# What makes species unique? The contribution of proteins with obscure features

**DOI:** 10.1186/gb-2006-7-7-r57

**Published:** 2006-07-19

**Authors:** Martin Gollery, Jeff Harper, John Cushman, Taliah Mittler, Thomas Girke, Jian-Kang Zhu, Julia Bailey-Serres, Ron Mittler

**Affiliations:** 1 Department of Biochemistry and Molecular Biology, University Of Nevada, Reno, NV 89557, USA; 2 Center for Plant Cell Biology, University Of California, Riverside, CA 92521, USA

## Abstract

**Background:**

Proteins with obscure features (POFs), which lack currently defined motifs or domains, represent between 18% and 38% of a typical eukaryotic proteome. To evaluate the contribution of this class of proteins to the diversity of eukaryotes, we performed a comparative analysis of the predicted proteomes derived from 10 different sequenced genomes, including budding and fission yeast, worm, fly, mosquito, *Arabidopsis*, rice, mouse, rat, and human.

**Results:**

Only 1,650 protein groups were found to be conserved among these proteomes (BLAST E-value threshold of 10^-6^). Of these, only three were designated as POFs. Surprisingly, we found that, on average, 60% of the POFs identified in these 10 proteomes (44,236 in total) were species specific. In contrast, only 7.5% of the proteins with defined features (PDFs) were species specific (17,554 in total). As a group, POFs appear similar to PDFs in their relative contribution to biological functions, as indicated by their expression, participation in protein-protein interactions and association with mutant phenotypes. However, POF have more predicted disordered structure than PDFs, implying that they may exhibit preferential involvement in species-specific regulatory and signaling networks.

**Conclusion:**

Because the majority of eukaryotic POFs are not well conserved, and by definition do not have defined domains or motifs upon which to formulate a functional working hypothesis, understanding their biochemical and biological functions will require species-specific investigations.

## Abstract 2

An analysis of proteins with obscure features in ten eukaryotic genomes revealed that the majority are species-specific.

## Background

Comparative analysis of eukaryotic genomes provides an unprecedented opportunity to investigate what makes a given species unique. The genetic mechanisms that generate species-specific differences include positively selected mutations that confer a fitness advantage, random fixation of selectively neutral mutations, and acquisition of new genes [1,2]. Divergence among species, therefore, includes variation in gene sequences, in particular those of regulatory genes, features of non-coding sequences and repetitive DNA, and gene number and repertoire [3,4]. To date, comparative genomics in eukaryotes has focused largely on genes that encode proteins with experimentally defined domains or motifs (proteins with defined features (PDFs)) [5,6]. Because the analysis of PDFs revealed a high degree of similarity among different species, it has been accepted widely that the uniqueness of a particular species was driven by changes in regulatory genes or elements [1-6], as opposed to the divergence of established coding sequences or the creation of new genes. This has led to a widespread perspective that just a few model organisms can provide the experimental foundation to assign functions to nearly every eukaryotic gene.

Noticeably lacking from the comparative analysis of eukaryotic genomes to date, however, is an analysis of the origins and functions of genes encoding proteins that currently lack defined motifs or domains (proteins with obscure features (POFs)) [7,8]. Expression profiling studies in different organisms suggested that POFs play an important role in many different biological processes. Nevertheless, their biological roles and origins remain poorly understood and elucidating their functions is currently a major goal of biological research in almost all organisms studied [7,8]. In this paper we examine the possibility that genes encoding POFs, which account for approximately one-quarter of all eukaryotic genes, play a role in determining differences among species. By analogy to the expectation that PDFs are often conserved among species [4-6], one might expect that POFs would show a parallel pattern of phylogenetic conservation. To test this assumption, we performed a comparative analysis of 10 different eukaryotic proteomes, including budding and fission yeast, worm, fruit fly, mosquito, *Arabidopsis*, rice, mouse, rat, and human. Surprisingly, in contrast to PDFs, we found that POFs include a much larger percentage of proteins that are highly divergent. Our results underscore the importance of delineating the origins and functions of POFs as an underlying cause of species specificity.

## Results

### Approximately one-quarter of eukaryotic proteins are POFs

**Figure 1:**
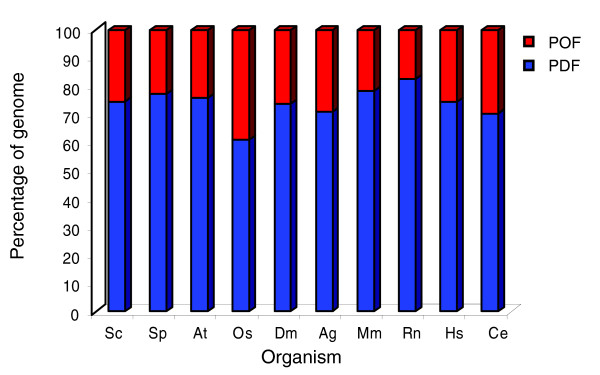
Representation of POFs in 10 different eukaryotic genomes. POFs represent 18% to 38% of the proteins in 10 different eukaryotic proteomes (*S. cerevisiae*, Sc; *S. pombe*, Sp; *A. thaliana*, At; *O. sativa*, Os; *D. melanogaster*, Dm; *A. gambiae*, Ag; *M. musculus*, Mm; *R. norvegicus*, Rn; *H. sapiens*, Hs; *C. elegans*, Ce). Proteomes were obtained and analyzed as described in Materials and methods.

Proteins were analyzed from ten different model proteomes and classified as POFs if they lacked an established domain or motif including domains of unknown function. The ten model proteomes were derived from gene models based on the genome sequences of *Saccharomyces cerevisiae *(Sc) and *Schizosaccharomyces pombe *(Sp), *Arabidopsis thaliana *(At), *Oryza sativa *(Os), *Drosophila melanogaster *(Dm), *Anopheles gambiae *(Ag), *Caenorhabditis elegans *(Ce), *Mus musculus *(Mm), *Rattus norvegicus *(Rn), and *Homo sapiens *(Hs). As shown in Figure 1, between 18% and 38% of all proteins (average 26%) predicted from each genome were classified as POFs.

### POFs are more divergent than PDFs

**Figure 2:**
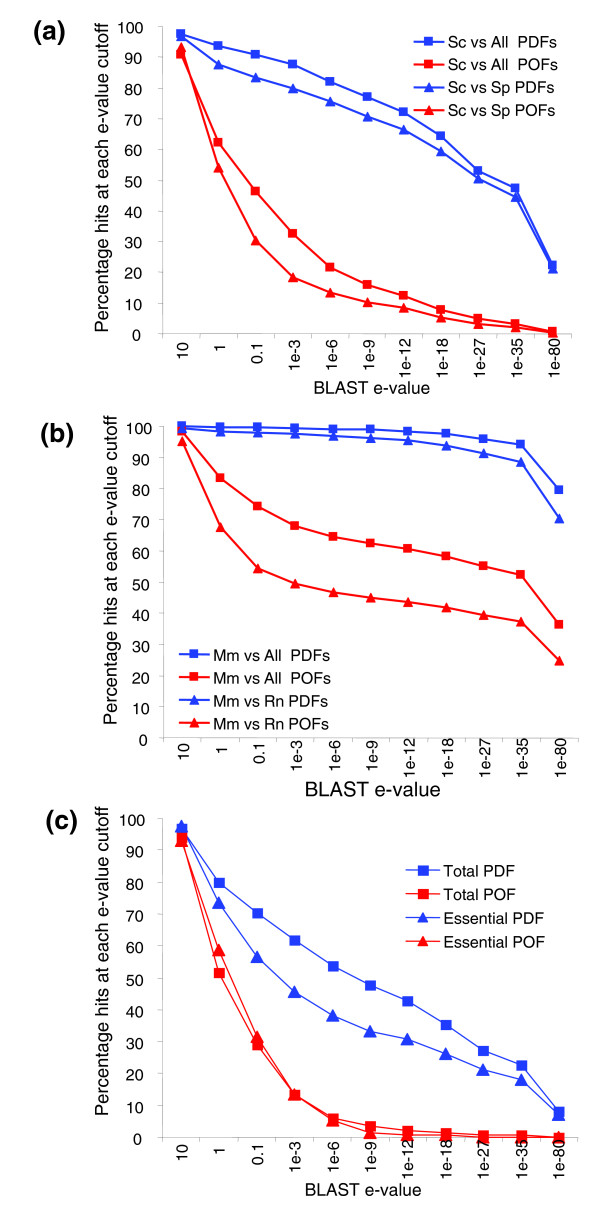
POFs are more divergent than PDFs in different eukaryotic proteomes.**(a) **Relative similarity among PDFs and POFs in *S. cerevisiae *(Sc) and *S. pombe *(Sp). **(b) **Relative similarity among PDFs and POFs in *M. musculus *(Mm) and *R. norvegicus *(Rn). **(c) **Relative similarity among total or essential PDFs and POFs in *S. cerevisiae *(Sc) and *C. elegans *(Ce). BLAST comparisons were performed as described in Materials and methods.

**Figure 3:**
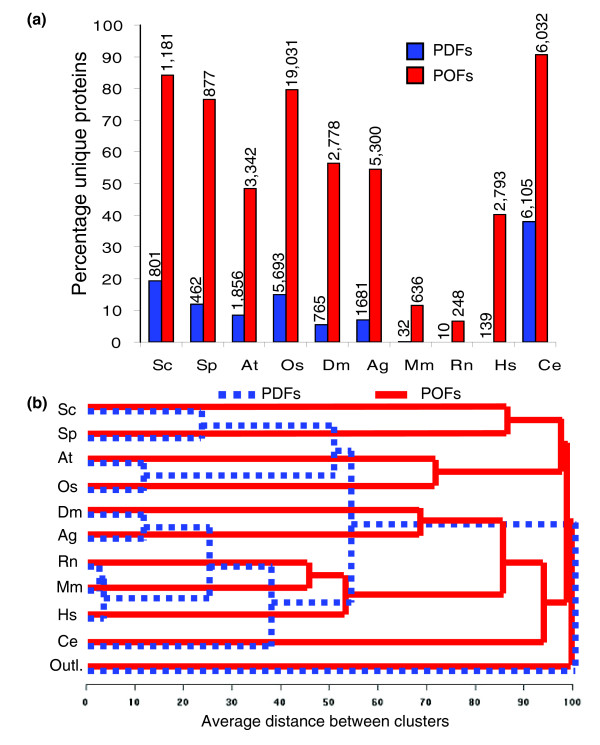
POFs are more likely to be species specific than PDFs. POFs are more likely to be species specific than PDFs among 10 different proteomes (*S. cerevisiae*, Sc; *S. pombe*, Sp; *A. thaliana*, At; *O. sativa*, Os; *D. melanogaster*, Dm; *A. gambiae*, Ag; *M. musculus*, Mm; *R. norvegicus*, Rn; *H. sapiens*, Hs; *C. elegans*, Ce). **(a) **Proportion of POFs and PDFs represented in unique protein sets determined among the 10 different proteomes. Specificity of a protein to a particular proteome was determined based on a BLAST e-value cutoff of 10^-6^. Numbers on top of bars donate the total number of proteins in each group. **(b) **Relationship trees among the 10 different proteomes shown in (a). Trees were constructed based on PDFs (dashed blue line) or POFs (red line). Proteome analyses, BLAST comparisons and tree construction were performed as described in Materials and methods. Outl., an outlier *E.coli *genome was used.

To evaluate the diversity among PDFs and POFs in different proteomes, we compared their sequence relatedness to each other using BLAST (Figure 2; Figures 1S to 3S in Additional data file 1). The percentage of related proteins was plotted as a function of similarity cutoff thresholds that ranged from non-stringent (BLAST E-values greater than 10^-6^) to stringent (from 10^-9 ^to 10^-80 ^or less). This method of plotting similarity differences permits the visualization of reproducible differences between PDFs and POFs across a wide range of cutoff thresholds. Unless noted otherwise, a BLAST similarity of greater than 10^-6 ^was used as the cutoff threshold for classifying a sequence as related. Using this similarity threshold, a total of 1,650 protein groups were found to be conserved among all 10 proteomes (Tables 1S and 2S in Additional data file 1). Surprisingly, only 3 of those (<0.2%) were POFs (as represented in *S. cerevisiae *by gi|6319274|, gi|6320573| and gi|6324048|).

Among the 10 proteomes, POFs always showed significantly more divergence than PDFs, as illustrated for *S. cerevisiae *and *S. pombe*, or *M. musculus *and *R. norvegicus *in Figure 2a, b, respectively (and documented for all proteomes in Figures 1S to 3S in in Additional data file 1). For example, in a comparison of proteomes from budding and fission yeast (Figure 2a), the percentage of similar POFs was typically five-fold less than PDFs, when evaluated with BLAST cutoff thresholds from 10^-6 ^to 10^-18^. This difference can also be illustrated by comparing the BLAST values that correspond to the point at which 50% of the PDFs or POFs show relatedness with other proteomes (referred to as the 50% similarity point). In the case of budding and fission yeast, these E-values correspond to approximately 1 for POFs and 10^-30 ^for PDFs. Using a 50% similarity point as a standard for comparison, the POFs from fission and budding yeast show 30 orders of magnitude higher divergence than PDFs. This higher divergence of POFs was also corroborated in comparisons of Sc with the nine other proteomes (Sc versus All, Figure 2a; Figures 1S and 2S in Additional data file 1). The same pattern of POFs divergence was seen in a pair-wise comparison of the two insect proteomes, the two plant proteomes, and the two vertebrate proteomes (Figure 2b; Figures 1S and 2S in Additional data file 1).

This relatively high divergence within the group of POFs was also true in a parallel analysis in which only proteins with essential functions were compared. In Figure 2c, the essential POFs and PDFs were compared from proteomes of Ce [9] and Sc [10]. These two organisms have been subjected to systematic deletion or RNAi analyses to define essential genes. In this comparison, essential POFs were still 3 to 9 orders of magnitude more dissimilar as shown by a comparison between the 50% similarity points.

For each proteome we identified the set of unique proteins not found in any of the other nine proteomes analyzed (Tables 3S and 4S in Additional data file 1). We then determined the relative proportion of POFs or PDFs designated as unique (BLAST cutoff of 10^-6^). As shown in Figure 3a, the relative percentage of POFs designated as unique was always higher than the percentage of PDFs. On average, we found that 60% of POFs (44,236 in total) are species-specific, in contrast to only 7.5% (17,554 in total) of the PDFs.

Average sequence similarity relationship trees constructed for all 10 proteomes, based on POFs or PDFs (Figure 3b), revealed that the divergence of POFs among the 10 different proteomes was consistently greater than that of PDFs, supporting the contention that POFs account for the majority of phylogenetically specific ORFs (Figure 3a).

### Comparative analysis of the human and chimpanzee proteomes

The recent publication of a draft chimpanzee (*Pan troglodytes *(Pt)) genome [11] provided us with a unique opportunity to compare the similarity of POFs and PDFs encoded in two proteomes that are estimated to have diverged only 5 to 7 million years ago. Compared to the degree of similarity among POFs from human and mouse, the degree of identity among POFs from human and chimpanzee was much higher (Figure 3S in Additional data file 1). To examine what proportion of human-specific proteins are POFs or PDFs we performed a BLAST search (E-value cutoff of 10^-6^) of all published sequences against the human proteome. Consistent with POFs representing the majority of species-specific proteins (Figure 3a), POFs accounted for all 27 expressed human-specific proteins (Table 5S in Additional data file 1) not observed in the genomes of any other organism.

### Relative contribution of POFs to biological functions

**Figure 4:**
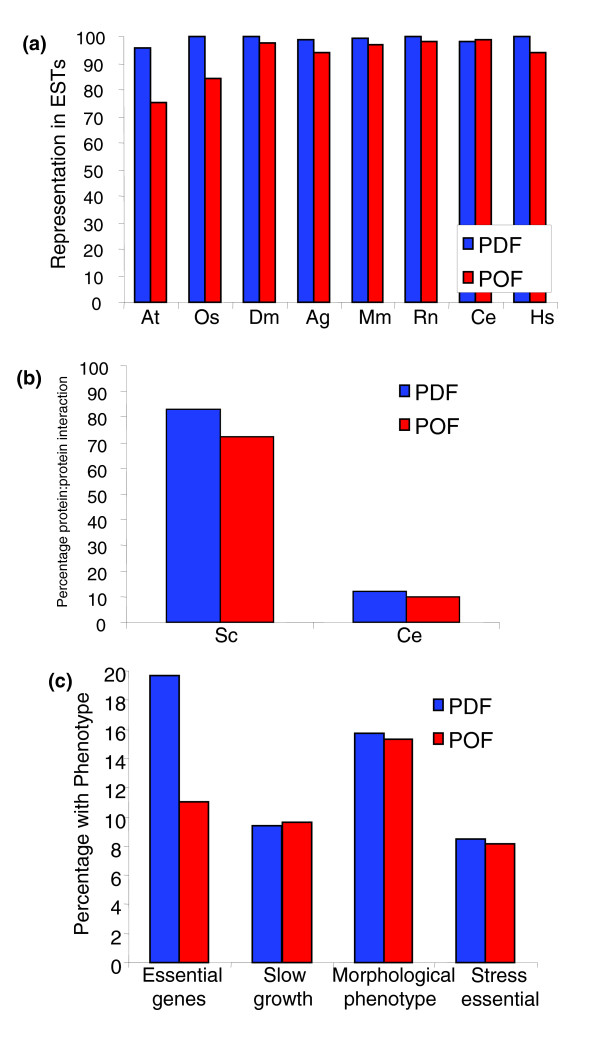
Relative contribution of POFs to biological functions. **(a) **Representation of POFs in EST libraries from different organisms (*A. thaliana*, At; *O. sativa*, Os; *D. melanogaster*, Dm; *A. gambiae*, Ag; *M. musculus*, Mm; *R. norvegicus*, Rn; *C. elegans*, Ce; *H. sapiens*, Hs). **(b) **Representation of POFs in protein-protein interaction networks in Sc and Ce. **(c) **Percent phenotypic penetrance of PDFs and POFs in Sc. EST libraries, protein-protein interaction data, insertional mutagenesis data, and sequence comparisons were obtained/performed as described in Materials and methods.

To evaluate POFs for their functional relevance, we first compared the percentage of PDFs and POFs that were represented in expressed sequence tag (EST) collections (Figure 4a). The six animal transcriptomes all showed a greater than 95% representation for transcripts encoding POFs. This high representation was only slightly less than that observed for PDFs. Whereas the two plant transcriptomes showed a somewhat larger difference, the POFs still showed a representation of greater than 75%. Thus, POFs are similar to PDFs in their representation as actively expressed mRNAs.

To compare the relative contributions of PDFs and POFs to protein-protein interaction networks, we examined the percentage representation of each protein class in global interaction data sets available for the Sc [12] and Ce [13] proteomes (Figure 4b). While the POFs showed a slightly reduced representation compared to PDFs, the relative differences were less than 7%.

To compare the relative phenotypic contribution of both protein groups, we examined the percent representation of corresponding mutant phenotypes from the genome-wide functional analyses conducted for *S. cerevisiae *(Sc) [10] (Figure 4c). With the exception of a potentially noteworthy two-fold lower representation of POFs in the essential gene categories, PDFs and POFs showed a similar percent contribution to other phenotypic categories. Similar results were found with *C. elegans *genome-wide functional analysis (Ce) [9] (data not shown).

Together, the findings presented in Figure 4 suggest that POFs, as a group, are not being mis-represented by an unusually high percentage of proteins being incorrectly predicted from inaccurate gene models. Rather, POFs appear comparable to PDFs in their relative contribution to an organism's repertoire of functional proteins.

### POFs are typically shorter and contain a higher content of disordered structure

**Figure 5:**
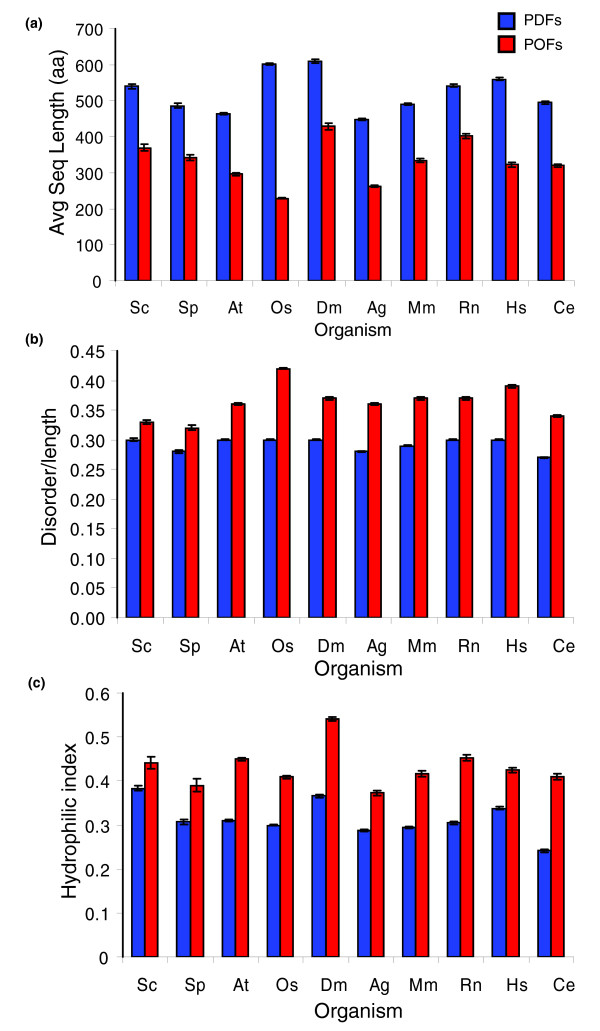
Distinct differences in several biophysical characteristics between POFs and PDFs. A comparison of PDFs and POFs shows distinct differences in several biophysical characteristics (*S. cerevisiae*, Sc; *S. pombe*, Sp; *A. thaliana*, At; *O. sativa*, Os; *D. melanogaster*, Dm; *A. gambiae*, Ag; *M. musculus*, Mm; *R. norvegicus*, Rn; *H. sapiens*, Hs; *C. elegans*, Ce). **(a) **Average length of PDFs and POFs from different species. **(b) **Structural disorder index (disorder/length) of PDFs and POFs from different species. **(c) **Hydrophilic index of PDFs and POFs from different species. Proteomes analyses were performed as described in Materials and methods.

To investigate if there are any structural characteristics other than established motifs and domains that might distinguish POFs from PDFs, the physical properties of the two groups of proteins were examined. Compared to PDFs, POFs as a group are 40% shorter (Figure 5a; ANOVA *p *< 0.001), have a higher percentage of disordered structure (Figure 5b; ANOVA *p *< 0.001), a higher content of hydrophilic residues (Figure 5c; ANOVA *p *< 0.001), a higher content of small amino acids (for example, proline and serine), glutamine, and arginine and a lower content of aliphatic (for example, isoleucine and valine) and aromatic (tyrosine) amino acids, and aspartic acid (Table 6S in Additional data file 1; ANOVA *p *< 0.01). Therefore, in addition to the absence of established motifs and domains, POFs, on average, have physical characteristics that further distinguish them from PDFs.

## Discussion

Assigning a role to proteins with unknown function is a major goal of current and future genomic research. Homology searches have traditionally been used to assign specific domain structures to proteins, thereby classifying them into protein families with putative function [5,6]. The distinction made in this paper between proteins with defined motifs or domains (PDFs), and those with undefined or obscure features (POFs) (Figure 1), underlined proteins that could not be assigned any known function by homology searches. Interestingly, POFs as a group were found to be shorter, more hydrophilic and more disordered than PDFs (Figure 5).

Our analysis of 10 different proteomes (Figures 1 to 3; Figures 1s to 3s in Additional data file 1) revealed a striking difference in conservation between PDFs and POFs. E-value plots show that this difference is evident whether similarities are measured with stringent or non-stringent criteria. With a minimum E-value similarity threshold of 10^-6^, a total of 44,236 phylogenetically specific POFs were identified (Figure 3a). In contrast, relatively few (17,544) PDFs were identified as phylogenetically specific. The opposite trend was observed for conserved proteins. Only 3 POF groups were conserved in all 10 proteomes, in contrast to 1,650 PDF groups (Tables 1S and 2S in Additional data file 1). In total, 60% of POFs appear to be phylogenetically restricted, in contrast to only 7.5% of PDFs.

One explanation as to why POFs show a higher degree of species specificity than PDFs is that POFs, in contrast to PDFs, could include a disproportionately higher number of proteins incorrectly predicted from pseudogenes or incorrect gene models. This could result in an artifact in which random sequences or non-functional proteins distort the overall diversity of this group of proteins. However, several lines of evidence presented here (Figures 2, 4 and 5) suggest that this trivial explanation is not the case. For example, even in the relatively well-characterized yeast genomes, the pattern of higher sequence diversification among the POFs is consistent with that seen in the larger more complex genomes. Furthermore, as a group, POFs appear similar to PDFs with respect to mRNA expression, phenotypic penetrance, and involvement in protein-protein interactions.

An additional and noteworthy characteristic supporting the contention that most POFs contribute functional activities is that, on average, POFs show a greater degree of predicted disordered structure (Figure 5). Empirical definitions of disordered structures have been derived from examining regions of proteins that fail to show a consistent or defined structure in a crystallized protein. These regions of disorder show a strong correlation with biochemical studies that suggest their involvement in protein-protein interactions, as well as in providing key regions for regulating a protein's activity via a structural conformation switch [14-18]. Importantly, the disorder prediction software programs used here do not provide high scores to random 'junk' DNA sequences, providing another line of evidence that gene models encoding POFs were not just artifactual predictions derived from 'junk' DNA. Rather, the high levels of disordered structures in POFs support their potential roles in regulatory networks in which protein conformational changes or protein-protein interactions are key.

Together, the above arguments strongly support the view that the average POF is just as likely to have a biological function as a protein with a defined motif or domain. We favor, therefore, a genetic explanation for the unusual diversity within the group of POFs. That explanation is that genes encoding the majority of POFs are arising *de novo *or diverging at an evolutionary rate much higher than genes encoding PDFs. In support of this possibility, POFs are consistently more divergent among different proteomes (Figure 3b), and are preferentially represented as singletons in the different genomes (Figure 4S in Additional data file 1).

There may be several distinct mechanisms contributing to the genetic diversity of POFs. For example, some POFs may have structures that are highly flexible and can diverge with few structural constraints. By contrast, some POFs may have conserved tertiary structures, but are nevertheless showing rapid divergence in their primary sequence. In either case, a widely conserved motif or distinct domain signature may never be found within the primary sequences of a large subset of the currently defined POFs. Nevertheless, some POFs may ultimately be found to have definable features. One reason that these features currently remain undefined may be related to the sociology of science. In general, scientists have focused their molecular research on relatively few organisms, and devoted most of their resources to in-depth studies of relatively few proteins. Those proteins or pathways are often chosen because of their general relevance to fundamental questions in a broad group of organisms or because these proteins exhibit strong evolutionary conservation and are judged on this basis to have greater intrinsic functional relevance. By contrast, the study of a species-specific protein is often a lonely pursuit. Another source of bias lies in the tendency inherent in classical biochemical methods, which are strongly biased towards the production and characterization of folded, active proteins that have highly ordered structures (for example, PDFs) and for which structural information is more readily obtained [19]. In contrast, disordered proteins (for example, POFs) are less well studied because they lack a readily recognized activity and structural information is more difficult to obtain for these proteins.

Previous work identified a class of proteins termed 'ORFans' that have no significant sequence similarity to any other open reading frame (ORF) and are, therefore, unique to a specific organism [20-22]. In contrast to the definition of POFs that is based on the presence of an observed domain or motif, the definition of an ORFan is based strictly on sequence homology. Thus, ORFans could include POFs as well as PDFs. Indeed, as shown in Figure 3a, POFs and PDFs accounted for 70.4% (42,218) and 29.6% (17,544) of all proteins unique among the 10 analyzed proteomes, respectively. Moreover, the majority of POFs from Mm and Rn were found to be similar (Figure 3a), suggesting that although some overlap exists between ORFans and POFs, homologs of many POFs can be found in similar genomes (Figure 3a). An interesting observation that was recently made for ORFans could also hold true for POFs. It was observed that some ORFans, although demonstrating no sequence homology to any known protein, could fold into a three-dimensional structure that resembled a protein with a known function [22]. In addition to being novel genes unique to an organism or a lineage, some POFs or ORFans could, therefore, be the result of convergent evolution. Thus, they might be distant members of known proteins, with similar functions and three-dimensional structure, but with sequences that have diverged beyond recognition.

## Conclusion

The advent of genome sequences has reinvigorated an effort to understand the origins of species specificity. This is a daunting challenge, emphasized by the fact that in the 10 proteomes analyzed here we identified 44,236 phylogenetically specific proteins with undefined or obscure features (POFs). In contrast to PDFs, which have established domains or motifs that can be used to formulate working hypotheses about a protein's function, advancing our understanding of POFs must proceed without such clues. Our analysis here provides an expectation that, on average, 60% of a eukaryote's set of POFs will be highly divergent, and that functional studies will ultimately need to be conducted on a species-specific basis. For example, the human genome encodes 27 proteins that currently cannot be found in genome sequences of any model organism, including the chimpanzee sequence (Table 5S in Additional data file 1). Consistent with expectations from this study, these human-specific proteins are all POFs. Eventually, the function of these unique proteins will need to be studied in humans. Our results support a general expectation that to understand the unique biology of a given organism will ultimately involve understanding the functions of an unexpectedly large number of proteins that have: no defined motifs or domains; are likely to have significant regions of disordered structure; and are restricted to a single species or a closely related phylogenetic branch.

## Materials and methods

### Protein data and definition of POFs

Gene models and proteomes for *Saccharomyces cerevisiae *(Sc), *Schizosaccharomyces pombe *(Sp), *Arabidopsis thaliana *(At), *Oryza sativa *(Os), *Drosophila melanogaster *(Dm), *Anopheles gambiae *(Ag), *Caenorhabditis elegans *(Ce), *Mus musculus *(Mm), *Rattus norvegicus *(Rn), and *Homo sapiens *(Hs) were downloaded from the NCBI website [23] and from TAIR [24] on 5 December, 2004. Gene models and proteomes for *Pan troglodytes *(Pt),*Mus musculus *(Mm), *Rattus norvegicus *(Rn), and *Homo sapiens *(Hs) were also downloaded from Ensembl [25] on 10 September, 2005.

To standardize the classification for which proteins are POFs and which are PDFs, we applied a consistent analysis method to all genes regardless of their current annotation. This analysis method involved an HMMPFAM [26] search against several major signature databases: PFAM [27], TIGRFAM [28], SMART [29], and Superfamily [30]. A protein sequence with a match to one or more of the models in any one of these databases, including domains of unknown function, was flagged as a PDF. Sequences with no matches to any one of the models in any database were flagged as a POF. The definition of POFs used in our work was similar to that used in [9,31].

### BLAST comparisons

BLAST comparisons of PDFs and POFs among different proteomes were performed using TeraBLAST running on an accelerated DeCypher server [32]. The comparisons of PDFs and POFs between each proteome and its respective collection of ESTs or between PDFs and POFs from each proteome and all other genomes translated in all reading frames were accomplished using TBLASTn [33]. ESTs were obtained on 5 December, 2004 from NCBI [23] except for *Arabidopsis*, which was downloaded at the same time from TAIR [24]. To examine the representation of PDFs and POFs from Sc or Ce in existing phenotypic studies, or existing protein-protein interaction datasets, POFs and PDFs, obtained as described above, were matched to existing datasets [9,10,12,13,34].

### Prediction of protein properties

Prediction of relative disorder for PDFs and POFs was performed with the DisEMBL 1.4 prediction program [35]. Due to the large numbers of proteins that were analyzed, we used DisEMBL locally rather than at the website [36]. To obtain an overall value for the percentage of proteins that were disordered, SAS V9.1 (SAS Institute Inc., Cary, NC, USA) was used to sum the total regions that were predicted to be disordered and to divide it by the length for each protein analyzed. Hydrophilic index and amino acid content were calculated with the *ad hoc *perl script hydrophil.pl, developed by Garay-Arroyo *et al*. [37] using the Kyte-Doolittle values for hydrophilicity. SAS was used to perform statistical analysis (descriptive statistics and ANOVA) for the hydrophilic index of POFs and PDFs from hydrophil.pl results (Figure 5c), for variations in amino acid content between POFs and PDFs (Table 6S in Additional data file 1), and for sequence length and relative disorder of POFs and PDFs (Figure 5a, b). Because the average length of the POFs was shorter than that of the PDFs, a length correction was used to eliminate bias in the scoring function of the program.

### 'All-against-all' comparisons and tree generation

'All-against-all' comparisons used to generate sets of species-specific proteins for both PDFs and POFs from Sc, Sp, At, Os, Dm, Ag, Ce, Mm, Rn and Hs were performed using TeraBLAST running on an accelerated DeCypher server [32], with a cutoff threshold of 10^-6^. A tree showing the relationships among Sc, Sp, At, Os, Dm, Ag, Ce, Mm, Rn and Hs proteomes was constructed using the reciprocal percentage of the number of genes that the organisms have in common. This tree was constructed using the SAS cluster procedure utilizing the average linkage method, and graphed using the SAS tree procedure [38]. Tree diagrams are discussed in the context of cluster analysis by Hartigan [39], and Everitt [40].

## Additional data files

The following additional data are available with the online version of this paper. Additional data file 1 contains supplemental figures and tables. Supplemental Figures 1-1 through 1-10 show the relative similarity among PDFs and POFs in all proteomes studied. Supplemental Figure 2 shows the relative similarity among PDFs and POFs in selected proteomes measured as percentage identity or percentage similarity. Supplemental Figure 3 shows the relative similarity among PDFs and POFs between Hs and Pt, compared to Hs and Mm. Supplemental Figure 4 shows cluster analysis of POFs and PDFs in selected proteomes. Supplemental Table 1 lists common POFs to all proteomes analyzed. Supplemental Table 2 lists common PDFs to all proteomes analyzed. Supplemental Table 3 lists unique PDFs from all proteomes analyzed. Supplemental Table 4 lists unique POFs from all proteomes analyzed. Supplemental Table 5 lists 27 unique Hs proteins with representation in EST databases. Supplemental Table 6 describes the amino acid content of POFs and PDFs from the different proteomes studied.

## Supplementary Material

Additional data file 1 Supplemental Figures 1-1 through 1-10 show the relative similarity among PDFs and POFs in all proteomes studied. Click here for file
