# Supplementary material for: What makes species unique? The contribution of proteins with obscure features
Source: Genome Biol. 2006 Jul 19;7(7):R57. doi: 10.1186/gb-2006-7-7-r57 (PMC1779552; doi:10.1186/gb-2006-7-7-r57)

Suppl. Fig. 1-1. Sc against all other proteomes

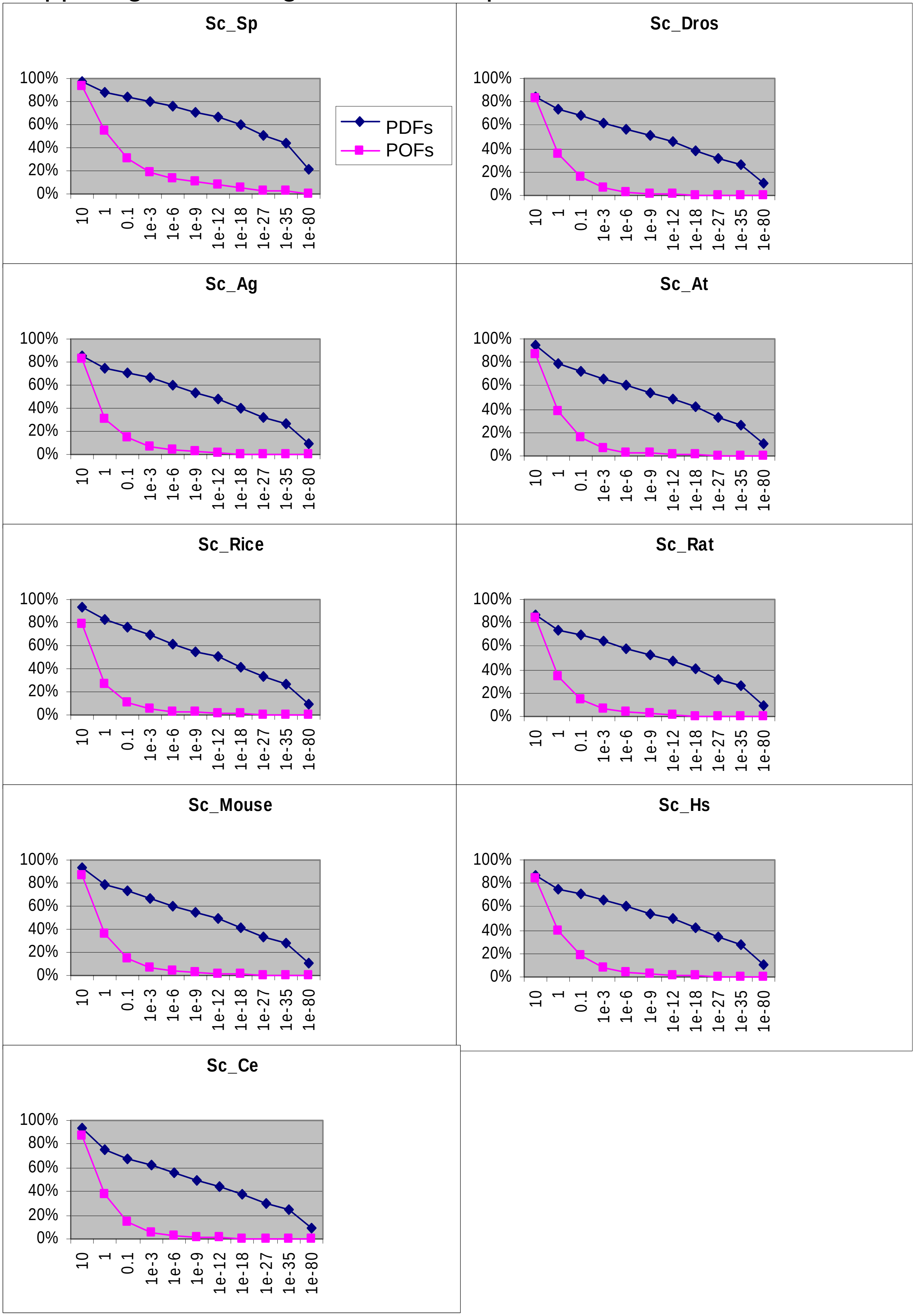

Suppl. Fig. 1-2. Sp against all other proteomes

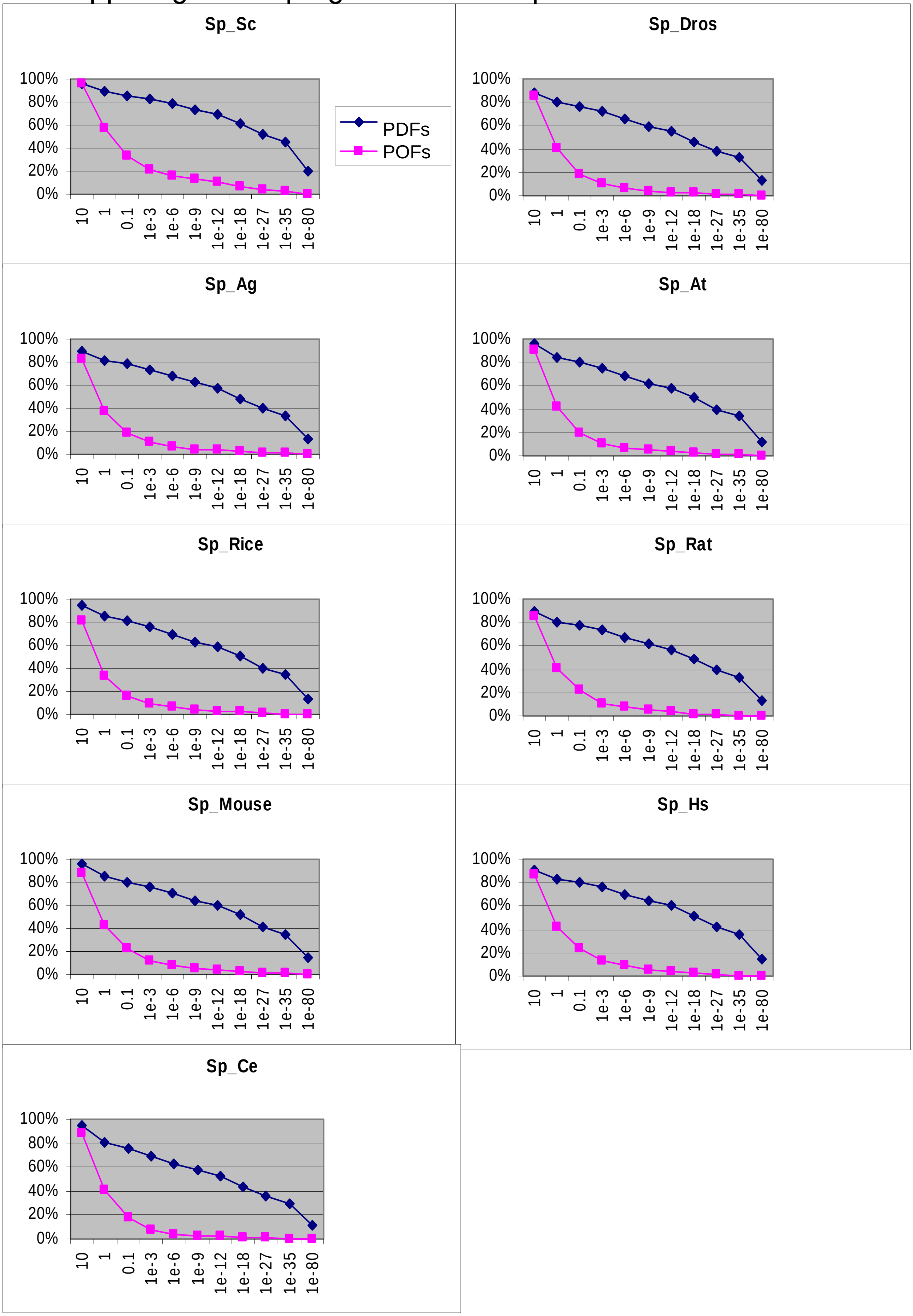

Suppl. Fig. 1-3. At against all other proteomes

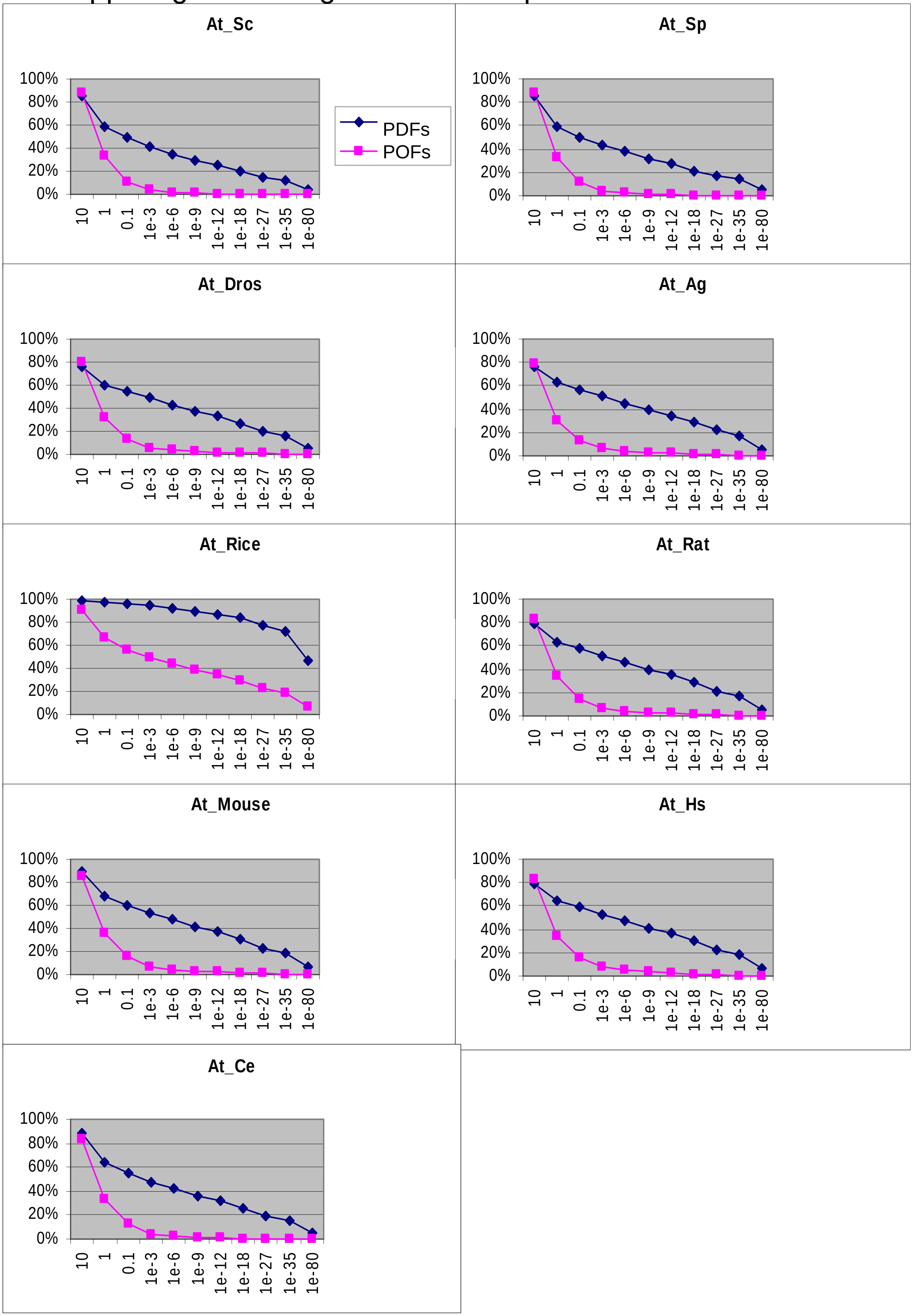

Suppl. Fig. 1-4. Os against all other proteomes

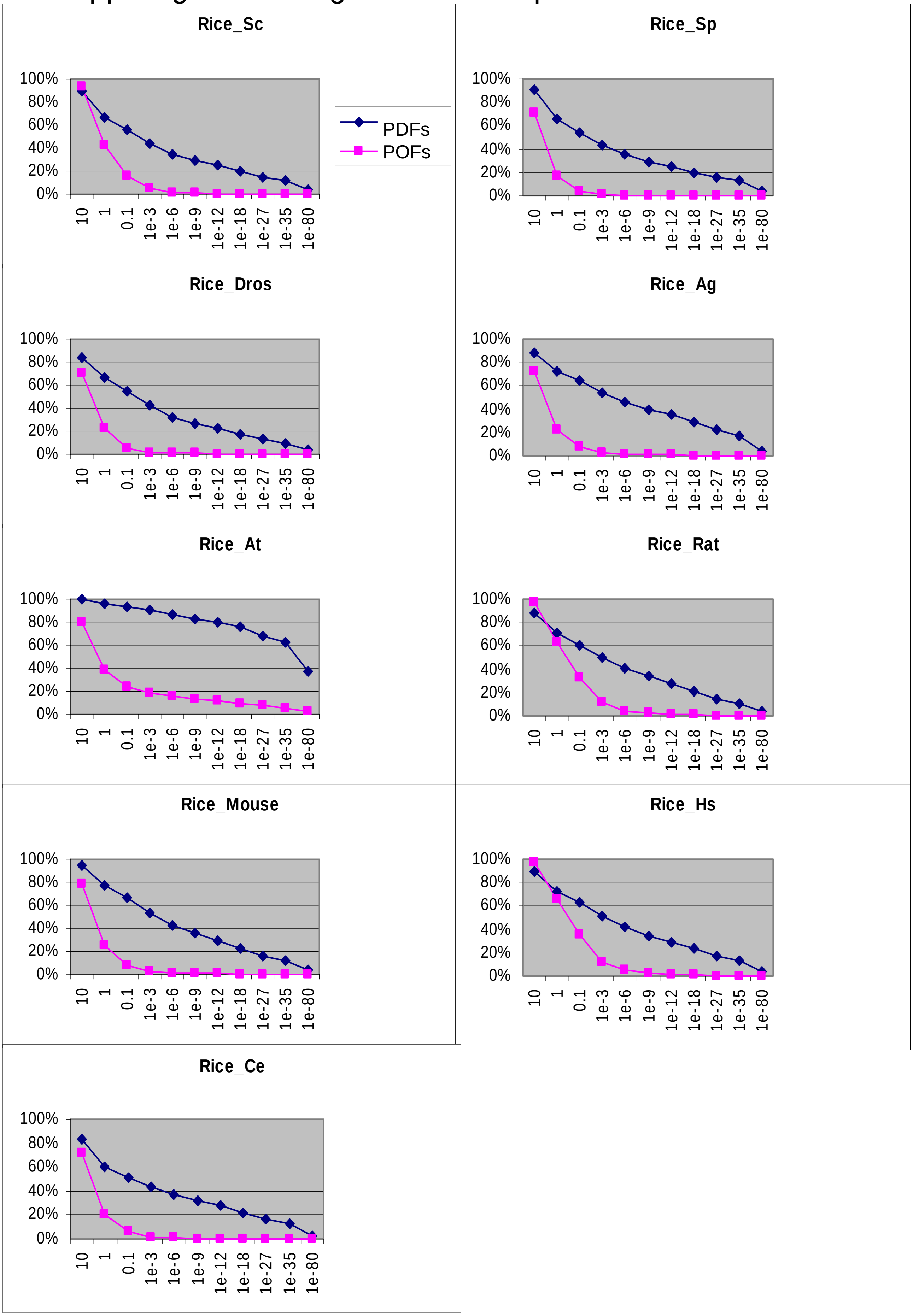

Suppl. Fig. 1-5. Dm against all other proteomes

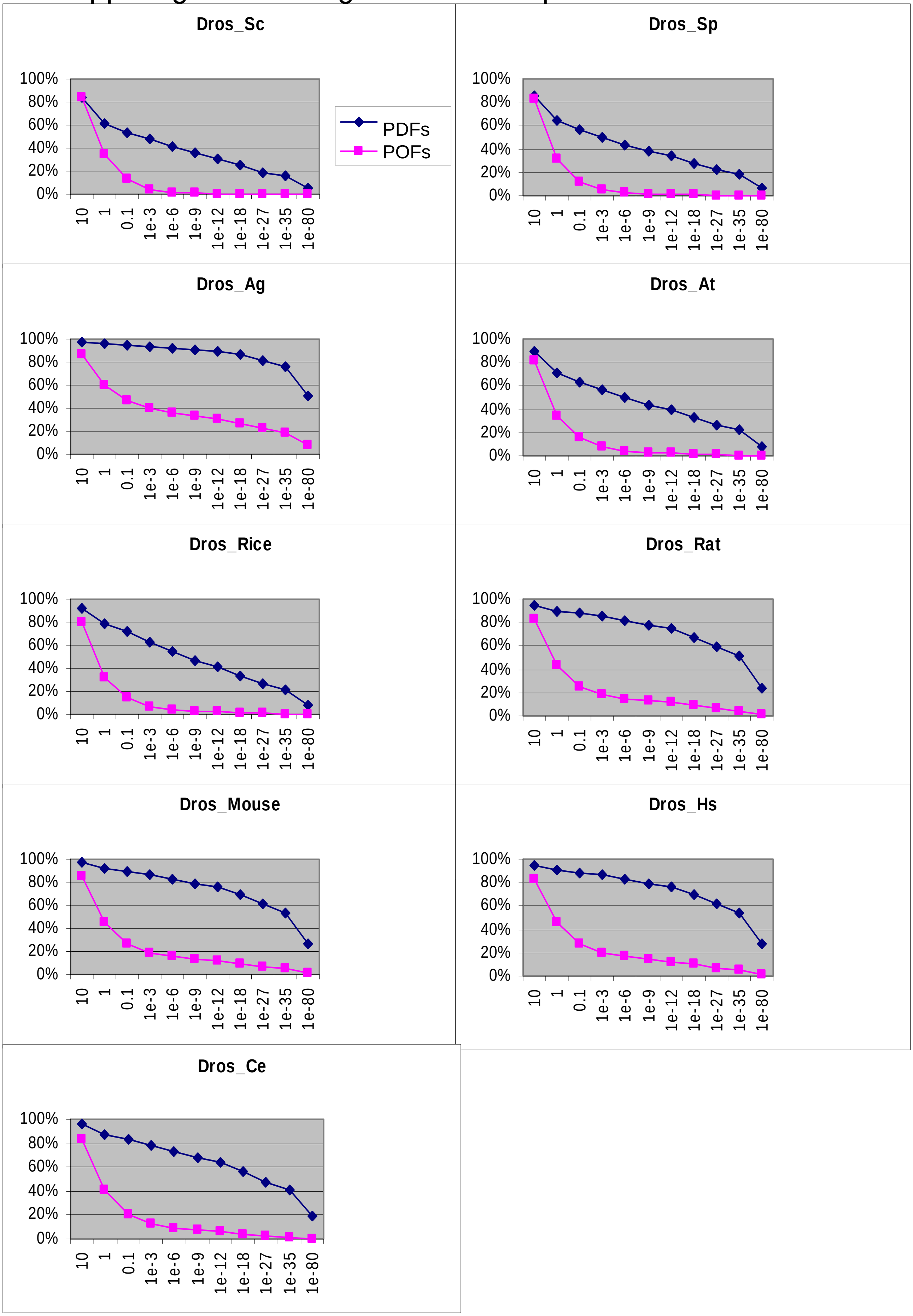

Suppl. Fig. 1-6. Ag against all other proteomes

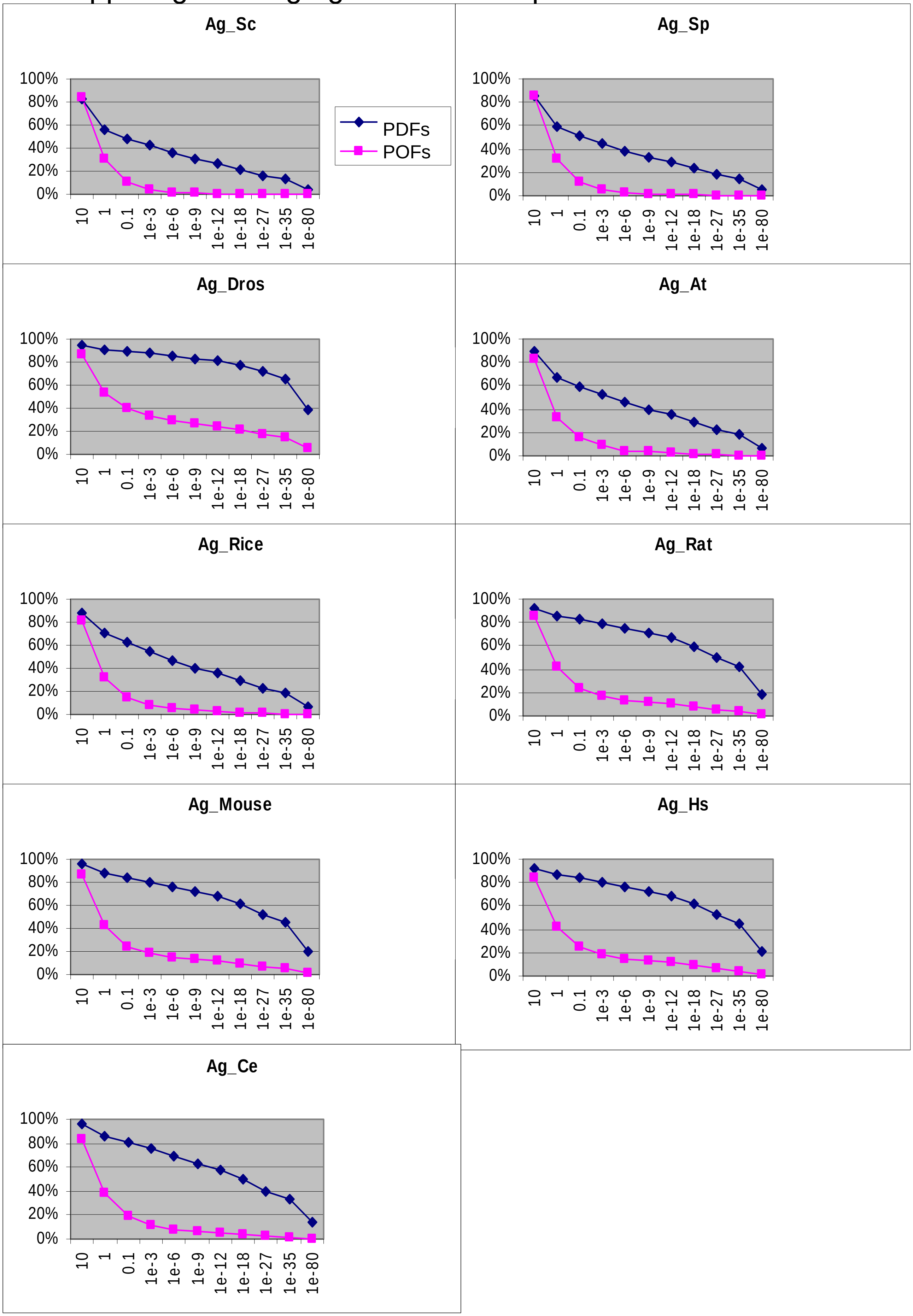

Suppl. Fig. 1-7. Mm against all other proteomes

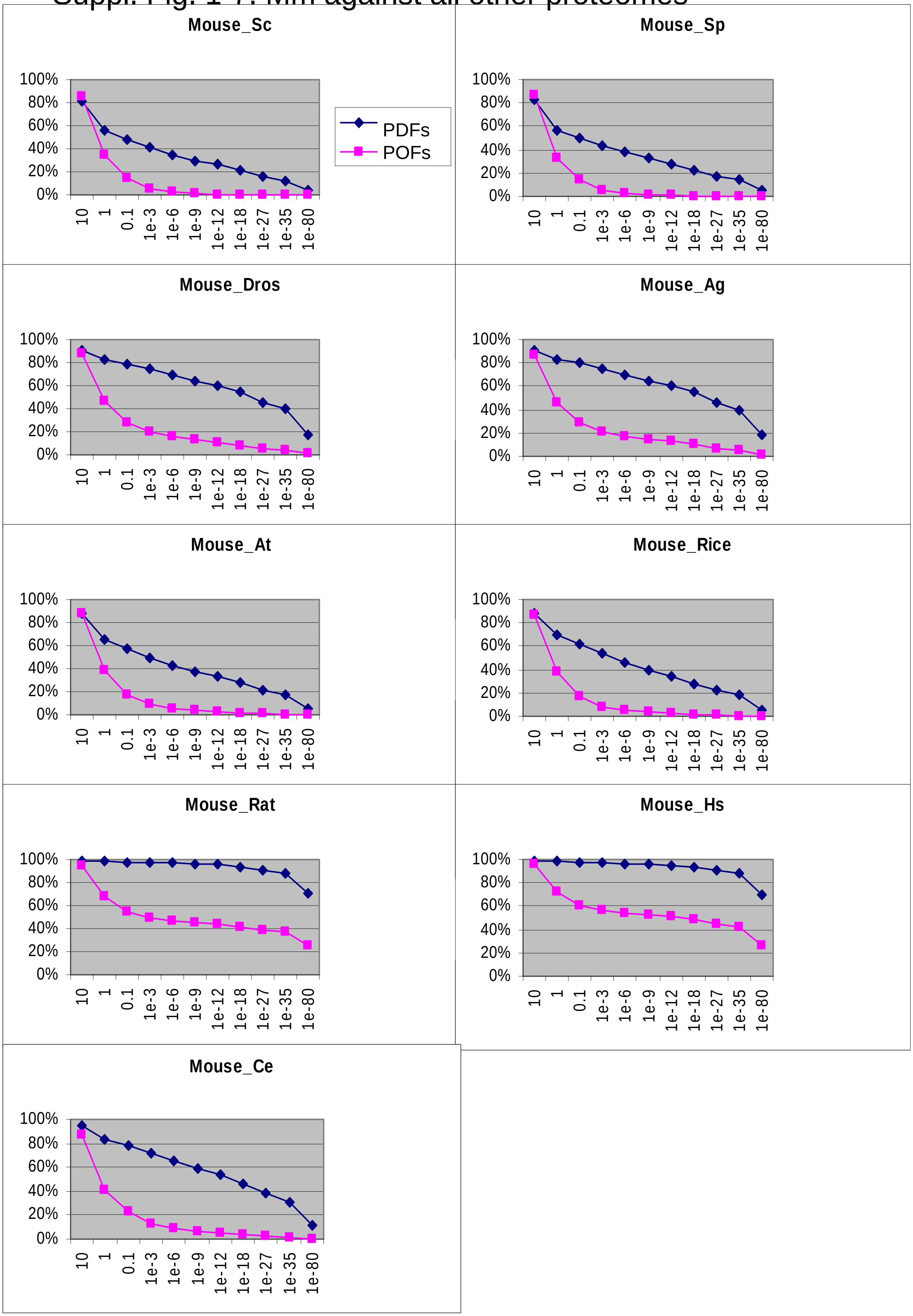

Suppl. Fig. 1-8. Rn against all other proteomes

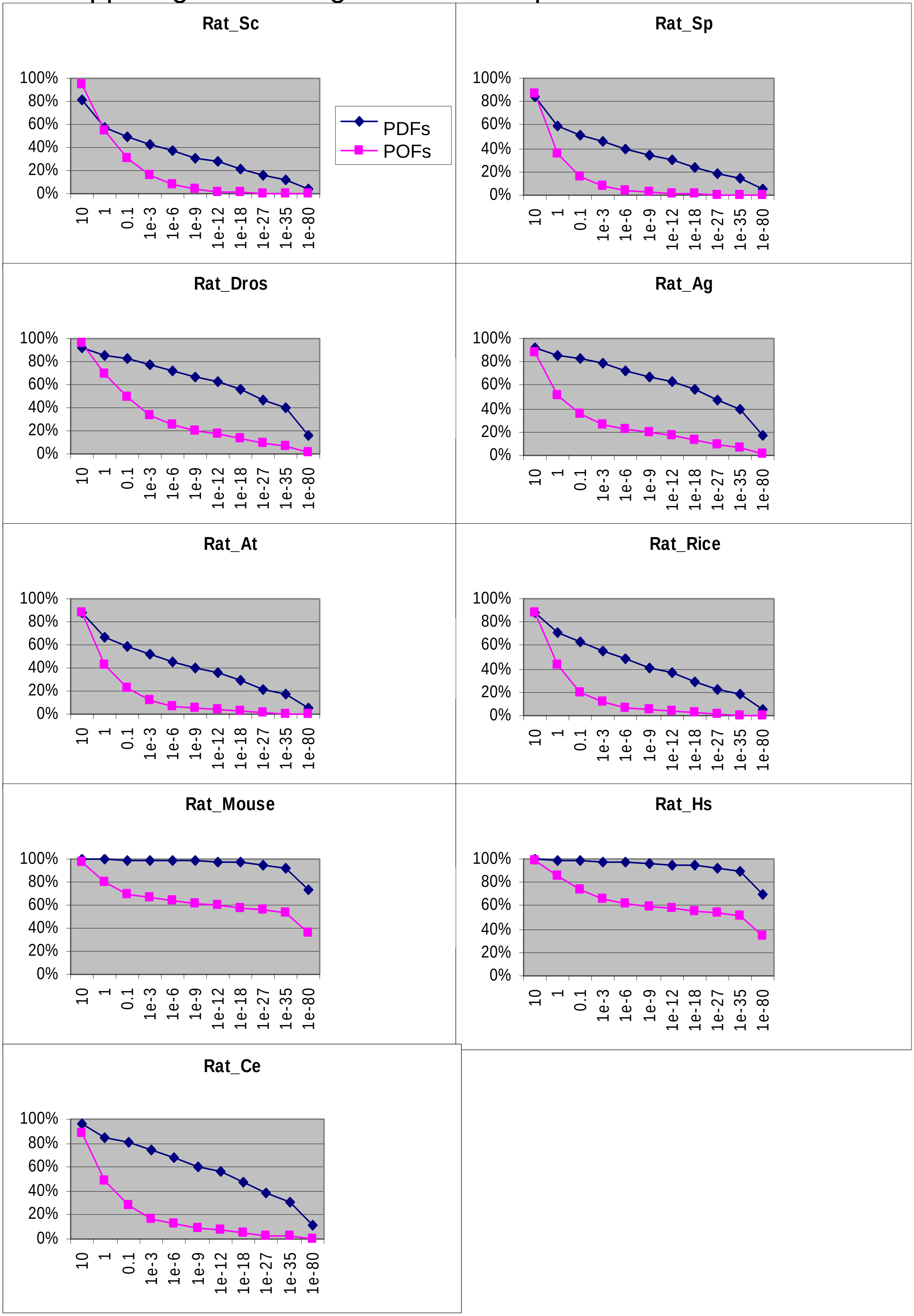

Suppl. Fig. 1-9. Hs against all other proteomes

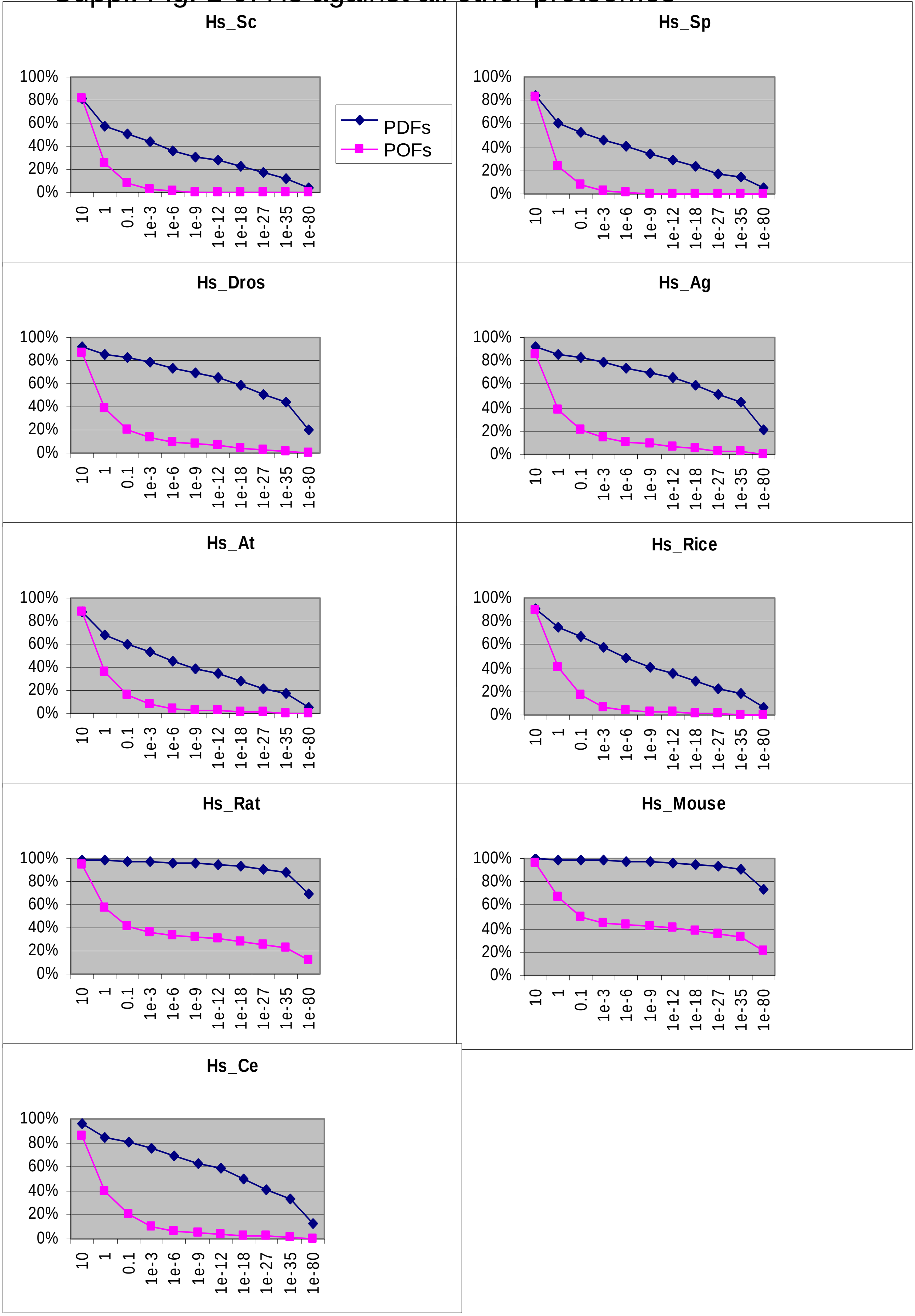

Suppl. Fig. 1-10. Ce against all other proteomes

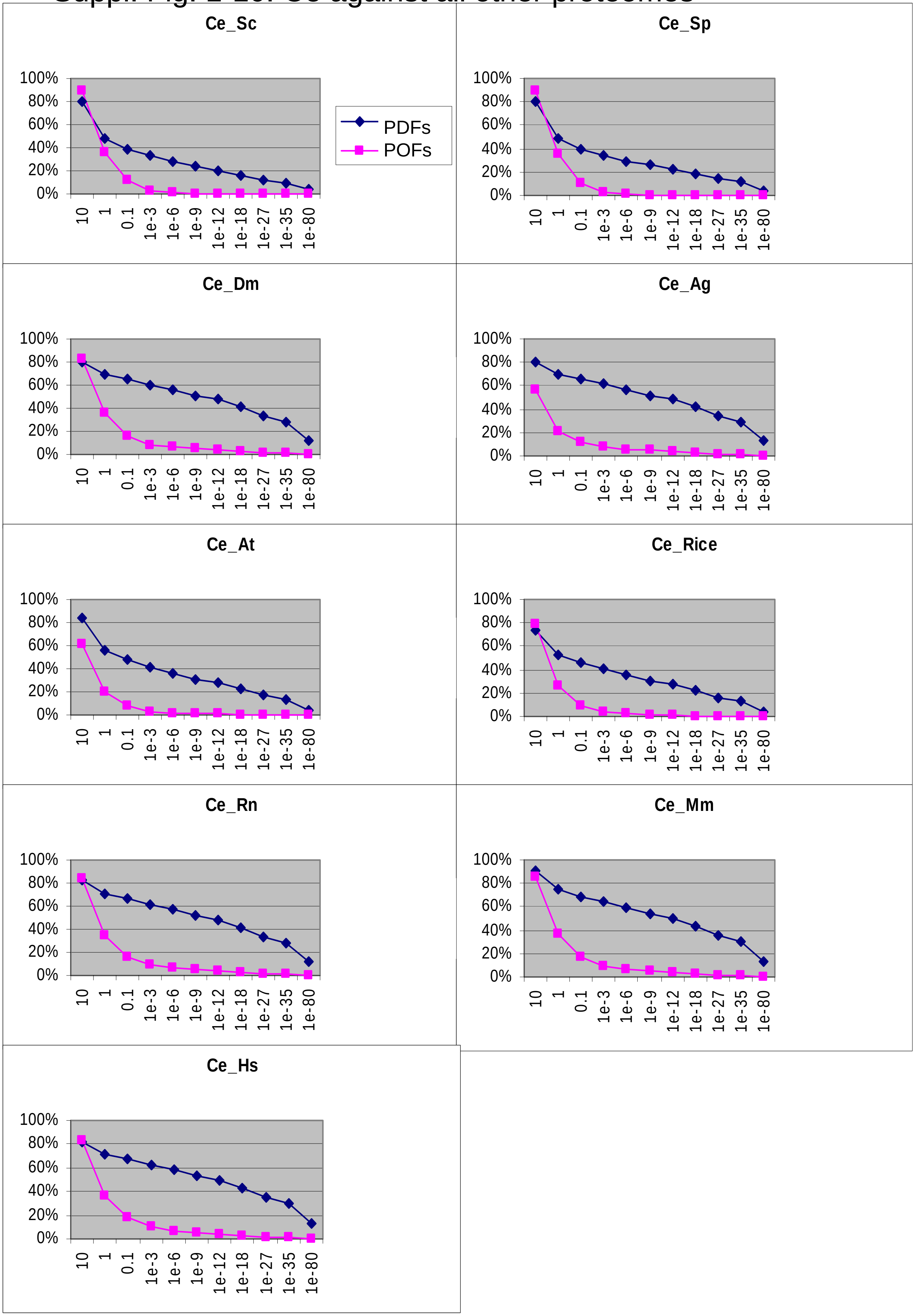

Suppl Fig. 2. Comparison of selected proteomes based on % identity (Iden) or % similarity (Sim).

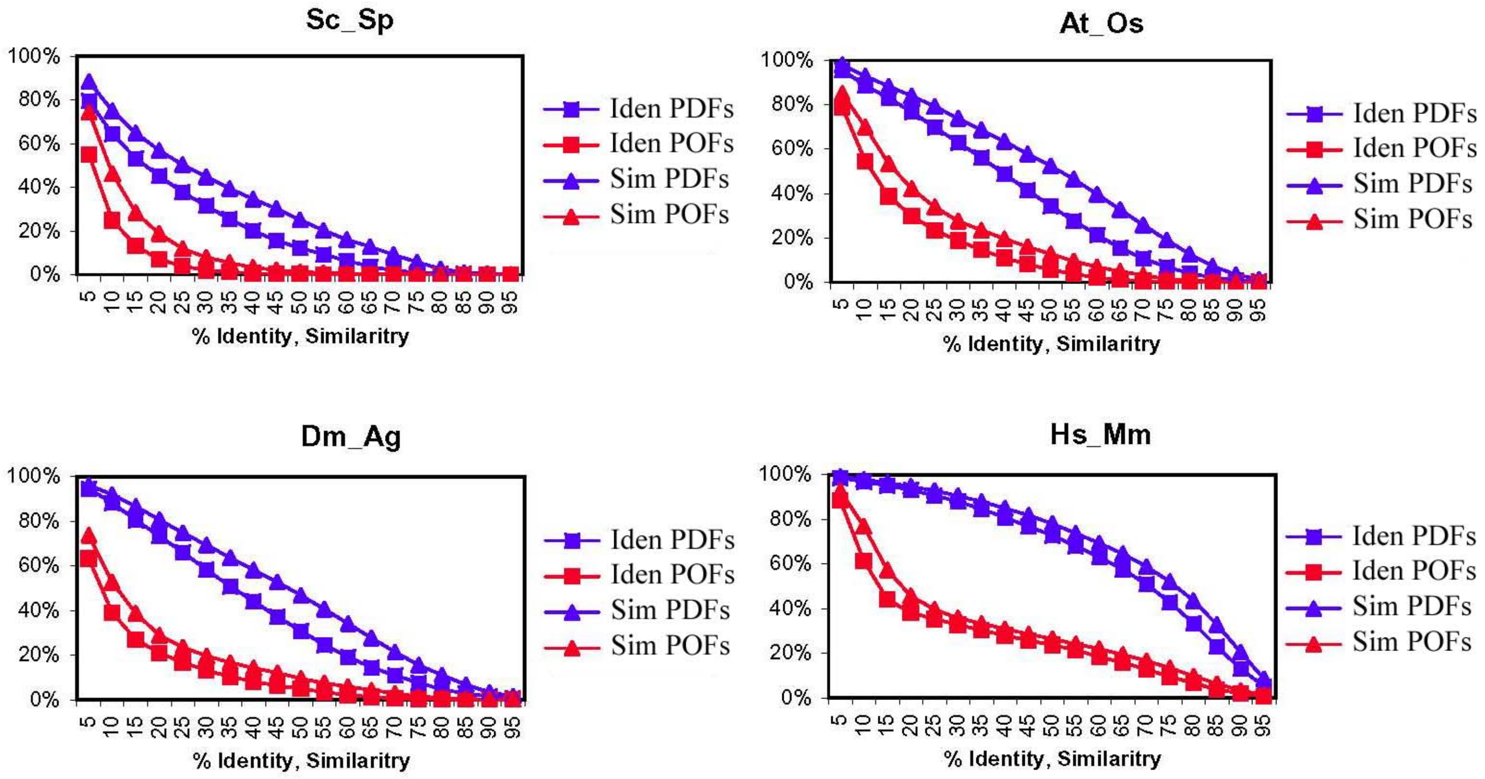

Suppl Fig. 3. Similarity between PDFs and POFs from Hs and Pt and between Hs and mouse (Mm).

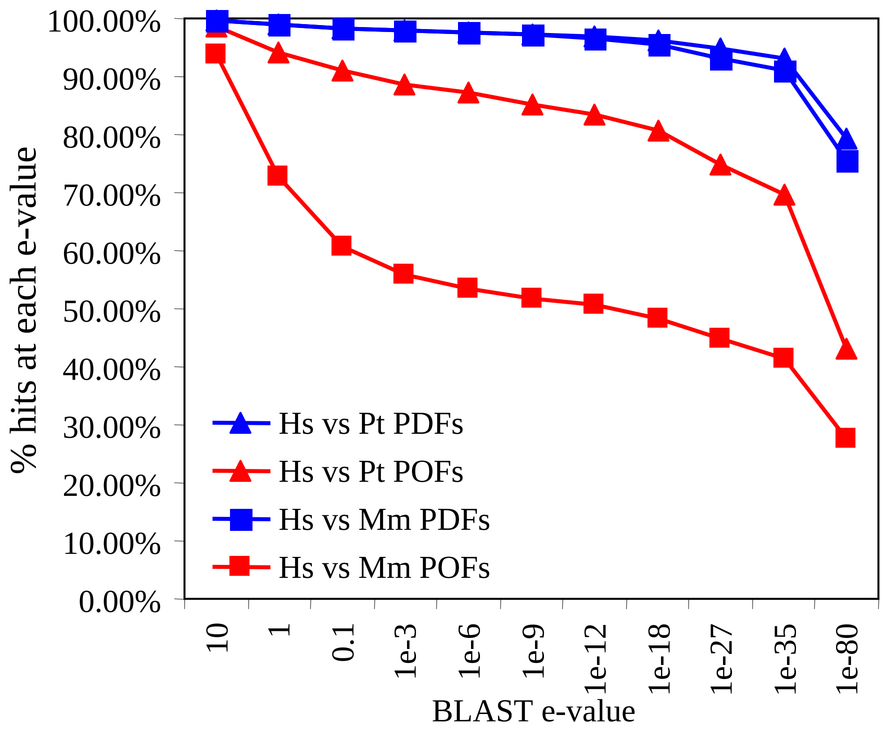

Suppl. Fig. 4. Cluster analysis of PDFs and POFs in 4 different proteomes showing that POFs are mainly represented as singletons.

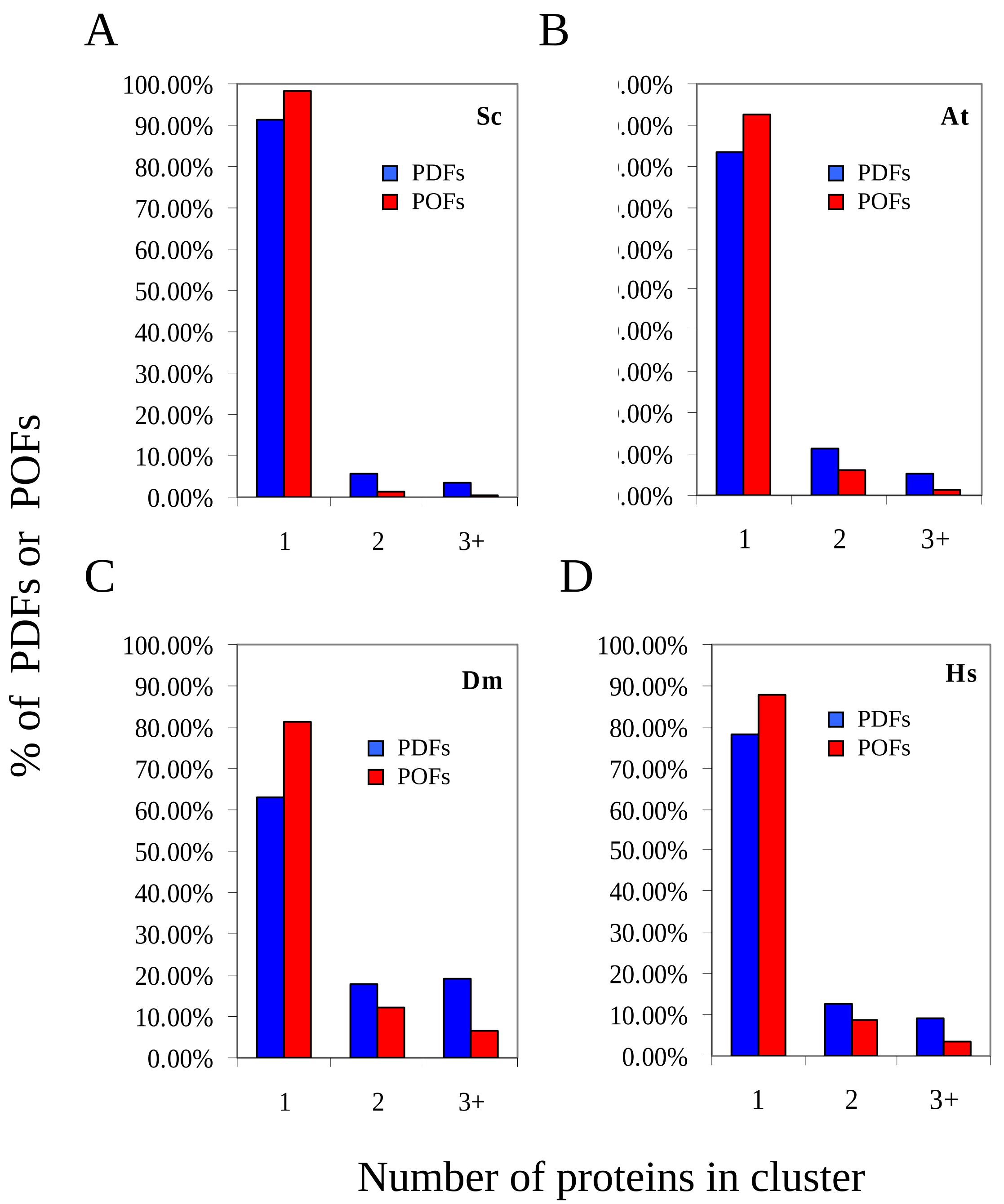

Suppl. Table 1. POFs common to all proteomes analyzed (1 donates a hit at a BLAST e-value of 10-6).

Sc POFs	Ag	At	Ce	Pt	Dm	Hs	Mm	Rn	Os	Sp	All	All without Pt
gi|6319274|	1	1	1	1	1	1	1	1	1	1	10	9
gi|6320573|	1	1	1	1	1	1	1	1	1	1	10	9
gi|6324048|	1	1	1	1	1	1	1	1	1	1	10	9
gi|6320491|	1	1	1	1		1	1	1	1	1	9	8
gi|6321483|	1	1	1	1	1	1	1		1	1	9	8
gi|6322843|	1	1	1	1		1	1	1	1	1	9	8
gi|6322948|	1	1	1	1	1	1	1	1		1	9	8
gi|6323080|	1	1	1	1		1	1	1	1	1	9	8
gi|6325386|	1	1	1	1		1	1	1	1	1	9	8
gi|6320562|		1	1	1	1	1	1	1	1		8	7
gi|6324087|	1			1	1	1	1	1	1	1	8	7
gi|6325426|	1	1		1		1	1	1	1	1	8	7
gi|37362680|		1	1			1	1	1	1	1	7	7
gi|6320646|	1	1	1		1		1		1	1	7	7
gi|6322465|	1	1			1		1	1	1	1	7	7
gi|6322669|		1	1			1	1	1	1	1	7	7
gi|6323573|		1	1		1	1	1	1	1		7	7
gi|6325012|	1	1			1		1	1	1	1	7	7
gi|6319581|	1			1		1	1	1	1	1	7	6
gi|6320505|	1		1	1	1	1			1	1	7	6
gi|6320791|	1			1	1	1	1	1		1	7	6
gi|6321264|	1			1	1	1	1	1		1	7	6
gi|6322042|	1		1	1	1		1	1		1	7	6
gi|6322594|		1	1	1		1	1	1		1	7	6
gi|6324190|		1	1	1		1	1	1	1		7	6
gi|6325159|	1		1	1	1	1	1			1	7	6
gi|6319600|	1				1	1	1	1		1	6	6
gi|6319930|			1		1		1	1	1	1	6	6
gi|6319978|	1				1	1	1	1		1	6	6
gi|6320353|	1		1			1		1	1	1	6	6
gi|6320681|	1	1	1		1				1	1	6	6
gi|6323623|	1	1			1		1		1	1	6	6
gi|6323655|		1			1	1	1	1	1		6	6
gi|6321372|	1			1		1	1	1		1	6	5
gi|6321503|	1			1		1	1	1		1	6	5
gi|6321569|		1	1	1	1	1				1	6	5
gi|6323143|	1			1	1	1	1			1	6	5
gi|14318473|		1					1	1	1	1	5	5
gi|6319366|	1	1			1				1	1	5	5
gi|6323487|	1					1	1	1		1	5	5
gi|6324347|	1	1			1				1	1	5	5
gi|6325382|	1	1					1	1		1	5	5
gi|6320735|				1		1	1	1		1	5	4
gi|6321913|	1			1		1	1	1			5	4
gi|6322076|				1	1	1	1	1			5	4
gi|6322927|				1		1	1	1		1	5	4
gi|9755334|		1	1	1	1				1		5	4
gi|6320293|					1		1	1	1		4	4
gi|6321557|			1		1			1		1	4	4

gi|6323692|	1						1	1		1	4	4
gi|6323978|	1		1		1					1	4	4
gi|6324077|	1	1							1	1	4	4
gi|6324084|	1				1		1			1	4	4
gi|6325374|						1	1	1		1	4	4
gi|14318550|				1		1	1	1			4	3
gi|6322447|	1	1		1					1		4	3
gi|6323230|				1	1		1			1	4	3
gi|6323674|		1		1	1	1					4	3
gi|10383809|	1		1							1	3	3
gi|14318495|	1				1					1	3	3
gi|41629682|			1		1					1	3	3
gi|6319544|	1				1					1	3	3
gi|6319804|	1				1			1			3	3
gi|6319994|							1	1		1	3	3
gi|6320380|		1							1	1	3	3
gi|6321588|		1							1	1	3	3
gi|6322124|	1	1	1								3	3
gi|6322494|	1		1							1	3	3
gi|6323677|						1	1	1			3	3
gi|6324202|					1		1	1			3	3
gi|6324382|			1				1	1			3	3
gi|6325111|							1	1		1	3	3
gi|6325150|		1							1	1	3	3
gi|6325413|		1							1	1	3	3
gi|14318538|			1	1		1					3	2
gi|6319818|				1					1	1	3	2
gi|6321316|				1		1				1	3	2
gi|6321713|		1		1						1	3	2
gi|6323877|				1			1	1			3	2
gi|6325306|				1		1	1				3	2
gi|6319759|		1							1		2	2
gi|6319760|									1	1	2	2
gi|6320098|							1			1	2	2
gi|6320104|		1				1					2	2
gi|6320208|						1				1	2	2
gi|6320620|							1			1	2	2
gi|6321711|									1	1	2	2
gi|6321926|									1	1	2	2
gi|6323116|	1		1								2	2
gi|6324069|									1	1	2	2
gi|6324104|									1	1	2	2
gi|6324157|			1							1	2	2
gi|6324238|						1				1	2	2
gi|6325002|		1	1								2	2
gi|6325362|	1									1	2	2
gi|44829553|				1	1						2	1
gi|6324465|			1	1							2	1
gi|10383774|	1										1	1
gi|10383784|						1					1	1
gi|10383800|										1	1	1
gi|10383807|										1	1	1

gi|13129166|				1	1	1
gi|14318470|				1	1	1
gi|41629677|		1			1	1
gi|42759851|				1	1	1
gi|44829555|				1	1	1
gi|6319273|				1	1	1
gi|6319309|				1	1	1
gi|6319381|				1	1	1
gi|6319539|	1				1	1
gi|6319582|				1	1	1
gi|6319638|				1	1	1
gi|6319648|				1	1	1
gi|6319670|				1	1	1
gi|6319692|				1	1	1
gi|6319767|				1	1	1
gi|6319989|				1	1	1
gi|6320055|				1	1	1
gi|6320136|				1	1	1
gi|6320166|				1	1	1
gi|6320167|				1	1	1
gi|6320231|				1	1	1
gi|6320381|				1	1	1
gi|6320399|		1			1	1
gi|6320501|			1		1	1
gi|6320516|				1	1	1
gi|6320553|				1	1	1
gi|6320555|				1	1	1
gi|6320568|				1	1	1
gi|6320575|				1	1	1
gi|6320585|				1	1	1
gi|6320642|	1				1	1
gi|6320672|				1	1	1
gi|6320770|				1	1	1
gi|6320830|				1	1	1
gi|6320838|				1	1	1
gi|6320860|				1	1	1
gi|6320916|				1	1	1
gi|6321179|				1	1	1
gi|6321190|				1	1	1
gi|6321220|				1	1	1
gi|6321266|				1	1	1
gi|6321309|				1	1	1
gi|6321312|				1	1	1
gi|6321451|				1	1	1
gi|6321513|				1	1	1
gi|6321523|				1	1	1
gi|6321536|				1	1	1
gi|6321596|				1	1	1
gi|6321613|				1	1	1
gi|6321654|				1	1	1
gi|6321668|				1	1	1
gi|6321708|				1	1	1

gi|6321712|							1	1	1
gi|6321761|							1	1	1
gi|6321823|	1							1	1
gi|6321825|							1	1	1
gi|6321889|							1	1	1
gi|6321971|							1	1	1
gi|6322034|							1	1	1
gi|6322053|							1	1	1
gi|6322081|							1	1	1
gi|6322095|							1	1	1
gi|6322098|							1	1	1
gi|6322101|							1	1	1
gi|6322104|							1	1	1
gi|6322140|							1	1	1
gi|6322148|							1	1	1
gi|6322201|							1	1	1
gi|6322209|							1	1	1
gi|6322254|						1		1	1
gi|6322290|							1	1	1
gi|6322379|							1	1	1
gi|6322421|							1	1	1
gi|6322457|							1	1	1
gi|6322578|							1	1	1
gi|6322600|							1	1	1
gi|6322711|							1	1	1
gi|6322741|							1	1	1
gi|6322801|							1	1	1
gi|6322804|					1			1	1
gi|6322836|							1	1	1
gi|6322873|							1	1	1
gi|6322918|							1	1	1
gi|6322941|							1	1	1
gi|6322959|							1	1	1
gi|6322993|				1				1	1
gi|6323005|							1	1	1
gi|6323023|							1	1	1
gi|6323097|			1					1	1
gi|6323113|							1	1	1
gi|6323179|							1	1	1
gi|6323232|							1	1	1
gi|6323254|							1	1	1
gi|6323444|	1							1	1
gi|6323527|							1	1	1
gi|6323533|							1	1	1
gi|6323636|							1	1	1
gi|6323716|							1	1	1
gi|6323732|		1						1	1
gi|6323738|							1	1	1
gi|6323771|							1	1	1
gi|6323777|							1	1	1
gi|6323824|			1					1	1
gi|6323856|							1	1	1

gi|6323929|							1	1	1
gi|6323951|							1	1	1
gi|6324002|	1							1	1
gi|6324033|						1		1	1
gi|6324038|							1	1	1
gi|6324050|							1	1	1
gi|6324123|							1	1	1
gi|6324235|							1	1	1
gi|6324250|							1	1	1
gi|6324259|							1	1	1
gi|6324274|					1			1	1
gi|6324304|		1						1	1
gi|6324345|							1	1	1
gi|6324348|							1	1	1
gi|6324367|							1	1	1
gi|6324481|							1	1	1
gi|6324547|							1	1	1
gi|6324554|							1	1	1
gi|6324565|							1	1	1
gi|6324591|							1	1	1
gi|6324994|							1	1	1
gi|6325001|							1	1	1
gi|6325023|							1	1	1
gi|6325129|							1	1	1
gi|6325172|							1	1	1
gi|6325227|				1				1	1
gi|6325253|							1	1	1
gi|6325406|							1	1	1
gi|6681845|							1	1	1
gi|7839165|							1	1	1
gi|6323126|			1					1	0
gi|6324430|			1					1	0

Suppl. Table 2. PDFs common to all proteomes analyzed (1 donates a hit at a BLAST e-value of 10-6).

Sc PDFs	Ag	At	Ce	Dm	Hs	Mm	Rn	Os	Sp	Pt	All	All, not chir
gi|10383767|	1	1	1	1	1	1	1	1	1	1	10	9
gi|10383773|	1	1	1	1	1	1	1	1	1	1	10	9
gi|10383780|	1	1	1	1	1	1	1	1	1	1	10	9
gi|10383781|	1	1	1	1	1	1	1	1	1	1	10	9
gi|10383790|	1	1	1	1	1	1	1	1	1	1	10	9
gi|10383793|	1	1	1	1	1	1	1	1	1	1	10	9
gi|10383804|	1	1	1	1	1	1	1	1	1	1	10	9
gi|10383805|	1	1	1	1	1	1	1	1	1	1	10	9
gi|10383806|	1	1	1	1	1	1	1	1	1	1	10	9
gi|10383810|	1	1	1	1	1	1	1	1	1	1	10	9
gi|10383811|	1	1	1	1	1	1	1	1	1	1	10	9
gi|13129156|	1	1	1	1	1	1	1	1	1	1	10	9
gi|14318437|	1	1	1	1	1	1	1	1	1	1	10	9
gi|14318438|	1	1	1	1	1	1	1	1	1	1	10	9
gi|14318474|	1	1	1	1	1	1	1	1	1	1	10	9
gi|14318479|	1	1	1	1	1	1	1	1	1	1	10	9
gi|14318480|	1	1	1	1	1	1	1	1	1	1	10	9
gi|14318481|	1	1	1	1	1	1	1	1	1	1	10	9
gi|14318482|	1	1	1	1	1	1	1	1	1	1	10	9
gi|14318483|	1	1	1	1	1	1	1	1	1	1	10	9
gi|14318484|	1	1	1	1	1	1	1	1	1	1	10	9
gi|14318486|	1	1	1	1	1	1	1	1	1	1	10	9
gi|14318497|	1	1	1	1	1	1	1	1	1	1	10	9
gi|14318501|	1	1	1	1	1	1	1	1	1	1	10	9
gi|14318502|	1	1	1	1	1	1	1	1	1	1	10	9
gi|14318504|	1	1	1	1	1	1	1	1	1	1	10	9
gi|14318510|	1	1	1	1	1	1	1	1	1	1	10	9
gi|14318513|	1	1	1	1	1	1	1	1	1	1	10	9
gi|14318514|	1	1	1	1	1	1	1	1	1	1	10	9
gi|14318517|	1	1	1	1	1	1	1	1	1	1	10	9
gi|14318519|	1	1	1	1	1	1	1	1	1	1	10	9
gi|14318521|	1	1	1	1	1	1	1	1	1	1	10	9
gi|14318522|	1	1	1	1	1	1	1	1	1	1	10	9
gi|14318526|	1	1	1	1	1	1	1	1	1	1	10	9
gi|14318527|	1	1	1	1	1	1	1	1	1	1	10	9
gi|14318528|	1	1	1	1	1	1	1	1	1	1	10	9
gi|14318531|	1	1	1	1	1	1	1	1	1	1	10	9
gi|14318532|	1	1	1	1	1	1	1	1	1	1	10	9
gi|14318536|	1	1	1	1	1	1	1	1	1	1	10	9
gi|14318541|	1	1	1	1	1	1	1	1	1	1	10	9
gi|14318545|	1	1	1	1	1	1	1	1	1	1	10	9
gi|14318553|	1	1	1	1	1	1	1	1	1	1	10	9
gi|14318554|	1	1	1	1	1	1	1	1	1	1	10	9
gi|14318555|	1	1	1	1	1	1	1	1	1	1	10	9
gi|14318557|	1	1	1	1	1	1	1	1	1	1	10	9
gi|14318562|	1	1	1	1	1	1	1	1	1	1	10	9
gi|14318575|	1	1	1	1	1	1	1	1	1	1	10	9
gi|14318576|	1	1	1	1	1	1	1	1	1	1	10	9
gi|14318578|	1	1	1	1	1	1	1	1	1	1	10	9
gi|14318580|	1	1	1	1	1	1	1	1	1	1	10	9
gi|16740527|	1	1	1	1	1	1	1	1	1	1	10	9
gi|27808705|	1	1	1	1	1	1	1	1	1	1	10	9
gi|27808711|	1	1	1	1	1	1	1	1	1	1	10	9
gi|27808712|	1	1	1	1	1	1	1	1	1	1	10	9
gi|27808716|	1	1	1	1	1	1	1	1	1	1	10	9
gi|31126970|	1	1	1	1	1	1	1	1	1	1	10	9
gi|37362609|	1	1	1	1	1	1	1	1	1	1	10	9
gi|37362612|	1	1	1	1	1	1	1	1	1	1	10	9
gi|37362615|	1	1	1	1	1	1	1	1	1	1	10	9
gi|37362617|	1	1	1	1	1	1	1	1	1	1	10	9
gi|37362622|	1	1	1	1	1	1	1	1	1	1	10	9
gi|37362623|	1	1	1	1	1	1	1	1	1	1	10	9
gi|37362631|	1	1	1	1	1	1	1	1	1	1	10	9
gi|37362632|	1	1	1	1	1	1	1	1	1	1	10	9
gi|37362641|	1	1	1	1	1	1	1	1	1	1	10	9
gi|37362644|	1	1	1	1	1	1	1	1	1	1	10	9
gi|37362649|	1	1	1	1	1	1	1	1	1	1	10	9

gi|37362650|	1	1	1	1	1	1	1	1	1	1	10	9
gi|37362658|	1	1	1	1	1	1	1	1	1	1	10	9
gi|37362659|	1	1	1	1	1	1	1	1	1	1	10	9
gi|37362660|	1	1	1	1	1	1	1	1	1	1	10	9
gi|37362669|	1	1	1	1	1	1	1	1	1	1	10	9
gi|37362674|	1	1	1	1	1	1	1	1	1	1	10	9
gi|37362677|	1	1	1	1	1	1	1	1	1	1	10	9
gi|37362683|	1	1	1	1	1	1	1	1	1	1	10	9
gi|37362684|	1	1	1	1	1	1	1	1	1	1	10	9
gi|37362688|	1	1	1	1	1	1	1	1	1	1	10	9
gi|37362691|	1	1	1	1	1	1	1	1	1	1	10	9
gi|37362699|	1	1	1	1	1	1	1	1	1	1	10	9
gi|37362703|	1	1	1	1	1	1	1	1	1	1	10	9
gi|41629673|	1	1	1	1	1	1	1	1	1	1	10	9
gi|41629691|	1	1	1	1	1	1	1	1	1	1	10	9
gi|41629693|	1	1	1	1	1	1	1	1	1	1	10	9
gi|42742173|	1	1	1	1	1	1	1	1	1	1	10	9
gi|42742257|	1	1	1	1	1	1	1	1	1	1	10	9
gi|42742289|	1	1	1	1	1	1	1	1	1	1	10	9
gi|42742307|	1	1	1	1	1	1	1	1	1	1	10	9
gi|42759859|	1	1	1	1	1	1	1	1	1	1	10	9
gi|42759860|	1	1	1	1	1	1	1	1	1	1	10	9
gi|42759862|	1	1	1	1	1	1	1	1	1	1	10	9
gi|46562124|	1	1	1	1	1	1	1	1	1	1	10	9
gi|50593116|	1	1	1	1	1	1	1	1	1	1	10	9
gi|50593214|	1	1	1	1	1	1	1	1	1	1	10	9
gi|50593215|	1	1	1	1	1	1	1	1	1	1	10	9
gi|50593217|	1	1	1	1	1	1	1	1	1	1	10	9
gi|52788561|	1	1	1	1	1	1	1	1	1	1	10	9
gi|52788562|	1	1	1	1	1	1	1	1	1	1	10	9
gi|52788568|	1	1	1	1	1	1	1	1	1	1	10	9
gi|52788570|	1	1	1	1	1	1	1	1	1	1	10	9
gi|6319258|	1	1	1	1	1	1	1	1	1	1	10	9
gi|6319260|	1	1	1	1	1	1	1	1	1	1	10	9
gi|6319264|	1	1	1	1	1	1	1	1	1	1	10	9
gi|6319268|	1	1	1	1	1	1	1	1	1	1	10	9
gi|6319270|	1	1	1	1	1	1	1	1	1	1	10	9
gi|6319279|	1	1	1	1	1	1	1	1	1	1	10	9
gi|6319281|	1	1	1	1	1	1	1	1	1	1	10	9
gi|6319282|	1	1	1	1	1	1	1	1	1	1	10	9
gi|6319287|	1	1	1	1	1	1	1	1	1	1	10	9
gi|6319289|	1	1	1	1	1	1	1	1	1	1	10	9
gi|6319290|	1	1	1	1	1	1	1	1	1	1	10	9
gi|6319293|	1	1	1	1	1	1	1	1	1	1	10	9
gi|6319294|	1	1	1	1	1	1	1	1	1	1	10	9
gi|6319298|	1	1	1	1	1	1	1	1	1	1	10	9
gi|6319300|	1	1	1	1	1	1	1	1	1	1	10	9
gi|6319302|	1	1	1	1	1	1	1	1	1	1	10	9
gi|6319303|	1	1	1	1	1	1	1	1	1	1	10	9
gi|6319307|	1	1	1	1	1	1	1	1	1	1	10	9
gi|6319314|	1	1	1	1	1	1	1	1	1	1	10	9
gi|6319315|	1	1	1	1	1	1	1	1	1	1	10	9
gi|6319319|	1	1	1	1	1	1	1	1	1	1	10	9
gi|6319320|	1	1	1	1	1	1	1	1	1	1	10	9
gi|6319321|	1	1	1	1	1	1	1	1	1	1	10	9
gi|6319327|	1	1	1	1	1	1	1	1	1	1	10	9
gi|6319328|	1	1	1	1	1	1	1	1	1	1	10	9
gi|6319352|	1	1	1	1	1	1	1	1	1	1	10	9
gi|6319353|	1	1	1	1	1	1	1	1	1	1	10	9
gi|6319363|	1	1	1	1	1	1	1	1	1	1	10	9
gi|6319370|	1	1	1	1	1	1	1	1	1	1	10	9
gi|6319378|	1	1	1	1	1	1	1	1	1	1	10	9
gi|6319380|	1	1	1	1	1	1	1	1	1	1	10	9
gi|6319383|	1	1	1	1	1	1	1	1	1	1	10	9
gi|6319384|	1	1	1	1	1	1	1	1	1	1	10	9
gi|6319387|	1	1	1	1	1	1	1	1	1	1	10	9
gi|6319389|	1	1	1	1	1	1	1	1	1	1	10	9
gi|6319393|	1	1	1	1	1	1	1	1	1	1	10	9
gi|6319395|	1	1	1	1	1	1	1	1	1	1	10	9
gi|6319396|	1	1	1	1	1	1	1	1	1	1	10	9

gi|6319399|	1	1	1	1	1	1	1	1	1	1	10	9
gi|6319407|	1	1	1	1	1	1	1	1	1	1	10	9
gi|6319408|	1	1	1	1	1	1	1	1	1	1	10	9
gi|6319414|	1	1	1	1	1	1	1	1	1	1	10	9
gi|6319415|	1	1	1	1	1	1	1	1	1	1	10	9
gi|6319419|	1	1	1	1	1	1	1	1	1	1	10	9
gi|6319421|	1	1	1	1	1	1	1	1	1	1	10	9
gi|6319422|	1	1	1	1	1	1	1	1	1	1	10	9
gi|6319424|	1	1	1	1	1	1	1	1	1	1	10	9
gi|6319426|	1	1	1	1	1	1	1	1	1	1	10	9
gi|6319430|	1	1	1	1	1	1	1	1	1	1	10	9
gi|6319431|	1	1	1	1	1	1	1	1	1	1	10	9
gi|6319432|	1	1	1	1	1	1	1	1	1	1	10	9
gi|6319434|	1	1	1	1	1	1	1	1	1	1	10	9
gi|6319435|	1	1	1	1	1	1	1	1	1	1	10	9
gi|6319441|	1	1	1	1	1	1	1	1	1	1	10	9
gi|6319445|	1	1	1	1	1	1	1	1	1	1	10	9
gi|6319447|	1	1	1	1	1	1	1	1	1	1	10	9
gi|6319448|	1	1	1	1	1	1	1	1	1	1	10	9
gi|6319449|	1	1	1	1	1	1	1	1	1	1	10	9
gi|6319450|	1	1	1	1	1	1	1	1	1	1	10	9
gi|6319455|	1	1	1	1	1	1	1	1	1	1	10	9
gi|6319463|	1	1	1	1	1	1	1	1	1	1	10	9
gi|6319470|	1	1	1	1	1	1	1	1	1	1	10	9
gi|6319471|	1	1	1	1	1	1	1	1	1	1	10	9
gi|6319473|	1	1	1	1	1	1	1	1	1	1	10	9
gi|6319474|	1	1	1	1	1	1	1	1	1	1	10	9
gi|6319478|	1	1	1	1	1	1	1	1	1	1	10	9
gi|6319481|	1	1	1	1	1	1	1	1	1	1	10	9
gi|6319482|	1	1	1	1	1	1	1	1	1	1	10	9
gi|6319483|	1	1	1	1	1	1	1	1	1	1	10	9
gi|6319491|	1	1	1	1	1	1	1	1	1	1	10	9
gi|6319493|	1	1	1	1	1	1	1	1	1	1	10	9
gi|6319494|	1	1	1	1	1	1	1	1	1	1	10	9
gi|6319499|	1	1	1	1	1	1	1	1	1	1	10	9
gi|6319500|	1	1	1	1	1	1	1	1	1	1	10	9
gi|6319502|	1	1	1	1	1	1	1	1	1	1	10	9
gi|6319503|	1	1	1	1	1	1	1	1	1	1	10	9
gi|6319505|	1	1	1	1	1	1	1	1	1	1	10	9
gi|6319508|	1	1	1	1	1	1	1	1	1	1	10	9
gi|6319513|	1	1	1	1	1	1	1	1	1	1	10	9
gi|6319522|	1	1	1	1	1	1	1	1	1	1	10	9
gi|6319529|	1	1	1	1	1	1	1	1	1	1	10	9
gi|6319533|	1	1	1	1	1	1	1	1	1	1	10	9
gi|6319535|	1	1	1	1	1	1	1	1	1	1	10	9
gi|6319540|	1	1	1	1	1	1	1	1	1	1	10	9
gi|6319554|	1	1	1	1	1	1	1	1	1	1	10	9
gi|6319555|	1	1	1	1	1	1	1	1	1	1	10	9
gi|6319556|	1	1	1	1	1	1	1	1	1	1	10	9
gi|6319558|	1	1	1	1	1	1	1	1	1	1	10	9
gi|6319560|	1	1	1	1	1	1	1	1	1	1	10	9
gi|6319562|	1	1	1	1	1	1	1	1	1	1	10	9
gi|6319564|	1	1	1	1	1	1	1	1	1	1	10	9
gi|6319565|	1	1	1	1	1	1	1	1	1	1	10	9
gi|6319573|	1	1	1	1	1	1	1	1	1	1	10	9
gi|6319579|	1	1	1	1	1	1	1	1	1	1	10	9
gi|6319580|	1	1	1	1	1	1	1	1	1	1	10	9
gi|6319585|	1	1	1	1	1	1	1	1	1	1	10	9
gi|6319589|	1	1	1	1	1	1	1	1	1	1	10	9
gi|6319590|	1	1	1	1	1	1	1	1	1	1	10	9
gi|6319593|	1	1	1	1	1	1	1	1	1	1	10	9
gi|6319594|	1	1	1	1	1	1	1	1	1	1	10	9
gi|6319597|	1	1	1	1	1	1	1	1	1	1	10	9
gi|6319603|	1	1	1	1	1	1	1	1	1	1	10	9
gi|6319610|	1	1	1	1	1	1	1	1	1	1	10	9
gi|6319612|	1	1	1	1	1	1	1	1	1	1	10	9
gi|6319615|	1	1	1	1	1	1	1	1	1	1	10	9
gi|6319618|	1	1	1	1	1	1	1	1	1	1	10	9
gi|6319619|	1	1	1	1	1	1	1	1	1	1	10	9
gi|6319625|	1	1	1	1	1	1	1	1	1	1	10	9

gi|6319630|	1	1	1	1	1	1	1	1	1	1	10	9
gi|6319631|	1	1	1	1	1	1	1	1	1	1	10	9
gi|6319635|	1	1	1	1	1	1	1	1	1	1	10	9
gi|6319636|	1	1	1	1	1	1	1	1	1	1	10	9
gi|6319641|	1	1	1	1	1	1	1	1	1	1	10	9
gi|6319646|	1	1	1	1	1	1	1	1	1	1	10	9
gi|6319651|	1	1	1	1	1	1	1	1	1	1	10	9
gi|6319658|	1	1	1	1	1	1	1	1	1	1	10	9
gi|6319659|	1	1	1	1	1	1	1	1	1	1	10	9
gi|6319664|	1	1	1	1	1	1	1	1	1	1	10	9
gi|6319666|	1	1	1	1	1	1	1	1	1	1	10	9
gi|6319668|	1	1	1	1	1	1	1	1	1	1	10	9
gi|6319669|	1	1	1	1	1	1	1	1	1	1	10	9
gi|6319673|	1	1	1	1	1	1	1	1	1	1	10	9
gi|6319675|	1	1	1	1	1	1	1	1	1	1	10	9
gi|6319679|	1	1	1	1	1	1	1	1	1	1	10	9
gi|6319685|	1	1	1	1	1	1	1	1	1	1	10	9
gi|6319689|	1	1	1	1	1	1	1	1	1	1	10	9
gi|6319695|	1	1	1	1	1	1	1	1	1	1	10	9
gi|6319698|	1	1	1	1	1	1	1	1	1	1	10	9
gi|6319699|	1	1	1	1	1	1	1	1	1	1	10	9
gi|6319706|	1	1	1	1	1	1	1	1	1	1	10	9
gi|6319711|	1	1	1	1	1	1	1	1	1	1	10	9
gi|6319712|	1	1	1	1	1	1	1	1	1	1	10	9
gi|6319713|	1	1	1	1	1	1	1	1	1	1	10	9
gi|6319714|	1	1	1	1	1	1	1	1	1	1	10	9
gi|6319718|	1	1	1	1	1	1	1	1	1	1	10	9
gi|6319720|	1	1	1	1	1	1	1	1	1	1	10	9
gi|6319721|	1	1	1	1	1	1	1	1	1	1	10	9
gi|6319722|	1	1	1	1	1	1	1	1	1	1	10	9
gi|6319729|	1	1	1	1	1	1	1	1	1	1	10	9
gi|6319738|	1	1	1	1	1	1	1	1	1	1	10	9
gi|6319751|	1	1	1	1	1	1	1	1	1	1	10	9
gi|6319761|	1	1	1	1	1	1	1	1	1	1	10	9
gi|6319766|	1	1	1	1	1	1	1	1	1	1	10	9
gi|6319771|	1	1	1	1	1	1	1	1	1	1	10	9
gi|6319772|	1	1	1	1	1	1	1	1	1	1	10	9
gi|6319791|	1	1	1	1	1	1	1	1	1	1	10	9
gi|6319796|	1	1	1	1	1	1	1	1	1	1	10	9
gi|6319806|	1	1	1	1	1	1	1	1	1	1	10	9
gi|6319809|	1	1	1	1	1	1	1	1	1	1	10	9
gi|6319814|	1	1	1	1	1	1	1	1	1	1	10	9
gi|6319816|	1	1	1	1	1	1	1	1	1	1	10	9
gi|6319844|	1	1	1	1	1	1	1	1	1	1	10	9
gi|6319850|	1	1	1	1	1	1	1	1	1	1	10	9
gi|6319853|	1	1	1	1	1	1	1	1	1	1	10	9
gi|6319872|	1	1	1	1	1	1	1	1	1	1	10	9
gi|6319896|	1	1	1	1	1	1	1	1	1	1	10	9
gi|6319903|	1	1	1	1	1	1	1	1	1	1	10	9
gi|6319914|	1	1	1	1	1	1	1	1	1	1	10	9
gi|6319925|	1	1	1	1	1	1	1	1	1	1	10	9
gi|6319926|	1	1	1	1	1	1	1	1	1	1	10	9
gi|6319935|	1	1	1	1	1	1	1	1	1	1	10	9
gi|6319937|	1	1	1	1	1	1	1	1	1	1	10	9
gi|6319955|	1	1	1	1	1	1	1	1	1	1	10	9
gi|6319956|	1	1	1	1	1	1	1	1	1	1	10	9
gi|6319967|	1	1	1	1	1	1	1	1	1	1	10	9
gi|6319972|	1	1	1	1	1	1	1	1	1	1	10	9
gi|6319975|	1	1	1	1	1	1	1	1	1	1	10	9
gi|6319982|	1	1	1	1	1	1	1	1	1	1	10	9
gi|6319984|	1	1	1	1	1	1	1	1	1	1	10	9
gi|6319985|	1	1	1	1	1	1	1	1	1	1	10	9
gi|6319987|	1	1	1	1	1	1	1	1	1	1	10	9
gi|6319993|	1	1	1	1	1	1	1	1	1	1	10	9
gi|6320006|	1	1	1	1	1	1	1	1	1	1	10	9
gi|6320007|	1	1	1	1	1	1	1	1	1	1	10	9
gi|6320009|	1	1	1	1	1	1	1	1	1	1	10	9
gi|6320011|	1	1	1	1	1	1	1	1	1	1	10	9
gi|6320013|	1	1	1	1	1	1	1	1	1	1	10	9
gi|6320016|	1	1	1	1	1	1	1	1	1	1	10	9

gi|6320023|	1	1	1	1	1	1	1	1	1	1	10	9
gi|6320027|	1	1	1	1	1	1	1	1	1	1	10	9
gi|6320033|	1	1	1	1	1	1	1	1	1	1	10	9
gi|6320035|	1	1	1	1	1	1	1	1	1	1	10	9
gi|6320036|	1	1	1	1	1	1	1	1	1	1	10	9
gi|6320038|	1	1	1	1	1	1	1	1	1	1	10	9
gi|6320039|	1	1	1	1	1	1	1	1	1	1	10	9
gi|6320041|	1	1	1	1	1	1	1	1	1	1	10	9
gi|6320042|	1	1	1	1	1	1	1	1	1	1	10	9
gi|6320046|	1	1	1	1	1	1	1	1	1	1	10	9
gi|6320047|	1	1	1	1	1	1	1	1	1	1	10	9
gi|6320052|	1	1	1	1	1	1	1	1	1	1	10	9
gi|6320053|	1	1	1	1	1	1	1	1	1	1	10	9
gi|6320054|	1	1	1	1	1	1	1	1	1	1	10	9
gi|6320056|	1	1	1	1	1	1	1	1	1	1	10	9
gi|6320058|	1	1	1	1	1	1	1	1	1	1	10	9
gi|6320059|	1	1	1	1	1	1	1	1	1	1	10	9
gi|6320061|	1	1	1	1	1	1	1	1	1	1	10	9
gi|6320063|	1	1	1	1	1	1	1	1	1	1	10	9
gi|6320064|	1	1	1	1	1	1	1	1	1	1	10	9
gi|6320066|	1	1	1	1	1	1	1	1	1	1	10	9
gi|6320067|	1	1	1	1	1	1	1	1	1	1	10	9
gi|6320070|	1	1	1	1	1	1	1	1	1	1	10	9
gi|6320077|	1	1	1	1	1	1	1	1	1	1	10	9
gi|6320078|	1	1	1	1	1	1	1	1	1	1	10	9
gi|6320079|	1	1	1	1	1	1	1	1	1	1	10	9
gi|6320089|	1	1	1	1	1	1	1	1	1	1	10	9
gi|6320095|	1	1	1	1	1	1	1	1	1	1	10	9
gi|6320099|	1	1	1	1	1	1	1	1	1	1	10	9
gi|6320100|	1	1	1	1	1	1	1	1	1	1	10	9
gi|6320101|	1	1	1	1	1	1	1	1	1	1	10	9
gi|6320102|	1	1	1	1	1	1	1	1	1	1	10	9
gi|6320103|	1	1	1	1	1	1	1	1	1	1	10	9
gi|6320106|	1	1	1	1	1	1	1	1	1	1	10	9
gi|6320113|	1	1	1	1	1	1	1	1	1	1	10	9
gi|6320116|	1	1	1	1	1	1	1	1	1	1	10	9
gi|6320119|	1	1	1	1	1	1	1	1	1	1	10	9
gi|6320120|	1	1	1	1	1	1	1	1	1	1	10	9
gi|6320121|	1	1	1	1	1	1	1	1	1	1	10	9
gi|6320124|	1	1	1	1	1	1	1	1	1	1	10	9
gi|6320125|	1	1	1	1	1	1	1	1	1	1	10	9
gi|6320128|	1	1	1	1	1	1	1	1	1	1	10	9
gi|6320129|	1	1	1	1	1	1	1	1	1	1	10	9
gi|6320132|	1	1	1	1	1	1	1	1	1	1	10	9
gi|6320137|	1	1	1	1	1	1	1	1	1	1	10	9
gi|6320139|	1	1	1	1	1	1	1	1	1	1	10	9
gi|6320142|	1	1	1	1	1	1	1	1	1	1	10	9
gi|6320143|	1	1	1	1	1	1	1	1	1	1	10	9
gi|6320145|	1	1	1	1	1	1	1	1	1	1	10	9
gi|6320148|	1	1	1	1	1	1	1	1	1	1	10	9
gi|6320156|	1	1	1	1	1	1	1	1	1	1	10	9
gi|6320164|	1	1	1	1	1	1	1	1	1	1	10	9
gi|6320168|	1	1	1	1	1	1	1	1	1	1	10	9
gi|6320172|	1	1	1	1	1	1	1	1	1	1	10	9
gi|6320173|	1	1	1	1	1	1	1	1	1	1	10	9
gi|6320175|	1	1	1	1	1	1	1	1	1	1	10	9
gi|6320179|	1	1	1	1	1	1	1	1	1	1	10	9
gi|6320181|	1	1	1	1	1	1	1	1	1	1	10	9
gi|6320185|	1	1	1	1	1	1	1	1	1	1	10	9
gi|6320187|	1	1	1	1	1	1	1	1	1	1	10	9
gi|6320189|	1	1	1	1	1	1	1	1	1	1	10	9
gi|6320190|	1	1	1	1	1	1	1	1	1	1	10	9
gi|6320193|	1	1	1	1	1	1	1	1	1	1	10	9
gi|6320197|	1	1	1	1	1	1	1	1	1	1	10	9
gi|6320198|	1	1	1	1	1	1	1	1	1	1	10	9
gi|6320204|	1	1	1	1	1	1	1	1	1	1	10	9
gi|6320205|	1	1	1	1	1	1	1	1	1	1	10	9
gi|6320207|	1	1	1	1	1	1	1	1	1	1	10	9
gi|6320212|	1	1	1	1	1	1	1	1	1	1	10	9
gi|6320214|	1	1	1	1	1	1	1	1	1	1	10	9

gi|6320215|	1	1	1	1	1	1	1	1	1	1	10	9
gi|6320224|	1	1	1	1	1	1	1	1	1	1	10	9
gi|6320226|	1	1	1	1	1	1	1	1	1	1	10	9
gi|6320228|	1	1	1	1	1	1	1	1	1	1	10	9
gi|6320241|	1	1	1	1	1	1	1	1	1	1	10	9
gi|6320242|	1	1	1	1	1	1	1	1	1	1	10	9
gi|6320243|	1	1	1	1	1	1	1	1	1	1	10	9
gi|6320244|	1	1	1	1	1	1	1	1	1	1	10	9
gi|6320245|	1	1	1	1	1	1	1	1	1	1	10	9
gi|6320248|	1	1	1	1	1	1	1	1	1	1	10	9
gi|6320250|	1	1	1	1	1	1	1	1	1	1	10	9
gi|6320255|	1	1	1	1	1	1	1	1	1	1	10	9
gi|6320259|	1	1	1	1	1	1	1	1	1	1	10	9
gi|6320264|	1	1	1	1	1	1	1	1	1	1	10	9
gi|6320265|	1	1	1	1	1	1	1	1	1	1	10	9
gi|6320267|	1	1	1	1	1	1	1	1	1	1	10	9
gi|6320269|	1	1	1	1	1	1	1	1	1	1	10	9
gi|6320274|	1	1	1	1	1	1	1	1	1	1	10	9
gi|6320280|	1	1	1	1	1	1	1	1	1	1	10	9
gi|6320289|	1	1	1	1	1	1	1	1	1	1	10	9
gi|6320296|	1	1	1	1	1	1	1	1	1	1	10	9
gi|6320297|	1	1	1	1	1	1	1	1	1	1	10	9
gi|6320298|	1	1	1	1	1	1	1	1	1	1	10	9
gi|6320301|	1	1	1	1	1	1	1	1	1	1	10	9
gi|6320302|	1	1	1	1	1	1	1	1	1	1	10	9
gi|6320303|	1	1	1	1	1	1	1	1	1	1	10	9
gi|6320304|	1	1	1	1	1	1	1	1	1	1	10	9
gi|6320312|	1	1	1	1	1	1	1	1	1	1	10	9
gi|6320317|	1	1	1	1	1	1	1	1	1	1	10	9
gi|6320325|	1	1	1	1	1	1	1	1	1	1	10	9
gi|6320327|	1	1	1	1	1	1	1	1	1	1	10	9
gi|6320331|	1	1	1	1	1	1	1	1	1	1	10	9
gi|6320334|	1	1	1	1	1	1	1	1	1	1	10	9
gi|6320339|	1	1	1	1	1	1	1	1	1	1	10	9
gi|6320350|	1	1	1	1	1	1	1	1	1	1	10	9
gi|6320352|	1	1	1	1	1	1	1	1	1	1	10	9
gi|6320355|	1	1	1	1	1	1	1	1	1	1	10	9
gi|6320359|	1	1	1	1	1	1	1	1	1	1	10	9
gi|6320363|	1	1	1	1	1	1	1	1	1	1	10	9
gi|6320374|	1	1	1	1	1	1	1	1	1	1	10	9
gi|6320377|	1	1	1	1	1	1	1	1	1	1	10	9
gi|6320382|	1	1	1	1	1	1	1	1	1	1	10	9
gi|6320389|	1	1	1	1	1	1	1	1	1	1	10	9
gi|6320391|	1	1	1	1	1	1	1	1	1	1	10	9
gi|6320393|	1	1	1	1	1	1	1	1	1	1	10	9
gi|6320395|	1	1	1	1	1	1	1	1	1	1	10	9
gi|6320396|	1	1	1	1	1	1	1	1	1	1	10	9
gi|6320400|	1	1	1	1	1	1	1	1	1	1	10	9
gi|6320402|	1	1	1	1	1	1	1	1	1	1	10	9
gi|6320410|	1	1	1	1	1	1	1	1	1	1	10	9
gi|6320414|	1	1	1	1	1	1	1	1	1	1	10	9
gi|6320417|	1	1	1	1	1	1	1	1	1	1	10	9
gi|6320418|	1	1	1	1	1	1	1	1	1	1	10	9
gi|6320430|	1	1	1	1	1	1	1	1	1	1	10	9
gi|6320431|	1	1	1	1	1	1	1	1	1	1	10	9
gi|6320432|	1	1	1	1	1	1	1	1	1	1	10	9
gi|6320438|	1	1	1	1	1	1	1	1	1	1	10	9
gi|6320440|	1	1	1	1	1	1	1	1	1	1	10	9
gi|6320444|	1	1	1	1	1	1	1	1	1	1	10	9
gi|6320448|	1	1	1	1	1	1	1	1	1	1	10	9
gi|6320449|	1	1	1	1	1	1	1	1	1	1	10	9
gi|6320450|	1	1	1	1	1	1	1	1	1	1	10	9
gi|6320453|	1	1	1	1	1	1	1	1	1	1	10	9
gi|6320461|	1	1	1	1	1	1	1	1	1	1	10	9
gi|6320462|	1	1	1	1	1	1	1	1	1	1	10	9
gi|6320469|	1	1	1	1	1	1	1	1	1	1	10	9
gi|6320470|	1	1	1	1	1	1	1	1	1	1	10	9
gi|6320473|	1	1	1	1	1	1	1	1	1	1	10	9
gi|6320474|	1	1	1	1	1	1	1	1	1	1	10	9
gi|6320475|	1	1	1	1	1	1	1	1	1	1	10	9

gi|6320478|	1	1	1	1	1	1	1	1	1	1	10	9
gi|6320486|	1	1	1	1	1	1	1	1	1	1	10	9
gi|6320489|	1	1	1	1	1	1	1	1	1	1	10	9
gi|6320490|	1	1	1	1	1	1	1	1	1	1	10	9
gi|6320498|	1	1	1	1	1	1	1	1	1	1	10	9
gi|6320499|	1	1	1	1	1	1	1	1	1	1	10	9
gi|6320503|	1	1	1	1	1	1	1	1	1	1	10	9
gi|6320504|	1	1	1	1	1	1	1	1	1	1	10	9
gi|6320506|	1	1	1	1	1	1	1	1	1	1	10	9
gi|6320510|	1	1	1	1	1	1	1	1	1	1	10	9
gi|6320511|	1	1	1	1	1	1	1	1	1	1	10	9
gi|6320518|	1	1	1	1	1	1	1	1	1	1	10	9
gi|6320519|	1	1	1	1	1	1	1	1	1	1	10	9
gi|6320535|	1	1	1	1	1	1	1	1	1	1	10	9
gi|6320538|	1	1	1	1	1	1	1	1	1	1	10	9
gi|6320541|	1	1	1	1	1	1	1	1	1	1	10	9
gi|6320546|	1	1	1	1	1	1	1	1	1	1	10	9
gi|6320548|	1	1	1	1	1	1	1	1	1	1	10	9
gi|6320549|	1	1	1	1	1	1	1	1	1	1	10	9
gi|6320550|	1	1	1	1	1	1	1	1	1	1	10	9
gi|6320552|	1	1	1	1	1	1	1	1	1	1	10	9
gi|6320572|	1	1	1	1	1	1	1	1	1	1	10	9
gi|6320576|	1	1	1	1	1	1	1	1	1	1	10	9
gi|6320583|	1	1	1	1	1	1	1	1	1	1	10	9
gi|6320584|	1	1	1	1	1	1	1	1	1	1	10	9
gi|6320593|	1	1	1	1	1	1	1	1	1	1	10	9
gi|6320594|	1	1	1	1	1	1	1	1	1	1	10	9
gi|6320595|	1	1	1	1	1	1	1	1	1	1	10	9
gi|6320598|	1	1	1	1	1	1	1	1	1	1	10	9
gi|6320602|	1	1	1	1	1	1	1	1	1	1	10	9
gi|6320612|	1	1	1	1	1	1	1	1	1	1	10	9
gi|6320614|	1	1	1	1	1	1	1	1	1	1	10	9
gi|6320617|	1	1	1	1	1	1	1	1	1	1	10	9
gi|6320618|	1	1	1	1	1	1	1	1	1	1	10	9
gi|6320624|	1	1	1	1	1	1	1	1	1	1	10	9
gi|6320625|	1	1	1	1	1	1	1	1	1	1	10	9
gi|6320632|	1	1	1	1	1	1	1	1	1	1	10	9
gi|6320635|	1	1	1	1	1	1	1	1	1	1	10	9
gi|6320639|	1	1	1	1	1	1	1	1	1	1	10	9
gi|6320644|	1	1	1	1	1	1	1	1	1	1	10	9
gi|6320649|	1	1	1	1	1	1	1	1	1	1	10	9
gi|6320655|	1	1	1	1	1	1	1	1	1	1	10	9
gi|6320658|	1	1	1	1	1	1	1	1	1	1	10	9
gi|6320661|	1	1	1	1	1	1	1	1	1	1	10	9
gi|6320662|	1	1	1	1	1	1	1	1	1	1	10	9
gi|6320663|	1	1	1	1	1	1	1	1	1	1	10	9
gi|6320665|	1	1	1	1	1	1	1	1	1	1	10	9
gi|6320667|	1	1	1	1	1	1	1	1	1	1	10	9
gi|6320668|	1	1	1	1	1	1	1	1	1	1	10	9
gi|6320674|	1	1	1	1	1	1	1	1	1	1	10	9
gi|6320679|	1	1	1	1	1	1	1	1	1	1	10	9
gi|6320685|	1	1	1	1	1	1	1	1	1	1	10	9
gi|6320698|	1	1	1	1	1	1	1	1	1	1	10	9
gi|6320699|	1	1	1	1	1	1	1	1	1	1	10	9
gi|6320704|	1	1	1	1	1	1	1	1	1	1	10	9
gi|6320705|	1	1	1	1	1	1	1	1	1	1	10	9
gi|6320708|	1	1	1	1	1	1	1	1	1	1	10	9
gi|6320710|	1	1	1	1	1	1	1	1	1	1	10	9
gi|6320715|	1	1	1	1	1	1	1	1	1	1	10	9
gi|6320718|	1	1	1	1	1	1	1	1	1	1	10	9
gi|6320720|	1	1	1	1	1	1	1	1	1	1	10	9
gi|6320724|	1	1	1	1	1	1	1	1	1	1	10	9
gi|6320726|	1	1	1	1	1	1	1	1	1	1	10	9
gi|6320727|	1	1	1	1	1	1	1	1	1	1	10	9
gi|6320731|	1	1	1	1	1	1	1	1	1	1	10	9
gi|6320732|	1	1	1	1	1	1	1	1	1	1	10	9
gi|6320740|	1	1	1	1	1	1	1	1	1	1	10	9
gi|6320745|	1	1	1	1	1	1	1	1	1	1	10	9
gi|6320764|	1	1	1	1	1	1	1	1	1	1	10	9
gi|6320766|	1	1	1	1	1	1	1	1	1	1	10	9

gi|6320777|	1	1	1	1	1	1	1	1	1	1	10	9
gi|6320779|	1	1	1	1	1	1	1	1	1	1	10	9
gi|6320781|	1	1	1	1	1	1	1	1	1	1	10	9
gi|6320783|	1	1	1	1	1	1	1	1	1	1	10	9
gi|6320784|	1	1	1	1	1	1	1	1	1	1	10	9
gi|6320793|	1	1	1	1	1	1	1	1	1	1	10	9
gi|6320796|	1	1	1	1	1	1	1	1	1	1	10	9
gi|6320801|	1	1	1	1	1	1	1	1	1	1	10	9
gi|6320803|	1	1	1	1	1	1	1	1	1	1	10	9
gi|6320804|	1	1	1	1	1	1	1	1	1	1	10	9
gi|6320805|	1	1	1	1	1	1	1	1	1	1	10	9
gi|6320806|	1	1	1	1	1	1	1	1	1	1	10	9
gi|6320808|	1	1	1	1	1	1	1	1	1	1	10	9
gi|6320809|	1	1	1	1	1	1	1	1	1	1	10	9
gi|6320811|	1	1	1	1	1	1	1	1	1	1	10	9
gi|6320813|	1	1	1	1	1	1	1	1	1	1	10	9
gi|6320814|	1	1	1	1	1	1	1	1	1	1	10	9
gi|6320816|	1	1	1	1	1	1	1	1	1	1	10	9
gi|6320831|	1	1	1	1	1	1	1	1	1	1	10	9
gi|6320839|	1	1	1	1	1	1	1	1	1	1	10	9
gi|6320841|	1	1	1	1	1	1	1	1	1	1	10	9
gi|6320842|	1	1	1	1	1	1	1	1	1	1	10	9
gi|6320844|	1	1	1	1	1	1	1	1	1	1	10	9
gi|6320846|	1	1	1	1	1	1	1	1	1	1	10	9
gi|6320849|	1	1	1	1	1	1	1	1	1	1	10	9
gi|6320850|	1	1	1	1	1	1	1	1	1	1	10	9
gi|6320852|	1	1	1	1	1	1	1	1	1	1	10	9
gi|6320853|	1	1	1	1	1	1	1	1	1	1	10	9
gi|6320854|	1	1	1	1	1	1	1	1	1	1	10	9
gi|6320858|	1	1	1	1	1	1	1	1	1	1	10	9
gi|6320861|	1	1	1	1	1	1	1	1	1	1	10	9
gi|6320863|	1	1	1	1	1	1	1	1	1	1	10	9
gi|6320865|	1	1	1	1	1	1	1	1	1	1	10	9
gi|6320867|	1	1	1	1	1	1	1	1	1	1	10	9
gi|6320869|	1	1	1	1	1	1	1	1	1	1	10	9
gi|6320874|	1	1	1	1	1	1	1	1	1	1	10	9
gi|6320882|	1	1	1	1	1	1	1	1	1	1	10	9
gi|6320887|	1	1	1	1	1	1	1	1	1	1	10	9
gi|6320888|	1	1	1	1	1	1	1	1	1	1	10	9
gi|6320894|	1	1	1	1	1	1	1	1	1	1	10	9
gi|6320904|	1	1	1	1	1	1	1	1	1	1	10	9
gi|6320912|	1	1	1	1	1	1	1	1	1	1	10	9
gi|6320914|	1	1	1	1	1	1	1	1	1	1	10	9
gi|6320917|	1	1	1	1	1	1	1	1	1	1	10	9
gi|6320918|	1	1	1	1	1	1	1	1	1	1	10	9
gi|6320922|	1	1	1	1	1	1	1	1	1	1	10	9
gi|6320925|	1	1	1	1	1	1	1	1	1	1	10	9
gi|6320926|	1	1	1	1	1	1	1	1	1	1	10	9
gi|6320930|	1	1	1	1	1	1	1	1	1	1	10	9
gi|6320934|	1	1	1	1	1	1	1	1	1	1	10	9
gi|6320941|	1	1	1	1	1	1	1	1	1	1	10	9
gi|6320942|	1	1	1	1	1	1	1	1	1	1	10	9
gi|6320945|	1	1	1	1	1	1	1	1	1	1	10	9
gi|6320946|	1	1	1	1	1	1	1	1	1	1	10	9
gi|6320947|	1	1	1	1	1	1	1	1	1	1	10	9
gi|6320949|	1	1	1	1	1	1	1	1	1	1	10	9
gi|6320950|	1	1	1	1	1	1	1	1	1	1	10	9
gi|6320954|	1	1	1	1	1	1	1	1	1	1	10	9
gi|6320963|	1	1	1	1	1	1	1	1	1	1	10	9
gi|6320966|	1	1	1	1	1	1	1	1	1	1	10	9
gi|6320969|	1	1	1	1	1	1	1	1	1	1	10	9
gi|6320972|	1	1	1	1	1	1	1	1	1	1	10	9
gi|6320973|	1	1	1	1	1	1	1	1	1	1	10	9
gi|6320976|	1	1	1	1	1	1	1	1	1	1	10	9
gi|6320978|	1	1	1	1	1	1	1	1	1	1	10	9
gi|6320980|	1	1	1	1	1	1	1	1	1	1	10	9
gi|6320983|	1	1	1	1	1	1	1	1	1	1	10	9
gi|6320988|	1	1	1	1	1	1	1	1	1	1	10	9
gi|6320989|	1	1	1	1	1	1	1	1	1	1	10	9
gi|6320992|	1	1	1	1	1	1	1	1	1	1	10	9

gi|6320994|	1	1	1	1	1	1	1	1	1	1	10	9
gi|6320996|	1	1	1	1	1	1	1	1	1	1	10	9
gi|6321002|	1	1	1	1	1	1	1	1	1	1	10	9
gi|6321004|	1	1	1	1	1	1	1	1	1	1	10	9
gi|6321007|	1	1	1	1	1	1	1	1	1	1	10	9
gi|6321011|	1	1	1	1	1	1	1	1	1	1	10	9
gi|6321012|	1	1	1	1	1	1	1	1	1	1	10	9
gi|6321013|	1	1	1	1	1	1	1	1	1	1	10	9
gi|6321014|	1	1	1	1	1	1	1	1	1	1	10	9
gi|6321017|	1	1	1	1	1	1	1	1	1	1	10	9
gi|6321018|	1	1	1	1	1	1	1	1	1	1	10	9
gi|6321019|	1	1	1	1	1	1	1	1	1	1	10	9
gi|6321020|	1	1	1	1	1	1	1	1	1	1	10	9
gi|6321022|	1	1	1	1	1	1	1	1	1	1	10	9
gi|6321024|	1	1	1	1	1	1	1	1	1	1	10	9
gi|6321025|	1	1	1	1	1	1	1	1	1	1	10	9
gi|6321027|	1	1	1	1	1	1	1	1	1	1	10	9
gi|6321183|	1	1	1	1	1	1	1	1	1	1	10	9
gi|6321184|	1	1	1	1	1	1	1	1	1	1	10	9
gi|6321198|	1	1	1	1	1	1	1	1	1	1	10	9
gi|6321200|	1	1	1	1	1	1	1	1	1	1	10	9
gi|6321203|	1	1	1	1	1	1	1	1	1	1	10	9
gi|6321210|	1	1	1	1	1	1	1	1	1	1	10	9
gi|6321218|	1	1	1	1	1	1	1	1	1	1	10	9
gi|6321221|	1	1	1	1	1	1	1	1	1	1	10	9
gi|6321228|	1	1	1	1	1	1	1	1	1	1	10	9
gi|6321230|	1	1	1	1	1	1	1	1	1	1	10	9
gi|6321231|	1	1	1	1	1	1	1	1	1	1	10	9
gi|6321232|	1	1	1	1	1	1	1	1	1	1	10	9
gi|6321235|	1	1	1	1	1	1	1	1	1	1	10	9
gi|6321238|	1	1	1	1	1	1	1	1	1	1	10	9
gi|6321244|	1	1	1	1	1	1	1	1	1	1	10	9
gi|6321248|	1	1	1	1	1	1	1	1	1	1	10	9
gi|6321249|	1	1	1	1	1	1	1	1	1	1	10	9
gi|6321253|	1	1	1	1	1	1	1	1	1	1	10	9
gi|6321254|	1	1	1	1	1	1	1	1	1	1	10	9
gi|6321258|	1	1	1	1	1	1	1	1	1	1	10	9
gi|6321259|	1	1	1	1	1	1	1	1	1	1	10	9
gi|6321260|	1	1	1	1	1	1	1	1	1	1	10	9
gi|6321265|	1	1	1	1	1	1	1	1	1	1	10	9
gi|6321267|	1	1	1	1	1	1	1	1	1	1	10	9
gi|6321271|	1	1	1	1	1	1	1	1	1	1	10	9
gi|6321275|	1	1	1	1	1	1	1	1	1	1	10	9
gi|6321280|	1	1	1	1	1	1	1	1	1	1	10	9
gi|6321289|	1	1	1	1	1	1	1	1	1	1	10	9
gi|6321291|	1	1	1	1	1	1	1	1	1	1	10	9
gi|6321295|	1	1	1	1	1	1	1	1	1	1	10	9
gi|6321301|	1	1	1	1	1	1	1	1	1	1	10	9
gi|6321302|	1	1	1	1	1	1	1	1	1	1	10	9
gi|6321303|	1	1	1	1	1	1	1	1	1	1	10	9
gi|6321311|	1	1	1	1	1	1	1	1	1	1	10	9
gi|6321315|	1	1	1	1	1	1	1	1	1	1	10	9
gi|6321318|	1	1	1	1	1	1	1	1	1	1	10	9
gi|6321319|	1	1	1	1	1	1	1	1	1	1	10	9
gi|6321322|	1	1	1	1	1	1	1	1	1	1	10	9
gi|6321332|	1	1	1	1	1	1	1	1	1	1	10	9
gi|6321333|	1	1	1	1	1	1	1	1	1	1	10	9
gi|6321335|	1	1	1	1	1	1	1	1	1	1	10	9
gi|6321338|	1	1	1	1	1	1	1	1	1	1	10	9
gi|6321339|	1	1	1	1	1	1	1	1	1	1	10	9
gi|6321341|	1	1	1	1	1	1	1	1	1	1	10	9
gi|6321343|	1	1	1	1	1	1	1	1	1	1	10	9
gi|6321344|	1	1	1	1	1	1	1	1	1	1	10	9
gi|6321347|	1	1	1	1	1	1	1	1	1	1	10	9
gi|6321351|	1	1	1	1	1	1	1	1	1	1	10	9
gi|6321358|	1	1	1	1	1	1	1	1	1	1	10	9
gi|6321360|	1	1	1	1	1	1	1	1	1	1	10	9
gi|6321362|	1	1	1	1	1	1	1	1	1	1	10	9
gi|6321364|	1	1	1	1	1	1	1	1	1	1	10	9
gi|6321368|	1	1	1	1	1	1	1	1	1	1	10	9

gi|6321369|	1	1	1	1	1	1	1	1	1	1	10	9
gi|6321373|	1	1	1	1	1	1	1	1	1	1	10	9
gi|6321374|	1	1	1	1	1	1	1	1	1	1	10	9
gi|6321375|	1	1	1	1	1	1	1	1	1	1	10	9
gi|6321376|	1	1	1	1	1	1	1	1	1	1	10	9
gi|6321380|	1	1	1	1	1	1	1	1	1	1	10	9
gi|6321384|	1	1	1	1	1	1	1	1	1	1	10	9
gi|6321390|	1	1	1	1	1	1	1	1	1	1	10	9
gi|6321394|	1	1	1	1	1	1	1	1	1	1	10	9
gi|6321395|	1	1	1	1	1	1	1	1	1	1	10	9
gi|6321407|	1	1	1	1	1	1	1	1	1	1	10	9
gi|6321408|	1	1	1	1	1	1	1	1	1	1	10	9
gi|6321416|	1	1	1	1	1	1	1	1	1	1	10	9
gi|6321419|	1	1	1	1	1	1	1	1	1	1	10	9
gi|6321421|	1	1	1	1	1	1	1	1	1	1	10	9
gi|6321424|	1	1	1	1	1	1	1	1	1	1	10	9
gi|6321427|	1	1	1	1	1	1	1	1	1	1	10	9
gi|6321430|	1	1	1	1	1	1	1	1	1	1	10	9
gi|6321432|	1	1	1	1	1	1	1	1	1	1	10	9
gi|6321434|	1	1	1	1	1	1	1	1	1	1	10	9
gi|6321435|	1	1	1	1	1	1	1	1	1	1	10	9
gi|6321436|	1	1	1	1	1	1	1	1	1	1	10	9
gi|6321440|	1	1	1	1	1	1	1	1	1	1	10	9
gi|6321444|	1	1	1	1	1	1	1	1	1	1	10	9
gi|6321447|	1	1	1	1	1	1	1	1	1	1	10	9
gi|6321449|	1	1	1	1	1	1	1	1	1	1	10	9
gi|6321456|	1	1	1	1	1	1	1	1	1	1	10	9
gi|6321457|	1	1	1	1	1	1	1	1	1	1	10	9
gi|6321464|	1	1	1	1	1	1	1	1	1	1	10	9
gi|6321465|	1	1	1	1	1	1	1	1	1	1	10	9
gi|6321471|	1	1	1	1	1	1	1	1	1	1	10	9
gi|6321474|	1	1	1	1	1	1	1	1	1	1	10	9
gi|6321477|	1	1	1	1	1	1	1	1	1	1	10	9
gi|6321480|	1	1	1	1	1	1	1	1	1	1	10	9
gi|6321485|	1	1	1	1	1	1	1	1	1	1	10	9
gi|6321489|	1	1	1	1	1	1	1	1	1	1	10	9
gi|6321497|	1	1	1	1	1	1	1	1	1	1	10	9
gi|6321498|	1	1	1	1	1	1	1	1	1	1	10	9
gi|6321501|	1	1	1	1	1	1	1	1	1	1	10	9
gi|6321510|	1	1	1	1	1	1	1	1	1	1	10	9
gi|6321520|	1	1	1	1	1	1	1	1	1	1	10	9
gi|6321522|	1	1	1	1	1	1	1	1	1	1	10	9
gi|6321525|	1	1	1	1	1	1	1	1	1	1	10	9
gi|6321528|	1	1	1	1	1	1	1	1	1	1	10	9
gi|6321529|	1	1	1	1	1	1	1	1	1	1	10	9
gi|6321531|	1	1	1	1	1	1	1	1	1	1	10	9
gi|6321537|	1	1	1	1	1	1	1	1	1	1	10	9
gi|6321538|	1	1	1	1	1	1	1	1	1	1	10	9
gi|6321540|	1	1	1	1	1	1	1	1	1	1	10	9
gi|6321545|	1	1	1	1	1	1	1	1	1	1	10	9
gi|6321546|	1	1	1	1	1	1	1	1	1	1	10	9
gi|6321552|	1	1	1	1	1	1	1	1	1	1	10	9
gi|6321556|	1	1	1	1	1	1	1	1	1	1	10	9
gi|6321562|	1	1	1	1	1	1	1	1	1	1	10	9
gi|6321563|	1	1	1	1	1	1	1	1	1	1	10	9
gi|6321571|	1	1	1	1	1	1	1	1	1	1	10	9
gi|6321572|	1	1	1	1	1	1	1	1	1	1	10	9
gi|6321574|	1	1	1	1	1	1	1	1	1	1	10	9
gi|6321584|	1	1	1	1	1	1	1	1	1	1	10	9
gi|6321587|	1	1	1	1	1	1	1	1	1	1	10	9
gi|6321591|	1	1	1	1	1	1	1	1	1	1	10	9
gi|6321594|	1	1	1	1	1	1	1	1	1	1	10	9
gi|6321599|	1	1	1	1	1	1	1	1	1	1	10	9
gi|6321611|	1	1	1	1	1	1	1	1	1	1	10	9
gi|6321612|	1	1	1	1	1	1	1	1	1	1	10	9
gi|6321619|	1	1	1	1	1	1	1	1	1	1	10	9
gi|6321623|	1	1	1	1	1	1	1	1	1	1	10	9
gi|6321624|	1	1	1	1	1	1	1	1	1	1	10	9
gi|6321631|	1	1	1	1	1	1	1	1	1	1	10	9
gi|6321632|	1	1	1	1	1	1	1	1	1	1	10	9

gi|6321633|	1	1	1	1	1	1	1	1	1	1	10	9
gi|6321634|	1	1	1	1	1	1	1	1	1	1	10	9
gi|6321639|	1	1	1	1	1	1	1	1	1	1	10	9
gi|6321641|	1	1	1	1	1	1	1	1	1	1	10	9
gi|6321643|	1	1	1	1	1	1	1	1	1	1	10	9
gi|6321646|	1	1	1	1	1	1	1	1	1	1	10	9
gi|6321647|	1	1	1	1	1	1	1	1	1	1	10	9
gi|6321648|	1	1	1	1	1	1	1	1	1	1	10	9
gi|6321650|	1	1	1	1	1	1	1	1	1	1	10	9
gi|6321653|	1	1	1	1	1	1	1	1	1	1	10	9
gi|6321657|	1	1	1	1	1	1	1	1	1	1	10	9
gi|6321659|	1	1	1	1	1	1	1	1	1	1	10	9
gi|6321662|	1	1	1	1	1	1	1	1	1	1	10	9
gi|6321682|	1	1	1	1	1	1	1	1	1	1	10	9
gi|6321683|	1	1	1	1	1	1	1	1	1	1	10	9
gi|6321684|	1	1	1	1	1	1	1	1	1	1	10	9
gi|6321685|	1	1	1	1	1	1	1	1	1	1	10	9
gi|6321689|	1	1	1	1	1	1	1	1	1	1	10	9
gi|6321691|	1	1	1	1	1	1	1	1	1	1	10	9
gi|6321692|	1	1	1	1	1	1	1	1	1	1	10	9
gi|6321693|	1	1	1	1	1	1	1	1	1	1	10	9
gi|6321694|	1	1	1	1	1	1	1	1	1	1	10	9
gi|6321695|	1	1	1	1	1	1	1	1	1	1	10	9
gi|6321696|	1	1	1	1	1	1	1	1	1	1	10	9
gi|6321697|	1	1	1	1	1	1	1	1	1	1	10	9
gi|6321700|	1	1	1	1	1	1	1	1	1	1	10	9
gi|6321704|	1	1	1	1	1	1	1	1	1	1	10	9
gi|6321706|	1	1	1	1	1	1	1	1	1	1	10	9
gi|6321709|	1	1	1	1	1	1	1	1	1	1	10	9
gi|6321710|	1	1	1	1	1	1	1	1	1	1	10	9
gi|6321715|	1	1	1	1	1	1	1	1	1	1	10	9
gi|6321716|	1	1	1	1	1	1	1	1	1	1	10	9
gi|6321717|	1	1	1	1	1	1	1	1	1	1	10	9
gi|6321720|	1	1	1	1	1	1	1	1	1	1	10	9
gi|6321752|	1	1	1	1	1	1	1	1	1	1	10	9
gi|6321754|	1	1	1	1	1	1	1	1	1	1	10	9
gi|6321768|	1	1	1	1	1	1	1	1	1	1	10	9
gi|6321772|	1	1	1	1	1	1	1	1	1	1	10	9
gi|6321773|	1	1	1	1	1	1	1	1	1	1	10	9
gi|6321775|	1	1	1	1	1	1	1	1	1	1	10	9
gi|6321776|	1	1	1	1	1	1	1	1	1	1	10	9
gi|6321777|	1	1	1	1	1	1	1	1	1	1	10	9
gi|6321780|	1	1	1	1	1	1	1	1	1	1	10	9
gi|6321786|	1	1	1	1	1	1	1	1	1	1	10	9
gi|6321787|	1	1	1	1	1	1	1	1	1	1	10	9
gi|6321791|	1	1	1	1	1	1	1	1	1	1	10	9
gi|6321792|	1	1	1	1	1	1	1	1	1	1	10	9
gi|6321793|	1	1	1	1	1	1	1	1	1	1	10	9
gi|6321795|	1	1	1	1	1	1	1	1	1	1	10	9
gi|6321796|	1	1	1	1	1	1	1	1	1	1	10	9
gi|6321798|	1	1	1	1	1	1	1	1	1	1	10	9
gi|6321799|	1	1	1	1	1	1	1	1	1	1	10	9
gi|6321800|	1	1	1	1	1	1	1	1	1	1	10	9
gi|6321801|	1	1	1	1	1	1	1	1	1	1	10	9
gi|6321803|	1	1	1	1	1	1	1	1	1	1	10	9
gi|6321807|	1	1	1	1	1	1	1	1	1	1	10	9
gi|6321808|	1	1	1	1	1	1	1	1	1	1	10	9
gi|6321809|	1	1	1	1	1	1	1	1	1	1	10	9
gi|6321812|	1	1	1	1	1	1	1	1	1	1	10	9
gi|6321813|	1	1	1	1	1	1	1	1	1	1	10	9
gi|6321815|	1	1	1	1	1	1	1	1	1	1	10	9
gi|6321816|	1	1	1	1	1	1	1	1	1	1	10	9
gi|6321817|	1	1	1	1	1	1	1	1	1	1	10	9
gi|6321819|	1	1	1	1	1	1	1	1	1	1	10	9
gi|6321822|	1	1	1	1	1	1	1	1	1	1	10	9
gi|6321826|	1	1	1	1	1	1	1	1	1	1	10	9
gi|6321828|	1	1	1	1	1	1	1	1	1	1	10	9
gi|6321829|	1	1	1	1	1	1	1	1	1	1	10	9
gi|6321832|	1	1	1	1	1	1	1	1	1	1	10	9
gi|6321837|	1	1	1	1	1	1	1	1	1	1	10	9

gi|6321848|	1	1	1	1	1	1	1	1	1	1	10	9
gi|6321857|	1	1	1	1	1	1	1	1	1	1	10	9
gi|6321859|	1	1	1	1	1	1	1	1	1	1	10	9
gi|6321864|	1	1	1	1	1	1	1	1	1	1	10	9
gi|6321865|	1	1	1	1	1	1	1	1	1	1	10	9
gi|6321866|	1	1	1	1	1	1	1	1	1	1	10	9
gi|6321870|	1	1	1	1	1	1	1	1	1	1	10	9
gi|6321874|	1	1	1	1	1	1	1	1	1	1	10	9
gi|6321880|	1	1	1	1	1	1	1	1	1	1	10	9
gi|6321882|	1	1	1	1	1	1	1	1	1	1	10	9
gi|6321883|	1	1	1	1	1	1	1	1	1	1	10	9
gi|6321884|	1	1	1	1	1	1	1	1	1	1	10	9
gi|6321886|	1	1	1	1	1	1	1	1	1	1	10	9
gi|6321888|	1	1	1	1	1	1	1	1	1	1	10	9
gi|6321890|	1	1	1	1	1	1	1	1	1	1	10	9
gi|6321894|	1	1	1	1	1	1	1	1	1	1	10	9
gi|6321896|	1	1	1	1	1	1	1	1	1	1	10	9
gi|6321900|	1	1	1	1	1	1	1	1	1	1	10	9
gi|6321902|	1	1	1	1	1	1	1	1	1	1	10	9
gi|6321903|	1	1	1	1	1	1	1	1	1	1	10	9
gi|6321904|	1	1	1	1	1	1	1	1	1	1	10	9
gi|6321911|	1	1	1	1	1	1	1	1	1	1	10	9
gi|6321912|	1	1	1	1	1	1	1	1	1	1	10	9
gi|6321914|	1	1	1	1	1	1	1	1	1	1	10	9
gi|6321915|	1	1	1	1	1	1	1	1	1	1	10	9
gi|6321921|	1	1	1	1	1	1	1	1	1	1	10	9
gi|6321927|	1	1	1	1	1	1	1	1	1	1	10	9
gi|6321934|	1	1	1	1	1	1	1	1	1	1	10	9
gi|6321942|	1	1	1	1	1	1	1	1	1	1	10	9
gi|6321955|	1	1	1	1	1	1	1	1	1	1	10	9
gi|6321956|	1	1	1	1	1	1	1	1	1	1	10	9
gi|6321957|	1	1	1	1	1	1	1	1	1	1	10	9
gi|6321958|	1	1	1	1	1	1	1	1	1	1	10	9
gi|6321960|	1	1	1	1	1	1	1	1	1	1	10	9
gi|6321962|	1	1	1	1	1	1	1	1	1	1	10	9
gi|6321963|	1	1	1	1	1	1	1	1	1	1	10	9
gi|6321964|	1	1	1	1	1	1	1	1	1	1	10	9
gi|6321965|	1	1	1	1	1	1	1	1	1	1	10	9
gi|6321968|	1	1	1	1	1	1	1	1	1	1	10	9
gi|6321977|	1	1	1	1	1	1	1	1	1	1	10	9
gi|6321980|	1	1	1	1	1	1	1	1	1	1	10	9
gi|6321987|	1	1	1	1	1	1	1	1	1	1	10	9
gi|6321997|	1	1	1	1	1	1	1	1	1	1	10	9
gi|6321998|	1	1	1	1	1	1	1	1	1	1	10	9
gi|6321999|	1	1	1	1	1	1	1	1	1	1	10	9
gi|6322000|	1	1	1	1	1	1	1	1	1	1	10	9
gi|6322002|	1	1	1	1	1	1	1	1	1	1	10	9
gi|6322012|	1	1	1	1	1	1	1	1	1	1	10	9
gi|6322031|	1	1	1	1	1	1	1	1	1	1	10	9
gi|6322036|	1	1	1	1	1	1	1	1	1	1	10	9
gi|6322038|	1	1	1	1	1	1	1	1	1	1	10	9
gi|6322043|	1	1	1	1	1	1	1	1	1	1	10	9
gi|6322048|	1	1	1	1	1	1	1	1	1	1	10	9
gi|6322049|	1	1	1	1	1	1	1	1	1	1	10	9
gi|6322054|	1	1	1	1	1	1	1	1	1	1	10	9
gi|6322058|	1	1	1	1	1	1	1	1	1	1	10	9
gi|6322065|	1	1	1	1	1	1	1	1	1	1	10	9
gi|6322066|	1	1	1	1	1	1	1	1	1	1	10	9
gi|6322067|	1	1	1	1	1	1	1	1	1	1	10	9
gi|6322073|	1	1	1	1	1	1	1	1	1	1	10	9
gi|6322079|	1	1	1	1	1	1	1	1	1	1	10	9
gi|6322082|	1	1	1	1	1	1	1	1	1	1	10	9
gi|6322084|	1	1	1	1	1	1	1	1	1	1	10	9
gi|6322088|	1	1	1	1	1	1	1	1	1	1	10	9
gi|6322096|	1	1	1	1	1	1	1	1	1	1	10	9
gi|6322097|	1	1	1	1	1	1	1	1	1	1	10	9
gi|6322100|	1	1	1	1	1	1	1	1	1	1	10	9
gi|6322108|	1	1	1	1	1	1	1	1	1	1	10	9
gi|6322115|	1	1	1	1	1	1	1	1	1	1	10	9
gi|6322116|	1	1	1	1	1	1	1	1	1	1	10	9

gi|6322120|	1	1	1	1	1	1	1	1	1	1	10	9
gi|6322123|	1	1	1	1	1	1	1	1	1	1	10	9
gi|6322128|	1	1	1	1	1	1	1	1	1	1	10	9
gi|6322137|	1	1	1	1	1	1	1	1	1	1	10	9
gi|6322141|	1	1	1	1	1	1	1	1	1	1	10	9
gi|6322143|	1	1	1	1	1	1	1	1	1	1	10	9
gi|6322145|	1	1	1	1	1	1	1	1	1	1	10	9
gi|6322146|	1	1	1	1	1	1	1	1	1	1	10	9
gi|6322147|	1	1	1	1	1	1	1	1	1	1	10	9
gi|6322151|	1	1	1	1	1	1	1	1	1	1	10	9
gi|6322154|	1	1	1	1	1	1	1	1	1	1	10	9
gi|6322155|	1	1	1	1	1	1	1	1	1	1	10	9
gi|6322159|	1	1	1	1	1	1	1	1	1	1	10	9
gi|6322163|	1	1	1	1	1	1	1	1	1	1	10	9
gi|6322168|	1	1	1	1	1	1	1	1	1	1	10	9
gi|6322171|	1	1	1	1	1	1	1	1	1	1	10	9
gi|6322177|	1	1	1	1	1	1	1	1	1	1	10	9
gi|6322182|	1	1	1	1	1	1	1	1	1	1	10	9
gi|6322185|	1	1	1	1	1	1	1	1	1	1	10	9
gi|6322188|	1	1	1	1	1	1	1	1	1	1	10	9
gi|6322189|	1	1	1	1	1	1	1	1	1	1	10	9
gi|6322191|	1	1	1	1	1	1	1	1	1	1	10	9
gi|6322192|	1	1	1	1	1	1	1	1	1	1	10	9
gi|6322194|	1	1	1	1	1	1	1	1	1	1	10	9
gi|6322195|	1	1	1	1	1	1	1	1	1	1	10	9
gi|6322217|	1	1	1	1	1	1	1	1	1	1	10	9
gi|6322226|	1	1	1	1	1	1	1	1	1	1	10	9
gi|6322228|	1	1	1	1	1	1	1	1	1	1	10	9
gi|6322242|	1	1	1	1	1	1	1	1	1	1	10	9
gi|6322247|	1	1	1	1	1	1	1	1	1	1	10	9
gi|6322261|	1	1	1	1	1	1	1	1	1	1	10	9
gi|6322264|	1	1	1	1	1	1	1	1	1	1	10	9
gi|6322270|	1	1	1	1	1	1	1	1	1	1	10	9
gi|6322271|	1	1	1	1	1	1	1	1	1	1	10	9
gi|6322272|	1	1	1	1	1	1	1	1	1	1	10	9
gi|6322274|	1	1	1	1	1	1	1	1	1	1	10	9
gi|6322284|	1	1	1	1	1	1	1	1	1	1	10	9
gi|6322286|	1	1	1	1	1	1	1	1	1	1	10	9
gi|6322293|	1	1	1	1	1	1	1	1	1	1	10	9
gi|6322294|	1	1	1	1	1	1	1	1	1	1	10	9
gi|6322296|	1	1	1	1	1	1	1	1	1	1	10	9
gi|6322297|	1	1	1	1	1	1	1	1	1	1	10	9
gi|6322299|	1	1	1	1	1	1	1	1	1	1	10	9
gi|6322306|	1	1	1	1	1	1	1	1	1	1	10	9
gi|6322307|	1	1	1	1	1	1	1	1	1	1	10	9
gi|6322318|	1	1	1	1	1	1	1	1	1	1	10	9
gi|6322320|	1	1	1	1	1	1	1	1	1	1	10	9
gi|6322321|	1	1	1	1	1	1	1	1	1	1	10	9
gi|6322323|	1	1	1	1	1	1	1	1	1	1	10	9
gi|6322325|	1	1	1	1	1	1	1	1	1	1	10	9
gi|6322328|	1	1	1	1	1	1	1	1	1	1	10	9
gi|6322331|	1	1	1	1	1	1	1	1	1	1	10	9
gi|6322333|	1	1	1	1	1	1	1	1	1	1	10	9
gi|6322335|	1	1	1	1	1	1	1	1	1	1	10	9
gi|6322336|	1	1	1	1	1	1	1	1	1	1	10	9
gi|6322337|	1	1	1	1	1	1	1	1	1	1	10	9
gi|6322341|	1	1	1	1	1	1	1	1	1	1	10	9
gi|6322346|	1	1	1	1	1	1	1	1	1	1	10	9
gi|6322349|	1	1	1	1	1	1	1	1	1	1	10	9
gi|6322350|	1	1	1	1	1	1	1	1	1	1	10	9
gi|6322355|	1	1	1	1	1	1	1	1	1	1	10	9
gi|6322357|	1	1	1	1	1	1	1	1	1	1	10	9
gi|6322359|	1	1	1	1	1	1	1	1	1	1	10	9
gi|6322366|	1	1	1	1	1	1	1	1	1	1	10	9
gi|6322380|	1	1	1	1	1	1	1	1	1	1	10	9
gi|6322383|	1	1	1	1	1	1	1	1	1	1	10	9
gi|6322387|	1	1	1	1	1	1	1	1	1	1	10	9
gi|6322388|	1	1	1	1	1	1	1	1	1	1	10	9
gi|6322391|	1	1	1	1	1	1	1	1	1	1	10	9
gi|6322392|	1	1	1	1	1	1	1	1	1	1	10	9

gi|6322399|	1	1	1	1	1	1	1	1	1	1	10	9
gi|6322401|	1	1	1	1	1	1	1	1	1	1	10	9
gi|6322408|	1	1	1	1	1	1	1	1	1	1	10	9
gi|6322409|	1	1	1	1	1	1	1	1	1	1	10	9
gi|6322411|	1	1	1	1	1	1	1	1	1	1	10	9
gi|6322416|	1	1	1	1	1	1	1	1	1	1	10	9
gi|6322425|	1	1	1	1	1	1	1	1	1	1	10	9
gi|6322426|	1	1	1	1	1	1	1	1	1	1	10	9
gi|6322427|	1	1	1	1	1	1	1	1	1	1	10	9
gi|6322430|	1	1	1	1	1	1	1	1	1	1	10	9
gi|6322434|	1	1	1	1	1	1	1	1	1	1	10	9
gi|6322436|	1	1	1	1	1	1	1	1	1	1	10	9
gi|6322446|	1	1	1	1	1	1	1	1	1	1	10	9
gi|6322452|	1	1	1	1	1	1	1	1	1	1	10	9
gi|6322455|	1	1	1	1	1	1	1	1	1	1	10	9
gi|6322458|	1	1	1	1	1	1	1	1	1	1	10	9
gi|6322459|	1	1	1	1	1	1	1	1	1	1	10	9
gi|6322461|	1	1	1	1	1	1	1	1	1	1	10	9
gi|6322464|	1	1	1	1	1	1	1	1	1	1	10	9
gi|6322466|	1	1	1	1	1	1	1	1	1	1	10	9
gi|6322468|	1	1	1	1	1	1	1	1	1	1	10	9
gi|6322469|	1	1	1	1	1	1	1	1	1	1	10	9
gi|6322484|	1	1	1	1	1	1	1	1	1	1	10	9
gi|6322491|	1	1	1	1	1	1	1	1	1	1	10	9
gi|6322492|	1	1	1	1	1	1	1	1	1	1	10	9
gi|6322495|	1	1	1	1	1	1	1	1	1	1	10	9
gi|6322497|	1	1	1	1	1	1	1	1	1	1	10	9
gi|6322500|	1	1	1	1	1	1	1	1	1	1	10	9
gi|6322505|	1	1	1	1	1	1	1	1	1	1	10	9
gi|6322507|	1	1	1	1	1	1	1	1	1	1	10	9
gi|6322508|	1	1	1	1	1	1	1	1	1	1	10	9
gi|6322511|	1	1	1	1	1	1	1	1	1	1	10	9
gi|6322517|	1	1	1	1	1	1	1	1	1	1	10	9
gi|6322518|	1	1	1	1	1	1	1	1	1	1	10	9
gi|6322519|	1	1	1	1	1	1	1	1	1	1	10	9
gi|6322523|	1	1	1	1	1	1	1	1	1	1	10	9
gi|6322524|	1	1	1	1	1	1	1	1	1	1	10	9
gi|6322525|	1	1	1	1	1	1	1	1	1	1	10	9
gi|6322526|	1	1	1	1	1	1	1	1	1	1	10	9
gi|6322528|	1	1	1	1	1	1	1	1	1	1	10	9
gi|6322529|	1	1	1	1	1	1	1	1	1	1	10	9
gi|6322531|	1	1	1	1	1	1	1	1	1	1	10	9
gi|6322532|	1	1	1	1	1	1	1	1	1	1	10	9
gi|6322537|	1	1	1	1	1	1	1	1	1	1	10	9
gi|6322549|	1	1	1	1	1	1	1	1	1	1	10	9
gi|6322552|	1	1	1	1	1	1	1	1	1	1	10	9
gi|6322554|	1	1	1	1	1	1	1	1	1	1	10	9
gi|6322555|	1	1	1	1	1	1	1	1	1	1	10	9
gi|6322556|	1	1	1	1	1	1	1	1	1	1	10	9
gi|6322557|	1	1	1	1	1	1	1	1	1	1	10	9
gi|6322563|	1	1	1	1	1	1	1	1	1	1	10	9
gi|6322564|	1	1	1	1	1	1	1	1	1	1	10	9
gi|6322565|	1	1	1	1	1	1	1	1	1	1	10	9
gi|6322569|	1	1	1	1	1	1	1	1	1	1	10	9
gi|6322570|	1	1	1	1	1	1	1	1	1	1	10	9
gi|6322577|	1	1	1	1	1	1	1	1	1	1	10	9
gi|6322579|	1	1	1	1	1	1	1	1	1	1	10	9
gi|6322581|	1	1	1	1	1	1	1	1	1	1	10	9
gi|6322582|	1	1	1	1	1	1	1	1	1	1	10	9
gi|6322585|	1	1	1	1	1	1	1	1	1	1	10	9
gi|6322591|	1	1	1	1	1	1	1	1	1	1	10	9
gi|6322605|	1	1	1	1	1	1	1	1	1	1	10	9
gi|6322608|	1	1	1	1	1	1	1	1	1	1	10	9
gi|6322618|	1	1	1	1	1	1	1	1	1	1	10	9
gi|6322619|	1	1	1	1	1	1	1	1	1	1	10	9
gi|6322631|	1	1	1	1	1	1	1	1	1	1	10	9
gi|6322633|	1	1	1	1	1	1	1	1	1	1	10	9
gi|6322634|	1	1	1	1	1	1	1	1	1	1	10	9
gi|6322636|	1	1	1	1	1	1	1	1	1	1	10	9
gi|6322637|	1	1	1	1	1	1	1	1	1	1	10	9

gi|6322639|	1	1	1	1	1	1	1	1	1	1	10	9
gi|6322640|	1	1	1	1	1	1	1	1	1	1	10	9
gi|6322651|	1	1	1	1	1	1	1	1	1	1	10	9
gi|6322652|	1	1	1	1	1	1	1	1	1	1	10	9
gi|6322654|	1	1	1	1	1	1	1	1	1	1	10	9
gi|6322655|	1	1	1	1	1	1	1	1	1	1	10	9
gi|6322658|	1	1	1	1	1	1	1	1	1	1	10	9
gi|6322659|	1	1	1	1	1	1	1	1	1	1	10	9
gi|6322667|	1	1	1	1	1	1	1	1	1	1	10	9
gi|6322668|	1	1	1	1	1	1	1	1	1	1	10	9
gi|6322675|	1	1	1	1	1	1	1	1	1	1	10	9
gi|6322676|	1	1	1	1	1	1	1	1	1	1	10	9
gi|6322680|	1	1	1	1	1	1	1	1	1	1	10	9
gi|6322682|	1	1	1	1	1	1	1	1	1	1	10	9
gi|6322683|	1	1	1	1	1	1	1	1	1	1	10	9
gi|6322688|	1	1	1	1	1	1	1	1	1	1	10	9
gi|6322689|	1	1	1	1	1	1	1	1	1	1	10	9
gi|6322693|	1	1	1	1	1	1	1	1	1	1	10	9
gi|6322698|	1	1	1	1	1	1	1	1	1	1	10	9
gi|6322699|	1	1	1	1	1	1	1	1	1	1	10	9
gi|6322700|	1	1	1	1	1	1	1	1	1	1	10	9
gi|6322701|	1	1	1	1	1	1	1	1	1	1	10	9
gi|6322702|	1	1	1	1	1	1	1	1	1	1	10	9
gi|6322704|	1	1	1	1	1	1	1	1	1	1	10	9
gi|6322709|	1	1	1	1	1	1	1	1	1	1	10	9
gi|6322710|	1	1	1	1	1	1	1	1	1	1	10	9
gi|6322714|	1	1	1	1	1	1	1	1	1	1	10	9
gi|6322715|	1	1	1	1	1	1	1	1	1	1	10	9
gi|6322720|	1	1	1	1	1	1	1	1	1	1	10	9
gi|6322722|	1	1	1	1	1	1	1	1	1	1	10	9
gi|6322723|	1	1	1	1	1	1	1	1	1	1	10	9
gi|6322728|	1	1	1	1	1	1	1	1	1	1	10	9
gi|6322729|	1	1	1	1	1	1	1	1	1	1	10	9
gi|6322733|	1	1	1	1	1	1	1	1	1	1	10	9
gi|6322736|	1	1	1	1	1	1	1	1	1	1	10	9
gi|6322743|	1	1	1	1	1	1	1	1	1	1	10	9
gi|6322745|	1	1	1	1	1	1	1	1	1	1	10	9
gi|6322748|	1	1	1	1	1	1	1	1	1	1	10	9
gi|6322749|	1	1	1	1	1	1	1	1	1	1	10	9
gi|6322750|	1	1	1	1	1	1	1	1	1	1	10	9
gi|6322755|	1	1	1	1	1	1	1	1	1	1	10	9
gi|6322762|	1	1	1	1	1	1	1	1	1	1	10	9
gi|6322765|	1	1	1	1	1	1	1	1	1	1	10	9
gi|6322769|	1	1	1	1	1	1	1	1	1	1	10	9
gi|6322770|	1	1	1	1	1	1	1	1	1	1	10	9
gi|6322771|	1	1	1	1	1	1	1	1	1	1	10	9
gi|6322772|	1	1	1	1	1	1	1	1	1	1	10	9
gi|6322777|	1	1	1	1	1	1	1	1	1	1	10	9
gi|6322783|	1	1	1	1	1	1	1	1	1	1	10	9
gi|6322792|	1	1	1	1	1	1	1	1	1	1	10	9
gi|6322794|	1	1	1	1	1	1	1	1	1	1	10	9
gi|6322806|	1	1	1	1	1	1	1	1	1	1	10	9
gi|6322811|	1	1	1	1	1	1	1	1	1	1	10	9
gi|6322815|	1	1	1	1	1	1	1	1	1	1	10	9
gi|6322823|	1	1	1	1	1	1	1	1	1	1	10	9
gi|6322828|	1	1	1	1	1	1	1	1	1	1	10	9
gi|6322830|	1	1	1	1	1	1	1	1	1	1	10	9
gi|6322831|	1	1	1	1	1	1	1	1	1	1	10	9
gi|6322833|	1	1	1	1	1	1	1	1	1	1	10	9
gi|6322834|	1	1	1	1	1	1	1	1	1	1	10	9
gi|6322835|	1	1	1	1	1	1	1	1	1	1	10	9
gi|6322842|	1	1	1	1	1	1	1	1	1	1	10	9
gi|6322844|	1	1	1	1	1	1	1	1	1	1	10	9
gi|6322847|	1	1	1	1	1	1	1	1	1	1	10	9
gi|6322852|	1	1	1	1	1	1	1	1	1	1	10	9
gi|6322853|	1	1	1	1	1	1	1	1	1	1	10	9
gi|6322854|	1	1	1	1	1	1	1	1	1	1	10	9
gi|6322855|	1	1	1	1	1	1	1	1	1	1	10	9
gi|6322861|	1	1	1	1	1	1	1	1	1	1	10	9
gi|6322866|	1	1	1	1	1	1	1	1	1	1	10	9

gi|6322869|	1	1	1	1	1	1	1	1	1	1	10	9
gi|6322876|	1	1	1	1	1	1	1	1	1	1	10	9
gi|6322878|	1	1	1	1	1	1	1	1	1	1	10	9
gi|6322883|	1	1	1	1	1	1	1	1	1	1	10	9
gi|6322901|	1	1	1	1	1	1	1	1	1	1	10	9
gi|6322904|	1	1	1	1	1	1	1	1	1	1	10	9
gi|6322905|	1	1	1	1	1	1	1	1	1	1	10	9
gi|6322908|	1	1	1	1	1	1	1	1	1	1	10	9
gi|6322910|	1	1	1	1	1	1	1	1	1	1	10	9
gi|6322912|	1	1	1	1	1	1	1	1	1	1	10	9
gi|6322921|	1	1	1	1	1	1	1	1	1	1	10	9
gi|6322924|	1	1	1	1	1	1	1	1	1	1	10	9
gi|6322925|	1	1	1	1	1	1	1	1	1	1	10	9
gi|6322932|	1	1	1	1	1	1	1	1	1	1	10	9
gi|6322934|	1	1	1	1	1	1	1	1	1	1	10	9
gi|6322937|	1	1	1	1	1	1	1	1	1	1	10	9
gi|6322939|	1	1	1	1	1	1	1	1	1	1	10	9
gi|6322946|	1	1	1	1	1	1	1	1	1	1	10	9
gi|6322947|	1	1	1	1	1	1	1	1	1	1	10	9
gi|6322956|	1	1	1	1	1	1	1	1	1	1	10	9
gi|6322957|	1	1	1	1	1	1	1	1	1	1	10	9
gi|6322978|	1	1	1	1	1	1	1	1	1	1	10	9
gi|6322980|	1	1	1	1	1	1	1	1	1	1	10	9
gi|6322984|	1	1	1	1	1	1	1	1	1	1	10	9
gi|6322987|	1	1	1	1	1	1	1	1	1	1	10	9
gi|6322988|	1	1	1	1	1	1	1	1	1	1	10	9
gi|6322989|	1	1	1	1	1	1	1	1	1	1	10	9
gi|6322992|	1	1	1	1	1	1	1	1	1	1	10	9
gi|6322994|	1	1	1	1	1	1	1	1	1	1	10	9
gi|6322997|	1	1	1	1	1	1	1	1	1	1	10	9
gi|6322999|	1	1	1	1	1	1	1	1	1	1	10	9
gi|6323001|	1	1	1	1	1	1	1	1	1	1	10	9
gi|6323004|	1	1	1	1	1	1	1	1	1	1	10	9
gi|6323009|	1	1	1	1	1	1	1	1	1	1	10	9
gi|6323011|	1	1	1	1	1	1	1	1	1	1	10	9
gi|6323014|	1	1	1	1	1	1	1	1	1	1	10	9
gi|6323016|	1	1	1	1	1	1	1	1	1	1	10	9
gi|6323018|	1	1	1	1	1	1	1	1	1	1	10	9
gi|6323019|	1	1	1	1	1	1	1	1	1	1	10	9
gi|6323021|	1	1	1	1	1	1	1	1	1	1	10	9
gi|6323028|	1	1	1	1	1	1	1	1	1	1	10	9
gi|6323030|	1	1	1	1	1	1	1	1	1	1	10	9
gi|6323033|	1	1	1	1	1	1	1	1	1	1	10	9
gi|6323037|	1	1	1	1	1	1	1	1	1	1	10	9
gi|6323047|	1	1	1	1	1	1	1	1	1	1	10	9
gi|6323048|	1	1	1	1	1	1	1	1	1	1	10	9
gi|6323050|	1	1	1	1	1	1	1	1	1	1	10	9
gi|6323053|	1	1	1	1	1	1	1	1	1	1	10	9
gi|6323054|	1	1	1	1	1	1	1	1	1	1	10	9
gi|6323056|	1	1	1	1	1	1	1	1	1	1	10	9
gi|6323060|	1	1	1	1	1	1	1	1	1	1	10	9
gi|6323062|	1	1	1	1	1	1	1	1	1	1	10	9
gi|6323063|	1	1	1	1	1	1	1	1	1	1	10	9
gi|6323067|	1	1	1	1	1	1	1	1	1	1	10	9
gi|6323072|	1	1	1	1	1	1	1	1	1	1	10	9
gi|6323077|	1	1	1	1	1	1	1	1	1	1	10	9
gi|6323087|	1	1	1	1	1	1	1	1	1	1	10	9
gi|6323088|	1	1	1	1	1	1	1	1	1	1	10	9
gi|6323089|	1	1	1	1	1	1	1	1	1	1	10	9
gi|6323090|	1	1	1	1	1	1	1	1	1	1	10	9
gi|6323095|	1	1	1	1	1	1	1	1	1	1	10	9
gi|6323098|	1	1	1	1	1	1	1	1	1	1	10	9
gi|6323099|	1	1	1	1	1	1	1	1	1	1	10	9
gi|6323104|	1	1	1	1	1	1	1	1	1	1	10	9
gi|6323110|	1	1	1	1	1	1	1	1	1	1	10	9
gi|6323112|	1	1	1	1	1	1	1	1	1	1	10	9
gi|6323114|	1	1	1	1	1	1	1	1	1	1	10	9
gi|6323115|	1	1	1	1	1	1	1	1	1	1	10	9
gi|6323118|	1	1	1	1	1	1	1	1	1	1	10	9
gi|6323119|	1	1	1	1	1	1	1	1	1	1	10	9

gi|6323121|	1	1	1	1	1	1	1	1	1	1	10	9
gi|6323125|	1	1	1	1	1	1	1	1	1	1	10	9
gi|6323135|	1	1	1	1	1	1	1	1	1	1	10	9
gi|6323136|	1	1	1	1	1	1	1	1	1	1	10	9
gi|6323142|	1	1	1	1	1	1	1	1	1	1	10	9
gi|6323145|	1	1	1	1	1	1	1	1	1	1	10	9
gi|6323147|	1	1	1	1	1	1	1	1	1	1	10	9
gi|6323158|	1	1	1	1	1	1	1	1	1	1	10	9
gi|6323165|	1	1	1	1	1	1	1	1	1	1	10	9
gi|6323172|	1	1	1	1	1	1	1	1	1	1	10	9
gi|6323175|	1	1	1	1	1	1	1	1	1	1	10	9
gi|6323177|	1	1	1	1	1	1	1	1	1	1	10	9
gi|6323182|	1	1	1	1	1	1	1	1	1	1	10	9
gi|6323192|	1	1	1	1	1	1	1	1	1	1	10	9
gi|6323196|	1	1	1	1	1	1	1	1	1	1	10	9
gi|6323197|	1	1	1	1	1	1	1	1	1	1	10	9
gi|6323200|	1	1	1	1	1	1	1	1	1	1	10	9
gi|6323201|	1	1	1	1	1	1	1	1	1	1	10	9
gi|6323203|	1	1	1	1	1	1	1	1	1	1	10	9
gi|6323209|	1	1	1	1	1	1	1	1	1	1	10	9
gi|6323214|	1	1	1	1	1	1	1	1	1	1	10	9
gi|6323215|	1	1	1	1	1	1	1	1	1	1	10	9
gi|6323217|	1	1	1	1	1	1	1	1	1	1	10	9
gi|6323224|	1	1	1	1	1	1	1	1	1	1	10	9
gi|6323225|	1	1	1	1	1	1	1	1	1	1	10	9
gi|6323226|	1	1	1	1	1	1	1	1	1	1	10	9
gi|6323229|	1	1	1	1	1	1	1	1	1	1	10	9
gi|6323235|	1	1	1	1	1	1	1	1	1	1	10	9
gi|6323236|	1	1	1	1	1	1	1	1	1	1	10	9
gi|6323237|	1	1	1	1	1	1	1	1	1	1	10	9
gi|6323239|	1	1	1	1	1	1	1	1	1	1	10	9
gi|6323241|	1	1	1	1	1	1	1	1	1	1	10	9
gi|6323246|	1	1	1	1	1	1	1	1	1	1	10	9
gi|6323251|	1	1	1	1	1	1	1	1	1	1	10	9
gi|6323259|	1	1	1	1	1	1	1	1	1	1	10	9
gi|6323263|	1	1	1	1	1	1	1	1	1	1	10	9
gi|6323269|	1	1	1	1	1	1	1	1	1	1	10	9
gi|6323272|	1	1	1	1	1	1	1	1	1	1	10	9
gi|6323273|	1	1	1	1	1	1	1	1	1	1	10	9
gi|6323275|	1	1	1	1	1	1	1	1	1	1	10	9
gi|6323276|	1	1	1	1	1	1	1	1	1	1	10	9
gi|6323277|	1	1	1	1	1	1	1	1	1	1	10	9
gi|6323278|	1	1	1	1	1	1	1	1	1	1	10	9
gi|6323280|	1	1	1	1	1	1	1	1	1	1	10	9
gi|6323282|	1	1	1	1	1	1	1	1	1	1	10	9
gi|6323288|	1	1	1	1	1	1	1	1	1	1	10	9
gi|6323289|	1	1	1	1	1	1	1	1	1	1	10	9
gi|6323291|	1	1	1	1	1	1	1	1	1	1	10	9
gi|6323294|	1	1	1	1	1	1	1	1	1	1	10	9
gi|6323298|	1	1	1	1	1	1	1	1	1	1	10	9
gi|6323304|	1	1	1	1	1	1	1	1	1	1	10	9
gi|6323305|	1	1	1	1	1	1	1	1	1	1	10	9
gi|6323306|	1	1	1	1	1	1	1	1	1	1	10	9
gi|6323307|	1	1	1	1	1	1	1	1	1	1	10	9
gi|6323318|	1	1	1	1	1	1	1	1	1	1	10	9
gi|6323320|	1	1	1	1	1	1	1	1	1	1	10	9
gi|6323324|	1	1	1	1	1	1	1	1	1	1	10	9
gi|6323330|	1	1	1	1	1	1	1	1	1	1	10	9
gi|6323333|	1	1	1	1	1	1	1	1	1	1	10	9
gi|6323335|	1	1	1	1	1	1	1	1	1	1	10	9
gi|6323336|	1	1	1	1	1	1	1	1	1	1	10	9
gi|6323337|	1	1	1	1	1	1	1	1	1	1	10	9
gi|6323340|	1	1	1	1	1	1	1	1	1	1	10	9
gi|6323355|	1	1	1	1	1	1	1	1	1	1	10	9
gi|6323360|	1	1	1	1	1	1	1	1	1	1	10	9
gi|6323365|	1	1	1	1	1	1	1	1	1	1	10	9
gi|6323371|	1	1	1	1	1	1	1	1	1	1	10	9
gi|6323376|	1	1	1	1	1	1	1	1	1	1	10	9
gi|6323377|	1	1	1	1	1	1	1	1	1	1	10	9
gi|6323379|	1	1	1	1	1	1	1	1	1	1	10	9

gi|6323386|	1	1	1	1	1	1	1	1	1	1	10	9
gi|6323389|	1	1	1	1	1	1	1	1	1	1	10	9
gi|6323394|	1	1	1	1	1	1	1	1	1	1	10	9
gi|6323399|	1	1	1	1	1	1	1	1	1	1	10	9
gi|6323401|	1	1	1	1	1	1	1	1	1	1	10	9
gi|6323402|	1	1	1	1	1	1	1	1	1	1	10	9
gi|6323409|	1	1	1	1	1	1	1	1	1	1	10	9
gi|6323411|	1	1	1	1	1	1	1	1	1	1	10	9
gi|6323414|	1	1	1	1	1	1	1	1	1	1	10	9
gi|6323416|	1	1	1	1	1	1	1	1	1	1	10	9
gi|6323418|	1	1	1	1	1	1	1	1	1	1	10	9
gi|6323420|	1	1	1	1	1	1	1	1	1	1	10	9
gi|6323429|	1	1	1	1	1	1	1	1	1	1	10	9
gi|6323430|	1	1	1	1	1	1	1	1	1	1	10	9
gi|6323431|	1	1	1	1	1	1	1	1	1	1	10	9
gi|6323433|	1	1	1	1	1	1	1	1	1	1	10	9
gi|6323437|	1	1	1	1	1	1	1	1	1	1	10	9
gi|6323438|	1	1	1	1	1	1	1	1	1	1	10	9
gi|6323441|	1	1	1	1	1	1	1	1	1	1	10	9
gi|6323442|	1	1	1	1	1	1	1	1	1	1	10	9
gi|6323451|	1	1	1	1	1	1	1	1	1	1	10	9
gi|6323462|	1	1	1	1	1	1	1	1	1	1	10	9
gi|6323464|	1	1	1	1	1	1	1	1	1	1	10	9
gi|6323465|	1	1	1	1	1	1	1	1	1	1	10	9
gi|6323470|	1	1	1	1	1	1	1	1	1	1	10	9
gi|6323471|	1	1	1	1	1	1	1	1	1	1	10	9
gi|6323474|	1	1	1	1	1	1	1	1	1	1	10	9
gi|6323480|	1	1	1	1	1	1	1	1	1	1	10	9
gi|6323481|	1	1	1	1	1	1	1	1	1	1	10	9
gi|6323482|	1	1	1	1	1	1	1	1	1	1	10	9
gi|6323483|	1	1	1	1	1	1	1	1	1	1	10	9
gi|6323505|	1	1	1	1	1	1	1	1	1	1	10	9
gi|6323509|	1	1	1	1	1	1	1	1	1	1	10	9
gi|6323510|	1	1	1	1	1	1	1	1	1	1	10	9
gi|6323511|	1	1	1	1	1	1	1	1	1	1	10	9
gi|6323526|	1	1	1	1	1	1	1	1	1	1	10	9
gi|6323534|	1	1	1	1	1	1	1	1	1	1	10	9
gi|6323544|	1	1	1	1	1	1	1	1	1	1	10	9
gi|6323546|	1	1	1	1	1	1	1	1	1	1	10	9
gi|6323547|	1	1	1	1	1	1	1	1	1	1	10	9
gi|6323552|	1	1	1	1	1	1	1	1	1	1	10	9
gi|6323554|	1	1	1	1	1	1	1	1	1	1	10	9
gi|6323560|	1	1	1	1	1	1	1	1	1	1	10	9
gi|6323562|	1	1	1	1	1	1	1	1	1	1	10	9
gi|6323565|	1	1	1	1	1	1	1	1	1	1	10	9
gi|6323566|	1	1	1	1	1	1	1	1	1	1	10	9
gi|6323567|	1	1	1	1	1	1	1	1	1	1	10	9
gi|6323571|	1	1	1	1	1	1	1	1	1	1	10	9
gi|6323575|	1	1	1	1	1	1	1	1	1	1	10	9
gi|6323576|	1	1	1	1	1	1	1	1	1	1	10	9
gi|6323577|	1	1	1	1	1	1	1	1	1	1	10	9
gi|6323583|	1	1	1	1	1	1	1	1	1	1	10	9
gi|6323585|	1	1	1	1	1	1	1	1	1	1	10	9
gi|6323587|	1	1	1	1	1	1	1	1	1	1	10	9
gi|6323606|	1	1	1	1	1	1	1	1	1	1	10	9
gi|6323613|	1	1	1	1	1	1	1	1	1	1	10	9
gi|6323615|	1	1	1	1	1	1	1	1	1	1	10	9
gi|6323617|	1	1	1	1	1	1	1	1	1	1	10	9
gi|6323619|	1	1	1	1	1	1	1	1	1	1	10	9
gi|6323625|	1	1	1	1	1	1	1	1	1	1	10	9
gi|6323627|	1	1	1	1	1	1	1	1	1	1	10	9
gi|6323630|	1	1	1	1	1	1	1	1	1	1	10	9
gi|6323642|	1	1	1	1	1	1	1	1	1	1	10	9
gi|6323643|	1	1	1	1	1	1	1	1	1	1	10	9
gi|6323646|	1	1	1	1	1	1	1	1	1	1	10	9
gi|6323651|	1	1	1	1	1	1	1	1	1	1	10	9
gi|6323653|	1	1	1	1	1	1	1	1	1	1	10	9
gi|6323657|	1	1	1	1	1	1	1	1	1	1	10	9
gi|6323664|	1	1	1	1	1	1	1	1	1	1	10	9
gi|6323678|	1	1	1	1	1	1	1	1	1	1	10	9

gi|6323685|	1	1	1	1	1	1	1	1	1	1	10	9
gi|6323699|	1	1	1	1	1	1	1	1	1	1	10	9
gi|6323701|	1	1	1	1	1	1	1	1	1	1	10	9
gi|6323713|	1	1	1	1	1	1	1	1	1	1	10	9
gi|6323720|	1	1	1	1	1	1	1	1	1	1	10	9
gi|6323726|	1	1	1	1	1	1	1	1	1	1	10	9
gi|6323730|	1	1	1	1	1	1	1	1	1	1	10	9
gi|6323731|	1	1	1	1	1	1	1	1	1	1	10	9
gi|6323736|	1	1	1	1	1	1	1	1	1	1	10	9
gi|6323740|	1	1	1	1	1	1	1	1	1	1	10	9
gi|6323748|	1	1	1	1	1	1	1	1	1	1	10	9
gi|6323749|	1	1	1	1	1	1	1	1	1	1	10	9
gi|6323751|	1	1	1	1	1	1	1	1	1	1	10	9
gi|6323752|	1	1	1	1	1	1	1	1	1	1	10	9
gi|6323755|	1	1	1	1	1	1	1	1	1	1	10	9
gi|6323756|	1	1	1	1	1	1	1	1	1	1	10	9
gi|6323757|	1	1	1	1	1	1	1	1	1	1	10	9
gi|6323763|	1	1	1	1	1	1	1	1	1	1	10	9
gi|6323768|	1	1	1	1	1	1	1	1	1	1	10	9
gi|6323775|	1	1	1	1	1	1	1	1	1	1	10	9
gi|6323776|	1	1	1	1	1	1	1	1	1	1	10	9
gi|6323779|	1	1	1	1	1	1	1	1	1	1	10	9
gi|6323787|	1	1	1	1	1	1	1	1	1	1	10	9
gi|6323788|	1	1	1	1	1	1	1	1	1	1	10	9
gi|6323791|	1	1	1	1	1	1	1	1	1	1	10	9
gi|6323795|	1	1	1	1	1	1	1	1	1	1	10	9
gi|6323813|	1	1	1	1	1	1	1	1	1	1	10	9
gi|6323814|	1	1	1	1	1	1	1	1	1	1	10	9
gi|6323817|	1	1	1	1	1	1	1	1	1	1	10	9
gi|6323818|	1	1	1	1	1	1	1	1	1	1	10	9
gi|6323819|	1	1	1	1	1	1	1	1	1	1	10	9
gi|6323821|	1	1	1	1	1	1	1	1	1	1	10	9
gi|6323822|	1	1	1	1	1	1	1	1	1	1	10	9
gi|6323830|	1	1	1	1	1	1	1	1	1	1	10	9
gi|6323837|	1	1	1	1	1	1	1	1	1	1	10	9
gi|6323840|	1	1	1	1	1	1	1	1	1	1	10	9
gi|6323844|	1	1	1	1	1	1	1	1	1	1	10	9
gi|6323846|	1	1	1	1	1	1	1	1	1	1	10	9
gi|6323848|	1	1	1	1	1	1	1	1	1	1	10	9
gi|6323861|	1	1	1	1	1	1	1	1	1	1	10	9
gi|6323863|	1	1	1	1	1	1	1	1	1	1	10	9
gi|6323869|	1	1	1	1	1	1	1	1	1	1	10	9
gi|6323872|	1	1	1	1	1	1	1	1	1	1	10	9
gi|6323873|	1	1	1	1	1	1	1	1	1	1	10	9
gi|6323880|	1	1	1	1	1	1	1	1	1	1	10	9
gi|6323882|	1	1	1	1	1	1	1	1	1	1	10	9
gi|6323885|	1	1	1	1	1	1	1	1	1	1	10	9
gi|6323886|	1	1	1	1	1	1	1	1	1	1	10	9
gi|6323896|	1	1	1	1	1	1	1	1	1	1	10	9
gi|6323899|	1	1	1	1	1	1	1	1	1	1	10	9
gi|6323903|	1	1	1	1	1	1	1	1	1	1	10	9
gi|6323904|	1	1	1	1	1	1	1	1	1	1	10	9
gi|6323916|	1	1	1	1	1	1	1	1	1	1	10	9
gi|6323923|	1	1	1	1	1	1	1	1	1	1	10	9
gi|6323928|	1	1	1	1	1	1	1	1	1	1	10	9
gi|6323932|	1	1	1	1	1	1	1	1	1	1	10	9
gi|6323933|	1	1	1	1	1	1	1	1	1	1	10	9
gi|6323944|	1	1	1	1	1	1	1	1	1	1	10	9
gi|6323947|	1	1	1	1	1	1	1	1	1	1	10	9
gi|6323954|	1	1	1	1	1	1	1	1	1	1	10	9
gi|6323955|	1	1	1	1	1	1	1	1	1	1	10	9
gi|6323958|	1	1	1	1	1	1	1	1	1	1	10	9
gi|6323959|	1	1	1	1	1	1	1	1	1	1	10	9
gi|6323962|	1	1	1	1	1	1	1	1	1	1	10	9
gi|6323968|	1	1	1	1	1	1	1	1	1	1	10	9
gi|6323969|	1	1	1	1	1	1	1	1	1	1	10	9
gi|6323974|	1	1	1	1	1	1	1	1	1	1	10	9
gi|6323985|	1	1	1	1	1	1	1	1	1	1	10	9
gi|6323999|	1	1	1	1	1	1	1	1	1	1	10	9
gi|6324000|	1	1	1	1	1	1	1	1	1	1	10	9

gi|6324001|	1	1	1	1	1	1	1	1	1	1	10	9
gi|6324003|	1	1	1	1	1	1	1	1	1	1	10	9
gi|6324005|	1	1	1	1	1	1	1	1	1	1	10	9
gi|6324006|	1	1	1	1	1	1	1	1	1	1	10	9
gi|6324009|	1	1	1	1	1	1	1	1	1	1	10	9
gi|6324011|	1	1	1	1	1	1	1	1	1	1	10	9
gi|6324012|	1	1	1	1	1	1	1	1	1	1	10	9
gi|6324022|	1	1	1	1	1	1	1	1	1	1	10	9
gi|6324025|	1	1	1	1	1	1	1	1	1	1	10	9
gi|6324027|	1	1	1	1	1	1	1	1	1	1	10	9
gi|6324028|	1	1	1	1	1	1	1	1	1	1	10	9
gi|6324030|	1	1	1	1	1	1	1	1	1	1	10	9
gi|6324031|	1	1	1	1	1	1	1	1	1	1	10	9
gi|6324036|	1	1	1	1	1	1	1	1	1	1	10	9
gi|6324039|	1	1	1	1	1	1	1	1	1	1	10	9
gi|6324041|	1	1	1	1	1	1	1	1	1	1	10	9
gi|6324042|	1	1	1	1	1	1	1	1	1	1	10	9
gi|6324055|	1	1	1	1	1	1	1	1	1	1	10	9
gi|6324062|	1	1	1	1	1	1	1	1	1	1	10	9
gi|6324066|	1	1	1	1	1	1	1	1	1	1	10	9
gi|6324067|	1	1	1	1	1	1	1	1	1	1	10	9
gi|6324079|	1	1	1	1	1	1	1	1	1	1	10	9
gi|6324082|	1	1	1	1	1	1	1	1	1	1	10	9
gi|6324085|	1	1	1	1	1	1	1	1	1	1	10	9
gi|6324088|	1	1	1	1	1	1	1	1	1	1	10	9
gi|6324103|	1	1	1	1	1	1	1	1	1	1	10	9
gi|6324107|	1	1	1	1	1	1	1	1	1	1	10	9
gi|6324109|	1	1	1	1	1	1	1	1	1	1	10	9
gi|6324110|	1	1	1	1	1	1	1	1	1	1	10	9
gi|6324120|	1	1	1	1	1	1	1	1	1	1	10	9
gi|6324122|	1	1	1	1	1	1	1	1	1	1	10	9
gi|6324125|	1	1	1	1	1	1	1	1	1	1	10	9
gi|6324126|	1	1	1	1	1	1	1	1	1	1	10	9
gi|6324128|	1	1	1	1	1	1	1	1	1	1	10	9
gi|6324129|	1	1	1	1	1	1	1	1	1	1	10	9
gi|6324140|	1	1	1	1	1	1	1	1	1	1	10	9
gi|6324143|	1	1	1	1	1	1	1	1	1	1	10	9
gi|6324144|	1	1	1	1	1	1	1	1	1	1	10	9
gi|6324146|	1	1	1	1	1	1	1	1	1	1	10	9
gi|6324149|	1	1	1	1	1	1	1	1	1	1	10	9
gi|6324151|	1	1	1	1	1	1	1	1	1	1	10	9
gi|6324155|	1	1	1	1	1	1	1	1	1	1	10	9
gi|6324160|	1	1	1	1	1	1	1	1	1	1	10	9
gi|6324161|	1	1	1	1	1	1	1	1	1	1	10	9
gi|6324166|	1	1	1	1	1	1	1	1	1	1	10	9
gi|6324168|	1	1	1	1	1	1	1	1	1	1	10	9
gi|6324175|	1	1	1	1	1	1	1	1	1	1	10	9
gi|6324182|	1	1	1	1	1	1	1	1	1	1	10	9
gi|6324191|	1	1	1	1	1	1	1	1	1	1	10	9
gi|6324194|	1	1	1	1	1	1	1	1	1	1	10	9
gi|6324197|	1	1	1	1	1	1	1	1	1	1	10	9
gi|6324208|	1	1	1	1	1	1	1	1	1	1	10	9
gi|6324215|	1	1	1	1	1	1	1	1	1	1	10	9
gi|6324217|	1	1	1	1	1	1	1	1	1	1	10	9
gi|6324218|	1	1	1	1	1	1	1	1	1	1	10	9
gi|6324222|	1	1	1	1	1	1	1	1	1	1	10	9
gi|6324224|	1	1	1	1	1	1	1	1	1	1	10	9
gi|6324227|	1	1	1	1	1	1	1	1	1	1	10	9
gi|6324228|	1	1	1	1	1	1	1	1	1	1	10	9
gi|6324231|	1	1	1	1	1	1	1	1	1	1	10	9
gi|6324232|	1	1	1	1	1	1	1	1	1	1	10	9
gi|6324233|	1	1	1	1	1	1	1	1	1	1	10	9
gi|6324236|	1	1	1	1	1	1	1	1	1	1	10	9
gi|6324237|	1	1	1	1	1	1	1	1	1	1	10	9
gi|6324239|	1	1	1	1	1	1	1	1	1	1	10	9
gi|6324241|	1	1	1	1	1	1	1	1	1	1	10	9
gi|6324252|	1	1	1	1	1	1	1	1	1	1	10	9
gi|6324254|	1	1	1	1	1	1	1	1	1	1	10	9
gi|6324256|	1	1	1	1	1	1	1	1	1	1	10	9
gi|6324257|	1	1	1	1	1	1	1	1	1	1	10	9

gi|6324258|	1	1	1	1	1	1	1	1	1	1	10	9
gi|6324260|	1	1	1	1	1	1	1	1	1	1	10	9
gi|6324262|	1	1	1	1	1	1	1	1	1	1	10	9
gi|6324265|	1	1	1	1	1	1	1	1	1	1	10	9
gi|6324268|	1	1	1	1	1	1	1	1	1	1	10	9
gi|6324269|	1	1	1	1	1	1	1	1	1	1	10	9
gi|6324275|	1	1	1	1	1	1	1	1	1	1	10	9
gi|6324279|	1	1	1	1	1	1	1	1	1	1	10	9
gi|6324280|	1	1	1	1	1	1	1	1	1	1	10	9
gi|6324283|	1	1	1	1	1	1	1	1	1	1	10	9
gi|6324291|	1	1	1	1	1	1	1	1	1	1	10	9
gi|6324297|	1	1	1	1	1	1	1	1	1	1	10	9
gi|6324298|	1	1	1	1	1	1	1	1	1	1	10	9
gi|6324306|	1	1	1	1	1	1	1	1	1	1	10	9
gi|6324307|	1	1	1	1	1	1	1	1	1	1	10	9
gi|6324308|	1	1	1	1	1	1	1	1	1	1	10	9
gi|6324312|	1	1	1	1	1	1	1	1	1	1	10	9
gi|6324314|	1	1	1	1	1	1	1	1	1	1	10	9
gi|6324319|	1	1	1	1	1	1	1	1	1	1	10	9
gi|6324321|	1	1	1	1	1	1	1	1	1	1	10	9
gi|6324322|	1	1	1	1	1	1	1	1	1	1	10	9
gi|6324326|	1	1	1	1	1	1	1	1	1	1	10	9
gi|6324327|	1	1	1	1	1	1	1	1	1	1	10	9
gi|6324328|	1	1	1	1	1	1	1	1	1	1	10	9
gi|6324332|	1	1	1	1	1	1	1	1	1	1	10	9
gi|6324334|	1	1	1	1	1	1	1	1	1	1	10	9
gi|6324338|	1	1	1	1	1	1	1	1	1	1	10	9
gi|6324339|	1	1	1	1	1	1	1	1	1	1	10	9
gi|6324342|	1	1	1	1	1	1	1	1	1	1	10	9
gi|6324343|	1	1	1	1	1	1	1	1	1	1	10	9
gi|6324346|	1	1	1	1	1	1	1	1	1	1	10	9
gi|6324354|	1	1	1	1	1	1	1	1	1	1	10	9
gi|6324358|	1	1	1	1	1	1	1	1	1	1	10	9
gi|6324359|	1	1	1	1	1	1	1	1	1	1	10	9
gi|6324362|	1	1	1	1	1	1	1	1	1	1	10	9
gi|6324363|	1	1	1	1	1	1	1	1	1	1	10	9
gi|6324364|	1	1	1	1	1	1	1	1	1	1	10	9
gi|6324366|	1	1	1	1	1	1	1	1	1	1	10	9
gi|6324370|	1	1	1	1	1	1	1	1	1	1	10	9
gi|6324371|	1	1	1	1	1	1	1	1	1	1	10	9
gi|6324375|	1	1	1	1	1	1	1	1	1	1	10	9
gi|6324376|	1	1	1	1	1	1	1	1	1	1	10	9
gi|6324378|	1	1	1	1	1	1	1	1	1	1	10	9
gi|6324380|	1	1	1	1	1	1	1	1	1	1	10	9
gi|6324381|	1	1	1	1	1	1	1	1	1	1	10	9
gi|6324398|	1	1	1	1	1	1	1	1	1	1	10	9
gi|6324400|	1	1	1	1	1	1	1	1	1	1	10	9
gi|6324417|	1	1	1	1	1	1	1	1	1	1	10	9
gi|6324432|	1	1	1	1	1	1	1	1	1	1	10	9
gi|6324433|	1	1	1	1	1	1	1	1	1	1	10	9
gi|6324438|	1	1	1	1	1	1	1	1	1	1	10	9
gi|6324444|	1	1	1	1	1	1	1	1	1	1	10	9
gi|6324445|	1	1	1	1	1	1	1	1	1	1	10	9
gi|6324446|	1	1	1	1	1	1	1	1	1	1	10	9
gi|6324449|	1	1	1	1	1	1	1	1	1	1	10	9
gi|6324450|	1	1	1	1	1	1	1	1	1	1	10	9
gi|6324451|	1	1	1	1	1	1	1	1	1	1	10	9
gi|6324452|	1	1	1	1	1	1	1	1	1	1	10	9
gi|6324457|	1	1	1	1	1	1	1	1	1	1	10	9
gi|6324459|	1	1	1	1	1	1	1	1	1	1	10	9
gi|6324460|	1	1	1	1	1	1	1	1	1	1	10	9
gi|6324472|	1	1	1	1	1	1	1	1	1	1	10	9
gi|6324478|	1	1	1	1	1	1	1	1	1	1	10	9
gi|6324479|	1	1	1	1	1	1	1	1	1	1	10	9
gi|6324482|	1	1	1	1	1	1	1	1	1	1	10	9
gi|6324493|	1	1	1	1	1	1	1	1	1	1	10	9
gi|6324496|	1	1	1	1	1	1	1	1	1	1	10	9
gi|6324506|	1	1	1	1	1	1	1	1	1	1	10	9
gi|6324510|	1	1	1	1	1	1	1	1	1	1	10	9
gi|6324511|	1	1	1	1	1	1	1	1	1	1	10	9

gi|6324513|	1	1	1	1	1	1	1	1	1	1	10	9
gi|6324527|	1	1	1	1	1	1	1	1	1	1	10	9
gi|6324533|	1	1	1	1	1	1	1	1	1	1	10	9
gi|6324535|	1	1	1	1	1	1	1	1	1	1	10	9
gi|6324539|	1	1	1	1	1	1	1	1	1	1	10	9
gi|6324540|	1	1	1	1	1	1	1	1	1	1	10	9
gi|6324550|	1	1	1	1	1	1	1	1	1	1	10	9
gi|6324552|	1	1	1	1	1	1	1	1	1	1	10	9
gi|6324555|	1	1	1	1	1	1	1	1	1	1	10	9
gi|6324557|	1	1	1	1	1	1	1	1	1	1	10	9
gi|6324562|	1	1	1	1	1	1	1	1	1	1	10	9
gi|6324564|	1	1	1	1	1	1	1	1	1	1	10	9
gi|6324568|	1	1	1	1	1	1	1	1	1	1	10	9
gi|6324569|	1	1	1	1	1	1	1	1	1	1	10	9
gi|6324570|	1	1	1	1	1	1	1	1	1	1	10	9
gi|6324571|	1	1	1	1	1	1	1	1	1	1	10	9
gi|6324575|	1	1	1	1	1	1	1	1	1	1	10	9
gi|6324578|	1	1	1	1	1	1	1	1	1	1	10	9
gi|6324579|	1	1	1	1	1	1	1	1	1	1	10	9
gi|6324580|	1	1	1	1	1	1	1	1	1	1	10	9
gi|6324585|	1	1	1	1	1	1	1	1	1	1	10	9
gi|6324588|	1	1	1	1	1	1	1	1	1	1	10	9
gi|6324594|	1	1	1	1	1	1	1	1	1	1	10	9
gi|6324974|	1	1	1	1	1	1	1	1	1	1	10	9
gi|6324987|	1	1	1	1	1	1	1	1	1	1	10	9
gi|6324989|	1	1	1	1	1	1	1	1	1	1	10	9
gi|6324993|	1	1	1	1	1	1	1	1	1	1	10	9
gi|6324996|	1	1	1	1	1	1	1	1	1	1	10	9
gi|6325004|	1	1	1	1	1	1	1	1	1	1	10	9
gi|6325006|	1	1	1	1	1	1	1	1	1	1	10	9
gi|6325007|	1	1	1	1	1	1	1	1	1	1	10	9
gi|6325009|	1	1	1	1	1	1	1	1	1	1	10	9
gi|6325016|	1	1	1	1	1	1	1	1	1	1	10	9
gi|6325018|	1	1	1	1	1	1	1	1	1	1	10	9
gi|6325020|	1	1	1	1	1	1	1	1	1	1	10	9
gi|6325021|	1	1	1	1	1	1	1	1	1	1	10	9
gi|6325022|	1	1	1	1	1	1	1	1	1	1	10	9
gi|6325024|	1	1	1	1	1	1	1	1	1	1	10	9
gi|6325029|	1	1	1	1	1	1	1	1	1	1	10	9
gi|6325030|	1	1	1	1	1	1	1	1	1	1	10	9
gi|6325036|	1	1	1	1	1	1	1	1	1	1	10	9
gi|6325038|	1	1	1	1	1	1	1	1	1	1	10	9
gi|6325039|	1	1	1	1	1	1	1	1	1	1	10	9
gi|6325044|	1	1	1	1	1	1	1	1	1	1	10	9
gi|6325045|	1	1	1	1	1	1	1	1	1	1	10	9
gi|6325047|	1	1	1	1	1	1	1	1	1	1	10	9
gi|6325052|	1	1	1	1	1	1	1	1	1	1	10	9
gi|6325053|	1	1	1	1	1	1	1	1	1	1	10	9
gi|6325058|	1	1	1	1	1	1	1	1	1	1	10	9
gi|6325061|	1	1	1	1	1	1	1	1	1	1	10	9
gi|6325074|	1	1	1	1	1	1	1	1	1	1	10	9
gi|6325078|	1	1	1	1	1	1	1	1	1	1	10	9
gi|6325079|	1	1	1	1	1	1	1	1	1	1	10	9
gi|6325085|	1	1	1	1	1	1	1	1	1	1	10	9
gi|6325087|	1	1	1	1	1	1	1	1	1	1	10	9
gi|6325090|	1	1	1	1	1	1	1	1	1	1	10	9
gi|6325093|	1	1	1	1	1	1	1	1	1	1	10	9
gi|6325097|	1	1	1	1	1	1	1	1	1	1	10	9
gi|6325100|	1	1	1	1	1	1	1	1	1	1	10	9
gi|6325102|	1	1	1	1	1	1	1	1	1	1	10	9
gi|6325104|	1	1	1	1	1	1	1	1	1	1	10	9
gi|6325105|	1	1	1	1	1	1	1	1	1	1	10	9
gi|6325106|	1	1	1	1	1	1	1	1	1	1	10	9
gi|6325107|	1	1	1	1	1	1	1	1	1	1	10	9
gi|6325114|	1	1	1	1	1	1	1	1	1	1	10	9
gi|6325116|	1	1	1	1	1	1	1	1	1	1	10	9
gi|6325117|	1	1	1	1	1	1	1	1	1	1	10	9
gi|6325122|	1	1	1	1	1	1	1	1	1	1	10	9
gi|6325125|	1	1	1	1	1	1	1	1	1	1	10	9
gi|6325138|	1	1	1	1	1	1	1	1	1	1	10	9

gi|6325140|	1	1	1	1	1	1	1	1	1	1	10	9
gi|6325141|	1	1	1	1	1	1	1	1	1	1	10	9
gi|6325144|	1	1	1	1	1	1	1	1	1	1	10	9
gi|6325147|	1	1	1	1	1	1	1	1	1	1	10	9
gi|6325151|	1	1	1	1	1	1	1	1	1	1	10	9
gi|6325153|	1	1	1	1	1	1	1	1	1	1	10	9
gi|6325157|	1	1	1	1	1	1	1	1	1	1	10	9
gi|6325160|	1	1	1	1	1	1	1	1	1	1	10	9
gi|6325161|	1	1	1	1	1	1	1	1	1	1	10	9
gi|6325164|	1	1	1	1	1	1	1	1	1	1	10	9
gi|6325166|	1	1	1	1	1	1	1	1	1	1	10	9
gi|6325167|	1	1	1	1	1	1	1	1	1	1	10	9
gi|6325168|	1	1	1	1	1	1	1	1	1	1	10	9
gi|6325175|	1	1	1	1	1	1	1	1	1	1	10	9
gi|6325176|	1	1	1	1	1	1	1	1	1	1	10	9
gi|6325178|	1	1	1	1	1	1	1	1	1	1	10	9
gi|6325183|	1	1	1	1	1	1	1	1	1	1	10	9
gi|6325194|	1	1	1	1	1	1	1	1	1	1	10	9
gi|6325196|	1	1	1	1	1	1	1	1	1	1	10	9
gi|6325198|	1	1	1	1	1	1	1	1	1	1	10	9
gi|6325199|	1	1	1	1	1	1	1	1	1	1	10	9
gi|6325206|	1	1	1	1	1	1	1	1	1	1	10	9
gi|6325209|	1	1	1	1	1	1	1	1	1	1	10	9
gi|6325212|	1	1	1	1	1	1	1	1	1	1	10	9
gi|6325215|	1	1	1	1	1	1	1	1	1	1	10	9
gi|6325217|	1	1	1	1	1	1	1	1	1	1	10	9
gi|6325220|	1	1	1	1	1	1	1	1	1	1	10	9
gi|6325221|	1	1	1	1	1	1	1	1	1	1	10	9
gi|6325226|	1	1	1	1	1	1	1	1	1	1	10	9
gi|6325228|	1	1	1	1	1	1	1	1	1	1	10	9
gi|6325229|	1	1	1	1	1	1	1	1	1	1	10	9
gi|6325231|	1	1	1	1	1	1	1	1	1	1	10	9
gi|6325234|	1	1	1	1	1	1	1	1	1	1	10	9
gi|6325235|	1	1	1	1	1	1	1	1	1	1	10	9
gi|6325240|	1	1	1	1	1	1	1	1	1	1	10	9
gi|6325247|	1	1	1	1	1	1	1	1	1	1	10	9
gi|6325248|	1	1	1	1	1	1	1	1	1	1	10	9
gi|6325249|	1	1	1	1	1	1	1	1	1	1	10	9
gi|6325257|	1	1	1	1	1	1	1	1	1	1	10	9
gi|6325260|	1	1	1	1	1	1	1	1	1	1	10	9
gi|6325261|	1	1	1	1	1	1	1	1	1	1	10	9
gi|6325267|	1	1	1	1	1	1	1	1	1	1	10	9
gi|6325268|	1	1	1	1	1	1	1	1	1	1	10	9
gi|6325270|	1	1	1	1	1	1	1	1	1	1	10	9
gi|6325273|	1	1	1	1	1	1	1	1	1	1	10	9
gi|6325276|	1	1	1	1	1	1	1	1	1	1	10	9
gi|6325278|	1	1	1	1	1	1	1	1	1	1	10	9
gi|6325281|	1	1	1	1	1	1	1	1	1	1	10	9
gi|6325285|	1	1	1	1	1	1	1	1	1	1	10	9
gi|6325286|	1	1	1	1	1	1	1	1	1	1	10	9
gi|6325288|	1	1	1	1	1	1	1	1	1	1	10	9
gi|6325290|	1	1	1	1	1	1	1	1	1	1	10	9
gi|6325291|	1	1	1	1	1	1	1	1	1	1	10	9
gi|6325293|	1	1	1	1	1	1	1	1	1	1	10	9
gi|6325296|	1	1	1	1	1	1	1	1	1	1	10	9
gi|6325297|	1	1	1	1	1	1	1	1	1	1	10	9
gi|6325298|	1	1	1	1	1	1	1	1	1	1	10	9
gi|6325300|	1	1	1	1	1	1	1	1	1	1	10	9
gi|6325302|	1	1	1	1	1	1	1	1	1	1	10	9
gi|6325305|	1	1	1	1	1	1	1	1	1	1	10	9
gi|6325309|	1	1	1	1	1	1	1	1	1	1	10	9
gi|6325311|	1	1	1	1	1	1	1	1	1	1	10	9
gi|6325323|	1	1	1	1	1	1	1	1	1	1	10	9
gi|6325325|	1	1	1	1	1	1	1	1	1	1	10	9
gi|6325326|	1	1	1	1	1	1	1	1	1	1	10	9
gi|6325329|	1	1	1	1	1	1	1	1	1	1	10	9
gi|6325331|	1	1	1	1	1	1	1	1	1	1	10	9
gi|6325337|	1	1	1	1	1	1	1	1	1	1	10	9
gi|6325338|	1	1	1	1	1	1	1	1	1	1	10	9
gi|6325343|	1	1	1	1	1	1	1	1	1	1	10	9

gi|6325345|	1	1	1	1	1	1	1	1	1	1	10	9
gi|6325351|	1	1	1	1	1	1	1	1	1	1	10	9
gi|6325359|	1	1	1	1	1	1	1	1	1	1	10	9
gi|6325360|	1	1	1	1	1	1	1	1	1	1	10	9
gi|6325361|	1	1	1	1	1	1	1	1	1	1	10	9
gi|6325364|	1	1	1	1	1	1	1	1	1	1	10	9
gi|6325365|	1	1	1	1	1	1	1	1	1	1	10	9
gi|6325367|	1	1	1	1	1	1	1	1	1	1	10	9
gi|6325368|	1	1	1	1	1	1	1	1	1	1	10	9
gi|6325369|	1	1	1	1	1	1	1	1	1	1	10	9
gi|6325370|	1	1	1	1	1	1	1	1	1	1	10	9
gi|6325375|	1	1	1	1	1	1	1	1	1	1	10	9
gi|6325376|	1	1	1	1	1	1	1	1	1	1	10	9
gi|6325377|	1	1	1	1	1	1	1	1	1	1	10	9
gi|6325379|	1	1	1	1	1	1	1	1	1	1	10	9
gi|6325389|	1	1	1	1	1	1	1	1	1	1	10	9
gi|6325399|	1	1	1	1	1	1	1	1	1	1	10	9
gi|6325403|	1	1	1	1	1	1	1	1	1	1	10	9
gi|6325419|	1	1	1	1	1	1	1	1	1	1	10	9
gi|6325423|	1	1	1	1	1	1	1	1	1	1	10	9
gi|6325431|	1	1	1	1	1	1	1	1	1	1	10	9
gi|6325434|	1	1	1	1	1	1	1	1	1	1	10	9
gi|6325435|	1	1	1	1	1	1	1	1	1	1	10	9
gi|6325438|	1	1	1	1	1	1	1	1	1	1	10	9
gi|6325439|	1	1	1	1	1	1	1	1	1	1	10	9
gi|6325440|	1	1	1	1	1	1	1	1	1	1	10	9
gi|6325445|	1	1	1	1	1	1	1	1	1	1	10	9
gi|6579192|	1	1	1	1	1	1	1	1	1	1	10	9
gi|6681846|	1	1	1	1	1	1	1	1	1	1	10	9
gi|6681847|	1	1	1	1	1	1	1	1	1	1	10	9
gi|6681848|	1	1	1	1	1	1	1	1	1	1	10	9
gi|6681849|	1	1	1	1	1	1	1	1	1	1	10	9
gi|7276233|	1	1	1	1	1	1	1	1	1	1	10	9
gi|9755329|	1	1	1	1	1	1	1	1	1	1	10	9
gi|9755331|	1	1	1	1	1	1	1	1	1	1	10	9
gi|9755332|	1	1	1	1	1	1	1	1	1	1	10	9
gi|9755335|	1	1	1	1	1	1	1	1	1	1	10	9
gi|9755336|	1	1	1	1	1	1	1	1	1	1	10	9
gi|9755341|	1	1	1	1	1	1	1	1	1	1	10	9
gi|9755344|	1	1	1	1	1	1	1	1	1	1	10	9
gi|14270688|	1	1	1	1	1	1	1	1	1		9	9
gi|42759861|	1	1	1	1	1	1	1	1	1		9	9
gi|6319304|	1	1	1	1	1	1	1	1	1		9	9
gi|6319705|	1	1	1	1	1	1	1	1	1		9	9
gi|6319895|	1	1	1	1	1	1	1	1	1		9	9
gi|6320881|	1	1	1	1	1	1	1	1	1		9	9
gi|6320899|	1	1	1	1	1	1	1	1	1		9	9
gi|6321005|	1	1	1	1	1	1	1	1	1		9	9
gi|6321610|	1	1	1	1	1	1	1	1	1		9	9
gi|6321860|	1	1	1	1	1	1	1	1	1		9	9
gi|6321959|	1	1	1	1	1	1	1	1	1		9	9
gi|6322138|	1	1	1	1	1	1	1	1	1		9	9
gi|6322167|	1	1	1	1	1	1	1	1	1		9	9
gi|6322559|	1	1	1	1	1	1	1	1	1		9	9
gi|6322656|	1	1	1	1	1	1	1	1	1		9	9
gi|6322657|	1	1	1	1	1	1	1	1	1		9	9
gi|6322705|	1	1	1	1	1	1	1	1	1		9	9
gi|6323383|	1	1	1	1	1	1	1	1	1		9	9
gi|6323415|	1	1	1	1	1	1	1	1	1		9	9
gi|6323632|	1	1	1	1	1	1	1	1	1		9	9
gi|6323665|	1	1	1	1	1	1	1	1	1		9	9
gi|6323693|	1	1	1	1	1	1	1	1	1		9	9
gi|6323724|	1	1	1	1	1	1	1	1	1		9	9
gi|6324475|	1	1	1	1	1	1	1	1	1		9	9
gi|6324524|	1	1	1	1	1	1	1	1	1		9	9
gi|6324530|	1	1	1	1	1	1	1	1	1		9	9
gi|6325313|	1	1	1	1	1	1	1	1	1		9	9
gi|10383789|	1	1	1		1	1	1	1	1	1	9	8
gi|10383794|	1	1	1	1	1	1	1	1		1	9	8
gi|14318476|	1		1	1	1	1	1	1	1	1	9	8

gi|14318503|	1	1	1	1	1	1		1	1	1	9	8
gi|21648335|	1	1	1	1	1	1		1	1	1	9	8
gi|37362616|	1	1		1	1	1	1	1	1	1	9	8
gi|37362618|	1	1	1	1	1		1	1	1	1	9	8
gi|37362651|	1	1		1	1	1	1	1	1	1	9	8
gi|37362665|	1	1	1	1	1	1	1	1		1	9	8
gi|37362671|	1	1		1	1	1	1	1	1	1	9	8
gi|41629676|	1		1	1	1	1	1	1	1	1	9	8
gi|42742308|	1	1	1	1	1	1		1	1	1	9	8
gi|6319391|	1	1	1	1	1	1		1	1	1	9	8
gi|6319392|	1		1	1	1	1	1	1	1	1	9	8
gi|6319413|		1	1	1	1	1	1	1	1	1	9	8
gi|6319416|	1	1		1	1	1	1	1	1	1	9	8
gi|6319475|	1	1	1	1	1	1		1	1	1	9	8
gi|6319488|	1	1	1		1	1	1	1	1	1	9	8
gi|6319509|	1	1	1	1	1	1		1	1	1	9	8
gi|6319520|	1	1	1		1	1	1	1	1	1	9	8
gi|6319534|	1	1		1	1	1	1	1	1	1	9	8
gi|6319586|	1	1	1	1	1	1		1	1	1	9	8
gi|6319601|	1	1	1	1		1	1	1	1	1	9	8
gi|6319611|	1	1	1	1	1	1		1	1	1	9	8
gi|6319621|	1	1	1	1		1	1	1	1	1	9	8
gi|6319655|	1	1	1	1	1	1	1	1		1	9	8
gi|6319672|	1	1	1	1	1	1	1		1	1	9	8
gi|6319687|	1	1		1	1	1	1	1	1	1	9	8
gi|6319704|	1	1	1	1	1	1	1	1		1	9	8
gi|6319719|	1	1		1	1	1	1	1	1	1	9	8
gi|6319724|	1	1	1	1	1	1		1	1	1	9	8
gi|6319728|	1	1	1		1	1	1	1	1	1	9	8
gi|6319731|	1		1	1	1	1	1	1	1	1	9	8
gi|6319743|	1	1	1	1	1	1	1		1	1	9	8
gi|6319768|	1	1	1	1	1	1	1		1	1	9	8
gi|6319773|	1	1	1	1	1	1	1	1		1	9	8
gi|6319793|	1	1		1	1	1	1	1	1	1	9	8
gi|6319882|	1		1	1	1	1	1	1	1	1	9	8
gi|6319890|	1	1	1	1	1	1		1	1	1	9	8
gi|6319892|	1	1	1	1	1	1		1	1	1	9	8
gi|6319905|	1	1	1	1	1	1	1	1		1	9	8
gi|6319908|	1	1	1	1	1		1	1	1	1	9	8
gi|6319965|	1	1	1	1	1		1	1	1	1	9	8
gi|6319996|	1	1		1	1	1	1	1	1	1	9	8
gi|6320000|	1	1	1	1	1	1		1	1	1	9	8
gi|6320083|	1	1	1	1	1	1		1	1	1	9	8
gi|6320122|	1		1	1	1	1	1	1	1	1	9	8
gi|6320151|	1		1	1	1	1	1	1	1	1	9	8
gi|6320159|	1	1	1	1	1	1	1		1	1	9	8
gi|6320176|	1	1		1	1	1	1	1	1	1	9	8
gi|6320183|	1	1		1	1	1	1	1	1	1	9	8
gi|6320196|	1	1	1		1	1	1	1	1	1	9	8
gi|6320222|	1	1	1	1	1		1	1	1	1	9	8
gi|6320223|	1	1	1	1	1	1	1	1		1	9	8
gi|6320249|	1	1		1	1	1	1	1	1	1	9	8
gi|6320252|	1	1		1	1	1	1	1	1	1	9	8
gi|6320285|	1	1	1		1	1	1	1	1	1	9	8
gi|6320310|	1	1	1	1	1	1	1		1	1	9	8
gi|6320333|	1	1	1	1	1	1	1		1	1	9	8
gi|6320368|	1	1		1	1	1	1	1	1	1	9	8
gi|6320434|	1	1	1	1	1		1	1	1	1	9	8
gi|6320442|		1	1	1	1	1	1	1	1	1	9	8
gi|6320458|	1	1	1	1	1	1	1	1		1	9	8
gi|6320471|	1	1		1	1	1	1	1	1	1	9	8
gi|6320493|	1	1	1	1	1	1	1	1		1	9	8
gi|6320500|	1	1	1	1	1	1	1	1		1	9	8
gi|6320507|	1	1	1	1	1		1	1	1	1	9	8
gi|6320517|	1	1		1	1	1	1	1	1	1	9	8
gi|6320533|	1		1	1	1	1	1	1	1	1	9	8
gi|6320537|	1	1		1	1	1	1	1	1	1	9	8
gi|6320540|	1		1	1	1	1	1	1	1	1	9	8
gi|6320590|	1		1	1	1	1	1	1	1	1	9	8
gi|6320603|	1	1		1	1	1	1	1	1	1	9	8

gi|6320637|	1	1	1	1	1	1		1	1	1	9	8
gi|6320656|	1	1		1	1	1	1	1	1	1	9	8
gi|6320785|	1	1	1	1	1	1	1		1	1	9	8
gi|6320789|	1	1	1	1	1	1		1	1	1	9	8
gi|6320797|	1		1	1	1	1	1	1	1	1	9	8
gi|6320798|		1	1	1	1	1	1	1	1	1	9	8
gi|6320815|	1	1	1	1	1	1		1	1	1	9	8
gi|6320821|	1	1	1		1	1	1	1	1	1	9	8
gi|6320826|	1	1	1	1	1	1	1	1		1	9	8
gi|6320834|	1	1	1	1	1	1		1	1	1	9	8
gi|6320835|		1	1	1	1	1	1	1	1	1	9	8
gi|6320859|	1	1	1	1	1	1	1	1		1	9	8
gi|6320866|	1		1	1	1	1	1	1	1	1	9	8
gi|6320991|	1	1	1	1	1		1	1	1	1	9	8
gi|6321206|	1	1	1	1	1	1		1	1	1	9	8
gi|6321229|	1	1	1	1	1	1	1		1	1	9	8
gi|6321233|	1	1	1	1	1	1	1	1		1	9	8
gi|6321243|	1	1	1		1	1	1	1	1	1	9	8
gi|6321296|	1	1		1	1	1	1	1	1	1	9	8
gi|6321297|	1	1	1	1	1	1		1	1	1	9	8
gi|6321326|	1	1		1	1	1	1	1	1	1	9	8
gi|6321337|	1	1		1	1	1	1	1	1	1	9	8
gi|6321342|	1	1	1	1	1	1	1	1		1	9	8
gi|6321355|	1		1	1	1	1	1	1	1	1	9	8
gi|6321383|	1	1	1	1	1	1	1		1	1	9	8
gi|6321391|	1	1	1	1	1	1	1		1	1	9	8
gi|6321398|	1	1		1	1	1	1	1	1	1	9	8
gi|6321411|		1	1	1	1	1	1	1	1	1	9	8
gi|6321415|	1	1	1	1	1	1	1	1		1	9	8
gi|6321461|	1	1		1	1	1	1	1	1	1	9	8
gi|6321475|	1	1		1	1	1	1	1	1	1	9	8
gi|6321491|	1	1	1	1	1	1		1	1	1	9	8
gi|6321493|	1		1	1	1	1	1	1	1	1	9	8
gi|6321515|	1	1	1	1	1	1		1	1	1	9	8
gi|6321527|	1	1	1		1	1	1	1	1	1	9	8
gi|6321533|	1	1	1		1	1	1	1	1	1	9	8
gi|6321547|	1	1	1		1	1	1	1	1	1	9	8
gi|6321666|		1	1	1	1	1	1	1	1	1	9	8
gi|6321671|	1	1	1	1	1	1		1	1	1	9	8
gi|6321677|	1	1		1	1	1	1	1	1	1	9	8
gi|6321681|	1	1		1	1	1	1	1	1	1	9	8
gi|6321687|	1		1	1	1	1	1	1	1	1	9	8
gi|6321688|	1	1	1	1	1	1	1		1	1	9	8
gi|6321701|	1	1		1	1	1	1	1	1	1	9	8
gi|6321724|	1	1	1	1	1	1	1		1	1	9	8
gi|6321755|	1	1	1	1	1	1	1	1		1	9	8
gi|6321757|	1	1	1	1	1	1	1	1		1	9	8
gi|6321782|	1		1	1	1	1	1	1	1	1	9	8
gi|6321789|	1	1		1	1	1	1	1	1	1	9	8
gi|6321806|	1	1		1	1	1	1	1	1	1	9	8
gi|6321820|	1	1	1	1	1	1	1		1	1	9	8
gi|6321836|	1	1	1	1	1	1	1	1		1	9	8
gi|6321841|		1	1	1	1	1	1	1	1	1	9	8
gi|6321861|	1	1	1	1	1	1	1	1		1	9	8
gi|6321867|	1		1	1	1	1	1	1	1	1	9	8
gi|6321872|	1		1	1	1	1	1	1	1	1	9	8
gi|6321878|	1	1	1		1	1	1	1	1	1	9	8
gi|6321881|	1	1	1	1	1	1		1	1	1	9	8
gi|6321891|	1	1	1	1	1		1	1	1	1	9	8
gi|6321909|	1	1		1	1	1	1	1	1	1	9	8
gi|6321982|	1	1	1	1	1	1	1		1	1	9	8
gi|6321994|	1		1	1	1	1	1	1	1	1	9	8
gi|6322057|	1		1	1	1	1	1	1	1	1	9	8
gi|6322078|	1	1	1	1	1	1	1		1	1	9	8
gi|6322110|	1	1	1		1	1	1	1	1	1	9	8
gi|6322142|	1	1		1	1	1	1	1	1	1	9	8
gi|6322184|	1	1	1	1	1	1	1		1	1	9	8
gi|6322196|	1	1	1	1	1	1	1		1	1	9	8
gi|6322198|	1	1	1	1	1	1		1	1	1	9	8
gi|6322199|	1	1	1	1	1	1	1		1	1	9	8

gi|6322218|	1	1	1	1	1	1	1	1		1	9	8
gi|6322265|	1		1	1	1	1	1	1	1	1	9	8
gi|6322289|	1	1		1	1	1	1	1	1	1	9	8
gi|6322352|	1	1	1	1	1		1	1	1	1	9	8
gi|6322382|	1	1	1	1	1	1	1	1		1	9	8
gi|6322393|	1	1	1	1	1	1	1	1		1	9	8
gi|6322431|	1	1	1	1	1	1	1		1	1	9	8
gi|6322467|	1	1	1	1	1		1	1	1	1	9	8
gi|6322493|	1	1	1	1	1		1	1	1	1	9	8
gi|6322504|		1	1	1	1	1	1	1	1	1	9	8
gi|6322510|	1	1	1		1	1	1	1	1	1	9	8
gi|6322583|	1	1	1	1	1		1	1	1	1	9	8
gi|6322638|	1	1		1	1	1	1	1	1	1	9	8
gi|6322660|	1	1	1	1	1	1	1	1		1	9	8
gi|6322664|	1		1	1	1	1	1	1	1	1	9	8
gi|6322697|	1	1		1	1	1	1	1	1	1	9	8
gi|6322759|	1	1		1	1	1	1	1	1	1	9	8
gi|6322810|	1	1	1	1	1	1	1	1		1	9	8
gi|6322824|	1	1		1	1	1	1	1	1	1	9	8
gi|6322826|		1	1	1	1	1	1	1	1	1	9	8
gi|6322839|	1		1	1	1	1	1	1	1	1	9	8
gi|6322840|	1	1		1	1	1	1	1	1	1	9	8
gi|6322860|	1	1	1	1	1	1	1		1	1	9	8
gi|6322864|	1	1	1	1	1	1	1	1		1	9	8
gi|6322882|	1		1	1	1	1	1	1	1	1	9	8
gi|6322889|	1	1	1	1	1	1	1		1	1	9	8
gi|6322952|		1	1	1	1	1	1	1	1	1	9	8
gi|6322990|	1		1	1	1	1	1	1	1	1	9	8
gi|6323010|	1	1	1		1	1	1	1	1	1	9	8
gi|6323017|	1		1	1	1	1	1	1	1	1	9	8
gi|6323057|	1	1	1		1	1	1	1	1	1	9	8
gi|6323101|	1		1	1	1	1	1	1	1	1	9	8
gi|6323128|	1	1	1		1	1	1	1	1	1	9	8
gi|6323132|	1	1		1	1	1	1	1	1	1	9	8
gi|6323150|	1	1	1	1	1	1	1	1		1	9	8
gi|6323160|	1	1	1	1	1	1	1		1	1	9	8
gi|6323176|	1	1	1	1	1	1		1	1	1	9	8
gi|6323204|	1	1	1	1	1	1		1	1	1	9	8
gi|6323268|		1	1	1	1	1	1	1	1	1	9	8
gi|6323322|	1	1		1	1	1	1	1	1	1	9	8
gi|6323354|	1	1	1	1	1	1	1		1	1	9	8
gi|6323367|	1	1	1	1	1	1	1		1	1	9	8
gi|6323368|	1	1	1	1	1	1		1	1	1	9	8
gi|6323381|	1	1	1	1	1	1	1	1		1	9	8
gi|6323412|	1	1	1	1	1	1	1		1	1	9	8
gi|6323419|	1	1	1	1	1	1	1		1	1	9	8
gi|6323450|	1	1	1	1	1	1		1	1	1	9	8
gi|6323454|	1	1	1	1	1	1	1	1		1	9	8
gi|6323492|	1	1	1	1	1	1	1	1		1	9	8
gi|6323541|		1	1	1	1	1	1	1	1	1	9	8
gi|6323543|	1	1		1	1	1	1	1	1	1	9	8
gi|6323579|	1	1	1	1	1	1	1		1	1	9	8
gi|6323639|	1	1		1	1	1	1	1	1	1	9	8
gi|6323660|	1	1	1	1	1	1	1		1	1	9	8
gi|6323686|	1	1	1	1	1	1	1		1	1	9	8
gi|6323706|	1	1	1		1	1	1	1	1	1	9	8
gi|6323725|	1	1	1		1	1	1	1	1	1	9	8
gi|6323760|	1	1	1	1	1	1		1	1	1	9	8
gi|6323769|	1	1	1		1	1	1	1	1	1	9	8
gi|6323780|	1	1	1	1	1	1	1	1		1	9	8
gi|6323853|	1	1	1	1	1	1		1	1	1	9	8
gi|6323866|	1	1	1	1	1	1	1	1		1	9	8
gi|6323879|		1	1	1	1	1	1	1	1	1	9	8
gi|6323889|	1	1	1	1	1	1		1	1	1	9	8
gi|6323892|	1	1		1	1	1	1	1	1	1	9	8
gi|6323950|	1	1	1	1	1	1		1	1	1	9	8
gi|6324016|	1	1	1	1	1	1	1	1		1	9	8
gi|6324049|	1	1	1		1	1	1	1	1	1	9	8
gi|6324054|	1		1	1	1	1	1	1	1	1	9	8
gi|6324058|	1	1	1	1	1	1	1		1	1	9	8

gi|6324074|		1	1	1	1	1	1	1	1	1	9	8
gi|6324089|	1	1	1		1	1	1	1	1	1	9	8
gi|6324201|	1	1	1	1	1	1	1		1	1	9	8
gi|6324203|	1	1		1	1	1	1	1	1	1	9	8
gi|6324211|	1	1	1		1	1	1	1	1	1	9	8
gi|6324219|	1	1	1	1	1	1		1	1	1	9	8
gi|6324301|	1		1	1	1	1	1	1	1	1	9	8
gi|6324305|	1		1	1	1	1	1	1	1	1	9	8
gi|6324323|	1	1		1	1	1	1	1	1	1	9	8
gi|6324325|	1	1	1	1	1	1	1		1	1	9	8
gi|6324330|	1		1	1	1	1	1	1	1	1	9	8
gi|6324357|	1		1	1	1	1	1	1	1	1	9	8
gi|6324360|	1	1	1	1	1	1		1	1	1	9	8
gi|6324374|	1		1	1	1	1	1	1	1	1	9	8
gi|6324437|	1	1	1	1		1	1	1	1	1	9	8
gi|6324448|	1	1	1	1	1	1		1	1	1	9	8
gi|6324474|	1	1	1		1	1	1	1	1	1	9	8
gi|6324498|	1	1	1	1	1	1	1	1		1	9	8
gi|6324512|	1	1	1		1	1	1	1	1	1	9	8
gi|6324514|	1	1		1	1	1	1	1	1	1	9	8
gi|6324521|	1	1	1	1	1	1	1		1	1	9	8
gi|6324574|	1	1	1	1	1	1	1	1		1	9	8
gi|6325068|	1	1	1	1	1	1		1	1	1	9	8
gi|6325080|	1	1	1	1	1	1	1	1		1	9	8
gi|6325103|	1	1	1	1	1	1	1	1		1	9	8
gi|6325110|	1	1	1	1	1	1	1	1		1	9	8
gi|6325123|	1		1	1	1	1	1	1	1	1	9	8
gi|6325126|	1	1	1		1	1	1	1	1	1	9	8
gi|6325135|	1		1	1	1	1	1	1	1	1	9	8
gi|6325137|	1	1	1	1	1	1	1	1		1	9	8
gi|6325162|	1	1	1	1	1	1	1	1		1	9	8
gi|6325171|	1	1	1	1	1	1		1	1	1	9	8
gi|6325192|	1	1	1	1	1		1	1	1	1	9	8
gi|6325255|	1	1	1	1	1	1		1	1	1	9	8
gi|6325256|		1	1	1	1	1	1	1	1	1	9	8
gi|6325272|	1	1	1	1	1	1	1		1	1	9	8
gi|6325282|	1	1		1	1	1	1	1	1	1	9	8
gi|6325292|	1	1	1	1	1	1		1	1	1	9	8
gi|6325307|	1	1	1	1	1	1		1	1	1	9	8
gi|6325315|	1	1	1		1	1	1	1	1	1	9	8
gi|6325330|	1	1		1	1	1	1	1	1	1	9	8
gi|6325339|	1	1		1	1	1	1	1	1	1	9	8
gi|6325394|	1		1	1	1	1	1	1	1	1	9	8
gi|6325398|	1	1	1	1	1	1	1	1		1	9	8
gi|6325418|	1	1	1	1	1	1	1	1		1	9	8
gi|6325441|	1	1	1	1	1	1	1		1	1	9	8
gi|6325444|	1		1	1	1	1	1	1	1	1	9	8
gi|6579194|	1	1	1	1	1		1	1	1	1	9	8
gi|9755327|	1	1	1	1	1	1	1	1		1	9	8
gi|14318489|	1	1	1	1	1	1	1	1			8	8
gi|6319256|	1	1	1	1	1		1	1	1		8	8
gi|6319516|	1	1	1		1	1	1	1	1		8	8
gi|6320200|	1	1	1	1		1	1	1	1		8	8
gi|6320371|	1	1		1	1	1	1	1	1		8	8
gi|6320788|	1	1	1	1		1	1	1	1		8	8
gi|6320843|	1	1	1	1	1	1	1		1		8	8
gi|6321010|	1	1	1	1	1		1	1	1		8	8
gi|6322004|		1	1	1	1	1	1	1	1		8	8
gi|6322474|	1	1	1	1		1	1	1	1		8	8
gi|6322887|	1	1	1	1		1	1	1	1		8	8
gi|6323146|	1	1	1	1		1	1	1	1		8	8
gi|6323162|	1	1		1	1	1	1	1	1		8	8
gi|6323329|	1	1		1	1	1	1	1	1		8	8
gi|6323568|	1	1		1	1	1	1	1	1		8	8
gi|6323723|	1	1	1	1	1	1	1	1			8	8
gi|6323739|	1	1	1	1		1	1	1	1		8	8
gi|6323843|	1	1	1	1		1	1	1	1		8	8
gi|6324187|	1	1	1	1		1	1	1	1		8	8
gi|6325132|	1	1		1	1	1	1	1	1		8	8
gi|10383778|	1		1	1	1	1	1		1	1	8	7

gi|10383801|	1		1	1	1	1	1		1	1	8	7
gi|12621476|	1	1			1	1	1	1	1	1	8	7
gi|14318465|		1	1		1	1	1	1	1	1	8	7
gi|14318466|		1	1		1	1	1	1	1	1	8	7
gi|14318475|	1	1		1	1	1		1	1	1	8	7
gi|14318485|		1	1		1	1	1	1	1	1	8	7
gi|14318498|	1		1	1	1	1	1		1	1	8	7
gi|14318551|	1		1	1	1	1	1		1	1	8	7
gi|14318569|	1		1	1	1	1	1		1	1	8	7
gi|14318577|	1	1	1	1	1		1	1		1	8	7
gi|15530184|		1	1		1	1	1	1	1	1	8	7
gi|27469358|	1		1	1	1	1	1		1	1	8	7
gi|37362625|	1		1	1	1	1	1		1	1	8	7
gi|37362640|	1		1	1	1	1	1		1	1	8	7
gi|37362708|		1	1	1	1	1	1		1	1	8	7
gi|41629687|	1	1			1	1	1	1	1	1	8	7
gi|41629689|	1		1	1	1	1	1		1	1	8	7
gi|6319295|	1		1	1	1	1	1		1	1	8	7
gi|6319312|	1		1		1	1	1	1	1	1	8	7
gi|6319492|	1		1	1	1	1	1		1	1	8	7
gi|6319498|	1	1	1	1	1			1	1	1	8	7
gi|6319511|	1	1	1	1	1			1	1	1	8	7
gi|6319553|	1	1	1		1		1	1	1	1	8	7
gi|6319622|	1	1		1	1	1		1	1	1	8	7
gi|6319623|		1	1		1	1	1	1	1	1	8	7
gi|6319647|	1		1	1	1	1	1		1	1	8	7
gi|6319660|	1	1		1	1	1	1	1		1	8	7
gi|6319697|	1		1	1	1	1	1		1	1	8	7
gi|6319737|	1		1	1	1	1	1		1	1	8	7
gi|6319753|	1	1	1	1	1	1	1			1	8	7
gi|6319758|	1		1	1	1	1	1		1	1	8	7
gi|6319776|	1		1	1	1	1	1		1	1	8	7
gi|6319795|	1	1		1	1	1	1	1		1	8	7
gi|6319847|	1		1	1	1	1	1		1	1	8	7
gi|6319911|		1	1		1	1	1	1	1	1	8	7
gi|6319919|	1	1	1	1	1	1	1			1	8	7
gi|6319976|	1		1	1	1	1	1		1	1	8	7
gi|6320001|	1		1	1	1	1	1		1	1	8	7
gi|6320091|	1	1	1	1	1	1		1		1	8	7
gi|6320107|	1	1		1	1	1	1		1	1	8	7
gi|6320152|	1		1		1	1	1	1	1	1	8	7
gi|6320163|	1		1	1	1	1	1		1	1	8	7
gi|6320220|	1		1	1	1	1	1		1	1	8	7
gi|6320314|	1	1		1	1	1	1	1		1	8	7
gi|6320330|		1	1		1	1	1	1	1	1	8	7
gi|6320345|	1		1	1	1	1	1		1	1	8	7
gi|6320349|	1	1		1	1	1		1	1	1	8	7
gi|6320378|	1		1	1	1	1	1		1	1	8	7
gi|6320422|	1		1	1	1	1	1		1	1	8	7
gi|6320424|	1		1	1	1	1	1		1	1	8	7
gi|6320452|	1		1	1	1	1	1		1	1	8	7
gi|6320459|	1		1	1	1	1	1		1	1	8	7
gi|6320464|	1	1			1	1	1	1	1	1	8	7
gi|6320530|	1		1	1	1	1	1		1	1	8	7
gi|6320531|	1	1	1		1		1	1	1	1	8	7
gi|6320580|	1		1	1	1	1	1		1	1	8	7
gi|6320581|	1		1	1	1	1	1		1	1	8	7
gi|6320582|	1	1		1	1	1	1	1		1	8	7
gi|6320586|	1	1	1	1	1	1			1	1	8	7
gi|6320605|	1	1		1	1	1		1	1	1	8	7
gi|6320725|	1		1	1	1	1	1		1	1	8	7
gi|6320738|	1		1	1	1	1	1		1	1	8	7
gi|6320794|	1	1		1	1	1		1	1	1	8	7
gi|6320856|	1		1	1	1	1	1		1	1	8	7
gi|6320879|	1		1	1	1	1	1		1	1	8	7
gi|6320889|	1	1		1	1	1		1	1	1	8	7
gi|6320892|	1		1	1	1	1	1		1	1	8	7
gi|6320913|	1	1			1	1	1	1	1	1	8	7
gi|6320931|	1		1	1	1	1	1		1	1	8	7
gi|6320952|	1		1	1	1	1	1		1	1	8	7

gi|6320999|	1		1	1	1	1	1		1	1	8	7
gi|6321003|	1		1	1	1	1	1		1	1	8	7
gi|6321246|	1	1		1	1	1	1	1		1	8	7
gi|6321323|	1		1	1	1	1	1		1	1	8	7
gi|6321403|	1		1	1	1	1	1		1	1	8	7
gi|6321437|		1	1		1	1	1	1	1	1	8	7
gi|6321452|	1		1	1	1	1	1		1	1	8	7
gi|6321468|	1		1	1	1	1	1		1	1	8	7
gi|6321473|	1	1			1	1	1	1	1	1	8	7
gi|6321484|	1		1	1	1	1	1		1	1	8	7
gi|6321495|	1		1	1	1	1	1	1		1	8	7
gi|6321496|	1		1	1	1	1	1		1	1	8	7
gi|6321504|	1		1	1	1	1	1		1	1	8	7
gi|6321535|	1		1	1	1		1	1	1	1	8	7
gi|6321575|	1		1	1	1	1	1		1	1	8	7
gi|6321602|	1		1	1	1	1	1		1	1	8	7
gi|6321608|	1	1		1	1	1	1		1	1	8	7
gi|6321626|	1		1	1	1	1	1		1	1	8	7
gi|6321627|	1			1	1	1	1	1	1	1	8	7
gi|6321655|	1	1		1	1	1	1		1	1	8	7
gi|6321656|	1		1	1	1	1	1		1	1	8	7
gi|6321672|	1		1	1	1	1	1		1	1	8	7
gi|6321679|	1		1	1	1	1	1		1	1	8	7
gi|6321723|	1		1	1	1	1	1		1	1	8	7
gi|6321726|	1		1	1	1	1	1		1	1	8	7
gi|6321731|	1		1	1	1	1	1		1	1	8	7
gi|6321756|		1	1	1	1	1	1	1		1	8	7
gi|6321770|	1	1	1		1	1		1	1	1	8	7
gi|6321784|	1	1	1		1	1	1		1	1	8	7
gi|6321785|	1		1	1	1	1	1		1	1	8	7
gi|6321804|		1		1	1	1	1	1	1	1	8	7
gi|6321842|	1		1	1	1	1	1		1	1	8	7
gi|6321899|	1		1	1	1	1	1		1	1	8	7
gi|6321905|		1	1		1	1	1	1	1	1	8	7
gi|6321924|	1		1	1	1	1	1		1	1	8	7
gi|6321937|	1	1	1		1	1	1		1	1	8	7
gi|6322021|	1		1	1	1	1	1		1	1	8	7
gi|6322032|	1		1	1	1	1	1		1	1	8	7
gi|6322060|	1		1	1	1	1	1		1	1	8	7
gi|6322063|	1		1	1	1	1		1	1	1	8	7
gi|6322156|	1		1	1	1	1	1		1	1	8	7
gi|6322166|	1		1	1	1	1	1		1	1	8	7
gi|6322241|	1		1	1	1	1	1		1	1	8	7
gi|6322245|	1		1	1	1	1	1		1	1	8	7
gi|6322253|	1		1	1	1	1	1		1	1	8	7
gi|6322268|	1			1	1	1	1	1	1	1	8	7
gi|6322327|	1	1			1	1	1	1	1	1	8	7
gi|6322351|	1		1	1	1	1	1		1	1	8	7
gi|6322360|	1		1	1	1	1	1		1	1	8	7
gi|6322364|	1	1	1		1	1		1	1	1	8	7
gi|6322405|	1		1	1	1	1	1		1	1	8	7
gi|6322509|	1	1		1	1	1		1	1	1	8	7
gi|6322536|	1		1	1	1	1	1		1	1	8	7
gi|6322586|		1	1		1	1	1	1	1	1	8	7
gi|6322588|	1		1	1	1	1	1		1	1	8	7
gi|6322592|	1			1	1	1	1	1	1	1	8	7
gi|6322603|	1			1	1	1	1	1	1	1	8	7
gi|6322746|		1	1		1	1	1	1	1	1	8	7
gi|6322797|	1	1		1	1	1		1	1	1	8	7
gi|6322832|	1		1	1	1	1	1		1	1	8	7
gi|6322885|	1		1	1	1	1	1		1	1	8	7
gi|6322907|	1		1	1	1	1	1		1	1	8	7
gi|6322945|		1	1	1	1		1	1	1	1	8	7
gi|6322975|	1	1		1	1	1	1	1		1	8	7
gi|6323002|	1	1			1	1	1	1	1	1	8	7
gi|6323045|	1		1	1	1	1	1		1	1	8	7
gi|6323086|	1	1	1	1	1	1		1		1	8	7
gi|6323103|	1	1		1	1	1	1		1	1	8	7
gi|6323117|	1			1	1	1	1	1	1	1	8	7
gi|6323195|		1		1	1	1	1	1	1	1	8	7

gi|6323205|	1		1	1	1	1	1		1	1	8	7
gi|6323220|	1		1	1	1	1	1		1	1	8	7
gi|6323222|	1		1	1	1	1	1		1	1	8	7
gi|6323238|	1		1	1	1	1	1		1	1	8	7
gi|6323274|	1		1	1	1	1	1		1	1	8	7
gi|6323302|	1	1		1	1	1		1	1	1	8	7
gi|6323314|	1	1	1	1	1	1	1			1	8	7
gi|6323341|	1		1	1	1	1	1		1	1	8	7
gi|6323346|	1		1	1	1	1	1		1	1	8	7
gi|6323391|	1		1	1	1	1	1		1	1	8	7
gi|6323460|	1		1	1	1	1	1		1	1	8	7
gi|6323504|	1	1			1	1	1	1	1	1	8	7
gi|6323514|	1		1	1	1	1	1		1	1	8	7
gi|6323559|	1		1	1	1	1	1		1	1	8	7
gi|6323570|		1	1		1	1	1	1	1	1	8	7
gi|6323572|		1	1		1	1	1	1	1	1	8	7
gi|6323580|	1	1		1	1	1	1	1		1	8	7
gi|6323581|	1		1	1	1	1	1		1	1	8	7
gi|6323614|	1		1	1	1	1	1	1		1	8	7
gi|6323620|		1	1		1	1	1	1	1	1	8	7
gi|6323644|	1		1	1	1	1	1		1	1	8	7
gi|6323679|	1		1	1	1	1	1		1	1	8	7
gi|6323691|	1			1	1	1	1	1	1	1	8	7
gi|6323722|	1	1		1	1	1		1	1	1	8	7
gi|6323729|	1	1	1		1	1		1	1	1	8	7
gi|6323832|	1	1	1		1	1	1	1		1	8	7
gi|6323836|	1		1	1	1	1	1		1	1	8	7
gi|6323857|	1		1	1	1	1	1		1	1	8	7
gi|6323864|	1	1	1		1	1		1	1	1	8	7
gi|6323887|	1	1	1		1	1	1	1		1	8	7
gi|6323890|	1		1	1	1	1	1		1	1	8	7
gi|6323891|	1		1	1	1	1	1		1	1	8	7
gi|6323930|	1	1		1	1	1	1		1	1	8	7
gi|6323934|	1		1	1	1	1	1		1	1	8	7
gi|6323948|	1		1	1	1	1		1	1	1	8	7
gi|6324024|	1	1			1	1	1	1	1	1	8	7
gi|6324043|	1		1	1	1	1		1	1	1	8	7
gi|6324086|	1		1	1	1	1	1		1	1	8	7
gi|6324091|	1		1	1	1	1	1		1	1	8	7
gi|6324261|	1		1	1	1	1	1		1	1	8	7
gi|6324267|	1			1	1	1	1	1	1	1	8	7
gi|6324273|	1		1	1	1	1	1		1	1	8	7
gi|6324289|	1		1	1		1	1	1	1	1	8	7
gi|6324344|	1	1			1	1	1	1	1	1	8	7
gi|6324392|	1	1		1	1	1	1	1		1	8	7
gi|6324416|	1		1	1	1	1	1		1	1	8	7
gi|6324427|	1	1		1	1	1	1		1	1	8	7
gi|6324470|		1		1	1	1	1	1	1	1	8	7
gi|6324480|		1	1		1	1	1	1	1	1	8	7
gi|6324501|	1		1	1	1	1	1		1	1	8	7
gi|6324504|	1		1	1	1	1	1		1	1	8	7
gi|6324516|	1	1		1	1	1	1	1		1	8	7
gi|6324532|	1		1	1	1	1	1		1	1	8	7
gi|6324561|	1		1	1	1	1	1		1	1	8	7
gi|6324577|	1		1	1	1	1		1	1	1	8	7
gi|6324992|		1		1	1	1	1	1	1	1	8	7
gi|6325026|	1		1	1	1	1	1		1	1	8	7
gi|6325031|	1		1	1	1	1	1		1	1	8	7
gi|6325043|	1		1	1	1		1	1	1	1	8	7
gi|6325060|		1	1	1	1	1	1		1	1	8	7
gi|6325073|	1	1			1	1	1	1	1	1	8	7
gi|6325081|		1	1		1	1	1	1	1	1	8	7
gi|6325083|	1		1	1	1	1	1		1	1	8	7
gi|6325146|	1		1	1	1	1	1		1	1	8	7
gi|6325170|	1	1		1	1	1	1	1		1	8	7
gi|6325188|	1		1	1	1	1	1	1		1	8	7
gi|6325213|		1	1		1	1	1	1	1	1	8	7
gi|6325219|	1		1	1	1	1	1		1	1	8	7
gi|6325242|	1		1	1	1	1	1		1	1	8	7
gi|6325251|	1	1	1	1	1	1		1		1	8	7

gi|6325390|	1	1	1		1	1	1		1	1	8	7
gi|6325412|	1		1	1	1	1	1		1	1	8	7
gi|6325447|	1			1	1	1	1	1	1	1	8	7
gi|6325449|	1	1	1	1	1			1	1	1	8	7
gi|6579193|	1	1		1	1	1		1	1	1	8	7
gi|7839181|	1	1	1	1	1	1			1	1	8	7
gi|9755337|		1	1		1	1	1	1	1	1	8	7
gi|37362614|	1			1	1	1	1	1	1		7	7
gi|37362639|	1	1	1	1		1		1	1		7	7
gi|37362667|	1	1	1		1	1		1	1		7	7
gi|41629686|	1	1	1			1	1	1	1		7	7
gi|42742174|	1	1	1			1	1	1	1		7	7
gi|50593504|		1	1		1	1	1	1	1		7	7
gi|6319278|	1		1	1	1	1	1		1		7	7
gi|6319995|	1	1	1		1	1		1	1		7	7
gi|6320565|	1	1	1	1		1		1	1		7	7
gi|6320610|		1	1	1	1	1	1	1			7	7
gi|6321442|	1	1		1	1		1	1	1		7	7
gi|6322125|	1	1	1	1		1	1		1		7	7
gi|6322611|	1	1	1	1	1	1			1		7	7
gi|6322763|	1		1	1	1	1	1		1		7	7
gi|6322791|	1	1		1		1	1	1	1		7	7
gi|6323299|	1		1	1	1	1	1		1		7	7
gi|6323603|	1		1	1	1	1		1	1		7	7
gi|6324019|	1	1	1	1			1	1	1		7	7
gi|6324242|	1	1		1	1	1	1		1		7	7
gi|6325014|	1		1	1	1	1	1		1		7	7
gi|6325193|	1	1	1	1		1		1	1		7	7
gi|10383797|	1	1	1	1				1	1	1	7	6
gi|14318478|	1	1	1	1				1	1	1	7	6
gi|14318490|	1	1	1	1	1		1			1	7	6
gi|14318537|	1		1	1	1	1	1			1	7	6
gi|14318540|	1		1	1	1	1	1			1	7	6
gi|19705600|	1	1			1	1	1	1		1	7	6
gi|22532998|	1		1	1	1	1			1	1	7	6
gi|37362692|	1		1	1	1		1		1	1	7	6
gi|41629674|	1			1	1	1	1		1	1	7	6
gi|50593425|		1			1	1	1	1	1	1	7	6
gi|6226533|	1		1	1	1	1	1			1	7	6
gi|6319276|	1			1	1	1	1		1	1	7	6
gi|6319337|	1		1	1	1	1	1			1	7	6
gi|6319372|	1		1	1	1	1	1			1	7	6
gi|6319497|	1		1		1	1	1		1	1	7	6
gi|6319678|	1		1	1	1	1	1			1	7	6
gi|6319694|		1			1	1	1	1	1	1	7	6
gi|6319709|	1		1		1	1	1		1	1	7	6
gi|6319835|		1	1		1	1	1		1	1	7	6
gi|6319961|	1		1		1	1	1		1	1	7	6
gi|6319963|	1			1	1	1	1		1	1	7	6
gi|6319971|		1	1		1	1	1		1	1	7	6
gi|6320008|	1				1	1	1	1	1	1	7	6
gi|6320060|	1				1	1	1	1	1	1	7	6
gi|6320085|	1			1	1	1	1		1	1	7	6
gi|6320109|	1			1	1	1	1		1	1	7	6
gi|6320203|	1		1	1	1	1			1	1	7	6
gi|6320346|		1			1	1	1	1	1	1	7	6
gi|6320488|	1		1	1	1	1			1	1	7	6
gi|6320526|		1	1		1	1	1		1	1	7	6
gi|6320543|	1	1			1	1	1	1		1	7	6
gi|6320544|		1			1	1	1	1	1	1	7	6
gi|6320545|		1			1	1	1	1	1	1	7	6
gi|6320579|		1	1	1	1	1	1			1	7	6
gi|6320657|	1	1			1	1	1	1		1	7	6
gi|6320689|	1			1	1	1	1		1	1	7	6
gi|6320771|	1	1		1	1	1	1			1	7	6
gi|6320824|	1	1			1		1	1	1	1	7	6
gi|6320883|		1			1	1	1	1	1	1	7	6
gi|6320890|	1			1	1	1	1		1	1	7	6
gi|6320977|	1			1	1	1	1		1	1	7	6
gi|6321217|	1			1	1	1	1		1	1	7	6

gi|6321236|	1			1	1	1	1		1	1	7	6
gi|6321285|	1			1	1	1	1		1	1	7	6
gi|6321354|		1			1	1	1	1	1	1	7	6
gi|6321371|		1			1	1	1	1	1	1	7	6
gi|6321458|		1		1	1	1	1		1	1	7	6
gi|6321492|	1		1	1	1	1	1			1	7	6
gi|6321507|	1			1	1	1	1		1	1	7	6
gi|6321550|	1		1		1	1	1		1	1	7	6
gi|6321614|		1			1	1	1	1	1	1	7	6
gi|6321620|	1		1		1	1	1		1	1	7	6
gi|6321638|	1			1	1	1	1		1	1	7	6
gi|6321760|	1		1	1	1	1	1			1	7	6
gi|6321774|	1	1			1	1	1		1	1	7	6
gi|6321821|		1			1	1	1	1	1	1	7	6
gi|6321863|		1			1	1	1	1	1	1	7	6
gi|6321929|	1			1	1	1	1		1	1	7	6
gi|6321952|		1			1	1	1	1	1	1	7	6
gi|6321984|		1			1	1	1	1	1	1	7	6
gi|6322001|		1		1	1	1	1	1		1	7	6
gi|6322062|	1	1	1		1	1	1			1	7	6
gi|6322094|	1	1			1	1	1		1	1	7	6
gi|6322103|			1	1	1	1		1	1	1	7	6
gi|6322126|		1			1	1	1	1	1	1	7	6
gi|6322183|	1			1	1	1	1	1		1	7	6
gi|6322224|				1	1	1	1	1	1	1	7	6
gi|6322324|	1		1	1	1	1	1			1	7	6
gi|6322361|	1		1	1	1	1	1			1	7	6
gi|6322373|		1			1	1	1	1	1	1	7	6
gi|6322389|	1		1	1	1		1		1	1	7	6
gi|6322415|	1		1	1	1		1		1	1	7	6
gi|6322420|	1			1	1	1	1		1	1	7	6
gi|6322424|		1			1	1	1	1	1	1	7	6
gi|6322615|		1			1	1	1	1	1	1	7	6
gi|6322739|	1	1	1	1	1				1	1	7	6
gi|6322758|	1		1	1	1	1	1			1	7	6
gi|6322788|	1			1	1	1	1		1	1	7	6
gi|6322827|	1		1	1	1		1		1	1	7	6
gi|6322881|	1			1	1	1	1		1	1	7	6
gi|6322911|	1		1	1	1	1	1			1	7	6
gi|6322916|		1		1	1	1	1		1	1	7	6
gi|6322923|		1			1	1	1	1	1	1	7	6
gi|6323085|		1			1	1	1	1	1	1	7	6
gi|6323244|	1	1			1	1	1		1	1	7	6
gi|6323271|	1		1		1	1	1		1	1	7	6
gi|6323287|	1		1	1	1	1	1			1	7	6
gi|6323403|	1			1	1	1	1		1	1	7	6
gi|6323530|	1		1		1	1	1		1	1	7	6
gi|6323542|	1				1	1	1	1	1	1	7	6
gi|6323563|	1			1	1	1	1		1	1	7	6
gi|6323592|	1		1		1	1	1		1	1	7	6
gi|6323599|	1		1	1	1	1	1			1	7	6
gi|6323680|	1			1	1	1	1		1	1	7	6
gi|6323715|				1	1	1	1	1	1	1	7	6
gi|6323717|	1	1		1	1	1	1			1	7	6
gi|6323778|	1			1	1	1	1		1	1	7	6
gi|6323800|	1	1			1	1	1		1	1	7	6
gi|6323895|	1		1		1	1		1	1	1	7	6
gi|6324037|	1	1			1	1		1	1	1	7	6
gi|6324045|	1	1			1		1	1	1	1	7	6
gi|6324080|	1	1		1	1	1		1		1	7	6
gi|6324097|	1			1	1	1		1	1	1	7	6
gi|6324108|	1			1	1	1	1		1	1	7	6
gi|6324176|	1	1		1	1	1		1		1	7	6
gi|6324204|	1		1	1	1	1	1			1	7	6
gi|6324303|		1		1	1	1	1		1	1	7	6
gi|6324356|		1			1	1	1	1	1	1	7	6
gi|6324365|		1		1	1	1	1		1	1	7	6
gi|6324453|	1		1	1	1	1	1			1	7	6
gi|6324515|	1		1	1	1	1	1			1	7	6
gi|6324534|				1	1	1	1	1	1	1	7	6

gi|6324546|		1	1	1	1		1	1		1	7	6
gi|6325034|		1			1	1	1	1	1	1	7	6
gi|6325057|		1			1	1	1	1	1	1	7	6
gi|6325065|		1	1		1	1	1	1		1	7	6
gi|6325067|		1			1	1	1	1	1	1	7	6
gi|6325092|		1			1	1	1	1	1	1	7	6
gi|6325169|	1				1	1	1	1	1	1	7	6
gi|6325173|	1			1	1	1	1		1	1	7	6
gi|6325211|	1	1	1		1	1	1			1	7	6
gi|6325237|			1	1	1	1	1		1	1	7	6
gi|6325254|		1		1	1	1		1	1	1	7	6
gi|6325352|	1		1	1	1	1	1			1	7	6
gi|6325420|	1			1	1	1	1		1	1	7	6
gi|6325442|	1		1	1	1	1	1			1	7	6
gi|6325450|	1	1			1	1	1	1		1	7	6
gi|7839183|				1	1	1	1	1	1	1	7	6
gi|9755339|	1	1	1	1	1				1	1	7	6
gi|10383787|	1	1		1		1	1	1			6	6
gi|14318477|	1	1	1	1				1	1		6	6
gi|6319602|	1	1	1	1				1	1		6	6
gi|6319951|		1			1	1	1	1	1		6	6
gi|6319958|		1			1	1	1	1	1		6	6
gi|6320030|	1	1	1	1				1	1		6	6
gi|6320351|		1			1	1	1	1	1		6	6
gi|6320388|	1	1		1		1		1	1		6	6
gi|6320643|	1		1			1	1	1	1		6	6
gi|6320648|	1		1	1	1	1	1				6	6
gi|6320680|	1	1	1	1				1	1		6	6
gi|6320965|				1	1	1	1	1	1		6	6
gi|6321031|	1	1	1	1				1	1		6	6
gi|6321334|	1	1	1	1				1	1		6	6
gi|6321559|	1	1	1	1				1	1		6	6
gi|6321719|			1	1	1	1	1		1		6	6
gi|6322035|	1		1	1		1	1		1		6	6
gi|6322077|		1			1	1	1	1	1		6	6
gi|6322190|		1			1	1	1	1	1		6	6
gi|6322227|	1	1	1	1				1	1		6	6
gi|6322695|	1	1		1		1	1		1		6	6
gi|6322951|	1	1	1	1				1	1		6	6
gi|6323156|		1			1	1	1	1	1		6	6
gi|6323512|	1	1				1	1	1	1		6	6
gi|6323537|	1	1	1	1				1	1		6	6
gi|6323540|		1	1	1	1			1	1		6	6
gi|6323553|	1	1				1	1	1	1		6	6
gi|6323654|	1		1	1		1	1		1		6	6
gi|6323917|	1	1	1	1				1	1		6	6
gi|6323998|		1			1	1	1	1	1		6	6
gi|6324111|	1	1			1	1	1		1		6	6
gi|6325356|	1	1	1	1				1	1		6	6
gi|14318464|	1			1	1		1		1	1	6	5
gi|14318494|	1	1			1		1		1	1	6	5
gi|14318524|			1		1	1	1		1	1	6	5
gi|14318566|				1	1	1	1		1	1	6	5
gi|27808714|	1			1	1	1	1			1	6	5
gi|33438870|	1	1		1	1	1				1	6	5
gi|37362670|				1	1	1	1		1	1	6	5
gi|41629671|	1			1	1	1	1			1	6	5
gi|41629672|	1				1	1	1		1	1	6	5
gi|42759852|	1	1			1	1	1			1	6	5
gi|50593118|		1			1	1	1	1		1	6	5
gi|6319451|			1	1	1		1		1	1	6	5
gi|6319454|	1				1	1	1		1	1	6	5
gi|6319557|	1			1	1	1	1			1	6	5
gi|6319587|			1		1	1	1		1	1	6	5
gi|6319765|			1		1	1	1		1	1	6	5
gi|6319854|	1				1	1	1		1	1	6	5
gi|6320097|	1			1	1	1	1			1	6	5
gi|6320292|			1		1	1	1		1	1	6	5
gi|6320319|					1	1	1	1	1	1	6	5
gi|6320326|	1				1	1	1		1	1	6	5

gi|6320344|				1	1	1	1		1	1	6	5
gi|6320596|			1		1	1	1		1	1	6	5
gi|6320597|				1	1	1	1		1	1	6	5
gi|6320939|				1	1	1	1		1	1	6	5
gi|6321257|		1			1	1	1		1	1	6	5
gi|6321356|		1			1	1	1	1		1	6	5
gi|6321388|		1			1	1		1	1	1	6	5
gi|6321389|	1			1	1	1			1	1	6	5
gi|6321426|		1			1	1	1		1	1	6	5
gi|6321601|	1				1	1		1	1	1	6	5
gi|6321975|	1			1	1	1	1			1	6	5
gi|6322020|	1				1	1	1		1	1	6	5
gi|6322186|				1	1	1	1		1	1	6	5
gi|6322239|	1				1	1	1		1	1	6	5
gi|6322266|	1	1		1		1	1			1	6	5
gi|6322480|	1		1		1	1	1			1	6	5
gi|6322598|			1		1	1	1		1	1	6	5
gi|6322768|				1	1	1	1		1	1	6	5
gi|6322803|		1	1	1	1				1	1	6	5
gi|6322812|	1	1			1	1			1	1	6	5
gi|6322820|	1	1				1	1		1	1	6	5
gi|6323052|				1	1		1	1	1	1	6	5
gi|6323074|	1				1	1	1		1	1	6	5
gi|6323134|	1	1		1	1	1				1	6	5
gi|6323163|	1	1			1			1	1	1	6	5
gi|6323180|	1	1		1	1			1		1	6	5
gi|6323270|		1			1	1		1	1	1	6	5
gi|6323382|		1			1	1		1	1	1	6	5
gi|6323428|	1				1	1	1		1	1	6	5
gi|6323669|	1		1	1	1				1	1	6	5
gi|6324188|			1		1	1	1		1	1	6	5
gi|6324266|	1			1	1	1			1	1	6	5
gi|6324293|	1				1	1	1		1	1	6	5
gi|6324302|	1				1	1	1		1	1	6	5
gi|6324324|		1			1	1	1		1	1	6	5
gi|6324393|	1				1	1	1		1	1	6	5
gi|6324491|			1	1	1	1	1			1	6	5
gi|6324590|			1		1	1	1		1	1	6	5
gi|6325075|	1			1	1		1		1	1	6	5
gi|6325119|				1	1	1	1		1	1	6	5
gi|6325128|		1			1	1		1	1	1	6	5
gi|6325197|		1			1	1	1		1	1	6	5
gi|6325280|	1				1	1	1		1	1	6	5
gi|27808704|		1			1	1	1	1			5	5
gi|37362635|	1	1	1					1	1		5	5
gi|6226520|	1	1	1		1			1			5	5
gi|6226529|	1	1	1		1			1			5	5
gi|6319267|		1			1	1	1	1			5	5
gi|6319322|	1				1	1	1		1		5	5
gi|6320026|				1		1	1	1	1		5	5
gi|6320202|	1	1				1	1		1		5	5
gi|6320279|	1	1		1				1	1		5	5
gi|6320386|	1				1	1	1		1		5	5
gi|6320616|	1			1		1	1		1		5	5
gi|6320673|		1				1	1	1	1		5	5
gi|6320719|			1	1		1	1		1		5	5
gi|6320908|	1	1	1					1	1		5	5
gi|6320956|		1				1	1	1	1		5	5
gi|6321247|	1		1			1	1		1		5	5
gi|6321439|	1	1	1					1	1		5	5
gi|6321790|	1	1	1					1	1		5	5
gi|6321818|		1			1	1	1		1		5	5
gi|6322402|	1			1		1	1		1		5	5
gi|6322562|	1		1		1	1			1		5	5
gi|6322724|	1			1		1	1		1		5	5
gi|6322848|	1	1	1					1	1		5	5
gi|6323138|	1	1		1				1	1		5	5
gi|6323144|	1	1		1				1	1		5	5
gi|6323404|				1		1	1	1	1		5	5
gi|6323596|		1	1	1				1	1		5	5

gi|6323703|	1	1		1				1	1		5	5
gi|6323765|	1		1	1	1				1		5	5
gi|6323786|	1	1		1				1	1		5	5
gi|6323980|	1	1	1					1	1		5	5
gi|6324431|		1			1	1	1		1		5	5
gi|6324486|	1	1	1					1	1		5	5
gi|6325049|		1			1		1	1	1		5	5
gi|6325245|		1			1	1		1	1		5	5
gi|6325263|	1	1	1					1	1		5	5
gi|6325319|	1	1	1					1	1		5	5
gi|6325396|	1	1		1				1	1		5	5
gi|6325424|		1				1	1	1	1		5	5
gi|6325425|	1	1		1				1	1		5	5
gi|14318463|		1			1	1	1			1	5	4
gi|27808713|					1	1	1		1	1	5	4
gi|6319410|	1		1					1	1	1	5	4
gi|6319436|					1	1		1	1	1	5	4
gi|6319708|					1	1	1		1	1	5	4
gi|6319734|			1		1	1	1			1	5	4
gi|6319893|					1	1	1		1	1	5	4
gi|6319901|					1	1	1		1	1	5	4
gi|6319904|			1		1	1			1	1	5	4
gi|6320003|	1				1			1	1	1	5	4
gi|6320112|		1				1	1		1	1	5	4
gi|6320216|		1				1		1	1	1	5	4
gi|6320253|		1			1			1	1	1	5	4
gi|6320322|			1		1	1		1		1	5	4
gi|6320463|		1			1		1		1	1	5	4
gi|6320542|	1				1	1			1	1	5	4
gi|6320559|			1				1	1	1	1	5	4
gi|6320600|					1	1	1		1	1	5	4
gi|6320840|			1		1	1	1			1	5	4
gi|6320862|			1		1	1	1			1	5	4
gi|6320895|				1	1	1	1			1	5	4
gi|6320981|					1	1	1		1	1	5	4
gi|6320987|					1	1	1		1	1	5	4
gi|6321016|		1			1			1	1	1	5	4
gi|6321196|					1	1	1		1	1	5	4
gi|6321282|					1	1	1		1	1	5	4
gi|6321524|		1			1			1	1	1	5	4
gi|6321629|					1	1	1		1	1	5	4
gi|6321673|					1	1	1		1	1	5	4
gi|6322087|					1	1	1		1	1	5	4
gi|6322127|					1	1	1		1	1	5	4
gi|6322158|		1			1	1			1	1	5	4
gi|6322220|	1				1	1			1	1	5	4
gi|6322363|					1	1	1		1	1	5	4
gi|6322485|			1		1	1	1			1	5	4
gi|6322533|					1	1	1		1	1	5	4
gi|6322607|	1	1						1	1	1	5	4
gi|6322880|					1	1	1		1	1	5	4
gi|6322906|		1			1			1	1	1	5	4
gi|6322943|	1		1	1		1				1	5	4
gi|6323073|		1			1			1	1	1	5	4
gi|6323261|			1		1	1	1			1	5	4
gi|6323616|					1		1	1	1	1	5	4
gi|6323682|		1			1	1	1			1	5	4
gi|6323719|					1	1	1		1	1	5	4
gi|6323744|					1	1	1		1	1	5	4
gi|6323858|					1	1	1		1	1	5	4
gi|6323927|					1	1	1		1	1	5	4
gi|6324076|	1				1	1		1		1	5	4
gi|6324148|					1	1	1		1	1	5	4
gi|6324288|	1			1	1	1				1	5	4
gi|6324434|					1	1	1		1	1	5	4
gi|6324576|					1	1	1		1	1	5	4
gi|6324991|		1		1	1	1				1	5	4
gi|6325066|			1			1	1		1	1	5	4
gi|6325154|			1		1	1	1			1	5	4
gi|6325241|					1	1	1		1	1	5	4

gi|6325289|	1			1			1		1	1	5	4
gi|10383755|			1	1			1		1		4	4
gi|10383772|	1	1						1	1		4	4
gi|37362654|					1		1	1	1		4	4
gi|6226521|	1	1	1		1						4	4
gi|6226527|	1	1			1			1			4	4
gi|6226530|	1	1	1		1						4	4
gi|6226531|	1	1	1		1						4	4
gi|6226532|	1	1	1		1						4	4
gi|6226540|	1	1	1		1						4	4
gi|6226542|	1	1	1		1						4	4
gi|6319351|	1					1	1		1		4	4
gi|6319569|	1					1	1		1		4	4
gi|6319653|	1	1						1	1		4	4
gi|6319725|	1	1						1	1		4	4
gi|6319775|			1				1	1	1		4	4
gi|6319954|		1	1					1	1		4	4
gi|6319991|		1		1				1	1		4	4
gi|6320073|			1	1				1	1		4	4
gi|6320131|	1				1		1		1		4	4
gi|6320221|		1		1				1	1		4	4
gi|6320332|	1	1						1	1		4	4
gi|6320369|	1	1		1					1		4	4
gi|6320472|	1			1	1				1		4	4
gi|6320482|		1	1					1	1		4	4
gi|6320527|	1		1	1					1		4	4
gi|6320587|	1		1	1					1		4	4
gi|6320750|		1	1					1	1		4	4
gi|6320851|	1					1	1		1		4	4
gi|6320893|	1	1						1	1		4	4
gi|6320935|	1	1						1	1		4	4
gi|6321000|	1			1		1			1		4	4
gi|6321181|	1		1	1					1		4	4
gi|6321346|	1	1		1					1		4	4
gi|6321401|	1		1	1					1		4	4
gi|6321429|	1	1						1	1		4	4
gi|6321517|	1					1	1		1		4	4
gi|6321548|		1				1		1	1		4	4
gi|6321609|		1	1					1	1		4	4
gi|6321853|		1				1	1		1		4	4
gi|6321973|		1	1					1	1		4	4
gi|6322075|	1	1						1	1		4	4
gi|6322121|					1	1	1		1		4	4
gi|6322222|	1	1	1					1			4	4
gi|6322368|	1		1	1		1					4	4
gi|6322381|	1		1	1					1		4	4
gi|6322476|	1	1						1	1		4	4
gi|6322543|		1				1		1	1		4	4
gi|6322560|		1				1		1	1		4	4
gi|6322602|	1	1						1	1		4	4
gi|6322620|		1	1					1	1		4	4
gi|6322708|	1		1	1					1		4	4
gi|6322779|		1	1	1					1		4	4
gi|6322922|	1	1						1	1		4	4
gi|6322966|	1	1		1				1			4	4
gi|6323051|	1			1				1	1		4	4
gi|6323129|					1	1	1		1		4	4
gi|6323157|	1	1		1					1		4	4
gi|6323362|	1	1		1					1		4	4
gi|6323531|		1	1					1	1		4	4
gi|6323635|		1	1					1	1		4	4
gi|6323746|	1	1		1				1			4	4
gi|6323839|					1	1	1		1		4	4
gi|6323859|				1	1		1		1		4	4
gi|6323878|						1	1	1	1		4	4
gi|6323937|			1			1	1	1			4	4
gi|6323983|	1	1		1				1			4	4
gi|6324021|					1	1		1	1		4	4
gi|6324070|		1	1	1					1		4	4
gi|6324100|	1			1				1	1		4	4

gi|6324212|	1	1	1					1			4	4
gi|6324386|	1			1		1			1		4	4
gi|6324399|		1	1					1	1		4	4
gi|6324421|		1	1					1	1		4	4
gi|6324429|	1	1						1	1		4	4
gi|6324517|	1	1						1	1		4	4
gi|6324566|	1			1				1	1		4	4
gi|6324997|	1	1						1	1		4	4
gi|6325086|		1	1					1	1		4	4
gi|6325088|	1			1		1			1		4	4
gi|6325112|			1	1			1		1		4	4
gi|6325283|	1			1		1	1				4	4
gi|6325312|			1		1	1			1		4	4
gi|6325378|	1	1						1	1		4	4
gi|10383753|					1	1	1			1	4	3
gi|10383785|					1	1	1			1	4	3
gi|27469361|					1		1		1	1	4	3
gi|6319257|	1				1	1				1	4	3
gi|6319616|				1	1		1			1	4	3
gi|6320024|			1		1	1				1	4	3
gi|6320233|		1				1	1			1	4	3
gi|6320513|					1	1			1	1	4	3
gi|6320660|					1	1			1	1	4	3
gi|6320775|					1	1			1	1	4	3
gi|6320919|					1	1	1			1	4	3
gi|6322047|			1		1				1	1	4	3
gi|6322144|					1	1	1			1	4	3
gi|6322187|					1	1			1	1	4	3
gi|6322538|					1	1	1			1	4	3
gi|6322666|					1	1			1	1	4	3
gi|6323171|	1		1				1			1	4	3
gi|6323279|			1			1			1	1	4	3
gi|6323435|					1	1			1	1	4	3
gi|6323883|			1		1				1	1	4	3
gi|6323949|		1			1			1		1	4	3
gi|6323975|	1	1							1	1	4	3
gi|6324174|		1			1	1				1	4	3
gi|6324245|					1	1			1	1	4	3
gi|6325050|			1			1			1	1	4	3
gi|6325322|			1	1					1	1	4	3
gi|6325349|		1			1			1		1	4	3
gi|6325402|			1		1	1				1	4	3
gi|6325421|					1	1	1			1	4	3
gi|10383761|		1						1	1		3	3
gi|10383770|	1	1						1			3	3
gi|14318460|		1						1	1		3	3
gi|14318520|	1	1						1			3	3
gi|20336767|	1	1						1			3	3
gi|37362656|	1	1							1		3	3
gi|37362661|		1						1	1		3	3
gi|37362687|		1						1	1		3	3
gi|42742256|		1						1	1		3	3
gi|6226519|	1		1		1						3	3
gi|6226522|	1		1		1						3	3
gi|6226523|	1		1		1						3	3
gi|6226524|	1		1		1						3	3
gi|6319323|	1	1						1			3	3
gi|6319326|		1						1	1		3	3
gi|6319365|			1		1	1					3	3
gi|6319369|	1	1						1			3	3
gi|6319429|		1						1	1		3	3
gi|6319438|		1						1	1		3	3
gi|6319468|	1	1						1			3	3
gi|6319486|	1	1						1			3	3
gi|6319495|		1						1	1		3	3
gi|6319526|		1						1	1		3	3
gi|6319577|		1						1	1		3	3
gi|6319733|		1						1	1		3	3
gi|6319764|		1						1	1		3	3
gi|6319837|		1						1	1		3	3

gi|6319849|		1						1	1		3	3
gi|6319902|		1			1		1				3	3
gi|6319949|	1	1						1			3	3
gi|6320002|		1	1					1			3	3
gi|6320049|				1				1	1		3	3
gi|6320071|		1						1	1		3	3
gi|6320075|		1						1	1		3	3
gi|6320118|		1						1	1		3	3
gi|6320123|		1						1	1		3	3
gi|6320138|		1						1	1		3	3
gi|6320210|		1						1	1		3	3
gi|6320235|		1						1	1		3	3
gi|6320379|		1						1	1		3	3
gi|6320443|		1						1	1		3	3
gi|6320454|			1					1	1		3	3
gi|6320497|		1						1	1		3	3
gi|6320561|		1						1	1		3	3
gi|6320588|		1						1	1		3	3
gi|6320619|		1				1		1			3	3
gi|6320659|		1		1		1					3	3
gi|6320695|		1						1	1		3	3
gi|6320714|	1			1					1		3	3
gi|6320723|		1	1					1			3	3
gi|6320833|			1	1					1		3	3
gi|6320901|		1						1	1		3	3
gi|6320936|		1						1	1		3	3
gi|6320959|	1		1					1			3	3
gi|6320985|	1	1						1			3	3
gi|6320990|		1						1	1		3	3
gi|6321008|	1	1						1			3	3
gi|6321021|						1	1		1		3	3
gi|6321182|		1						1	1		3	3
gi|6321193|	1							1	1		3	3
gi|6321214|		1						1	1		3	3
gi|6321281|		1	1						1		3	3
gi|6321290|		1						1	1		3	3
gi|6321305|	1	1						1			3	3
gi|6321307|			1					1	1		3	3
gi|6321361|		1						1	1		3	3
gi|6321412|		1						1	1		3	3
gi|6321428|		1						1	1		3	3
gi|6321469|		1						1	1		3	3
gi|6321583|		1						1	1		3	3
gi|6321593|		1						1	1		3	3
gi|6321617|		1						1	1		3	3
gi|6321644|		1						1	1		3	3
gi|6321649|		1						1	1		3	3
gi|6321725|		1						1	1		3	3
gi|6321729|		1						1	1		3	3
gi|6321765|		1						1	1		3	3
gi|6321766|	1		1	1							3	3
gi|6321907|	1		1						1		3	3
gi|6321938|	1			1					1		3	3
gi|6321941|		1						1	1		3	3
gi|6321969|			1	1					1		3	3
gi|6321983|		1						1	1		3	3
gi|6322003|					1	1		1			3	3
gi|6322010|	1	1						1			3	3
gi|6322026|		1	1					1			3	3
gi|6322027|		1	1					1			3	3
gi|6322029|		1						1	1		3	3
gi|6322044|		1						1	1		3	3
gi|6322111|							1	1	1		3	3
gi|6322119|		1						1	1		3	3
gi|6322139|		1						1	1		3	3
gi|6322169|		1						1	1		3	3
gi|6322180|		1						1	1		3	3
gi|6322219|		1						1	1		3	3
gi|6322243|		1						1	1		3	3
gi|6322249|		1						1	1		3	3

gi|6322329|					1	1	1				3	3
gi|6322332|		1						1	1		3	3
gi|6322347|	1	1						1			3	3
gi|6322367|		1						1	1		3	3
gi|6322460|		1	1					1			3	3
gi|6322487|	1	1						1			3	3
gi|6322489|	1	1						1			3	3
gi|6322576|		1						1	1		3	3
gi|6322599|	1	1						1			3	3
gi|6322644|		1				1			1		3	3
gi|6322674|		1						1	1		3	3
gi|6322718|		1	1						1		3	3
gi|6322756|		1						1	1		3	3
gi|6322782|	1	1							1		3	3
gi|6322790|	1	1							1		3	3
gi|6322795|			1			1			1		3	3
gi|6322817|		1						1	1		3	3
gi|6322825|		1						1	1		3	3
gi|6322845|		1		1					1		3	3
gi|6322903|		1						1	1		3	3
gi|6322929|		1						1	1		3	3
gi|6322942|		1						1	1		3	3
gi|6323034|		1						1	1		3	3
gi|6323064|	1	1						1			3	3
gi|6323159|		1						1	1		3	3
gi|6323173|		1						1	1		3	3
gi|6323218|		1		1				1			3	3
gi|6323266|		1						1	1		3	3
gi|6323316|		1						1	1		3	3
gi|6323321|		1						1	1		3	3
gi|6323357|	1		1						1		3	3
gi|6323374|		1						1	1		3	3
gi|6323387|	1	1						1			3	3
gi|6323393|		1						1	1		3	3
gi|6323452|		1						1	1		3	3
gi|6323472|		1						1	1		3	3
gi|6323515|		1						1	1		3	3
gi|6323517|					1		1		1		3	3
gi|6323597|	1	1						1			3	3
gi|6323601|	1	1						1			3	3
gi|6323638|		1						1	1		3	3
gi|6323684|	1	1						1			3	3
gi|6323688|	1	1						1			3	3
gi|6323694|	1	1						1			3	3
gi|6323707|		1						1	1		3	3
gi|6323742|		1						1	1		3	3
gi|6323743|		1						1	1		3	3
gi|6323785|		1						1	1		3	3
gi|6323794|		1						1	1		3	3
gi|6323801|					1	1	1				3	3
gi|6323831|		1						1	1		3	3
gi|6323847|		1						1	1		3	3
gi|6323876|		1						1	1		3	3
gi|6323905|		1	1					1			3	3
gi|6323906|		1						1	1		3	3
gi|6323922|		1						1	1		3	3
gi|6323939|		1						1	1		3	3
gi|6323961|	1		1						1		3	3
gi|6323965|		1						1	1		3	3
gi|6323973|		1						1	1		3	3
gi|6323996|		1						1	1		3	3
gi|6324008|		1						1	1		3	3
gi|6324065|		1						1	1		3	3
gi|6324098|		1						1	1		3	3
gi|6324117|		1						1	1		3	3
gi|6324132|		1	1						1		3	3
gi|6324137|			1	1					1		3	3
gi|6324206|		1						1	1		3	3
gi|6324213|	1		1						1		3	3
gi|6324225|		1						1	1		3	3

gi|6324230|		1						1	1		3	3
gi|6324248|		1						1	1		3	3
gi|6324264|			1					1	1		3	3
gi|6324272|		1						1	1		3	3
gi|6324287|		1						1	1		3	3
gi|6324292|		1						1	1		3	3
gi|6324296|		1						1	1		3	3
gi|6324335|		1						1	1		3	3
gi|6324395|		1						1	1		3	3
gi|6324484|						1	1		1		3	3
gi|6324488|		1						1	1		3	3
gi|6324508|		1						1	1		3	3
gi|6324518|		1			1				1		3	3
gi|6324573|		1						1	1		3	3
gi|6324982|	1	1						1			3	3
gi|6325032|		1						1	1		3	3
gi|6325041|	1			1					1		3	3
gi|6325042|		1						1	1		3	3
gi|6325244|		1						1	1		3	3
gi|6325317|		1						1	1		3	3
gi|6325384|		1						1	1		3	3
gi|6325446|			1				1	1			3	3
gi|6325452|		1						1	1		3	3
gi|7839150|	1	1						1			3	3
gi|7839152|	1	1						1			3	3
gi|7839154|	1	1						1			3	3
gi|7839158|	1	1						1			3	3
gi|7839160|	1	1						1			3	3
gi|7839162|	1	1						1			3	3
gi|7839164|	1	1						1			3	3
gi|7839171|	1	1						1			3	3
gi|7839173|	1	1						1			3	3
gi|7839176|	1	1						1			3	3
gi|7839178|	1	1						1			3	3
gi|7839180|	1	1						1			3	3
gi|7839185|	1	1						1			3	3
gi|7839187|	1	1						1			3	3
gi|7839190|	1	1						1			3	3
gi|7839194|	1	1						1			3	3
gi|7839197|	1	1						1			3	3
gi|7839205|	1	1						1			3	3
gi|7839207|	1	1						1			3	3
gi|7839209|	1	1						1			3	3
gi|7839211|	1	1						1			3	3
gi|33438857|					1	1				1	3	2
gi|37362647|						1			1	1	3	2
gi|52788569|						1			1	1	3	2
gi|52788571|						1			1	1	3	2
gi|6319899|				1					1	1	3	2
gi|6320492|					1		1			1	3	2
gi|6320627|							1		1	1	3	2
gi|6321722|					1	1				1	3	2
gi|6323311|	1				1					1	3	2
gi|6323909|					1	1				1	3	2
gi|6324458|	1								1	1	3	2
gi|6325179|					1				1	1	3	2
gi|6325385|					1	1				1	3	2
gi|9755328|					1	1				1	3	2
gi|14318459|		1						1			2	2
gi|14318462|		1						1			2	2
gi|14318491|		1						1			2	2
gi|14318506|			1						1		2	2
gi|14318518|								1	1		2	2
gi|14318525|								1	1		2	2
gi|14318529|		1						1			2	2
gi|16740526|	1								1		2	2
gi|27808706|				1		1					2	2
gi|37362621|								1	1		2	2
gi|37362706|		1						1			2	2
gi|41629668|								1	1		2	2

gi|42742058|			1						1		2	2
gi|6319299|						1			1		2	2
gi|6319433|	1								1		2	2
gi|6319452|			1						1		2	2
gi|6319504|		1						1			2	2
gi|6319523|							1		1		2	2
gi|6319567|						1			1		2	2
gi|6319568|	1								1		2	2
gi|6319591|				1					1		2	2
gi|6319650|	1								1		2	2
gi|6319677|			1						1		2	2
gi|6319723|		1		1							2	2
gi|6319770|		1							1		2	2
gi|6319810|				1					1		2	2
gi|6319940|		1						1			2	2
gi|6319941|								1	1		2	2
gi|6320019|		1							1		2	2
gi|6320045|		1						1			2	2
gi|6320157|		1							1		2	2
gi|6320180|	1								1		2	2
gi|6320229|							1		1		2	2
gi|6320246|			1						1		2	2
gi|6320256|		1						1			2	2
gi|6320258|						1			1		2	2
gi|6320321|		1						1			2	2
gi|6320347|		1							1		2	2
gi|6320372|	1								1		2	2
gi|6320406|			1						1		2	2
gi|6320441|		1		1							2	2
gi|6320467|								1	1		2	2
gi|6320496|		1							1		2	2
gi|6320560|		1						1			2	2
gi|6320622|	1			1							2	2
gi|6320697|						1			1		2	2
gi|6320742|	1								1		2	2
gi|6320857|		1							1		2	2
gi|6320896|		1							1		2	2
gi|6320958|		1							1		2	2
gi|6321204|		1							1		2	2
gi|6321226|		1						1			2	2
gi|6321251|						1			1		2	2
gi|6321283|		1							1		2	2
gi|6321294|		1							1		2	2
gi|6321304|		1						1			2	2
gi|6321308|								1	1		2	2
gi|6321324|		1						1			2	2
gi|6321399|			1						1		2	2
gi|6321420|		1						1			2	2
gi|6321566|			1					1			2	2
gi|6321577|		1							1		2	2
gi|6321622|				1					1		2	2
gi|6321628|		1						1			2	2
gi|6321771|		1							1		2	2
gi|6321811|			1						1		2	2
gi|6321824|		1						1			2	2
gi|6321830|			1		1						2	2
gi|6321839|								1	1		2	2
gi|6321892|		1							1		2	2
gi|6321898|		1						1			2	2
gi|6321940|			1						1		2	2
gi|6322011|	1								1		2	2
gi|6322070|								1	1		2	2
gi|6322118|		1						1			2	2
gi|6322229|	1								1		2	2
gi|6322263|			1						1		2	2
gi|6322316|		1						1			2	2
gi|6322369|	1								1		2	2
gi|6322406|		1						1			2	2
gi|6322550|								1	1		2	2
gi|6322613|		1						1			2	2

gi|6322673|		1							1		2	2
gi|6322725|								1	1		2	2
gi|6322735|			1						1		2	2
gi|6322764|					1				1		2	2
gi|6322919|		1						1			2	2
gi|6322933|	1								1		2	2
gi|6322950|		1						1			2	2
gi|6322972|			1						1		2	2
gi|6322985|			1						1		2	2
gi|6323024|								1	1		2	2
gi|6323032|		1							1		2	2
gi|6323041|		1						1			2	2
gi|6323155|		1							1		2	2
gi|6323208|		1	1								2	2
gi|6323249|		1						1			2	2
gi|6323267|	1								1		2	2
gi|6323315|								1	1		2	2
gi|6323331|								1	1		2	2
gi|6323443|				1					1		2	2
gi|6323455|	1								1		2	2
gi|6323458|	1								1		2	2
gi|6323681|	1								1		2	2
gi|6323700|			1						1		2	2
gi|6323718|		1						1			2	2
gi|6323761|		1		1							2	2
gi|6323806|		1							1		2	2
gi|6323842|		1						1			2	2
gi|6323897|		1							1		2	2
gi|6323941|	1								1		2	2
gi|6323970|	1								1		2	2
gi|6323984|	1								1		2	2
gi|6323995|		1						1			2	2
gi|6324017|								1	1		2	2
gi|6324156|	1	1									2	2
gi|6324200|			1						1		2	2
gi|6324317|		1						1			2	2
gi|6324361|		1							1		2	2
gi|6324436|			1						1		2	2
gi|6324503|						1			1		2	2
gi|6324975|	1								1		2	2
gi|6325131|		1							1		2	2
gi|6325299|		1							1		2	2
gi|6325414|		1							1		2	2
gi|6325451|					1				1		2	2
gi|6325455|		1							1		2	2
gi|37362636|					1					1	2	1
gi|6319657|									1	1	2	1
gi|6319820|			1							1	2	1
gi|6320209|					1					1	2	1
gi|6322449|					1					1	2	1
gi|6323915|					1					1	2	1
gi|6324205|									1	1	2	1
gi|10383750|									1		1	1
gi|10383760|									1		1	1
gi|10383766|									1		1	1
gi|10383782|							1				1	1
gi|10383791|									1		1	1
gi|10383795|				1							1	1
gi|10383798|									1		1	1
gi|10383802|									1		1	1
gi|10383803|									1		1	1
gi|12621478|									1		1	1
gi|13129164|									1		1	1
gi|14318461|									1		1	1
gi|14318467|									1		1	1
gi|14318469|									1		1	1
gi|14318544|									1		1	1
gi|14318548|									1		1	1
gi|14318558|									1		1	1
gi|14627173|									1		1	1

gi|16198342|						1	1	1
gi|27808701|						1	1	1
gi|37362610|						1	1	1
gi|37362624|						1	1	1
gi|37362638|				1			1	1
gi|37362690|						1	1	1
gi|37362700|				1			1	1
gi|37362705|						1	1	1
gi|41629669|						1	1	1
gi|42742288|						1	1	1
gi|50593216|						1	1	1
gi|52788576|						1	1	1
gi|52788579|						1	1	1
gi|52788581|						1	1	1
gi|52788584|						1	1	1
gi|6319250|						1	1	1
gi|6319265|						1	1	1
gi|6319277|						1	1	1
gi|6319283|						1	1	1
gi|6319286|						1	1	1
gi|6319310|						1	1	1
gi|6319362|						1	1	1
gi|6319374|						1	1	1
gi|6319386|						1	1	1
gi|6319411|						1	1	1
gi|6319420|						1	1	1
gi|6319439|						1	1	1
gi|6319456|						1	1	1
gi|6319459|						1	1	1
gi|6319464|						1	1	1
gi|6319476|						1	1	1
gi|6319480|						1	1	1
gi|6319507|						1	1	1
gi|6319512|						1	1	1
gi|6319517|						1	1	1
gi|6319528|						1	1	1
gi|6319530|						1	1	1
gi|6319542|						1	1	1
gi|6319543|						1	1	1
gi|6319546|						1	1	1
gi|6319552|						1	1	1
gi|6319570|						1	1	1
gi|6319595|						1	1	1
gi|6319608|						1	1	1
gi|6319613|						1	1	1
gi|6319629|						1	1	1
gi|6319634|					1		1	1
gi|6319637|						1	1	1
gi|6319656|						1	1	1
gi|6319676|						1	1	1
gi|6319682|						1	1	1
gi|6319684|						1	1	1
gi|6319690|						1	1	1
gi|6319691|						1	1	1
gi|6319701|						1	1	1
gi|6319726|						1	1	1
gi|6319742|						1	1	1
gi|6319748|						1	1	1
gi|6319754|			1				1	1
gi|6319763|	1						1	1
gi|6319774|						1	1	1
gi|6319783|						1	1	1
gi|6319801|						1	1	1
gi|6319802|						1	1	1
gi|6319836|		1					1	1
gi|6319855|						1	1	1
gi|6319865|						1	1	1
gi|6319869|						1	1	1
gi|6319876|						1	1	1
gi|6319912|						1	1	1

<table>
<tr><th></th><th></th><th></th><th></th><th></th><th></th><th></th><th></th><th></th></tr>
<tr><td>gi|6319931|</td><td></td><td></td><td></td><td></td><td></td><td>1</td><td>1</td><td>1</td></tr>
<tr><td>gi|6319943|</td><td></td><td></td><td></td><td></td><td></td><td>1</td><td>1</td><td>1</td></tr>
<tr><td>gi|6319945|</td><td></td><td></td><td></td><td></td><td></td><td>1</td><td>1</td><td>1</td></tr>
<tr><td>gi|6319950|</td><td></td><td></td><td></td><td></td><td></td><td>1</td><td>1</td><td>1</td></tr>
<tr><td>gi|6319957|</td><td></td><td></td><td></td><td></td><td></td><td>1</td><td>1</td><td>1</td></tr>
<tr><td>gi|6319966|</td><td></td><td></td><td></td><td></td><td></td><td>1</td><td>1</td><td>1</td></tr>
<tr><td>gi|6319977|</td><td></td><td></td><td></td><td></td><td></td><td>1</td><td>1</td><td>1</td></tr>
<tr><td>gi|6319986|</td><td></td><td></td><td></td><td></td><td></td><td>1</td><td>1</td><td>1</td></tr>
<tr><td>gi|6319988|</td><td></td><td></td><td></td><td></td><td></td><td>1</td><td>1</td><td>1</td></tr>
<tr><td>gi|6319992|</td><td></td><td></td><td></td><td></td><td></td><td>1</td><td>1</td><td>1</td></tr>
<tr><td>gi|6319998|</td><td></td><td></td><td></td><td></td><td></td><td>1</td><td>1</td><td>1</td></tr>
<tr><td>gi|6320020|</td><td></td><td></td><td></td><td></td><td></td><td>1</td><td>1</td><td>1</td></tr>
<tr><td>gi|6320031|</td><td></td><td></td><td></td><td></td><td></td><td>1</td><td>1</td><td>1</td></tr>
<tr><td>gi|6320034|</td><td></td><td></td><td></td><td></td><td></td><td>1</td><td>1</td><td>1</td></tr>
<tr><td>gi|6320081|</td><td></td><td></td><td></td><td></td><td></td><td>1</td><td>1</td><td>1</td></tr>
<tr><td>gi|6320086|</td><td></td><td></td><td></td><td></td><td></td><td>1</td><td>1</td><td>1</td></tr>
<tr><td>gi|6320087|</td><td></td><td></td><td></td><td></td><td></td><td>1</td><td>1</td><td>1</td></tr>
<tr><td>gi|6320090|</td><td></td><td></td><td></td><td></td><td></td><td>1</td><td>1</td><td>1</td></tr>
<tr><td>gi|6320092|</td><td>1</td><td></td><td></td><td></td><td></td><td></td><td>1</td><td>1</td></tr>
<tr><td>gi|6320094|</td><td></td><td></td><td></td><td></td><td></td><td>1</td><td>1</td><td>1</td></tr>
<tr><td>gi|6320141|</td><td></td><td></td><td></td><td></td><td></td><td>1</td><td>1</td><td>1</td></tr>
<tr><td>gi|6320147|</td><td></td><td></td><td></td><td></td><td></td><td>1</td><td>1</td><td>1</td></tr>
<tr><td>gi|6320161|</td><td></td><td></td><td></td><td></td><td></td><td>1</td><td>1</td><td>1</td></tr>
<tr><td>gi|6320174|</td><td></td><td></td><td></td><td></td><td></td><td>1</td><td>1</td><td>1</td></tr>
<tr><td>gi|6320184|</td><td></td><td></td><td></td><td></td><td></td><td>1</td><td>1</td><td>1</td></tr>
<tr><td>gi|6320230|</td><td></td><td></td><td></td><td>1</td><td></td><td></td><td>1</td><td>1</td></tr>
<tr><td>gi|6320236|</td><td></td><td></td><td></td><td></td><td></td><td>1</td><td>1</td><td>1</td></tr>
<tr><td>gi|6320237|</td><td></td><td></td><td></td><td></td><td></td><td>1</td><td>1</td><td>1</td></tr>
<tr><td>gi|6320240|</td><td></td><td></td><td></td><td></td><td></td><td>1</td><td>1</td><td>1</td></tr>
<tr><td>gi|6320251|</td><td></td><td></td><td></td><td></td><td></td><td>1</td><td>1</td><td>1</td></tr>
<tr><td>gi|6320260|</td><td></td><td></td><td></td><td></td><td></td><td>1</td><td>1</td><td>1</td></tr>
<tr><td>gi|6320266|</td><td></td><td></td><td></td><td></td><td></td><td>1</td><td>1</td><td>1</td></tr>
<tr><td>gi|6320268|</td><td></td><td></td><td></td><td></td><td></td><td>1</td><td>1</td><td>1</td></tr>
<tr><td>gi|6320286|</td><td></td><td></td><td></td><td></td><td></td><td>1</td><td>1</td><td>1</td></tr>
<tr><td>gi|6320295|</td><td></td><td></td><td></td><td></td><td></td><td>1</td><td>1</td><td>1</td></tr>
<tr><td>gi|6320309|</td><td></td><td></td><td></td><td></td><td></td><td>1</td><td>1</td><td>1</td></tr>
<tr><td>gi|6320311|</td><td></td><td></td><td></td><td></td><td></td><td>1</td><td>1</td><td>1</td></tr>
<tr><td>gi|6320348|</td><td></td><td></td><td></td><td></td><td></td><td>1</td><td>1</td><td>1</td></tr>
<tr><td>gi|6320362|</td><td></td><td></td><td></td><td></td><td></td><td>1</td><td>1</td><td>1</td></tr>
<tr><td>gi|6320364|</td><td></td><td></td><td></td><td></td><td></td><td>1</td><td>1</td><td>1</td></tr>
<tr><td>gi|6320366|</td><td></td><td></td><td></td><td></td><td></td><td>1</td><td>1</td><td>1</td></tr>
<tr><td>gi|6320383|</td><td></td><td></td><td></td><td></td><td></td><td>1</td><td>1</td><td>1</td></tr>
<tr><td>gi|6320397|</td><td></td><td></td><td></td><td></td><td></td><td>1</td><td>1</td><td>1</td></tr>
<tr><td>gi|6320404|</td><td></td><td></td><td></td><td></td><td></td><td>1</td><td>1</td><td>1</td></tr>
<tr><td>gi|6320419|</td><td></td><td></td><td></td><td></td><td></td><td>1</td><td>1</td><td>1</td></tr>
<tr><td>gi|6320427|</td><td></td><td></td><td>1</td><td></td><td></td><td></td><td>1</td><td>1</td></tr>
<tr><td>gi|6320451|</td><td></td><td></td><td></td><td></td><td></td><td>1</td><td>1</td><td>1</td></tr>
<tr><td>gi|6320512|</td><td></td><td></td><td></td><td></td><td></td><td>1</td><td>1</td><td>1</td></tr>
<tr><td>gi|6320514|</td><td></td><td></td><td></td><td></td><td></td><td>1</td><td>1</td><td>1</td></tr>
<tr><td>gi|6320522|</td><td></td><td></td><td></td><td></td><td></td><td>1</td><td>1</td><td>1</td></tr>
<tr><td>gi|6320528|</td><td></td><td></td><td></td><td></td><td></td><td>1</td><td>1</td><td>1</td></tr>
<tr><td>gi|6320539|</td><td></td><td></td><td></td><td></td><td></td><td>1</td><td>1</td><td>1</td></tr>
<tr><td>gi|6320569|</td><td></td><td></td><td></td><td></td><td></td><td>1</td><td>1</td><td>1</td></tr>
<tr><td>gi|6320589|</td><td></td><td></td><td></td><td></td><td>1</td><td></td><td>1</td><td>1</td></tr>
<tr><td>gi|6320592|</td><td></td><td></td><td></td><td></td><td></td><td>1</td><td>1</td><td>1</td></tr>
<tr><td>gi|6320601|</td><td></td><td></td><td></td><td></td><td></td><td>1</td><td>1</td><td>1</td></tr>
<tr><td>gi|6320606|</td><td></td><td></td><td></td><td></td><td></td><td>1</td><td>1</td><td>1</td></tr>
<tr><td>gi|6320607|</td><td></td><td></td><td></td><td></td><td></td><td>1</td><td>1</td><td>1</td></tr>
<tr><td>gi|6320613|</td><td></td><td></td><td></td><td></td><td></td><td>1</td><td>1</td><td>1</td></tr>
<tr><td>gi|6320633|</td><td></td><td></td><td></td><td></td><td></td><td>1</td><td>1</td><td>1</td></tr>
<tr><td>gi|6320652|</td><td></td><td></td><td></td><td></td><td></td><td>1</td><td>1</td><td>1</td></tr>
<tr><td>gi|6320676|</td><td></td><td></td><td></td><td></td><td></td><td>1</td><td>1</td><td>1</td></tr>
<tr><td>gi|6320691|</td><td></td><td></td><td></td><td></td><td></td><td>1</td><td>1</td><td>1</td></tr>
<tr><td>gi|6320711|</td><td></td><td>1</td><td></td><td></td><td></td><td></td><td>1</td><td>1</td></tr>
<tr><td>gi|6320713|</td><td></td><td></td><td></td><td></td><td></td><td>1</td><td>1</td><td>1</td></tr>
<tr><td>gi|6320717|</td><td></td><td></td><td></td><td></td><td></td><td>1</td><td>1</td><td>1</td></tr>
<tr><td>gi|6320730|</td><td></td><td></td><td></td><td></td><td></td><td>1</td><td>1</td><td>1</td></tr>
<tr><td>gi|6320746|</td><td>1</td><td></td><td></td><td></td><td></td><td></td><td>1</td><td>1</td></tr>
<tr><td>gi|6320768|</td><td></td><td></td><td></td><td>1</td><td></td><td></td><td>1</td><td>1</td></tr>
<tr><td>gi|6320772|</td><td></td><td></td><td></td><td></td><td></td><td>1</td><td>1</td><td>1</td></tr>
</table>

gi|6320780|									1		1	1
gi|6320795|								1			1	1
gi|6320799|									1		1	1
gi|6320864|									1		1	1
gi|6320875|									1		1	1
gi|6320877|									1		1	1
gi|6320880|								1			1	1
gi|6320885|									1		1	1
gi|6320891|									1		1	1
gi|6320909|									1		1	1
gi|6320932|									1		1	1
gi|6320943|									1		1	1
gi|6320955|		1									1	1
gi|6320957|									1		1	1
gi|6320960|									1		1	1
gi|6320962|	1										1	1
gi|6320993|									1		1	1
gi|6321023|		1									1	1
gi|6321032|									1		1	1
gi|6321185|		1									1	1
gi|6321189|									1		1	1
gi|6321194|									1		1	1
gi|6321195|									1		1	1
gi|6321208|									1		1	1
gi|6321213|									1		1	1
gi|6321223|									1		1	1
gi|6321269|									1		1	1
gi|6321272|									1		1	1
gi|6321274|									1		1	1
gi|6321299|									1		1	1
gi|6321314|				1							1	1
gi|6321327|									1		1	1
gi|6321352|									1		1	1
gi|6321353|									1		1	1
gi|6321382|									1		1	1
gi|6321400|									1		1	1
gi|6321425|									1		1	1
gi|6321446|									1		1	1
gi|6321502|									1		1	1
gi|6321519|									1		1	1
gi|6321530|									1		1	1
gi|6321564|									1		1	1
gi|6321582|									1		1	1
gi|6321589|									1		1	1
gi|6321606|									1		1	1
gi|6321625|									1		1	1
gi|6321663|									1		1	1
gi|6321698|									1		1	1
gi|6321721|									1		1	1
gi|6321727|									1		1	1
gi|6321740|									1		1	1
gi|6321747|									1		1	1
gi|6321748|									1		1	1
gi|6321764|									1		1	1
gi|6321814|									1		1	1
gi|6321838|									1		1	1
gi|6321849|									1		1	1
gi|6321854|									1		1	1
gi|6321858|									1		1	1
gi|6321862|									1		1	1
gi|6321877|							1				1	1
gi|6321879|									1		1	1
gi|6321906|									1		1	1
gi|6321908|				1							1	1
gi|6321948|									1		1	1
gi|6321966|									1		1	1
gi|6321972|									1		1	1
gi|6321992|									1		1	1
gi|6321995|									1		1	1
gi|6321996|									1		1	1

gi|6322025|				1	1	1
gi|6322041|				1	1	1
gi|6322061|				1	1	1
gi|6322068|				1	1	1
gi|6322071|				1	1	1
gi|6322080|				1	1	1
gi|6322083|				1	1	1
gi|6322086|				1	1	1
gi|6322092|				1	1	1
gi|6322106|				1	1	1
gi|6322202|	1				1	1
gi|6322203|		1			1	1
gi|6322221|				1	1	1
gi|6322223|				1	1	1
gi|6322225|				1	1	1
gi|6322230|				1	1	1
gi|6322255|				1	1	1
gi|6322258|				1	1	1
gi|6322278|				1	1	1
gi|6322281|				1	1	1
gi|6322295|				1	1	1
gi|6322298|			1		1	1
gi|6322322|				1	1	1
gi|6322334|				1	1	1
gi|6322345|				1	1	1
gi|6322353|				1	1	1
gi|6322354|				1	1	1
gi|6322356|				1	1	1
gi|6322375|				1	1	1
gi|6322377|				1	1	1
gi|6322378|				1	1	1
gi|6322390|				1	1	1
gi|6322398|				1	1	1
gi|6322412|				1	1	1
gi|6322450|				1	1	1
gi|6322454|		1			1	1
gi|6322502|				1	1	1
gi|6322512|				1	1	1
gi|6322520|				1	1	1
gi|6322535|				1	1	1
gi|6322544|				1	1	1
gi|6322561|				1	1	1
gi|6322571|				1	1	1
gi|6322572|				1	1	1
gi|6322574|				1	1	1
gi|6322590|				1	1	1
gi|6322593|				1	1	1
gi|6322604|				1	1	1
gi|6322609|	1				1	1
gi|6322612|				1	1	1
gi|6322616|				1	1	1
gi|6322629|				1	1	1
gi|6322671|				1	1	1
gi|6322678|				1	1	1
gi|6322694|				1	1	1
gi|6322706|				1	1	1
gi|6322732|				1	1	1
gi|6322766|				1	1	1
gi|6322786|				1	1	1
gi|6322805|				1	1	1
gi|6322837|				1	1	1
gi|6322849|				1	1	1
gi|6322850|				1	1	1
gi|6322870|				1	1	1
gi|6322871|				1	1	1
gi|6322875|				1	1	1
gi|6322892|				1	1	1
gi|6322895|				1	1	1
gi|6322914|				1	1	1
gi|6322915|				1	1	1

gi|6322920|					1	1	1
gi|6322935|					1	1	1
gi|6322958|					1	1	1
gi|6322967|					1	1	1
gi|6322968|		1				1	1
gi|6322970|					1	1	1
gi|6322996|					1	1	1
gi|6323000|					1	1	1
gi|6323007|					1	1	1
gi|6323039|					1	1	1
gi|6323043|					1	1	1
gi|6323075|					1	1	1
gi|6323076|					1	1	1
gi|6323084|					1	1	1
gi|6323094|				1		1	1
gi|6323096|		1				1	1
gi|6323107|					1	1	1
gi|6323127|					1	1	1
gi|6323149|					1	1	1
gi|6323167|					1	1	1
gi|6323193|					1	1	1
gi|6323206|					1	1	1
gi|6323207|	1					1	1
gi|6323211|					1	1	1
gi|6323212|					1	1	1
gi|6323242|		1				1	1
gi|6323243|					1	1	1
gi|6323247|					1	1	1
gi|6323257|					1	1	1
gi|6323301|		1				1	1
gi|6323323|					1	1	1
gi|6323350|					1	1	1
gi|6323351|					1	1	1
gi|6323370|					1	1	1
gi|6323375|					1	1	1
gi|6323449|					1	1	1
gi|6323457|					1	1	1
gi|6323459|					1	1	1
gi|6323489|					1	1	1
gi|6323493|					1	1	1
gi|6323518|					1	1	1
gi|6323520|					1	1	1
gi|6323521|					1	1	1
gi|6323532|					1	1	1
gi|6323539|					1	1	1
gi|6323557|					1	1	1
gi|6323558|					1	1	1
gi|6323590|					1	1	1
gi|6323611|			1			1	1
gi|6323626|					1	1	1
gi|6323648|					1	1	1
gi|6323650|					1	1	1
gi|6323659|					1	1	1
gi|6323661|					1	1	1
gi|6323662|			1			1	1
gi|6323663|					1	1	1
gi|6323666|					1	1	1
gi|6323670|					1	1	1
gi|6323676|					1	1	1
gi|6323683|					1	1	1
gi|6323710|					1	1	1
gi|6323735|					1	1	1
gi|6323737|					1	1	1
gi|6323747|					1	1	1
gi|6323789|		1				1	1
gi|6323808|					1	1	1
gi|6323823|					1	1	1
gi|6323871|					1	1	1
gi|6323884|					1	1	1
gi|6323893|					1	1	1

gi|6323894|					1	1	1
gi|6323900|					1	1	1
gi|6323918|					1	1	1
gi|6323924|					1	1	1
gi|6323935|					1	1	1
gi|6323936|					1	1	1
gi|6323940|					1	1	1
gi|6323942|					1	1	1
gi|6323960|					1	1	1
gi|6323967|					1	1	1
gi|6323981|					1	1	1
gi|6323997|					1	1	1
gi|6324014|					1	1	1
gi|6324023|					1	1	1
gi|6324052|					1	1	1
gi|6324056|					1	1	1
gi|6324057|					1	1	1
gi|6324059|					1	1	1
gi|6324061|					1	1	1
gi|6324078|					1	1	1
gi|6324081|					1	1	1
gi|6324105|					1	1	1
gi|6324114|					1	1	1
gi|6324172|					1	1	1
gi|6324177|					1	1	1
gi|6324181|		1				1	1
gi|6324192|					1	1	1
gi|6324195|					1	1	1
gi|6324214|					1	1	1
gi|6324221|			1			1	1
gi|6324263|					1	1	1
gi|6324276|					1	1	1
gi|6324281|					1	1	1
gi|6324300|					1	1	1
gi|6324318|					1	1	1
gi|6324329|					1	1	1
gi|6324340|					1	1	1
gi|6324353|	1					1	1
gi|6324355|					1	1	1
gi|6324383|					1	1	1
gi|6324388|					1	1	1
gi|6324402|				1		1	1
gi|6324408|	1					1	1
gi|6324415|					1	1	1
gi|6324440|					1	1	1
gi|6324442|					1	1	1
gi|6324443|					1	1	1
gi|6324447|	1					1	1
gi|6324485|					1	1	1
gi|6324494|					1	1	1
gi|6324507|					1	1	1
gi|6324522|					1	1	1
gi|6324531|					1	1	1
gi|6324541|					1	1	1
gi|6324543|					1	1	1
gi|6324553|					1	1	1
gi|6324563|					1	1	1
gi|6324592|					1	1	1
gi|6324976|					1	1	1
gi|6324981|					1	1	1
gi|6324990|					1	1	1
gi|6325008|					1	1	1
gi|6325017|					1	1	1
gi|6325025|					1	1	1
gi|6325028|					1	1	1
gi|6325035|					1	1	1
gi|6325048|					1	1	1
gi|6325072|					1	1	1
gi|6325142|					1	1	1
gi|6325181|					1	1	1

gi|6325200|								1		1	1
gi|6325203|								1		1	1
gi|6325204|								1		1	1
gi|6325207|								1		1	1
gi|6325236|							1			1	1
gi|6325238|								1		1	1
gi|6325265|								1		1	1
gi|6325279|								1		1	1
gi|6325336|								1		1	1
gi|6325354|								1		1	1
gi|6325363|		1								1	1
gi|6325371|								1		1	1
gi|6325393|								1		1	1
gi|6325405|	1									1	1
gi|6325417|								1		1	1
gi|6325430|								1		1	1
gi|6325448|								1		1	1
gi|6681850|								1		1	1
gi|6862571|								1		1	1
gi|9755342|								1		1	1
gi|6324461|									1	1	0

Suppl Table 3. Unique PDFs from the different proteomes

Ag	At	Ce	Dm	Hs	Mm	Os	Rn	Sc	Sp
gi|10242302|	lcl|At1g01410.1	lcl|6R55.1b CE26787 WBGene(	gi|17136300|	gi|10567818|ref|NP_066387.1|	gi|12963503|ref|NP_075618.1|	gi|6700006|ref|Os01g01050|	gi|11120684|ref|NP_068508.1|	gi|10383757|ref|NP_009880.2|	gi|19075175|ref|NP_587675.1|
gi|11359828|	lcl|At1g02030.1	lcl|AC3.1 CE33589 WBGene00	gi|17136328|	gi|11024641|ref|NP_066946.1|	gi|14010851|ref|NP_113575.1|	gi|6700010|ref|Os01g01090|	gi|11560135|ref|NP_071636.1|	gi|10383763|ref|NP_009903.2|	gi|19075176|ref|NP_587676.1|
gi|1245440|	lcl|At1g02160.1	lcl|AC3.3 CE05133 WBGene00	gi|17136348|	gi|13435137|ref|NP_073152.1|	gi|20823588|ref|XP_140860.1|	gi|6700021|ref|Os01g01200|	gi|34852460|ref|XP_346604.1|	gi|10383764|ref|NP_065436.1|	gi|19075180|ref|NP_587680.1|
gi|13537670|	lcl|At1g02750.1	lcl|AC3.4 CE37872 WBGene00	gi|17136350|	gi|13994345|ref|NP_114156.1|	gi|20831538|ref|XP_132752.1|	gi|6700024|ref|Os01g01230|	gi|34853107|ref|XP_238313.2|	gi|10383765|ref|NP_009904.2|	gi|19075187|ref|NP_587687.1|
gi|1561563|	lcl|At1g02770.1	lcl|AC3.9 CE37873 WBGene00	gi|17136544|	gi|14192939|ref|NP_115793.1|	gi|21327671|ref|NP_631971.1|	gi|6700026|ref|Os01g01250|	gi|34858916|ref|XP_342540.1|	gi|10383771|ref|NP_009910.2|	gi|19075200|ref|NP_587700.1|
gi|16266047|	lcl|At1g03300.1	lcl|AH6.10 CE01451 WBGene0	gi|17136626|	gi|15826850|ref|NP_055081.1|	gi|21361232|ref|NP_631965.1|	gi|6700085|ref|Os01g01820|	gi|34858932|ref|XP_342541.1|	gi|10383775|ref|NP_009914.2|	gi|19075213|ref|NP_587713.1|
gi|17016225|	lcl|At1g03710.1	lcl|AH6.11 CE01452 WBGene0	gi|17136852|	gi|17402929|ref|NP_478109.1|	gi|21703772|ref|NP_663361.1|	gi|6700090|ref|Os01g01860|	gi|34861941|ref|XP_344999.1|	gi|10383782|ref|NP_009940.2|	gi|19075243|ref|NP_587743.1|
gi|17016226|	lcl|At1g03720.1	lcl|AH6.12 CE01453 WBGene0	gi|17136860|	gi|17402933|ref|NP_478113.1|	gi|21703896|ref|NP_663424.1|	gi|6700102|ref|Os01g01970|	gi|34869483|ref|XP_345550.1|	gi|10383786|ref|NP_009944.2|	gi|19075250|ref|NP_587750.1|
gi|17016227|	lcl|At1g04645.1	lcl|AH6.14 CE01455 WBGene0	gi|17137016|	gi|17459291|ref|XP_066457.1|	gi|33695146|ref|NP_892042.1|	gi|6700115|ref|Os01g02070|	gi|34881259|ref|XP_344191.1|	gi|10383796|ref|NP_009964.2|	gi|19075254|ref|NP_587754.1|
gi|17016228|	lcl|At1g05080.1	lcl|AH6.4 CE01458 WBGene00	gi|17137018|	gi|17486607|ref|XP_060054.1|	gi|34147129|ref|NP_898946.1|	gi|6700131|ref|Os01g02210|	gi|8394475|ref|NP_058753.1|	gi|10383799|ref|NP_009967.2|	gi|19075256|ref|NP_587756.1|
gi|17105036|	lcl|At1g05540.1	lcl|AH6.6 CE01460 WBGene00	gi|17137180|	gi|17738294|ref|NP_478102.1|	gi|38050510|ref|XP_285102.2|	gi|6700137|ref|Os01g02270|		gi|10383808|ref|NP_010003.2|	gi|19075276|ref|NP_587776.1|
gi|18140725|	lcl|At1g05550.1	lcl|AH6.7 CE01461 WBGene00	gi|17137662|	gi|17939348|ref|NP_525128.1|	gi|38074201|ref|XP_357163.1|	gi|6700148|ref|Os01g02380|		gi|12621478|ref|NP_075208.1|	gi|19075290|ref|NP_587790.1|
gi|18140729|	lcl|At1g05615.1	lcl|AH6.8 CE01462 WBGene00	gi|17530857|	gi|18589706|ref|XP_085830.1|	gi|38075705|ref|XP_356710.1|	gi|6700158|ref|Os01g02480|		gi|13129151|ref|NP_076873.1|	gi|19075292|ref|NP_587792.1|
gi|18378603|	lcl|At1g05770.1	lcl|AH9.4 CE30841 WBGene00	gi|17647129|	gi|19743940|ref|NP_569734.2|	gi|38079390|ref|XP_358737.1|	gi|6700161|ref|Os01g02510|		gi|13129188|ref|NP_076910.1|	gi|19075308|ref|NP_587808.1|
gi|18389891|	lcl|At1g05920.1	lcl|B0001.2 CE36776 WBGene(	gi|17647179|	gi|19747269|ref|NP_570132.1|	gi|38080069|ref|XP_357478.1|	gi|6700162|ref|Os01g02520|		gi|13432314|ref|NP_085069.1|	gi|19075318|ref|NP_587818.1|
gi|18389895|	lcl|At1g05930.1	lcl|B0019.1 CE32294 WBGene(	gi|17647181|	gi|19747271|ref|NP_573440.1|	gi|38082064|ref|XP_354991.1|	gi|6700173|ref|Os01g02630|		gi|14270687|ref|NP_009484.2|	gi|19075320|ref|NP_587820.1|
gi|21288426|	lcl|At1g06910.1	lcl|B0019.2 CE37075 WBGene(	gi|17647187|	gi|19747273|ref|NP_570131.1|	gi|38082066|ref|XP_128535.2|	gi|6700174|ref|Os01g02640|		gi|14318435|ref|NP_116577.1|	gi|19075325|ref|NP_587825.1|
gi|21289770|	lcl|At1g08160.1	lcl|B0024.1 CE05146 WBGene(	gi|17647277|	gi|19747281|ref|NP_597674.1|	gi|38083679|ref|XP_111637.3|	gi|6700176|ref|Os01g02660|		gi|14318453|ref|NP_116589.1|	gi|19075326|ref|NP_587826.1|
gi|21289900|	lcl|At1g08740.1	lcl|B0024.3 CE31281 WBGene(	gi|17647279|	gi|19747283|ref|NP_570133.1|	gi|38083920|ref|XP_355749.1|	gi|6700208|ref|Os01g02980|		gi|14318457|ref|NP_116593.1|	gi|19075331|ref|NP_587831.1|
gi|21289938|	lcl|At1g09245.1	lcl|B0024.4 CE05149 WBGene(	gi|17647541|	gi|20143935|ref|NP_005050.2|	gi|38085051|ref|XP_358742.1|	gi|6700230|ref|Os01g03180|		gi|14318458|ref|NP_116594.1|	gi|19075339|ref|NP_587839.1|
gi|21290031|	lcl|At1g09360.1	lcl|B0034.5 CE02413 WBGene(	gi|17647583|	gi|20302158|ref|NP_619540.1|	gi|38089696|ref|XP_356137.1|	gi|6700234|ref|Os01g03220|		gi|14318467|ref|NP_116603.1|	gi|19075344|ref|NP_587844.1|
gi|21290052|	lcl|At1g09370.1	lcl|B0047.1 CE36680 WBGene(	gi|17647587|	gi|20357544|ref|NP_579872.1|	gi|38090523|ref|XP_356467.1|	gi|6700243|ref|Os01g03310|		gi|14318471|ref|NP_116606.1|	gi|19075346|ref|NP_587846.1|
gi|21290071|	lcl|At1g09650.1	lcl|B0047.2 CE34627 WBGene(	gi|17647767|	gi|20553917|ref|XP_114723.1|	gi|38093409|ref|XP_285626.2|	gi|6700244|ref|Os01g03320|		gi|14318499|ref|NP_116633.1|	gi|19075370|ref|NP_587870.1|
gi|21290107|	lcl|At1g09665.1	lcl|B0047.3 CE07675 WBGene(	gi|17647783|	gi|21359860|ref|NP_002814.2|	gi|38093572|ref|XP_358067.1|	gi|6700245|ref|Os01g03330|		gi|14318525|ref|NP_116658.1|	gi|19075385|ref|NP_587885.1|
gi|21290143|	lcl|At1g10110.1	lcl|B0047.4 CE07676 WBGene(	gi|17647793|	gi|21389429|ref|NP_653216.1|	gi|38093978|ref|XP_358114.1|	gi|6700246|ref|Os01g03340|		gi|14318552|ref|NP_116685.1|	gi|19075393|ref|NP_587893.1|
gi|21290186|	lcl|At1g10770.1	lcl|B0047.5 CE34628 WBGene(	gi|17648069|	gi|21699070|ref|NP_660292.1|	gi|38097201|ref|XP_356311.1|	gi|6700248|ref|Os01g03360|		gi|14318556|ref|NP_116689.1|	gi|19075396|ref|NP_587896.1|
gi|21290317|	lcl|At1g11230.1	lcl|B0198.3 CE37308 WBGene(	gi|17648071|	gi|22027489|ref|NP_663695.1|	gi|6753624|ref|NP_034160.1|	gi|6700250|ref|Os01g03380|		gi|14318559|ref|NP_116692.1|	gi|19075431|ref|NP_587931.1|
gi|21290514|	lcl|At1g11270.1	lcl|B0205.1 CE36908 WBGene(	gi|17648075|	gi|22027492|ref|NP_663697.1|	gi|6755424|ref|NP_035459.1|	gi|6700251|ref|Os01g03390|		gi|16740526|ref|NP_444296.1|	gi|19075443|ref|NP_587943.1|
gi|21290717|	lcl|At1g11765.1	lcl|B0205.2 CE17316 WBGene(	gi|17648077|	gi|22027496|ref|NP_663698.1|	gi|7110705|ref|NP_032998.1|	gi|6700253|ref|Os01g03410|		gi|18702303|ref|NP_570898.1|	gi|19075447|ref|NP_587947.1|
gi|21294658|	lcl|At1g11810.1	lcl|B0205.9 CE17323 WBGene(	gi|17648079|	gi|22748971|ref|NP_689673.1|	gi|7305557|ref|NP_038800.1|	gi|6700271|ref|Os01g03590|		gi|20336768|ref|NP_620388.1|	gi|19075476|ref|NP_587976.1|
gi|21295704|	lcl|At1g11925.1	lcl|B0207.11 CE07689 WBGene	gi|17737403|	gi|25188187|ref|NP_742001.1|	gi|7305561|ref|NP_038804.1|	gi|6700282|ref|Os01g03680|		gi|22532997|ref|NP_683715.1|	gi|19075486|ref|NP_587986.1|
gi|21297739|	lcl|At1g12170.1	lcl|B0212.2 CE37692 WBGene(	gi|17737437|	gi|27499037|ref|XP_210876.1|	gi|9910188|ref|NP_064294.1|	gi|6700293|ref|Os01g03790|		gi|27469359|ref|NP_009388.2|	gi|19075526|ref|NP_588026.1|
gi|21299458|	lcl|At1g12180.1	lcl|B0212.3 CE16769 WBGene(	gi|17737477|	gi|27734825|ref|NP_775968.1|		gi|6700300|ref|Os01g03860|		gi|27808706|ref|NP_011456.2|	gi|19075562|ref|NP_588062.1|
gi|21300507|	lcl|At1g12190.1	lcl|B0213.7 CE16778 WBGene(	gi|17737523|	gi|27735085|ref|NP_775763.1|		gi|6700302|ref|Os01g03880|		gi|27808707|ref|NP_011470.2|	gi|19075563|ref|NP_588063.1|
gi|21302771|	lcl|At1g12490.1	lcl|B0213.8 CE35206 WBGene(	gi|17737595|	gi|28372507|ref|NP_776258.1|		gi|6700319|ref|Os01g04040|		gi|33438788|ref|NP_878070.1|	gi|19075575|ref|NP_588075.1|
gi|21952522|	lcl|At1g12540.1	lcl|B0213.9 CE16780 WBGene(	gi|17737629|	gi|28416915|ref|NP_000579.2|		gi|6700320|ref|Os01g04050|		gi|33438825|ref|NP_878107.1|	gi|19075588|ref|NP_588088.1|
gi|21952523|	lcl|At1g13200.1	lcl|B0222.1 CE06692 WBGene(	gi|17737845|	gi|29737092|ref|XP_295205.1|		gi|6700324|ref|Os01g04090|		gi|33438857|ref|NP_878145.1|	gi|19075600|ref|NP_588100.1|
gi|21952524|	lcl|At1g13590.1	lcl|B0222.5 CE06696 WBGene(	gi|17737877|	gi|29739183|ref|XP_295166.1|		gi|6700329|ref|Os01g04140|		gi|33438872|ref|NP_878160.1|	gi|19075601|ref|NP_588101.1|
gi|22506871|	lcl|At1g14480.1	lcl|B0222.6 CE30585 WBGene(	gi|17737989|	gi|29742396|ref|XP_291169.1|		gi|6700399|ref|Os01g04770|		gi|37362628|ref|NP_010137.2|	gi|19075605|ref|NP_588105.1|
gi|2731364|	lcl|At1g14770.1	lcl|B0222.7 CE06698 WBGene(	gi|17738061|	gi|30425486|ref|NP_848629.1|		gi|6700401|ref|Os01g04790|		gi|37362636|ref|NP_010751.2|	gi|19075609|ref|NP_588109.1|
gi|27414019|	lcl|At1g14940.1	lcl|B0222.8 CE06699 WBGene(	gi|17738079|	gi|30725873|ref|NP_851321.1|		gi|6700408|ref|Os01g04850|		gi|37362656|ref|NP_011717.2|	gi|19075628|ref|NP_588128.1|
gi|27414021|	lcl|At1g14950.1	lcl|B0238.11 CE07701 WBGene	gi|17738201|	gi|30795231|ref|NP_006308.3|		gi|6700429|ref|Os01g05020|		gi|37362662|ref|NP_012192.2|	gi|19075652|ref|NP_588152.1|
gi|27414031|	lcl|At1g14960.1	lcl|B0238.12 CE07702 WBGene	gi|17738223|	gi|32698952|ref|NP_872366.1|		gi|6700431|ref|Os01g05040|		gi|37362679|ref|NP_013300.2|	gi|19075656|ref|NP_588156.1|
gi|27414043|	lcl|At1g15560.1	lcl|B0238.14 CE20446 WBGene	gi|17864116|	gi|33667032|ref|NP_892016.1|		gi|6700454|ref|Os01g05270|		gi|37362685|ref|NP_014083.2|	gi|19075665|ref|NP_588165.1|
gi|27414051|	lcl|At1g15680.1	lcl|B0238.3 CE35208 WBGene(	gi|17864118|	gi|37550374|ref|XP_208333.2|		gi|6700460|ref|Os01g05330|		gi|37362700|ref|NP_015114.2|	gi|19075666|ref|NP_588166.1|
gi|27414061|	lcl|At1g16705.1	lcl|B0238.5 CE07695 WBGene(	gi|17864478|	gi|37555989|ref|XP_066069.2|		gi|6700467|ref|Os01g05400|		gi|37362701|ref|NP_015177.2|	gi|19075672|ref|NP_588172.1|
gi|27414069|	lcl|At1g16940.1	lcl|B0238.6 CE07696 WBGene(	gi|17865831|	gi|38348370|ref|NP_940943.1|		gi|6700482|ref|Os01g05540|		gi|37362704|ref|NP_015410.2|	gi|19075691|ref|NP_588191.1|
gi|27414073|	lcl|At1g17610.1	lcl|B0238.8 CE37949 WBGene(	gi|17975553|	gi|38348416|ref|NP_940973.1|		gi|6700483|ref|Os01g05540|		gi|37362706|ref|NP_015418.2|	gi|19075702|ref|NP_588202.1|
gi|27414075|	lcl|At1g18050.1	lcl|B0244.10 CE26788 WBGen	gi|17975555|	gi|41147122|ref|XP_371736.1|		gi|6700494|ref|Os01g05650|		gi|41629685|ref|NP_011923.2|	gi|19075704|ref|NP_588204.1|
gi|27414079|	lcl|At1g19060.1	lcl|B0244.6 CE01753 WBGene(	gi|17986055|	gi|41148132|ref|XP_374752.1|		gi|6700507|ref|Os01g05780|		gi|41629692|ref|NP_011317.2|	gi|19075729|ref|NP_588229.1|
gi|27414081|	lcl|At1g19160.1	lcl|B0244.7 CE26790 WBGene(	gi|17986065|	gi|41148526|ref|XP_373234.1|		gi|6700518|ref|Os01g05880|		gi|42742057|ref|NP_010156.2|	gi|19075732|ref|NP_588232.1|
gi|27414083|	lcl|At1g19410.1	lcl|B0250.10 CE36577 WBGen	gi|17986069|	gi|41148856|ref|XP_372102.1|		gi|6700519|ref|Os01g05890|		gi|42742254|ref|NP_012375.2|	gi|19075740|ref|NP_588240.1|
gi|27414085|	lcl|At1g19460.1	lcl|B0250.6 CE18482 WBGene(	gi|17986137|	gi|41148858|ref|XP_372103.1|		gi|6700538|ref|Os01g06080|		gi|42742255|ref|NP_012376.2|	gi|19075758|ref|NP_588258.1|
gi|27414087|	lcl|At1g19470.1	lcl|B0280.10 CE29533 WBGen	gi|18543251|	gi|41148868|ref|XP_373297.1|		gi|6700541|ref|Os01g06110|		gi|42742287|ref|NP_013306.2|	gi|19075759|ref|NP_588259.1|
gi|27414089|	lcl|At1g19610.1	lcl|B0280.13 CE37472 WBGen	gi|18543297|	gi|41149017|ref|XP_373284.1|		gi|6700547|ref|Os01g06170|		gi|42742288|ref|NP_013417.2|	gi|19075764|ref|NP_588264.1|
gi|27414091|	lcl|At1g19930.1	lcl|B0280.2 CE37473 WBGene(	gi|18858007|	gi|41149272|ref|XP_372337.1|		gi|6700550|ref|Os01g06200|		gi|42759852|ref|NP_009919.3|	gi|19075775|ref|NP_588275.1|
gi|27414093|	lcl|At1g20240.1	lcl|B0280.5 CE31868 WBGene(	gi|18858043|	gi|41149482|ref|XP_372432.1|		gi|6700556|ref|Os01g06260|		gi|50593114|ref|NP_009349.2|	gi|19075777|ref|NP_588277.1|
gi|27414095|	lcl|At1g20360.1	lcl|B0281.3 CE07714 WBGene(	gi|18859643|	gi|41149786|ref|XP_370669.1|		gi|6700566|ref|Os01g06340|		gi|50593117|ref|NP_009487.2|	gi|19075786|ref|NP_588286.1|
gi|27414097|	lcl|At1g20400.1	lcl|B0281.4 CE32096 WBGene(	gi|18859825|	gi|41149832|ref|XP_370709.1|		gi|6700567|ref|Os01g06350|		gi|50593118|ref|NP_009618.2|	gi|19075794|ref|NP_588294.1|
gi|27414099|	lcl|At1g20450.1	lcl|B0281.8 CE07718 WBGene(	gi|18859895|	gi|41149978|ref|XP_373587.1|		gi|6700568|ref|Os01g06360|		gi|50593139|ref|NP_010314.2|	gi|19075806|ref|NP_588306.1|
gi|27414101|	lcl|At1g20450.2	lcl|B0284.4 CE00652 WBGene(	gi|18860519|	gi|41150187|ref|XP_373617.1|		gi|6700569|ref|Os01g06370|		gi|50593140|ref|NP_010465.2|	gi|19075807|ref|NP_588307.1|
gi|27414103|	lcl|At1g20520.1	lcl|B0285.7 CE00646 WBGene(	gi|18860537|	gi|41150197|ref|XP_373623.1|		gi|6700571|ref|Os01g06390|		gi|50882584|ref|NP_011999.2|	gi|19075827|ref|NP_588327.1|
gi|27414105|	lcl|At1g20600.1	lcl|B0286.6 CE36969 WBGene(	gi|18921139|	gi|41150418|ref|XP_290712.2|		gi|6700573|ref|Os01g06410|		gi|52788563|ref|YP_087097.1|	gi|19075834|ref|NP_588334.1|
gi|27414107|	lcl|At1g20790.1	lcl|B0294.1 CE19656 WBGene(	gi|19549366|	gi|41151259|ref|XP_373903.1|		gi|6700595|ref|Os01g06630|		gi|52788564|ref|YP_087098.1|	gi|19075835|ref|NP_588335.1|
gi|2833288|	lcl|At1g20795.1	lcl|B0303.2 CE00536 WBGene(	gi|19920452|	gi|41151296|ref|XP_373913.1|		gi|6700606|ref|Os01g06740|		gi|52788565|ref|YP_087099.1|	gi|19075861|ref|NP_588361.1|
gi|2842719|	lcl|At1g20800.1	lcl|B0303.8 CE00001 WBGene(	gi|19920546|	gi|41151363|ref|XP_372880.1|		gi|6700652|ref|Os01g07160|		gi|52788566|ref|YP_087100.1|	gi|19075896|ref|NP_588396.1|
gi|30173544|	lcl|At1g20870.1	lcl|B0304.7 CE33510 WBGene(	gi|19920584|	gi|41151501|ref|XP_372212.1|		gi|6700666|ref|Os01g07300|		gi|52788569|ref|YP_087103.1|	gi|19075906|ref|NP_588406.1|
gi|30173555|	lcl|At1g20940.1	lcl|B0304.8 CE33511 WBGene(	gi|19920630|	gi|41151523|ref|XP_373352.1|		gi|6700696|ref|Os01g07600|		gi|52788571|ref|YP_087105.1|	gi|19075907|ref|NP_588407.1|
gi|30173565|	lcl|At1g21030.1	lcl|B0310.1 CE03874 WBGene(	gi|19920726|	gi|41188271|ref|XP_372770.1|		gi|6700724|ref|Os01g07820|		gi|52788573|ref|YP_087107.1|	gi|19075908|ref|NP_588408.1|
gi|30174155|	lcl|At1g21090.1	lcl|B0310.4 CE03877 WBGene(	gi|19920778|	gi|41190379|ref|XP_209180.3|		gi|6700735|ref|Os01g07930|		gi|52788574|ref|YP_087108.1|	gi|19075919|ref|NP_588419.1|
gi|30174198|	lcl|At1g21990.1	lcl|B0334.1 CE02932 WBGene(	gi|19921154|	gi|41191441|ref|XP_374022.1|		gi|6700747|ref|Os01g08010|		gi|6226526|ref|NP_009312.1|	gi|19075923|ref|NP_588423.1|
gi|30174246|	lcl|At1g22080.1	lcl|B0334.6 CE30473 WBGene(	gi|19921236|	gi|41195533|ref|XP_373053.1|		gi|6700753|ref|Os01g08070|		gi|6226534|ref|NP_009320.1|	gi|19075927|ref|NP_588427.1|
gi|30174304|	lcl|At1g22090.1	lcl|B0334.7 CE35926 WBGene(	gi|19921296|	gi|41201794|ref|XP_372469.1|		gi|6700764|ref|Os01g08180|		gi|6226538|ref|NP_009324.1|	gi|19075929|ref|NP_588429.1|
gi|30174535|	lcl|At1g23120.1	lcl|B0336.1 CE26743 WBGene(	gi|19921334|	gi|41202679|ref|XP_058611.8|		gi|6700826|ref|Os01g08730|		gi|6226541|ref|NP_009327.1|	gi|19075949|ref|NP_588449.1|
gi|30174941|	lcl|At1g23350.1	lcl|B0336.7a CE29546 WBGen(	gi|19921346|	gi|41206139|ref|XP_370952.1|		gi|6700836|ref|Os01g08830|		gi|6319249|ref|NP_009332.1|	gi|19075950|ref|NP_588450.1|

gi|30175084|	lcl|At1g23570.1	lcl|B0336.7b CE37741 WBGen	gi|19921582|	gi|41222646|ref|XP_372141.1|	gi|6700837|ref|Os01g08830|	gi|6319255|ref|NP_009338.1|	gi|19075954|ref|NP_588454.1|
gi|30175171|	lcl|At1g23580.1	lcl|B0348.1 CE17326 WBGene	gi|19921814|	gi|42655808|ref|XP_377601.1|	gi|6700844|ref|Os01g08900|	gi|6319297|ref|NP_009380.1|	gi|19075960|ref|NP_588460.1|
gi|30175210|	lcl|At1g23590.1	lcl|B0348.2 CE32297 WBGene	gi|19921946|	gi|42656154|ref|XP_371384.2|	gi|6700857|ref|Os01g09020|	gi|6319299|ref|NP_009382.1|	gi|19075970|ref|NP_588470.1|
gi|30175280|	lcl|At1g23610.1	lcl|B0361.9 CE00838 WBGene	gi|19922024|	gi|42656158|ref|XP_375897.1|	gi|6700864|ref|Os01g09090|	gi|6319311|ref|NP_009394.1|	gi|19075982|ref|NP_588482.1|
gi|30175326|	lcl|At1g23680.1	lcl|B0365.2 CE07720 WBGene	gi|19922150|	gi|42656291|ref|XP_376048.1|	gi|6700872|ref|Os01g09160|	gi|6319317|ref|NP_009400.1|	gi|19075988|ref|NP_588488.1|
gi|30175327|	lcl|At1g23930.1	lcl|B0365.5 CE07722 WBGene	gi|19922274|	gi|42658241|ref|XP_379797.1|	gi|6700889|ref|Os01g09330|	gi|6319324|ref|NP_009407.1|	gi|19075993|ref|NP_588493.1|
gi|30175329|	lcl|At1g23950.1	lcl|B0391.11 CE24768 WBGen	gi|19922498|	gi|42659049|ref|XP_376930.1|	gi|6700897|ref|Os01g09410|	gi|6319329|ref|NP_009412.1|	gi|19076000|ref|NP_588500.1|
gi|30175392|	lcl|At1g23960.1	lcl|B0391.12 CE33020 WBGen	gi|19922520|	gi|42659094|ref|XP_376861.1|	gi|6700910|ref|Os01g09530|	gi|6319330|ref|NP_009413.1|	gi|19076007|ref|NP_588507.1|
gi|30175526|	lcl|At1g23970.1	lcl|B0391.3 CE17332 WBGene	gi|19922636|	gi|42659098|ref|XP_379639.1|	gi|6700921|ref|Os01g09610|	gi|6319331|ref|NP_009414.1|	gi|19076020|ref|NP_588520.1|
gi|30175920|	lcl|At1g23970.2	lcl|B0391.4 CE33021 WBGene	gi|19922742|	gi|42659116|ref|XP_376872.1|	gi|6700926|ref|Os01g09660|	gi|6319332|ref|NP_009415.1|	gi|19076028|ref|NP_588528.1|
gi|30175923|	lcl|At1g24000.1	lcl|B0391.5 CE24769 WBGene	gi|19922896|	gi|42659688|ref|XP_374899.1|	gi|6700958|ref|Os01g09980|	gi|6319333|ref|NP_009416.1|	gi|19076035|ref|NP_588535.1|
gi|30176009|	lcl|At1g24010.1	lcl|B0391.6 CE33753 WBGene	gi|19922966|	gi|42659695|ref|XP_374902.1|	gi|6700973|ref|Os01g10120|	gi|6319334|ref|NP_009418.1|	gi|19076043|ref|NP_588543.1|
gi|30176100|	lcl|At1g24220.1	lcl|B0391.9 CE17337 WBGene	gi|19922988|	gi|42660018|ref|XP_378342.1|	gi|6700984|ref|Os01g10230|	gi|6319336|ref|NP_009419.1|	gi|19076056|ref|NP_588556.1|
gi|30176147|	lcl|At1g25886.1	lcl|B0393.3 CE00856 WBGene	gi|19923048|	gi|42660711|ref|XP_208863.5|	gi|6701003|ref|Os01g10390|	gi|6319341|ref|NP_009424.1|	gi|19076069|ref|NP_588569.1|
gi|30176465|	lcl|At1g26510.1	lcl|B0393.6 CE33964 WBGene	gi|20128823|	gi|42660718|ref|XP_290671.5|	gi|6701077|ref|Os01g11080|	gi|6319356|ref|NP_009438.1|	gi|19111857|ref|NP_595065.1|
gi|30176563|	lcl|At1g26590.1	lcl|B0393.7 CE31767 WBGene	gi|20128831|	gi|42660748|ref|XP_375316.1|	gi|6701102|ref|Os01g11330|	gi|6319365|ref|NP_009447.1|	gi|19111877|ref|NP_595085.1|
gi|30176693|	lcl|At1g26680.1	lcl|B0399.2 CE36626 WBGene	gi|20128875|	gi|42660757|ref|XP_375319.1|	gi|6701120|ref|Os01g11510|	gi|6319368|ref|NP_009451.1|	gi|19111882|ref|NP_595090.1|
gi|30177070|	lcl|At1g26795.1	lcl|B0414.1 CE37176 WBGene	gi|20128955|	gi|42660927|ref|XP_208891.5|	gi|6701186|ref|Os01g12150|	gi|6319398|ref|NP_009479.1|	gi|19111906|ref|NP_595114.1|
gi|30177506|	lcl|At1g26797.1	lcl|B0414.3 CE07733 WBGene	gi|20128975|	gi|42660929|ref|XP_370958.2|	gi|6701195|ref|Os01g12230|	gi|6319411|ref|NP_009493.1|	gi|19111913|ref|NP_595121.1|
gi|30177722|	lcl|At1g26950.1	lcl|B0416.2 CE02432 WBGene	gi|20129271|	gi|42660931|ref|XP_375325.1|	gi|6701204|ref|Os01g12310|	gi|6319417|ref|NP_009499.1|	gi|19111919|ref|NP_595127.1|
gi|30177754|	lcl|At1g27270.1	lcl|B0432.1 CE07739 WBGene	gi|20129275|	gi|42660943|ref|XP_370939.2|	gi|6701208|ref|Os01g12350|	gi|6319446|ref|NP_009528.1|	gi|19111923|ref|NP_595131.1|
gi|30177755|	lcl|At1g27380.1	lcl|B0432.12 CE07750 WBGen	gi|20129395|	gi|42660957|ref|XP_290670.4|	gi|6701233|ref|Os01g12600|	gi|6319462|ref|NP_009544.1|	gi|19111935|ref|NP_595143.1|
gi|30177833|	lcl|At1g27380.2	lcl|B0432.9 CE34245 WBGene	gi|20129415|	gi|42661313|ref|XP_371089.2|	gi|6701236|ref|Os01g12630|	gi|6319466|ref|NP_009548.1|	gi|19111939|ref|NP_595147.1|
gi|30178005|	lcl|At1g27780.1	lcl|B0454.1 CE20455 WBGene	gi|20129485|	gi|42661582|ref|XP_378794.1|	gi|6701344|ref|Os01g13630|	gi|6319467|ref|NP_009550.1|	gi|19111941|ref|NP_595149.1|
gi|30178006|	lcl|At1g27800.1	lcl|B0454.10 CE25741 WBGen	gi|20129543|	gi|42661630|ref|XP_375614.1|	gi|6701350|ref|Os01g13680|	gi|6319472|ref|NP_009554.1|	gi|19111953|ref|NP_595161.1|
gi|30178161|	lcl|At1g27810.1	lcl|B0454.2 CE07752 WBGene	gi|20129551|	gi|42662557|ref|XP_372224.2|	gi|6701364|ref|Os01g13810|	gi|6319476|ref|NP_009558.1|	gi|19111961|ref|NP_595170.1|
gi|30178303|	lcl|At1g27860.1	lcl|B0454.3 CE25742 WBGene	gi|20129569|	gi|42662688|ref|XP_377051.1|	gi|6701373|ref|Os01g13900|	gi|6319479|ref|NP_009561.1|	gi|19111969|ref|NP_595177.1|
gi|30178523|	lcl|At1g28410.1	lcl|B0454.4 CE25743 WBGene	gi|20129589|	gi|4502677|ref|NP_001770.1|	gi|6701374|ref|Os01g13910|	gi|6319485|ref|NP_009568.1|	gi|19111977|ref|NP_595185.1|
gi|30178570|	lcl|At1g28500.1	lcl|B0454.5 CE37697 WBGene	gi|20129719|	gi|4503087|ref|NP_001882.1|	gi|6701382|ref|Os01g13990|	gi|6319489|ref|NP_009571.1|	gi|19111993|ref|NP_595201.1|
gi|30178721|	lcl|At1g29180.1	lcl|B0454.8 CE07758 WBGene	gi|20129833|	gi|4503305|ref|NP_001917.1|	gi|6701412|ref|Os01g14270|	gi|6319496|ref|NP_009578.1|	gi|19112007|ref|NP_595215.1|
gi|30178841|	lcl|At1g29240.1	lcl|B0454.9 CE07759 WBGene	gi|20129967|	gi|4503877|ref|NP_001459.1|	gi|6701415|ref|Os01g14300|	gi|6319507|ref|NP_009589.1|	gi|19112029|ref|NP_595237.1|
gi|30178978|	lcl|At1g29600.1	lcl|B0457.1c CE36086 WBGen	gi|20129999|	gi|4503879|ref|NP_001463.1|	gi|6701432|ref|Os01g14460|	gi|6319518|ref|NP_009600.1|	gi|19112039|ref|NP_595247.1|
gi|30178979|	lcl|At1g30135.1	lcl|B0462.1 CE15554 WBGene	gi|20130041|	gi|4503883|ref|NP_001465.1|	gi|6701442|ref|Os01g14560|	gi|6319527|ref|NP_009609.1|	gi|19112058|ref|NP_595266.1|
gi|30178980|	lcl|At1g30150.1	lcl|B0462.3 CE15556 WBGene	gi|20130099|	gi|4503885|ref|NP_001466.1|	gi|6701461|ref|Os01g14710|	gi|6319541|ref|NP_009623.1|	gi|19112063|ref|NP_595271.1|
gi|30179433|	lcl|At1g30160.1	lcl|B0464.8 CE36465 WBGene	gi|20130277|	gi|4503887|ref|NP_001467.1|	gi|6701462|ref|Os01g14720|	gi|6319572|ref|NP_009654.1|	gi|19112065|ref|NP_595273.1|
gi|309066|	lcl|At1g30170.1	lcl|B0478.3 CE06711 WBGene	gi|20130279|	gi|4503889|ref|NP_001468.1|	gi|6701463|ref|Os01g14730|	gi|6319595|ref|NP_009677.1|	gi|19112070|ref|NP_595278.1|
gi|31194145|	lcl|At1g30780.1	lcl|B0491.2 CE02104 WBGene	gi|20130329|	gi|4504227|ref|NP_002091.1|	gi|6701465|ref|Os01g14750|	gi|6319605|ref|NP_009687.1|	gi|19112090|ref|NP_595298.1|
gi|31194161|	lcl|At1g30790.1	lcl|B0491.4 CE02106 WBGene	gi|20130369|	gi|4505433|ref|NP_002512.1|	gi|6701488|ref|Os01g14940|	gi|6319607|ref|NP_009689.1|	gi|19112101|ref|NP_595309.1|
gi|31194163|	lcl|At1g30800.1	lcl|B0495.1 CE06712 WBGene	gi|20130427|	gi|4505593|ref|NP_003776.1|	gi|6701516|ref|Os01g15190|	gi|6319626|ref|NP_009708.1|	gi|19112104|ref|NP_595312.1|
gi|31194171|	lcl|At1g30920.1	lcl|B0495.6 CE01764 WBGene	gi|21355417|	gi|4506111|ref|NP_002753.1|	gi|6701524|ref|Os01g15270|	gi|6319627|ref|NP_009709.1|	gi|19112105|ref|NP_595313.1|
gi|31194179|	lcl|At1g30925.1	lcl|B0495.8b CE29949 WBGen	gi|21355449|	gi|4506885|ref|NP_002999.1|	gi|6701530|ref|Os01g15330|	gi|6319632|ref|NP_009714.1|	gi|19112108|ref|NP_595316.1|
gi|31194181|	lcl|At1g30930.1	lcl|B0496.1 CE06713 WBGene	gi|21355493|	gi|4507261|ref|NP_003145.1|	gi|6701550|ref|Os01g15500|	gi|6319634|ref|NP_009716.1|	gi|19112116|ref|NP_595324.1|
gi|31194183|	lcl|At1g30935.1	lcl|B0496.2 CE06714 WBGene	gi|21355563|	gi|4557034|ref|NP_002409.1|	gi|6701573|ref|Os01g15730|	gi|6319642|ref|NP_009724.1|	gi|19112146|ref|NP_595354.1|
gi|31194189|	lcl|At1g30990.1	lcl|B0496.5 CE06717 WBGene	gi|21355777|	gi|4557683|ref|NP_000207.1|	gi|6701575|ref|Os01g15750|	gi|6319645|ref|NP_009727.1|	gi|19112156|ref|NP_595364.1|
gi|31194199|	lcl|At1g31000.1	lcl|B0507.1 CE28815 WBGene	gi|21355803|	gi|4758146|ref|NP_004075.1|	gi|6701590|ref|Os01g15880|	gi|6319681|ref|NP_009763.1|	gi|19112164|ref|NP_595372.1|
gi|31194229|	lcl|At1g31080.1	lcl|B0507.11 CE36422 WBGen	gi|21355807|	gi|4826692|ref|NP_004933.1|	gi|6701639|ref|Os01g16310|	gi|6319690|ref|NP_009772.1|	gi|19112170|ref|NP_595378.1|
gi|31194247|	lcl|At1g31090.1	lcl|B0507.4 CE35211 WBGene	gi|21355841|	gi|4885179|ref|NP_005208.1|	gi|6701646|ref|Os01g16380|	gi|6319701|ref|NP_009782.1|	gi|19112178|ref|NP_595386.1|
gi|31194257|	lcl|At1g31240.1	lcl|B0507.6 CE07765 WBGene	gi|21355913|	gi|4885635|ref|NP_005416.1|	gi|6701654|ref|Os01g16460|	gi|6319716|ref|NP_009798.1|	gi|19112180|ref|NP_595388.1|
gi|31194263|	lcl|At1g31370.1	lcl|B0511.3 CE17342 WBGene	gi|21356037|	gi|5031781|ref|NP_005523.1|	gi|6701656|ref|Os01g16480|	gi|6319717|ref|NP_009799.1|	gi|19112182|ref|NP_595390.1|
gi|31194265|	lcl|At1g31510.1	lcl|B0513.6 CE15562 WBGene	gi|21356057|	gi|5901986|ref|NP_008934.1|	gi|6701686|ref|Os01g16780|	gi|6319734|ref|NP_009816.1|	gi|19112191|ref|NP_595399.1|
gi|31194273|	lcl|At1g31540.1	lcl|B0524.4 CE20466 WBGene	gi|21356135|	gi|5902014|ref|NP_008916.1|	gi|6701696|ref|Os01g16880|	gi|6319735|ref|NP_009817.1|	gi|19112196|ref|NP_595404.1|
gi|31194287|	lcl|At1g32030.1	lcl|B0524.6 CE20468 WBGene	gi|21356271|	gi|6005741|ref|NP_009092.1|	gi|6701711|ref|Os01g17030|	gi|6319747|ref|NP_009829.1|	gi|19112204|ref|NP_595412.1|
gi|31194295|	lcl|At1g32140.1	lcl|B0545.3 CE17356 WBGene	gi|21356367|	gi|7108344|ref|NP_006424.2|	gi|6701712|ref|Os01g17040|	gi|6319748|ref|NP_009830.1|	gi|19112234|ref|NP_595442.1|
gi|31194297|	lcl|At1g32375.1	lcl|B0545.4 CE17357 WBGene	gi|21356383|	gi|7108346|ref|NP_036615.1|	gi|6701713|ref|Os01g17050|	gi|6319750|ref|NP_009832.1|	gi|19112241|ref|NP_595449.1|
gi|31194299|	lcl|At1g32420.1	lcl|B0546.3 CE16794 WBGene	gi|21356557|	gi|7661586|ref|NP_056409.1|	gi|6701714|ref|Os01g17050|	gi|6319754|ref|NP_009837.1|	gi|19112245|ref|NP_595453.1|
gi|31194301|	lcl|At1g32430.1	lcl|B0546.5 CE34640 WBGene	gi|21356583|	gi|7705379|ref|NP_036328.1|	gi|6701718|ref|Os01g17080|	gi|6319756|ref|NP_009838.1|	gi|19112256|ref|NP_595464.1|
gi|31194313|	lcl|At1g32600.1	lcl|B0547.4 CE07772 WBGene	gi|21356623|	gi|7706551|ref|NP_057596.1|	gi|6701739|ref|Os01g17270|	gi|6319763|ref|NP_009845.1|	gi|19112271|ref|NP_595479.1|
gi|31194315|	lcl|At1g32660.1	lcl|B0554.2 CE07774 WBGene	gi|21356705|	gi|8922686|ref|NP_060698.1|	gi|6701743|ref|Os01g17310|	gi|6319777|ref|NP_009860.1|	gi|19112275|ref|NP_595483.1|
gi|31194323|	lcl|At1g32830.1	lcl|B0554.4 CE07776 WBGene	gi|21356799|	gi|8923452|ref|NP_060312.1|	gi|6701747|ref|Os01g17350|	gi|6319779|ref|NP_009861.1|	gi|19112289|ref|NP_595497.1|
gi|31194325|	lcl|At1g32840.1	lcl|B0554.6 CE07778 WBGene	gi|21356867|	gi|8923756|ref|NP_060872.1|	gi|6701749|ref|Os01g17370|	gi|6319810|ref|NP_009891.1|	gi|19112315|ref|NP_595523.1|
gi|31194333|	lcl|At1g33010.1	lcl|B0554.7 CE25744 WBGene	gi|21356963|	gi|8924176|ref|NP_061000.1|	gi|6701750|ref|Os01g17380|	gi|6319813|ref|NP_009894.1|	gi|19112318|ref|NP_595526.1|
gi|31194341|	lcl|At1g33050.1	lcl|B0563.2 CE35212 WBGene	gi|21356965|	gi|9910196|ref|NP_064546.1|	gi|6701756|ref|Os01g17440|	gi|6319817|ref|NP_009898.1|	gi|19112329|ref|NP_595537.1|
gi|31194355|	lcl|At1g33050.2	lcl|B0563.6a CE29551 WBGen	gi|21356967|	gi|9910354|ref|NP_064532.1|	gi|6701757|ref|Os01g17450|	gi|6319852|ref|NP_009933.1|	gi|19112332|ref|NP_595540.1|
gi|31194371|	lcl|At1g33070.1	lcl|B0563.6b CE33513 WBGen	gi|21357001|	gi|9966899|ref|NP_065144.1|	gi|6701765|ref|Os01g18020|	gi|6319865|ref|NP_009946.1|	gi|19112335|ref|NP_595543.1|
gi|31194375|	lcl|At1g33130.1	lcl|B0564.11 CE36970 WBGen	gi|21357097|		gi|6701766|ref|Os01g18030|	gi|6319866|ref|NP_009947.1|	gi|19112398|ref|NP_595606.1|
gi|31194389|	lcl|At1g33530.1	lcl|BE10.3 CE15566 WBGene0	gi|21357379|		gi|6701783|ref|Os01g18200|	gi|6319867|ref|NP_009948.1|	gi|19112400|ref|NP_595608.1|
gi|31194391|	lcl|At1g33570.1	lcl|C01B12.1 CE07789 WBGen	gi|21357479|		gi|6701789|ref|Os01g18260|	gi|6319931|ref|NP_010012.1|	gi|19112404|ref|NP_595612.1|
gi|31194403|	lcl|At1g34480.1	lcl|C01B12.2 CE32107 WBGen	gi|21357485|		gi|6701818|ref|Os01g18550|	gi|6319933|ref|NP_010014.1|	gi|19112418|ref|NP_595626.1|
gi|31194405|	lcl|At1g34530.1	lcl|C01B4.1 CE20470 WBGene	gi|21357549|		gi|6701833|ref|Os01g18680|	gi|6319938|ref|NP_010019.1|	gi|19112420|ref|NP_595628.1|
gi|31194407|	lcl|At1g34560.1	lcl|C01B4.10 CE20479 WBGen	gi|21357617|		gi|6701841|ref|Os01g18760|	gi|6319939|ref|NP_010020.1|	gi|19112428|ref|NP_595636.1|
gi|31194413|	lcl|At1g34610.1	lcl|C01B4.2 CE36778 WBGene	gi|21357619|		gi|6701842|ref|Os01g18770|	gi|6319943|ref|NP_010024.1|	gi|19112433|ref|NP_595641.1|
gi|31194415|	lcl|At1g34710.1	lcl|C01B4.3 CE20472 WBGene	gi|21357645|		gi|6701868|ref|Os01g18980|	gi|6319944|ref|NP_010025.1|	gi|19112448|ref|NP_595656.1|
gi|31194423|	lcl|At1g34720.1	lcl|C01B4.5 CE24773 WBGene	gi|21357647|		gi|6701874|ref|Os01g19040|	gi|6319945|ref|NP_010026.1|	gi|19112466|ref|NP_595674.1|
gi|31194425|	lcl|At1g34740.1	lcl|C01B7.1a CE29553 WBGen	gi|21357785|		gi|6701877|ref|Os01g19070|	gi|6319948|ref|NP_010029.1|	gi|19112472|ref|NP_595680.1|
gi|31194427|	lcl|At1g34842.1	lcl|C01B7.1b CE30719 WBGen	gi|21357967|		gi|6701881|ref|Os01g19110|	gi|6319950|ref|NP_010031.1|	gi|19112481|ref|NP_595689.1|
gi|31194431|	lcl|At1g35090.1	lcl|C01B7.7 CE06731 WBGene	gi|21358033|		gi|6701893|ref|Os01g19200|	gi|6319953|ref|NP_010033.1|	gi|19112487|ref|NP_595695.1|
gi|31194433|	lcl|At1g35110.1	lcl|C01B9.1 CE17359 WBGene	gi|21358055|		gi|6701915|ref|Os01g19420|	gi|6319974|ref|NP_010054.1|	gi|19112510|ref|NP_595718.1|

gi|31194443|	lcl|At1g35310.1	lcl|C01C10.1 CE28881 WBGen	gi|21358247|	gi|6701932|ref|Os01g19590|	gi|6319979|ref|NP_010059.1|	gi|19112520|ref|NP_595728.1|
gi|31194453|	lcl|At1g35365.1	lcl|C01C10.4 CE28882 WBGen	gi|21358409|	gi|6701939|ref|Os01g19660|	gi|6319981|ref|NP_010061.1|	gi|19112523|ref|NP_595731.1|
gi|31194455|	lcl|At1g35400.1	lcl|C01F1.4 CE06745 WBGene	gi|21358411|	gi|6701944|ref|Os01g19710|	gi|6320022|ref|NP_010102.1|	gi|19112525|ref|NP_595733.1|
gi|31194457|	lcl|At1g35410.1	lcl|C01F6.4 CE02953 WBGene	gi|21358485|	gi|6701963|ref|Os01g19900|	gi|6320045|ref|NP_010125.1|	gi|19112527|ref|NP_595735.1|
gi|31194465|	lcl|At1g35600.1	lcl|C01F6.5 CE02954 WBGene	gi|21358611|	gi|6701972|ref|Os01g19990|	gi|6320050|ref|NP_010131.1|	gi|19112546|ref|NP_595754.1|
gi|31194467|	lcl|At1g35610.1	lcl|C01F6.8a CE17360 WBGen	gi|21686963|	gi|6701977|ref|Os01g20040|	gi|6320072|ref|NP_010152.1|	gi|19112553|ref|NP_595761.1|
gi|31194473|	lcl|At1g35650.1	lcl|C01F6.8b CE30069 WBGen	gi|21686993|	gi|6701978|ref|Os01g20050|	gi|6320080|ref|NP_010160.1|	gi|19112566|ref|NP_595774.1|
gi|31194475|	lcl|At1g35770.1	lcl|C01G10.1 CE07810 WBGen	gi|21687080|	gi|6701980|ref|Os01g20070|	gi|6320081|ref|NP_010161.1|	gi|19112567|ref|NP_595775.1|
gi|31194481|	lcl|At1g35930.1	lcl|C01G10.12 CE07821 WBGe	gi|22023972|	gi|6701981|ref|Os01g20080|	gi|6320087|ref|NP_010167.1|	gi|19112592|ref|NP_595800.1|
gi|31194485|	lcl|At1g35950.1	lcl|C01G10.14 CE07823 WBGe	gi|22023974|	gi|6701988|ref|Os01g20150|	gi|6320096|ref|NP_010176.1|	gi|19112610|ref|NP_595818.1|
gi|31194489|	lcl|At1g36190.1	lcl|C01G10.2 CE07811 WBGen	gi|22023986|	gi|6701992|ref|Os01g20190|	gi|6320111|ref|NP_010191.1|	gi|19112616|ref|NP_595824.1|
gi|31194497|	lcl|At1g36260.1	lcl|C01G10.3 CE36264 WBGen	gi|22024075|	gi|6702010|ref|Os01g20850|	gi|6320115|ref|NP_010195.1|	gi|19112633|ref|NP_595841.1|
gi|31194511|	lcl|At1g36430.1	lcl|C01G12.1 CE15571 WBGen	gi|22026783|	gi|6702035|ref|Os01g21090|	gi|6320117|ref|NP_010197.1|	gi|19112646|ref|NP_595854.1|
gi|31194513|	lcl|At1g36460.1	lcl|C01G12.7 CE31150 WBGen	gi|22026840|	gi|6702057|ref|Os01g21290|	gi|6320138|ref|NP_010218.1|	gi|19112650|ref|NP_595858.1|
gi|31194519|	lcl|At1g36480.1	lcl|C01G5.4 CE23512 WBGene	gi|22026893|	gi|6702079|ref|Os01g21490|	gi|6320144|ref|NP_010224.1|	gi|19112653|ref|NP_595861.1|
gi|31194541|	lcl|At1g36510.1	lcl|C01G6.5 CE00867 WBGene	gi|22026898|	gi|6702080|ref|Os01g21500|	gi|6320146|ref|NP_010226.1|	gi|19112654|ref|NP_595862.1|
gi|31194543|	lcl|At1g36810.1	lcl|C02A12.2 CE07826 WBGen	gi|22026979|	gi|6702111|ref|Os01g21810|	gi|6320149|ref|NP_010229.1|	gi|19112685|ref|NP_595893.1|
gi|31194551|	lcl|At1g36910.1	lcl|C02A12.3 CE07827 WBGen	gi|24158424|	gi|6702144|ref|Os01g22120|	gi|6320154|ref|NP_010234.1|	gi|19112693|ref|NP_595901.1|
gi|31194555|	lcl|At1g37020.1	lcl|C02A12.4 CE07828 WBGen	gi|24580560|	gi|6702146|ref|Os01g22140|	gi|6320155|ref|NP_010235.1|	gi|19112704|ref|NP_595912.1|
gi|31194559|	lcl|At1g37050.1	lcl|C02A12.5 CE36779 WBGen	gi|24580684|	gi|6702152|ref|Os01g22200|	gi|6320158|ref|NP_010238.1|	gi|19112722|ref|NP_595930.1|
gi|31194563|	lcl|At1g37160.1	lcl|C02A12.6 CE07830 WBGen	gi|24580778|	gi|6702173|ref|Os01g22400|	gi|6320161|ref|NP_010241.1|	gi|19112726|ref|NP_595934.1|
gi|31194583|	lcl|At1g37275.1	lcl|C02B10.3 CE29018 WBGen	gi|24580886|	gi|6702174|ref|Os01g22410|	gi|6320186|ref|NP_010266.1|	gi|19112727|ref|NP_595935.1|
gi|31194595|	lcl|At1g39110.1	lcl|C02B10.4 CE16801 WBGen	gi|24581132|	gi|6702176|ref|Os01g22430|	gi|6320201|ref|NP_010281.1|	gi|19112753|ref|NP_595961.1|
gi|31194603|	lcl|At1g39270.1	lcl|C02B8.6 CE29656 WBGene	gi|24581182|	gi|6702181|ref|Os01g22480|	gi|6320230|ref|NP_010310.1|	gi|19112755|ref|NP_595963.1|
gi|31194605|	lcl|At1g39430.1	lcl|C02C2.1 CE06754 WBGene	gi|24581185|	gi|6702187|ref|Os01g22540|	gi|6320258|ref|NP_010337.1|	gi|19112760|ref|NP_595968.1|
gi|31194607|	lcl|At1g39990.1	lcl|C02C6.3 CE29169 WBGene	gi|24581224|	gi|6702191|ref|Os01g22580|	gi|6320263|ref|NP_010343.1|	gi|19112766|ref|NP_595974.1|
gi|31194621|	lcl|At1g40105.1	lcl|C02E7.13 CE16805 WBGen	gi|24581473|	gi|6702195|ref|Os01g22620|	gi|6320268|ref|NP_010348.1|	gi|19112787|ref|NP_595995.1|
gi|31194629|	lcl|At1g40109.1	lcl|C02E7.2 CE17362 WBGene	gi|24581499|	gi|6702208|ref|Os01g22740|	gi|6320272|ref|NP_010352.1|	gi|19112810|ref|NP_596018.1|
gi|31194633|	lcl|At1g40310.1	lcl|C02E7.3 CE07840 WBGene	gi|24581514|	gi|6702217|ref|Os01g22830|	gi|6320273|ref|NP_010353.1|	gi|19112820|ref|NP_596028.1|
gi|31194637|	lcl|At1g41910.1	lcl|C02E7.4 CE07841 WBGene	gi|24581516|	gi|6702219|ref|Os01g22850|	gi|6320276|ref|NP_010356.1|	gi|19112821|ref|NP_596029.1|
gi|31194643|	lcl|At1g41930.1	lcl|C02E7.5 CE07842 WBGene	gi|24581711|	gi|6702240|ref|Os01g23050|	gi|6320281|ref|NP_010361.1|	gi|19112822|ref|NP_596030.1|
gi|31194651|	lcl|At1g42150.1	lcl|C02E7.9 CE25748 WBGene	gi|24581949|	gi|6702242|ref|Os01g23070|	gi|6320286|ref|NP_010366.1|	gi|19112829|ref|NP_596037.1|
gi|31194655|	lcl|At1g42200.1	lcl|C02F5.11 CE25750 WBGen	gi|24581962|	gi|6702243|ref|Os01g23080|	gi|6320291|ref|NP_010371.1|	gi|19112833|ref|NP_596041.1|
gi|31194657|	lcl|At1g42250.1	lcl|C02F5.8 CE00044 WBGene	gi|24581964|	gi|6702246|ref|Os01g23110|	gi|6320294|ref|NP_010374.1|	gi|19112866|ref|NP_596074.1|
gi|31194665|	lcl|At1g42360.1	lcl|C02H6.2 CE07858 WBGene	gi|24581996|	gi|6702272|ref|Os01g23370|	gi|6320306|ref|NP_010386.1|	gi|19112870|ref|NP_596078.1|
gi|31194671|	lcl|At1g42370.1	lcl|C02H7.2 CE06758 WBGene	gi|24582034|	gi|6702279|ref|Os01g23440|	gi|6320308|ref|NP_010388.1|	gi|19112897|ref|NP_596105.1|
gi|31194673|	lcl|At1g42400.1	lcl|C03A3.3 CE05194 WBGene	gi|24582096|	gi|6702289|ref|Os01g23540|	gi|6320309|ref|NP_010389.1|	gi|19112900|ref|NP_596108.1|
gi|31194677|	lcl|At1g42410.1	lcl|C03A7.10 CE07868 WBGen	gi|24582123|	gi|6702291|ref|Os01g23560|	gi|6320311|ref|NP_010391.1|	gi|19112911|ref|NP_596119.1|
gi|31194721|	lcl|At1g42460.1	lcl|C03A7.13 CE07871 WBGen	gi|24582162|	gi|6702306|ref|Os01g23690|	gi|6320324|ref|NP_010404.1|	gi|19112917|ref|NP_596125.1|
gi|31194723|	lcl|At1g42500.1	lcl|C03A7.14 CE07872 WBGen	gi|24582205|	gi|6702312|ref|Os01g23750|	gi|6320328|ref|NP_010408.1|	gi|19112919|ref|NP_596127.1|
gi|31194725|	lcl|At1g43040.1	lcl|C03A7.3 CE34521 WBGene	gi|24582276|	gi|6702313|ref|Os01g23760|	gi|6320336|ref|NP_010416.1|	gi|19112942|ref|NP_596150.1|
gi|31194727|	lcl|At1g43300.1	lcl|C03A7.4 CE07862 WBGene	gi|24582319|	gi|6702314|ref|Os01g23770|	gi|6320337|ref|NP_010417.1|	gi|19112957|ref|NP_596165.1|
gi|31194729|	lcl|At1g43740.1	lcl|C03A7.5 CE35215 WBGene	gi|24582516|	gi|6702315|ref|Os01g23780|	gi|6320347|ref|NP_010427.1|	gi|19112958|ref|NP_596166.1|
gi|31194731|	lcl|At1g43750.1	lcl|C03A7.6 CE35216 WBGene	gi|24582826|	gi|6702319|ref|Os01g23820|	gi|6320356|ref|NP_010436.1|	gi|19112976|ref|NP_596184.1|
gi|31194735|	lcl|At1g43840.1	lcl|C03A7.7 CE07865 WBGene	gi|24582864|	gi|6702333|ref|Os01g23960|	gi|6320357|ref|NP_010437.1|	gi|19112984|ref|NP_596192.1|
gi|31194739|	lcl|At1g43880.1	lcl|C03A7.8 CE07866 WBGene	gi|24583142|	gi|6702346|ref|Os01g24080|	gi|6320367|ref|NP_010447.1|	gi|19112985|ref|NP_596193.1|
gi|31194741|	lcl|At1g44020.1	lcl|C03A7.9 CE37951 WBGene	gi|24583202|	gi|6702351|ref|Os01g24130|	gi|6320375|ref|NP_010455.1|	gi|19113014|ref|NP_596222.1|
gi|31194743|	lcl|At1g44030.1	lcl|C03B1.12 CE03912 WBGen	gi|24583456|	gi|6702356|ref|Os01g24180|	gi|6320376|ref|NP_010456.1|	gi|19113026|ref|NP_596234.1|
gi|31194747|	lcl|At1g44050.1	lcl|C03B1.13 CE28529 WBGen	gi|24583535|	gi|6702358|ref|Os01g24200|	gi|6320379|ref|NP_010459.1|	gi|19113028|ref|NP_596236.1|
gi|31194751|	lcl|At1g44060.1	lcl|C03B1.4 CE03906 WBGene	gi|24583539|	gi|6702362|ref|Os01g24240|	gi|6320392|ref|NP_010472.1|	gi|19113034|ref|NP_596242.1|
gi|31194777|	lcl|At1g44810.1	lcl|C03C11.1 CE07873 WBGen	gi|24583616|	gi|6702368|ref|Os01g24300|	gi|6320403|ref|NP_010483.1|	gi|19113043|ref|NP_596251.1|
gi|31194779|	lcl|At1g44860.1	lcl|C03C11.2 CE07874 WBGen	gi|24583743|	gi|6702371|ref|Os01g24330|	gi|6320404|ref|NP_010484.1|	gi|19113044|ref|NP_596252.1|
gi|31194783|	lcl|At1g44880.1	lcl|C03D6.4 CE05198 WBGene	gi|24583867|	gi|6702374|ref|Os01g24360|	gi|6320412|ref|NP_010492.1|	gi|19113055|ref|NP_596263.1|
gi|31194787|	lcl|At1g45015.1	lcl|C03E10.5 CE15585 WBGen	gi|24583925|	gi|6702376|ref|Os01g24380|	gi|6320413|ref|NP_010493.1|	gi|19113063|ref|NP_596271.1|
gi|31194789|	lcl|At1g45080.1	lcl|C03G6.1 CE07875 WBGene	gi|24584148|	gi|6702380|ref|Os01g24420|	gi|6320419|ref|NP_010499.1|	gi|19113077|ref|NP_596285.1|
gi|31194791|	lcl|At1g45090.1	lcl|C03G6.11 CE35951 WBGen	gi|24584348|	gi|6702389|ref|Os01g24510|	gi|6320423|ref|NP_010503.1|	gi|19113079|ref|NP_596287.1|
gi|31194797|	lcl|At1g45190.1	lcl|C03G6.13 CE07887 WBGen	gi|24584350|	gi|6702391|ref|Os01g24530|	gi|6320425|ref|NP_010505.1|	gi|19113088|ref|NP_596296.1|
gi|31194801|	lcl|At1g46408.1	lcl|C03G6.16 CE07890 WBGen	gi|24584494|	gi|6702400|ref|Os01g24620|	gi|6320427|ref|NP_010507.1|	gi|19113089|ref|NP_596297.1|
gi|31194803|	lcl|At1g46696.1	lcl|C03G6.17 CE35218 WBGen	gi|24584506|	gi|6702404|ref|Os01g24660|	gi|6320439|ref|NP_010519.1|	gi|19113091|ref|NP_596299.1|
gi|31194805|	lcl|At1g46840.1	lcl|C03G6.18 CE37078 WBGen	gi|24584512|	gi|6702408|ref|Os01g24700|	gi|6320441|ref|NP_010521.1|	gi|19113094|ref|NP_596302.1|
gi|31194813|	lcl|At1g46912.1	lcl|C03G6.2 CE07876 WBGene	gi|24584525|	gi|6702409|ref|Os01g24710|	gi|6320460|ref|NP_010540.1|	gi|19113128|ref|NP_596336.1|
gi|31194815|	lcl|At1g46984.1	lcl|C03G6.3 CE07877 WBGene	gi|24584556|	gi|6702427|ref|Os01g24890|	gi|6320465|ref|NP_010545.1|	gi|19113131|ref|NP_596339.1|
gi|31194817|	lcl|At1g47300.1	lcl|C03G6.5 CE28386 WBGene	gi|24584601|	gi|6702437|ref|Os01g24990|	gi|6320466|ref|NP_010546.1|	gi|19113144|ref|NP_596352.1|
gi|31194841|	lcl|At1g47350.1	lcl|C03G6.6 CE36265 WBGene	gi|24584603|	gi|6702441|ref|Os01g25030|	gi|6320479|ref|NP_010559.1|	gi|19113159|ref|NP_596367.1|
gi|31194843|	lcl|At1g47370.1	lcl|C03G6.7 CE07881 WBGene	gi|24584632|	gi|6702444|ref|Os01g25060|	gi|6320492|ref|NP_010572.1|	gi|19113181|ref|NP_596389.1|
gi|31194845|	lcl|At1g47390.1	lcl|C03G6.9 CE07883 WBGene	gi|24584634|	gi|6702450|ref|Os01g25120|	gi|6320508|ref|NP_010588.1|	gi|19113182|ref|NP_596390.1|
gi|31194847|	lcl|At1g47450.1	lcl|C03H5.1 CE32109 WBGene	gi|24584742|	gi|6702453|ref|Os01g25150|	gi|6320509|ref|NP_010589.1|	gi|19113190|ref|NP_596398.1|
gi|31194861|	lcl|At1g47470.1	lcl|C03H5.4 CE07897 WBGene	gi|24584774|	gi|6702459|ref|Os01g25210|	gi|6320515|ref|NP_010595.1|	gi|19113230|ref|NP_596438.1|
gi|31194863|	lcl|At1g47540.1	lcl|C03H5.6 CE07899 WBGene	gi|24584776|	gi|6702466|ref|Os01g25280|	gi|6320536|ref|NP_010616.1|	gi|19113240|ref|NP_596448.1|
gi|31194893|	lcl|At1g47730.1	lcl|C04B4.3 CE05205 WBGene	gi|24584936|	gi|6702472|ref|Os01g25340|	gi|6320554|ref|NP_010634.1|	gi|19113248|ref|NP_596456.1|
gi|31194895|	lcl|At1g47765.1	lcl|C04C11.1a CE05209 WBGe	gi|24585177|	gi|6702479|ref|Os01g25410|	gi|6320556|ref|NP_010636.1|	gi|19113263|ref|NP_596471.1|
gi|31194903|	lcl|At1g47790.1	lcl|C04C11.2 CE05210 WBGen	gi|24585197|	gi|6702498|ref|Os01g25590|	gi|6320569|ref|NP_010649.1|	gi|19113288|ref|NP_596496.1|
gi|31194913|	lcl|At1g47800.1	lcl|C04C3.1 CE37183 WBGene	gi|24585215|	gi|6702504|ref|Os01g25640|	gi|6320570|ref|NP_010650.1|	gi|19113292|ref|NP_596500.1|
gi|31194927|	lcl|At1g47810.1	lcl|C04C3.6 CE16812 WBGene	gi|24585280|	gi|6702507|ref|Os01g25670|	gi|6320571|ref|NP_010651.1|	gi|19113329|ref|NP_596537.1|
gi|31194933|	lcl|At1g47920.1	lcl|C04C3.7 CE16813 WBGene	gi|24585288|	gi|6702510|ref|Os01g25700|	gi|6320577|ref|NP_010657.1|	gi|19113340|ref|NP_596548.1|
gi|31194935|	lcl|At1g47960.1	lcl|C04E12.1 CE35219 WBGen	gi|24585337|	gi|6702532|ref|Os01g25900|	gi|6320589|ref|NP_010669.1|	gi|19113344|ref|NP_596552.1|
gi|31194945|	lcl|At1g48010.1	lcl|C04E12.10 CE16823 WBGe	gi|24585479|	gi|6702537|ref|Os01g25950|	gi|6320611|ref|NP_010691.1|	gi|19113347|ref|NP_596555.1|

gi|31194951|	lcl|At1g48020.1	lcl|C04E12.2 CE16815 WBGen	gi|24585481|	gi|6702565|ref|Os01g26230|	gi|6320622|ref|NP_010702.1|	gi|19113369|ref|NP_596577.1|
gi|31194955|	lcl|At1g48060.1	lcl|C04E12.4 CE30721 WBGen	gi|24585510|	gi|6702575|ref|Os01g26330|	gi|6320623|ref|NP_010703.1|	gi|19113374|ref|NP_596582.1|
gi|31194957|	lcl|At1g48150.1	lcl|C04E12.5 CE35221 WBGen	gi|24585531|	gi|6702593|ref|Os01g26990|	gi|6320629|ref|NP_010709.1|	gi|19113383|ref|NP_596591.1|
gi|31194963|	lcl|At1g48180.1	lcl|C04E12.6 CE16819 WBGen	gi|24585535|	gi|6702602|ref|Os01g27080|	gi|6320630|ref|NP_010710.1|	gi|19113392|ref|NP_596600.1|
gi|31194965|	lcl|At1g48500.1	lcl|C04E12.8 CE16821 WBGen	gi|24585537|	gi|6702637|ref|Os01g27430|	gi|6320636|ref|NP_010716.1|	gi|19113395|ref|NP_596603.1|
gi|31194969|	lcl|At1g48620.1	lcl|C04E12.9 CE16822 WBGen	gi|24585737|	gi|6702641|ref|Os01g27470|	gi|6320640|ref|NP_010720.1|	gi|19113416|ref|NP_596624.1|
gi|31194971|	lcl|At1g49090.1	lcl|C04E6.10 CE25754 WBGen	gi|24585739|	gi|6702654|ref|Os01g27600|	gi|6320652|ref|NP_010732.1|	gi|19113419|ref|NP_596627.1|
gi|31194977|	lcl|At1g49420.1	lcl|C04E6.12 CE07917 WBGen	gi|24585749|	gi|6702660|ref|Os01g27660|	gi|6320666|ref|NP_010746.1|	gi|19113430|ref|NP_596638.1|
gi|31194979|	lcl|At1g49480.1	lcl|C04E6.2 CE07907 WBGene	gi|24586153|	gi|6702662|ref|Os01g27680|	gi|6320677|ref|NP_010757.1|	gi|19113431|ref|NP_596639.1|
gi|31194981|	lcl|At1g49790.1	lcl|C04E6.5 CE27053 WBGene	gi|24586155|	gi|6702674|ref|Os01g27800|	gi|6320687|ref|NP_010767.1|	gi|19113446|ref|NP_596654.1|
gi|31194983|	lcl|At1g50190.1	lcl|C04E6.6 CE34652 WBGene	gi|24586157|	gi|6702685|ref|Os01g27910|	gi|6320692|ref|NP_010772.1|	gi|19113455|ref|NP_596663.1|
gi|31194985|	lcl|At1g50690.1	lcl|C04E6.9 CE25755 WBGene	gi|24586562|	gi|6702687|ref|Os01g27930|	gi|6320696|ref|NP_010776.1|	gi|19113476|ref|NP_596684.1|
gi|31194987|	lcl|At1g50780.1	lcl|C04E7.2 CE28816 WBGene	gi|24586566|	gi|6702689|ref|Os01g27950|	gi|6320706|ref|NP_010786.1|	gi|19113493|ref|NP_596701.1|
gi|31194989|	lcl|At1g50850.1	lcl|C04F1.1 CE17364 WBGene	gi|24586568|	gi|6702696|ref|Os01g28010|	gi|6320709|ref|NP_010789.1|	gi|19113506|ref|NP_596714.1|
gi|31194991|	lcl|At1g50870.1	lcl|C04F12.6 CE19679 WBGen	gi|24586570|	gi|6702697|ref|Os01g28020|	gi|6320722|ref|NP_010802.1|	gi|19113508|ref|NP_596716.1|
gi|31194995|	lcl|At1g50880.1	lcl|C04F2.1 CE17367 WBGene	gi|24586574|	gi|6702702|ref|Os01g28070|	gi|6320729|ref|NP_010808.1|	gi|19113510|ref|NP_596718.1|
gi|31194997|	lcl|At1g51055.1	lcl|C04F2.4 CE17368 WBGene	gi|24638847|	gi|6702708|ref|Os01g28130|	gi|6320739|ref|NP_010819.1|	gi|19113512|ref|NP_596720.1|
gi|31194999|	lcl|At1g51090.1	lcl|C04F5.9 CE19673 WBGene	gi|24639004|	gi|6702715|ref|Os01g28190|	gi|6320748|ref|NP_010828.1|	gi|19113540|ref|NP_596748.1|
gi|31195001|	lcl|At1g51230.1	lcl|C04G2.1 CE05211 WBGene	gi|24639333|	gi|6702718|ref|Os01g28220|	gi|6320751|ref|NP_010831.1|	gi|19113544|ref|NP_596752.1|
gi|31195005|	lcl|At1g51240.1	lcl|C04G2.11 CE05219 WBGen	gi|24639396|	gi|6702726|ref|Os01g28300|	gi|6320758|ref|NP_010837.1|	gi|19113552|ref|NP_596760.1|
gi|31195007|	lcl|At1g51250.1	lcl|C04G2.4 CE03853 WBGene	gi|24639399|	gi|6702738|ref|Os01g28420|	gi|6320759|ref|NP_010836.1|	gi|19113567|ref|NP_596775.1|
gi|31195009|	lcl|At1g51280.1	lcl|C04G6.2 CE27792 WBGene	gi|24639432|	gi|6702756|ref|Os01g28600|	gi|6320782|ref|NP_010861.1|	gi|19113568|ref|NP_596776.1|
gi|31195013|	lcl|At1g51290.1	lcl|C04G6.5 CE06766 WBGene	gi|24639651|	gi|6702784|ref|Os01g28800|	gi|6320786|ref|NP_010865.1|	gi|19113580|ref|NP_596788.1|
gi|31195017|	lcl|At1g51320.1	lcl|C04H5.2 CE15588 WBGene	gi|24639659|	gi|6702790|ref|Os01g28860|	gi|6320787|ref|NP_010866.1|	gi|19113590|ref|NP_596798.1|
gi|31195019|	lcl|At1g51670.1	lcl|C05A9.3 CE05223 WBGene	gi|24639703|	gi|6702795|ref|Os01g28910|	gi|6320792|ref|NP_010871.1|	gi|19113600|ref|NP_596808.1|
gi|31195021|	lcl|At1g52020.1	lcl|C05B5.6 CE33966 WBGene	gi|24639723|	gi|6702800|ref|Os01g28960|	gi|6320795|ref|NP_010874.1|	gi|19113626|ref|NP_596834.1|
gi|31195025|	lcl|At1g52060.1	lcl|C05C10.1 CE01465 WBGen	gi|24639762|	gi|6702811|ref|Os01g29070|	gi|6320810|ref|NP_010889.1|	gi|19113633|ref|NP_596841.1|
gi|31195027|	lcl|At1g52110.1	lcl|C05C12.1 CE02965 WBGen	gi|24639830|	gi|6702814|ref|Os01g29100|	gi|6320817|ref|NP_010896.1|	gi|19113634|ref|NP_596842.1|
gi|31195029|	lcl|At1g52450.1	lcl|C05C8.8 CE07926 WBGene	gi|24639974|	gi|6702816|ref|Os01g29120|	gi|6320822|ref|NP_010901.1|	gi|19113642|ref|NP_596850.1|
gi|31195033|	lcl|At1g52480.1	lcl|C05C9.1 CE33968 WBGene	gi|24640019|	gi|6702823|ref|Os01g29190|	gi|6320828|ref|NP_010907.1|	gi|19113645|ref|NP_596853.1|
gi|31195035|	lcl|At1g52490.1	lcl|C05D10.4a CE30423 WBGe	gi|24640031|	gi|6702824|ref|Os01g29200|	gi|6320837|ref|NP_010916.1|	gi|19113652|ref|NP_596860.1|
gi|31195041|	lcl|At1g52670.1	lcl|C05D10.4b CE30424 WBGe	gi|24640155|	gi|6702830|ref|Os01g29260|	gi|6320847|ref|NP_010926.1|	gi|19113658|ref|NP_596866.1|
gi|31195045|	lcl|At1g52850.1	lcl|C05D10.4c CE30425 WBGe	gi|24640194|	gi|6702835|ref|Os01g29310|	gi|6320848|ref|NP_010927.1|	gi|19113675|ref|NP_592763.1|
gi|31195047|	lcl|At1g52950.1	lcl|C05D11.10 CE01129 WBGe	gi|24640211|	gi|6702841|ref|Os01g29370|	gi|6320880|ref|NP_010959.1|	gi|19113678|ref|NP_592766.1|
gi|31195051|	lcl|At1g52970.1	lcl|C05D11.2 CE24785 WBGen	gi|24640213|	gi|6702856|ref|Os01g29510|	gi|6320885|ref|NP_010964.1|	gi|19113681|ref|NP_592769.1|
gi|31195055|	lcl|At1g53340.1	lcl|C05D12.2 CE32301 WBGen	gi|24640399|	gi|6702859|ref|Os01g29540|	gi|6320886|ref|NP_010965.1|	gi|19113688|ref|NP_592776.1|
gi|31195061|	lcl|At1g53360.1	lcl|C05D12.3 CE36974 WBGen	gi|24640411|	gi|6702863|ref|Os01g29580|	gi|6320897|ref|NP_010976.1|	gi|19113690|ref|NP_592778.1|
gi|31195063|	lcl|At1g53370.1	lcl|C05D2.1d CE33555 WBGen	gi|24640413|	gi|6702869|ref|Os01g29640|	gi|6320902|ref|NP_010981.1|	gi|19113698|ref|NP_592786.1|
gi|31195065|	lcl|At1g53550.1	lcl|C05D9.2 CE07928 WBGene	gi|24640415|	gi|6702876|ref|Os01g29710|	gi|6320903|ref|NP_010982.1|	gi|19113713|ref|NP_592801.1|
gi|31195079|	lcl|At1g53980.1	lcl|C05E11.6 CE35383 WBGen	gi|24640464|	gi|6702878|ref|Os01g29730|	gi|6320905|ref|NP_010984.1|	gi|19113716|ref|NP_592804.1|
gi|31195081|	lcl|At1g54070.1	lcl|C05E4.10 CE25756 WBGen	gi|24640564|	gi|6702885|ref|Os01g29800|	gi|6320906|ref|NP_010985.1|	gi|19113728|ref|NP_592816.1|
gi|31195085|	lcl|At1g54240.1	lcl|C05E4.11 CE07944 WBGen	gi|24640593|	gi|6702891|ref|Os01g29850|	gi|6320907|ref|NP_010986.1|	gi|19113738|ref|NP_592826.1|
gi|31195087|	lcl|At1g54550.1	lcl|C05E4.12 CE07945 WBGen	gi|24640689|	gi|6702901|ref|Os01g30940|	gi|6320933|ref|NP_011012.1|	gi|19113748|ref|NP_592836.1|
gi|31195091|	lcl|At1g54620.1	lcl|C05E4.13 CE07946 WBGen	gi|24640695|	gi|6702908|ref|Os01g31010|	gi|6320948|ref|NP_011027.1|	gi|19113753|ref|NP_592841.1|
gi|31195099|	lcl|At1g54760.1	lcl|C05E4.14 CE36912 WBGen	gi|24640759|	gi|6702910|ref|Os01g31030|	gi|6320955|ref|NP_011034.1|	gi|19113756|ref|NP_592844.1|
gi|31195105|	lcl|At1g54840.1	lcl|C05E4.2 CE25757 WBGene	gi|24640769|	gi|6702921|ref|Os01g31140|	gi|6320958|ref|NP_011037.1|	gi|19113792|ref|NP_592880.1|
gi|31195109|	lcl|At1g54850.1	lcl|C05E4.4 CE25758 WBGene	gi|24640773|	gi|6702923|ref|Os01g31160|	gi|6320962|ref|NP_011041.1|	gi|19113799|ref|NP_592887.1|
gi|31195111|	lcl|At1g54980.1	lcl|C05E4.6 CE25759 WBGene	gi|24641049|	gi|6702931|ref|Os01g31240|	gi|6320964|ref|NP_011043.1|	gi|19113800|ref|NP_592888.1|
gi|31195113|	lcl|At1g55010.1	lcl|C05E7.4 CE34155 WBGene	gi|24641066|	gi|6702936|ref|Os01g31290|	gi|6320971|ref|NP_011050.1|	gi|19113809|ref|NP_592897.1|
gi|31195121|	lcl|At1g55070.1	lcl|C06A12.5 CE18489 WBGen	gi|24641178|	gi|6702948|ref|Os01g31410|	gi|6320974|ref|NP_011053.1|	gi|19113840|ref|NP_592928.1|
gi|31195135|	lcl|At1g55380.1	lcl|C06A5.1 CE07948 WBGene	gi|24641353|	gi|6702950|ref|Os01g31430|	gi|6320979|ref|NP_011058.1|	gi|19113842|ref|NP_592930.1|
gi|31195137|	lcl|At1g55390.1	lcl|C06A5.4 CE07951 WBGene	gi|24641355|	gi|6702969|ref|Os01g31620|	gi|6321001|ref|NP_011080.1|	gi|19113855|ref|NP_592943.1|
gi|31195139|	lcl|At1g55410.1	lcl|C06A5.8a CE07955 WBGen	gi|24641497|	gi|6702972|ref|Os01g31650|	gi|6321021|ref|NP_011100.1|	gi|19113867|ref|NP_592955.1|
gi|31195147|	lcl|At1g55420.1	lcl|C06A5.9 CE34249 WBGene	gi|24641517|	gi|6702973|ref|Os01g31660|	gi|6321023|ref|NP_011102.1|	gi|19113882|ref|NP_592970.1|
gi|31195149|	lcl|At1g55430.1	lcl|C06A8.7 CE24789 WBGene	gi|24641550|	gi|6702979|ref|Os01g31710|	gi|6321030|ref|NP_011109.1|	gi|19113892|ref|NP_592980.1|
gi|31195153|	lcl|At1g55440.1	lcl|C06A8.8a CE34656 WBGen	gi|24641622|	gi|6702982|ref|Os01g31740|	gi|6321033|ref|NP_011112.1|	gi|19113913|ref|NP_593001.1|
gi|31195159|	lcl|At1g55770.1	lcl|C06B3.1 CE15593 WBGene	gi|24641626|	gi|6703015|ref|Os01g32050|	gi|6321174|ref|NP_011251.1|	gi|19113940|ref|NP_593028.1|
gi|31195161|	lcl|At1g55950.1	lcl|C06B3.10 CE07967 WBGen	gi|24641646|	gi|6703016|ref|Os01g32060|	gi|6321176|ref|NP_011253.1|	gi|19113941|ref|NP_593029.1|
gi|31195163|	lcl|At1g56400.1	lcl|C06B3.11 CE07968 WBGen	gi|24641648|	gi|6703021|ref|Os01g32100|	gi|6321178|ref|NP_011255.1|	gi|19113946|ref|NP_593034.1|
gi|31195165|	lcl|At1g56610.1	lcl|C06B3.9 CE15594 WBGene	gi|24641781|	gi|6703032|ref|Os01g32210|	gi|6321180|ref|NP_011257.1|	gi|19113982|ref|NP_593070.1|
gi|31195167|	lcl|At1g56620.1	lcl|C06B8.1 CE07969 WBGene	gi|24641796|	gi|6703053|ref|Os01g32400|	gi|6321205|ref|NP_011282.1|	gi|19113983|ref|NP_593071.1|
gi|31195169|	lcl|At1g57580.1	lcl|C06B8.10 CE34469 WBGen	gi|24641798|	gi|6703055|ref|Os01g32420|	gi|6321209|ref|NP_011286.1|	gi|19113986|ref|NP_593074.1|
gi|31195171|	lcl|At1g57630.1	lcl|C06B8.2a CE32303 WBGen	gi|24641862|	gi|6703068|ref|Os01g32550|	gi|6321212|ref|NP_011289.1|	gi|19113996|ref|NP_593084.1|
gi|31195173|	lcl|At1g57670.1	lcl|C06B8.2b CE32304 WBGen	gi|24642002|	gi|6703070|ref|Os01g32570|	gi|6321215|ref|NP_011292.1|	gi|19114014|ref|NP_593102.1|
gi|31195175|	lcl|At1g57690.1	lcl|C06B8.4 CE07972 WBGene	gi|24642099|	gi|6703071|ref|Os01g32580|	gi|6321225|ref|NP_011302.1|	gi|19114015|ref|NP_593103.1|
gi|31195187|	lcl|At1g57780.1	lcl|C06B8.6 CE35953 WBGene	gi|24642123|	gi|6703072|ref|Os01g32590|	gi|6321241|ref|NP_011318.1|	gi|19114017|ref|NP_593105.1|
gi|31195189|	lcl|At1g57830.1	lcl|C06B8.9 CE33755 WBGene	gi|24642189|	gi|6703081|ref|Os01g32680|	gi|6321252|ref|NP_011329.1|	gi|19114021|ref|NP_593109.1|
gi|31195191|	lcl|At1g57850.1	lcl|C06C3.3 CE35823 WBGene	gi|24642231|	gi|6703103|ref|Os01g32890|	gi|6321262|ref|NP_011339.1|	gi|19114029|ref|NP_593117.1|
gi|31195195|	lcl|At1g58090.1	lcl|C06C3.6 CE35954 WBGene	gi|24642254|	gi|6703131|ref|Os01g33160|	gi|6321278|ref|NP_011355.1|	gi|19114030|ref|NP_593118.1|
gi|31195201|	lcl|At1g58160.1	lcl|C06C3.8 CE35824 WBGene	gi|24642271|	gi|6703153|ref|Os01g33380|	gi|6321283|ref|NP_011360.1|	gi|19114044|ref|NP_593132.1|
gi|31195203|	lcl|At1g59630.1	lcl|C06C6.1 CE15595 WBGene	gi|24642278|	gi|6703155|ref|Os01g33400|	gi|6321284|ref|NP_011361.1|	gi|19114049|ref|NP_593137.1|
gi|31195207|	lcl|At1g59675.1	lcl|C06C6.2 CE15596 WBGene	gi|24642365|	gi|6703158|ref|Os01g33430|	gi|6321305|ref|NP_011382.1|	gi|19114072|ref|NP_593160.1|
gi|31195217|	lcl|At1g59680.1	lcl|C06C6.3 CE35384 WBGene	gi|24642525|	gi|6703167|ref|Os01g33520|	gi|6321307|ref|NP_011384.1|	gi|19114079|ref|NP_593167.1|
gi|31195221|	lcl|At1g59810.1	lcl|C06C6.5a CE15599 WBGen	gi|24642527|	gi|6703168|ref|Os01g33530|	gi|6321314|ref|NP_011391.1|	gi|19114080|ref|NP_593168.1|
gi|31195223|	lcl|At1g60040.1	lcl|C06C6.5b CE29171 WBGen	gi|24642683|	gi|6703177|ref|Os01g33620|	gi|6321327|ref|NP_011404.1|	gi|19114083|ref|NP_593171.1|
gi|31195229|	lcl|At1g60180.1	lcl|C06C6.6 CE15600 WBGene	gi|24642685|	gi|6703178|ref|Os01g33630|	gi|6321328|ref|NP_011405.1|	gi|19114090|ref|NP_593178.1|
gi|31195231|	lcl|At1g60320.1	lcl|C06C6.7 CE15601 WBGene	gi|24642817|	gi|6703183|ref|Os01g33680|	gi|6321331|ref|NP_011408.1|	gi|19114102|ref|NP_593190.1|

gi|31195233|	lcl|At1g60370.1	lcl|C06C6.8 CE17372 WBGene	gi|24642884|	gi|6703185|ref|Os01g33700|	gi|6321349|ref|NP_011426.1|	gi|19114105|ref|NP_593193.1|
gi|31195253|	lcl|At1g60570.1	lcl|C06C6.9 CE17373 WBGene	gi|24643020|	gi|6703217|ref|Os01g34020|	gi|6321357|ref|NP_011434.1|	gi|19114142|ref|NP_593230.1|
gi|31195255|	lcl|At1g60760.1	lcl|C06E1.7 CE30483 WBGene	gi|24643047|	gi|6703218|ref|Os01g34030|	gi|6321381|ref|NP_011458.1|	gi|19114145|ref|NP_593233.1|
gi|31195257|	lcl|At1g60880.1	lcl|C06E2.8 CE35386 WBGene	gi|24643066|	gi|6703237|ref|Os01g34220|	gi|6321385|ref|NP_011462.1|	gi|19114162|ref|NP_593250.1|
gi|31195259|	lcl|At1g60920.1	lcl|C06E4.7 CE34658 WBGene	gi|24643068|	gi|6703242|ref|Os01g34270|	gi|6321387|ref|NP_011464.1|	gi|19114169|ref|NP_593257.1|
gi|31195267|	lcl|At1g60990.1	lcl|C06E7.1b CE37186 WBGen	gi|24643076|	gi|6703251|ref|Os01g34360|	gi|6321409|ref|NP_011486.1|	gi|19114171|ref|NP_593259.1|
gi|31195269|	lcl|At1g61060.1	lcl|C06E7.7 CE33361 WBGene	gi|24643102|	gi|6703273|ref|Os01g34580|	gi|6321410|ref|NP_011487.1|	gi|19114184|ref|NP_593272.1|
gi|31195275|	lcl|At1g61230.1	lcl|C06E8.5 CE00801 WBGene	gi|24643172|	gi|6703291|ref|Os01g34720|	gi|6321417|ref|NP_011494.1|	gi|19114186|ref|NP_593274.1|
gi|31195277|	lcl|At1g61320.1	lcl|C06G1.1 CE27054 WBGene	gi|24643200|	gi|6703293|ref|Os01g34740|	gi|6321418|ref|NP_011495.1|	gi|19114195|ref|NP_593283.1|
gi|31195287|	lcl|At1g61330.1	lcl|C06G3.4 CE07980 WBGene	gi|24643209|	gi|6703295|ref|Os01g34760|	gi|6321422|ref|NP_011499.1|	gi|19114201|ref|NP_593289.1|
gi|31195301|	lcl|At1g61540.1	lcl|C06G3.6 CE07982 WBGene	gi|24643367|	gi|6703296|ref|Os01g34770|	gi|6321425|ref|NP_011502.1|	gi|19114216|ref|NP_593304.1|
gi|31195305|	lcl|At1g61563.1	lcl|C06G4.1 CE28203 WBGene	gi|24643435|	gi|6703301|ref|Os01g34820|	gi|6321441|ref|NP_011518.1|	gi|19114221|ref|NP_593309.1|
gi|31195317|	lcl|At1g61566.1	lcl|C06G8.4 CE05232 WBGene	gi|24643445|	gi|6703329|ref|Os01g35080|	gi|6321450|ref|NP_011527.1|	gi|19114225|ref|NP_593313.1|
gi|31195319|	lcl|At1g61710.1	lcl|C06H2.5 CE05237 WBGene	gi|24643505|	gi|6703331|ref|Os01g35100|	gi|6321454|ref|NP_011531.1|	gi|19114239|ref|NP_593327.1|
gi|31195323|	lcl|At1g61830.1	lcl|C06H2.7 CE34661 WBGene	gi|24643507|	gi|6703342|ref|Os01g35210|	gi|6321459|ref|NP_011537.1|	gi|19114257|ref|NP_593345.1|
gi|31195325|	lcl|At1g61840.1	lcl|C06H5.1 CE27668 WBGene	gi|24643578|	gi|6703347|ref|Os01g35260|	gi|6321481|ref|NP_011558.1|	gi|19114274|ref|NP_593362.1|
gi|31195329|	lcl|At1g61930.1	lcl|C06H5.2 CE15604 WBGene	gi|24643741|	gi|6703348|ref|Os01g35270|	gi|6321508|ref|NP_011585.1|	gi|19114286|ref|NP_593374.1|
gi|31195343|	lcl|At1g62030.1	lcl|C06H5.7 CE31877 WBGene	gi|24643867|	gi|6703358|ref|Os01g35370|	gi|6321509|ref|NP_011586.1|	gi|19114292|ref|NP_593380.1|
gi|31195345|	lcl|At1g62270.1	lcl|C07A12.1a CE29567 WBGe	gi|24643879|	gi|6703359|ref|Os01g35380|	gi|6321512|ref|NP_011589.1|	gi|19114294|ref|NP_593382.1|
gi|31195349|	lcl|At1g62490.1	lcl|C07A12.1b CE25763 WBGe	gi|24643976|	gi|6703361|ref|Os01g35400|	gi|6321517|ref|NP_011594.1|	gi|19114298|ref|NP_593386.1|
gi|31195351|	lcl|At1g62975.1	lcl|C07A12.5a CE36266 WBGe	gi|24644047|	gi|6703364|ref|Os01g35430|	gi|6321530|ref|NP_011607.1|	gi|19114318|ref|NP_593406.1|
gi|31195359|	lcl|At1g63190.1	lcl|C07A12.5b CE36267 WBGe	gi|24644112|	gi|6703368|ref|Os01g35470|	gi|6321534|ref|NP_011611.1|	gi|19114334|ref|NP_593422.1|
gi|31195361|	lcl|At1g63200.1	lcl|C07A9.1 CE37745 WBGene	gi|24644121|	gi|6703377|ref|Os01g35560|	gi|6321555|ref|NP_011632.1|	gi|19114351|ref|NP_593439.1|
gi|31195363|	lcl|At1g63205.1	lcl|C07A9.12 CE37746 WBGen	gi|24644214|	gi|6703389|ref|Os01g35670|	gi|6321595|ref|NP_011672.1|	gi|19114361|ref|NP_593449.1|
gi|31195365|	lcl|At1g63870.1	lcl|C07D10.3 CE34662 WBGen	gi|24644327|	gi|6703395|ref|Os01g35730|	gi|6321597|ref|NP_011674.1|	gi|19114363|ref|NP_593451.1|
gi|31195373|	lcl|At1g64130.1	lcl|C07E3.4 CE02120 WBGene	gi|24644498|	gi|6703424|ref|Os01g36020|	gi|6321616|ref|NP_011693.1|	gi|19114421|ref|NP_593509.1|
gi|31195375|	lcl|At1g64540.1	lcl|C07E3.5 CE02121 WBGene	gi|24644500|	gi|6703434|ref|Os01g36100|	gi|6321622|ref|NP_011699.1|	gi|19114429|ref|NP_593517.1|
gi|31195377|	lcl|At1g64840.1	lcl|C07E3.6 CE35825 WBGene	gi|24644507|	gi|6703438|ref|Os01g36140|	gi|6321637|ref|NP_011714.1|	gi|19114466|ref|NP_593554.1|
gi|31195379|	lcl|At1g65050.1	lcl|C07E3.8 CE02123 WBGene	gi|24644517|	gi|6703442|ref|Os01g36180|	gi|6321642|ref|NP_011719.1|	gi|19114489|ref|NP_593577.1|
gi|31195381|	lcl|At1g65080.1	lcl|C07G1.2 CE34664 WBGene	gi|24644596|	gi|6703455|ref|Os01g36310|	gi|6321645|ref|NP_011722.1|	gi|19114516|ref|NP_593604.1|
gi|31195387|	lcl|At1g65110.1	lcl|C07G1.4b CE32573 WBGen	gi|24644626|	gi|6703464|ref|Os01g36380|	gi|6321651|ref|NP_011728.1|	gi|19114518|ref|NP_593606.1|
gi|31195391|	lcl|At1g65150.1	lcl|C07G2.1a CE00665 WBGen	gi|24644653|	gi|6703466|ref|Os01g36400|	gi|6321652|ref|NP_011729.1|	gi|19114522|ref|NP_593610.1|
gi|31195395|	lcl|At1g65180.1	lcl|C07G2.1b CE33971 WBGen	gi|24644655|	gi|6703470|ref|Os01g36440|	gi|6321661|ref|NP_011738.1|	gi|19114525|ref|NP_593613.1|
gi|31195401|	lcl|At1g65760.1	lcl|C07G2.2a CE33597 WBGen	gi|24644762|	gi|6703471|ref|Os01g36450|	gi|6321669|ref|NP_011746.1|	gi|19114528|ref|NP_593616.1|
gi|31195403|	lcl|At1g65770.1	lcl|C07G2.2b CE19688 WBGen	gi|24644894|	gi|6703493|ref|Os01g36670|	gi|6321672|ref|NP_011749.1|	gi|19114533|ref|NP_593621.1|
gi|31195405|	lcl|At1g66000.1	lcl|C07G2.2c CE33598 WBGen	gi|24645182|	gi|6703499|ref|Os01g36730|	gi|6321686|ref|NP_011763.1|	gi|19114569|ref|NP_593657.1|
gi|31195407|	lcl|At1g66045.1	lcl|C07G3.3 CE07994 WBGene	gi|24645189|	gi|6703500|ref|Os01g36740|	gi|6321702|ref|NP_011779.1|	gi|19114605|ref|NP_593693.1|
gi|31195409|	lcl|At1g66060.1	lcl|C07G3.4 CE07995 WBGene	gi|24645501|	gi|6703503|ref|Os01g36770|	gi|6321718|ref|NP_011795.1|	gi|19114612|ref|NP_593700.1|
gi|31195411|	lcl|At1g66090.1	lcl|C07G3.5 CE07996 WBGene	gi|24645511|	gi|6703506|ref|Os01g36800|	gi|6321722|ref|NP_011799.1|	gi|19114622|ref|NP_593710.1|
gi|31195413|	lcl|At1g66110.1	lcl|C07G3.6 CE07997 WBGene	gi|24645587|	gi|6703511|ref|Os01g36850|	gi|6321727|ref|NP_011804.1|	gi|19114624|ref|NP_593712.1|
gi|31195421|	lcl|At1g66420.1	lcl|C07G3.7 CE07998 WBGene	gi|24645722|	gi|6703519|ref|Os01g36910|	gi|6321732|ref|NP_011810.1|	gi|19114627|ref|NP_593715.1|
gi|31195423|	lcl|At1g66440.1	lcl|C07G3.8 CE07999 WBGene	gi|24645732|	gi|6703533|ref|Os01g37050|	gi|6321734|ref|NP_011811.1|	gi|19114644|ref|NP_593732.1|
gi|31195425|	lcl|At1g66450.1	lcl|C07H6.1 CE34522 WBGene	gi|24645897|	gi|6703536|ref|Os01g37080|	gi|6321737|ref|NP_011813.1|	gi|19114674|ref|NP_593762.1|
gi|31195427|	lcl|At1g66490.1	lcl|C07H6.2 CE00756 WBGene	gi|24645990|	gi|6703545|ref|Os01g37160|	gi|6321739|ref|NP_011815.1|	gi|19114694|ref|NP_593782.1|
gi|31195429|	lcl|At1g66640.1	lcl|C08A9.6 CE06788 WBGene	gi|24646240|	gi|6703552|ref|Os01g37230|	gi|6321741|ref|NP_011817.1|	gi|19114702|ref|NP_593790.1|
gi|31195433|	lcl|At1g67130.1	lcl|C08A9.7 CE06789 WBGene	gi|24646267|	gi|6703554|ref|Os01g37250|	gi|6321743|ref|NP_011819.1|	gi|19114703|ref|NP_593791.1|
gi|31195447|	lcl|At1g67160.1	lcl|C08A9.8 CE06790 WBGene	gi|24646478|	gi|6703555|ref|Os01g37260|	gi|6321744|ref|NP_011820.1|	gi|19114729|ref|NP_593817.1|
gi|31195459|	lcl|At1g67450.1	lcl|C08A9.9 CE06791 WBGene	gi|24646494|	gi|6703559|ref|Os01g37300|	gi|6321745|ref|NP_011821.1|	gi|19114745|ref|NP_593833.1|
gi|31195461|	lcl|At1g67455.1	lcl|C08B6.12 CE34665 WBGen	gi|24646530|	gi|6703561|ref|Os01g37320|	gi|6321748|ref|NP_011824.1|	gi|19114769|ref|NP_593857.1|
gi|31195469|	lcl|At1g67623.1	lcl|C08B6.13 CE37807 WBGen	gi|24646589|	gi|6703565|ref|Os01g37360|	gi|6321753|ref|NP_011829.1|	gi|19114782|ref|NP_593870.1|
gi|31195473|	lcl|At1g68960.1	lcl|C08B6.5 CE05242 WBGene	gi|24646722|	gi|6703572|ref|Os01g37430|	gi|6321758|ref|NP_011834.1|	gi|19114827|ref|NP_593915.1|
gi|31195477|	lcl|At1g69090.1	lcl|C08C3.4 CE00069 WBGene	gi|24646756|	gi|6703584|ref|Os01g37550|	gi|6321759|ref|NP_011835.1|	gi|19114831|ref|NP_593919.1|
gi|31195479|	lcl|At1g69150.1	lcl|C08E3.10 CE34666 WBGen	gi|24646774|	gi|6703587|ref|Os01g37570|	gi|6321762|ref|NP_011838.1|	gi|19114857|ref|NP_593945.1|
gi|31195487|	lcl|At1g69550.1	lcl|C08E3.12 CE08015 WBGen	gi|24646782|	gi|6703590|ref|Os01g37600|	gi|6321764|ref|NP_011840.1|	gi|19114859|ref|NP_593947.1|
gi|31195489|	lcl|At1g69650.1	lcl|C08E3.2 CE08005 WBGene	gi|24646788|	gi|6703597|ref|Os01g37670|	gi|6321769|ref|NP_011845.1|	gi|19114862|ref|NP_593950.1|
gi|31195497|	lcl|At1g69660.1	lcl|C08E3.4 CE32119 WBGene	gi|24646857|	gi|6703602|ref|Os01g37720|	gi|6321779|ref|NP_011855.1|	gi|19114869|ref|NP_593957.1|
gi|31195503|	lcl|At1g70020.1	lcl|C08E3.5 CE34667 WBGene	gi|24646947|	gi|6703620|ref|Os01g37880|	gi|6321794|ref|NP_011870.1|	gi|19114871|ref|NP_593959.1|
gi|31195505|	lcl|At1g70040.1	lcl|C08E3.6 CE08009 WBGene	gi|24647187|	gi|6703625|ref|Os01g37930|	gi|6321811|ref|NP_011887.1|	gi|19114872|ref|NP_593960.1|
gi|31195509|	lcl|At1g70120.1	lcl|C08E3.7 CE08010 WBGene	gi|24647189|	gi|6703636|ref|Os01g38040|	gi|6321827|ref|NP_011903.1|	gi|19114875|ref|NP_593963.1|
gi|31195515|	lcl|At1g70380.1	lcl|C08E3.8 CE08011 WBGene	gi|24647209|	gi|6703639|ref|Os01g38070|	gi|6321833|ref|NP_011909.1|	gi|19114876|ref|NP_593964.1|
gi|31195519|	lcl|At1g70390.1	lcl|C08E3.9 CE08012 WBGene	gi|24647224|	gi|6703641|ref|Os01g38090|	gi|6321834|ref|NP_011910.1|	gi|19114881|ref|NP_593969.1|
gi|31195525|	lcl|At1g70540.1	lcl|C08E8.1 CE17375 WBGene	gi|24647227|	gi|6703648|ref|Os01g38160|	gi|6321843|ref|NP_011919.1|	gi|19114888|ref|NP_593976.1|
gi|31195529|	lcl|At1g70700.1	lcl|C08F1.1 CE36683 WBGene	gi|24647276|	gi|6703658|ref|Os01g38260|	gi|6321852|ref|NP_011928.1|	gi|19114889|ref|NP_593977.1|
gi|31195531|	lcl|At1g70870.1	lcl|C08F1.3 CE08018 WBGene	gi|24647278|	gi|6703662|ref|Os01g38300|	gi|6321858|ref|NP_011934.1|	gi|19114898|ref|NP_593986.1|
gi|31195533|	lcl|At1g70960.1	lcl|C08F1.4a CE27059 WBGen	gi|24647280|	gi|6703675|ref|Os01g38430|	gi|6321873|ref|NP_011949.1|	gi|19114900|ref|NP_593988.1|
gi|31195541|	lcl|At1g70970.1	lcl|C08F1.4b CE31771 WBGen	gi|24647411|	gi|6703678|ref|Os01g38460|	gi|6321876|ref|NP_011952.1|	gi|19114912|ref|NP_594000.1|
gi|31195561|	lcl|At1g71200.1	lcl|C08F1.5 CE31772 WBGene	gi|24647413|	gi|6703687|ref|Os01g38550|	gi|6321877|ref|NP_011953.1|	gi|19114922|ref|NP_594010.1|
gi|31195575|	lcl|At1g71320.1	lcl|C08F11.11 CE17388 WBGe	gi|24647450|	gi|6703712|ref|Os01g38790|	gi|6321897|ref|NP_011973.1|	gi|19114940|ref|NP_594028.1|
gi|31195581|	lcl|At1g72290.1	lcl|C08F11.12 CE17389 WBGe	gi|24647452|	gi|6703714|ref|Os01g38810|	gi|6321901|ref|NP_011977.1|	gi|19114950|ref|NP_594038.1|
gi|31195587|	lcl|At1g72870.1	lcl|C08F11.4 CE34524 WBGen	gi|24647479|	gi|6703718|ref|Os01g38850|	gi|6321908|ref|NP_011984.1|	gi|19114955|ref|NP_594043.1|
gi|31195589|	lcl|At1g72900.1	lcl|C08F11.5 CE17383 WBGen	gi|24647582|	gi|6703738|ref|Os01g39010|	gi|6321916|ref|NP_011992.1|	gi|19114958|ref|NP_594046.1|
gi|31195607|	lcl|At1g73325.1	lcl|C08F11.7 CE17384 WBGen	gi|24647685|	gi|6703755|ref|Os01g39180|	gi|6321919|ref|NP_011995.1|	gi|19114959|ref|NP_594047.1|
gi|31195621|	lcl|At1g73330.1	lcl|C08F11.9 CE35388 WBGen	gi|24647729|	gi|6703756|ref|Os01g39190|	gi|6321930|ref|NP_012006.1|	gi|19114964|ref|NP_594052.1|
gi|31195627|	lcl|At1g75720.1	lcl|C08F8.5 CE05252 WBGene	gi|24647823|	gi|6703785|ref|Os01g39460|	gi|6321931|ref|NP_012007.1|	gi|19114983|ref|NP_594071.1|
gi|31195629|	lcl|At1g75830.1	lcl|C08G5.1 CE36684 WBGene	gi|24648004|	gi|6703786|ref|Os01g39470|	gi|6321940|ref|NP_012016.1|	gi|19114996|ref|NP_594084.1|
gi|31195631|	lcl|At1g76220.1	lcl|C08G5.3 CE19692 WBGene	gi|24648008|	gi|6703814|ref|Os01g39740|	gi|6321944|ref|NP_012020.1|	gi|19115014|ref|NP_594102.1|
gi|31195637|	lcl|At1g76440.1	lcl|C08G9.1 CE31155 WBGene	gi|24648042|	gi|6703844|ref|Os01g40040|	gi|6321946|ref|NP_012022.1|	gi|19115015|ref|NP_594103.1|

gi|31195643|	lcl|At1g76770.1	lcl|C08H9.11 CE02996 WBGen	gi|24648156|	gi|6703854|ref|Os01g40130|	gi|6321949|ref|NP_012025.1|	gi|19115025|ref|NP_594113.1|
gi|31195653|	lcl|At1g76830.1	lcl|C08H9.14 CE02999 WBGen	gi|24648163|	gi|6703857|ref|Os01g40160|	gi|6321950|ref|NP_012026.1|	gi|19115046|ref|NP_594134.1|
gi|31195657|	lcl|At1g77650.1	lcl|C08H9.6 CE02992 WBGen	gi|24648171|	gi|6703866|ref|Os01g40240|	gi|6321966|ref|NP_012042.1|	gi|19115048|ref|NP_594136.1|
gi|31195707|	lcl|At1g77815.1	lcl|C09B9.6 CE06798 WBGene	gi|24648173|	gi|6703899|ref|Os01g40550|	gi|6321969|ref|NP_012045.1|	gi|19115056|ref|NP_594144.1|
gi|31195713|	lcl|At1g77830.1	lcl|C09B9.7 CE06799 WBGene	gi|24648181|	gi|6703926|ref|Os01g40780|	gi|6321976|ref|NP_012052.1|	gi|19115074|ref|NP_594162.1|
gi|31195741|	lcl|At1g78310.1	lcl|C09D1.2 CE34253 WBGene	gi|24648393|	gi|6703928|ref|Os01g40800|	gi|6321983|ref|NP_012059.1|	gi|19115084|ref|NP_594172.1|
gi|31195759|	lcl|At1g78355.1	lcl|C09E7.1 CE25766 WBGene	gi|24648500|	gi|6703936|ref|Os01g40880|	gi|6321986|ref|NP_012062.1|	gi|19115085|ref|NP_594173.1|
gi|31195761|	lcl|At1g78410.1	lcl|C09E7.4 CE25768 WBGene	gi|24648561|	gi|6703938|ref|Os01g40900|	gi|6321990|ref|NP_012066.1|	gi|19115122|ref|NP_594210.1|
gi|31195773|	lcl|At1g78470.1	lcl|C09E7.5 CE25769 WBGene	gi|24648563|	gi|6703943|ref|Os01g40950|	gi|6321993|ref|NP_012069.1|	gi|19115143|ref|NP_594231.1|
gi|31195781|	lcl|At1g78640.1	lcl|C09E7.7 CE25771 WBGene	gi|24648566|	gi|6703945|ref|Os01g40970|	gi|6322003|ref|NP_012079.1|	gi|19115168|ref|NP_594256.1|
gi|31195791|	lcl|At1g78730.1	lcl|C09E7.8a CE32121 WBGen	gi|24648570|	gi|6703954|ref|Os01g41060|	gi|6322005|ref|NP_012081.1|	gi|19115181|ref|NP_594269.1|
gi|31195797|	lcl|At1g78740.1	lcl|C09E7.8b CE32122 WBGen	gi|24648751|	gi|6703956|ref|Os01g41080|	gi|6322007|ref|NP_012083.1|	gi|19115213|ref|NP_594301.1|
gi|31195799|	lcl|At1g78840.1	lcl|C09E7.9 CE29078 WBGene	gi|24648753|	gi|6703974|ref|Os01g41260|	gi|6322018|ref|NP_012093.1|	gi|19115234|ref|NP_594322.1|
gi|31195805|	lcl|At1g80450.1	lcl|C09E8.2a CE19348 WBGen	gi|24649183|	gi|6703975|ref|Os01g41270|	gi|6322040|ref|NP_012115.1|	gi|19115251|ref|NP_594339.1|
gi|31195823|	lcl|At1g80970.1	lcl|C09E8.2b CE32123 WBGen	gi|24649185|	gi|6703982|ref|Os01g41340|	gi|6322051|ref|NP_012126.1|	gi|19115273|ref|NP_594361.1|
gi|31195853|	lcl|At1g80990.1	lcl|C09F12.3 CE33973 WBGen	gi|24649665|	gi|6703984|ref|Os01g41360|	gi|6322052|ref|NP_012127.1|	gi|19115275|ref|NP_594363.1|
gi|31195859|	lcl|At2g01360.1	lcl|C09G1.4 CE08042 WBGene	gi|24649675|	gi|6703998|ref|Os01g41500|	gi|6322090|ref|NP_012165.1|	gi|19115279|ref|NP_594367.1|
gi|31195861|	lcl|At2g01390.1	lcl|C09G12.10 CE37083 WBGe	gi|24649681|	gi|6704018|ref|Os01g41700|	gi|6322122|ref|NP_012197.1|	gi|19115293|ref|NP_594381.1|
gi|31195871|	lcl|At2g01520.1	lcl|C09G12.11 CE37084 WBGe	gi|24649770|	gi|6704066|ref|Os01g42170|	gi|6322133|ref|NP_012208.1|	gi|19115304|ref|NP_594392.1|
gi|31195873|	lcl|At2g01530.1	lcl|C09G12.16 CE37190 WBGe	gi|24649842|	gi|6704083|ref|Os01g42340|	gi|6322136|ref|NP_012211.1|	gi|19115311|ref|NP_594399.1|
gi|31195877|	lcl|At2g01560.1	lcl|C09G12.2 CE16836 WBGen	gi|24650068|	gi|6704102|ref|Os01g42510|	gi|6322150|ref|NP_012225.1|	gi|19115328|ref|NP_594416.1|
gi|31195885|	lcl|At2g01700.1	lcl|C09G12.3 CE37085 WBGen	gi|24650273|	gi|6704106|ref|Os01g42540|	gi|6322153|ref|NP_012228.1|	gi|19115330|ref|NP_594418.1|
gi|31195887|	lcl|At2g02030.1	lcl|C09G12.6 CE37191 WBGen	gi|24650342|	gi|6704142|ref|Os01g42860|	gi|6322172|ref|NP_012247.1|	gi|19115339|ref|NP_594427.1|
gi|31195895|	lcl|At2g02200.1	lcl|C09G5.3 CE01483 WBGene	gi|24650344|	gi|6704185|ref|Os01g43200|	gi|6322173|ref|NP_012248.1|	gi|19115344|ref|NP_594432.1|
gi|31195911|	lcl|At2g02210.1	lcl|C09G5.4 CE01484 WBGene	gi|24650346|	gi|6704196|ref|Os01g43310|	gi|6322174|ref|NP_012249.1|	gi|19115349|ref|NP_594437.1|
gi|31195913|	lcl|At2g02610.1	lcl|C09G5.5 CE01485 WBGene	gi|24650348|	gi|6704236|ref|Os01g43670|	gi|6322176|ref|NP_012251.1|	gi|19115356|ref|NP_594444.1|
gi|31195915|	lcl|At2g02620.1	lcl|C09G9.2 CE35389 WBGene	gi|24650350|	gi|6704251|ref|Os01g43820|	gi|6322179|ref|NP_012254.1|	gi|19115365|ref|NP_594453.1|
gi|31195921|	lcl|At2g02630.1	lcl|C09G9.3 CE03002 WBGene	gi|24650352|	gi|6704252|ref|Os01g43830|	gi|6322197|ref|NP_012272.1|	gi|19115379|ref|NP_594467.1|
gi|31195929|	lcl|At2g02640.1	lcl|C09G9.7 CE03006 WBGene	gi|24650354|	gi|6704268|ref|Os01g43990|	gi|6322202|ref|NP_012277.1|	gi|19115380|ref|NP_594468.1|
gi|31195935|	lcl|At2g02660.1	lcl|C09H10.10 CE23538 WBGe	gi|24650356|	gi|6704271|ref|Os01g44020|	gi|6322203|ref|NP_012278.1|	gi|19115381|ref|NP_594469.1|
gi|31195937|	lcl|At2g02680.1	lcl|C09H10.6 CE03007 WBGen	gi|24650358|	gi|6704297|ref|Os01g44250|	gi|6322205|ref|NP_012280.1|	gi|19115382|ref|NP_594470.1|
gi|31195945|	lcl|At2g02690.1	lcl|C09H10.8 CE35828 WBGen	gi|24650363|	gi|6704319|ref|Os01g44950|	gi|6322208|ref|NP_012283.1|	gi|19115384|ref|NP_594472.1|
gi|31195949|	lcl|At2g02700.1	lcl|C09H5.3 CE08045 WBGene	gi|24650373|	gi|6704347|ref|Os01g45220|	gi|6322216|ref|NP_012291.1|	gi|19115408|ref|NP_594496.1|
gi|31195955|	lcl|At2g02890.1	lcl|C09H5.4 CE08046 WBGene	gi|24650393|	gi|6704369|ref|Os01g45430|	gi|6322221|ref|NP_012296.1|	gi|19115409|ref|NP_594497.1|
gi|31195957|	lcl|At2g03020.1	lcl|C09H5.5 CE08047 WBGene	gi|24650471|	gi|6704384|ref|Os01g45580|	gi|6322230|ref|NP_012305.1|	gi|19115412|ref|NP_594500.1|
gi|31195967|	lcl|At2g03030.1	lcl|C09H5.6 CE08048 WBGene	gi|24650587|	gi|6704387|ref|Os01g45610|	gi|6322232|ref|NP_012307.1|	gi|19115423|ref|NP_594511.1|
gi|31195973|	lcl|At2g03300.1	lcl|C09H5.9 CE08051 WBGene	gi|24650796|	gi|6704391|ref|Os01g45650|	gi|6322233|ref|NP_012308.1|	gi|19115434|ref|NP_594522.1|
gi|31195975|	lcl|At2g03460.1	lcl|C09H6.1a CE30487 WBGen	gi|24650851|	gi|6704394|ref|Os01g45680|	gi|6322238|ref|NP_012312.1|	gi|19115441|ref|NP_594529.1|
gi|31195977|	lcl|At2g03610.1	lcl|C09H6.1b CE15609 WBGen	gi|24650860|	gi|6704408|ref|Os01g45800|	gi|6322244|ref|NP_012318.1|	gi|19115451|ref|NP_594539.1|
gi|31195981|	lcl|At2g04037.1	lcl|C10B5.1 CE30722 WBGene	gi|24650864|	gi|6704438|ref|Os01g46100|	gi|6322248|ref|NP_012322.1|	gi|19115457|ref|NP_594545.1|
gi|31195985|	lcl|At2g04041.1	lcl|C10C5.2 CE03010 WBGene	gi|24650887|	gi|6704463|ref|Os01g46330|	gi|6322252|ref|NP_012326.1|	gi|19115463|ref|NP_594551.1|
gi|31195993|	lcl|At2g04170.1	lcl|C10E2.1 CE35393 WBGene	gi|24650910|	gi|6704474|ref|Os01g46440|	gi|6322258|ref|NP_012332.1|	gi|19115464|ref|NP_594552.1|
gi|31195995|	lcl|At2g04190.1	lcl|C10G11.2 CE20497 WBGen	gi|24651028|	gi|6704480|ref|Os01g46500|	gi|6322260|ref|NP_012334.1|	gi|19115515|ref|NP_594603.1|
gi|31195997|	lcl|At2g04210.1	lcl|C10G11.3 CE20498 WBGen	gi|24651060|	gi|6704486|ref|Os01g46560|	gi|6322275|ref|NP_012349.1|	gi|19115520|ref|NP_594608.1|
gi|31195999|	lcl|At2g04395.1	lcl|C10G11.4 CE08083 WBGen	gi|24651062|	gi|6704514|ref|Os01g46780|	gi|6322282|ref|NP_012356.1|	gi|19115530|ref|NP_594618.1|
gi|31196001|	lcl|At2g04500.1	lcl|C10G11.7 CE08086 WBGen	gi|24651064|	gi|6704526|ref|Os01g46890|	gi|6322287|ref|NP_012361.1|	gi|19115534|ref|NP_594622.1|
gi|31196031|	lcl|At2g04680.1	lcl|C10G8.1 CE08072 WBGene	gi|24651098|	gi|6704539|ref|Os01g47020|	gi|6322298|ref|NP_012372.1|	gi|19115546|ref|NP_594634.1|
gi|31196043|	lcl|At2g04730.1	lcl|C10G8.2 CE08073 WBGene	gi|24651100|	gi|6704550|ref|Os01g47130|	gi|6322304|ref|NP_012378.1|	gi|19115558|ref|NP_594646.1|
gi|31196045|	lcl|At2g04810.1	lcl|C10G8.3 CE20494 WBGene	gi|24651179|	gi|6704553|ref|Os01g47160|	gi|6322305|ref|NP_012379.1|	gi|19115561|ref|NP_594649.1|
gi|31196047|	lcl|At2g04920.1	lcl|C10G8.4 CE08075 WBGene	gi|24651240|	gi|6704561|ref|Os01g47240|	gi|6322310|ref|NP_012384.1|	gi|19115616|ref|NP_594704.1|
gi|31196057|	lcl|At2g04970.1	lcl|C10H11.7 CE08096 WBGen	gi|24651529|	gi|6704640|ref|Os01g47890|	gi|6322312|ref|NP_012386.1|	gi|19115631|ref|NP_594719.1|
gi|31196165|	lcl|At2g04980.1	lcl|C11E4.3 CE08103 WBGene	gi|24651794|	gi|6704645|ref|Os01g47940|	gi|6322315|ref|NP_012389.1|	gi|19115673|ref|NP_594761.1|
gi|31196201|	lcl|At2g04990.1	lcl|C11E4.4 CE08104 WBGene	gi|24651875|	gi|6704648|ref|Os01g47970|	gi|6322329|ref|NP_012403.1|	gi|19115684|ref|NP_594772.1|
gi|31196225|	lcl|At2g05450.1	lcl|C11G6.1 CE05255 WBGene	gi|24651879|	gi|6704661|ref|Os01g48090|	gi|6322330|ref|NP_012404.1|	gi|19115689|ref|NP_594777.1|
gi|31196229|	lcl|At2g05480.1	lcl|C11G6.2 CE05256 WBGene	gi|24651893|	gi|6704678|ref|Os01g48250|	gi|6322358|ref|NP_012432.1|	gi|19115691|ref|NP_594779.1|
gi|31196237|	lcl|At2g05560.1	lcl|C11G6.4b CE37954 WBGen	gi|24651958|	gi|6704684|ref|Os01g48310|	gi|6322362|ref|NP_012436.1|	gi|19115702|ref|NP_594790.1|
gi|31196247|	lcl|At2g05570.1	lcl|C11H1.5 CE37315 WBGene	gi|24652131|	gi|6704702|ref|Os01g48480|	gi|6322371|ref|NP_012445.1|	gi|19115726|ref|NP_594814.1|
gi|31196255|	lcl|At2g05870.1	lcl|C11H1.9a CE05264 WBGen	gi|24652238|	gi|6704724|ref|Os01g48690|	gi|6322372|ref|NP_012446.1|	gi|19115730|ref|NP_594818.1|
gi|31196257|	lcl|At2g05880.1	lcl|C11H1.9b CE37316 WBGen	gi|24652254|	gi|6704750|ref|Os01g48880|	gi|6322375|ref|NP_012450.1|	gi|19115742|ref|NP_594830.1|
gi|31196259|	lcl|At2g05890.1	lcl|C12D5.10 CE35229 WBGen	gi|24652427|	gi|6704816|ref|Os01g49500|	gi|6322400|ref|NP_012474.1|	gi|19115746|ref|NP_594834.1|
gi|31196321|	lcl|At2g05970.1	lcl|C12D5.11 CE32585 WBGen	gi|24652587|	gi|6704868|ref|Os01g49970|	gi|6322404|ref|NP_012478.1|	gi|19115770|ref|NP_594858.1|
gi|31196343|	lcl|At2g06090.1	lcl|C12D5.3 CE06805 WBGene	gi|24652648|	gi|6704870|ref|Os01g49990|	gi|6322413|ref|NP_012487.1|	gi|19115777|ref|NP_594865.1|
gi|31196373|	lcl|At2g06120.1	lcl|C12D5.4 CE06806 WBGene	gi|24652727|	gi|6704931|ref|Os01g50550|	gi|6322414|ref|NP_012488.1|	gi|19115781|ref|NP_594869.1|
gi|31196501|	lcl|At2g06150.1	lcl|C12D5.5 CE06807 WBGene	gi|24652787|	gi|6704941|ref|Os01g50650|	gi|6322417|ref|NP_012491.1|	gi|19115784|ref|NP_594872.1|
gi|31196545|	lcl|At2g06180.1	lcl|C12D5.8b CE32587 WBGen	gi|24652806|	gi|6704942|ref|Os01g50660|	gi|6322419|ref|NP_012493.1|	gi|19115805|ref|NP_594893.1|
gi|31196615|	lcl|At2g06330.1	lcl|C12D5.9 CE06811 WBGene	gi|24652808|	gi|6704943|ref|Os01g50670|	gi|6322449|ref|NP_012523.1|	gi|19115824|ref|NP_594912.1|
gi|31196621|	lcl|At2g06430.1	lcl|C12D8.12 CE05276 WBGen	gi|24652810|	gi|6704958|ref|Os01g50790|	gi|6322454|ref|NP_012528.1|	gi|19115826|ref|NP_594914.1|
gi|31196713|	lcl|At2g06490.1	lcl|C12D8.4 CE05268 WBGene	gi|24652812|	gi|6704960|ref|Os01g50810|	gi|6322470|ref|NP_012544.1|	gi|19115827|ref|NP_594915.1|
gi|31196717|	lcl|At2g06610.1	lcl|C12D8.8 CE05271 WBGene	gi|24652814|	gi|6704976|ref|Os01g50970|	gi|6322486|ref|NP_012561.1|	gi|19115835|ref|NP_594923.1|
gi|31196721|	lcl|At2g06690.1	lcl|C13A10.2 CE08124 WBGen	gi|24653029|	gi|6704978|ref|Os01g50990|	gi|6322488|ref|NP_012563.1|	gi|19115838|ref|NP_594926.1|
gi|31196737|	lcl|At2g06720.1	lcl|C13A2.1 CE35231 WBGene	gi|24653345|	gi|6704989|ref|Os01g51100|	gi|6322502|ref|NP_012576.1|	gi|19115847|ref|NP_594935.1|
gi|31196803|	lcl|At2g06800.1	lcl|C13A2.10 CE08121 WBGen	gi|24653354|	gi|6705032|ref|Os01g51500|	gi|6322515|ref|NP_012589.1|	gi|19115880|ref|NP_594968.1|
gi|31196819|	lcl|At2g06845.1	lcl|C13A2.11 CE36783 WBGen	gi|24653540|	gi|6705065|ref|Os01g51830|	gi|6322522|ref|NP_012596.1|	gi|19115890|ref|NP_594978.1|
gi|31196831|	lcl|At2g06860.1	lcl|C13A2.2 CE08114 WBGene	gi|24653610|	gi|6705122|ref|Os01g52270|	gi|6322534|ref|NP_012608.1|	gi|19115902|ref|NP_594990.1|
gi|31196833|	lcl|At2g06965.1	lcl|C13A2.3 CE35232 WBGene	gi|24653618|	gi|6705123|ref|Os01g52280|	gi|6322544|ref|NP_012618.1|	gi|19115925|ref|NP_595013.1|
gi|31196837|	lcl|At2g06980.1	lcl|C13A2.4 CE08116 WBGene	gi|24653620|	gi|6705124|ref|Os01g52290|	gi|6322545|ref|NP_012619.1|	gi|19115941|ref|NP_595029.1|
gi|31196859|	lcl|At2g07120.1	lcl|C13A2.5 CE08117 WBGene	gi|24653622|	gi|6705126|ref|Os01g52310|	gi|6322547|ref|NP_012621.1|	gi|19115944|ref|NP_595032.1|

gi|31196901|	lcl|At2g07140.1	lcl|C13A2.6 CE08118 WBGene	gi|24653635|	gi|6705127|ref|Os01g52320|	gi|6322548|ref|NP_012622.1|	gi|19115947|ref|NP_595035.1|
gi|31196905|	lcl|At2g07200.1	lcl|C13A2.9 CE08120 WBGene	gi|24653714|	gi|6705131|ref|Os01g52360|	gi|6322558|ref|NP_012632.1|	gi|19115950|ref|NP_595038.1|
gi|31196957|	lcl|At2g07240.1	lcl|C13B7.1 CE21939 WBGene	gi|24653777|	gi|6705133|ref|Os01g52380|	gi|6322566|ref|NP_012640.1|	
gi|31197047|	lcl|At2g07260.1	lcl|C13B7.2 CE25775 WBGene	gi|24654144|	gi|6705174|ref|Os01g52730|	gi|6322567|ref|NP_012641.1|	
gi|31197115|	lcl|At2g07380.1	lcl|C13B7.3 CE24800 WBGene	gi|24654146|	gi|6705175|ref|Os01g52740|	gi|6322584|ref|NP_012658.1|	
gi|31197129|	lcl|At2g07390.1	lcl|C13B7.4 CE21942 WBGene	gi|24654162|	gi|6705194|ref|Os01g52930|	gi|6322589|ref|NP_012663.1|	
gi|31197163|	lcl|At2g07455.1	lcl|C13B7.5 CE20503 WBGene	gi|24654226|	gi|6705197|ref|Os01g52960|	gi|6322609|ref|NP_012683.1|	
gi|31197199|	lcl|At2g07630.1	lcl|C13B9.1 CE32795 WBGene	gi|24654228|	gi|6705198|ref|Os01g52970|	gi|6322610|ref|NP_012684.1|	
gi|31197419|	lcl|At2g07700.1	lcl|C13C4.7 CE34685 WBGene	gi|24654237|	gi|6705199|ref|Os01g52980|	gi|6322614|ref|NP_012688.1|	
gi|31197431|	lcl|At2g07770.1	lcl|C13D9.1 CE08134 WBGene	gi|24654261|	gi|6705217|ref|Os01g53160|	gi|6322621|ref|NP_012695.1|	
gi|31197453|	lcl|At2g09910.1	lcl|C13D9.2 CE08135 WBGene	gi|24654284|	gi|6705228|ref|Os01g53270|	gi|6322625|ref|NP_012698.1|	
gi|31197483|	lcl|At2g09960.1	lcl|C13D9.3 CE08136 WBGene	gi|24654313|	gi|6705250|ref|Os01g53480|	gi|6322627|ref|NP_012700.1|	
gi|31197485|	lcl|At2g10000.1	lcl|C13D9.4 CE08137 WBGene	gi|24654315|	gi|6705282|ref|Os01g53780|	gi|6322628|ref|NP_012701.1|	
gi|31197487|	lcl|At2g10060.1	lcl|C13D9.5 CE08138 WBGene	gi|24654374|	gi|6705328|ref|Os01g54150|	gi|6322629|ref|NP_012702.1|	
gi|31197489|	lcl|At2g10350.1	lcl|C13D9.6 CE08139 WBGene	gi|24654673|	gi|6705341|ref|Os01g54250|	gi|6322630|ref|NP_012703.1|	
gi|31197491|	lcl|At2g10370.1	lcl|C13F10.6 CE08147 WBGen	gi|24654703|	gi|6705344|ref|Os01g54280|	gi|6322632|ref|NP_012705.1|	
gi|31197493|	lcl|At2g10460.1	lcl|C13G3.2 CE05278 WBGene	gi|24654706|	gi|6705386|ref|Os01g54650|	gi|6322635|ref|NP_012708.1|	
gi|31197497|	lcl|At2g10465.1	lcl|C14A4.15 CE33031 WBGen	gi|24654740|	gi|6705389|ref|Os01g54680|	gi|6322641|ref|NP_012714.1|	
gi|31197501|	lcl|At2g10490.1	lcl|C14A4.8 CE02149 WBGene	gi|24654744|	gi|6705403|ref|Os01g54820|	gi|6322662|ref|NP_012735.1|	
gi|31197527|	lcl|At2g10500.1	lcl|C14A6.3 CE18495 WBGene	gi|24654749|	gi|6705406|ref|Os01g54850|	gi|6322663|ref|NP_012736.1|	
gi|31197529|	lcl|At2g10640.1	lcl|C14B1.3 CE00900 WBGene	gi|24654758|	gi|6705444|ref|Os01g55210|	gi|6322677|ref|NP_012750.1|	
gi|31197537|	lcl|At2g10840.1	lcl|C14B9.6a CE36784 WBGen	gi|24654782|	gi|6705466|ref|Os01g55410|	gi|6322684|ref|NP_012757.1|	
gi|31197703|	lcl|At2g11010.1	lcl|C14B9.6b CE29577 WBGen	gi|24654832|	gi|6705472|ref|Os01g55470|	gi|6322685|ref|NP_012758.1|	
gi|31197739|	lcl|At2g11110.1	lcl|C14B9.6c CE30603 WBGen	gi|24654848|	gi|6705490|ref|Os01g55630|	gi|6322690|ref|NP_012763.1|	
gi|31197741|	lcl|At2g11120.1	lcl|C14C10.4 CE05283 WBGen	gi|24654872|	gi|6705524|ref|Os01g55920|	gi|6322721|ref|NP_012794.1|	
gi|31197875|	lcl|At2g11150.1	lcl|C14C10.7 CE28433 WBGen	gi|24654876|	gi|6705525|ref|Os01g55930|	gi|6322737|ref|NP_012810.1|	
gi|31197973|	lcl|At2g11170.1	lcl|C14C11.4 CE27683 WBGen	gi|24654895|	gi|6705569|ref|Os01g56340|	gi|6322742|ref|NP_012815.1|	
gi|31198097|	lcl|At2g11200.1	lcl|C14C11.5 CE06824 WBGen	gi|24654901|	gi|6705578|ref|Os01g56410|	gi|6322754|ref|NP_012827.1|	
gi|31198099|	lcl|At2g11210.1	lcl|C14C6.1 CE36375 WBGene	gi|24654905|	gi|6705613|ref|Os01g56730|	gi|6322760|ref|NP_012833.1|	
gi|31198101|	lcl|At2g11345.1	lcl|C14C6.10 CE33600 WBGen	gi|24654908|	gi|6705614|ref|Os01g56740|	gi|6322776|ref|NP_012849.1|	
gi|31198103|	lcl|At2g11360.1	lcl|C14C6.12 CE35236 WBGen	gi|24654912|	gi|6705625|ref|Os01g56840|	gi|6322780|ref|NP_012853.1|	
gi|31198227|	lcl|At2g11480.1	lcl|C14C6.2 CE08157 WBGene	gi|24654916|	gi|6705628|ref|Os01g56870|	gi|6322781|ref|NP_012854.1|	
gi|31198253|	lcl|At2g11490.1	lcl|C14C6.3 CE35237 WBGene	gi|24655153|	gi|6705659|ref|Os01g57130|	gi|6322795|ref|NP_012868.1|	
gi|31198261|	lcl|At2g11560.1	lcl|C14C6.5 CE08160 WBGene	gi|24655338|	gi|6705688|ref|Os01g57400|	gi|6322803|ref|NP_012876.1|	
gi|311985|	lcl|At2g11650.1	lcl|C14C6.9 CE36376 WBGene	gi|24655540|	gi|6705727|ref|Os01g57760|	gi|6322808|ref|NP_012881.1|	
gi|31198747|	lcl|At2g11690.1	lcl|C14H10.4 CE17403 WBGen	gi|24655771|	gi|6705729|ref|Os01g57780|	gi|6322819|ref|NP_012892.1|	
gi|31198749|	lcl|At2g12060.1	lcl|C15A11.1 CE08169 WBGen	gi|24655776|	gi|6705732|ref|Os01g57810|	gi|6322841|ref|NP_012914.1|	
gi|31198767|	lcl|At2g12100.1	lcl|C15A11.5 CE08173 WBGen	gi|24655939|	gi|6705748|ref|Os01g57970|	gi|6322846|ref|NP_012919.1|	
gi|31198921|	lcl|At2g12140.1	lcl|C15A11.6 CE08174 WBGen	gi|24655956|	gi|6705756|ref|Os01g58050|	gi|6322896|ref|NP_012969.1|	
gi|31199165|	lcl|At2g12240.1	lcl|C15A7.1 CE03021 WBGene	gi|24655970|	gi|6705758|ref|Os01g58070|	gi|6322897|ref|NP_012970.1|	
gi|31199171|	lcl|At2g12300.1	lcl|C15C7.2 CE03988 WBGene	gi|24655973|	gi|6705763|ref|Os01g58100|	gi|6322913|ref|NP_012986.1|	
gi|31199195|	lcl|At2g12520.1	lcl|C15C7.4 CE03990 WBGene	gi|24655982|	gi|6705827|ref|Os01g58720|	gi|6322917|ref|NP_012990.1|	
gi|31199299|	lcl|At2g12870.1	lcl|C15C8.4 CE20506 WBGene	gi|24656040|	gi|6705859|ref|Os01g59040|	gi|6322931|ref|NP_013004.1|	
gi|31199527|	lcl|At2g12880.1	lcl|C15C8.5 CE05289 WBGene	gi|24656059|	gi|6705900|ref|Os01g59410|	gi|6322935|ref|NP_013008.1|	
gi|31199575|	lcl|At2g12980.1	lcl|C15F1.5 CE20510 WBGene	gi|24656168|	gi|6705915|ref|Os01g59540|	gi|6322949|ref|NP_013022.1|	
gi|31199589|	lcl|At2g13070.1	lcl|C15H11.1 CE08178 WBGen	gi|24656210|	gi|6705925|ref|Os01g59640|	gi|6322951|ref|NP_013024.1|	
gi|31199861|	lcl|At2g13130.1	lcl|C15H11.5 CE08182 WBGen	gi|24656242|	gi|6705977|ref|Os01g60090|	gi|6322955|ref|NP_013028.1|	
gi|31199937|	lcl|At2g13160.1	lcl|C15H7.3 CE00081 WBGene	gi|24656247|	gi|6705989|ref|Os01g60210|	gi|6322964|ref|NP_013036.1|	
gi|31200013|	lcl|At2g13310.1	lcl|C15H9.11 CE27686 WBGen	gi|24656292|	gi|6706011|ref|Os01g60400|	gi|6322968|ref|NP_013040.1|	
gi|31200051|	lcl|At2g13630.1	lcl|C15H9.2 CE35401 WBGene	gi|24656547|	gi|6706029|ref|Os01g60560|	gi|6322971|ref|NP_013043.1|	
gi|31200309|	lcl|At2g13640.1	lcl|C15H9.3 CE03995 WBGene	gi|24656553|	gi|6706034|ref|Os01g60610|	gi|6322976|ref|NP_013048.1|	
gi|31200437|	lcl|At2g13750.1	lcl|C16A11.7 CE34690 WBGen	gi|24656668|	gi|6706068|ref|Os01g60920|	gi|6322977|ref|NP_013049.1|	
gi|31200501|	lcl|At2g13870.1	lcl|C16A11.8 CE19358 WBGen	gi|24656733|	gi|6706076|ref|Os01g61000|	gi|6322982|ref|NP_013054.1|	
gi|31200513|	lcl|At2g13890.1	lcl|C16B8.4 CE30248 WBGene	gi|24656738|	gi|6706144|ref|Os01g61650|	gi|6323003|ref|NP_013075.1|	
gi|31200515|	lcl|At2g13900.1	lcl|C16C4.10 CE08206 WBGen	gi|24656741|	gi|6706150|ref|Os01g61700|	gi|6323020|ref|NP_013092.1|	
gi|31200553|	lcl|At2g13910.1	lcl|C16C4.11 CE08207 WBGen	gi|24656744|	gi|6706227|ref|Os01g62340|	gi|6323029|ref|NP_013101.1|	
gi|31200615|	lcl|At2g13920.1	lcl|C16C4.12 CE08208 WBGen	gi|24656764|	gi|6706274|ref|Os01g62720|	gi|6323035|ref|NP_013107.1|	
gi|31200621|	lcl|At2g13950.1	lcl|C16C4.13 CE36789 WBGen	gi|24656794|	gi|6706276|ref|Os01g62740|	gi|6323041|ref|NP_013113.1|	
gi|31200641|	lcl|At2g13980.1	lcl|C16C4.14 CE08210 WBGen	gi|24656799|	gi|6706309|ref|Os01g63040|	gi|6323042|ref|NP_013114.1|	
gi|31200649|	lcl|At2g14010.1	lcl|C16C4.15 CE08211 WBGen	gi|24656954|	gi|6706316|ref|Os01g63110|	gi|6323044|ref|NP_013116.1|	
gi|31200683|	lcl|At2g14070.1	lcl|C16C4.16 CE08212 WBGen	gi|24657070|	gi|6706318|ref|Os01g63130|	gi|6323061|ref|NP_013133.1|	
gi|31200767|	lcl|At2g14130.1	lcl|C16C4.2 CE32309 WBGene	gi|24657072|	gi|6706319|ref|Os01g63140|	gi|6323066|ref|NP_013138.1|	
gi|31200841|	lcl|At2g14140.1	lcl|C16C4.3 CE08199 WBGene	gi|24657351|	gi|6706337|ref|Os01g63300|	gi|6323075|ref|NP_013147.1|	
gi|31200949|	lcl|At2g14160.1	lcl|C16C4.4 CE34692 WBGene	gi|24657367|	gi|6706339|ref|Os01g63320|	gi|6323076|ref|NP_013148.1|	
gi|31201163|	lcl|At2g14230.1	lcl|C16C4.5 CE08201 WBGene	gi|24657504|	gi|6706340|ref|Os01g63330|	gi|6323078|ref|NP_013150.1|	
gi|31201189|	lcl|At2g14290.1	lcl|C16C4.6 CE08202 WBGene	gi|24657616|	gi|6706361|ref|Os01g63530|	gi|6323106|ref|NP_013178.1|	
gi|31201271|	lcl|At2g14330.1	lcl|C16C4.8 CE08204 WBGene	gi|24657624|	gi|6706372|ref|Os01g63640|	gi|6323109|ref|NP_013181.1|	
gi|31201395|	lcl|At2g14450.1	lcl|C16C4.9 CE08205 WBGene	gi|24657631|	gi|6706385|ref|Os01g63740|	gi|6323137|ref|NP_013209.1|	
gi|31201501|	lcl|At2g14500.1	lcl|C16C8.11 CE08222 WBGen	gi|24657638|	gi|6706413|ref|Os01g64010|	gi|6323150|ref|NP_013222.1|	
gi|31201529|	lcl|At2g14710.1	lcl|C16C8.13 CE08224 WBGen	gi|24657645|	gi|6706417|ref|Os01g64050|	gi|6323157|ref|NP_013229.1|	
gi|31201661|	lcl|At2g14770.1	lcl|C16C8.14 CE08225 WBGen	gi|24657727|	gi|6706427|ref|Os01g64150|	gi|6323171|ref|NP_013243.1|	
gi|31201665|	lcl|At2g14780.1	lcl|C16C8.16 CE08227 WBGen	gi|24657739|	gi|6706434|ref|Os01g64220|	gi|6323178|ref|NP_013250.1|	
gi|31201693|	lcl|At2g15060.1	lcl|C16C8.17 CE08228 WBGen	gi|24657747|	gi|6706463|ref|Os01g64510|	gi|6323181|ref|NP_013253.1|	
gi|31201819|	lcl|At2g15110.1	lcl|C16C8.4 CE08215 WBGene	gi|24657905|	gi|6706473|ref|Os01g64610|	gi|6323194|ref|NP_013266.1|	
gi|31201887|	lcl|At2g15140.1	lcl|C16C8.5 CE08216 WBGene	gi|24657939|	gi|6706582|ref|Os01g65510|	gi|6323207|ref|NP_013279.1|	
gi|31202117|	lcl|At2g15180.1	lcl|C16D2.1 CE15622 WBGene	gi|24657959|	gi|6706622|ref|Os01g65910|	gi|6323208|ref|NP_013280.1|	
gi|31202119|	lcl|At2g15190.1	lcl|C16D9.1 CE27695 WBGene	gi|24657966|	gi|6706639|ref|Os01g66080|	gi|6323216|ref|NP_013288.1|	

gi|31202145|	lcl|At2g15200.1	lcl|C16D9.4 CE06840 WBGene	gi|24657974|	gi|6706676|ref|Os01g66450|	gi|6323242|ref|NP_013314.1|
gi|31202237|	lcl|At2g15640.1	lcl|C16D9.5 CE35239 WBGene	gi|24657989|	gi|6706688|ref|Os01g66570|	gi|6323255|ref|NP_013327.1|
gi|31202241|	lcl|At2g15740.1	lcl|C16D9.7 CE37955 WBGene	gi|24658046|	gi|6706785|ref|Os01g67390|	gi|6323257|ref|NP_013329.1|
gi|31202245|	lcl|At2g15750.1	lcl|C17A2.2 CE08240 WBGene	gi|24658054|	gi|6706831|ref|Os01g67810|	gi|6323285|ref|NP_013357.1|
gi|31202261|	lcl|At2g16010.1	lcl|C17A2.4 CE08242 WBGene	gi|24658081|	gi|6706832|ref|Os01g67810|	gi|6323296|ref|NP_013368.1|
gi|31202277|	lcl|At2g16100.1	lcl|C17A2.5 CE08243 WBGene	gi|24658135|	gi|6706834|ref|Os01g67830|	gi|6323308|ref|NP_013380.1|
gi|31202283|	lcl|At2g16160.1	lcl|C17A2.6 CE08244 WBGene	gi|24658342|	gi|6706846|ref|Os01g67940|	gi|6323323|ref|NP_013395.1|
gi|31202285|	lcl|At2g16180.1	lcl|C17A2.7 CE08245 WBGene	gi|24658344|	gi|6706860|ref|Os01g68080|	gi|6323338|ref|NP_013410.1|
gi|31202299|	lcl|At2g16210.1	lcl|C17B7.1 CE16849 WBGene	gi|24658349|	gi|6706869|ref|Os01g68170|	gi|6323339|ref|NP_013411.1|
gi|31202303|	lcl|At2g16220.1	lcl|C17B7.10 CE16858 WBGen	gi|24658429|	gi|6706887|ref|Os01g68340|	gi|6323344|ref|NP_013416.1|
gi|31202429|	lcl|At2g16290.1	lcl|C17B7.11 CE16859 WBGen	gi|24658754|	gi|6706903|ref|Os01g68470|	gi|6323350|ref|NP_013422.1|
gi|31202443|	lcl|At2g16330.1	lcl|C17B7.12 CE35242 WBGen	gi|24659163|	gi|6706914|ref|Os01g68580|	gi|6323356|ref|NP_013428.1|
gi|31202511|	lcl|At2g16365.1	lcl|C17B7.2 CE16850 WBGene	gi|24659177|	gi|6706921|ref|Os01g68650|	gi|6323357|ref|NP_013429.1|
gi|31202715|	lcl|At2g16450.1	lcl|C17B7.3 CE16851 WBGene	gi|24659182|	gi|6706923|ref|Os01g68670|	gi|6323363|ref|NP_013436.1|
gi|31202723|	lcl|At2g16520.1	lcl|C17B7.4 CE16852 WBGene	gi|24659306|	gi|6706945|ref|Os01g68890|	gi|6323370|ref|NP_013441.1|
gi|31202725|	lcl|At2g16900.1	lcl|C17B7.5 CE16853 WBGene	gi|24659319|	gi|6706990|ref|Os01g69820|	gi|6323384|ref|NP_013456.1|
gi|31202739|	lcl|At2g17030.1	lcl|C17B7.7 CE16855 WBGene	gi|24659331|	gi|6707013|ref|Os01g70030|	gi|6323395|ref|NP_013467.1|
gi|31202741|	lcl|At2g17036.1	lcl|C17B7.8 CE16856 WBGene	gi|24659632|	gi|6707034|ref|Os01g70240|	gi|6323396|ref|NP_013468.1|
gi|31202869|	lcl|At2g17310.1	lcl|C17B7.9 CE16857 WBGene	gi|24659858|	gi|6707081|ref|Os01g70650|	gi|6323400|ref|NP_013472.1|
gi|31202999|	lcl|At2g17590.1	lcl|C17C3.10 CE04030 WBGen	gi|24659887|	gi|6707084|ref|Os01g70680|	gi|6323406|ref|NP_013479.1|
gi|31203011|	lcl|At2g17600.1	lcl|C17C3.11 CE34696 WBGen	gi|24660118|	gi|6707091|ref|Os01g70750|	gi|6323423|ref|NP_013495.1|
gi|31203045|	lcl|At2g17680.1	lcl|C17C3.13 CE03984 WBGen	gi|24660124|	gi|6707106|ref|Os01g70900|	gi|6323425|ref|NP_013497.1|
gi|31203049|	lcl|At2g17690.1	lcl|C17C3.15 CE27069 WBGen	gi|24660144|	gi|6707127|ref|Os01g71090|	gi|6323426|ref|NP_013498.1|
gi|31203087|	lcl|At2g17770.1	lcl|C17C3.18 CE35405 WBGen	gi|24660536|	gi|6707132|ref|Os01g71130|	gi|6323427|ref|NP_013499.1|
gi|31203403|	lcl|At2g17830.1	lcl|C17C3.19 CE35406 WBGen	gi|24660728|	gi|6707144|ref|Os01g71250|	gi|6323439|ref|NP_013511.1|
gi|31203407|	lcl|At2g18490.1	lcl|C17C3.2 CE03984 WBGene	gi|24660919|	gi|6707164|ref|Os01g71430|	gi|6323456|ref|NP_013528.1|
gi|31203453|	lcl|At2g18780.1	lcl|C17C3.20 CE35407 WBGen	gi|24661166|	gi|6707166|ref|Os01g71450|	gi|6323473|ref|NP_013545.1|
gi|31203629|	lcl|At2g18810.1	lcl|C17C3.4 CE04024 WBGene	gi|24661742|	gi|6707167|ref|Os01g71460|	gi|6323475|ref|NP_013547.1|
gi|31203785|	lcl|At2g19200.1	lcl|C17C3.5 CE04025 WBGene	gi|24661749|	gi|6707172|ref|Os01g71510|	gi|6323479|ref|NP_013551.1|
gi|31203909|	lcl|At2g19310.1	lcl|C17C3.6 CE04026 WBGene	gi|24661763|	gi|6707175|ref|Os01g71540|	gi|6323484|ref|NP_013556.1|
gi|31203917|	lcl|At2g19630.1	lcl|C17C3.7 CE04027 WBGene	gi|24661848|	gi|6707180|ref|Os01g71590|	gi|6323485|ref|NP_013557.1|
gi|31204009|	lcl|At2g19650.1	lcl|C17C3.8 CE04028 WBGene	gi|24661852|	gi|6707187|ref|Os01g71660|	gi|6323488|ref|NP_013560.1|
gi|31204093|	lcl|At2g19660.1	lcl|C17D12.3 CE17407 WBGen	gi|24661856|	gi|6707212|ref|Os01g71910|	gi|6323493|ref|NP_013565.1|
gi|31204433|	lcl|At2g19890.1	lcl|C17D12.5 CE30492 WBGen	gi|24661860|	gi|6707229|ref|Os01g72060|	gi|6323494|ref|NP_013566.1|
gi|31204437|	lcl|At2g20145.1	lcl|C17E7.1 CE08261 WBGene	gi|24661940|	gi|6707230|ref|Os01g72070|	gi|6323496|ref|NP_013569.1|
gi|31204439|	lcl|At2g20380.1	lcl|C17E7.10 CE35243 WBGen	gi|24662201|	gi|6707231|ref|Os01g72080|	gi|6323503|ref|NP_013574.1|
gi|31204441|	lcl|At2g20620.1	lcl|C17E7.11 CE25781 WBGen	gi|24662205|	gi|6707317|ref|Os01g72900|	gi|6323507|ref|NP_013578.1|
gi|31204549|	lcl|At2g20660.1	lcl|C17E7.2 CE08262 WBGene	gi|24662650|	gi|6707318|ref|Os01g72910|	gi|6323525|ref|NP_013596.1|
gi|31204573|	lcl|At2g20720.1	lcl|C17E7.3 CE35244 WBGene	gi|24662654|	gi|6707336|ref|Os01g73060|	gi|6323528|ref|NP_013599.1|
gi|31204577|	lcl|At2g20950.1	lcl|C17E7.7 CE17412 WBGene	gi|24662669|	gi|6707341|ref|Os01g73100|	gi|6323536|ref|NP_013607.1|
gi|31204579|	lcl|At2g20960.1	lcl|C17F4.1 CE08274 WBGene	gi|24662989|	gi|6707356|ref|Os01g73250|	gi|6323539|ref|NP_013610.1|
gi|31204633|	lcl|At2g21655.1	lcl|C17F4.10 CE37200 WBGen	gi|24663019|	gi|6707360|ref|Os01g73290|	gi|6323551|ref|NP_013622.1|
gi|31204649|	lcl|At2g21680.1	lcl|C17F4.4 CE35955 WBGene	gi|24663027|	gi|6707365|ref|Os01g73340|	gi|6323561|ref|NP_013632.1|
gi|31204729|	lcl|At2g21830.1	lcl|C17F4.5 CE27075 WBGene	gi|24663035|	gi|6707370|ref|Os01g73390|	gi|6323569|ref|NP_013640.1|
gi|31204763|	lcl|At2g21840.1	lcl|C17G10.5 CE06846 WBGen	gi|24663047|	gi|6707419|ref|Os01g73850|	gi|6323574|ref|NP_013645.1|
gi|31204835|	lcl|At2g21850.1	lcl|C17H1.1 CE15623 WBGene	gi|24663544|	gi|6707443|ref|Os01g74050|	gi|6323589|ref|NP_013660.1|
gi|31204837|	lcl|At2g21930.1	lcl|C17H1.8 CE15630 WBGene	gi|24663568|	gi|6707444|ref|Os01g74060|	gi|6323590|ref|NP_013661.1|
gi|31204839|	lcl|At2g22050.1	lcl|C17H11.1 CE29584 WBGen	gi|24663607|	gi|6707451|ref|Os01g74130|	gi|6323592|ref|NP_013663.1|
gi|31204885|	lcl|At2g22150.1	lcl|C17H12.11 CE16871 WBGe	gi|24663611|	gi|6707459|ref|Os01g74210|	gi|6323598|ref|NP_013669.1|
gi|31204911|	lcl|At2g22180.1	lcl|C17H12.12 CE16872 WBGe	gi|24663748|	gi|6707460|ref|Os01g74220|	gi|6323602|ref|NP_013673.1|
gi|31204975|	lcl|At2g22860.1	lcl|C17H12.13 CE37201 WBGe	gi|24663752|	gi|6707465|ref|Os01g74270|	gi|6323607|ref|NP_013679.1|
gi|31205015|	lcl|At2g23100.1	lcl|C17H12.5 CE16866 WBGen	gi|24663878|	gi|6707503|ref|Os01g74620|	gi|6323611|ref|NP_013682.1|
gi|31205021|	lcl|At2g23160.1	lcl|C17H12.6 CE16867 WBGen	gi|24663882|	gi|6707512|ref|Os01g74690|	gi|6323612|ref|NP_013683.1|
gi|31205023|	lcl|At2g23400.1	lcl|C17H12.8 CE19360 WBGen	gi|24664143|	gi|6800006|ref|Os02g01060|	gi|6323622|ref|NP_013693.1|
gi|31205057|	lcl|At2g23710.1	lcl|C18A11.4 CE04035 WBGen	gi|24664166|	gi|6800009|ref|Os02g01080|	gi|6323628|ref|NP_013699.1|
gi|31205261|	lcl|At2g24250.1	lcl|C18A11.5a CE35708 WBGe	gi|24664476|	gi|6800042|ref|Os02g01400|	gi|6323641|ref|NP_013712.1|
gi|31205263|	lcl|At2g24510.1	lcl|C18A11.5b CE35709 WBGe	gi|24664744|	gi|6800044|ref|Os02g01420|	gi|6323645|ref|NP_013716.1|
gi|31205551|	lcl|At2g24650.1	lcl|C18A11.5c CE24807 WBGe	gi|24664748|	gi|6800056|ref|Os02g01540|	gi|6323647|ref|NP_013718.1|
gi|31205687|	lcl|At2g24670.1	lcl|C18A3.5c CE27710 WBGen	gi|24664752|	gi|6800057|ref|Os02g01550|	gi|6323658|ref|NP_013729.1|
gi|31205695|	lcl|At2g24680.1	lcl|C18B10.1 CE17416 WBGen	gi|24664764|	gi|6800060|ref|Os02g01580|	gi|6323661|ref|NP_013732.1|
gi|31206229|	lcl|At2g24690.1	lcl|C18B10.2 CE08296 WBGen	gi|24664768|	gi|6800068|ref|Os02g01650|	gi|6323662|ref|NP_013733.1|
gi|31206233|	lcl|At2g24700.1	lcl|C18B10.3 CE35245 WBGen	gi|24664772|	gi|6800071|ref|Os02g01680|	gi|6323668|ref|NP_013739.1|
gi|31206269|	lcl|At2g24870.1	lcl|C18B10.4 CE08298 WBGen	gi|24664776|	gi|6800114|ref|Os02g02090|	gi|6323675|ref|NP_013746.1|
gi|31206317|	lcl|At2g24880.1	lcl|C18B10.5 CE29178 WBGen	gi|24664817|	gi|6800130|ref|Os02g02250|	gi|6323676|ref|NP_013747.1|
gi|31206335|	lcl|At2g24900.1	lcl|C18B10.6 CE08300 WBGen	gi|24665166|	gi|6800210|ref|Os02g02940|	gi|6323683|ref|NP_013754.1|
gi|31206499|	lcl|At2g24920.1	lcl|C18B10.7 CE25782 WBGen	gi|24665169|	gi|6800211|ref|Os02g02940|	gi|6323687|ref|NP_013758.1|
gi|31206605|	lcl|At2g24930.1	lcl|C18B10.8 CE23556 WBGen	gi|24665172|	gi|6800242|ref|Os02g03140|	gi|6323689|ref|NP_013760.1|
gi|31206607|	lcl|At2g25690.1	lcl|C18B12.5 CE08309 WBGen	gi|24665176|	gi|6800243|ref|Os02g03150|	gi|6323695|ref|NP_013766.1|
gi|31206661|	lcl|At2g26010.1	lcl|C18B2.1 CE04040 WBGene	gi|24665179|	gi|6800245|ref|Os02g03170|	gi|6323712|ref|NP_013783.1|
gi|31206773|	lcl|At2g26160.1	lcl|C18B2.2 CE29179 WBGene	gi|24665193|	gi|6800246|ref|Os02g03180|	gi|6323714|ref|NP_013785.1|
gi|31206859|	lcl|At2g26320.1	lcl|C18B2.5a CE04044 WBGen	gi|24665197|	gi|6800247|ref|Os02g03190|	gi|6323734|ref|NP_013805.1|
gi|31206861|	lcl|At2g26370.1	lcl|C18B2.5b CE30872 WBGen	gi|24665205|	gi|6800297|ref|Os02g03660|	gi|6323737|ref|NP_013808.1|
gi|31206963|	lcl|At2g26940.1	lcl|C18C4.1 CE36271 WBGene	gi|24665225|	gi|6800312|ref|Os02g03810|	gi|6323744|ref|NP_013815.1|
gi|31207065|	lcl|At2g27260.1	lcl|C18C4.9 CE29586 WBGene	gi|24665295|	gi|6800329|ref|Os02g03980|	gi|6323753|ref|NP_013824.1|
gi|31207235|	lcl|At2g27375.1	lcl|C18D4.2 CE15632 WBGene	gi|24665297|	gi|6800345|ref|Os02g04140|	gi|6323762|ref|NP_013833.1|
gi|31207431|	lcl|At2g27410.1	lcl|C18D4.4 CE15634 WBGene	gi|24665363|	gi|6800360|ref|Os02g04280|	gi|6323781|ref|NP_013852.1|
gi|31207479|	lcl|At2g27505.1	lcl|C18D4.5 CE37478 WBGene	gi|24665932|	gi|6800361|ref|Os02g04290|	gi|6323801|ref|NP_013872.1|

gi|31207485|	lcl|At2g27520.1	lcl|C18D4.8 CE31882 WBGene	gi|24666084|	gi|6800383|ref|Os02g04470|	gi|6323802|ref|NP_013873.1|
gi|31207539|	lcl|At2g27630.1	lcl|C18D4.9 CE18511 WBGene	gi|24666105|	gi|6800395|ref|Os02g04580|	gi|6323804|ref|NP_013875.1|
gi|31207587|	lcl|At2g27670.1	lcl|C18F10.4 CE33270 WBGen	gi|24666167|	gi|6800421|ref|Os02g04830|	gi|6323806|ref|NP_013877.1|
gi|31207623|	lcl|At2g27780.1	lcl|C18F10.5 CE33032 WBGen	gi|24666171|	gi|6800440|ref|Os02g05020|	gi|6323816|ref|NP_013887.1|
gi|31207747|	lcl|At2g28355.1	lcl|C18F10.6 CE02491 WBGen	gi|24666274|	gi|6800455|ref|Os02g05170|	gi|6323820|ref|NP_013891.1|
gi|31207789|	lcl|At2g28460.1	lcl|C18F10.8 CE06858 WBGen	gi|24666334|	gi|6800461|ref|Os02g05230|	gi|6323828|ref|NP_013899.1|
gi|31207997|	lcl|At2g29240.1	lcl|C18F10.9 CE30609 WBGen	gi|24666552|	gi|6800476|ref|Os02g05380|	gi|6323834|ref|NP_013905.1|
gi|31208175|	lcl|At2g29600.1	lcl|C18G1.5 CE17421 WBGene	gi|24666682|	gi|6800477|ref|Os02g05390|	gi|6323841|ref|NP_013912.1|
gi|31208265|	lcl|At2g29610.1	lcl|C18G1.6 CE17422 WBGene	gi|24666972|	gi|6800498|ref|Os02g05570|	gi|6323855|ref|NP_013926.1|
gi|31208267|	lcl|At2g29770.1	lcl|C18G1.7 CE17423 WBGene	gi|24666997|	gi|6800500|ref|Os02g05590|	gi|6323867|ref|NP_013938.1|
gi|31208269|	lcl|At2g29780.1	lcl|C18G1.8 CE17424 WBGene	gi|24667020|	gi|6800501|ref|Os02g05600|	gi|6323888|ref|NP_013959.1|
gi|31208273|	lcl|At2g29800.1	lcl|C18H2.1 CE29670 WBGene	gi|24667196|	gi|6800530|ref|Os02g05860|	gi|6323902|ref|NP_013972.1|
gi|31208275|	lcl|At2g29810.1	lcl|C18H2.2 CE32132 WBGene	gi|24667234|	gi|6800550|ref|Os02g06040|	gi|6323918|ref|NP_013989.1|
gi|31208385|	lcl|At2g29820.1	lcl|C18H2.3 CE30611 WBGene	gi|24667246|	gi|6800556|ref|Os02g06100|	gi|6323920|ref|NP_013991.1|
gi|31208387|	lcl|At2g29830.1	lcl|C18H2.4 CE00762 WBGene	gi|24667250|	gi|6800579|ref|Os02g06330|	gi|6323931|ref|NP_014002.1|
gi|31208391|	lcl|At2g29860.1	lcl|C18H2.5 CE30612 WBGene	gi|24667254|	gi|6800594|ref|Os02g06470|	gi|6323936|ref|NP_014007.1|
gi|31208437|	lcl|At2g29930.1	lcl|C18H7.3 CE17428 WBGene	gi|24667258|	gi|6800600|ref|Os02g06520|	gi|6323937|ref|NP_014008.1|
gi|31208495|	lcl|At2g30615.1	lcl|C18H7.6 CE17431 WBGene	gi|24667262|	gi|6800603|ref|Os02g06550|	gi|6323940|ref|NP_014011.1|
gi|31208497|	lcl|At2g30990.1	lcl|C18H7.8 CE17433 WBGene	gi|24667619|	gi|6800616|ref|Os02g06670|	gi|6323942|ref|NP_014013.1|
gi|31208499|	lcl|At2g31420.1	lcl|C18H7.9 CE17434 WBGene	gi|24668026|	gi|6800634|ref|Os02g06830|	gi|6323943|ref|NP_014014.1|
gi|31208611|	lcl|At2g31430.1	lcl|C18H9.3 CE01803 WBGene	gi|24668194|	gi|6800663|ref|Os02g07090|	gi|6323945|ref|NP_014016.1|
gi|31208683|	lcl|At2g31460.1	lcl|C23G10.5 CE04051 WBGen	gi|24668206|	gi|6800664|ref|Os02g07100|	gi|6323957|ref|NP_014028.1|
gi|31208713|	lcl|At2g31470.1	lcl|C23G10.8 CE25784 WBGen	gi|24668302|	gi|6800674|ref|Os02g07190|	gi|6323964|ref|NP_014035.1|
gi|31208717|	lcl|At2g31480.1	lcl|C23H5.1 CE17436 WBGene	gi|24668397|	gi|6800700|ref|Os02g07400|	gi|6323970|ref|NP_014041.1|
gi|31208843|	lcl|At2g31720.1	lcl|C23H5.2b CE17437 WBGen	gi|24668519|	gi|6800711|ref|Os02g07510|	gi|6323987|ref|NP_014058.1|
gi|31209017|	lcl|At2g32140.1	lcl|C23H5.9 CE17442 WBGene	gi|24668616|	gi|6800713|ref|Os02g07530|	gi|6323993|ref|NP_014063.1|
gi|31209089|	lcl|At2g32310.1	lcl|C24A3.1 CE04058 WBGene	gi|24668820|	gi|6800715|ref|Os02g07550|	gi|6323994|ref|NP_014064.1|
gi|31209139|	lcl|At2g32350.1	lcl|C24A8.3 CE27080 WBGene	gi|24668824|	gi|6800716|ref|Os02g07560|	gi|6324018|ref|NP_014088.1|
gi|31209147|	lcl|At2g32360.1	lcl|C24B5.1 CE36981 WBGene	gi|24762394|	gi|6800722|ref|Os02g07620|	gi|6324040|ref|NP_014110.1|
gi|31209149|	lcl|At2g32470.1	lcl|C24B5.5 CE20523 WBGene	gi|24762430|	gi|6800749|ref|Os02g07860|	gi|6324046|ref|NP_014116.1|
gi|31209247|	lcl|At2g32820.1	lcl|C24B9.1 CE25785 WBGene	gi|24762556|	gi|6800757|ref|Os02g07930|	gi|6324051|ref|NP_014121.1|
gi|31209325|	lcl|At2g32905.1	lcl|C24B9.10 CE33271 WBGen	gi|24762646|	gi|6800762|ref|Os02g07970|	gi|6324064|ref|NP_014134.1|
gi|31209327|	lcl|At2g33000.1	lcl|C24B9.11 CE33272 WBGen	gi|24762656|	gi|6800797|ref|Os02g08280|	gi|6324072|ref|NP_014142.1|
gi|31209501|	lcl|At2g33010.1	lcl|C24B9.12 CE33273 WBGen	gi|24762659|	gi|6800808|ref|Os02g08390|	gi|6324095|ref|NP_014165.1|
gi|31209697|	lcl|At2g33090.1	lcl|C24B9.14 CE33274 WBGen	gi|24762697|	gi|6800823|ref|Os02g08540|	gi|6324099|ref|NP_014169.1|
gi|31209725|	lcl|At2g33130.1	lcl|C24B9.15 CE33275 WBGen	gi|24762745|	gi|6800879|ref|Os02g09560|	gi|6324112|ref|NP_014182.1|
gi|31209873|	lcl|At2g33190.1	lcl|C24B9.16 CE36426 WBGen	gi|28571341|	gi|6800892|ref|Os02g09690|	gi|6324113|ref|NP_014183.1|
gi|31209895|	lcl|At2g33200.1	lcl|C24B9.2 CE37956 WBGene	gi|28571343|	gi|6800915|ref|Os02g09910|	gi|6324119|ref|NP_014189.1|
gi|31209935|	lcl|At2g33300.1	lcl|C24B9.4 CE17446 WBGene	gi|28571345|	gi|6800970|ref|Os02g10420|	gi|6324135|ref|NP_014205.1|
gi|31209945|	lcl|At2g33655.1	lcl|C24B9.5 CE35830 WBGene	gi|28571503|	gi|6800988|ref|Os02g10550|	gi|6324138|ref|NP_014208.1|
gi|31209961|	lcl|At2g33780.1	lcl|C24B9.6 CE17448 WBGene	gi|28571530|	gi|6800991|ref|Os02g10580|	gi|6324142|ref|NP_014212.1|
gi|31210029|	lcl|At2g33790.1	lcl|C24B9.7 CE33276 WBGene	gi|28571579|	gi|6800994|ref|Os02g10600|	gi|6324147|ref|NP_014217.1|
gi|31210221|	lcl|At2g34130.1	lcl|C24B9.8 CE17450 WBGene	gi|28571621|	gi|6801025|ref|Os02g10900|	gi|6324152|ref|NP_014222.1|
gi|31210229|	lcl|At2g34170.1	lcl|C24D10.1 CE34705 WBGen	gi|28571708|	gi|6801031|ref|Os02g10940|	gi|6324156|ref|NP_014226.1|
gi|31210285|	lcl|At2g34220.1	lcl|C24D10.6 CE16878 WBGen	gi|28571712|	gi|6801049|ref|Os02g11090|	gi|6324169|ref|NP_014239.1|
gi|31210403|	lcl|At2g34230.1	lcl|C24D10.7 CE16879 WBGen	gi|28571925|	gi|6801050|ref|Os02g11100|	gi|6324172|ref|NP_014242.1|
gi|31210449|	lcl|At2g34240.1	lcl|C24F3.5 CE19700 WBGene	gi|28572995|	gi|6801053|ref|Os02g11120|	gi|6324174|ref|NP_014244.1|
gi|31210465|	lcl|At2g34280.1	lcl|C24G6.7 CE17463 WBGene	gi|28573105|	gi|6801057|ref|Os02g11650|	gi|6324180|ref|NP_014250.1|
gi|31210627|	lcl|At2g34540.1	lcl|C24G7.2 CE37320 WBGene	gi|28573261|	gi|6801079|ref|Os02g11860|	gi|6324181|ref|NP_014251.1|
gi|31210731|	lcl|At2g34820.1	lcl|C24H10.1 CE04066 WBGen	gi|28573320|	gi|6801081|ref|Os02g11880|	gi|6324198|ref|NP_014268.1|
gi|31210813|	lcl|At2g35310.1	lcl|C24H11.4 CE18527 WBGen	gi|28573354|	gi|6801167|ref|Os02g12710|	gi|6324207|ref|NP_014277.1|
gi|31210817|	lcl|At2g35730.1	lcl|C24H12.1 CE08356 WBGen	gi|28573389|	gi|6801195|ref|Os02g12990|	gi|6324221|ref|NP_014291.1|
gi|31210915|	lcl|At2g36340.1	lcl|C24H12.11 CE08365 WBGe	gi|28573412|	gi|6801197|ref|Os02g13010|	gi|6324234|ref|NP_014304.1|
gi|31210979|	lcl|At2g36402.1	lcl|C24H12.9 CE34707 WBGen	gi|28573466|	gi|6801221|ref|Os02g13250|	gi|6324244|ref|NP_014314.1|
gi|31211021|	lcl|At2g36650.1	lcl|C25A1.10 CE08376 WBGen	gi|28573517|	gi|6801246|ref|Os02g13500|	gi|6324277|ref|NP_014347.1|
gi|31211063|	lcl|At2g37140.1	lcl|C25A1.9 CE18532 WBGene	gi|28573568|	gi|6801257|ref|Os02g13610|	gi|6324313|ref|NP_014383.1|
gi|31211095|	lcl|At2g37435.1	lcl|C25B8.5 CE35409 WBGene	gi|28573604|	gi|6801311|ref|Os02g14100|	gi|6324337|ref|NP_014407.1|
gi|31211099|	lcl|At2g37480.1	lcl|C25B8.7 CE30878 WBGene	gi|28573654|	gi|6801323|ref|Os02g14210|	gi|6324355|ref|NP_014425.1|
gi|31211177|	lcl|At2g37810.1	lcl|C25D7.1 CE08387 WBGene	gi|28573666|	gi|6801334|ref|Os02g14320|	gi|6324379|ref|NP_014449.1|
gi|31211189|	lcl|At2g37960.1	lcl|C25D7.12 CE08397 WBGen	gi|28573732|	gi|6801342|ref|Os02g14400|	gi|6324385|ref|NP_014455.1|
gi|31211191|	lcl|At2g38040.1	lcl|C25D7.15 CE34708 WBGen	gi|28573820|	gi|6801358|ref|Os02g14550|	gi|6324387|ref|NP_014457.1|
gi|31211193|	lcl|At2g38040.2	lcl|C25D7.2 CE08388 WBGene	gi|28574216|	gi|6801431|ref|Os02g15280|	gi|6324388|ref|NP_014458.1|
gi|31211197|	lcl|At2g38500.1	lcl|C25D7.4 CE08390 WBGene	gi|28574220|	gi|6801432|ref|Os02g15290|	gi|6324391|ref|NP_014461.1|
gi|31211213|	lcl|At2g38590.1	lcl|C25D7.5 CE08391 WBGene	gi|28574222|	gi|6801436|ref|Os02g15330|	gi|6324394|ref|NP_014464.1|
gi|31211231|	lcl|At2g38900.1	lcl|C25E10.10 CE06872 WBGe	gi|28574224|	gi|6801451|ref|Os02g15480|	gi|6324396|ref|NP_014466.1|
gi|31211321|	lcl|At2g39100.1	lcl|C25E10.7 CE06869 WBGen	gi|28574226|	gi|6801452|ref|Os02g15490|	gi|6324397|ref|NP_014467.1|
gi|31211343|	lcl|At2g39490.1	lcl|C25F9.1 CE31883 WBGene	gi|28574228|	gi|6801482|ref|Os02g15790|	gi|6324403|ref|NP_014473.1|
gi|31211567|	lcl|At2g39790.1	lcl|C25F9.2 CE17467 WBGene	gi|28574230|	gi|6801506|ref|Os02g16030|	gi|6324404|ref|NP_014474.1|
gi|31211601|	lcl|At2g39975.1	lcl|C25F9.7 CE17471 WBGene	gi|28574257|	gi|6801510|ref|Os02g16070|	gi|6324408|ref|NP_014477.1|
gi|31211605|	lcl|At2g40440.1	lcl|C25G4.1 CE05302 WBGene	gi|28574338|	gi|6801513|ref|Os02g16100|	gi|6324409|ref|NP_014478.1|
gi|31211761|	lcl|At2g40500.1	lcl|C25G4.11 CE28207 WBGen	gi|28574340|	gi|6801514|ref|Os02g16110|	gi|6324412|ref|NP_014481.1|
gi|31211899|	lcl|At2g40745.1	lcl|C25G4.3 CE05304 WBGene	gi|28574342|	gi|6801515|ref|Os02g16120|	gi|6324414|ref|NP_014483.1|
gi|31211947|	lcl|At2g40910.1	lcl|C25G4.6 CE05307 WBGene	gi|28574346|	gi|6801525|ref|Os02g16220|	gi|6324419|ref|NP_014488.1|
gi|31211951|	lcl|At2g40920.1	lcl|C25G4.7 CE05308 WBGene	gi|28574354|	gi|6801528|ref|Os02g16250|	gi|6324420|ref|NP_014489.1|
gi|31211963|	lcl|At2g40925.1	lcl|C25G4.8 CE05309 WBGene	gi|28574379|	gi|6801529|ref|Os02g16260|	gi|6324423|ref|NP_014492.1|
gi|31211973|	lcl|At2g41010.1	lcl|C25G6.2 CE04083 WBGene	gi|28574523|	gi|6801531|ref|Os02g16280|	gi|6324425|ref|NP_014494.1|
gi|31211981|	lcl|At2g41180.1	lcl|C25G6.3 CE04084 WBGene	gi|28574571|	gi|6801537|ref|Os02g16340|	gi|6324428|ref|NP_014497.1|

<table>
<tr><td>gi|31212005|</td><td>lcl|At2g41360.1</td><td>lcl|C25H3.6a CE27094 WBGen</td><td>gi|28574692|</td><td>gi|6801538|ref|Os02g16350|</td><td>gi|6324434|ref|NP_014503.1|</td></tr>
<tr><td>gi|31212035|</td><td>lcl|At2g41590.1</td><td>lcl|C25H3.6b CE31315 WBGen</td><td>gi|28574739|</td><td>gi|6801540|ref|Os02g16370|</td><td>gi|6324447|ref|NP_014516.1|</td></tr>
<tr><td>gi|31212081|</td><td>lcl|At2g41600.1</td><td>lcl|C26B2.8 CE04094 WBGene</td><td>gi|28574753|</td><td>gi|6801542|ref|Os02g16390|</td><td>gi|6324456|ref|NP_014525.1|</td></tr>
<tr><td>gi|31212241|</td><td>lcl|At2g41910.1</td><td>lcl|C26B9.6 CE35410 WBGene</td><td>gi|28574816|</td><td>gi|6801545|ref|Os02g16420|</td><td>gi|6324458|ref|NP_014527.1|</td></tr>
<tr><td>gi|31212321|</td><td>lcl|At2g42060.1</td><td>lcl|C26C6.4 CE05313 WBGene</td><td>gi|28574928|</td><td>gi|6801546|ref|Os02g16430|</td><td>gi|6324461|ref|NP_014530.1|</td></tr>
<tr><td>gi|31212341|</td><td>lcl|At2g43220.1</td><td>lcl|C26C6.7 CE05316 WBGene</td><td>gi|28574978|</td><td>gi|6801562|ref|Os02g16590|</td><td>gi|6324467|ref|NP_014536.1|</td></tr>
<tr><td>gi|31212355|</td><td>lcl|At2g43260.1</td><td>lcl|C26D10.5a CE03028 WBGe</td><td>gi|28575009|</td><td>gi|6801579|ref|Os02g16760|</td><td>gi|6324471|ref|NP_014540.1|</td></tr>
<tr><td>gi|31212377|</td><td>lcl|At2g43440.1</td><td>lcl|C26D10.5b CE30881 WBGe</td><td>gi|45549016|</td><td>gi|6801589|ref|Os02g16860|</td><td>gi|6324477|ref|NP_014546.1|</td></tr>
<tr><td>gi|31212379|</td><td>lcl|At2g43510.1</td><td>lcl|C26D10.5d CE32594 WBGe</td><td>gi|45549172|</td><td>gi|6801602|ref|Os02g16990|</td><td>gi|6324483|ref|NP_014552.1|</td></tr>
<tr><td>gi|31212385|</td><td>lcl|At2g43520.1</td><td>lcl|C26E1.3 CE31885 WBGene</td><td>gi|45549221|</td><td>gi|6801607|ref|Os02g17040|</td><td>gi|6324497|ref|NP_014566.1|</td></tr>
<tr><td>gi|31212389|</td><td>lcl|At2g43530.1</td><td>lcl|C26E6.1 CE01157 WBGene</td><td>gi|45549336|</td><td>gi|6801614|ref|Os02g17110|</td><td>gi|6324500|ref|NP_014569.1|</td></tr>
<tr><td>gi|31212541|</td><td>lcl|At2g43535.1</td><td>lcl|C26E6.2 CE32143 WBGene</td><td>gi|45550094|</td><td>gi|6801619|ref|Os02g17160|</td><td>gi|6324505|ref|NP_014574.1|</td></tr>
<tr><td>gi|31212557|</td><td>lcl|At2g43550.1</td><td>lcl|C26F1.5 CE06879 WBGene</td><td>gi|45550155|</td><td>gi|6801624|ref|Os02g17210|</td><td>gi|6324507|ref|NP_014576.1|</td></tr>
<tr><td>gi|31212595|</td><td>lcl|At2g43730.1</td><td>lcl|C26F1.7 CE06881 WBGene</td><td>gi|45550182|</td><td>gi|6801646|ref|Os02g17420|</td><td>gi|6324518|ref|NP_014587.1|</td></tr>
<tr><td>gi|31212613|</td><td>lcl|At2g43740.1</td><td>lcl|C27A2.2b CE29188 WBGen</td><td>gi|45550204|</td><td>gi|6801653|ref|Os02g17490|</td><td>gi|6324522|ref|NP_014591.1|</td></tr>
<tr><td>gi|31212651|</td><td>lcl|At2g43745.1</td><td>lcl|C27A7.2 CE31886 WBGene</td><td>gi|45550359|</td><td>gi|6801677|ref|Os02g17730|</td><td>gi|6324541|ref|NP_014610.1|</td></tr>
<tr><td>gi|31212661|</td><td>lcl|At2g44030.1</td><td>lcl|C27A7.7 CE08410 WBGene</td><td>gi|45550427|</td><td>gi|6801678|ref|Os02g17740|</td><td>gi|6324545|ref|NP_014614.1|</td></tr>
<tr><td>gi|31212727|</td><td>lcl|At2g44390.1</td><td>lcl|C27A7.8 CE34711 WBGene</td><td>gi|45550486|</td><td>gi|6801683|ref|Os02g17790|</td><td>gi|6324551|ref|NP_014620.1|</td></tr>
<tr><td>gi|31212791|</td><td>lcl|At2g44630.1</td><td>lcl|C27A7.9 CE34712 WBGene</td><td>gi|45550510|</td><td>gi|6801685|ref|Os02g17810|</td><td>gi|6324558|ref|NP_014627.1|</td></tr>
<tr><td>gi|31212841|</td><td>lcl|At2g44700.1</td><td>lcl|C27B7.5 CE03034 WBGene</td><td>gi|45550513|</td><td>gi|6801730|ref|Os02g18200|</td><td>gi|6324581|ref|NP_014650.1|</td></tr>
<tr><td>gi|31212909|</td><td>lcl|At2g45406.1</td><td>lcl|C27B7.7 CE03036 WBGene</td><td>gi|45550584|</td><td>gi|6801734|ref|Os02g18240|</td><td>gi|6324583|ref|NP_014652.1|</td></tr>
<tr><td>gi|31213099|</td><td>lcl|At2g45760.1</td><td>lcl|C27C12.3 CE20532 WBGen</td><td>gi|45550599|</td><td>gi|6801736|ref|Os02g18260|</td><td>gi|6324584|ref|NP_014653.1|</td></tr>
<tr><td>gi|31213273|</td><td>lcl|At2g45940.1</td><td>lcl|C27C12.5 CE31421 WBGen</td><td>gi|45550671|</td><td>gi|6801738|ref|Os02g18280|</td><td>gi|6324593|ref|NP_014662.1|</td></tr>
<tr><td>gi|31213297|</td><td>lcl|At2g46190.1</td><td>lcl|C27C7.5 CE24813 WBGene</td><td>gi|45550703|</td><td>gi|6801776|ref|Os02g18660|</td><td>gi|6324973|ref|NP_015041.1|</td></tr>
<tr><td>gi|31213317|</td><td>lcl|At2g46330.1</td><td>lcl|C27C7.7 CE18537 WBGene</td><td>gi|45550712|</td><td>gi|6801782|ref|Os02g18720|</td><td>gi|6324976|ref|NP_015044.1|</td></tr>
<tr><td>gi|31213329|</td><td>lcl|At2g46505.1</td><td>lcl|C27C7.8 CE18538 WBGene</td><td>gi|45550753|</td><td>gi|6801785|ref|Os02g18750|</td><td>gi|6324984|ref|NP_015052.1|</td></tr>
<tr><td>gi|31213331|</td><td>lcl|At2g46670.1</td><td>lcl|C27D6.10 CE06890 WBGen</td><td>gi|45550761|</td><td>gi|6801799|ref|Os02g18890|</td><td>gi|6324999|ref|NP_015067.1|</td></tr>
<tr><td>gi|31213333|</td><td>lcl|At2g47050.1</td><td>lcl|C27D6.6 CE06886 WBGene</td><td>gi|45550845|</td><td>gi|6801813|ref|Os02g19030|</td><td>gi|6325010|ref|NP_015078.1|</td></tr>
<tr><td>gi|31213373|</td><td>lcl|At2g47340.1</td><td>lcl|C27D6.7 CE06887 WBGene</td><td>gi|45550857|</td><td>gi|6801818|ref|Os02g19080|</td><td>gi|6325011|ref|NP_015079.1|</td></tr>
<tr><td>gi|31213483|</td><td>lcl|At2g47540.1</td><td>lcl|C27D6.8 CE06888 WBGene</td><td>gi|45550988|</td><td>gi|6801820|ref|Os02g19100|</td><td>gi|6325017|ref|NP_015085.1|</td></tr>
<tr><td>gi|31213590|</td><td>lcl|At3g01030.1</td><td>lcl|C27D6.9 CE06889 WBGene</td><td>gi|45551059|</td><td>gi|6801830|ref|Os02g19200|</td><td>gi|6325037|ref|NP_015105.1|</td></tr>
<tr><td>gi|31213692|</td><td>lcl|At3g01185.1</td><td>lcl|C27D8.2 CE08421 WBGene</td><td>gi|45551098|</td><td>gi|6801836|ref|Os02g19250|</td><td>gi|6325040|ref|NP_015108.1|</td></tr>
<tr><td>gi|31213762|</td><td>lcl|At3g03030.1</td><td>lcl|C27D8.4 CE30618 WBGene</td><td>gi|45551127|</td><td>gi|6801842|ref|Os02g19310|</td><td>gi|6325043|ref|NP_015111.1|</td></tr>
<tr><td>gi|31213989|</td><td>lcl|At3g03730.1</td><td>lcl|C27F2.1 CE26874 WBGene</td><td>gi|45551499|</td><td>gi|6801846|ref|Os02g19350|</td><td>gi|6325060|ref|NP_015128.1|</td></tr>
<tr><td>gi|31214073|</td><td>lcl|At3g03847.1</td><td>lcl|C27F2.6 CE01173 WBGene</td><td>gi|45551520|</td><td>gi|6801849|ref|Os02g19380|</td><td>gi|6325062|ref|NP_015130.1|</td></tr>
<tr><td>gi|31214079|</td><td>lcl|At3g04210.1</td><td>lcl|C27F2.7 CE29190 WBGene</td><td>gi|45551636|</td><td>gi|6801851|ref|Os02g19400|</td><td>gi|6325069|ref|NP_015137.1|</td></tr>
<tr><td>gi|31214205|</td><td>lcl|At3g04250.1</td><td>lcl|C27H5.5 CE06893 WBGene</td><td>gi|45551838|</td><td>gi|6801852|ref|Os02g19410|</td><td>gi|6325084|ref|NP_015152.1|</td></tr>
<tr><td>gi|31214857|</td><td>lcl|At3g04320.1</td><td>lcl|C27H5.6 CE34715 WBGene</td><td>gi|45551915|</td><td>gi|6801858|ref|Os02g19470|</td><td>gi|6325089|ref|NP_015157.1|</td></tr>
<tr><td>gi|31215150|</td><td>lcl|At3g04330.1</td><td>lcl|C28A5.1 CE00675 WBGene</td><td>gi|45552281|</td><td>gi|6801859|ref|Os02g19480|</td><td>gi|6325118|ref|NP_015186.1|</td></tr>
<tr><td>gi|31215392|</td><td>lcl|At3g04540.1</td><td>lcl|C28A5.2 CE00676 WBGene</td><td>gi|45552317|</td><td>gi|6801887|ref|Os02g19730|</td><td>gi|6325121|ref|NP_015188.1|</td></tr>
<tr><td>gi|31215921|</td><td>lcl|At3g04660.1</td><td>lcl|C28A5.6 CE00673 WBGene</td><td>gi|45552367|</td><td>gi|6801922|ref|Os02g20070|</td><td>gi|6325124|ref|NP_015192.1|</td></tr>
<tr><td>gi|31215934|</td><td>lcl|At3g04890.1</td><td>lcl|C28C12.13 CE29485 WBGe</td><td>gi|45552391|</td><td>gi|6801927|ref|Os02g20120|</td><td>gi|6325130|ref|NP_015198.1|</td></tr>
<tr><td>gi|31215937|</td><td>lcl|At3g05440.1</td><td>lcl|C28C12.3 CE04110 WBGen</td><td>gi|45552425|</td><td>gi|6801932|ref|Os02g20170|</td><td>gi|6325134|ref|NP_015202.1|</td></tr>
<tr><td>gi|31216184|</td><td>lcl|At3g05450.1</td><td>lcl|C28C12.4 CE04111 WBGen</td><td>gi|45552427|</td><td>gi|6801934|ref|Os02g20190|</td><td>gi|6325139|ref|NP_015207.1|</td></tr>
<tr><td>gi|31216413|</td><td>lcl|At3g05685.1</td><td>lcl|C28C12.5 CE04112 WBGen</td><td>gi|45552481|</td><td>gi|6801941|ref|Os02g20260|</td><td>gi|6325152|ref|NP_015220.1|</td></tr>
<tr><td>gi|31216535|</td><td>lcl|At3g06160.1</td><td>lcl|C28C12.7a CE29486 WBGe</td><td>gi|45552483|</td><td>gi|6801983|ref|Os02g20650|</td><td>gi|6325155|ref|NP_015224.1|</td></tr>
<tr><td>gi|31216698|</td><td>lcl|At3g06220.1</td><td>lcl|C28C12.7b CE31323 WBGe</td><td>gi|45552485|</td><td>gi|6802034|ref|Os02g21130|</td><td>gi|6325182|ref|NP_015250.1|</td></tr>
<tr><td>gi|31216793|</td><td>lcl|At3g06240.1</td><td>lcl|C28C12.8 CE04115 WBGen</td><td>gi|45552487|</td><td>gi|6802036|ref|Os02g21150|</td><td>gi|6325184|ref|NP_015253.1|</td></tr>
<tr><td>gi|31216875|</td><td>lcl|At3g06520.1</td><td>lcl|C28D4.10 CE34716 WBGen</td><td>gi|45552537|</td><td>gi|6802037|ref|Os02g21160|</td><td>gi|6325187|ref|NP_015255.1|</td></tr>
<tr><td>gi|31216938|</td><td>lcl|At3g06570.1</td><td>lcl|C28D4.4 CE25787 WBGene</td><td>gi|45552551|</td><td>gi|6802044|ref|Os02g21230|</td><td>gi|6325224|ref|NP_015292.1|</td></tr>
<tr><td>gi|31216950|</td><td>lcl|At3g06990.1</td><td>lcl|C28D4.5 CE25788 WBGene</td><td>gi|45552575|</td><td>gi|6802081|ref|Os02g21600|</td><td>gi|6325225|ref|NP_015293.1|</td></tr>
<tr><td>gi|31216985|</td><td>lcl|At3g07000.1</td><td>lcl|C28D4.7 CE25789 WBGene</td><td>gi|45552597|</td><td>gi|6802094|ref|Os02g21720|</td><td>gi|6325236|ref|NP_015304.1|</td></tr>
<tr><td>gi|31216995|</td><td>lcl|At3g07450.1</td><td>lcl|C28D4.8 CE25790 WBGene</td><td>gi|45552599|</td><td>gi|6802101|ref|Os02g21790|</td><td>gi|6325241|ref|NP_015309.1|</td></tr>
<tr><td>gi|31217055|</td><td>lcl|At3g07870.1</td><td>lcl|C28F5.2 CE01816 WBGene</td><td>gi|45552633|</td><td>gi|6802123|ref|Os02g22000|</td><td>gi|6325246|ref|NP_015314.1|</td></tr>
<tr><td>gi|31217143|</td><td>lcl|At3g08750.1</td><td>lcl|C28G1.2 CE33520 WBGene</td><td>gi|45552643|</td><td>gi|6802126|ref|Os02g22030|</td><td>gi|6325250|ref|NP_015318.1|</td></tr>
<tr><td>gi|31217519|</td><td>lcl|At3g08810.1</td><td>lcl|C28G1.4 CE04121 WBGene</td><td>gi|45552657|</td><td>gi|6802128|ref|Os02g22050|</td><td>gi|6325252|ref|NP_015320.1|</td></tr>
<tr><td>gi|31217639|</td><td>lcl|At3g09035.1</td><td>lcl|C28G1.5 CE37204 WBGene</td><td>gi|45552755|</td><td>gi|6802157|ref|Os02g22300|</td><td>gi|6325258|ref|NP_015326.1|</td></tr>
<tr><td>gi|31217670|</td><td>lcl|At3g09160.1</td><td>lcl|C28G1.6 CE30885 WBGene</td><td>gi|45552757|</td><td>gi|6802167|ref|Os02g22390|</td><td>gi|6325264|ref|NP_015332.1|</td></tr>
<tr><td>gi|31217876|</td><td>lcl|At3g09170.1</td><td>lcl|C28H8.5 CE01827 WBGene</td><td>gi|45552785|</td><td>gi|6802170|ref|Os02g22420|</td><td>gi|6325274|ref|NP_015342.1|</td></tr>
<tr><td>gi|31217958|</td><td>lcl|At3g09390.1</td><td>lcl|C28H8.8 CE29196 WBGene</td><td>gi|45553019|</td><td>gi|6802171|ref|Os02g22430|</td><td>gi|6325275|ref|NP_015343.1|</td></tr>
<tr><td>gi|31218063|</td><td>lcl|At3g09870.1</td><td>lcl|C29E4.5 CE29682 WBGene</td><td>gi|45553083|</td><td>gi|6802181|ref|Os02g22530|</td><td>gi|6325277|ref|NP_015345.1|</td></tr>
<tr><td>gi|31218692|</td><td>lcl|At3g10100.1</td><td>lcl|C29E6.1a CE05332 WBGen</td><td>gi|45553089|</td><td>gi|6802183|ref|Os02g22550|</td><td>gi|6325312|ref|NP_015380.1|</td></tr>
<tr><td>gi|31219562|</td><td>lcl|At3g10240.1</td><td>lcl|C29E6.1b CE37323 WBGen</td><td>gi|45553125|</td><td>gi|6802184|ref|Os02g22560|</td><td>gi|6325318|ref|NP_015386.1|</td></tr>
<tr><td>gi|31221170|</td><td>lcl|At3g10430.1</td><td>lcl|C29E6.3 CE17478 WBGene</td><td>gi|45553135|</td><td>gi|6802194|ref|Os02g22660|</td><td>gi|6325322|ref|NP_015390.1|</td></tr>
<tr><td>gi|31221504|</td><td>lcl|At3g10440.1</td><td>lcl|C29E6.4 CE05335 WBGene</td><td>gi|45553487|</td><td>gi|6802202|ref|Os02g22740|</td><td>gi|6325324|ref|NP_015392.1|</td></tr>
<tr><td>gi|31222334|</td><td>lcl|At3g10460.1</td><td>lcl|C29F3.4 CE34525 WBGene</td><td>gi|45553489|</td><td>gi|6802204|ref|Os02g22760|</td><td>gi|6325341|ref|NP_015409.1|</td></tr>
<tr><td>gi|31222471|</td><td>lcl|At3g10510.1</td><td>lcl|C29F3.5 CE08439 WBGene</td><td>gi|45553780|</td><td>gi|6802209|ref|Os02g22810|</td><td>gi|6325372|ref|NP_015440.1|</td></tr>
<tr><td>gi|31222481|</td><td>lcl|At3g10595.1</td><td>lcl|C29F3.6 CE34526 WBGene</td><td>gi|45553953|</td><td>gi|6802227|ref|Os02g23980|</td><td>gi|6325405|ref|NP_015473.1|</td></tr>
<tr><td>gi|31222489|</td><td>lcl|At3g10750.1</td><td>lcl|C29F3.7 CE31888 WBGene</td><td>gi|45554104|</td><td>gi|6802228|ref|Os02g23990|</td><td>gi|6325410|ref|NP_015478.1|</td></tr>
<tr><td>gi|31222496|</td><td>lcl|At3g10790.1</td><td>lcl|C29F4.1 CE03038 WBGene</td><td>gi|45554348|</td><td>gi|6802229|ref|Os02g24000|</td><td>gi|6325421|ref|NP_015489.1|</td></tr>
<tr><td>gi|31222503|</td><td>lcl|At3g10845.1</td><td>lcl|C29F5.7 CE02514 WBGene</td><td>gi|45554360|</td><td>gi|6802236|ref|Os02g24070|</td><td>gi|6325427|ref|NP_015495.1|</td></tr>
<tr><td>gi|31222512|</td><td>lcl|At3g11370.1</td><td>lcl|C29F7.1 CE08442 WBGene</td><td>gi|45554381|</td><td>gi|6802260|ref|Os02g24300|</td><td>gi|6325443|ref|NP_015511.1|</td></tr>
<tr><td>gi|31222536|</td><td>lcl|At3g11390.1</td><td>lcl|C29F7.2 CE35414 WBGene</td><td>gi|45554508|</td><td>gi|6802261|ref|Os02g24310|</td><td>gi|6325446|ref|NP_015514.1|</td></tr>
<tr><td>gi|31222545|</td><td>lcl|At3g11770.1</td><td>lcl|C29F9.10 CE08458 WBGen</td><td>gi|45555264|</td><td>gi|6802272|ref|Os02g24420|</td><td>gi|6325451|ref|NP_015519.1|</td></tr>
<tr><td>gi|31222597|</td><td>lcl|At3g11990.1</td><td>lcl|C29F9.11 CE08459 WBGene00016225 status:Predicted SW:Q9TZC0 protein_id:AAC68752.1 /QuerySize=305</td><td></td><td>gi|6802278|ref|Os02g24480|</td><td>gi|6325454|ref|NP_015522.1|</td></tr>
<tr><td>gi|31223290|</td><td>lcl|At3g12450.1</td><td>lcl|C29F9.13 CE08461 WBGene00016227 status:Partially_confirmed SW:Q9TZB4 protein_id:AAC68755.1 /QuerySize=143</td><td></td><td>gi|6802283|ref|Os02g24530|</td><td>gi|6325458|ref|NP_015526.1|</td></tr>
<tr><td>gi|31223720|</td><td>lcl|At3g12775.1</td><td>lcl|C29G2.4 CE37876 WBGene00016232 locus:srt-4 status:Predicted /QuerySize=354</td><td></td><td>gi|6802310|ref|Os02g24800|</td><td>gi|6325462|ref|NP_015530.1|</td></tr>
</table>

gi|31223954|	lcl|At3g12850.1	lcl|C29H12.5 CE02518 WBGene00016236 status:Predicted SW:Q18313 protein_id:AAC46697.1 /QuerySize=706	gi|6802318|ref|Os02g24880|	gi|6381999|ref|NP_009311.2|
gi|31224095|	lcl|At3g12880.1	lcl|C30A5.10a CE29202 WBGene00016242 status:Partially_confirmed SW:Q95QT0 protein_id:AAL02451.1 /QuerySize=298	gi|6802328|ref|Os02g24980|	gi|6862572|ref|NP_036195.1|
gi|31224291|	lcl|At3g13140.1	lcl|C30A5.10b CE29203 WBGene00016242 status:Confirmed SW:Q95QS9 protein_id:AAL02452.1 /QuerySize=231	gi|6802330|ref|Os02g25000|	gi|7839146|ref|NP_058136.1|
gi|31225472|	lcl|At3g13260.1	lcl|C30A5.2 CE00094 WBGene00004342 locus:rfs-1 status:Partially_confirmed SW:P34348 protein_id:AAB59172.1 /QuerySize	gi|6802337|ref|Os02g25070|	gi|7839149|ref|NP_058139.1|
gi|31226189|	lcl|At3g13280.1	lcl|C30A5.5 CE00096 WBGene00004899 locus:snb-5 status:Partially_confirmed SW:P34351 protein_id:AAB59178.1 /QuerySiz	gi|6802341|ref|Os02g25110|	gi|7839151|ref|NP_058141.1|
gi|31226193|	lcl|At3g13480.1	lcl|C30B5.5 CE02524 WBGene00016246 status:Partially_confirmed SW:Q18321 protein_id:AAK31469.1 /QuerySize=790	gi|6802355|ref|Os02g25250|	gi|7839153|ref|NP_058143.1|
gi|31226451|	lcl|At3g13590.1	lcl|C30B5.7 CE02526 WBGene00016248 status:Partially_confirmed SW:Q18323 protein_id:AAK31471.1 /QuerySize=350	gi|6802361|ref|Os02g25310|	gi|7839157|ref|NP_058147.1|
gi|31226868|	lcl|At3g13680.1	lcl|C30E1.5 CE08472 WBGene00016254 status:Partially_confirmed SW:O17333 protein_id:AAB71250.1 /QuerySize=319	gi|6802362|ref|Os02g25320|	gi|7839159|ref|NP_058149.1|
gi|31226931|	lcl|At3g13760.1	lcl|C30E1.8 CE08475 WBGene00016257 status:Predicted SW:O17332 protein_id:AAB71253.1 /QuerySize=424	gi|6802365|ref|Os02g25350|	gi|7839161|ref|NP_058151.1|
gi|31227094|	lcl|At3g13820.1	lcl|C30F2.1 CE17480 WBGene00000760 locus:col-187 collagen status:Partially_confirmed SW:Q18327 protein_id:CAA94580.1	gi|6802367|ref|Os02g25370|	gi|7839163|ref|NP_058153.1|
gi|31227163|	lcl|At3g13830.1	lcl|C30F2.2 CE31422 WBGene00007816 Zinc finger	gi|6802369|ref|Os02g25390|	gi|7839167|ref|NP_058155.1|
gi|31227169|	lcl|At3g14220.1	lcl|C30G12.1 CE29685 WBGene00016273 status:Predicted SW:Q09255 protein_id:AAL16307.1 /QuerySize=469	gi|6802374|ref|Os02g25440|	gi|7839168|ref|NP_058156.1|
gi|31227358|	lcl|At3g14500.1	lcl|C30G12.4 CE37485 WBGene00016276 status:Partially_confirmed SW:Q09257 protein_id:AAC46673.2 /QuerySize=258	gi|6802382|ref|Os02g25520|	gi|7839169|ref|NP_058157.1|
gi|31227554|	lcl|At3g14710.1	lcl|C30G12.6a CE29205 WBGene00016277 status:Partially_confirmed SW:Q09259 protein_id:AAL02454.1 /QuerySize=1100	gi|6802386|ref|Os02g25560|	gi|7839170|ref|NP_058159.1|
gi|31227579|	lcl|At3g15530.1	lcl|C30G12.6b CE29206 WBGene00016277 status:Partially_confirmed SW:Q09259 protein_id:AAL02455.1 /QuerySize=1097	gi|6802403|ref|Os02g25730|	gi|7839172|ref|NP_058161.1|
gi|31227590|	lcl|At3g15530.2	lcl|C30G4.2 CE06898 WBGene00016268 status:Partially_confirmed SW:Q18330 protein_id:AAA93420.1 /QuerySize=192	gi|6802410|ref|Os02g25800|	gi|7839174|ref|NP_058162.1|
gi|31227718|	lcl|At3g15600.1	lcl|C30G4.6 CE06902 WBGene00016271 status:Predicted SW:Q18335 protein_id:AAA93425.1 /QuerySize=353	gi|6802435|ref|Os02g26050|	gi|7839175|ref|NP_058164.1|
gi|31227725|	lcl|At3g15690.2	lcl|C30H6.3 CE31161 WBGene00007821 status:Confirmed SW:O45274 protein_id:CAB02807.2 /QuerySize=208	gi|6802440|ref|Os02g26100|	gi|7839177|ref|NP_058166.1|
gi|31227846|	lcl|At3g15960.1	lcl|C30H6.4 CE15648 WBGene00007822 Lectin C-type domain short and long forms status:Predicted SW:O45275 protein_id:C	gi|6802457|ref|Os02g26270|	gi|7839184|ref|NP_058170.1|
gi|31227853|	lcl|At3g16210.1	lcl|C30H6.5 CE15649 WBGene00007823 status:Predicted SW:O45277 protein_id:CAB02811.1 /QuerySize=501	gi|6802470|ref|Os02g26400|	gi|7839186|ref|NP_058172.1|
gi|31227909|	lcl|At3g16555.1	lcl|C31A11.3 CE15654 WBGene00007831 status:Predicted SW:O45280 protein_id:CAB05685.1 /QuerySize=405	gi|6802514|ref|Os02g26760|	gi|7839188|ref|NP_058173.1|
gi|31228103|	lcl|At3g16580.1	lcl|C31A11.4 CE15655 WBGene00007832 status:Predicted SW:O45281 protein_id:CAB05686.1 /QuerySize=282	gi|6802518|ref|Os02g26800|	gi|7839189|ref|NP_058175.1|
gi|31228107|	lcl|At3g16590.1	lcl|C31A11.6 CE15657 WBGene00007834 status:Partially_confirmed SW:O62081 protein_id:CAB05688.1 /QuerySize=330	gi|6802520|ref|Os02g26820|	gi|7839191|ref|NP_058176.1|
gi|31228462|	lcl|At3g16740.1	lcl|C31A11.9 CE15659 WBGene00006247 locus:str-214 status:Predicted SW:O45284 protein_id:CAB05691.1 /QuerySize=352	gi|6802531|ref|Os02g26930|	gi|7839193|ref|NP_058179.1|
gi|31228476|	lcl|At3g16820.1	lcl|C31B8.1 CE35250 WBGene00016279 status:Partially_confirmed SW:O16908 protein_id:AAG23974.2 /QuerySize=186	gi|6802533|ref|Os02g26950|	gi|7839195|ref|NP_058180.1|
gi|31228782|	lcl|At3g16880.1	lcl|C31B8.11 CE26694 WBGene00005261 locus:srh-38 7TM chemoreceptor	gi|6802563|ref|Os02g27240|	gi|7839196|ref|NP_058182.1|
gi|31228950|	lcl|At3g16970.1	lcl|C31B8.12 CE35251 WBGene00016285 status:Partially_confirmed SW:O16907 protein_id:AAG23971.2 /QuerySize=338	gi|6802566|ref|Os02g27270|	gi|7839204|ref|NP_058190.1|
gi|31228976|	lcl|At3g17010.1	lcl|C31B8.13 CE08493 WBGene00005453 locus:srh-247 7TM chemoreceptor	gi|6802567|ref|Os02g27280|	gi|7839206|ref|NP_058192.1|
gi|31229057|	lcl|At3g17080.1	lcl|C31B8.2 CE35252 WBGene00016280 status:Partially_confirmed SW:O16904 protein_id:AAG23973.2 /QuerySize=226	gi|6802581|ref|Os02g27400|	gi|7839208|ref|NP_058194.1|
gi|31229419|	lcl|At3g17130.1	lcl|C31B8.6 CE08486 WBGene00006111 locus:str-46 7TM chemoreceptor	gi|6802583|ref|Os02g27430|	gi|7839210|ref|NP_058196.1|
gi|31229655|	lcl|At3g17150.1	lcl|C31B8.7 CE08487 WBGene00016282 status:Partially_confirmed SW:O16900 protein_id:AAG23977.1 /QuerySize=474	gi|6802609|ref|Os02g27630|	gi|9755328|ref|NP_010092.2|
gi|31229826|	lcl|At3g17220.1	lcl|C31B8.9 CE36796 WBGene00016284 status:Predicted SW:O16902 protein_id:AAG23972.2 /QuerySize=241	gi|6802611|ref|Os02g27650|	gi|9755333|ref|NP_011482.2|
gi|31230122|	lcl|At3g17225.1	lcl|C31C9.1a CE32597 WBGene00006404 locus:tag-10 apical gut membrane protein status:Confirmed SW:O01986 protein_id:	gi|6802615|ref|Os02g27690|	gi|9755338|ref|NP_013312.2|
gi|31230457|	lcl|At3g17230.1	lcl|C31C9.1b CE32598 WBGene00006404 locus:tag-10 status:Confirmed SW:Q8I0D2 protein_id:CAD57698.1 /QuerySize=455	gi|6802617|ref|Os02g27710|	
gi|31230591|	lcl|At3g17265.1	lcl|C31C9.3 CE08498 WBGene00007837 status:Partially_confirmed SW:O45285 protein_id:CAB05695.1 /QuerySize=377	gi|6802629|ref|Os02g27830|	
gi|31230679|	lcl|At3g17270.1	lcl|C31C9.4 CE37207 WBGene00007838 status:Predicted SW:O45286 protein_id:CAB05696.4 /QuerySize=592	gi|6802630|ref|Os02g27840|	
gi|31230911|	lcl|At3g17280.1	lcl|C31C9.6 CE32599 WBGene00007840 status:Partially_confirmed SW:Q8I4M6 protein_id:CAD57687.1 /QuerySize=965	gi|6802653|ref|Os02g28070|	
gi|31231006|	lcl|At3g17320.1	lcl|C31E10.1 CE08501 WBGene00007842 status:Predicted SW:Q93310 protein_id:CAB01727.1 /QuerySize=350	gi|6802661|ref|Os02g28150|	
gi|31231045|	lcl|At3g17400.1	lcl|C31E10.3 CE28540 WBGene00007844 status:Predicted SW:Q93312 protein_id:CAB01729.2 /QuerySize=410	gi|6802666|ref|Os02g28200|	
gi|31231146|	lcl|At3g17480.1	lcl|C31E10.4 CE08504 WBGene00007845 status:Partially_confirmed SW:Q93313 protein_id:CAB01730.1 /QuerySize=177	gi|6802671|ref|Os02g28250|	
gi|31231153|	lcl|At3g17490.1	lcl|C31G12.2 CE15661 WBGene00007851 Lectin C-type domain short and long forms status:Partially_confirmed SW:O17630 p	gi|6802678|ref|Os02g28320|	
gi|31231159|	lcl|At3g17500.1	lcl|C31H1.2 CE34722 WBGene00016287 status:Partially_confirmed SW:Q18337 protein_id:AAA83606.2 /QuerySize=345	gi|6802697|ref|Os02g28510|	
gi|31231285|	lcl|At3g17530.1	lcl|C31H1.5 CE34723 WBGene00016288 status:Partially_confirmed SW:Q18339 protein_id:AAA83608.2 /QuerySize=315	gi|6802700|ref|Os02g28540|	
gi|31231332|	lcl|At3g17540.1	lcl|C31H2.2 CE04130 WBGene00001070 locus:dpy-8 collagen alpha (weak) status:Confirmed SW:Q18345 protein_id:AAA8333	gi|6802711|ref|Os02g28650|	
gi|31231558|	lcl|At3g17560.1	lcl|C31H5.1 CE34471 WBGene00007854 status:Partially_confirmed SW:O62084 protein_id:CAB07844.2 /QuerySize=336	gi|6802725|ref|Os02g28790|	
gi|31231683|	lcl|At3g17570.1	lcl|C32B5.2 CE08510 WBGene00005479 locus:srh-274 7TM chemoreceptor	gi|6802733|ref|Os02g28870|	
gi|31231975|	lcl|At3g17620.1	lcl|C32C4.5 CE31427 WBGene00003114 locus:mab-23 status:Confirmed SW:Q8MQA9 protein_id:CAD44103.1 /QuerySize=17	gi|6802742|ref|Os02g28960|	
gi|31232026|	lcl|At3g17710.1	lcl|C32D5.10 CE29688 WBGene00016317 status:Partially_confirmed SW:Q09268 protein_id:AAC46798.2 /QuerySize=610	gi|6802743|ref|Os02g28970|	
gi|31232453|	lcl|At3g18035.1	lcl|C32D5.11 CE02527 WBGene00016318 status:Partially_confirmed SW:Q18355 protein_id:AAC46800.1 /QuerySize=770	gi|6802752|ref|Os02g29050|	
gi|31232791|	lcl|At3g18150.1	lcl|C32D5.12 CE01841 WBGene00016319 status:Partially_confirmed SW:Q09491 protein_id:AAC46799.1 /QuerySize=357	gi|6802791|ref|Os02g29430|	
gi|31233189|	lcl|At3g18320.1	lcl|C32D5.8a CE01848 WBGene00016316 Thioredoxin status:Confirmed SW:Q09489 protein_id:AAC46796.1 /QuerySize=140	gi|6802809|ref|Os02g29610|	
gi|31233360|	lcl|At3g18330.1	lcl|C32D5.8b CE37326 WBGene00016316 status:Confirmed SW:Q65ZJ7 protein_id:AAU20849.1 /QuerySize=167	gi|6802816|ref|Os02g29680|	
gi|31233367|	lcl|At3g18340.1	lcl|C32E8.1 CE08525 WBGene00016320 status:Partially_confirmed SW:P91131 protein_id:AAB42321.1 /QuerySize=154	gi|6802817|ref|Os02g29690|	
gi|31233430|	lcl|At3g18360.1	lcl|C32H11.1 CE19708 WBGene00007864 status:Partially_confirmed SW:Q9XTE4 protein_id:CAA97437.1 /QuerySize=462	gi|6802818|ref|Os02g29700|	
gi|31233434|	lcl|At3g18650.1	lcl|C32H11.10 CE19717 WBGene00007873 locus:dod-21 status:Partially_confirmed SW:Q9U3N6 protein_id:CAB05136.1 /Que	gi|6802821|ref|Os02g29730|	
gi|31233747|	lcl|At3g18690.1	lcl|C32H11.11 CE19715 WBGene00007874 status:Predicted SW:Q9XTN7 protein_id:CAB05137.1 /QuerySize=195	gi|6802822|ref|Os02g29740|	
gi|31233977|	lcl|At3g18910.1	lcl|C32H11.12 CE19718 WBGene00007875 locus:dod-24 status:Partially_confirmed SW:Q9XUH3 protein_id:CAB05138.1 /Que	gi|6802825|ref|Os02g29770|	
gi|31234749|	lcl|At3g18980.1	lcl|C32H11.13 CE19719 WBGene00007876 status:Partially_confirmed SW:Q9XUH2 protein_id:CAB05139.1 /QuerySize=359	gi|6802829|ref|Os02g29810|	
gi|31234754|	lcl|At3g19070.1	lcl|C32H11.2 CE19709 WBGene00007865 locus:srz-15 status:Predicted SW:Q9XUH1 protein_id:CAB05140.1 /QuerySize=332	gi|6802843|ref|Os02g29950|	
gi|31235209|	lcl|At3g19085.1	lcl|C32H11.3 CE19710 WBGene00007866 status:Partially_confirmed SW:Q9XUH8 protein_id:CAB05130.1 /QuerySize=344	gi|6802850|ref|Os02g30020|	
gi|31235215|	lcl|At3g19150.1	lcl|C32H11.4 CE19711 WBGene00007867 status:Partially_confirmed SW:Q9XUH7 protein_id:CAB05131.1 /QuerySize=346	gi|6802860|ref|Os02g30120|	
gi|31235220|	lcl|At3g19410.1	lcl|C32H11.5 CE19712 WBGene00007868 status:Confirmed SW:Q9XUH6 protein_id:CAB05132.1 /QuerySize=194	gi|6802888|ref|Os02g30390|	
gi|31236101|	lcl|At3g19470.1	lcl|C32H11.6 CE19713 WBGene00007869 status:Predicted SW:Q9XUH5 protein_id:CAB05133.1 /QuerySize=194	gi|6802897|ref|Os02g30480|	
gi|31236213|	lcl|At3g19515.1	lcl|C32H11.8 CE19715 WBGene00007871 status:Predicted SW:Q9XTN7 protein_id:CAB05134.1 /QuerySize=195	gi|6802918|ref|Os02g30670|	
gi|31236810|	lcl|At3g19520.1	lcl|C32H11.9 CE19716 WBGene00007872 status:Partially_confirmed SW:Q9XUH4 protein_id:CAB05135.1 /QuerySize=369	gi|6802922|ref|Os02g30710|	
gi|31236907|	lcl|At3g19880.1	lcl|C33A11.1 CE24824 WBGene00007877 Ank repeat status:Confirmed SW:Q93318 protein_id:CAB61033.1 /QuerySize=611	gi|6802927|ref|Os02g30750|	
gi|31237018|	lcl|At3g19890.1	lcl|C33A12.10 CE05356 WBGene00005667 locus:sru-4 7TM receptor status:Partially_confirmed SW:Q18364 protein_id:CAA92	gi|6802929|ref|Os02g30770|	
gi|31237219|	lcl|At3g20030.1	lcl|C33A12.11 CE05357 WBGene00005666 locus:sru-3 7TM receptor status:Partially_confirmed SW:Q18366 protein_id:CAA92	gi|6802957|ref|Os02g31050|	
gi|31237507|	lcl|At3g20370.1	lcl|C33A12.12 CE15665 WBGene00007887 Leucine Rich Repeat (2 copies) (2 domains) status:Partially_confirmed SW:Q1836	gi|6802970|ref|Os02g31170|	
gi|31237611|	lcl|At3g20380.1	lcl|C33A12.13 CE15666 WBGene00005665 locus:sru-2 status:Partially_confirmed SW:O17633 protein_id:CAA92796.1 /Query	gi|6802981|ref|Os02g31270|	
gi|31237687|	lcl|At3g20590.1	lcl|C33A12.14 CE34527 WBGene00005664 locus:sru-1 status:Partially_confirmed SW:O17634 protein_id:CAA92797.2 /Query	gi|6802985|ref|Os02g31300|	
gi|31237823|	lcl|At3g20600.1	lcl|C33A12.15 CE35419 WBGene00007888 Transthyretin-like family status:Partially_confirmed SW:O17635 protein_id:CAA927	gi|6803123|ref|Os02g33120|	
gi|31238151|	lcl|At3g20610.1	lcl|C33A12.16 CE32157 WBGene00007889 status:Predicted SW:Q18362 protein_id:CAA92799.2 /QuerySize=266	gi|6803133|ref|Os02g33210|	
gi|31238386|	lcl|At3g20620.1	lcl|C33A12.8 CE05354 WBGene00005669 locus:sru-6 7TM receptor status:Predicted SW:Q18363 protein_id:CAA92792.1 /Que	gi|6803138|ref|Os02g33260|	
gi|31238631|	lcl|At3g20690.1	lcl|C33C12.5 CE08553 WBGene00016337 status:Partially_confirmed SW:O16577 protein_id:AAB66057.1 /QuerySize=461	gi|6803144|ref|Os02g33310|	
gi|31238937|	lcl|At3g20700.1	lcl|C33C12.6 CE08554 WBGene00016338 locus:srb-14 status:Partially_confirmed SW:O16578 protein_id:AAB66058.1 /Query	gi|6803145|ref|Os02g33320|	
gi|31238953|	lcl|At3g20710.1	lcl|C33D9.2 CE03043 WBGene00007897 C-lectin binding domain status:Predicted SW:Q18373 protein_id:CAA92283.1 /Query	gi|6803150|ref|Os02g33370|	

gi|31239159|	lcl|At3g21120.1	lcl|C33D9.4 CE03045 WBGene00005698 locus:sru-35 status:Predicted SW:Q18375 protein_id:CAA92285.1 /QuerySize=323	gi|6803174|ref|Os02g33600|
gi|31239187|	lcl|At3g21130.1	lcl|C33E10.1 CE34265 WBGene00016345 status:Confirmed SW:O61801 protein_id:AAC16976.2 /QuerySize=74	gi|6803199|ref|Os02g33840|
gi|31239235|	lcl|At3g21170.1	lcl|C33E10.2 CE17491 WBGene00016346 status:Partially_confirmed SW:O61800 protein_id:AAC16977.1 /QuerySize=305	gi|6803202|ref|Os02g33870|
gi|31239305|	lcl|At3g21410.1	lcl|C33E10.6 CE23567 WBGene00016348 status:Predicted SW:Q9N504 protein_id:AAK84500.1 /QuerySize=1014	gi|6803204|ref|Os02g33890|
gi|31239435|	lcl|At3g21490.1	lcl|C33E10.8 CE35420 WBGene00016349 status:Partially_confirmed SW:O61798 protein_id:AAC16980.2 /QuerySize=303	gi|6803211|ref|Os02g33960|
gi|31239521|	lcl|At3g22100.1	lcl|C33F10.13 CE29694 WBGene00016359 status:Partially_confirmed SW:Q95QS7 protein_id:AAL16311.1 /QuerySize=363	gi|6803214|ref|Os02g33990|
gi|31239631|	lcl|At3g22350.1	lcl|C33F10.9 CE02806 WBGene00003435 locus:msp-40 major sperm protein status:Confirmed SW:P53017 protein_id:AAK314	gi|6803216|ref|Os02g34010|
gi|31239689|	lcl|At3g22421.1	lcl|C33G3.3 CE08559 WBGene00007903 status:Partially_confirmed SW:Q93323 protein_id:CAB01736.1 /QuerySize=409	gi|6803232|ref|Os02g34170|
gi|31239715|	lcl|At3g22570.1	lcl|C33G8.1 CE34730 WBGene00005874 locus:srw-127 7TM chemoreceptor	gi|6803236|ref|Os02g34210|
gi|31239817|	lcl|At3g22580.1	lcl|C33G8.10 CE06919 WBGene00016367 zinc finger portein status:Partially_confirmed SW:Q18395 protein_id:AAC25853.1 /Q	gi|6803248|ref|Os02g34330|
gi|31239931|	lcl|At3g22650.1	lcl|C33G8.11 CE34731 WBGene00003697 locus:nhr-107 nuclear hormone receptor status:Confirmed SW:Q86PJ2 protein_id:A	gi|6803264|ref|Os02g34490|
gi|31239963|	lcl|At3g22700.1	lcl|C33G8.13 CE27365 WBGene00016369 status:Partially_confirmed SW:Q95ZX7 protein_id:AAK84501.1 /QuerySize=176	gi|6803373|ref|Os02g35520|
gi|31239965|	lcl|At3g22710.1	lcl|C33G8.8 CE06917 WBGene00016365 zinc finger protein status:Partially_confirmed SW:Q18393 protein_id:AAC25852.1 /Qu	gi|6803377|ref|Os02g35560|
gi|31239969|	lcl|At3g22720.1	lcl|C33H5.1 CE36582 WBGene00016370 status:Predicted SW:Q18417 protein_id:AAA82262.2 /QuerySize=295	gi|6803387|ref|Os02g35650|
gi|31240147|	lcl|At3g22730.1	lcl|C33H5.15 CE34732 WBGene00016381 locus:sgo-1 status:Partially_confirmed SW:Q18412 protein_id:AAA82271.2 /QueryS	gi|6803423|ref|Os02g36010|
gi|31240283|	lcl|At3g22770.1	lcl|C33H5.2 CE04146 WBGene00016371 status:Partially_confirmed SW:Q18416 protein_id:AAA82261.1 /QuerySize=507	gi|6803431|ref|Os02g36090|
gi|31240477|	lcl|At3g22870.1	lcl|C34B2.2 CE16890 WBGene00016387 locus:kbp-5 status:Confirmed SW:O44956 protein_id:AAB97534.1 /QuerySize=135	gi|6803489|ref|Os02g36650|
gi|31240577|	lcl|At3g22940.1	lcl|C34B4.2 CE36688 WBGene00007907 Activin types I and II receptor domain status:Partially_confirmed SW:Q18419 protein_	gi|6803557|ref|Os02g37300|
gi|31240705|	lcl|At3g23140.1	lcl|C34B4.3 CE36583 WBGene00007908 status:Partially_confirmed SW:Q6BES4 protein_id:CAH04707.1 /QuerySize=362	gi|6803578|ref|Os02g37510|
gi|31240717|	lcl|At3g23175.1	lcl|C34B4.4 CE36689 WBGene00007909 status:Predicted SW:Q6BES5 protein_id:CAH04726.1 /QuerySize=560	gi|6803590|ref|Os02g37620|
gi|31240901|	lcl|At3g23260.1	lcl|C34B4.5 CE36690 WBGene00007910 status:Predicted SW:Q6BES6 protein_id:CAH04725.1 /QuerySize=300	gi|6803601|ref|Os02g37730|
gi|31240907|	lcl|At3g23320.1	lcl|C34B7.1 CE27817 WBGene00007911 status:Partially_confirmed SW:P90769 protein_id:CAB05700.2 /QuerySize=440	gi|6803602|ref|Os02g37740|
gi|31240945|	lcl|At3g23370.1	lcl|C34C12.1 CE01509 WBGene00007920 status:Predicted SW:Q09494 protein_id:CAA87099.1 /QuerySize=472	gi|6803618|ref|Os02g37900|
gi|31240953|	lcl|At3g23420.1	lcl|C34C12.6 CE01514 WBGene00007925 status:Partially_confirmed SW:Q09270 protein_id:CAA87097.1 /QuerySize=392	gi|6803662|ref|Os02g38280|
gi|31241063|	lcl|At3g23685.1	lcl|C34C6.1 CE33033 WBGene00005172 locus:srg-15 G-protein coupled receptor	gi|6803672|ref|Os02g38370|
gi|31241119|	lcl|At3g23880.1	lcl|C34D1.2 CE08570 WBGene00007930 DM DNA binding domain status:Partially_confirmed SW:Q18435 protein_id:CAB0149	gi|6803685|ref|Os02g38500|
gi|31241197|	lcl|At3g23950.1	lcl|C34D1.5 CE18541 WBGene00007932 Basic region plus leucine zipper transcription factors status:Partially_confirmed SW:Q	gi|6803692|ref|Os02g38570|
gi|31241239|	lcl|At3g23960.1	lcl|C34D4.10 CE17504 WBGene00016402 status:Partially_confirmed SW:Q18440 protein_id:AAB00692.1 /QuerySize=362	gi|6803694|ref|Os02g38590|
gi|31241307|	lcl|At3g24060.1	lcl|C34D4.3 CE06924 WBGene00016399 status:Confirmed SW:Q18451 protein_id:AAB00703.1 /QuerySize=117	gi|6803702|ref|Os02g38670|
gi|31241355|	lcl|At3g24065.1	lcl|C34D4.8 CE24826 WBGene00006113 locus:str-48 7TM chemoreceptor	gi|6803707|ref|Os02g38720|
gi|31241905|	lcl|At3g24070.1	lcl|C34F11.1 CE04162 WBGene00016413 status:Partially_confirmed SW:Q18466 protein_id:AAA85765.1 /QuerySize=226	gi|6803718|ref|Os02g38830|
gi|31241969|	lcl|At3g24150.1	lcl|C34F11.4 CE02806 WBGene00003443 locus:msp-50 major sperm protein status:Partially_confirmed SW:P53017 protein_id:	gi|6803748|ref|Os02g39120|
gi|31242045|	lcl|At3g24270.1	lcl|C34F11.6 CE04166 WBGene00003442 locus:msp-49 major sperm protein status:Confirmed SW:Q18461 protein_id:AAA857	gi|6803759|ref|Os02g39230|
gi|31242047|	lcl|At3g24370.1	lcl|C34F6.10 CE15679 WBGene00007944 Fibronectin type III domain (4 domains) status:Partially_confirmed SW:O17637 prote	gi|6803760|ref|Os02g39240|
gi|31242049|	lcl|At3g24390.1	lcl|C34F6.2 CE15671 WBGene00000751 locus:col-178 collagen status:Confirmed SW:O17641 protein_id:CAB03941.1 /QueryS	gi|6803762|ref|Os02g39260|
gi|31242051|	lcl|At3g24580.1	lcl|C34F6.3 CE15672 WBGene00000752 locus:col-179 collagen status:Partially_confirmed SW:O17642 protein_id:CAB03942.1	gi|6803779|ref|Os02g39430|
gi|31242391|	lcl|At3g24610.1	lcl|C34F6.9 CE15678 WBGene00007943 Ubiquitin carboxyl-terminal hydrolases family 2 status:Partially_confirmed SW:O1764	gi|6803782|ref|Os02g39460|
gi|31242767|	lcl|At3g24700.1	lcl|C34G6.2 CE08574 WBGene00016419 status:Confirmed SW:O01497 protein_id:AAB52481.1 /QuerySize=751	gi|6803851|ref|Os02g40050|
gi|31242919|	lcl|At3g24760.1	lcl|C34G6.6a CE29699 WBGene00016422 status:Partially_confirmed SW:O01494 protein_id:AAB52479.2 /QuerySize=1065	gi|6803888|ref|Os02g40360|
gi|31242949|	lcl|At3g24850.1	lcl|C34G6.6b CE30432 WBGene00016422 status:Partially_confirmed SW:Q8T3B7 protein_id:AAM15558.1 /QuerySize=1069	gi|6803890|ref|Os02g40380|
gi|31243075|	lcl|At3g25080.1	lcl|C34H4.1 CE25804 WBGene00016424 status:Partially_confirmed SW:O45144 protein_id:AAC04390.3 /QuerySize=397	gi|6803892|ref|Os02g40400|
gi|31243099|	lcl|At3g25090.1	lcl|C34H4.2 CE31328 WBGene00016425 status:Confirmed SW:O45143 protein_id:AAC04391.2 /QuerySize=449	gi|6803899|ref|Os02g40470|
gi|31247928|	lcl|At3g25170.1	lcl|C34H4.3 CE27820 WBGene00016426 status:Confirmed SW:O45142 protein_id:AAC04392.2 /QuerySize=399	gi|6803937|ref|Os02g40850|
gi|31247964|	lcl|At3g25460.1	lcl|C34H4.4a CE27821 WBGene00000681 locus:col-107 cuticular collagen status:Confirmed SW:O45140 protein_id:AAC04393	gi|6803947|ref|Os02g40930|
gi|31247976|	lcl|At3g25550.1	lcl|C34H4.4b CE20539 WBGene00000681 locus:col-107 status:Confirmed SW:Q86S35 protein_id:AAO44912.1 /QuerySize=294	gi|6803948|ref|Os02g40940|
gi|31247978|	lcl|At3g25717.1	lcl|C35A5.4 CE35422 WBGene00007948 status:Partially_confirmed SW:Q18471 protein_id:CAA94905.2 /QuerySize=427	gi|6803979|ref|Os02g41690|
gi|31247980|	lcl|At3g25750.1	lcl|C35A5.5 CE37810 WBGene00007949 status:Predicted SW:Q18473 protein_id:CAA94907.1 /QuerySize=520	gi|6803994|ref|Os02g41840|
gi|31248010|	lcl|At3g25850.1	lcl|C35B8.1 CE02534 WBGene00000748 locus:col-175 Cuticle collagen status:Confirmed SW:Q18480 protein_id:AAC46553.1 /	gi|6804005|ref|Os02g41930|
gi|31248014|	lcl|At3g26010.1	lcl|C35D10.11 CE01192 WBGene00016447 status:Confirmed SW:Q27317 protein_id:AAA62567.1 /QuerySize=109	gi|6804009|ref|Os02g41970|
gi|31248016|	lcl|At3g26090.1	lcl|C35D10.15 CE27829 WBGene00016451 status:Partially_confirmed SW:Q95QR9 protein_id:AAK68224.1 /QuerySize=407	gi|6804012|ref|Os02g42000|
gi|31248076|	lcl|At3g26240.1	lcl|C35D10.9 CE01203 WBGene00000418 locus:ced-4 status:Confirmed SW:P30429 protein_id:AAA62564.1 /QuerySize=549	gi|6804052|ref|Os02g42390|
gi|31248114|	lcl|At3g26250.1	lcl|C35D6.1 CE15681 WBGene00005436 locus:srh-228 status:Predicted SW:O46344 protein_id:CAB05144.1 /QuerySize=336	gi|6804053|ref|Os02g42400|
gi|32699417|	lcl|At3g26260.1	lcl|C35D6.10 CE37328 WBGene00023513 locus:srz-71 status:Predicted SW:Q5WRL8 protein_id:CAH60757.1 /QuerySize=310	gi|6804058|ref|Os02g42450|
gi|3282590|	lcl|At3g26450.1	lcl|C35D6.2 CE15681 WBGene00005435 locus:srh-227 status:Predicted SW:O46344 protein_id:CAB05145.1 /QuerySize=336	gi|6804080|ref|Os02g42630|
gi|33355867|	lcl|At3g26460.1	lcl|C35D6.5 CE15684 WBGene00007962 status:Predicted SW:O45290 protein_id:CAB05148.1 /QuerySize=271	gi|6804094|ref|Os02g42760|
gi|33355869|	lcl|At3g26470.1	lcl|C35D6.9 CE37329 WBGene00023508 locus:srz-38 status:Predicted SW:Q5WRL9 protein_id:CAH60756.1 /QuerySize=304	gi|6804102|ref|Os02g42840|
gi|33355873|	lcl|At3g26530.1	lcl|C35E7.9 CE17522 WBGene00016461 status:Partially_confirmed SW:O61765 protein_id:AAC17524.1 /QuerySize=301	gi|6804145|ref|Os02g43270|
gi|33355875|	lcl|At3g26550.1	lcl|C36B1.11 CE05378 WBGene00007978 status:Confirmed SW:Q93345 protein_id:CAB02276.1 /QuerySize=327	gi|6804188|ref|Os02g43670|
gi|33355881|	lcl|At3g26860.1	lcl|C36B1.9 CE05376 WBGene00007976 Zinc finger	gi|6804238|ref|Os02g44060|
gi|33355887|	lcl|At3g26870.1	lcl|C36B7.7 CE04178 WBGene00001841 locus:hen-1 status:Confirmed SW:Q966P4 protein_id:AAK68234.1 /QuerySize=117	gi|6804246|ref|Os02g44130|
gi|33355889|	lcl|At3g26880.1	lcl|C36C5.12 CE08601 WBGene00016481 status:Predicted SW:O16406 protein_id:AAB65934.1 /QuerySize=219	gi|6804248|ref|Os02g44150|
gi|3378529|	lcl|At3g26930.1	lcl|C36C5.14 CE08603 WBGene00016483 status:Partially_confirmed SW:O16408 protein_id:AAB65923.1 /QuerySize=201	gi|6804250|ref|Os02g44170|
gi|3378531|	lcl|At3g27070.1	lcl|C36C5.15 CE08604 WBGene00016484 status:Partially_confirmed SW:O16410 protein_id:AAB65922.1 /QuerySize=203	gi|6804276|ref|Os02g44390|
gi|3617766|	lcl|At3g27473.1	lcl|C36C5.3 CE08592 WBGene00016472 status:Predicted SW:O16411 protein_id:AAB65927.1 /QuerySize=225	gi|6804279|ref|Os02g44420|
gi|37220718|	lcl|At3g27480.1	lcl|C36C5.4 CE08593 WBGene00016473 status:Predicted SW:O16409 protein_id:AAB65928.1 /QuerySize=201	gi|6804282|ref|Os02g44450|
gi|37220720|	lcl|At3g27490.1	lcl|C36C5.5 CE08594 WBGene00016474 status:Partially_confirmed SW:O16405 protein_id:AAB65924.1 /QuerySize=204	gi|6804329|ref|Os02g44870|
gi|37220722|	lcl|At3g27510.1	lcl|C36C5.9 CE08598 WBGene00016478 locus:srt-72 status:Predicted SW:Q8ITZ4 protein_id:AAN73879.1 /QuerySize=178	gi|6804341|ref|Os02g44990|
gi|37220724|	lcl|At3g27600.1	lcl|C36C9.2 CE19721 WBGene00006550 locus:tbx-31 status:Predicted SW:Q9TZL0 protein_id:AAC67425.1 /QuerySize=747	gi|6804344|ref|Os02g45020|
gi|37220730|	lcl|At3g27680.1	lcl|C36C9.3 CE19722 WBGene00016486 status:Partially_confirmed SW:Q9TZK9 protein_id:AAC67423.1 /QuerySize=410	gi|6804365|ref|Os02g45210|
gi|37220732|	lcl|At3g27900.1	lcl|C36C9.4 CE19723 WBGene00016487 status:Predicted SW:Q9TZK7 protein_id:AAC67426.1 /QuerySize=671	gi|6804418|ref|Os02g45720|
gi|37220734|	lcl|At3g27910.1	lcl|C36E8.2 CE30888 WBGene00007981 status:Confirmed SW:O62089 protein_id:CAA84649.2 /QuerySize=254	gi|6804426|ref|Os02g45800|
gi|37703114|	lcl|At3g28010.1	lcl|C36F7.5 CE08609 WBGene00007986 status:Partially_confirmed SW:Q93351 protein_id:CAB02815.1 /QuerySize=322	gi|6804447|ref|Os02g46010|
gi|37703116|	lcl|At3g28220.1	lcl|C36H8.1 CE05380 WBGene00007987 major sperm protein like status:Partially_confirmed SW:Q18503 protein_id:CAA93481	gi|6804451|ref|Os02g46050|
gi|37703118|	lcl|At3g28320.1	lcl|C36H8.2 CE24831 WBGene00002128 locus:inx-6 UNC-7 like status:Confirmed SW:Q9U3N4 protein_id:CAB60997.1 /Query	gi|6804476|ref|Os02g46290|
gi|37777692|	lcl|At3g28650.1	lcl|C37A2.7 CE30433 WBGene00016493 ribosomal protein status:Confirmed SW:O01504 protein_id:AAB52450.2 /QuerySize=	gi|6804483|ref|Os02g46360|
gi|37777694|	lcl|At3g28780.1	lcl|C37A5.9 CE30125 WBGene00004202 locus:pry-1 status:Confirmed SW:O62090 protein_id:CAB07332.2 /QuerySize=586	gi|6804492|ref|Os02g46430|
gi|37777696|	lcl|At3g28790.1	lcl|C37C3.13 CE32820 WBGene00016505 status:Confirmed SW:Q8I7H9 protein_id:AAN84848.1 /QuerySize=143	gi|6804506|ref|Os02g46570|

<table>
<tr><td>gi|37777698|</td><td>lcl|At3g28810.1</td><td>lcl|C37C3.4 CE34270 WBGene00001697 locus:grd-8 status:Partially_confirmed SW:Q22914 protein_id:AAC25861.2 /QuerySiz</td><td>gi|6804518|ref|Os02g46690|</td></tr>
<tr><td>gi|37777700|</td><td>lcl|At3g28820.1</td><td>lcl|C37C3.7 CE32822 WBGene00016499 status:Partially_confirmed SW:Q22913 protein_id:AAN84850.1 /QuerySize=356</td><td>gi|6804538|ref|Os02g46870|</td></tr>
<tr><td>gi|37777702|</td><td>lcl|At3g28830.1</td><td>lcl|C37C3.9 CE27368 WBGene00016501 status:Confirmed SW:Q22917 protein_id:AAC25864.2 /QuerySize=313</td><td>gi|6804540|ref|Os02g46890|</td></tr>
<tr><td>gi|37777704|</td><td>lcl|At3g28840.1</td><td>lcl|C37H5.9a CE08632 WBGene00003528 locus:nas-9 status:Partially_confirmed SW:P91137 protein_id:AAB42372.1 /QuerySi</td><td>gi|6804600|ref|Os02g47450|</td></tr>
<tr><td>gi|37777706|</td><td>lcl|At3g28958.1</td><td>lcl|C38C10.3 CE00106 WBGene00008001 status:Partially_confirmed SW:Q03568 protein_id:CAA79547.1 /QuerySize=301</td><td>gi|6804628|ref|Os02g47700|</td></tr>
<tr><td>gi|37811677|</td><td>lcl|At3g29050.1</td><td>lcl|C38C10.4 CE00107 WBGene00001689 locus:gpr-2 status:Partially_confirmed SW:Q03569 protein_id:CAA79548.1 /QuerySi</td><td>gi|6804630|ref|Os02g47720|</td></tr>
<tr><td>gi|37811679|</td><td>lcl|At3g29340.1</td><td>lcl|C38C3.1 CE33279 WBGene00005691 locus:sru-28 7TM chemoreceptor</td><td>gi|6804681|ref|Os02g48230|</td></tr>
<tr><td>gi|37811681|</td><td>lcl|At3g29650.1</td><td>lcl|C38C3.2 CE08638 WBGene00005690 locus:sru-27 7TM chemoreceptor</td><td>gi|6804683|ref|Os02g48250|</td></tr>
<tr><td>gi|37811683|</td><td>lcl|At3g29660.1</td><td>lcl|C38C3.4a CE30257 WBGene00016513 protein kinase status:Confirmed SW:O17074 protein_id:AAB70335.2 /QuerySize=14</td><td>gi|6804700|ref|Os02g48410|</td></tr>
<tr><td>gi|37811685|</td><td>lcl|At3g29730.1</td><td>lcl|C38C3.7 CE08644 WBGene00016515 status:Predicted SW:O17078 protein_id:AAB70336.1 /QuerySize=642</td><td>gi|6804702|ref|Os02g48430|</td></tr>
<tr><td>gi|37811687|</td><td>lcl|At3g29740.1</td><td>lcl|C38C6.4 CE15696 WBGene00007997 locus:sre-13 status:Predicted SW:O45300 protein_id:CAB07564.1 /QuerySize=338</td><td>gi|6804763|ref|Os02g49010|</td></tr>
<tr><td>gi|37962892|</td><td>lcl|At3g29787.1</td><td>lcl|C38D9.1 CE19727 WBGene00008009 status:Partially_confirmed SW:Q9XVC5 protein_id:CAB03947.1 /QuerySize=404</td><td>gi|6804796|ref|Os02g49290|</td></tr>
<tr><td>gi|37962894|</td><td>lcl|At3g29830.1</td><td>lcl|C38D9.4 CE19730 WBGene00008012 status:Predicted SW:Q9XVC2 protein_id:CAB03950.1 /QuerySize=345</td><td>gi|6804818|ref|Os02g49500|</td></tr>
<tr><td>gi|37962896|</td><td>lcl|At3g30310.1</td><td>lcl|C38D9.5 CE19731 WBGene00008013 status:Predicted SW:Q9XTC7 protein_id:CAB07494.1 /QuerySize=1213</td><td>gi|6804826|ref|Os02g49580|</td></tr>
<tr><td>gi|37962898|</td><td>lcl|At3g30396.1</td><td>lcl|C38D9.6 CE24834 WBGene00008014 status:Partially_confirmed SW:Q9U3N1 protein_id:CAB63437.1 /QuerySize=658</td><td>gi|6804857|ref|Os02g49890|</td></tr>
<tr><td>gi|37962900|</td><td>lcl|At3g30440.1</td><td>lcl|C38D9.7 CE24835 WBGene00008015 status:Partially_confirmed SW:Q9U3N2 protein_id:CAB63436.1 /QuerySize=405</td><td>gi|6804865|ref|Os02g49970|</td></tr>
<tr><td>gi|37962902|</td><td>lcl|At3g30450.1</td><td>lcl|C38D9.9 CE24837 WBGene00008017 status:Predicted SW:Q9U3N0 protein_id:CAB63438.1 /QuerySize=660</td><td>gi|6804876|ref|Os02g50080|</td></tr>
<tr><td>gi|37962904|</td><td>lcl|At3g30470.1</td><td>lcl|C39B10.1 CE02157 WBGene00008021 G-protein coupled receptor status:Partially_confirmed SW:Q18517 protein_id:CAA9</td><td>gi|6804930|ref|Os02g50610|</td></tr>
<tr><td>gi|38196217|</td><td>lcl|At3g30500.1</td><td>lcl|C39B5.1 CE37211 WBGene00016519 locus:srz-56 status:Predicted SW:Q9N5W5 protein_id:AAF39766.2 /QuerySize=356</td><td>gi|6804959|ref|Os02g50870|</td></tr>
<tr><td>gi|38196219|</td><td>lcl|At3g30550.1</td><td>lcl|C39B5.11 CE23577 WBGene00005144 locus:srd-67 7TM chemoreceptor</td><td>gi|6805001|ref|Os02g51230|</td></tr>
<tr><td>gi|38196221|</td><td>lcl|At3g30600.1</td><td>lcl|C39B5.12 CE24838 WBGene00005142 locus:srd-65 7TM chemoreceptor</td><td>gi|6805009|ref|Os02g51310|</td></tr>
<tr><td>gi|38196223|</td><td>lcl|At3g30620.1</td><td>lcl|C39B5.3 CE23579 WBGene00016521 status:Partially_confirmed SW:Q9N5W9 protein_id:AAF39768.1 /QuerySize=286</td><td>gi|6805033|ref|Os02g51530|</td></tr>
<tr><td>gi|40019405|</td><td>lcl|At3g30640.1</td><td>lcl|C39B5.4 CE23580 WBGene00016522 status:Partially_confirmed SW:Q9N5X0 protein_id:AAF39769.1 /QuerySize=303</td><td>gi|6805055|ref|Os02g51740|</td></tr>
<tr><td>gi|40019409|</td><td>lcl|At3g30760.1</td><td>lcl|C39B5.7 CE23582 WBGene00016525 status:Partially_confirmed SW:Q9N5X2 protein_id:AAF39771.1 /QuerySize=291</td><td>gi|6805091|ref|Os02g52070|</td></tr>
<tr><td>gi|40019411|</td><td>lcl|At3g30780.1</td><td>lcl|C39B5.8 CE20555 WBGene00016526 status:Predicted SW:Q9N5X3 protein_id:AAF39764.1 /QuerySize=322</td><td>gi|6805095|ref|Os02g52110|</td></tr>
<tr><td>gi|40019415|</td><td>lcl|At3g30810.1</td><td>lcl|C39B5.9 CE29219 WBGene00016527 status:Partially_confirmed SW:Q9N5W8 protein_id:AAF39772.2 /QuerySize=309</td><td>gi|6805141|ref|Os02g52480|</td></tr>
<tr><td>gi|40019417|</td><td>lcl|At3g31314.1</td><td>lcl|C39D10.8a CE29713 WBGene00016535 status:Confirmed SW:Q95QR8 protein_id:AAL16315.1 /QuerySize=133</td><td>gi|6805146|ref|Os02g52530|</td></tr>
<tr><td>gi|40019421|</td><td>lcl|At3g31403.1</td><td>lcl|C39D10.8b CE29714 WBGene00016535 status:Confirmed SW:Q95QR7 protein_id:AAL16316.1 /QuerySize=137</td><td>gi|6805147|ref|Os02g52540|</td></tr>
<tr><td>gi|40019423|</td><td>lcl|At3g31909.1</td><td>lcl|C39D10.8c CE30501 WBGene00016535 status:Partially_confirmed SW:Q8T3E9 protein_id:AAM15559.1 /QuerySize=131</td><td>gi|6805194|ref|Os02g52980|</td></tr>
<tr><td>gi|40019425|</td><td>lcl|At3g31930.1</td><td>lcl|C39E9.1 CE05384 WBGene00008026 testis-specific protein TPX-1 like status:Predicted SW:Q18542 protein_id:CAA94334.1</td><td>gi|6805213|ref|Os02g53150|</td></tr>
<tr><td>gi|40019427|</td><td>lcl|At3g31950.1</td><td>lcl|C39E9.12 CE05395 WBGene00008035 status:Partially_confirmed SW:Q18546 protein_id:CAA94338.1 /QuerySize=444</td><td>gi|6805256|ref|Os02g53540|</td></tr>
<tr><td>gi|40019429|</td><td>lcl|At3g32010.1</td><td>lcl|C39E9.4 CE05387 WBGene00008028 testis-specific protein TPX-1 like status:Partially_confirmed SW:Q18538 protein_id:CA</td><td>gi|6805262|ref|Os02g53600|</td></tr>
<tr><td>gi|40019433|</td><td>lcl|At3g32130.1</td><td>lcl|C39E9.6 CE05389 WBGene00008030 testis-specific protein TPX-1 like status:Partially_confirmed SW:Q18540 protein_id:CA</td><td>gi|6805264|ref|Os02g53620|</td></tr>
<tr><td>gi|40204912|</td><td>lcl|At3g32210.1</td><td>lcl|C39E9.7 CE35427 WBGene00008031 sodium\/phosphate transporter status:Partially_confirmed SW:Q18544 protein_id:CA</td><td>gi|6805265|ref|Os02g53630|</td></tr>
<tr><td>gi|4127333|</td><td>lcl|At3g32240.1</td><td>lcl|C39E9.9 CE05392 WBGene00000706 locus:col-132 collagen status:Partially_confirmed SW:Q18536 protein_id:CAA94328.1</td><td>gi|6805298|ref|Os02g53930|</td></tr>
<tr><td>gi|422409|</td><td>lcl|At3g32260.1</td><td>lcl|C39H7.5 CE06944 WBGene00016543 status:Predicted SW:Q18551 protein_id:AAB00672.1 /QuerySize=263</td><td>gi|6805334|ref|Os02g54220|</td></tr>
<tr><td>gi|4538887|</td><td>lcl|At3g32270.1</td><td>lcl|C39H7.6 CE25807 WBGene00005090 locus:srd-12 7TM chemoreceptor</td><td>gi|6805339|ref|Os02g54270|</td></tr>
<tr><td>gi|4538889|</td><td>lcl|At3g32677.1</td><td>lcl|C39H7.7 CE25808 WBGene00005091 locus:srd-13 7TM chemoreceptor</td><td>gi|6805353|ref|Os02g54400|</td></tr>
<tr><td>gi|4538891|</td><td>lcl|At3g32880.1</td><td>lcl|C40A11.1 CE19364 WBGene00016544 status:Partially_confirmed SW:Q9TZA4 protein_id:AAC68759.1 /QuerySize=321</td><td>gi|6805369|ref|Os02g54550|</td></tr>
<tr><td>gi|461558|</td><td>lcl|At3g32900.1</td><td>lcl|C40A11.2 CE34742 WBGene00016545 status:Partially_confirmed SW:Q9TZA7 protein_id:AAC68760.3 /QuerySize=213</td><td>gi|6805413|ref|Os02g54950|</td></tr>
<tr><td>gi|46395677|</td><td>lcl|At3g32950.1</td><td>lcl|C40A11.5 CE19368 WBGene00016548 protein-tyrosine phosphatase status:Predicted SW:Q9TZB1 protein_id:AAC68761.1</td><td>gi|6805469|ref|Os02g55480|</td></tr>
<tr><td>gi|46395901|</td><td>lcl|At3g33067.1</td><td>lcl|C40D2.1 CE19373 WBGene00016554 status:Predicted SW:O76559 protein_id:AAC26264.1 /QuerySize=208</td><td>gi|6805532|ref|Os02g56070|</td></tr>
<tr><td>gi|46395902|</td><td>lcl|At3g42083.1</td><td>lcl|C40D2.2 CE19374 WBGene00016555 possible trans-splice site at 5004 status:Confirmed SW:O76558 protein_id:AAC26265</td><td>gi|6805541|ref|Os02g56160|</td></tr>
<tr><td>gi|464261|</td><td>lcl|At3g42110.1</td><td>lcl|C40D2.3 CE19375 WBGene00016556 status:Partially_confirmed SW:O76557 protein_id:AAC26266.1 /QuerySize=441</td><td>gi|6805594|ref|Os02g56660|</td></tr>
<tr><td>gi|47779236|</td><td>lcl|At3g42190.1</td><td>lcl|C40D2.4 CE19376 WBGene00016557 status:Predicted SW:O76560 protein_id:AAC26267.1 /QuerySize=147</td><td>gi|6805601|ref|Os02g56730|</td></tr>
<tr><td>gi|48994194|</td><td>lcl|At3g42230.1</td><td>lcl|C40H1.2 CE00110 WBGene00008038 status:Partially_confirmed SW:Q03572 protein_id:CAA79553.1 /QuerySize=208</td><td>gi|6805607|ref|Os02g56780|</td></tr>
<tr><td>gi|48994198|</td><td>lcl|At3g42253.1</td><td>lcl|C40H1.5 CE00113 WBGene00008040 Transthyretin-like family status:Partially_confirmed SW:Q03575 protein_id:CAA79556</td><td>gi|6805622|ref|Os02g56930|</td></tr>
<tr><td>gi|54066769|</td><td>lcl|At3g42280.1</td><td>lcl|C40H1.7 CE33603 WBGene00008042 status:Partially_confirmed SW:Q9BIA1 protein_id:CAC35885.2 /QuerySize=388</td><td>gi|6805623|ref|Os02g56940|</td></tr>
<tr><td>gi|55233397|</td><td>lcl|At3g42400.1</td><td>lcl|C40H1.8 CE34743 WBGene00008043 status:Predicted SW:Q7YX61 protein_id:CAE17740.1 /QuerySize=342</td><td>gi|6805662|ref|Os02g57280|</td></tr>
<tr><td>gi|55233422|</td><td>lcl|At3g42410.1</td><td>lcl|C40H1.9 CE34744 WBGene00008044 status:Predicted SW:Q7YX60 protein_id:CAE17741.1 /QuerySize=335</td><td>gi|6805663|ref|Os02g57280|</td></tr>
<tr><td>gi|55233423|</td><td>lcl|At3g42440.1</td><td>lcl|C40H5.3 CE33982 WBGene00008046 status:Partially_confirmed SW:Q9U3M9 protein_id:CAB03954.3 /QuerySize=377</td><td>gi|6805664|ref|Os02g57280|</td></tr>
<tr><td>gi|55233501|</td><td>lcl|At3g42445.1</td><td>lcl|C40H5.4 CE08652 WBGene00008047 B.subtilis penicillin-binding protein like status:Partially_confirmed SW:Q9U3M7 protei</td><td>gi|6805665|ref|Os02g57280|</td></tr>
<tr><td>gi|55233506|</td><td>lcl|At3g42460.1</td><td>lcl|C40H5.7 CE08655 WBGene00008049 EGF-domain protein status:Predicted SW:Q9BIA0 protein_id:CAB03958.1 /QuerySiz</td><td>gi|6805666|ref|Os02g57280|</td></tr>
<tr><td>gi|55233646|</td><td>lcl|At3g42470.1</td><td>lcl|C41C4.1 CE01516 WBGene00008050 status:Partially_confirmed SW:Q09272 protein_id:CAA88098.1 /QuerySize=329</td><td>gi|6805735|ref|Os02g57860|</td></tr>
<tr><td>gi|55233674|</td><td>lcl|At3g42500.1</td><td>lcl|C41C4.2 CE37883 WBGene00005150 locus:sre-2 status:Partially_confirmed SW:Q09273 protein_id:CAA88099.3 /QuerySiz</td><td>gi|6805736|ref|Os02g57870|</td></tr>
<tr><td>gi|55233675|</td><td>lcl|At3g42580.1</td><td>lcl|C41C4.3 CE32324 WBGene00008051 Leucine Rich Repeat (2 copies) (2 domains) status:Partially_confirmed SW:Q09498 p</td><td>gi|6805737|ref|Os02g57880|</td></tr>
<tr><td>gi|55233713|</td><td>lcl|At3g42620.1</td><td>lcl|C41C4.6 CE01521 WBGene00006739 locus:ulp-4 status:Partially_confirmed SW:Q09275 protein_id:CAA88104.1 /QuerySiz</td><td>gi|6805738|ref|Os02g57890|</td></tr>
<tr><td>gi|55233714|</td><td>lcl|At3g42624.1</td><td>lcl|C41G11.1 CE30894 WBGene00016568 status:Partially_confirmed SW:Q18561 protein_id:AAA85743.2 /QuerySize=417</td><td>gi|6805740|ref|Os02g57910|</td></tr>
<tr><td>gi|55233715|</td><td>lcl|At3g42650.1</td><td>lcl|C41G11.4a CE29716 WBGene00016570 G-protein coupled receptor status:Partially_confirmed SW:Q18564 protein_id:AAA</td><td>gi|6805745|ref|Os02g57960|</td></tr>
<tr><td>gi|55233716|</td><td>lcl|At3g42690.1</td><td>lcl|C41G11.4b CE30896 WBGene00016570 status:Partially_confirmed SW:Q8MQA6 protein_id:AAM51526.1 /QuerySize=514</td><td>gi|6805754|ref|Os02g58040|</td></tr>
<tr><td>gi|55233808|</td><td>lcl|At3g42716.1</td><td>lcl|C41G11.4c CE35428 WBGene00016570 status:Partially_confirmed SW:Q7JP11 protein_id:AAQ65208.1 /QuerySize=557</td><td>gi|6805824|ref|Os02g58700|</td></tr>
<tr><td>gi|55233875|</td><td>lcl|At3g42720.1</td><td>lcl|C41G6.10 CE23586 WBGene00005537 locus:sri-25 7TM chemoreceptor</td><td>gi|6805832|ref|Os02g58780|</td></tr>
<tr><td>gi|55233891|</td><td>lcl|At3g42722.1</td><td>lcl|C41G6.11 CE23587 WBGene00005536 locus:sri-24 7TM chemoreceptor</td><td>gi|6900007|ref|Os03g01060|</td></tr>
<tr><td>gi|55233915|</td><td>lcl|At3g42730.1</td><td>lcl|C41G6.12 CE15709 WBGene00008058 status:Predicted SW:O17664 protein_id:CAB02836.1 /QuerySize=215</td><td>gi|6900008|ref|Os03g01070|</td></tr>
<tr><td>gi|55233993|</td><td>lcl|At3g42760.1</td><td>lcl|C41G6.14 CE23588 WBGene00005538 locus:sri-26 7TM chemoreceptor</td><td>gi|6900043|ref|Os03g01390|</td></tr>
<tr><td>gi|55234015|</td><td>lcl|At3g42770.1</td><td>lcl|C41G6.15 CE23589 WBGene00005760 locus:srw-13 7TM chemoreceptor</td><td>gi|6900044|ref|Os03g01400|</td></tr>
<tr><td>gi|55234094|</td><td>lcl|At3g42820.1</td><td>lcl|C41G6.16 CE36472 WBGene00008060 status:Predicted SW:Q6BES1 protein_id:CAH04642.1 /QuerySize=357</td><td>gi|6900058|ref|Os03g01540|</td></tr>
<tr><td>gi|55234145|</td><td>lcl|At3g42910.1</td><td>lcl|C41G6.2 CE33762 WBGene00005759 locus:srw-12 7TM chemoreceptor</td><td>gi|6900143|ref|Os03g02210|</td></tr>
<tr><td>gi|55234153|</td><td>lcl|At3g42916.1</td><td>lcl|C41G6.3 CE15701 WBGene00005762 locus:srw-15 7TM chemoreceptor</td><td>gi|6900158|ref|Os03g02340|</td></tr>
<tr><td>gi|55234164|</td><td>lcl|At3g42930.1</td><td>lcl|C41G6.5 CE15702 WBGene00008056 Zinc finger</td><td>gi|6900160|ref|Os03g02360|</td></tr>
<tr><td>gi|55234272|</td><td>lcl|At3g42980.1</td><td>lcl|C41G6.6 CE34530 WBGene00008057 status:Partially_confirmed SW:O17659 protein_id:CAB02831.3 /QuerySize=364</td><td>gi|6900188|ref|Os03g02630|</td></tr>
<tr><td>gi|55234320|</td><td>lcl|At3g43040.1</td><td>lcl|C41G6.7 CE33763 WBGene00005771 locus:srw-24 7TM chemoreceptor</td><td>gi|6900253|ref|Os03g03220|</td></tr>
<tr><td>gi|55234336|</td><td>lcl|At3g43100.1</td><td>lcl|C41G6.8 CE33764 WBGene00005761 locus:srw-14 7TM chemoreceptor</td><td>gi|6900254|ref|Os03g03230|</td></tr>
<tr><td>gi|55234337|</td><td>lcl|At3g43130.1</td><td>lcl|C41G6.9 CE15706 WBGene00005381 locus:srh-165 status:Predicted SW:O17662 protein_id:CAB02834.1 /QuerySize=297</td><td>gi|6900255|ref|Os03g03240|</td></tr>
<tr><td>gi|55234338|</td><td>lcl|At3g43170.1</td><td>lcl|C41G7.1a CE08665 WBGene00004887 locus:smn-1 Human muscular atrophy protein like status:Confirmed SW:Q93365 pro</td><td>gi|6900308|ref|Os03g03740|</td></tr>
<tr><td>gi|55234345|</td><td>lcl|At3g43180.1</td><td>lcl|C41G7.1b CE27843 WBGene00004887 locus:smn-1 Human muscular atrophy protein like status:Confirmed SW:Q9U758 pro</td><td>gi|6900334|ref|Os03g03970|</td></tr>
<tr><td>gi|55234378|</td><td>lcl|At3g43355.1</td><td>lcl|C41H7.3 CE08674 WBGene00016573 status:Partially_confirmed SW:O17152 protein_id:AAO38668.1 /QuerySize=373</td><td>gi|6900341|ref|Os03g04040|</td></tr>
</table>

<table>
<tr><td>gi|55234381|</td><td>lcl|At3g43370.1</td><td>lcl|C41H7.4 CE08675 WBGene00016574 status:Partially_confirmed SW:O17151 protein_id:AAB70932.1 /QuerySize=331</td><td>gi|6900412|ref|Os03g04720|</td></tr>
<tr><td>gi|55234393|</td><td>lcl|At3g43380.1</td><td>lcl|C41H7.5 CE32164 WBGene00016575 status:Partially_confirmed SW:O17150 protein_id:AAB70936.2 /QuerySize=226</td><td>gi|6900419|ref|Os03g04790|</td></tr>
<tr><td>gi|55234404|</td><td>lcl|At3g43430.1</td><td>lcl|C41H7.6 CE08677 WBGene00016576 status:Confirmed SW:O17149 protein_id:AAB70937.1 /QuerySize=321</td><td>gi|6900424|ref|Os03g04840|</td></tr>
<tr><td>gi|55234450|</td><td>lcl|At3g43440.1</td><td>lcl|C41H7.8 CE33365 WBGene00016578 status:Predicted SW:Q86S48 protein_id:AAO38669.1 /QuerySize=363</td><td>gi|6900427|ref|Os03g04870|</td></tr>
<tr><td>gi|55234465|</td><td>lcl|At3g43460.1</td><td>lcl|C42C1.1 CE16907 WBGene00016579 locus:sre-14 status:Partially_confirmed SW:O44974 protein_id:AAB97543.1 /QuerySi</td><td>gi|6900431|ref|Os03g04910|</td></tr>
<tr><td>gi|55234505|</td><td>lcl|At3g43563.1</td><td>lcl|C42C1.11b CE33564 WBGene00016589 status:Confirmed SW:Q86GU5 protein_id:AAO61433.1 /QuerySize=157</td><td>gi|6900444|ref|Os03g05000|</td></tr>
<tr><td>gi|55234511|</td><td>lcl|At3g43730.1</td><td>lcl|C42C1.16 CE29223 WBGene00016593 status:Partially_confirmed SW:Q95X51 protein_id:AAL02465.1 /QuerySize=300</td><td>gi|6900494|ref|Os03g05460|</td></tr>
<tr><td>gi|55234542|</td><td>lcl|At3g43862.1</td><td>lcl|C42C1.7 CE29224 WBGene00016585 status:Partially_confirmed SW:O44963 protein_id:AAB97548.2 /QuerySize=710</td><td>gi|6900515|ref|Os03g05670|</td></tr>
<tr><td>gi|55234588|</td><td>lcl|At3g43890.1</td><td>lcl|C42C1.9 CE26913 WBGene00016587 status:Partially_confirmed SW:O44966 protein_id:AAK72294.1 /QuerySize=267</td><td>gi|6900579|ref|Os03g06270|</td></tr>
<tr><td>gi|55234603|</td><td>lcl|At3g44060.1</td><td>lcl|C42D4.1 CE04189 WBGene00016594 status:Confirmed SW:Q18577 protein_id:AAA83352.1 /QuerySize=251</td><td>gi|6900610|ref|Os03g06550|</td></tr>
<tr><td>gi|55234699|</td><td>lcl|At3g44080.1</td><td>lcl|C42D4.11 CE04199 WBGene00016597 status:Partially_confirmed SW:Q18574 protein_id:AAA83349.1 /QuerySize=297</td><td>gi|6900644|ref|Os03g06840|</td></tr>
<tr><td>gi|55234700|</td><td>lcl|At3g44090.1</td><td>lcl|C42D4.3 CE04191 WBGene00016596 status:Confirmed SW:Q18572 protein_id:AAA83347.1 /QuerySize=255</td><td>gi|6900645|ref|Os03g06850|</td></tr>
<tr><td>gi|55234702|</td><td>lcl|At3g44120.1</td><td>lcl|C42D4.4 CE24841 WBGene00006109 locus:str-44 7TM chemoreceptor</td><td>gi|6900662|ref|Os03g07020|</td></tr>
<tr><td>gi|55234703|</td><td>lcl|At3g44130.1</td><td>lcl|C42D4.5 CE25809 WBGene00006069 locus:str-1 7TM chemoreceptor</td><td>gi|6900664|ref|Os03g07040|</td></tr>
<tr><td>gi|55234737|</td><td>lcl|At3g44180.1</td><td>lcl|C42D4.9 CE24842 WBGene00006251 locus:str-220 7TM chemoreceptor</td><td>gi|6900667|ref|Os03g07070|</td></tr>
<tr><td>gi|55234814|</td><td>lcl|At3g44360.1</td><td>lcl|C43C3.1 CE01523 WBGene00002067 locus:ifp-1 intermediate filament protein status:Confirmed SW:Q09501 protein_id:CA</td><td>gi|6900724|ref|Os03g07610|</td></tr>
<tr><td>gi|55234816|</td><td>lcl|At3g44410.1</td><td>lcl|C43C3.3 CE23591 WBGene00001124 locus:dyf-8 status:Partially_confirmed SW:Q09276 protein_id:CAA87330.2 /QuerySiz</td><td>gi|6900725|ref|Os03g07620|</td></tr>
<tr><td>gi|55234911|</td><td>lcl|At3g44500.1</td><td>lcl|C43D7.2 CE18547 WBGene00008066 status:Partially_confirmed SW:Q9XVB8 protein_id:CAB03963.1 /QuerySize=307</td><td>gi|6900727|ref|Os03g07640|</td></tr>
<tr><td>gi|55234943|</td><td>lcl|At3g44713.1</td><td>lcl|C43D7.6 CE35959 WBGene00005418 locus:srh-208 status:Predicted SW:Q9XVB7 protein_id:CAB03964.2 /QuerySize=338</td><td>gi|6900731|ref|Os03g07680|</td></tr>
<tr><td>gi|55234948|</td><td>lcl|At3g44735.1</td><td>lcl|C43D7.9 CE35960 WBGene00008071 status:Predicted SW:Q7JKV5 protein_id:CAE53731.1 /QuerySize=277</td><td>gi|6900742|ref|Os03g07780|</td></tr>
<tr><td>gi|55234949|</td><td>lcl|At3g44770.1</td><td>lcl|C43E11.2b CE32609 WBGene00016602 status:Confirmed /QuerySize=183</td><td>gi|6900786|ref|Os03g08160|</td></tr>
<tr><td>gi|55234955|</td><td>lcl|At3g44780.1</td><td>lcl|C43E11.5 CE08683 WBGene00016605 status:Partially_confirmed /QuerySize=368</td><td>gi|6900801|ref|Os03g08310|</td></tr>
<tr><td>gi|55234958|</td><td>lcl|At3g44800.1</td><td>lcl|C43F9.6 CE19734 WBGene00008074 Sodium and potassium ATPases status:Partially_confirmed SW:Q9XUG9 protein_id:</td><td>gi|6900802|ref|Os03g08320|</td></tr>
<tr><td>gi|55234959|</td><td>lcl|At3g44805.1</td><td>lcl|C43G2.5 CE34751 WBGene00006621 locus:try-3 peptidase status:Partially_confirmed SW:Q94176 protein_id:AAB09110.4</td><td>gi|6900803|ref|Os03g08330|</td></tr>
<tr><td>gi|55235027|</td><td>lcl|At3g44810.1</td><td>lcl|C43H6.5 CE35431 WBGene00016618 status:Partially_confirmed SW:Q18584 protein_id:AAA96083.2 /QuerySize=327</td><td>gi|6900938|ref|Os03g09950|</td></tr>
<tr><td>gi|55235049|</td><td>lcl|At3g45350.1</td><td>lcl|C43H6.6 CE06957 WBGene00016619 status:Partially_confirmed SW:Q95ZX0 protein_id:AAK84504.1 /QuerySize=156</td><td>gi|6900970|ref|Os03g10270|</td></tr>
<tr><td>gi|55235067|</td><td>lcl|At3g45460.1</td><td>lcl|C44B12.4 CE36427 WBGene00005904 locus:srx-13 7TM chemoreceptor</td><td>gi|6900992|ref|Os03g10450|</td></tr>
<tr><td>gi|55235076|</td><td>lcl|At3g45490.1</td><td>lcl|C44B12.6 CE16922 WBGene00016639 status:Predicted SW:O44146 protein_id:AAB88329.1 /QuerySize=162</td><td>gi|6901001|ref|Os03g10540|</td></tr>
<tr><td>gi|55235081|</td><td>lcl|At3g45525.1</td><td>lcl|C44B12.7 CE16923 WBGene00016640 reverse transcriptase status:Predicted SW:O44147 protein_id:AAB88330.1 /QuerySi</td><td>gi|6901002|ref|Os03g10540|</td></tr>
<tr><td>gi|55235089|</td><td>lcl|At3g45530.1</td><td>lcl|C44B12.8 CE36428 WBGene00005905 locus:srx-14 7TM chemoreceptor</td><td>gi|6901066|ref|Os03g11160|</td></tr>
<tr><td>gi|55235101|</td><td>lcl|At3g45800.1</td><td>lcl|C44B7.5 CE02543 WBGene00016627 status:Partially_confirmed SW:Q18594 protein_id:AAA68336.1 /QuerySize=236</td><td>gi|6901094|ref|Os03g11430|</td></tr>
<tr><td>gi|55235151|</td><td>lcl|At3g45840.1</td><td>lcl|C44B9.4 CE30897 WBGene00008081 S.pombe hypothetical protein C27F7.07C like status:Partially_confirmed SW:Q18605</td><td>gi|6901134|ref|Os03g11790|</td></tr>
<tr><td>gi|55235169|</td><td>lcl|At3g46190.1</td><td>lcl|C44B9.6 CE37756 WBGene00043996 status:Predicted SW:Q5R3S7 protein_id:CAI06058.1 /QuerySize=326</td><td>gi|6901135|ref|Os03g11790|</td></tr>
<tr><td>gi|55235216|</td><td>lcl|At3g46470.1</td><td>lcl|C44C10.1 CE05408 WBGene00000753 locus:col-180 collagen status:Partially_confirmed SW:Q18620 protein_id:CAA93642</td><td>gi|6901143|ref|Os03g11870|</td></tr>
<tr><td>gi|55235231|</td><td>lcl|At3g46580.1</td><td>lcl|C44C10.10 CE05417 WBGene00008090 DNA repair protein RECN like status:Predicted SW:Q18618 protein_id:CAA93640.</td><td>gi|6901144|ref|Os03g11880|</td></tr>
<tr><td>gi|55235243|</td><td>lcl|At3g46800.1</td><td>lcl|C44C10.8 CE05415 WBGene00001981 locus:hnd-1 Helix-loop-helix DNA-binding domain status:Partially_confirmed SW:Q1</td><td>gi|6901163|ref|Os03g12070|</td></tr>
<tr><td>gi|55235324|</td><td>lcl|At3g46810.1</td><td>lcl|C44C3.1 CE08707 WBGene00005871 locus:srw-124 7TM chemoreceptor</td><td>gi|6901164|ref|Os03g12070|</td></tr>
<tr><td>gi|55235357|</td><td>lcl|At3g46860.1</td><td>lcl|C44C3.11 CE34754 WBGene00005870 locus:srw-123 7TM chemoreceptor</td><td>gi|6901166|ref|Os03g12090|</td></tr>
<tr><td>gi|55235465|</td><td>lcl|At3g46910.1</td><td>lcl|C44C3.2 CE08708 WBGene00005876 locus:srw-129 7TM chemoreceptor</td><td>gi|6901167|ref|Os03g12100|</td></tr>
<tr><td>gi|55235469|</td><td>lcl|At3g46990.1</td><td>lcl|C44C3.3 CE32611 WBGene00005885 locus:srw-138 7TM chemoreceptor</td><td>gi|6901176|ref|Os03g12190|</td></tr>
<tr><td>gi|55235568|</td><td>lcl|At3g47020.1</td><td>lcl|C44C3.5 CE08711 WBGene00005865 locus:srw-118 7TM chemoreceptor</td><td>gi|6901177|ref|Os03g12200|</td></tr>
<tr><td>gi|55235578|</td><td>lcl|At3g47030.1</td><td>lcl|C44C3.6 CE08712 WBGene00005864 locus:srw-117 7TM chemoreceptor</td><td>gi|6901191|ref|Os03g12340|</td></tr>
<tr><td>gi|55235589|</td><td>lcl|At3g47130.1</td><td>lcl|C44C3.7 CE34531 WBGene00005886 locus:srw-139 7TM chemoreceptor</td><td>gi|6901209|ref|Os03g12480|</td></tr>
<tr><td>gi|55235592|</td><td>lcl|At3g47140.1</td><td>lcl|C44C3.9 CE24849 WBGene00005309 locus:srh-88 7TM chemoreceptor</td><td>gi|6901263|ref|Os03g12990|</td></tr>
<tr><td>gi|55235594|</td><td>lcl|At3g47150.1</td><td>lcl|C44F1.5 CE33567 WBGene00000070 locus:acy-3 adenylyl cyclase status:Partially_confirmed SW:Q18628 protein_id:CAA8</td><td>gi|6901362|ref|Os03g13890|</td></tr>
<tr><td>gi|55235598|</td><td>lcl|At3g47260.1</td><td>lcl|C44H4.5 CE08726 WBGene00006524 locus:tap-1 Protein phosphatase 2C status:Confirmed SW:Q93375 protein_id:CAB01</td><td>gi|6901364|ref|Os03g13910|</td></tr>
<tr><td>gi|55235619|</td><td>lcl|At3g47270.1</td><td>lcl|C44H9.2 CE05419 WBGene00008098 NAM7 like protein status:Predicted SW:Q18630 protein_id:CAA99783.1 /QuerySize=</td><td>gi|6901395|ref|Os03g14150|</td></tr>
<tr><td>gi|55235654|</td><td>lcl|At3g47330.1</td><td>lcl|C45B11.4 CE05428 WBGene00005295 locus:srh-74 status:Partially_confirmed SW:Q18640 protein_id:CAA98432.1 /Query</td><td>gi|6901432|ref|Os03g14490|</td></tr>
<tr><td>gi|55235680|</td><td>lcl|At3g47670.1</td><td>lcl|C45E5.2 CE08730 WBGene00016665 status:Predicted SW:Q18644 protein_id:AAC24288.1 /QuerySize=469</td><td>gi|6901459|ref|Os03g14750|</td></tr>
<tr><td>gi|55235709|</td><td>lcl|At3g48400.1</td><td>lcl|C45E5.6a CE08734 WBGene00003636 locus:nhr-46 nuclear hormone receptor status:Confirmed SW:Q18646 protein_id:AA</td><td>gi|6901461|ref|Os03g14770|</td></tr>
<tr><td>gi|55235767|</td><td>lcl|At3g48840.1</td><td>lcl|C45E5.6c CE29227 WBGene00003636 locus:nhr-46 status:Partially_confirmed SW:Q95QR3 protein_id:AAL02467.1 /Query</td><td>gi|6901463|ref|Os03g14790|</td></tr>
<tr><td>gi|55235776|</td><td>lcl|At3g49020.1</td><td>lcl|C45G3.1 CE15710 WBGene00008107 Actinin-type actin-binding domain containing proteins status:Partially_confirmed SW:</td><td>gi|6901532|ref|Os03g15380|</td></tr>
<tr><td>gi|55235790|</td><td>lcl|At3g49330.1</td><td>lcl|C45G7.1 CE17548 WBGene00016668 status:Predicted SW:O76359 protein_id:AAC19180.1 /QuerySize=145</td><td>gi|6901534|ref|Os03g15400|</td></tr>
</table>

Suppl Table 4. Unique POFs from the different proteomes

Ag	At	Ce	Dm	Hs	Mm	Os	Rn	Sc	Sp
gi|103014|pir||	lcl|At1g01400.1	lcl|2L52.1 CE32090 WBGene00(	gi|17136224|	gi|10092604|ref|NP_065388.1|	gi|10181136|ref|NP_065616.1|	gi|6700015|ref|Os01g01140|	gi|11177908|ref|NP_068634.1|	gi|10383751|ref|NP_009865.2|	gi|19075173|ref|NP_587673.1|
gi|13509402|emb|CAC35450.1|	lcl|At1g01725.1	lcl|3R5.1 CE24758 WBGene000	gi|17136358|	gi|10190658|ref|NP_061192.1|	gi|10181186|ref|NP_065587.1|	gi|6700034|ref|Os01g01330|	gi|12083639|ref|NP_073179.1|	gi|10383754|ref|NP_009871.2|	gi|19075174|ref|NP_587674.1|
gi|13509404|emb|CAC35451.1|	lcl|At1g01730.1	lcl|6R55.2 CE19653 WBGene00	gi|17136392|	gi|10337609|ref|NP_066274.1|	gi|10946780|ref|NP_067392.1|	gi|6700038|ref|Os01g01370|	gi|13242289|ref|NP_077360.1|	gi|10383756|ref|NP_009879.2|	gi|19075181|ref|NP_587681.1|
gi|13509406|emb|CAC35452.1|	lcl|At1g01810.1	lcl|AC7.3 CE07653 WBGene000	gi|17136398|	gi|10835085|ref|NP_005942.1|	gi|10947018|ref|NP_067264.1|	gi|6700046|ref|Os01g01440|	gi|16758858|ref|NP_446420.1|	gi|10383758|ref|NP_009881.2|	gi|19075199|ref|NP_587699.1|
gi|1352587|sp|P34856|	lcl|At1g01840.1	lcl|AC8.10 CE37737 WBGene00	gi|17136464|	gi|10835230|ref|NP_005941.1|	gi|11230808|ref|NP_068698.1|	gi|6700049|ref|Os01g01460|	gi|20301948|ref|NP_620181.1|	gi|10383759|ref|NP_009889.2|	gi|19075219|ref|NP_587719.1|
gi|13537660|emb|CAC35519.1|	lcl|At1g01990.1	lcl|AC8.11 CE20439 WBGene00	gi|17136522|	gi|10835232|ref|NP_005943.1|	gi|11967951|ref|NP_071869.1|	gi|6700056|ref|Os01g01530|	gi|22129747|ref|NP_671484.1|	gi|10383762|ref|NP_009902.2|	gi|19075223|ref|NP_587723.1|
gi|13537662|emb|CAC35520.1|	lcl|At1g02210.1	lcl|AC8.12 CE20441 WBGene00	gi|17136532|	gi|10835250|ref|NP_006499.1|	gi|13384802|ref|NP_079689.1|	gi|6700057|ref|Os01g01540|	gi|22129757|ref|NP_671483.1|	gi|10383769|ref|NP_065437.1|	gi|19075227|ref|NP_587727.1|
gi|13537664|emb|CAC35521.1|	lcl|At1g02320.1	lcl|AC8.3 CE37737 WBGene000	gi|17136584|	gi|10835254|ref|NP_006509.1|	gi|13384862|ref|NP_079733.1|	gi|6700061|ref|Os01g01580|	gi|22219442|ref|NP_671741.1|	gi|10383774|ref|NP_009913.2|	gi|19075232|ref|NP_587732.1|
gi|13537666|emb|CAC35522.1|	lcl|At1g02350.1	lcl|AC8.4 CE20439 WBGene000	gi|17136586|	gi|10863983|ref|NP_067005.1|	gi|13385178|ref|NP_084539.1|	gi|6700062|ref|Os01g01590|	gi|24025627|ref|NP_705888.1|	gi|10383777|ref|NP_009922.2|	gi|19075233|ref|NP_587733.1|
gi|13537668|emb|CAC35523.1|	lcl|At1g02405.1	lcl|AC8.5 CE20440 WBGene000	gi|17136588|	gi|10864019|ref|NP_067031.1|	gi|13385362|ref|NP_080153.1|	gi|6700066|ref|Os01g01630|	gi|25453418|ref|NP_742062.1|	gi|10383783|ref|NP_009941.2|	gi|19075234|ref|NP_587734.1|
gi|13537672|emb|CAC35525.1|	lcl|At1g02450.1	lcl|AC8.6 CE23495 WBGene000	gi|17136610|	gi|10864035|ref|NP_067048.1|	gi|13385710|ref|NP_080480.1|	gi|6700070|ref|Os01g01670|	gi|27659746|ref|XP_238062.1|	gi|10383784|ref|NP_009942.2|	gi|19075238|ref|NP_587738.1|
gi|13537674|emb|CAC35526.1|	lcl|At1g02490.1	lcl|AC8.7 CE20441 WBGene000	gi|17136760|	gi|10947105|ref|NP_055233.1|	gi|13385782|ref|NP_080563.1|	gi|6700071|ref|Os01g01680|	gi|27660238|ref|XP_232590.1|	gi|10383792|ref|NP_009959.2|	gi|19075253|ref|NP_587753.1|
gi|13537676|emb|CAC35527.1|	lcl|At1g02540.1	lcl|AH10.2 CE07656 WBGene00	gi|17136772|	gi|10947107|ref|NP_036600.2|	gi|13385788|ref|NP_080568.1|	gi|6700076|ref|Os01g01730|	gi|27660362|ref|XP_237701.1|	gi|13112038|ref|NP_076437.1|	gi|19075261|ref|NP_587761.1|
gi|13568940|gb|AAK30821.1|	lcl|At1g02570.1	lcl|AH10.3 CE07657 WBGene00	gi|17136844|	gi|10947109|ref|NP_065575.1|	gi|13385880|ref|NP_080670.1|	gi|6700078|ref|Os01g01750|	gi|27662474|ref|XP_238433.1|	gi|13129152|ref|NP_076874.1|	gi|19075262|ref|NP_587762.1|
gi|13568942|gb|AAK30822.1|	lcl|At1g02710.1	lcl|AH10.4 CE07658 WBGene00	gi|17136984|	gi|11036650|ref|NP_066579.1|	gi|13386084|ref|NP_080872.1|	gi|6700079|ref|Os01g01760|	gi|27662624|ref|XP_238512.1|	gi|13129162|ref|NP_076884.1|	gi|19075264|ref|NP_587764.1|
gi|13568944|gb|AAK30823.1|	lcl|At1g02965.1	lcl|AH6.3 CE01457 WBGene000	gi|17136992|	gi|11056010|ref|NP_067648.1|	gi|13386138|ref|NP_080925.1|	gi|6700084|ref|Os01g01810|	gi|27662944|ref|XP_238451.1|	gi|13129167|ref|NP_076889.1|	gi|19075268|ref|NP_587768.1|
gi|13568946|gb|AAK30824.1|	lcl|At1g03170.1	lcl|AH9.3 CE03859 WBGene000	gi|17137022|	gi|11141883|ref|NP_068584.1|	gi|13386166|ref|NP_081087.1|	gi|6700085|ref|Os01g01820|	gi|27664374|ref|XP_213766.1|	gi|13129168|ref|NP_076890.1|	gi|19075272|ref|NP_587772.1|
gi|13568948|gb|AAK30825.1|	lcl|At1g03200.1	lcl|B0001.1 CE32786 WBGene0	gi|17137102|	gi|11345480|ref|NP_068760.1|	gi|13386198|ref|NP_081363.1|	gi|6700092|ref|Os01g01880|	gi|27665630|ref|XP_238530.1|	gi|13129169|ref|NP_076891.1|	gi|19075273|ref|NP_587773.1|
gi|13568950|gb|AAK30826.1|	lcl|At1g03240.1	lcl|B0001.3 CE29523 WBGene0	gi|17137110|	gi|11345488|ref|NP_068764.1|	gi|13386200|ref|NP_081413.1|	gi|6700095|ref|Os01g01910|	gi|27665636|ref|XP_238528.1|	gi|13129172|ref|NP_076894.1|	gi|19075278|ref|NP_587778.1|
gi|13568952|gb|AAK30827.1|	lcl|At1g03320.1	lcl|B0001.5 CE29524 WBGene0	gi|17137120|	gi|11545827|ref|NP_071395.1|	gi|13386232|ref|NP_081764.1|	gi|6700098|ref|Os01g01930|	gi|27666126|ref|XP_237837.1|	gi|13129173|ref|NP_076895.1|	gi|19075283|ref|NP_587783.1|
gi|13568954|gb|AAK30828.1|	lcl|At1g03420.1	lcl|B0001.6 CE31863 WBGene0	gi|17137296|	gi|11545926|ref|NP_068841.1|	gi|13386234|ref|NP_081837.1|	gi|6700099|ref|Os01g01940|	gi|27666626|ref|XP_237748.1|	gi|13129176|ref|NP_076898.1|	gi|19075287|ref|NP_587787.1|
gi|13568956|gb|AAK30829.1|	lcl|At1g03490.1	lcl|B0001.7 CE05145 WBGene0	gi|17137716|	gi|11560154|ref|NP_071382.1|	gi|13386254|ref|NP_081995.1|	gi|6700100|ref|Os01g01950|	gi|27666844|ref|XP_237751.1|	gi|13129177|ref|NP_076899.1|	gi|19075289|ref|NP_587789.1|
gi|13568958|gb|AAK30830.1|	lcl|At1g03660.1	lcl|B0001.8 CE31864 WBGene0	gi|17137718|	gi|11641251|ref|NP_071740.1|	gi|13386262|ref|NP_082120.1|	gi|6700103|ref|Os01g01980|	gi|27667126|ref|XP_238461.1|	gi|13129179|ref|NP_076901.1|	gi|19075293|ref|NP_587793.1|
gi|13568960|gb|AAK30831.1|	lcl|At1g03820.1	lcl|B0024.10 CE05154 WBGene	gi|17137744|	gi|11693180|ref|NP_071770.1|	gi|13386320|ref|NP_082830.1|	gi|6700104|ref|Os01g01990|	gi|27667258|ref|XP_216754.1|	gi|13129181|ref|NP_076903.1|	gi|19075296|ref|NP_587796.1|
gi|13568962|gb|AAK30832.1|	lcl|At1g04330.1	lcl|B0025.4 CE30120 WBGene0	gi|17313268|	gi|11863128|ref|NP_065206.1|	gi|13386374|ref|NP_083475.1|	gi|6700106|ref|Os01g02010|	gi|27668210|ref|XP_238467.1|	gi|13129182|ref|NP_076904.1|	gi|19075298|ref|NP_587798.1|
gi|13568964|gb|AAK30833.1|	lcl|At1g04445.1	lcl|B0034.1 CE02409 WBGene0	gi|17316365|	gi|11968049|ref|NP_071937.1|	gi|13386448|ref|NP_084281.1|	gi|6700111|ref|Os01g02030|	gi|27673001|ref|XP_237782.1|	gi|13129183|ref|NP_076905.1|	gi|19075299|ref|NP_587799.1|
gi|13568966|gb|AAK30834.1|	lcl|At1g04660.1	lcl|B0034.2 CE02410 WBGene0	gi|17530859|	gi|12056975|ref|NP_073154.1|	gi|13386450|ref|NP_084282.1|	gi|6700115|ref|Os01g02070|	gi|27674271|ref|XP_238578.1|	gi|13129184|ref|NP_076906.1|	gi|19075304|ref|NP_587804.1|
gi|13568968|gb|AAK30835.1|	lcl|At1g04670.1	lcl|B0034.3b CE27049 WBGene	gi|17647127|	gi|12232407|ref|NP_073584.1|	gi|13878213|ref|NP_113562.1|	gi|6700123|ref|Os01g02130|	gi|27675326|ref|XP_238077.1|	gi|13129187|ref|NP_076909.1|	gi|19075314|ref|NP_587814.1|
gi|13568970|gb|AAK30836.1|	lcl|At1g04800.1	lcl|B0034.4 CE02412 WBGene0	gi|17647147|	gi|12232427|ref|NP_073595.1|	gi|13928676|ref|NP_113687.1|	gi|6700124|ref|Os01g02140|	gi|27675330|ref|XP_238632.1|	gi|14270689|ref|NP_116327.1|	gi|19075319|ref|NP_587819.1|
gi|13568972|gb|AAK30837.1|	lcl|At1g04930.1	lcl|B0035.15 CE05170 WBGene	gi|17647205|	gi|12545372|ref|NP_001309.2|	gi|13928682|ref|NP_035304.2|	gi|6700125|ref|Os01g02150|	gi|27679384|ref|XP_237911.1|	gi|14318436|ref|NP_011628.2|	gi|19075327|ref|NP_587827.1|
gi|13568974|gb|AAK30838.1|	lcl|At1g05040.1	lcl|B0035.6 CE20443 WBGene0	gi|17647271|	gi|12597651|ref|NP_075058.1|	gi|14389425|ref|NP_035552.1|	gi|6700126|ref|Os01g02160|	gi|27679448|ref|XP_237909.1|	gi|14318451|ref|NP_116587.1|	gi|19075342|ref|NP_587842.1|
gi|13568976|gb|AAK30839.1|	lcl|At1g05065.1	lcl|B0041.1 CE07666 WBGene0	gi|17647281|	gi|13027610|ref|NP_076428.1|	gi|14389427|ref|NP_035553.1|	gi|6700147|ref|Os01g02370|	gi|27681103|ref|XP_238022.1|	gi|14318452|ref|NP_116588.1|	gi|19075343|ref|NP_587843.1|
gi|13568978|gb|AAK30840.1|	lcl|At1g05090.1	lcl|B0041.2a CE27563 WBGene	gi|17647363|	gi|13027790|ref|NP_073627.1|	gi|15042955|ref|NP_149410.1|	gi|6700155|ref|Os01g02450|	gi|27681647|ref|XP_238575.1|	gi|14318454|ref|NP_116590.1|	gi|19075348|ref|NP_587848.1|
gi|159643|gb|AAA29368.1|	lcl|At1g05135.1	lcl|B0041.2b CE29526 WBGene	gi|17647433|	gi|13027820|ref|NP_072056.1|	gi|15987830|ref|NP_082864.1|	gi|6700157|ref|Os01g02470|	gi|27681709|ref|XP_237992.1|	gi|14318455|ref|NP_116591.1|	gi|19075354|ref|NP_587854.1|
gi|17026155|emb|CAD12039.1|	lcl|At1g05135.2	lcl|B0041.2c CE33546 WBGene(	gi|17647637|	gi|13128986|ref|NP_076940.1|	gi|16716599|ref|NP_444495.1|	gi|6700159|ref|Os01g02490|	gi|27682937|ref|XP_238028.1|	gi|14318468|ref|NP_116576.1|	gi|19075360|ref|NP_587860.1|
gi|17026157|emb|CAD12040.1|	lcl|At1g05290.1	lcl|B0041.8 CE29528 WBGene0	gi|17647701|	gi|13129014|ref|NP_076954.1|	gi|18700022|ref|NP_570953.1|	gi|6700172|ref|Os01g02620|	gi|27689915|ref|XP_238220.1|	gi|14318488|ref|NP_116622.1|	gi|19075366|ref|NP_587867.1|
gi|18140727|gb|AAL60411.1|	lcl|At1g05330.1	lcl|B0198.2 CE35377 WBGene0	gi|17647911|	gi|13129112|ref|NP_077009.1|	gi|18875366|ref|NP_573477.1|	gi|6700181|ref|Os01g02710|	gi|27689951|ref|XP_238219.1|	gi|14318508|ref|NP_116641.1|	gi|19075373|ref|NP_587873.1|
gi|18158594|gb|AAL59657.1|	lcl|At1g05340.1	lcl|B0205.10 CE17324 WBGene	gi|17648035|	gi|13162285|ref|NP_076938.1|	gi|19526966|ref|NP_598551.1|	gi|6700195|ref|Os01g02850|	gi|27690866|ref|XP_237817.1|	gi|14318509|ref|NP_116643.1|	gi|19075399|ref|NP_587899.1|
gi|18389879|gb|AAL68776.1|	lcl|At1g05430.1	lcl|B0205.5 CE17319 WBGene0	gi|17737381|	gi|13236555|ref|NP_077290.1|	gi|19527118|ref|NP_598662.1|	gi|6700205|ref|Os01g02950|	gi|27692719|ref|XP_238238.1|	gi|14318511|ref|NP_116645.1|	gi|19075405|ref|NP_587905.1|
gi|18389893|gb|AAL68783.1|	lcl|At1g05450.1	lcl|B0205.8 CE17322 WBGene0	gi|17737433|	gi|13375650|ref|NP_078790.1|	gi|20336730|ref|NP_620396.1|	gi|6700206|ref|Os01g02960|	gi|27694177|ref|XP_214024.1|	gi|14318523|ref|NP_116656.1|	gi|19075408|ref|NP_587908.1|
gi|18389897|gb|AAL68785.1|	lcl|At1g05490.1	lcl|B0207.10 CE07688 WBGene	gi|17737475|	gi|13375680|ref|NP_078806.1|	gi|20341372|ref|XP_112611.1|	gi|6700207|ref|Os01g02970|	gi|27699072|ref|XP_238267.1|	gi|14318530|ref|NP_116663.1|	gi|19075411|ref|NP_587911.1|
gi|18389899|gb|AAL68786.1|	lcl|At1g05575.1	lcl|B0207.2 CE30406 WBGene0	gi|17737563|	gi|13375688|ref|NP_078810.1|	gi|20341766|ref|XP_110600.1|	gi|6700209|ref|Os01g02990|	gi|27702772|ref|XP_238275.1|	gi|14318533|ref|NP_116666.1|	gi|19075412|ref|NP_587912.1|
gi|18389901|gb|AAL68787.1|	lcl|At1g05950.1	lcl|B0207.5 CE32093 WBGene0	gi|17737621|	gi|13375731|ref|NP_078839.1|	gi|20347918|ref|XP_112564.1|	gi|6700211|ref|Os01g03010|	gi|27704202|ref|XP_238185.1|	gi|14318534|ref|NP_116667.1|	gi|19075414|ref|NP_587914.1|
gi|18389903|gb|AAL68788.1|	lcl|At1g06010.1	lcl|B0207.8 CE07686 WBGene0	gi|17737695|	gi|13375830|ref|NP_078890.1|	gi|20820688|ref|XP_149734.1|	gi|6700216|ref|Os01g03060|	gi|27704548|ref|XP_238190.1|	gi|14318535|ref|NP_116668.1|	gi|19075415|ref|NP_587915.1|
gi|18389905|gb|AAL68789.1|	lcl|At1g06225.1	lcl|B0207.9 CE07687 WBGene0	gi|17737805|	gi|13375864|ref|NP_078909.1|	gi|20822551|ref|XP_149590.1|	gi|6700225|ref|Os01g03130|	gi|27705080|ref|XP_238298.1|	gi|14318538|ref|NP_116671.1|	gi|19075426|ref|NP_587926.1|
gi|18389907|gb|AAL68790.1|	lcl|At1g06320.1	lcl|B0213.17 CE30583 WBGene	gi|17737809|	gi|13375962|ref|NP_078964.1|	gi|20824257|ref|XP_149113.1|	gi|6700232|ref|Os01g03200|	gi|27709880|ref|XP_238631.1|	gi|14318539|ref|NP_116672.1|	gi|19075427|ref|NP_587927.1|
gi|18389909|gb|AAL68791.1|	lcl|At1g06420.1	lcl|B0213.2 CE16773 WBGene0	gi|17737871|	gi|13375979|ref|NP_078972.1|	gi|20824551|ref|XP_131958.1|	gi|6700233|ref|Os01g03210|	gi|27713557|ref|XP_238385.1|	gi|14318549|ref|NP_116682.1|	gi|19075439|ref|NP_587939.1|
gi|18389911|gb|AAL68792.1|	lcl|At1g06500.1	lcl|B0213.3 CE16774 WBGene0	gi|17737929|	gi|13375993|ref|NP_078979.1|	gi|20826020|ref|XP_149603.1|	gi|6700236|ref|Os01g03240|	gi|27713850|ref|XP_238390.1|	gi|14318560|ref|NP_116693.1|	gi|19075445|ref|NP_587945.1|
gi|18389913|gb|AAL68793.1|	lcl|At1g06540.1	lcl|B0213.4 CE16775 WBGene0	gi|17737959|	gi|13376025|ref|NP_078996.1|	gi|20826741|ref|XP_149427.1|	gi|6700237|ref|Os01g03250|	gi|27716027|ref|XP_238408.1|	gi|14318561|ref|NP_116694.1|	gi|19075460|ref|NP_587960.1|
gi|18389915|gb|AAL68794.1|	lcl|At1g06810.1	lcl|B0213.5 CE16776 WBGene0	gi|17738017|	gi|13376050|ref|NP_079008.1|	gi|20831184|ref|XP_149617.1|	gi|6700238|ref|Os01g03260|	gi|27718061|ref|XP_238492.1|	gi|14318564|ref|NP_116697.1|	gi|19075462|ref|NP_587962.1|
gi|18389917|gb|AAL68795.1|	lcl|At1g06925.1	lcl|B0213.6 CE16777 WBGene0	gi|17738191|	gi|13376076|ref|NP_079024.1|	gi|20831256|ref|XP_149794.1|	gi|6700240|ref|Os01g03280|	gi|27719331|ref|XP_235797.1|	gi|14318567|ref|NP_116700.1|	gi|19075463|ref|NP_587963.1|
gi|18873404|emb|CAA76824.2|	lcl|At1g06930.1	lcl|B0218.7 CE06690 WBGene0	gi|17738205|	gi|13376113|ref|NP_079044.1|	gi|20833781|ref|XP_149010.1|	gi|6700247|ref|Os01g03350|	gi|27719975|ref|XP_217103.1|	gi|14318568|ref|NP_116701.1|	gi|19075465|ref|NP_587965.1|
gi|19571840|emb|CAD27474.1|	lcl|At1g07135.1	lcl|B0222.10 CE30121 WBGene	gi|17738209|	gi|13376168|ref|NP_079072.1|	gi|20834965|ref|XP_131510.1|	gi|6700257|ref|Os01g03450|	gi|27722157|ref|XP_238621.1|	gi|14318571|ref|NP_116704.1|	gi|19075469|ref|NP_587969.1|
gi|19571843|emb|CAD27477.1|	lcl|At1g07190.1	lcl|B0228.1 CE01742 WBGene0	gi|17738281|	gi|13376220|ref|NP_079099.1|	gi|20837110|ref|XP_130264.1|	gi|6700260|ref|Os01g03480|	gi|27731315|ref|XP_238105.1|	gi|14318574|ref|NP_116707.1|	gi|19075470|ref|NP_587970.1|
gi|19571844|emb|CAD27478.1|	lcl|At1g07300.1	lcl|B0228.4b CE37469 WBGene	gi|17864114|	gi|13376263|ref|NP_079122.1|	gi|20837297|ref|XP_149149.1|	gi|6700266|ref|Os01g03540|	gi|27733121|ref|XP_224527.1|	gi|15530185|ref|NP_219498.1|	gi|19075473|ref|NP_587973.1|
gi|19571845|emb|CAD27479.1|	lcl|At1g07330.1	lcl|B0228.4d CE37471 WBGene	gi|17864160|	gi|13376269|ref|NP_079125.1|	gi|20837699|ref|XP_132190.1|	gi|6700278|ref|Os01g03660|	gi|34851218|ref|XP_346969.1|	gi|21450619|ref|NP_631876.1|	gi|19075474|ref|NP_587974.1|
gi|19571846|emb|CAD27480.1|	lcl|At1g07473.1	lcl|B0228.6 CE31283 WBGene0	gi|17864276|	gi|13376322|ref|NP_079156.1|	gi|20837956|ref|XP_149162.1|	gi|6700279|ref|Os01g03660|	gi|34851561|ref|XP_346522.1|	gi|23270398|ref|NP_690844.1|	gi|19075475|ref|NP_587975.1|
gi|19572382|emb|CAD27928.1|	lcl|At1g07476.1	lcl|B0228.8 CE01748 WBGene0	gi|17864406|	gi|13376327|ref|NP_079158.1|	gi|20840678|ref|XP_131534.1|	gi|6700291|ref|Os01g03770|	gi|34851799|ref|XP_346975.1|	gi|23270400|ref|NP_690846.1|	gi|19075487|ref|NP_587987.1|
gi|19572984|emb|CAD28124.1|	lcl|At1g07490.1	lcl|B0228.9 CE01749 WBGene0	gi|17864574|	gi|13376335|ref|NP_079162.1|	gi|20841153|ref|XP_149473.1|	gi|6700295|ref|Os01g03810|	gi|34852029|ref|XP_346595.1|	gi|23270401|ref|NP_690847.1|	gi|19075488|ref|NP_587988.1|
gi|19577387|emb|CAD27769.1|	lcl|At1g07500.1	lcl|B0238.9 CE32558 WBGene0	gi|17864582|	gi|13376343|ref|NP_079167.1|	gi|20846679|ref|XP_149023.1|	gi|6700299|ref|Os01g03850|	gi|34852043|ref|XP_347001.1|	gi|24416034|ref|NP_710144.1|	gi|19075490|ref|NP_587990.1|
gi|19577389|emb|CAD27770.1|	lcl|At1g07600.1	lcl|B0240.2 CE31865 WBGene0	gi|17864622|	gi|13376376|ref|NP_079188.1|	gi|20848025|ref|XP_129823.1|	gi|6700304|ref|Os01g03900|	gi|34852153|ref|XP_346598.1|	gi|27469362|ref|NP_010782.2|	gi|19075498|ref|NP_587998.1|
gi|21288213|gb|EAA00534.1|	lcl|At1g07610.1	lcl|B0244.11 CE26789 WBGene	gi|17933528|	gi|13376380|ref|NP_079190.1|	gi|20849381|ref|XP_149714.1|	gi|6700306|ref|Os01g03920|	gi|34852177|ref|XP_347004.1|	gi|27808702|ref|NP_009656.2|	gi|19075502|ref|NP_588002.1|
gi|21288273|gb|EAA00594.1|	lcl|At1g07680.1	lcl|B0244.4 CE34517 WBGene0	gi|17933544|	gi|13376384|ref|NP_079192.1|	gi|20853846|ref|XP_147687.1|	gi|6700307|ref|Os01g03930|	gi|34852221|ref|XP_347005.1|	gi|27808709|ref|NP_012515.2|	gi|19075506|ref|NP_588006.1|
gi|21288396|gb|EAA00717.1|	lcl|At1g07690.1	lcl|B0244.5 CE02415 WBGene0	gi|17939404|	gi|13376404|ref|NP_079205.1|	gi|20855076|ref|XP_129929.1|	gi|6700308|ref|Os01g03940|	gi|34852231|ref|XP_347006.1|	gi|28913320|ref|NP_796378.1|	gi|19075507|ref|NP_588007.1|
gi|21289163|gb|EAA01456.1|	lcl|At1g07860.1	lcl|B0244.9 CE01756 WBGene0	gi|17975585|	gi|13376435|ref|NP_079226.1|	gi|20856183|ref|XP_149067.1|	gi|6700310|ref|Os01g03960|	gi|34853437|ref|XP_347385.1|	gi|33438754|ref|NP_878038.1|	gi|19075509|ref|NP_588009.1|
gi|21289773|gb|EAA01918.1|	lcl|At1g08020.1	lcl|B0250.2 CE18479 WBGene0	gi|17977690|	gi|13376453|ref|NP_079237.1|	gi|20856615|ref|XP_129957.1|	gi|6700311|ref|Os01g03970|	gi|34853783|ref|XP_346978.1|	gi|33438755|ref|NP_878039.1|	gi|19075514|ref|NP_588014.1|
gi|21290063|gb|EAA02208.1|	lcl|At1g08035.1	lcl|B0250.4 CE18480 WBGene0	gi|17981733|	gi|13376466|ref|NP_079246.1|	gi|20858155|ref|XP_127107.1|	gi|6700312|ref|Os01g03980|	gi|34853785|ref|XP_346979.1|	gi|33438756|ref|NP_878040.1|	gi|19075517|ref|NP_588017.1|
gi|21290088|gb|EAA02233.1|	lcl|At1g08180.1	lcl|B0250.8 CE18484 WBGene0	gi|17986021|	gi|13376474|ref|NP_079250.1|	gi|20861286|ref|XP_149251.1|	gi|6700313|ref|Os01g03990|	gi|34853962|ref|XP_346685.1|	gi|33438757|ref|NP_878041.1|	gi|19075519|ref|NP_588019.1|
gi|21290982|gb|EAA03127.1|	lcl|At1g08580.1	lcl|B0252.5 CE02421 WBGene0	gi|17986063|	gi|13376476|ref|NP_079252.1|	gi|20861433|ref|XP_150060.1|	gi|6700314|ref|Os01g04000|	gi|34854086|ref|XP_344829.1|	gi|33438758|ref|NP_878042.1|	gi|19075521|ref|NP_588021.1|
gi|21291505|gb|EAA03650.1|	lcl|At1g08910.1	lcl|B0252.6 CE34632 WBGene0	gi|17986081|	gi|13376478|ref|NP_079253.1|	gi|20863456|ref|XP_134133.1|	gi|6700315|ref|Os01g04010|	gi|34854094|ref|XP_344833.1|	gi|33438761|ref|NP_878043.1|	gi|19075522|ref|NP_588022.1|
gi|21291587|gb|EAA03732.1|	lcl|At1g08985.1	lcl|B0261.5 CE07707 WBGene0	gi|17986103|	gi|13376480|ref|NP_079254.1|	gi|20864095|ref|XP_149287.1|	gi|6700321|ref|Os01g04060|	gi|34854102|ref|XP_344835.1|	gi|33438762|ref|NP_878044.1|	gi|19075530|ref|NP_588030.1|
gi|21292251|gb|EAA04396.1|	lcl|At1g09415.1	lcl|B0261.6 CE29532 WBGene0	gi|17986153|	gi|13376492|ref|NP_079264.1|	gi|20864452|ref|XP_150076.1|	gi|6700323|ref|Os01g04080|	gi|34854143|ref|XP_347010.1|	gi|33438763|ref|NP_878045.1|	gi|19075531|ref|NP_588031.1|
gi|21293572|gb|EAA05717.1|	lcl|At1g09990.1	lcl|B0261.7 CE07709 WBGene0	gi|17986155|	gi|13376515|ref|NP_079278.1|	gi|20866347|ref|XP_137325.1|	gi|6700325|ref|Os01g04100|	gi|34854188|ref|XP_238225.2|	gi|33438764|ref|NP_878046.1|	gi|19075532|ref|NP_588032.1|
gi|21294045|gb|EAA06190.1|	lcl|At1g09995.1	lcl|B0261.8 CE34240 WBGene0	gi|18110125|	gi|13376521|ref|NP_079281.1|	gi|20878396|ref|XP_149397.1|	gi|6700331|ref|Os01g04160|	gi|34854252|ref|XP_227100.2|	gi|33438765|ref|NP_878047.1|	gi|19075538|ref|NP_588038.1|
gi|21294374|gb|EAA06519.1|	lcl|At1g10100.1	lcl|B0273.1 CE16787 WBGene0	gi|18543213|	gi|13376527|ref|NP_079284.1|	gi|20879998|ref|XP_147490.1|	gi|6700332|ref|Os01g04170|	gi|34854931|ref|XP_346694.1|	gi|33438766|ref|NP_878048.1|	gi|19075543|ref|NP_588043.1|
gi|21294423|gb|EAA06568.1|	lcl|At1g10140.1	lcl|B0280.7 CE29535 WBGene0	gi|18543223|	gi|13376533|ref|NP_079288.1|	gi|20881263|ref|XP_126190.1|	gi|6700333|ref|Os01g04180|	gi|34855146|ref|XP_347042.1|	gi|33438768|ref|NP_878050.1|	gi|19075545|ref|NP_588045.1|
gi|21294533|gb|EAA06678.1|	lcl|At1g10155.1	lcl|B0281.1 CE07712 WBGene0	gi|18543241|	gi|13376543|ref|NP_079300.1|	gi|20881493|ref|XP_127874.1|	gi|6700341|ref|Os01g04240|	gi|34855169|ref|XP_346659.1|	gi|33438769|ref|NP_878051.1|	gi|19075549|ref|NP_588049.1|
gi|21294602|gb|EAA06747.1|	lcl|At1g10220.1	lcl|B0284.2 CE00651 WBGene0	gi|18543265|	gi|13376546|ref|NP_079301.1|	gi|20886605|ref|XP_150130.1|	gi|6700342|ref|Os01g04250|	gi|34855758|ref|XP_346983.1|	gi|33438770|ref|NP_878052.1|	gi|19075560|ref|NP_588060.1|
gi|21295079|gb|EAA07224.1|	lcl|At1g10420.1	lcl|B0284.3 CE28525 WBGene0	gi|18543279|	gi|13376548|ref|NP_079302.1|	gi|20889493|ref|XP_127223.1|	gi|6700344|ref|Os01g04270|	gi|34855760|ref|XP_346984.1|	gi|33438771|ref|NP_878053.1|	gi|19075564|ref|NP_588064.1|
gi|21295549|gb|EAA07694.1|	lcl|At1g10455.1	lcl|B0285.3 CE00667 WBGene0	gi|18543289|	gi|13376552|ref|NP_079305.1|	gi|20890236|ref|XP_148917.1|	gi|6700348|ref|Os01g04310|	gi|34855873|ref|XP_347045.1|	gi|33438772|ref|NP_878054.1|	gi|19075571|ref|NP_588071.1|
gi|21296540|gb|EAA08685.1|	lcl|At1g10480.1	lcl|B0286.1 CE30066 WBGene0	gi|18543303|	gi|13376556|ref|NP_079307.1|	gi|20890790|ref|XP_150190.1|	gi|6700349|ref|Os01g04320|	gi|34855890|ref|XP_346698.1|	gi|33438773|ref|NP_878055.1|	gi|19075576|ref|NP_588076.1|
gi|21296735|gb|EAA08880.1|	lcl|At1g10690.1	lcl|B0294.3 CE35707 WBGene0	gi|18543307|	gi|13376558|ref|NP_079308.1|	gi|20892241|ref|XP_148706.1|	gi|6700356|ref|Os01g04390|	gi|34856303|ref|XP_346988.1|	gi|33438774|ref|NP_878056.1|	gi|19075580|ref|NP_588080.1|
gi|21296755|gb|EAA08900.1|	lcl|At1g10710.1	lcl|B0302.1c CE35209 WBGene(	gi|18543309|	gi|13376562|ref|NP_079310.1|	gi|20892247|ref|XP_148040.1|	gi|6700358|ref|Os01g04410|	gi|34856440|ref|XP_346665.1|	gi|33438775|ref|NP_878057.1|	gi|19075582|ref|NP_588082.1|
gi|21296952|gb|EAA09097.1|	lcl|At1g10800.1	lcl|B0302.2 CE03867 WBGene0	gi|18857979|	gi|13376566|ref|NP_079317.1|	gi|20894803|ref|XP_147733.1|	gi|6700359|ref|Os01g04420|	gi|34856567|ref|XP_347034.1|	gi|33438776|ref|NP_878058.1|	gi|19075583|ref|NP_588083.1|
gi|21298324|gb|EAA10469.1|	lcl|At1g10990.1	lcl|B0302.4 CE35210 WBGene0	gi|18858015|	gi|13376580|ref|NP_079327.1|	gi|20895692|ref|XP_147763.1|	gi|6700360|ref|Os01g04430|	gi|34856693|ref|XP_347036.1|	gi|33438777|ref|NP_878059.1|	gi|19075608|ref|NP_588108.1|
gi|21299083|gb|EAA11228.1|	lcl|At1g10990.2	lcl|B0302.5 CE03870 WBGene0	gi|18858029|	gi|13376589|ref|NP_079332.1|	gi|20896247|ref|XP_128328.1|	gi|6700361|ref|Os01g04440|	gi|34856739|ref|XP_346624.1|	gi|33438778|ref|NP_878060.1|	gi|19075613|ref|NP_588113.1|
gi|21299285|gb|EAA11430.1|	lcl|At1g11070.1	lcl|B0303.14 CE00003 WBGene	gi|18858035|	gi|13376615|ref|NP_079347.1|	gi|20899220|ref|XP_148771.1|	gi|6700364|ref|Os01g04470|	gi|34856973|ref|XP_342252.1|	gi|33438779|ref|NP_878061.1|	gi|19075618|ref|NP_588118.1|
gi|21300459|gb|EAA12604.1|	lcl|At1g11120.1	lcl|B0304.2 CE02424 WBGene0	gi|18858047|	gi|13376642|ref|NP_079362.1|	gi|20900556|ref|XP_128699.1|	gi|6700367|ref|Os01g04500|	gi|34857058|ref|XP_347014.1|	gi|33438780|ref|NP_878062.1|	gi|19075620|ref|NP_588120.1|
gi|21300593|gb|EAA12738.1|	lcl|At1g11145.1	lcl|B0304.4 CE03872 WBGene0	gi|18858075|	gi|13376654|ref|NP_079369.1|	gi|20900722|ref|XP_128729.1|	gi|6700368|ref|Os01g04510|	gi|34857064|ref|XP_347015.1|	gi|33438781|ref|NP_878063.1|	gi|19075621|ref|NP_588121.1|
gi|21301145|gb|EAA13290.1|	lcl|At1g11470.1	lcl|B0304.5 CE33508 WBGene0	gi|18858083|	gi|13376667|ref|NP_079376.1|	gi|20901251|ref|XP_148825.1|	gi|6700370|ref|Os01g04530|	gi|34857076|ref|XP_342262.1|	gi|33438782|ref|NP_878064.1|	gi|19075625|ref|NP_588125.1|
gi|21301540|gb|EAA13685.1|	lcl|At1g11655.1	lcl|B0304.6 CE34634 WBGene0	gi|18858085|	gi|13376673|ref|NP_079381.1|	gi|20901299|ref|XP_148821.1|	gi|6700373|ref|Os01g04560|	gi|34857253|ref|XP_346990.1|	gi|33438783|ref|NP_878065.1|	gi|19075630|ref|NP_588130.1|
gi|21301585|gb|EAA13730.1|	lcl|At1g11700.1	lcl|B0304.9 CE33512 WBGene0	gi|18858125|	gi|13376695|ref|NP_079393.1|	gi|20909184|ref|XP_138788.1|	gi|6700379|ref|Os01g04610|	gi|34857807|ref|XP_346994.1|	gi|33438784|ref|NP_878066.1|	gi|19075631|ref|NP_588131.1|
gi|21301847|gb|EAA13992.1|	lcl|At1g11850.1	lcl|B0310.3 CE03876 WBGene0	gi|18858147|	gi|13376697|ref|NP_079394.1|	gi|20909336|ref|XP_127486.1|	gi|6700386|ref|Os01g04670|	gi|34857892|ref|XP_347016.1|	gi|33438785|ref|NP_878067.1|	gi|19075635|ref|NP_588135.1|
gi|21301878|gb|EAA14023.1|	lcl|At1g11850.2	lcl|B0310.6 CE03879 WBGene0	gi|18859647|	gi|13376729|ref|NP_079419.1|	gi|20910029|ref|XP_129392.1|	gi|6700388|ref|Os01g04690|	gi|34858067|ref|XP_347018.1|	gi|33438786|ref|NP_878068.1|	gi|19075637|ref|NP_588137.1|
gi|21302314|gb|EAA14459.1|	lcl|At1g11970.1	lcl|B0334.10 CE19660 WBGene	gi|18859649|	gi|13386482|ref|NP_085049.1|	gi|20984017|ref|XP_135884.1|	gi|6700389|ref|Os01g04700|	gi|34858071|ref|XP_347019.1|	gi|33438787|ref|NP_878069.1|	gi|19075638|ref|NP_588138.1|
gi|21302760|gb|EAA14905.1|	lcl|At1g12080.1	lcl|B0334.11a CE19661 WBGen	gi|18859655|	gi|13386488|ref|NP_085052.1|	gi|20984045|ref|XP_135902.1|	gi|6700396|ref|Os01g04740|	gi|34858119|ref|XP_345231.1|	gi|33438789|ref|NP_878071.1|	gi|19075648|ref|NP_588148.1|
gi|21436527|emb|CAD29631.1|	lcl|At1g12080.2	lcl|B0334.11b CE32788 WBGen	gi|18859669|	gi|13435155|ref|NP_079318.1|	gi|20985049|ref|XP_142014.1|	gi|6700398|ref|Os01g04760|	gi|34858290|ref|XP_346717.1|	gi|33438790|ref|NP_878072.1|	gi|19075650|ref|NP_588150.1|
gi|2731365|gb|AAD12200.1|	lcl|At1g12660.1	lcl|B0334.13 CE34635 WBGene	gi|18859675|	gi|13443002|ref|NP_079360.1|	gi|20985593|ref|XP_136059.1|	gi|6700406|ref|Os01g04830|	gi|34858294|ref|XP_346715.1|	gi|33438791|ref|NP_878073.1|	gi|19075659|ref|NP_588159.1|
gi|28396160|gb|AAO39757.1|	lcl|At1g12670.1	lcl|B0336.12 CE00780 WBGene	gi|18859681|	gi|13540608|ref|NP_110441.1|	gi|20986026|ref|XP_136127.1|	gi|6700424|ref|Os01g04970|	gi|34858521|ref|XP_347055.1|	gi|33438792|ref|NP_878074.1|	gi|19075663|ref|NP_588163.1|
gi|28396162|gb|AAO39758.1|	lcl|At1g12725.1	lcl|B0336.9c CE31144 WBGene(	gi|18859715|	gi|13562106|ref|NP_110519.1|	gi|20986256|ref|XP_150444.1|	gi|6700427|ref|Os01g05000|	gi|34858523|ref|XP_238384.2|	gi|33438793|ref|NP_878075.1|	gi|19075664|ref|NP_588164.1|
gi|28569872|dbj|BAC57911.1|	lcl|At1g12810.1	lcl|B0336.9e CE31146 WBGene	gi|18859717|	gi|13569843|ref|NP_057251.2|	gi|21311889|ref|NP_080882.1|	gi|6700437|ref|Os01g05100|	gi|34858525|ref|XP_347056.1|	gi|33438794|ref|NP_878076.1|	gi|19075673|ref|NP_588173.1|
gi|2997643|gb|AAC08530.1|	lcl|At1g12830.1	lcl|B0344.2 CE33963 WBGene0	gi|18859725|	gi|13569917|ref|NP_112206.1|	gi|21311955|ref|NP_083071.1|	gi|6700441|ref|Os01g05140|	gi|34858635|ref|XP_346668.1|	gi|33438795|ref|NP_878077.1|	gi|19075674|ref|NP_588174.1|
gi|30173553|gb|EAA43052.1|	lcl|At1g12845.1	lcl|B0348.5 CE17330 WBGene0	gi|18859745|	gi|13654286|ref|NP_112491.1|	gi|21311957|ref|NP_083074.1|	gi|6700442|ref|Os01g05150|	gi|34858938|ref|XP_347037.1|	gi|33438796|ref|NP_878078.1|	gi|19075675|ref|NP_588175.1|
gi|30173554|gb|EAA43053.1|	lcl|At1g13245.1	lcl|B0350.3 CE06706 WBGene0	gi|18859747|	gi|13775164|ref|NP_112232.1|	gi|21312191|ref|NP_082620.1|	gi|6700443|ref|Os01g05160|	gi|34859136|ref|XP_345429.1|	gi|33438797|ref|NP_878079.1|	gi|19075676|ref|NP_588176.1|
gi|30173563|gb|EAA43059.1|	lcl|At1g13310.1	lcl|B0353.1 CE27571 WBGene0	gi|18859755|	gi|13775196|ref|NP_112575.1|	gi|21312476|ref|NP_081541.1|	gi|6700444|ref|Os01g05170|	gi|34859352|ref|XP_345449.1|	gi|33438798|ref|NP_878080.1|	gi|19075677|ref|NP_588177.1|
gi|30173580|gb|EAA43065.1|	lcl|At1g13360.1	lcl|B0361.3 CE34242 WBGene0	gi|18859763|	gi|13899223|ref|NP_113604.1|	gi|21312716|ref|NP_083187.1|	gi|6700445|ref|Os01g05180|	gi|34859752|ref|XP_347020.1|	gi|33438799|ref|NP_878081.1|	gi|19075678|ref|NP_588178.1|
gi|30173588|gb|EAA01659.2|	lcl|At1g13390.1	lcl|B0361.4 CE00751 WBGene0	gi|18859865|	gi|13994368|ref|NP_114168.1|	gi|21312904|ref|NP_080521.1|	gi|6700446|ref|Os01g05190|	gi|34859859|ref|XP_347022.1|	gi|33438800|ref|NP_878082.1|	gi|19075679|ref|NP_588179.1|
gi|30173597|gb|EAA01561.2|	lcl|At1g13390.2	lcl|B0379.1 CE20452 WBGene0	gi|18859867|	gi|13994372|ref|NP_114170.1|	gi|21312942|ref|NP_081996.1|	gi|6700448|ref|Os01g05210|	gi|34859947|ref|XP_347023.1|	gi|33438801|ref|NP_878083.1|	gi|19075682|ref|NP_588182.1|
gi|30173750|gb|EAA43143.1|	lcl|At1g13620.1	lcl|B0379.2 CE07725 WBGene0	gi|18859881|	gi|14042933|ref|NP_114410.1|	gi|21313056|ref|NP_080321.1|	gi|6700449|ref|Os01g05220|	gi|34860005|ref|XP_344950.1|	gi|33438802|ref|NP_878084.1|	gi|19075687|ref|NP_588187.1|
gi|30173751|gb|EAA43144.1|	lcl|At1g13670.1	lcl|B0379.3a CE30474 WBGene	gi|18859887|	gi|14042962|ref|NP_114424.1|	gi|21313126|ref|NP_083807.1|	gi|6700450|ref|Os01g05230|	gi|34860051|ref|XP_219386.2|	gi|33438803|ref|NP_878085.1|	gi|19075696|ref|NP_588196.1|
gi|30173779|gb|EAA43159.1|	lcl|At1g14180.1	lcl|B0379.3b CE30475 WBGene	gi|18859931|	gi|14149651|ref|NP_055226.1|	gi|21313178|ref|NP_083863.1|	gi|6700451|ref|Os01g05240|	gi|34860246|ref|XP_345290.1|	gi|33438804|ref|NP_878086.1|	gi|19075699|ref|NP_588199.1|
gi|30173780|gb|EAA43160.1|	lcl|At1g14280.1	lcl|B0379.6 CE07729 WBGene0	gi|18859939|	gi|14149708|ref|NP_056436.1|	gi|21313350|ref|NP_082072.1|	gi|6700452|ref|Os01g05250|	gi|34860300|ref|XP_347039.1|	gi|33438805|ref|NP_878087.1|	gi|19075700|ref|NP_588200.1|
gi|30173781|gb|EAA43161.1|	lcl|At1g14760.1	lcl|B0379.7 CE23508 WBGene0	gi|18859947|	gi|14149833|ref|NP_115531.1|	gi|21313352|ref|NP_080005.1|	gi|6700453|ref|Os01g05260|	gi|34860370|ref|XP_347096.1|	gi|33438806|ref|NP_878088.1|	gi|19075710|ref|NP_588210.1|
gi|30173783|gb|EAA43162.1|	lcl|At1g15190.1	lcl|B0391.10 CE17338 WBGene	gi|18859977|	gi|14149906|ref|NP_115585.1|	gi|21313546|ref|NP_079748.1|	gi|6700455|ref|Os01g05280|	gi|34860675|ref|XP_342574.1|	gi|33438807|ref|NP_878089.1|	gi|19075717|ref|NP_588217.1|
gi|30173793|gb|EAA01366.2|	lcl|At1g15260.1	lcl|B0393.4 CE00857 WBGene0	gi|18859979|	gi|14149948|ref|NP_115614.1|	gi|21313656|ref|NP_082121.1|	gi|6700456|ref|Os01g05290|	gi|34860755|ref|XP_230851.2|	gi|33438808|ref|NP_878090.1|	gi|19075721|ref|NP_588221.1|
gi|30173818|gb|EAA43179.1|	lcl|At1g15320.1	lcl|B0393.8 CE32101 WBGene0	gi|18860017|	gi|14149956|ref|NP_115618.1|	gi|21313670|ref|NP_082199.1|	gi|6700457|ref|Os01g05300|	gi|34860967|ref|XP_347025.1|	gi|33438809|ref|NP_878091.1|	gi|19075728|ref|NP_588228.1|
gi|30173830|gb|EAA43184.1|	lcl|At1g15385.1	lcl|B0403.1 CE03881 WBGene0	gi|18860021|	gi|14149960|ref|NP_115621.1|	gi|21357427|ref|NP_570940.1|	gi|6700458|ref|Os01g05310|	gi|34861180|ref|XP_346682.1|	gi|33438810|ref|NP_878092.1|	gi|19075731|ref|NP_588231.1|
gi|30173868|gb|EAA43203.1|	lcl|At1g15415.1	lcl|B0403.3 CE03883 WBGene0	gi|18860093|	gi|14149964|ref|NP_115623.1|	gi|21362297|ref|NP_653096.1|	gi|6700461|ref|Os01g05340|	gi|34862010|ref|XP_347075.1|	gi|33438811|ref|NP_878093.1|	gi|19075745|ref|NP_588245.1|
gi|30173872|gb|EAA43207.1|	lcl|At1g15600.1	lcl|B0403.5 CE36085 WBGene0	gi|18921205|	gi|14149977|ref|NP_115630.1|	gi|21362367|ref|NP_653138.1|	gi|6700462|ref|Os01g05350|	gi|34862341|ref|XP_238484.2|	gi|33438812|ref|NP_878094.1|	gi|19075754|ref|NP_588254.1|
gi|30173876|gb|EAA43210.1|	lcl|At1g15630.1	lcl|B0403.6 CE37603 WBGene0	gi|19584583|	gi|14149995|ref|NP_115638.1|	gi|21426859|ref|NP_570927.1|	gi|6700463|ref|Os01g05360|	gi|34862379|ref|XP_347082.1|	gi|33438813|ref|NP_878095.1|	gi|19075757|ref|NP_588257.1|
gi|30173902|gb|EAA43219.1|	lcl|At1g15800.1	lcl|B0410.1 CE06707 WBGene0	gi|19920368|	gi|14150031|ref|NP_115663.1|	gi|21426861|ref|NP_570942.1|	gi|6700465|ref|Os01g05380|	gi|34862408|ref|XP_347083.1|	gi|33438814|ref|NP_878096.1|	gi|19075772|ref|NP_588272.1|
gi|30173954|gb|EAA43246.1|	lcl|At1g15825.1	lcl|B0410.3 CE28814 WBGene0	gi|19920378|	gi|14150052|ref|NP_115673.1|	gi|21426873|ref|NP_570943.1|	gi|6700466|ref|Os01g05390|	gi|34862575|ref|XP_346582.1|	gi|33438815|ref|NP_878097.1|	gi|19075776|ref|NP_588276.1|
gi|30173956|gb|EAA43248.1|	lcl|At1g15830.1	lcl|B0416.3 CE02433 WBGene0	gi|19920396|	gi|14150058|ref|NP_115677.1|	gi|21426875|ref|NP_570946.1|	gi|6700468|ref|Os01g05410|	gi|34862726|ref|XP_346999.1|	gi|33438816|ref|NP_878098.1|	gi|19075779|ref|NP_588279.1|
gi|30173957|gb|EAA43249.1|	lcl|At1g15840.1	lcl|B0416.7 CE02437 WBGene0	gi|19920424|	gi|14150227|ref|NP_115767.1|	gi|21492626|ref|NP_659569.1|	gi|6700472|ref|Os01g05450|	gi|34862994|ref|XP_347099.1|	gi|33438817|ref|NP_878099.1|	gi|19075780|ref|NP_588280.1|
gi|30173958|gb|EAA43250.1|	lcl|At1g16025.1	lcl|B0432.10 CE07748 WBGene	gi|19920432|	gi|14165278|ref|NP_115788.1|	gi|21536218|ref|NP_079696.1|	gi|6700473|ref|Os01g05460|	gi|34863054|ref|XP_343372.1|	gi|33438818|ref|NP_878100.1|	gi|19075781|ref|NP_588281.1|
gi|30173960|gb|EAA43252.1|	lcl|At1g16500.1	lcl|B0432.11 CE07749 WBGene	gi|19920492|	gi|14165280|ref|NP_115789.1|	gi|21539595|ref|NP_079777.1|	gi|6700479|ref|Os01g05510|	gi|34863058|ref|XP_347000.1|	gi|33438819|ref|NP_878101.1|	gi|19075790|ref|NP_588290.1|
gi|30173961|gb|EAA43253.1|	lcl|At1g16730.1	lcl|B0432.3 CE07741 WBGene0	gi|19920520|	gi|14192931|ref|NP_112160.1|	gi|21539629|ref|NP_082898.1|	gi|6700480|ref|Os01g05520|	gi|34863142|ref|XP_347101.1|	gi|33438820|ref|NP_878102.1|	gi|19075792|ref|NP_588292.1|
gi|30173963|gb|EAA43254.1|	lcl|At1g16790.1	lcl|B0432.6 CE34636 WBGene0	gi|19920552|	gi|14210498|ref|NP_115879.1|	gi|21539645|ref|NP_076356.1|	gi|6700484|ref|Os01g05550|	gi|34864216|ref|XP_345043.1|	gi|33438821|ref|NP_878103.1|	gi|19075793|ref|NP_588293.1|
gi|30173974|gb|EAA43258.1|	lcl|At1g16850.1	lcl|B0432.7 CE07745 WBGene0	gi|19920576|	gi|14210534|ref|NP_115913.1|	gi|21729763|ref|NP_081352.1|	gi|6700485|ref|Os01g05560|	gi|34864370|ref|XP_238145.2|	gi|33438822|ref|NP_878104.1|	gi|19075795|ref|NP_588295.1|
gi|30173976|gb|EAA43260.1|	lcl|At1g16950.1	lcl|B0457.2 CE32790 WBGene0	gi|19920594|	gi|14211869|ref|NP_115952.1|	gi|21729769|ref|NP_083072.1|	gi|6700486|ref|Os01g05570|	gi|34864831|ref|XP_217647.2|	gi|33438823|ref|NP_878105.1|	gi|19075797|ref|NP_588297.1|
gi|30173977|gb|EAA43261.1|	lcl|At1g17090.1	lcl|B0457.4 CE02948 WBGene0	gi|19920604|	gi|14211881|ref|NP_115959.1|	gi|21729782|ref|NP_083866.1|	gi|6700487|ref|Os01g05580|	gi|34864939|ref|XP_345059.1|	gi|33438824|ref|NP_878106.1|	gi|19075799|ref|NP_588299.1|
gi|30173978|gb|EAA43262.1|	lcl|At1g17300.1	lcl|B0457.6 CE34638 WBGene0	gi|19920608|	gi|14211917|ref|NP_115978.1|	gi|21735457|ref|NP_081867.1|	gi|6700506|ref|Os01g05770|	gi|34864949|ref|XP_345061.1|	gi|33438826|ref|NP_878108.1|	gi|19075801|ref|NP_588301.1|

gi|30173979|gb|EAA43263.1|	lcl|At1g17400.1	lcl|B0462.4 CE34639 WBGene0	gi|19920692|	gi|14249146|ref|NP_116014.1|	gi|22122581|ref|NP_666192.1|	gi|6700515|ref|Os01g05850|	gi|34865405|ref|XP_347384.1|	gi|33438827|ref|NP_878115.1|	gi|19075804|ref|NP_588304.1|
gi|30173980|gb|EAA43264.1|	lcl|At1g17510.1	lcl|B0464.3 CE00017 WBGene0	gi|19920774|	gi|14249184|ref|NP_116033.1|	gi|25020122|ref|XP_205860.1|	gi|6700521|ref|Os01g05910|	gi|34865432|ref|XP_347077.1|	gi|33438828|ref|NP_878116.1|	gi|19075817|ref|NP_588317.1|
gi|30173981|gb|EAA43265.1|	lcl|At1g17780.1	lcl|B0491.5 CE02107 WBGene0	gi|19920802|	gi|14249192|ref|NP_116037.1|	gi|25022417|ref|XP_207086.1|	gi|6700522|ref|Os01g05920|	gi|34865506|ref|XP_236440.2|	gi|33438830|ref|NP_878118.1|	gi|19075819|ref|NP_588319.1|
gi|30173982|gb|EAA43266.1|	lcl|At1g17900.1	lcl|B0491.6 CE02108 WBGene0	gi|19920870|	gi|14249202|ref|NP_116042.1|	gi|25032796|ref|XP_195432.1|	gi|6700534|ref|Os01g06040|	gi|34865512|ref|XP_345961.1|	gi|33438836|ref|NP_878124.1|	gi|19075825|ref|NP_588325.1|
gi|30173983|gb|EAA43267.1|	lcl|At1g18220.1	lcl|B0495.9 CE01767 WBGene0	gi|19920974|	gi|14249212|ref|NP_116047.1|	gi|25033006|ref|XP_196132.1|	gi|6700542|ref|Os01g06120|	gi|34865601|ref|XP_236497.2|	gi|33438837|ref|NP_878125.1|	gi|19075841|ref|NP_588341.1|
gi|30173984|gb|EAA43268.1|	lcl|At1g18290.1	lcl|B0496.3c CE37179 WBGene	gi|19921040|	gi|14249218|ref|NP_116050.1|	gi|25047711|ref|XP_196251.1|	gi|6700563|ref|Os01g06310|	gi|34865661|ref|XP_345496.1|	gi|33438841|ref|NP_878129.1|	gi|19075842|ref|NP_588342.1|
gi|30173985|gb|EAA43269.1|	lcl|At1g18380.1	lcl|B0496.4 CE06716 WBGene0	gi|19921106|	gi|14249266|ref|NP_116075.1|	gi|25049719|ref|XP_196362.1|	gi|6700565|ref|Os01g06330|	gi|34866056|ref|XP_342796.1|	gi|33438842|ref|NP_878130.1|	gi|19075855|ref|NP_588355.1|
gi|30174120|gb|EAA43308.1|	lcl|At1g18510.1	lcl|B0496.6 CE06718 WBGene0	gi|19921110|	gi|14249274|ref|NP_116079.1|	gi|25051959|ref|XP_196011.1|	gi|6700570|ref|Os01g06380|	gi|34866199|ref|XP_243273.2|	gi|33438843|ref|NP_878131.1|	gi|19075858|ref|NP_588358.1|
gi|30174121|gb|EAA43309.1|	lcl|At1g18620.1	lcl|B0507.10 CE28875 WBGene	gi|19921148|	gi|14249302|ref|NP_116094.1|	gi|25052316|ref|XP_196072.1|	gi|6700572|ref|Os01g06400|	gi|34866681|ref|XP_346001.1|	gi|33438844|ref|NP_878132.1|	gi|19075863|ref|NP_588363.1|
gi|30174124|gb|EAA43311.1|	lcl|At1g18810.1	lcl|B0507.3 CE07762 WBGene0	gi|19921152|	gi|14249310|ref|NP_116099.1|	gi|25070249|ref|XP_196141.1|	gi|6700574|ref|Os01g06420|	gi|34866920|ref|XP_347089.1|	gi|33438845|ref|NP_878133.1|	gi|19075865|ref|NP_588365.1|
gi|30174125|gb|EAA43312.1|	lcl|At1g19010.1	lcl|B0507.5 CE07764 WBGene0	gi|19921162|	gi|14249312|ref|NP_116100.1|	gi|25072211|ref|XP_196060.1|	gi|6700575|ref|Os01g06430|	gi|34867096|ref|XP_347060.1|	gi|33438846|ref|NP_878134.1|	gi|19075866|ref|NP_588366.1|
gi|30174138|gb|EAA43315.1|	lcl|At1g19010.2	lcl|B0507.7 CE07766 WBGene0	gi|19921264|	gi|14249384|ref|NP_116140.1|	gi|27229070|ref|NP_081032.1|	gi|6700576|ref|Os01g06440|	gi|34867102|ref|XP_342815.1|	gi|33438847|ref|NP_878135.1|	gi|19075867|ref|NP_588367.1|
gi|30174199|gb|EAA43332.1|	lcl|At1g19020.1	lcl|B0507.8 CE28876 WBGene0	gi|19921306|	gi|14249404|ref|NP_116150.1|	gi|27369573|ref|NP_766020.1|	gi|6700589|ref|Os01g06570|	gi|34867132|ref|XP_342819.1|	gi|33438848|ref|NP_878136.1|	gi|19075875|ref|NP_588375.1|
gi|30174214|gb|EAA43338.1|	lcl|At1g19040.1	lcl|B0507.9 CE07768 WBGene0	gi|19921326|	gi|14249406|ref|NP_116151.1|	gi|27369623|ref|NP_766045.1|	gi|6700593|ref|Os01g06610|	gi|34867326|ref|XP_346781.1|	gi|33438849|ref|NP_878137.1|	gi|19075899|ref|NP_588399.1|
gi|30174220|gb|EAA43343.1|	lcl|At1g19240.1	lcl|B0511.11 CE26852 WBGene	gi|19921340|	gi|14249466|ref|NP_116182.1|	gi|27734056|ref|NP_775543.1|	gi|6700594|ref|Os01g06620|	gi|34867367|ref|XP_347078.1|	gi|33438850|ref|NP_878138.1|	gi|19075900|ref|NP_588400.1|
gi|30174222|gb|EAA43344.1|	lcl|At1g19500.1	lcl|B0511.2 CE17341 WBGene0	gi|19921344|	gi|14249512|ref|NP_116206.1|	gi|27734084|ref|NP_775588.1|	gi|6700597|ref|Os01g06650|	gi|34867476|ref|XP_346810.1|	gi|33438851|ref|NP_878139.1|	gi|19075902|ref|NP_588402.1|
gi|30174228|gb|EAA43346.1|	lcl|At1g19530.1	lcl|B0511.9a CE26854 WBGene	gi|19921370|	gi|14249564|ref|NP_116234.1|	gi|28202003|ref|NP_780418.1|	gi|6700601|ref|Os01g06690|	gi|34867636|ref|XP_346911.1|	gi|33438852|ref|NP_878140.1|	gi|19075904|ref|NP_588404.1|
gi|30174233|gb|EAA43347.1|	lcl|At1g19620.1	lcl|B0511.9b CE17348 WBGene	gi|19921372|	gi|14249642|ref|NP_116275.1|	gi|28202009|ref|NP_780436.1|	gi|6700603|ref|Os01g06710|	gi|34867659|ref|XP_347091.1|	gi|33438853|ref|NP_878141.1|	gi|19075912|ref|NP_588412.1|
gi|30174321|gb|EAA14944.2|	lcl|At1g19960.1	lcl|B0513.1 CE15557 WBGene0	gi|19921378|	gi|14249644|ref|NP_116276.1|	gi|28316778|ref|NP_783610.1|	gi|6700610|ref|Os01g06780|	gi|34867739|ref|XP_347079.1|	gi|33438854|ref|NP_878142.1|	gi|19075918|ref|NP_588418.1|
gi|30174341|gb|EAA15084.2|	lcl|At1g20070.1	lcl|B0513.4 CE15560 WBGene0	gi|19921404|	gi|14249658|ref|NP_116284.1|	gi|28316800|ref|NP_783623.1|	gi|6700612|ref|Os01g06800|	gi|34867785|ref|XP_238474.2|	gi|33438855|ref|NP_878143.1|	gi|19075928|ref|NP_588428.1|
gi|30174352|gb|EAA43398.1|	lcl|At1g20100.1	lcl|B0523.3 CE20462 WBGene0	gi|19921414|	gi|14249662|ref|NP_116286.1|	gi|28316802|ref|NP_783624.1|	gi|6700615|ref|Os01g06830|	gi|34867815|ref|XP_238472.2|	gi|33438856|ref|NP_878144.1|	gi|19075939|ref|NP_588439.1|
gi|30174405|gb|EAA43429.1|	lcl|At1g20280.1	lcl|B0524.1 CE20463 WBGene0	gi|19921418|	gi|14249674|ref|NP_116292.1|	gi|28477140|ref|XP_283186.1|	gi|6700617|ref|Os01g06850|	gi|34867934|ref|XP_346786.1|	gi|33438858|ref|NP_878146.1|	gi|19075956|ref|NP_588456.1|
gi|30174406|gb|EAA43430.1|	lcl|At1g20310.1	lcl|B0524.3 CE33914 WBGene0	gi|19921426|	gi|14249688|ref|NP_116299.1|	gi|28477764|ref|XP_283202.1|	gi|6700623|ref|Os01g06910|	gi|34868162|ref|XP_347093.1|	gi|33438859|ref|NP_878147.1|	gi|19075961|ref|NP_588461.1|
gi|30174407|gb|EAA43431.1|	lcl|At1g20430.1	lcl|B0524.5 CE20467 WBGene0	gi|19921432|	gi|14249700|ref|NP_116305.1|	gi|28478069|ref|XP_286602.1|	gi|6700625|ref|Os01g06930|	gi|34868270|ref|XP_345873.1|	gi|33438860|ref|NP_878148.1|	gi|19075974|ref|NP_588474.1|
gi|30174408|gb|EAA43432.1|	lcl|At1g20440.1	lcl|B0547.2 CE07771 WBGene0	gi|19921446|	gi|14249706|ref|NP_116309.1|	gi|28479398|ref|XP_287006.1|	gi|6700627|ref|Os01g06950|	gi|34868328|ref|XP_346739.1|	gi|33438861|ref|NP_878149.1|	gi|19075984|ref|NP_588484.1|
gi|30174409|gb|EAA43433.1|	lcl|At1g20460.1	lcl|B0554.1 CE07773 WBGene0	gi|19921554|	gi|14249722|ref|NP_116317.1|	gi|28479748|ref|XP_286615.1|	gi|6700629|ref|Os01g06970|	gi|34868722|ref|XP_346883.1|	gi|33438862|ref|NP_878150.1|	gi|19075986|ref|NP_588486.1|
gi|30174414|gb|EAA43436.1|	lcl|At1g20690.1	lcl|B0554.3 CE07775 WBGene0	gi|19921570|	gi|14269578|ref|NP_116324.1|	gi|28482273|ref|XP_287042.1|	gi|6700655|ref|Os01g07190|	gi|34868734|ref|XP_346884.1|	gi|33438863|ref|NP_878151.1|	gi|19075989|ref|NP_588489.1|
gi|30174468|gb|EAA43458.1|	lcl|At1g20730.1	lcl|B0563.1 CE02439 WBGene0	gi|19921586|	gi|14269584|ref|NP_116326.1|	gi|28484299|ref|XP_287801.1|	gi|6700663|ref|Os01g07270|	gi|34868851|ref|XP_345889.1|	gi|33438864|ref|NP_878152.1|	gi|19076011|ref|NP_588512.1|
gi|30174476|gb|EAA43462.1|	lcl|At1g20740.1	lcl|B0563.5 CE02443 WBGene0	gi|19921590|	gi|14670379|ref|NP_116783.1|	gi|28484584|ref|XP_284420.1|	gi|6700668|ref|Os01g07320|	gi|34868872|ref|XP_347094.1|	gi|33438865|ref|NP_878153.1|	gi|19076033|ref|NP_588533.1|
gi|30174491|gb|EAA14514.2|	lcl|At1g21020.1	lcl|B0563.8 CE02446 WBGene0	gi|19921722|	gi|14861858|ref|NP_149060.1|	gi|28484903|ref|XP_287099.1|	gi|6700669|ref|Os01g07330|	gi|34868991|ref|XP_233051.2|	gi|33438866|ref|NP_878154.1|	gi|19076034|ref|NP_588534.1|
gi|30174503|gb|EAA43468.1|	lcl|At1g21220.1	lcl|B0564.7 CE36971 WBGene0	gi|19921798|	gi|15029524|ref|NP_056458.1|	gi|28486726|ref|XP_286688.1|	gi|6700682|ref|Os01g07460|	gi|34869015|ref|XP_347062.1|	gi|33438867|ref|NP_878155.1|	gi|19076038|ref|NP_588538.1|
gi|30174549|gb|EAA43498.1|	lcl|At1g21280.1	lcl|BE10.1 CE17358 WBGene00	gi|19921842|	gi|15082222|ref|NP_149049.1|	gi|28486771|ref|XP_286697.1|	gi|6700683|ref|Os01g07470|	gi|34869090|ref|XP_346419.1|	gi|33438868|ref|NP_878156.1|	gi|19076044|ref|NP_588544.1|
gi|30174554|gb|EAA43501.1|	lcl|At1g21290.1	lcl|BE10.4 CE15567 WBGene00	gi|19921844|	gi|15082254|ref|NP_149986.1|	gi|28486841|ref|XP_286696.1|	gi|6700687|ref|Os01g07510|	gi|34869291|ref|XP_346912.1|	gi|33438869|ref|NP_878157.1|	gi|19076045|ref|NP_588545.1|
gi|30174596|gb|EAA43519.1|	lcl|At1g21310.1	lcl|C01A2.1 CE07783 WBGene0	gi|19921850|	gi|15187164|ref|NP_150593.1|	gi|28487466|ref|XP_287155.1|	gi|6700691|ref|Os01g07550|	gi|34869373|ref|XP_344025.1|	gi|33438871|ref|NP_878159.1|	gi|19076067|ref|NP_588567.1|
gi|30174597|gb|EAA43520.1|	lcl|At1g21323.1	lcl|C01A2.2 CE07784 WBGene0	gi|19921858|	gi|15619001|ref|NP_203695.1|	gi|28491755|ref|XP_287254.1|	gi|6700698|ref|Os01g07620|	gi|34869636|ref|XP_237832.2|	gi|33438873|ref|NP_878161.1|	gi|19076071|ref|NP_588571.1|
gi|30174625|gb|EAA43542.1|	lcl|At1g21390.1	lcl|C01A2.6 CE07788 WBGene0	gi|19921876|	gi|15619003|ref|NP_203696.1|	gi|28492788|ref|XP_148381.2|	gi|6700702|ref|Os01g07650|	gi|34870602|ref|XP_237761.2|	gi|33438874|ref|NP_878162.1|	gi|19076072|ref|NP_588572.1|
gi|30174626|gb|EAA43543.1|	lcl|At1g21475.1	lcl|C01A2.7 CE30236 WBGene0	gi|19921932|	gi|16118229|ref|NP_149461.2|	gi|28493815|ref|XP_286747.1|	gi|6700703|ref|Os01g07660|	gi|34870630|ref|XP_346885.1|	gi|33438875|ref|NP_878163.1|	gi|19111846|ref|NP_595054.1|
gi|30174627|gb|EAA43544.1|	lcl|At1g21510.1	lcl|C01B10.5b CE26856 WBGen	gi|19922016|	gi|16118240|ref|NP_114167.2|	gi|28494056|ref|XP_203243.2|	gi|6700704|ref|Os01g07670|	gi|34870709|ref|XP_346887.1|	gi|33438876|ref|NP_878164.1|	gi|19111864|ref|NP_595072.1|
gi|30174629|gb|EAA43546.1|	lcl|At1g21520.1	lcl|C01B10.5c CE26857 WBGen	gi|19922020|	gi|16306527|ref|NP_391991.1|	gi|28495241|ref|XP_137059.3|	gi|6700705|ref|Os01g07680|	gi|34870719|ref|XP_346888.1|	gi|33438877|ref|NP_878165.1|	gi|19111876|ref|NP_595084.1|
gi|30174631|gb|EAA43548.1|	lcl|At1g21580.1	lcl|C01B10.5e CE33357 WBGen	gi|19922156|	gi|16418363|ref|NP_443093.1|	gi|28495330|ref|XP_282906.1|	gi|6700708|ref|Os01g07710|	gi|34870843|ref|XP_237773.2|	gi|33438878|ref|NP_878166.1|	gi|19111879|ref|NP_595087.1|
gi|30174633|gb|EAA14463.2|	lcl|At1g21695.1	lcl|C01B10.6a CE29552 WBGen	gi|19922180|	gi|16418367|ref|NP_443095.1|	gi|28498447|ref|XP_287392.1|	gi|6700731|ref|Os01g07890|	gi|34870870|ref|XP_346892.1|	gi|33438879|ref|NP_878167.1|	gi|19111880|ref|NP_595088.1|
gi|30174642|gb|EAA43555.1|	lcl|At1g21830.1	lcl|C01B10.6b CE31149 WBGen	gi|19922182|	gi|16418373|ref|NP_443103.1|	gi|28512103|ref|XP_284085.1|	gi|6700732|ref|Os01g07900|	gi|34870901|ref|XP_238409.2|	gi|33438880|ref|NP_878168.1|	gi|19111888|ref|NP_595096.1|
gi|30174742|gb|EAA43598.1|	lcl|At1g21940.1	lcl|C01B10.7 CE06738 WBGene	gi|19922208|	gi|16418443|ref|NP_443184.1|	gi|28514434|ref|XP_286372.1|	gi|6700749|ref|Os01g08030|	gi|34871341|ref|XP_343890.1|	gi|33438891|ref|NP_878179.1|	gi|19111895|ref|NP_595103.1|
gi|30174784|gb|EAA43611.1|	lcl|At1g21950.1	lcl|C01B12.4 CE34642 WBGene	gi|19922248|	gi|16506301|ref|NP_443726.1|	gi|28521646|ref|XP_286430.1|	gi|6700751|ref|Os01g08050|	gi|34871445|ref|XP_347065.1|	gi|33438892|ref|NP_878180.1|	gi|19111903|ref|NP_595111.1|
gi|30174901|gb|EAA13608.2|	lcl|At1g22010.1	lcl|C01B12.7 CE07795 WBGene	gi|19922260|	gi|16596690|ref|NP_444276.1|	gi|28524582|ref|XP_148862.2|	gi|6700771|ref|Os01g08240|	gi|34871710|ref|XP_346918.1|	gi|33438893|ref|NP_878181.1|	gi|19111934|ref|NP_595142.1|
gi|30174945|gb|EAA13607.2|	lcl|At1g22110.1	lcl|C01B12.8 CE07796 WBGene	gi|19922272|	gi|16751921|ref|NP_444513.1|	gi|28526367|ref|XP_147774.2|	gi|6700779|ref|Os01g08300|	gi|34871728|ref|XP_346920.1|	gi|33438894|ref|NP_878182.1|	gi|19111936|ref|NP_595144.1|
gi|30174950|gb|EAA43692.1|	lcl|At1g22120.1	lcl|C01B7.3 CE06727 WBGene0	gi|19922300|	gi|16876437|ref|NP_473358.1|	gi|28526553|ref|XP_287648.1|	gi|6700785|ref|Os01g08360|	gi|34871759|ref|XP_347066.1|	gi|33438895|ref|NP_878183.1|	gi|19111942|ref|NP_595150.1|
gi|30174951|gb|EAA43693.1|	lcl|At1g22250.1	lcl|C01C10.2a CE02448 WBGer	gi|19922308|	gi|16876449|ref|NP_473370.1|	gi|28526729|ref|XP_132905.2|	gi|6700788|ref|Os01g08390|	gi|34871860|ref|XP_347067.1|	gi|33438896|ref|NP_878184.1|	gi|19111944|ref|NP_595152.1|
gi|30174961|gb|EAA43698.1|	lcl|At1g22335.1	lcl|C01C10.2b CE37077 WBGer	gi|19922428|	gi|17136145|ref|NP_478106.1|	gi|28526871|ref|XP_286553.1|	gi|6700790|ref|Os01g08410|	gi|34872096|ref|XP_346894.1|	gi|33578046|ref|NP_878109.1|	gi|19111946|ref|NP_595154.1|
gi|30175042|gb|EAA13254.2|	lcl|At1g22420.1	lcl|C01C4.1 CE29554 WBGene(	gi|19922472|	gi|17158005|ref|NP_477521.1|	gi|28527655|ref|XP_141598.2|	gi|6700798|ref|Os01g08490|	gi|34872199|ref|XP_347069.1|	gi|33578047|ref|NP_878110.1|	gi|19111948|ref|NP_595156.1|
gi|30175065|gb|EAA43748.1|	lcl|At1g22470.1	lcl|C01C4.2 CE03888 WBGene(	gi|19922572|	gi|17158013|ref|NP_478064.1|	gi|28528695|ref|XP_288109.1|	gi|6700802|ref|Os01g08530|	gi|34872481|ref|XP_347070.1|	gi|33578048|ref|NP_878111.1|	gi|19111949|ref|NP_595157.1|
gi|30175075|gb|EAA43753.1|	lcl|At1g22590.1	lcl|C01F1.5 CE06746 WBGene0	gi|19922640|	gi|17158019|ref|NP_478068.1|	gi|28544736|ref|XP_287637.1|	gi|6700808|ref|Os01g08590|	gi|34872710|ref|XP_347071.1|	gi|37362611|ref|NP_009391.2|	gi|19111960|ref|NP_595168.1|
gi|30175257|gb|EAA12647.2|	lcl|At1g22680.1	lcl|C01F4.1 CE07797 WBGene0	gi|19922676|	gi|17158021|ref|NP_478069.1|	gi|28546148|ref|XP_287378.1|	gi|6700809|ref|Os01g08600|	gi|34872777|ref|XP_347072.1|	gi|37362619|ref|NP_009653.2|	gi|19111964|ref|NP_595172.1|
gi|30175286|gb|EAA43843.1|	lcl|At1g22885.1	lcl|C01F6.2 CE26968 WBGene0	gi|19922686|	gi|17196629|ref|NP_477516.1|	gi|28549010|ref|XP_287971.1|	gi|6700811|ref|Os01g08620|	gi|34873015|ref|XP_346897.1|	gi|37362620|ref|NP_009680.2|	gi|19111966|ref|NP_595174.1|
gi|30175295|gb|EAA43848.1|	lcl|At1g22890.1	lcl|C01G10.13 CE07822 WBGer	gi|19922692|	gi|17435004|ref|XP_065316.1|	gi|28551351|ref|XP_286926.1|	gi|6700812|ref|Os01g08630|	gi|34873017|ref|XP_347073.1|	gi|37362626|ref|NP_009924.3|	gi|19111974|ref|NP_595182.1|
gi|30175316|gb|EAA12603.2|	lcl|At1g23040.1	lcl|C01G10.15 CE34643 WBGer	gi|19922694|	gi|17437351|ref|XP_059396.1|	gi|28892673|ref|NP_795871.1|	gi|6700813|ref|Os01g08640|	gi|34873364|ref|XP_347383.1|	gi|37362629|ref|NP_010142.2|	gi|19111976|ref|NP_595184.1|
gi|30175425|gb|EAA43910.1|	lcl|At1g23050.1	lcl|C01G10.16 CE34644 WBGer	gi|19922758|	gi|17443793|ref|XP_060509.1|	gi|28892685|ref|NP_795877.1|	gi|6700814|ref|Os01g08650|	gi|34873467|ref|XP_346899.1|	gi|37362630|ref|NP_010214.2|	gi|19111979|ref|NP_595187.1|
gi|30175448|gb|EAA43923.1|	lcl|At1g23270.1	lcl|C01G10.4 CE07813 WBGene	gi|19922762|	gi|17446770|ref|XP_059462.1|	gi|28892689|ref|NP_795879.1|	gi|6700816|ref|Os01g08670|	gi|34873574|ref|XP_346946.1|	gi|37362633|ref|NP_010604.2|	gi|19111980|ref|NP_595188.1|
gi|30175485|gb|EAA43944.1|	lcl|At1g23510.1	lcl|C01G10.5 CE07814 WBGene	gi|19922800|	gi|17452744|ref|XP_058332.1|	gi|28892695|ref|NP_795883.1|	gi|6700827|ref|Os01g08740|	gi|34873603|ref|XP_346406.1|	gi|37362634|ref|NP_010671.2|	gi|19111987|ref|NP_595195.1|
gi|30175539|gb|EAA43972.1|	lcl|At1g23530.1	lcl|C01G10.6 CE07815 WBGene	gi|19922806|	gi|17462609|ref|XP_059608.1|	gi|28892697|ref|NP_795884.1|	gi|6700832|ref|Os01g08790|	gi|34873936|ref|XP_346904.1|	gi|37362637|ref|NP_010763.2|	gi|19111988|ref|NP_595196.1|
gi|30175546|gb|EAA43974.1|	lcl|At1g23650.1	lcl|C01G12.10 CE34645 WBGer	gi|19922810|	gi|17480197|ref|XP_058857.1|	gi|28892739|ref|NP_795908.1|	gi|6700838|ref|Os01g08840|	gi|34874234|ref|XP_237943.2|	gi|37362642|ref|NP_010949.2|	gi|19111996|ref|NP_595204.1|
gi|30175685|gb|EAA44041.1|	lcl|At1g23710.1	lcl|C01G12.11 CE34646 WBGer	gi|19922820|	gi|17738298|ref|NP_478104.1|	gi|28892743|ref|NP_795910.1|	gi|6700841|ref|Os01g08870|	gi|34874373|ref|XP_347111.1|	gi|37362643|ref|NP_011006.2|	gi|19112000|ref|NP_595208.1|
gi|30175719|gb|EAA11014.2|	lcl|At1g23720.1	lcl|C01G12.2 CE15572 WBGene	gi|19922900|	gi|18105010|ref|NP_055133.2|	gi|28892749|ref|NP_795915.1|	gi|6700848|ref|Os01g08940|	gi|34874513|ref|XP_346936.1|	gi|37362648|ref|NP_011264.2|	gi|19112008|ref|NP_595216.1|

gi|30177937|gb|EAA45110.1|	lcl|At1g30795.1	lcl|C04B4.1 CE05203 WBGene0	gi|20129965|	gi|20557668|ref|XP_117044.1|	gi|29244520|ref|NP_808563.1|	gi|6701086|ref|Os01g11170|	gi|6319442|ref|NP_009524.1|	gi|19112477|ref|NP_595685.1|
gi|30177942|gb|EAA06688.2|	lcl|At1g30835.1	lcl|C04B4.2 CE05204 WBGene0	gi|20130003|	gi|20557889|ref|XP_117100.1|	gi|29244536|ref|NP_808570.1|	gi|6701087|ref|Os01g11180|	gi|6319443|ref|NP_009525.1|	gi|19112483|ref|NP_595691.1|
gi|30178003|gb|EAA06199.2|	lcl|At1g30850.1	lcl|C04B4.4 CE05206 WBGene0	gi|20130069|	gi|20558546|ref|XP_085724.2|	gi|29244540|ref|NP_808572.1|	gi|6701088|ref|Os01g11190|	gi|6319453|ref|NP_009535.1|	gi|19112488|ref|NP_595696.1|
gi|30178015|gb|EAA45143.1|	lcl|At1g30880.1	lcl|C04B4.5 CE05207 WBGene0	gi|20130075|	gi|20559323|ref|XP_117056.1|	gi|29244564|ref|NP_808582.1|	gi|6701090|ref|Os01g11210|	gi|6319457|ref|NP_009539.1|	gi|19112497|ref|NP_595705.1|
gi|30178017|gb|EAA06571.2|	lcl|At1g30922.1	lcl|C04B4.6 CE05208 WBGene0	gi|20130095|	gi|20562090|ref|XP_117014.1|	gi|29244568|ref|NP_808584.1|	gi|6701105|ref|Os01g11360|	gi|6319461|ref|NP_009543.1|	gi|19112504|ref|NP_595712.1|
gi|30178038|gb|EAA06422.2|	lcl|At1g30940.1	lcl|C04C3.4 CE16810 WBGene(	gi|20130129|	gi|20562450|ref|XP_117030.1|	gi|30039724|ref|NP_780604.1|	gi|6701108|ref|Os01g11390|	gi|6319477|ref|NP_009559.1|	gi|19112515|ref|NP_595723.1|
gi|30178065|gb|EAA45167.1|	lcl|At1g31040.1	lcl|C04E6.11 CE36911 WBGene	gi|20130163|	gi|20631961|ref|NP_620117.1|	gi|30424583|ref|NP_776107.1|	gi|6701109|ref|Os01g11400|	gi|6319487|ref|NP_009569.1|	gi|19112533|ref|NP_595741.1|
gi|30178070|gb|EAA45169.1|	lcl|At1g31060.1	lcl|C04E6.13 CE32041 WBGene	gi|20130165|	gi|20911037|ref|NP_624312.1|	gi|30424693|ref|NP_778192.1|	gi|6701112|ref|Os01g11430|	gi|6319490|ref|NP_009572.1|	gi|19112534|ref|NP_595742.1|
gi|30178094|gb|EAA06158.2|	lcl|At1g31150.1	lcl|C04E6.3 CE36782 WBGene0	gi|20130179|	gi|21040357|ref|NP_620580.1|	gi|30424720|ref|NP_780326.1|	gi|6701113|ref|Os01g11440|	gi|6319510|ref|NP_009592.1|	gi|19112535|ref|NP_595743.1|
gi|30178110|gb|EAA45187.1|	lcl|At1g31163.1	lcl|C04E6.4 CE35222 WBGene0	gi|20130201|	gi|21281685|ref|NP_640343.1|	gi|30424778|ref|NP_780378.1|	gi|6701116|ref|Os01g11470|	gi|6319514|ref|NP_009596.1|	gi|19112536|ref|NP_595744.1|
gi|30178164|gb|EAA45215.1|	lcl|At1g31200.1	lcl|C04E6.8 CE07913 WBGene0	gi|20130219|	gi|21281689|ref|NP_079209.1|	gi|30424804|ref|NP_780401.1|	gi|6701122|ref|Os01g11530|	gi|6319519|ref|NP_009601.1|	gi|19112549|ref|NP_595757.1|
gi|30178167|gb|EAA06108.2|	lcl|At1g31270.1	lcl|C04E7.1 CE03918 WBGene0	gi|20130411|	gi|21314711|ref|NP_076953.2|	gi|30424824|ref|NP_780412.1|	gi|6701123|ref|Os01g11540|	gi|6319521|ref|NP_009603.1|	gi|19112555|ref|NP_595763.1|
gi|30178168|gb|EAA05987.2|	lcl|At1g31335.1	lcl|C04E7.3 CE03920 WBGene0	gi|20130417|	gi|21359957|ref|NP_078845.2|	gi|30424832|ref|NP_780417.1|	gi|6701125|ref|Os01g11560|	gi|6319525|ref|NP_009606.1|	gi|19112557|ref|NP_595765.1|
gi|30178169|gb|EAA45216.1|	lcl|At1g31380.1	lcl|C04E7.4 CE30851 WBGene0	gi|20270397|	gi|21362026|ref|NP_078952.2|	gi|30424846|ref|NP_780425.1|	gi|6701133|ref|Os01g11640|	gi|6319531|ref|NP_009613.1|	gi|19112559|ref|NP_595767.1|
gi|30178226|gb|EAA45240.1|	lcl|At1g31520.1	lcl|C04F12.5 CE19678 WBGene	gi|21355193|	gi|21362054|ref|NP_078786.2|	gi|30424848|ref|NP_780427.1|	gi|6701138|ref|Os01g11690|	gi|6319538|ref|NP_009619.1|	gi|19112562|ref|NP_595770.1|
gi|30178274|gb|EAA05813.2|	lcl|At1g31580.1	lcl|C04F12.7 CE19680 WBGene	gi|21355275|	gi|21389543|ref|NP_653264.1|	gi|30424854|ref|NP_780430.1|	gi|6701141|ref|Os01g11720|	gi|6319545|ref|NP_009627.1|	gi|19112575|ref|NP_595783.1|
gi|30178427|gb|EAA45333.1|	lcl|At1g31620.1	lcl|C04F5.2 CE35223 WBGene0	gi|21355309|	gi|21389551|ref|NP_653268.1|	gi|30424864|ref|NP_780439.1|	gi|6701146|ref|Os01g11770|	gi|6319550|ref|NP_009632.1|	gi|19112577|ref|NP_595785.1|
gi|30178514|gb|EAA05020.2|	lcl|At1g31750.1	lcl|C04F5.3 CE27791 WBGene0	gi|21355351|	gi|21450729|ref|NP_659450.1|	gi|30424894|ref|NP_780462.1|	gi|6701147|ref|Os01g11780|	gi|6319551|ref|NP_009633.1|	gi|19112578|ref|NP_595786.1|
gi|30178522|gb|EAA45355.1|	lcl|At1g31814.1	lcl|C04F5.4 CE19668 WBGene0	gi|21355381|	gi|21450745|ref|NP_659459.1|	gi|30424902|ref|NP_780466.1|	gi|6701149|ref|Os01g11800|	gi|6319566|ref|NP_009648.1|	gi|19112581|ref|NP_595789.1|
gi|30178527|gb|EAA45359.1|	lcl|At1g31960.1	lcl|C04F5.5 CE19669 WBGene0	gi|21355395|	gi|21450765|ref|NP_659470.1|	gi|30424920|ref|NP_780476.1|	gi|6701151|ref|Os01g11820|	gi|6319578|ref|NP_009660.1|	gi|19112582|ref|NP_595790.1|
gi|30178536|gb|EAA04854.2|	lcl|At1g31990.1	lcl|C04F5.6 CE19670 WBGene0	gi|21355445|	gi|21450812|ref|NP_659487.1|	gi|30424954|ref|NP_780497.1|	gi|6701154|ref|Os01g11850|	gi|6319583|ref|NP_009665.1|	gi|19112589|ref|NP_595797.1|
gi|30178546|gb|EAA45369.1|	lcl|At1g32000.1	lcl|C04F6.2 CE03922 WBGene0	gi|21355465|	gi|21464131|ref|NP_653090.1|	gi|30424956|ref|NP_780498.1|	gi|6701156|ref|Os01g11870|	gi|6319596|ref|NP_009678.1|	gi|19112593|ref|NP_595801.1|
gi|30178606|gb|EAA45393.1|	lcl|At1g32010.1	lcl|C04G2.5 CE05213 WBGene(	gi|21355667|	gi|21536342|ref|NP_065577.2|	gi|30424968|ref|NP_780506.1|	gi|6701158|ref|Os01g11890|	gi|6319604|ref|NP_009686.1|	gi|19112594|ref|NP_595802.1|
gi|30178769|gb|EAA45436.1|	lcl|At1g32040.1	lcl|C04G2.8 CE05216 WBGene(	gi|21355673|	gi|21536344|ref|NP_065578.2|	gi|30425014|ref|NP_780539.1|	gi|6701162|ref|Os01g11930|	gi|6319606|ref|NP_009688.1|	gi|19112598|ref|NP_595806.1|
gi|30178771|gb|EAA45438.1|	lcl|At1g32290.1	lcl|C04G2.9 CE05217 WBGene(	gi|21355745|	gi|21536346|ref|NP_065579.2|	gi|30425016|ref|NP_780540.1|	gi|6701167|ref|Os01g11970|	gi|6319614|ref|NP_009696.1|	gi|19112599|ref|NP_595807.1|
gi|30178791|gb|EAA04303.2|	lcl|At1g32390.1	lcl|C04G6.10 CE27649 WBGene	gi|21355783|	gi|21553333|ref|NP_660163.1|	gi|30425056|ref|NP_780563.1|	gi|6701170|ref|Os01g12000|	gi|6319624|ref|NP_009706.1|	gi|19112603|ref|NP_595811.1|
gi|30178844|gb|EAA45477.1|	lcl|At1g32460.1	lcl|C04G6.11 CE27650 WBGene	gi|21355785|	gi|21553339|ref|NP_660162.1|	gi|30425084|ref|NP_780585.1|	gi|6701171|ref|Os01g12010|	gi|6319628|ref|NP_009710.1|	gi|19112605|ref|NP_595813.1|
gi|30178845|gb|EAA45478.1|	lcl|At1g32570.1	lcl|C04G6.6 CE06767 WBGene(	gi|21355811|	gi|21687024|ref|NP_660361.1|	gi|30425094|ref|NP_780597.1|	gi|6701175|ref|Os01g12050|	gi|6319639|ref|NP_009721.1|	gi|19112609|ref|NP_595817.1|
gi|30178846|gb|EAA45479.1|	lcl|At1g32585.1	lcl|C04G6.7 CE27652 WBGene(	gi|21355877|	gi|21687070|ref|NP_660347.1|	gi|30425114|ref|NP_780613.1|	gi|6701176|ref|Os01g12060|	gi|6319640|ref|NP_009722.1|	gi|19112618|ref|NP_595826.1|
gi|30178896|gb|EAA45494.1|	lcl|At1g32610.1	lcl|C04H4.1 CE34653 WBGene(	gi|21355891|	gi|21687074|ref|NP_660299.1|	gi|30425118|ref|NP_780616.1|	gi|6701180|ref|Os01g12100|	gi|6319644|ref|NP_009726.1|	gi|19112630|ref|NP_595838.1|
gi|30178897|gb|EAA45495.1|	lcl|At1g32630.1	lcl|C04H5.7 CE27793 WBGene(	gi|21355893|	gi|21687123|ref|NP_660357.1|	gi|30425126|ref|NP_780625.1|	gi|6701181|ref|Os01g12110|	gi|6319661|ref|NP_009743.1|	gi|19112635|ref|NP_595843.1|
gi|30178898|gb|EAA45496.1|	lcl|At1g32650.1	lcl|C04H5.8 CE36467 WBGene(	gi|21355931|	gi|21687149|ref|NP_660321.1|	gi|30425134|ref|NP_780631.1|	gi|6701182|ref|Os01g12110|	gi|6319662|ref|NP_009744.1|	gi|19112641|ref|NP_595849.1|
gi|30178984|gb|EAA45535.1|	lcl|At1g32670.1	lcl|C05A9.2 CE05222 WBGene0	gi|21355939|	gi|21687220|ref|NP_660317.1|	gi|30520275|ref|NP_848909.1|	gi|6701183|ref|Os01g12120|	gi|6319665|ref|NP_009747.1|	gi|19112643|ref|NP_595851.1|
gi|30178988|gb|EAA45538.1|	lcl|At1g32680.1	lcl|C05B5.1 CE00620 WBGene0	gi|21355983|	gi|21687241|ref|NP_660351.1|	gi|30725802|ref|NP_849258.1|	gi|6701185|ref|Os01g12140|	gi|6319671|ref|NP_009753.1|	gi|19112645|ref|NP_595853.1|
gi|30178990|gb|EAA45540.1|	lcl|At1g32690.1	lcl|C05B5.2 CE00878 WBGene0	gi|21356051|	gi|21717830|ref|NP_663631.1|	gi|31560549|ref|NP_035598.2|	gi|6701196|ref|Os01g12240|	gi|6319674|ref|NP_009756.1|	gi|19112647|ref|NP_595855.1|
gi|30178998|gb|EAA45545.1|	lcl|At1g32720.1	lcl|C05B5.3 CE17369 WBGene0	gi|21356059|	gi|21735609|ref|NP_663303.1|	gi|31981480|ref|NP_035606.2|	gi|6701197|ref|Os01g12250|	gi|6319680|ref|NP_009762.1|	gi|19112648|ref|NP_595856.1|
gi|30179087|gb|EAA45588.1|	lcl|At1g32920.1	lcl|C05B5.4 CE00880 WBGene0	gi|21356119|	gi|22035659|ref|NP_663694.1|	gi|33239385|ref|NP_796107.1|	gi|6701199|ref|Os01g12270|	gi|6319688|ref|NP_009770.1|	gi|19112652|ref|NP_595860.1|
gi|30179120|gb|EAA45607.1|	lcl|At1g32975.1	lcl|C05B5.5 CE37184 WBGene0	gi|21356133|	gi|22041028|ref|XP_173083.1|	gi|33239389|ref|NP_778164.1|	gi|6701206|ref|Os01g12330|	gi|6319693|ref|NP_009775.1|	gi|19112664|ref|NP_595872.1|
gi|30179131|gb|EAA03417.2|	lcl|At1g33000.1	lcl|C05B5.8 CE33967 WBGene0	gi|21356233|	gi|22041208|ref|XP_173063.1|	gi|33239405|ref|NP_778159.1|	gi|6701218|ref|Os01g12450|	gi|6319702|ref|NP_009784.1|	gi|19112666|ref|NP_595874.1|
gi|30179207|gb|EAA03247.2|	lcl|At1g33135.1	lcl|C05B5.9 CE37185 WBGene0	gi|21356355|	gi|22041744|ref|XP_171008.1|	gi|33468863|ref|NP_032656.1|	gi|6701223|ref|Os01g12500|	gi|6319707|ref|NP_009789.1|	gi|19112674|ref|NP_595882.1|
gi|30179360|gb|EAA45745.1|	lcl|At1g33450.1	lcl|C05C10.5a CE01469 WBGer	gi|21356435|	gi|22042062|ref|XP_173120.1|	gi|33468869|ref|NP_033326.1|	gi|6701224|ref|Os01g12510|	gi|6319715|ref|NP_009797.1|	gi|19112679|ref|NP_595887.1|
gi|30179372|gb|EAA45754.1|	lcl|At1g33475.1	lcl|C05C10.5b CE35822 WBGer	gi|21356453|	gi|22042071|ref|XP_173119.1|	gi|33468933|ref|NP_071706.1|	gi|6701234|ref|Os01g12610|	gi|6319727|ref|NP_009809.1|	gi|19112680|ref|NP_595888.1|
gi|30179461|gb|EAA45796.1|	lcl|At1g33640.1	lcl|C05C10.7 CE31875 WBGene	gi|21356461|	gi|22042869|ref|XP_173068.1|	gi|33469017|ref|NP_084413.1|	gi|6701235|ref|Os01g12620|	gi|6319730|ref|NP_009812.1|	gi|19112687|ref|NP_595895.1|
gi|30179479|gb|EAA45807.1|	lcl|At1g33690.1	lcl|C05C12.4 CE02967 WBGene	gi|21356493|	gi|22043463|ref|XP_173015.1|	gi|34147063|ref|NP_898913.1|	gi|6701251|ref|Os01g12780|	gi|6319732|ref|NP_009814.1|	gi|19112694|ref|NP_595902.1|
gi|30179550|gb|EAA45857.1|	lcl|At1g33820.1	lcl|C05C12.5 CE02968 WBGene	gi|21356501|	gi|22044545|ref|XP_173105.1|	gi|34147089|ref|NP_898930.1|	gi|6701259|ref|Os01g12840|	gi|6319736|ref|NP_009818.1|	gi|19112696|ref|NP_595904.1|
gi|30179581|gb|EAA45877.1|	lcl|At1g33860.1	lcl|C05C12.6 CE02969 WBGene	gi|21356517|	gi|22044797|ref|XP_173135.1|	gi|34147097|ref|NP_898934.1|	gi|6701260|ref|Os01g12850|	gi|6319740|ref|NP_009821.1|	gi|19112703|ref|NP_595911.1|
gi|30179593|gb|EAA45882.1|	lcl|At1g34010.1	lcl|C05C8.4 CE07922 WBGene(	gi|21356529|	gi|22045460|ref|XP_173036.1|	gi|34147113|ref|NP_898943.1|	gi|6701264|ref|Os01g12890|	gi|6319745|ref|NP_009827.1|	gi|19112706|ref|NP_595914.1|
gi|30179622|gb|EAA45897.1|	lcl|At1g34095.1	lcl|C05C8.9a CE28884 WBGene	gi|21356585|	gi|22047802|ref|XP_173140.1|	gi|34147117|ref|NP_898945.1|	gi|6701272|ref|Os01g12960|	gi|6319749|ref|NP_009831.1|	gi|19112708|ref|NP_595916.1|
gi|30179642|gb|EAA02028.2|	lcl|At1g34280.1	lcl|C05C8.9b CE29481 WBGene	gi|21356627|	gi|22049633|ref|XP_172917.1|	gi|34147127|ref|NP_898949.1|	gi|6701275|ref|Os01g12990|	gi|6319752|ref|NP_009834.1|	gi|19112711|ref|NP_595919.1|
gi|30179647|gb|EAA45912.1|	lcl|At1g34315.1	lcl|C05C9.2 CE31405 WBGene(	gi|21356645|	gi|22052661|ref|XP_173160.1|	gi|34147131|ref|NP_898951.1|	gi|6701277|ref|Os01g13010|	gi|6319762|ref|NP_009844.1|	gi|19112734|ref|NP_595942.1|
gi|30179723|gb|EAA45964.1|	lcl|At1g34400.1	lcl|C05C9.3 CE02963 WBGene(	gi|21356733|	gi|22054274|ref|XP_173166.1|	gi|34147133|ref|NP_898948.1|	gi|6701278|ref|Os01g13020|	gi|6319789|ref|NP_009870.1|	gi|19112739|ref|NP_595947.1|
gi|31194083|ref|XP_305989.1|	lcl|At1g34440.1	lcl|C05D10.1a CE24783 WBGer	gi|21356755|	gi|22062992|ref|XP_172855.1|	gi|34147141|ref|NP_898953.1|	gi|6701280|ref|Os01g13040|	gi|6319792|ref|NP_009873.1|	gi|19112769|ref|NP_595977.1|
gi|31194089|ref|XP_305992.1|	lcl|At1g34590.1	lcl|C05D10.1b CE30420 WBGer	gi|21356769|	gi|22129788|ref|NP_060433.1|	gi|34147145|ref|NP_898955.1|	gi|6701284|ref|Os01g13080|	gi|6319794|ref|NP_009875.1|	gi|19112770|ref|NP_595978.1|
gi|31194091|ref|XP_305993.1|	lcl|At1g34730.1	lcl|C05D10.1c CE37698 WBGen	gi|21356815|	gi|22208973|ref|NP_665909.1|	gi|34147187|ref|NP_898982.1|	gi|6701285|ref|Os01g13090|	gi|6319797|ref|NP_009878.1|	gi|19112772|ref|NP_595980.1|
gi|31194101|ref|XP_305998.1|	lcl|At1g34910.1	lcl|C05D12.4 CE02972 WBGene	gi|21356973|	gi|22208975|ref|NP_665910.1|	gi|34147256|ref|NP_899015.1|	gi|6701286|ref|Os01g13110|	gi|6319805|ref|NP_009886.1|	gi|19112776|ref|NP_595984.1|
gi|31194105|ref|XP_306000.1|	lcl|At1g35030.1	lcl|C05D12.7 CE37875 WBGene	gi|21356977|	gi|22208979|ref|NP_665912.1|	gi|34147258|ref|NP_899023.1|	gi|6701296|ref|Os01g13180|	gi|6319845|ref|NP_009926.1|	gi|19112780|ref|NP_595988.1|
gi|31194109|ref|XP_306002.1|	lcl|At1g35040.1	lcl|C05D2.5 CE26863 WBGene(	gi|21356979|	gi|22208992|ref|NP_665863.1|	gi|34147278|ref|NP_899033.1|	gi|6701303|ref|Os01g13240|	gi|6319848|ref|NP_009929.1|	gi|19112782|ref|NP_595990.1|
gi|31194117|ref|XP_306006.1|	lcl|At1g35080.1	lcl|C05D2.6 CE32115 WBGene(	gi|21356987|	gi|22212940|ref|NP_671516.1|	gi|34147286|ref|NP_899034.1|	gi|6701308|ref|Os01g13290|	gi|6319868|ref|NP_009949.1|	gi|19112785|ref|NP_595993.1|
gi|31194127|ref|XP_306011.1|	lcl|At1g35100.1	lcl|C05D2.8 CE32116 WBGene(	gi|21357141|	gi|22380639|ref|NP_683516.1|	gi|34147308|ref|NP_899046.1|	gi|6701312|ref|Os01g13330|	gi|6319891|ref|NP_009972.1|	gi|19112789|ref|NP_595997.1|
gi|31194129|ref|XP_306012.1|	lcl|At1g35143.1	lcl|C05D9.4 CE07930 WBGene(	gi|21357151|	gi|22380643|ref|NP_683517.1|	gi|34303966|ref|NP_899123.1|	gi|6701318|ref|Os01g13380|	gi|6319894|ref|NP_009975.1|	gi|19112791|ref|NP_595999.1|
gi|31194131|ref|XP_306013.1|	lcl|At1g35183.1	lcl|C05D9.7 CE07933 WBGene(	gi|21357153|	gi|22507417|ref|NP_004667.2|	gi|34303969|ref|NP_899125.1|	gi|6701321|ref|Os01g13410|	gi|6319900|ref|NP_009981.1|	gi|19112797|ref|NP_596005.1|
gi|31194141|ref|XP_306018.1|	lcl|At1g35210.1	lcl|C05D9.9 CE27653 WBGene(	gi|21357175|	gi|22748665|ref|NP_689504.1|	gi|34303973|ref|NP_899124.1|	gi|6701324|ref|Os01g13440|	gi|6319920|ref|NP_010001.1|	gi|19112798|ref|NP_596006.1|
gi|31194143|ref|XP_306019.1|	lcl|At1g35230.1	lcl|C05E11.2 CE03932 WBGene	gi|21357201|	gi|22748759|ref|NP_689563.1|	gi|34303975|ref|NP_899129.1|	gi|6701325|ref|Os01g13450|	gi|6319924|ref|NP_010005.1|	gi|19112799|ref|NP_596007.1|
gi|31194159|ref|XP_306027.1|	lcl|At1g35320.1	lcl|C05E11.3 CE33029 WBGene	gi|21357227|	gi|22748851|ref|NP_689612.1|	gi|34303981|ref|NP_899128.1|	gi|6701330|ref|Os01g13490|	gi|6319928|ref|NP_010009.1|	gi|19112811|ref|NP_596019.1|
gi|31194165|ref|XP_306030.1|	lcl|At1g35375.1	lcl|C05E11.7 CE03937 WBGene	gi|21357285|	gi|22748899|ref|NP_689638.1|	gi|34303989|ref|NP_899132.1|	gi|6701331|ref|Os01g13500|	gi|6319942|ref|NP_010023.1|	gi|19112814|ref|NP_596022.1|
gi|31194167|ref|XP_306031.1|	lcl|At1g35430.1	lcl|C05E11.8 CE03938 WBGene	gi|21357315|	gi|22748953|ref|NP_689667.1|	gi|34304024|ref|NP_899073.1|	gi|6701332|ref|Os01g13510|	gi|6319960|ref|NP_010040.1|	gi|19112817|ref|NP_596025.1|
gi|31194169|ref|XP_306032.1|	lcl|At1g35480.1	lcl|C05E4.7 CE07941 WBGene0	gi|21357343|	gi|22748965|ref|NP_689674.1|	gi|34304028|ref|NP_899075.1|	gi|6701339|ref|Os01g13580|	gi|6319962|ref|NP_010042.1|	gi|19112837|ref|NP_596045.1|
gi|31194173|ref|XP_306034.1|	lcl|At1g35500.1	lcl|C05E7.1 CE23522 WBGene0	gi|21357345|	gi|22748981|ref|NP_689682.1|	gi|34304050|ref|NP_899088.1|	gi|6701345|ref|Os01g13640|	gi|6319964|ref|NP_010044.1|	gi|19112840|ref|NP_596048.1|
gi|31194175|ref|XP_306035.1|	lcl|At1g35513.1	lcl|C05E7.2 CE02976 WBGene0	gi|21357357|	gi|22748983|ref|NP_689679.1|	gi|34304054|ref|NP_899090.1|	gi|6701346|ref|Os01g13650|	gi|6319968|ref|NP_010048.1|	gi|19112843|ref|NP_596051.1|
gi|31194177|ref|XP_306036.1|	lcl|At1g35612.1	lcl|C05E7.3 CE34154 WBGene0	gi|21357417|	gi|22749011|ref|NP_689693.1|	gi|34304056|ref|NP_899091.1|	gi|6701347|ref|Os01g13660|	gi|6319969|ref|NP_010049.1|	gi|19112868|ref|NP_596076.1|
gi|31194195|ref|XP_306045.1|	lcl|At1g35614.1	lcl|C05G5.2 CE05225 WBGene(	gi|21357497|	gi|22749017|ref|NP_689701.1|	gi|34304075|ref|NP_899100.1|	gi|6701348|ref|Os01g13660|	gi|6319970|ref|NP_010050.1|	gi|19112874|ref|NP_596082.1|
gi|31194201|ref|XP_306048.1|	lcl|At1g35617.1	lcl|C05G5.3 CE30853 WBGene(	gi|21357505|	gi|22749043|ref|NP_689710.1|	gi|38049314|ref|XP_356574.1|	gi|6701349|ref|Os01g13670|	gi|6319978|ref|NP_010058.1|	gi|19112875|ref|NP_596083.1|
gi|31194211|ref|XP_306053.1|	lcl|At1g35640.1	lcl|C06A1.2 CE02115 WBGene0	gi|21357585|	gi|22749045|ref|NP_689715.1|	gi|38049450|ref|XP_356579.1|	gi|6701361|ref|Os01g13780|	gi|6319983|ref|NP_010063.1|	gi|19112880|ref|NP_596088.1|
gi|31194213|ref|XP_306054.1|	lcl|At1g35740.1	lcl|C06A1.6 CE20484 WBGene0	gi|21357587|	gi|22749053|ref|NP_689720.1|	gi|38049460|ref|XP_138001.2|	gi|6701362|ref|Os01g13790|	gi|6319990|ref|NP_010070.1|	gi|19112884|ref|NP_596092.1|
gi|31194215|ref|XP_306055.1|	lcl|At1g35820.1	lcl|C06A12.8 CE34655 WBGene	gi|21357649|	gi|22749059|ref|NP_689719.1|	gi|38049485|ref|XP_129804.2|	gi|6701366|ref|Os01g13830|	gi|6319997|ref|NP_010077.1|	gi|19112920|ref|NP_596128.1|
gi|31194217|ref|XP_306056.1|	lcl|At1g35880.1	lcl|C06A5.10 CE07957 WBGene	gi|21357669|	gi|22749063|ref|NP_689721.1|	gi|38049495|ref|XP_358537.1|	gi|6701367|ref|Os01g13840|	gi|6319999|ref|NP_010079.1|	gi|19112921|ref|NP_596129.1|
gi|31194219|ref|XP_306057.1|	lcl|At1g35890.1	lcl|C06A5.2 CE07949 WBGene0	gi|21357683|	gi|22749121|ref|NP_689754.1|	gi|38049541|ref|XP_358538.1|	gi|6701368|ref|Os01g13850|	gi|6320004|ref|NP_010084.1|	gi|19112927|ref|NP_596135.1|
gi|31194231|ref|XP_306063.1|	lcl|At1g35900.1	lcl|C06A5.5 CE07952 WBGene0	gi|21357741|	gi|22749155|ref|NP_689767.1|	gi|38049555|ref|XP_358539.1|	gi|6701369|ref|Os01g13860|	gi|6320014|ref|NP_010095.1|	gi|19112929|ref|NP_596137.1|
gi|31194235|ref|XP_306065.1|	lcl|At1g36020.1	lcl|C06A5.6 CE07953 WBGene0	gi|21357827|	gi|22749167|ref|NP_689774.1|	gi|38049563|ref|XP_149025.3|	gi|6701370|ref|Os01g13870|	gi|6320018|ref|NP_010098.1|	gi|19112931|ref|NP_596139.1|
gi|31194239|ref|XP_306067.1|	lcl|At1g36030.1	lcl|C06A5.8b CE33360 WBGene	gi|21357841|	gi|22749173|ref|NP_689782.1|	gi|38049571|ref|XP_358540.1|	gi|6701371|ref|Os01g13880|	gi|6320021|ref|NP_010101.1|	gi|19112934|ref|NP_596142.1|
gi|31194249|ref|XP_306072.1|	lcl|At1g36040.1	lcl|C06A6.2a CE31152 WBGene	gi|21357869|	gi|22749177|ref|NP_689784.1|	gi|38049650|ref|XP_358541.1|	gi|6701375|ref|Os01g13920|	gi|6320025|ref|NP_010105.1|	gi|19112943|ref|NP_596151.1|
gi|31194251|ref|XP_306073.1|	lcl|At1g36080.1	lcl|C06A6.2b CE31153 WBGene	gi|21357901|	gi|22749185|ref|NP_689788.1|	gi|38049652|ref|XP_358542.1|	gi|6701376|ref|Os01g13930|	gi|6320028|ref|NP_010108.1|	gi|19112944|ref|NP_596152.1|
gi|31194253|ref|XP_306074.1|	lcl|At1g36100.1	lcl|C06A6.7 CE35952 WBGene0	gi|21357915|	gi|22749195|ref|NP_689789.1|	gi|38049678|ref|XP_358543.1|	gi|6701379|ref|Os01g13960|	gi|6320032|ref|NP_010112.1|	gi|19112949|ref|NP_596157.1|
gi|31194259|ref|XP_306077.1|	lcl|At1g36230.1	lcl|C06A8.2 CE31292 WBGene0	gi|21357917|	gi|22749201|ref|NP_689793.1|	gi|38049700|ref|XP_357109.1|	gi|6701383|ref|Os01g14000|	gi|6320044|ref|NP_010124.1|	gi|19112952|ref|NP_596160.1|
gi|31194261|ref|XP_306078.1|	lcl|At1g36395.1	lcl|C06A8.3 CE02454 WBGene0	gi|21357937|	gi|22749313|ref|NP_689857.1|	gi|38050323|ref|XP_358331.1|	gi|6701390|ref|Os01g14060|	gi|6320069|ref|NP_010149.1|	gi|19112953|ref|NP_596161.1|
gi|31194267|ref|XP_306081.1|	lcl|At1g36600.1	lcl|C06A8.5 CE02456 WBGene0	gi|21357949|	gi|22749317|ref|NP_689859.1|	gi|38050351|ref|XP_358544.1|	gi|6701397|ref|Os01g14120|	gi|6320074|ref|NP_010154.1|	gi|19112962|ref|NP_596170.1|
gi|31194275|ref|XP_306085.1|	lcl|At1g36640.1	lcl|C06A8.8b CE32117 WBGene	gi|21357969|	gi|22749343|ref|NP_689874.1|	gi|38073266|ref|XP_358332.1|	gi|6701398|ref|Os01g14130|	gi|6320082|ref|NP_010162.1|	gi|19112967|ref|NP_596176.1|
gi|31194281|ref|XP_306088.1|	lcl|At1g36670.1	lcl|C06B3.6 CE07963 WBGene0	gi|21358035|	gi|22749345|ref|NP_689875.1|	gi|38073320|ref|XP_358546.1|	gi|6701401|ref|Os01g14160|	gi|6320093|ref|NP_010173.1|	gi|19112968|ref|NP_596175.1|
gi|31194305|ref|XP_306100.1|	lcl|At1g36675.1	lcl|C06B3.7 CE28430 WBGene0	gi|21358085|	gi|22749349|ref|NP_689877.1|	gi|38073354|ref|XP_358733.1|	gi|6701402|ref|Os01g14170|	gi|6320104|ref|NP_010184.1|	gi|19112974|ref|NP_596182.1|
gi|31194309|ref|XP_306102.1|	lcl|At1g36745.1	lcl|C06B8.11 CE34657 WBGene	gi|21358087|	gi|22749355|ref|NP_689882.1|	gi|38073591|ref|XP_358424.1|	gi|6701403|ref|Os01g14180|	gi|6320105|ref|NP_010185.1|	gi|19112980|ref|NP_596188.1|
gi|31194329|ref|XP_306112.1|	lcl|At1g36756.1	lcl|C06C3.4 CE32568 WBGene(	gi|21358109|	gi|22749375|ref|NP_689897.1|	gi|38073646|ref|XP_149003.2|	gi|6701406|ref|Os01g14210|	gi|6320114|ref|NP_010194.1|	gi|19112982|ref|NP_596190.1|
gi|31194331|ref|XP_306113.1|	lcl|At1g36763.1	lcl|C06C3.7 CE00888 WBGene(	gi|21358111|	gi|22749423|ref|NP_689927.1|	gi|38073730|ref|XP_358427.1|	gi|6701407|ref|Os01g14220|	gi|6320127|ref|NP_010207.1|	gi|19112996|ref|NP_596204.1|
gi|31194335|ref|XP_306115.1|	lcl|At1g36820.1	lcl|C06E1.1 CE00054 WBGene0	gi|21358219|	gi|22749445|ref|NP_689941.1|	gi|38073989|ref|XP_357153.1|	gi|6701408|ref|Os01g14230|	gi|6320130|ref|NP_010210.1|	gi|19113008|ref|NP_596216.1|
gi|31194349|ref|XP_306122.1|	lcl|At1g36900.1	lcl|C06E1.11 CE00056 WBGene	gi|21358285|	gi|22749457|ref|NP_689951.1|	gi|38074169|ref|XP_129819.2|	gi|6701409|ref|Os01g14240|	gi|6320150|ref|NP_010230.1|	gi|19113009|ref|NP_596217.1|
gi|31194363|ref|XP_306129.1|	lcl|At1g36920.1	lcl|C06E1.5 CE00060 WBGene0	gi|21358287|	gi|22749463|ref|NP_689959.1|	gi|38074180|ref|XP_358734.1|	gi|6701410|ref|Os01g14250|	gi|6320160|ref|NP_010240.1|	gi|19113017|ref|NP_596225.1|
gi|31194367|ref|XP_306131.1|	lcl|At1g36925.1	lcl|C06E1.6 CE00061 WBGene0	gi|21358329|	gi|22749483|ref|NP_689970.1|	gi|38074324|ref|XP_358437.1|	gi|6701413|ref|Os01g14280|	gi|6320165|ref|NP_010245.1|	gi|19113024|ref|NP_596232.1|
gi|31194373|ref|XP_306134.1|	lcl|At1g36940.1	lcl|C06E1.9 CE24790 WBGene0	gi|21358333|	gi|22749507|ref|NP_689981.1|	gi|38074511|ref|XP_358441.1|	gi|6701414|ref|Os01g14290|	gi|6320170|ref|NP_010249.1|	gi|19113027|ref|NP_596235.1|
gi|31194377|ref|XP_306136.1|	lcl|At1g36960.1	lcl|C06E2.1 CE03944 WBGene0	gi|21358339|	gi|22749511|ref|NP_689984.1|	gi|38074557|ref|XP_205896.3|	gi|6701416|ref|Os01g14310|	gi|6320178|ref|NP_010257.1|	gi|19113030|ref|NP_596238.1|
gi|31194381|ref|XP_306138.1|	lcl|At1g36970.1	lcl|C06E2.2 CE03945 WBGene0	gi|21358357|	gi|22749523|ref|NP_689990.1|	gi|38074764|ref|XP_356667.1|	gi|6701417|ref|Os01g14320|	gi|6320191|ref|NP_010271.1|	gi|19113040|ref|NP_596248.1|
gi|31194385|ref|XP_306140.1|	lcl|At1g37000.1	lcl|C06E2.5 CE30595 WBGene0	gi|21358367|	gi|22758132|ref|NP_689855.1|	gi|38074869|ref|XP_358444.1|	gi|6701418|ref|Os01g14330|	gi|6320192|ref|NP_010272.1|	gi|19113042|ref|NP_596250.1|
gi|31194393|ref|XP_306144.1|	lcl|At1g37010.1	lcl|C06E4.1 CE03949 WBGene0	gi|21358375|	gi|23097313|ref|NP_689553.1|	gi|38074899|ref|XP_138684.2|	gi|6701419|ref|Os01g14340|	gi|6320199|ref|NP_010279.1|	gi|19113053|ref|NP_596261.1|
gi|31194417|ref|XP_306156.1|	lcl|At1g37015.1	lcl|C06E4.2 CE03950 WBGene0	gi|21358383|	gi|23110980|ref|NP_690594.1|	gi|38074918|ref|XP_358561.1|	gi|6701420|ref|Os01g14350|	gi|6320206|ref|NP_010286.1|	gi|19113054|ref|NP_596262.1|
gi|31194429|ref|XP_306162.1|	lcl|At1g37037.1	lcl|C06E4.8 CE03956 WBGene0	gi|21358385|	gi|23308513|ref|NP_694585.1|	gi|38075046|ref|XP_358445.1|	gi|6701421|ref|Os01g14360|	gi|6320217|ref|NP_010297.1|	gi|19113073|ref|NP_596281.1|
gi|31194447|ref|XP_306171.1|	lcl|At1g37045.1	lcl|C06E7.2 CE33516 WBGene0	gi|21358387|	gi|23308521|ref|NP_694559.1|	gi|38075069|ref|XP_358448.1|	gi|6701424|ref|Os01g14380|	gi|6320219|ref|NP_010299.1|	gi|19113080|ref|NP_596288.1|
gi|31194449|ref|XP_306172.1|	lcl|At1g37058.1	lcl|C06E7.4 CE29026 WBGene0	gi|21358389|	gi|23308525|ref|NP_694589.1|	gi|38075222|ref|XP_130628.2|	gi|6701425|ref|Os01g14390|	gi|6320225|ref|NP_010305.1|	gi|19113085|ref|NP_596293.1|
gi|31194459|ref|XP_306177.1|	lcl|At1g38380.1	lcl|C06E7.6 CE03962 WBGene0	gi|21358399|	gi|23308527|ref|NP_694562.1|	gi|38075362|ref|XP_147773.2|	gi|6701426|ref|Os01g14400|	gi|6320238|ref|NP_010318.1|	gi|19113097|ref|NP_596305.1|
gi|31194471|ref|XP_306183.1|	lcl|At1g38630.1	lcl|C06E8.1 CE00726 WBGene0	gi|21358401|	gi|23308529|ref|NP_694576.1|	gi|38075365|ref|XP_358449.1|	gi|6701429|ref|Os01g14430|	gi|6320239|ref|NP_010319.1|	gi|19113100|ref|NP_596308.1|
gi|31194487|ref|XP_306191.1|	lcl|At1g38790.1	lcl|C06G1.2 CE03965 WBGene(	gi|21358423|	gi|23308531|ref|NP_694554.1|	gi|38075405|ref|XP_358730.1|	gi|6701433|ref|Os01g14470|	gi|6320247|ref|NP_010327.1|	gi|19113113|ref|NP_596321.1|
gi|31194495|ref|XP_306195.1|	lcl|At1g38950.1	lcl|C06G1.4 CE03967 WBGene(	gi|21358425|	gi|23308533|ref|NP_694556.1|	gi|38075407|ref|XP_358731.1|	gi|6701436|ref|Os01g14500|	gi|6320261|ref|NP_010341.1|	gi|19113124|ref|NP_596332.1|
gi|31194501|ref|XP_306198.1|	lcl|At1g39350.1	lcl|C06G3.12 CE32570 WBGene	gi|21358429|	gi|23308537|ref|NP_694583.1|	gi|38075429|ref|XP_110478.3|	gi|6701448|ref|Os01g14600|	gi|6320262|ref|NP_010342.1|	gi|19113125|ref|NP_596333.1|
gi|31194503|ref|XP_306199.1|	lcl|At1g39750.1	lcl|C06G3.3 CE07979 WBGene(	gi|21358441|	gi|23308539|ref|NP_694586.1|	gi|38075619|ref|XP_358570.1|	gi|6701449|ref|Os01g14620|	gi|6320270|ref|NP_010350.1|	gi|19113127|ref|NP_596335.1|
gi|31194509|ref|XP_306202.1|	lcl|At1g40080.1	lcl|C06G4.2c CE00517 WBGene	gi|21358465|	gi|23308541|ref|NP_694572.1|	gi|38075687|ref|XP_358573.1|	gi|6701451|ref|Os01g14640|	gi|6320271|ref|NP_010351.1|	gi|19113134|ref|NP_596342.1|
gi|31194521|ref|XP_306208.1|	lcl|At1g40087.1	lcl|C06G4.4 CE00549 WBGene(	gi|21358467|	gi|23308547|ref|NP_694584.1|	gi|38075751|ref|XP_358574.1|	gi|6701459|ref|Os01g14680|	gi|6320275|ref|NP_010355.1|	gi|19113139|ref|NP_596347.1|
gi|31194531|ref|XP_306213.1|	lcl|At1g40111.1	lcl|C06H2.6 CE29664 WBGene(	gi|21358487|	gi|23308549|ref|NP_694577.1|	gi|38075861|ref|XP_357269.1|	gi|6701464|ref|Os01g14740|	gi|6320278|ref|NP_010358.1|	gi|19113149|ref|NP_596357.1|
gi|31194537|ref|XP_306216.1|	lcl|At1g40115.1	lcl|C07A12.2 CE03970 WBGene	gi|21358489|	gi|23308555|ref|NP_694561.1|	gi|38075916|ref|XP_358454.1|	gi|6701466|ref|Os01g14760|	gi|6320282|ref|NP_010362.1|	gi|19113158|ref|NP_596366.1|
gi|31194549|ref|XP_306222.1|	lcl|At1g40125.1	lcl|C07A9.10 CE00496 WBGene	gi|21358509|	gi|23308557|ref|NP_694582.1|	gi|38076059|ref|XP_358457.1|	gi|6701468|ref|Os01g14780|	gi|6320283|ref|NP_010363.1|	gi|19113175|ref|NP_596383.1|
gi|31194567|ref|XP_306231.1|	lcl|At1g40129.1	lcl|C07A9.9 CE00493 WBGene0	gi|21358539|	gi|23308569|ref|NP_694588.1|	gi|38076113|ref|XP_356733.1|	gi|6701470|ref|Os01g14800|	gi|6320284|ref|NP_010364.1|	gi|19113178|ref|NP_596386.1|
gi|31194569|ref|XP_306232.1|	lcl|At1g40133.1	lcl|C07B5.2 CE00892 WBGene0	gi|21358541|	gi|23308581|ref|NP_694555.1|	gi|38076119|ref|XP_355391.1|	gi|6701476|ref|Os01g14850|	gi|6320287|ref|NP_010367.1|	gi|19113179|ref|NP_596387.1|
gi|31194579|ref|XP_306237.1|	lcl|At1g40230.1	lcl|C07B5.3 CE00893 WBGene0	gi|21358581|	gi|23308583|ref|NP_694553.1|	gi|38076227|ref|XP_130824.2|	gi|6701481|ref|Os01g14880|	gi|6320290|ref|NP_010370.1|	gi|19113186|ref|NP_596394.1|
gi|31194581|ref|XP_306238.1|	lcl|At1g41650.1	lcl|C07B5.6 CE17374 WBGene0	gi|21358589|	gi|23308591|ref|NP_694542.1|	gi|38076266|ref|XP_358350.1|	gi|6701484|ref|Os01g14910|	gi|6320305|ref|NP_010385.1|	gi|19113187|ref|NP_596395.1|
gi|31194589|ref|XP_306242.1|	lcl|At1g41750.1	lcl|C07C7.1 CE02982 WBGene(	gi|21358623|	gi|23308695|ref|NP_694535.1|	gi|38076348|ref|XP_358459.1|	gi|6701488|ref|Os01g14940|	gi|6320313|ref|NP_010393.1|	gi|19113188|ref|NP_596396.1|
gi|31194599|ref|XP_306247.1|	lcl|At1g41770.1	lcl|C07D10.1 CE27056 WBGene	gi|21358639|	gi|23397431|ref|NP_694935.1|	gi|38076367|ref|XP_358460.1|	gi|6701491|ref|Os01g14970|	gi|6320315|ref|NP_010395.1|	gi|19113193|ref|NP_596402.1|
gi|31194601|ref|XP_306248.1|	lcl|At1g41810.1	lcl|C07D10.5 CE36628 WBGene	gi|21358649|	gi|23397449|ref|NP_694936.1|	gi|38076396|ref|XP_358463.1|	gi|6701500|ref|Os01g15030|	gi|6320318|ref|NP_010398.1|	gi|19113195|ref|NP_596403.1|
gi|31194623|ref|XP_306259.1|	lcl|At1g41820.1	lcl|C07D8.2 CE06772 WBGene(	gi|21617857|	gi|23397485|ref|NP_694956.1|	gi|38076402|ref|XP_358464.1|	gi|6701503|ref|Os01g15060|	gi|6320323|ref|NP_010403.1|	gi|19113198|ref|NP_596406.1|
gi|31194625|ref|XP_306260.1|	lcl|At1g41855.1	lcl|C07D8.3 CE06773 WBGene(	gi|21686961|	gi|23397494|ref|NP_694959.1|	gi|38076486|ref|XP_356752.1|	gi|6701504|ref|Os01g15070|	gi|6320329|ref|NP_010409.1|	gi|19113207|ref|NP_596415.1|
gi|31194631|ref|XP_306263.1|	lcl|At1g41860.1	lcl|C07E3.10 CE15607 WBGene	gi|21686965|	gi|23397498|ref|NP_694961.1|	gi|38076564|ref|XP_358582.1|	gi|6701505|ref|Os01g15080|	gi|6320335|ref|NP_010415.1|	gi|19113221|ref|NP_596429.1|
gi|31194649|ref|XP_306272.1|	lcl|At1g41870.1	lcl|C07E3.1b CE23526 WBGene	gi|21686983|	gi|23397520|ref|NP_694970.1|	gi|38076570|ref|XP_358584.1|	gi|6701506|ref|Os01g15090|	gi|6320340|ref|NP_010421.1|	gi|19113228|ref|NP_596436.1|
gi|31194661|ref|XP_306278.1|	lcl|At1g41900.1	lcl|C07E3.3 CE32305 WBGene0	gi|21687078|	gi|23397530|ref|NP_694973.1|	gi|38076593|ref|XP_357304.1|	gi|6701507|ref|Os01g15100|	gi|6320342|ref|NP_010422.1|	gi|19113231|ref|NP_596439.1|
gi|31194669|ref|XP_306282.1|	lcl|At1g42040.1	lcl|C07F11.2 CE23528 WBGene	gi|21687131|	gi|23397532|ref|NP_694976.1|	gi|38076656|ref|XP_358469.1|	gi|6701511|ref|Os01g15140|	gi|6320353|ref|NP_010434.1|	gi|19113235|ref|NP_596443.1|
gi|31194681|ref|XP_306288.1|	lcl|At1g42080.1	lcl|C07G1.6 CE06782 WBGene(	gi|21687169|	gi|23397534|ref|NP_694978.1|	gi|38077020|ref|XP_358470.1|	gi|6701512|ref|Os01g15150|	gi|6320360|ref|NP_010440.1|	gi|19113241|ref|NP_596449.1|
gi|31194697|ref|XP_306296.1|	lcl|At1g42190.1	lcl|C07G1.7 CE06783 WBGene(	gi|21687194|	gi|23397538|ref|NP_694980.1|	gi|38077033|ref|XP_358471.1|	gi|6701513|ref|Os01g15160|	gi|6320370|ref|NP_010450.1|	gi|19113246|ref|NP_596454.1|
gi|31194699|ref|XP_306297.1|	lcl|At1g42220.1	lcl|C07G3.10 CE27669 WBGene	gi|21687204|	gi|23503321|ref|NP_699206.1|	gi|38077066|ref|XP_358590.1|	gi|6701514|ref|Os01g15170|	gi|6320373|ref|NP_010453.1|	gi|19113254|ref|NP_596462.1|
gi|31194703|ref|XP_306299.1|	lcl|At1g42420.1	lcl|C07G3.2 CE07993 WBGene(	gi|21687206|	gi|23503335|ref|NP_694983.1|	gi|38077116|ref|XP_128070.4|	gi|6701517|ref|Os01g15200|	gi|6320384|ref|NP_010464.1|	gi|19113261|ref|NP_596469.1|
gi|31194711|ref|XP_306303.1|	lcl|At1g42515.1	lcl|C07H4.1 CE02986 WBGene(	gi|21687216|	gi|23510431|ref|NP_690615.1|	gi|38077126|ref|XP_358473.1|	gi|6701518|ref|Os01g15210|	gi|6320387|ref|NP_010467.1|	gi|19113266|ref|NP_596474.1|

gi|31194717|ref|XP_306306.1|	lcl|At1g42580.1	lcl|C07H6.6 CE30240 WBGene0	gi|21699032|	gi|23510434|ref|NP_690616.1|	gi|38077132|ref|XP_358474.1|	gi|6701519|ref|Os01g15220|	gi|6320390|ref|NP_010470.1|	gi|19113274|ref|NP_596482.1|
gi|31194763|ref|XP_306329.1|	lcl|At1g42610.1	lcl|C07H6.9 CE29075 WBGene0	gi|21699042|	gi|23592236|ref|NP_703155.1|	gi|38077138|ref|XP_358475.1|	gi|6701520|ref|Os01g15230|	gi|6320399|ref|NP_010478.1|	gi|19113282|ref|NP_596490.1|
gi|31194765|ref|XP_306330.1|	lcl|At1g42630.1	lcl|C08A9.3 CE06785 WBGene0	gi|21699046|	gi|23592244|ref|NP_703156.1|	gi|38077221|ref|XP_356794.1|	gi|6701521|ref|Os01g15240|	gi|6320401|ref|NP_010481.1|	gi|19113284|ref|NP_596492.1|
gi|31194767|ref|XP_306331.1|	lcl|At1g42640.1	lcl|C08A9.4 CE06786 WBGene0	gi|22024071|	gi|23943878|ref|NP_115629.1|	gi|38077223|ref|XP_356795.1|	gi|6701522|ref|Os01g15250|	gi|6320407|ref|NP_010487.1|	gi|19113300|ref|NP_596508.1|
gi|31194769|ref|XP_306332.1|	lcl|At1g42650.1	lcl|C08A9.5 CE06787 WBGene0	gi|22024236|	gi|23943884|ref|NP_115662.1|	gi|38077236|ref|XP_358592.1|	gi|6701533|ref|Os01g15360|	gi|6320409|ref|NP_010488.1|	gi|19113301|ref|NP_596509.1|
gi|31194781|ref|XP_306338.1|	lcl|At1g42700.1	lcl|C08B6.10 CE05247 WBGene	gi|22024244|	gi|24234760|ref|NP_705611.1|	gi|38077275|ref|XP_358596.1|	gi|6701534|ref|Os01g15370|	gi|6320415|ref|NP_010496.1|	gi|19113303|ref|NP_596511.1|
gi|31194785|ref|XP_306340.1|	lcl|At1g42740.1	lcl|C08B6.11 CE31297 WBGene	gi|22024259|	gi|24234763|ref|NP_705612.1|	gi|38077302|ref|XP_357330.1|	gi|6701536|ref|Os01g15390|	gi|6320428|ref|NP_010508.1|	gi|19113309|ref|NP_596517.1|
gi|31194795|ref|XP_306345.1|	lcl|At1g42960.1	lcl|C08B6.2 CE31298 WBGene0	gi|22024283|	gi|24308424|ref|NP_714920.1|	gi|38077395|ref|XP_356801.1|	gi|6701537|ref|Os01g15400|	gi|6320429|ref|NP_010509.1|	gi|19113311|ref|NP_596519.1|
gi|31194827|ref|XP_306361.1|	lcl|At1g43200.1	lcl|C08B6.6 CE33972 WBGene0	gi|22024402|	gi|24371250|ref|NP_715631.1|	gi|38077469|ref|XP_358481.1|	gi|6701539|ref|Os01g15420|	gi|6320433|ref|NP_010513.1|	gi|19113312|ref|NP_596520.1|
gi|31194835|ref|XP_306365.1|	lcl|At1g43205.1	lcl|C08E3.1 CE08004 WBGene0	gi|22026856|	gi|24371254|ref|NP_715633.1|	gi|38077577|ref|XP_358598.1|	gi|6701540|ref|Os01g15430|	gi|6320435|ref|NP_010515.1|	gi|19113325|ref|NP_596533.1|
gi|31194839|ref|XP_306367.1|	lcl|At1g43220.1	lcl|C08E3.11 CE08014 WBGene	gi|22026928|	gi|24371258|ref|NP_715632.1|	gi|38077726|ref|XP_356835.1|	gi|6701541|ref|Os01g15440|	gi|6320437|ref|NP_010517.1|	gi|19113327|ref|NP_596535.1|
gi|31194855|ref|XP_306375.1|	lcl|At1g43230.1	lcl|C08E3.13 CE08004 WBGene	gi|22026962|	gi|24371262|ref|NP_715634.1|	gi|38077749|ref|XP_358601.1|	gi|6701551|ref|Os01g15510|	gi|6320445|ref|NP_010525.1|	gi|19113336|ref|NP_596544.1|
gi|31194857|ref|XP_306376.1|	lcl|At1g43290.1	lcl|C08E8.3 CE17377 WBGene0	gi|23397405|	gi|24371264|ref|NP_715636.1|	gi|38077803|ref|XP_358485.1|	gi|6701556|ref|Os01g15560|	gi|6320447|ref|NP_010526.1|	gi|19113350|ref|NP_596558.1|
gi|31194859|ref|XP_306377.1|	lcl|At1g43320.1	lcl|C08E8.4 CE17378 WBGene0	gi|23397619|	gi|24415992|ref|NP_722468.1|	gi|38077884|ref|XP_358602.1|	gi|6701557|ref|Os01g15570|	gi|6320455|ref|NP_010535.1|	gi|19113359|ref|NP_596567.1|
gi|31194865|ref|XP_306380.1|	lcl|At1g43415.1	lcl|C08E8.5 CE34668 WBGene0	gi|24580543|	gi|25121989|ref|NP_109588.2|	gi|38077945|ref|XP_356845.1|	gi|6701559|ref|Os01g15590|	gi|6320457|ref|NP_010537.1|	gi|19113362|ref|NP_596570.1|
gi|31194867|ref|XP_306381.1|	lcl|At1g43680.1	lcl|C08E8.6 CE34669 WBGene0	gi|24580591|	gi|25188181|ref|NP_742002.1|	gi|38078100|ref|XP_133529.2|	gi|6701561|ref|Os01g15610|	gi|6320468|ref|NP_010548.1|	gi|19113371|ref|NP_596579.1|
gi|31194887|ref|XP_306391.1|	lcl|At1g43720.1	lcl|C08F1.10 CE31770 WBGene	gi|24580601|	gi|25777673|ref|NP_740751.1|	gi|38078316|ref|XP_358608.1|	gi|6701568|ref|Os01g15680|	gi|6320481|ref|NP_010561.1|	gi|19113378|ref|NP_596586.1|
gi|31194905|ref|XP_306400.1|	lcl|At1g43777.1	lcl|C08F1.6 CE08021 WBGene0	gi|24580603|	gi|26006855|ref|NP_742148.1|	gi|38078318|ref|XP_358609.1|	gi|6701574|ref|Os01g15740|	gi|6320483|ref|NP_010563.1|	gi|19113386|ref|NP_596594.1|
gi|31194909|ref|XP_306402.1|	lcl|At1g43790.1	lcl|C08F1.8 CE08023 WBGene0	gi|24580610|	gi|27363470|ref|NP_758958.1|	gi|38078453|ref|XP_355501.1|	gi|6701582|ref|Os01g15800|	gi|6320485|ref|NP_010565.1|	gi|19113389|ref|NP_596597.1|
gi|31194911|ref|XP_306403.1|	lcl|At1g43810.1	lcl|C08F11.1 CE17379 WBGene	gi|24580623|	gi|27363482|ref|NP_758956.1|	gi|38078500|ref|XP_143884.2|	gi|6701594|ref|Os01g15920|	gi|6320487|ref|NP_010567.1|	gi|19113400|ref|NP_596608.1|
gi|31194915|ref|XP_306405.1|	lcl|At1g43870.1	lcl|C08F11.10 CE17387 WBGen	gi|24580650|	gi|27414495|ref|NP_005938.1|	gi|38078775|ref|XP_358614.1|	gi|6701596|ref|Os01g15940|	gi|6320494|ref|NP_010574.1|	gi|19113408|ref|NP_596616.1|
gi|31194923|ref|XP_306409.1|	lcl|At1g43895.1	lcl|C08F11.13 CE27799 WBGen	gi|24580680|	gi|27477066|ref|NP_775260.1|	gi|38078781|ref|XP_358615.1|	gi|6701597|ref|Os01g15950|	gi|6320501|ref|NP_010581.1|	gi|19113415|ref|NP_596623.1|
gi|31194959|ref|XP_306427.1|	lcl|At1g43920.1	lcl|C08F11.2 CE17380 WBGene	gi|24580693|	gi|27477380|ref|XP_211707.1|	gi|38078926|ref|XP_358619.1|	gi|6701598|ref|Os01g15960|	gi|6320502|ref|NP_010582.1|	gi|19113420|ref|NP_596628.1|
gi|31194975|ref|XP_306435.1|	lcl|At1g43940.1	lcl|C08F11.3 CE35387 WBGene	gi|24580695|	gi|27477439|ref|XP_211805.1|	gi|38079117|ref|XP_357416.1|	gi|6701599|ref|Os01g15970|	gi|6320516|ref|NP_010596.1|	gi|19113429|ref|NP_596637.1|
gi|31195011|ref|XP_306453.1|	lcl|At1g43970.1	lcl|C08F8.3 CE30596 WBGene0	gi|24580697|	gi|27477610|ref|XP_211837.1|	gi|38079119|ref|XP_357417.1|	gi|6701601|ref|Os01g15990|	gi|6320520|ref|NP_010600.1|	gi|19113432|ref|NP_596640.1|
gi|31195015|ref|XP_306455.1|	lcl|At1g44080.1	lcl|C08F8.9 CE34670 WBGene0	gi|24580702|	gi|27477615|ref|XP_211768.1|	gi|38079524|ref|XP_357447.1|	gi|6701608|ref|Os01g16060|	gi|6320521|ref|NP_010601.1|	gi|19113440|ref|NP_596648.1|
gi|31195031|ref|XP_306463.1|	lcl|At1g44085.1	lcl|C08G5.2 CE34671 WBGene0	gi|24580708|	gi|27477655|ref|XP_209563.1|	gi|38079688|ref|XP_358489.1|	gi|6701609|ref|Os01g16070|	gi|6320523|ref|NP_010603.1|	gi|19113451|ref|NP_596659.1|
gi|31195043|ref|XP_306469.1|	lcl|At1g44222.1	lcl|C08G5.5 CE19694 WBGene0	gi|24580748|	gi|27477865|ref|XP_211843.1|	gi|38079764|ref|XP_358632.1|	gi|6701616|ref|Os01g16140|	gi|6320525|ref|NP_010605.1|	gi|19113466|ref|NP_596674.1|
gi|31195049|ref|XP_306472.1|	lcl|At1g44478.1	lcl|C08G5.6 CE19695 WBGene0	gi|24580756|	gi|27478066|ref|XP_211853.1|	gi|38079782|ref|XP_358491.1|	gi|6701632|ref|Os01g16250|	gi|6320529|ref|NP_010609.1|	gi|19113473|ref|NP_596681.1|
gi|31195059|ref|XP_306477.1|	lcl|At1g44608.1	lcl|C08H9.15 CE34673 WBGene	gi|24580762|	gi|27478326|ref|XP_212170.1|	gi|38079789|ref|XP_358492.1|	gi|6701636|ref|Os01g16280|	gi|6320557|ref|NP_010637.1|	gi|19113482|ref|NP_596690.1|
gi|31195069|ref|XP_306482.1|	lcl|At1g44740.1	lcl|C08H9.4 CE35826 WBGene0	gi|24580788|	gi|27478394|ref|XP_212094.1|	gi|38079814|ref|XP_354966.1|	gi|6701638|ref|Os01g16300|	gi|6320558|ref|NP_010638.1|	gi|19113485|ref|NP_596693.1|
gi|31195089|ref|XP_306492.1|	lcl|At1g44850.1	lcl|C09B7.2 CE08027 WBGene0	gi|24580806|	gi|27478818|ref|XP_211529.1|	gi|38079972|ref|XP_356904.1|	gi|6701644|ref|Os01g16360|	gi|6320564|ref|NP_010644.1|	gi|19113495|ref|NP_596703.1|
gi|31195097|ref|XP_306496.1|	lcl|At1g44870.1	lcl|C09B8.3 CE02463 WBGene0	gi|24580829|	gi|27478974|ref|XP_211557.1|	gi|38080139|ref|XP_359228.1|	gi|6701650|ref|Os01g16420|	gi|6320574|ref|NP_010654.1|	gi|19113503|ref|NP_596711.1|
gi|31195103|ref|XP_306499.1|	lcl|At1g44875.1	lcl|C09B8.5 CE02465 WBGene0	gi|24580832|	gi|27479014|ref|XP_211983.1|	gi|38080183|ref|XP_358901.1|	gi|6701652|ref|Os01g16440|	gi|6320578|ref|NP_010658.1|	gi|19113504|ref|NP_596712.1|
gi|31195123|ref|XP_306509.1|	lcl|At1g44890.1	lcl|C09B8.8 CE30243 WBGene0	gi|24580859|	gi|27479155|ref|XP_211988.1|	gi|38080213|ref|XP_358912.1|	gi|6701664|ref|Os01g16560|	gi|6320599|ref|NP_010679.1|	gi|19113509|ref|NP_596717.1|
gi|31195125|ref|XP_306510.1|	lcl|At1g44930.1	lcl|C09B9.1 CE06793 WBGene0	gi|24580863|	gi|27479241|ref|XP_212123.1|	gi|38080219|ref|XP_358914.1|	gi|6701667|ref|Os01g16590|	gi|6320615|ref|NP_010695.1|	gi|19113511|ref|NP_596719.1|
gi|31195129|ref|XP_306512.1|	lcl|At1g44990.1	lcl|C09B9.2 CE06794 WBGene0	gi|24580872|	gi|27479311|ref|XP_211995.1|	gi|38080257|ref|XP_358925.1|	gi|6701670|ref|Os01g16620|	gi|6320628|ref|NP_010708.1|	gi|19113515|ref|NP_596723.1|
gi|31195133|ref|XP_306514.1|	lcl|At1g45248.1	lcl|C09D4.2 CE08031 WBGene0	gi|24580874|	gi|27479405|ref|XP_211086.1|	gi|38080384|ref|XP_358738.1|	gi|6701671|ref|Os01g16630|	gi|6320631|ref|NP_010711.1|	gi|19113519|ref|NP_596727.1|
gi|31195151|ref|XP_306523.1|	lcl|At1g45403.1	lcl|C09D4.6 CE08035 WBGene0	gi|24580876|	gi|27479547|ref|XP_211092.1|	gi|38080495|ref|XP_357501.1|	gi|6701672|ref|Os01g16640|	gi|6320645|ref|NP_010725.1|	gi|19113520|ref|NP_596728.1|
gi|31195157|ref|XP_306526.1|	lcl|At1g46336.1	lcl|C09E7.2 CE25767 WBGene0	gi|24580878|	gi|27479549|ref|XP_211108.1|	gi|38080564|ref|XP_358945.1|	gi|6701684|ref|Os01g16760|	gi|6320647|ref|NP_010727.1|	gi|19113541|ref|NP_596749.1|
gi|31195177|ref|XP_306536.1|	lcl|At1g47280.1	lcl|C09E7.6 CE29077 WBGene0	gi|24580880|	gi|27479553|ref|XP_211089.1|	gi|38080612|ref|XP_359234.1|	gi|6701688|ref|Os01g16800|	gi|6320651|ref|NP_010731.1|	gi|19113547|ref|NP_596755.1|
gi|31195181|ref|XP_306538.1|	lcl|At1g47395.1	lcl|C09E8.1a CE31299 WBGene	gi|24580898|	gi|27479555|ref|XP_211090.1|	gi|38080626|ref|XP_359235.1|	gi|6701692|ref|Os01g16840|	gi|6320654|ref|NP_010734.1|	gi|19113551|ref|NP_596759.1|
gi|31195199|ref|XP_306547.1|	lcl|At1g47400.1	lcl|C09E8.1b CE31879 WBGene	gi|24580933|	gi|27480144|ref|XP_211749.1|	gi|38080664|ref|XP_359236.1|	gi|6701706|ref|Os01g16980|	gi|6320669|ref|NP_010749.1|	gi|19113554|ref|NP_596762.1|
gi|31195215|ref|XP_306555.1|	lcl|At1g47410.1	lcl|C09E9.2 CE08037 WBGene0	gi|24580937|	gi|27480484|ref|XP_209640.1|	gi|38080678|ref|XP_358825.1|	gi|6701707|ref|Os01g16990|	gi|6320670|ref|NP_010750.1|	gi|19113558|ref|NP_596766.1|
gi|31195219|ref|XP_306557.1|	lcl|At1g47465.1	lcl|C09F12.1 CE08040 WBGene	gi|24580939|	gi|27480511|ref|XP_211573.1|	gi|38080723|ref|XP_358957.1|	gi|6701719|ref|Os01g17090|	gi|6320684|ref|NP_010764.1|	gi|19113560|ref|NP_596768.1|
gi|31195235|ref|XP_306565.1|	lcl|At1g47485.1	lcl|C09F12.2 CE31409 WBGene	gi|24580943|	gi|27480566|ref|XP_211923.1|	gi|38080745|ref|XP_358966.1|	gi|6701720|ref|Os01g17100|	gi|6320686|ref|NP_010766.1|	gi|19113562|ref|NP_596770.1|
gi|31195237|ref|XP_306566.1|	lcl|At1g47495.1	lcl|C09F5.1 CE29572 WBGene0	gi|24580955|	gi|27481115|ref|XP_212067.1|	gi|38080755|ref|XP_358970.1|	gi|6701721|ref|Os01g17110|	gi|6320688|ref|NP_010768.1|	gi|19113573|ref|NP_596781.1|
gi|31195249|ref|XP_306572.1|	lcl|At1g47660.1	lcl|C09F5.3 CE01775 WBGene0	gi|24580961|	gi|27481158|ref|XP_211518.1|	gi|38080763|ref|XP_355639.1|	gi|6701724|ref|Os01g17140|	gi|6320690|ref|NP_010770.1|	gi|19113574|ref|NP_596782.1|
gi|31195251|ref|XP_306573.1|	lcl|At1g47680.1	lcl|C09F9.1 CE17392 WBGene0	gi|24580976|	gi|27481243|ref|XP_211896.1|	gi|38080781|ref|XP_358639.1|	gi|6701725|ref|Os01g17150|	gi|6320701|ref|NP_010781.1|	gi|19113578|ref|NP_596786.1|
gi|31195265|ref|XP_306580.1|	lcl|At1g47690.1	lcl|C09F9.4 CE17395 WBGene0	gi|24580982|	gi|27482550|ref|XP_211079.1|	gi|38080833|ref|XP_359240.1|	gi|6701733|ref|Os01g17210|	gi|6320703|ref|NP_010783.1|	gi|19113588|ref|NP_596796.1|
gi|31195289|ref|XP_306592.1|	lcl|At1g47700.1	lcl|C09G1.1 CE37313 WBGene0	gi|24580984|	gi|27483467|ref|XP_211174.1|	gi|38080845|ref|XP_358984.1|	gi|6701734|ref|Os01g17220|	gi|6320707|ref|NP_010787.1|	gi|19113591|ref|NP_596799.1|
gi|31195297|ref|XP_306596.1|	lcl|At1g47770.1	lcl|C09G1.2 CE02127 WBGene0	gi|24580996|	gi|27483665|ref|XP_212013.1|	gi|38080849|ref|XP_358986.1|	gi|6701735|ref|Os01g17230|	gi|6320712|ref|NP_010792.1|	gi|19113593|ref|NP_596801.1|
gi|31195309|ref|XP_306602.1|	lcl|At1g47783.1	lcl|C09G1.3 CE02128 WBGene0	gi|24581004|	gi|27484057|ref|XP_211339.1|	gi|38080891|ref|XP_359002.1|	gi|6701742|ref|Os01g17300|	gi|6320721|ref|NP_010800.1|	gi|19113594|ref|NP_596802.1|
gi|31195333|ref|XP_306614.1|	lcl|At1g47813.1	lcl|C09G1.5 CE37314 WBGene0	gi|24581029|	gi|27484364|ref|XP_211345.1|	gi|38080901|ref|XP_359005.1|	gi|6701753|ref|Os01g17410|	gi|6320734|ref|NP_010814.1|	gi|19113606|ref|NP_596814.1|
gi|31195347|ref|XP_306621.1|	lcl|At1g47970.1	lcl|C09G12.5 CE16839 WBGene	gi|24581032|	gi|27484665|ref|XP_211197.1|	gi|38080992|ref|XP_359024.1|	gi|6701754|ref|Os01g17420|	gi|6320737|ref|NP_010817.1|	gi|19113607|ref|NP_596815.1|
gi|31195353|ref|XP_306624.1|	lcl|At1g48070.1	lcl|C09G4.4 CE03981 WBGene0	gi|24581071|	gi|27485050|ref|XP_211422.1|	gi|38081008|ref|XP_359030.1|	gi|6701761|ref|Os01g17980|	gi|6320741|ref|NP_010821.1|	gi|19113608|ref|NP_596816.1|
gi|31195371|ref|XP_306633.1|	lcl|At1g48095.1	lcl|C09G5.7 CE01487 WBGene0	gi|24581091|	gi|27485088|ref|XP_211413.1|	gi|38081014|ref|XP_359033.1|	gi|6701764|ref|Os01g18010|	gi|6320743|ref|NP_010823.1|	gi|19113616|ref|NP_596824.1|
gi|31195389|ref|XP_306642.1|	lcl|At1g48145.1	lcl|C09G5.8 CE25774 WBGene0	gi|24581122|	gi|27485210|ref|XP_211432.1|	gi|38081032|ref|XP_355645.1|	gi|6701771|ref|Os01g18080|	gi|6320749|ref|NP_010829.1|	gi|19113622|ref|NP_596830.1|
gi|31195415|ref|XP_306655.1|	lcl|At1g48190.1	lcl|C09G9.1 CE03000 WBGene0	gi|24581148|	gi|27485565|ref|XP_211028.1|	gi|38081060|ref|XP_359046.1|	gi|6701772|ref|Os01g18090|	gi|6320760|ref|NP_010839.1|	gi|19113625|ref|NP_596833.1|
gi|31195419|ref|XP_306657.1|	lcl|At1g48250.1	lcl|C09G9.5 CE03004 WBGene0	gi|24581170|	gi|27486286|ref|XP_211694.1|	gi|38081086|ref|XP_359052.1|	gi|6701776|ref|Os01g18130|	gi|6320762|ref|NP_010841.1|	gi|19113637|ref|NP_596845.1|
gi|31195451|ref|XP_306673.1|	lcl|At1g48330.1	lcl|C09G9.8 CE34677 WBGene0	gi|24581208|	gi|27497893|ref|XP_211764.1|	gi|38081092|ref|XP_357514.1|	gi|6701777|ref|Os01g18140|	gi|6320763|ref|NP_010842.1|	gi|19113640|ref|NP_596848.1|
gi|31195465|ref|XP_306680.1|	lcl|At1g48390.1	lcl|C09H10.1 CE28533 WBGene	gi|24581303|	gi|27498383|ref|XP_212022.1|	gi|38081148|ref|XP_359069.1|	gi|6701779|ref|Os01g18160|	gi|6320778|ref|NP_010857.1|	gi|19113646|ref|NP_596854.1|
gi|31195471|ref|XP_306683.1|	lcl|At1g48405.1	lcl|C09H10.5 CE28534 WBGene	gi|24581343|	gi|27499116|ref|XP_169057.2|	gi|38081164|ref|XP_359076.1|	gi|6701782|ref|Os01g18190|	gi|6320800|ref|NP_010879.1|	gi|19113655|ref|NP_596863.1|
gi|31195483|ref|XP_306689.1|	lcl|At1g48560.1	lcl|C09H10.7 CE02136 WBGene	gi|24581370|	gi|27499155|ref|XP_210906.1|	gi|38081209|ref|XP_359081.1|	gi|6701788|ref|Os01g18250|	gi|6320812|ref|NP_010891.1|	gi|19113656|ref|NP_596864.1|
gi|31195507|ref|XP_306701.1|	lcl|At1g48610.1	lcl|C09H10.9 CE03008 WBGene	gi|24581372|	gi|27499638|ref|XP_211040.1|	gi|38081213|ref|XP_359083.1|	gi|6701793|ref|Os01g18300|	gi|6320818|ref|NP_010897.1|	gi|19113657|ref|NP_596865.1|
gi|31195523|ref|XP_306709.1|	lcl|At1g48730.1	lcl|C09H5.1 CE08043 WBGene0	gi|24581374|	gi|27500052|ref|XP_211244.1|	gi|38081215|ref|XP_359084.1|	gi|6701794|ref|Os01g18310|	gi|6320819|ref|NP_010898.1|	gi|19113676|ref|NP_592764.1|
gi|31195539|ref|XP_306717.1|	lcl|At1g48770.1	lcl|C10A4.2 CE02470 WBGene0	gi|24581411|	gi|27500084|ref|XP_211251.1|	gi|38081217|ref|XP_359085.1|	gi|6701796|ref|Os01g18330|	gi|6320820|ref|NP_010899.1|	gi|19113677|ref|NP_592765.1|
gi|31195543|ref|XP_306719.1|	lcl|At1g48780.1	lcl|C10A4.3 CE02471 WBGene0	gi|24581415|	gi|27500536|ref|XP_097065.2|	gi|38081239|ref|XP_359094.1|	gi|6701797|ref|Os01g18340|	gi|6320832|ref|NP_010911.1|	gi|19113682|ref|NP_592770.1|
gi|31195547|ref|XP_306721.1|	lcl|At1g49005.1	lcl|C10A4.4 CE02472 WBGene0	gi|24581425|	gi|27501308|ref|XP_210035.1|	gi|38081349|ref|XP_359146.1|	gi|6701798|ref|Os01g18350|	gi|6320836|ref|NP_010915.1|	gi|19113686|ref|NP_592774.1|
gi|31195551|ref|XP_306723.1|	lcl|At1g49110.1	lcl|C10A4.5 CE29079 WBGene0	gi|24581438|	gi|27501415|ref|XP_212326.1|	gi|38081393|ref|XP_359153.1|	gi|6701800|ref|Os01g18370|	gi|6320845|ref|NP_010924.1|	gi|19113687|ref|NP_592775.1|
gi|31195555|ref|XP_306725.1|	lcl|At1g49150.1	lcl|C10A4.6 CE02474 WBGene0	gi|24581449|	gi|27734691|ref|NP_775940.1|	gi|38081399|ref|XP_359156.1|	gi|6701801|ref|Os01g18380|	gi|6320855|ref|NP_010934.1|	gi|19113691|ref|NP_592779.1|
gi|31195557|ref|XP_306726.1|	lcl|At1g49245.1	lcl|C10A4.7 CE02475 WBGene0	gi|24581497|	gi|27734705|ref|NP_775939.1|	gi|38081431|ref|XP_359170.1|	gi|6701802|ref|Os01g18390|	gi|6320860|ref|NP_010939.1|	gi|19113701|ref|NP_592789.1|
gi|31195571|ref|XP_306733.1|	lcl|At1g49260.1	lcl|C10B5.3 CE35228 WBGene0	gi|24581522|	gi|27734707|ref|NP_775881.1|	gi|38081433|ref|XP_359171.1|	gi|6701804|ref|Os01g18410|	gi|6320868|ref|NP_010947.1|	gi|19113703|ref|NP_592791.1|
gi|31195573|ref|XP_306734.1|	lcl|At1g49310.1	lcl|C10C5.7 CE34678 WBGene0	gi|24581572|	gi|27734732|ref|NP_775888.1|	gi|38081439|ref|XP_359174.1|	gi|6701806|ref|Os01g18430|	gi|6320871|ref|NP_010950.1|	gi|19113706|ref|NP_592794.1|
gi|31195579|ref|XP_306737.1|	lcl|At1g49410.1	lcl|C10C6.3 CE35392 WBGene0	gi|24581574|	gi|27734742|ref|NP_775892.1|	gi|38081453|ref|XP_359181.1|	gi|6701808|ref|Os01g18450|	gi|6320872|ref|NP_010951.1|	gi|19113707|ref|NP_592795.1|
gi|31195585|ref|XP_306740.1|	lcl|At1g49500.1	lcl|C10C6.7 CE32794 WBGene0	gi|24581576|	gi|27734763|ref|NP_775875.1|	gi|38081483|ref|XP_359195.1|	gi|6701809|ref|Os01g18460|	gi|6320873|ref|NP_010952.1|	gi|19113709|ref|NP_592797.1|
gi|31195595|ref|XP_306745.1|	lcl|At1g49510.1	lcl|C10E2.2 CE08061 WBGene0	gi|24581600|	gi|27734787|ref|NP_775861.1|	gi|38081492|ref|XP_359197.1|	gi|6701812|ref|Os01g18490|	gi|6320876|ref|NP_010955.1|	gi|19113710|ref|NP_592798.1|
gi|31195597|ref|XP_306746.1|	lcl|At1g49680.1	lcl|C10E2.4 CE08063 WBGene0	gi|24581694|	gi|27734795|ref|NP_775938.1|	gi|38081515|ref|XP_358885.1|	gi|6701813|ref|Os01g18500|	gi|6320884|ref|NP_010963.1|	gi|19113723|ref|NP_592811.1|
gi|31195599|ref|XP_306747.1|	lcl|At1g49700.1	lcl|C10E2.5 CE35394 WBGene0	gi|24581704|	gi|27734803|ref|NP_775967.1|	gi|38081557|ref|XP_359213.1|	gi|6701816|ref|Os01g18530|	gi|6320900|ref|NP_010979.1|	gi|19113732|ref|NP_592820.1|
gi|31195601|ref|XP_306748.1|	lcl|At1g49800.1	lcl|C10F3.1 CE08066 WBGene0	gi|24581713|	gi|27734813|ref|NP_775850.1|	gi|38081627|ref|XP_196166.2|	gi|6701817|ref|Os01g18540|	gi|6320910|ref|NP_010990.1|	gi|19113751|ref|NP_592839.1|
gi|31195603|ref|XP_306749.1|	lcl|At1g49830.1	lcl|C10F3.7 CE32581 WBGene0	gi|24581719|	gi|27734817|ref|NP_775851.1|	gi|38081670|ref|XP_289726.2|	gi|6701819|ref|Os01g18560|	gi|6320915|ref|NP_010994.1|	gi|19113762|ref|NP_592850.1|
gi|31195625|ref|XP_306760.1|	lcl|At1g49930.1	lcl|C10G11.1 CE34257 WBGene	gi|24581742|	gi|27734819|ref|NP_775848.1|	gi|38081694|ref|XP_358497.1|	gi|6701821|ref|Os01g18580|	gi|6320920|ref|NP_010999.1|	gi|19113767|ref|NP_592855.1|
gi|31195645|ref|XP_306770.1|	lcl|At1g49940.1	lcl|C10G11.10 CE08089 WBGen	gi|24581787|	gi|27734821|ref|NP_775849.1|	gi|38081723|ref|XP_358498.1|	gi|6701824|ref|Os01g18610|	gi|6320921|ref|NP_011000.1|	gi|19113770|ref|NP_592858.1|
gi|31195667|ref|XP_306781.1|	lcl|At1g49990.1	lcl|C10G11.6 CE32124 WBGene	gi|24581789|	gi|27734835|ref|NP_775842.1|	gi|38081876|ref|XP_132351.2|	gi|6701837|ref|Os01g18720|	gi|6320923|ref|NP_011002.1|	gi|19113772|ref|NP_592861.1|
gi|31195679|ref|XP_306787.1|	lcl|At1g50080.1	lcl|C10G11.9 CE08088 WBGene	gi|24581877|	gi|27734843|ref|NP_775932.1|	gi|38082055|ref|XP_128528.2|	gi|6701838|ref|Os01g18730|	gi|6320924|ref|NP_011003.1|	gi|19113780|ref|NP_592868.1|
gi|31195681|ref|XP_306788.1|	lcl|At1g50160.1	lcl|C10G8.8a CE08079 WBGene	gi|24581895|	gi|27734915|ref|NP_775812.1|	gi|38082129|ref|XP_358499.1|	gi|6701843|ref|Os01g18780|	gi|6320929|ref|NP_011008.1|	gi|19113784|ref|NP_592872.1|
gi|31195685|ref|XP_306790.1|	lcl|At1g50220.1	lcl|C10G8.8b CE32584 WBGene	gi|24581929|	gi|27734963|ref|NP_775793.1|	gi|38082156|ref|XP_358502.1|	gi|6701844|ref|Os01g18790|	gi|6320938|ref|NP_011017.1|	gi|19113787|ref|NP_592875.1|
gi|31195687|ref|XP_306791.1|	lcl|At1g50290.1	lcl|C10H11.10 CE08099 WBGen	gi|24581931|	gi|27734969|ref|NP_775948.1|	gi|38082186|ref|XP_358504.1|	gi|6701849|ref|Os01g18810|	gi|6320940|ref|NP_011019.1|	gi|19113801|ref|NP_592889.1|
gi|31195695|ref|XP_306795.1|	lcl|At1g50340.1	lcl|C11D2.1 CE24799 WBGene0	gi|24581970|	gi|27734975|ref|NP_775923.1|	gi|38082307|ref|XP_358507.1|	gi|6701850|ref|Os01g18820|	gi|6320951|ref|NP_011030.1|	gi|19113803|ref|NP_592891.1|
gi|31195697|ref|XP_306796.1|	lcl|At1g50350.1	lcl|C11D2.3 CE32307 WBGene0	gi|24581976|	gi|27735002|ref|NP_775944.1|	gi|38082671|ref|XP_358649.1|	gi|6701851|ref|Os01g18830|	gi|6320953|ref|NP_011032.1|	gi|19113811|ref|NP_592899.1|
gi|31195699|ref|XP_306797.1|	lcl|At1g50470.1	lcl|C11E4.7 CE08107 WBGene0	gi|24581978|	gi|27735031|ref|NP_775910.1|	gi|38082688|ref|XP_357569.1|	gi|6701864|ref|Os01g18940|	gi|6320961|ref|NP_011040.1|	gi|19113813|ref|NP_592901.1|
gi|31195701|ref|XP_306798.1|	lcl|At1g50530.1	lcl|C11E4.8 CE34679 WBGene0	gi|24581980|	gi|27735087|ref|NP_775915.1|	gi|38083083|ref|XP_359415.1|	gi|6701866|ref|Os01g18960|	gi|6320975|ref|NP_011054.1|	gi|19113814|ref|NP_592902.1|
gi|31195709|ref|XP_306802.1|	lcl|At1g50800.1	lcl|C11G10.1 CE03015 WBGene	gi|24581983|	gi|27735105|ref|NP_775769.1|	gi|38083235|ref|XP_148827.2|	gi|6701869|ref|Os01g18990|	gi|6320984|ref|NP_011063.1|	gi|19113816|ref|NP_592904.1|
gi|31195711|ref|XP_306803.1|	lcl|At1g50930.1	lcl|C11G10.2 CE03016 WBGene	gi|24582000|	gi|27735123|ref|NP_775958.1|	gi|38083591|ref|XP_358513.1|	gi|6701871|ref|Os01g19010|	gi|6320986|ref|NP_011065.1|	gi|19113827|ref|NP_592915.1|
gi|31195717|ref|XP_306806.1|	lcl|At1g51000.1	lcl|C11H1.8 CE31414 WBGene0	gi|24582010|	gi|27735135|ref|NP_775784.1|	gi|38083642|ref|XP_355074.1|	gi|6701876|ref|Os01g19060|	gi|6320995|ref|NP_011074.1|	gi|19113830|ref|NP_592918.1|
gi|31195719|ref|XP_306807.1|	lcl|At1g51010.1	lcl|C12D12.1a CE27679 WBGen	gi|24582025|	gi|27735141|ref|NP_775946.1|	gi|38083681|ref|XP_358650.1|	gi|6701878|ref|Os01g19080|	gi|6320997|ref|NP_011076.1|	gi|19113837|ref|NP_592925.1|
gi|31195723|ref|XP_306809.1|	lcl|At1g51030.1	lcl|C12D12.1b CE29576 WBGen	gi|24582030|	gi|27735147|ref|NP_775955.1|	gi|38083760|ref|XP_357597.1|	gi|6701879|ref|Os01g19090|	gi|6320998|ref|NP_011077.1|	gi|19113839|ref|NP_592927.1|
gi|31195729|ref|XP_306812.1|	lcl|At1g51355.1	lcl|C12D12.3 CE06815 WBGene	gi|24582041|	gi|27754180|ref|NP_776088.1|	gi|38083785|ref|XP_358518.1|	gi|6701891|ref|Os01g19180|	gi|6321006|ref|NP_011085.1|	gi|19113846|ref|NP_592934.1|
gi|31195819|ref|XP_306857.1|	lcl|At1g51400.1	lcl|C12D12.4 CE06816 WBGene	gi|24582046|	gi|27764904|ref|NP_775183.1|	gi|38083837|ref|XP_357010.1|	gi|6701896|ref|Os01g19230|	gi|6321009|ref|NP_011088.1|	gi|19113849|ref|NP_592937.1|
gi|31195821|ref|XP_306858.1|	lcl|At1g51430.1	lcl|C12D8.13 CE34680 WBGene	gi|24582048|	gi|28144912|ref|NP_777369.1|	gi|38083896|ref|XP_358519.1|	gi|6701898|ref|Os01g19250|	gi|6321015|ref|NP_011094.1|	gi|19113850|ref|NP_592938.1|
gi|31195833|ref|XP_306864.1|	lcl|At1g51530.1	lcl|C12D8.14 CE34681 WBGene	gi|24582050|	gi|28212220|ref|NP_780778.1|	gi|38084306|ref|XP_358741.1|	gi|6701900|ref|Os01g19270|	gi|6321028|ref|NP_011107.1|	gi|19113856|ref|NP_592944.1|
gi|31195847|ref|XP_306871.1|	lcl|At1g51915.1	lcl|C12D8.15 CE34682 WBGene	gi|24582059|	gi|28212280|ref|NP_777603.1|	gi|38084421|ref|XP_358521.1|	gi|6701901|ref|Os01g19280|	gi|6321034|ref|NP_011113.1|	gi|19113858|ref|NP_592946.1|
gi|31195855|ref|XP_306875.1|	lcl|At1g51920.1	lcl|C12D8.16 CE34683 WBGene	gi|24582061|	gi|28269683|ref|NP_783315.1|	gi|38084524|ref|XP_358366.1|	gi|6701903|ref|Os01g19300|	gi|6321035|ref|NP_011114.1|	gi|19113866|ref|NP_592954.1|
gi|31195857|ref|XP_306876.1|	lcl|At1g51970.1	lcl|C12D8.17 CE34684 WBGene	gi|24582065|	gi|28269705|ref|NP_777639.1|	gi|38084754|ref|XP_358523.1|	gi|6701905|ref|Os01g19320|	gi|6321037|ref|NP_011116.1|	gi|19113868|ref|NP_592956.1|
gi|31195869|ref|XP_306882.1|	lcl|At1g52010.1	lcl|C12D8.6 CE05270 WBGene0	gi|24582164|	gi|28274703|ref|NP_777561.1|	gi|38084843|ref|XP_358526.1|	gi|6701907|ref|Os01g19340|	gi|6321175|ref|NP_011252.1|	gi|19113870|ref|NP_592958.1|
gi|31195893|ref|XP_306894.1|	lcl|At1g52090.1	lcl|C12D8.9 CE34157 WBGene0	gi|24582166|	gi|28274705|ref|NP_777611.1|	gi|38084936|ref|XP_358367.1|	gi|6701908|ref|Os01g19350|	gi|6321188|ref|NP_011265.1|	gi|19113883|ref|NP_592971.1|
gi|31195899|ref|XP_306897.1|	lcl|At1g52140.1	lcl|C13A10.1 CE08123 WBGene	gi|24582168|	gi|28372517|ref|NP_778251.1|	gi|38084942|ref|XP_358653.1|	gi|6701910|ref|Os01g19370|	gi|6321207|ref|NP_011284.1|	gi|19113887|ref|NP_592975.1|
gi|31195901|ref|XP_306898.1|	lcl|At1g52270.1	lcl|C13A2.7 CE35233 WBGene0	gi|24582170|	gi|28376666|ref|NP_777566.1|	gi|38085009|ref|XP_358655.1|	gi|6701923|ref|Os01g19500|	gi|6321211|ref|NP_011288.1|	gi|19113909|ref|NP_592997.1|
gi|31195903|ref|XP_306899.1|	lcl|At1g52390.1	lcl|C13B7.6 CE27681 WBGene0	gi|24582178|	gi|28395037|ref|NP_783316.1|	gi|38085017|ref|XP_358656.1|	gi|6701926|ref|Os01g19530|	gi|6321216|ref|NP_011293.1|	gi|19113910|ref|NP_592998.1|
gi|31195907|ref|XP_306901.1|	lcl|At1g52410.1	lcl|C13C12.2 CE08133 WBGene	gi|24582186|	gi|28395041|ref|NP_775934.1|	gi|38085081|ref|XP_358528.1|	gi|6701927|ref|Os01g19540|	gi|6321220|ref|NP_011296.1|	gi|19113922|ref|NP_593010.1|
gi|31195909|ref|XP_306902.1|	lcl|At1g52410.2	lcl|C13E3.1 CE30246 WBGene0	gi|24582207|	gi|28395053|ref|NP_776181.1|	gi|38085155|ref|XP_358529.1|	gi|6701928|ref|Os01g19550|	gi|6321263|ref|NP_011340.1|	gi|19113923|ref|NP_593011.1|
gi|31195927|ref|XP_306911.1|	lcl|At1g52415.1	lcl|C13F10.1a CE29482 WBGen	gi|24582212|	gi|28557701|ref|NP_787076.1|	gi|38085193|ref|XP_355168.1|	gi|6701930|ref|Os01g19570|	gi|6321268|ref|NP_011345.1|	gi|19113926|ref|NP_593014.1|
gi|31195939|ref|XP_306917.1|	lcl|At1g52550.1	lcl|C13F10.1b CE29483 WBGen	gi|24582214|	gi|28557713|ref|NP_787085.1|	gi|38085195|ref|XP_358531.1|	gi|6701931|ref|Os01g19580|	gi|6321270|ref|NP_011347.1|	gi|19113932|ref|NP_593020.1|
gi|31196003|ref|XP_306949.1|	lcl|At1g52565.1	lcl|C13F10.7 CE08148 WBGene	gi|24582255|	gi|28557715|ref|NP_787087.1|	gi|38085344|ref|XP_357075.1|	gi|6701936|ref|Os01g19630|	gi|6321276|ref|NP_011353.1|	gi|19113939|ref|NP_593027.1|
gi|31196019|ref|XP_306957.1|	lcl|At1g52680.1	lcl|C13G3.1 CE05277 WBGene0	gi|24582283|	gi|28557719|ref|NP_787089.1|	gi|38085357|ref|XP_355821.1|	gi|6701937|ref|Os01g19640|	gi|6321277|ref|NP_011354.1|	gi|19113942|ref|NP_593030.1|
gi|31196021|ref|XP_306958.1|	lcl|At1g52710.1	lcl|C13G5.2 CE29086 WBGene0	gi|24582317|	gi|28557723|ref|NP_787091.1|	gi|38085435|ref|XP_357678.1|	gi|6701940|ref|Os01g19670|	gi|6321279|ref|NP_011356.1|	gi|19113956|ref|NP_593044.1|
gi|31196025|ref|XP_306960.1|	lcl|At1g52840.1	lcl|C14A11.2 CE08150 WBGene	gi|24582333|	gi|28557725|ref|NP_787092.1|	gi|38085466|ref|XP_358660.1|	gi|6701946|ref|Os01g19730|	gi|6321287|ref|NP_011364.1|	gi|19113964|ref|NP_593052.1|
gi|31196039|ref|XP_306967.1|	lcl|At1g53035.1	lcl|C14A11.5 CE08153 WBGene	gi|24582335|	gi|28557727|ref|NP_787093.1|	gi|38085480|ref|XP_358534.1|	gi|6701951|ref|Os01g19780|	gi|6321292|ref|NP_011369.1|	gi|19113972|ref|NP_593060.1|
gi|31196059|ref|XP_306977.1|	lcl|At1g53180.1	lcl|C14A11.6 CE08154 WBGene	gi|24582346|	gi|28557729|ref|NP_787094.1|	gi|38085611|ref|XP_355833.1|	gi|6701952|ref|Os01g19790|	gi|6321298|ref|NP_011375.1|	gi|19113975|ref|NP_593063.1|
gi|31196075|ref|XP_306985.1|	lcl|At1g53265.1	lcl|C14A4.12a CE30599 WBGen	gi|24582362|	gi|28557735|ref|NP_787097.1|	gi|38085613|ref|XP_355834.1|	gi|6701954|ref|Os01g19810|	gi|6321300|ref|NP_011377.1|	gi|19113987|ref|NP_593075.1|
gi|31196087|ref|XP_306991.1|	lcl|At1g53325.1	lcl|C14A4.12b CE02141 WBGen	gi|24582383|	gi|28557737|ref|NP_787099.1|	gi|38085626|ref|XP_355835.1|	gi|6701956|ref|Os01g19830|	gi|6321309|ref|NP_011386.1|	gi|19113993|ref|NP_593081.1|
gi|31196091|ref|XP_306993.1|	lcl|At1g53480.1	lcl|C14A4.6 CE02147 WBGene0	gi|24582389|	gi|28557753|ref|NP_787108.1|	gi|38085736|ref|XP_357693.1|	gi|6701959|ref|Os01g19860|	gi|6321312|ref|NP_011389.1|	gi|19113994|ref|NP_593082.1|
gi|31196101|ref|XP_306998.1|	lcl|At1g53610.1	lcl|C14A4.7a CE02148 WBGene	gi|24582391|	gi|28557757|ref|NP_787110.1|	gi|38085891|ref|XP_159101.2|	gi|6701961|ref|Os01g19880|	gi|6321316|ref|NP_011393.1|	gi|19114000|ref|NP_593088.1|
gi|31196113|ref|XP_307004.1|	lcl|At1g53620.1	lcl|C14A4.7b CE36101 WBGene	gi|24582402|	gi|28557759|ref|NP_787111.1|	gi|38085930|ref|XP_358662.1|	gi|6701964|ref|Os01g19910|	gi|6321317|ref|NP_011394.1|	gi|19114016|ref|NP_593104.1|
gi|31196115|ref|XP_307005.1|	lcl|At1g53625.1	lcl|C14A4.9 CE02150 WBGene0	gi|24582404|	gi|28557763|ref|NP_787113.1|	gi|38086020|ref|XP_358386.1|	gi|6701968|ref|Os01g19950|	gi|6321321|ref|NP_011398.1|	gi|19114019|ref|NP_593107.1|
gi|31196145|ref|XP_307020.1|	lcl|At1g53640.1	lcl|C14A6.5 CE18496 WBGene0	gi|24582429|	gi|28557765|ref|NP_787114.1|	gi|38086024|ref|XP_358715.1|	gi|6701971|ref|Os01g19980|	gi|6321325|ref|NP_011402.1|	gi|19114025|ref|NP_593113.1|
gi|31196149|ref|XP_307022.1|	lcl|At1g53705.1	lcl|C14A6.6 CE18497 WBGene0	gi|24582439|	gi|28557767|ref|NP_787115.1|	gi|38086119|ref|XP_358201.1|	gi|6701973|ref|Os01g20000|	gi|6321330|ref|NP_011407.1|	gi|19114038|ref|NP_593126.1|
gi|31196153|ref|XP_307024.1|	lcl|At1g53935.1	lcl|C14A6.7 CE18498 WBGene0	gi|24582448|	gi|28557769|ref|NP_787116.1|	gi|38086293|ref|XP_288148.2|	gi|6701974|ref|Os01g20010|	gi|6321340|ref|NP_011417.1|	gi|19114040|ref|NP_593128.1|
gi|31196155|ref|XP_307025.1|	lcl|At1g53970.1	lcl|C14A6.8 CE18499 WBGene0	gi|24582453|	gi|28557771|ref|NP_787117.1|	gi|38086300|ref|XP_356326.1|	gi|6701975|ref|Os01g20020|	gi|6321348|ref|NP_011425.1|	gi|19114045|ref|NP_593133.1|
gi|31196171|ref|XP_307033.1|	lcl|At1g54120.1	lcl|C14B1.2 CE00899 WBGene0	gi|24582464|	gi|28557775|ref|NP_787119.1|	gi|38086338|ref|XP_356332.1|	gi|6701979|ref|Os01g20060|	gi|6321359|ref|NP_011436.1|	gi|19114061|ref|NP_593149.1|
gi|31196189|ref|XP_307042.1|	lcl|At1g54200.1	lcl|C14B1.6 CE31415 WBGene0	gi|24582469|	gi|28603832|ref|NP_776157.1|	gi|38086380|ref|XP_358210.1|	gi|6701983|ref|Os01g20100|	gi|6321363|ref|NP_011440.1|	gi|19114066|ref|NP_593155.1|
gi|31196191|ref|XP_307043.1|	lcl|At1g54215.1	lcl|C14B1.8 CE00905 WBGene0	gi|24582471|	gi|28603862|ref|NP_777599.1|	gi|38086472|ref|XP_358718.1|	gi|6701987|ref|Os01g20140|	gi|6321367|ref|NP_011444.1|	gi|19114067|ref|NP_593154.1|
gi|31196193|ref|XP_307044.1|	lcl|At1g54230.1	lcl|C14B1.9 CE00906 WBGene0	gi|24582473|	gi|28866947|ref|NP_005940.1|	gi|38086474|ref|XP_358719.1|	gi|6701990|ref|Os01g20170|	gi|6321377|ref|NP_011454.1|	gi|19114068|ref|NP_593156.1|
gi|31196195|ref|XP_307045.1|	lcl|At1g54410.1	lcl|C14B4.1 CE15616 WBGene0	gi|24582518|	gi|28866960|ref|NP_005937.1|	gi|38086478|ref|XP_356344.1|	gi|6701991|ref|Os01g20180|	gi|6321378|ref|NP_011455.1|	gi|19114089|ref|NP_593177.1|

gi|31196197|ref|XP_307046.1|	lcl|At1g54420.1	lcl|C14B4.2 CE18500 WBGene0	gi|24582528|	gi|28866966|ref|NP_789846.1|	gi|38086482|ref|XP_356346.1|	gi|6701994|ref|Os01g20700|	gi|6321402|ref|NP_011479.1|	gi|19114091|ref|NP_593179.1|
gi|31196199|ref|XP_307047.1|	lcl|At1g54530.1	lcl|C14B9.10 CE30600 WBGene	gi|24582530|	gi|29126236|ref|NP_777607.2|	gi|38086521|ref|XP_358666.1|	gi|6701995|ref|Os01g20710|	gi|6321406|ref|NP_011483.1|	gi|19114107|ref|NP_593195.1|
gi|31196207|ref|XP_307051.1|	lcl|At1g54640.1	lcl|C14B9.3 CE26868 WBGene0	gi|24582571|	gi|29171298|ref|NP_808217.1|	gi|38086754|ref|XP_282884.2|	gi|6701999|ref|Os01g20750|	gi|6321423|ref|NP_011500.1|	gi|19114110|ref|NP_593198.1|
gi|31196235|ref|XP_307065.1|	lcl|At1g54700.1	lcl|C14C10.2 CE28432 WBGene	gi|24582594|	gi|29727359|ref|XP_295034.1|	gi|38086819|ref|XP_358670.1|	gi|6702003|ref|Os01g20780|	gi|6321433|ref|NP_011510.1|	gi|19114112|ref|NP_593200.1|
gi|31196249|ref|XP_307072.1|	lcl|At1g54720.1	lcl|C14C10.3 CE05282 WBGene	gi|24582596|	gi|29729488|ref|XP_295091.1|	gi|38086860|ref|XP_358674.1|	gi|6702004|ref|Os01g20790|	gi|6321445|ref|NP_011522.1|	gi|19114127|ref|NP_593215.1|
gi|31196251|ref|XP_307073.1|	lcl|At1g54880.1	lcl|C14C10.6 CE28204 WBGene	gi|24582626|	gi|29730134|ref|XP_295059.1|	gi|38086892|ref|XP_136124.2|	gi|6702005|ref|Os01g20800|	gi|6321451|ref|NP_011528.1|	gi|19114138|ref|NP_593226.1|
gi|31196261|ref|XP_307078.1|	lcl|At1g54923.1	lcl|C14C11.1 CE06820 WBGene	gi|24582637|	gi|29730467|ref|XP_295126.1|	gi|38086972|ref|XP_358722.1|	gi|6702006|ref|Os01g20810|	gi|6321453|ref|NP_011530.1|	gi|19114139|ref|NP_593227.1|
gi|31196277|ref|XP_307086.1|	lcl|At1g54926.1	lcl|C14C11.2 CE06821 WBGene	gi|24582639|	gi|29730669|ref|XP_295097.1|	gi|38087044|ref|XP_358675.1|	gi|6702007|ref|Os01g20820|	gi|6321463|ref|NP_011540.1|	gi|19114140|ref|NP_593228.1|
gi|31196279|ref|XP_307087.1|	lcl|At1g54950.1	lcl|C14C11.7 CE06826 WBGene	gi|24582705|	gi|29731087|ref|XP_295062.1|	gi|38087119|ref|XP_358248.1|	gi|6702012|ref|Os01g20870|	gi|6321467|ref|NP_011544.1|	gi|19114141|ref|NP_593229.1|
gi|31196285|ref|XP_307090.1|	lcl|At1g55220.1	lcl|C14C11.8 CE36269 WBGene	gi|24582707|	gi|29731719|ref|XP_291017.1|	gi|38087184|ref|XP_358388.1|	gi|6702014|ref|Os01g20890|	gi|6321470|ref|NP_011547.1|	gi|19114144|ref|NP_593232.1|
gi|31196287|ref|XP_307091.1|	lcl|At1g55330.1	lcl|C14C6.13 CE08168 WBGene	gi|24582746|	gi|29731973|ref|XP_291075.1|	gi|38087197|ref|XP_141928.3|	gi|6702017|ref|Os01g20920|	gi|6321472|ref|NP_011549.1|	gi|19114170|ref|NP_593258.1|
gi|31196293|ref|XP_307094.1|	lcl|At1g55365.1	lcl|C14E2.1 CE35396 WBGene0	gi|24582788|	gi|29732624|ref|XP_295257.1|	gi|38087203|ref|XP_358723.1|	gi|6702021|ref|Os01g20960|	gi|6321478|ref|NP_011555.1|	gi|19114178|ref|NP_593266.1|
gi|31196297|ref|XP_307096.1|	lcl|At1g55400.1	lcl|C14E2.3 CE06830 WBGene0	gi|24582793|	gi|29733992|ref|XP_295261.1|	gi|38087249|ref|XP_358724.1|	gi|6702026|ref|Os01g21000|	gi|6321479|ref|NP_011556.1|	gi|19114187|ref|NP_593275.1|
gi|31196303|ref|XP_307099.1|	lcl|At1g55540.1	lcl|C14E2.5 CE35397 WBGene0	gi|24582813|	gi|29734413|ref|XP_114481.4|	gi|38087309|ref|XP_358262.1|	gi|6702028|ref|Os01g21020|	gi|6321482|ref|NP_011559.1|	gi|19114188|ref|NP_593276.1|
gi|31196315|ref|XP_307105.1|	lcl|At1g55710.1	lcl|C14F11.3 CE29579 WBGene	gi|24582828|	gi|29736038|ref|XP_290609.1|	gi|38087322|ref|XP_358389.1|	gi|6702032|ref|Os01g21060|	gi|6321486|ref|NP_011563.1|	gi|19114194|ref|NP_593282.1|
gi|31196323|ref|XP_307109.1|	lcl|At1g55780.1	lcl|C14F11.4a CE37197 WBGen	gi|24582830|	gi|29736050|ref|XP_294743.1|	gi|38087857|ref|XP_358681.1|	gi|6702036|ref|Os01g21100|	gi|6321490|ref|NP_011567.1|	gi|19114196|ref|NP_593284.1|
gi|31196325|ref|XP_307110.1|	lcl|At1g55990.1	lcl|C14F11.4b CE37198 WBGen	gi|24582832|	gi|29736726|ref|XP_295252.1|	gi|38087915|ref|XP_132963.2|	gi|6702037|ref|Os01g21110|	gi|6321499|ref|NP_011576.1|	gi|19114206|ref|NP_593294.1|
gi|31196329|ref|XP_307112.1|	lcl|At1g56085.1	lcl|C14F11.7 CE27685 WBGene	gi|24582839|	gi|29738587|ref|XP_294854.1|	gi|38088201|ref|XP_358683.1|	gi|6702039|ref|Os01g21130|	gi|6321503|ref|NP_011580.1|	gi|19114223|ref|NP_593311.1|
gi|31196331|ref|XP_307113.1|	lcl|At1g56100.1	lcl|C14F5.1a CE01780 WBGene	gi|24582843|	gi|29739185|ref|XP_295169.1|	gi|38088967|ref|XP_358685.1|	gi|6702040|ref|Os01g21140|	gi|6321514|ref|NP_011591.1|	gi|19114246|ref|NP_593334.1|
gi|31196333|ref|XP_307114.1|	lcl|At1g56200.1	lcl|C14F5.1b CE29172 WBGene	gi|24582850|	gi|29740258|ref|XP_294750.1|	gi|38089045|ref|XP_358687.1|	gi|6702041|ref|Os01g21150|	gi|6321516|ref|NP_011593.1|	gi|19114251|ref|NP_593339.1|
gi|31196337|ref|XP_307116.1|	lcl|At1g56270.1	lcl|C14F5.3c CE37091 WBGene	gi|24582860|	gi|29740707|ref|XP_295178.1|	gi|38089083|ref|XP_287764.2|	gi|6702045|ref|Os01g21170|	gi|6321518|ref|NP_011595.1|	gi|19114266|ref|NP_593354.1|
gi|31196345|ref|XP_307120.1|	lcl|At1g56530.1	lcl|C14H10.2 CE23543 WBGene	gi|24582922|	gi|29740944|ref|XP_294666.1|	gi|38089087|ref|XP_357852.1|	gi|6702048|ref|Os01g21200|	gi|6321521|ref|NP_011598.1|	gi|19114270|ref|NP_593358.1|
gi|31196351|ref|XP_307123.1|	lcl|At1g56555.1	lcl|C15A11.2 CE08170 WBGene	gi|24582991|	gi|29741438|ref|XP_294910.1|	gi|38089123|ref|XP_357864.1|	gi|6702050|ref|Os01g21220|	gi|6321526|ref|NP_011603.1|	gi|19114271|ref|NP_593359.1|
gi|31196355|ref|XP_307125.1|	lcl|At1g56660.1	lcl|C15A11.4 CE08172 WBGene	gi|24583000|	gi|29741464|ref|XP_294914.1|	gi|38089134|ref|XP_356063.1|	gi|6702054|ref|Os01g21260|	gi|6321536|ref|NP_011613.1|	gi|19114273|ref|NP_593361.1|
gi|31196361|ref|XP_307128.1|	lcl|At1g57565.1	lcl|C15A11.7 CE08175 WBGene	gi|24583002|	gi|29741963|ref|XP_294778.1|	gi|38089139|ref|XP_150054.2|	gi|6702063|ref|Os01g21340|	gi|6321539|ref|NP_011616.1|	gi|19114276|ref|NP_593364.1|
gi|31196363|ref|XP_307129.1|	lcl|At1g57906.1	lcl|C15B12.2 CE02484 WBGene	gi|24583020|	gi|29742514|ref|XP_294922.1|	gi|38089143|ref|XP_358688.1|	gi|6702064|ref|Os01g21350|	gi|6321541|ref|NP_011618.1|	gi|19114278|ref|NP_593366.1|
gi|31196365|ref|XP_307130.1|	lcl|At1g58070.1	lcl|C15B12.3 CE02485 WBGene	gi|24583029|	gi|29743451|ref|XP_294906.1|	gi|38089229|ref|XP_356075.1|	gi|6702066|ref|Os01g21370|	gi|6321542|ref|NP_011619.1|	gi|19114288|ref|NP_593376.1|
gi|31196369|ref|XP_307132.1|	lcl|At1g58150.1	lcl|C15B12.4 CE30864 WBGene	gi|24583033|	gi|29744195|ref|XP_293097.1|	gi|38089291|ref|XP_357887.1|	gi|6702068|ref|Os01g21390|	gi|6321543|ref|NP_011620.1|	gi|19114291|ref|NP_593379.1|
gi|31196375|ref|XP_307135.1|	lcl|At1g58225.1	lcl|C15B12.6 CE37610 WBGene	gi|24583055|	gi|29744348|ref|XP_294723.1|	gi|38089427|ref|XP_358377.1|	gi|6702070|ref|Os01g21410|	gi|6321549|ref|NP_011626.1|	gi|19114299|ref|NP_593387.1|
gi|31196381|ref|XP_307138.1|	lcl|At1g58235.1	lcl|C15C6.1 CE18501 WBGene0	gi|24583061|	gi|29744654|ref|XP_295007.1|	gi|38089429|ref|XP_358376.1|	gi|6702071|ref|Os01g21410|	gi|6321560|ref|NP_011637.1|	gi|19114302|ref|NP_593390.1|
gi|31196383|ref|XP_307139.1|	lcl|At1g58420.1	lcl|C15C6.2a CE18502 WBGene	gi|24583065|	gi|29745033|ref|XP_294680.1|	gi|38089487|ref|XP_358693.1|	gi|6702073|ref|Os01g21430|	gi|6321565|ref|NP_011642.1|	gi|19114305|ref|NP_593393.1|
gi|31196395|ref|XP_307145.1|	lcl|At1g58460.1	lcl|C15C6.2b CE18503 WBGene	gi|24583067|	gi|29745453|ref|XP_295270.1|	gi|38089500|ref|XP_358694.1|	gi|6702074|ref|Os01g21440|	gi|6321567|ref|NP_011644.1|	gi|19114307|ref|NP_593395.1|
gi|31196407|ref|XP_307151.1|	lcl|At1g59171.1	lcl|C15C6.3 CE18504 WBGene0	gi|24583081|	gi|29745745|ref|XP_295017.1|	gi|38089541|ref|XP_358695.1|	gi|6702075|ref|Os01g21450|	gi|6321568|ref|NP_011645.1|	gi|19114310|ref|NP_593398.1|
gi|31196409|ref|XP_307152.1|	lcl|At1g59535.1	lcl|C15C7.5 CE03991 WBGene0	gi|24583095|	gi|29745935|ref|XP_294688.1|	gi|38089647|ref|XP_358005.1|	gi|6702077|ref|Os01g21470|	gi|6321569|ref|NP_011646.1|	gi|19114313|ref|NP_593401.1|
gi|31196415|ref|XP_307155.1|	lcl|At1g59590.1	lcl|C15C7.6 CE37092 WBGene0	gi|24583104|	gi|29746498|ref|XP_294692.1|	gi|38089698|ref|XP_356140.1|	gi|6702078|ref|Os01g21480|	gi|6321573|ref|NP_011650.1|	gi|19114324|ref|NP_593412.1|
gi|31196417|ref|XP_307156.1|	lcl|At1g59690.1	lcl|C15C8.1 CE05285 WBGene0	gi|24583115|	gi|30017465|ref|NP_835222.1|	gi|38089868|ref|XP_358702.1|	gi|6702085|ref|Os01g21550|	gi|6321579|ref|NP_011656.1|	gi|19114327|ref|NP_593415.1|
gi|31196419|ref|XP_307157.1|	lcl|At1g59722.1	lcl|C15C8.6 CE05290 WBGene0	gi|24583122|	gi|30156012|ref|XP_099034.2|	gi|38089876|ref|XP_358704.1|	gi|6702087|ref|Os01g21570|	gi|6321580|ref|NP_011657.1|	gi|19114328|ref|NP_593416.1|
gi|31196435|ref|XP_307165.1|	lcl|At1g59835.1	lcl|C15C8.7 CE27580 WBGene0	gi|24583128|	gi|30179913|ref|NP_778212.2|	gi|38089915|ref|XP_134987.2|	gi|6702094|ref|Os01g21640|	gi|6321581|ref|NP_011658.1|	gi|19114331|ref|NP_593419.1|
gi|31196439|ref|XP_307167.1|	lcl|At1g59885.1	lcl|C15F1.2 CE23545 WBGene0	gi|24583148|	gi|30240939|ref|NP_005835.2|	gi|38089917|ref|XP_356169.1|	gi|6702096|ref|Os01g21660|	gi|6321585|ref|NP_011662.1|	gi|19114344|ref|NP_593432.1|
gi|31196441|ref|XP_307168.1|	lcl|At1g59920.1	lcl|C15F1.3a CE23546 WBGene	gi|24583200|	gi|30315662|ref|NP_847885.1|	gi|38090028|ref|XP_357965.1|	gi|6702098|ref|Os01g21680|	gi|6321586|ref|NP_011663.1|	gi|19114345|ref|NP_593433.1|
gi|31196447|ref|XP_307171.1|	lcl|At1g59930.1	lcl|C15F1.3b CE23547 WBGene	gi|24583208|	gi|30350204|ref|NP_848028.1|	gi|38090110|ref|XP_356189.1|	gi|6702100|ref|Os01g21700|	gi|6321592|ref|NP_011669.1|	gi|19114352|ref|NP_593440.1|
gi|31196449|ref|XP_307172.1|	lcl|At1g60000.2	lcl|C15F1.8 CE23551 WBGene0	gi|24583230|	gi|30350206|ref|NP_848029.1|	gi|38090158|ref|XP_358707.1|	gi|6702102|ref|Os01g21720|	gi|6321600|ref|NP_011677.1|	gi|19114354|ref|NP_593442.1|
gi|31196457|ref|XP_307176.1|	lcl|At1g60075.1	lcl|C15H11.10 CE34686 WBGen	gi|24583232|	gi|30350208|ref|NP_848031.1|	gi|38090191|ref|XP_357973.1|	gi|6702103|ref|Os01g21730|	gi|6321604|ref|NP_011681.1|	gi|19114373|ref|NP_593461.1|
gi|31196461|ref|XP_307178.1|	lcl|At1g60210.1	lcl|C15H11.11 CE34687 WBGen	gi|24583234|	gi|30350210|ref|NP_848033.1|	gi|38090314|ref|XP_358710.1|	gi|6702104|ref|Os01g21740|	gi|6321605|ref|NP_011682.1|	gi|19114375|ref|NP_593463.1|
gi|31196471|ref|XP_307183.1|	lcl|At1g60240.1	lcl|C15H7.4 CE00082 WBGene0	gi|24583236|	gi|30350212|ref|NP_848030.1|	gi|38090480|ref|XP_358302.1|	gi|6702105|ref|Os01g21750|	gi|6321607|ref|NP_011684.1|	gi|19114381|ref|NP_593469.1|
gi|31196481|ref|XP_307188.1|	lcl|At1g60250.1	lcl|C15H9.10 CE06836 WBGene	gi|24583250|	gi|30350214|ref|NP_848032.1|	gi|38090500|ref|XP_358390.1|	gi|6702106|ref|Os01g21760|	gi|6321613|ref|NP_011690.1|	gi|19114385|ref|NP_593473.1|
gi|31196483|ref|XP_307189.1|	lcl|At1g60280.1	lcl|C15H9.4 CE03996 WBGene0	gi|24583332|	gi|30350216|ref|NP_848034.1|	gi|38090504|ref|XP_358391.1|	gi|6702107|ref|Os01g21770|	gi|6321618|ref|NP_011695.1|	gi|19114389|ref|NP_593477.1|
gi|31196487|ref|XP_307191.1|	lcl|At1g60300.1	lcl|C16A11.2b CE36979 WBGen	gi|24583334|	gi|30387642|ref|NP_848133.1|	gi|38090555|ref|XP_358392.1|	gi|6702108|ref|Os01g21780|	gi|6321635|ref|NP_011712.1|	gi|19114397|ref|NP_593485.1|
gi|31196495|ref|XP_307195.1|	lcl|At1g60340.1	lcl|C16A11.5 CE34689 WBGene	gi|24583341|	gi|30387646|ref|NP_848131.1|	gi|38090587|ref|XP_358394.1|	gi|6702109|ref|Os01g21790|	gi|6321636|ref|NP_011713.1|	gi|19114399|ref|NP_593487.1|
gi|31196505|ref|XP_307200.1|	lcl|At1g60350.1	lcl|C16A11.6 CE36787 WBGene	gi|24583372|	gi|30387656|ref|NP_848126.1|	gi|38090684|ref|XP_358397.1|	gi|6702110|ref|Os01g21800|	gi|6321654|ref|NP_011731.1|	gi|19114400|ref|NP_593488.1|
gi|31196507|ref|XP_307201.1|	lcl|At1g60380.1	lcl|C16B8.2 CE30247 WBGene0	gi|24583477|	gi|30410033|ref|NP_848522.1|	gi|38090686|ref|XP_358398.1|	gi|6702113|ref|Os01g21830|	gi|6321660|ref|NP_011737.1|	gi|19114401|ref|NP_593489.1|
gi|31196525|ref|XP_307210.1|	lcl|At1g60870.1	lcl|C16B8.3 CE04013 WBGene0	gi|24583494|	gi|30410037|ref|NP_848521.1|	gi|38090717|ref|XP_358400.1|	gi|6702117|ref|Os01g21870|	gi|6321665|ref|NP_011742.1|	gi|19114405|ref|NP_593493.1|
gi|31196527|ref|XP_307211.1|	lcl|At1g60985.1	lcl|C16C10.13 CE34691 WBGen	gi|24583498|	gi|30410041|ref|NP_848520.1|	gi|38090734|ref|XP_356486.1|	gi|6702118|ref|Os01g21880|	gi|6321674|ref|NP_011751.1|	gi|19114413|ref|NP_593501.1|
gi|31196543|ref|XP_307219.1|	lcl|At1g61080.1	lcl|C16C10.9 CE01500 WBGene	gi|24583502|	gi|30410043|ref|NP_848517.1|	gi|38090804|ref|XP_359227.1|	gi|6702119|ref|Os01g21890|	gi|6321675|ref|NP_011752.1|	gi|19114418|ref|NP_593506.1|
gi|31196547|ref|XP_307221.1|	lcl|At1g61090.1	lcl|C16C4.1 CE36788 WBGene0	gi|24583504|	gi|30410045|ref|NP_848515.1|	gi|38091294|ref|XP_358403.1|	gi|6702121|ref|Os01g21910|	gi|6321676|ref|NP_011753.1|	gi|19114419|ref|NP_593507.1|
gi|31196567|ref|XP_307231.1|	lcl|At1g61095.1	lcl|C16C4.7 CE08203 WBGene0	gi|24583541|	gi|30410047|ref|NP_848516.1|	gi|38091310|ref|XP_358404.1|	gi|6702122|ref|Os01g21920|	gi|6321678|ref|NP_011755.1|	gi|19114420|ref|NP_593508.1|
gi|31196573|ref|XP_307234.1|	lcl|At1g61160.1	lcl|C16C8.10 CE08221 WBGene	gi|24583563|	gi|30425386|ref|NP_848558.1|	gi|38091315|ref|XP_358405.1|	gi|6702123|ref|Os01g21930|	gi|6321690|ref|NP_011767.1|	gi|19114427|ref|NP_593515.1|
gi|31196575|ref|XP_307235.1|	lcl|At1g61170.1	lcl|C16C8.12 CE34693 WBGene	gi|24583565|	gi|30425430|ref|NP_848598.1|	gi|38091576|ref|XP_358728.1|	gi|6702125|ref|Os01g21950|	gi|6321705|ref|NP_011782.1|	gi|19114428|ref|NP_593516.1|
gi|31196585|ref|XP_307240.1|	lcl|At1g61200.1	lcl|C16C8.19 CE28205 WBGene	gi|24583567|	gi|30425450|ref|NP_848608.1|	gi|38091764|ref|XP_358408.1|	gi|6702128|ref|Os01g21980|	gi|6321712|ref|NP_011789.1|	gi|19114431|ref|NP_593519.1|
gi|31196591|ref|XP_307243.1|	lcl|At1g61255.1	lcl|C16C8.7 CE08218 WBGene0	gi|24583569|	gi|30425452|ref|NP_848609.1|	gi|38091774|ref|XP_112540.2|	gi|6702130|ref|Os01g22000|	gi|6321738|ref|NP_011814.1|	gi|19114434|ref|NP_593522.1|
gi|31196593|ref|XP_307244.1|	lcl|At1g61920.1	lcl|C16C8.8 CE08219 WBGene0	gi|24583573|	gi|30425454|ref|NP_848610.1|	gi|38091932|ref|XP_358414.1|	gi|6702134|ref|Os01g22020|	gi|6321749|ref|NP_011825.1|	gi|19114439|ref|NP_593527.1|
gi|31196597|ref|XP_307246.1|	lcl|At1g62000.1	lcl|C16C8.9 CE08220 WBGene0	gi|24583579|	gi|30425462|ref|NP_848614.1|	gi|38091999|ref|XP_358415.1|	gi|6702135|ref|Os01g22030|	gi|6321767|ref|NP_011843.1|	gi|19114440|ref|NP_593528.1|
gi|31196599|ref|XP_307247.1|	lcl|At1g62045.1	lcl|C16D6.1 CE08230 WBGene0	gi|24583590|	gi|30425466|ref|NP_848616.1|	gi|38093514|ref|XP_358049.1|	gi|6702136|ref|Os01g22040|	gi|6321778|ref|NP_011854.1|	gi|19114444|ref|NP_593532.1|
gi|31196601|ref|XP_307248.1|	lcl|At1g62060.1	lcl|C16D6.3 CE08232 WBGene0	gi|24583614|	gi|30425474|ref|NP_848623.1|	gi|38093600|ref|XP_356255.1|	gi|6702140|ref|Os01g22080|	gi|6321788|ref|NP_011864.1|	gi|19114445|ref|NP_593533.1|
gi|31196605|ref|XP_307250.1|	lcl|At1g62070.1	lcl|C16D9.2b CE32799 WBGene	gi|24583618|	gi|30425476|ref|NP_848624.1|	gi|38093921|ref|XP_358103.1|	gi|6702141|ref|Os01g22090|	gi|6321802|ref|NP_011878.1|	gi|19114454|ref|NP_593542.1|
gi|31196607|ref|XP_307251.1|	lcl|At1g62080.1	lcl|C16D9.3 CE32801 WBGene0	gi|24583620|	gi|30425478|ref|NP_848625.1|	gi|38093936|ref|XP_358107.1|	gi|6702147|ref|Os01g22150|	gi|6321805|ref|NP_011881.1|	gi|19114455|ref|NP_593543.1|
gi|31196609|ref|XP_307252.1|	lcl|At1g62210.1	lcl|C16D9.8 CE06844 WBGene0	gi|24583622|	gi|30425480|ref|NP_848626.1|	gi|38094079|ref|XP_142623.2|	gi|6702148|ref|Os01g22160|	gi|6321810|ref|NP_011886.1|	gi|19114457|ref|NP_593545.1|
gi|31196619|ref|XP_307257.1|	lcl|At1g62220.1	lcl|C16D9.9 CE27696 WBGene0	gi|24583654|	gi|30425484|ref|NP_848628.1|	gi|38094639|ref|XP_358151.1|	gi|6702153|ref|Os01g22210|	gi|6321823|ref|NP_011899.1|	gi|19114474|ref|NP_593562.1|
gi|31196625|ref|XP_307260.1|	lcl|At1g62225.1	lcl|C16H3.3a CE27697 WBGene	gi|24583660|	gi|30425488|ref|NP_848630.1|	gi|38095519|ref|XP_358163.1|	gi|6702154|ref|Os01g22220|	gi|6321825|ref|NP_011901.1|	gi|19114485|ref|NP_593573.1|
gi|31196627|ref|XP_307261.1|	lcl|At1g62240.1	lcl|C16H3.3b CE08237 WBGene	gi|24583703|	gi|30425494|ref|NP_848633.1|	gi|38097051|ref|XP_135584.2|	gi|6702161|ref|Os01g22290|	gi|6321831|ref|NP_011907.1|	gi|19114488|ref|NP_593576.1|
gi|31196631|ref|XP_307263.1|	lcl|At1g62480.1	lcl|C17C3.1c CE27072 WBGene	gi|24583705|	gi|30425508|ref|NP_848641.1|	gi|6678708|ref|NP_032534.1|	gi|6702163|ref|Os01g22310|	gi|6321835|ref|NP_011911.1|	gi|19114499|ref|NP_593587.1|
gi|31196633|ref|XP_307264.1|	lcl|At1g62690.1	lcl|C17C3.1d CE27073 WBGene	gi|24583717|	gi|30425549|ref|NP_848664.1|	gi|6678942|ref|NP_032657.1|	gi|6702164|ref|Os01g22320|	gi|6321845|ref|NP_011921.1|	gi|19114502|ref|NP_593590.1|
gi|31196639|ref|XP_307267.1|	lcl|At1g62920.1	lcl|C17C3.9 CE34699 WBGene0	gi|24583724|	gi|30520316|ref|NP_849160.1|	gi|6678962|ref|NP_032670.1|	gi|6702167|ref|Os01g22350|	gi|6321851|ref|NP_011927.1|	gi|19114503|ref|NP_593591.1|
gi|31196657|ref|XP_307276.1|	lcl|At1g62935.1	lcl|C17D12.7 CE23554 WBGene	gi|24583726|	gi|30520357|ref|NP_848931.1|	gi|6679305|ref|NP_032859.1|	gi|6702170|ref|Os01g22380|	gi|6321869|ref|NP_011945.1|	gi|19114508|ref|NP_593596.1|
gi|31196661|ref|XP_307278.1|	lcl|At1g63105.1	lcl|C17E4.1 CE08250 WBGene0	gi|24583751|	gi|30581150|ref|NP_847901.1|	gi|6679307|ref|NP_032860.1|	gi|6702177|ref|Os01g22440|	gi|6321871|ref|NP_011947.1|	gi|19114511|ref|NP_593599.1|
gi|31196663|ref|XP_307279.1|	lcl|At1g63240.1	lcl|C17E4.10 CE32589 WBGene	gi|24583753|	gi|30581152|ref|NP_847902.1|	gi|6679309|ref|NP_032861.1|	gi|6702178|ref|Os01g22450|	gi|6321875|ref|NP_011951.1|	gi|19114530|ref|NP_593618.1|
gi|31196667|ref|XP_307281.1|	lcl|At1g63245.1	lcl|C17E4.2 CE08251 WBGene0	gi|24583756|	gi|31377614|ref|NP_612437.2|	gi|6680025|ref|NP_032158.1|	gi|6702179|ref|Os01g22460|	gi|6321893|ref|NP_011969.1|	gi|19114534|ref|NP_593622.1|
gi|31196677|ref|XP_307286.1|	lcl|At1g63530.1	lcl|C17E4.4 CE08253 WBGene0	gi|24583758|	gi|31377654|ref|NP_116159.2|	gi|6680269|ref|NP_032309.1|	gi|6702180|ref|Os01g22470|	gi|6321895|ref|NP_011971.1|	gi|19114540|ref|NP_593628.1|
gi|31196683|ref|XP_307289.1|	lcl|At1g63670.1	lcl|C17E7.12 CE27698 WBGene	gi|24583822|	gi|31563390|ref|NP_851785.1|	gi|6753962|ref|NP_034398.1|	gi|6702186|ref|Os01g22530|	gi|6321910|ref|NP_011986.1|	gi|19114550|ref|NP_593638.1|
gi|31196697|ref|XP_307296.1|	lcl|At1g63960.1	lcl|C17E7.13 CE27699 WBGene	gi|24583856|	gi|31563392|ref|NP_851786.1|	gi|6754144|ref|NP_034526.1|	gi|6702199|ref|Os01g22650|	gi|6321918|ref|NP_011994.1|	gi|19114553|ref|NP_593641.1|
gi|31196707|ref|XP_307301.1|	lcl|At1g64050.1	lcl|C17E7.4 CE08264 WBGene0	gi|24583860|	gi|31563394|ref|NP_851787.1|	gi|6754496|ref|NP_034803.1|	gi|6702203|ref|Os01g22690|	gi|6321925|ref|NP_012001.1|	gi|19114555|ref|NP_593643.1|
gi|31196725|ref|XP_307310.1|	lcl|At1g64080.1	lcl|C17E7.9a CE27700 WBGene	gi|24583881|	gi|31563396|ref|NP_851788.1|	gi|6754498|ref|NP_034806.1|	gi|6702206|ref|Os01g22720|	gi|6321928|ref|NP_012004.1|	gi|19114564|ref|NP_593652.1|
gi|31196727|ref|XP_307311.1|	lcl|At1g64105.1	lcl|C17E7.9b CE27701 WBGene	gi|24583890|	gi|31563398|ref|NP_851789.1|	gi|6754892|ref|NP_035070.1|	gi|6702210|ref|Os01g22760|	gi|6321933|ref|NP_012009.1|	gi|19114567|ref|NP_593655.1|
gi|31196743|ref|XP_307319.1|	lcl|At1g64290.1	lcl|C17F3.1 CE36270 WBGene0	gi|24583894|	gi|31563402|ref|NP_851800.1|	gi|6755056|ref|NP_035210.1|	gi|6702211|ref|Os01g22770|	gi|6321935|ref|NP_012011.1|	gi|19114570|ref|NP_593658.1|
gi|31196745|ref|XP_307320.1|	lcl|At1g64320.1	lcl|C17F3.3 CE17414 WBGene0	gi|24583896|	gi|31563404|ref|NP_851802.1|	gi|6755146|ref|NP_036152.1|	gi|6702215|ref|Os01g22810|	gi|6321936|ref|NP_012012.1|	gi|19114576|ref|NP_593664.1|
gi|31196749|ref|XP_307322.1|	lcl|At1g64340.1	lcl|C17F4.2 CE08275 WBGene0	gi|24583906|	gi|31563406|ref|NP_851803.1|	gi|6755254|ref|NP_035352.1|	gi|6702216|ref|Os01g22820|	gi|6321943|ref|NP_012019.1|	gi|19114584|ref|NP_593672.1|
gi|31196759|ref|XP_307327.1|	lcl|At1g64360.1	lcl|C17F4.3 CE08276 WBGene0	gi|24583912|	gi|31563408|ref|NP_851804.1|	gi|6755630|ref|NP_035599.1|	gi|6702220|ref|Os01g22860|	gi|6321945|ref|NP_012021.1|	gi|19114585|ref|NP_593673.1|
gi|31196777|ref|XP_307336.1|	lcl|At1g64370.1	lcl|C17F4.7 CE27076 WBGene0	gi|24583927|	gi|31563410|ref|NP_851805.1|	gi|6755632|ref|NP_035600.1|	gi|6702222|ref|Os01g22880|	gi|6321947|ref|NP_012023.1|	gi|19114587|ref|NP_593675.1|
gi|31196781|ref|XP_307338.1|	lcl|At1g64405.1	lcl|C17G1.1 CE08280 WBGene0	gi|24583929|	gi|31563412|ref|NP_851806.1|	gi|6755634|ref|NP_035601.1|	gi|6702227|ref|Os01g22930|	gi|6321951|ref|NP_012027.1|	gi|19114588|ref|NP_593676.1|
gi|31196785|ref|XP_307340.1|	lcl|At1g64490.1	lcl|C17G1.2 CE08281 WBGene0	gi|24583931|	gi|31563414|ref|NP_851807.1|	gi|6755636|ref|NP_035602.1|	gi|6702230|ref|Os01g22960|	gi|6321953|ref|NP_012029.1|	gi|19114591|ref|NP_593679.1|
gi|31196795|ref|XP_307345.1|	lcl|At1g64560.1	lcl|C17G1.4a CE08283 WBGene	gi|24583935|	gi|31563416|ref|NP_851809.1|	gi|6755638|ref|NP_035603.1|	gi|6702234|ref|Os01g22990|	gi|6321954|ref|NP_012030.1|	gi|19114595|ref|NP_593683.1|
gi|31196797|ref|XP_307346.1|	lcl|At1g64700.1	lcl|C17G1.4b CE37318 WBGene	gi|24583958|	gi|31563418|ref|NP_851810.1|	gi|6755640|ref|NP_035604.1|	gi|6702235|ref|Os01g23000|	gi|6321961|ref|NP_012037.1|	gi|19114600|ref|NP_593688.1|
gi|31196799|ref|XP_307347.1|	lcl|At1g64750.1	lcl|C17G1.5 CE31416 WBGene0	gi|24584001|	gi|31563420|ref|NP_851811.1|	gi|6755642|ref|NP_035605.1|	gi|6702236|ref|Os01g23010|	gi|6321978|ref|NP_012054.1|	gi|19114601|ref|NP_593689.1|
gi|31196801|ref|XP_307348.1|	lcl|At1g64800.1	lcl|C17G10.3 CE19359 WBGene	gi|24584007|	gi|31563422|ref|NP_851812.1|	gi|6755646|ref|NP_035607.1|	gi|6702238|ref|Os01g23030|	gi|6321979|ref|NP_012055.1|	gi|19114618|ref|NP_593706.1|
gi|31196807|ref|XP_307351.1|	lcl|At1g64870.1	lcl|C17G10.6a CE01790 WBGen	gi|24584009|	gi|31563424|ref|NP_851813.1|	gi|6755871|ref|NP_035765.1|	gi|6702241|ref|Os01g23060|	gi|6321981|ref|NP_012057.1|	gi|19114621|ref|NP_593709.1|
gi|31196811|ref|XP_307353.1|	lcl|At1g65342.1	lcl|C17G10.6b CE36104 WBGen	gi|24584039|	gi|31563426|ref|NP_851814.1|	gi|7110625|ref|NP_034546.1|	gi|6702245|ref|Os01g23100|	gi|6321985|ref|NP_012061.1|	gi|19114623|ref|NP_593711.1|
gi|31196813|ref|XP_307354.1|	lcl|At1g65445.1	lcl|C17G10.7 CE01791 WBGene	gi|24584049|	gi|31563428|ref|NP_851815.1|	gi|7305131|ref|NP_038944.1|	gi|6702249|ref|Os01g23140|	gi|6321989|ref|NP_012065.1|	gi|19114625|ref|NP_593713.1|
gi|31196815|ref|XP_307355.1|	lcl|At1g65490.1	lcl|C17H1.2 CE15624 WBGene0	gi|24584051|	gi|31563430|ref|NP_851816.1|	gi|7305285|ref|NP_038630.1|	gi|6702250|ref|Os01g23150|	gi|6321991|ref|NP_012067.1|	gi|19114629|ref|NP_593717.1|
gi|31196817|ref|XP_307356.1|	lcl|At1g65500.1	lcl|C17H1.3 CE15625 WBGene0	gi|24584053|	gi|31563432|ref|NP_851817.1|	gi|7305287|ref|NP_038631.1|	gi|6702252|ref|Os01g23170|	gi|6322008|ref|NP_012084.1|	gi|19114631|ref|NP_593719.1|
gi|31196825|ref|XP_307360.1|	lcl|At1g65510.1	lcl|C17H1.4 CE15626 WBGene0	gi|24584056|	gi|31563434|ref|NP_851818.1|	gi|7305409|ref|NP_038665.1|	gi|6702253|ref|Os01g23180|	gi|6322015|ref|NP_012091.1|	gi|19114633|ref|NP_593721.1|
gi|31196835|ref|XP_307365.1|	lcl|At1g65740.1	lcl|C17H1.5 CE15627 WBGene0	gi|24584073|	gi|31745172|ref|NP_006772.2|	gi|7657283|ref|NP_056623.1|	gi|6702254|ref|Os01g23190|	gi|6322022|ref|NP_012097.1|	gi|19114635|ref|NP_593723.1|
gi|31196843|ref|XP_307369.1|	lcl|At1g65845.1	lcl|C17H1.6 CE15628 WBGene0	gi|24584086|	gi|31791000|ref|NP_853655.1|	gi|7710034|ref|NP_057869.1|	gi|6702257|ref|Os01g23220|	gi|6322030|ref|NP_012105.1|	gi|19114640|ref|NP_593728.1|
gi|31196871|ref|XP_307383.1|	lcl|At1g66145.1	lcl|C17H1.7 CE15629 WBGene0	gi|24584090|	gi|31791010|ref|NP_853650.1|		gi|6702259|ref|Os01g23240|	gi|6322033|ref|NP_012108.1|	gi|19114647|ref|NP_593735.1|
gi|31196873|ref|XP_307384.1|	lcl|At1g66190.1	lcl|C17H1.9 CE34700 WBGene0	gi|24584124|	gi|31791012|ref|NP_853651.1|		gi|6702261|ref|Os01g23260|	gi|6322034|ref|NP_012109.1|	gi|19114650|ref|NP_593738.1|
gi|31196877|ref|XP_307386.1|	lcl|At1g66235.1	lcl|C17H11.2 CE27703 WBGene	gi|24584131|	gi|31791014|ref|NP_853647.1|		gi|6702264|ref|Os01g23290|	gi|6322037|ref|NP_012112.1|	gi|19114654|ref|NP_593742.1|
gi|31196881|ref|XP_307388.1|	lcl|At1g66245.1	lcl|C17H11.5 CE08293 WBGene	gi|24584133|	gi|31791016|ref|NP_853648.1|		gi|6702265|ref|Os01g23300|	gi|6322039|ref|NP_012114.1|	gi|19114655|ref|NP_593743.1|
gi|31196885|ref|XP_307390.1|	lcl|At1g66790.1	lcl|C17H12.10 CE16870 WBGen	gi|24584135|	gi|31791018|ref|NP_853645.1|		gi|6702266|ref|Os01g23310|	gi|6322045|ref|NP_012120.1|	gi|19114657|ref|NP_593745.1|
gi|31196887|ref|XP_307391.1|	lcl|At1g66820.1	lcl|C18A11.1 CE04032 WBGene	gi|24584146|	gi|31791020|ref|NP_853646.1|		gi|6702267|ref|Os01g23320|	gi|6322055|ref|NP_012130.1|	gi|19114658|ref|NP_593746.1|
gi|31196889|ref|XP_307392.1|	lcl|At1g66890.1	lcl|C18A11.2 CE04033 WBGene	gi|24584169|	gi|31791022|ref|NP_853642.1|		gi|6702269|ref|Os01g23340|	gi|6322056|ref|NP_012131.1|	gi|19114660|ref|NP_593748.1|
gi|31196893|ref|XP_307394.1|	lcl|At1g66940.1	lcl|C18A11.3 CE04034 WBGene	gi|24584177|	gi|31791024|ref|NP_853643.1|		gi|6702270|ref|Os01g23350|	gi|6322059|ref|NP_012134.1|	gi|19114662|ref|NP_593750.1|
gi|31196899|ref|XP_307397.1|	lcl|At1g67020.1	lcl|C18A11.6 CE04037 WBGene	gi|24584198|	gi|31791026|ref|NP_853640.1|		gi|6702271|ref|Os01g23360|	gi|6322064|ref|NP_012139.1|	gi|19114667|ref|NP_593755.1|
gi|31196903|ref|XP_307399.1|	lcl|At1g67025.1	lcl|C18A3.10 CE31306 WBGene	gi|24584238|	gi|31791028|ref|NP_853641.1|		gi|6702274|ref|Os01g23390|	gi|6322069|ref|NP_012144.1|	gi|19114669|ref|NP_593757.1|
gi|31196907|ref|XP_307401.1|	lcl|At1g67035.1	lcl|C18A3.4a CE01796 WBGene	gi|24584257|	gi|31791030|ref|NP_853638.1|		gi|6702275|ref|Os01g23400|	gi|6322072|ref|NP_012147.1|	gi|19114670|ref|NP_593758.1|
gi|31196923|ref|XP_307409.1|	lcl|At1g67040.1	lcl|C18A3.4b CE29484 WBGene	gi|24584269|	gi|31791032|ref|NP_853639.1|		gi|6702278|ref|Os01g23430|	gi|6322074|ref|NP_012149.1|	gi|19114673|ref|NP_593761.1|
gi|31196931|ref|XP_307413.1|	lcl|At1g67050.1	lcl|C18A3.4c CE32129 WBGene	gi|24584274|	gi|31791034|ref|NP_853636.1|		gi|6702280|ref|Os01g23450|	gi|6322081|ref|NP_012156.1|	gi|19114678|ref|NP_593766.1|
gi|31196933|ref|XP_307414.1|	lcl|At1g67350.1	lcl|C18A3.7 CE01799 WBGene0	gi|24584287|	gi|31791047|ref|NP_857598.1|		gi|6702281|ref|Os01g23460|	gi|6322089|ref|NP_012164.1|	gi|19114681|ref|NP_593769.1|
gi|31196935|ref|XP_307415.1|	lcl|At1g67540.1	lcl|C18A3.9 CE29177 WBGene0	gi|24584302|	gi|31982871|ref|NP_620585.2|		gi|6702283|ref|Os01g23480|	gi|6322093|ref|NP_012168.1|	gi|19114683|ref|NP_593771.1|
gi|31196951|ref|XP_307423.1|	lcl|At1g67635.1	lcl|C18B12.1 CE18508 WBGene	gi|24584337|	gi|32140180|ref|NP_859014.1|		gi|6702285|ref|Os01g23500|	gi|6322095|ref|NP_012170.1|	gi|19114685|ref|NP_593773.1|
gi|31196955|ref|XP_307425.1|	lcl|At1g67670.1	lcl|C18B2.3 CE27077 WBGene0	gi|24584343|	gi|32171194|ref|NP_859078.1|		gi|6702286|ref|Os01g23510|	gi|6322099|ref|NP_012174.1|	gi|19114686|ref|NP_593774.1|
gi|31196963|ref|XP_307429.1|	lcl|At1g67790.1	lcl|C18C4.5a CE30729 WBGene	gi|24584357|	gi|32171203|ref|NP_859073.1|		gi|6702287|ref|Os01g23520|	gi|6322102|ref|NP_012177.1|	gi|19114687|ref|NP_593775.1|
gi|31196967|ref|XP_307431.1|	lcl|At1g67855.1	lcl|C18C4.5b CE35246 WBGene	gi|24584361|	gi|32171211|ref|NP_859068.1|		gi|6702292|ref|Os01g23570|	gi|6322104|ref|NP_012179.1|	gi|19114693|ref|NP_593781.1|
gi|31196995|ref|XP_307445.1|	lcl|At1g67860.1	lcl|C18C4.6 CE06851 WBGene0	gi|24584363|	gi|32698835|ref|NP_872305.1|		gi|6702295|ref|Os01g23600|	gi|6322107|ref|NP_012182.1|	gi|19114706|ref|NP_593794.1|
gi|31197003|ref|XP_307449.1|	lcl|At1g67865.1	lcl|C18C4.7 CE06852 WBGene0	gi|24584368|	gi|32698859|ref|NP_872316.1|		gi|6702301|ref|Os01g23650|	gi|6322113|ref|NP_012188.1|	gi|19114714|ref|NP_593802.1|
gi|31197007|ref|XP_307451.1|	lcl|At1g67870.1	lcl|C18D1.3 CE01503 WBGene0	gi|24584415|	gi|32698870|ref|NP_872322.1|		gi|6702302|ref|Os01g23660|	gi|6322114|ref|NP_012189.1|	gi|19114717|ref|NP_593805.1|
gi|31197009|ref|XP_307452.1|	lcl|At1g67910.1	lcl|C18D1.4 CE34701 WBGene0	gi|24584417|	gi|32698900|ref|NP_872341.1|		gi|6702307|ref|Os01g23700|	gi|6322129|ref|NP_012204.1|	gi|19114719|ref|NP_593807.1|
gi|31197027|ref|XP_307461.1|	lcl|At1g67920.1	lcl|C18D11.1 CE18512 WBGene	gi|24584419|	gi|32698906|ref|NP_872340.1|		gi|6702308|ref|Os01g23710|	gi|6322132|ref|NP_012207.1|	gi|19114721|ref|NP_593809.1|
gi|31197037|ref|XP_307466.1|	lcl|At1g68240.1	lcl|C18D11.3 CE18514 WBGene	gi|24584421|	gi|32698924|ref|NP_872356.1|		gi|6702309|ref|Os01g23720|	gi|6322134|ref|NP_012209.1|	gi|19114732|ref|NP_593821.1|
gi|31197053|ref|XP_307474.1|	lcl|At1g68250.1	lcl|C18D11.6 CE33974 WBGene	gi|24584427|	gi|32698946|ref|NP_872364.1|		gi|6702316|ref|Os01g23790|	gi|6322149|ref|NP_012224.1|	gi|19114741|ref|NP_593829.1|
gi|31197055|ref|XP_307475.1|	lcl|At1g68330.1	lcl|C18D4.1 CE15631 WBGene0	gi|24584429|	gi|32698958|ref|NP_872373.1|		gi|6702317|ref|Os01g23800|	gi|6322152|ref|NP_012227.1|	gi|19114756|ref|NP_593844.1|
gi|31197057|ref|XP_307476.1|	lcl|At1g68340.1	lcl|C18D4.3 CE15633 WBGene0	gi|24584432|	gi|32698960|ref|NP_872370.1|		gi|6702318|ref|Os01g23810|	gi|6322160|ref|NP_012235.1|	gi|19114757|ref|NP_593845.1|
gi|31197059|ref|XP_307477.1|	lcl|At1g68350.1	lcl|C18D4.6a CE32310 WBGene	gi|24584434|	gi|32698968|ref|NP_872374.1|		gi|6702320|ref|Os01g23830|	gi|6322162|ref|NP_012237.1|	gi|19114759|ref|NP_593847.1|
gi|31197065|ref|XP_307480.1|	lcl|At1g68430.1	lcl|C18D4.6b CE32311 WBGene	gi|24584440|	gi|32698972|ref|NP_872376.1|		gi|6702322|ref|Os01g23850|	gi|6322165|ref|NP_012240.1|	gi|19114761|ref|NP_593849.1|
gi|31197067|ref|XP_307481.1|	lcl|At1g68440.1	lcl|C18D4.6c CE36469 WBGene	gi|24584442|	gi|32698974|ref|NP_872382.1|		gi|6702323|ref|Os01g23860|	gi|6322170|ref|NP_012245.1|	gi|19114762|ref|NP_593850.1|
gi|31197073|ref|XP_307484.1|	lcl|At1g68490.1	lcl|C18E3.1 CE27712 WBGene0	gi|24584444|	gi|32698986|ref|NP_872388.1|		gi|6702324|ref|Os01g23870|	gi|6322175|ref|NP_012250.1|	gi|19114763|ref|NP_593851.1|

gi|31197081|ref|XP_307488.1| lcl|At1g68500.1 lcl|C18E3.3 CE08312 WBGene0 gi|24584456| gi|32698988|ref|NP_872385.1| gi|6702325|ref|Os01g23880| gi|6322181|ref|NP_012256.1| gi|19114767|ref|NP_593855.1|
gi|31197083|ref|XP_307489.1| lcl|At1g68585.1 lcl|C18E3.4 CE08313 WBGene0 gi|24584498| gi|32698990|ref|NP_872390.1| gi|6702326|ref|Os01g23890| gi|6322193|ref|NP_012268.1| gi|19114768|ref|NP_593856.1|
gi|31197107|ref|XP_307501.1| lcl|At1g68680.1 lcl|C18E3.7b CE31307 WBGene gi|24584519| gi|32698994|ref|NP_872391.1| gi|6702328|ref|Os01g23910| gi|6322200|ref|NP_012275.1| gi|19114776|ref|NP_593864.1|
gi|31197109|ref|XP_307502.1| lcl|At1g68700.1 lcl|C18E3.9 CE27716 WBGene0 gi|24584531| gi|32698996|ref|NP_872389.1| gi|6702329|ref|Os01g23920| gi|6322206|ref|NP_012281.1| gi|19114790|ref|NP_593878.1|
gi|31197113|ref|XP_307504.1| lcl|At1g68725.1 lcl|C18E9.3a CE32804 WBGene gi|24584534| gi|32699003|ref|NP_872392.1| gi|6702331|ref|Os01g23940| gi|6322207|ref|NP_012282.1| gi|19114820|ref|NP_593908.1|
gi|31197123|ref|XP_307509.1| lcl|At1g68765.1 lcl|C18E9.3b CE20519 WBGene gi|24584536| gi|32699011|ref|NP_872397.1| gi|6702332|ref|Os01g23950| gi|6322212|ref|NP_012287.1| gi|19114821|ref|NP_593909.1|
gi|31197125|ref|XP_307510.1| lcl|At1g68795.1 lcl|C18E9.5 CE34704 WBGene0 gi|24584545| gi|32699013|ref|NP_872404.1| gi|6702336|ref|Os01g23990| gi|6322215|ref|NP_012290.1| gi|19114822|ref|NP_593910.1|
gi|31197145|ref|XP_307520.1| lcl|At1g68845.1 lcl|C18E9.8 CE05300 WBGene0 gi|24584547| gi|32699017|ref|NP_872406.1| gi|6702349|ref|Os01g24110| gi|6322250|ref|NP_012325.1| gi|19114830|ref|NP_593918.1|
gi|31197149|ref|XP_307522.1| lcl|At1g68870.1 lcl|C18E9.9 CE05301 WBGene0 gi|24584573| gi|32699021|ref|NP_872410.1| gi|6702350|ref|Os01g24120| gi|6322256|ref|NP_012330.1| gi|19114833|ref|NP_593921.1|
gi|31197151|ref|XP_307523.1| lcl|At1g68875.1 lcl|C18F10.2 CE30607 WBGene gi|24584595| gi|32699030|ref|NP_872411.1| gi|6702353|ref|Os01g24150| gi|6322269|ref|NP_012343.1| gi|19114837|ref|NP_593925.1|
gi|31197179|ref|XP_307537.1| lcl|At1g68935.1 lcl|C18G1.1 CE17417 WBGene0 gi|24584597| gi|32699062|ref|NP_872432.1| gi|6702354|ref|Os01g24160| gi|6322276|ref|NP_012350.1| gi|19114849|ref|NP_593937.1|
gi|31197189|ref|XP_307542.1| lcl|At1g68945.1 lcl|C18G1.4a CE17420 WBGene gi|24584599| gi|32699068|ref|NP_872435.1| gi|6702355|ref|Os01g24170| gi|6322277|ref|NP_012351.1| gi|19114851|ref|NP_593939.1|
gi|31197191|ref|XP_307543.1| lcl|At1g69050.1 lcl|C18G1.4b CE27717 WBGene gi|24584636| gi|32699145|ref|NP_872290.1| gi|6702357|ref|Os01g24190| gi|6322279|ref|NP_012354.1| gi|19114852|ref|NP_593940.1|
gi|31197195|ref|XP_307545.1| lcl|At1g69160.1 lcl|C18G1.9 CE17425 WBGene0 gi|24584639| gi|32967303|ref|NP_872602.1| gi|6702361|ref|Os01g24230| gi|6322288|ref|NP_012362.1| gi|19114858|ref|NP_593946.1|
gi|31197215|ref|XP_307555.1| lcl|At1g69280.1 lcl|C18H7.5 CE17430 WBGene0 gi|24584641| gi|33300645|ref|NP_877430.1| gi|6702365|ref|Os01g24270| gi|6322291|ref|NP_012365.1| gi|19114866|ref|NP_593954.1|
gi|31197227|ref|XP_307561.1| lcl|At1g69320.1 lcl|C18H7.7 CE17432 WBGene0 gi|24584643| gi|33300647|ref|NP_877431.1| gi|6702366|ref|Os01g24280| gi|6322300|ref|NP_012374.1| gi|19114868|ref|NP_593956.1|
gi|31197231|ref|XP_307563.1| lcl|At1g69470.1 lcl|C18H9.1 CE01801 WBGene0 gi|24584760| gi|33438590|ref|NP_149042.1| gi|6702367|ref|Os01g24290| gi|6322303|ref|NP_012377.1| gi|19114873|ref|NP_593961.1|
gi|31197243|ref|XP_307569.1| lcl|At1g69760.1 lcl|C18H9.2 CE01802 WBGene0 gi|24584771| gi|33457299|ref|NP_878251.1| gi|6702372|ref|Os01g24340| gi|6322313|ref|NP_012387.1| gi|19114879|ref|NP_593967.1|
gi|31197247|ref|XP_307571.1| lcl|At1g69970.1 lcl|C18H9.6 CE01806 WBGene0 gi|24584778| gi|33667099|ref|NP_878903.1| gi|6702373|ref|Os01g24350| gi|6322314|ref|NP_012388.1| gi|19114890|ref|NP_593978.1|
gi|31197253|ref|XP_307574.1| lcl|At1g70220.1 lcl|C23F12.3 CE04047 WBGene gi|24584780| gi|34147409|ref|NP_115660.2| gi|6702375|ref|Os01g24370| gi|6322317|ref|NP_012391.1| gi|19114905|ref|NP_593993.1|
gi|31197293|ref|XP_307594.1| lcl|At1g70270.1 lcl|C23F12.4 CE04048 WBGene gi|24584786| gi|34916008|ref|NP_919262.1| gi|6702377|ref|Os01g24390| gi|6322338|ref|NP_012412.1| gi|19114918|ref|NP_594006.1|
gi|31197295|ref|XP_307595.1| lcl|At1g70350.1 lcl|C23G10.10 CE04056 WBGer gi|24584788| gi|34916012|ref|NP_919264.1| gi|6702379|ref|Os01g24410| gi|6322339|ref|NP_012413.1| gi|19114919|ref|NP_594007.1|
gi|31197297|ref|XP_307596.1| lcl|At1g70420.1 lcl|C23G10.11 CE04057 WBGer gi|24584790| gi|34916018|ref|NP_919266.1| gi|6702381|ref|Os01g24430| gi|6322342|ref|NP_012417.1| gi|19114935|ref|NP_594023.1|
gi|31197299|ref|XP_307597.1| lcl|At1g70470.1 lcl|C23H3.1 CE08320 WBGene0 gi|24584796| gi|34916030|ref|NP_919280.1| gi|6702382|ref|Os01g24440| gi|6322344|ref|NP_012418.1| gi|19114937|ref|NP_594025.1|
gi|31197301|ref|XP_307598.1| lcl|At1g70895.1 lcl|C23H3.2a CE27078 WBGene gi|24584800| gi|34916038|ref|NP_919286.1| gi|6702383|ref|Os01g24450| gi|6322348|ref|NP_012422.1| gi|19114941|ref|NP_594029.1|
gi|31197303|ref|XP_307599.1| lcl|At1g70985.1 lcl|C23H3.2b CE08321 WBGene gi|24584820| gi|34916040|ref|NP_919281.1| gi|6702387|ref|Os01g24490| gi|6322384|ref|NP_012458.1| gi|19114957|ref|NP_594045.1|
gi|31197325|ref|XP_307610.1| lcl|At1g70990.1 lcl|C23H3.5 CE08324 WBGene0 gi|24584828| gi|34916044|ref|NP_919285.1| gi|6702388|ref|Os01g24500| gi|6322385|ref|NP_012459.1| gi|19114960|ref|NP_594048.1|
gi|31197339|ref|XP_307617.1| lcl|At1g71235.1 lcl|C23H5.2a CE32591 WBGene gi|24584849| gi|34996519|ref|NP_919308.1| gi|6702390|ref|Os01g24520| gi|6322395|ref|NP_012469.1| gi|19114961|ref|NP_594049.1|
gi|31197341|ref|XP_307618.1| lcl|At1g71290.1 lcl|C23H5.3 CE17438 WBGene0 gi|24584865| gi|34996533|ref|NP_919305.1| gi|6702396|ref|Os01g24580| gi|6322397|ref|NP_012470.1| gi|19114962|ref|NP_594050.1|
gi|31197353|ref|XP_307624.1| lcl|At1g71470.1 lcl|C23H5.8a CE17440 WBGene gi|24584887| gi|37537558|ref|NP_931045.1| gi|6702397|ref|Os01g24590| gi|6322403|ref|NP_012477.1| gi|19114963|ref|NP_594051.1|
gi|31197355|ref|XP_307625.1| lcl|At1g71910.1 lcl|C23H5.8b CE17441 WBGene gi|24584891| gi|37537692|ref|NP_002714.2| gi|6702403|ref|Os01g24650| gi|6322410|ref|NP_012484.1| gi|19114968|ref|NP_594056.1|
gi|31197387|ref|XP_307641.1| lcl|At1g71970.1 lcl|C24A1.1 CE32138 WBGene0 gi|24584893| gi|37538595|ref|XP_168521.3| gi|6702405|ref|Os01g24670| gi|6322418|ref|NP_012492.1| gi|19114973|ref|NP_594061.1|
gi|31197389|ref|XP_307642.1| lcl|At1g72080.1 lcl|C24A11.1 CE20521 WBGene gi|24584897| gi|37539574|ref|XP_036612.2| gi|6702410|ref|Os01g24720| gi|6322422|ref|NP_012496.1| gi|19114976|ref|NP_594064.1|
gi|31197397|ref|XP_307646.1| lcl|At1g72240.1 lcl|C24A11.2 CE08343 WBGene gi|24584899| gi|37539846|ref|XP_117514.2| gi|6702411|ref|Os01g24730| gi|6322423|ref|NP_012497.1| gi|19114977|ref|NP_594065.1|
gi|31197405|ref|XP_307650.1| lcl|At1g72490.1 lcl|C24A11.5 CE08346 WBGene gi|24584901| gi|37540869|ref|XP_096864.2| gi|6702414|ref|Os01g24760| gi|6322433|ref|NP_012507.1| gi|19114982|ref|NP_594070.1|
gi|31197413|ref|XP_307654.1| lcl|At1g72580.1 lcl|C24A11.6 CE08347 WBGene gi|24584916| gi|37541728|ref|XP_291974.2| gi|6702415|ref|Os01g24770| gi|6322435|ref|NP_012509.1| gi|19115034|ref|NP_594122.1|
gi|31197429|ref|XP_307662.1| lcl|At1g72600.1 lcl|C24A3.2a CE04059 WBGene gi|24584923| gi|37542496|ref|XP_211291.2| gi|6702421|ref|Os01g24830| gi|6322438|ref|NP_012511.1| gi|19115036|ref|NP_594124.1|
gi|31197445|ref|XP_307670.1| lcl|At1g72790.1 lcl|C24A3.2b CE29184 WBGene gi|24584932| gi|37542882|ref|XP_211009.2| gi|6702422|ref|Os01g24840| gi|6322456|ref|NP_012530.1| gi|19115037|ref|NP_594125.1|
gi|31197459|ref|XP_307677.1| lcl|At1g73090.1 lcl|C24A3.8 CE04065 WBGene0 gi|24584944| gi|37544032|ref|XP_172968.2| gi|6702425|ref|Os01g24870| gi|6322462|ref|NP_012536.1| gi|19115038|ref|NP_594126.1|
gi|31197463|ref|XP_307679.1| lcl|At1g73120.1 lcl|C24A8.5 CE08341 WBGene0 gi|24584950| gi|37544457|ref|XP_294894.2| gi|6702428|ref|Os01g24900| gi|6322463|ref|NP_012537.1| gi|19115039|ref|NP_594127.1|
gi|31197467|ref|XP_307681.1| lcl|At1g73130.1 lcl|C24B5.2b CE30732 WBGene gi|24584961| gi|37544514|ref|XP_209027.2| gi|6702431|ref|Os01g24930| gi|6322471|ref|NP_012545.1| gi|19115044|ref|NP_594132.1|
gi|31197481|ref|XP_307688.1| lcl|At1g73165.1 lcl|C24B9.9 CE17451 WBGene0 gi|24584987| gi|37544657|ref|XP_170840.2| gi|6702435|ref|Os01g24970| gi|6322475|ref|NP_012549.1| gi|19115049|ref|NP_594137.1|
gi|31197505|ref|XP_307700.1| lcl|At1g73177.1 lcl|C24D10.2 CE16875 WBGene gi|24584992| gi|37544665|ref|XP_290743.2| gi|6702440|ref|Os01g25020| gi|6322481|ref|NP_012555.1| gi|19115052|ref|NP_594140.1|
gi|31197531|ref|XP_307713.1| lcl|At1g73510.1 lcl|C24D10.4 CE16876 WBGene gi|24585004| gi|37545646|ref|XP_211408.2| gi|6702443|ref|Os01g25050| gi|6322490|ref|NP_012564.1| gi|19115058|ref|NP_594146.1|
gi|31197533|ref|XP_307714.1| lcl|At1g73770.1 lcl|C24D10.5 CE16877 WBGene gi|24585006| gi|37546095|ref|XP_063138.2| gi|6702445|ref|Os01g25070| gi|6322498|ref|NP_012573.1| gi|19115075|ref|NP_594163.1|
gi|31197561|ref|XP_307728.1| lcl|At1g74045.1 lcl|C24G6.1 CE17456 WBGene0 gi|24585031| gi|37546418|ref|XP_298053.2| gi|6702446|ref|Os01g25080| gi|6322501|ref|NP_012575.1| gi|19115076|ref|NP_594164.1|
gi|31197563|ref|XP_307729.1| lcl|At1g74055.1 lcl|C24H10.2 CE04067 WBGene gi|24585033| gi|37546512|ref|XP_088677.2| gi|6702455|ref|Os01g25170| gi|6322503|ref|NP_012577.1| gi|19115088|ref|NP_594176.1|
gi|31197565|ref|XP_307730.1| lcl|At1g74140.1 lcl|C24H10.3 CE04068 WBGene gi|24585040| gi|37546627|ref|XP_351949.1| gi|6702458|ref|Os01g25200| gi|6322506|ref|NP_012580.1| gi|19115100|ref|NP_594188.1|
gi|31197577|ref|XP_307736.1| lcl|At1g74220.1 lcl|C24H10.4 CE04069 WBGene gi|24585089| gi|37546638|ref|XP_293449.2| gi|6702460|ref|Os01g25220| gi|6322513|ref|NP_012587.1| gi|19115110|ref|NP_594198.1|
gi|31197581|ref|XP_307738.1| lcl|At1g74860.1 lcl|C24H11.5 CE18528 WBGene gi|24585100| gi|37546654|ref|XP_067076.2| gi|6702461|ref|Os01g25230| gi|6322514|ref|NP_012588.1| gi|19115111|ref|NP_594199.1|
gi|31197595|ref|XP_307745.1| lcl|At1g75150.1 lcl|C24H11.9 CE36687 WBGene gi|24585120| gi|37546905|ref|XP_087097.2| gi|6702463|ref|Os01g25250| gi|6322516|ref|NP_012590.1| gi|19115114|ref|NP_594202.1|
gi|31197653|ref|XP_307774.1| lcl|At1g75190.1 lcl|C24H12.10 CE36792 WBGer gi|24585158| gi|37547305|ref|XP_211509.2| gi|6702464|ref|Os01g25260| gi|6322521|ref|NP_012595.1| gi|19115129|ref|NP_594217.1|
gi|31197661|ref|XP_307778.1| lcl|At1g75360.1 lcl|C24H12.2 CE36793 WBGene gi|24585160| gi|37548318|ref|XP_060171.3| gi|6702467|ref|Os01g25290| gi|6322527|ref|NP_012601.1| gi|19115132|ref|NP_594220.1|
gi|31197715|ref|XP_307805.1| lcl|At1g75550.1 lcl|C24H12.5a CE08359 WBGer gi|24585162| gi|37549243|ref|XP_086308.2| gi|6702468|ref|Os01g25300| gi|6322539|ref|NP_012613.1| gi|19115136|ref|NP_594224.1|
gi|31197719|ref|XP_307807.1| lcl|At1g75730.1 lcl|C24H12.5b CE27729 WBGer gi|24585164| gi|37549349|ref|XP_087141.2| gi|6702480|ref|Os01g25420| gi|6322540|ref|NP_012614.1| gi|19115137|ref|NP_594225.1|
gi|31197733|ref|XP_307814.1| lcl|At1g75770.1 lcl|C24H12.6 CE34706 WBGene gi|24585169| gi|37549487|ref|XP_294802.3| gi|6702481|ref|Os01g25430| gi|6322541|ref|NP_012615.1| gi|19115147|ref|NP_594235.1|
gi|31197735|ref|XP_307815.1| lcl|At1g75810.1 lcl|C24H12.7 CE08361 WBGene gi|24585171| gi|37549883|ref|XP_098350.2| gi|6702494|ref|Os01g25550| gi|6322542|ref|NP_012616.1| gi|19115153|ref|NP_594241.1|
gi|31197737|ref|XP_307816.1| lcl|At1g75860.1 lcl|C24H12.8 CE08362 WBGene gi|24585181| gi|37550340|ref|XP_295146.2| gi|6702502|ref|Os01g25620| gi|6322553|ref|NP_012627.1| gi|19115154|ref|NP_594242.1|
gi|31197757|ref|XP_307826.1| lcl|At1g75870.1 lcl|C25A1.15 CE08381 WBGene gi|24585186| gi|37550410|ref|XP_209719.3| gi|6702506|ref|Os01g25660| gi|6322568|ref|NP_012642.1| gi|19115156|ref|NP_594244.1|
gi|31197767|ref|XP_307831.1| lcl|At1g76070.1 lcl|C25A11.1 CE37203 WBGene gi|24585188| gi|37550614|ref|XP_211816.3| gi|6702509|ref|Os01g25690| gi|6322573|ref|NP_012648.1| gi|19115157|ref|NP_594245.1|
gi|31197777|ref|XP_307836.1| lcl|At1g76180.1 lcl|C25A11.2 CE30873 WBGene gi|24585228| gi|37550798|ref|XP_030445.3| gi|6702518|ref|Os01g25770| gi|6322575|ref|NP_012649.1| gi|19115160|ref|NP_594248.1|
gi|31197779|ref|XP_307837.1| lcl|At1g76185.1 lcl|C25A11.4b CE27084 WBGen gi|24585240| gi|37551121|ref|XP_173164.2| gi|6702521|ref|Os01g25800| gi|6322578|ref|NP_012652.1| gi|19115166|ref|NP_594254.1|
gi|31197781|ref|XP_307838.1| lcl|At1g76230.1 lcl|C25A11.4c CE27085 WBGen gi|24585242| gi|37552321|ref|XP_059047.2| gi|6702527|ref|Os01g25850| gi|6322580|ref|NP_012654.1| gi|19115172|ref|NP_594260.1|
gi|31197785|ref|XP_307840.1| lcl|At1g76705.1 lcl|C25A8.1 CE08382 WBGene0 gi|24585273| gi|37552399|ref|XP_294438.3| gi|6702529|ref|Os01g25870| gi|6322595|ref|NP_012669.1| gi|19115176|ref|NP_594264.1|
gi|31197787|ref|XP_307841.1| lcl|At1g76740.1 lcl|C25A8.2 CE31313 WBGene0 gi|24585277| gi|37552412|ref|XP_209889.2| gi|6702533|ref|Os01g25910| gi|6322596|ref|NP_012670.1| gi|19115179|ref|NP_594267.1|
gi|31197789|ref|XP_307842.1| lcl|At1g76820.1 lcl|C25D7.10 CE27731 WBGene gi|24585327| gi|37552465|ref|XP_294456.2| gi|6702534|ref|Os01g25920| gi|6322601|ref|NP_012675.1| gi|19115185|ref|NP_594273.1|
gi|31197809|ref|XP_307852.1| lcl|At1g76840.1 lcl|C25D7.3 CE08389 WBGene0 gi|24585329| gi|37552488|ref|XP_059909.2| gi|6702536|ref|Os01g25940| gi|6322643|ref|NP_012716.1| gi|19115187|ref|NP_594275.1|
gi|31197811|ref|XP_307853.1| lcl|At1g76910.1 lcl|C25D7.9 CE08395 WBGene0 gi|24585343| gi|37552858|ref|XP_086622.2| gi|6702538|ref|Os01g25960| gi|6322645|ref|NP_012718.1| gi|19115190|ref|NP_594278.1|
gi|31197843|ref|XP_307869.1| lcl|At1g76930.1 lcl|C25E10.11 CE06873 WBGen gi|24585348| gi|37555985|ref|XP_293121.2| gi|6702543|ref|Os01g26010| gi|6322648|ref|NP_012721.1| gi|19115192|ref|NP_594280.1|
gi|31197845|ref|XP_307870.1| lcl|At1g76930.2 lcl|C25E10.13 CE37094 WBGen gi|24585350| gi|37556091|ref|XP_097753.3| gi|6702548|ref|Os01g26060| gi|6322661|ref|NP_012734.1| gi|19115193|ref|NP_594281.1|
gi|31197849|ref|XP_307872.1| lcl|At1g76960.1 lcl|C25F6.1 CE02494 WBGene0 gi|24585353| gi|37558316|ref|XP_097792.2| gi|6702549|ref|Os01g26070| gi|6322665|ref|NP_012738.1| gi|19115199|ref|NP_594287.1|
gi|31197851|ref|XP_307873.1| lcl|At1g76965.1 lcl|C25F6.6 CE02498 WBGene0 gi|24585356| gi|37558993|ref|XP_117213.2| gi|6702550|ref|Os01g26080| gi|6322672|ref|NP_012745.1| gi|19115206|ref|NP_594294.1|
gi|31197855|ref|XP_307875.1| lcl|At1g77400.1 lcl|C25F9.4 CE33975 WBGene0 gi|24585363| gi|37559467|ref|XP_066243.2| gi|6702551|ref|Os01g26090| gi|6322681|ref|NP_012754.1| gi|19115208|ref|NP_594296.1|
gi|31197891|ref|XP_307893.1| lcl|At1g77655.1 lcl|C25F9.5 CE17469 WBGene0 gi|24585394| gi|37563667|ref|XP_086894.3| gi|6702552|ref|Os01g26100| gi|6322687|ref|NP_012760.1| gi|19115215|ref|NP_594303.1|
gi|31197909|ref|XP_307902.1| lcl|At1g77765.1 lcl|C25F9.6 CE17470 WBGene0 gi|24585401| gi|37563675|ref|XP_209413.2| gi|6702553|ref|Os01g26110| gi|6322707|ref|NP_012780.1| gi|19115220|ref|NP_594308.1|
gi|31197919|ref|XP_307907.1| lcl|At1g77910.1 lcl|C25F9.8 CE17472 WBGene0 gi|24585403| gi|37620214|ref|NP_932344.1| gi|6702557|ref|Os01g26150| gi|6322711|ref|NP_012784.1| gi|19115223|ref|NP_594311.1|
gi|31197939|ref|XP_307917.1| lcl|At1g77960.1 lcl|C25F9.9 CE17473 WBGene0 gi|24585430| gi|37675381|ref|NP_922942.1| gi|6702560|ref|Os01g26180| gi|6322712|ref|NP_012785.1| gi|19115235|ref|NP_594323.1|
gi|31197951|ref|XP_307923.1| lcl|At1g77970.1 lcl|C25G4.5 CE08398 WBGene0 gi|24585442| gi|37694067|ref|NP_922946.1| gi|6702567|ref|Os01g26250| gi|6322716|ref|NP_012789.1| gi|19115236|ref|NP_594324.1|
gi|31197955|ref|XP_307925.1| lcl|At1g77980.1 lcl|C25G6.1 CE04082 WBGene0 gi|24585444| gi|37704384|ref|NP_775689.1| gi|6702571|ref|Os01g26290| gi|6322719|ref|NP_012792.1| gi|19115241|ref|NP_594329.1|
gi|31197993|ref|XP_307944.1| lcl|At1g78030.1 lcl|C25G6.4 CE37095 WBGene0 gi|24585446| gi|38016133|ref|NP_932345.1| gi|6702577|ref|Os01g26350| gi|6322727|ref|NP_012800.1| gi|19115242|ref|NP_594330.1|
gi|31197999|ref|XP_307947.1| lcl|At1g79170.1 lcl|C25H3.10a CE27090 WBGer gi|24585448| gi|38044110|ref|NP_937824.1| gi|6702579|ref|Os01g26370| gi|6322731|ref|NP_012803.1| gi|19115243|ref|NP_594331.1|
gi|31198033|ref|XP_307964.1| lcl|At1g79615.1 lcl|C25H3.10b CE37612 WBGer gi|24585450| gi|38093641|ref|NP_938013.1| gi|6702580|ref|Os01g26380| gi|6322740|ref|NP_012813.1| gi|19115250|ref|NP_594338.1|
gi|31198059|ref|XP_307977.1| lcl|At1g79780.1 lcl|C25H3.12 CE27092 WBGene gi|24585452| gi|38093665|ref|NP_938025.1| gi|6702582|ref|Os01g26400| gi|6322741|ref|NP_012814.1| gi|19115254|ref|NP_594342.1|
gi|31198063|ref|XP_307979.1| lcl|At1g80180.1 lcl|C25H3.5 CE29185 WBGene0 gi|24585526| gi|38348198|ref|NP_940843.1| gi|6702583|ref|Os01g26410| gi|6322744|ref|NP_012817.1| gi|19115259|ref|NP_594347.1|
gi|31198121|ref|XP_308008.1| lcl|At1g80245.1 lcl|C25H3.6c CE36525 WBGene gi|24585529| gi|38348252|ref|NP_940868.1| gi|6702588|ref|Os01g26950| gi|6322751|ref|NP_012824.1| gi|19115260|ref|NP_594348.1|
gi|31198123|ref|XP_308009.1| lcl|At1g80610.1 lcl|C25H3.9a CE29186 WBGene gi|24585584| gi|38348274|ref|NP_940881.1| gi|6702589|ref|Os01g26960| gi|6322753|ref|NP_012826.1| gi|19115261|ref|NP_594349.1|
gi|31198149|ref|XP_308022.1| lcl|At1g80800.1 lcl|C25H3.9b CE31316 WBGene gi|24585597| gi|38348364|ref|NP_940932.1| gi|6702597|ref|Os01g27030| gi|6322757|ref|NP_012830.1| gi|19115262|ref|NP_594350.1|
gi|31198153|ref|XP_308024.1| lcl|At1g80865.1 lcl|C26B2.2 CE04088 WBGene0 gi|24585618| gi|38348368|ref|NP_940934.1| gi|6702600|ref|Os01g27060| gi|6322761|ref|NP_012834.1| gi|19115265|ref|NP_594353.1|
gi|31198167|ref|XP_308031.1| lcl|At2g01031.1 lcl|C26B2.5 CE04091 WBGene0 gi|24585620| gi|38348372|ref|NP_940939.1| gi|6702605|ref|Os01g27110| gi|6322773|ref|NP_012846.1| gi|19115266|ref|NP_594354.1|
gi|31198175|ref|XP_308035.1| lcl|At2g01310.1 lcl|C26B2.7 CE04093 WBGene0 gi|24585658| gi|38348378|ref|NP_940945.1| gi|6702607|ref|Os01g27130| gi|6322775|ref|NP_012848.1| gi|19115267|ref|NP_594355.1|
gi|31198179|ref|XP_308037.1| lcl|At2g01400.1 lcl|C26B9.2 CE04096 WBGene0 gi|24585692| gi|38373680|ref|NP_941391.1| gi|6702612|ref|Os01g27180| gi|6322778|ref|NP_012851.1| gi|19115285|ref|NP_594373.1|
gi|31198197|ref|XP_308046.1| lcl|At2g01505.1 lcl|C26B9.3 CE29187 WBGene0 gi|24585701| gi|38490573|ref|NP_941964.1| gi|6702614|ref|Os01g27200| gi|6322785|ref|NP_012858.1| gi|19115290|ref|NP_594378.1|
gi|31198199|ref|XP_308047.1| lcl|At2g01800.1 lcl|C26B9.7 CE27733 WBGene0 gi|24585705| gi|38490577|ref|NP_941963.1| gi|6702616|ref|Os01g27220| gi|6322787|ref|NP_012860.1| gi|19115291|ref|NP_594379.1|
gi|31198203|ref|XP_308049.1| lcl|At2g01870.1 lcl|C26C6.9 CE34710 WBGene0 gi|24585707| gi|38490583|ref|NP_941968.1| gi|6702621|ref|Os01g27270| gi|6322789|ref|NP_012862.1| gi|19115298|ref|NP_594386.1|
gi|31198223|ref|XP_308059.1| lcl|At2g01960.1 lcl|C26D10.5c CE31159 WBGen gi|24585732| gi|38502314|ref|NP_942148.1| gi|6702627|ref|Os01g27330| gi|6322793|ref|NP_012866.1| gi|19115307|ref|NP_594395.1|
gi|31198229|ref|XP_308062.1| lcl|At2g02280.1 lcl|C26D10.7 CE36985 WBGene gi|24585734| gi|38708011|ref|NP_944606.1| gi|6702631|ref|Os01g27370| gi|6322796|ref|NP_012869.1| gi|19115312|ref|NP_594400.1|
gi|31198231|ref|XP_308063.1| lcl|At2g02350.1 lcl|C26E1.1 CE08400 WBGene0 gi|24585759| gi|40068065|ref|NP_478060.2| gi|6702634|ref|Os01g27400| gi|6322799|ref|NP_012872.1| gi|19115316|ref|NP_594404.1|
gi|31198233|ref|XP_308064.1| lcl|At2g02440.1 lcl|C26E1.2 CE36581 WBGene0 gi|24585761| gi|40068067|ref|NP_951037.1| gi|6702635|ref|Os01g27410| gi|6322800|ref|NP_012873.1| gi|19115317|ref|NP_594405.1|
gi|31198235|ref|XP_308065.1| lcl|At2g02490.1 lcl|C26E6.7a CE01165 WBGene gi|24585767| gi|40254437|ref|NP_001205.2| gi|6702639|ref|Os01g27450| gi|6322801|ref|NP_012874.1| gi|19115320|ref|NP_594408.1|
gi|31198237|ref|XP_308066.1| lcl|At2g02515.1 lcl|C26E6.7b CE32145 WBGene gi|24585791| gi|40254819|ref|NP_005544.2| gi|6702644|ref|Os01g27500| gi|6322804|ref|NP_012877.1| gi|19115327|ref|NP_594415.1|
gi|31198243|ref|XP_308069.1| lcl|At2g02520.1 lcl|C26F1.10 CE30733 WBGene gi|24585869| gi|40254900|ref|NP_060157.2| gi|6702645|ref|Os01g27510| gi|6322809|ref|NP_012882.1| gi|19115340|ref|NP_594428.1|
gi|31198245|ref|XP_308070.1| lcl|At2g02530.1 lcl|C26F1.1a CE32807 WBGene gi|24585902| gi|40255083|ref|NP_659472.2| gi|6702647|ref|Os01g27530| gi|6322813|ref|NP_012886.1| gi|19115343|ref|NP_594431.1|
gi|31198247|ref|XP_308071.1| lcl|At2g02670.1 lcl|C26F1.1b CE35249 WBGene gi|24585906| gi|40255115|ref|NP_694574.2| gi|6702648|ref|Os01g27540| gi|6322814|ref|NP_012887.1| gi|19115347|ref|NP_594435.1|
gi|31198249|ref|XP_308072.1| lcl|At2g02795.1 lcl|C26F1.3 CE06877 WBGene0 gi|24585908| gi|40255129|ref|NP_689902.2| gi|6702650|ref|Os01g27560| gi|6322829|ref|NP_012902.1| gi|19115359|ref|NP_594447.1|
gi|31198285|ref|XP_308090.1| lcl|At2g02835.1 lcl|C26G2.2 CE37322 WBGene0 gi|24585943| gi|40548382|ref|NP_954857.1| gi|6702651|ref|Os01g27570| gi|6322838|ref|NP_012911.1| gi|19115374|ref|NP_594462.1|
gi|31198305|ref|XP_308100.1| lcl|At2g02840.1 lcl|C27A12.10 CE36794 WBGen gi|24585974| gi|40548384|ref|NP_954858.1| gi|6702656|ref|Os01g27620| gi|6322857|ref|NP_012930.1| gi|19115386|ref|NP_594474.1|
gi|31198315|ref|XP_308105.1| lcl|At2g02950.1 lcl|C27A12.4 CE08413 WBGene gi|24585979| gi|40786396|ref|NP_955370.1| gi|6702663|ref|Os01g27690| gi|6322859|ref|NP_012932.1| gi|19115390|ref|NP_594478.1|
gi|31198317|ref|XP_308106.1| lcl|At2g03010.1 lcl|C27A2.3 CE04103 WBGene0 gi|24586002| gi|40786414|ref|NP_955375.1| gi|6702665|ref|Os01g27710| gi|6322862|ref|NP_012935.1| gi|19115392|ref|NP_594480.1|
gi|31198319|ref|XP_308107.1| lcl|At2g03180.1 lcl|C27A2.5 CE04105 WBGene0 gi|24586042| gi|40789308|ref|NP_955524.1| gi|6702670|ref|Os01g27760| gi|6322863|ref|NP_012936.1| gi|19115398|ref|NP_594486.1|
gi|31198333|ref|XP_308114.1| lcl|At2g03230.1 lcl|C27A7.5b CE20528 WBGene gi|24586082| gi|40804755|ref|NP_955630.1| gi|6702676|ref|Os01g27820| gi|6322867|ref|NP_012940.1| gi|19115425|ref|NP_594513.1|
gi|31198343|ref|XP_308119.1| lcl|At2g03310.1 lcl|C27A7.6 CE08409 WBGene0 gi|24586096| gi|40805108|ref|NP_116565.2| gi|6702677|ref|Os01g27830| gi|6322868|ref|NP_012941.1| gi|19115435|ref|NP_594523.1|
gi|31198361|ref|XP_308128.1| lcl|At2g03320.1 lcl|C27B7.9 CE34713 WBGene0 gi|24586133| gi|41054804|ref|NP_116273.2| gi|6702678|ref|Os01g27840| gi|6322872|ref|NP_012945.1| gi|19115436|ref|NP_594524.1|
gi|31198367|ref|XP_308131.1| lcl|At2g03540.1 lcl|C27C12.1 CE05318 WBGene gi|24586165| gi|41057796|ref|XP_374308.1| gi|6702679|ref|Os01g27850| gi|6322874|ref|NP_012947.1| gi|19115446|ref|NP_594534.1|
gi|31198373|ref|XP_308134.1| lcl|At2g03570.1 lcl|C27C7.1 CE19705 WBGene0 gi|24586167| gi|41057811|ref|XP_374309.1| gi|6702680|ref|Os01g27860| gi|6322877|ref|NP_012950.1| gi|19115477|ref|NP_594565.1|
gi|31198389|ref|XP_308142.1| lcl|At2g03580.1 lcl|C27C7.2 CE18533 WBGene0 gi|24586184| gi|41057813|ref|XP_373856.1| gi|6702682|ref|Os01g27870| gi|6322879|ref|NP_012952.1| gi|19115479|ref|NP_594567.1|
gi|31198409|ref|XP_308152.1| lcl|At2g03830.1 lcl|C27D6.1 CE01811 WBGene0 gi|24586186| gi|41057821|ref|XP_373857.1| gi|6702691|ref|Os01g27970| gi|6322890|ref|NP_012963.1| gi|19115480|ref|NP_594568.1|
gi|31198423|ref|XP_308159.1| lcl|At2g03850.1 lcl|C27D6.3 CE01813 WBGene0 gi|24586193| gi|41058028|ref|XP_371283.1| gi|6702692|ref|Os01g27980| gi|6322893|ref|NP_012967.1| gi|19115486|ref|NP_594574.1|
gi|31198435|ref|XP_308165.1| lcl|At2g04000.1 lcl|C27D8.3 CE08422 WBGene0 gi|24586209| gi|41058037|ref|XP_374364.1| gi|6702700|ref|Os01g28050| gi|6322899|ref|NP_012972.1| gi|19115487|ref|NP_594575.1|
gi|31198441|ref|XP_308168.1| lcl|At2g04025.1 lcl|C27D9.1 CE06891 WBGene0 gi|24586211| gi|41058104|ref|XP_373884.1| gi|6702705|ref|Os01g28100| gi|6322902|ref|NP_012975.1| gi|19115490|ref|NP_594578.1|
gi|31198461|ref|XP_308178.1| lcl|At2g04063.1 lcl|C27D9.2 CE32313 WBGene0 gi|24586222| gi|41058113|ref|XP_373885.1| gi|6702707|ref|Os01g28120| gi|6322928|ref|NP_013001.1| gi|19115493|ref|NP_594581.1|
gi|31198503|ref|XP_308199.1| lcl|At2g04320.1 lcl|C27H2.2 CE15640 WBGene0 gi|24586226| gi|41058121|ref|XP_373886.1| gi|6702710|ref|Os01g28150| gi|6322930|ref|NP_013003.1| gi|19115501|ref|NP_594590.1|
gi|31198517|ref|XP_308206.1| lcl|At2g04330.1 lcl|C27H6.3 CE31321 WBGene0 gi|24586228| gi|41058215|ref|XP_373825.1| gi|6702711|ref|Os01g28150| gi|6322936|ref|NP_013009.1| gi|19115503|ref|NP_594591.1|
gi|31198557|ref|XP_308226.1| lcl|At2g04370.1 lcl|C28A5.5 CE00662 WBGene0 gi|24586309| gi|41058233|ref|XP_373826.1| gi|6702712|ref|Os01g28160| gi|6322938|ref|NP_013011.1| gi|19115513|ref|NP_594601.1|
gi|31198561|ref|XP_308228.1| lcl|At2g04380.1 lcl|C28C12.1 CE04108 WBGene gi|24586324| gi|41058267|ref|XP_373894.1| gi|6702714|ref|Os01g28180| gi|6322941|ref|NP_013014.1| gi|19115523|ref|NP_594611.1|
gi|31198565|ref|XP_308230.1| lcl|At2g04410.1 lcl|C28C12.11 CE29192 WBGer gi|24586332| gi|41058274|ref|XP_373848.1| gi|6702717|ref|Os01g28210| gi|6322954|ref|NP_013027.1| gi|19115527|ref|NP_594615.1|
gi|31198573|ref|XP_308234.1| lcl|At2g04460.1 lcl|C28C12.12 CE28891 WBGer gi|24586338| gi|41058278|ref|XP_373849.1| gi|6702719|ref|Os01g28230| gi|6322974|ref|NP_013046.1| gi|19115528|ref|NP_594616.1|
gi|31198619|ref|XP_308257.1| lcl|At2g04495.1 lcl|C28C12.2 CE04109 WBGene gi|24586367| gi|41058280|ref|XP_373850.1| gi|6702722|ref|Os01g28260| gi|6322979|ref|NP_013051.1| gi|19115529|ref|NP_594617.1|
gi|31198621|ref|XP_308258.1| lcl|At2g04515.1 lcl|C28F5.1 CE01815 WBGene0 gi|24586369| gi|41103623|ref|XP_373853.1| gi|6702724|ref|Os01g28280| gi|6322986|ref|NP_013058.1| gi|19115533|ref|NP_594621.1|
gi|31198629|ref|XP_308262.1| lcl|At2g04600.1 lcl|C28H8.2 CE32812 WBGene0 gi|24586394| gi|41103626|ref|XP_373854.1| gi|6702727|ref|Os01g28310| gi|6322993|ref|NP_013065.1| gi|19115554|ref|NP_594642.1|
gi|31198631|ref|XP_308263.1| lcl|At2g04675.1 lcl|C28H8.7 CE01829 WBGene0 gi|24586427| gi|41104163|ref|XP_373855.1| gi|6702728|ref|Os01g28320| gi|6322995|ref|NP_013067.1| gi|19115559|ref|NP_594647.1|
gi|31198643|ref|XP_308269.1| lcl|At2g04795.1 lcl|C28H8.9b CE32813 WBGene gi|24586437| gi|41106694|ref|XP_373867.1| gi|6702730|ref|Os01g28340| gi|6323006|ref|NP_013078.1| gi|19115567|ref|NP_594655.1|
gi|31198645|ref|XP_308270.1| lcl|At2g04800.1 lcl|C29A12.1 CE05326 WBGene gi|24586470| gi|41106714|ref|XP_371314.1| gi|6702731|ref|Os01g28350| gi|6323015|ref|NP_013087.1| gi|19115571|ref|NP_594659.1|
gi|31198657|ref|XP_308276.1| lcl|At2g04830.1 lcl|C29E4.11 CE29197 WBGene gi|24586479| gi|41107410|ref|XP_373839.1| gi|6702732|ref|Os01g28360| gi|6323022|ref|NP_013094.1| gi|19115574|ref|NP_594662.1|
gi|31198667|ref|XP_308281.1| lcl|At2g04840.1 lcl|C29E4.12 CE30619 WBGene gi|24586485| gi|41107412|ref|XP_373840.1| gi|6702734|ref|Os01g28380| gi|6323025|ref|NP_013097.1| gi|19115588|ref|NP_594676.1|

gi|31198683|ref|XP_308289.1|	lcl|At2g04870.1	lcl|C29E4.13 CE37482 WBGene	gi|24586496|	gi|41107457|ref|XP_373844.1|	gi|6702737|ref|Os01g28410|	gi|6323026|ref|NP_013098.1|	gi|19115600|ref|NP_594688.1|
gi|31198687|ref|XP_308291.1|	lcl|At2g05000.1	lcl|C29E4.2 CE30620 WBGene0	gi|24586506|	gi|41107465|ref|XP_373845.1|	gi|6702739|ref|Os01g28430|	gi|6323027|ref|NP_013099.1|	gi|19115601|ref|NP_594689.1|
gi|31198725|ref|XP_308310.1|	lcl|At2g05025.1	lcl|C29E4.4 CE37484 WBGene0	gi|24586535|	gi|41107472|ref|XP_373846.1|	gi|6702742|ref|Os01g28460|	gi|6323031|ref|NP_013103.1|	gi|19115603|ref|NP_594691.1|
gi|31198727|ref|XP_308311.1|	lcl|At2g05030.1	lcl|C29E4.9 CE29199 WBGene0	gi|24586546|	gi|41107495|ref|XP_373862.1|	gi|6702745|ref|Os01g28490|	gi|6323038|ref|NP_013110.1|	gi|19115608|ref|NP_594696.1|
gi|31198737|ref|XP_308316.1|	lcl|At2g05270.1	lcl|C29F3.3 CE08437 WBGene0	gi|24586548|	gi|41107518|ref|XP_373883.1|	gi|6702747|ref|Os01g28510|	gi|6323046|ref|NP_013118.1|	gi|19115609|ref|NP_594697.1|
gi|31198765|ref|XP_308330.1|	lcl|At2g05290.1	lcl|C29F4.3 CE03040 WBGene0	gi|24586550|	gi|41107684|ref|XP_373832.1|	gi|6702748|ref|Os01g28520|	gi|6323049|ref|NP_013121.1|	gi|19115610|ref|NP_594698.1|
gi|31198833|ref|XP_308364.1|	lcl|At2g05350.1	lcl|C29F5.1 CE02508 WBGene0	gi|24586552|	gi|41107706|ref|XP_373822.1|	gi|6702749|ref|Os01g28530|	gi|6323058|ref|NP_013130.1|	gi|19115613|ref|NP_594701.1|
gi|31198871|ref|XP_308383.1|	lcl|At2g05360.1	lcl|C29F5.2 CE36105 WBGene0	gi|24586558|	gi|41107708|ref|XP_373823.1|	gi|6702752|ref|Os01g28560|	gi|6323059|ref|NP_013131.1|	gi|19115621|ref|NP_594709.1|
gi|31198931|ref|XP_308413.1|	lcl|At2g05380.1	lcl|C29F5.3 CE02510 WBGene0	gi|24586560|	gi|41107712|ref|XP_374362.1|	gi|6702754|ref|Os01g28580|	gi|6323065|ref|NP_013137.1|	gi|19115623|ref|NP_594711.1|
gi|31198937|ref|XP_308416.1|	lcl|At2g05400.1	lcl|C29F5.4 CE33602 WBGene0	gi|24586564|	gi|41107716|ref|XP_373820.1|	gi|6702755|ref|Os01g28590|	gi|6323068|ref|NP_013140.1|	gi|19115632|ref|NP_594720.1|
gi|31198965|ref|XP_308430.1|	lcl|At2g05430.1	lcl|C29F5.5 CE02512 WBGene0	gi|24586572|	gi|41107722|ref|XP_373821.1|	gi|6702757|ref|Os01g28610|	gi|6323070|ref|NP_013141.1|	gi|19115635|ref|NP_594723.1|
gi|31199035|ref|XP_308465.1|	lcl|At2g05440.1	lcl|C29F7.7 CE08448 WBGene0	gi|24586605|	gi|41109027|ref|XP_373835.1|	gi|6702761|ref|Os01g28650|	gi|6323071|ref|NP_013143.1|	gi|19115637|ref|NP_594725.1|
gi|31199061|ref|XP_308478.1|	lcl|At2g05440.2	lcl|C29F9.1 CE08449 WBGene0	gi|24586616|	gi|41109031|ref|XP_373836.1|	gi|6702762|ref|Os01g28660|	gi|6323079|ref|NP_013151.1|	gi|19115638|ref|NP_594726.1|
gi|31199101|ref|XP_308498.1|	lcl|At2g05460.1	lcl|C29F9.12 CE08460 WBGene	gi|24586630|	gi|41109034|ref|XP_373837.1|	gi|6702763|ref|Os01g28670|	gi|6323081|ref|NP_013153.1|	gi|19115647|ref|NP_594735.1|
gi|31199107|ref|XP_308501.1|	lcl|At2g05470.1	lcl|C29F9.14 CE29487 WBGene	gi|24586640|	gi|41109283|ref|XP_373824.1|	gi|6702774|ref|Os01g28700|	gi|6323082|ref|NP_013154.1|	gi|19115655|ref|NP_594743.1|
gi|31199109|ref|XP_308502.1|	lcl|At2g05500.1	lcl|C29F9.2 CE08450 WBGene0	gi|24638484|	gi|41110093|ref|XP_373889.1|	gi|6702775|ref|Os01g28710|	gi|6323092|ref|NP_013164.1|	gi|19115656|ref|NP_594744.1|
gi|31199111|ref|XP_308503.1|	lcl|At2g05510.1	lcl|C29F9.3a CE28896 WBGene	gi|24638519|	gi|41110099|ref|XP_373890.1|	gi|6702776|ref|Os01g28720|	gi|6323093|ref|NP_013165.1|	gi|19115661|ref|NP_594749.1|
gi|31199113|ref|XP_308504.1|	lcl|At2g05520.1	lcl|C29F9.3b CE30623 WBGene	gi|24638571|	gi|41110137|ref|XP_373893.1|	gi|6702779|ref|Os01g28750|	gi|6323097|ref|NP_013169.1|	gi|19115662|ref|NP_594750.1|
gi|31199117|ref|XP_308506.1|	lcl|At2g05530.1	lcl|C29F9.3c CE30624 WBGene	gi|24638573|	gi|41111277|ref|XP_373828.1|	gi|6702781|ref|Os01g28770|	gi|6323100|ref|NP_013172.1|	gi|19115663|ref|NP_594751.1|
gi|31199119|ref|XP_308507.1|	lcl|At2g05540.1	lcl|C29F9.4 CE08452 WBGene0	gi|24638575|	gi|41112046|ref|XP_373872.1|	gi|6702782|ref|Os01g28780|	gi|6323102|ref|NP_013174.1|	gi|19115667|ref|NP_594755.1|
gi|31199121|ref|XP_308508.1|	lcl|At2g05580.1	lcl|C29F9.8 CE08456 WBGene0	gi|24638712|	gi|41112067|ref|XP_373873.1|	gi|6702783|ref|Os01g28790|	gi|6323108|ref|NP_013180.1|	gi|19115672|ref|NP_594760.1|
gi|31199123|ref|XP_308509.1|	lcl|At2g05600.1	lcl|C29F9.9 CE08457 WBGene0	gi|24638728|	gi|41112851|ref|XP_373879.1|	gi|6702785|ref|Os01g28810|	gi|6323111|ref|NP_013183.1|	gi|19115676|ref|NP_594764.1|
gi|31199145|ref|XP_308520.1|	lcl|At2g05645.1	lcl|C29G2.1 CE08462 WBGene0	gi|24638748|	gi|41112861|ref|XP_373880.1|	gi|6702786|ref|Os01g28820|	gi|6323120|ref|NP_013192.1|	gi|19115681|ref|NP_594769.1|
gi|31199169|ref|XP_308532.1|	lcl|At2g05647.1	lcl|C29G2.2 CE08463 WBGene0	gi|24638763|	gi|41113276|ref|XP_373858.1|	gi|6702787|ref|Os01g28830|	gi|6323123|ref|NP_013195.1|	gi|19115687|ref|NP_594775.1|
gi|31199189|ref|XP_308542.1|	lcl|At2g05752.1	lcl|C29G2.3 CE08464 WBGene0	gi|24638815|	gi|41114092|ref|XP_373896.1|	gi|6702789|ref|Os01g28850|	gi|6323124|ref|NP_013196.1|	gi|19115694|ref|NP_594782.1|
gi|31199207|ref|XP_308551.1|	lcl|At2g05915.1	lcl|C29G2.6 CE08467 WBGene0	gi|24638822|	gi|41114107|ref|XP_294993.3|	gi|6702793|ref|Os01g28890|	gi|6323131|ref|NP_013203.1|	gi|19115696|ref|NP_594784.1|
gi|31199231|ref|XP_308563.1|	lcl|At2g05950.1	lcl|C29H12.6 CE29683 WBGene	gi|24638824|	gi|41114111|ref|XP_295598.3|	gi|6702796|ref|Os01g28920|	gi|6323133|ref|NP_013205.1|	gi|19115707|ref|NP_594795.1|
gi|31199237|ref|XP_308566.1|	lcl|At2g06095.1	lcl|C30A5.6 CE29204 WBGene0	gi|24638826|	gi|41114124|ref|XP_371355.1|	gi|6702797|ref|Os01g28930|	gi|6323139|ref|NP_013211.1|	gi|19115714|ref|NP_594802.1|
gi|31199273|ref|XP_308584.1|	lcl|At2g06140.1	lcl|C30B5.1 CE37325 WBGene0	gi|24638828|	gi|41114796|ref|XP_373859.1|	gi|6702798|ref|Os01g28940|	gi|6323148|ref|NP_013220.1|	gi|19115724|ref|NP_594812.1|
gi|31199275|ref|XP_308585.1|	lcl|At2g06165.1	lcl|C30B5.6 CE32155 WBGene0	gi|24638864|	gi|41114914|ref|XP_373814.1|	gi|6702801|ref|Os01g28970|	gi|6323154|ref|NP_013226.1|	gi|19115729|ref|NP_594817.1|
gi|31199279|ref|XP_308587.1|	lcl|At2g06220.1	lcl|C30C11.1 CE00100 WBGene	gi|24638866|	gi|41114917|ref|XP_373815.1|	gi|6702807|ref|Os01g29030|	gi|6323161|ref|NP_013233.1|	gi|19115732|ref|NP_594820.1|
gi|31199287|ref|XP_308591.1|	lcl|At2g06230.1	lcl|C30D11.2 CE00908 WBGene	gi|24638908|	gi|41114968|ref|XP_373817.1|	gi|6702808|ref|Os01g29040|	gi|6323164|ref|NP_013236.1|	gi|19115750|ref|NP_594838.1|
gi|31199315|ref|XP_308605.1|	lcl|At2g06260.1	lcl|C30E1.2 CE08469 WBGene0	gi|24638954|	gi|41115072|ref|XP_373818.1|	gi|6702810|ref|Os01g29060|	gi|6323166|ref|NP_013238.1|	gi|19115753|ref|NP_594841.1|
gi|31199329|ref|XP_308612.1|	lcl|At2g06390.1	lcl|C30E1.3 CE08470 WBGene0	gi|24638956|	gi|41115261|ref|XP_373827.1|	gi|6702818|ref|Os01g29140|	gi|6323168|ref|NP_013240.1|	gi|19115756|ref|NP_594844.1|
gi|31199331|ref|XP_308613.1|	lcl|At2g06420.1	lcl|C30E1.6 CE35415 WBGene0	gi|24638961|	gi|41115523|ref|XP_373864.1|	gi|6702821|ref|Os01g29170|	gi|6323170|ref|NP_013242.1|	gi|19115757|ref|NP_594845.1|
gi|31199413|ref|XP_308654.1|	lcl|At2g06480.1	lcl|C30E1.7 CE08474 WBGene0	gi|24638963|	gi|41117716|ref|XP_371478.1|	gi|6702822|ref|Os01g29180|	gi|6323174|ref|NP_013246.1|	gi|19115767|ref|NP_594855.1|
gi|31199417|ref|XP_308656.1|	lcl|At2g06555.1	lcl|C30F12.3 CE16883 WBGene	gi|24638974|	gi|41118743|ref|XP_374002.1|	gi|6702825|ref|Os01g29210|	gi|6323183|ref|NP_013255.1|	gi|19115772|ref|NP_594860.1|
gi|31199449|ref|XP_308672.1|	lcl|At2g06570.1	lcl|C30F12.4 CE16884 WBGene	gi|24638976|	gi|41118746|ref|XP_374003.1|	gi|6702833|ref|Os01g29290|	gi|6323202|ref|NP_013274.1|	gi|19115780|ref|NP_594868.1|
gi|31199451|ref|XP_308673.1|	lcl|At2g06620.1	lcl|C30F12.5 CE16885 WBGene	gi|24638978|	gi|41118757|ref|XP_374005.1|	gi|6702834|ref|Os01g29300|	gi|6323210|ref|NP_013282.1|	gi|19115782|ref|NP_594870.1|
gi|31199463|ref|XP_308679.1|	lcl|At2g06630.1	lcl|C30F2.3 CE35416 WBGene0	gi|24638980|	gi|41118760|ref|XP_374006.1|	gi|6702839|ref|Os01g29350|	gi|6323219|ref|NP_013291.1|	gi|19115787|ref|NP_594875.1|
gi|31199467|ref|XP_308681.1|	lcl|At2g06640.1	lcl|C30F2.4 CE34718 WBGene0	gi|24638991|	gi|41119848|ref|XP_373998.1|	gi|6702840|ref|Os01g29360|	gi|6323221|ref|NP_013293.1|	gi|19115788|ref|NP_594876.1|
gi|31199477|ref|XP_308686.1|	lcl|At2g06645.1	lcl|C30F2.5 CE34719 WBGene0	gi|24638993|	gi|41120020|ref|XP_374029.1|	gi|6702846|ref|Os01g29410|	gi|6323223|ref|NP_013295.1|	gi|19115796|ref|NP_594884.1|
gi|31199499|ref|XP_308697.1|	lcl|At2g06700.1	lcl|C30F8.3 CE30255 WBGene0	gi|24639008|	gi|41123795|ref|XP_373975.1|	gi|6702849|ref|Os01g29440|	gi|6323233|ref|NP_013305.1|	gi|19115799|ref|NP_594887.1|
gi|31199503|ref|XP_308699.1|	lcl|At2g06730.1	lcl|C30G12.3 CE01834 WBGene	gi|24639011|	gi|41123816|ref|XP_373977.1|	gi|6702853|ref|Os01g29480|	gi|6323248|ref|NP_013320.1|	gi|19115802|ref|NP_594890.1|
gi|31199505|ref|XP_308700.1|	lcl|At2g06735.1	lcl|C30G4.4a CE37205 WBGene	gi|24639023|	gi|41123820|ref|XP_373978.1|	gi|6702855|ref|Os01g29500|	gi|6323250|ref|NP_013322.1|	gi|19115812|ref|NP_594900.1|
gi|31199509|ref|XP_308702.1|	lcl|At2g06750.1	lcl|C30G4.4b CE37206 WBGene	gi|24639027|	gi|41123976|ref|XP_373973.1|	gi|6702860|ref|Os01g29550|	gi|6323252|ref|NP_013324.1|	gi|19115818|ref|NP_594906.1|
gi|31199521|ref|XP_308708.1|	lcl|At2g06775.1	lcl|C30G4.5 CE06901 WBGene0	gi|24639029|	gi|41124485|ref|XP_373995.1|	gi|6702862|ref|Os01g29570|	gi|6323253|ref|NP_013325.1|	gi|19115821|ref|NP_594909.1|
gi|31199539|ref|XP_308717.1|	lcl|At2g06820.1	lcl|C30H6.10 CE31160 WBGene	gi|24639050|	gi|41125702|ref|XP_374011.1|	gi|6702865|ref|Os01g29600|	gi|6323256|ref|NP_013328.1|	gi|19115822|ref|NP_594910.1|
gi|31199559|ref|XP_308727.1|	lcl|At2g06902.1	lcl|C30H6.11 CE34720 WBGene	gi|24639052|	gi|41125718|ref|XP_374012.1|	gi|6702866|ref|Os01g29610|	gi|6323262|ref|NP_013334.1|	gi|19115823|ref|NP_594911.1|
gi|31199571|ref|XP_308733.1|	lcl|At2g06904.1	lcl|C30H6.9 CE19707 WBGene0	gi|24639070|	gi|41125736|ref|XP_371568.1|	gi|6702868|ref|Os01g29630|	gi|6323283|ref|NP_013355.1|	gi|19115825|ref|NP_594913.1|
gi|31199581|ref|XP_308738.1|	lcl|At2g06906.1	lcl|C31B8.4 CE08484 WBGene0	gi|24639072|	gi|41125742|ref|XP_374013.1|	gi|6702874|ref|Os01g29690|	gi|6323286|ref|NP_013358.1|	gi|19115834|ref|NP_594922.1|
gi|31199583|ref|XP_308739.1|	lcl|At2g06908.1	lcl|C31C9.7 CE34721 WBGene0	gi|24639074|	gi|41125748|ref|XP_374014.1|	gi|6702877|ref|Os01g29720|	gi|6323292|ref|NP_013364.1|	gi|19115839|ref|NP_594927.1|
gi|31199595|ref|XP_308745.1|	lcl|At2g06915.1	lcl|C31E10.2 CE08502 WBGene	gi|24639076|	gi|41126355|ref|XP_374007.1|	gi|6702882|ref|Os01g29770|	gi|6323293|ref|NP_013365.1|	gi|19115840|ref|NP_594928.1|
gi|31199605|ref|XP_308750.1|	lcl|At2g06970.1	lcl|C31E10.5 CE08505 WBGene	gi|24639079|	gi|41126358|ref|XP_374008.1|	gi|6702884|ref|Os01g29790|	gi|6323295|ref|NP_013367.1|	gi|19115845|ref|NP_594933.1|
gi|31199607|ref|XP_308751.1|	lcl|At2g07000.1	lcl|C31E10.6 CE08506 WBGene	gi|24639081|	gi|41126367|ref|XP_374009.1|	gi|6702886|ref|Os01g29810|	gi|6323297|ref|NP_013369.1|	gi|19115856|ref|NP_594944.1|
gi|31199617|ref|XP_308756.1|	lcl|At2g07090.1	lcl|C31G12.1 CE15660 WBGene	gi|24639086|	gi|41126370|ref|XP_374010.1|	gi|6702888|ref|Os01g29830|	gi|6323303|ref|NP_013375.1|	gi|19115864|ref|NP_594952.1|
gi|31199673|ref|XP_308784.1|	lcl|At2g07130.1	lcl|C31G12.3 CE15662 WBGene	gi|24639092|	gi|41126919|ref|XP_374028.1|	gi|6702889|ref|Os01g29830|	gi|6323312|ref|NP_013385.1|	gi|19115865|ref|NP_594953.1|
gi|31199675|ref|XP_308785.1|	lcl|At2g07190.1	lcl|C31G12.4 CE15663 WBGene	gi|24639122|	gi|41126926|ref|XP_371605.1|	gi|6702892|ref|Os01g29860|	gi|6323317|ref|NP_013389.1|	gi|19115872|ref|NP_594960.1|
gi|31199679|ref|XP_308787.1|	lcl|At2g07215.1	lcl|C31H1.1 CE04122 WBGene0	gi|24639157|	gi|41127707|ref|XP_373974.1|	gi|6702895|ref|Os01g29890|	gi|6323319|ref|NP_013391.1|	gi|19115875|ref|NP_594963.1|
gi|31199685|ref|XP_308790.1|	lcl|At2g07280.1	lcl|C31H1.6a CE31326 WBGene	gi|24639160|	gi|41133989|ref|XP_374016.1|	gi|6702898|ref|Os01g29920|	gi|6323326|ref|NP_013398.1|	gi|19115876|ref|NP_594964.1|
gi|31199741|ref|XP_308818.1|	lcl|At2g07290.1	lcl|C31H1.6b CE32600 WBGene	gi|24639166|	gi|41134018|ref|XP_374017.1|	gi|6702906|ref|Os01g30990|	gi|6323328|ref|NP_013400.1|	gi|19115885|ref|NP_594973.1|
gi|31199759|ref|XP_308827.1|	lcl|At2g07300.1	lcl|C31H1.7 CE04127 WBGene0	gi|24639168|	gi|41137122|ref|XP_374042.1|	gi|6702912|ref|Os01g31050|	gi|6323332|ref|NP_013404.1|	gi|19115888|ref|NP_594976.1|
gi|31199769|ref|XP_308832.1|	lcl|At2g07310.1	lcl|C31H1.8 CE27808 WBGene0	gi|24639170|	gi|41137142|ref|XP_371653.1|	gi|6702916|ref|Os01g31090|	gi|6323343|ref|NP_013415.1|	gi|19115893|ref|NP_594981.1|
gi|31199779|ref|XP_308837.1|	lcl|At2g07505.1	lcl|C31H2.3 CE35417 WBGene0	gi|24639172|	gi|41144270|ref|XP_374033.1|	gi|6702917|ref|Os01g31100|	gi|6323347|ref|NP_013419.1|	gi|19115897|ref|NP_594985.1|
gi|31199781|ref|XP_308838.1|	lcl|At2g07510.1	lcl|C31H5.4 CE17484 WBGene0	gi|24639208|	gi|41144311|ref|XP_374035.1|	gi|6702919|ref|Os01g31120|	gi|6323352|ref|NP_013424.1|	gi|19115898|ref|NP_594986.1|
gi|31199793|ref|XP_308844.1|	lcl|At2g07672.1	lcl|C31H5.5 CE17485 WBGene0	gi|24639210|	gi|41144323|ref|XP_371623.1|	gi|6702920|ref|Os01g31130|	gi|6323358|ref|NP_013430.1|	gi|19115914|ref|NP_595002.1|
gi|31199807|ref|XP_308851.1|	lcl|At2g07673.1	lcl|C31H5.7 CE34724 WBGene0	gi|24639228|	gi|41144329|ref|XP_374036.1|	gi|6702924|ref|Os01g31170|	gi|6323359|ref|NP_013431.1|	gi|19115916|ref|NP_595004.1|
gi|31199811|ref|XP_308853.1|	lcl|At2g07674.1	lcl|C32A3.1a CE27810 WBGene	gi|24639232|	gi|41144339|ref|XP_374037.1|	gi|6702927|ref|Os01g31200|	gi|6323361|ref|NP_013433.1|	gi|19115922|ref|NP_595010.1|
gi|31199813|ref|XP_308854.1|	lcl|At2g07676.1	lcl|C32A3.1b CE01505 WBGene	gi|24639246|	gi|41144355|ref|XP_374038.1|	gi|6702928|ref|Os01g31210|	gi|6323373|ref|NP_013445.1|	gi|19115931|ref|NP_595019.1|
gi|31199819|ref|XP_308857.1|	lcl|At2g07678.1	lcl|C32A3.2 CE01506 WBGene0	gi|24639260|	gi|41144858|ref|XP_371683.1|	gi|6702935|ref|Os01g31280|	gi|6323378|ref|NP_013450.1|	gi|19115942|ref|NP_595030.1|
gi|31199831|ref|XP_308863.1|	lcl|At2g07679.1	lcl|C32A9.1 CE02152 WBGene0	gi|24639301|	gi|41144865|ref|XP_374070.1|	gi|6702937|ref|Os01g31300|	gi|6323385|ref|NP_013457.1|	gi|19115943|ref|NP_595031.1|
gi|31199835|ref|XP_308865.1|	lcl|At2g07691.1	lcl|C32B5.10 CE08518 WBGene	gi|24639304|	gi|41144870|ref|XP_374071.1|	gi|6702938|ref|Os01g31310|	gi|6323388|ref|NP_013460.1|	gi|19115945|ref|NP_595033.1|
gi|31199837|ref|XP_308866.1|	lcl|At2g07692.1	lcl|C32B5.11 CE08519 WBGene	gi|24639314|	gi|41144884|ref|XP_371684.1|	gi|6702940|ref|Os01g31330|	gi|6323392|ref|NP_013464.1|	gi|19115948|ref|NP_595036.1|
gi|31199841|ref|XP_308868.1|	lcl|At2g07701.1	lcl|C32B5.12 CE31426 WBGene	gi|24639343|	gi|41146526|ref|XP_374045.1|	gi|6702941|ref|Os01g31340|	gi|6323405|ref|NP_013477.1|	gi|19115949|ref|NP_595037.1|
gi|31199843|ref|XP_308869.1|	lcl|At2g07702.1	lcl|C32B5.14 CE08522 WBGene	gi|24639402|	gi|41146542|ref|XP_374046.1|	gi|6702945|ref|Os01g31380|	gi|6323408|ref|NP_013480.1|	gi|19115951|ref|NP_595039.1|
gi|31199867|ref|XP_308881.1|	lcl|At2g07705.1	lcl|C32B5.15 CE08523 WBGene	gi|24639404|	gi|41146554|ref|XP_374048.1|	gi|6702953|ref|Os01g31460|	gi|6323413|ref|NP_013485.1|	
gi|31199869|ref|XP_308882.1|	lcl|At2g07706.1	lcl|C32B5.16 CE08524 WBGene	gi|24639420|	gi|41146561|ref|XP_374049.1|	gi|6702955|ref|Os01g31480|	gi|6323417|ref|NP_013489.1|	
gi|31199871|ref|XP_308883.1|	lcl|At2g07708.1	lcl|C32B5.1a CE25802 WBGene	gi|24639430|	gi|41146572|ref|XP_374050.1|	gi|6702956|ref|Os01g31490|	gi|6323422|ref|NP_013494.1|	
gi|31199951|ref|XP_308923.1|	lcl|At2g07710.1	lcl|C32B5.1b CE32314 WBGene	gi|24639434|	gi|41146574|ref|XP_374051.1|	gi|6702957|ref|Os01g31500|	gi|6323424|ref|NP_013496.1|	
gi|31199965|ref|XP_308930.1|	lcl|At2g07713.1	lcl|C32B5.3 CE08511 WBGene0	gi|24639436|	gi|41146580|ref|XP_374052.1|	gi|6702959|ref|Os01g31520|	gi|6323436|ref|NP_013508.1|	
gi|31199979|ref|XP_308937.1|	lcl|At2g07719.1	lcl|C32B5.4 CE08512 WBGene0	gi|24639438|	gi|41146582|ref|XP_371665.1|	gi|6702960|ref|Os01g31530|	gi|6323440|ref|NP_013512.1|	
gi|31199997|ref|XP_308946.1|	lcl|At2g07720.1	lcl|C32B5.5 CE34725 WBGene0	gi|24639440|	gi|41146584|ref|XP_371666.1|	gi|6702963|ref|Os01g31560|	gi|6323445|ref|NP_013517.1|	
gi|31200015|ref|XP_308955.1|	lcl|At2g07721.1	lcl|C32B5.6 CE08514 WBGene0	gi|24639442|	gi|41146588|ref|XP_374053.1|	gi|6702964|ref|Os01g31570|	gi|6323446|ref|NP_013518.1|	
gi|31200053|ref|XP_308974.1|	lcl|At2g07722.1	lcl|C32B5.8 CE08516 WBGene0	gi|24639448|	gi|41146590|ref|XP_374054.1|	gi|6702966|ref|Os01g31590|	gi|6323447|ref|NP_013519.1|	
gi|31200057|ref|XP_308976.1|	lcl|At2g07724.1	lcl|C32B5.9 CE08517 WBGene0	gi|24639459|	gi|41146600|ref|XP_374055.1|	gi|6702967|ref|Os01g31600|	gi|6323453|ref|NP_013525.1|	
gi|31200065|ref|XP_308980.1|	lcl|At2g07728.1	lcl|C32C4.3 CE37878 WBGene0	gi|24639466|	gi|41146604|ref|XP_374056.1|	gi|6702974|ref|Os01g31670|	gi|6323463|ref|NP_013535.1|	
gi|31200083|ref|XP_308989.1|	lcl|At2g07738.1	lcl|C32D5.1 CE34726 WBGene0	gi|24639498|	gi|41146614|ref|XP_374060.1|	gi|6702978|ref|Os01g31700|	gi|6323468|ref|NP_013540.1|	
gi|31200089|ref|XP_308992.1|	lcl|At2g07749.1	lcl|C32D5.4 CE01844 WBGene0	gi|24639500|	gi|41146629|ref|XP_374062.1|	gi|6702980|ref|Os01g31720|	gi|6323469|ref|NP_013541.1|	
gi|31200093|ref|XP_308994.1|	lcl|At2g07760.1	lcl|C32D5.7 CE01847 WBGene0	gi|24639502|	gi|41146649|ref|XP_374064.1|	gi|6702986|ref|Os01g31780|	gi|6323476|ref|NP_013548.1|	
gi|31200099|ref|XP_308997.1|	lcl|At2g07771.1	lcl|C32E12.1 CE04133 WBGene	gi|24639527|	gi|41146651|ref|XP_374065.1|	gi|6702987|ref|Os01g31790|	gi|6323486|ref|NP_013558.1|	
gi|31200107|ref|XP_309001.1|	lcl|At2g07772.1	lcl|C32E12.3 CE30495 WBGene	gi|24639529|	gi|41146653|ref|XP_374066.1|	gi|6702990|ref|Os01g31820|	gi|6323491|ref|NP_013562.1|	
gi|31200109|ref|XP_309002.1|	lcl|At2g07774.1	lcl|C32E12.4 CE30496 WBGene	gi|24639555|	gi|41146655|ref|XP_374067.1|	gi|6702991|ref|Os01g31830|	gi|6323495|ref|NP_013567.1|	
gi|31200113|ref|XP_309004.1|	lcl|At2g07775.1	lcl|C32E8.10f CE23566 WBGen	gi|24639598|	gi|41146669|ref|XP_374082.1|	gi|6702993|ref|Os01g31850|	gi|6323506|ref|NP_013577.1|	
gi|31200119|ref|XP_309007.1|	lcl|At2g07776.1	lcl|C32E8.4 CE29692 WBGene0	gi|24639601|	gi|41146673|ref|XP_028217.2|	gi|6702994|ref|Os01g31860|	gi|6323516|ref|NP_013587.1|	
gi|31200125|ref|XP_309010.1|	lcl|At2g07981.1	lcl|C32F10.4 CE08541 WBGene	gi|24639603|	gi|41146691|ref|XP_371710.1|	gi|6702998|ref|Os01g31880|	gi|6323522|ref|NP_013593.1|	
gi|31200157|ref|XP_309026.1|	lcl|At2g08986.1	lcl|C32H11.7 CE19714 WBGene	gi|24639605|	gi|41146693|ref|XP_371711.1|	gi|6702999|ref|Os01g31890|	gi|6323523|ref|NP_013594.1|	
gi|31200159|ref|XP_309027.1|	lcl|At2g09388.1	lcl|C33A12.19 CE33757 WBGen	gi|24639613|	gi|41146697|ref|XP_374088.1|	gi|6703001|ref|Os01g31910|	gi|6323524|ref|NP_013595.1|	
gi|31200165|ref|XP_309030.1|	lcl|At2g09840.1	lcl|C33A12.2 CE05348 WBGene	gi|24639621|	gi|41146700|ref|XP_374089.1|	gi|6703002|ref|Os01g31920|	gi|6323533|ref|NP_013604.1|	
gi|31200167|ref|XP_309031.1|	lcl|At2g09865.1	lcl|C33A12.4 CE05350 WBGene	gi|24639632|	gi|41146704|ref|XP_374090.1|	gi|6703003|ref|Os01g31930|	gi|6323548|ref|NP_013619.1|	
gi|31200179|ref|XP_309037.1|	lcl|At2g09900.1	lcl|C33B4.2 CE01507 WBGene0	gi|24639641|	gi|41146706|ref|XP_374091.1|	gi|6703005|ref|Os01g31950|	gi|6323556|ref|NP_013627.1|	
gi|31200195|ref|XP_309045.1|	lcl|At2g10020.1	lcl|C33B4.4 CE34727 WBGene0	gi|24639645|	gi|41146710|ref|XP_374092.1|	gi|6703006|ref|Os01g31960|	gi|6323564|ref|NP_013635.1|	
gi|31200197|ref|XP_309046.1|	lcl|At2g10050.1	lcl|C33B4.5 CE34728 WBGene0	gi|24639647|	gi|41146715|ref|XP_374093.1|	gi|6703007|ref|Os01g31970|	gi|6323578|ref|NP_013649.1|	
gi|31200241|ref|XP_309068.1|	lcl|At2g10070.1	lcl|C33C12.1 CE08549 WBGene	gi|24639649|	gi|41146717|ref|XP_374094.1|	gi|6703009|ref|Os01g31990|	gi|6323582|ref|NP_013653.1|	
gi|31200247|ref|XP_309071.1|	lcl|At2g10105.1	lcl|C33C12.10 CE31428 WBGer	gi|24639653|	gi|41146755|ref|XP_374096.1|	gi|6703010|ref|Os01g32000|	gi|6323588|ref|NP_013659.1|	
gi|31200289|ref|XP_309092.1|	lcl|At2g10110.1	lcl|C33C12.4 CE31429 WBGene	gi|24639655|	gi|41146757|ref|XP_374097.1|	gi|6703025|ref|Os01g32140|	gi|6323591|ref|NP_013662.1|	
gi|31200293|ref|XP_309094.1|	lcl|At2g10175.1	lcl|C33C12.7 CE34729 WBGene	gi|24639657|	gi|41146761|ref|XP_374098.1|	gi|6703026|ref|Os01g32150|	gi|6323593|ref|NP_013664.1|	
gi|31200301|ref|XP_309098.1|	lcl|At2g10340.1	lcl|C33D3.3 CE02154 WBGene0	gi|24639661|	gi|41146771|ref|XP_374100.1|	gi|6703027|ref|Os01g32160|	gi|6323595|ref|NP_013666.1|	
gi|31200305|ref|XP_309100.1|	lcl|At2g10360.1	lcl|C33D3.4 CE02155 WBGene0	gi|24639669|	gi|41146773|ref|XP_374101.1|	gi|6703029|ref|Os01g32180|	gi|6323600|ref|NP_013671.1|	
gi|31200307|ref|XP_309101.1|	lcl|At2g10380.1	lcl|C33D3.5 CE31431 WBGene0	gi|24639677|	gi|41146779|ref|XP_374103.1|	gi|6703031|ref|Os01g32200|	gi|6323604|ref|NP_013675.1|	
gi|31200315|ref|XP_309105.1|	lcl|At2g10390.1	lcl|C33D9.3a CE33758 WBGene	gi|24639679|	gi|41146781|ref|XP_374104.1|	gi|6703037|ref|Os01g32260|	gi|6323610|ref|NP_013681.1|	
gi|31200317|ref|XP_309106.1|	lcl|At2g10470.1	lcl|C33D9.3b CE33759 WBGene	gi|24639683|	gi|41146793|ref|XP_374367.1|	gi|6703038|ref|Os01g32270|	gi|6323618|ref|NP_013689.1|	
gi|31200329|ref|XP_309112.1|	lcl|At2g10550.1	lcl|C33D9.5 CE37808 WBGene0	gi|24639690|	gi|41146795|ref|XP_293715.4|	gi|6703043|ref|Os01g32310|	gi|6323621|ref|NP_013692.1|	
gi|31200331|ref|XP_309113.1|	lcl|At2g10555.1	lcl|C33D9.6 CE33760 WBGene0	gi|24639701|	gi|41146797|ref|XP_374368.1|	gi|6703046|ref|Os01g32340|	gi|6323631|ref|NP_013702.1|	
gi|31200335|ref|XP_309115.1|	lcl|At2g10602.1	lcl|C33D9.8 CE03048 WBGene0	gi|24639707|	gi|41146799|ref|XP_374369.1|	gi|6703051|ref|Os01g32380|	gi|6323634|ref|NP_013705.1|	
gi|31200347|ref|XP_309121.1|	lcl|At2g10750.1	lcl|C33D9.9 CE29589 WBGene0	gi|24639709|	gi|41146810|ref|XP_373026.1|	gi|6703054|ref|Os01g32410|	gi|6323637|ref|NP_013708.1|	
gi|31200351|ref|XP_309123.1|	lcl|At2g10805.1	lcl|C33E10.5 CE17492 WBGene	gi|24639711|	gi|41146826|ref|XP_374076.1|	gi|6703056|ref|Os01g32430|	gi|6323640|ref|NP_013711.1|	
gi|31200377|ref|XP_309136.1|	lcl|At2g10850.1	lcl|C33F10.1 CE29209 WBGene	gi|24639754|	gi|41146832|ref|XP_374075.1|	gi|6703057|ref|Os01g32440|	gi|6323652|ref|NP_013723.1|	
gi|31200389|ref|XP_309142.1|	lcl|At2g10870.1	lcl|C33F10.11 CE04143 WBGen	gi|24639756|	gi|41146853|ref|XP_371688.1|	gi|6703058|ref|Os01g32450|	gi|6323656|ref|NP_013727.1|	
gi|31200391|ref|XP_309143.1|	lcl|At2g10880.1	lcl|C33F10.4b CE02531 WBGen	gi|24639760|	gi|41146857|ref|XP_374077.1|	gi|6703059|ref|Os01g32460|	gi|6323667|ref|NP_013738.1|	
gi|31200403|ref|XP_309149.1|	lcl|At2g10885.1	lcl|C33G3.5 CE34158 WBGene0	gi|24639766|	gi|41146862|ref|XP_374078.1|	gi|6703060|ref|Os01g32470|	gi|6323672|ref|NP_013743.1|	
gi|31200409|ref|XP_309152.1|	lcl|At2g10900.1	lcl|C33G3.6 CE31432 WBGene0	gi|24639768|	gi|41146868|ref|XP_371690.1|	gi|6703062|ref|Os01g32490|	gi|6323674|ref|NP_013744.1|	
gi|31200449|ref|XP_309172.1|	lcl|At2g10920.1	lcl|C33G8.2 CE06911 WBGene0	gi|24639770|	gi|41146873|ref|XP_374079.1|	gi|6703064|ref|Os01g32510|	gi|6323692|ref|NP_013763.1|	
gi|31200457|ref|XP_309176.1|	lcl|At2g10930.1	lcl|C33G8.3 CE06912 WBGene0	gi|24639774|	gi|41146911|ref|XP_374105.1|	gi|6703065|ref|Os01g32520|	gi|6323696|ref|NP_013767.1|	
gi|31200477|ref|XP_309186.1|	lcl|At2g10965.1	lcl|C33G8.4 CE06913 WBGene0	gi|24639794|	gi|41146917|ref|XP_374106.1|	gi|6703067|ref|Os01g32540|	gi|6323698|ref|NP_013769.1|	
gi|31200503|ref|XP_309199.1|	lcl|At2g10970.1	lcl|C33G8.5 CE06914 WBGene0	gi|24639796|	gi|41146922|ref|XP_374108.1|	gi|6703069|ref|Os01g32560|	gi|6323704|ref|NP_013775.1|	
gi|31200509|ref|XP_309202.1|	lcl|At2g10975.1	lcl|C33H5.10 CE26878 WBGene	gi|24639810|	gi|41146926|ref|XP_371726.1|	gi|6703075|ref|Os01g32620|	gi|6323705|ref|NP_013776.1|	
gi|31200517|ref|XP_309206.1|	lcl|At2g11005.1	lcl|C33H5.11 CE29696 WBGene	gi|24639812|	gi|41146930|ref|XP_374111.1|	gi|6703077|ref|Os01g32640|	gi|6323708|ref|NP_013779.1|	
gi|31200519|ref|XP_309207.1|	lcl|At2g11015.1	lcl|C33H5.13 CE04156 WBGene	gi|24639814|	gi|41146932|ref|XP_374147.1|	gi|6703078|ref|Os01g32650|	gi|6323709|ref|NP_013780.1|	
gi|31200527|ref|XP_309211.1|	lcl|At2g11090.1	lcl|C33H5.3 CE04147 WBGene0	gi|24639822|	gi|41146947|ref|XP_374148.1|	gi|6703080|ref|Os01g32670|	gi|6323711|ref|NP_013782.1|	
gi|31200543|ref|XP_309219.1|	lcl|At2g11130.1	lcl|C34B2.1 CE36631 WBGene0	gi|24639841|	gi|41146949|ref|XP_374149.1|	gi|6703088|ref|Os01g32740|	gi|6323727|ref|NP_013798.1|	
gi|31200567|ref|XP_309231.1|	lcl|At2g11135.1	lcl|C34B2.11 CE29210 WBGene	gi|24639869|	gi|41146951|ref|XP_374150.1|	gi|6703090|ref|Os01g32760|	gi|6323732|ref|NP_013803.1|	
gi|31200583|ref|XP_309239.1|	lcl|At2g11140.1	lcl|C34B2.9 CE27816 WBGene0	gi|24639876|	gi|41146955|ref|XP_371783.1|	gi|6703095|ref|Os01g32810|	gi|6323741|ref|NP_013812.1|	
gi|31200597|ref|XP_309246.1|	lcl|At2g11160.1	lcl|C34C12.2 CE03056 WBGene	gi|24639878|	gi|41146977|ref|XP_374129.1|	gi|6703098|ref|Os01g32840|	gi|6323745|ref|NP_013816.1|	
gi|31200599|ref|XP_309247.1|	lcl|At2g11165.1	lcl|C34C12.7 CE01515 WBGene	gi|24639885|	gi|41146983|ref|XP_374131.1|	gi|6703100|ref|Os01g32860|	gi|6323754|ref|NP_013825.1|	

gi|31200601|ref|XP_309248.1|	lcl|At2g11260.1	lcl|C34C12.9 CE34734 WBGene	gi|24639891|	gi|41146988|ref|XP_374144.1|	gi|6703104|ref|Os01g32900|	gi|6323758|ref|NP_013829.1|
gi|31200607|ref|XP_309251.1|	lcl|At2g11370.1	lcl|C34C6.2 CE03050 WBGene0	gi|24639900|	gi|41146998|ref|XP_374145.1|	gi|6703105|ref|Os01g32910|	gi|6323759|ref|NP_013830.1|
gi|31200617|ref|XP_309256.1|	lcl|At2g11390.1	lcl|C34C6.7 CE03055 WBGene0	gi|24639904|	gi|41147000|ref|XP_374146.1|	gi|6703108|ref|Os01g32940|	gi|6323764|ref|NP_013835.1|
gi|31200619|ref|XP_309257.1|	lcl|At2g11405.1	lcl|C34D1.4 CE23568 WBGene0	gi|24639924|	gi|41147008|ref|XP_371763.1|	gi|6703109|ref|Os01g32950|	gi|6323766|ref|NP_013837.1|
gi|31200629|ref|XP_309262.1|	lcl|At2g11462.1	lcl|C34D4.1 CE27742 WBGene0	gi|24639962|	gi|41147014|ref|XP_371765.1|	gi|6703110|ref|Os01g32960|	gi|6323772|ref|NP_013843.1|
gi|31200631|ref|XP_309263.1|	lcl|At2g11540.1	lcl|C34D4.11 CE17505 WBGene	gi|24639968|	gi|41147024|ref|XP_374132.1|	gi|6703128|ref|Os01g33130|	gi|6323774|ref|NP_013845.1|
gi|31200633|ref|XP_309264.1|	lcl|At2g11570.1	lcl|C34D4.13 CE34735 WBGene	gi|24639986|	gi|41147028|ref|XP_374133.1|	gi|6703130|ref|Os01g33150|	gi|6323782|ref|NP_013853.1|
gi|31200635|ref|XP_309265.1|	lcl|At2g11600.1	lcl|C34E10.8 CE32158 WBGene	gi|24639988|	gi|41147037|ref|XP_371769.1|	gi|6703133|ref|Os01g33180|	gi|6323784|ref|NP_013854.1|
gi|31200639|ref|XP_309267.1|	lcl|At2g11620.1	lcl|C34E10.9 CE01189 WBGene	gi|24640009|	gi|41147043|ref|XP_374135.1|	gi|6703136|ref|Os01g33210|	gi|6323793|ref|NP_013864.1|
gi|31200653|ref|XP_309274.1|	lcl|At2g11626.1	lcl|C34E11.3 CE08573 WBGene	gi|24640011|	gi|41147045|ref|XP_374136.1|	gi|6703142|ref|Os01g33270|	gi|6323796|ref|NP_013867.1|
gi|31200665|ref|XP_309280.1|	lcl|At2g11775.1	lcl|C34E11.4 CE34736 WBGene	gi|24640021|	gi|41147047|ref|XP_371772.1|	gi|6703143|ref|Os01g33280|	gi|6323797|ref|NP_013868.1|
gi|31200669|ref|XP_309282.1|	lcl|At2g11830.1	lcl|C34E7.1 CE36355 WBGene0	gi|24640128|	gi|41147053|ref|XP_374137.1|	gi|6703144|ref|Os01g33290|	gi|6323798|ref|NP_013869.1|
gi|31200673|ref|XP_309284.1|	lcl|At2g11910.1	lcl|C34E7.3 CE03060 WBGene0	gi|24640190|	gi|41147057|ref|XP_374139.1|	gi|6703146|ref|Os01g33310|	gi|6323805|ref|NP_013876.1|
gi|31200675|ref|XP_309285.1|	lcl|At2g11910.2	lcl|C34E7.4 CE03061 WBGene0	gi|24640220|	gi|41147059|ref|XP_374140.1|	gi|6703149|ref|Os01g33340|	gi|6323807|ref|NP_013878.1|
gi|31200677|ref|XP_309286.1|	lcl|At2g12110.1	lcl|C34F11.2 CE04163 WBGene	gi|24640262|	gi|41147061|ref|XP_374141.1|	gi|6703154|ref|Os01g33390|	gi|6323809|ref|NP_013880.1|
gi|31200707|ref|XP_309301.1|	lcl|At2g12120.1	lcl|C34F11.8 CE36986 WBGene	gi|24640293|	gi|41147079|ref|XP_374121.1|	gi|6703160|ref|Os01g33450|	gi|6323811|ref|NP_013882.1|
gi|31200717|ref|XP_309306.1|	lcl|At2g12130.1	lcl|C34F6.11 CE34737 WBGene	gi|24640295|	gi|41147108|ref|XP_371729.1|	gi|6703162|ref|Os01g33470|	gi|6323812|ref|NP_013883.1|
gi|31200721|ref|XP_309308.1|	lcl|At2g12170.1	lcl|C34F6.5 CE15674 WBGene0	gi|24640298|	gi|41147112|ref|XP_371731.1|	gi|6703163|ref|Os01g33480|	gi|6323815|ref|NP_013886.1|
gi|31200723|ref|XP_309309.1|	lcl|At2g12230.1	lcl|C34F6.6 CE15675 WBGene0	gi|24640342|	gi|41147120|ref|XP_374118.1|	gi|6703164|ref|Os01g33490|	gi|6323824|ref|NP_013895.1|
gi|31200811|ref|XP_309353.1|	lcl|At2g12250.1	lcl|C34F6.7 CE15676 WBGene0	gi|24640360|	gi|41147128|ref|XP_374120.1|	gi|6703166|ref|Os01g33510|	gi|6323826|ref|NP_013897.1|
gi|31200815|ref|XP_309355.1|	lcl|At2g12320.1	lcl|C34G6.3 CE08575 WBGene0	gi|24640362|	gi|41147130|ref|XP_371738.1|	gi|6703170|ref|Os01g33550|	gi|6323829|ref|NP_013900.1|
gi|31200847|ref|XP_309371.1|	lcl|At2g12350.1	lcl|C34H4.5 CE31436 WBGene0	gi|24640368|	gi|41147176|ref|XP_374124.1|	gi|6703171|ref|Os01g33560|	gi|6323833|ref|NP_013904.1|
gi|31200849|ref|XP_309372.1|	lcl|At2g12405.1	lcl|C35A11.2 CE35253 WBGene	gi|24640372|	gi|41147198|ref|XP_374127.1|	gi|6703173|ref|Os01g33580|	gi|6323835|ref|NP_013906.1|
gi|31200869|ref|XP_309382.1|	lcl|At2g12420.1	lcl|C35A11.3 CE08582 WBGene	gi|24640374|	gi|41147201|ref|XP_374128.1|	gi|6703175|ref|Os01g33600|	gi|6323838|ref|NP_013909.1|
gi|31200877|ref|XP_309386.1|	lcl|At2g12505.1	lcl|C35A5.10 CE37809 WBGene	gi|24640377|	gi|41147206|ref|XP_371759.1|	gi|6703180|ref|Os01g33650|	gi|6323851|ref|NP_013922.1|
gi|31200885|ref|XP_309390.1|	lcl|At2g12610.1	lcl|C35A5.11 CE37879 WBGene	gi|24640379|	gi|41147238|ref|XP_374152.1|	gi|6703182|ref|Os01g33670|	gi|6323854|ref|NP_013925.1|
gi|31200891|ref|XP_309393.1|	lcl|At2g12685.1	lcl|C35A5.6 CE05364 WBGene0	gi|24640381|	gi|41147254|ref|XP_371743.1|	gi|6703186|ref|Os01g33710|	gi|6323860|ref|NP_013931.1|
gi|31200903|ref|XP_309399.1|	lcl|At2g12695.1	lcl|C35B1.2a CE29213 WBGene	gi|24640383|	gi|41147264|ref|XP_371749.1|	gi|6703187|ref|Os01g33720|	gi|6323862|ref|NP_013933.1|
gi|31200913|ref|XP_309404.1|	lcl|At2g12700.1	lcl|C35B1.2b CE33521 WBGene	gi|24640385|	gi|41147278|ref|XP_374178.1|	gi|6703191|ref|Os01g33760|	gi|6323865|ref|NP_013936.1|
gi|31200915|ref|XP_309405.1|	lcl|At2g12875.1	lcl|C35B1.2c CE35834 WBGene	gi|24640401|	gi|41147293|ref|XP_374154.1|	gi|6703192|ref|Os01g33770|	gi|6323874|ref|NP_013945.1|
gi|31200951|ref|XP_309423.1|	lcl|At2g12905.1	lcl|C35B1.3 CE16901 WBGene0	gi|24640421|	gi|41147297|ref|XP_374156.1|	gi|6703197|ref|Os01g33820|	gi|6323875|ref|NP_013946.1|
gi|31200955|ref|XP_309425.1|	lcl|At2g12910.1	lcl|C35B1.4 CE16902 WBGene0	gi|24640508|	gi|41147301|ref|XP_374158.1|	gi|6703198|ref|Os01g33830|	gi|6323881|ref|NP_013952.1|
gi|31200959|ref|XP_309427.1|	lcl|At2g12935.1	lcl|C35B1.7 CE27825 WBGene0	gi|24640516|	gi|41147309|ref|XP_371797.1|	gi|6703201|ref|Os01g33860|	gi|6323907|ref|NP_013978.1|
gi|31201023|ref|XP_309459.1|	lcl|At2g12945.1	lcl|C35B1.8 CE34738 WBGene0	gi|24640531|	gi|41147316|ref|XP_374172.1|	gi|6703202|ref|Os01g33870|	gi|6323908|ref|NP_013979.1|
gi|31201025|ref|XP_309460.1|	lcl|At2g13115.1	lcl|C35C5.1 CE18542 WBGene0	gi|24640555|	gi|41147325|ref|XP_374173.1|	gi|6703203|ref|Os01g33880|	gi|6323911|ref|NP_013982.1|
gi|31201029|ref|XP_309462.1|	lcl|At2g13125.1	lcl|C35C5.8a CE28208 WBGene	gi|24640566|	gi|41147329|ref|XP_374174.1|	gi|6703205|ref|Os01g33900|	gi|6323912|ref|NP_013983.1|
gi|31201031|ref|XP_309463.1|	lcl|At2g13145.1	lcl|C35C5.8b CE34161 WBGene	gi|24640570|	gi|41147335|ref|XP_374175.1|	gi|6703207|ref|Os01g33920|	gi|6323913|ref|NP_013984.1|
gi|31201033|ref|XP_309464.1|	lcl|At2g13175.1	lcl|C35C5.9 CE31437 WBGene0	gi|24640572|	gi|41147341|ref|XP_374176.1|	gi|6703209|ref|Os01g33940|	gi|6323914|ref|NP_013985.1|
gi|31201035|ref|XP_309465.1|	lcl|At2g13270.1	lcl|C35D10.13 CE01194 WBGer	gi|24640578|	gi|41147343|ref|XP_374177.1|	gi|6703213|ref|Os01g33980|	gi|6323919|ref|NP_013990.1|
gi|31201037|ref|XP_309466.1|	lcl|At2g13320.1	lcl|C35D10.1b CE30626 WBGer	gi|24640584|	gi|41147352|ref|XP_374194.1|	gi|6703220|ref|Os01g34050|	gi|6323921|ref|NP_013992.1|
gi|31201049|ref|XP_309472.1|	lcl|At2g13335.1	lcl|C35D10.3 CE01197 WBGene	gi|24640601|	gi|41147356|ref|XP_374186.1|	gi|6703222|ref|Os01g34070|	gi|6323926|ref|NP_013997.1|
gi|31201051|ref|XP_309473.1|	lcl|At2g13400.1	lcl|C35D10.7a CE32160 WBGer	gi|24640603|	gi|41147358|ref|XP_374187.1|	gi|6703224|ref|Os01g34090|	gi|6323938|ref|NP_014009.1|
gi|31201053|ref|XP_309474.1|	lcl|At2g13410.1	lcl|C35D10.7b CE32161 WBGer	gi|24640605|	gi|41147360|ref|XP_374188.1|	gi|6703225|ref|Os01g34100|	gi|6323951|ref|NP_014022.1|
gi|31201055|ref|XP_309475.1|	lcl|At2g13430.1	lcl|C35D10.8 CE29702 WBGene	gi|24640607|	gi|41147362|ref|XP_374189.1|	gi|6703226|ref|Os01g34110|	gi|6323953|ref|NP_014024.1|
gi|31201073|ref|XP_309484.1|	lcl|At2g13500.1	lcl|C35D6.3 CE15682 WBGene0	gi|24640613|	gi|41147364|ref|XP_374190.1|	gi|6703227|ref|Os01g34120|	gi|6323956|ref|NP_014027.1|
gi|31201157|ref|XP_309526.1|	lcl|At2g13510.1	lcl|C35E7.1 CE36273 WBGene0	gi|24640623|	gi|41147366|ref|XP_374191.1|	gi|6703228|ref|Os01g34130|	gi|6323971|ref|NP_014042.1|
gi|31201167|ref|XP_309531.1|	lcl|At2g13550.1	lcl|C35E7.11 CE27830 WBGene	gi|24640667|	gi|41147372|ref|XP_371858.1|	gi|6703230|ref|Os01g34150|	gi|6323972|ref|NP_014043.1|
gi|31201193|ref|XP_309544.1|	lcl|At2g13660.1	lcl|C35E7.2a CE29215 WBGene	gi|24640683|	gi|41147374|ref|XP_374192.1|	gi|6703231|ref|Os01g34160|	gi|6323976|ref|NP_014047.1|
gi|31201201|ref|XP_309548.1|	lcl|At2g13730.1	lcl|C35E7.2b CE29216 WBGene	gi|24640685|	gi|41147382|ref|XP_374193.1|	gi|6703233|ref|Os01g34180|	gi|6324007|ref|NP_014077.1|
gi|31201203|ref|XP_309549.1|	lcl|At2g13760.1	lcl|C35E7.3 CE17516 WBGene0	gi|24640687|	gi|41147398|ref|XP_374161.1|	gi|6703236|ref|Os01g34210|	gi|6324015|ref|NP_014085.1|
gi|31201213|ref|XP_309554.1|	lcl|At2g13865.1	lcl|C35E7.4 CE29704 WBGene0	gi|24640699|	gi|41147404|ref|XP_374196.1|	gi|6703238|ref|Os01g34230|	gi|6324029|ref|NP_014099.1|
gi|31201235|ref|XP_309565.1|	lcl|At2g13880.1	lcl|C35E7.5a CE17518 WBGene	gi|24640701|	gi|41147426|ref|XP_374198.1|	gi|6703239|ref|Os01g34240|	gi|6324033|ref|NP_014102.1|
gi|31201241|ref|XP_309568.1|	lcl|At2g14000.1	lcl|C35E7.5b CE32817 WBGene	gi|24640710|	gi|41147456|ref|XP_372004.1|	gi|6703241|ref|Os01g34260|	gi|6324034|ref|NP_014104.1|
gi|31201245|ref|XP_309570.1|	lcl|At2g14020.1	lcl|C35E7.6 CE17519 WBGene0	gi|24640740|	gi|41147458|ref|XP_372005.1|	gi|6703243|ref|Os01g34280|	gi|6324035|ref|NP_014105.1|
gi|31201255|ref|XP_309575.1|	lcl|At2g14095.1	lcl|C35E7.7 CE17520 WBGene0	gi|24640747|	gi|41147465|ref|XP_374370.1|	gi|6703245|ref|Os01g34300|	gi|6324047|ref|NP_014117.1|
gi|31201273|ref|XP_309584.1|	lcl|At2g14240.1	lcl|C35E7.8 CE17521 WBGene0	gi|24640752|	gi|41147481|ref|XP_374218.1|	gi|6703247|ref|Os01g34320|	gi|6324060|ref|NP_014130.1|
gi|31201287|ref|XP_309591.1|	lcl|At2g14270.1	lcl|C36A4.10 CE33979 WBGene	gi|24640756|	gi|41147545|ref|XP_374199.1|	gi|6703252|ref|Os01g34370|	gi|6324068|ref|NP_014138.1|
gi|31201291|ref|XP_309593.1|	lcl|At2g14340.1	lcl|C36B1.6 CE37752 WBGene0	gi|24640777|	gi|41147547|ref|XP_374236.1|	gi|6703259|ref|Os01g34440|	gi|6324071|ref|NP_014141.1|
gi|31201295|ref|XP_309595.1|	lcl|At2g14350.1	lcl|C36B1.8a CE34472 WBGene	gi|24640785|	gi|41147615|ref|XP_374222.1|	gi|6703261|ref|Os01g34460|	gi|6324075|ref|NP_014145.1|
gi|31201297|ref|XP_309596.1|	lcl|At2g14360.1	lcl|C36B1.8b CE34473 WBGene	gi|24640798|	gi|41147637|ref|XP_374226.1|	gi|6703262|ref|Os01g34470|	gi|6324083|ref|NP_014153.1|
gi|31201299|ref|XP_309597.1|	lcl|At2g14390.1	lcl|C36B7.3 CE04174 WBGene0	gi|24640812|	gi|41147641|ref|XP_374227.1|	gi|6703265|ref|Os01g34500|	gi|6324092|ref|NP_014162.1|
gi|31201311|ref|XP_309603.1|	lcl|At2g14410.1	lcl|C36B7.4 CE25805 WBGene0	gi|24640824|	gi|41147690|ref|XP_374710.1|	gi|6703266|ref|Os01g34510|	gi|6324093|ref|NP_014163.1|
gi|31201313|ref|XP_309604.1|	lcl|At2g14460.1	lcl|C36C5.1 CE23572 WBGene0	gi|24640836|	gi|41147692|ref|XP_374711.1|	gi|6703267|ref|Os01g34520|	gi|6324096|ref|NP_014166.1|
gi|31201315|ref|XP_309605.1|	lcl|At2g14590.1	lcl|C36C5.10 CE08599 WBGene	gi|24640867|	gi|41147748|ref|XP_374715.1|	gi|6703269|ref|Os01g34540|	gi|6324104|ref|NP_014174.1|
gi|31201329|ref|XP_309612.1|	lcl|At2g14600.1	lcl|C36C5.11 CE08600 WBGene	gi|24640883|	gi|41147788|ref|XP_374721.1|	gi|6703270|ref|Os01g34550|	gi|6324115|ref|NP_014185.1|
gi|31201331|ref|XP_309613.1|	lcl|At2g14630.1	lcl|C36C5.2 CE08591 WBGene0	gi|24640885|	gi|41147790|ref|XP_374722.1|	gi|6703274|ref|Os01g34590|	gi|6324118|ref|NP_014188.1|
gi|31201347|ref|XP_309621.1|	lcl|At2g14700.1	lcl|C36C5.6 CE29217 WBGene0	gi|24640887|	gi|41147806|ref|XP_290925.2|	gi|6703275|ref|Os01g34600|	gi|6324130|ref|NP_014200.1|
gi|31201361|ref|XP_309628.1|	lcl|At2g14760.1	lcl|C36C5.7 CE08596 WBGene0	gi|24640889|	gi|41147808|ref|XP_374434.1|	gi|6703276|ref|Os01g34610|	gi|6324133|ref|NP_014203.1|
gi|31201383|ref|XP_309639.1|	lcl|At2g14800.1	lcl|C36C5.8 CE08597 WBGene0	gi|24640891|	gi|41147898|ref|XP_374730.1|	gi|6703284|ref|Os01g34650|	gi|6324136|ref|NP_014206.1|
gi|31201385|ref|XP_309640.1|	lcl|At2g14810.1	lcl|C36C9.1 CE19720 WBGene0	gi|24640893|	gi|41147940|ref|XP_374734.1|	gi|6703285|ref|Os01g34660|	gi|6324139|ref|NP_014209.1|
gi|31201391|ref|XP_309643.1|	lcl|At2g14890.1	lcl|C36C9.5 CE19724 WBGene0	gi|24640895|	gi|41147964|ref|XP_374735.1|	gi|6703290|ref|Os01g34710|	gi|6324141|ref|NP_014211.1|
gi|31201397|ref|XP_309646.1|	lcl|At2g15000.1	lcl|C36E6.1a CE30887 WBGene	gi|24640897|	gi|41148008|ref|XP_374741.1|	gi|6703296|ref|Os01g34770|	gi|6324153|ref|NP_014223.1|
gi|31201409|ref|XP_309652.1|	lcl|At2g15000.2	lcl|C36H8.3 CE05382 WBGene0	gi|24640899|	gi|41148130|ref|XP_374751.1|	gi|6703298|ref|Os01g34790|	gi|6324162|ref|NP_014232.1|
gi|31201413|ref|XP_309654.1|	lcl|At2g15185.1	lcl|C37A2.2 CE27747 WBGene0	gi|24640914|	gi|41148273|ref|XP_374638.1|	gi|6703305|ref|Os01g34860|	gi|6324163|ref|NP_014233.1|
gi|31201429|ref|XP_309662.1|	lcl|At2g15327.1	lcl|C37A2.5a CE27748 WBGene	gi|24640918|	gi|41148407|ref|XP_374589.1|	gi|6703310|ref|Os01g34910|	gi|6324164|ref|NP_014234.1|
gi|31201467|ref|XP_309681.1|	lcl|At2g15340.1	lcl|C37A2.5b CE32818 WBGene	gi|24640928|	gi|41148409|ref|XP_374761.1|	gi|6703313|ref|Os01g34940|	gi|6324165|ref|NP_014235.1|
gi|31201473|ref|XP_309684.1|	lcl|At2g15345.1	lcl|C37A2.8a CE08617 WBGene	gi|24640936|	gi|41148413|ref|XP_374591.1|	gi|6703318|ref|Os01g34980|	gi|6324170|ref|NP_014240.1|
gi|31201493|ref|XP_309694.1|	lcl|At2g15360.1	lcl|C37A2.8b CE29218 WBGene	gi|24640947|	gi|41148425|ref|XP_374764.1|	gi|6703319|ref|Os01g34990|	gi|6324171|ref|NP_014241.1|
gi|31201503|ref|XP_309699.1|	lcl|At2g15420.1	lcl|C37A5.2 CE17529 WBGene0	gi|24640959|	gi|41148429|ref|XP_374258.1|	gi|6703320|ref|Os01g35000|	gi|6324173|ref|NP_014243.1|
gi|31201577|ref|XP_309736.1|	lcl|At2g15535.1	lcl|C37A5.4 CE17530 WBGene0	gi|24640961|	gi|41148431|ref|XP_374259.1|	gi|6703321|ref|Os01g35010|	gi|6324178|ref|NP_014248.1|
gi|31201679|ref|XP_309787.1|	lcl|At2g15550.1	lcl|C37A5.7 CE37488 WBGene0	gi|24640963|	gi|41148437|ref|XP_374261.1|	gi|6703322|ref|Os01g35020|	gi|6324183|ref|NP_014253.1|
gi|31201681|ref|XP_309788.1|	lcl|At2g15600.1	lcl|C37A5.8 CE17532 WBGene0	gi|24640965|	gi|41148449|ref|XP_374262.1|	gi|6703332|ref|Os01g35110|	gi|6324184|ref|NP_014254.1|
gi|31201709|ref|XP_309802.1|	lcl|At2g15670.1	lcl|C37C3.1 CE06926 WBGene0	gi|24640967|	gi|41148460|ref|XP_374267.1|	gi|6703334|ref|Os01g35130|	gi|6324185|ref|NP_014255.1|
gi|31201713|ref|XP_309804.1|	lcl|At2g15720.1	lcl|C37C3.10 CE06935 WBGene	gi|24640971|	gi|41148464|ref|XP_374284.1|	gi|6703336|ref|Os01g35150|	gi|6324193|ref|NP_014263.1|
gi|31201765|ref|XP_309830.1|	lcl|At2g15760.1	lcl|C37C3.11 CE06936 WBGene	gi|24640973|	gi|41148468|ref|XP_374285.1|	gi|6703345|ref|Os01g35240|	gi|6324196|ref|NP_014266.1|
gi|31201815|ref|XP_309855.1|	lcl|At2g15800.1	lcl|C37C3.12 CE32819 WBGene	gi|24640977|	gi|41148472|ref|XP_374286.1|	gi|6703352|ref|Os01g35310|	gi|6324209|ref|NP_014280.1|
gi|31201821|ref|XP_309858.1|	lcl|At2g15815.1	lcl|C37H5.10 CE08633 WBGene	gi|24640984|	gi|41148474|ref|XP_374287.1|	gi|6703353|ref|Os01g35320|	gi|6324226|ref|NP_014296.1|
gi|31201831|ref|XP_309863.1|	lcl|At2g15830.1	lcl|C37H5.11 CE08634 WBGene	gi|24640990|	gi|41148486|ref|XP_372009.1|	gi|6703354|ref|Os01g35330|	gi|6324229|ref|NP_014299.1|
gi|31201835|ref|XP_309865.1|	lcl|At2g15930.1	lcl|C37H5.13b CE27835 WBGer	gi|24640996|	gi|41148490|ref|XP_374243.1|	gi|6703355|ref|Os01g35340|	gi|6324243|ref|NP_014313.1|
gi|31201839|ref|XP_309867.1|	lcl|At2g15960.1	lcl|C37H5.4 CE33278 WBGene0	gi|24640998|	gi|41148494|ref|XP_374244.1|	gi|6703365|ref|Os01g35440|	gi|6324249|ref|NP_014319.1|
gi|31201843|ref|XP_309869.1|	lcl|At2g16015.1	lcl|C38C10.6 CE34740 WBGene	gi|24641004|	gi|41148502|ref|XP_374245.1|	gi|6703367|ref|Os01g35460|	gi|6324251|ref|NP_014321.1|
gi|31201877|ref|XP_309886.1|	lcl|At2g16020.1	lcl|C38C3.3 CE29711 WBGene0	gi|24641006|	gi|41148522|ref|XP_374371.1|	gi|6703370|ref|Os01g35490|	gi|6324253|ref|NP_014323.1|
gi|31201881|ref|XP_309888.1|	lcl|At2g16170.1	lcl|C38C3.6 CE08643 WBGene0	gi|24641008|	gi|41148536|ref|XP_374251.1|	gi|6703373|ref|Os01g35520|	gi|6324255|ref|NP_014325.1|
gi|31201893|ref|XP_309894.1|	lcl|At2g16300.1	lcl|C38C3.8 CE08645 WBGene0	gi|24641010|	gi|41148550|ref|XP_374254.1|	gi|6703374|ref|Os01g35530|	gi|6324259|ref|NP_014329.1|
gi|31201895|ref|XP_309895.1|	lcl|At2g16340.1	lcl|C38C5.1 CE35423 WBGene0	gi|24641021|	gi|41148552|ref|XP_374255.1|	gi|6703376|ref|Os01g35550|	gi|6324271|ref|NP_014340.1|
gi|31201897|ref|XP_309896.1|	lcl|At2g16350.1	lcl|C38C6.3 CE15695 WBGene0	gi|24641042|	gi|41148554|ref|XP_374256.1|	gi|6703378|ref|Os01g35570|	gi|6324274|ref|NP_014344.1|
gi|31201899|ref|XP_309897.1|	lcl|At2g16410.1	lcl|C38C6.5 CE35424 WBGene0	gi|24641113|	gi|41148556|ref|XP_374257.1|	gi|6703379|ref|Os01g35580|	gi|6324278|ref|NP_014348.1|
gi|31201905|ref|XP_309900.1|	lcl|At2g16485.1	lcl|C38C6.6 CE15698 WBGene0	gi|24641115|	gi|41148584|ref|XP_372024.1|	gi|6703380|ref|Os01g35580|	gi|6324282|ref|NP_014352.1|
gi|31201911|ref|XP_309903.1|	lcl|At2g16575.1	lcl|C38D4.1a CE17538 WBGene	gi|24641129|	gi|41148586|ref|XP_374247.1|	gi|6703381|ref|Os01g35590|	gi|6324286|ref|NP_014356.1|
gi|31201921|ref|XP_309908.1|	lcl|At2g16590.1	lcl|C38D4.1b CE00915 WBGene	gi|24641166|	gi|41148588|ref|XP_374248.1|	gi|6703382|ref|Os01g35600|	gi|6324294|ref|NP_014364.1|
gi|31201923|ref|XP_309909.1|	lcl|At2g16820.1	lcl|C38D4.3 CE36378 WBGene0	gi|24641168|	gi|41148598|ref|XP_374250.1|	gi|6703383|ref|Os01g35610|	gi|6324295|ref|NP_014365.1|
gi|31201925|ref|XP_309910.1|	lcl|At2g16830.1	lcl|C38D4.4 CE00917 WBGene0	gi|24641170|	gi|41148635|ref|XP_374263.1|	gi|6703387|ref|Os01g35650|	gi|6324304|ref|NP_014374.1|
gi|31201929|ref|XP_309912.1|	lcl|At2g16835.1	lcl|C38D4.7 CE00920 WBGene0	gi|24641172|	gi|41148637|ref|XP_374264.1|	gi|6703390|ref|Os01g35680|	gi|6324309|ref|NP_014379.1|
gi|31201933|ref|XP_309914.1|	lcl|At2g17300.1	lcl|C38D9.2 CE19728 WBGene0	gi|24641174|	gi|41148639|ref|XP_373248.1|	gi|6703392|ref|Os01g35700|	gi|6324310|ref|NP_014380.1|
gi|31201935|ref|XP_309915.1|	lcl|At2g17305.1	lcl|C38D9.3 CE19729 WBGene0	gi|24641211|	gi|41148644|ref|XP_374276.1|	gi|6703397|ref|Os01g35750|	gi|6324320|ref|NP_014390.1|
gi|31201937|ref|XP_309916.1|	lcl|At2g17540.1	lcl|C38D9.8 CE24836 WBGene0	gi|24641213|	gi|41148652|ref|XP_372055.1|	gi|6703398|ref|Os01g35760|	gi|6324331|ref|NP_014401.1|
gi|31201945|ref|XP_309920.1|	lcl|At2g17710.1	lcl|C38H2.3 CE34741 WBGene0	gi|24641215|	gi|41148661|ref|XP_374277.1|	gi|6703401|ref|Os01g35790|	gi|6324336|ref|NP_014406.1|
gi|31201951|ref|XP_309923.1|	lcl|At2g17785.1	lcl|C39B10.3 CE03079 WBGene	gi|24641235|	gi|41148665|ref|XP_374279.1|	gi|6703403|ref|Os01g35810|	gi|6324341|ref|NP_014411.1|
gi|31201959|ref|XP_309927.1|	lcl|At2g17940.1	lcl|C39B10.4 CE02159 WBGene	gi|24641237|	gi|41148669|ref|XP_374280.1|	gi|6703405|ref|Os01g35830|	gi|6324349|ref|NP_014419.1|
gi|31201961|ref|XP_309928.1|	lcl|At2g17960.1	lcl|C39B10.5 CE02160 WBGene	gi|24641243|	gi|41148671|ref|XP_374281.1|	gi|6703406|ref|Os01g35840|	gi|6324350|ref|NP_014420.1|
gi|31201975|ref|XP_309935.1|	lcl|At2g18070.1	lcl|C39B5.10 CE23576 WBGene	gi|24641245|	gi|41148674|ref|XP_374282.1|	gi|6703409|ref|Os01g35870|	gi|6324351|ref|NP_014421.1|
gi|31201977|ref|XP_309936.1|	lcl|At2g18115.1	lcl|C39B5.2 CE23578 WBGene0	gi|24641251|	gi|41148695|ref|XP_374268.1|	gi|6703410|ref|Os01g35880|	gi|6324368|ref|NP_014438.1|
gi|31202007|ref|XP_309951.1|	lcl|At2g18200.1	lcl|C39D10.1 CE04180 WBGene	gi|24641261|	gi|41148697|ref|XP_374269.1|	gi|6703412|ref|Os01g35900|	gi|6324372|ref|NP_014442.1|
gi|31202021|ref|XP_309958.1|	lcl|At2g18210.1	lcl|C39D10.11 CE37618 WBGer	gi|24641284|	gi|41148711|ref|XP_372045.1|	gi|6703413|ref|Os01g35910|	gi|6324373|ref|NP_014443.1|
gi|31202027|ref|XP_309961.1|	lcl|At2g18270.1	lcl|C39D10.2 CE04181 WBGene	gi|24641287|	gi|41148716|ref|XP_374271.1|	gi|6703414|ref|Os01g35920|	gi|6324377|ref|NP_014447.1|
gi|31202037|ref|XP_309966.1|	lcl|At2g18320.1	lcl|C39D10.5 CE27105 WBGene	gi|24641302|	gi|41148722|ref|XP_374272.1|	gi|6703415|ref|Os01g35930|	gi|6324384|ref|NP_014454.1|
gi|31202069|ref|XP_309982.1|	lcl|At2g18440.1	lcl|C39D10.6 CE35425 WBGene	gi|24641304|	gi|41148732|ref|XP_374274.1|	gi|6703416|ref|Os01g35940|	gi|6324389|ref|NP_014459.1|
gi|31202095|ref|XP_309995.1|	lcl|At2g18610.1	lcl|C39D10.9 CE30502 WBGene	gi|24641338|	gi|41148734|ref|XP_374275.1|	gi|6703417|ref|Os01g35950|	gi|6324390|ref|NP_014460.1|
gi|31202101|ref|XP_309998.1|	lcl|At2g18680.1	lcl|C39E6.1 CE35426 WBGene0	gi|24641340|	gi|41148754|ref|XP_374372.1|	gi|6703418|ref|Os01g35960|	gi|6324418|ref|NP_014487.1|
gi|31202131|ref|XP_310013.1|	lcl|At2g18690.1	lcl|C39E6.2 CE06938 WBGene0	gi|24641367|	gi|41148762|ref|XP_374266.1|	gi|6703420|ref|Os01g35980|	gi|6324424|ref|NP_014493.1|
gi|31202141|ref|XP_310018.1|	lcl|At2g18830.1	lcl|C39E9.11 CE20559 WBGene	gi|24641414|	gi|41148793|ref|XP_374295.1|	gi|6703421|ref|Os01g35990|	gi|6324435|ref|NP_014504.1|
gi|31202149|ref|XP_310022.1|	lcl|At2g18920.1	lcl|C39E9.8a CE27838 WBGene	gi|24641416|	gi|41148802|ref|XP_373300.1|	gi|6703425|ref|Os01g36030|	gi|6324441|ref|NP_014510.1|
gi|31202151|ref|XP_310023.1|	lcl|At2g18930.1	lcl|C39E9.8b CE05391 WBGene	gi|24641420|	gi|41148806|ref|XP_373302.1|	gi|6703428|ref|Os01g36060|	gi|6324455|ref|NP_014524.1|
gi|31202153|ref|XP_310024.1|	lcl|At2g18970.1	lcl|C39F7.1 CE16904 WBGene0	gi|24641445|	gi|41148822|ref|XP_374307.1|	gi|6703435|ref|Os01g36110|	gi|6324462|ref|NP_014531.1|
gi|31202155|ref|XP_310025.1|	lcl|At2g19020.1	lcl|C39H7.4 CE06943 WBGene0	gi|24641493|	gi|41148850|ref|XP_372100.1|	gi|6703436|ref|Os01g36120|	gi|6324463|ref|NP_014532.1|
gi|31202157|ref|XP_310026.1|	lcl|At2g19030.1	lcl|C40A11.10 CE27839 WBGen	gi|24641495|	gi|41148864|ref|XP_373439.1|	gi|6703437|ref|Os01g36130|	gi|6324464|ref|NP_014533.1|
gi|31202159|ref|XP_310027.1|	lcl|At2g19040.1	lcl|C40A11.8 CE36798 WBGene	gi|24641499|	gi|41148898|ref|XP_373328.1|	gi|6703439|ref|Os01g36150|	gi|6324465|ref|NP_014534.1|
gi|31202161|ref|XP_310029.1|	lcl|At2g19140.1	lcl|C40A11.9 CE19372 WBGene	gi|24641503|	gi|41148906|ref|XP_373331.1|	gi|6703444|ref|Os01g36200|	gi|6324468|ref|NP_014537.1|
gi|31202163|ref|XP_310030.1|	lcl|At2g19180.1	lcl|C40C9.3 CE05400 WBGene0	gi|24641505|	gi|41148922|ref|XP_374296.1|	gi|6703446|ref|Os01g36220|	gi|6324481|ref|NP_014550.1|
gi|31202165|ref|XP_310031.1|	lcl|At2g19220.1	lcl|C40C9.4 CE05401 WBGene0	gi|24641509|	gi|41148924|ref|XP_372175.1|	gi|6703447|ref|Os01g36230|	gi|6324489|ref|NP_014558.1|
gi|31202167|ref|XP_310032.1|	lcl|At2g19290.1	lcl|C40H5.2 CE08650 WBGene0	gi|24641519|	gi|41148945|ref|XP_374289.1|	gi|6703451|ref|Os01g36270|	gi|6324490|ref|NP_014559.1|
gi|31202169|ref|XP_310033.1|	lcl|At2g19300.1	lcl|C41A3.2a CE26675 WBGene	gi|24641523|	gi|41148947|ref|XP_374290.1|	gi|6703457|ref|Os01g36330|	gi|6324495|ref|NP_014564.1|
gi|31202171|ref|XP_310034.1|	lcl|At2g19320.1	lcl|C41A3.2b CE27108 WBGene	gi|24641529|	gi|41148963|ref|XP_374291.1|	gi|6703458|ref|Os01g36340|	gi|6324502|ref|NP_014571.1|
gi|31202173|ref|XP_310035.1|	lcl|At2g19370.1	lcl|C41C4.9 CE34745 WBGene0	gi|24641531|	gi|41148965|ref|XP_374292.1|	gi|6703467|ref|Os01g36410|	gi|6324509|ref|NP_014578.1|

gi|31202175|ref|XP_310036.1|	lcl|At2g19420.1	lcl|C41D11.1 CE08658 WBGene	gi|24641537|	gi|41148990|ref|XP_374294.1|	gi|6703473|ref|Os01g36470|	gi|6324519|ref|NP_014588.1|
gi|31202187|ref|XP_310042.1|	lcl|At2g19700.1	lcl|C41D11.3 CE08660 WBGene	gi|24641573|	gi|41149034|ref|XP_374300.1|	gi|6703474|ref|Os01g36480|	gi|6324520|ref|NP_014589.1|
gi|31202189|ref|XP_310043.1|	lcl|At2g19850.1	lcl|C41D11.4 CE32827 WBGene	gi|24641575|	gi|41149036|ref|XP_374301.1|	gi|6703475|ref|Os01g36490|	gi|6324529|ref|NP_014598.1|
gi|31202191|ref|XP_310044.1|	lcl|At2g20070.1	lcl|C41D11.5 CE27841 WBGene	gi|24641624|	gi|41149047|ref|XP_374302.1|	gi|6703477|ref|Os01g36510|	gi|6324536|ref|NP_014606.1|
gi|31202193|ref|XP_310045.1|	lcl|At2g20090.1	lcl|C41D11.6 CE08663 WBGene	gi|24641628|	gi|41149049|ref|XP_374303.1|	gi|6703478|ref|Os01g36520|	gi|6324542|ref|NP_014611.1|
gi|31202195|ref|XP_310046.1|	lcl|At2g20150.1	lcl|C41D7.1 CE08656 WBGene	gi|24641642|	gi|41149053|ref|XP_374304.1|	gi|6703491|ref|Os01g36650|	gi|6324544|ref|NP_014613.1|
gi|31202209|ref|XP_310053.1|	lcl|At2g20250.1	lcl|C41G6.13 CE17539 WBGene	gi|24641644|	gi|41149071|ref|XP_374297.1|	gi|6703495|ref|Os01g36690|	gi|6324547|ref|NP_014616.1|
gi|31202253|ref|XP_310075.1|	lcl|At2g20465.1	lcl|C41G7.6 CE17540 WBGene	gi|24641650|	gi|41149073|ref|XP_374298.1|	gi|6703496|ref|Os01g36700|	gi|6324548|ref|NP_014617.1|
gi|31202259|ref|XP_310078.1|	lcl|At2g20480.1	lcl|C41G7.7 CE08671 WBGene	gi|24641652|	gi|41149075|ref|XP_374299.1|	gi|6703502|ref|Os01g36760|	gi|6324549|ref|NP_014618.1|
gi|31202291|ref|XP_310094.1|	lcl|At2g20500.1	lcl|C41H7.1 CE32605 WBGene	gi|24641675|	gi|41149086|ref|XP_373460.1|	gi|6703507|ref|Os01g36810|	gi|6324556|ref|NP_014625.1|
gi|31202305|ref|XP_310101.1|	lcl|At2g20595.1	lcl|C41H7.2 CE32162 WBGene	gi|24641706|	gi|41149090|ref|XP_373481.1|	gi|6703508|ref|Os01g36820|	gi|6324559|ref|NP_014628.1|
gi|31202311|ref|XP_310104.1|	lcl|At2g20613.1	lcl|C42C1.12 CE16918 WBGene	gi|24641713|	gi|41149094|ref|XP_373480.1|	gi|6703516|ref|Os01g36880|	gi|6324567|ref|NP_014636.1|
gi|31202331|ref|XP_310114.1|	lcl|At2g20616.1	lcl|C42C1.3 CE16909 WBGene	gi|24641715|	gi|41149111|ref|XP_370566.1|	gi|6703526|ref|Os01g36980|	gi|6324582|ref|NP_014651.1|
gi|31202339|ref|XP_310118.1|	lcl|At2g20625.1	lcl|C42C1.4b CE36276 WBGene	gi|24641732|	gi|41149119|ref|XP_373468.1|	gi|6703527|ref|Os01g36990|	gi|6324586|ref|NP_014655.1|
gi|31202351|ref|XP_310124.1|	lcl|At2g20805.1	lcl|C42C1.6 CE16912 WBGene	gi|24641741|	gi|41149127|ref|XP_373469.1|	gi|6703531|ref|Os01g37030|	gi|6324977|ref|NP_015045.1|
gi|31202367|ref|XP_310132.1|	lcl|At2g20870.1	lcl|C42D4.13 CE34746 WBGene	gi|24641774|	gi|41149129|ref|XP_370570.1|	gi|6703532|ref|Os01g37040|	gi|6324978|ref|NP_015046.1|
gi|31202379|ref|XP_310138.1|	lcl|At2g20970.1	lcl|C42D8.1 CE27844 WBGene	gi|24641788|	gi|41149133|ref|XP_373470.1|	gi|6703544|ref|Os01g37150|	gi|6324983|ref|NP_015051.1|
gi|31202413|ref|XP_310155.1|	lcl|At2g21000.1	lcl|C42D8.5b CE37212 WBGene	gi|24641792|	gi|41149145|ref|XP_373472.1|	gi|6703547|ref|Os01g37180|	gi|6324986|ref|NP_015054.1|
gi|31202421|ref|XP_310159.1|	lcl|At2g21185.1	lcl|C43D7.4 CE18548 WBGene	gi|24641794|	gi|41149154|ref|XP_373474.1|	gi|6703548|ref|Os01g37190|	gi|6324988|ref|NP_015056.1|
gi|31202425|ref|XP_310161.1|	lcl|At2g21195.1	lcl|C43D7.5 CE18549 WBGene	gi|24641800|	gi|41149157|ref|XP_373475.1|	gi|6703549|ref|Os01g37200|	gi|6324998|ref|NP_015066.1|
gi|31202437|ref|XP_310167.1|	lcl|At2g21237.1	lcl|C43D7.7 CE18551 WBGene	gi|24641802|	gi|41149173|ref|XP_373477.1|	gi|6703550|ref|Os01g37210|	gi|6325000|ref|NP_015068.1|
gi|31202453|ref|XP_310175.1|	lcl|At2g21490.1	lcl|C43D7.8 CE18552 WBGene	gi|24641805|	gi|41149234|ref|XP_373478.1|	gi|6703551|ref|Os01g37220|	gi|6325001|ref|NP_015069.1|
gi|31202459|ref|XP_310178.1|	lcl|At2g21560.1	lcl|C43E11.11 CE32607 WBGen	gi|24641807|	gi|41149242|ref|XP_373444.1|	gi|6703556|ref|Os01g37270|	gi|6325002|ref|NP_015070.1|
gi|31202473|ref|XP_310185.1|	lcl|At2g21780.1	lcl|C43E11.12 CE32608 WBGen	gi|24641849|	gi|41149244|ref|XP_373445.1|	gi|6703557|ref|Os01g37280|	gi|6325005|ref|NP_015073.1|
gi|31202491|ref|XP_310194.1|	lcl|At2g21920.1	lcl|C43E11.3 CE37885 WBGene	gi|24641900|	gi|41149246|ref|XP_373446.1|	gi|6703558|ref|Os01g37290|	gi|6325013|ref|NP_015081.1|
gi|31202517|ref|XP_310207.1|	lcl|At2g21980.1	lcl|C43E11.6c CE27847 WBGen	gi|24641902|	gi|41149250|ref|XP_374349.1|	gi|6703562|ref|Os01g37330|	gi|6325015|ref|NP_015083.1|
gi|31202531|ref|XP_310214.1|	lcl|At2g22000.1	lcl|C43F9.4 CE35429 WBGene0	gi|24641909|	gi|41149256|ref|XP_373447.1|	gi|6703563|ref|Os01g37340|	gi|6325023|ref|NP_015091.1|
gi|31202539|ref|XP_310218.1|	lcl|At2g22060.1	lcl|C43F9.5 CE19733 WBGene0	gi|24641911|	gi|41149296|ref|XP_372340.1|	gi|6703566|ref|Os01g37370|	gi|6325027|ref|NP_015095.1|
gi|31202543|ref|XP_310220.1|	lcl|At2g22080.1	lcl|C43F9.7 CE19735 WBGene0	gi|24641915|	gi|41149315|ref|XP_373463.1|	gi|6703568|ref|Os01g37390|	gi|6325033|ref|NP_015101.1|
gi|31202549|ref|XP_310223.1|	lcl|At2g22320.1	lcl|C43G2.3 CE36277 WBGene	gi|24641919|	gi|41149319|ref|XP_373464.1|	gi|6703579|ref|Os01g37500|	gi|6325054|ref|NP_015122.1|
gi|31202551|ref|XP_310224.1|	lcl|At2g22340.1	lcl|C43H6.1 CE37619 WBGene	gi|24641921|	gi|41149323|ref|XP_373465.1|	gi|6703581|ref|Os01g37520|	gi|6325055|ref|NP_015123.1|
gi|31202571|ref|XP_310234.1|	lcl|At2g22470.1	lcl|C43H6.4 CE33917 WBGene	gi|24641929|	gi|41149325|ref|XP_373466.1|	gi|6703582|ref|Os01g37530|	gi|6325056|ref|NP_015124.1|
gi|31202577|ref|XP_310237.1|	lcl|At2g22510.1	lcl|C44B11.4 CE29719 WBGene	gi|24641931|	gi|41149349|ref|XP_373448.1|	gi|6703592|ref|Os01g37620|	gi|6325063|ref|NP_015131.1|
gi|31202579|ref|XP_310238.1|	lcl|At2g22520.1	lcl|C44B11.6 CE30628 WBGene	gi|24641933|	gi|41149355|ref|XP_373450.1|	gi|6703594|ref|Os01g37640|	gi|6325064|ref|NP_015132.1|
gi|31202581|ref|XP_310239.1|	lcl|At2g22720.1	lcl|C44B12.1 CE27850 WBGene	gi|24641955|	gi|41149364|ref|XP_370538.1|	gi|6703596|ref|Os01g37660|	gi|6325071|ref|NP_015138.1|
gi|31202589|ref|XP_310243.1|	lcl|At2g22820.1	lcl|C44B12.3 CE31332 WBGene	gi|24641986|	gi|41149376|ref|XP_373451.1|	gi|6703598|ref|Os01g37680|	gi|6325077|ref|NP_015145.1|
gi|31202615|ref|XP_310256.1|	lcl|At2g22940.1	lcl|C44B12.5 CE16921 WBGene	gi|24642053|	gi|41149384|ref|XP_373452.1|	gi|6703600|ref|Os01g37700|	gi|6325091|ref|NP_015159.1|
gi|31202637|ref|XP_310267.1|	lcl|At2g23040.1	lcl|C44B7.3 CE32328 WBGene0	gi|24642063|	gi|41149390|ref|XP_373453.1|	gi|6703601|ref|Os01g37710|	gi|6325094|ref|NP_015162.1|
gi|31202657|ref|XP_310277.1|	lcl|At2g23110.1	lcl|C44B9.2 CE05404 WBGene0	gi|24642068|	gi|41149398|ref|XP_373454.1|	gi|6703603|ref|Os01g37730|	gi|6325095|ref|NP_015163.1|
gi|31202661|ref|XP_310279.1|	lcl|At2g23120.1	lcl|C44B9.3 CE05405 WBGene0	gi|24642103|	gi|41149406|ref|XP_372309.1|	gi|6703604|ref|Os01g37740|	gi|6325096|ref|NP_015164.1|
gi|31202671|ref|XP_310284.1|	lcl|At2g23130.1	lcl|C44B9.5 CE37755 WBGene0	gi|24642111|	gi|41149410|ref|XP_370585.1|	gi|6703608|ref|Os01g37780|	gi|6325098|ref|NP_015166.1|
gi|31202673|ref|XP_310285.1|	lcl|At2g23270.1	lcl|C44C1.1 CE35432 WBGene	gi|24642113|	gi|41149436|ref|XP_373513.1|	gi|6703611|ref|Os01g37810|	gi|6325099|ref|NP_015167.1|
gi|31202675|ref|XP_310286.1|	lcl|At2g23430.1	lcl|C44C1.6 CE30899 WBGene	gi|24642115|	gi|41149438|ref|XP_370658.1|	gi|6703622|ref|Os01g37900|	gi|6325101|ref|NP_015169.1|
gi|31202677|ref|XP_310287.1|	lcl|At2g23440.1	lcl|C44C10.11 CE37713 WBGer	gi|24642117|	gi|41149445|ref|XP_373515.1|	gi|6703624|ref|Os01g37920|	gi|6325108|ref|NP_015176.1|
gi|31202679|ref|XP_310288.1|	lcl|At2g23480.1	lcl|C44C10.6 CE05413 WBGene	gi|24642121|	gi|41149462|ref|XP_373516.1|	gi|6703627|ref|Os01g37950|	gi|6325113|ref|NP_015181.1|
gi|31202681|ref|XP_310289.1|	lcl|At2g23490.1	lcl|C44C10.9 CE18553 WBGene	gi|24642128|	gi|41149464|ref|XP_373517.1|	gi|6703629|ref|Os01g37970|	gi|6325127|ref|NP_015195.1|
gi|31202729|ref|XP_310313.1|	lcl|At2g23670.1	lcl|C44C8.1 CE17541 WBGene	gi|24642134|	gi|41149472|ref|XP_373519.1|	gi|6703632|ref|Os01g38000|	gi|6325133|ref|NP_015201.1|
gi|31202731|ref|XP_310314.1|	lcl|At2g23755.1	lcl|C44C8.2 CE17542 WBGene	gi|24642166|	gi|41149474|ref|XP_373520.1|	gi|6703634|ref|Os01g38020|	gi|6325136|ref|NP_015204.1|
gi|31202733|ref|XP_310315.1|	lcl|At2g23920.1	lcl|C44C8.3 CE17542 WBGene	gi|24642187|	gi|41149517|ref|XP_373500.1|	gi|6703635|ref|Os01g38030|	gi|6325145|ref|NP_015213.1|
gi|31202735|ref|XP_310316.1|	lcl|At2g23985.1	lcl|C44C8.4 CE17543 WBGene	gi|24642197|	gi|41149552|ref|XP_373509.1|	gi|6703640|ref|Os01g38080|	gi|6325149|ref|NP_015217.1|
gi|31202737|ref|XP_310317.1|	lcl|At2g24030.1	lcl|C44E4.2 CE08716 WBGene0	gi|24642200|	gi|41149569|ref|XP_370642.1|	gi|6703642|ref|Os01g38100|	gi|6325158|ref|NP_015226.1|
gi|31202743|ref|XP_310320.1|	lcl|At2g24080.1	lcl|C44F1.1 CE34163 WBGene0	gi|24642202|	gi|41149572|ref|XP_373511.1|	gi|6703645|ref|Os01g38130|	gi|6325172|ref|NP_015240.1|
gi|31202745|ref|XP_310321.1|	lcl|At2g24255.1	lcl|C44H4.7a CE31445 WBGene	gi|24642209|	gi|41149574|ref|XP_373512.1|	gi|6703652|ref|Os01g38200|	gi|6325180|ref|NP_015248.1|
gi|31202751|ref|XP_310324.1|	lcl|At2g24285.1	lcl|C44H4.7b CE31446 WBGene	gi|24642227|	gi|41149595|ref|XP_373483.1|	gi|6703653|ref|Os01g38210|	gi|6325186|ref|NP_015254.1|
gi|31202789|ref|XP_310343.1|	lcl|At2g24310.1	lcl|C44H4.8 CE31447 WBGene	gi|24642233|	gi|41149597|ref|XP_373484.1|	gi|6703654|ref|Os01g38220|	gi|6325189|ref|NP_015257.1|
gi|31202791|ref|XP_310344.1|	lcl|At2g24340.1	lcl|C44H9.5 CE05421 WBGene	gi|24642235|	gi|41149604|ref|XP_373485.1|	gi|6703657|ref|Os01g38250|	gi|6325190|ref|NP_015258.1|
gi|31202793|ref|XP_310345.1|	lcl|At2g24460.1	lcl|C44H9.6 CE05422 WBGene	gi|24642237|	gi|41149650|ref|XP_373486.1|	gi|6703660|ref|Os01g38280|	gi|6325191|ref|NP_015259.1|
gi|31202801|ref|XP_310349.1|	lcl|At2g24762.1	lcl|C44H9.7a CE05423 WBGene	gi|24642312|	gi|41149652|ref|XP_373487.1|	gi|6703661|ref|Os01g38290|	gi|6325201|ref|NP_015269.1|
gi|31202803|ref|XP_310350.1|	lcl|At2g24780.1	lcl|C44H9.7b CE36109 WBGene	gi|24642361|	gi|41149661|ref|XP_373489.1|	gi|6703663|ref|Os01g38310|	gi|6325202|ref|NP_015270.1|
gi|31202817|ref|XP_310357.1|	lcl|At2g24910.1	lcl|C44H9.8 CE30630 WBGene	gi|24642404|	gi|41149684|ref|XP_373539.1|	gi|6703665|ref|Os01g38330|	gi|6325205|ref|NP_015273.1|
gi|31202829|ref|XP_310363.1|	lcl|At2g24915.1	lcl|C45B11.5 CE34755 WBGene	gi|24642406|	gi|41149694|ref|XP_373540.1|	gi|6703667|ref|Os01g38350|	gi|6325208|ref|NP_015276.1|
gi|31202851|ref|XP_310374.1|	lcl|At2g24945.1	lcl|C45B2.1 CE27853 WBGene0	gi|24642408|	gi|41149698|ref|XP_373541.1|	gi|6703668|ref|Os01g38360|	gi|6325210|ref|NP_015278.1|
gi|31202873|ref|XP_310385.1|	lcl|At2g24980.1	lcl|C45B2.2 CE02552 WBGene0	gi|24642414|	gi|41149706|ref|XP_373542.1|	gi|6703671|ref|Os01g38390|	gi|6325216|ref|NP_015284.1|
gi|31202883|ref|XP_310390.1|	lcl|At2g25250.1	lcl|C45B2.3 CE02553 WBGene0	gi|24642496|	gi|41149751|ref|XP_373530.1|	gi|6703677|ref|Os01g38450|	gi|6325218|ref|NP_015286.1|
gi|31202899|ref|XP_310398.1|	lcl|At2g25510.1	lcl|C45B2.8 CE29225 WBGene0	gi|24642498|	gi|41149753|ref|XP_373531.1|	gi|6703679|ref|Os01g38470|	gi|6325222|ref|NP_015291.1|
gi|31202901|ref|XP_310399.1|	lcl|At2g25565.1	lcl|C45E1.4 CE36633 WBGene0	gi|24642501|	gi|41149766|ref|XP_373533.1|	gi|6703684|ref|Os01g38520|	gi|6325230|ref|NP_015298.1|
gi|31202903|ref|XP_310400.1|	lcl|At2g25670.1	lcl|C45E5.4 CE08732 WBGene0	gi|24642503|	gi|41149772|ref|XP_373556.1|	gi|6703688|ref|Os01g38560|	gi|6325233|ref|NP_015301.1|
gi|31202923|ref|XP_310410.1|	lcl|At2g25670.2	lcl|C45G3.4 CE17547 WBGene	gi|24642543|	gi|41149782|ref|XP_373522.1|	gi|6703689|ref|Os01g38570|	gi|6325239|ref|NP_015307.1|
gi|31202961|ref|XP_310429.1|	lcl|At2g25735.1	lcl|C45G3.5 CE37812 WBGene	gi|24642553|	gi|41149800|ref|XP_373524.1|	gi|6703691|ref|Os01g38590|	gi|6325243|ref|NP_015311.1|
gi|31202963|ref|XP_310430.1|	lcl|At2g25990.1	lcl|C45G7.5 CE37490 WBGene	gi|24642567|	gi|41149816|ref|XP_373525.1|	gi|6703692|ref|Os01g38600|	gi|6325262|ref|NP_015330.1|
gi|31202971|ref|XP_310434.1|	lcl|At2g26110.1	lcl|C45G7.7 CE37491 WBGene	gi|24642571|	gi|41149834|ref|XP_373553.1|	gi|6703697|ref|Os01g38640|	gi|6325266|ref|NP_015334.1|
gi|31202975|ref|XP_310436.1|	lcl|At2g26120.1	lcl|C45G9.10a CE01851 WBGer	gi|24642583|	gi|41149842|ref|XP_373554.1|	gi|6703698|ref|Os01g38650|	gi|6325284|ref|NP_015352.1|
gi|31202979|ref|XP_310438.1|	lcl|At2g26520.1	lcl|C45G9.10b CE37887 WBGer	gi|24642590|	gi|41149844|ref|XP_373555.1|	gi|6703704|ref|Os01g38710|	gi|6325287|ref|NP_015355.1|
gi|31202991|ref|XP_310444.1|	lcl|At2g26530.1	lcl|C45G9.11 CE27856 WBGene	gi|24642592|	gi|41149846|ref|XP_372480.1|	gi|6703706|ref|Os01g38730|	gi|6325303|ref|NP_015371.1|
gi|31203001|ref|XP_310449.1|	lcl|At2g26810.1	lcl|C45G9.12 CE01853 WBGene	gi|24642622|	gi|41149848|ref|XP_374352.1|	gi|6703708|ref|Os01g38750|	gi|6325306|ref|NP_015374.1|
gi|31203015|ref|XP_310456.1|	lcl|At2g26880.1	lcl|C45G9.13 CE30632 WBGene	gi|24642626|	gi|41149858|ref|XP_373521.1|	gi|6703710|ref|Os01g38770|	gi|6325314|ref|NP_015382.1|
gi|31203019|ref|XP_310458.1|	lcl|At2g27180.1	lcl|C45G9.4 CE01855 WBGene	gi|24642628|	gi|41149869|ref|XP_373545.1|	gi|6703713|ref|Os01g38800|	gi|6325320|ref|NP_015388.1|
gi|31203021|ref|XP_310459.1|	lcl|At2g27250.1	lcl|C45G9.5 CE01856 WBGene	gi|24642662|	gi|41149873|ref|XP_373546.1|	gi|6703719|ref|Os01g38860|	gi|6325327|ref|NP_015395.1|
gi|31203041|ref|XP_310469.1|	lcl|At2g27310.1	lcl|C45G9.6a CE30633 WBGene	gi|24642693|	gi|41149879|ref|XP_373547.1|	gi|6703720|ref|Os01g38870|	gi|6325328|ref|NP_015396.1|
gi|31203043|ref|XP_310470.1|	lcl|At2g27380.1	lcl|C45G9.6b CE30634 WBGene	gi|24642703|	gi|41149881|ref|XP_373548.1|	gi|6703721|ref|Os01g38880|	gi|6325333|ref|NP_015400.1|
gi|31203047|ref|XP_310472.1|	lcl|At2g27385.1	lcl|C45G9.8 CE01859 WBGene	gi|24642708|	gi|41149903|ref|XP_373571.1|	gi|6703722|ref|Os01g38890|	gi|6325335|ref|NP_015403.1|
gi|31203051|ref|XP_310474.1|	lcl|At2g27390.1	lcl|C45G9.9 CE01860 WBGene	gi|24642730|	gi|41149905|ref|XP_373572.1|	gi|6703726|ref|Os01g38930|	gi|6325340|ref|NP_015408.1|
gi|31203053|ref|XP_310475.1|	lcl|At2g27535.1	lcl|C45H4.13 CE35961 WBGene	gi|24642738|	gi|41149907|ref|XP_373573.1|	gi|6703727|ref|Os01g38940|	gi|6325358|ref|NP_015426.1|
gi|31203073|ref|XP_310485.1|	lcl|At2g27540.1	lcl|C45H4.14a CE08747 WBGer	gi|24642740|	gi|41149909|ref|XP_373574.1|	gi|6703729|ref|Os01g38960|	gi|6325366|ref|NP_015434.1|
gi|31203083|ref|XP_310490.1|	lcl|At2g27620.1	lcl|C45H4.14b CE32829 WBGer	gi|24642742|	gi|41149919|ref|XP_373575.1|	gi|6703736|ref|Os01g39000|	gi|6325373|ref|NP_015441.1|
gi|31203105|ref|XP_310501.1|	lcl|At2g27640.1	lcl|C46A5.5 CE08755 WBGene0	gi|24642744|	gi|41149936|ref|XP_373577.1|	gi|6703737|ref|Os01g39000|	gi|6325380|ref|NP_015449.1|
gi|31203109|ref|XP_310503.1|	lcl|At2g27775.1	lcl|C46A5.6 CE08756 WBGene0	gi|24642746|	gi|41149940|ref|XP_373578.1|	gi|6703740|ref|Os01g39030|	gi|6325391|ref|NP_015459.1|
gi|31203123|ref|XP_310510.1|	lcl|At2g27775.2	lcl|C46A5.8 CE08757 WBGene0	gi|24642752|	gi|41149946|ref|XP_370734.1|	gi|6703743|ref|Os01g39060|	gi|6325392|ref|NP_015460.1|
gi|31203127|ref|XP_310512.1|	lcl|At2g28140.1	lcl|C46C11.2 CE27860 WBGene	gi|24642754|	gi|41149964|ref|XP_373583.1|	gi|6703745|ref|Os01g39080|	gi|6325397|ref|NP_015465.1|
gi|31203129|ref|XP_310513.1|	lcl|At2g28330.1	lcl|C46C11.3 CE04218 WBGene	gi|24642756|	gi|41149970|ref|XP_373585.1|	gi|6703746|ref|Os01g39090|	gi|6325400|ref|NP_015469.1|
gi|31203137|ref|XP_310517.1|	lcl|At2g28410.1	lcl|C46C2.3 CE03082 WBGene	gi|24642758|	gi|41149972|ref|XP_370758.1|	gi|6703754|ref|Os01g39170|	gi|6325406|ref|NP_015474.1|
gi|31203151|ref|XP_310524.1|	lcl|At2g28440.1	lcl|C46C2.5 CE28725 WBGene	gi|24642761|	gi|41149982|ref|XP_373588.1|	gi|6703758|ref|Os01g39210|	gi|6325409|ref|NP_015477.1|
gi|31203175|ref|XP_310536.1|	lcl|At2g28540.1	lcl|C46C2.6 CE34757 WBGene	gi|24642767|	gi|41150000|ref|XP_373624.1|	gi|6703760|ref|Os01g39230|	gi|6325411|ref|NP_015479.1|
gi|31203187|ref|XP_310542.1|	lcl|At2g28570.1	lcl|C46E1.1 CE17556 WBGene0	gi|24642773|	gi|41150025|ref|XP_373625.1|	gi|6703761|ref|Os01g39240|	gi|6325415|ref|NP_015483.1|
gi|31203189|ref|XP_310543.1|	lcl|At2g28725.1	lcl|C46E10.1 CE36110 WBGene	gi|24642775|	gi|41150027|ref|XP_373626.1|	gi|6703767|ref|Os01g39300|	gi|6325416|ref|NP_015484.1|
gi|31203209|ref|XP_310553.1|	lcl|At2g28820.1	lcl|C46E10.2 CE08760 WBGene	gi|24642799|	gi|41150037|ref|XP_370895.1|	gi|6703770|ref|Os01g39320|	gi|6325422|ref|NP_015490.1|
gi|31203219|ref|XP_310558.1|	lcl|At2g28870.1	lcl|C46E10.3 CE08761 WBGene	gi|24642810|	gi|41150043|ref|XP_370898.1|	gi|6703772|ref|Os01g39340|	gi|6325429|ref|NP_015497.1|
gi|31203221|ref|XP_310559.1|	lcl|At2g29125.1	lcl|C46E10.4 CE29724 WBGene	gi|24642812|	gi|41150051|ref|XP_373645.1|	gi|6703774|ref|Os01g39360|	gi|6325432|ref|NP_015500.1|
gi|31203223|ref|XP_310560.1|	lcl|At2g29180.1	lcl|C46E10.5 CE08763 WBGene	gi|24642843|	gi|41150053|ref|XP_373646.1|	gi|6703778|ref|Os01g39390|	gi|6325437|ref|NP_015505.1|
gi|31203225|ref|XP_310561.1|	lcl|At2g29620.1	lcl|C46F11.5a CE31448 WBGen	gi|24642845|	gi|41150055|ref|XP_373647.1|	gi|6703780|ref|Os01g39410|	gi|6325457|ref|NP_015525.1|
gi|31203231|ref|XP_310564.1|	lcl|At2g29790.1	lcl|C46F11.5b CE31449 WBGen	gi|24642850|	gi|41150059|ref|XP_373622.1|	gi|6703788|ref|Os01g39490|	gi|6325460|ref|NP_015528.1|
gi|31203241|ref|XP_310569.1|	lcl|At2g29850.1	lcl|C46F2.1 CE17557 WBGene0	gi|24642882|	gi|41150063|ref|XP_374354.1|	gi|6703789|ref|Os01g39500|	gi|6325461|ref|NP_015529.1|
gi|31203251|ref|XP_310574.1|	lcl|At2g29880.1	lcl|C46G7.1 CE37213 WBGene	gi|24642920|	gi|41150079|ref|XP_370911.1|	gi|6703790|ref|Os01g39510|	gi|6681845|ref|NP_031358.1|
gi|31203265|ref|XP_310581.1|	lcl|At2g29920.1	lcl|C46G7.2 CE08778 WBGene	gi|24642922|	gi|41150116|ref|XP_370829.1|	gi|6703791|ref|Os01g39520|	gi|7839147|ref|NP_058137.1|
gi|31203271|ref|XP_310584.1|	lcl|At2g29995.1	lcl|C46G7.3 CE08779 WBGene	gi|24642926|	gi|41150156|ref|XP_370886.1|	gi|6703798|ref|Os01g39590|	gi|7839166|ref|NP_058154.1|
gi|31203275|ref|XP_310586.1|	lcl|At2g30030.1	lcl|C46G7.4b CE08781 WBGene	gi|24642930|	gi|41150160|ref|XP_370887.1|	gi|6703801|ref|Os01g39620|	gi|7839179|ref|NP_058134.1|
gi|31203277|ref|XP_310587.1|	lcl|At2g30350.1	lcl|C46H11.1 CE08783 WBGene	gi|24642965|	gi|41150174|ref|XP_370894.1|	gi|6703803|ref|Os01g39640|	gi|9755326|ref|NP_061491.1|
gi|31203279|ref|XP_310588.1|	lcl|At2g30430.1	lcl|C46H11.10a CE34272 WBGe	gi|24642967|	gi|41150178|ref|XP_372586.1|	gi|6703807|ref|Os01g39680|	gi|9755330|ref|NP_061492.1|
gi|31203295|ref|XP_310596.1|	lcl|At2g30480.1	lcl|C46H11.10b CE34273 WBGe	gi|24642974|	gi|41150236|ref|XP_373666.1|	gi|6703808|ref|Os01g39680|	gi|9755340|ref|NP_061493.1|
gi|31203309|ref|XP_310603.1|	lcl|At2g30560.1	lcl|C46H3.1 CE04221 WBGene	gi|24642980|	gi|41150240|ref|XP_373667.1|	gi|6703809|ref|Os01g39690|	gi|9755343|ref|NP_061494.1|
gi|31203317|ref|XP_310607.1|	lcl|At2g30680.1	lcl|C47A4.5 CE19740 WBGene0	gi|24642982|	gi|41150252|ref|XP_373668.1|	gi|6703810|ref|Os01g39700|	gi|9755359|ref|NP_014595.2|
gi|31203323|ref|XP_310610.1|	lcl|At2g30760.1	lcl|C47B2.8 CE17569 WBGene0	gi|24642984|	gi|41150279|ref|XP_373685.1|	gi|6703811|ref|Os01g39710|	
gi|31203327|ref|XP_310612.1|	lcl|At2g30925.1	lcl|C47C12.2 CE08795 WBGene	gi|24642986|	gi|41150281|ref|XP_373686.1|	gi|6703815|ref|Os01g39750|	
gi|31203341|ref|XP_310619.1|	lcl|At2g30930.1	lcl|C47D12.5 CE05433 WBGene	gi|24642988|	gi|41150285|ref|XP_373687.1|	gi|6703816|ref|Os01g39760|	
gi|31203345|ref|XP_310621.1|	lcl|At2g30960.1	lcl|C47D2.1 CE06961 WBGene	gi|24642990|	gi|41150287|ref|XP_373683.1|	gi|6703824|ref|Os01g39840|	
gi|31203361|ref|XP_310629.1|	lcl|At2g31035.1	lcl|C47E12.11 CE05455 WBGen	gi|24642992|	gi|41150289|ref|XP_373684.1|	gi|6703827|ref|Os01g39870|	
gi|31203379|ref|XP_310638.1|	lcl|At2g31081.1	lcl|C47E12.12 CE34761 WBGen	gi|24642994|	gi|41150300|ref|XP_373702.1|	gi|6703828|ref|Os01g39880|	
gi|31203381|ref|XP_310639.1|	lcl|At2g31082.1	lcl|C47E12.13 CE34762 WBGen	gi|24642996|	gi|41150302|ref|XP_373703.1|	gi|6703830|ref|Os01g39900|	
gi|31203387|ref|XP_310642.1|	lcl|At2g31083.1	lcl|C47E12.9 CE32612 WBGene	gi|24642998|	gi|41150312|ref|XP_374357.1|	gi|6703831|ref|Os01g39910|	
gi|31203401|ref|XP_310649.1|	lcl|At2g31085.1	lcl|C47E8.1 CE35837 WBGene0	gi|24643000|	gi|41150316|ref|XP_374358.1|	gi|6703832|ref|Os01g39920|	
gi|31203411|ref|XP_310654.1|	lcl|At2g31090.1	lcl|C47E8.6 CE35839 WBGene0	gi|24643024|	gi|41150320|ref|XP_373660.1|	gi|6703834|ref|Os01g39940|	
gi|31203417|ref|XP_310657.1|	lcl|At2g31130.1	lcl|C47E8.9 CE35840 WBGene0	gi|24643026|	gi|41150324|ref|XP_370932.1|	gi|6703835|ref|Os01g39950|	
gi|31203425|ref|XP_310661.1|	lcl|At2g31590.1	lcl|C47F8.7 CE17576 WBGene0	gi|24643029|	gi|41150340|ref|XP_373700.1|	gi|6703838|ref|Os01g39980|	
gi|31203429|ref|XP_310663.1|	lcl|At2g31700.1	lcl|C48B4.10 CE33604 WBGene	gi|24643031|	gi|41150346|ref|XP_372646.1|	gi|6703839|ref|Os01g39990|	
gi|31203431|ref|XP_310664.1|	lcl|At2g31850.1	lcl|C48B4.11 CE00487 WBGene	gi|24643063|	gi|41150350|ref|XP_373681.1|	gi|6703842|ref|Os01g40020|	
gi|31203459|ref|XP_310678.1|	lcl|At2g31930.1	lcl|C48B4.12a CE20576 WBGen	gi|24643074|	gi|41150352|ref|XP_373682.1|	gi|6703843|ref|Os01g40030|	
gi|31203467|ref|XP_310682.1|	lcl|At2g32190.1	lcl|C48B4.12b CE20577 WBGen	gi|24643100|	gi|41150362|ref|XP_373692.1|	gi|6703849|ref|Os01g40080|	
gi|31203483|ref|XP_310690.1|	lcl|At2g32200.1	lcl|C48B4.13 CE34763 WBGene	gi|24643121|	gi|41150382|ref|XP_373695.1|	gi|6703859|ref|Os01g40180|	
gi|31203485|ref|XP_310691.1|	lcl|At2g32210.1	lcl|C48B4.3 CE00490 WBGene0	gi|24643123|	gi|41150384|ref|XP_373696.1|	gi|6703860|ref|Os01g40190|	
gi|31203495|ref|XP_310696.1|	lcl|At2g32235.1	lcl|C48B4.6 CE00482 WBGene0	gi|24643126|	gi|41150386|ref|XP_373697.1|	gi|6703863|ref|Os01g40210|	
gi|31203503|ref|XP_310700.1|	lcl|At2g32690.1	lcl|C48B4.7 CE00483 WBGene0	gi|24643132|	gi|41150390|ref|XP_373672.1|	gi|6703864|ref|Os01g40220|	
gi|31203517|ref|XP_310707.1|	lcl|At2g32890.1	lcl|C48B4.8 CE00484 WBGene0	gi|24643145|	gi|41150412|ref|XP_373673.1|	gi|6703869|ref|Os01g40270|	
gi|31203531|ref|XP_310714.1|	lcl|At2g33250.1	lcl|C48B4.9 CE00485 WBGene0	gi|24643147|	gi|41150414|ref|XP_373674.1|	gi|6703871|ref|Os01g40290|	
gi|31203535|ref|XP_310716.1|	lcl|At2g33390.1	lcl|C48B6.10 CE27868 WBGene	gi|24643154|	gi|41150426|ref|XP_373676.1|	gi|6703879|ref|Os01g40350|	

gi|31203541|ref|XP_310719.1| lcl|At2g33400.1 lcl|C48B6.3 CE08805 WBGene0 gi|24643161| gi|41150436|ref|XP_370974.1| gi|6703890|ref|Os01g40460|
gi|31203547|ref|XP_310722.1| lcl|At2g33520.1 lcl|C48B6.8 CE32166 WBGene0 gi|24643168| gi|41150442|ref|XP_373677.1| gi|6703895|ref|Os01g40510|
gi|31203551|ref|XP_310724.1| lcl|At2g33690.1 lcl|C48B6.9 CE08811 WBGene0 gi|24643176| gi|41150470|ref|XP_372626.1| gi|6703896|ref|Os01g40520|
gi|31203553|ref|XP_310725.1| lcl|At2g33720.1 lcl|C48D1.5 CE34764 WBGene0 gi|24643182| gi|41150488|ref|XP_373654.1| gi|6703912|ref|Os01g40650|
gi|31203557|ref|XP_310727.1| lcl|At2g33850.1 lcl|C48D5.3 CE34765 WBGene0 gi|24643184| gi|41150492|ref|XP_373655.1| gi|6703921|ref|Os01g40730|
gi|31203561|ref|XP_310729.1| lcl|At2g34010.1 lcl|C48E7.1 CE08816 WBGene0 gi|24643198| gi|41150494|ref|XP_370924.1| gi|6703922|ref|Os01g40740|
gi|31203563|ref|XP_310730.1| lcl|At2g34100.1 lcl|C48E7.7 CE08822 WBGene0 gi|24643203| gi|41150500|ref|XP_373657.1| gi|6703923|ref|Os01g40750|
gi|31203567|ref|XP_310732.1| lcl|At2g34110.1 lcl|C48G7.1 CE08825 WBGene0 gi|24643219| gi|41150509|ref|XP_373659.1| gi|6703924|ref|Os01g40760|
gi|31203573|ref|XP_310735.1| lcl|At2g34120.1 lcl|C49A1.1 CE08827 WBGene0 gi|24643230| gi|41150529|ref|XP_373679.1| gi|6703927|ref|Os01g40790|
gi|31203579|ref|XP_310738.1| lcl|At2g34270.1 lcl|C49A1.10 CE28542 WBGene gi|24643248| gi|41150541|ref|XP_373744.1| gi|6703931|ref|Os01g40830|
gi|31203581|ref|XP_310739.1| lcl|At2g34310.1 lcl|C49A9.10 CE33568 WBGene gi|24643278| gi|41150543|ref|XP_373745.1| gi|6703939|ref|Os01g40910|
gi|31203583|ref|XP_310740.1| lcl|At2g34310.2 lcl|C49A9.9b CE33570 WBGene gi|24643301| gi|41150545|ref|XP_373746.1| gi|6703940|ref|Os01g40920|
gi|31203591|ref|XP_310744.1| lcl|At2g34310.3 lcl|C49C3.15 CE37493 WBGene gi|24643345| gi|41150559|ref|XP_373749.1| gi|6703941|ref|Os01g40930|
gi|31203611|ref|XP_310754.1| lcl|At2g34315.1 lcl|C49C3.3 CE19742 WBGene0 gi|24643354| gi|41150564|ref|XP_373750.1| gi|6703942|ref|Os01g40940|
gi|31203619|ref|XP_310758.1| lcl|At2g34330.1 lcl|C49C3.6 CE19745 WBGene0 gi|24643417| gi|41150566|ref|XP_373751.1| gi|6703950|ref|Os01g41020|
gi|31203625|ref|XP_310761.1| lcl|At2g34530.1 lcl|C49C3.7 CE19746 WBGene0 gi|24643421| gi|41150584|ref|XP_373760.1| gi|6703958|ref|Os01g41100|
gi|31203637|ref|XP_310767.1| lcl|At2g34550.1 lcl|C49C3.8 CE19747 WBGene0 gi|24643423| gi|41150586|ref|XP_373761.1| gi|6703962|ref|Os01g41140|
gi|31203639|ref|XP_310768.1| lcl|At2g34580.1 lcl|C49C3.9 CE19748 WBGene0 gi|24643437| gi|41150591|ref|XP_373707.1| gi|6703965|ref|Os01g41170|
gi|31203641|ref|XP_310769.1| lcl|At2g34600.1 lcl|C49C8.2 CE33571 WBGene0 gi|24643439| gi|41150593|ref|XP_373708.1| gi|6703966|ref|Os01g41180|
gi|31203649|ref|XP_310773.1| lcl|At2g34610.1 lcl|C49C8.3 CE08838 WBGene0 gi|24643441| gi|41150610|ref|XP_371008.1| gi|6703971|ref|Os01g41230|
gi|31203667|ref|XP_310782.1| lcl|At2g34800.1 lcl|C49C8.6 CE35963 WBGene0 gi|24643443| gi|41150637|ref|XP_373713.1| gi|6703978|ref|Os01g41300|
gi|31203677|ref|XP_310787.1| lcl|At2g34870.1 lcl|C49D10.11 CE29230 WBGer gi|24643476| gi|41150646|ref|XP_371018.1| gi|6703980|ref|Os01g41320|
gi|31203683|ref|XP_310790.1| lcl|At2g34910.1 lcl|C49D10.4 CE08845 WBGene gi|24643478| gi|41150648|ref|XP_085606.3| gi|6703981|ref|Os01g41330|
gi|31203695|ref|XP_310796.1| lcl|At2g35070.1 lcl|C49F5.3 CE08854 WBGene0 gi|24643481| gi|41150650|ref|XP_373714.1| gi|6703983|ref|Os01g41350|
gi|31203723|ref|XP_310810.1| lcl|At2g35075.1 lcl|C49F5.6 CE08857 WBGene0 gi|24643497| gi|41150654|ref|XP_373715.1| gi|6703985|ref|Os01g41370|
gi|31203737|ref|XP_310817.1| lcl|At2g35080.1 lcl|C49F5.7 CE31455 WBGene0 gi|24643499| gi|41150676|ref|XP_373717.1| gi|6703986|ref|Os01g41380|
gi|31203751|ref|XP_310824.1| lcl|At2g35090.1 lcl|C49F5.8 CE30909 WBGene0 gi|24643534| gi|41150678|ref|XP_373718.1| gi|6703987|ref|Os01g41390|
gi|31203757|ref|XP_310827.1| lcl|At2g35215.1 lcl|C49F8.1 CE05456 WBGene0 gi|24643540| gi|41150706|ref|XP_373728.1| gi|6703989|ref|Os01g41410|
gi|31203779|ref|XP_310838.1| lcl|At2g35260.1 lcl|C49G7.5 CE08863 WBGene0 gi|24643544| gi|41150716|ref|XP_373734.1| gi|6703992|ref|Os01g41440|
gi|31203789|ref|XP_310843.1| lcl|At2g35280.1 lcl|C49G7.6 CE36802 WBGene0 gi|24643550| gi|41150721|ref|XP_373722.1| gi|6703995|ref|Os01g41470|
gi|31203801|ref|XP_310849.1| lcl|At2g35470.1 lcl|C49G9.1 CE18555 WBGene0 gi|24643552| gi|41150731|ref|XP_373723.1| gi|6704002|ref|Os01g41540|
gi|31203805|ref|XP_310851.1| lcl|At2g35733.1 lcl|C49H3.12 CE04238 WBGene gi|24643557| gi|41150733|ref|XP_373724.1| gi|6704004|ref|Os01g41560|
gi|31203821|ref|XP_310859.1| lcl|At2g35750.1 lcl|C49H3.3 CE04229 WBGene0 gi|24643564| gi|41150759|ref|XP_371081.1| gi|6704016|ref|Os01g41680|
gi|31203829|ref|XP_310863.1| lcl|At2g35850.1 lcl|C50A2.3 CE17581 WBGene0 gi|24643576| gi|41150763|ref|XP_373753.1| gi|6704022|ref|Os01g41740|
gi|31203857|ref|XP_310877.1| lcl|At2g35870.1 lcl|C50B6.1 CE08870 WBGene0 gi|24643580| gi|41150767|ref|XP_373754.1| gi|6704027|ref|Os01g41790|
gi|31203865|ref|XP_310881.1| lcl|At2g35950.1 lcl|C50B6.3 CE08872 WBGene0 gi|24643584| gi|41150769|ref|XP_373755.1| gi|6704033|ref|Os01g41850|
gi|31203867|ref|XP_310882.1| lcl|At2g36040.1 lcl|C50B8.4 CE23606 WBGene0 gi|24643586| gi|41150771|ref|XP_371083.1| gi|6704034|ref|Os01g41860|
gi|31203883|ref|XP_310890.1| lcl|At2g36120.1 lcl|C50B8.5 CE36920 WBGene0 gi|24643588| gi|41150775|ref|XP_373757.1| gi|6704040|ref|Os01g41920|
gi|31203887|ref|XP_310892.1| lcl|At2g36220.1 lcl|C50B8.6 CE34767 WBGene0 gi|24643591| gi|41150777|ref|XP_294867.4| gi|6704043|ref|Os01g41950|
gi|31203889|ref|XP_310893.1| lcl|At2g36295.1 lcl|C50C10.1 CE05459 WBGene gi|24643593| gi|41150781|ref|XP_373758.1| gi|6704045|ref|Os01g41970|
gi|31203893|ref|XP_310895.1| lcl|At2g36420.1 lcl|C50C3.12 CE30635 WBGene gi|24643603| gi|41150785|ref|XP_373705.1| gi|6704046|ref|Os01g41980|
gi|31203905|ref|XP_310901.1| lcl|At2g36440.1 lcl|C50D2.1 CE27874 WBGene0 gi|24643630| gi|41150787|ref|XP_373719.1| gi|6704059|ref|Os01g42100|
gi|31203915|ref|XP_310906.1| lcl|At2g36920.1 lcl|C50D2.6 CE34768 WBGene0 gi|24643634| gi|41150795|ref|XP_371028.1| gi|6704062|ref|Os01g42130|
gi|31203927|ref|XP_310912.1| lcl|At2g36940.1 lcl|C50E10.1 CE28141 WBGene gi|24643650| gi|41150805|ref|XP_373721.1| gi|6704063|ref|Os01g42140|
gi|31203953|ref|XP_310925.1| lcl|At2g37070.1 lcl|C50E10.4 CE32334 WBGene gi|24643652| gi|41150819|ref|XP_373726.1| gi|6704064|ref|Os01g42150|
gi|31203955|ref|XP_310926.1| lcl|At2g37100.1 lcl|C50E3.12 CE08905 WBGene gi|24643666| gi|41150829|ref|XP_373763.1| gi|6704065|ref|Os01g42160|
gi|31203961|ref|XP_310929.1| lcl|At2g37120.1 lcl|C50E3.13 CE08906 WBGene gi|24643668| gi|41150831|ref|XP_371088.1| gi|6704067|ref|Os01g42180|
gi|31203969|ref|XP_310933.1| lcl|At2g37300.1 lcl|C50E3.2 CE08895 WBGene0 gi|24643670| gi|41150839|ref|XP_373765.1| gi|6704070|ref|Os01g42210|
gi|31203989|ref|XP_310943.1| lcl|At2g37530.1 lcl|C50E3.5 CE30738 WBGene0 gi|24643729| gi|41150847|ref|XP_373766.1| gi|6704071|ref|Os01g42220|
gi|31204007|ref|XP_310952.1| lcl|At2g37610.1 lcl|C50E3.6 CE37620 WBGene0 gi|24643731| gi|41150851|ref|XP_373767.1| gi|6704080|ref|Os01g42310|
gi|31204023|ref|XP_310960.1| lcl|At2g37750.1 lcl|C50E3.8 CE08901 WBGene0 gi|24643800| gi|41150859|ref|XP_373769.1| gi|6704085|ref|Os01g42360|
gi|31204027|ref|XP_310962.1| lcl|At2g37830.1 lcl|C50E3.9 CE08902 WBGene0 gi|24643820| gi|41150867|ref|XP_373770.1| gi|6704088|ref|Os01g42390|
gi|31204031|ref|XP_310964.1| lcl|At2g37910.1 lcl|C50F2.10 CE27876 WBGene gi|24643824| gi|41150869|ref|XP_373771.1| gi|6704091|ref|Os01g42420|
gi|31204079|ref|XP_310988.1| lcl|At2g38140.1 lcl|C50F2.2 CE27877 WBGene0 gi|24643842| gi|41150875|ref|XP_371106.1| gi|6704093|ref|Os01g42440|
gi|31204081|ref|XP_310989.1| lcl|At2g38160.1 lcl|C50F2.4 CE08910 WBGene0 gi|24643844| gi|41150877|ref|XP_373772.1| gi|6704094|ref|Os01g42450|
gi|31204083|ref|XP_310990.1| lcl|At2g38185.1 lcl|C50F2.7 CE08913 WBGene0 gi|24643984| gi|41150881|ref|XP_373773.1| gi|6704103|ref|Os01g42510|
gi|31204095|ref|XP_310996.1| lcl|At2g38200.1 lcl|C50F2.9 CE27879 WBGene0 gi|24644003| gi|41150894|ref|XP_373774.1| gi|6704107|ref|Os01g42550|
gi|31204101|ref|XP_310999.1| lcl|At2g38220.1 lcl|C50F4.4 CE33985 WBGene0 gi|24644024| gi|41150903|ref|XP_374359.1| gi|6704108|ref|Os01g42560|
gi|31204103|ref|XP_311000.1| lcl|At2g38350.1 lcl|C50F4.6 CE05471 WBGene0 gi|24644026| gi|41150915|ref|XP_373783.1| gi|6704109|ref|Os01g42570|
gi|31204109|ref|XP_311003.1| lcl|At2g38365.1 lcl|C50F4.8 CE05472 WBGene0 gi|24644028| gi|41150917|ref|XP_373784.1| gi|6704110|ref|Os01g42580|
gi|31204125|ref|XP_311011.1| lcl|At2g38570.1 lcl|C50F4.9 CE05473 WBGene0 gi|24644030| gi|41150925|ref|XP_372700.1| gi|6704112|ref|Os01g42600|
gi|31204127|ref|XP_311012.1| lcl|At2g38690.1 lcl|C50F7.2 CE04240 WBGene0 gi|24644032| gi|41150927|ref|XP_374361.1| gi|6704120|ref|Os01g42660|
gi|31204129|ref|XP_311013.1| lcl|At2g38790.1 lcl|C50F7.3 CE04241 WBGene0 gi|24644038| gi|41150933|ref|XP_373782.1| gi|6704121|ref|Os01g42670|
gi|31204133|ref|XP_311015.1| lcl|At2g38823.1 lcl|C50F7.5 CE04243 WBGene0 gi|24644040| gi|41150941|ref|XP_373778.1| gi|6704126|ref|Os01g42720|
gi|31204167|ref|XP_311032.1| lcl|At2g38826.1 lcl|C50F7.9 CE04247 WBGene0 gi|24644068| gi|41150945|ref|XP_373779.1| gi|6704129|ref|Os01g42750|
gi|31204177|ref|XP_311037.1| lcl|At2g38890.1 lcl|C50H11.8 CE35258 WBGene gi|24644077| gi|41150947|ref|XP_373780.1| gi|6704139|ref|Os01g42840|
gi|31204181|ref|XP_311039.1| lcl|At2g38930.1 lcl|C50H2.10 CE30127 WBGene gi|24644079| gi|41150961|ref|XP_373800.1| gi|6704145|ref|Os01g42890|
gi|31204183|ref|XP_311040.1| lcl|At2g38980.1 lcl|C50H2.12 CE34770 WBGene gi|24644081| gi|41150963|ref|XP_373801.1| gi|6704147|ref|Os01g42910|
gi|31204185|ref|XP_311041.1| lcl|At2g39160.1 lcl|C50H2.13 CE34771 WBGene gi|24644089| gi|41150965|ref|XP_373802.1| gi|6704149|ref|Os01g42930|
gi|31204191|ref|XP_311044.1| lcl|At2g39500.1 lcl|C50H2.2a CE36586 WBGene gi|24644091| gi|41150982|ref|XP_373803.1| gi|6704150|ref|Os01g42940|
gi|31204201|ref|XP_311049.1| lcl|At2g39520.1 lcl|C50H2.2b CE05480 WBGene gi|24644095| gi|41151016|ref|XP_371121.1| gi|6704174|ref|Os01g43110|
gi|31204207|ref|XP_311052.1| lcl|At2g39560.1 lcl|C50H2.4 CE05482 WBGene0 gi|24644105| gi|41151018|ref|XP_373785.1| gi|6704184|ref|Os01g43190|
gi|31204215|ref|XP_311056.1| lcl|At2g39680.1 lcl|C50H2.5 CE05483 WBGene0 gi|24644119| gi|41151024|ref|XP_371122.1| gi|6704189|ref|Os01g43240|
gi|31204229|ref|XP_311063.1| lcl|At2g39855.1 lcl|C51E3.10 CE34772 WBGene gi|24644129| gi|41151031|ref|XP_373787.1| gi|6704191|ref|Os01g43260|
gi|31204237|ref|XP_311067.1| lcl|At2g39870.1 lcl|C52B11.4 CE04260 WBGene gi|24644189| gi|41151039|ref|XP_373788.1| gi|6704194|ref|Os01g43290|
gi|31204253|ref|XP_311075.1| lcl|At2g39920.1 lcl|C52B9.10 CE30260 WBGene gi|24644209| gi|41151045|ref|XP_373789.1| gi|6704207|ref|Os01g43400|
gi|31204279|ref|XP_311088.1| lcl|At2g40020.1 lcl|C52B9.5 CE33366 WBGene0 gi|24644222| gi|41151048|ref|XP_373790.1| gi|6704210|ref|Os01g43430|
gi|31204291|ref|XP_311094.1| lcl|At2g40475.1 lcl|C52D10.1 CE17586 WBGene gi|24644274| gi|41151073|ref|XP_373793.1| gi|6704212|ref|Os01g43450|
gi|31204301|ref|XP_311099.1| lcl|At2g40530.1 lcl|C52D10.11 CE17594 WBGer gi|24644330| gi|41151109|ref|XP_373795.1| gi|6704214|ref|Os01g43470|
gi|31204319|ref|XP_311108.1| lcl|At2g40765.1 lcl|C52D10.2 CE17587 WBGene gi|24644347| gi|41151116|ref|XP_373796.1| gi|6704218|ref|Os01g43500|
gi|31204327|ref|XP_311112.1| lcl|At2g40955.1 lcl|C52D10.3 CE36281 WBGene gi|24644374| gi|41151120|ref|XP_371147.1| gi|6704219|ref|Os01g43510|
gi|31204333|ref|XP_311115.1| lcl|At2g41230.1 lcl|C52E2.2 CE20597 WBGene0 gi|24644426| gi|41151122|ref|XP_373797.1| gi|6704224|ref|Os01g43560|
gi|31204335|ref|XP_311116.1| lcl|At2g41260.1 lcl|C52E2.8 CE25832 WBGene0 gi|24644432| gi|41151197|ref|XP_373920.1| gi|6704225|ref|Os01g43570|
gi|31204341|ref|XP_311119.1| lcl|At2g41280.1 lcl|C52G5.3 CE34773 WBGene0 gi|24644452| gi|41151199|ref|XP_373921.1| gi|6704229|ref|Os01g43600|
gi|31204351|ref|XP_311124.1| lcl|At2g41390.1 lcl|C53A3.1 CE16941 WBGene0 gi|24644474| gi|41151201|ref|XP_373922.1| gi|6704231|ref|Os01g43620|
gi|31204355|ref|XP_311126.1| lcl|At2g41400.1 lcl|C53A5.1 CE08950 WBGene0 gi|24644515| gi|41151207|ref|XP_371405.1| gi|6704235|ref|Os01g43660|
gi|31204357|ref|XP_311127.1| lcl|At2g41420.1 lcl|C53B4.2 CE32836 WBGene0 gi|24644552| gi|41151215|ref|XP_373914.1| gi|6704238|ref|Os01g43690|
gi|31204361|ref|XP_311129.1| lcl|At2g41440.1 lcl|C53B7.3 CE06973 WBGene0 gi|24644555| gi|41151224|ref|XP_373915.1| gi|6704246|ref|Os01g43770|
gi|31204367|ref|XP_311132.1| lcl|At2g41550.1 lcl|C53B7.6 CE06976 WBGene0 gi|24644557| gi|41151233|ref|XP_373916.1| gi|6704248|ref|Os01g43790|
gi|31204375|ref|XP_311136.1| lcl|At2g41650.1 lcl|C53C11.2 CE04265 WBGene gi|24644564| gi|41151237|ref|XP_373917.1| gi|6704255|ref|Os01g43860|
gi|31204417|ref|XP_311157.1| lcl|At2g41730.1 lcl|C53C7.3 CE34167 WBGene0 gi|24644566| gi|41151239|ref|XP_371403.1| gi|6704257|ref|Os01g43880|
gi|31204449|ref|XP_311173.1| lcl|At2g41780.1 lcl|C53C9.1 CE36282 WBGene0 gi|24644634| gi|41151270|ref|XP_373905.1| gi|6704259|ref|Os01g43900|
gi|31204453|ref|XP_311175.1| lcl|At2g42050.1 lcl|C53C9.2 CE27882 WBGene0 gi|24644638| gi|41151272|ref|XP_373906.1| gi|6704261|ref|Os01g43920|
gi|31204455|ref|XP_311176.1| lcl|At2g42110.1 lcl|C53D5.1a CE27883 WBGene gi|24644640| gi|41151274|ref|XP_373907.1| gi|6704264|ref|Os01g43950|
gi|31204481|ref|XP_311189.1| lcl|At2g42260.1 lcl|C53D5.1b CE27884 WBGene gi|24644646| gi|41151280|ref|XP_373909.1| gi|6704265|ref|Os01g43960|
gi|31204483|ref|XP_311190.1| lcl|At2g42340.1 lcl|C53D5.1c CE23609 WBGene gi|24644681| gi|41151282|ref|XP_373910.1| gi|6704269|ref|Os01g44000|
gi|31204485|ref|XP_311191.1| lcl|At2g42530.1 lcl|C53D5.3 CE20607 WBGene0 gi|24644700| gi|41151292|ref|XP_374365.1| gi|6704270|ref|Os01g44010|
gi|31204507|ref|XP_311202.1| lcl|At2g42540.1 lcl|C53D6.10 CE36990 WBGene gi|24644702| gi|41151309|ref|XP_373925.1| gi|6704272|ref|Os01g44030|
gi|31204511|ref|XP_311204.1| lcl|At2g42540.2 lcl|C53D6.5 CE05490 WBGene0 gi|24644710| gi|41151311|ref|XP_373926.1| gi|6704275|ref|Os01g44060|
gi|31204513|ref|XP_311205.1| lcl|At2g42560.1 lcl|C53D6.8 CE34774 WBGene0 gi|24644757| gi|41151315|ref|XP_373928.1| gi|6704280|ref|Os01g44100|
gi|31204517|ref|XP_311207.1| lcl|At2g42860.1 lcl|C54A12.3 CE01867 WBGene gi|24644776| gi|41151321|ref|XP_371411.1| gi|6704282|ref|Os01g44120|
gi|31204543|ref|XP_311220.1| lcl|At2g42870.1 lcl|C54C6.4 CE05495 WBGene0 gi|24644803| gi|41151327|ref|XP_373931.1| gi|6704284|ref|Os01g44140|
gi|31204545|ref|XP_311221.1| lcl|At2g42885.1 lcl|C54C6.5 CE05496 WBGene0 gi|24644805| gi|41151331|ref|XP_373933.1| gi|6704286|ref|Os01g44160|
gi|31204547|ref|XP_311222.1| lcl|At2g42955.1 lcl|C54C8.2 CE08965 WBGene0 gi|24644809| gi|41151347|ref|XP_373938.1| gi|6704288|ref|Os01g44180|
gi|31204551|ref|XP_311224.1| lcl|At2g43060.1 lcl|C54C8.6 CE08969 WBGene0 gi|24644813| gi|41151353|ref|XP_373941.1| gi|6704289|ref|Os01g44190|
gi|31204565|ref|XP_311231.1| lcl|At2g43150.1 lcl|C54C8.9 CE08971 WBGene0 gi|24644815| gi|41151369|ref|XP_373943.1| gi|6704299|ref|Os01g44270|
gi|31204587|ref|XP_311242.1| lcl|At2g43270.1 lcl|C54D1.7 CE30917 WBGene0 gi|24644824| gi|41151375|ref|XP_373944.1| gi|6704300|ref|Os01g44280|
gi|31204589|ref|XP_311243.1| lcl|At2g43310.1 lcl|C54D10.4 CE05500 WBGene gi|24644897| gi|41151377|ref|XP_371423.1| gi|6704301|ref|Os01g44290|
gi|31204591|ref|XP_311244.1| lcl|At2g43380.1 lcl|C54D10.7 CE05503 WBGene gi|24644905| gi|41151430|ref|XP_373947.1| gi|6704302|ref|Os01g44300|
gi|31204595|ref|XP_311246.1| lcl|At2g43390.1 lcl|C54D10.8 CE35439 WBGene gi|24644944| gi|41151432|ref|XP_371437.1| gi|6704311|ref|Os01g44380|
gi|31204605|ref|XP_311251.1| lcl|At2g43450.1 lcl|C54D10.9 CE05505 WBGene gi|24644946| gi|41151440|ref|XP_373964.1| gi|6704318|ref|Os01g44940|
gi|31204609|ref|XP_311253.1| lcl|At2g43990.1 lcl|C54D2.2 CE02559 WBGene0 gi|24644954| gi|41151442|ref|XP_373965.1| gi|6704325|ref|Os01g45000|
gi|31204651|ref|XP_311274.1| lcl|At2g44000.1 lcl|C54E10.6 CE15734 WBGene gi|24645011| gi|41151444|ref|XP_373966.1| gi|6704326|ref|Os01g45010|
gi|31204655|ref|XP_311276.1| lcl|At2g44010.1 lcl|C54E4.1 CE16943 WBGene0 gi|24645013| gi|41151451|ref|XP_373967.1| gi|6704333|ref|Os01g45080|
gi|31204657|ref|XP_311277.1| lcl|At2g44080.1 lcl|C54E4.4 CE29238 WBGene0 gi|24645015| gi|41151453|ref|XP_373968.1| gi|6704337|ref|Os01g45120|
gi|31204661|ref|XP_311279.1| lcl|At2g44195.1 lcl|C54F6.11 CE08983 WBGene gi|24645037| gi|41151455|ref|XP_373969.1| gi|6704338|ref|Os01g45130|
gi|31204679|ref|XP_311288.1| lcl|At2g44735.1 lcl|C54F6.12 CE35259 WBGene gi|24645065| gi|41151459|ref|XP_373970.1| gi|6704340|ref|Os01g45150|
gi|31204685|ref|XP_311291.1| lcl|At2g45135.1 lcl|C54F6.5 CE08977 WBGene0 gi|24645067| gi|41151477|ref|XP_373942.1| gi|6704341|ref|Os01g45160|
gi|31204691|ref|XP_311294.1| lcl|At2g45403.1 lcl|C54G10.1 CE05516 WBGene gi|24645091| gi|41151507|ref|XP_373345.1| gi|6704342|ref|Os01g45170|
gi|31204697|ref|XP_311297.1| lcl|At2g45450.1 lcl|C54G4.2 CE05508 WBGene0 gi|24645141| gi|41151511|ref|XP_374326.1| gi|6704350|ref|Os01g45250|
gi|31204707|ref|XP_311302.1| lcl|At2g45780.1 lcl|C54G4.3 CE05509 WBGene0 gi|24645175| gi|41151535|ref|XP_374328.1| gi|6704351|ref|Os01g45260|
gi|31204719|ref|XP_311308.1| lcl|At2g45860.1 lcl|C54G4.5 CE23613 WBGene0 gi|24645177| gi|41151545|ref|XP_374331.1| gi|6704352|ref|Os01g45270|
gi|31204745|ref|XP_311321.1| lcl|At2g45900.1 lcl|C54G4.9 CE05515 WBGene0 gi|24645184| gi|41151598|ref|XP_374323.1| gi|6704356|ref|Os01g45300|
gi|31204779|ref|XP_311338.1| lcl|At2g45930.1 lcl|C54G6.2 CE36636 WBGene0 gi|24645203| gi|41151633|ref|XP_374337.1| gi|6704357|ref|Os01g45310|
gi|31204795|ref|XP_311346.1| lcl|At2g46360.1 lcl|C54G6.3 CE36637 WBGene0 gi|24645270| gi|41151640|ref|XP_374338.1| gi|6704360|ref|Os01g45340|
gi|31204809|ref|XP_311353.1| lcl|At2g46375.1 lcl|C54G7.1 CE04269 WBGene0 gi|24645272| gi|41151644|ref|XP_374339.1| gi|6704367|ref|Os01g45410|
gi|31204823|ref|XP_311360.1| lcl|At2g46390.1 lcl|C54H2.4 CE35441 WBGene0 gi|24645274| gi|41151646|ref|XP_374340.1| gi|6704370|ref|Os01g45440|
gi|31204833|ref|XP_311365.1| lcl|At2g46490.1 lcl|C55A1.11 CE33044 WBGene gi|24645276| gi|41151700|ref|XP_373369.1| gi|6704373|ref|Os01g45470|
gi|31204841|ref|XP_311369.1| lcl|At2g46535.1 lcl|C55A1.4 CE08990 WBGene0 gi|24645285| gi|41151706|ref|XP_373382.1| gi|6704374|ref|Os01g45480|
gi|31204845|ref|XP_311371.1| lcl|At2g46630.1 lcl|C55A1.6 CE08992 WBGene0 gi|24645356| gi|41151708|ref|XP_372274.1| gi|6704376|ref|Os01g45500|
gi|31204861|ref|XP_311379.1| lcl|At2g46640.1 lcl|C55A1.7 CE08990 WBGene0 gi|24645358| gi|41151710|ref|XP_374341.1| gi|6704380|ref|Os01g45540|
gi|31204881|ref|XP_311389.1| lcl|At2g46735.1 lcl|C55A1.8 CE34533 WBGene0 gi|24645360| gi|41151712|ref|XP_374342.1| gi|6704386|ref|Os01g45600|
gi|31204889|ref|XP_311393.1| lcl|At2g46830.2 lcl|C55A1.9 CE08995 WBGene0 gi|24645362| gi|41151714|ref|XP_374343.1| gi|6704389|ref|Os01g45630|
gi|31204929|ref|XP_311413.1| lcl|At2g46940.1 lcl|C55A6.11 CE34778 WBGene gi|24645372| gi|41151716|ref|XP_372275.1| gi|6704390|ref|Os01g45640|

gi|31204945|ref|XP_311421.1|	lcl|At2g46980.1	lcl|C55A6.12 CE37818 WBGene	gi|24645448|	gi|41151747|ref|XP_374377.1|	gi|6704393|ref|Os01g45670|
gi|31204963|ref|XP_311430.1|	lcl|At2g46980.2	lcl|C55B6.4 CE09010 WBGene0	gi|24645483|	gi|41151749|ref|XP_374378.1|	gi|6704396|ref|Os01g45700|
gi|31204985|ref|XP_311441.1|	lcl|At2g47200.1	lcl|C55B7.11 CE09022 WBGene	gi|24645485|	gi|41151753|ref|XP_374379.1|	gi|6704397|ref|Os01g45710|
gi|31205033|ref|XP_311465.1|	lcl|At2g47270.1	lcl|C55B7.9 CE09020 WBGene0	gi|24645519|	gi|41151757|ref|XP_373398.1|	gi|6704398|ref|Os01g45720|
gi|31205045|ref|XP_311471.1|	lcl|At2g47360.1	lcl|C55C2.3 CE36638 WBGene0	gi|24645523|	gi|41151763|ref|XP_293460.4|	gi|6704405|ref|Os01g45770|
gi|31205063|ref|XP_311480.1|	lcl|At2g47530.1	lcl|C55C2.4 CE36639 WBGene0	gi|24645545|	gi|41151769|ref|XP_372295.1|	gi|6704406|ref|Os01g45780|
gi|31205087|ref|XP_311492.1|	lcl|At2g47660.1	lcl|C55C3.2 CE36587 WBGene0	gi|24645549|	gi|41151805|ref|XP_374317.1|	gi|6704407|ref|Os01g45790|
gi|31205107|ref|XP_311502.1|	lcl|At2g47720.1	lcl|C55C3.3 CE29734 WBGene0	gi|24645551|	gi|41151808|ref|XP_372190.1|	gi|6704410|ref|Os01g45820|
gi|31205177|ref|XP_311537.1|	lcl|At2g47930.1	lcl|C55C3.6 CE29736 WBGene0	gi|24645557|	gi|41151812|ref|XP_374318.1|	gi|6704413|ref|Os01g45850|
gi|31205225|ref|XP_311561.1|	lcl|At2g47950.1	lcl|C55C3.7 CE37496 WBGene0	gi|24645576|	gi|41188093|ref|XP_373877.1|	gi|6704415|ref|Os01g45870|
gi|31205237|ref|XP_311567.1|	lcl|At2g48090.1	lcl|C56C10.11 CE28900 WBGen	gi|24645669|	gi|41188104|ref|XP_373878.1|	gi|6704417|ref|Os01g45890|
gi|31205269|ref|XP_311583.1|	lcl|At3g01230.1	lcl|C56C10.12 CE02566 WBGen	gi|24645671|	gi|41188117|ref|XP_372807.1|	gi|6704420|ref|Os01g45920|
gi|31205285|ref|XP_311591.1|	lcl|At3g01240.1	lcl|C56C10.5 CE33522 WBGene	gi|24645673|	gi|41188260|ref|XP_373831.1|	gi|6704421|ref|Os01g45930|
gi|31205287|ref|XP_311592.1|	lcl|At3g01250.1	lcl|C56E10.3 CE30922 WBGene	gi|24645675|	gi|41188451|ref|XP_373881.1|	gi|6704422|ref|Os01g45940|
gi|31205289|ref|XP_311593.1|	lcl|At3g01430.1	lcl|C56E6.3a CE04275 WBGene	gi|24645695|	gi|41188459|ref|XP_373882.1|	gi|6704423|ref|Os01g45950|
gi|31205297|ref|XP_311597.1|	lcl|At3g01513.1	lcl|C56E6.3b CE35261 WBGene	gi|24645697|	gi|41188794|ref|XP_373870.1|	gi|6704425|ref|Os01g45970|
gi|31205303|ref|XP_311600.1|	lcl|At3g01516.1	lcl|C56E6.7 CE37622 WBGene0	gi|24645704|	gi|41188812|ref|XP_373871.1|	gi|6704426|ref|Os01g45980|
gi|31205307|ref|XP_311602.1|	lcl|At3g01670.1	lcl|C56G2.9 CE30641 WBGene0	gi|24645724|	gi|41190371|ref|XP_373980.1|	gi|6704429|ref|Os01g46010|
gi|31205309|ref|XP_311603.1|	lcl|At3g01680.1	lcl|CC4.2 CE36118 WBGene000	gi|24645745|	gi|41190381|ref|XP_373981.1|	gi|6704433|ref|Os01g46050|
gi|31205313|ref|XP_311605.1|	lcl|At3g01700.1	lcl|CC8.1 CE20616 WBGene000	gi|24645747|	gi|41190393|ref|XP_373982.1|	gi|6704443|ref|Os01g46130|
gi|31205333|ref|XP_311615.1|	lcl|At3g01730.1	lcl|CD4.1 CE16949 WBGene000	gi|24645749|	gi|41190396|ref|XP_373983.1|	gi|6704444|ref|Os01g46140|
gi|31205341|ref|XP_311619.1|	lcl|At3g01960.1	lcl|CD4.5 CE36811 WBGene000	gi|24645755|	gi|41190400|ref|XP_373984.1|	gi|6704446|ref|Os01g46160|
gi|31205343|ref|XP_311620.1|	lcl|At3g02120.1	lcl|CE7X_3.1 CE32170 WBGene	gi|24645812|	gi|41190403|ref|XP_373985.1|	gi|6704447|ref|Os01g46170|
gi|31205361|ref|XP_311629.1|	lcl|At3g02125.1	lcl|CE7X_3.2 CE32171 WBGene	gi|24645853|	gi|41190407|ref|XP_371492.1|	gi|6704457|ref|Os01g46270|
gi|31205381|ref|XP_311639.1|	lcl|At3g02170.1	lcl|D1005.3 CE06999 WBGene0	gi|24645911|	gi|41190412|ref|XP_371493.1|	gi|6704458|ref|Os01g46280|
gi|31205407|ref|XP_311652.1|	lcl|At3g02240.1	lcl|D1005.5 CE35444 WBGene0	gi|24645967|	gi|41190419|ref|XP_373986.1|	gi|6704460|ref|Os01g46300|
gi|31205409|ref|XP_311653.1|	lcl|At3g02390.1	lcl|D1005.6 CE07002 WBGene0	gi|24646028|	gi|41190473|ref|XP_373991.1|	gi|6704461|ref|Os01g46310|
gi|31205411|ref|XP_311654.1|	lcl|At3g02480.1	lcl|D1007.10a CE09045 WBGen	gi|24646030|	gi|41190480|ref|XP_373992.1|	gi|6704466|ref|Os01g46360|
gi|31205419|ref|XP_311658.1|	lcl|At3g02500.1	lcl|D1007.10b CE36921 WBGen	gi|24646052|	gi|41191411|ref|XP_374020.1|	gi|6704472|ref|Os01g46420|
gi|31205443|ref|XP_311671.1|	lcl|At3g02555.1	lcl|D1007.10c CE34282 WBGen	gi|24646058|	gi|41191423|ref|XP_371585.1|	gi|6704475|ref|Os01g46450|
gi|31205445|ref|XP_311672.1|	lcl|At3g02670.1	lcl|D1007.13 CE09048 WBGene	gi|24646086|	gi|41191431|ref|XP_374021.1|	gi|6704476|ref|Os01g46460|
gi|31205477|ref|XP_311689.1|	lcl|At3g03020.1	lcl|D1007.14 CE34283 WBGene	gi|24646142|	gi|41191455|ref|XP_374023.1|	gi|6704477|ref|Os01g46470|
gi|31205481|ref|XP_311691.1|	lcl|At3g03170.1	lcl|D1007.16 CE23617 WBGene	gi|24646144|	gi|41191469|ref|XP_371593.1|	gi|6704478|ref|Os01g46480|
gi|31205507|ref|XP_311705.1|	lcl|At3g03405.1	lcl|D1007.3 CE09038 WBGene0	gi|24646167|	gi|41191481|ref|XP_374025.1|	gi|6704479|ref|Os01g46490|
gi|31205511|ref|XP_311707.1|	lcl|At3g03776.1	lcl|D1007.4 CE09039 WBGene0	gi|24646197|	gi|41191493|ref|XP_374026.1|	gi|6704483|ref|Os01g46530|
gi|31205521|ref|XP_311712.1|	lcl|At3g03870.1	lcl|D1007.9 CE09044 WBGene0	gi|24646199|	gi|41191873|ref|XP_372978.1|	gi|6704485|ref|Os01g46550|
gi|31205539|ref|XP_311721.1|	lcl|At3g04040.1	lcl|D1009.4 CE04288 WBGene0	gi|24646225|	gi|41194213|ref|XP_374068.1|	gi|6704494|ref|Os01g46620|
gi|31205541|ref|XP_311722.1|	lcl|At3g04270.1	lcl|D1014.4 CE07006 WBGene0	gi|24646232|	gi|41194216|ref|XP_374069.1|	gi|6704497|ref|Os01g46640|
gi|31205545|ref|XP_311724.1|	lcl|At3g04310.1	lcl|D1025.1 CE17609 WBGene0	gi|24646257|	gi|41194255|ref|XP_373005.1|	gi|6704498|ref|Os01g46650|
gi|31205563|ref|XP_311733.1|	lcl|At3g04410.1	lcl|D1025.4 CE17612 WBGene0	gi|24646261|	gi|41195515|ref|XP_374112.1|	gi|6704500|ref|Os01g46670|
gi|31205565|ref|XP_311734.1|	lcl|At3g04640.1	lcl|D1025.6 CE17612 WBGene0	gi|24646275|	gi|41195522|ref|XP_374115.1|	gi|6704501|ref|Os01g46680|
gi|31205577|ref|XP_311740.1|	lcl|At3g05080.1	lcl|D1025.7 CE31459 WBGene0	gi|24646310|	gi|41196204|ref|XP_371845.1|	gi|6704502|ref|Os01g46690|
gi|31205619|ref|XP_311761.1|	lcl|At3g05460.1	lcl|D1025.8 CE34779 WBGene0	gi|24646328|	gi|41196249|ref|XP_374184.1|	gi|6704509|ref|Os01g46730|
gi|31205621|ref|XP_311762.1|	lcl|At3g05730.1	lcl|D1025.9 CE34779 WBGene0	gi|24646332|	gi|41196251|ref|XP_374185.1|	gi|6704510|ref|Os01g46740|
gi|31205623|ref|XP_311763.1|	lcl|At3g05800.1	lcl|D1044.4 CE01207 WBGene0	gi|24646337|	gi|41197060|ref|XP_374162.1|	gi|6704515|ref|Os01g46790|
gi|31205653|ref|XP_311778.1|	lcl|At3g05935.1	lcl|D1044.5 CE01208 WBGene0	gi|24646339|	gi|41197072|ref|XP_374163.1|	gi|6704518|ref|Os01g46820|
gi|31205665|ref|XP_311784.1|	lcl|At3g05980.1	lcl|D1044.6 CE32173 WBGene0	gi|24646358|	gi|41197084|ref|XP_371808.1|	gi|6704519|ref|Os01g46830|
gi|31205667|ref|XP_311785.1|	lcl|At3g06020.1	lcl|D1053.3 CE03101 WBGene0	gi|24646373|	gi|41197087|ref|XP_371809.1|	gi|6704528|ref|Os01g46910|
gi|31205673|ref|XP_311788.1|	lcl|At3g06070.1	lcl|D1054.10 CE05528 WBGene	gi|24646375|	gi|41197091|ref|XP_374164.1|	gi|6704533|ref|Os01g46960|
gi|31205675|ref|XP_311789.1|	lcl|At3g06090.1	lcl|D1054.11 CE05529 WBGene	gi|24646418|	gi|41197096|ref|XP_374165.1|	gi|6704536|ref|Os01g46990|
gi|31205677|ref|XP_311790.1|	lcl|At3g06145.1	lcl|D1054.5 CE35446 WBGene0	gi|24646430|	gi|41197123|ref|XP_374167.1|	gi|6704538|ref|Os01g47010|
gi|31205683|ref|XP_311793.1|	lcl|At3g06280.1	lcl|D1054.9a CE05527 WBGene	gi|24646467|	gi|41197130|ref|XP_374168.1|	gi|6704543|ref|Os01g47060|
gi|31205689|ref|XP_311796.1|	lcl|At3g06360.1	lcl|D1054.9b CE20626 WBGene	gi|24646485|	gi|41197135|ref|XP_374169.1|	gi|6704546|ref|Os01g47090|
gi|31205697|ref|XP_311800.1|	lcl|At3g06545.1	lcl|D1054.9c CE31897 WBGene	gi|24646505|	gi|41197147|ref|XP_374170.1|	gi|6704548|ref|Os01g47110|
gi|31205699|ref|XP_311801.1|	lcl|At3g06600.1	lcl|D1069.1 CE17613 WBGene0	gi|24646539|	gi|41197150|ref|XP_374171.1|	gi|6704549|ref|Os01g47120|
gi|31205701|ref|XP_311802.1|	lcl|At3g06750.1	lcl|D1079.1 CE09057 WBGene0	gi|24646541|	gi|41199228|ref|XP_374232.1|	gi|6704551|ref|Os01g47140|
gi|31205703|ref|XP_311803.1|	lcl|At3g06780.1	lcl|D1081.5 CE05537 WBGene0	gi|24646543|	gi|41199566|ref|XP_372157.1|	gi|6704558|ref|Os01g47210|
gi|31205707|ref|XP_311805.1|	lcl|At3g06840.1	lcl|D1081.6 CE05538 WBGene0	gi|24646545|	gi|41199578|ref|XP_372160.1|	gi|6704560|ref|Os01g47230|
gi|31205715|ref|XP_311809.1|	lcl|At3g06870.1	lcl|D1081.7 CE05539 WBGene0	gi|24646547|	gi|41199581|ref|XP_374315.1|	gi|6704564|ref|Os01g47270|
gi|31205717|ref|XP_311810.1|	lcl|At3g06895.1	lcl|D1086.1 CE09059 WBGene0	gi|24646549|	gi|41200334|ref|XP_373502.1|	gi|6704566|ref|Os01g47290|
gi|31205719|ref|XP_311811.1|	lcl|At3g07195.1	lcl|D1086.10 CE37962 WBGene	gi|24646566|	gi|41200339|ref|XP_373503.1|	gi|6704567|ref|Os01g47300|
gi|31205731|ref|XP_311817.1|	lcl|At3g07230.1	lcl|D1086.12a CE37964 WBGen	gi|24646568|	gi|41200363|ref|XP_373505.1|	gi|6704568|ref|Os01g47300|
gi|31205751|ref|XP_311827.1|	lcl|At3g07280.1	lcl|D1086.12b CE37965 WBGen	gi|24646570|	gi|41200366|ref|XP_373506.1|	gi|6704569|ref|Os01g47310|
gi|31205753|ref|XP_311828.1|	lcl|At3g07568.1	lcl|D1086.2 CE09060 WBGene0	gi|24646585|	gi|41200375|ref|XP_373507.1|	gi|6704577|ref|Os01g47380|
gi|31205755|ref|XP_311829.1|	lcl|At3g07580.1	lcl|D1086.4 CE09062 WBGene0	gi|24646587|	gi|41200853|ref|XP_373490.1|	gi|6704578|ref|Os01g47390|
gi|31205759|ref|XP_311831.1|	lcl|At3g07710.1	lcl|D1086.5 CE09063 WBGene0	gi|24646591|	gi|41200861|ref|XP_373491.1|	gi|6704584|ref|Os01g47440|
gi|31205777|ref|XP_311840.1|	lcl|At3g07730.1	lcl|D1086.6 CE37966 WBGene0	gi|24646593|	gi|41200871|ref|XP_373492.1|	gi|6704588|ref|Os01g47480|
gi|31205819|ref|XP_311861.1|	lcl|At3g09130.1	lcl|D1086.7 CE17618 WBGene0	gi|24646595|	gi|41200890|ref|XP_373494.1|	gi|6704593|ref|Os01g47500|
gi|31205835|ref|XP_311869.1|	lcl|At3g09280.1	lcl|D1086.8 CE34781 WBGene0	gi|24646597|	gi|41200898|ref|XP_373495.1|	gi|6704595|ref|Os01g47520|
gi|31205843|ref|XP_311873.1|	lcl|At3g09430.1	lcl|D2005.2 CE09066 WBGene0	gi|24646600|	gi|41200905|ref|XP_373498.1|	gi|6704603|ref|Os01g47570|
gi|31205849|ref|XP_311876.1|	lcl|At3g09730.1	lcl|D2005.4 CE17619 WBGene0	gi|24646602|	gi|41200911|ref|XP_373499.1|	gi|6704605|ref|Os01g47590|
gi|31205875|ref|XP_311889.1|	lcl|At3g09735.1	lcl|D2005.6 CE37497 WBGene0	gi|24646705|	gi|41201707|ref|XP_373534.1|	gi|6704613|ref|Os01g47670|
gi|31205877|ref|XP_311890.1|	lcl|At3g09750.1	lcl|D2007.1 CE00126 WBGene0	gi|24646720|	gi|41201709|ref|XP_373535.1|	gi|6704618|ref|Os01g47700|
gi|31205885|ref|XP_311894.1|	lcl|At3g09860.1	lcl|D2007.3 CE00128 WBGene0	gi|24646737|	gi|41201717|ref|XP_172889.2|	gi|6704620|ref|Os01g47720|
gi|31205911|ref|XP_311907.1|	lcl|At3g09922.1	lcl|D2007.5 CE00130 WBGene0	gi|24646749|	gi|41201731|ref|XP_373537.1|	gi|6704638|ref|Os01g47870|
gi|31205913|ref|XP_311908.1|	lcl|At3g10116.1	lcl|D2024.4 CE04293 WBGene0	gi|24646758|	gi|41202054|ref|XP_373527.1|	gi|6704639|ref|Os01g47880|
gi|31205943|ref|XP_311923.1|	lcl|At3g10120.1	lcl|D2024.5a CE27896 WBGene	gi|24646766|	gi|41202573|ref|XP_373557.1|	gi|6704642|ref|Os01g47910|
gi|31205961|ref|XP_311932.1|	lcl|At3g10525.1	lcl|D2024.5b CE31460 WBGene	gi|24646770|	gi|41202577|ref|XP_373558.1|	gi|6704643|ref|Os01g47920|
gi|31205965|ref|XP_311934.1|	lcl|At3g10830.1	lcl|D2030.10b CE30741 WBGen	gi|24646772|	gi|41202582|ref|XP_373559.1|	gi|6704644|ref|Os01g47930|
gi|31206005|ref|XP_311954.1|	lcl|At3g10930.1	lcl|D2030.11 CE30926 WBGene	gi|24646784|	gi|41202592|ref|XP_373560.1|	gi|6704646|ref|Os01g47950|
gi|31206015|ref|XP_311959.1|	lcl|At3g10990.1	lcl|D2030.12 CE34784 WBGene	gi|24646829|	gi|41202608|ref|XP_373561.1|	gi|6704652|ref|Os01g48010|
gi|31206017|ref|XP_311960.1|	lcl|At3g11060.1	lcl|D2045.1a CE37889 WBGene	gi|24646831|	gi|41202611|ref|XP_373562.1|	gi|6704654|ref|Os01g48030|
gi|31206019|ref|XP_311961.1|	lcl|At3g11160.1	lcl|D2045.1b CE37967 WBGene	gi|24646833|	gi|41202614|ref|XP_373563.1|	gi|6704660|ref|Os01g48080|
gi|31206029|ref|XP_311966.1|	lcl|At3g11300.1	lcl|D2062.4 CE29744 WBGene0	gi|24646835|	gi|41202618|ref|XP_370721.1|	gi|6704662|ref|Os01g48100|
gi|31206047|ref|XP_311975.1|	lcl|At3g11640.1	lcl|D2062.5 CE09092 WBGene0	gi|24646837|	gi|41202659|ref|XP_373566.1|	gi|6704663|ref|Os01g48110|
gi|31206057|ref|XP_311980.1|	lcl|At3g11690.1	lcl|D2062.6 CE09093 WBGene0	gi|24646845|	gi|41202661|ref|XP_373567.1|	gi|6704664|ref|Os01g48120|
gi|31206061|ref|XP_311982.1|	lcl|At3g11745.1	lcl|D2062.7 CE09094 WBGene0	gi|24646847|	gi|41202669|ref|XP_373568.1|	gi|6704667|ref|Os01g48150|
gi|31206081|ref|XP_311992.1|	lcl|At3g11860.1	lcl|D2085.7 CE03110 WBGene0	gi|24646849|	gi|41202671|ref|XP_373569.1|	gi|6704668|ref|Os01g48160|
gi|31206107|ref|XP_312005.1|	lcl|At3g12190.1	lcl|D2092.6 CE09105 WBGene0	gi|24646851|	gi|41202717|ref|XP_372494.1|	gi|6704676|ref|Os01g48230|
gi|31206111|ref|XP_312007.1|	lcl|At3g12320.1	lcl|D2092.8 CE09107 WBGene0	gi|24646853|	gi|41203707|ref|XP_373593.1|	gi|6704677|ref|Os01g48240|
gi|31206117|ref|XP_312010.1|	lcl|At3g12510.1	lcl|D2096.10 CE35264 WBGene	gi|24646884|	gi|41203762|ref|XP_370769.1|	gi|6704679|ref|Os01g48260|
gi|31206119|ref|XP_312011.1|	lcl|At3g12545.1	lcl|D2096.12 CE33609 WBGene	gi|24646891|	gi|41203764|ref|XP_373599.1|	gi|6704688|ref|Os01g48340|
gi|31206121|ref|XP_312012.1|	lcl|At3g12840.1	lcl|D2096.5 CE35265 WBGene0	gi|24646895|	gi|41203767|ref|XP_373600.1|	gi|6704690|ref|Os01g48360|
gi|31206123|ref|XP_312013.1|	lcl|At3g12910.1	lcl|D2096.6 CE04304 WBGene0	gi|24646928|	gi|41203786|ref|XP_373602.1|	gi|6704694|ref|Os01g48400|
gi|31206153|ref|XP_312028.1|	lcl|At3g13130.1	lcl|D2096.9 CE04307 WBGene0	gi|24646930|	gi|41203789|ref|XP_373603.1|	gi|6704697|ref|Os01g48430|
gi|31206175|ref|XP_312039.1|	lcl|At3g13175.1	lcl|DC2.4 CE35266 WBGene000	gi|24646972|	gi|41203794|ref|XP_373604.1|	gi|6704701|ref|Os01g48470|
gi|31206183|ref|XP_312043.1|	lcl|At3g13222.1	lcl|DH11.2 CE27900 WBGene00	gi|24646975|	gi|41203797|ref|XP_373605.1|	gi|6704705|ref|Os01g48510|
gi|31206185|ref|XP_312044.1|	lcl|At3g13227.1	lcl|DH11.5a CE32343 WBGene0	gi|24647006|	gi|41203799|ref|XP_373606.1|	gi|6704707|ref|Os01g48530|
gi|31206293|ref|XP_312098.1|	lcl|At3g13240.1	lcl|DH11.5b CE32344 WBGene0	gi|24647058|	gi|41203811|ref|XP_373607.1|	gi|6704709|ref|Os01g48550|
gi|31206309|ref|XP_312106.1|	lcl|At3g13275.1	lcl|DH11.5c CE32345 WBGene0	gi|24647141|	gi|41203819|ref|XP_373609.1|	gi|6704710|ref|Os01g48560|
gi|31206313|ref|XP_312108.1|	lcl|At3g13360.1	lcl|DY3.5 CE15749 WBGene000	gi|24647148|	gi|41203822|ref|XP_373610.1|	gi|6704713|ref|Os01g48590|
gi|31206337|ref|XP_312120.1|	lcl|At3g13370.1	lcl|DY3.8 CE36431 WBGene000	gi|24647172|	gi|41203827|ref|XP_373611.1|	gi|6704719|ref|Os01g48650|
gi|31206355|ref|XP_312129.1|	lcl|At3g13420.1	lcl|E01B7.1 CE20645 WBGene0	gi|24647211|	gi|41203857|ref|XP_370785.1|	gi|6704727|ref|Os01g48710|
gi|31206363|ref|XP_312133.1|	lcl|At3g13435.1	lcl|E01G4.3 CE09110 WBGene0	gi|24647216|	gi|41203901|ref|XP_372524.1|	gi|6704729|ref|Os01g48730|
gi|31206365|ref|XP_312134.1|	lcl|At3g13500.1	lcl|E01G4.4 CE09111 WBGene0	gi|24647220|	gi|41204873|ref|XP_373627.1|	gi|6704736|ref|Os01g48780|
gi|31206371|ref|XP_312137.1|	lcl|At3g13520.1	lcl|E01G4.5 CE23623 WBGene0	gi|24647222|	gi|41204913|ref|XP_373628.1|	gi|6704742|ref|Os01g48820|
gi|31206399|ref|XP_312151.1|	lcl|At3g13630.1	lcl|E01G4.6 CE15755 WBGene0	gi|24647254|	gi|41204933|ref|XP_373630.1|	gi|6704746|ref|Os01g48840|
gi|31206431|ref|XP_312167.1|	lcl|At3g13674.1	lcl|E01G6.2 CE23624 WBGene0	gi|24647270|	gi|41204940|ref|XP_373631.1|	gi|6704751|ref|Os01g48890|
gi|31206461|ref|XP_312190.1|	lcl|At3g13845.1	lcl|E01H11.3 CE29246 WBGene	gi|24647272|	gi|41204948|ref|XP_373632.1|	gi|6704758|ref|Os01g48940|
gi|31206465|ref|XP_312192.1|	lcl|At3g13950.1	lcl|E02A10.4 CE31898 WBGene	gi|24647282|	gi|41204970|ref|XP_373634.1|	gi|6704759|ref|Os01g48950|
gi|31206483|ref|XP_312201.1|	lcl|At3g13965.1	lcl|E02C12.11 CE04320 WBGen	gi|24647289|	gi|41204977|ref|XP_373635.1|	gi|6704761|ref|Os01g48970|
gi|31206495|ref|XP_312210.1|	lcl|At3g13980.1	lcl|E02H1.5 CE01540 WBGene0	gi|24647310|	gi|41204984|ref|XP_373637.1|	gi|6704769|ref|Os01g49040|
gi|31206501|ref|XP_312213.1|	lcl|At3g14030.1	lcl|E02H1.8 CE34789 WBGene0	gi|24647314|	gi|41204992|ref|XP_373638.1|	gi|6704770|ref|Os01g49050|
gi|31206533|ref|XP_312229.1|	lcl|At3g14060.1	lcl|E02H4.2 CE35447 WBGene0	gi|24647328|	gi|41204999|ref|XP_373639.1|	gi|6704772|ref|Os01g49070|
gi|31206561|ref|XP_312243.1|	lcl|At3g14190.1	lcl|E02H4.4 CE05550 WBGene0	gi|24647353|	gi|41205010|ref|XP_370883.1|	gi|6704773|ref|Os01g49080|
gi|31206577|ref|XP_312253.1|	lcl|At3g14340.1	lcl|E02H4.5 CE35847 WBGene0	gi|24647374|	gi|41205024|ref|XP_374355.1|	gi|6704774|ref|Os01g49090|
gi|31206597|ref|XP_312263.1|	lcl|At3g14395.1	lcl|E02H9.1 CE09118 WBGene0	gi|24647385|	gi|41205032|ref|XP_374356.1|	gi|6704778|ref|Os01g49130|
gi|31206609|ref|XP_312271.1|	lcl|At3g14480.1	lcl|E02H9.6 CE09123 WBGene0	gi|24647389|	gi|41205081|ref|XP_372585.1|	gi|6704782|ref|Os01g49170|
gi|31206621|ref|XP_312277.1|	lcl|At3g14560.1	lcl|E02H9.7 CE09124 WBGene0	gi|24647403|	gi|41205608|ref|XP_370906.1|	gi|6704786|ref|Os01g49210|
gi|31206625|ref|XP_312279.1|	lcl|At3g14670.1	lcl|E02H9.9 CE09126 WBGene0	gi|24647405|	gi|41205613|ref|XP_373648.1|	gi|6704787|ref|Os01g49220|
gi|31206637|ref|XP_312285.1|	lcl|At3g14840.1	lcl|E03A3.8 CE02960 WBGene0	gi|24647424|	gi|41205623|ref|XP_373650.1|	gi|6704791|ref|Os01g49260|
gi|31206649|ref|XP_312291.1|	lcl|At3g15115.1	lcl|E03D2.1 CE16958 WBGene0	gi|24647427|	gi|41206118|ref|XP_373670.1|	gi|6704796|ref|Os01g49310|
gi|31206653|ref|XP_312293.1|	lcl|At3g15250.1	lcl|E03D2.2a CE16959 WBGene	gi|24647468|	gi|41206134|ref|XP_373671.1|	gi|6704797|ref|Os01g49310|
gi|31206665|ref|XP_312299.1|	lcl|At3g15300.1	lcl|E03D2.2b CE27903 WBGene	gi|24647470|	gi|41206407|ref|XP_373688.1|	gi|6704803|ref|Os01g49370|
gi|31206677|ref|XP_312305.1|	lcl|At3g15357.1	lcl|E03G2.1 CE03118 WBGene0	gi|24647472|	gi|41206409|ref|XP_373689.1|	gi|6704806|ref|Os01g49400|
gi|31206679|ref|XP_312306.1|	lcl|At3g15400.1	lcl|E03H12.5 CE09144 WBGene	gi|24647474|	gi|41206412|ref|XP_373690.1|	gi|6704807|ref|Os01g49410|
gi|31206691|ref|XP_312312.1|	lcl|At3g15420.1	lcl|E03H12.7 CE09146 WBGene	gi|24647516|	gi|41206417|ref|XP_373691.1|	gi|6704812|ref|Os01g49460|
gi|31206693|ref|XP_312313.1|	lcl|At3g15440.1	lcl|E04A4.3 CE34534 WBGene0	gi|24647528|	gi|41206446|ref|XP_370987.1|	gi|6704814|ref|Os01g49480|
gi|31206695|ref|XP_312314.1|	lcl|At3g15690.1	lcl|E04A4.6 CE16967 WBGene0	gi|24647538|	gi|41206453|ref|XP_372640.1|	gi|6704820|ref|Os01g49540|
gi|31206703|ref|XP_312318.1|	lcl|At3g15760.1	lcl|E04D5.1b CE37821 WBGene	gi|24647572|	gi|41206622|ref|XP_373698.1|	gi|6704821|ref|Os01g49550|
gi|31206707|ref|XP_312320.1|	lcl|At3g15780.1	lcl|E04D5.5 CE34791 WBGene0	gi|24647574|	gi|41206625|ref|XP_373699.1|	gi|6704822|ref|Os01g49560|
gi|31206719|ref|XP_312326.1|	lcl|At3g15860.1	lcl|E04F6.10 CE35267 WBGene	gi|24647588|	gi|41206813|ref|XP_373730.1|	gi|6704823|ref|Os01g49570|
gi|31206723|ref|XP_312328.1|	lcl|At3g15910.1	lcl|E04F6.12 CE01213 WBGene	gi|24647601|	gi|41206815|ref|XP_373731.1|	gi|6704824|ref|Os01g49580|
gi|31206729|ref|XP_312331.1|	lcl|At3g16020.1	lcl|E04F6.6 CE27909 WBGene0	gi|24647603|	gi|41207717|ref|XP_373776.1|	gi|6704826|ref|Os01g49600|

gi|31206763|ref|XP_312348.1|	lcl|At3g16040.1	lcl|E04F6.8 CE01220 WBGene0	gi|24647607|	gi|41208727|ref|XP_373808.1|	gi|6704827|ref|Os01g49610|
gi|31206767|ref|XP_312350.1|	lcl|At3g16070.1	lcl|E04F6.9 CE01221 WBGene0	gi|24647650|	gi|41208774|ref|XP_373810.1|	gi|6704838|ref|Os01g49700|
gi|31206775|ref|XP_312354.1|	lcl|At3g16750.1	lcl|EGAP1.1 CE04322 WBGene(	gi|24647652|	gi|41208782|ref|XP_371187.1|	gi|6704841|ref|Os01g49730|
gi|31206777|ref|XP_312355.1|	lcl|At3g16855.1	lcl|EGAP2.1 CE35268 WBGene(	gi|24647654|	gi|41208826|ref|XP_373811.1|	gi|6704843|ref|Os01g49750|
gi|31206805|ref|XP_312369.1|	lcl|At3g16930.1	lcl|EGAP2.2 CE04324 WBGene(	gi|24647660|	gi|41208863|ref|XP_373812.1|	gi|6704848|ref|Os01g49780|
gi|31206815|ref|XP_312374.1|	lcl|At3g16960.1	lcl|EGAP5.1 CE04326 WBGene(	gi|24647687|	gi|41209342|ref|XP_373936.1|	gi|6704849|ref|Os01g49790|
gi|31206817|ref|XP_312375.1|	lcl|At3g17050.1	lcl|EGAP798.1 CE25840 WBGe	gi|24647694|	gi|41209801|ref|XP_371455.1|	gi|6704850|ref|Os01g49800|
gi|31206823|ref|XP_312378.1|	lcl|At3g17110.1	lcl|E_BE45912.2 CE33578 WBG	gi|24647707|	gi|41209818|ref|XP_373950.1|	gi|6704855|ref|Os01g49840|
gi|31206903|ref|XP_312418.1|	lcl|At3g17140.1	lcl|F01D4.5a CE29035 WBGene	gi|24647709|	gi|41209832|ref|XP_373951.1|	gi|6704857|ref|Os01g49860|
gi|31206905|ref|XP_312419.1|	lcl|At3g17160.1	lcl|F01D4.5b CE29036 WBGene	gi|24647711|	gi|41209835|ref|XP_373952.1|	gi|6704859|ref|Os01g49880|
gi|31206983|ref|XP_312458.1|	lcl|At3g17190.1	lcl|F01D4.7 CE09159 WBGene0	gi|24647717|	gi|41209838|ref|XP_373953.1|	gi|6704864|ref|Os01g49930|
gi|31206985|ref|XP_312459.1|	lcl|At3g17460.1	lcl|F01F1.11 CE26883 WBGene	gi|24647719|	gi|41209845|ref|XP_373954.1|	gi|6704867|ref|Os01g49960|
gi|31207007|ref|XP_312470.1|	lcl|At3g17520.1	lcl|F01F1.14 CE01227 WBGene	gi|24647721|	gi|41209854|ref|XP_373955.1|	gi|6704871|ref|Os01g50000|
gi|31207025|ref|XP_312479.1|	lcl|At3g17580.1	lcl|F01F1.1b CE26885 WBGene	gi|24647723|	gi|41209859|ref|XP_373957.1|	gi|6704886|ref|Os01g50120|
gi|31207043|ref|XP_312488.1|	lcl|At3g17890.1	lcl|F01G10.4 CE09166 WBGene	gi|24647725|	gi|41209861|ref|XP_373958.1|	gi|6704888|ref|Os01g50140|
gi|31207051|ref|XP_312492.1|	lcl|At3g17950.1	lcl|F01G10.9 CE35849 WBGene	gi|24647727|	gi|41210272|ref|XP_374333.1|	gi|6704893|ref|Os01g50190|
gi|31207053|ref|XP_312493.1|	lcl|At3g18120.1	lcl|F01G12.2a CE04332 WBGen	gi|24647751|	gi|41210564|ref|XP_374325.1|	gi|6704895|ref|Os01g50210|
gi|31207063|ref|XP_312498.1|	lcl|At3g18250.1	lcl|F01G12.2b CE30932 WBGen	gi|24647791|	gi|41222009|ref|XP_374200.1|	gi|6704897|ref|Os01g50230|
gi|31207173|ref|XP_312553.1|	lcl|At3g18295.1	lcl|F01G4.4 CE35453 WBGene0	gi|24647801|	gi|41222060|ref|XP_374203.1|	gi|6704898|ref|Os01g50240|
gi|31207175|ref|XP_312554.1|	lcl|At3g18300.1	lcl|F02A9.1a CE00131 WBGene	gi|24647831|	gi|41222066|ref|XP_374204.1|	gi|6704899|ref|Os01g50250|
gi|31207177|ref|XP_312555.1|	lcl|At3g18485.1	lcl|F02A9.1b CE00132 WBGene	gi|24647858|	gi|41222659|ref|XP_374305.1|	gi|6704900|ref|Os01g50260|
gi|31207179|ref|XP_312556.1|	lcl|At3g18510.1	lcl|F02C12.3 CE23626 WBGene	gi|24647893|	gi|41222807|ref|XP_373735.1|	gi|6704901|ref|Os01g50270|
gi|31207181|ref|XP_312557.1|	lcl|At3g18540.1	lcl|F02C9.1 CE09173 WBGene0	gi|24647942|	gi|41222824|ref|XP_373737.1|	gi|6704902|ref|Os01g50280|
gi|31207261|ref|XP_312597.1|	lcl|At3g18700.1	lcl|F02D10.1 CE03136 WBGene	gi|24647944|	gi|41222847|ref|XP_373740.1|	gi|6704903|ref|Os01g50290|
gi|31207281|ref|XP_312607.1|	lcl|At3g19030.1	lcl|F02D10.2 CE03137 WBGene	gi|24647952|	gi|41222849|ref|XP_371073.1|	gi|6704904|ref|Os01g50300|
gi|31207307|ref|XP_312620.1|	lcl|At3g19055.1	lcl|F02D10.3 CE03138 WBGene	gi|24647995|	gi|41281603|ref|NP_277055.1|	gi|6704905|ref|Os01g50310|
gi|31207311|ref|XP_312622.1|	lcl|At3g19200.1	lcl|F02D10.6 CE03141 WBGene	gi|24648017|	gi|41282245|ref|NP_958779.1|	gi|6704911|ref|Os01g50350|
gi|31207349|ref|XP_312641.1|	lcl|At3g19530.1	lcl|F02D8.1 CE36124 WBGene0	gi|24648024|	gi|41327160|ref|NP_898828.1|	gi|6704914|ref|Os01g50380|
gi|31207351|ref|XP_312642.1|	lcl|At3g19650.1	lcl|F02D8.5 CE34794 WBGene0	gi|24648026|	gi|41327689|ref|NP_955807.1|	gi|6704919|ref|Os01g50430|
gi|31207353|ref|XP_312643.1|	lcl|At3g19660.1	lcl|F02E11.2 CE36816 WBGene	gi|24648028|	gi|41349482|ref|NP_005030.2|	gi|6704921|ref|Os01g50450|
gi|31207369|ref|XP_312651.1|	lcl|At3g19780.1	lcl|F02E8.4 CE07019 WBGene0	gi|24648037|	gi|41349484|ref|NP_955385.1|	gi|6704927|ref|Os01g50510|
gi|31207389|ref|XP_312661.1|	lcl|At3g19790.1	lcl|F02E9.1 CE09183 WBGene0	gi|24648044|	gi|41349486|ref|NP_955386.1|	gi|6704932|ref|Os01g50560|
gi|31207391|ref|XP_312662.1|	lcl|At3g20155.1	lcl|F02E9.3 CE34291 WBGene0	gi|24648059|	gi|41349488|ref|NP_006240.3|	gi|6704936|ref|Os01g50600|
gi|31207415|ref|XP_312674.1|	lcl|At3g20340.1	lcl|F02E9.5 CE23628 WBGene0	gi|24648063|	gi|41352703|ref|NP_958801.1|	gi|6704939|ref|Os01g50630|
gi|31207423|ref|XP_312678.1|	lcl|At3g20362.1	lcl|F02H6.1 CE17625 WBGene0	gi|24648065|	gi|41462406|ref|NP_958803.2|	gi|6704948|ref|Os01g50710|
gi|31207427|ref|XP_312680.1|	lcl|At3g20400.1	lcl|F02H6.2 CE17626 WBGene0	gi|24648074|	gi|41680644|ref|NP_963841.1|	gi|6704951|ref|Os01g50740|
gi|31207433|ref|XP_312683.1|	lcl|At3g20470.1	lcl|F02H6.3a CE17627 WBGene	gi|24648081|	gi|42475965|ref|NP_037442.2|	gi|6704957|ref|Os01g50780|
gi|31207435|ref|XP_312684.1|	lcl|At3g20555.1	lcl|F02H6.3b CE27914 WBGene	gi|24648083|	gi|42476018|ref|NP_060384.2|	gi|6704959|ref|Os01g50800|
gi|31207439|ref|XP_312686.1|	lcl|At3g20850.1	lcl|F02H6.4 CE17628 WBGene0	gi|24648085|	gi|42476155|ref|NP_002815.2|	gi|6704964|ref|Os01g50850|
gi|31207447|ref|XP_312690.1|	lcl|At3g20865.1	lcl|F07A11.1 CE03143 WBGene	gi|24648087|	gi|42600569|ref|NP_976056.1|	gi|6704966|ref|Os01g50870|
gi|31207453|ref|XP_312693.1|	lcl|At3g20900.1	lcl|F07A11.3 CE03145 WBGene	gi|24648091|	gi|42655600|ref|XP_378933.1|	gi|6704967|ref|Os01g50880|
gi|31207459|ref|XP_312696.1|	lcl|At3g20980.1	lcl|F07A5.2 CE05555 WBGene0	gi|24648093|	gi|42655602|ref|XP_378934.1|	gi|6704968|ref|Os01g50890|
gi|31207463|ref|XP_312698.1|	lcl|At3g21000.1	lcl|F07A5.4 CE28905 WBGene0	gi|24648127|	gi|42655615|ref|XP_375841.1|	gi|6704971|ref|Os01g50920|
gi|31207467|ref|XP_312700.1|	lcl|At3g21055.1	lcl|F07A5.6 CE32852 WBGene0	gi|24648136|	gi|42655617|ref|XP_375842.1|	gi|6704974|ref|Os01g50950|
gi|31207475|ref|XP_312704.1|	lcl|At3g21570.1	lcl|F07B7.1 CE21283 WBGene0	gi|24648147|	gi|42655619|ref|XP_375843.1|	gi|6704979|ref|Os01g51000|
gi|31207477|ref|XP_312705.1|	lcl|At3g21680.1	lcl|F07B7.2 CE21282 WBGene0	gi|24648158|	gi|42655629|ref|XP_086360.4|	gi|6704982|ref|Os01g51030|
gi|31207489|ref|XP_312711.1|	lcl|At3g21710.1	lcl|F07B7.7 CE07602 WBGene0	gi|24648195|	gi|42655645|ref|XP_378889.1|	gi|6704986|ref|Os01g51070|
gi|31207493|ref|XP_312713.1|	lcl|At3g22070.1	lcl|F07C3.2 CE07024 WBGene0	gi|24648205|	gi|42655647|ref|XP_375774.1|	gi|6704990|ref|Os01g51110|
gi|31207501|ref|XP_312717.1|	lcl|At3g22090.1	lcl|F07C3.5 CE07027 WBGene0	gi|24648266|	gi|42655655|ref|XP_378873.1|	gi|6705004|ref|Os01g51240|
gi|31207509|ref|XP_312721.1|	lcl|At3g22140.1	lcl|F07C3.9 CE07030 WBGene0	gi|24648281|	gi|42655657|ref|XP_375744.1|	gi|6705007|ref|Os01g51270|
gi|31207513|ref|XP_312723.1|	lcl|At3g22160.1	lcl|F07C4.7 CE09206 WBGene0	gi|24648296|	gi|42655665|ref|XP_378877.1|	gi|6705013|ref|Os01g51310|
gi|31207529|ref|XP_312731.1|	lcl|At3g22210.1	lcl|F07C6.2 CE05563 WBGene0	gi|24648298|	gi|42655667|ref|XP_378879.1|	gi|6705015|ref|Os01g51330|
gi|31207545|ref|XP_312739.1|	lcl|At3g22231.1	lcl|F07C6.3 CE05564 WBGene0	gi|24648312|	gi|42655669|ref|XP_378880.1|	gi|6705017|ref|Os01g51350|
gi|31207551|ref|XP_312742.1|	lcl|At3g22235.1	lcl|F07D3.2 CE29252 WBGene0	gi|24648317|	gi|42655686|ref|XP_378840.1|	gi|6705019|ref|Os01g51370|
gi|31207575|ref|XP_312754.1|	lcl|At3g22240.1	lcl|F07E5.1 CE09214 WBGene0	gi|24648325|	gi|42655688|ref|XP_378841.1|	gi|6705026|ref|Os01g51440|
gi|31207595|ref|XP_312764.1|	lcl|At3g22510.1	lcl|F07E5.4 CE09216 WBGene0	gi|24648353|	gi|42655690|ref|XP_378842.1|	gi|6705028|ref|Os01g51460|
gi|31207605|ref|XP_312769.1|	lcl|At3g23040.1	lcl|F07E5.9 CE09221 WBGene0	gi|24648370|	gi|42655694|ref|XP_378843.1|	gi|6705029|ref|Os01g51470|
gi|31207625|ref|XP_312779.1|	lcl|At3g23170.1	lcl|F07F6.2 CE35272 WBGene0	gi|24648377|	gi|42655711|ref|XP_373866.2|	gi|6705030|ref|Os01g51480|
gi|31207627|ref|XP_312780.1|	lcl|At3g23172.1	lcl|F07F6.5 CE01897 WBGene0	gi|24648405|	gi|42655719|ref|XP_373868.2|	gi|6705031|ref|Os01g51490|
gi|31207643|ref|XP_312788.1|	lcl|At3g23245.1	lcl|F07F6.7 CE29956 WBGene0	gi|24648407|	gi|42655721|ref|XP_375802.1|	gi|6705033|ref|Os01g51510|
gi|31207645|ref|XP_312789.1|	lcl|At3g23295.1	lcl|F07F6.8 CE30073 WBGene0	gi|24648498|	gi|42655723|ref|XP_375803.1|	gi|6705034|ref|Os01g51520|
gi|31207657|ref|XP_312795.1|	lcl|At3g23450.1	lcl|F07G6.1 CE07034 WBGene(	gi|24648514|	gi|42655725|ref|XP_378901.1|	gi|6705038|ref|Os01g51560|
gi|31207659|ref|XP_312796.1|	lcl|At3g23650.1	lcl|F07G6.2 CE07035 WBGene(	gi|24648574|	gi|42655736|ref|XP_378855.1|	gi|6705040|ref|Os01g51580|
gi|31207667|ref|XP_312800.1|	lcl|At3g23680.1	lcl|F07G6.3 CE07036 WBGene(	gi|24648597|	gi|42655742|ref|XP_378858.1|	gi|6705041|ref|Os01g51590|
gi|31207671|ref|XP_312802.1|	lcl|At3g23720.1	lcl|F07G6.5 CE07038 WBGene(	gi|24648637|	gi|42655749|ref|XP_378860.1|	gi|6705042|ref|Os01g51600|
gi|31207675|ref|XP_312804.1|	lcl|At3g23850.1	lcl|F07G6.8 CE07041 WBGene(	gi|24648702|	gi|42655752|ref|XP_378861.1|	gi|6705048|ref|Os01g51660|
gi|31207697|ref|XP_312815.1|	lcl|At3g23930.1	lcl|F07H5.10 CE28906 WBGene	gi|24648761|	gi|42655759|ref|XP_373843.2|	gi|6705049|ref|Os01g51670|
gi|31207699|ref|XP_312816.1|	lcl|At3g23970.1	lcl|F07H5.12 CE03161 WBGene	gi|24648765|	gi|42655778|ref|XP_378865.1|	gi|6705050|ref|Os01g51680|
gi|31207711|ref|XP_312822.1|	lcl|At3g24100.1	lcl|F07H5.13 CE29037 WBGene	gi|24648794|	gi|42655780|ref|XP_378866.1|	gi|6705054|ref|Os01g51720|
gi|31207721|ref|XP_312827.1|	lcl|At3g24225.1	lcl|F07H5.6 CE03155 WBGene0	gi|24648814|	gi|42655788|ref|XP_373847.2|	gi|6705055|ref|Os01g51730|
gi|31207745|ref|XP_312839.1|	lcl|At3g24250.1	lcl|F07H5.8 CE03157 WBGene0	gi|24648816|	gi|42655794|ref|XP_378893.1|	gi|6705056|ref|Os01g51740|
gi|31207755|ref|XP_312844.1|	lcl|At3g24260.1	lcl|F08B1.3 CE27921 WBGene0	gi|24648818|	gi|42655796|ref|XP_378894.1|	gi|6705073|ref|Os01g51900|
gi|31207769|ref|XP_312851.1|	lcl|At3g24280.1	lcl|F08B12.4 CE26708 WBGene	gi|24648820|	gi|42655810|ref|XP_378899.1|	gi|6705074|ref|Os01g51910|
gi|31207803|ref|XP_312868.1|	lcl|At3g24380.1	lcl|F08B12.5 CE34796 WBGene	gi|24648822|	gi|42655814|ref|XP_373865.2|	gi|6705080|ref|Os01g51930|
gi|31207805|ref|XP_312869.1|	lcl|At3g24510.1	lcl|F08B4.3 CE07044 WBGene0	gi|24648824|	gi|42655817|ref|XP_378183.1|	gi|6705081|ref|Os01g51940|
gi|31207819|ref|XP_312876.1|	lcl|At3g24517.1	lcl|F08B4.4 CE07045 WBGene0	gi|24648834|	gi|42655819|ref|XP_378929.1|	gi|6705083|ref|Os01g51960|
gi|31207823|ref|XP_312878.1|	lcl|At3g24535.1	lcl|F08B6.1 CE20655 WBGene0	gi|24648838|	gi|42655821|ref|XP_378930.1|	gi|6705084|ref|Os01g51970|
gi|31207835|ref|XP_312884.1|	lcl|At3g24630.1	lcl|F08C6.5 CE02594 WBGene0	gi|24648855|	gi|42655826|ref|XP_375833.1|	gi|6705089|ref|Os01g52020|
gi|31207853|ref|XP_312893.1|	lcl|At3g24640.1	lcl|F08D12.12 CE37221 WBGen	gi|24648865|	gi|42655830|ref|XP_378820.1|	gi|6705091|ref|Os01g52040|
gi|31207879|ref|XP_312906.1|	lcl|At3g24750.1	lcl|F08D12.13 CE37339 WBGen	gi|24648869|	gi|42655833|ref|XP_375707.1|	gi|6705093|ref|Os01g52060|
gi|31207889|ref|XP_312911.1|	lcl|At3g24770.1	lcl|F08F1.3 CE09250 WBGene0	gi|24648902|	gi|42655841|ref|XP_375681.1|	gi|6705095|ref|Os01g52080|
gi|31207903|ref|XP_312918.1|	lcl|At3g25130.1	lcl|F08F1.4a CE27928 WBGene	gi|24648907|	gi|42655849|ref|XP_378828.1|	gi|6705105|ref|Os01g52120|
gi|31207911|ref|XP_312922.1|	lcl|At3g25165.1	lcl|F08F1.4b CE34293 WBGene	gi|24648909|	gi|42655851|ref|XP_377556.1|	gi|6705108|ref|Os01g52150|
gi|31207951|ref|XP_312942.1|	lcl|At3g25200.1	lcl|F08F3.1 CE09257 WBGene0	gi|24648911|	gi|42655866|ref|XP_378917.1|	gi|6705125|ref|Os01g52300|
gi|31207961|ref|XP_312947.1|	lcl|At3g25545.1	lcl|F08F3.10 CE32855 WBGene	gi|24648927|	gi|42655868|ref|XP_378918.1|	gi|6705130|ref|Os01g52350|
gi|31207975|ref|XP_312954.1|	lcl|At3g25590.1	lcl|F08F3.8 CE09263 WBGene0	gi|24648933|	gi|42655870|ref|XP_378919.1|	gi|6705132|ref|Os01g52370|
gi|31208005|ref|XP_312969.1|	lcl|At3g25630.1	lcl|F08F3.9a CE30743 WBGene	gi|24648936|	gi|42655872|ref|XP_378921.1|	gi|6705137|ref|Os01g52420|
gi|31208007|ref|XP_312970.1|	lcl|At3g25655.1	lcl|F08F3.9b CE09264 WBGene	gi|24648938|	gi|42655877|ref|XP_375821.1|	gi|6705139|ref|Os01g52440|
gi|31208027|ref|XP_312980.1|	lcl|At3g25727.1	lcl|F08F8.1 CE02597 WBGene0	gi|24648940|	gi|42655880|ref|XP_378923.1|	gi|6705157|ref|Os01g52590|
gi|31208039|ref|XP_312986.1|	lcl|At3g25870.1	lcl|F08F8.10a CE30648 WBGen	gi|24648942|	gi|42655884|ref|XP_378852.1|	gi|6705158|ref|Os01g52600|
gi|31208051|ref|XP_312992.1|	lcl|At3g25882.1	lcl|F08F8.10b CE37222 WBGen	gi|24648958|	gi|42655889|ref|XP_375713.1|	gi|6705168|ref|Os01g52670|
gi|31208083|ref|XP_313008.1|	lcl|At3g25905.1	lcl|F08F8.4 CE27932 WBGene0	gi|24648981|	gi|42655893|ref|XP_377585.1|	gi|6705171|ref|Os01g52700|
gi|31208105|ref|XP_313019.1|	lcl|At3g26110.1	lcl|F08F8.5 CE02601 WBGene0	gi|24648983|	gi|42655897|ref|XP_378848.1|	gi|6705177|ref|Os01g52760|
gi|31208123|ref|XP_313028.1|	lcl|At3g26235.1	lcl|F08F8.6 CE27933 WBGene0	gi|24649003|	gi|42655903|ref|XP_378831.1|	gi|6705183|ref|Os01g52820|
gi|31208127|ref|XP_313030.1|	lcl|At3g26390.1	lcl|F08F8.9a CE27936 WBGene	gi|24649080|	gi|42655905|ref|XP_378832.1|	gi|6705186|ref|Os01g52850|
gi|31208141|ref|XP_313037.1|	lcl|At3g26595.1	lcl|F08F8.9b CE27937 WBGene	gi|24649095|	gi|42655909|ref|XP_378186.1|	gi|6705188|ref|Os01g52870|
gi|31208155|ref|XP_313044.1|	lcl|At3g26616.1	lcl|F08F8.9c CE30651 WBGene(	gi|24649097|	gi|42655911|ref|XP_375845.1|	gi|6705192|ref|Os01g52910|
gi|31208191|ref|XP_313062.1|	lcl|At3g26800.1	lcl|F08G12.3 CE03168 WBGene	gi|24649128|	gi|42655914|ref|XP_375846.1|	gi|6705200|ref|Os01g52990|
gi|31208215|ref|XP_313074.1|	lcl|At3g26855.1	lcl|F08G12.8 CE03172 WBGene	gi|24649150|	gi|42655940|ref|XP_378187.1|	gi|6705202|ref|Os01g53010|
gi|31208217|ref|XP_313075.1|	lcl|At3g27025.1	lcl|F08G2.4 CE19776 WBGene0	gi|24649156|	gi|42655945|ref|XP_378909.1|	gi|6705204|ref|Os01g53030|
gi|31208223|ref|XP_313078.1|	lcl|At3g27250.1	lcl|F08G2.5 CE19777 WBGene0	gi|24649166|	gi|42655947|ref|XP_378910.1|	gi|6705213|ref|Os01g53120|
gi|31208281|ref|XP_313107.1|	lcl|At3g27370.1	lcl|F08G2.7 CE19779 WBGene0	gi|24649171|	gi|42655958|ref|XP_378925.1|	gi|6705219|ref|Os01g53180|
gi|31208283|ref|XP_313108.1|	lcl|At3g27410.1	lcl|F08G2.8 CE19780 WBGene0	gi|24649189|	gi|42655972|ref|XP_378874.1|	gi|6705220|ref|Os01g53190|
gi|31208301|ref|XP_313117.1|	lcl|At3g27420.1	lcl|F08G5.1 CE05571 WBGene0	gi|24649230|	gi|42655974|ref|XP_378876.1|	gi|6705224|ref|Os01g53230|
gi|31208303|ref|XP_313118.1|	lcl|At3g27520.1	lcl|F08G5.3a CE32857 WBGene	gi|24649232|	gi|42655978|ref|XP_378886.1|	gi|6705232|ref|Os01g53310|
gi|31208305|ref|XP_313119.1|	lcl|At3g27590.1	lcl|F08G5.3b CE35455 WBGene	gi|24649252|	gi|42655987|ref|XP_378941.1|	gi|6705235|ref|Os01g53340|
gi|31208313|ref|XP_313123.1|	lcl|At3g27630.1	lcl|F08H9.12 CE37343 WBGene	gi|24649256|	gi|42655990|ref|XP_375856.1|	gi|6705237|ref|Os01g53360|
gi|31208315|ref|XP_313124.1|	lcl|At3g27800.1	lcl|F08H9.2 CE09266 WBGene0	gi|24649258|	gi|42655992|ref|XP_371353.2|	gi|6705245|ref|Os01g53440|
gi|31208317|ref|XP_313125.1|	lcl|At3g27880.1	lcl|F09A5.3 CE03175 WBGene0	gi|24649316|	gi|42655995|ref|XP_378945.1|	gi|6705251|ref|Os01g53490|
gi|31208347|ref|XP_313140.1|	lcl|At3g27990.1	lcl|F09B12.1a CE23633 WBGen	gi|24649321|	gi|42655997|ref|XP_378946.1|	gi|6705256|ref|Os01g53530|
gi|31208363|ref|XP_313148.1|	lcl|At3g28090.1	lcl|F09B12.1b CE23634 WBGen	gi|24649323|	gi|42656012|ref|XP_378837.1|	gi|6705257|ref|Os01g53540|
gi|31208395|ref|XP_313164.1|	lcl|At3g28110.1	lcl|F09B9.4 CE37344 WBGene0	gi|24649325|	gi|42656034|ref|XP_378815.1|	gi|6705258|ref|Os01g53550|
gi|31208411|ref|XP_313172.1|	lcl|At3g28120.1	lcl|F09C11.1 CE16970 WBGene	gi|24649345|	gi|42656036|ref|XP_375667.1|	gi|6705273|ref|Os01g53690|
gi|31208459|ref|XP_313196.1|	lcl|At3g28170.1	lcl|F09C12.6 CE35456 WBGene	gi|24649369|	gi|42656038|ref|XP_375668.1|	gi|6705278|ref|Os01g53740|
gi|31208465|ref|XP_313199.1|	lcl|At3g28190.1	lcl|F09C6.10 CE18583 WBGene	gi|24649444|	gi|42656042|ref|XP_374153.2|	gi|6705280|ref|Os01g53760|
gi|31208473|ref|XP_313203.1|	lcl|At3g28240.1	lcl|F09C6.3 CE18581 WBGene0	gi|24649475|	gi|42656044|ref|XP_375670.1|	gi|6705281|ref|Os01g53770|
gi|31208477|ref|XP_313205.1|	lcl|At3g28260.1	lcl|F09E10.1 CE29254 WBGene	gi|24649532|	gi|42656046|ref|XP_375671.1|	gi|6705286|ref|Os01g53820|
gi|31208481|ref|XP_313207.1|	lcl|At3g28280.1	lcl|F09E10.7b CE34537 WBGen	gi|24649592|	gi|42656048|ref|XP_378822.1|	gi|6705289|ref|Os01g53850|
gi|31208483|ref|XP_313208.1|	lcl|At3g28310.1	lcl|F09E5.10 CE02605 WBGene	gi|24649594|	gi|42656054|ref|XP_378823.1|	gi|6705290|ref|Os01g53860|
gi|31208501|ref|XP_313217.1|	lcl|At3g28330.1	lcl|F09E5.11 CE02606 WBGene	gi|24649620|	gi|42656056|ref|XP_378824.1|	gi|6705304|ref|Os01g53940|
gi|31208511|ref|XP_313222.1|	lcl|At3g28400.1	lcl|F09E5.12 CE02607 WBGene	gi|24649638|	gi|42656058|ref|XP_378825.1|	gi|6705305|ref|Os01g53950|
gi|31208519|ref|XP_313226.1|	lcl|At3g28420.1	lcl|F09E5.13 CE02608 WBGene	gi|24649640|	gi|42656065|ref|XP_378838.1|	gi|6705307|ref|Os01g53970|
gi|31208527|ref|XP_313230.1|	lcl|At3g28455.1	lcl|F09E5.14 CE31780 WBGene	gi|24649692|	gi|42656069|ref|XP_378892.1|	gi|6705308|ref|Os01g53980|
gi|31208541|ref|XP_313237.1|	lcl|At3g28530.1	lcl|F09E5.16 CE32362 WBGene	gi|24649719|	gi|42656073|ref|XP_375779.1|	gi|6705311|ref|Os01g54000|
gi|31208543|ref|XP_313238.1|	lcl|At3g28550.1	lcl|F09E5.7 CE02614 WBGene0	gi|24649739|	gi|42656079|ref|XP_371301.2|	gi|6705319|ref|Os01g54060|
gi|31208549|ref|XP_313241.1|	lcl|At3g28590.1	lcl|F09E5.9 CE02616 WBGene0	gi|24649747|	gi|42656087|ref|XP_378950.1|	gi|6705320|ref|Os01g54070|
gi|31208553|ref|XP_313243.1|	lcl|At3g28750.1	lcl|F09E8.8 CE34798 WBGene0	gi|24649753|	gi|42656091|ref|XP_378951.1|	gi|6705332|ref|Os01g54190|
gi|31208555|ref|XP_313244.1|	lcl|At3g28800.1	lcl|F09F7.1 CE29258 WBGene0	gi|24649773|	gi|42656093|ref|XP_375875.1|	gi|6705333|ref|Os01g54190|
gi|31208563|ref|XP_313248.1|	lcl|At3g28925.1	lcl|F09F7.6 CE01238 WBGene0	gi|24649815|	gi|42656095|ref|XP_375876.1|	gi|6705334|ref|Os01g54200|
gi|31208583|ref|XP_313258.1|	lcl|At3g29033.1	lcl|F09F7.8 CE01240 WBGene0	gi|24649818|	gi|42656104|ref|XP_378890.1|	gi|6705336|ref|Os01g54210|
gi|31208585|ref|XP_313259.1|	lcl|At3g29034.1	lcl|F09F9.1 CE04346 WBGene0	gi|24649820|	gi|42656106|ref|XP_378891.1|	gi|6705347|ref|Os01g54310|
gi|31208587|ref|XP_313260.1|	lcl|At3g29080.1	lcl|F09F9.2 CE04347 WBGene0	gi|24649822|	gi|42656111|ref|XP_378897.1|	gi|6705348|ref|Os01g54320|
gi|31208591|ref|XP_313262.1|	lcl|At3g29140.1	lcl|F09F9.3 CE04348 WBGene0	gi|24649867|	gi|42656113|ref|XP_378898.1|	gi|6705349|ref|Os01g54330|
gi|31208597|ref|XP_313265.1|	lcl|At3g29210.1	lcl|F09G2.9 CE09304 WBGene0	gi|24649869|	gi|42656127|ref|XP_378903.1|	gi|6705350|ref|Os01g54340|

<table>
<tr><td>gi|31208615|ref|XP_313274.1|</td><td>lcl|At3g29300.1</td><td>lcl|F09G8.7 CE00142 WBGene0</td><td>gi|24649871|</td><td>gi|42656133|ref|XP_378905.1|</td><td>gi|6705354|ref|Os01g54360|</td></tr>
<tr><td>gi|31208621|ref|XP_313277.1|</td><td>lcl|At3g29305.1</td><td>lcl|F09G8.9 CE00144 WBGene0</td><td>gi|24649893|</td><td>gi|42656149|ref|XP_378949.1|</td><td>gi|6705359|ref|Os01g54410|</td></tr>
<tr><td>gi|31208623|ref|XP_313278.1|</td><td>lcl|At3g29310.1</td><td>lcl|F10C1.1 CE02617 WBGene0</td><td>gi|24649908|</td><td>gi|42656186|ref|XP_378954.1|</td><td>gi|6705364|ref|Os01g54460|</td></tr>
<tr><td>gi|31208657|ref|XP_313295.1|</td><td>lcl|At3g29370.1</td><td>lcl|F10C1.3 CE02620 WBGene0</td><td>gi|24649947|</td><td>gi|42656188|ref|XP_378955.1|</td><td>gi|6705369|ref|Os01g54500|</td></tr>
<tr><td>gi|31208675|ref|XP_313304.1|</td><td>lcl|At3g29420.1</td><td>lcl|F10C1.8b CE31904 WBGene</td><td>gi|24649954|</td><td>gi|42656190|ref|XP_379078.1|</td><td>gi|6705392|ref|Os01g54710|</td></tr>
<tr><td>gi|31208687|ref|XP_313310.1|</td><td>lcl|At3g29520.1</td><td>lcl|F10C2.2 CE09306 WBGene0</td><td>gi|24650052|</td><td>gi|42656193|ref|XP_379079.1|</td><td>gi|6705393|ref|Os01g54720|</td></tr>
<tr><td>gi|31208691|ref|XP_313312.1|</td><td>lcl|At3g29560.1</td><td>lcl|F10D11.2 CE32366 WBGene</td><td>gi|24650066|</td><td>gi|42656196|ref|XP_379080.1|</td><td>gi|6705395|ref|Os01g54740|</td></tr>
<tr><td>gi|31208703|ref|XP_313318.1|</td><td>lcl|At3g29570.1</td><td>lcl|F10D11.3 CE09325 WBGene</td><td>gi|24650090|</td><td>gi|42656199|ref|XP_377732.1|</td><td>gi|6705396|ref|Os01g54750|</td></tr>
<tr><td>gi|31208715|ref|XP_313324.1|</td><td>lcl|At3g29600.1</td><td>lcl|F10D11.4 CE09326 WBGene</td><td>gi|24650092|</td><td>gi|42656203|ref|XP_379122.1|</td><td>gi|6705397|ref|Os01g54760|</td></tr>
<tr><td>gi|31208809|ref|XP_313371.1|</td><td>lcl|At3g29610.1</td><td>lcl|F10D7.4 CE04354 WBGene0</td><td>gi|24650099|</td><td>gi|42656205|ref|XP_376121.1|</td><td>gi|6705398|ref|Os01g54770|</td></tr>
<tr><td>gi|31208823|ref|XP_313378.1|</td><td>lcl|At3g29636.1</td><td>lcl|F10E7.2 CE35460 WBGene0</td><td>gi|24650118|</td><td>gi|42656207|ref|XP_379123.1|</td><td>gi|6705399|ref|Os01g54780|</td></tr>
<tr><td>gi|31208827|ref|XP_313380.1|</td><td>lcl|At3g29700.1</td><td>lcl|F10E7.3 CE36819 WBGene0</td><td>gi|24650152|</td><td>gi|42656215|ref|XP_372950.2|</td><td>gi|6705401|ref|Os01g54800|</td></tr>
<tr><td>gi|31208837|ref|XP_313385.1|</td><td>lcl|At3g29710.1</td><td>lcl|F10E7.6 CE04361 WBGene0</td><td>gi|24650159|</td><td>gi|42656226|ref|XP_379113.1|</td><td>gi|6705405|ref|Os01g54840|</td></tr>
<tr><td>gi|31208839|ref|XP_313386.1|</td><td>lcl|At3g29780.1</td><td>lcl|F10E9.1 CE00145 WBGene0</td><td>gi|24650202|</td><td>gi|42656228|ref|XP_379114.1|</td><td>gi|6705420|ref|Os01g54980|</td></tr>
<tr><td>gi|31208863|ref|XP_313398.1|</td><td>lcl|At3g29785.1</td><td>lcl|F10E9.10 CE28908 WBGene</td><td>gi|24650208|</td><td>gi|42656239|ref|XP_379117.1|</td><td>gi|6705422|ref|Os01g55000|</td></tr>
<tr><td>gi|31208869|ref|XP_313401.1|</td><td>lcl|At3g29786.1</td><td>lcl|F10E9.11 CE29491 WBGene</td><td>gi|24650232|</td><td>gi|42656243|ref|XP_379119.1|</td><td>gi|6705423|ref|Os01g55010|</td></tr>
<tr><td>gi|31208873|ref|XP_313403.1|</td><td>lcl|At3g29790.1</td><td>lcl|F10E9.12 CE29492 WBGene</td><td>gi|24650263|</td><td>gi|42656257|ref|XP_376190.1|</td><td>gi|6705424|ref|Os01g55020|</td></tr>
<tr><td>gi|31208893|ref|XP_313413.1|</td><td>lcl|At3g29791.1</td><td>lcl|F10E9.2 CE24893 WBGene0</td><td>gi|24650265|</td><td>gi|42656259|ref|XP_376191.1|</td><td>gi|6705429|ref|Os01g55060|</td></tr>
<tr><td>gi|31208909|ref|XP_313421.1|</td><td>lcl|At3g29796.1</td><td>lcl|F10E9.3 CE29959 WBGene0</td><td>gi|24650307|</td><td>gi|42656261|ref|XP_379166.1|</td><td>gi|6705431|ref|Os01g55080|</td></tr>
<tr><td>gi|31208915|ref|XP_313424.1|</td><td>lcl|At3g30150.1</td><td>lcl|F10E9.5 CE36128 WBGene0</td><td>gi|24650309|</td><td>gi|42656263|ref|XP_378190.1|</td><td>gi|6705435|ref|Os01g55120|</td></tr>
<tr><td>gi|31208923|ref|XP_313428.1|</td><td>lcl|At3g30160.1</td><td>lcl|F10E9.8 CE29756 WBGene0</td><td>gi|24650319|</td><td>gi|42656283|ref|XP_379085.1|</td><td>gi|6705436|ref|Os01g55130|</td></tr>
<tr><td>gi|31208933|ref|XP_313433.1|</td><td>lcl|At3g30220.1</td><td>lcl|F10F2.9 CE00954 WBGene0</td><td>gi|24650338|</td><td>gi|42656285|ref|XP_379086.1|</td><td>gi|6705437|ref|Os01g55140|</td></tr>
<tr><td>gi|31208939|ref|XP_313436.1|</td><td>lcl|At3g30230.1</td><td>lcl|F10G2.4 CE09332 WBGene0</td><td>gi|24650340|</td><td>gi|42656294|ref|XP_376049.1|</td><td>gi|6705439|ref|Os01g55160|</td></tr>
<tr><td>gi|31208943|ref|XP_313438.1|</td><td>lcl|At3g30240.1</td><td>lcl|F10G7.6 CE02630 WBGene0</td><td>gi|24650366|</td><td>gi|42656300|ref|XP_379089.1|</td><td>gi|6705441|ref|Os01g55180|</td></tr>
<tr><td>gi|31208945|ref|XP_313439.1|</td><td>lcl|At3g30250.1</td><td>lcl|F10G7.9a CE32371 WBGene</td><td>gi|24650391|</td><td>gi|42656302|ref|XP_376051.1|</td><td>gi|6705445|ref|Os01g55220|</td></tr>
<tr><td>gi|31208959|ref|XP_313446.1|</td><td>lcl|At3g30320.1</td><td>lcl|F10G7.9b CE32372 WBGene</td><td>gi|24650395|</td><td>gi|42656306|ref|XP_379094.1|</td><td>gi|6705446|ref|Os01g55230|</td></tr>
<tr><td>gi|31208961|ref|XP_313447.1|</td><td>lcl|At3g30330.1</td><td>lcl|F10G8.2 CE36532 WBGene0</td><td>gi|24650425|</td><td>gi|42656312|ref|XP_379096.1|</td><td>gi|6705448|ref|Os01g55250|</td></tr>
<tr><td>gi|31208963|ref|XP_313448.1|</td><td>lcl|At3g30350.1</td><td>lcl|F11A1.2 CE03180 WBGene0</td><td>gi|24650433|</td><td>gi|42656314|ref|XP_379097.1|</td><td>gi|6705457|ref|Os01g55330|</td></tr>
<tr><td>gi|31208965|ref|XP_313449.1|</td><td>lcl|At3g30360.1</td><td>lcl|F11A5.13 CE15791 WBGene</td><td>gi|24650439|</td><td>gi|42656316|ref|XP_379098.1|</td><td>gi|6705463|ref|Os01g55380|</td></tr>
<tr><td>gi|31208979|ref|XP_313456.1|</td><td>lcl|At3g30370.1</td><td>lcl|F11A5.15 CE34801 WBGene</td><td>gi|24650457|</td><td>gi|42656321|ref|XP_379099.1|</td><td>gi|6705465|ref|Os01g55400|</td></tr>
<tr><td>gi|31208983|ref|XP_313458.1|</td><td>lcl|At3g30410.1</td><td>lcl|F11A5.6 CE15784 WBGene0</td><td>gi|24650459|</td><td>gi|42656325|ref|XP_379100.1|</td><td>gi|6705471|ref|Os01g55460|</td></tr>
<tr><td>gi|31209069|ref|XP_313501.1|</td><td>lcl|At3g30430.1</td><td>lcl|F11C1.2 CE31472 WBGene0</td><td>gi|24650461|</td><td>gi|42656328|ref|XP_379101.1|</td><td>gi|6705499|ref|Os01g55720|</td></tr>
<tr><td>gi|31209071|ref|XP_313502.1|</td><td>lcl|At3g30480.1</td><td>lcl|F11C7.5 CE17657 WBGene0</td><td>gi|24650463|</td><td>gi|42656334|ref|XP_379102.1|</td><td>gi|6705505|ref|Os01g55760|</td></tr>
<tr><td>gi|31209073|ref|XP_313503.1|</td><td>lcl|At3g30490.1</td><td>lcl|F11D11.10 CE15801 WBGen</td><td>gi|24650497|</td><td>gi|42656346|ref|XP_379106.1|</td><td>gi|6705507|ref|Os01g55790|</td></tr>
<tr><td>gi|31209095|ref|XP_313514.1|</td><td>lcl|At3g30510.1</td><td>lcl|F11D5.1a CE27948 WBGene</td><td>gi|24650545|</td><td>gi|42656350|ref|XP_376072.1|</td><td>gi|6705515|ref|Os01g55840|</td></tr>
<tr><td>gi|31209097|ref|XP_313515.1|</td><td>lcl|At3g30520.1</td><td>lcl|F11D5.1b CE27949 WBGene</td><td>gi|24650555|</td><td>gi|42656354|ref|XP_376074.1|</td><td>gi|6705516|ref|Os01g55850|</td></tr>
<tr><td>gi|31209099|ref|XP_313516.1|</td><td>lcl|At3g30580.1</td><td>lcl|F11D5.6 CE35463 WBGene0</td><td>gi|24650602|</td><td>gi|42656360|ref|XP_379108.1|</td><td>gi|6705531|ref|Os01g55990|</td></tr>
<tr><td>gi|31209105|ref|XP_313519.1|</td><td>lcl|At3g30590.1</td><td>lcl|F11E6.10 CE34802 WBGene</td><td>gi|24650613|</td><td>gi|42656373|ref|XP_379068.1|</td><td>gi|6705534|ref|Os01g56020|</td></tr>
<tr><td>gi|31209107|ref|XP_313520.1|</td><td>lcl|At3g30610.1</td><td>lcl|F11E6.11 CE37345 WBGene</td><td>gi|24650615|</td><td>gi|42656375|ref|XP_379069.1|</td><td>gi|6705538|ref|Os01g56060|</td></tr>
<tr><td>gi|31209109|ref|XP_313521.1|</td><td>lcl|At3g30645.1</td><td>lcl|F11E6.3 CE19783 WBGene0</td><td>gi|24650623|</td><td>gi|42656379|ref|XP_373972.2|</td><td>gi|6705542|ref|Os01g56080|</td></tr>
<tr><td>gi|31209115|ref|XP_313524.1|</td><td>lcl|At3g30650.1</td><td>lcl|F11E6.4 CE31167 WBGene0</td><td>gi|24650628|</td><td>gi|42656381|ref|XP_376094.1|</td><td>gi|6705550|ref|Os01g56160|</td></tr>
<tr><td>gi|31209135|ref|XP_313534.1|</td><td>lcl|At3g30660.1</td><td>lcl|F11E6.6 CE35464 WBGene0</td><td>gi|24650630|</td><td>gi|42656396|ref|XP_379111.1|</td><td>gi|6705551|ref|Os01g56170|</td></tr>
<tr><td>gi|31209169|ref|XP_313551.1|</td><td>lcl|At3g30670.1</td><td>lcl|F11E6.7 CE19787 WBGene0</td><td>gi|24650634|</td><td>gi|42656406|ref|XP_376117.1|</td><td>gi|6705561|ref|Os01g56260|</td></tr>
<tr><td>gi|31209173|ref|XP_313553.1|</td><td>lcl|At3g30690.1</td><td>lcl|F11E6.9 CE31168 WBGene0</td><td>gi|24650636|</td><td>gi|42656408|ref|XP_379121.1|</td><td>gi|6705564|ref|Os01g56290|</td></tr>
<tr><td>gi|31209223|ref|XP_313578.1|</td><td>lcl|At3g30700.1</td><td>lcl|F11G11.10 CE31783 WBGen</td><td>gi|24650638|</td><td>gi|42656412|ref|XP_379109.1|</td><td>gi|6705581|ref|Os01g56440|</td></tr>
<tr><td>gi|31209233|ref|XP_313583.1|</td><td>lcl|At3g30710.1</td><td>lcl|F11G11.4 CE07057 WBGene</td><td>gi|24650640|</td><td>gi|42656417|ref|XP_379169.1|</td><td>gi|6705585|ref|Os01g56480|</td></tr>
<tr><td>gi|31209259|ref|XP_313596.1|</td><td>lcl|At3g30720.1</td><td>lcl|F11G11.5 CE07058 WBGene</td><td>gi|24650678|</td><td>gi|42656419|ref|XP_376195.1|</td><td>gi|6705587|ref|Os01g56500|</td></tr>
<tr><td>gi|31209265|ref|XP_313599.1|</td><td>lcl|At3g30725.1</td><td>lcl|F12A10.1 CE01904 WBGene</td><td>gi|24650686|</td><td>gi|42656423|ref|XP_379136.1|</td><td>gi|6705605|ref|Os01g56650|</td></tr>
<tr><td>gi|31209291|ref|XP_313612.1|</td><td>lcl|At3g30750.1</td><td>lcl|F12A10.2 CE01905 WBGene</td><td>gi|24650689|</td><td>gi|42656451|ref|XP_379141.1|</td><td>gi|6705606|ref|Os01g56660|</td></tr>
<tr><td>gi|31209301|ref|XP_313617.1|</td><td>lcl|At3g30751.1</td><td>lcl|F12A10.6 CE01909 WBGene</td><td>gi|24650691|</td><td>gi|42656460|ref|XP_379131.1|</td><td>gi|6705608|ref|Os01g56680|</td></tr>
<tr><td>gi|31209305|ref|XP_313619.1|</td><td>lcl|At3g30755.1</td><td>lcl|F12A10.7 CE01910 WBGene</td><td>gi|24650693|</td><td>gi|42656462|ref|XP_379133.1|</td><td>gi|6705609|ref|Os01g56690|</td></tr>
<tr><td>gi|31209311|ref|XP_313622.1|</td><td>lcl|At3g30766.1</td><td>lcl|F12A10.8 CE31784 WBGene</td><td>gi|24650695|</td><td>gi|42656471|ref|XP_379135.1|</td><td>gi|6705610|ref|Os01g56700|</td></tr>
<tr><td>gi|31209317|ref|XP_313625.1|</td><td>lcl|At3g30770.1</td><td>lcl|F12D9.1a CE30947 WBGene</td><td>gi|24650699|</td><td>gi|42656480|ref|XP_376186.1|</td><td>gi|6705611|ref|Os01g56710|</td></tr>
<tr><td>gi|31209319|ref|XP_313626.1|</td><td>lcl|At3g30816.1</td><td>lcl|F12D9.1b CE02633 WBGene</td><td>gi|24650719|</td><td>gi|42656482|ref|XP_379161.1|</td><td>gi|6705612|ref|Os01g56720|</td></tr>
<tr><td>gi|31209321|ref|XP_313627.1|</td><td>lcl|At3g30820.1</td><td>lcl|F12D9.2 CE02634 WBGene0</td><td>gi|24650721|</td><td>gi|42656484|ref|XP_379163.1|</td><td>gi|6705615|ref|Os01g56750|</td></tr>
<tr><td>gi|31209323|ref|XP_313628.1|</td><td>lcl|At3g30830.1</td><td>lcl|F12E12.1 CE26805 WBGene</td><td>gi|24650733|</td><td>gi|42656488|ref|XP_379164.1|</td><td>gi|6705617|ref|Os01g56770|</td></tr>
<tr><td>gi|31209349|ref|XP_313641.1|</td><td>lcl|At3g30840.1</td><td>lcl|F12E12.2 CE20677 WBGene</td><td>gi|24650737|</td><td>gi|42656490|ref|XP_376189.1|</td><td>gi|6705626|ref|Os01g56850|</td></tr>
<tr><td>gi|31209407|ref|XP_313670.1|</td><td>lcl|At3g30845.1</td><td>lcl|F12E12.3 CE26806 WBGene</td><td>gi|24650773|</td><td>gi|42656492|ref|XP_379072.1|</td><td>gi|6705632|ref|Os01g56900|</td></tr>
<tr><td>gi|31209409|ref|XP_313671.1|</td><td>lcl|At3g30848.1</td><td>lcl|F12E12.6 CE35466 WBGene</td><td>gi|24650780|</td><td>gi|42656494|ref|XP_376031.1|</td><td>gi|6705633|ref|Os01g56900|</td></tr>
<tr><td>gi|31209421|ref|XP_313677.1|</td><td>lcl|At3g31300.1</td><td>lcl|F12F6.1 CE34803 WBGene0</td><td>gi|24650788|</td><td>gi|42656496|ref|XP_376032.1|</td><td>gi|6705635|ref|Os01g56920|</td></tr>
<tr><td>gi|31209435|ref|XP_313684.1|</td><td>lcl|At3g31310.1</td><td>lcl|F12F6.2 CE05590 WBGene0</td><td>gi|24650804|</td><td>gi|42656499|ref|XP_379073.1|</td><td>gi|6705647|ref|Os01g57030|</td></tr>
<tr><td>gi|31209441|ref|XP_313687.1|</td><td>lcl|At3g31320.1</td><td>lcl|F13A2.1 CE09363 WBGene0</td><td>gi|24650826|</td><td>gi|42656501|ref|XP_379074.1|</td><td>gi|6705648|ref|Os01g57040|</td></tr>
<tr><td>gi|31209489|ref|XP_313711.1|</td><td>lcl|At3g31330.1</td><td>lcl|F13A2.2 CE32373 WBGene0</td><td>gi|24650849|</td><td>gi|42656503|ref|XP_376033.1|</td><td>gi|6705651|ref|Os01g57070|</td></tr>
<tr><td>gi|31209499|ref|XP_313716.1|</td><td>lcl|At3g31350.1</td><td>lcl|F13A2.3 CE09365 WBGene0</td><td>gi|24650866|</td><td>gi|42656505|ref|XP_379075.1|</td><td>gi|6705652|ref|Os01g57080|</td></tr>
<tr><td>gi|31209517|ref|XP_313725.1|</td><td>lcl|At3g31370.1</td><td>lcl|F13A2.4 CE09366 WBGene0</td><td>gi|24650895|</td><td>gi|42656514|ref|XP_379077.1|</td><td>gi|6705660|ref|Os01g57140|</td></tr>
<tr><td>gi|31209527|ref|XP_313730.1|</td><td>lcl|At3g31400.1</td><td>lcl|F13A2.5 CE09367 WBGene0</td><td>gi|24650902|</td><td>gi|42656516|ref|XP_377728.1|</td><td>gi|6705661|ref|Os01g57150|</td></tr>
<tr><td>gi|31209529|ref|XP_313731.1|</td><td>lcl|At3g31406.1</td><td>lcl|F13A7.11 CE15816 WBGene</td><td>gi|24650904|</td><td>gi|42656518|ref|XP_378191.1|</td><td>gi|6705663|ref|Os01g57170|</td></tr>
<tr><td>gi|31209533|ref|XP_313733.1|</td><td>lcl|At3g31410.1</td><td>lcl|F13B12.3 CE35467 WBGene</td><td>gi|24650906|</td><td>gi|42656534|ref|XP_379145.1|</td><td>gi|6705664|ref|Os01g57180|</td></tr>
<tr><td>gi|31209535|ref|XP_313734.1|</td><td>lcl|At3g31460.1</td><td>lcl|F13B12.7 CE35468 WBGene</td><td>gi|24650979|</td><td>gi|42656536|ref|XP_379146.1|</td><td>gi|6705665|ref|Os01g57190|</td></tr>
<tr><td>gi|31209537|ref|XP_313735.1|</td><td>lcl|At3g31540.1</td><td>lcl|F13B6.1 CE16973 WBGene0</td><td>gi|24650999|</td><td>gi|42656538|ref|XP_379147.1|</td><td>gi|6705666|ref|Os01g57200|</td></tr>
<tr><td>gi|31209541|ref|XP_313737.1|</td><td>lcl|At3g31910.1</td><td>lcl|F13B6.2 CE16974 WBGene0</td><td>gi|24651085|</td><td>gi|42656575|ref|XP_379154.1|</td><td>gi|6705673|ref|Os01g57250|</td></tr>
<tr><td>gi|31209553|ref|XP_313743.1|</td><td>lcl|At3g31915.1</td><td>lcl|F13B6.3 CE31169 WBGene0</td><td>gi|24651104|</td><td>gi|42656582|ref|XP_379156.1|</td><td>gi|6705678|ref|Os01g57300|</td></tr>
<tr><td>gi|31209557|ref|XP_313745.1|</td><td>lcl|At3g31940.1</td><td>lcl|F13B9.1a CE37100 WBGene</td><td>gi|24651106|</td><td>gi|42656590|ref|XP_379158.1|</td><td>gi|6705681|ref|Os01g57330|</td></tr>
<tr><td>gi|31209559|ref|XP_313746.1|</td><td>lcl|At3g31955.1</td><td>lcl|F13B9.1b CE30949 WBGene</td><td>gi|24651111|</td><td>gi|42656592|ref|XP_379159.1|</td><td>gi|6705686|ref|Os01g57380|</td></tr>
<tr><td>gi|31209589|ref|XP_313761.1|</td><td>lcl|At3g32020.1</td><td>lcl|F13B9.1c CE35469 WBGene</td><td>gi|24651133|</td><td>gi|42656603|ref|XP_377778.1|</td><td>gi|6705687|ref|Os01g57390|</td></tr>
<tr><td>gi|31209601|ref|XP_313767.1|</td><td>lcl|At3g32050.1</td><td>lcl|F13B9.2 CE04379 WBGene0</td><td>gi|24651173|</td><td>gi|42656613|ref|XP_379167.1|</td><td>gi|6705692|ref|Os01g57430|</td></tr>
<tr><td>gi|31209625|ref|XP_313779.1|</td><td>lcl|At3g32070.1</td><td>lcl|F13C5.3 CE35470 WBGene0</td><td>gi|24651177|</td><td>gi|42656620|ref|XP_379168.1|</td><td>gi|6705699|ref|Os01g57500|</td></tr>
<tr><td>gi|31209627|ref|XP_313780.1|</td><td>lcl|At3g32080.1</td><td>lcl|F13C5.4 CE19386 WBGene0</td><td>gi|24651209|</td><td>gi|42656628|ref|XP_376118.1|</td><td>gi|6705701|ref|Os01g57520|</td></tr>
<tr><td>gi|31209649|ref|XP_313791.1|</td><td>lcl|At3g32090.1</td><td>lcl|F13C5.5 CE19387 WBGene0</td><td>gi|24651253|</td><td>gi|42656632|ref|XP_379170.1|</td><td>gi|6705702|ref|Os01g57530|</td></tr>
<tr><td>gi|31209665|ref|XP_313799.1|</td><td>lcl|At3g32100.1</td><td>lcl|F13C5.6a CE27424 WBGene</td><td>gi|24651295|</td><td>gi|42656636|ref|XP_376243.1|</td><td>gi|6705710|ref|Os01g57590|</td></tr>
<tr><td>gi|31209695|ref|XP_313814.1|</td><td>lcl|At3g32120.1</td><td>lcl|F13C5.6b CE29760 WBGene</td><td>gi|24651297|</td><td>gi|42656638|ref|XP_379197.1|</td><td>gi|6705715|ref|Os01g57640|</td></tr>
<tr><td>gi|31209699|ref|XP_313816.1|</td><td>lcl|At3g32140.1</td><td>lcl|F13D12.10 CE32374 WBGen</td><td>gi|24651326|</td><td>gi|42656640|ref|XP_379198.1|</td><td>gi|6705717|ref|Os01g57660|</td></tr>
<tr><td>gi|31209731|ref|XP_313832.1|</td><td>lcl|At3g32150.1</td><td>lcl|F13D12.3 CE03197 WBGene</td><td>gi|24651328|</td><td>gi|42656665|ref|XP_379172.1|</td><td>gi|6705719|ref|Os01g57680|</td></tr>
<tr><td>gi|31209745|ref|XP_313839.1|</td><td>lcl|At3g32160.1</td><td>lcl|F13D12.5 CE02184 WBGene</td><td>gi|24651371|</td><td>gi|42656667|ref|XP_379173.1|</td><td>gi|6705726|ref|Os01g57750|</td></tr>
<tr><td>gi|31209757|ref|XP_313845.1|</td><td>lcl|At3g32180.1</td><td>lcl|F13D12.8 CE20682 WBGene</td><td>gi|24651375|</td><td>gi|42656671|ref|XP_379175.1|</td><td>gi|6705735|ref|Os01g57840|</td></tr>
<tr><td>gi|31209769|ref|XP_313851.1|</td><td>lcl|At3g32190.1</td><td>lcl|F13D2.1 CE37347 WBGene0</td><td>gi|24651428|</td><td>gi|42656676|ref|XP_379177.1|</td><td>gi|6705762|ref|Os01g58090|</td></tr>
<tr><td>gi|31209793|ref|XP_313863.1|</td><td>lcl|At3g32200.1</td><td>lcl|F13D2.4 CE03196 WBGene0</td><td>gi|24651473|</td><td>gi|42656681|ref|XP_376206.1|</td><td>gi|6705766|ref|Os01g58130|</td></tr>
<tr><td>gi|31209829|ref|XP_313881.1|</td><td>lcl|At3g32220.1</td><td>lcl|F13E9.10 CE05612 WBGene</td><td>gi|24651479|</td><td>gi|42656687|ref|XP_379179.1|</td><td>gi|6705767|ref|Os01g58140|</td></tr>
<tr><td>gi|31209831|ref|XP_313882.1|</td><td>lcl|At3g32300.1</td><td>lcl|F13E9.11 CE35471 WBGene</td><td>gi|24651489|</td><td>gi|42656697|ref|XP_376212.1|</td><td>gi|6705768|ref|Os01g58150|</td></tr>
<tr><td>gi|31209833|ref|XP_313883.1|</td><td>lcl|At3g32394.1</td><td>lcl|F13E9.12 CE30657 WBGene</td><td>gi|24651515|</td><td>gi|42656699|ref|XP_379180.1|</td><td>gi|6705769|ref|Os01g58160|</td></tr>
<tr><td>gi|31209859|ref|XP_313896.1|</td><td>lcl|At3g32896.1</td><td>lcl|F13E9.2 CE05604 WBGene0</td><td>gi|24651524|</td><td>gi|42656701|ref|XP_379181.1|</td><td>gi|6705770|ref|Os01g58170|</td></tr>
<tr><td>gi|31209875|ref|XP_313904.1|</td><td>lcl|At3g32902.1</td><td>lcl|F13E9.5 CE35472 WBGene0</td><td>gi|24651526|</td><td>gi|42656703|ref|XP_379182.1|</td><td>gi|6705776|ref|Os01g58210|</td></tr>
<tr><td>gi|31209883|ref|XP_313908.1|</td><td>lcl|At3g32903.1</td><td>lcl|F13E9.6 CE05608 WBGene0</td><td>gi|24651534|</td><td>gi|42656705|ref|XP_379183.1|</td><td>gi|6705777|ref|Os01g58230|</td></tr>
<tr><td>gi|31209889|ref|XP_313911.1|</td><td>lcl|At3g32904.1</td><td>lcl|F13E9.7 CE05609 WBGene0</td><td>gi|24651539|</td><td>gi|42656707|ref|XP_379184.1|</td><td>gi|6705779|ref|Os01g58250|</td></tr>
<tr><td>gi|31209899|ref|XP_313916.1|</td><td>lcl|At3g32910.1</td><td>lcl|F13E9.9 CE35473 WBGene0</td><td>gi|24651541|</td><td>gi|42656720|ref|XP_379189.1|</td><td>gi|6705784|ref|Os01g58300|</td></tr>
<tr><td>gi|31209929|ref|XP_313931.1|</td><td>lcl|At3g32960.1</td><td>lcl|F13G3.10 CE17660 WBGene</td><td>gi|24651587|</td><td>gi|42656725|ref|XP_379190.1|</td><td>gi|6705785|ref|Os01g58310|</td></tr>
<tr><td>gi|31209933|ref|XP_313933.1|</td><td>lcl|At3g33064.1</td><td>lcl|F13G3.12 CE34806 WBGene</td><td>gi|24651610|</td><td>gi|42656731|ref|XP_376227.1|</td><td>gi|6705786|ref|Os01g58320|</td></tr>
<tr><td>gi|31209975|ref|XP_313954.1|</td><td>lcl|At3g33073.1</td><td>lcl|F13H10.1 CE05622 WBGene</td><td>gi|24651629|</td><td>gi|42656735|ref|XP_379192.1|</td><td>gi|6705787|ref|Os01g58330|</td></tr>
<tr><td>gi|31209977|ref|XP_313955.1|</td><td>lcl|At3g33080.1</td><td>lcl|F13H10.6 CE30343 WBGene</td><td>gi|24651651|</td><td>gi|42656741|ref|XP_379194.1|</td><td>gi|6705788|ref|Os01g58340|</td></tr>
<tr><td>gi|31209991|ref|XP_313962.1|</td><td>lcl|At3g33131.1</td><td>lcl|F13H8.1a CE02635 WBGene</td><td>gi|24651731|</td><td>gi|42656745|ref|XP_376239.1|</td><td>gi|6705789|ref|Os01g58350|</td></tr>
<tr><td>gi|31209993|ref|XP_313963.1|</td><td>lcl|At3g33230.1</td><td>lcl|F13H8.1b CE37349 WBGene</td><td>gi|24651733|</td><td>gi|42656748|ref|XP_379195.1|</td><td>gi|6705791|ref|Os01g58370|</td></tr>
<tr><td>gi|31210007|ref|XP_313970.1|</td><td>lcl|At3g33293.1</td><td>lcl|F13H8.6 CE02640 WBGene0</td><td>gi|24651735|</td><td>gi|42656750|ref|XP_376241.1|</td><td>gi|6705796|ref|Os01g58410|</td></tr>
<tr><td>gi|31210031|ref|XP_313982.1|</td><td>lcl|At3g33393.1</td><td>lcl|F13H8.8 CE35477 WBGene0</td><td>gi|24651763|</td><td>gi|42656752|ref|XP_379196.1|</td><td>gi|6705798|ref|Os01g58430|</td></tr>
<tr><td>gi|31210033|ref|XP_313983.1|</td><td>lcl|At3g33448.1</td><td>lcl|F14B6.1 CE17663 WBGene0</td><td>gi|24651770|</td><td>gi|42656759|ref|XP_379244.1|</td><td>gi|6705799|ref|Os01g58440|</td></tr>
<tr><td>gi|31210035|ref|XP_313984.1|</td><td>lcl|At3g33494.1</td><td>lcl|F14B6.2 CE25855 WBGene0</td><td>gi|24651785|</td><td>gi|42656761|ref|XP_376292.1|</td><td>gi|6705800|ref|Os01g58450|</td></tr>
<tr><td>gi|31210043|ref|XP_313988.1|</td><td>lcl|At3g33572.1</td><td>lcl|F14B6.3 CE17665 WBGene0</td><td>gi|24651799|</td><td>gi|42656767|ref|XP_379247.1|</td><td>gi|6705801|ref|Os01g58460|</td></tr>
<tr><td>gi|31210045|ref|XP_313989.1|</td><td>lcl|At3g42060.1</td><td>lcl|F14B8.2 CE02645 WBGene0</td><td>gi|24651891|</td><td>gi|42656778|ref|XP_379201.1|</td><td>gi|6705803|ref|Os01g58480|</td></tr>
<tr><td>gi|31210051|ref|XP_313992.1|</td><td>lcl|At3g42070.1</td><td>lcl|F14B8.4 CE02647 WBGene0</td><td>gi|24651926|</td><td>gi|42656780|ref|XP_376256.1|</td><td>gi|6705804|ref|Os01g58490|</td></tr>
<tr><td>gi|31210053|ref|XP_313993.1|</td><td>lcl|At3g42080.1</td><td>lcl|F14B8.5b CE29763 WBGene</td><td>gi|24651935|</td><td>gi|42656786|ref|XP_298151.3|</td><td>gi|6705809|ref|Os01g58540|</td></tr>
<tr><td>gi|31210059|ref|XP_313996.1|</td><td>lcl|At3g42090.1</td><td>lcl|F14B8.6 CE02649 WBGene0</td><td>gi|24651939|</td><td>gi|42656788|ref|XP_379205.1|</td><td>gi|6705814|ref|Os01g58590|</td></tr>
<tr><td>gi|31210115|ref|XP_314024.1|</td><td>lcl|At3g42120.1</td><td>lcl|F14D12.1a CE29382 WBGen</td><td>gi|24651944|</td><td>gi|42656795|ref|XP_376257.1|</td><td>gi|6705815|ref|Os01g58600|</td></tr>
<tr><td>gi|31210125|ref|XP_314029.1|</td><td>lcl|At3g42130.1</td><td>lcl|F14D12.1b CE29269 WBGen</td><td>gi|24651950|</td><td>gi|42656800|ref|XP_379207.1|</td><td>gi|6705832|ref|Os01g58770|</td></tr>
<tr><td>gi|31210131|ref|XP_314032.1|</td><td>lcl|At3g42140.1</td><td>lcl|F14D2.10 CE16985 WBGene</td><td>gi|24651963|</td><td>gi|42656811|ref|XP_379210.1|</td><td>gi|6705835|ref|Os01g58800|</td></tr>
<tr><td>gi|31210133|ref|XP_314033.1|</td><td>lcl|At3g42155.1</td><td>lcl|F14D2.14 CE35480 WBGene</td><td>gi|24652045|</td><td>gi|42656820|ref|XP_379213.1|</td><td>gi|6705837|ref|Os01g58820|</td></tr>
<tr><td>gi|31210135|ref|XP_314034.1|</td><td>lcl|At3g42200.1</td><td>lcl|F14D2.5 CE16980 WBGene0</td><td>gi|24652055|</td><td>gi|42656827|ref|XP_379214.1|</td><td>gi|6705839|ref|Os01g58840|</td></tr>
<tr><td>gi|31210137|ref|XP_314035.1|</td><td>lcl|At3g42210.1</td><td>lcl|F14D7.10 CE34807 WBGene</td><td>gi|24652078|</td><td>gi|42656832|ref|XP_376268.1|</td><td>gi|6705843|ref|Os01g58880|</td></tr>
<tr><td>gi|31210145|ref|XP_314039.1|</td><td>lcl|At3g42240.1</td><td>lcl|F14D7.11 CE34808 WBGene</td><td>gi|24652094|</td><td>gi|42656844|ref|XP_376269.1|</td><td>gi|6705845|ref|Os01g58900|</td></tr>
<tr><td>gi|31210155|ref|XP_314044.1|</td><td>lcl|At3g42254.1</td><td>lcl|F14D7.2 CE09380 WBGene0</td><td>gi|24652096|</td><td>gi|42656847|ref|XP_295096.3|</td><td>gi|6705848|ref|Os01g58930|</td></tr>
<tr><td>gi|31210159|ref|XP_314046.1|</td><td>lcl|At3g42300.1</td><td>lcl|F14D7.3 CE09381 WBGene0</td><td>gi|24652101|</td><td>gi|42656855|ref|XP_379228.1|</td><td>gi|6705849|ref|Os01g58940|</td></tr>
<tr><td>gi|31210161|ref|XP_314047.1|</td><td>lcl|At3g42310.1</td><td>lcl|F14D7.5 CE09383 WBGene0</td><td>gi|24652107|</td><td>gi|42656859|ref|XP_379229.1|</td><td>gi|6705853|ref|Os01g58980|</td></tr>
<tr><td>gi|31210167|ref|XP_314050.1|</td><td>lcl|At3g42330.1</td><td>lcl|F14D7.7 CE09385 WBGene0</td><td>gi|24652138|</td><td>gi|42656863|ref|XP_379230.1|</td><td>gi|6705856|ref|Os01g59010|</td></tr>
<tr><td>gi|31210175|ref|XP_314054.1|</td><td>lcl|At3g42350.1</td><td>lcl|F14D7.8 CE09386 WBGene0</td><td>gi|24652169|</td><td>gi|42656869|ref|XP_379231.1|</td><td>gi|6705858|ref|Os01g59030|</td></tr>
<tr><td>gi|31210179|ref|XP_314056.1|</td><td>lcl|At3g42380.1</td><td>lcl|F14D7.9 CE36481 WBGene0</td><td>gi|24652186|</td><td>gi|42656873|ref|XP_379233.1|</td><td>gi|6705874|ref|Os01g59170|</td></tr>
<tr><td>gi|31210183|ref|XP_314058.1|</td><td>lcl|At3g42390.1</td><td>lcl|F14F11.2 CE03210 WBGene</td><td>gi|24652188|</td><td>gi|42656882|ref|XP_379234.1|</td><td>gi|6705876|ref|Os01g59190|</td></tr>
<tr><td>gi|31210205|ref|XP_314069.1|</td><td>lcl|At3g42430.1</td><td>lcl|F14F3.4 CE34811 WBGene0</td><td>gi|24652190|</td><td>gi|42656884|ref|XP_379235.1|</td><td>gi|6705881|ref|Os01g59240|</td></tr>
<tr><td>gi|31210207|ref|XP_314070.1|</td><td>lcl|At3g42436.1</td><td>lcl|F14F7.4 CE28822 WBGene0</td><td>gi|24652192|</td><td>gi|42656891|ref|XP_379240.1|</td><td>gi|6705882|ref|Os01g59250|</td></tr>
<tr><td>gi|31210223|ref|XP_314078.1|</td><td>lcl|At3g42450.1</td><td>lcl|F14F8.4 CE37503 WBGene0</td><td>gi|24652201|</td><td>gi|42656893|ref|XP_376290.1|</td><td>gi|6705884|ref|Os01g59270|</td></tr>
<tr><td>gi|31210233|ref|XP_314083.1|</td><td>lcl|At3g42480.1</td><td>lcl|F14F8.8 CE18592 WBGene0</td><td>gi|24652242|</td><td>gi|42656921|ref|XP_379263.1|</td><td>gi|6705885|ref|Os01g59280|</td></tr>
<tr><td>gi|31210261|ref|XP_314097.1|</td><td>lcl|At3g42490.1</td><td>lcl|F14F9.2 CE09388 WBGene0</td><td>gi|24652246|</td><td>gi|42656923|ref|XP_379264.1|</td><td>gi|6705886|ref|Os01g59290|</td></tr>
<tr><td>gi|31210313|ref|XP_314123.1|</td><td>lcl|At3g42510.1</td><td>lcl|F14F9.3 CE09389 WBGene0</td><td>gi|24652248|</td><td>gi|42656932|ref|XP_379271.1|</td><td>gi|6705887|ref|Os01g59300|</td></tr>
<tr><td>gi|31210337|ref|XP_314135.1|</td><td>lcl|At3g42520.1</td><td>lcl|F14F9.4 CE09390 WBGene0</td><td>gi|24652371|</td><td>gi|42656945|ref|XP_379273.1|</td><td>gi|6705888|ref|Os01g59310|</td></tr>
<tr><td>gi|31210345|ref|XP_314139.1|</td><td>lcl|At3g42530.1</td><td>lcl|F14F9.6 CE36823 WBGene0</td><td>gi|24652375|</td><td>gi|42656949|ref|XP_379274.1|</td><td>gi|6705889|ref|Os01g59320|</td></tr>
<tr><td>gi|31210355|ref|XP_314144.1|</td><td>lcl|At3g42540.1</td><td>lcl|F14H12.2 CE07062 WBGene</td><td>gi|24652381|</td><td>gi|42656953|ref|XP_376339.1|</td><td>gi|6705896|ref|Os01g59370|</td></tr>
<tr><td>gi|31210387|ref|XP_314160.1|</td><td>lcl|At3g42556.1</td><td>lcl|F14H12.6 CE25857 WBGene</td><td>gi|24652406|</td><td>gi|42656956|ref|XP_371705.2|</td><td>gi|6705898|ref|Os01g59390|</td></tr>
</table>

gi|31210415|ref|XP_314174.1|	lcl|At3g42560.1	lcl|F14H12.7 CE07066 WBGene	gi|24652413|	gi|42656958|ref|XP_379275.1|	gi|6705899|ref|Os01g59400|
gi|31210427|ref|XP_314180.1|	lcl|At3g42590.1	lcl|F14H12.8 CE07067 WBGene	gi|24652419|	gi|42656965|ref|XP_379276.1|	gi|6705901|ref|Os01g59420|
gi|31210429|ref|XP_314181.1|	lcl|At3g42600.1	lcl|F14H3.3 CE23651 WBGene0	gi|24652421|	gi|42656978|ref|XP_379278.1|	gi|6705902|ref|Os01g59430|
gi|31210441|ref|XP_314187.1|	lcl|At3g42610.1	lcl|F14H3.4 CE15829 WBGene0	gi|24652423|	gi|42656980|ref|XP_376347.1|	gi|6705906|ref|Os01g59460|
gi|31210447|ref|XP_314190.1|	lcl|At3g42680.1	lcl|F14H3.5 CE15830 WBGene0	gi|24652425|	gi|42656983|ref|XP_379280.1|	gi|6705907|ref|Os01g59470|
gi|31210479|ref|XP_314206.1|	lcl|At3g42700.1	lcl|F14H3.6 CE23652 WBGene0	gi|24652429|	gi|42656992|ref|XP_379287.1|	gi|6705908|ref|Os01g59480|
gi|31210489|ref|XP_314211.1|	lcl|At3g42710.1	lcl|F14H3.9 CE15833 WBGene0	gi|24652467|	gi|42656994|ref|XP_379288.1|	gi|6705926|ref|Os01g59650|
gi|31210527|ref|XP_314230.1|	lcl|At3g42723.1	lcl|F14H8.2 CE09394 WBGene0	gi|24652518|	gi|42657006|ref|XP_379267.1|	gi|6705933|ref|Os01g59700|
gi|31210539|ref|XP_314236.1|	lcl|At3g42725.1	lcl|F14H8.4 CE09395 WBGene0	gi|24652569|	gi|42657008|ref|XP_379268.1|	gi|6705934|ref|Os01g59710|
gi|31210543|ref|XP_314238.1|	lcl|At3g42740.1	lcl|F14H8.5 CE09396 WBGene0	gi|24652578|	gi|42657023|ref|XP_374099.2|	gi|6705935|ref|Os01g59720|
gi|31210557|ref|XP_314245.1|	lcl|At3g42750.1	lcl|F15A2.7 CE05636 WBGene0	gi|24652592|	gi|42657025|ref|XP_379294.1|	gi|6705940|ref|Os01g59770|
gi|31210561|ref|XP_314247.1|	lcl|At3g42780.1	lcl|F15A4.11 CE23655 WBGene	gi|24652596|	gi|42657028|ref|XP_379295.1|	gi|6705955|ref|Os01g59900|
gi|31210585|ref|XP_314259.1|	lcl|At3g42786.1	lcl|F15A4.12 CE23656 WBGene	gi|24652606|	gi|42657030|ref|XP_379298.1|	gi|6705962|ref|Os01g59940|
gi|31210621|ref|XP_314277.1|	lcl|At3g42792.1	lcl|F15A4.5 CE23657 WBGene0	gi|24652609|	gi|42657033|ref|XP_379299.1|	gi|6705964|ref|Os01g59960|
gi|31210625|ref|XP_314279.1|	lcl|At3g42800.1	lcl|F15A8.1 CE09400 WBGene0	gi|24652626|	gi|42657037|ref|XP_374080.2|	gi|6705965|ref|Os01g59970|
gi|31210665|ref|XP_314299.1|	lcl|At3g42810.1	lcl|F15A8.4 CE07068 WBGene0	gi|24652628|	gi|42657039|ref|XP_376318.1|	gi|6705971|ref|Os01g60030|
gi|31210671|ref|XP_314302.1|	lcl|At3g42843.1	lcl|F15B10.3 CE16989 WBGene	gi|24652630|	gi|42657046|ref|XP_379249.1|	gi|6705981|ref|Os01g60130|
gi|31210673|ref|XP_314303.1|	lcl|At3g42870.1	lcl|F15B9.6 CE09411 WBGene0	gi|24652634|	gi|42657048|ref|XP_376299.1|	gi|6705983|ref|Os01g60150|
gi|31210709|ref|XP_314321.1|	lcl|At3g42920.1	lcl|F15D3.1b CE24904 WBGene	gi|24652636|	gi|42657059|ref|XP_378193.1|	gi|6705984|ref|Os01g60160|
gi|31210711|ref|XP_314322.1|	lcl|At3g42940.1	lcl|F15D3.5 CE15852 WBGene0	gi|24652638|	gi|42657066|ref|XP_379254.1|	gi|6705986|ref|Os01g60180|
gi|31210805|ref|XP_314369.1|	lcl|At3g42970.1	lcl|F15D3.8 CE34479 WBGene0	gi|24652654|	gi|42657068|ref|XP_379255.1|	gi|6706000|ref|Os01g60290|
gi|31210807|ref|XP_314370.1|	lcl|At3g42990.1	lcl|F15D4.2 CE09415 WBGene0	gi|24652676|	gi|42657072|ref|XP_379252.1|	gi|6706001|ref|Os01g60300|
gi|31210809|ref|XP_314371.1|	lcl|At3g43000.1	lcl|F15D4.3 CE37223 WBGene0	gi|24652682|	gi|42657076|ref|XP_376325.1|	gi|6706002|ref|Os01g60310|
gi|31210821|ref|XP_314377.1|	lcl|At3g43010.1	lcl|F15D4.5 CE36131 WBGene0	gi|24652684|	gi|42657080|ref|XP_379265.1|	gi|6706008|ref|Os01g60370|
gi|31210823|ref|XP_314378.1|	lcl|At3g43020.1	lcl|F15D4.6 CE36132 WBGene0	gi|24652686|	gi|42657092|ref|XP_379256.1|	gi|6706010|ref|Os01g60390|
gi|31210827|ref|XP_314380.1|	lcl|At3g43030.1	lcl|F15D4.8 CE34815 WBGene0	gi|24652733|	gi|42657100|ref|XP_376307.1|	gi|6706019|ref|Os01g60460|
gi|31210831|ref|XP_314382.1|	lcl|At3g43060.1	lcl|F15E11.1 CE16999 WBGene	gi|24652768|	gi|42657106|ref|XP_379258.1|	gi|6706020|ref|Os01g60470|
gi|31210847|ref|XP_314390.1|	lcl|At3g43070.1	lcl|F15E11.12 CE35965 WBGen	gi|24652816|	gi|42657108|ref|XP_379260.1|	gi|6706023|ref|Os01g60500|
gi|31210853|ref|XP_314393.1|	lcl|At3g43110.1	lcl|F15E11.13 CE25224 WBGen	gi|24652840|	gi|42657136|ref|XP_379270.1|	gi|6706024|ref|Os01g60510|
gi|31210859|ref|XP_314396.1|	lcl|At3g43140.1	lcl|F15E11.14 CE16999 WBGen	gi|24652884|	gi|42657148|ref|XP_376364.1|	gi|6706026|ref|Os01g60530|
gi|31210877|ref|XP_314405.1|	lcl|At3g43150.1	lcl|F15E11.15a CE35276 WBGe	gi|24652920|	gi|42657150|ref|XP_376365.1|	gi|6706028|ref|Os01g60550|
gi|31210899|ref|XP_314416.1|	lcl|At3g43153.1	lcl|F15E11.15b CE33283 WBGe	gi|24652928|	gi|42657152|ref|XP_376366.1|	gi|6706030|ref|Os01g60570|
gi|31210905|ref|XP_314419.1|	lcl|At3g43160.1	lcl|F15E6.3 CE16992 WBGene0	gi|24652932|	gi|42657154|ref|XP_379303.1|	gi|6706036|ref|Os01g60630|
gi|31210907|ref|XP_314420.1|	lcl|At3g43260.1	lcl|F15E6.4 CE16993 WBGene0	gi|24652934|	gi|42657156|ref|XP_374107.2|	gi|6706041|ref|Os01g60680|
gi|31210913|ref|XP_314423.1|	lcl|At3g43280.1	lcl|F15E6.8 CE16997 WBGene0	gi|24652972|	gi|42657159|ref|XP_379306.1|	gi|6706042|ref|Os01g60690|
gi|31210917|ref|XP_314425.1|	lcl|At3g43290.1	lcl|F15G9.1a CE01549 WBGene	gi|24652987|	gi|42657164|ref|XP_374109.2|	gi|6706044|ref|Os01g60710|
gi|31210929|ref|XP_314431.1|	lcl|At3g43302.1	lcl|F15G9.1b CE23660 WBGene	gi|24652999|	gi|42657166|ref|XP_379309.1|	gi|6706045|ref|Os01g60720|
gi|31210933|ref|XP_314433.1|	lcl|At3g43310.1	lcl|F15G9.1c CE32382 WBGene	gi|24653005|	gi|42657170|ref|XP_295155.3|	gi|6706051|ref|Os01g60750|
gi|31210939|ref|XP_314436.1|	lcl|At3g43320.1	lcl|F15G9.2 CE01550 WBGene0	gi|24653007|	gi|42657173|ref|XP_376370.1|	gi|6706052|ref|Os01g60760|
gi|31210965|ref|XP_314449.1|	lcl|At3g43390.1	lcl|F15G9.3 CE01551 WBGene0	gi|24653042|	gi|42657176|ref|XP_379313.1|	gi|6706056|ref|Os01g60800|
gi|31210969|ref|XP_314451.1|	lcl|At3g43410.1	lcl|F15G9.5 CE23661 WBGene0	gi|24653053|	gi|42657182|ref|XP_374113.2|	gi|6706063|ref|Os01g60870|
gi|31210971|ref|XP_314452.1|	lcl|At3g43420.1	lcl|F15G9.6 CE34816 WBGene0	gi|24653084|	gi|42657184|ref|XP_374114.2|	gi|6706065|ref|Os01g60890|
gi|31210973|ref|XP_314453.1|	lcl|At3g43433.1	lcl|F15H10.10 CE37823 WBGen	gi|24653092|	gi|42657186|ref|XP_379317.1|	gi|6706069|ref|Os01g60930|
gi|31210977|ref|XP_314455.1|	lcl|At3g43450.1	lcl|F15H10.4 CE31908 WBGene	gi|24653104|	gi|42657188|ref|XP_379318.1|	gi|6706070|ref|Os01g60940|
gi|31210981|ref|XP_314457.1|	lcl|At3g43470.1	lcl|F15H10.5 CE37824 WBGene	gi|24653172|	gi|42657190|ref|XP_379320.1|	gi|6706100|ref|Os01g61220|
gi|31210983|ref|XP_314458.1|	lcl|At3g43480.1	lcl|F15H10.6 CE05643 WBGene	gi|24653174|	gi|42657193|ref|XP_379371.1|	gi|6706104|ref|Os01g61260|
gi|31210985|ref|XP_314459.1|	lcl|At3g43500.1	lcl|F15H10.9 CE37825 WBGene	gi|24653178|	gi|42657196|ref|XP_376440.1|	gi|6706106|ref|Os01g61280|
gi|31210989|ref|XP_314461.1|	lcl|At3g43528.1	lcl|F16B12.4 CE34173 WBGene	gi|24653205|	gi|42657198|ref|XP_379372.1|	gi|6706107|ref|Os01g61290|
gi|31211013|ref|XP_314473.1|	lcl|At3g43560.1	lcl|F16B12.6 CE32383 WBGene	gi|24653242|	gi|42657206|ref|XP_379373.1|	gi|6706108|ref|Os01g61300|
gi|31211027|ref|XP_314480.1|	lcl|At3g43580.1	lcl|F16B3.2 CE36927 WBGene0	gi|24653272|	gi|42657208|ref|XP_376443.1|	gi|6706112|ref|Os01g61340|
gi|31211059|ref|XP_314496.1|	lcl|At3g43640.1	lcl|F16B4.5a CE17015 WBGene	gi|24653295|	gi|42657213|ref|XP_379377.1|	gi|6706114|ref|Os01g61360|
gi|31211069|ref|XP_314501.1|	lcl|At3g43650.1	lcl|F16B4.5b CE27968 WBGene	gi|24653322|	gi|42657215|ref|XP_379378.1|	gi|6706115|ref|Os01g61370|
gi|31211083|ref|XP_314508.1|	lcl|At3g43680.1	lcl|F16B4.7 CE17017 WBGene0	gi|24653328|	gi|42657226|ref|XP_379343.1|	gi|6706121|ref|Os01g61430|
gi|31211117|ref|XP_314525.1|	lcl|At3g43682.1	lcl|F16C3.2 CE23665 WBGene0	gi|24653331|	gi|42657234|ref|XP_379347.1|	gi|6706123|ref|Os01g61450|
gi|31211121|ref|XP_314527.1|	lcl|At3g43684.1	lcl|F16C3.3 CE36929 WBGene0	gi|24653333|	gi|42657251|ref|XP_379363.1|	gi|6706130|ref|Os01g61520|
gi|31211129|ref|XP_314531.1|	lcl|At3g43690.1	lcl|F16F9.2 CE30956 WBGene0	gi|24653335|	gi|42657255|ref|XP_379364.1|	gi|6706131|ref|Os01g61530|
gi|31211181|ref|XP_314560.1|	lcl|At3g43760.1	lcl|F16F9.3 CE09442 WBGene0	gi|24653350|	gi|42657257|ref|XP_379366.1|	gi|6706132|ref|Os01g61540|
gi|31211185|ref|XP_314562.1|	lcl|At3g43770.1	lcl|F16G10.5 CE35490 WBGene	gi|24653352|	gi|42657259|ref|XP_379368.1|	gi|6706138|ref|Os01g61600|
gi|31211203|ref|XP_314573.1|	lcl|At3g43780.1	lcl|F16H11.2 CE04397 WBGene	gi|24653358|	gi|42657269|ref|XP_376419.1|	gi|6706157|ref|Os01g61730|
gi|31211221|ref|XP_314583.1|	lcl|At3g43870.1	lcl|F16H6.10 CE27753 WBGene	gi|24653362|	gi|42657285|ref|XP_379355.1|	gi|6706159|ref|Os01g61750|
gi|31211229|ref|XP_314587.1|	lcl|At3g43880.1	lcl|F16H6.6 CE18602 WBGene0	gi|24653376|	gi|42657287|ref|XP_379359.1|	gi|6706161|ref|Os01g61770|
gi|31211233|ref|XP_314589.1|	lcl|At3g43900.1	lcl|F16H6.8 CE18604 WBGene0	gi|24653380|	gi|42657296|ref|XP_376394.1|	gi|6706168|ref|Os01g61840|
gi|31211235|ref|XP_314590.1|	lcl|At3g43910.1	lcl|F16H6.9 CE18605 WBGene0	gi|24653412|	gi|42657298|ref|XP_376395.1|	gi|6706169|ref|Os01g61850|
gi|31211237|ref|XP_314591.1|	lcl|At3g43940.1	lcl|F17A2.1 CE03212 WBGene0	gi|24653414|	gi|42657300|ref|XP_378194.1|	gi|6706171|ref|Os01g61870|
gi|31211241|ref|XP_314593.1|	lcl|At3g43950.1	lcl|F17A2.13 CE33999 WBGene	gi|24653416|	gi|42657302|ref|XP_379334.1|	gi|6706184|ref|Os01g61950|
gi|31211245|ref|XP_314595.1|	lcl|At3g43970.1	lcl|F17A2.4 CE03214 WBGene0	gi|24653418|	gi|42657305|ref|XP_379336.1|	gi|6706196|ref|Os01g62050|
gi|31211247|ref|XP_314596.1|	lcl|At3g44040.1	lcl|F17C11.1 CE31341 WBGene	gi|24653420|	gi|42657315|ref|XP_376387.1|	gi|6706201|ref|Os01g62090|
gi|31211249|ref|XP_314597.1|	lcl|At3g44070.1	lcl|F17C11.11 CE26809 WBGen	gi|24653439|	gi|42657321|ref|XP_379321.1|	gi|6706204|ref|Os01g62110|
gi|31211251|ref|XP_314598.1|	lcl|At3g44115.1	lcl|F17C11.13 CE37352 WBGen	gi|24653446|	gi|42657323|ref|XP_379322.1|	gi|6706205|ref|Os01g62120|
gi|31211253|ref|XP_314599.1|	lcl|At3g44140.1	lcl|F17C11.2 CE05649 WBGene	gi|24653448|	gi|42657325|ref|XP_379323.1|	gi|6706207|ref|Os01g62140|
gi|31211257|ref|XP_314601.1|	lcl|At3g44170.1	lcl|F17C11.4 CE05651 WBGene	gi|24653450|	gi|42657327|ref|XP_379324.1|	gi|6706210|ref|Os01g62170|
gi|31211259|ref|XP_314602.1|	lcl|At3g44210.1	lcl|F17C11.6 CE37353 WBGene	gi|24653463|	gi|42657331|ref|XP_379325.1|	gi|6706211|ref|Os01g62180|
gi|31211261|ref|XP_314603.1|	lcl|At3g44230.1	lcl|F17C8.7 CE35180 WBGene0	gi|24653473|	gi|42657333|ref|XP_379326.1|	gi|6706220|ref|Os01g62270|
gi|31211279|ref|XP_314609.1|	lcl|At3g44235.1	lcl|F17E9.2 CE17023 WBGene0	gi|24653475|	gi|42657335|ref|XP_379327.1|	gi|6706221|ref|Os01g62280|
gi|31211291|ref|XP_314615.1|	lcl|At3g44270.1	lcl|F17E9.3 CE34817 WBGene0	gi|24653530|	gi|42657344|ref|XP_379331.1|	gi|6706229|ref|Os01g62360|
gi|31211293|ref|XP_314616.1|	lcl|At3g44370.1	lcl|F17E9.4 CE27971 WBGene0	gi|24653576|	gi|42657346|ref|XP_379332.1|	gi|6706233|ref|Os01g62400|
gi|31211295|ref|XP_314617.1|	lcl|At3g44430.1	lcl|F17E9.5 CE07072 WBGene0	gi|24653612|	gi|42657348|ref|XP_379333.1|	gi|6706244|ref|Os01g62450|
gi|31211297|ref|XP_314618.1|	lcl|At3g44440.1	lcl|F17H10.2 CE03227 WBGene	gi|24653629|	gi|42657381|ref|XP_374125.2|	gi|6706252|ref|Os01g62520|
gi|31211315|ref|XP_314627.1|	lcl|At3g44470.1	lcl|F17H10.4 CE05658 WBGene	gi|24653631|	gi|42657388|ref|XP_379339.1|	gi|6706253|ref|Os01g62530|
gi|31211317|ref|XP_314628.1|	lcl|At3g44570.1	lcl|F18A1.2 CE27972 WBGene0	gi|24653633|	gi|42657393|ref|XP_379340.1|	gi|6706254|ref|Os01g62540|
gi|31211323|ref|XP_314631.1|	lcl|At3g44580.1	lcl|F18A1.3a CE27973 WBGene	gi|24653651|	gi|42657401|ref|XP_379380.1|	gi|6706255|ref|Os01g62550|
gi|31211327|ref|XP_314633.1|	lcl|At3g44690.1	lcl|F18A1.3b CE27974 WBGene	gi|24653653|	gi|42657404|ref|XP_376453.1|	gi|6706256|ref|Os01g62560|
gi|31211339|ref|XP_314639.1|	lcl|At3g44716.1	lcl|F18A1.3c CE32627 WBGene	gi|24653662|	gi|42657408|ref|XP_379381.1|	gi|6706270|ref|Os01g62680|
gi|31211345|ref|XP_314642.1|	lcl|At3g44755.1	lcl|F18A1.3d CE37001 WBGene	gi|24653669|	gi|42657411|ref|XP_379382.1|	gi|6706271|ref|Os01g62690|
gi|31211355|ref|XP_314647.1|	lcl|At3g44760.1	lcl|F18A1.4a CE27975 WBGene	gi|24653676|	gi|42657413|ref|XP_371205.2|	gi|6706272|ref|Os01g62700|
gi|31211369|ref|XP_314654.1|	lcl|At3g44935.1	lcl|F18A1.4b CE27976 WBGene	gi|24653678|	gi|42657415|ref|XP_376458.1|	gi|6706275|ref|Os01g62730|
gi|31211389|ref|XP_314664.1|	lcl|At3g44940.1	lcl|F18A1.6a CE04406 WBGene	gi|24653680|	gi|42657417|ref|XP_371793.2|	gi|6706280|ref|Os01g62770|
gi|31211405|ref|XP_314672.1|	lcl|At3g44950.1	lcl|F18A1.6b CE27977 WBGene	gi|24653688|	gi|42657429|ref|XP_291144.5|	gi|6706286|ref|Os01g62830|
gi|31211431|ref|XP_314685.1|	lcl|At3g44960.1	lcl|F18A1.7 CE32628 WBGene0	gi|24653690|	gi|42657449|ref|XP_379430.1|	gi|6706298|ref|Os01g62930|
gi|31211433|ref|XP_314686.1|	lcl|At3g44980.1	lcl|F18A1.8 CE04408 WBGene0	gi|24653692|	gi|42657461|ref|XP_379432.1|	gi|6706299|ref|Os01g62940|
gi|31211475|ref|XP_314707.1|	lcl|At3g45110.1	lcl|F18A11.3 CE09453 WBGene	gi|24653711|	gi|42657463|ref|XP_379433.1|	gi|6706301|ref|Os01g62960|
gi|31211479|ref|XP_314709.1|	lcl|At3g45120.1	lcl|F18A11.4 CE20689 WBGene	gi|24653720|	gi|42657468|ref|XP_379434.1|	gi|6706302|ref|Os01g62970|
gi|31211525|ref|XP_314732.1|	lcl|At3g45160.1	lcl|F18A12.2 CE36826 WBGene	gi|24653724|	gi|42657472|ref|XP_379435.1|	gi|6706307|ref|Os01g63020|
gi|31211529|ref|XP_314734.1|	lcl|At3g45200.1	lcl|F18C12.3 CE15869 WBGene	gi|24653755|	gi|42657476|ref|XP_379436.1|	gi|6706312|ref|Os01g63070|
gi|31211535|ref|XP_314737.1|	lcl|At3g45230.1	lcl|F18C12.4 CE34818 WBGene	gi|24653762|	gi|42657478|ref|XP_379437.1|	gi|6706343|ref|Os01g63360|
gi|31211541|ref|XP_314740.1|	lcl|At3g45250.1	lcl|F18C5.10 CE31790 WBGene	gi|24653793|	gi|42657480|ref|XP_379438.1|	gi|6706344|ref|Os01g63370|
gi|31211545|ref|XP_314742.1|	lcl|At3g45320.1	lcl|F18C5.5 CE02654 WBGene0	gi|24653858|	gi|42657487|ref|XP_376541.1|	gi|6706346|ref|Os01g63390|
gi|31211551|ref|XP_314745.1|	lcl|At3g45360.1	lcl|F18C5.8 CE02657 WBGene0	gi|24653862|	gi|42657499|ref|XP_379384.1|	gi|6706351|ref|Os01g63430|
gi|31211561|ref|XP_314750.1|	lcl|At3g45370.1	lcl|F18E2.6 CE34819 WBGene0	gi|24653869|	gi|42657501|ref|XP_379386.1|	gi|6706352|ref|Os01g63440|
gi|31211563|ref|XP_314751.1|	lcl|At3g45380.1	lcl|F18E3.11 CE36537 WBGene	gi|24653871|	gi|42657503|ref|XP_379391.1|	gi|6706353|ref|Os01g63450|
gi|31211569|ref|XP_314754.1|	lcl|At3g45443.1	lcl|F18E3.7b CE32864 WBGene	gi|24653873|	gi|42657505|ref|XP_379392.1|	gi|6706357|ref|Os01g63490|
gi|31211579|ref|XP_314759.1|	lcl|At3g45500.1	lcl|F18E9.1 CE02658 WBGene0	gi|24653875|	gi|42657509|ref|XP_376463.1|	gi|6706358|ref|Os01g63500|
gi|31211609|ref|XP_314774.1|	lcl|At3g45730.1	lcl|F18E9.2 CE02659 WBGene0	gi|24653890|	gi|42657511|ref|XP_379393.1|	gi|6706364|ref|Os01g63560|
gi|31211611|ref|XP_314775.1|	lcl|At3g45755.1	lcl|F18E9.3 CE35494 WBGene0	gi|24653897|	gi|42657521|ref|XP_379424.1|	gi|6706365|ref|Os01g63570|
gi|31211613|ref|XP_314776.1|	lcl|At3g45820.1	lcl|F18E9.4 CE02661 WBGene0	gi|24653899|	gi|42657532|ref|XP_376555.1|	gi|6706367|ref|Os01g63590|
gi|31211631|ref|XP_314785.1|	lcl|At3g45910.1	lcl|F18E9.7 CE28823 WBGene0	gi|24653929|	gi|42657534|ref|XP_379456.1|	gi|6706368|ref|Os01g63600|
gi|31211633|ref|XP_314786.1|	lcl|At3g46150.1	lcl|F18F11.2 CE17685 WBGene	gi|24653935|	gi|42657537|ref|XP_379458.1|	gi|6706375|ref|Os01g63670|
gi|31211635|ref|XP_314787.1|	lcl|At3g46300.1	lcl|F18G5.1 CE04409 WBGene0	gi|24653940|	gi|42657541|ref|XP_376557.1|	gi|6706378|ref|Os01g63700|
gi|31211651|ref|XP_314795.1|	lcl|At3g46310.1	lcl|F18G5.2 CE04410 WBGene0	gi|24653982|	gi|42657543|ref|XP_376549.1|	gi|6706383|ref|Os01g63720|
gi|31211653|ref|XP_314796.1|	lcl|At3g46360.1	lcl|F18G5.5 CE04413 WBGene0	gi|24654035|	gi|42657551|ref|XP_379450.1|	gi|6706384|ref|Os01g63730|
gi|31211659|ref|XP_314799.1|	lcl|At3g46380.1	lcl|F18G5.6 CE04414 WBGene0	gi|24654055|	gi|42657554|ref|XP_379452.1|	gi|6706386|ref|Os01g63750|
gi|31211663|ref|XP_314801.1|	lcl|At3g46390.1	lcl|F18H3.4 CE23671 WBGene0	gi|24654090|	gi|42657556|ref|XP_379453.1|	gi|6706391|ref|Os01g63790|
gi|31211667|ref|XP_314803.1|	lcl|At3g46750.1	lcl|F19B10.1 CE09468 WBGene	gi|24654099|	gi|42657560|ref|XP_379454.1|	gi|6706398|ref|Os01g63860|
gi|31211687|ref|XP_314813.1|	lcl|At3g46770.1	lcl|F19B10.10 CE09477 WBGen	gi|24654101|	gi|42657564|ref|XP_087928.4|	gi|6706400|ref|Os01g63880|
gi|31211691|ref|XP_314815.1|	lcl|At3g46880.1	lcl|F19B10.11 CE09478 WBGen	gi|24654107|	gi|42657566|ref|XP_379459.1|	gi|6706404|ref|Os01g63920|
gi|31211695|ref|XP_314817.1|	lcl|At3g47070.1	lcl|F19B10.2 CE32630 WBGene	gi|24654109|	gi|42657568|ref|XP_379460.1|	gi|6706416|ref|Os01g64040|
gi|31211697|ref|XP_314818.1|	lcl|At3g47100.1	lcl|F19B10.3 CE09470 WBGene	gi|24654116|	gi|42657573|ref|XP_379395.1|	gi|6706425|ref|Os01g64130|
gi|31211701|ref|XP_314820.1|	lcl|At3g47230.1	lcl|F19B10.4 CE09471 WBGene	gi|24654122|	gi|42657581|ref|XP_379398.1|	gi|6706430|ref|Os01g64180|
gi|31211705|ref|XP_314822.1|	lcl|At3g47240.1	lcl|F19B10.5 CE09472 WBGene	gi|24654150|	gi|42657583|ref|XP_376471.1|	gi|6706433|ref|Os01g64210|
gi|31211707|ref|XP_314823.1|	lcl|At3g47295.1	lcl|F19B10.6 CE09473 WBGene	gi|24654152|	gi|42657590|ref|XP_376474.1|	gi|6706435|ref|Os01g64230|
gi|31211737|ref|XP_314838.1|	lcl|At3g47320.1	lcl|F19B2.5 CE20697 WBGene0	gi|24654154|	gi|42657594|ref|XP_379401.1|	gi|6706436|ref|Os01g64240|
gi|31211767|ref|XP_314853.1|	lcl|At3g47410.1	lcl|F19B2.7 CE20699 WBGene0	gi|24654167|	gi|42657613|ref|XP_376480.1|	gi|6706441|ref|Os01g64290|
gi|31211777|ref|XP_314858.1|	lcl|At3g47510.1	lcl|F19B6.3 CE05668 WBGene0	gi|24654366|	gi|42657619|ref|XP_376486.1|	gi|6706444|ref|Os01g64320|
gi|31211795|ref|XP_314869.1|	lcl|At3g47836.1	lcl|F19C6.5 CE01558 WBGene0	gi|24654376|	gi|42657623|ref|XP_376488.1|	gi|6706445|ref|Os01g64330|
gi|31211801|ref|XP_314873.1|	lcl|At3g47920.1	lcl|F19C7.5 CE04419 WBGene0	gi|24654378|	gi|42657625|ref|XP_376491.1|	gi|6706446|ref|Os01g64340|
gi|31211843|ref|XP_314905.1|	lcl|At3g47970.1	lcl|F19C7.6 CE04420 WBGene0	gi|24654428|	gi|42657627|ref|XP_379406.1|	gi|6706447|ref|Os01g64350|
gi|31211845|ref|XP_314907.1|	lcl|At3g48185.1	lcl|F19D8.2 CE37356 WBGene0	gi|24654454|	gi|42657631|ref|XP_379409.1|	gi|6706450|ref|Os01g64380|
gi|31211853|ref|XP_314911.1|	lcl|At3g48220.1	lcl|F19F10.10 CE09490 WBGen	gi|24654465|	gi|42657633|ref|XP_379410.1|	gi|6706451|ref|Os01g64390|
gi|31211859|ref|XP_314914.1|	lcl|At3g48490.1	lcl|F19F10.11a CE09491 WBGe	gi|24654479|	gi|42657642|ref|XP_376499.1|	gi|6706452|ref|Os01g64400|
gi|31211861|ref|XP_314915.1|	lcl|At3g48565.1	lcl|F19F10.11b CE09492 WBGe	gi|24654524|	gi|42657646|ref|XP_379413.1|	gi|6706456|ref|Os01g64440|
gi|31211865|ref|XP_314917.1|	lcl|At3g48640.1	lcl|F19F10.8 CE36829 WBGene	gi|24654562|	gi|42657662|ref|XP_379417.1|	gi|6706458|ref|Os01g64460|
gi|31211871|ref|XP_314920.1|	lcl|At3g48650.1	lcl|F19G12.4 CE07087 WBGene	gi|24654578|	gi|42657668|ref|XP_379461.1|	gi|6706462|ref|Os01g64500|
gi|31211877|ref|XP_314923.1|	lcl|At3g48675.1	lcl|F19H6.5 CE35497 WBGene0	gi|24654621|	gi|42657670|ref|XP_379462.1|	gi|6706465|ref|Os01g64530|
gi|31211885|ref|XP_314927.1|	lcl|At3g48970.1	lcl|F19H6.6 CE03237 WBGene0	gi|24654634|	gi|42657672|ref|XP_376558.1|	gi|6706467|ref|Os01g64550|

gi|31211907|ref|XP_314938.1|	lcl|At3g49070.1	lcl|F19H6.7 CE34821 WBGene0	gi|24654646|	gi|42657674|ref|XP_379463.1|	gi|6706470|ref|Os01g64580|
gi|31211915|ref|XP_314942.1|	lcl|At3g49230.1	lcl|F19H8.2 CE15871 WBGene0	gi|24654660|	gi|42657676|ref|XP_376560.1|	gi|6706488|ref|Os01g64710|
gi|31211917|ref|XP_314943.1|	lcl|At3g49270.1	lcl|F20A1.1 CE07091 WBGene0	gi|24654669|	gi|42657684|ref|XP_379492.1|	gi|6706491|ref|Os01g64740|
gi|31211939|ref|XP_314954.1|	lcl|At3g49280.1	lcl|F20A1.10 CE07100 WBGene	gi|24654684|	gi|42657686|ref|XP_376614.1|	gi|6706503|ref|Os01g64800|
gi|31211949|ref|XP_314959.1|	lcl|At3g49300.1	lcl|F20A1.6a CE29766 WBGene	gi|24654688|	gi|42657690|ref|XP_376610.1|	gi|6706521|ref|Os01g64950|
gi|31211957|ref|XP_314963.1|	lcl|At3g49305.1	lcl|F20A1.6b CE37002 WBGene	gi|24654692|	gi|42657692|ref|XP_376611.1|	gi|6706528|ref|Os01g65020|
gi|31211959|ref|XP_314964.1|	lcl|At3g49440.1	lcl|F20A1.8 CE07098 WBGene0	gi|24654696|	gi|42657696|ref|XP_376613.1|	gi|6706530|ref|Os01g65040|
gi|31211961|ref|XP_314965.1|	lcl|At3g49460.1	lcl|F20B10.3 CE35499 WBGene	gi|24654722|	gi|42657700|ref|XP_379488.1|	gi|6706531|ref|Os01g65050|
gi|31211965|ref|XP_314967.1|	lcl|At3g49490.1	lcl|F20B4.2 CE07101 WBGene0	gi|24654724|	gi|42657702|ref|XP_379489.1|	gi|6706544|ref|Os01g65130|
gi|31211969|ref|XP_314969.1|	lcl|At3g49540.1	lcl|F20B4.3 CE07102 WBGene0	gi|24654754|	gi|42657704|ref|XP_379490.1|	gi|6706547|ref|Os01g65160|
gi|31211977|ref|XP_314973.1|	lcl|At3g49570.1	lcl|F20B4.4 CE07103 WBGene0	gi|24654765|	gi|42657706|ref|XP_379533.1|	gi|6706548|ref|Os01g65170|
gi|31211985|ref|XP_314977.1|	lcl|At3g49770.1	lcl|F20B6.4 CE04426 WBGene0	gi|24654802|	gi|42657708|ref|XP_379537.1|	gi|6706558|ref|Os01g65270|
gi|31211987|ref|XP_314978.1|	lcl|At3g49780.1	lcl|F20B6.5 CE04427 WBGene0	gi|24654817|	gi|42657710|ref|XP_379534.1|	gi|6706559|ref|Os01g65280|
gi|31211995|ref|XP_314982.1|	lcl|At3g49820.1	lcl|F20B6.7 CE04429 WBGene0	gi|24654821|	gi|42657716|ref|XP_379535.1|	gi|6706565|ref|Os01g65340|
gi|31211997|ref|XP_314983.1|	lcl|At3g50250.1	lcl|F20B6.9 CE27985 WBGene0	gi|24654824|	gi|42657723|ref|XP_379536.1|	gi|6706567|ref|Os01g65360|
gi|31211999|ref|XP_314984.1|	lcl|At3g50290.2	lcl|F20C5.3 CE35500 WBGene0	gi|24654854|	gi|42657727|ref|XP_376615.1|	gi|6706570|ref|Os01g65390|
gi|31212001|ref|XP_314985.1|	lcl|At3g50320.1	lcl|F20C5.5 CE03242 WBGene0	gi|24654856|	gi|42657729|ref|XP_379493.1|	gi|6706574|ref|Os01g65430|
gi|31212003|ref|XP_314986.1|	lcl|At3g50373.1	lcl|F20C5.6 CE03243 WBGene0	gi|24654859|	gi|42657731|ref|XP_379494.1|	gi|6706578|ref|Os01g65470|
gi|31212013|ref|XP_314991.1|	lcl|At3g50540.1	lcl|F20C5.7 CE33776 WBGene0	gi|24654866|	gi|42657733|ref|XP_376616.1|	gi|6706585|ref|Os01g65540|
gi|31212069|ref|XP_315019.1|	lcl|At3g50550.1	lcl|F20D1.10 CE23677 WBGene	gi|24654879|	gi|42657740|ref|XP_376619.1|	gi|6706588|ref|Os01g65570|
gi|31212073|ref|XP_315021.1|	lcl|At3g50550.2	lcl|F20D1.6 CE09499 WBGene0	gi|24654883|	gi|42657743|ref|XP_379495.1|	gi|6706593|ref|Os01g65620|
gi|31212077|ref|XP_315023.1|	lcl|At3g50570.1	lcl|F20D12.7 CE29768 WBGene	gi|24654922|	gi|42657747|ref|XP_379496.1|	gi|6706595|ref|Os01g65640|
gi|31212087|ref|XP_315028.1|	lcl|At3g50580.1	lcl|F20D6.10 CE35282 WBGene	gi|24654924|	gi|42657755|ref|XP_379498.1|	gi|6706601|ref|Os01g65700|
gi|31212093|ref|XP_315031.1|	lcl|At3g50610.1	lcl|F20G2.6 CE34823 WBGene0	gi|24654928|	gi|42657758|ref|XP_379499.1|	gi|6706602|ref|Os01g65710|
gi|31212103|ref|XP_315036.1|	lcl|At3g50640.1	lcl|F20G4.2 CE05674 WBGene0	gi|24654930|	gi|42657763|ref|XP_379501.1|	gi|6706606|ref|Os01g65750|
gi|31212111|ref|XP_315040.1|	lcl|At3g50900.1	lcl|F20H11.6 CE20703 WBGene	gi|24654934|	gi|42657765|ref|XP_379502.1|	gi|6706608|ref|Os01g65770|
gi|31212123|ref|XP_315046.1|	lcl|At3g50970.1	lcl|F21A3.4 CE09518 WBGene0	gi|24654940|	gi|42657775|ref|XP_379503.1|	gi|6706612|ref|Os01g65810|
gi|31212153|ref|XP_315061.1|	lcl|At3g50980.1	lcl|F21A9.1 CE34297 WBGene0	gi|24654942|	gi|42657803|ref|XP_374242.2|	gi|6706613|ref|Os01g65820|
gi|31212161|ref|XP_315065.1|	lcl|At3g51180.1	lcl|F21C10.10 CE30758 WBGen	gi|24654946|	gi|42657807|ref|XP_379467.1|	gi|6706618|ref|Os01g65870|
gi|31212185|ref|XP_315077.1|	lcl|At3g51220.1	lcl|F21C10.11 CE27987 WBGen	gi|24654950|	gi|42657811|ref|XP_379469.1|	gi|6706624|ref|Os01g65930|
gi|31212213|ref|XP_315091.1|	lcl|At3g51410.1	lcl|F21C10.5 CE07120 WBGene	gi|24654954|	gi|42657815|ref|XP_376565.1|	gi|6706626|ref|Os01g65950|
gi|31212215|ref|XP_315092.1|	lcl|At3g51500.1	lcl|F21C10.8a CE07123 WBGen	gi|24654973|	gi|42657821|ref|XP_379471.1|	gi|6706629|ref|Os01g65980|
gi|31212243|ref|XP_315106.1|	lcl|At3g51750.1	lcl|F21C10.8b CE35283 WBGen	gi|24654977|	gi|42657823|ref|XP_376568.1|	gi|6706637|ref|Os01g66060|
gi|31212283|ref|XP_315126.1|	lcl|At3g51760.1	lcl|F21C3.4 CE37005 WBGene0	gi|24654985|	gi|42657825|ref|XP_379472.1|	gi|6706646|ref|Os01g66150|
gi|31212295|ref|XP_315132.1|	lcl|At3g52110.1	lcl|F21C3.6 CE05681 WBGene0	gi|24654990|	gi|42657827|ref|XP_376569.1|	gi|6706652|ref|Os01g66210|
gi|31212299|ref|XP_315134.1|	lcl|At3g52360.1	lcl|F21D12.2 CE02667 WBGene	gi|24655051|	gi|42657853|ref|XP_379474.1|	gi|6706654|ref|Os01g66230|
gi|31212305|ref|XP_315137.1|	lcl|At3g52420.1	lcl|F21D5.9 CE03251 WBGene0	gi|24655053|	gi|42657858|ref|XP_379476.1|	gi|6706657|ref|Os01g66260|
gi|31212309|ref|XP_315139.1|	lcl|At3g52480.1	lcl|F21D9.2 CE23679 WBGene0	gi|24655055|	gi|42657864|ref|XP_376586.1|	gi|6706662|ref|Os01g66310|
gi|31212313|ref|XP_315141.1|	lcl|At3g52510.1	lcl|F21D9.3 CE18629 WBGene0	gi|24655059|	gi|42657868|ref|XP_379478.1|	gi|6706663|ref|Os01g66320|
gi|31212317|ref|XP_315143.1|	lcl|At3g52520.1	lcl|F21D9.8 CE18633 WBGene0	gi|24655063|	gi|42657872|ref|XP_379479.1|	gi|6706665|ref|Os01g66340|
gi|31212319|ref|XP_315144.1|	lcl|At3g52550.1	lcl|F21E9.2 CE09526 WBGene0	gi|24655091|	gi|42657876|ref|XP_379480.1|	gi|6706671|ref|Os01g66400|
gi|31212343|ref|XP_315156.1|	lcl|At3g52620.1	lcl|F21E9.4 CE09528 WBGene0	gi|24655109|	gi|42657878|ref|XP_379481.1|	gi|6706672|ref|Os01g66410|
gi|31212349|ref|XP_315159.1|	lcl|At3g52700.1	lcl|F21E9.6 CE17690 WBGene0	gi|24655117|	gi|42657880|ref|XP_379482.1|	gi|6706674|ref|Os01g66430|
gi|31212361|ref|XP_315165.1|	lcl|At3g52710.1	lcl|F21F12.1 CE09546 WBGene	gi|24655121|	gi|42657886|ref|XP_379483.1|	gi|6706675|ref|Os01g66440|
gi|31212373|ref|XP_315171.1|	lcl|At3g52770.1	lcl|F21F3.4 CE09534 WBGene0	gi|24655142|	gi|42657896|ref|XP_376595.1|	gi|6706679|ref|Os01g66480|
gi|31212375|ref|XP_315172.1|	lcl|At3g52830.1	lcl|F21F3.6 CE09536 WBGene0	gi|24655170|	gi|42657911|ref|XP_379486.1|	gi|6706684|ref|Os01g66530|
gi|31212437|ref|XP_315203.1|	lcl|At3g53070.1	lcl|F21F8.5 CE19404 WBGene0	gi|24655174|	gi|42657915|ref|XP_376605.1|	gi|6706685|ref|Os01g66540|
gi|31212461|ref|XP_315215.1|	lcl|At3g53235.1	lcl|F21F8.6 CE35285 WBGene0	gi|24655187|	gi|42657917|ref|XP_379487.1|	gi|6706686|ref|Os01g66550|
gi|31212463|ref|XP_315216.1|	lcl|At3g53370.1	lcl|F21G4.5 CE09551 WBGene0	gi|24655189|	gi|42657919|ref|XP_376607.1|	gi|6706688|ref|Os01g66570|
gi|31212465|ref|XP_315217.1|	lcl|At3g53670.1	lcl|F21G4.6 CE37360 WBGene0	gi|24655220|	gi|42657952|ref|XP_379526.1|	gi|6706697|ref|Os01g66650|
gi|31212485|ref|XP_315227.1|	lcl|At3g54060.1	lcl|F21H12.2 CE01913 WBGene	gi|24655366|	gi|42657981|ref|XP_379527.1|	gi|6706698|ref|Os01g66660|
gi|31212501|ref|XP_315235.1|	lcl|At3g54330.1	lcl|F21H12.3 CE36830 WBGene	gi|24655396|	gi|42657983|ref|XP_379528.1|	gi|6706699|ref|Os01g66670|
gi|31212517|ref|XP_315243.1|	lcl|At3g54520.1	lcl|F21H7.12 CE31910 WBGene	gi|24655401|	gi|42657985|ref|XP_379529.1|	gi|6706707|ref|Os01g66750|
gi|31212523|ref|XP_315246.1|	lcl|At3g54530.1	lcl|F21H7.2 CE15875 WBGene0	gi|24655413|	gi|42657989|ref|XP_379530.1|	gi|6706709|ref|Os01g66770|
gi|31212525|ref|XP_315247.1|	lcl|At3g54580.1	lcl|F21H7.3 CE19801 WBGene0	gi|24655429|	gi|42657993|ref|XP_376725.1|	gi|6706710|ref|Os01g66780|
gi|31212529|ref|XP_315249.1|	lcl|At3g54590.1	lcl|F22A3.4 CE04440 WBGene0	gi|24655457|	gi|42657995|ref|XP_379531.1|	gi|6706712|ref|Os01g66800|
gi|31212533|ref|XP_315251.1|	lcl|At3g54680.1	lcl|F22B3.9 CE34824 WBGene0	gi|24655519|	gi|42657997|ref|XP_379532.1|	gi|6706713|ref|Os01g66810|
gi|31212555|ref|XP_315262.1|	lcl|At3g54730.1	lcl|F22B5.4 CE02199 WBGene0	gi|24655524|	gi|42658014|ref|XP_378002.1|	gi|6706725|ref|Os01g66880|
gi|31212561|ref|XP_315265.1|	lcl|At3g55690.1	lcl|F22B5.6 CE02201 WBGene0	gi|24655544|	gi|42658018|ref|XP_379504.1|	gi|6706730|ref|Os01g66910|
gi|31212563|ref|XP_315266.1|	lcl|At3g55790.1	lcl|F22B7.1 CE00153 WBGene0	gi|24655549|	gi|42658027|ref|XP_376643.1|	gi|6706734|ref|Os01g66950|
gi|31212567|ref|XP_315268.1|	lcl|At3g55860.1	lcl|F22B7.2 CE00155 WBGene0	gi|24655558|	gi|42658043|ref|XP_379507.1|	gi|6706741|ref|Os01g66990|
gi|31212599|ref|XP_315284.1|	lcl|At3g55900.1	lcl|F22B7.3 CE00156 WBGene0	gi|24655659|	gi|42658045|ref|XP_379508.1|	gi|6706751|ref|Os01g67070|
gi|31212603|ref|XP_315286.1|	lcl|At3g55910.1	lcl|F22B7.4 CE00157 WBGene0	gi|24655664|	gi|42658051|ref|XP_376651.1|	gi|6706752|ref|Os01g67080|
gi|31212615|ref|XP_315292.1|	lcl|At3g56250.1	lcl|F22D3.2c CE37762 WBGene	gi|24655682|	gi|42658060|ref|XP_379511.1|	gi|6706759|ref|Os01g67150|
gi|31212629|ref|XP_315299.1|	lcl|At3g56260.1	lcl|F22D3.4 CE02673 WBGene0	gi|24655753|	gi|42658077|ref|XP_379512.1|	gi|6706770|ref|Os01g67260|
gi|31212667|ref|XP_315318.1|	lcl|At3g56270.1	lcl|F22D3.5 CE02674 WBGene0	gi|24655789|	gi|42658086|ref|XP_379514.1|	gi|6706771|ref|Os01g67270|
gi|31212675|ref|XP_315322.1|	lcl|At3g56390.1	lcl|F22D6.14 CE23680 WBGene	gi|24655806|	gi|42658094|ref|XP_379516.1|	gi|6706772|ref|Os01g67280|
gi|31212677|ref|XP_315323.1|	lcl|At3g56410.1	lcl|F22D6.15 CE34825 WBGene	gi|24655815|	gi|42658098|ref|XP_379518.1|	gi|6706774|ref|Os01g67300|
gi|31212707|ref|XP_315338.1|	lcl|At3g56410.2	lcl|F22D6.7 CE05688 WBGene0	gi|24655819|	gi|42658102|ref|XP_376670.1|	gi|6706784|ref|Os01g67380|
gi|31212735|ref|XP_315352.1|	lcl|At3g56520.1	lcl|F22E12.3 CE05697 WBGene	gi|24655832|	gi|42658104|ref|XP_295213.4|	gi|6706786|ref|Os01g67400|
gi|31212741|ref|XP_315355.1|	lcl|At3g56610.1	lcl|F22E5.17 CE27992 WBGene	gi|24655838|	gi|42658116|ref|XP_379521.1|	gi|6706790|ref|Os01g67440|
gi|31212759|ref|XP_315364.1|	lcl|At3g56670.1	lcl|F22E5.2 CE09554 WBGene0	gi|24655843|	gi|42658125|ref|XP_379523.1|	gi|6706807|ref|Os01g67610|
gi|31212767|ref|XP_315368.1|	lcl|At3g56810.1	lcl|F22E5.20 CE33623 WBGene	gi|24655845|	gi|42658132|ref|XP_379524.1|	gi|6706808|ref|Os01g67620|
gi|31212785|ref|XP_315377.1|	lcl|At3g56890.1	lcl|F22E5.7 CE27993 WBGene0	gi|24655848|	gi|42658178|ref|XP_380082.1|	gi|6706812|ref|Os01g67660|
gi|31212807|ref|XP_315388.1|	lcl|At3g56910.1	lcl|F22E5.9 CE09561 WBGene0	gi|24655853|	gi|42658182|ref|XP_380085.1|	gi|6706814|ref|Os01g67680|
gi|31212839|ref|XP_315404.1|	lcl|At3g57110.1	lcl|F22F4.1 CE07126 WBGene0	gi|24655888|	gi|42658186|ref|XP_379772.1|	gi|6706816|ref|Os01g67700|
gi|31212843|ref|XP_315406.1|	lcl|At3g57160.1	lcl|F22F4.4 CE07129 WBGene0	gi|24655891|	gi|42658192|ref|XP_380087.1|	gi|6706817|ref|Os01g67710|
gi|31212847|ref|XP_315408.1|	lcl|At3g57250.1	lcl|F22F4.5 CE35504 WBGene0	gi|24655893|	gi|42658194|ref|XP_379775.1|	gi|6706826|ref|Os01g67760|
gi|31212863|ref|XP_310028.1|	lcl|At3g57400.1	lcl|F22F7.6 CE35286 WBGene0	gi|24655901|	gi|42658196|ref|XP_380088.1|	gi|6706828|ref|Os01g67780|
gi|31212871|ref|XP_312169.1|	lcl|At3g57440.1	lcl|F22G12.1 CE15888 WBGene	gi|24655905|	gi|42658198|ref|XP_379776.1|	gi|6706829|ref|Os01g67790|
gi|31212873|ref|XP_312170.1|	lcl|At3g57583.1	lcl|F22H10.2 CE09571 WBGene	gi|24655930|	gi|42658229|ref|XP_380089.1|	gi|6706831|ref|Os01g67810|
gi|31212887|ref|XP_312202.1|	lcl|At3g57586.1	lcl|F22H10.3 CE09572 WBGene	gi|24655991|	gi|42658237|ref|XP_380091.1|	gi|6706832|ref|Os01g67810|
gi|31212903|ref|XP_314548.1|	lcl|At3g57690.1	lcl|F22H10.6 CE09575 WBGene	gi|24655999|	gi|42658243|ref|XP_379798.1|	gi|6706835|ref|Os01g67840|
gi|31212915|ref|XP_314861.1|	lcl|At3g57850.1	lcl|F23A7.1 CE09576 WBGene0	gi|24656021|	gi|42658249|ref|XP_380094.1|	gi|6706842|ref|Os01g67900|
gi|31212919|ref|XP_314871.1|	lcl|At3g57860.1	lcl|F23A7.3 CE09577 WBGene0	gi|24656034|	gi|42658252|ref|XP_380095.1|	gi|6706843|ref|Os01g67910|
gi|31212925|ref|XP_314888.1|	lcl|At3g57900.1	lcl|F23A7.4 CE09578 WBGene0	gi|24656038|	gi|42658256|ref|XP_380096.1|	gi|6706845|ref|Os01g67930|
gi|31212943|ref|XP_314906.1|	lcl|At3g57930.1	lcl|F23A7.5 CE34175 WBGene0	gi|24656043|	gi|42658258|ref|XP_380097.1|	gi|6706846|ref|Os01g67940|
gi|31212945|ref|XP_315416.1|	lcl|At3g58080.1	lcl|F23A7.6 CE26811 WBGene0	gi|24656051|	gi|42658260|ref|XP_380098.1|	gi|6706862|ref|Os01g68100|
gi|31212999|ref|XP_315443.1|	lcl|At3g58230.1	lcl|F23B12.3 CE09595 WBGene	gi|24656056|	gi|42658266|ref|XP_380099.1|	gi|6706863|ref|Os01g68110|
gi|31213003|ref|XP_315445.1|	lcl|At3g58280.1	lcl|F23B12.9 CE35714 WBGene	gi|24656074|	gi|42658277|ref|XP_379809.1|	gi|6706865|ref|Os01g68130|
gi|31213007|ref|XP_315447.1|	lcl|At3g58300.1	lcl|F23B2.5a CE09585 WBGene	gi|24656078|	gi|42658302|ref|XP_380105.1|	gi|6706870|ref|Os01g68180|
gi|31213009|ref|XP_315448.1|	lcl|At3g58320.1	lcl|F23B2.5b CE32045 WBGene	gi|24656081|	gi|42658306|ref|XP_379825.1|	gi|6706871|ref|Os01g68190|
gi|31213035|ref|XP_315461.1|	lcl|At3g58330.1	lcl|F23B2.5c CE32046 WBGene	gi|24656107|	gi|42658309|ref|XP_380106.1|	gi|6706873|ref|Os01g68210|
gi|31213069|ref|XP_315478.1|	lcl|At3g58370.1	lcl|F23B2.7 CE17698 WBGene0	gi|24656139|	gi|42658311|ref|XP_379827.1|	gi|6706876|ref|Os01g68230|
gi|31213071|ref|XP_315479.1|	lcl|At3g58540.1	lcl|F23B2.8 CE32182 WBGene0	gi|24656191|	gi|42658316|ref|XP_379831.1|	gi|6706877|ref|Os01g68240|
gi|31213077|ref|XP_315482.1|	lcl|At3g58770.1	lcl|F23C8.1 CE34298 WBGene0	gi|24656203|	gi|42658318|ref|XP_379832.1|	gi|6706878|ref|Os01g68250|
gi|31213145|ref|XP_315516.1|	lcl|At3g58840.1	lcl|F23C8.11 CE20719 WBGene	gi|24656208|	gi|42658322|ref|XP_379835.1|	gi|6706880|ref|Os01g68270|
gi|31213149|ref|XP_315518.1|	lcl|At3g58850.1	lcl|F23C8.12 CE20720 WBGene	gi|24656213|	gi|42658327|ref|XP_380107.1|	gi|6706882|ref|Os01g68290|
gi|31213153|ref|XP_315520.1|	lcl|At3g58870.1	lcl|F23C8.3 CE30515 WBGene0	gi|24656235|	gi|42658331|ref|XP_380108.1|	gi|6706883|ref|Os01g68300|
gi|31213163|ref|XP_315525.1|	lcl|At3g59370.1	lcl|F23D12.1 CE05699 WBGene	gi|24656259|	gi|42658333|ref|XP_380109.1|	gi|6706896|ref|Os01g68400|
gi|31213185|ref|XP_315536.1|	lcl|At3g59430.1	lcl|F23D12.3 CE05701 WBGene	gi|24656267|	gi|42658343|ref|XP_380111.1|	gi|6706912|ref|Os01g68560|
gi|31213203|ref|XP_315545.1|	lcl|At3g59460.1	lcl|F23D12.4 CE05702 WBGene	gi|24656273|	gi|42658345|ref|XP_379842.1|	gi|6706913|ref|Os01g68570|
gi|31213217|ref|XP_315552.1|	lcl|At3g59860.1	lcl|F23D12.6 CE32388 WBGene	gi|24656275|	gi|42658375|ref|XP_379843.1|	gi|6706915|ref|Os01g68590|
gi|31213219|ref|XP_315553.1|	lcl|At3g59880.1	lcl|F23D12.7 CE34826 WBGene	gi|24656280|	gi|42658377|ref|XP_380112.1|	gi|6706920|ref|Os01g68640|
gi|31213223|ref|XP_315555.1|	lcl|At3g59900.1	lcl|F23F1.10 CE36433 WBGene	gi|24656299|	gi|42658379|ref|XP_380113.1|	gi|6706925|ref|Os01g68690|
gi|31213241|ref|XP_315564.1|	lcl|At3g59930.1	lcl|F23F1.3 CE31795 WBGene0	gi|24656337|	gi|42658381|ref|XP_379844.1|	gi|6706928|ref|Os01g68720|
gi|31213253|ref|XP_315570.1|	lcl|At3g60060.1	lcl|F23F1.4 CE32635 WBGene0	gi|24656369|	gi|42658389|ref|XP_379848.1|	gi|6706929|ref|Os01g68730|
gi|31213259|ref|XP_315573.1|	lcl|At3g60200.1	lcl|F23F12.12 CE29282 WBGen	gi|24656426|	gi|42658393|ref|XP_380114.1|	gi|6706930|ref|Os01g68740|
gi|31213267|ref|XP_315577.1|	lcl|At3g60230.1	lcl|F23F12.4 CE01251 WBGene	gi|24656466|	gi|42658399|ref|XP_379852.1|	gi|6706934|ref|Os01g68780|
gi|31213277|ref|XP_315582.1|	lcl|At3g60380.1	lcl|F23F12.7 CE01254 WBGene	gi|24656474|	gi|42658403|ref|XP_380115.1|	gi|6706936|ref|Os01g68800|
gi|31213279|ref|XP_315583.1|	lcl|At3g60410.1	lcl|F23F12.8 CE01255 WBGene	gi|24656479|	gi|42658411|ref|XP_380117.1|	gi|6706938|ref|Os01g68820|
gi|31213283|ref|XP_315585.1|	lcl|At3g60560.1	lcl|F23F12.9b CE32389 WBGen	gi|24656484|	gi|42658413|ref|XP_380118.1|	gi|6706941|ref|Os01g68850|
gi|31213301|ref|XP_315594.1|	lcl|At3g60650.1	lcl|F23H11.2 CE34542 WBGene	gi|24656529|	gi|42658419|ref|XP_380120.1|	gi|6706944|ref|Os01g68880|
gi|31213311|ref|XP_315599.1|	lcl|At3g60760.1	lcl|F23H11.4b CE32390 WBGen	gi|24656532|	gi|42658421|ref|XP_380121.1|	gi|6706947|ref|Os01g68910|
gi|31213313|ref|XP_315600.1|	lcl|At3g60890.1	lcl|F23H11.5 CE09613 WBGene	gi|24656558|	gi|42658429|ref|XP_380122.1|	gi|6706954|ref|Os01g68980|
gi|31213325|ref|XP_315606.1|	lcl|At3g60930.1	lcl|F23H11.6 CE09614 WBGene	gi|24656673|	gi|42658469|ref|XP_380125.1|	gi|6706956|ref|Os01g69000|
gi|31213327|ref|XP_315607.1|	lcl|At3g61660.1	lcl|F23H11.7 CE27998 WBGene	gi|24656683|	gi|42658481|ref|XP_380126.1|	gi|6706958|ref|Os01g69020|
gi|31213349|ref|XP_315618.1|	lcl|At3g61780.1	lcl|F23H12.3 CE31912 WBGene	gi|24656841|	gi|42658483|ref|XP_379879.1|	gi|6706972|ref|Os01g69150|
gi|31213351|ref|XP_315619.1|	lcl|At3g61840.1	lcl|F23H12.7 CE05710 WBGene	gi|24656869|	gi|42658497|ref|XP_380128.1|	gi|6706974|ref|Os01g69170|
gi|31213357|ref|XP_315622.1|	lcl|At3g61920.1	lcl|F23H12.8 CE31913 WBGene	gi|24656913|	gi|42658500|ref|XP_380129.1|	gi|6706983|ref|Os01g69260|
gi|31213359|ref|XP_315623.1|	lcl|At3g61930.1	lcl|F23H12.9 CE31914 WBGene	gi|24656921|	gi|42658505|ref|XP_379891.1|	gi|6706986|ref|Os01g69290|
gi|31213365|ref|XP_315626.1|	lcl|At3g62320.1	lcl|F25B3.5 CE31491 WBGene0	gi|24656946|	gi|42658513|ref|XP_380133.1|	gi|6706987|ref|Os01g69790|
gi|31213369|ref|XP_315628.1|	lcl|At3g62350.1	lcl|F25B4.4 CE09622 WBGene0	gi|24656951|	gi|42658529|ref|XP_380134.1|	gi|6706992|ref|Os01g69840|
gi|31213381|ref|XP_315634.1|	lcl|At3g62380.1	lcl|F25B5.2 CE01919 WBGene0	gi|24656982|	gi|42658565|ref|XP_380135.1|	gi|6706994|ref|Os01g69860|
gi|31213391|ref|XP_315639.1|	lcl|At3g62400.1	lcl|F25B5.3e CE30664 WBGene	gi|24657043|	gi|42658577|ref|XP_380137.1|	gi|6707006|ref|Os01g69960|
gi|31213393|ref|XP_315640.1|	lcl|At3g62400.2	lcl|F25B5.7c CE29285 WBGene	gi|24657046|	gi|42658581|ref|XP_380138.1|	gi|6707010|ref|Os01g70000|
gi|31213399|ref|XP_315643.1|	lcl|At3g62480.1	lcl|F25D7.4 CE09632 WBGene0	gi|24657112|	gi|42658591|ref|XP_380140.1|	gi|6707017|ref|Os01g70070|
gi|31213417|ref|XP_315652.1|	lcl|At3g62490.1	lcl|F25E2.2 CE37106 WBGene0	gi|24657117|	gi|42658593|ref|XP_380141.1|	gi|6707022|ref|Os01g70120|
gi|31213419|ref|XP_315653.1|	lcl|At3g62500.1	lcl|F25E5.1 CE07135 WBGene0	gi|24657135|	gi|42658597|ref|XP_380143.1|	gi|6707025|ref|Os01g70150|
gi|31213447|ref|XP_315667.1|	lcl|At3g62680.1	lcl|F25E5.8a CE07142 WBGene	gi|24657155|	gi|42658599|ref|XP_379919.1|	gi|6707041|ref|Os01g70280|
gi|31213477|ref|XP_315682.1|	lcl|At3g62990.1	lcl|F25E5.8b CE32876 WBGene	gi|24657158|	gi|42658601|ref|XP_379920.1|	gi|6707042|ref|Os01g70290|
gi|31213503|ref|XP_315695.1|	lcl|At3g63020.1	lcl|F25E5.9 CE07143 WBGene0	gi|24657177|	gi|42658617|ref|XP_380147.1|	gi|6707048|ref|Os01g70340|
gi|31213523|ref|XP_315705.1|	lcl|At3g63040.1	lcl|F25F6.1 CE09634 WBGene0	gi|24657191|	gi|42658622|ref|XP_380148.1|	gi|6707049|ref|Os01g70350|
gi|31213545|ref|XP_315716.1|	lcl|At3g63050.1	lcl|F25G6.1 CE32877 WBGene0	gi|24657197|	gi|42658630|ref|XP_380149.1|	gi|6707054|ref|Os01g70400|

gi|31213547|ref|XP_315717.1|	lcl|At3g63100.1	lcl|F25H5.2 CE15897 WBGene0	gi|24657200|	gi|42658638|ref|XP_380150.1|	gi|6707058|ref|Os01g70420|
gi|31213551|ref|XP_315719.1|	lcl|At3g63160.1	lcl|F25H5.5 CE15901 WBGene0	gi|24657243|	gi|42658646|ref|XP_380151.1|	gi|6707060|ref|Os01g70440|
gi|31213608|ref|XP_315729.1|	lcl|At3g63180.1	lcl|F25H8.5b CE35291 WBGene	gi|24657294|	gi|42658668|ref|XP_380152.1|	gi|6707061|ref|Os01g70450|
gi|31213631|ref|XP_315735.1|	lcl|At3g63230.1	lcl|F25H8.7 CE26700 WBGene0	gi|24657322|	gi|42658670|ref|XP_380153.1|	gi|6707063|ref|Os01g70470|
gi|31213638|ref|XP_315736.1|	lcl|At3g63420.1	lcl|F26A1.1 CE27757 WBGene0	gi|24657340|	gi|42658674|ref|XP_380154.1|	gi|6707066|ref|Os01g70500|
gi|31213678|ref|XP_315745.1|	lcl|At3g63420.2	lcl|F26A1.6 CE02687 WBGene0	gi|24657369|	gi|42658678|ref|XP_379975.1|	gi|6707077|ref|Os01g70610|
gi|31213720|ref|XP_315753.1|	lcl|At3g63430.1	lcl|F26A1.7 CE02688 WBGene0	gi|24657389|	gi|42658680|ref|XP_380155.1|	gi|6707078|ref|Os01g70620|
gi|31213751|ref|XP_315758.1|	lcl|At4g00232.1	lcl|F26A1.8 CE02689 WBGene0	gi|24657398|	gi|42658682|ref|XP_380156.1|	gi|6707080|ref|Os01g70640|
gi|31213775|ref|XP_315762.1|	lcl|At4g00280.1	lcl|F26A1.9 CE29287 WBGene0	gi|24657457|	gi|42658690|ref|XP_380157.1|	gi|6707088|ref|Os01g70720|
gi|31213839|ref|XP_315769.1|	lcl|At4g00310.1	lcl|F26A10.1 CE02691 WBGene	gi|24657462|	gi|42658746|ref|XP_380077.1|	gi|6707090|ref|Os01g70740|
gi|31213881|ref|XP_315775.1|	lcl|At4g00440.1	lcl|F26A3.5 CE09670 WBGene0	gi|24657470|	gi|42658748|ref|XP_380158.1|	gi|6707107|ref|Os01g70910|
gi|31213899|ref|XP_315778.1|	lcl|At4g00525.1	lcl|F26A3.6 CE09671 WBGene0	gi|24657489|	gi|42658750|ref|XP_380159.1|	gi|6707116|ref|Os01g70980|
gi|31214007|ref|XP_315794.1|	lcl|At4g00530.1	lcl|F26A3.7 CE09672 WBGene0	gi|24657496|	gi|42658756|ref|XP_380160.1|	gi|6707120|ref|Os01g71020|
gi|31214060|ref|XP_315802.1|	lcl|At4g00580.1	lcl|F26B1.1 CE30517 WBGene0	gi|24657588|	gi|42658763|ref|XP_380162.1|	gi|6707121|ref|Os01g71030|
gi|31214065|ref|XP_315803.1|	lcl|At4g00670.1	lcl|F26C11.3 CE37833 WBGene	gi|24657619|	gi|42658767|ref|XP_374260.2|	gi|6707122|ref|Os01g71040|
gi|31214110|ref|XP_315810.1|	lcl|At4g00695.1	lcl|F26D11.4 CE17708 WBGene	gi|24657627|	gi|42658770|ref|XP_376761.1|	gi|6707134|ref|Os01g71150|
gi|31214140|ref|XP_315812.1|	lcl|At4g00770.1	lcl|F26D11.6 CE17710 WBGene	gi|24657634|	gi|42658779|ref|XP_209910.5|	gi|6707136|ref|Os01g71170|
gi|31214155|ref|XP_315814.1|	lcl|At4g00890.1	lcl|F26D12.1d CE36589 WBGen	gi|24657641|	gi|42658786|ref|XP_376801.1|	gi|6707140|ref|Os01g71210|
gi|31214233|ref|XP_315825.1|	lcl|At4g00920.1	lcl|F26D2.16 CE34828 WBGene	gi|24657659|	gi|42658792|ref|XP_379539.1|	gi|6707152|ref|Os01g71330|
gi|31214240|ref|XP_315826.1|	lcl|At4g00930.1	lcl|F26D2.2 CE33925 WBGene0	gi|24657671|	gi|42658794|ref|XP_379540.1|	gi|6707156|ref|Os01g71360|
gi|31214247|ref|XP_315827.1|	lcl|At4g00950.1	lcl|F26D2.3b CE36138 WBGene	gi|24657689|	gi|42658796|ref|XP_379541.1|	gi|6707157|ref|Os01g71370|
gi|31214254|ref|XP_315828.1|	lcl|At4g00955.1	lcl|F26E4.2 CE09686 WBGene0	gi|24657697|	gi|42658798|ref|XP_372010.2|	gi|6707159|ref|Os01g71390|
gi|31214275|ref|XP_315831.1|	lcl|At4g01245.1	lcl|F26E4.4 CE09688 WBGene0	gi|24657699|	gi|42658802|ref|XP_379543.1|	gi|6707165|ref|Os01g71440|
gi|31214282|ref|XP_315832.1|	lcl|At4g01340.1	lcl|F26F12.2 CE07147 WBGene	gi|24657705|	gi|42658804|ref|XP_379545.1|	gi|6707169|ref|Os01g71480|
gi|31214319|ref|XP_315837.1|	lcl|At4g01490.1	lcl|F26F12.3a CE36538 WBGen	gi|24657713|	gi|42658808|ref|XP_379547.1|	gi|6707170|ref|Os01g71490|
gi|31214384|ref|XP_315844.1|	lcl|At4g01525.1	lcl|F26F12.3b CE20728 WBGen	gi|24657732|	gi|42658812|ref|XP_379548.1|	gi|6707171|ref|Os01g71500|
gi|31214540|ref|XP_315862.1|	lcl|At4g01535.1	lcl|F26F12.3c CE37765 WBGen	gi|24657755|	gi|42658821|ref|XP_379550.1|	gi|6707176|ref|Os01g71550|
gi|31214570|ref|XP_315866.1|	lcl|At4g01735.1	lcl|F26F12.4 CE07149 WBGene	gi|24657763|	gi|42658823|ref|XP_378007.1|	gi|6707178|ref|Os01g71570|
gi|31214626|ref|XP_315873.1|	lcl|At4g01895.1	lcl|F26F12.5a CE32881 WBGen	gi|24657766|	gi|42658827|ref|XP_378009.1|	gi|6707179|ref|Os01g71580|
gi|31214645|ref|XP_315875.1|	lcl|At4g01960.1	lcl|F26F12.5b CE07150 WBGen	gi|24657770|	gi|42658836|ref|XP_376795.1|	gi|6707181|ref|Os01g71600|
gi|31214717|ref|XP_315887.1|	lcl|At4g02000.1	lcl|F26F2.1 CE19813 WBGene0	gi|24657774|	gi|42658844|ref|XP_374283.2|	gi|6707184|ref|Os01g71630|
gi|31214739|ref|XP_315891.1|	lcl|At4g02040.1	lcl|F26F2.2 CE19814 WBGene0	gi|24657785|	gi|42658846|ref|XP_379608.1|	gi|6707185|ref|Os01g71640|
gi|31214759|ref|XP_315895.1|	lcl|At4g02090.1	lcl|F26F2.3 CE19815 WBGene0	gi|24657793|	gi|42658848|ref|XP_379609.1|	gi|6707194|ref|Os01g71730|
gi|31214772|ref|XP_315897.1|	lcl|At4g02140.1	lcl|F26F2.4 CE19816 WBGene0	gi|24657821|	gi|42658858|ref|XP_379559.1|	gi|6707196|ref|Os01g71750|
gi|31214799|ref|XP_315902.1|	lcl|At4g02160.1	lcl|F26F2.5 CE19817 WBGene0	gi|24657850|	gi|42658859|ref|XP_374252.2|	gi|6707200|ref|Os01g71790|
gi|31214806|ref|XP_315903.1|	lcl|At4g02170.1	lcl|F26F4.13 CE30766 WBGene	gi|24657862|	gi|42658864|ref|XP_379562.1|	gi|6707208|ref|Os01g71870|
gi|31214837|ref|XP_315908.1|	lcl|At4g02240.1	lcl|F26F4.2 CE29288 WBGene0	gi|24657875|	gi|42658873|ref|XP_379551.1|	gi|6707209|ref|Os01g71880|
gi|31214866|ref|XP_315914.1|	lcl|At4g02270.1	lcl|F26F4.6 CE29290 WBGene0	gi|24657883|	gi|42658878|ref|XP_379552.1|	gi|6707213|ref|Os01g71920|
gi|31214873|ref|XP_315915.1|	lcl|At4g02425.1	lcl|F26F4.9a CE31918 WBGene	gi|24657899|	gi|42658883|ref|XP_379553.1|	gi|6707215|ref|Os01g71940|
gi|31214877|ref|XP_315916.1|	lcl|At4g02465.1	lcl|F26F4.9b CE29292 WBGene	gi|24657910|	gi|42658885|ref|XP_379554.1|	gi|6707216|ref|Os01g71950|
gi|31214921|ref|XP_315927.1|	lcl|At4g02810.1	lcl|F26G1.2 CE02694 WBGene0	gi|24658020|	gi|42658897|ref|XP_376809.1|	gi|6707242|ref|Os01g72180|
gi|31214933|ref|XP_315930.1|	lcl|At4g02830.1	lcl|F26G5.1 CE28008 WBGene0	gi|24658040|	gi|42658901|ref|XP_374123.2|	gi|6707268|ref|Os01g72440|
gi|31215012|ref|XP_315946.1|	lcl|At4g02870.1	lcl|F26H11.1 CE26712 WBGene	gi|24658052|	gi|42658903|ref|XP_379573.1|	gi|6707271|ref|Os01g72470|
gi|31215093|ref|XP_315961.1|	lcl|At4g02910.1	lcl|F26H11.4 CE15911 WBGene	gi|24658062|	gi|42658908|ref|XP_376785.1|	gi|6707283|ref|Os01g72590|
gi|31215112|ref|XP_315965.1|	lcl|At4g03040.1	lcl|F26H9.1 CE09706 WBGene0	gi|24658088|	gi|42658914|ref|XP_379592.1|	gi|6707287|ref|Os01g72630|
gi|31215137|ref|XP_315970.1|	lcl|At4g03165.1	lcl|F27B10.1 CE30769 WBGene	gi|24658105|	gi|42658916|ref|XP_379594.1|	gi|6707288|ref|Os01g72640|
gi|31215145|ref|XP_315972.1|	lcl|At4g03205.2	lcl|F27B3.6 CE24925 WBGene0	gi|24658118|	gi|42658922|ref|XP_379595.1|	gi|6707290|ref|Os01g72660|
gi|31215177|ref|XP_315978.1|	lcl|At4g03305.1	lcl|F27B3.7 CE29293 WBGene0	gi|24658148|	gi|42658924|ref|XP_376791.1|	gi|6707291|ref|Os01g72670|
gi|31215235|ref|XP_315987.1|	lcl|At4g03540.1	lcl|F27C1.1 CE09714 WBGene0	gi|24658193|	gi|42658926|ref|XP_379596.1|	gi|6707297|ref|Os01g72720|
gi|31215241|ref|XP_315988.1|	lcl|At4g03580.1	lcl|F27C1.10 CE30521 WBGene	gi|24658255|	gi|42658928|ref|XP_379597.1|	gi|6707310|ref|Os01g72840|
gi|31215246|ref|XP_315989.1|	lcl|At4g03620.1	lcl|F27C1.4 CE09717 WBGene0	gi|24658262|	gi|42658936|ref|XP_372058.2|	gi|6707318|ref|Os01g72910|
gi|31215250|ref|XP_315990.1|	lcl|At4g03670.1	lcl|F27C8.3 CE03268 WBGene0	gi|24658319|	gi|42658938|ref|XP_379603.1|	gi|6707319|ref|Os01g72920|
gi|31215268|ref|XP_315993.1|	lcl|At4g03680.1	lcl|F27D4.2 CE36140 WBGene0	gi|24658370|	gi|42658940|ref|XP_379605.1|	gi|6707331|ref|Os01g73010|
gi|31215321|ref|XP_316003.1|	lcl|At4g03700.1	lcl|F27D4.6 CE32394 WBGene0	gi|24658375|	gi|42658947|ref|XP_376771.1|	gi|6707333|ref|Os01g73030|
gi|31215333|ref|XP_316005.1|	lcl|At4g03720.1	lcl|F27D9.3 CE02700 WBGene0	gi|24658386|	gi|42658953|ref|XP_379582.1|	gi|6707335|ref|Os01g73050|
gi|31215337|ref|XP_316006.1|	lcl|At4g03735.1	lcl|F27D9.4 CE02701 WBGene0	gi|24658441|	gi|42658961|ref|XP_379583.1|	gi|6707338|ref|Os01g73080|
gi|31215347|ref|XP_316008.1|	lcl|At4g03740.1	lcl|F27E5.3 CE01564 WBGene0	gi|24658492|	gi|42658963|ref|XP_379584.1|	gi|6707339|ref|Os01g73080|
gi|31215356|ref|XP_316010.1|	lcl|At4g03750.1	lcl|F27E5.4 CE30964 WBGene0	gi|24658523|	gi|42658965|ref|XP_379586.1|	gi|6707340|ref|Os01g73090|
gi|31215376|ref|XP_316014.1|	lcl|At4g03760.1	lcl|F27E5.5 CE37898 WBGene0	gi|24658546|	gi|42658967|ref|XP_379587.1|	gi|6707342|ref|Os01g73110|
gi|31215398|ref|XP_316019.1|	lcl|At4g03775.1	lcl|F27E5.8 CE37899 WBGene0	gi|24658548|	gi|42658990|ref|XP_372037.2|	gi|6707349|ref|Os01g73180|
gi|31215427|ref|XP_316025.1|	lcl|At4g03780.1	lcl|F28A10.4 CE19409 WBGene	gi|24658553|	gi|42658995|ref|XP_379676.1|	gi|6707352|ref|Os01g73210|
gi|31215447|ref|XP_316030.1|	lcl|At4g03890.1	lcl|F28A10.7 CE19412 WBGene	gi|24658726|	gi|42658997|ref|XP_376924.1|	gi|6707355|ref|Os01g73240|
gi|31215546|ref|XP_316050.1|	lcl|At4g03940.1	lcl|F28B1.1 CE15913 WBGene0	gi|24658729|	gi|42658999|ref|XP_379677.1|	gi|6707357|ref|Os01g73260|
gi|31215551|ref|XP_316051.1|	lcl|At4g03965.1	lcl|F28B1.2 CE15914 WBGene0	gi|24658779|	gi|42659001|ref|XP_379678.1|	gi|6707358|ref|Os01g73270|
gi|31215567|ref|XP_316053.1|	lcl|At4g03975.1	lcl|F28B1.3 CE15915 WBGene0	gi|24658827|	gi|42659005|ref|XP_378087.1|	gi|6707364|ref|Os01g73330|
gi|31215576|ref|XP_316054.1|	lcl|At4g04155.1	lcl|F28B1.4 CE15916 WBGene0	gi|24658901|	gi|42659011|ref|XP_379643.1|	gi|6707367|ref|Os01g73360|
gi|31215652|ref|XP_316068.1|	lcl|At4g04160.1	lcl|F28B1.5 CE15917 WBGene0	gi|24658907|	gi|42659020|ref|XP_379644.1|	gi|6707371|ref|Os01g73400|
gi|31215677|ref|XP_316073.1|	lcl|At4g04165.1	lcl|F28B12.1 CE02702 WBGene	gi|24658910|	gi|42659022|ref|XP_376880.1|	gi|6707375|ref|Os01g73440|
gi|31215681|ref|XP_316074.1|	lcl|At4g04273.1	lcl|F28B3.10 CE28924 WBGene	gi|24658916|	gi|42659028|ref|XP_376885.1|	gi|6707380|ref|Os01g73490|
gi|31215689|ref|XP_316076.1|	lcl|At4g04276.1	lcl|F28B3.2 CE09740 WBGene0	gi|24658964|	gi|42659030|ref|XP_373305.2|	gi|6707397|ref|Os01g73640|
gi|31215696|ref|XP_316077.1|	lcl|At4g04394.1	lcl|F28B3.3 CE09741 WBGene0	gi|24658981|	gi|42659032|ref|XP_379645.1|	gi|6707398|ref|Os01g73650|
gi|31215730|ref|XP_316085.1|	lcl|At4g04396.1	lcl|F28B3.6a CE28925 WBGene	gi|24659010|	gi|42659036|ref|XP_379647.1|	gi|6707400|ref|Os01g73670|
gi|31215736|ref|XP_316086.1|	lcl|At4g04398.1	lcl|F28B3.6b CE33384 WBGene	gi|24659066|	gi|42659038|ref|XP_374313.2|	gi|6707401|ref|Os01g73670|
gi|31215757|ref|XP_316090.1|	lcl|At4g04400.1	lcl|F28B4.1 CE35510 WBGene0	gi|24659079|	gi|42659044|ref|XP_379682.1|	gi|6707412|ref|Os01g73780|
gi|31215760|ref|XP_316091.1|	lcl|At4g04407.1	lcl|F28B4.4 CE28223 WBGene0	gi|24659098|	gi|42659051|ref|XP_376931.1|	gi|6707414|ref|Os01g73800|
gi|31215771|ref|XP_316093.1|	lcl|At4g04423.1	lcl|F28C10.1 CE07154 WBGene	gi|24659119|	gi|42659060|ref|XP_376838.1|	gi|6707416|ref|Os01g73820|
gi|31215775|ref|XP_316094.1|	lcl|At4g04525.1	lcl|F28C12.1 CE33385 WBGene	gi|24659135|	gi|42659064|ref|XP_376840.1|	gi|6707417|ref|Os01g73830|
gi|31215815|ref|XP_316103.1|	lcl|At4g04550.1	lcl|F28C12.4 CE09751 WBGene	gi|24659201|	gi|42659068|ref|XP_376843.1|	gi|6707418|ref|Os01g73840|
gi|31215827|ref|XP_316105.1|	lcl|At4g04660.1	lcl|F28C12.6 CE09753 WBGene	gi|24659208|	gi|42659077|ref|XP_376850.1|	gi|6707420|ref|Os01g73860|
gi|31215853|ref|XP_316112.1|	lcl|At4g04730.1	lcl|F28C12.7 CE33386 WBGene	gi|24659221|	gi|42659079|ref|XP_378064.1|	gi|6707429|ref|Os01g73920|
gi|31215886|ref|XP_316119.1|	lcl|At4g04745.1	lcl|F28C6.10 CE09747 WBGene	gi|24659231|	gi|42659087|ref|XP_376856.1|	gi|6707431|ref|Os01g73940|
gi|31215906|ref|XP_316124.1|	lcl|At4g04820.1	lcl|F28C6.5 CE03276 WBGene0	gi|24659260|	gi|42659089|ref|XP_379638.1|	gi|6707448|ref|Os01g74100|
gi|31215925|ref|XP_316129.1|	lcl|At4g05070.1	lcl|F28C6.9 CE03280 WBGene0	gi|24659282|	gi|42659100|ref|XP_379640.1|	gi|6707450|ref|Os01g74120|
gi|31215927|ref|XP_316130.1|	lcl|At4g05095.1	lcl|F28D1.11 CE20732 WBGene	gi|24659329|	gi|42659110|ref|XP_379641.1|	gi|6707468|ref|Os01g74300|
gi|31215930|ref|XP_316131.1|	lcl|At4g05136.1	lcl|F28D1.2 CE05742 WBGene0	gi|24659343|	gi|42659114|ref|XP_379642.1|	gi|6707476|ref|Os01g74380|
gi|31215944|ref|XP_316134.1|	lcl|At4g05145.1	lcl|F28D1.6 CE05746 WBGene0	gi|24659350|	gi|42659122|ref|XP_379637.1|	gi|6707477|ref|Os01g74390|
gi|31215948|ref|XP_316135.1|	lcl|At4g05290.1	lcl|F28D9.4 CE23687 WBGene0	gi|24659448|	gi|42659133|ref|XP_379648.1|	gi|6707482|ref|Os01g74430|
gi|31215952|ref|XP_316136.1|	lcl|At4g05360.1	lcl|F28E10.1a CE37716 WBGen	gi|24659455|	gi|42659143|ref|XP_379684.1|	gi|6707510|ref|Os01g74670|
gi|31215957|ref|XP_316137.1|	lcl|At4g05370.1	lcl|F28E10.1b CE33631 WBGen	gi|24659500|	gi|42659149|ref|XP_379617.1|	gi|6800006|ref|Os02g01060|
gi|31215995|ref|XP_316145.1|	lcl|At4g05523.1	lcl|F28E10.1c CE33632 WBGen	gi|24659521|	gi|42659151|ref|XP_379618.1|	gi|6800014|ref|Os02g01120|
gi|31216041|ref|XP_316155.1|	lcl|At4g05553.1	lcl|F28E10.1d CE33633 WBGen	gi|24659529|	gi|42659153|ref|XP_379619.1|	gi|6800015|ref|Os02g01130|
gi|31216045|ref|XP_316156.1|	lcl|At4g05560.1	lcl|F28E10.4 CE04461 WBGene	gi|24659670|	gi|42659155|ref|XP_379622.1|	gi|6800031|ref|Os02g01290|
gi|31216094|ref|XP_316165.1|	lcl|At4g05580.1	lcl|F28F5.1 CE00716 WBGene0	gi|24659681|	gi|42659170|ref|XP_379625.1|	gi|6800034|ref|Os02g01320|
gi|31216099|ref|XP_316166.1|	lcl|At4g05581.1	lcl|F28F5.6 CE30772 WBGene0	gi|24659693|	gi|42659175|ref|XP_379627.1|	gi|6800039|ref|Os02g01370|
gi|31216110|ref|XP_316168.1|	lcl|At4g05583.1	lcl|F28F8.5 CE09759 WBGene0	gi|24659736|	gi|42659177|ref|XP_376822.1|	gi|6800043|ref|Os02g01410|
gi|31216123|ref|XP_316170.1|	lcl|At4g05616.1	lcl|F28F9.2 CE09763 WBGene0	gi|24659741|	gi|42659181|ref|XP_379628.1|	gi|6800055|ref|Os02g01530|
gi|31216157|ref|XP_316178.1|	lcl|At4g05630.1	lcl|F28F9.3 CE36590 WBGene0	gi|24659750|	gi|42659183|ref|XP_379629.1|	gi|6800062|ref|Os02g01600|
gi|31216204|ref|XP_316188.1|	lcl|At4g05631.1	lcl|F28H1.1 CE09766 WBGene0	gi|24659824|	gi|42659196|ref|XP_379630.1|	gi|6800066|ref|Os02g01630|
gi|31216223|ref|XP_316191.1|	lcl|At4g05632.1	lcl|F28H1.5 CE09770 WBGene0	gi|24659834|	gi|42659211|ref|XP_379635.1|	gi|6800069|ref|Os02g01660|
gi|31216226|ref|XP_316192.1|	lcl|At4g05633.1	lcl|F28H6.7 CE18652 WBGene0	gi|24659839|	gi|42659215|ref|XP_379636.1|	gi|6800073|ref|Os02g01700|
gi|31216232|ref|XP_316193.1|	lcl|At4g05636.1	lcl|F29A7.4 CE36835 WBGene0	gi|24659843|	gi|42659221|ref|XP_378199.1|	gi|6800078|ref|Os02g01750|
gi|31216301|ref|XP_316207.1|	lcl|At4g05640.1	lcl|F29A7.5 CE09776 WBGene0	gi|24659944|	gi|42659223|ref|XP_379656.1|	gi|6800080|ref|Os02g01770|
gi|31216305|ref|XP_316208.1|	lcl|At4g06475.1	lcl|F29A7.6 CE09777 WBGene0	gi|24660015|	gi|42659226|ref|XP_379657.1|	gi|6800081|ref|Os02g01780|
gi|31216310|ref|XP_316209.1|	lcl|At4g06476.1	lcl|F29B9.11 CE09789 WBGene	gi|24660020|	gi|42659245|ref|XP_379664.1|	gi|6800082|ref|Os02g01790|
gi|31216319|ref|XP_316210.1|	lcl|At4g06477.1	lcl|F29B9.7 CE09785 WBGene0	gi|24660025|	gi|42659247|ref|XP_379665.1|	gi|6800083|ref|Os02g01790|
gi|31216351|ref|XP_316214.1|	lcl|At4g06478.1	lcl|F29B9.8 CE09786 WBGene0	gi|24660031|	gi|42659255|ref|XP_379667.1|	gi|6800085|ref|Os02g01810|
gi|31216356|ref|XP_316215.1|	lcl|At4g06480.1	lcl|F29C12.1a CE19820 WBGen	gi|24660112|	gi|42659268|ref|XP_379668.1|	gi|6800089|ref|Os02g01850|
gi|31216404|ref|XP_316225.1|	lcl|At4g06490.1	lcl|F29C12.1b CE37902 WBGen	gi|24660246|	gi|42659270|ref|XP_372142.2|	gi|6800091|ref|Os02g01870|
gi|31216433|ref|XP_316231.1|	lcl|At4g06526.1	lcl|F29C12.3 CE20734 WBGene	gi|24660252|	gi|42659276|ref|XP_374306.2|	gi|6800094|ref|Os02g01900|
gi|31216471|ref|XP_316238.1|	lcl|At4g06597.1	lcl|F29C12.6 CE19824 WBGene	gi|24660308|	gi|42659278|ref|XP_379671.1|	gi|6800095|ref|Os02g01910|
gi|31216565|ref|XP_316258.1|	lcl|At4g06603.1	lcl|F29C4.4 CE36591 WBGene0	gi|24660345|	gi|42659280|ref|XP_379672.1|	gi|6800105|ref|Os02g02010|
gi|31216568|ref|XP_316259.1|	lcl|At4g06672.1	lcl|F29C6.1 CE13912 WBGene0	gi|24660375|	gi|42659300|ref|XP_379650.1|	gi|6800107|ref|Os02g02030|
gi|31216572|ref|XP_316260.1|	lcl|At4g06678.1	lcl|F29D10.1 CE05760 WBGene	gi|24660512|	gi|42659303|ref|XP_379651.1|	gi|6800112|ref|Os02g02070|
gi|31216578|ref|XP_316261.1|	lcl|At4g06680.1	lcl|F29D10.2 CE05761 WBGene	gi|24660544|	gi|42659309|ref|XP_379655.1|	gi|6800113|ref|Os02g02080|
gi|31216582|ref|XP_316262.1|	lcl|At4g06684.1	lcl|F29G6.3a CE36483 WBGene	gi|24660583|	gi|42659324|ref|XP_374847.1|	gi|6800121|ref|Os02g02160|
gi|31216587|ref|XP_316263.1|	lcl|At4g06688.1	lcl|F29G6.3b CE18655 WBGene	gi|24660587|	gi|42659326|ref|XP_378259.1|	gi|6800123|ref|Os02g02180|
gi|31216591|ref|XP_316264.1|	lcl|At4g06690.1	lcl|F29G6.3c CE36484 WBGene	gi|24660595|	gi|42659328|ref|XP_378260.1|	gi|6800129|ref|Os02g02240|
gi|31216655|ref|XP_316279.1|	lcl|At4g06706.1	lcl|F29G9.1 CE35297 WBGene0	gi|24660720|	gi|42659331|ref|XP_378257.1|	gi|6800131|ref|Os02g02260|
gi|31216686|ref|XP_316284.1|	lcl|At4g06716.1	lcl|F30A10.11 CE34833 WBGen	gi|24660724|	gi|42659333|ref|XP_378255.1|	gi|6800132|ref|Os02g02270|
gi|31216708|ref|XP_316288.1|	lcl|At4g06724.1	lcl|F30A10.12 CE34834 WBGen	gi|24660738|	gi|42659346|ref|XP_378240.1|	gi|6800133|ref|Os02g02270|
gi|31216766|ref|XP_316298.1|	lcl|At4g06728.1	lcl|F30A10.2 CE09803 WBGene	gi|24660742|	gi|42659362|ref|XP_378247.1|	gi|6800134|ref|Os02g02280|
gi|31216804|ref|XP_316303.1|	lcl|At4g06740.1	lcl|F30F8.1 CE09813 WBGene0	gi|24660750|	gi|42659378|ref|XP_378250.1|	gi|6800136|ref|Os02g02300|
gi|31216890|ref|XP_316321.1|	lcl|At4g07031.1	lcl|F30F8.5 CE37511 WBGene0	gi|24660754|	gi|42659382|ref|XP_378251.1|	gi|6800139|ref|Os02g02330|
gi|31216958|ref|XP_316334.1|	lcl|At4g07350.1	lcl|F30F8.7 CE09818 WBGene0	gi|24660788|	gi|42659403|ref|XP_210860.3|	gi|6800154|ref|Os02g02440|
gi|31217029|ref|XP_316347.1|	lcl|At4g07380.1	lcl|F30H5.2 CE01926 WBGene0	gi|24660954|	gi|42659411|ref|XP_378223.1|	gi|6800175|ref|Os02g02630|
gi|31217038|ref|XP_316349.1|	lcl|At4g07435.1	lcl|F31A3.3 CE07160 WBGene0	gi|24661014|	gi|42659417|ref|XP_378227.1|	gi|6800177|ref|Os02g02650|
gi|31217064|ref|XP_316355.1|	lcl|At4g07452.1	lcl|F31A3.5 CE35513 WBGene0	gi|24661077|	gi|42659423|ref|XP_374840.1|	gi|6800206|ref|Os02g02900|
gi|31217180|ref|XP_316378.1|	lcl|At4g07460.1	lcl|F31A9.1 CE07162 WBGene0	gi|24661128|	gi|42659428|ref|XP_378224.1|	gi|6800207|ref|Os02g02910|
gi|31217218|ref|XP_316386.1|	lcl|At4g07485.1	lcl|F31A9.2 CE07163 WBGene0	gi|24661158|	gi|42659438|ref|XP_378200.1|	gi|6800241|ref|Os02g03130|
gi|31217292|ref|XP_316400.1|	lcl|At4g07493.1	lcl|F31A9.4 CE07165 WBGene0	gi|24661162|	gi|42659440|ref|XP_378201.1|	gi|6800253|ref|Os02g03250|
gi|31217303|ref|XP_316403.1|	lcl|At4g07494.1	lcl|F31B12.2 CE03282 WBGene	gi|24661170|	gi|42659442|ref|XP_378202.1|	gi|6800258|ref|Os02g03300|
gi|31217307|ref|XP_316404.1|	lcl|At4g07500.1	lcl|F31B12.4 CE34836 WBGene	gi|24661173|	gi|42659444|ref|XP_378203.1|	gi|6800264|ref|Os02g03360|
gi|31217334|ref|XP_316409.1|	lcl|At4g07515.1	lcl|F31B9.2 CE17728 WBGene0	gi|24661177|	gi|42659446|ref|XP_378207.1|	gi|6800265|ref|Os02g03370|
gi|31217339|ref|XP_316410.1|	lcl|At4g07520.1	lcl|F31B9.3 CE17729 WBGene0	gi|24661229|	gi|42659451|ref|XP_378208.1|	gi|6800267|ref|Os02g03390|

gi|31217355|ref|XP_316412.1|	lcl|At4g07521.1	lcl|F31B9.4 CE34837 WBGene0	gi|24661322|	gi|42659453|ref|XP_374766.1|	gi|6800278|ref|Os02g03480|
gi|31217362|ref|XP_316413.1|	lcl|At4g07523.1	lcl|F31C3.6a CE24930 WBGene	gi|24661379|	gi|42659455|ref|XP_374767.1|	gi|6800279|ref|Os02g03490|
gi|31217387|ref|XP_316416.1|	lcl|At4g07524.1	lcl|F31D4.8 CE15934 WBGene0	gi|24661471|	gi|42659460|ref|XP_378210.1|	gi|6800280|ref|Os02g03500|
gi|31217436|ref|XP_316425.1|	lcl|At4g07525.1	lcl|F31D4.9 CE23694 WBGene0	gi|24661475|	gi|42659462|ref|XP_378211.1|	gi|6800281|ref|Os02g03510|
gi|31217446|ref|XP_316427.1|	lcl|At4g07526.1	lcl|F31D5.3c CE31797 WBGene	gi|24661495|	gi|42659466|ref|XP_374769.1|	gi|6800282|ref|Os02g03520|
gi|31217457|ref|XP_316430.1|	lcl|At4g07550.1	lcl|F31D5.3d CE31798 WBGene	gi|24661499|	gi|42659477|ref|XP_377136.1|	gi|6800295|ref|Os02g03640|
gi|31217486|ref|XP_316436.1|	lcl|At4g07650.1	lcl|F31E3.2c CE29301 WBGene	gi|24661597|	gi|42659486|ref|XP_378261.1|	gi|6800296|ref|Os02g03650|
gi|31217490|ref|XP_316437.1|	lcl|At4g07666.1	lcl|F31E3.6 CE29302 WBGene0	gi|24661662|	gi|42659490|ref|XP_377142.1|	gi|6800301|ref|Os02g03700|
gi|31217530|ref|XP_316444.1|	lcl|At4g07675.1	lcl|F31E8.1 CE01928 WBGene0	gi|24661666|	gi|42659498|ref|XP_378228.1|	gi|6800302|ref|Os02g03710|
gi|31217562|ref|XP_316449.1|	lcl|At4g07700.1	lcl|F31E8.5 CE35516 WBGene0	gi|24661674|	gi|42659503|ref|XP_378230.1|	gi|6800308|ref|Os02g03770|
gi|31217614|ref|XP_316462.1|	lcl|At4g07706.1	lcl|F31E9.4 CE15938 WBGene0	gi|24661726|	gi|42659512|ref|XP_378232.1|	gi|6800309|ref|Os02g03780|
gi|31217678|ref|XP_316478.1|	lcl|At4g07710.1	lcl|F31E9.6 CE23695 WBGene0	gi|24661738|	gi|42659523|ref|XP_378235.1|	gi|6800311|ref|Os02g03800|
gi|31217696|ref|XP_316482.1|	lcl|At4g07720.1	lcl|F31F6.2 CE05774 WBGene0	gi|24661864|	gi|42659525|ref|XP_378236.1|	gi|6800314|ref|Os02g03830|
gi|31217719|ref|XP_316488.1|	lcl|At4g07730.1	lcl|F31F6.4a CE05776 WBGene	gi|24661868|	gi|42659527|ref|XP_378238.1|	gi|6800323|ref|Os02g03920|
gi|31217743|ref|XP_316494.1|	lcl|At4g07740.1	lcl|F31F6.4b CE29960 WBGene	gi|24661904|	gi|42659540|ref|XP_378215.1|	gi|6800331|ref|Os02g04000|
gi|31217786|ref|XP_316503.1|	lcl|At4g07825.1	lcl|F31F7.3 CE09841 WBGene0	gi|24661970|	gi|42659550|ref|XP_374781.1|	gi|6800344|ref|Os02g04130|
gi|31217799|ref|XP_316506.1|	lcl|At4g07846.1	lcl|F32A11.3 CE17739 WBGene	gi|24662024|	gi|42659559|ref|XP_378218.1|	gi|6800351|ref|Os02g04190|
gi|31217805|ref|XP_316507.1|	lcl|At4g07868.1	lcl|F32A6.1 CE04469 WBGene0	gi|24662028|	gi|42659561|ref|XP_378219.1|	gi|6800353|ref|Os02g04210|
gi|31217817|ref|XP_316512.1|	lcl|At4g07880.1	lcl|F32A7.5a CE09846 WBGene	gi|24662105|	gi|42659566|ref|XP_374786.1|	gi|6800354|ref|Os02g04220|
gi|31217828|ref|XP_316516.1|	lcl|At4g07890.1	lcl|F32A7.5b CE34178 WBGene	gi|24662109|	gi|42659572|ref|XP_370584.2|	gi|6800368|ref|Os02g04350|
gi|31217861|ref|XP_316520.1|	lcl|At4g07900.1	lcl|F32A7.5c CE34179 WBGene	gi|24662113|	gi|42659580|ref|XP_378308.1|	gi|6800369|ref|Os02g04360|
gi|31217864|ref|XP_316521.1|	lcl|At4g07910.1	lcl|F32B4.5 CE17745 WBGene0	gi|24662121|	gi|42659589|ref|XP_374932.1|	gi|6800370|ref|Os02g04370|
gi|31217885|ref|XP_316526.1|	lcl|At4g07920.1	lcl|F32B5.2 CE36147 WBGene0	gi|24662143|	gi|42659595|ref|XP_378309.1|	gi|6800371|ref|Os02g04380|
gi|31217903|ref|XP_316528.1|	lcl|At4g07930.1	lcl|F32B5.4 CE09852 WBGene0	gi|24662147|	gi|42659597|ref|XP_378311.1|	gi|6800372|ref|Os02g04390|
gi|31217924|ref|XP_316531.1|	lcl|At4g07932.1	lcl|F32B5.6c CE33387 WBGene	gi|24662151|	gi|42659599|ref|XP_378312.1|	gi|6800373|ref|Os02g04400|
gi|31217928|ref|XP_316532.1|	lcl|At4g07940.1	lcl|F32B5.6d CE36150 WBGene	gi|24662155|	gi|42659607|ref|XP_378313.1|	gi|6800374|ref|Os02g04410|
gi|31217934|ref|XP_316533.1|	lcl|At4g07943.1	lcl|F32B6.4 CE20742 WBGene0	gi|24662197|	gi|42659611|ref|XP_374937.1|	gi|6800375|ref|Os02g04420|
gi|31217938|ref|XP_316534.1|	lcl|At4g07944.1	lcl|F32B6.5 CE09860 WBGene0	gi|24662209|	gi|42659615|ref|XP_378316.1|	gi|6800376|ref|Os02g04420|
gi|31217976|ref|XP_316543.1|	lcl|At4g07965.1	lcl|F32D1.6 CE09870 WBGene0	gi|24662216|	gi|42659620|ref|XP_378317.1|	gi|6800377|ref|Os02g04420|
gi|31218028|ref|XP_316551.1|	lcl|At4g07970.1	lcl|F32D1.7 CE09871 WBGene0	gi|24662243|	gi|42659630|ref|XP_378320.1|	gi|6800388|ref|Os02g04520|
gi|31218209|ref|XP_316613.1|	lcl|At4g08013.1	lcl|F32D1.8 CE35301 WBGene0	gi|24662262|	gi|42659632|ref|XP_378321.1|	gi|6800394|ref|Os02g04570|
gi|31218244|ref|XP_316631.1|	lcl|At4g08025.1	lcl|F32D8.2 CE05781 WBGene0	gi|24662274|	gi|42659637|ref|XP_378325.1|	gi|6800395|ref|Os02g04580|
gi|31218255|ref|XP_316634.1|	lcl|At4g08097.1	lcl|F32E10.7 CE24935 WBGene	gi|24662278|	gi|42659641|ref|XP_378326.1|	gi|6800397|ref|Os02g04600|
gi|31218321|ref|XP_316645.1|	lcl|At4g08098.1	lcl|F32E10.9 CE37717 WBGene	gi|24662313|	gi|42659645|ref|XP_378327.1|	gi|6800398|ref|Os02g04610|
gi|31218421|ref|XP_316658.1|	lcl|At4g08110.1	lcl|F32G8.2 CE05791 WBGene0	gi|24662337|	gi|42659656|ref|XP_374885.1|	gi|6800399|ref|Os02g04620|
gi|31218437|ref|XP_316660.1|	lcl|At4g08111.1	lcl|F32G8.3 CE05792 WBGene0	gi|24662376|	gi|42659669|ref|XP_374890.1|	gi|6800410|ref|Os02g04720|
gi|31218451|ref|XP_316662.1|	lcl|At4g08113.1	lcl|F32H2.3 CE09878 WBGene0	gi|24662380|	gi|42659674|ref|XP_374893.1|	gi|6800415|ref|Os02g04770|
gi|31218535|ref|XP_316673.1|	lcl|At4g08115.1	lcl|F32H2.7 CE09882 WBGene0	gi|24662384|	gi|42659686|ref|XP_378297.1|	gi|6800417|ref|Os02g04790|
gi|31218550|ref|XP_316675.1|	lcl|At4g08130.1	lcl|F32H5.3a CE17751 WBGene	gi|24662386|	gi|42659717|ref|XP_378300.1|	gi|6800423|ref|Os02g04850|
gi|31218601|ref|XP_316683.1|	lcl|At4g08240.1	lcl|F32H5.3b CE09887 WBGene	gi|24662495|	gi|42659723|ref|XP_374906.1|	gi|6800425|ref|Os02g04870|
gi|31218613|ref|XP_316686.1|	lcl|At4g08240.2	lcl|F32H5.5 CE33066 WBGene0	gi|24662499|	gi|42659725|ref|XP_378301.1|	gi|6800426|ref|Os02g04880|
gi|31218616|ref|XP_316687.1|	lcl|At4g08270.1	lcl|F32H5.8 CE31926 WBGene0	gi|24662503|	gi|42659742|ref|XP_378303.1|	gi|6800427|ref|Os02g04890|
gi|31218671|ref|XP_316695.1|	lcl|At4g08336.1	lcl|F33A8.10 CE34840 WBGene	gi|24662543|	gi|42659744|ref|XP_378304.1|	gi|6800428|ref|Os02g04900|
gi|31218706|ref|XP_316700.1|	lcl|At4g08370.1	lcl|F33A8.2 CE32885 WBGene0	gi|24662547|	gi|42659752|ref|XP_379741.1|	gi|6800434|ref|Os02g04960|
gi|31218711|ref|XP_316701.1|	lcl|At4g08380.1	lcl|F33A8.7 CE23701 WBGene0	gi|24662551|	gi|42659758|ref|XP_374857.1|	gi|6800437|ref|Os02g04990|
gi|31218724|ref|XP_316703.1|	lcl|At4g08395.1	lcl|F33A8.9 CE35183 WBGene0	gi|24662630|	gi|42659760|ref|XP_374858.1|	gi|6800438|ref|Os02g05000|
gi|31218730|ref|XP_316704.1|	lcl|At4g08400.1	lcl|F33C8.2 CE28555 WBGene0	gi|24662634|	gi|42659762|ref|XP_378262.1|	gi|6800439|ref|Os02g05010|
gi|31218760|ref|XP_316707.1|	lcl|At4g08410.1	lcl|F33C8.4 CE37513 WBGene0	gi|24662696|	gi|42659770|ref|XP_378299.1|	gi|6800445|ref|Os02g05070|
gi|31218818|ref|XP_316715.1|	lcl|At4g08510.1	lcl|F33D11.2 CE09892 WBGene	gi|24662704|	gi|42659790|ref|XP_378266.1|	gi|6800446|ref|Os02g05080|
gi|31218831|ref|XP_316717.1|	lcl|At4g08555.1	lcl|F33D11.6 CE09896 WBGene	gi|24662761|	gi|42659794|ref|XP_374864.1|	gi|6800447|ref|Os02g05090|
gi|31218843|ref|XP_316719.1|	lcl|At4g08593.1	lcl|F33D11.8 CE29785 WBGene	gi|24662834|	gi|42659805|ref|XP_378271.1|	gi|6800450|ref|Os02g05120|
gi|31218849|ref|XP_316720.1|	lcl|At4g08600.1	lcl|F33D11.9a CE37904 WBGen	gi|24662858|	gi|42659807|ref|XP_378272.1|	gi|6800451|ref|Os02g05130|
gi|31218921|ref|XP_316729.1|	lcl|At4g08605.1	lcl|F33D4.6a CE33634 WBGene	gi|24662869|	gi|42659809|ref|XP_378273.1|	gi|6800452|ref|Os02g05140|
gi|31218930|ref|XP_316730.1|	lcl|At4g08655.1	lcl|F33D4.6b CE37228 WBGene	gi|24662899|	gi|42659815|ref|XP_378276.1|	gi|6800457|ref|Os02g05190|
gi|31218983|ref|XP_316736.1|	lcl|At4g08710.1	lcl|F33E11.2 CE28293 WBGene	gi|24662903|	gi|42659819|ref|XP_378277.1|	gi|6800458|ref|Os02g05200|
gi|31219039|ref|XP_316743.1|	lcl|At4g08730.1	lcl|F33E2.4 CE34180 WBGene0	gi|24662973|	gi|42659823|ref|XP_378279.1|	gi|6800459|ref|Os02g05210|
gi|31219068|ref|XP_316747.1|	lcl|At4g08740.1	lcl|F33E2.5 CE09906 WBGene0	gi|24662981|	gi|42659825|ref|XP_378280.1|	gi|6800462|ref|Os02g05240|
gi|31219073|ref|XP_316748.1|	lcl|At4g08760.1	lcl|F33H1.3 CE01569 WBGene0	gi|24663011|	gi|42659834|ref|XP_378283.1|	gi|6800463|ref|Os02g05250|
gi|31219100|ref|XP_316752.1|	lcl|At4g08874.1	lcl|F33H1.6 CE37907 WBGene0	gi|24663135|	gi|42659839|ref|XP_378286.1|	gi|6800465|ref|Os02g05270|
gi|31219107|ref|XP_316753.1|	lcl|At4g08876.1	lcl|F33H2.8 CE37514 WBGene0	gi|24663224|	gi|42659841|ref|XP_378288.1|	gi|6800466|ref|Os02g05280|
gi|31219135|ref|XP_316758.1|	lcl|At4g09030.1	lcl|F34D10.3 CE00963 WBGene	gi|24663228|	gi|42659843|ref|XP_374877.1|	gi|6800467|ref|Os02g05290|
gi|31219148|ref|XP_316760.1|	lcl|At4g09170.1	lcl|F34D10.4 CE17770 WBGene	gi|24663296|	gi|42659893|ref|XP_378371.1|	gi|6800468|ref|Os02g05300|
gi|31219154|ref|XP_316761.1|	lcl|At4g09210.1	lcl|F34D10.8 CE33636 WBGene	gi|24663352|	gi|42659895|ref|XP_378372.1|	gi|6800473|ref|Os02g05350|
gi|31219160|ref|XP_316762.1|	lcl|At4g09220.1	lcl|F34H10.2 CE01572 WBGene	gi|24663388|	gi|42659897|ref|XP_378374.1|	gi|6800474|ref|Os02g05360|
gi|31219178|ref|XP_316764.1|	lcl|At4g09260.1	lcl|F34H10.3 CE01573 WBGene	gi|24663454|	gi|42659904|ref|XP_378379.1|	gi|6800480|ref|Os02g05420|
gi|31219206|ref|XP_316768.1|	lcl|At4g09270.1	lcl|F34H10.4 CE01574 WBGene	gi|24663458|	gi|42659915|ref|XP_378381.1|	gi|6800481|ref|Os02g05430|
gi|31219212|ref|XP_316769.1|	lcl|At4g09290.1	lcl|F34H10.5 CE34842 WBGene	gi|24663462|	gi|42659934|ref|XP_378353.1|	gi|6800484|ref|Os02g05460|
gi|31219220|ref|XP_316770.1|	lcl|At4g09390.1	lcl|F35A5.1 CE04485 WBGene0	gi|24663466|	gi|42659936|ref|XP_378354.1|	gi|6800494|ref|Os02g05530|
gi|31219227|ref|XP_316771.1|	lcl|At4g09700.1	lcl|F35A5.2 CE04486 WBGene0	gi|24663548|	gi|42659945|ref|XP_378355.1|	gi|6800495|ref|Os02g05540|
gi|31219257|ref|XP_316775.1|	lcl|At4g09840.1	lcl|F35A5.4 CE37642 WBGene0	gi|24663556|	gi|42659956|ref|XP_378358.1|	gi|6800508|ref|Os02g05650|
gi|31219280|ref|XP_316778.1|	lcl|At4g09850.1	lcl|F35A5.5 CE04489 WBGene0	gi|24663564|	gi|42659958|ref|XP_378360.1|	gi|6800510|ref|Os02g05670|
gi|31219326|ref|XP_316785.1|	lcl|At4g09860.1	lcl|F35B12.3 CE31929 WBGene	gi|24663572|	gi|42659973|ref|XP_378362.1|	gi|6800520|ref|Os02g05760|
gi|31219331|ref|XP_316786.1|	lcl|At4g09880.1	lcl|F35B12.5 CE27758 WBGene	gi|24663576|	gi|42659975|ref|XP_378363.1|	gi|6800521|ref|Os02g05770|
gi|31219409|ref|XP_316796.1|	lcl|At4g09970.1	lcl|F35B12.7 CE37515 WBGene	gi|24663679|	gi|42659980|ref|XP_378365.1|	gi|6800523|ref|Os02g05790|
gi|31219431|ref|XP_316799.1|	lcl|At4g09970.2	lcl|F35B12.9 CE31931 WBGene	gi|24663691|	gi|42659982|ref|XP_378366.1|	gi|6800525|ref|Os02g05810|
gi|31219447|ref|XP_316801.1|	lcl|At4g10230.1	lcl|F35B3.3 CE07170 WBGene0	gi|24663698|	gi|42659985|ref|XP_378367.1|	gi|6800534|ref|Os02g05890|
gi|31219471|ref|XP_316804.1|	lcl|At4g10290.1	lcl|F35C11.1 CE02208 WBGene	gi|24663710|	gi|42659987|ref|XP_378368.1|	gi|6800552|ref|Os02g06060|
gi|31219478|ref|XP_316805.1|	lcl|At4g10330.1	lcl|F35C11.4 CE03284 WBGene	gi|24663722|	gi|42659995|ref|XP_378398.1|	gi|6800554|ref|Os02g06080|
gi|31219485|ref|XP_316806.1|	lcl|At4g10470.1	lcl|F35C11.6 CE02212 WBGene	gi|24663726|	gi|42660000|ref|XP_378336.1|	gi|6800563|ref|Os02g06170|
gi|31219493|ref|XP_316807.1|	lcl|At4g10660.1	lcl|F35C12.1 CE09913 WBGene	gi|24663740|	gi|42660012|ref|XP_378339.1|	gi|6800577|ref|Os02g06310|
gi|31219500|ref|XP_316808.1|	lcl|At4g10700.1	lcl|F35C5.1 CE35520 WBGene0	gi|24663744|	gi|42660016|ref|XP_378340.1|	gi|6800589|ref|Os02g06420|
gi|31219571|ref|XP_316815.1|	lcl|At4g10810.1	lcl|F35C5.10 CE15951 WBGene	gi|24663755|	gi|42660020|ref|XP_378343.1|	gi|6800591|ref|Os02g06440|
gi|31219617|ref|XP_316822.1|	lcl|At4g10820.1	lcl|F35C5.4 CE15944 WBGene0	gi|24663791|	gi|42660033|ref|XP_378346.1|	gi|6800592|ref|Os02g06450|
gi|31219786|ref|XP_316833.1|	lcl|At4g10845.1	lcl|F35C8.8 CE04500 WBGene0	gi|24663799|	gi|42660038|ref|XP_378349.1|	gi|6800596|ref|Os02g06480|
gi|31219818|ref|XP_316838.1|	lcl|At4g10860.1	lcl|F35D11.1 CE02712 WBGene	gi|24663820|	gi|42660056|ref|XP_378389.1|	gi|6800599|ref|Os02g06510|
gi|31219825|ref|XP_316839.1|	lcl|At4g10870.1	lcl|F35D11.2a CE32647 WBGen	gi|24663913|	gi|42660058|ref|XP_378390.1|	gi|6800604|ref|Os02g06560|
gi|31219839|ref|XP_316841.1|	lcl|At4g10880.1	lcl|F35D11.2b CE31802 WBGen	gi|24663924|	gi|42660061|ref|XP_378392.1|	gi|6800609|ref|Os02g06610|
gi|31219871|ref|XP_316845.1|	lcl|At4g10910.1	lcl|F35D11.5 CE29789 WBGene	gi|24663930|	gi|42660064|ref|XP_378393.1|	gi|6800617|ref|Os02g06680|
gi|31219901|ref|XP_316849.1|	lcl|At4g11020.1	lcl|F35D2.1 CE02723 WBGene0	gi|24663934|	gi|42660066|ref|XP_378394.1|	gi|6800620|ref|Os02g06710|
gi|31220047|ref|XP_316869.1|	lcl|At4g11100.1	lcl|F35D2.2 CE36839 WBGene0	gi|24664000|	gi|42660068|ref|XP_290579.3|	gi|6800623|ref|Os02g06730|
gi|31220122|ref|XP_316878.1|	lcl|At4g11385.1	lcl|F35E12.9 CE32404 WBGene	gi|24664004|	gi|42660073|ref|XP_378328.1|	gi|6800626|ref|Os02g06760|
gi|31220139|ref|XP_316881.1|	lcl|At4g11510.1	lcl|F35F10.14 CE35866 WBGen	gi|24664058|	gi|42660075|ref|XP_378329.1|	gi|6800627|ref|Os02g06770|
gi|31220156|ref|XP_316884.1|	lcl|At4g11780.1	lcl|F35F11.2 CE24943 WBGene	gi|24664113|	gi|42660077|ref|XP_378330.1|	gi|6800628|ref|Os02g06770|
gi|31220184|ref|XP_316888.1|	lcl|At4g11870.1	lcl|F35F11.3 CE24944 WBGene	gi|24664132|	gi|42660079|ref|XP_378331.1|	gi|6800629|ref|Os02g06780|
gi|31220217|ref|XP_316893.1|	lcl|At4g11930.1	lcl|F35G12.12 CE31499 WBGen	gi|24664136|	gi|42660081|ref|XP_378332.1|	gi|6800630|ref|Os02g06790|
gi|31220277|ref|XP_316900.1|	lcl|At4g11940.1	lcl|F35G12.5 CE00974 WBGene	gi|24664154|	gi|42660083|ref|XP_374952.1|	gi|6800631|ref|Os02g06800|
gi|31220284|ref|XP_316901.1|	lcl|At4g12005.1	lcl|F35G2.3 CE05810 WBGene0	gi|24664158|	gi|42660093|ref|XP_373549.2|	gi|6800636|ref|Os02g06850|
gi|31220295|ref|XP_316903.1|	lcl|At4g12220.1	lcl|F35G2.5 CE35524 WBGene0	gi|24664162|	gi|42660095|ref|XP_374999.1|	gi|6800640|ref|Os02g06900|
gi|31220303|ref|XP_316904.1|	lcl|At4g12350.1	lcl|F35H10.2 CE04502 WBGene	gi|24664183|	gi|42660101|ref|XP_378388.1|	gi|6800648|ref|Os02g06960|
gi|31220314|ref|XP_316906.1|	lcl|At4g12380.1	lcl|F35H10.3 CE36592 WBGene	gi|24664203|	gi|42660113|ref|XP_375033.1|	gi|6800649|ref|Os02g06970|
gi|31220337|ref|XP_316909.1|	lcl|At4g12540.1	lcl|F35H10.5 CE04505 WBGene	gi|24664207|	gi|42660117|ref|XP_378428.1|	gi|6800650|ref|Os02g06980|
gi|31220346|ref|XP_316910.1|	lcl|At4g12580.1	lcl|F35H12.1 CE04510 WBGene	gi|24664241|	gi|42660123|ref|XP_378430.1|	gi|6800651|ref|Os02g06990|
gi|31220375|ref|XP_316914.1|	lcl|At4g12930.1	lcl|F35H12.6 CE30973 WBGene	gi|24664245|	gi|42660128|ref|XP_378431.1|	gi|6800652|ref|Os02g07000|
gi|31220408|ref|XP_316917.1|	lcl|At4g12940.1	lcl|F35H12.7 CE30974 WBGene	gi|24664253|	gi|42660133|ref|XP_375007.1|	gi|6800653|ref|Os02g07010|
gi|31220453|ref|XP_316923.1|	lcl|At4g12990.1	lcl|F35H8.1 CE35867 WBGene0	gi|24664257|	gi|42660134|ref|XP_116980.4|	gi|6800654|ref|Os02g07020|
gi|31220460|ref|XP_316924.1|	lcl|At4g13150.1	lcl|F35H8.4 CE35868 WBGene0	gi|24664261|	gi|42660136|ref|XP_167908.2|	gi|6800667|ref|Os02g07130|
gi|31220465|ref|XP_316925.1|	lcl|At4g13195.1	lcl|F36A2.10 CE09949 WBGene	gi|24664351|	gi|42660138|ref|XP_378399.1|	gi|6800668|ref|Os02g07140|
gi|31220494|ref|XP_316928.1|	lcl|At4g13235.1	lcl|F36A2.11 CE34181 WBGene	gi|24664365|	gi|42660144|ref|XP_378404.1|	gi|6800690|ref|Os02g07300|
gi|31220531|ref|XP_316933.1|	lcl|At4g13320.1	lcl|F36A2.12 CE37232 WBGene	gi|24664403|	gi|42660147|ref|XP_375013.1|	gi|6800692|ref|Os02g07320|
gi|31220567|ref|XP_316936.1|	lcl|At4g13390.1	lcl|F36A2.14 CE37233 WBGene	gi|24664407|	gi|42660150|ref|XP_378405.1|	gi|6800699|ref|Os02g07390|
gi|31220576|ref|XP_316938.1|	lcl|At4g13470.1	lcl|F36A2.7 CE09946 WBGene0	gi|24664411|	gi|42660153|ref|XP_378407.1|	gi|6800704|ref|Os02g07440|
gi|31220599|ref|XP_316943.1|	lcl|At4g13520.1	lcl|F36A4.11 CE07186 WBGene	gi|24664415|	gi|42660158|ref|XP_378410.1|	gi|6800706|ref|Os02g07460|
gi|31220659|ref|XP_316951.1|	lcl|At4g13540.1	lcl|F36D1.4 CE18665 WBGene0	gi|24664419|	gi|42660160|ref|XP_375018.1|	gi|6800708|ref|Os02g07470|
gi|31220714|ref|XP_316959.1|	lcl|At4g13615.1	lcl|F36D1.5 CE34305 WBGene0	gi|24664423|	gi|42660165|ref|XP_378411.1|	gi|6800710|ref|Os02g07500|
gi|31220735|ref|XP_316962.1|	lcl|At4g13690.1	lcl|F36D1.7 CE18668 WBGene0	gi|24664430|	gi|42660167|ref|XP_378412.1|	gi|6800718|ref|Os02g07580|
gi|31220753|ref|XP_316964.1|	lcl|At4g14020.1	lcl|F36D1.9 CE36434 WBGene0	gi|24664498|	gi|42660170|ref|XP_378413.1|	gi|6800719|ref|Os02g07590|
gi|31220762|ref|XP_316965.1|	lcl|At4g14120.1	lcl|F36D3.1 CE25872 WBGene0	gi|24664502|	gi|42660174|ref|XP_378414.1|	gi|6800724|ref|Os02g07640|
gi|31220793|ref|XP_316969.1|	lcl|At4g14165.1	lcl|F36D4.1 CE33072 WBGene0	gi|24664506|	gi|42660176|ref|XP_375023.1|	gi|6800730|ref|Os02g07690|
gi|31220832|ref|XP_316972.1|	lcl|At4g14200.1	lcl|F36D4.6 CE07194 WBGene0	gi|24664510|	gi|42660179|ref|XP_378416.1|	gi|6800731|ref|Os02g07700|
gi|31220850|ref|XP_316974.1|	lcl|At4g14380.1	lcl|F36F12.3 CE35869 WBGene	gi|24664518|	gi|42660187|ref|XP_375027.1|	gi|6800732|ref|Os02g07710|
gi|31220861|ref|XP_316975.1|	lcl|At4g14450.1	lcl|F36F12.4 CE17784 WBGene	gi|24664600|	gi|42660189|ref|XP_378419.1|	gi|6800743|ref|Os02g07800|
gi|31220908|ref|XP_316981.1|	lcl|At4g14530.1	lcl|F36F12.7 CE17787 WBGene	gi|24664621|	gi|42660195|ref|XP_378421.1|	gi|6800764|ref|Os02g07990|
gi|31220913|ref|XP_316982.1|	lcl|At4g14650.1	lcl|F36F2.2 CE15976 WBGene0	gi|24664639|	gi|42660197|ref|XP_375031.1|	gi|6800765|ref|Os02g08000|
gi|31220921|ref|XP_316983.1|	lcl|At4g14810.1	lcl|F36F2.7 CE34845 WBGene0	gi|24664740|	gi|42660208|ref|XP_378434.1|	gi|6800767|ref|Os02g08020|
gi|31220947|ref|XP_316986.1|	lcl|At4g15096.1	lcl|F36F2.8 CE34846 WBGene0	gi|24664759|	gi|42660210|ref|XP_378436.1|	gi|6800769|ref|Os02g08040|
gi|31220955|ref|XP_316987.1|	lcl|At4g15120.1	lcl|F36G3.1 CE15979 WBGene0	gi|24664802|	gi|42660212|ref|XP_378437.1|	gi|6800770|ref|Os02g08050|
gi|31220962|ref|XP_316988.1|	lcl|At4g15150.1	lcl|F36G9.7 CE15984 WBGene0	gi|24664852|	gi|42660214|ref|XP_375038.1|	gi|6800771|ref|Os02g08060|
gi|31220971|ref|XP_316989.1|	lcl|At4g15460.1	lcl|F36H1.10 CE34850 WBGene	gi|24664860|	gi|42660216|ref|XP_370733.2|	gi|6800773|ref|Os02g08080|
gi|31220978|ref|XP_316990.1|	lcl|At4g15563.1	lcl|F36H1.11 CE34851 WBGene	gi|24664864|	gi|42660218|ref|XP_378439.1|	gi|6800785|ref|Os02g08160|
gi|31220985|ref|XP_316991.1|	lcl|At4g15620.1	lcl|F36H1.12 CE34852 WBGene	gi|24664998|	gi|42660225|ref|XP_378441.1|	gi|6800786|ref|Os02g08170|
gi|31220989|ref|XP_316992.1|	lcl|At4g15650.1	lcl|F36H1.5 CE05816 WBGene0	gi|24665078|	gi|42660227|ref|XP_377306.1|	gi|6800790|ref|Os02g08210|
gi|31220996|ref|XP_316993.1|	lcl|At4g15710.1	lcl|F36H1.9 CE34853 WBGene0	gi|24665081|	gi|42660250|ref|XP_378449.1|	gi|6800791|ref|Os02g08220|
gi|31221025|ref|XP_316996.1|	lcl|At4g15765.1	lcl|F36H12.1 CE09973 WBGene	gi|24665109|	gi|42660259|ref|XP_378452.1|	gi|6800794|ref|Os02g08250|
gi|31221038|ref|XP_316998.1|	lcl|At4g15820.1	lcl|F36H12.13 CE09983 WBGen	gi|24665112|	gi|42660261|ref|XP_375076.1|	gi|6800812|ref|Os02g08430|

gi|31221046|ref|XP_316999.1|	lcl|At4g15950.1	lcl|F36H12.14 CE09984 WBGen	gi|24665115|	gi|42660265|ref|XP_378453.1|	gi|6800815|ref|Os02g08460|
gi|31221054|ref|XP_317000.1|	lcl|At4g15990.1	lcl|F36H12.15 CE09985 WBGen	gi|24665119|	gi|42660275|ref|XP_378455.1|	gi|6800816|ref|Os02g08470|
gi|31221065|ref|XP_317001.1|	lcl|At4g16000.1	lcl|F36H12.16 CE09986 WBGen	gi|24665128|	gi|42660277|ref|XP_378456.1|	gi|6800825|ref|Os02g09050|
gi|31221103|ref|XP_317007.1|	lcl|At4g16040.1	lcl|F36H12.17 CE09987 WBGen	gi|24665132|	gi|42660280|ref|XP_378457.1|	gi|6800830|ref|Os02g09090|
gi|31221121|ref|XP_317009.1|	lcl|At4g16090.1	lcl|F36H12.2 CE34854 WBGene	gi|24665135|	gi|42660288|ref|XP_375081.1|	gi|6800835|ref|Os02g09140|
gi|31221144|ref|XP_317012.1|	lcl|At4g16095.1	lcl|F36H12.4 CE31691 WBGene	gi|24665141|	gi|42660290|ref|XP_378460.1|	gi|6800837|ref|Os02g09160|
gi|31221230|ref|XP_317023.1|	lcl|At4g16140.1	lcl|F36H12.5 CE09977 WBGene	gi|24665144|	gi|42660300|ref|XP_378462.1|	gi|6800843|ref|Os02g09210|
gi|31221314|ref|XP_317031.1|	lcl|At4g16141.1	lcl|F36H5.10 CE29040 WBGene	gi|24665147|	gi|42660306|ref|XP_375088.1|	gi|6800845|ref|Os02g09230|
gi|31221323|ref|XP_317034.1|	lcl|At4g16215.1	lcl|F36H5.4 CE09961 WBGene0	gi|24665151|	gi|42660314|ref|XP_378465.1|	gi|6800850|ref|Os02g09280|
gi|31221331|ref|XP_317035.1|	lcl|At4g16240.1	lcl|F36H9.5 CE09971 WBGene0	gi|24665154|	gi|42660326|ref|XP_378467.1|	gi|6800852|ref|Os02g09300|
gi|31221337|ref|XP_317036.1|	lcl|At4g16400.1	lcl|F36H9.8 CE33080 WBGene0	gi|24665157|	gi|42660330|ref|XP_378470.1|	gi|6800873|ref|Os02g09500|
gi|31221386|ref|XP_317040.1|	lcl|At4g16447.1	lcl|F37A4.3 CE00709 WBGene0	gi|24665160|	gi|42660332|ref|XP_375099.1|	gi|6800875|ref|Os02g09520|
gi|31221400|ref|XP_317041.1|	lcl|At4g16460.1	lcl|F37A4.6 CE00706 WBGene0	gi|24665182|	gi|42660337|ref|XP_378473.1|	gi|6800877|ref|Os02g09540|
gi|31221406|ref|XP_317042.1|	lcl|At4g16515.1	lcl|F37A8.1 CE00986 WBGene0	gi|24665186|	gi|42660344|ref|XP_378476.1|	gi|6800880|ref|Os02g09570|
gi|31221495|ref|XP_317053.1|	lcl|At4g16610.1	lcl|F37A8.2 CE20754 WBGene0	gi|24665209|	gi|42660346|ref|XP_378477.1|	gi|6800886|ref|Os02g09630|
gi|31221519|ref|XP_317056.1|	lcl|At4g16695.1	lcl|F37A8.4 CE00989 WBGene0	gi|24665213|	gi|42660351|ref|XP_378482.1|	gi|6800887|ref|Os02g09640|
gi|31221625|ref|XP_317067.1|	lcl|At4g16790.1	lcl|F37B12.1 CE03289 WBGene	gi|24665217|	gi|42660354|ref|XP_096733.3|	gi|6800889|ref|Os02g09660|
gi|31221632|ref|XP_317068.1|	lcl|At4g16840.1	lcl|F37B4.10 CE17799 WBGene	gi|24665300|	gi|42660357|ref|XP_211088.2|	gi|6800894|ref|Os02g09710|
gi|31221663|ref|XP_317072.1|	lcl|At4g16980.1	lcl|F37C12.10 CE00823 WBGen	gi|24665303|	gi|42660361|ref|XP_378487.1|	gi|6800896|ref|Os02g09730|
gi|31221808|ref|XP_317092.1|	lcl|At4g17250.1	lcl|F37C12.13b CE30780 WBGe	gi|24665335|	gi|42660366|ref|XP_378488.1|	gi|6800899|ref|Os02g09760|
gi|31221847|ref|XP_317099.1|	lcl|At4g17310.1	lcl|F37C12.14 CE00822 WBGen	gi|24665360|	gi|42660368|ref|XP_378489.1|	gi|6800909|ref|Os02g09850|
gi|31221858|ref|XP_317100.1|	lcl|At4g17440.1	lcl|F37C4.4a CE17047 WBGene	gi|24665402|	gi|42660372|ref|XP_370779.2|	gi|6800910|ref|Os02g09860|
gi|31222024|ref|XP_317103.1|	lcl|At4g17590.1	lcl|F37C4.4b CE31505 WBGene	gi|24665469|	gi|42660374|ref|XP_378490.1|	gi|6800912|ref|Os02g09880|
gi|31222030|ref|XP_317104.1|	lcl|At4g17840.1	lcl|F37C4.5a CE17048 WBGene	gi|24665489|	gi|42660380|ref|XP_378491.1|	gi|6800927|ref|Os02g10030|
gi|31222036|ref|XP_317105.1|	lcl|At4g17930.1	lcl|F37C4.5b CE33640 WBGene	gi|24665493|	gi|42660402|ref|XP_375171.1|	gi|6800942|ref|Os02g10170|
gi|31222071|ref|XP_317110.1|	lcl|At4g18000.1	lcl|F37C4.7 CE17050 WBGene0	gi|24665569|	gi|42660404|ref|XP_375174.1|	gi|6800947|ref|Os02g10210|
gi|31222095|ref|XP_317118.1|	lcl|At4g18070.1	lcl|F37H8.1 CE15994 WBGene0	gi|24665591|	gi|42660417|ref|XP_378506.1|	gi|6800951|ref|Os02g10250|
gi|31222142|ref|XP_317133.1|	lcl|At4g18070.2	lcl|F37H8.2 CE23705 WBGene0	gi|24665607|	gi|42660419|ref|XP_375183.1|	gi|6800959|ref|Os02g10330|
gi|31222147|ref|XP_317134.1|	lcl|At4g18080.1	lcl|F38A1.13 CE10026 WBGene	gi|24665626|	gi|42660429|ref|XP_378507.1|	gi|6800963|ref|Os02g10370|
gi|31222290|ref|XP_317159.1|	lcl|At4g18090.1	lcl|F38A1.6 CE37370 WBGene0	gi|24665657|	gi|42660431|ref|XP_375190.1|	gi|6800969|ref|Os02g10410|
gi|31222305|ref|XP_317160.1|	lcl|At4g18280.1	lcl|F38A1.9 CE10020 WBGene0	gi|24665685|	gi|42660433|ref|XP_375191.1|	gi|6800974|ref|Os02g10450|
gi|31222439|ref|XP_317178.1|	lcl|At4g18310.1	lcl|F38A5.14 CE34856 WBGene	gi|24665704|	gi|42660446|ref|XP_378511.1|	gi|6800975|ref|Os02g10460|
gi|31222518|ref|XP_317188.1|	lcl|At4g18320.1	lcl|F38A5.3b CE33642 WBGene	gi|24665748|	gi|42660455|ref|XP_375203.1|	gi|6800983|ref|Os02g10500|
gi|31222524|ref|XP_317189.1|	lcl|At4g18335.1	lcl|F38A5.7 CE10038 WBGene0	gi|24665799|	gi|42660460|ref|XP_085231.4|	gi|6800989|ref|Os02g10560|
gi|31222698|ref|XP_317214.1|	lcl|At4g18395.1	lcl|F38A5.8 CE10040 WBGene0	gi|24665821|	gi|42660462|ref|XP_378514.1|	gi|6800990|ref|Os02g10570|
gi|31222781|ref|XP_317224.1|	lcl|At4g18400.1	lcl|F38A6.4 CE34857 WBGene0	gi|24665825|	gi|42660464|ref|XP_378515.1|	gi|6800995|ref|Os02g10610|
gi|31222787|ref|XP_317225.1|	lcl|At4g18420.1	lcl|F38B6.1 CE04514 WBGene0	gi|24665832|	gi|42660467|ref|XP_378516.1|	gi|6800996|ref|Os02g10620|
gi|31222803|ref|XP_317226.1|	lcl|At4g18490.1	lcl|F38B6.2 CE04515 WBGene0	gi|24665836|	gi|42660469|ref|XP_378517.1|	gi|6801001|ref|Os02g10670|
gi|31222838|ref|XP_317230.1|	lcl|At4g18500.1	lcl|F38B6.7 CE35526 WBGene0	gi|24665850|	gi|42660476|ref|XP_375210.1|	gi|6801002|ref|Os02g10680|
gi|31222878|ref|XP_317234.1|	lcl|At4g18510.1	lcl|F38C2.1 CE16001 WBGene0	gi|24665950|	gi|42660480|ref|XP_370871.2|	gi|6801007|ref|Os02g10730|
gi|31222885|ref|XP_317235.1|	lcl|At4g18580.1	lcl|F38E1.10 CE04528 WBGene	gi|24665958|	gi|42660483|ref|XP_116971.4|	gi|6801009|ref|Os02g10750|
gi|31222925|ref|XP_317240.1|	lcl|At4g18740.1	lcl|F38E1.11 CE04529 WBGene	gi|24665962|	gi|42660485|ref|XP_378522.1|	gi|6801022|ref|Os02g10870|
gi|31223029|ref|XP_317254.1|	lcl|At4g18850.1	lcl|F38E11.6a CE33780 WBGen	gi|24665992|	gi|42660490|ref|XP_378523.1|	gi|6801024|ref|Os02g10890|
gi|31223036|ref|XP_317255.1|	lcl|At4g18860.1	lcl|F38E11.6b CE33781 WBGen	gi|24666049|	gi|42660496|ref|XP_378525.1|	gi|6801030|ref|Os02g10930|
gi|31223061|ref|XP_317258.1|	lcl|At4g19095.1	lcl|F38E11.7 CE34013 WBGene	gi|24666088|	gi|42660502|ref|XP_378528.1|	gi|6801032|ref|Os02g10950|
gi|31223163|ref|XP_317271.1|	lcl|At4g19160.1	lcl|F38E11.9 CE26625 WBGene	gi|24666118|	gi|42660504|ref|XP_378529.1|	gi|6801033|ref|Os02g10960|
gi|31223184|ref|XP_317274.1|	lcl|At4g19200.1	lcl|F38E9.4 CE07200 WBGene0	gi|24666129|	gi|42660506|ref|XP_373636.2|	gi|6801035|ref|Os02g10980|
gi|31223227|ref|XP_317281.1|	lcl|At4g19240.1	lcl|F38E9.6 CE07202 WBGene0	gi|24666133|	gi|42660519|ref|XP_378535.1|	gi|6801039|ref|Os02g11010|
gi|31223285|ref|XP_317289.1|	lcl|At4g19260.1	lcl|F38H4.1 CE10058 WBGene0	gi|24666218|	gi|42660525|ref|XP_375226.1|	gi|6801048|ref|Os02g11080|
gi|31223387|ref|XP_317302.1|	lcl|At4g19270.1	lcl|F38H4.3 CE10062 WBGene0	gi|24666222|	gi|42660542|ref|XP_377388.1|	gi|6801063|ref|Os02g11710|
gi|31223440|ref|XP_317308.1|	lcl|At4g19280.1	lcl|F39B2.4a CE28023 WBGene	gi|24666226|	gi|42660544|ref|XP_377390.1|	gi|6801069|ref|Os02g11770|
gi|31223520|ref|XP_317318.1|	lcl|At4g19290.1	lcl|F39B2.4b CE28024 WBGene	gi|24666251|	gi|42660548|ref|XP_378172.1|	gi|6801072|ref|Os02g11800|
gi|31223548|ref|XP_317321.1|	lcl|At4g19305.1	lcl|F39B3.3 CE10090 WBGene0	gi|24666266|	gi|42660550|ref|XP_378538.1|	gi|6801073|ref|Os02g11810|
gi|31223562|ref|XP_317323.1|	lcl|At4g19320.1	lcl|F39D8.1a CE17810 WBGene	gi|24666282|	gi|42660554|ref|XP_375246.1|	gi|6801080|ref|Os02g11870|
gi|31223635|ref|XP_317332.1|	lcl|At4g19340.1	lcl|F39D8.1b CE23707 WBGene	gi|24666286|	gi|42660560|ref|XP_375249.1|	gi|6801083|ref|Os02g11900|
gi|31223699|ref|XP_317340.1|	lcl|At4g19430.1	lcl|F39D8.1c CE17812 WBGene	gi|24666303|	gi|42660562|ref|XP_370899.2|	gi|6801084|ref|Os02g11910|
gi|31223707|ref|XP_317341.1|	lcl|At4g19470.1	lcl|F39D8.2 CE34185 WBGene0	gi|24666307|	gi|42660564|ref|XP_375252.1|	gi|6801085|ref|Os02g11920|
gi|31223714|ref|XP_317342.1|	lcl|At4g19480.1	lcl|F39F10.4 CE07206 WBGene	gi|24666319|	gi|42660568|ref|XP_172931.4|	gi|6801090|ref|Os02g11970|
gi|31223733|ref|XP_317345.1|	lcl|At4g19550.1	lcl|F39F10.5 CE07207 WBGene	gi|24666326|	gi|42660570|ref|XP_378542.1|	gi|6801091|ref|Os02g11970|
gi|31223777|ref|XP_317353.1|	lcl|At4g19620.1	lcl|F39H12.1 CE35527 WBGene	gi|24666330|	gi|42660579|ref|XP_375263.1|	gi|6801093|ref|Os02g11990|
gi|31223782|ref|XP_317354.1|	lcl|At4g19980.1	lcl|F39H12.2 CE07209 WBGene	gi|24666358|	gi|42660581|ref|XP_378544.1|	gi|6801094|ref|Os02g12000|
gi|31223878|ref|XP_317366.1|	lcl|At4g20160.1	lcl|F39H2.1 CE30530 WBGene0	gi|24666421|	gi|42660583|ref|XP_378545.1|	gi|6801096|ref|Os02g12020|
gi|31223940|ref|XP_317374.1|	lcl|At4g20220.1	lcl|F39H2.4 CE17814 WBGene0	gi|24666454|	gi|42660589|ref|XP_378549.1|	gi|6801099|ref|Os02g12050|
gi|31223967|ref|XP_317378.1|	lcl|At4g20250.1	lcl|F40A3.1 CE10142 WBGene0	gi|24666472|	gi|42660597|ref|XP_378173.1|	gi|6801101|ref|Os02g12070|
gi|31224000|ref|XP_317382.1|	lcl|At4g20290.1	lcl|F40A3.2 CE10144 WBGene0	gi|24666479|	gi|42660601|ref|XP_375266.1|	gi|6801102|ref|Os02g12080|
gi|31224020|ref|XP_317384.1|	lcl|At4g20420.1	lcl|F40A3.4 CE33643 WBGene0	gi|24666567|	gi|42660603|ref|XP_378550.1|	gi|6801103|ref|Os02g12090|
gi|31224089|ref|XP_317393.1|	lcl|At4g20470.1	lcl|F40A3.6 CE10152 WBGene0	gi|24666619|	gi|42660605|ref|XP_378551.1|	gi|6801108|ref|Os02g12140|
gi|31224152|ref|XP_317402.1|	lcl|At4g20500.1	lcl|F40C5.2 CE23710 WBGene0	gi|24666694|	gi|42660609|ref|XP_375269.1|	gi|6801109|ref|Os02g12150|
gi|31224162|ref|XP_317403.1|	lcl|At4g20510.1	lcl|F40D4.13 CE19847 WBGene	gi|24666721|	gi|42660613|ref|XP_373615.2|	gi|6801111|ref|Os02g12170|
gi|31224246|ref|XP_317415.1|	lcl|At4g20690.1	lcl|F40E12.1 CE36843 WBGene	gi|24666733|	gi|42660622|ref|XP_378493.1|	gi|6801112|ref|Os02g12180|
gi|31224322|ref|XP_317426.1|	lcl|At4g20715.1	lcl|F40E3.2 CE34307 WBGene0	gi|24666737|	gi|42660624|ref|XP_294775.3|	gi|6801115|ref|Os02g12210|
gi|31224377|ref|XP_317431.1|	lcl|At4g20880.1	lcl|F40E3.3 CE10162 WBGene0	gi|24666741|	gi|42660628|ref|XP_378494.1|	gi|6801117|ref|Os02g12230|
gi|31224383|ref|XP_317432.1|	lcl|At4g21215.1	lcl|F40E3.6 CE10168 WBGene0	gi|24666745|	gi|42660630|ref|XP_375153.1|	gi|6801118|ref|Os02g12240|
gi|31224460|ref|XP_317443.1|	lcl|At4g21500.1	lcl|F40F11.2 CE28557 WBGene	gi|24666749|	gi|42660633|ref|XP_375154.1|	gi|6801119|ref|Os02g12250|
gi|31224504|ref|XP_317448.1|	lcl|At4g21740.1	lcl|F40F11.3 CE05862 WBGene	gi|24666753|	gi|42660636|ref|XP_373616.2|	gi|6801120|ref|Os02g12260|
gi|31224509|ref|XP_317449.1|	lcl|At4g21780.1	lcl|F40F12.3 CE00618 WBGene	gi|24666758|	gi|42660641|ref|XP_378496.1|	gi|6801121|ref|Os02g12270|
gi|31224516|ref|XP_317450.1|	lcl|At4g21865.1	lcl|F40F12.4 CE00569 WBGene	gi|24666762|	gi|42660643|ref|XP_294765.3|	gi|6801122|ref|Os02g12280|
gi|31224526|ref|XP_317451.1|	lcl|At4g21890.1	lcl|F40F8.3 CE05843 WBGene0	gi|24666770|	gi|42660645|ref|XP_375235.1|	gi|6801127|ref|Os02g12330|
gi|31224535|ref|XP_317452.1|	lcl|At4g21920.1	lcl|F40F8.4 CE05844 WBGene0	gi|24666850|	gi|42660653|ref|XP_375240.1|	gi|6801132|ref|Os02g12370|
gi|31224541|ref|XP_317453.1|	lcl|At4g21950.1	lcl|F40F8.5 CE32888 WBGene0	gi|24666854|	gi|42660655|ref|XP_370881.2|	gi|6801138|ref|Os02g12430|
gi|31224567|ref|XP_317456.1|	lcl|At4g22020.1	lcl|F40F8.7 CE05846 WBGene0	gi|24666886|	gi|42660667|ref|XP_375163.1|	gi|6801141|ref|Os02g12460|
gi|31224606|ref|XP_317460.1|	lcl|At4g22160.1	lcl|F40G12.11 CE10196 WBGen	gi|24666944|	gi|42660685|ref|XP_375272.1|	gi|6801142|ref|Os02g12470|
gi|31224610|ref|XP_317461.1|	lcl|At4g22160.2	lcl|F40G9.5 CE19852 WBGene0	gi|24667104|	gi|42660691|ref|XP_375275.1|	gi|6801143|ref|Os02g12480|
gi|31224629|ref|XP_317463.1|	lcl|At4g22165.1	lcl|F40G9.6 CE19853 WBGene0	gi|24667113|	gi|42660695|ref|XP_378553.1|	gi|6801144|ref|Os02g12490|
gi|31224699|ref|XP_317473.1|	lcl|At4g22210.1	lcl|F40G9.7 CE19854 WBGene0	gi|24667117|	gi|42660697|ref|XP_375277.1|	gi|6801145|ref|Os02g12490|
gi|31224873|ref|XP_317500.1|	lcl|At4g22212.1	lcl|F40G9.8 CE19855 WBGene0	gi|24667121|	gi|42660709|ref|XP_378564.1|	gi|6801146|ref|Os02g12500|
gi|31224923|ref|XP_317505.1|	lcl|At4g22214.1	lcl|F40H3.2 CE35530 WBGene0	gi|24667186|	gi|42660721|ref|XP_378567.1|	gi|6801147|ref|Os02g12510|
gi|31224937|ref|XP_317507.1|	lcl|At4g22217.1	lcl|F40H3.3 CE19864 WBGene0	gi|24667211|	gi|42660729|ref|XP_373669.2|	gi|6801152|ref|Os02g12560|
gi|31224973|ref|XP_317512.1|	lcl|At4g22230.1	lcl|F40H3.6 CE28318 WBGene0	gi|24667215|	gi|42660741|ref|XP_372615.2|	gi|6801155|ref|Os02g12590|
gi|31224987|ref|XP_317514.1|	lcl|At4g22230.2	lcl|F40H6.1 CE01278 WBGene0	gi|24667242|	gi|42660746|ref|XP_378571.1|	gi|6801160|ref|Os02g12640|
gi|31225013|ref|XP_317517.1|	lcl|At4g22235.1	lcl|F40H6.2 CE01279 WBGene0	gi|24667306|	gi|42660752|ref|XP_378573.1|	gi|6801167|ref|Os02g12710|
gi|31225071|ref|XP_317525.1|	lcl|At4g22400.1	lcl|F41B4.1 CE30980 WBGene0	gi|24667314|	gi|42660769|ref|XP_378599.1|	gi|6801179|ref|Os02g12830|
gi|31225126|ref|XP_317532.1|	lcl|At4g22420.1	lcl|F41B4.2a CE30981 WBGene	gi|24667458|	gi|42660777|ref|XP_378601.1|	gi|6801182|ref|Os02g12860|
gi|31225139|ref|XP_317533.1|	lcl|At4g22430.1	lcl|F41B4.2b CE07212 WBGene	gi|24667517|	gi|42660782|ref|XP_378606.1|	gi|6801189|ref|Os02g12930|
gi|31225217|ref|XP_317544.1|	lcl|At4g22440.1	lcl|F41B4.3 CE07213 WBGene0	gi|24667577|	gi|42660786|ref|XP_375360.1|	gi|6801191|ref|Os02g12950|
gi|31225233|ref|XP_317546.1|	lcl|At4g22480.1	lcl|F41C3.6 CE29797 WBGene0	gi|24667627|	gi|42660788|ref|XP_378607.1|	gi|6801192|ref|Os02g12960|
gi|31225240|ref|XP_317547.1|	lcl|At4g22500.1	lcl|F41C3.7 CE02735 WBGene0	gi|24667631|	gi|42660798|ref|XP_378608.1|	gi|6801198|ref|Os02g13020|
gi|31225441|ref|XP_317572.1|	lcl|At4g22510.1	lcl|F41C3.8 CE29798 WBGene0	gi|24667642|	gi|42660804|ref|XP_378609.1|	gi|6801199|ref|Os02g13030|
gi|31225517|ref|XP_317582.1|	lcl|At4g22520.1	lcl|F41C6.2 CE04539 WBGene0	gi|24667670|	gi|42660814|ref|XP_378592.1|	gi|6801200|ref|Os02g13040|
gi|31225524|ref|XP_317583.1|	lcl|At4g22600.1	lcl|F41C6.3 CE04540 WBGene0	gi|24667674|	gi|42660820|ref|XP_378595.1|	gi|6801201|ref|Os02g13050|
gi|31225645|ref|XP_317597.1|	lcl|At4g22650.1	lcl|F41C6.6 CE04543 WBGene0	gi|24667699|	gi|42660822|ref|XP_375379.1|	gi|6801203|ref|Os02g13070|
gi|31225651|ref|XP_317598.1|	lcl|At4g22800.1	lcl|F41D3.7 CE16026 WBGene0	gi|24667715|	gi|42660824|ref|XP_378628.1|	gi|6801204|ref|Os02g13080|
gi|31225689|ref|XP_317602.1|	lcl|At4g23020.1	lcl|F41D3.8 CE16027 WBGene0	gi|24667741|	gi|42660831|ref|XP_378630.1|	gi|6801205|ref|Os02g13090|
gi|31225864|ref|XP_317625.1|	lcl|At4g23090.1	lcl|F41E6.1 CE10244 WBGene0	gi|24667752|	gi|42660833|ref|XP_378631.1|	gi|6801208|ref|Os02g13120|
gi|31225891|ref|XP_317629.1|	lcl|At4g23110.1	lcl|F41E6.10 CE10262 WBGene	gi|24667756|	gi|42660835|ref|XP_375383.1|	gi|6801217|ref|Os02g13210|
gi|31225953|ref|XP_317636.1|	lcl|At4g23330.1	lcl|F41E6.11 CE10264 WBGene	gi|24667771|	gi|42660837|ref|XP_378632.1|	gi|6801224|ref|Os02g13280|
gi|31225958|ref|XP_317637.1|	lcl|At4g23493.1	lcl|F41E6.12 CE37770 WBGene	gi|24667778|	gi|42660839|ref|XP_378633.1|	gi|6801228|ref|Os02g13320|
gi|31225998|ref|XP_317641.1|	lcl|At4g23760.1	lcl|F41E6.7 CE10256 WBGene0	gi|24667804|	gi|42660841|ref|XP_378634.1|	gi|6801235|ref|Os02g13390|
gi|31226018|ref|XP_317643.1|	lcl|At4g23770.1	lcl|F41E7.4 CE03304 WBGene0	gi|24667868|	gi|42660847|ref|XP_373704.2|	gi|6801240|ref|Os02g13440|
gi|31226058|ref|XP_317649.1|	lcl|At4g23780.1	lcl|F41E7.5 CE03305 WBGene0	gi|24667872|	gi|42660849|ref|XP_375386.1|	gi|6801241|ref|Os02g13450|
gi|31226108|ref|XP_317658.1|	lcl|At4g23870.1	lcl|F41E7.7 CE03307 WBGene0	gi|24667917|	gi|42660851|ref|XP_378637.1|	gi|6801244|ref|Os02g13480|
gi|31226140|ref|XP_317662.1|	lcl|At4g23880.1	lcl|F41E7.8 CE19867 WBGene0	gi|24667966|	gi|42660855|ref|XP_378558.1|	gi|6801250|ref|Os02g13540|
gi|31226184|ref|XP_317668.1|	lcl|At4g23885.1	lcl|F41E7.9 CE34858 WBGene0	gi|24668010|	gi|42660861|ref|XP_373662.2|	gi|6801251|ref|Os02g13550|
gi|31226253|ref|XP_317680.1|	lcl|At4g23970.1	lcl|F41F3.1 CE07215 WBGene0	gi|24668022|	gi|42660871|ref|XP_378562.1|	gi|6801255|ref|Os02g13590|
gi|31226330|ref|XP_317691.1|	lcl|At4g24030.1	lcl|F41F3.3 CE07217 WBGene0	gi|24668030|	gi|42660875|ref|XP_375378.1|	gi|6801256|ref|Os02g13600|
gi|31226339|ref|XP_317692.1|	lcl|At4g24265.1	lcl|F41G3.1 CE01282 WBGene0	gi|24668034|	gi|42660877|ref|XP_378620.1|	gi|6801259|ref|Os02g13630|
gi|31226346|ref|XP_317693.1|	lcl|At4g24275.1	lcl|F41G3.2 CE01286 WBGene0	gi|24668038|	gi|42660879|ref|XP_378621.1|	gi|6801263|ref|Os02g13670|
gi|31226418|ref|XP_317703.1|	lcl|At4g24300.1	lcl|F41G3.3 CE01287 WBGene0	gi|24668070|	gi|42660881|ref|XP_378623.1|	gi|6801265|ref|Os02g13690|
gi|31226433|ref|XP_317706.1|	lcl|At4g24370.1	lcl|F41G3.6 CE28933 WBGene0	gi|24668091|	gi|42660883|ref|XP_378625.1|	gi|6801266|ref|Os02g13700|
gi|31226447|ref|XP_317708.1|	lcl|At4g24410.1	lcl|F41G4.4 CE34556 WBGene0	gi|24668241|	gi|42660885|ref|XP_378626.1|	gi|6801268|ref|Os02g13720|
gi|31226509|ref|XP_317720.1|	lcl|At4g24500.1	lcl|F41G4.5 CE34557 WBGene0	gi|24668338|	gi|42660887|ref|XP_373701.2|	gi|6801270|ref|Os02g13740|
gi|31226541|ref|XP_317726.1|	lcl|At4g24500.2	lcl|F41G4.7 CE29799 WBGene0	gi|24668342|	gi|42660889|ref|XP_378586.1|	gi|6801272|ref|Os02g13760|
gi|31226552|ref|XP_317728.1|	lcl|At4g24600.1	lcl|F41G4.8 CE04553 WBGene0	gi|24668346|	gi|42660891|ref|XP_378588.1|	gi|6801275|ref|Os02g13790|
gi|31226592|ref|XP_317736.1|	lcl|At4g24700.1	lcl|F41H10.12 CE29499 WBGen	gi|24668350|	gi|42660893|ref|XP_378589.1|	gi|6801278|ref|Os02g13820|
gi|31226626|ref|XP_317738.1|	lcl|At4g24950.1	lcl|F41H10.2 CE10274 WBGene	gi|24668369|	gi|42660895|ref|XP_375353.1|	gi|6801283|ref|Os02g13860|
gi|31226652|ref|XP_317742.1|	lcl|At4g25060.1	lcl|F41H10.3a CE10276 WBGen	gi|24668373|	gi|42660898|ref|XP_378590.1|	gi|6801285|ref|Os02g13880|
gi|31226668|ref|XP_317744.1|	lcl|At4g25330.1	lcl|F41H10.3b CE31694 WBGen	gi|24668429|	gi|42660904|ref|XP_373693.2|	gi|6801289|ref|Os02g13920|
gi|31226676|ref|XP_317745.1|	lcl|At4g25430.1	lcl|F41H10.9 CE32414 WBGene	gi|24668448|	gi|42660915|ref|XP_378617.1|	gi|6801291|ref|Os02g13940|
gi|31226691|ref|XP_317748.1|	lcl|At4g25510.1	lcl|F42A10.5 CE01296 WBGene	gi|24668478|	gi|42660923|ref|XP_377447.1|	gi|6801295|ref|Os02g13980|
gi|31226699|ref|XP_317750.1|	lcl|At4g25580.1	lcl|F42A10.7 CE01298 WBGene	gi|24668499|	gi|42660933|ref|XP_378576.1|	gi|6801302|ref|Os02g14010|
gi|31226769|ref|XP_317761.1|	lcl|At4g25760.1	lcl|F42A6.1 CE34558 WBGene0	gi|24668504|	gi|42660935|ref|XP_378577.1|	gi|6801303|ref|Os02g14020|
gi|31226773|ref|XP_317762.1|	lcl|At4g26030.1	lcl|F42A6.2 CE17054 WBGene0	gi|24668555|	gi|42660937|ref|XP_378578.1|	gi|6801304|ref|Os02g14030|
gi|31226787|ref|XP_317764.1|	lcl|At4g26040.1	lcl|F42A6.3 CE17055 WBGene0	gi|24668559|	gi|42660955|ref|XP_375334.1|	gi|6801305|ref|Os02g14040|
gi|31226810|ref|XP_317769.1|	lcl|At4g26130.1	lcl|F42A8.1 CE01578 WBGene0	gi|24668589|	gi|42660962|ref|XP_378582.1|	gi|6801307|ref|Os02g14060|

gi|31226824|ref|XP_317772.1|	lcl|At4g26290.1	lcl|F42A9.3 CE07225 WBGene0	gi|24668596|	gi|42660982|ref|XP_375349.1|	gi|6801309|ref|Os02g14080|
gi|31226832|ref|XP_317774.1|	lcl|At4g26320.1	lcl|F42A9.6 CE34859 WBGene0	gi|24668623|	gi|42661030|ref|XP_378700.1|	gi|6801322|ref|Os02g14200|
gi|31226836|ref|XP_317775.1|	lcl|At4g26450.1	lcl|F42A9.8 CE07229 WBGene0	gi|24668627|	gi|42661032|ref|XP_378701.1|	gi|6801325|ref|Os02g14230|
gi|31226839|ref|XP_317776.1|	lcl|At4g26460.1	lcl|F42A9.9 CE34860 WBGene0	gi|24668631|	gi|42661034|ref|XP_378703.1|	gi|6801326|ref|Os02g14240|
gi|31226877|ref|XP_317784.1|	lcl|At4g26960.1	lcl|F42C5.10 CE28393 WBGene	gi|24668635|	gi|42661036|ref|XP_378705.1|	gi|6801332|ref|Os02g14300|
gi|31226948|ref|XP_317798.1|	lcl|At4g27330.1	lcl|F42C5.3 CE04556 WBGene0	gi|24668650|	gi|42661043|ref|XP_378706.1|	gi|6801333|ref|Os02g14310|
gi|31226952|ref|XP_317799.1|	lcl|At4g27410.1	lcl|F42C5.6 CE33645 WBGene0	gi|24668654|	gi|42661045|ref|XP_375491.1|	gi|6801335|ref|Os02g14330|
gi|31226962|ref|XP_317801.1|	lcl|At4g27510.1	lcl|F42E11.3 CE03310 WBGene	gi|24668658|	gi|42661047|ref|XP_378708.1|	gi|6801336|ref|Os02g14340|
gi|31226999|ref|XP_317808.1|	lcl|At4g27530.1	lcl|F42E8.1 CE10302 WBGene0	gi|24668735|	gi|42661049|ref|XP_378709.1|	gi|6801337|ref|Os02g14350|
gi|31227024|ref|XP_317812.1|	lcl|At4g27580.1	lcl|F42F12.1 CE31938 WBGene	gi|24668743|	gi|42661058|ref|XP_378712.1|	gi|6801344|ref|Os02g14420|
gi|31227053|ref|XP_317817.1|	lcl|At4g27652.1	lcl|F42F12.10 CE31940 WBGen	gi|24668747|	gi|42661066|ref|XP_378642.1|	gi|6801347|ref|Os02g14440|
gi|31227141|ref|XP_317833.1|	lcl|At4g27654.1	lcl|F42F12.6 CE31939 WBGene	gi|24668828|	gi|42661070|ref|XP_378643.1|	gi|6801348|ref|Os02g14450|
gi|31227197|ref|XP_317843.1|	lcl|At4g27657.1	lcl|F42F12.7 CE31939 WBGene	gi|24668840|	gi|42661085|ref|XP_378646.1|	gi|6801350|ref|Os02g14470|
gi|31227225|ref|XP_317848.1|	lcl|At4g27660.1	lcl|F42F12.8 CE31940 WBGene	gi|24668848|	gi|42661095|ref|XP_378648.1|	gi|6801353|ref|Os02g14500|
gi|31227421|ref|XP_317879.1|	lcl|At4g27810.1	lcl|F42F12.9 CE31941 WBGene	gi|24668885|	gi|42661106|ref|XP_375410.1|	gi|6801354|ref|Os02g14510|
gi|31227445|ref|XP_317883.1|	lcl|At4g27850.1	lcl|F42G2.3 CE10310 WBGene0	gi|24762327|	gi|42661108|ref|XP_378650.1|	gi|6801364|ref|Os02g14610|
gi|31227470|ref|XP_317887.1|	lcl|At4g27930.1	lcl|F42G4.2 CE37914 WBGene0	gi|24762381|	gi|42661111|ref|XP_375412.1|	gi|6801365|ref|Os02g14620|
gi|31227475|ref|XP_317888.1|	lcl|At4g28085.1	lcl|F42G4.5 CE35535 WBGene0	gi|24762383|	gi|42661113|ref|XP_378653.1|	gi|6801367|ref|Os02g14640|
gi|31227489|ref|XP_317890.1|	lcl|At4g28160.1	lcl|F42G4.6 CE20776 WBGene0	gi|24762385|	gi|42661121|ref|XP_378655.1|	gi|6801368|ref|Os02g14650|
gi|31227594|ref|XP_317909.1|	lcl|At4g28170.1	lcl|F42G4.7 CE34861 WBGene0	gi|24762392|	gi|42661124|ref|XP_378656.1|	gi|6801369|ref|Os02g14660|
gi|31227600|ref|XP_317910.1|	lcl|At4g28180.1	lcl|F42G8.10a CE17069 WBGen	gi|24762403|	gi|42661152|ref|XP_375452.1|	gi|6801370|ref|Os02g14670|
gi|31227638|ref|XP_317918.1|	lcl|At4g28210.1	lcl|F42G8.10b CE33646 WBGen	gi|24762528|	gi|42661154|ref|XP_378675.1|	gi|6801374|ref|Os02g14710|
gi|31227693|ref|XP_317928.1|	lcl|At4g28230.1	lcl|F42G8.5 CE17064 WBGene0	gi|24762600|	gi|42661158|ref|XP_378676.1|	gi|6801377|ref|Os02g14740|
gi|31227800|ref|XP_317945.1|	lcl|At4g28240.1	lcl|F42G9.4 CE30789 WBGene0	gi|24762608|	gi|42661160|ref|XP_373733.2|	gi|6801378|ref|Os02g14750|
gi|31227829|ref|XP_317949.1|	lcl|At4g28290.1	lcl|F42H10.5 CE00167 WBGene	gi|24762630|	gi|42661162|ref|XP_379749.1|	gi|6801383|ref|Os02g14800|
gi|31227891|ref|XP_317959.1|	lcl|At4g28460.1	lcl|F42H10.9 CE24970 WBGene	gi|24762661|	gi|42661168|ref|XP_378678.1|	gi|6801385|ref|Os02g14820|
gi|31227932|ref|XP_317966.1|	lcl|At4g28810.1	lcl|F43B10.1 CE28323 WBGene	gi|24762690|	gi|42661176|ref|XP_378680.1|	gi|6801386|ref|Os02g14830|
gi|31227949|ref|XP_317969.1|	lcl|At4g28870.1	lcl|F43B10.2 CE35536 WBGene	gi|24762722|	gi|42661182|ref|XP_378682.1|	gi|6801388|ref|Os02g14850|
gi|31227962|ref|XP_317971.1|	lcl|At4g28930.1	lcl|F43C11.10 CE35930 WBGen	gi|24762747|	gi|42661188|ref|XP_378683.1|	gi|6801391|ref|Os02g14880|
gi|31227969|ref|XP_317972.1|	lcl|At4g28960.1	lcl|F43C11.3 CE20783 WBGene	gi|24762753|	gi|42661190|ref|XP_378684.1|	gi|6801392|ref|Os02g14890|
gi|31228011|ref|XP_317979.1|	lcl|At4g29020.1	lcl|F43C11.6 CE20780 WBGene	gi|24762790|	gi|42661192|ref|XP_378685.1|	gi|6801399|ref|Os02g14960|
gi|31228015|ref|XP_317980.1|	lcl|At4g29030.1	lcl|F43C11.9 CE23724 WBGene	gi|24762792|	gi|42661194|ref|XP_378686.1|	gi|6801400|ref|Os02g14970|
gi|31228030|ref|XP_317983.1|	lcl|At4g29110.1	lcl|F43D2.2 CE10336 WBGene0	gi|24762803|	gi|42661201|ref|XP_378688.1|	gi|6801406|ref|Os02g15030|
gi|31228044|ref|XP_317986.1|	lcl|At4g29200.1	lcl|F43D2.3 CE10338 WBGene0	gi|24762828|	gi|42661203|ref|XP_375475.1|	gi|6801407|ref|Os02g15040|
gi|31228049|ref|XP_317987.1|	lcl|At4g29280.1	lcl|F43E2.11 CE36848 WBGene	gi|28571123|	gi|42661212|ref|XP_378689.1|	gi|6801408|ref|Os02g15050|
gi|31228092|ref|XP_317996.1|	lcl|At4g29285.1	lcl|F43E2.6a CE32662 WBGene	gi|28571127|	gi|42661214|ref|XP_378692.1|	gi|6801416|ref|Os02g15130|
gi|31228140|ref|XP_318005.1|	lcl|At4g29290.1	lcl|F43E2.6b CE32663 WBGene	gi|28571165|	gi|42661221|ref|XP_378693.1|	gi|6801419|ref|Os02g15160|
gi|31228145|ref|XP_318006.1|	lcl|At4g29300.1	lcl|F43E2.7a CE33087 WBGene	gi|28571277|	gi|42661226|ref|XP_378694.1|	gi|6801423|ref|Os02g15200|
gi|31228165|ref|XP_318009.1|	lcl|At4g30050.1	lcl|F43E2.7b CE32664 WBGene	gi|28571279|	gi|42661242|ref|XP_378664.1|	gi|6801424|ref|Os02g15210|
gi|31228170|ref|XP_318010.1|	lcl|At4g30070.1	lcl|F43E2.9 CE10350 WBGene0	gi|28571310|	gi|42661250|ref|XP_375434.1|	gi|6801428|ref|Os02g15250|
gi|31228177|ref|XP_318011.1|	lcl|At4g30180.1	lcl|F43G6.10 CE20790 WBGene	gi|28571356|	gi|42661252|ref|XP_378667.1|	gi|6801429|ref|Os02g15260|
gi|31228187|ref|XP_318013.1|	lcl|At4g30230.1	lcl|F43G6.2 CE02220 WBGene0	gi|28571385|	gi|42661254|ref|XP_375436.1|	gi|6801433|ref|Os02g15300|
gi|31228246|ref|XP_318024.1|	lcl|At4g30390.1	lcl|F43G6.4 CE31700 WBGene0	gi|28571392|	gi|42661259|ref|XP_378668.1|	gi|6801440|ref|Os02g15370|
gi|31228297|ref|XP_318032.1|	lcl|At4g30450.1	lcl|F43G6.7 CE02225 WBGene0	gi|28571400|	gi|42661269|ref|XP_378713.1|	gi|6801444|ref|Os02g15410|
gi|31228313|ref|XP_318034.1|	lcl|At4g30460.1	lcl|F43G6.9 CE18675 WBGene0	gi|28571424|	gi|42661273|ref|XP_375494.1|	gi|6801445|ref|Os02g15420|
gi|31228317|ref|XP_318035.1|	lcl|At4g30630.1	lcl|F43G9.13 CE27163 WBGene	gi|28571426|	gi|42661277|ref|XP_375496.1|	gi|6801447|ref|Os02g15440|
gi|31228481|ref|XP_318063.1|	lcl|At4g30670.1	lcl|F43G9.8 CE34308 WBGene0	gi|28571428|	gi|42661287|ref|XP_378660.1|	gi|6801448|ref|Os02g15450|
gi|31228488|ref|XP_318064.1|	lcl|At4g30730.1	lcl|F43H9.4 CE27381 WBGene0	gi|28571433|	gi|42661298|ref|XP_378661.1|	gi|6801449|ref|Os02g15460|
gi|31228521|ref|XP_318070.1|	lcl|At4g30740.1	lcl|F44A2.5a CE33088 WBGene	gi|28571454|	gi|42661301|ref|XP_378639.1|	gi|6801456|ref|Os02g15530|
gi|31228626|ref|XP_318087.1|	lcl|At4g30750.1	lcl|F44A2.7 CE35308 WBGene0	gi|28571456|	gi|42661309|ref|XP_378723.1|	gi|6801457|ref|Os02g15540|
gi|31228662|ref|XP_318092.1|	lcl|At4g30970.1	lcl|F44A6.3 CE03324 WBGene0	gi|28571511|	gi|42661315|ref|XP_378724.1|	gi|6801460|ref|Os02g15570|
gi|31228668|ref|XP_318093.1|	lcl|At4g31030.1	lcl|F44A6.4 CE37373 WBGene0	gi|28571513|	gi|42661322|ref|XP_378728.1|	gi|6801462|ref|Os02g15590|
gi|31228675|ref|XP_318094.1|	lcl|At4g31260.1	lcl|F44A6.5 CE03326 WBGene0	gi|28571535|	gi|42661336|ref|XP_378730.1|	gi|6801470|ref|Os02g15670|
gi|31228845|ref|XP_318120.1|	lcl|At4g31280.1	lcl|F44B9.2 CE00171 WBGene0	gi|28571545|	gi|42661341|ref|XP_378734.1|	gi|6801471|ref|Os02g15680|
gi|31228849|ref|XP_318121.1|	lcl|At4g31430.1	lcl|F44B9.4c CE32419 WBGene	gi|28571547|	gi|42661343|ref|XP_375527.1|	gi|6801473|ref|Os02g15700|
gi|31228871|ref|XP_318125.1|	lcl|At4g31730.1	lcl|F44B9.7 CE29043 WBGene0	gi|28571549|	gi|42661345|ref|XP_378735.1|	gi|6801475|ref|Os02g15720|
gi|31228875|ref|XP_318126.1|	lcl|At4g31875.1	lcl|F44D12.6 CE03330 WBGene	gi|28571552|	gi|42661349|ref|XP_378738.1|	gi|6801480|ref|Os02g15770|
gi|31228883|ref|XP_318127.1|	lcl|At4g31960.1	lcl|F44D12.8 CE03331 WBGene	gi|28571555|	gi|42661357|ref|XP_373775.2|	gi|6801481|ref|Os02g15780|
gi|31228887|ref|XP_318128.1|	lcl|At4g32020.1	lcl|F44E2.2c CE00180 WBGene	gi|28571597|	gi|42661361|ref|XP_378741.1|	gi|6801483|ref|Os02g15800|
gi|31228910|ref|XP_318133.1|	lcl|At4g32030.1	lcl|F44E2.7a CE29802 WBGene	gi|28571599|	gi|42661367|ref|XP_378760.1|	gi|6801486|ref|Os02g15830|
gi|31228921|ref|XP_318135.1|	lcl|At4g32080.1	lcl|F44E2.7b CE30994 WBGene	gi|28571601|	gi|42661369|ref|XP_378763.1|	gi|6801487|ref|Os02g15840|
gi|31228937|ref|XP_318138.1|	lcl|At4g32100.1	lcl|F44E2.7c CE30995 WBGene	gi|28571613|	gi|42661371|ref|XP_377496.1|	gi|6801488|ref|Os02g15850|
gi|31228941|ref|XP_318139.1|	lcl|At4g32230.1	lcl|F44E2.7d CE24975 WBGene	gi|28571635|	gi|42661377|ref|XP_378742.1|	gi|6801492|ref|Os02g15890|
gi|31228974|ref|XP_318147.1|	lcl|At4g32240.1	lcl|F44E2.8 CE32420 WBGene0	gi|28571660|	gi|42661388|ref|XP_378745.1|	gi|6801494|ref|Os02g15910|
gi|31228982|ref|XP_318150.1|	lcl|At4g32280.1	lcl|F44E2.9 CE26895 WBGene0	gi|28571662|	gi|42661390|ref|XP_378746.1|	gi|6801495|ref|Os02g15920|
gi|31229029|ref|XP_318155.1|	lcl|At4g32695.1	lcl|F44E5.1 CE18676 WBGene0	gi|28571714|	gi|42661402|ref|XP_378747.1|	gi|6801499|ref|Os02g15960|
gi|31229045|ref|XP_318158.1|	lcl|At4g32740.1	lcl|F44E7.3 CE10400 WBGene0	gi|28571741|	gi|42661406|ref|XP_378756.1|	gi|6801500|ref|Os02g15970|
gi|31229091|ref|XP_318165.1|	lcl|At4g32860.1	lcl|F44E7.5a CE33093 WBGene	gi|28571753|	gi|42661410|ref|XP_378757.1|	gi|6801505|ref|Os02g16020|
gi|31229110|ref|XP_318168.1|	lcl|At4g33310.1	lcl|F44E7.5b CE33094 WBGene	gi|28571755|	gi|42661412|ref|XP_378758.1|	gi|6801519|ref|Os02g16160|
gi|31229155|ref|XP_318174.1|	lcl|At4g33560.1	lcl|F44F1.2 CE10414 WBGene0	gi|28571782|	gi|42661414|ref|XP_375545.1|	gi|6801521|ref|Os02g16180|
gi|31229165|ref|XP_318176.1|	lcl|At4g33570.1	lcl|F44F1.4 CE10418 WBGene0	gi|28571790|	gi|42661418|ref|XP_378750.1|	gi|6801526|ref|Os02g16230|
gi|31229176|ref|XP_318178.1|	lcl|At4g33610.1	lcl|F44F1.5 CE10420 WBGene0	gi|28571862|	gi|42661420|ref|XP_378751.1|	gi|6801532|ref|Os02g16290|
gi|31229196|ref|XP_318181.1|	lcl|At4g33660.1	lcl|F44F1.6a CE23728 WBGene	gi|28571863|	gi|42661422|ref|XP_378753.1|	gi|6801534|ref|Os02g16310|
gi|31229231|ref|XP_318186.1|	lcl|At4g33666.1	lcl|F44F1.6b CE23729 WBGene	gi|28571880|	gi|42661424|ref|XP_378754.1|	gi|6801535|ref|Os02g16320|
gi|31229241|ref|XP_318190.1|	lcl|At4g33740.1	lcl|F44F1.7 CE10424 WBGene0	gi|28571898|	gi|42661428|ref|XP_378755.1|	gi|6801541|ref|Os02g16380|
gi|31229247|ref|XP_318193.1|	lcl|At4g33740.2	lcl|F44F4.10 CE00997 WBGene	gi|28571903|	gi|42661555|ref|XP_378178.1|	gi|6801544|ref|Os02g16410|
gi|31229258|ref|XP_318198.1|	lcl|At4g33750.1	lcl|F44F4.3 CE00999 WBGene0	gi|28571910|	gi|42661558|ref|XP_378786.1|	gi|6801547|ref|Os02g16440|
gi|31229265|ref|XP_318199.1|	lcl|At4g33800.1	lcl|F44F4.9 CE01004 WBGene0	gi|28571948|	gi|42661560|ref|XP_378787.1|	gi|6801549|ref|Os02g16460|
gi|31229273|ref|XP_318201.1|	lcl|At4g33960.1	lcl|F44G4.2 CE02229 WBGene0	gi|28571982|	gi|42661565|ref|XP_378791.1|	gi|6801550|ref|Os02g16470|
gi|31229281|ref|XP_318203.1|	lcl|At4g33980.1	lcl|F44G4.3 CE02230 WBGene0	gi|28572044|	gi|42661569|ref|XP_378793.1|	gi|6801551|ref|Os02g16480|
gi|31229287|ref|XP_318204.1|	lcl|At4g34265.1	lcl|F44G4.5 CE02232 WBGene0	gi|28572046|	gi|42661577|ref|XP_375599.1|	gi|6801552|ref|Os02g16490|
gi|31229291|ref|XP_318205.1|	lcl|At4g34550.1	lcl|F44G4.6 CE28558 WBGene0	gi|28572055|	gi|42661585|ref|XP_378795.1|	gi|6801556|ref|Os02g16530|
gi|31229299|ref|XP_318207.1|	lcl|At4g34560.1	lcl|F44G4.7 CE37837 WBGene0	gi|28572118|	gi|42661587|ref|XP_378796.1|	gi|6801559|ref|Os02g16560|
gi|31229317|ref|XP_318210.1|	lcl|At4g34600.1	lcl|F45B8.3 CE23731 WBGene0	gi|28572960|	gi|42661600|ref|XP_378798.1|	gi|6801560|ref|Os02g16570|
gi|31229352|ref|XP_318216.1|	lcl|At4g34630.1	lcl|F45D11.10 CE23733 WBGen	gi|28573008|	gi|42661605|ref|XP_378799.1|	gi|6801563|ref|Os02g16600|
gi|31229357|ref|XP_318217.1|	lcl|At4g34690.1	lcl|F45D11.9 CE23741 WBGene	gi|28573129|	gi|42661609|ref|XP_211447.2|	gi|6801572|ref|Os02g16690|
gi|31229399|ref|XP_318223.1|	lcl|At4g35165.1	lcl|F45D3.1 CE10468 WBGene0	gi|28573171|	gi|42661613|ref|XP_375608.1|	gi|6801574|ref|Os02g16710|
gi|31229407|ref|XP_318224.1|	lcl|At4g35170.1	lcl|F45D3.2 CE10470 WBGene0	gi|28573175|	gi|42661617|ref|XP_378800.1|	gi|6801577|ref|Os02g16740|
gi|31229411|ref|XP_318225.1|	lcl|At4g35400.1	lcl|F45D3.3 CE10472 WBGene0	gi|28573177|	gi|42661619|ref|XP_378801.1|	gi|6801578|ref|Os02g16750|
gi|31229472|ref|XP_318234.1|	lcl|At4g35510.1	lcl|F45D3.4 CE10474 WBGene0	gi|28573179|	gi|42661620|ref|XP_097351.3|	gi|6801580|ref|Os02g16770|
gi|31229519|ref|XP_318241.1|	lcl|At4g35580.1	lcl|F45E1.1 CE01938 WBGene0	gi|28573234|	gi|42661632|ref|XP_373805.2|	gi|6801592|ref|Os02g16890|
gi|31229526|ref|XP_318242.1|	lcl|At4g35725.1	lcl|F45E1.2 CE01939 WBGene0	gi|28573259|	gi|42661635|ref|XP_378804.1|	gi|6801593|ref|Os02g16900|
gi|31229533|ref|XP_318243.1|	lcl|At4g36170.1	lcl|F45E1.4 CE01941 WBGene0	gi|28573268|	gi|42661637|ref|XP_378805.1|	gi|6801594|ref|Os02g16910|
gi|31229554|ref|XP_318246.1|	lcl|At4g36230.1	lcl|F45E1.5 CE10486 WBGene0	gi|28573282|	gi|42661639|ref|XP_378806.1|	gi|6801596|ref|Os02g16930|
gi|31229568|ref|XP_318248.1|	lcl|At4g36370.1	lcl|F45E12.6 CE02742 WBGene	gi|28573308|	gi|42661646|ref|XP_378807.1|	gi|6801605|ref|Os02g17020|
gi|31229623|ref|XP_318256.1|	lcl|At4g36460.1	lcl|F45E4.10a CE31701 WBGen	gi|28573393|	gi|42661657|ref|XP_378810.1|	gi|6801606|ref|Os02g17030|
gi|31229628|ref|XP_318257.1|	lcl|At4g36500.1	lcl|F45E4.10b CE31702 WBGen	gi|28573439|	gi|42661659|ref|XP_375629.1|	gi|6801615|ref|Os02g17120|
gi|31229636|ref|XP_318259.1|	lcl|At4g36510.1	lcl|F45E4.3b CE24981 WBGene	gi|28573448|	gi|42661675|ref|XP_378812.1|	gi|6801616|ref|Os02g17130|
gi|31229643|ref|XP_318260.1|	lcl|At4g36560.1	lcl|F45E4.4 CE10498 WBGene0	gi|28573486|	gi|42661723|ref|XP_375658.1|	gi|6801620|ref|Os02g17170|
gi|31229660|ref|XP_318263.1|	lcl|At4g36655.1	lcl|F45E4.5 CE35313 WBGene0	gi|28573562|	gi|42661836|ref|XP_375548.1|	gi|6801621|ref|Os02g17180|
gi|31229680|ref|XP_318266.1|	lcl|At4g36925.1	lcl|F45E4.8 CE10506 WBGene0	gi|28573563|	gi|42661838|ref|XP_375549.1|	gi|6801623|ref|Os02g17200|
gi|31229708|ref|XP_318272.1|	lcl|At4g37290.1	lcl|F45E6.1 CE03336 WBGene0	gi|28573606|	gi|42661846|ref|XP_375551.1|	gi|6801625|ref|Os02g17220|
gi|31229736|ref|XP_318277.1|	lcl|At4g37295.1	lcl|F45E6.6 CE17830 WBGene0	gi|28573609|	gi|42661848|ref|XP_378765.1|	gi|6801629|ref|Os02g17260|
gi|31229755|ref|XP_318280.1|	lcl|At4g37450.1	lcl|F45G2.7 CE16050 WBGene0	gi|28573621|	gi|42661855|ref|XP_378766.1|	gi|6801630|ref|Os02g17270|
gi|31229762|ref|XP_318281.1|	lcl|At4g37685.1	lcl|F45H10.4 CE10548 WBGene	gi|28573623|	gi|42661860|ref|XP_378767.1|	gi|6801633|ref|Os02g17300|
gi|31229792|ref|XP_318285.1|	lcl|At4g37700.1	lcl|F45H10.5 CE32890 WBGene	gi|28573639|	gi|42661862|ref|XP_378769.1|	gi|6801638|ref|Os02g17340|
gi|31229821|ref|XP_318289.1|	lcl|At4g37810.1	lcl|F45H11.3 CE33785 WBGene	gi|28573657|	gi|42661864|ref|XP_378770.1|	gi|6801642|ref|Os02g17380|
gi|31229851|ref|XP_318295.1|	lcl|At4g37860.1	lcl|F45H7.1 CE28934 WBGene0	gi|28573658|	gi|42661951|ref|XP_378180.1|	gi|6801645|ref|Os02g17410|
gi|31229857|ref|XP_318296.1|	lcl|At4g38060.1	lcl|F46A8.6 CE34187 WBGene0	gi|28573659|	gi|42661953|ref|XP_378776.1|	gi|6801648|ref|Os02g17440|
gi|31229864|ref|XP_318297.1|	lcl|At4g38080.1	lcl|F46A8.7 CE10570 WBGene0	gi|28573670|	gi|42661957|ref|XP_378777.1|	gi|6801649|ref|Os02g17450|
gi|31229912|ref|XP_318305.1|	lcl|At4g38330.1	lcl|F46A9.1 CE10572 WBGene0	gi|28573671|	gi|42661992|ref|XP_378780.1|	gi|6801651|ref|Os02g17470|
gi|31229929|ref|XP_318308.1|	lcl|At4g38410.1	lcl|F46A9.2 CE10574 WBGene0	gi|28573719|	gi|42662006|ref|XP_375583.1|	gi|6801652|ref|Os02g17480|
gi|31229933|ref|XP_318309.1|	lcl|At4g38820.1	lcl|F46B3.14 CE18693 WBGene	gi|28573721|	gi|42662011|ref|XP_378783.1|	gi|6801655|ref|Os02g17510|
gi|31229938|ref|XP_318310.1|	lcl|At4g38980.1	lcl|F46B3.15 CE18694 WBGene	gi|28573723|	gi|42662015|ref|XP_375589.1|	gi|6801658|ref|Os02g17540|
gi|31229945|ref|XP_318311.1|	lcl|At4g39130.1	lcl|F46B3.16 CE20809 WBGene	gi|28573725|	gi|42662021|ref|XP_378784.1|	gi|6801660|ref|Os02g17560|
gi|31229954|ref|XP_318312.1|	lcl|At4g39320.1	lcl|F46B3.17 CE33787 WBGene	gi|28573727|	gi|42662023|ref|XP_373798.2|	gi|6801662|ref|Os02g17580|
gi|31229969|ref|XP_318314.1|	lcl|At4g39360.1	lcl|F46B3.9 CE18691 WBGene0	gi|28573731|	gi|42662082|ref|XP_378992.1|	gi|6801663|ref|Os02g17590|
gi|31229976|ref|XP_318315.1|	lcl|At4g39675.1	lcl|F46B6.10 CE05876 WBGene	gi|28573737|	gi|42662084|ref|XP_378993.1|	gi|6801665|ref|Os02g17610|
gi|31229984|ref|XP_318316.1|	lcl|At4g39745.1	lcl|F46B6.11 CE05877 WBGene	gi|28573747|	gi|42662087|ref|XP_378994.1|	gi|6801667|ref|Os02g17630|
gi|31229990|ref|XP_318317.1|	lcl|At4g39750.1	lcl|F46B6.2 CE05868 WBGene0	gi|28573753|	gi|42662089|ref|XP_375925.1|	gi|6801670|ref|Os02g17660|
gi|31229997|ref|XP_318318.1|	lcl|At4g39900.1	lcl|F46B6.5a CE33788 WBGene	gi|28573755|	gi|42662218|ref|XP_378978.1|	gi|6801672|ref|Os02g17680|
gi|31230005|ref|XP_318319.1|	lcl|At4g39930.1	lcl|F46B6.5b CE33789 WBGene	gi|28573757|	gi|42662220|ref|XP_378980.1|	gi|6801673|ref|Os02g17690|
gi|31230012|ref|XP_318320.1|	lcl|At4g40070.1	lcl|F46B6.5c CE34023 WBGene	gi|28573759|	gi|42662222|ref|XP_375917.1|	gi|6801681|ref|Os02g17770|
gi|31230033|ref|XP_318323.1|	lcl|At4g40090.1	lcl|F46B6.9 CE26975 WBGene0	gi|28573807|	gi|42662226|ref|XP_378981.1|	gi|6801687|ref|Os02g17830|
gi|31230040|ref|XP_318324.1|	lcl|At5g01080.1	lcl|F46C3.2 CE03341 WBGene0	gi|28573818|	gi|42662228|ref|XP_378982.1|	gi|6801688|ref|Os02g17840|
gi|31230048|ref|XP_318325.1|	lcl|At5g01225.1	lcl|F46C5.1 CE03343 WBGene0	gi|28573822|	gi|42662230|ref|XP_378983.1|	gi|6801693|ref|Os02g17890|
gi|31230054|ref|XP_318326.1|	lcl|At5g01350.1	lcl|F46C5.10 CE32891 WBGene	gi|28573836|	gi|42662232|ref|XP_378985.1|	gi|6801699|ref|Os02g17930|
gi|31230096|ref|XP_318335.1|	lcl|At5g01370.1	lcl|F46C5.2 CE03344 WBGene0	gi|28573954|	gi|42662239|ref|XP_211627.2|	gi|6801710|ref|Os02g18010|
gi|31230148|ref|XP_318346.1|	lcl|At5g01570.1	lcl|F46C5.4 CE03346 WBGene0	gi|28573980|	gi|42662245|ref|XP_378988.1|	gi|6801711|ref|Os02g18020|
gi|31230181|ref|XP_318350.1|	lcl|At5g01590.1	lcl|F46C5.7 CE03348 WBGene0	gi|28573999|	gi|42662249|ref|XP_375902.1|	gi|6801714|ref|Os02g18050|
gi|31230187|ref|XP_318351.1|	lcl|At5g01910.1	lcl|F46C8.1 CE04576 WBGene0	gi|28574004|	gi|42662257|ref|XP_378957.1|	gi|6801719|ref|Os02g18100|
gi|31230200|ref|XP_318353.1|	lcl|At5g02000.1	lcl|F46C8.3 CE28334 WBGene0	gi|28574006|	gi|42662260|ref|XP_373904.2|	gi|6801720|ref|Os02g18110|
gi|31230226|ref|XP_318357.1|	lcl|At5g02020.1	lcl|F46C8.8 CE35539 WBGene0	gi|28574018|	gi|42662268|ref|XP_378961.1|	gi|6801722|ref|Os02g18130|
gi|31230244|ref|XP_318360.1|	lcl|At5g02020.2	lcl|F46E10.11 CE25890 WBGen	gi|28574039|	gi|42662270|ref|XP_378964.1|	gi|6801724|ref|Os02g18150|

gi|31230298|ref|XP_318366.1|	lcl|At5g02090.1	lcl|F46E10.2 CE20813 WBGene	gi|28574055|	gi|42662274|ref|XP_378969.1|	gi|6801725|ref|Os02g18160|
gi|31230431|ref|XP_318385.1|	lcl|At5g02160.1	lcl|F46F11.7 CE36162 WBGene	gi|28574063|	gi|42662277|ref|XP_378970.1|	gi|6801726|ref|Os02g18170|
gi|31230475|ref|XP_318392.1|	lcl|At5g02200.1	lcl|F46F11.8 CE26921 WBGene	gi|28574075|	gi|42662279|ref|XP_378971.1|	gi|6801729|ref|Os02g18190|
gi|31230489|ref|XP_318394.1|	lcl|At5g02390.1	lcl|F46F2.3 CE05880 WBGene0	gi|28574081|	gi|42662283|ref|XP_375912.1|	gi|6801732|ref|Os02g18220|
gi|31230499|ref|XP_318396.1|	lcl|At5g02420.1	lcl|F46F2.4 CE05881 WBGene0	gi|28574083|	gi|42662285|ref|XP_378973.1|	gi|6801735|ref|Os02g18250|
gi|31230517|ref|XP_318399.1|	lcl|At5g02440.1	lcl|F46F2.5 CE34866 WBGene0	gi|28574085|	gi|42662289|ref|XP_378974.1|	gi|6801740|ref|Os02g18300|
gi|31230543|ref|XP_318404.1|	lcl|At5g02520.1	lcl|F46F3.3 CE10590 WBGene0	gi|28574087|	gi|42662296|ref|XP_378976.1|	gi|6801745|ref|Os02g18350|
gi|31230595|ref|XP_318414.1|	lcl|At5g02550.1	lcl|F46F5.16 CE19432 WBGene	gi|28574096|	gi|42662302|ref|XP_378989.1|	gi|6801749|ref|Os02g18390|
gi|31230837|ref|XP_318435.1|	lcl|At5g02650.1	lcl|F46F5.5 CE19421 WBGene0	gi|28574137|	gi|42662304|ref|XP_375929.1|	gi|6801750|ref|Os02g18400|
gi|31230880|ref|XP_318442.1|	lcl|At5g02670.1	lcl|F46F5.6 CE19422 WBGene0	gi|28574139|	gi|42662314|ref|XP_375935.1|	gi|6801752|ref|Os02g18420|
gi|31230889|ref|XP_318443.1|	lcl|At5g02690.1	lcl|F46F5.8 CE19424 WBGene0	gi|28574170|	gi|42662319|ref|XP_379002.1|	gi|6801757|ref|Os02g18470|
gi|31230905|ref|XP_318446.1|	lcl|At5g02720.1	lcl|F46F5.9 CE19425 WBGene0	gi|28574212|	gi|42662321|ref|XP_379003.1|	gi|6801759|ref|Os02g18490|
gi|31230915|ref|XP_318448.1|	lcl|At5g03050.1	lcl|F46F6.1b CE18698 WBGene	gi|28574218|	gi|42662324|ref|XP_379004.1|	gi|6801767|ref|Os02g18570|
gi|31230999|ref|XP_318450.1|	lcl|At5g03060.1	lcl|F46F6.3 CE02236 WBGene0	gi|28574232|	gi|42662327|ref|XP_379006.1|	gi|6801768|ref|Os02g18580|
gi|31231018|ref|XP_318453.1|	lcl|At5g03090.1	lcl|F46G10.2 CE31521 WBGene	gi|28574235|	gi|42662330|ref|XP_373932.2|	gi|6801770|ref|Os02g18600|
gi|31231060|ref|XP_318460.1|	lcl|At5g03110.1	lcl|F46G11.2 CE37647 WBGene	gi|28574249|	gi|42662332|ref|XP_379009.1|	gi|6801771|ref|Os02g18610|
gi|31231211|ref|XP_318483.1|	lcl|At5g03120.1	lcl|F46G11.4 CE28337 WBGene	gi|28574251|	gi|42662334|ref|XP_375941.1|	gi|6801772|ref|Os02g18620|
gi|31231241|ref|XP_318489.1|	lcl|At5g03130.1	lcl|F46H5.2a CE30801 WBGene	gi|28574313|	gi|42662336|ref|XP_379011.1|	gi|6801773|ref|Os02g18630|
gi|31231252|ref|XP_318491.1|	lcl|At5g03190.1	lcl|F46H5.2b CE34024 WBGene	gi|28574344|	gi|42662340|ref|XP_379014.1|	gi|6801774|ref|Os02g18640|
gi|31231276|ref|XP_318495.1|	lcl|At5g03210.1	lcl|F46H5.2c CE34025 WBGene	gi|28574348|	gi|42662342|ref|XP_379015.1|	gi|6801781|ref|Os02g18710|
gi|31231321|ref|XP_318504.1|	lcl|At5g03400.1	lcl|F46H5.7a CE04593 WBGene	gi|28574364|	gi|42662344|ref|XP_379016.1|	gi|6801785|ref|Os02g18750|
gi|31231326|ref|XP_318505.1|	lcl|At5g03420.1	lcl|F46H5.7b CE31170 WBGene	gi|28574376|	gi|42662346|ref|XP_379017.1|	gi|6801787|ref|Os02g18770|
gi|31231349|ref|XP_318509.1|	lcl|At5g03545.1	lcl|F46H5.7c CE34026 WBGene	gi|28574378|	gi|42662348|ref|XP_379018.1|	gi|6801788|ref|Os02g18780|
gi|31231360|ref|XP_318511.1|	lcl|At5g03550.1	lcl|F46H6.4 CE04598 WBGene0	gi|28574460|	gi|42662350|ref|XP_379020.1|	gi|6801789|ref|Os02g18790|
gi|31231362|ref|XP_318512.1|	lcl|At5g03670.1	lcl|F46H6.5 CE37113 WBGene0	gi|28574466|	gi|42662352|ref|XP_379021.1|	gi|6801790|ref|Os02g18800|
gi|31231515|ref|XP_318539.1|	lcl|At5g03710.1	lcl|F47A4.2 CE16058 WBGene0	gi|28574473|	gi|42662354|ref|XP_379022.1|	gi|6801796|ref|Os02g18860|
gi|31231595|ref|XP_318551.1|	lcl|At5g03890.1	lcl|F47B10.3 CE03353 WBGene	gi|28574503|	gi|42662356|ref|XP_379023.1|	gi|6801801|ref|Os02g18910|
gi|31231707|ref|XP_318574.1|	lcl|At5g03920.1	lcl|F47B10.4 CE03354 WBGene	gi|28574505|	gi|42662358|ref|XP_371418.2|	gi|6801802|ref|Os02g18920|
gi|31231764|ref|XP_318589.1|	lcl|At5g03930.1	lcl|F47B10.5 CE37376 WBGene	gi|28574547|	gi|42662360|ref|XP_375945.1|	gi|6801806|ref|Os02g18960|
gi|31231820|ref|XP_318598.1|	lcl|At5g03950.1	lcl|F47B10.6 CE03356 WBGene	gi|28574577|	gi|42662362|ref|XP_379025.1|	gi|6801811|ref|Os02g19010|
gi|31231853|ref|XP_318604.1|	lcl|At5g03960.1	lcl|F47B10.8 CE32424 WBGene	gi|28574604|	gi|42662364|ref|XP_373939.2|	gi|6801812|ref|Os02g19020|
gi|31231891|ref|XP_318610.1|	lcl|At5g04030.1	lcl|F47B7.6 CE02747 WBGene0	gi|28574606|	gi|42662374|ref|XP_379012.1|	gi|6801815|ref|Os02g19050|
gi|31231926|ref|XP_318616.1|	lcl|At5g04190.1	lcl|F47B7.7 CE35544 WBGene0	gi|28574613|	gi|42662376|ref|XP_375946.1|	gi|6801817|ref|Os02g19070|
gi|31231947|ref|XP_318620.1|	lcl|At5g04300.1	lcl|F47B8.1 CE10632 WBGene0	gi|28574745|	gi|42662378|ref|XP_379029.1|	gi|6801821|ref|Os02g19110|
gi|31231953|ref|XP_318621.1|	lcl|At5g04470.1	lcl|F47B8.11 CE10652 WBGene	gi|28574747|	gi|42662380|ref|XP_379030.1|	gi|6801822|ref|Os02g19120|
gi|31231957|ref|XP_318622.1|	lcl|At5g04650.1	lcl|F47B8.2 CE10634 WBGene0	gi|28574749|	gi|42662386|ref|XP_379032.1|	gi|6801826|ref|Os02g19160|
gi|31232104|ref|XP_318649.1|	lcl|At5g04750.1	lcl|F47B8.3 CE10636 WBGene0	gi|28574751|	gi|42662391|ref|XP_375953.1|	gi|6801835|ref|Os02g19240|
gi|31232109|ref|XP_318650.1|	lcl|At5g04790.1	lcl|F47B8.4 CE10638 WBGene0	gi|28574766|	gi|42662412|ref|XP_378189.1|	gi|6801837|ref|Os02g19260|
gi|31232113|ref|XP_318651.1|	lcl|At5g04880.1	lcl|F47D12.6 CE01950 WBGene	gi|28574771|	gi|42662416|ref|XP_379036.1|	gi|6801840|ref|Os02g19290|
gi|31232128|ref|XP_318653.1|	lcl|At5g05020.1	lcl|F47F2.2 CE04604 WBGene0	gi|28574780|	gi|42662441|ref|XP_379040.1|	gi|6801843|ref|Os02g19320|
gi|31232163|ref|XP_318658.1|	lcl|At5g05220.1	lcl|F47F2.3 CE35545 WBGene0	gi|28574810|	gi|42662445|ref|XP_379041.1|	gi|6801845|ref|Os02g19340|
gi|31232181|ref|XP_318661.1|	lcl|At5g05230.1	lcl|F47F6.1a CE29325 WBGene	gi|28574820|	gi|42662455|ref|XP_379046.1|	gi|6801847|ref|Os02g19360|
gi|31232206|ref|XP_318665.1|	lcl|At5g05250.1	lcl|F47F6.4 CE10710 WBGene0	gi|28574828|	gi|42662457|ref|XP_376003.1|	gi|6801849|ref|Os02g19380|
gi|31232211|ref|XP_318666.1|	lcl|At5g05300.1	lcl|F47G3.1 CE29327 WBGene0	gi|28574875|	gi|42662467|ref|XP_379052.1|	gi|6801857|ref|Os02g19460|
gi|31232230|ref|XP_318668.1|	lcl|At5g05430.1	lcl|F47G3.3 CE04608 WBGene0	gi|28574876|	gi|42662477|ref|XP_379054.1|	gi|6801867|ref|Os02g19540|
gi|31232309|ref|XP_318680.1|	lcl|At5g05640.1	lcl|F47G4.5 CE18703 WBGene0	gi|28574903|	gi|42662481|ref|XP_379055.1|	gi|6801869|ref|Os02g19560|
gi|31232320|ref|XP_318682.1|	lcl|At5g05965.1	lcl|F47G4.8 CE18706 WBGene0	gi|28575008|	gi|42662504|ref|XP_376019.1|	gi|6801874|ref|Os02g19610|
gi|31232357|ref|XP_318691.1|	lcl|At5g06010.1	lcl|F47G6.3 CE34310 WBGene0	gi|45549029|	gi|42662506|ref|XP_376020.1|	gi|6801877|ref|Os02g19640|
gi|31232366|ref|XP_318693.1|	lcl|At5g06040.1	lcl|F47G9.6 CE05887 WBGene0	gi|45549117|	gi|42662511|ref|XP_379060.1|	gi|6801880|ref|Os02g19660|
gi|31232378|ref|XP_318695.1|	lcl|At5g06190.1	lcl|F48A11.2 CE36855 WBGene	gi|45549188|	gi|42662513|ref|XP_376021.1|	gi|6801883|ref|Os02g19690|
gi|31232382|ref|XP_318696.1|	lcl|At5g06280.1	lcl|F48A11.3 CE10726 WBGene	gi|45549253|	gi|42662515|ref|XP_379064.1|	gi|6801884|ref|Os02g19700|
gi|31232387|ref|XP_318697.1|	lcl|At5g06380.1	lcl|F48A11.4 CE32668 WBGene	gi|45549293|	gi|42662519|ref|XP_379065.1|	gi|6801885|ref|Os02g19710|
gi|31232393|ref|XP_318698.1|	lcl|At5g06630.1	lcl|F48A9.1 CE10716 WBGene0	gi|45549295|	gi|42662521|ref|XP_376022.1|	gi|6801888|ref|Os02g19740|
gi|31232410|ref|XP_318700.1|	lcl|At5g06640.1	lcl|F48A9.2 CE10718 WBGene0	gi|45549306|	gi|42662534|ref|XP_379721.1|	gi|6801892|ref|Os02g19780|
gi|31232422|ref|XP_318702.1|	lcl|At5g06890.1	lcl|F48B9.1 CE04609 WBGene0	gi|45549344|	gi|42662538|ref|XP_379702.1|	gi|6801905|ref|Os02g19900|
gi|31232447|ref|XP_318707.1|	lcl|At5g06980.1	lcl|F48B9.2 CE04610 WBGene0	gi|45549357|	gi|42662542|ref|XP_379703.1|	gi|6801906|ref|Os02g19910|
gi|31232456|ref|XP_318709.1|	lcl|At5g06980.2	lcl|F48B9.3 CE04611 WBGene0	gi|45549363|	gi|42662544|ref|XP_379704.1|	gi|6801908|ref|Os02g19930|
gi|31232494|ref|XP_318715.1|	lcl|At5g07170.1	lcl|F48B9.4 CE30276 WBGene0	gi|45549389|	gi|42662552|ref|XP_379705.1|	gi|6801910|ref|Os02g19950|
gi|31232507|ref|XP_318717.1|	lcl|At5g07240.1	lcl|F48B9.8 CE04616 WBGene0	gi|45549457|	gi|42662563|ref|XP_378118.1|	gi|6801911|ref|Os02g19960|
gi|31232520|ref|XP_318719.1|	lcl|At5g07490.1	lcl|F48C1.3 CE10736 WBGene0	gi|45549577|	gi|42662566|ref|XP_377000.1|	gi|6801913|ref|Os02g19980|
gi|31232560|ref|XP_318725.1|	lcl|At5g07510.1	lcl|F48C1.4 CE10738 WBGene0	gi|45550130|	gi|42662568|ref|XP_377002.1|	gi|6801915|ref|Os02g20000|
gi|31232574|ref|XP_318728.1|	lcl|At5g07520.1	lcl|F48C1.5 CE10740 WBGene0	gi|45550134|	gi|42662572|ref|XP_378116.1|	gi|6801918|ref|Os02g20030|
gi|31232584|ref|XP_318729.1|	lcl|At5g07530.1	lcl|F48C1.6 CE10742 WBGene0	gi|45550187|	gi|42662587|ref|XP_379717.1|	gi|6801919|ref|Os02g20040|
gi|31232598|ref|XP_318731.1|	lcl|At5g07540.1	lcl|F48C1.7 CE10744 WBGene0	gi|45550358|	gi|42662624|ref|XP_379714.1|	gi|6801921|ref|Os02g20060|
gi|31232660|ref|XP_318739.1|	lcl|At5g07540.2	lcl|F48C1.8 CE10746 WBGene0	gi|45550383|	gi|42662631|ref|XP_379715.1|	gi|6801924|ref|Os02g20090|
gi|31232724|ref|XP_318749.1|	lcl|At5g07600.1	lcl|F48C1.9 CE31350 WBGene0	gi|45550403|	gi|42662635|ref|XP_379716.1|	gi|6801928|ref|Os02g20130|
gi|31232749|ref|XP_318753.1|	lcl|At5g07730.1	lcl|F48C11.3 CE10752 WBGene	gi|45550408|	gi|42662639|ref|XP_377053.1|	gi|6801930|ref|Os02g20150|
gi|31232806|ref|XP_318762.1|	lcl|At5g08010.1	lcl|F48D6.4a CE28400 WBGene	gi|45550417|	gi|42662650|ref|XP_379695.1|	gi|6801933|ref|Os02g20180|
gi|31232912|ref|XP_318777.1|	lcl|At5g08090.1	lcl|F48D6.4b CE07261 WBGene	gi|45550439|	gi|42662652|ref|XP_379696.1|	gi|6801938|ref|Os02g20230|
gi|31232918|ref|XP_318778.1|	lcl|At5g08141.1	lcl|F48D6.4c CE29328 WBGene	gi|45550461|	gi|42662663|ref|XP_379697.1|	gi|6801944|ref|Os02g20290|
gi|31232926|ref|XP_318782.1|	lcl|At5g08150.1	lcl|F48E3.6 CE02753 WBGene0	gi|45550476|	gi|42662686|ref|XP_379728.1|	gi|6801945|ref|Os02g20300|
gi|31232959|ref|XP_318789.1|	lcl|At5g08210.1	lcl|F48E3.9 CE26615 WBGene0	gi|45550499|	gi|42662694|ref|XP_379694.1|	gi|6801949|ref|Os02g20340|
gi|31232970|ref|XP_318793.1|	lcl|At5g08220.1	lcl|F48F5.2 CE34030 WBGene0	gi|45550517|	gi|42662703|ref|XP_379730.1|	gi|6801953|ref|Os02g20370|
gi|31232972|ref|XP_318794.1|	lcl|At5g08240.1	lcl|F48F5.3 CE18709 WBGene0	gi|45550522|	gi|42662707|ref|XP_379693.1|	gi|6801954|ref|Os02g20380|
gi|31232974|ref|XP_318795.1|	lcl|At5g08480.1	lcl|F48F5.6 CE35876 WBGene0	gi|45550540|	gi|42662709|ref|XP_379760.1|	gi|6801955|ref|Os02g20380|
gi|31232976|ref|XP_318796.1|	lcl|At5g08480.2	lcl|F48F7.4 CE05891 WBGene0	gi|45550545|	gi|42662713|ref|XP_376978.1|	gi|6801962|ref|Os02g20450|
gi|31233048|ref|XP_318808.1|	lcl|At5g08770.1	lcl|F48F7.5 CE05892 WBGene0	gi|45550548|	gi|42662715|ref|XP_379722.1|	gi|6801967|ref|Os02g20500|
gi|31233065|ref|XP_318811.1|	lcl|At5g09460.1	lcl|F48F7.6 CE05893 WBGene0	gi|45550558|	gi|42662717|ref|XP_379723.1|	gi|6801968|ref|Os02g20510|
gi|31233076|ref|XP_318813.1|	lcl|At5g09480.1	lcl|F48G7.13 CE10778 WBGene	gi|45550575|	gi|42662722|ref|XP_377076.1|	gi|6801970|ref|Os02g20530|
gi|31233122|ref|XP_318821.1|	lcl|At5g09520.1	lcl|F48G7.2 CE33099 WBGene0	gi|45550588|	gi|42662725|ref|XP_292122.4|	gi|6801972|ref|Os02g20550|
gi|31233209|ref|XP_318834.1|	lcl|At5g09940.1	lcl|F48G7.4 CE10760 WBGene0	gi|45550598|	gi|42662729|ref|XP_379700.1|	gi|6801973|ref|Os02g20560|
gi|31233276|ref|XP_318845.1|	lcl|At5g09980.1	lcl|F49B2.3 CE16074 WBGene0	gi|45550640|	gi|42662749|ref|XP_377098.1|	gi|6801985|ref|Os02g20670|
gi|31233291|ref|XP_318848.1|	lcl|At5g09990.1	lcl|F49B2.4 CE28560 WBGene0	gi|45550688|	gi|42662751|ref|XP_377102.1|	gi|6801986|ref|Os02g20680|
gi|31233372|ref|XP_318860.1|	lcl|At5g10040.1	lcl|F49C12.11 CE03371 WBGen	gi|45550702|	gi|42662755|ref|XP_379735.1|	gi|6801988|ref|Os02g20700|
gi|31233377|ref|XP_318861.1|	lcl|At5g10110.1	lcl|F49C12.15 CE03375 WBGen	gi|45550708|	gi|42662757|ref|XP_379761.1|	gi|6801989|ref|Os02g20710|
gi|31233407|ref|XP_318867.1|	lcl|At5g10430.1	lcl|F49C5.3 CE37917 WBGene0	gi|45550714|	gi|42662761|ref|XP_377087.1|	gi|6801991|ref|Os02g20730|
gi|31233465|ref|XP_318880.1|	lcl|At5g10590.1	lcl|F49C5.5 CE16082 WBGene0	gi|45550732|	gi|42662765|ref|XP_379736.1|	gi|6801992|ref|Os02g20740|
gi|31233505|ref|XP_318888.1|	lcl|At5g10660.1	lcl|F49C5.7 CE16084 WBGene0	gi|45550745|	gi|42662774|ref|XP_379686.1|	gi|6801993|ref|Os02g20750|
gi|31233525|ref|XP_318892.1|	lcl|At5g10670.1	lcl|F49D11.2 CE17076 WBGene	gi|45550747|	gi|42662776|ref|XP_376968.1|	gi|6801996|ref|Os02g20780|
gi|31233564|ref|XP_318897.1|	lcl|At5g10695.1	lcl|F49D11.7 CE17081 WBGene	gi|45550757|	gi|42662780|ref|XP_379688.1|	gi|6801997|ref|Os02g20790|
gi|31233569|ref|XP_318898.1|	lcl|At5g10850.1	lcl|F49E10.3 CE07264 WBGene	gi|45550762|	gi|42662784|ref|XP_379690.1|	gi|6801999|ref|Os02g20810|
gi|31233634|ref|XP_318910.1|	lcl|At5g11070.1	lcl|F49E10.4a CE35716 WBGen	gi|45550766|	gi|42662786|ref|XP_379691.1|	gi|6802000|ref|Os02g20820|
gi|31233644|ref|XP_318915.1|	lcl|At5g11120.1	lcl|F49E10.4b CE31176 WBGen	gi|45550827|	gi|42662788|ref|XP_379692.1|	gi|6802001|ref|Os02g20830|
gi|31233657|ref|XP_318920.1|	lcl|At5g11220.1	lcl|F49E11.8 CE05904 WBGene	gi|45550863|	gi|4502761|ref|NP_001794.1|	gi|6802007|ref|Os02g20880|
gi|31233671|ref|XP_318924.1|	lcl|At5g11360.1	lcl|F49E12.12 CE34871 WBGen	gi|45550878|	gi|4503085|ref|NP_001881.1|	gi|6802009|ref|Os02g20900|
gi|31233714|ref|XP_318934.1|	lcl|At5g11440.1	lcl|F49E12.2 CE03379 WBGene	gi|45550889|	gi|4503119|ref|NP_001318.1|	gi|6802010|ref|Os02g20910|
gi|31233724|ref|XP_318938.1|	lcl|At5g11530.1	lcl|F49E12.8 CE03384 WBGene	gi|45550892|	gi|4503181|ref|NP_003919.1|	gi|6802011|ref|Os02g20920|
gi|31233728|ref|XP_318939.1|	lcl|At5g11550.1	lcl|F49E7.2 CE10784 WBGene0	gi|45550899|	gi|4504057|ref|NP_001492.1|	gi|6802015|ref|Os02g20960|
gi|31233868|ref|XP_318965.1|	lcl|At5g11630.1	lcl|F49E8.6 CE10796 WBGene0	gi|45550915|	gi|4504231|ref|NP_002093.1|	gi|6802018|ref|Os02g20990|
gi|31233875|ref|XP_318966.1|	lcl|At5g11740.1	lcl|F49E8.7a CE10798 WBGene	gi|45550917|	gi|4504433|ref|NP_002122.1|	gi|6802019|ref|Os02g21000|
gi|31233878|ref|XP_318967.1|	lcl|At5g11830.1	lcl|F49E8.7b CE37238 WBGene	gi|45550931|	gi|4504529|ref|NP_002150.1|	gi|6802022|ref|Os02g21030|
gi|31233885|ref|XP_318968.1|	lcl|At5g11990.1	lcl|F49F1.12 CE17095 WBGene	gi|45550941|	gi|4504693|ref|NP_003660.1|	gi|6802024|ref|Os02g21050|
gi|31233895|ref|XP_318970.1|	lcl|At5g12050.1	lcl|F49F1.3 CE25894 WBGene0	gi|45550942|	gi|4504711|ref|NP_002186.1|	gi|6802033|ref|Os02g21120|
gi|31233900|ref|XP_318971.1|	lcl|At5g12235.1	lcl|F49H12.2 CE20838 WBGene	gi|45550986|	gi|4504739|ref|NP_003483.1|	gi|6802038|ref|Os02g21170|
gi|31233911|ref|XP_318972.1|	lcl|At5g12240.1	lcl|F49H12.4 CE34564 WBGene	gi|45550994|	gi|4505183|ref|NP_002405.1|	gi|6802039|ref|Os02g21180|
gi|31233919|ref|XP_318973.1|	lcl|At5g12360.1	lcl|F49H6.11 CE18720 WBGene	gi|45551013|	gi|4506769|ref|NP_002954.1|	gi|6802043|ref|Os02g21220|
gi|31233941|ref|XP_318976.1|	lcl|At5g12880.1	lcl|F49H6.12 CE19879 WBGene	gi|45551014|	gi|4557347|ref|NP_001178.1|	gi|6802048|ref|Os02g21270|
gi|31234056|ref|XP_318992.1|	lcl|At5g12990.1	lcl|F49H6.8 CE18717 WBGene0	gi|45551029|	gi|4557381|ref|NP_001204.1|	gi|6802050|ref|Os02g21290|
gi|31234061|ref|XP_318993.1|	lcl|At5g13250.1	lcl|F52A8.1 CE05908 WBGene0	gi|45551079|	gi|4557653|ref|NP_000191.1|	gi|6802052|ref|Os02g21310|
gi|31234101|ref|XP_319003.1|	lcl|At5g13310.1	lcl|F52A8.3a CE05910 WBGene	gi|45551082|	gi|4557725|ref|NP_000418.1|	gi|6802054|ref|Os02g21330|
gi|31234105|ref|XP_319004.1|	lcl|At5g13470.1	lcl|F52A8.3b CE37019 WBGene	gi|45551093|	gi|4757964|ref|NP_004056.1|	gi|6802058|ref|Os02g21370|
gi|31234164|ref|XP_319015.1|	lcl|At5g13590.1	lcl|F52A8.6b CE28936 WBGene	gi|45551095|	gi|4758100|ref|NP_004700.1|	gi|6802060|ref|Os02g21390|
gi|31234240|ref|XP_319029.1|	lcl|At5g13825.1	lcl|F52B11.5 CE18725 WBGene	gi|45551109|	gi|4759256|ref|NP_004900.1|	gi|6802062|ref|Os02g21410|
gi|31234262|ref|XP_319030.1|	lcl|At5g14090.1	lcl|F52B11.6 CE18726 WBGene	gi|45551115|	gi|4759304|ref|NP_004670.1|	gi|6802063|ref|Os02g21420|
gi|31234275|ref|XP_319032.1|	lcl|At5g14160.1	lcl|F52C6.13 CE17099 WBGene	gi|45551144|	gi|4826944|ref|NP_005033.1|	gi|6802065|ref|Os02g21440|
gi|31234319|ref|XP_319039.1|	lcl|At5g14330.1	lcl|F52C6.14 CE10826 WBGene	gi|45551148|	gi|4885059|ref|NP_005153.1|	gi|6802066|ref|Os02g21450|
gi|31234351|ref|XP_319045.1|	lcl|At5g14380.1	lcl|F52C6.2 CE32669 WBGene0	gi|45551154|	gi|5031565|ref|NP_005741.1|	gi|6802068|ref|Os02g21470|
gi|31234377|ref|XP_319050.1|	lcl|At5g14410.1	lcl|F52C9.7 CE04619 WBGene0	gi|45551194|	gi|5031655|ref|NP_005858.1|	gi|6802069|ref|Os02g21480|
gi|31234426|ref|XP_319059.1|	lcl|At5g14560.1	lcl|F52C9.8a CE30802 WBGene	gi|45551195|	gi|5031859|ref|NP_005878.1|	gi|6802071|ref|Os02g21500|
gi|31234435|ref|XP_319060.1|	lcl|At5g14690.1	lcl|F52D1.2 CE35717 WBGene0	gi|45551409|	gi|5031935|ref|NP_005812.1|	gi|6802078|ref|Os02g21570|
gi|31234510|ref|XP_319074.1|	lcl|At5g14730.1	lcl|F52D2.4 CE23757 WBGene0	gi|45551411|	gi|5174495|ref|NP_006012.1|	gi|6802082|ref|Os02g21610|
gi|31234529|ref|XP_319077.1|	lcl|At5g14830.1	lcl|F52D2.5 CE35718 WBGene0	gi|45551443|	gi|5174693|ref|NP_005979.1|	gi|6802086|ref|Os02g21650|
gi|31234554|ref|XP_319080.1|	lcl|At5g14840.1	lcl|F52D2.6 CE20842 WBGene0	gi|45551473|	gi|5174762|ref|NP_005945.1|	gi|6802089|ref|Os02g21670|
gi|31234583|ref|XP_319084.1|	lcl|At5g14930.1	lcl|F52D2.7 CE23758 WBGene0	gi|45551500|	gi|5174764|ref|NP_005944.1|	gi|6802090|ref|Os02g21680|
gi|31234617|ref|XP_319090.1|	lcl|At5g15000.1	lcl|F52E1.12 CE04640 WBGene	gi|45551524|	gi|5453625|ref|NP_006356.1|	gi|6802091|ref|Os02g21690|
gi|31234627|ref|XP_319091.1|	lcl|At5g15060.1	lcl|F52E1.3 CE04631 WBGene0	gi|45551557|	gi|5453756|ref|NP_006307.1|	gi|6802096|ref|Os02g21740|
gi|31234632|ref|XP_319092.1|	lcl|At5g15190.1	lcl|F52E1.5 CE33105 WBGene0	gi|45551811|	gi|5454008|ref|NP_006385.1|	gi|6802098|ref|Os02g21760|
gi|31234688|ref|XP_319099.1|	lcl|At5g15190.2	lcl|F52E1.6 CE04634 WBGene0	gi|45551819|	gi|5729869|ref|NP_006534.1|	gi|6802099|ref|Os02g21770|
gi|31234764|ref|XP_319112.1|	lcl|At5g15320.1	lcl|F52E1.9 CE35324 WBGene0	gi|45551835|	gi|5729962|ref|NP_006676.1|	gi|6802104|ref|Os02g21820|
gi|31234791|ref|XP_319117.1|	lcl|At5g15360.1	lcl|F52E10.2 CE03394 WBGene	gi|45551919|	gi|5729966|ref|NP_006665.1|	gi|6802105|ref|Os02g21830|
gi|31234801|ref|XP_319119.1|	lcl|At5g15420.1	lcl|F52E10.3 CE03395 WBGene	gi|45551931|	gi|5730061|ref|NP_006683.1|	gi|6802107|ref|Os02g21850|
gi|31234806|ref|XP_319120.1|	lcl|At5g15560.1	lcl|F52E10.4 CE03396 WBGene	gi|45551934|	gi|5730063|ref|NP_006533.1|	gi|6802108|ref|Os02g21860|
gi|31234825|ref|XP_319124.1|	lcl|At5g15580.1	lcl|F52E4.5 CE07272 WBGene0	gi|45551947|	gi|5802976|ref|NP_006820.1|	gi|6802109|ref|Os02g21870|
gi|31234848|ref|XP_319128.1|	lcl|At5g15690.1	lcl|F52E4.6 CE07273 WBGene0	gi|45551967|	gi|5881413|ref|NP_000492.1|	gi|6802113|ref|Os02g21910|

gi|31234859|ref|XP_319130.1|	lcl|At5g15725.1	lcl|F52E4.8 CE09875 WBGene0	gi|45551971|	gi|5901966|ref|NP_009002.1|	gi|6802116|ref|Os02g21930|
gi|31234884|ref|XP_319136.1|	lcl|At5g15802.1	lcl|F52F10.1 CE36858 WBGene	gi|45551991|	gi|5902120|ref|NP_008876.1|	gi|6802122|ref|Os02g21990|
gi|31234965|ref|XP_319158.1|	lcl|At5g15960.1	lcl|F52F12.5 CE10848 WBGene	gi|45552002|	gi|5921995|ref|NP_005880.1|	gi|6802124|ref|Os02g22010|
gi|31234980|ref|XP_319160.1|	lcl|At5g15970.1	lcl|F52G2.2 CE17848 WBGene0	gi|45552071|	gi|6005802|ref|NP_009175.1|	gi|6802133|ref|Os02g22100|
gi|31235089|ref|XP_319179.1|	lcl|At5g15990.1	lcl|F52G2.3 CE35553 WBGene0	gi|45552095|	gi|6005878|ref|NP_009036.1|	gi|6802134|ref|Os02g22100|
gi|31235118|ref|XP_319183.1|	lcl|At5g16030.1	lcl|F52G3.4 CE10860 WBGene0	gi|45552113|	gi|6005910|ref|NP_009164.1|	gi|6802135|ref|Os02g22100|
gi|31235176|ref|XP_319191.1|	lcl|At5g16100.1	lcl|F52H2.1 CE34312 WBGene0	gi|45552247|	gi|6382067|ref|NP_009295.1|	gi|6802138|ref|Os02g22110|
gi|31235183|ref|XP_319192.1|	lcl|At5g16110.1	lcl|F52H2.3 CE10868 WBGene0	gi|45552307|	gi|6466464|ref|NP_015560.1|	gi|6802139|ref|Os02g22120|
gi|31235192|ref|XP_319193.1|	lcl|At5g16160.1	lcl|F53A2.2 CE16094 WBGene0	gi|45552321|	gi|6680081|ref|NP_031380.1|	gi|6802142|ref|Os02g22150|
gi|31235196|ref|XP_319194.1|	lcl|At5g16200.1	lcl|F53A2.3 CE16095 WBGene0	gi|45552327|	gi|6912578|ref|NP_036522.1|	gi|6802147|ref|Os02g22200|
gi|31235203|ref|XP_319195.1|	lcl|At5g16285.1	lcl|F53A2.9 CE16101 WBGene0	gi|45552329|	gi|6912700|ref|NP_036383.1|	gi|6802154|ref|Os02g22270|
gi|31235250|ref|XP_319204.1|	lcl|At5g16430.1	lcl|F53A3.1 CE10880 WBGene0	gi|45552331|	gi|7019343|ref|NP_037362.1|	gi|6802160|ref|Os02g22330|
gi|31235312|ref|XP_319215.1|	lcl|At5g17130.1	lcl|F53A3.4 CE28443 WBGene0	gi|45552333|	gi|7106371|ref|NP_037475.1|	gi|6802162|ref|Os02g22350|
gi|31235320|ref|XP_319217.1|	lcl|At5g17150.1	lcl|F53A3.6 CE30999 WBGene0	gi|45552365|	gi|7305625|ref|NP_038480.1|	gi|6802172|ref|Os02g22440|
gi|31235351|ref|XP_319227.1|	lcl|At5g17180.1	lcl|F53A9.1 CE02760 WBGene0	gi|45552381|	gi|7656859|ref|NP_055301.1|	gi|6802173|ref|Os02g22450|
gi|31235395|ref|XP_319237.1|	lcl|At5g17300.1	lcl|F53A9.2 CE02762 WBGene0	gi|45552387|	gi|7657021|ref|NP_055364.1|	gi|6802174|ref|Os02g22460|
gi|31235406|ref|XP_319239.1|	lcl|At5g17340.1	lcl|F53A9.3 CE30140 WBGene0	gi|45552389|	gi|7657156|ref|NP_055169.1|	gi|6802175|ref|Os02g22470|
gi|31235420|ref|XP_319241.1|	lcl|At5g17360.1	lcl|F53A9.4 CE30141 WBGene0	gi|45552395|	gi|7657158|ref|NP_055171.1|	gi|6802177|ref|Os02g22490|
gi|31235573|ref|XP_319263.1|	lcl|At5g17590.1	lcl|F53A9.6 CE02766 WBGene0	gi|45552399|	gi|7657259|ref|NP_055471.1|	gi|6802179|ref|Os02g22510|
gi|31235738|ref|XP_319292.1|	lcl|At5g17650.1	lcl|F53A9.7 CE02767 WBGene0	gi|45552401|	gi|7657685|ref|NP_055172.1|	gi|6802182|ref|Os02g22540|
gi|31235782|ref|XP_319300.1|	lcl|At5g17950.1	lcl|F53A9.8 CE02768 WBGene0	gi|45552409|	gi|7661600|ref|NP_056397.1|	gi|6802186|ref|Os02g22580|
gi|31235788|ref|XP_319301.1|	lcl|At5g18040.1	lcl|F53A9.9 CE02769 WBGene0	gi|45552423|	gi|7661692|ref|NP_056215.1|	gi|6802187|ref|Os02g22590|
gi|31235809|ref|XP_319304.1|	lcl|At5g18065.1	lcl|F53B1.3 CE04643 WBGene0	gi|45552435|	gi|7661724|ref|NP_056187.1|	gi|6802188|ref|Os02g22600|
gi|31235817|ref|XP_319305.1|	lcl|At5g18150.1	lcl|F53B2.3 CE05917 WBGene0	gi|45552507|	gi|7661750|ref|NP_054866.1|	gi|6802190|ref|Os02g22620|
gi|31235962|ref|XP_319328.1|	lcl|At5g18310.1	lcl|F53B2.8 CE28030 WBGene0	gi|45552523|	gi|7661752|ref|NP_054867.1|	gi|6802195|ref|Os02g22670|
gi|31235982|ref|XP_319331.1|	lcl|At5g18310.2	lcl|F53B3.3 CE37117 WBGene0	gi|45552549|	gi|7661760|ref|NP_054871.1|	gi|6802197|ref|Os02g22690|
gi|31235987|ref|XP_319332.1|	lcl|At5g18690.1	lcl|F53B3.6 CE04446 WBGene0	gi|45552553|	gi|7661780|ref|NP_054881.1|	gi|6802199|ref|Os02g22710|
gi|31236012|ref|XP_319338.1|	lcl|At5g18850.1	lcl|F53B6.8 CE23765 WBGene0	gi|45552609|	gi|7661782|ref|NP_054882.1|	gi|6802203|ref|Os02g22750|
gi|31236030|ref|XP_319342.1|	lcl|At5g18920.1	lcl|F53B6.9 CE30539 WBGene0	gi|45552611|	gi|7661814|ref|NP_054898.1|	gi|6802205|ref|Os02g22770|
gi|31236096|ref|XP_319354.1|	lcl|At5g19190.1	lcl|F53B7.4 CE05923 WBGene0	gi|45552613|	gi|7661924|ref|NP_055607.1|	gi|6802210|ref|Os02g22820|
gi|31236106|ref|XP_319356.1|	lcl|At5g19260.1	lcl|F53B7.7 CE34879 WBGene0	gi|45552617|	gi|7662520|ref|NP_054832.1|	gi|6802212|ref|Os02g23830|
gi|31236163|ref|XP_319367.1|	lcl|At5g19340.1	lcl|F53B8.1 CE16102 WBGene0	gi|45552625|	gi|7662526|ref|NP_054835.1|	gi|6802219|ref|Os02g23900|
gi|31236260|ref|XP_319382.1|	lcl|At5g19800.1	lcl|F53C11.4 CE17853 WBGene	gi|45552627|	gi|7662536|ref|NP_054840.1|	gi|6802220|ref|Os02g23910|
gi|31236266|ref|XP_319383.1|	lcl|At5g19810.1	lcl|F53C11.5 CE17854 WBGene	gi|45552631|	gi|7662548|ref|NP_054845.1|	gi|6802221|ref|Os02g23920|
gi|31236310|ref|XP_319390.1|	lcl|At5g20100.1	lcl|F53C3.7 CE19445 WBGene0	gi|45552641|	gi|7662552|ref|NP_054846.1|	gi|6802222|ref|Os02g23930|
gi|31236447|ref|XP_319411.1|	lcl|At5g20120.1	lcl|F53C3.8 CE19446 WBGene0	gi|45552671|	gi|7662560|ref|NP_054848.1|	gi|6802223|ref|Os02g23940|
gi|31236458|ref|XP_319413.1|	lcl|At5g20130.1	lcl|F53E10.3 CE36861 WBGene	gi|45552789|	gi|7662574|ref|NP_054852.1|	gi|6802225|ref|Os02g23960|
gi|31236463|ref|XP_319414.1|	lcl|At5g20200.1	lcl|F53E10.4 CE10926 WBGene	gi|45552795|	gi|7662576|ref|NP_054853.1|	gi|6802230|ref|Os02g24010|
gi|31236542|ref|XP_319432.1|	lcl|At5g20370.1	lcl|F53E10.5 CE10928 WBGene	gi|45552839|	gi|7662578|ref|NP_054854.1|	gi|6802232|ref|Os02g24030|
gi|31236546|ref|XP_319433.1|	lcl|At5g20460.1	lcl|F53F1.4 CE10938 WBGene0	gi|45552867|	gi|7662611|ref|NP_054818.1|	gi|6802233|ref|Os02g24040|
gi|31236560|ref|XP_319435.1|	lcl|At5g20635.1	lcl|F53F1.5 CE10940 WBGene0	gi|45552869|	gi|7662621|ref|NP_054823.1|	gi|6802234|ref|Os02g24050|
gi|31236564|ref|XP_319436.1|	lcl|At5g20760.1	lcl|F53F1.6 CE10942 WBGene0	gi|45552871|	gi|7662625|ref|NP_054825.1|	gi|6802239|ref|Os02g24100|
gi|31236652|ref|XP_319452.1|	lcl|At5g20770.1	lcl|F53F10.5 CE10988 WBGene	gi|45552877|	gi|7662635|ref|NP_054830.1|	gi|6802241|ref|Os02g24120|
gi|31236726|ref|XP_319466.1|	lcl|At5g20790.1	lcl|F53F4.1 CE10954 WBGene0	gi|45552933|	gi|7662661|ref|NP_054787.1|	gi|6802246|ref|Os02g24160|
gi|31236745|ref|XP_319469.1|	lcl|At5g21020.1	lcl|F53F4.12 CE36385 WBGene	gi|45552959|	gi|7662663|ref|NP_054863.1|	gi|6802250|ref|Os02g24200|
gi|31236826|ref|XP_319482.1|	lcl|At5g21110.1	lcl|F53F4.13 CE10978 WBGene	gi|45552969|	gi|7662665|ref|NP_054788.1|	gi|6802251|ref|Os02g24210|
gi|31236832|ref|XP_319483.1|	lcl|At5g21125.1	lcl|F53F4.15 CE34880 WBGene	gi|45552971|	gi|7662675|ref|NP_056184.1|	gi|6802252|ref|Os02g24220|
gi|31236836|ref|XP_319484.1|	lcl|At5g21280.1	lcl|F53F4.16 CE36935 WBGene	gi|45552999|	gi|7705431|ref|NP_057017.1|	gi|6802254|ref|Os02g24240|
gi|31236879|ref|XP_319493.1|	lcl|At5g21910.1	lcl|F53F4.2 CE10956 WBGene0	gi|45553021|	gi|7705656|ref|NP_056938.1|	gi|6802256|ref|Os02g24260|
gi|31236884|ref|XP_319494.1|	lcl|At5g21940.1	lcl|F53F4.4 CE10960 WBGene0	gi|45553033|	gi|7705871|ref|NP_057265.1|	gi|6802259|ref|Os02g24290|
gi|31236914|ref|XP_319500.1|	lcl|At5g22150.1	lcl|F53F4.6 CE10964 WBGene0	gi|45553035|	gi|7705933|ref|NP_057410.1|	gi|6802267|ref|Os02g24370|
gi|31236940|ref|XP_319511.1|	lcl|At5g22160.1	lcl|F53F8.6 CE34881 WBGene0	gi|45553037|	gi|7705945|ref|NP_057433.1|	gi|6802268|ref|Os02g24380|
gi|31236952|ref|XP_319514.1|	lcl|At5g22170.1	lcl|F53G12.4 CE36696 WBGene	gi|45553041|	gi|7705969|ref|NP_057464.1|	gi|6802269|ref|Os02g24390|
gi|31236961|ref|XP_319516.1|	lcl|At5g22180.1	lcl|F53G12.8 CE36697 WBGene	gi|45553043|	gi|7705971|ref|NP_057467.1|	gi|6802270|ref|Os02g24400|
gi|31236965|ref|XP_319517.1|	lcl|At5g22190.1	lcl|F53G12.9 CE30376 WBGene	gi|45553045|	gi|7705973|ref|NP_057496.1|	gi|6802271|ref|Os02g24410|
gi|31236995|ref|XP_319523.1|	lcl|At5g22210.1	lcl|F53G2.2 CE36862 WBGene0	gi|45553047|	gi|7705995|ref|NP_057533.1|	gi|6802274|ref|Os02g24440|
gi|31237005|ref|XP_319525.1|	lcl|At5g22270.1	lcl|F53G2.3 CE36863 WBGene0	gi|45553049|	gi|7706198|ref|NP_057730.1|	gi|6802275|ref|Os02g24450|
gi|31237030|ref|XP_319529.1|	lcl|At5g22310.1	lcl|F53G2.4a CE32673 WBGene	gi|45553051|	gi|7706587|ref|NP_057674.1|	gi|6802276|ref|Os02g24460|
gi|31237130|ref|XP_319547.1|	lcl|At5g22390.1	lcl|F53G2.4b CE32674 WBGene	gi|45553053|	gi|7706743|ref|NP_057296.1|	gi|6802277|ref|Os02g24470|
gi|31237161|ref|XP_319553.1|	lcl|At5g22430.1	lcl|F53G2.8 CE11004 WBGene0	gi|45553097|	gi|7706759|ref|NP_057462.1|	gi|6802279|ref|Os02g24490|
gi|31237216|ref|XP_319564.1|	lcl|At5g22520.1	lcl|F53H1.4b CE33651 WBGene	gi|45553155|	gi|7706761|ref|NP_057463.1|	gi|6802280|ref|Os02g24500|
gi|31237223|ref|XP_319566.1|	lcl|At5g22530.1	lcl|F53H1.4c CE33652 WBGene	gi|45553157|	gi|7706771|ref|NP_056939.1|	gi|6802284|ref|Os02g24540|
gi|31237228|ref|XP_319567.1|	lcl|At5g22545.1	lcl|F53H2.2 CE19887 WBGene0	gi|45553171|	gi|8393250|ref|NP_059114.1|	gi|6802286|ref|Os02g24560|
gi|31237241|ref|XP_319570.1|	lcl|At5g22555.1	lcl|F53H2.3 CE19888 WBGene0	gi|45553173|	gi|8393384|ref|NP_058643.1|	gi|6802291|ref|Os02g24610|
gi|31237257|ref|XP_319574.1|	lcl|At5g22650.2	lcl|F53H4.2 CE11030 WBGene0	gi|45553175|	gi|8922128|ref|NP_060068.1|	gi|6802296|ref|Os02g24660|
gi|31237355|ref|XP_319594.1|	lcl|At5g22680.1	lcl|F53H4.3 CE11032 WBGene0	gi|45553185|	gi|8922147|ref|NP_060076.1|	gi|6802301|ref|Os02g24710|
gi|31237389|ref|XP_319599.1|	lcl|At5g22930.1	lcl|F53H4.4 CE17863 WBGene0	gi|45553205|	gi|8922153|ref|NP_061175.1|	gi|6802305|ref|Os02g24750|
gi|31237409|ref|XP_319602.1|	lcl|At5g22970.1	lcl|F54A3.2 CE32675 WBGene0	gi|45553207|	gi|8922157|ref|NP_060927.1|	gi|6802311|ref|Os02g24810|
gi|31237431|ref|XP_319606.1|	lcl|At5g22990.1	lcl|F54A3.6 CE25902 WBGene0	gi|45553223|	gi|8922159|ref|NP_060005.1|	gi|6802312|ref|Os02g24820|
gi|31237457|ref|XP_319611.1|	lcl|At5g23160.1	lcl|F54A5.2 CE20860 WBGene0	gi|45553231|	gi|8922172|ref|NP_060892.1|	gi|6802313|ref|Os02g24830|
gi|31237464|ref|XP_319613.1|	lcl|At5g23180.1	lcl|F54A5.3c CE20863 WBGene	gi|45553239|	gi|8922175|ref|NP_060067.1|	gi|6802317|ref|Os02g24870|
gi|31237478|ref|XP_319616.1|	lcl|At5g23405.1	lcl|F54B11.10 CE05934 WBGen	gi|45553245|	gi|8922198|ref|NP_060446.1|	gi|6802321|ref|Os02g24910|
gi|31237491|ref|XP_319620.1|	lcl|At5g23405.2	lcl|F54B11.4 CE05928 WBGene	gi|45553261|	gi|8922259|ref|NP_060481.1|	gi|6802322|ref|Os02g24920|
gi|31237521|ref|XP_319625.1|	lcl|At5g23460.1	lcl|F54B11.7 CE05931 WBGene	gi|45553309|	gi|8922309|ref|NP_060508.1|	gi|6802323|ref|Os02g24930|
gi|31237533|ref|XP_319627.1|	lcl|At5g23640.1	lcl|F54B11.8 CE05932 WBGene	gi|45553311|	gi|8922459|ref|NP_060581.1|	gi|6802324|ref|Os02g24940|
gi|31237581|ref|XP_319636.1|	lcl|At5g24250.1	lcl|F54B11.9 CE05933 WBGene	gi|45553331|	gi|8922474|ref|NP_060588.1|	gi|6802326|ref|Os02g24960|
gi|31237606|ref|XP_319639.1|	lcl|At5g24313.1	lcl|F54B3.2 CE01590 WBGene0	gi|45553349|	gi|8922677|ref|NP_060694.1|	gi|6802331|ref|Os02g25010|
gi|31237750|ref|XP_319661.1|	lcl|At5g24316.1	lcl|F54B8.4 CE16106 WBGene0	gi|45553367|	gi|8922779|ref|NP_060747.1|	gi|6802333|ref|Os02g25030|
gi|31237754|ref|XP_319662.1|	lcl|At5g24355.1	lcl|F54B8.5 CE16107 WBGene0	gi|45553393|	gi|8922879|ref|NP_060799.1|	gi|6802335|ref|Os02g25050|
gi|31237782|ref|XP_319665.1|	lcl|At5g24460.1	lcl|F54C1.3a CE11046 WBGene	gi|45553433|	gi|8922919|ref|NP_060820.1|	gi|6802338|ref|Os02g25080|
gi|31237802|ref|XP_319668.1|	lcl|At5g24480.1	lcl|F54C1.3b CE36305 WBGene	gi|45553453|	gi|8923015|ref|NP_060088.1|	gi|6802342|ref|Os02g25120|
gi|31237827|ref|XP_319672.1|	lcl|At5g24500.1	lcl|F54C1.8 CE34315 WBGene0	gi|45553455|	gi|8923026|ref|NP_060094.1|	gi|6802343|ref|Os02g25130|
gi|31237833|ref|XP_319673.1|	lcl|At5g24570.1	lcl|F54C1.9 CE23768 WBGene0	gi|45553495|	gi|8923036|ref|NP_060100.1|	gi|6802344|ref|Os02g25140|
gi|31237838|ref|XP_319674.1|	lcl|At5g24590.1	lcl|F54C4.3 CE19892 WBGene0	gi|45553497|	gi|8923120|ref|NP_060144.1|	gi|6802345|ref|Os02g25150|
gi|31237841|ref|XP_319675.1|	lcl|At5g24640.1	lcl|F54C4.4 CE19893 WBGene0	gi|45553518|	gi|8923150|ref|NP_060159.1|	gi|6802346|ref|Os02g25160|
gi|31237845|ref|XP_319676.1|	lcl|At5g24880.1	lcl|F54C8.6 CE28832 WBGene0	gi|45553641|	gi|8923287|ref|NP_060226.1|	gi|6802349|ref|Os02g25190|
gi|31237868|ref|XP_319680.1|	lcl|At5g24915.1	lcl|F54C9.3 CE02253 WBGene0	gi|45553715|	gi|8923352|ref|NP_060262.1|	gi|6802350|ref|Os02g25200|
gi|31237889|ref|XP_319684.1|	lcl|At5g24920.1	lcl|F54C9.7 CE02257 WBGene0	gi|45553971|	gi|8923382|ref|NP_060278.1|	gi|6802351|ref|Os02g25210|
gi|31237905|ref|XP_319688.1|	lcl|At5g24980.1	lcl|F54D10.4 CE35977 WBGene	gi|45554124|	gi|8923435|ref|NP_060304.1|	gi|6802354|ref|Os02g25240|
gi|31238017|ref|XP_319709.1|	lcl|At5g25000.1	lcl|F54D10.5 CE19455 WBGene	gi|45554163|	gi|8923623|ref|NP_060398.1|	gi|6802357|ref|Os02g25270|
gi|31238180|ref|XP_319723.1|	lcl|At5g25200.1	lcl|F54D11.3 CE35325 WBGene	gi|45554838|	gi|8923630|ref|NP_060402.1|	gi|6802360|ref|Os02g25300|
gi|31238192|ref|XP_319725.1|	lcl|At5g25210.1	lcl|F54D11.4 CE29392 WBGene	gi|45554849|	gi|8923682|ref|NP_060429.1|	gi|6802366|ref|Os02g25360|
gi|31238246|ref|XP_319736.1|	lcl|At5g25240.1	lcl|F54D12.2 CE17867 WBGene	gi|45555160|	gi|8923688|ref|NP_060432.1|	gi|6802370|ref|Os02g25400|
gi|31238302|ref|XP_319743.1|	lcl|At5g25280.1	lcl|F54D12.4 CE17869 WBGene	gi|45555171|	gi|8923806|ref|NP_060940.1|	gi|6802371|ref|Os02g25410|
gi|31238441|ref|XP_319774.1|	lcl|At5g25280.2	lcl|F54D12.5 CE36864 WBGene	gi|45555580|	gi|8923907|ref|NP_061164.1|	gi|6802372|ref|Os02g25420|
gi|31238473|ref|XP_319781.1|	lcl|At5g25425.1	lcl|F54D12.9 CE36307 WBGene	gi|45555703|	gi|8923972|ref|NP_061032.1|	gi|6802376|ref|Os02g25460|
gi|31238496|ref|XP_319786.1|	lcl|At5g25475.1	lcl|F54D5.15a CE32896 WBGen	gi|45555814|	gi|8923995|ref|NP_061038.1|	gi|6802380|ref|Os02g25500|
gi|31238522|ref|XP_319792.1|	lcl|At5g25475.2	lcl|F54D5.15b CE37239 WBGen	gi|45555825|	gi|8924005|ref|NP_061042.1|	gi|6802381|ref|Os02g25510|
gi|31238579|ref|XP_319806.1|	lcl|At5g25600.1	lcl|F54D5.15c CE37240 WBGen	gi|45555930|	gi|8924026|ref|NP_061048.1|	gi|6802383|ref|Os02g25530|
gi|31238605|ref|XP_319811.1|	lcl|At5g25750.1	lcl|F54D5.2 CE36491 WBGene0	gi|45556010|	gi|8924054|ref|NP_061073.1|	gi|6802387|ref|Os02g25570|
gi|31238642|ref|XP_319816.1|	lcl|At5g25870.1	lcl|F54D5.3 CE28033 WBGene00010049 status:Confirmed SW:Q		gi|8924092|ref|NP_060980.1|	gi|6802389|ref|Os02g25590|
gi|31238666|ref|XP_319820.1|	lcl|At5g25920.1	lcl|F54D5.4 CE03409 WBGene00010050 status:Confirmed SW:Q		gi|8924096|ref|NP_061078.1|	gi|6802390|ref|Os02g25600|
gi|31238687|ref|XP_319823.1|	lcl|At5g26020.1	lcl|F54D5.5a CE36386 WBGene00010051 status:Partially_confir		gi|8924100|ref|NP_061080.1|	gi|6802391|ref|Os02g25610|
gi|31238733|ref|XP_319832.1|	lcl|At5g26070.1	lcl|F54D5.5b CE37920 WBGene00010051 status:Partially_confir		gi|8924110|ref|NP_060982.1|	gi|6802392|ref|Os02g25620|
gi|31238743|ref|XP_319834.1|	lcl|At5g26080.1	lcl|F54D8.5 CE01311 WBGene00018801 status:Partially_confirm		gi|8924147|ref|NP_060990.1|	gi|6802395|ref|Os02g25650|
gi|31238749|ref|XP_319837.1|	lcl|At5g26100.1	lcl|F54D8.6 CE31000 WBGene00018802 status:Partially_confirm		gi|9506643|ref|NP_061906.1|	gi|6802398|ref|Os02g25680|
gi|31238772|ref|XP_319842.1|	lcl|At5g26230.1	lcl|F54E2.2 CE17106 WBGene00018824 status:Partially_confirm		gi|9506923|ref|NP_061933.1|	gi|6802399|ref|Os02g25690|
gi|31238835|ref|XP_319857.1|	lcl|At5g26270.1	lcl|F54E4.3 CE11078 WBGene00010059 status:Partially_confirm		gi|9624988|ref|NP_062553.1|	gi|6802405|ref|Os02g25750|
gi|31238840|ref|XP_319858.1|	lcl|At5g26350.1	lcl|F54E7.5 CE01316 WBGene00018828 status:Partially_confirm		gi|9910206|ref|NP_064600.1|	gi|6802406|ref|Os02g25760|
gi|31238845|ref|XP_319859.1|	lcl|At5g26620.1	lcl|F54E7.6 CE01317 WBGene00018829 status:Partially_confirm		gi|9910226|ref|NP_064564.1|	gi|6802419|ref|Os02g25890|
gi|31238847|ref|XP_319860.1|	lcl|At5g26692.1	lcl|F54E7.8 CE26898 WBGene00018830 status:Confirmed SW:Q		gi|9910448|ref|NP_064622.1|	gi|6802422|ref|Os02g25920|
gi|31238872|ref|XP_319868.1|	lcl|At5g26717.1	lcl|F54F12.2 CE16115 WBGene00010073 status:Predicted SW:C		gi|9910450|ref|NP_064635.1|	gi|6802428|ref|Os02g25980|
gi|31238909|ref|XP_319876.1|	lcl|At5g26720.1	lcl|F54F2.2b CE26933 WBGene00006975 locus:zfp-1 status:Part		gi|9910496|ref|NP_064566.1|	gi|6802431|ref|Os02g26010|
gi|31238913|ref|XP_319877.1|	lcl|At5g26731.1	lcl|F54F2.5b CE32435 WBGene00018833 status:Confirmed SW:		gi|9945310|ref|NP_006241.1|	gi|6802437|ref|Os02g26070|
gi|31238935|ref|XP_319881.1|	lcl|At5g26790.1	lcl|F54F2.6 CE00199 WBGene00018834 status:Predicted SW:P3		gi|9966915|ref|NP_065200.1|	gi|6802442|ref|Os02g26120|
gi|31238943|ref|XP_319885.1|	lcl|At5g26800.1	lcl|F54F7.2 CE37524 WBGene00010064 status:Predicted SW:Q20789 protein_id:CAA91755.2 /QuerySize=322			gi|6802443|ref|Os02g26130|
gi|31238961|ref|XP_319894.1|	lcl|At5g26840.1	lcl|F54F7.3 CE03418 WBGene00010065 status:Partially_confirmed SW:Q20790 protein_id:CAA91756.1 /QuerySize=189			gi|6802445|ref|Os02g26150|
gi|31238981|ref|XP_319904.1|	lcl|At5g26890.1	lcl|F54F7.4 CE03419 WBGene00006965 locus:xtr-1 TRA-2 like protein status:Predicted SW:Q20791 protein_id:CAA91757.1 /Quer			gi|6802450|ref|Os02g26200|
gi|31238983|ref|XP_319905.1|	lcl|At5g26970.1	lcl|F54F7.6 CE03421 WBGene00010066 status:Partially_confirmed SW:Q20793 protein_id:CAA91759.1 /QuerySize=311			gi|6802452|ref|Os02g26220|
gi|31238985|ref|XP_319906.1|	lcl|At5g27020.1	lcl|F54F7.8 CE34882 WBGene00010068 status:Predicted SW:Q7YX02 protein_id:CAE17847.1 /QuerySize=209			gi|6802453|ref|Os02g26230|
gi|31238991|ref|XP_319909.1|	lcl|At5g27040.1	lcl|F54H12.2 CE00515 WBGene00018842 status:Partially_confirmed SW:P34456 protein_id:AAK84536.1 /QuerySize=419			gi|6802455|ref|Os02g26250|
gi|31238993|ref|XP_319910.1|	lcl|At5g27160.1	lcl|F54H12.4 CE00546 WBGene00018844 status:Partially_confirmed SW:P34458 protein_id:AAA28053.1 /QuerySize=343			gi|6802456|ref|Os02g26260|
gi|31239011|ref|XP_319919.1|	lcl|At5g27170.1	lcl|F55A11.8 CE05950 WBGene00010081 status:Partially_confirmed SW:Q20803 protein_id:CAA96661.1 /QuerySize=297			gi|6802458|ref|Os02g26280|
gi|31239025|ref|XP_319926.1|	lcl|At5g27180.1	lcl|F55A12.10 CE28451 WBGene00018867 status:Partially_confirmed SW:Q95R14 protein_id:AAK73876.1 /QuerySize=220			gi|6802460|ref|Os02g26300|
gi|31239035|ref|XP_319931.1|	lcl|At5g27260.1	lcl|F55A12.2a CE29396 WBGene00018863 status:Confirmed SW:O01760 protein_id:AAB54128.2 /QuerySize=175			gi|6802461|ref|Os02g26310|
gi|31239041|ref|XP_319934.1|	lcl|At5g27340.1	lcl|F55A12.2b CE30540 WBGene00018863 status:Confirmed SW:Q8T3H1 protein_id:AAM15579.1 /QuerySize=250			gi|6802463|ref|Os02g26330|
gi|31239073|ref|XP_319950.1|	lcl|At5g27440.1	lcl|F55A12.2c CE34317 WBGene00018863 status:Confirmed SW:Q7Z133 protein_id:AAP68939.1 /QuerySize=307			gi|6802468|ref|Os02g26380|
gi|31239081|ref|XP_319954.1|	lcl|At5g27500.1	lcl|F55A12.2d CE34318 WBGene00018863 status:Confirmed SW:Q7Z132 protein_id:AAP68940.1 /QuerySize=39			gi|6802469|ref|Os02g26390|
gi|31239095|ref|XP_319961.1|	lcl|At5g27590.1	lcl|F55A12.5 CE11125 WBGene00018864 status:Partially_confirmed SW:O01756 protein_id:AAB54123.1 /QuerySize=849			gi|6802477|ref|Os02g26470|
gi|31239127|ref|XP_319977.1|	lcl|At5g27606.1	lcl|F55A12.6 CE36649 WBGene00018865 status:Predicted SW:O01754 protein_id:AAB54124.2 /QuerySize=423			gi|6802479|ref|Os02g26490|
gi|31239191|ref|XP_320009.1|	lcl|At5g27800.1	lcl|F55A3.2 CE17112 WBGene00018848 status:Partially_confirmed SW:Q9N5R8 protein_id:AAF39834.1 /QuerySize=786			gi|6802482|ref|Os02g26520|
gi|31239207|ref|XP_320017.1|	lcl|At5g27890.1	lcl|F55A4.10 CE35556 WBGene00018861 status:Partially_confirmed SW:Q95Q52 protein_id:AAL06028.2 /QuerySize=633			gi|6802483|ref|Os02g26530|
gi|31239225|ref|XP_320026.1|	lcl|At5g28070.1	lcl|F55A4.2 CE31181 WBGene00018854 status:Partially_confirmed SW:Q94236 protein_id:AAB07560.2 /QuerySize=368			gi|6802484|ref|Os02g26540|
gi|31239249|ref|XP_320038.1|	lcl|At5g28090.1	lcl|F55A4.3 CE11103 WBGene00018855 status:Partially_confirmed SW:Q94237 protein_id:AAB07561.1 /QuerySize=70			gi|6802487|ref|Os02g26570|

<table>
<tr><td>gi|31239251|ref|XP_320039.1|</td><td>lcl|At5g28110.1</td><td>lcl|F55B11.2 CE32898 WBGene00010084 status:Partially_confirmed SW:O17891 protein_id:CAB05901.2 /QuerySize=220</td><td>gi|6802488|ref|Os02g26580|</td></tr>
<tr><td>gi|31239259|ref|XP_320043.1|</td><td>lcl|At5g28120.1</td><td>lcl|F55B11.3 CE36167 WBGene00010085 status:Partially_confirmed SW:O17893 protein_id:CAB05903.3 /QuerySize=231</td><td>gi|6802489|ref|Os02g26590|</td></tr>
<tr><td>gi|31239261|ref|XP_320044.1|</td><td>lcl|At5g28130.1</td><td>lcl|F55B11.5 CE36168 WBGene00010087 status:Predicted SW:Q7JKF1 protein_id:CAE54897.1 /QuerySize=203</td><td>gi|6802491|ref|Os02g26610|</td></tr>
<tr><td>gi|31239263|ref|XP_320045.1|</td><td>lcl|At5g28140.1</td><td>lcl|F55C12.4 CE19468 WBGene00018871 status:Confirmed SW:Q20808 protein_id:AAC71166.1 /QuerySize=220</td><td>gi|6802494|ref|Os02g26640|</td></tr>
<tr><td>gi|31239265|ref|XP_320046.1|</td><td>lcl|At5g28170.1</td><td>lcl|F55C5.10 CE34884 WBGene00010098 status:Predicted SW:Q7YTM4 protein_id:CAE17828.1 /QuerySize=164</td><td>gi|6802498|ref|Os02g26670|</td></tr>
<tr><td>gi|31239267|ref|XP_320047.1|</td><td>lcl|At5g28190.1</td><td>lcl|F55C7.2 CE11170 WBGene00018868 status:Partially_confirmed SW:P91335 protein_id:AAC71107.1 /QuerySize=88</td><td>gi|6802499|ref|Os02g26680|</td></tr>
<tr><td>gi|31239269|ref|XP_320048.1|</td><td>lcl|At5g28200.1</td><td>lcl|F55C9.5 CE18737 WBGene00010102 status:Partially_confirmed SW:Q9XUZ0 protein_id:CAB04472.1 /QuerySize=87</td><td>gi|6802511|ref|Os02g26730|</td></tr>
<tr><td>gi|31239289|ref|XP_320058.1|</td><td>lcl|At5g28240.1</td><td>lcl|F55D1.1 CE07275 WBGene00018875 status:Predicted SW:Q20825 protein_id:AAA93413.1 /QuerySize=91</td><td>gi|6802517|ref|Os02g26790|</td></tr>
<tr><td>gi|31239297|ref|XP_320062.1|</td><td>lcl|At5g28256.1</td><td>lcl|F55D1.2 CE07276 WBGene00018876 status:Predicted SW:Q20824 protein_id:AAA93412.1 /QuerySize=92</td><td>gi|6802521|ref|Os02g26830|</td></tr>
<tr><td>gi|31239301|ref|XP_320064.1|</td><td>lcl|At5g28260.1</td><td>lcl|F55D12.1 CE05955 WBGene00010110 status:Partially_confirmed SW:Q20830 protein_id:CAA99859.1 /QuerySize=141</td><td>gi|6802528|ref|Os02g26900|</td></tr>
<tr><td>gi|31239317|ref|XP_320072.1|</td><td>lcl|At5g28270.1</td><td>lcl|F55D12.6 CE17876 WBGene00010114 status:Partially_confirmed SW:O62264 protein_id:CAA99863.1 /QuerySize=179</td><td>gi|6802529|ref|Os02g26910|</td></tr>
<tr><td>gi|31239347|ref|XP_320087.1|</td><td>lcl|At5g28295.1</td><td>lcl|F55E10.1 CE02781 WBGene00018881 status:Predicted SW:Q20839 protein_id:AAC46530.1 /QuerySize=103</td><td>gi|6802534|ref|Os02g26960|</td></tr>
<tr><td>gi|31239355|ref|XP_320091.1|</td><td>lcl|At5g28320.1</td><td>lcl|F55E10.2 CE02782 WBGene00018882 status:Predicted SW:Q20838 protein_id:AAC46531.1 /QuerySize=97</td><td>gi|6802535|ref|Os02g26970|</td></tr>
<tr><td>gi|31239363|ref|XP_320095.1|</td><td>lcl|At5g28400.1</td><td>lcl|F55E10.4 CE02784 WBGene00018883 status:Predicted SW:Q20836 protein_id:AAC46533.1 /QuerySize=113</td><td>gi|6802541|ref|Os02g27020|</td></tr>
<tr><td>gi|31239377|ref|XP_320102.1|</td><td>lcl|At5g28410.1</td><td>lcl|F55E10.5 CE02785 WBGene00018884 status:Predicted SW:Q20837 protein_id:AAC46534.1 /QuerySize=423</td><td>gi|6802543|ref|Os02g27040|</td></tr>
<tr><td>gi|31239379|ref|XP_320103.1|</td><td>lcl|At5g28420.1</td><td>lcl|F55F1.1 CE30147 WBGene00018887 status:Predicted SW:Q20843 protein_id:AAL65789.1 /QuerySize=95</td><td>gi|6802547|ref|Os02g27080|</td></tr>
<tr><td>gi|31239381|ref|XP_320104.1|</td><td>lcl|At5g28430.1</td><td>lcl|F55F1.2 CE07278 WBGene00018888 status:Predicted SW:Q20842 protein_id:AAB00618.1 /QuerySize=141</td><td>gi|6802553|ref|Os02g27140|</td></tr>
<tr><td>gi|31239389|ref|XP_320108.1|</td><td>lcl|At5g28463.1</td><td>lcl|F55F3.4 CE18747 WBGene00010118 status:Predicted SW:Q9XUY3 protein_id:CAB04479.1 /QuerySize=881</td><td>gi|6802554|ref|Os02g27150|</td></tr>
<tr><td>gi|31239393|ref|XP_320110.1|</td><td>lcl|At5g28480.1</td><td>lcl|F55F8.6 CE11198 WBGene00018894 status:Confirmed SW:P91344 protein_id:AAB37810.1 /QuerySize=161</td><td>gi|6802556|ref|Os02g27170|</td></tr>
<tr><td>gi|31239399|ref|XP_320113.1|</td><td>lcl|At5g28482.1</td><td>lcl|F55F8.8 CE11202 WBGene00018896 status:Partially_confirmed SW:P91347 protein_id:AAB37813.1 /QuerySize=230</td><td>gi|6802557|ref|Os02g27180|</td></tr>
<tr><td>gi|31239413|ref|XP_320120.1|</td><td>lcl|At5g28484.1</td><td>lcl|F55G1.1 CE34885 WBGene00018899 status:Partially_confirmed SW:Q20850 protein_id:AAB00651.2 /QuerySize=256</td><td>gi|6802558|ref|Os02g27190|</td></tr>
<tr><td>gi|31239415|ref|XP_320121.1|</td><td>lcl|At5g28545.1</td><td>lcl|F55G1.15 CE35980 WBGene00018907 status:Partially_confirmed SW:Q20854 protein_id:AAB00655.4 /QuerySize=340</td><td>gi|6802560|ref|Os02g27210|</td></tr>
<tr><td>gi|31239423|ref|XP_320125.1|</td><td>lcl|At5g28560.1</td><td>lcl|F55G1.4 CE07281 WBGene00018900 locus:rod-1 status:Partially_confirmed SW:Q20849 protein_id:AAB00646.1 /QuerySize=2</td><td>gi|6802564|ref|Os02g27250|</td></tr>
<tr><td>gi|31239425|ref|XP_320126.1|</td><td>lcl|At5g28570.1</td><td>lcl|F55G1.6 CE34886 WBGene00018902 status:Partially_confirmed SW:Q20846 protein_id:AAB00643.2 /QuerySize=350</td><td>gi|6802565|ref|Os02g27260|</td></tr>
<tr><td>gi|31239427|ref|XP_320127.1|</td><td>lcl|At5g28580.1</td><td>lcl|F55G1.7 CE07284 WBGene00018903 status:Partially_confirmed SW:Q20844 protein_id:AAB00641.1 /QuerySize=307</td><td>gi|6802568|ref|Os02g27290|</td></tr>
<tr><td>gi|31239443|ref|XP_320135.1|</td><td>lcl|At5g28590.1</td><td>lcl|F55G11.1 CE19899 WBGene00010122 status:Partially_confirmed SW:Q9XTF2 protein_id:CAB05223.1 /QuerySize=337</td><td>gi|6802572|ref|Os02g27330|</td></tr>
<tr><td>gi|31239475|ref|XP_320151.1|</td><td>lcl|At5g28600.1</td><td>lcl|F55G11.10 CE18755 WBGene00010129 status:Predicted SW:Q9XUD9 protein_id:CAB05217.1 /QuerySize=280</td><td>gi|6802576|ref|Os02g27370|</td></tr>
<tr><td>gi|31239497|ref|XP_320162.1|</td><td>lcl|At5g28610.1</td><td>lcl|F55G7.1 CE03423 WBGene00010119 status:Partially_confirmed SW:Q20855 protein_id:CAA90609.1 /QuerySize=395</td><td>gi|6802579|ref|Os02g27410|</td></tr>
<tr><td>gi|31239505|ref|XP_320166.1|</td><td>lcl|At5g28615.1</td><td>lcl|F55G7.3 CE03425 WBGene00010121 status:Predicted SW:Q20857 protein_id:CAA90611.1 /QuerySize=212</td><td>gi|6802580|ref|Os02g27400|</td></tr>
<tr><td>gi|31239507|ref|XP_320167.1|</td><td>lcl|At5g28620.1</td><td>lcl|F55H12.2 CE32440 WBGene00010133 status:Confirmed SW:Q9XVM4 protein_id:CAB03141.2 /QuerySize=57</td><td>gi|6802587|ref|Os02g27450|</td></tr>
<tr><td>gi|31239511|ref|XP_320169.1|</td><td>lcl|At5g28622.1</td><td>lcl|F55H12.4 CE11212 WBGene00010135 status:Confirmed SW:P90889 protein_id:CAB03140.1 /QuerySize=208</td><td>gi|6802599|ref|Os02g27530|</td></tr>
<tr><td>gi|31239515|ref|XP_320171.1|</td><td>lcl|At5g28630.1</td><td>lcl|F55H2.7 CE34033 WBGene00010132 status:Partially_confirmed SW:P34467 protein_id:CAA81604.3 /QuerySize=379</td><td>gi|6802601|ref|Os02g27550|</td></tr>
<tr><td>gi|31239565|ref|XP_320196.1|</td><td>lcl|At5g28694.1</td><td>lcl|F56A11.6 CE36868 WBGene00018926 status:Partially_confirmed SW:O44519 protein_id:AAB92077.2 /QuerySize=442</td><td>gi|6802602|ref|Os02g27560|</td></tr>
<tr><td>gi|31239567|ref|XP_320197.1|</td><td>lcl|At5g28710.1</td><td>lcl|F56A11.7 CE33655 WBGene00018927 status:Predicted SW:Q86MG6 protein_id:AAO91713.1 /QuerySize=126</td><td>gi|6802603|ref|Os02g27570|</td></tr>
<tr><td>gi|31239569|ref|XP_320198.1|</td><td>lcl|At5g28720.1</td><td>lcl|F56A3.1 CE28460 WBGene00018908 status:Partially_confirmed SW:P91352 protein_id:AAB37805.2 /QuerySize=783</td><td>gi|6802604|ref|Os02g27580|</td></tr>
<tr><td>gi|31239575|ref|XP_320201.1|</td><td>lcl|At5g28785.1</td><td>lcl|F56A3.3a CE11218 WBGene00003792 locus:npp-6 status:Partially_confirmed SW:P91350 protein_id:AAB37803.1 /QuerySize=</td><td>gi|6802605|ref|Os02g27590|</td></tr>
<tr><td>gi|31239605|ref|XP_320216.1|</td><td>lcl|At5g28790.1</td><td>lcl|F56A3.3b CE30281 WBGene00003792 locus:npp-6 status:Partially_confirmed SW:Q8T8M4 protein_id:AAL77180.1 /QuerySize</td><td>gi|6802606|ref|Os02g27600|</td></tr>
<tr><td>gi|31239613|ref|XP_320220.1|</td><td>lcl|At5g28800.1</td><td>lcl|F56A6.4 CE34319 WBGene00018923 status:Partially_confirmed SW:Q9GZG5 protein_id:AAF99975.3 /QuerySize=479</td><td>gi|6802607|ref|Os02g27610|</td></tr>
<tr><td>gi|31239629|ref|XP_320228.1|</td><td>lcl|At5g28810.1</td><td>lcl|F56A8.3b CE16123 WBGene00010139 status:Confirmed SW:O45574 protein_id:CAB05743.1 /QuerySize=254</td><td>gi|6802612|ref|Os02g27660|</td></tr>
<tr><td>gi|31239675|ref|XP_320251.1|</td><td>lcl|At5g28820.1</td><td>lcl|F56A8.4 CE16124 WBGene00010140 status:Partially_confirmed SW:O45575 protein_id:CAB05744.1 /QuerySize=275</td><td>gi|6802613|ref|Os02g27670|</td></tr>
<tr><td>gi|31239687|ref|XP_320257.1|</td><td>lcl|At5g28823.1</td><td>lcl|F56A8.8 CE16128 WBGene00010142 status:Partially_confirmed SW:O45578 protein_id:CAB05748.1 /QuerySize=248</td><td>gi|6802616|ref|Os02g27700|</td></tr>
<tr><td>gi|31239731|ref|XP_320279.1|</td><td>lcl|At5g28860.1</td><td>lcl|F56B3.11b CE25908 WBGene00018934 status:Partially_confirmed SW:Q9BKP0 protein_id:AAK29832.1 /QuerySize=188</td><td>gi|6802624|ref|Os02g27780|</td></tr>
<tr><td>gi|31239777|ref|XP_320302.1|</td><td>lcl|At5g28870.1</td><td>lcl|F56B3.3 CE17884 WBGene00018929 status:Partially_confirmed SW:O45112 protein_id:AAK29828.1 /QuerySize=225</td><td>gi|6802632|ref|Os02g27860|</td></tr>
<tr><td>gi|31239785|ref|XP_320306.1|</td><td>lcl|At5g28880.1</td><td>lcl|F56B3.4a CE29969 WBGene00004824 locus:skr-18 status:Partially_confirmed SW:O45107 protein_id:AAL32223.1 /QuerySize=</td><td>gi|6802635|ref|Os02g27890|</td></tr>
<tr><td>gi|31239787|ref|XP_320307.1|</td><td>lcl|At5g28920.1</td><td>lcl|F56B3.4b CE37243 WBGene00004824 locus:skr-18 status:Partially_confirmed SW:Q688B1 protein_id:AAU05566.1 /QuerySize</td><td>gi|6802636|ref|Os02g27900|</td></tr>
<tr><td>gi|31239807|ref|XP_320317.1|</td><td>lcl|At5g28930.1</td><td>lcl|F56B3.9 CE34321 WBGene00018933 status:Partially_confirmed SW:O45111 protein_id:AAK29826.3 /QuerySize=169</td><td>gi|6802637|ref|Os02g27910|</td></tr>
<tr><td>gi|31239865|ref|XP_320346.1|</td><td>lcl|At5g28990.1</td><td>lcl|F56B6.6 CE04666 WBGene00018937 status:Predicted SW:Q22996 protein_id:AAB04561.1 /QuerySize=120</td><td>gi|6802643|ref|Os02g27970|</td></tr>
<tr><td>gi|31239869|ref|XP_320348.1|</td><td>lcl|At5g29010.1</td><td>lcl|F56C11.4 CE30543 WBGene00018956 status:Partially_confirmed SW:Q8T7Y8 protein_id:AAM15580.1 /QuerySize=229</td><td>gi|6802644|ref|Os02g27980|</td></tr>
<tr><td>gi|31239879|ref|XP_320353.1|</td><td>lcl|At5g29020.1</td><td>lcl|F56C11.5 CE34322 WBGene00018957 status:Partially_confirmed SW:O44976 protein_id:AAB97557.2 /QuerySize=230</td><td>gi|6802645|ref|Os02g27990|</td></tr>
<tr><td>gi|31239899|ref|XP_320363.1|</td><td>lcl|At5g29024.1</td><td>lcl|F56C3.1 CE17892 WBGene00018938 status:Predicted SW:O61760 protein_id:AAC17004.1 /QuerySize=286</td><td>gi|6802646|ref|Os02g28000|</td></tr>
<tr><td>gi|31239977|ref|XP_320402.1|</td><td>lcl|At5g29030.1</td><td>lcl|F56C3.3 CE17894 WBGene00018940 status:Predicted SW:O61753 protein_id:AAC17006.1 /QuerySize=166</td><td>gi|6802649|ref|Os02g28030|</td></tr>
<tr><td>gi|31239995|ref|XP_320411.1|</td><td>lcl|At5g29034.1</td><td>lcl|F56C3.4 CE17895 WBGene00018941 status:Predicted SW:O61754 protein_id:AAC17003.1 /QuerySize=344</td><td>gi|6802651|ref|Os02g28050|</td></tr>
<tr><td>gi|31240025|ref|XP_320426.1|</td><td>lcl|At5g29040.1</td><td>lcl|F56C3.5 CE17896 WBGene00018942 status:Predicted SW:O61755 protein_id:AAC17002.1 /QuerySize=211</td><td>gi|6802655|ref|Os02g28090|</td></tr>
<tr><td>gi|31240043|ref|XP_320435.1|</td><td>lcl|At5g29050.1</td><td>lcl|F56C3.6 CE17897 WBGene00018943 status:Partially_confirmed SW:O61756 protein_id:AAC17001.1 /QuerySize=399</td><td>gi|6802656|ref|Os02g28100|</td></tr>
<tr><td>gi|31240097|ref|XP_320462.1|</td><td>lcl|At5g29060.1</td><td>lcl|F56C3.7 CE17898 WBGene00018944 status:Predicted SW:O61757 protein_id:AAC17007.1 /QuerySize=64</td><td>gi|6802658|ref|Os02g28120|</td></tr>
<tr><td>gi|31240105|ref|XP_320466.1|</td><td>lcl|At5g29070.1</td><td>lcl|F56C3.8 CE17899 WBGene00018945 status:Partially_confirmed SW:O61759 protein_id:AAC17008.1 /QuerySize=69</td><td>gi|6802660|ref|Os02g28140|</td></tr>
<tr><td>gi|31240111|ref|XP_320469.1|</td><td>lcl|At5g29080.1</td><td>lcl|F56C4.1 CE35557 WBGene00010144 status:Partially_confirmed SW:Q20859 protein_id:CAA94288.2 /QuerySize=243</td><td>gi|6802662|ref|Os02g28160|</td></tr>
<tr><td>gi|31240119|ref|XP_320473.1|</td><td>lcl|At5g29090.1</td><td>lcl|F56C4.2 CE35558 WBGene00010145 status:Partially_confirmed SW:Q20860 protein_id:CAA94289.2 /QuerySize=183</td><td>gi|6802665|ref|Os02g28190|</td></tr>
<tr><td>gi|31240125|ref|XP_320476.1|</td><td>lcl|At5g29210.1</td><td>lcl|F56C9.11 CE31004 WBGene00018954 status:Confirmed SW:Q8WQE8 protein_id:AAL50325.2 /QuerySize=246</td><td>gi|6802667|ref|Os02g28210|</td></tr>
<tr><td>gi|31240141|ref|XP_320484.1|</td><td>lcl|At5g29570.1</td><td>lcl|F56C9.6 CE01325 WBGene00018950 status:Partially_confirmed SW:Q20863 protein_id:AAK18961.1 /QuerySize=329</td><td>gi|6802669|ref|Os02g28230|</td></tr>
<tr><td>gi|31240145|ref|XP_320486.1|</td><td>lcl|At5g29602.1</td><td>lcl|F56C9.8 CE01327 WBGene00018952 status:Partially_confirmed SW:Q20866 protein_id:AAK18963.1 /QuerySize=182</td><td>gi|6802673|ref|Os02g28270|</td></tr>
<tr><td>gi|31240161|ref|XP_320494.1|</td><td>lcl|At5g29613.1</td><td>lcl|F56D1.2 CE01970 WBGene00018960 status:Partially_confirmed SW:Q10128 protein_id:AAA81100.1 /QuerySize=718</td><td>gi|6802674|ref|Os02g28280|</td></tr>
<tr><td>gi|31240179|ref|XP_320503.1|</td><td>lcl|At5g29890.1</td><td>lcl|F56D12.5b CE33404 WBGene00006924 locus:vig-1 status:Confirmed SW:Q8MNX8 protein_id:AAM34394.2 /QuerySize=165</td><td>gi|6802675|ref|Os02g28290|</td></tr>
<tr><td>gi|31240195|ref|XP_320511.1|</td><td>lcl|At5g30341.1</td><td>lcl|F56D2.3 CE11230 WBGene00018965 status:Partially_confirmed SW:Q20873 protein_id:AAB52682.1 /QuerySize=412</td><td>gi|6802677|ref|Os02g28310|</td></tr>
<tr><td>gi|31240203|ref|XP_320515.1|</td><td>lcl|At5g30520.1</td><td>lcl|F56D2.8 CE31006 WBGene00018968 status:Partially_confirmed SW:O01420 protein_id:AAB52684.2 /QuerySize=133</td><td>gi|6802682|ref|Os02g28360|</td></tr>
<tr><td>gi|31240257|ref|XP_320542.1|</td><td>lcl|At5g30942.1</td><td>lcl|F56D3.1 CE29410 WBGene00018969 status:Partially_confirmed SW:Q20877 protein_id:AAA68355.2 /QuerySize=441</td><td>gi|6802683|ref|Os02g28370|</td></tr>
<tr><td>gi|31240259|ref|XP_320543.1|</td><td>lcl|At5g31685.1</td><td>lcl|F56D5.2 CE05968 WBGene00010147 status:Predicted SW:Q20879 protein_id:CAA93498.1 /QuerySize=440</td><td>gi|6802689|ref|Os02g28430|</td></tr>
<tr><td>gi|31240267|ref|XP_320547.1|</td><td>lcl|At5g31752.1</td><td>lcl|F56D5.5 CE05961 WBGene00010149 status:Partially_confirmed SW:Q20885 protein_id:CAA93504.1 /QuerySize=244</td><td>gi|6802691|ref|Os02g28450|</td></tr>
<tr><td>gi|31240275|ref|XP_320551.1|</td><td>lcl|At5g31753.1</td><td>lcl|F56D5.6 CE33797 WBGene00010150 status:Partially_confirmed SW:Q20881 protein_id:CAA93500.3 /QuerySize=449</td><td>gi|6802692|ref|Os02g28460|</td></tr>
<tr><td>gi|31240277|ref|XP_320552.1|</td><td>lcl|At5g31770.1</td><td>lcl|F56D6.6 CE17910 WBGene00006488 locus:srz-62 status:Predicted SW:Q8ITX7 protein_id:AAN73864.1 /QuerySize=266</td><td>gi|6802694|ref|Os02g28480|</td></tr>
<tr><td>gi|31240279|ref|XP_320553.1|</td><td>lcl|At5g31787.1</td><td>lcl|F56E10.1 CE19901 WBGene00018975 status:Partially_confirmed SW:Q9TXN9 protein_id:AAC69220.1 /QuerySize=358</td><td>gi|6802695|ref|Os02g28490|</td></tr>
<tr><td>gi|31240281|ref|XP_320554.1|</td><td>lcl|At5g31838.1</td><td>lcl|F56F11.1 CE11276 WBGene00018988 status:Partially_confirmed SW:Q9TZ62 protein_id:AAK21408.1 /QuerySize=115</td><td>gi|6802696|ref|Os02g28500|</td></tr>
<tr><td>gi|31240291|ref|XP_320559.1|</td><td>lcl|At5g31873.1</td><td>lcl|F56F11.2 CE11278 WBGene00018989 status:Partially_confirmed SW:Q9TZ63 protein_id:AAK21406.1 /QuerySize=401</td><td>gi|6802702|ref|Os02g28560|</td></tr>
<tr><td>gi|31240307|ref|XP_320567.1|</td><td>lcl|At5g32161.1</td><td>lcl|F56F11.5 CE36169 WBGene00018992 status:Partially_confirmed SW:Q9TZ66 protein_id:AAK21410.2 /QuerySize=509</td><td>gi|6802703|ref|Os02g28570|</td></tr>
<tr><td>gi|31240387|ref|XP_320607.1|</td><td>lcl|At5g32169.1</td><td>lcl|F56F3.1 CE17911 WBGene00004132 locus:pqn-45 status:Partially_confirmed SW:Q20898 protein_id:CAA83608.1 /QuerySize=</td><td>gi|6802704|ref|Os02g28580|</td></tr>
<tr><td>gi|31240399|ref|XP_320613.1|</td><td>lcl|At5g32312.1</td><td>lcl|F56F3.3 CE00678 WBGene00010154 status:Predicted SW:Q20897 protein_id:CAA83604.1 /QuerySize=113</td><td>gi|6802705|ref|Os02g28590|</td></tr>
<tr><td>gi|31240437|ref|XP_320632.1|</td><td>lcl|At5g32365.1</td><td>lcl|F56F4.3 CE11264 WBGene00018980 status:Partially_confirmed SW:O01843 protein_id:AAB54200.1 /QuerySize=701</td><td>gi|6802710|ref|Os02g28640|</td></tr>
<tr><td>gi|31240481|ref|XP_320654.1|</td><td>lcl|At5g32400.1</td><td>lcl|F56F4.4 CE11266 WBGene00018981 status:Predicted SW:O01841 protein_id:AAB54201.1 /QuerySize=228</td><td>gi|6802712|ref|Os02g28660|</td></tr>
<tr><td>gi|31240483|ref|XP_320655.1|</td><td>lcl|At5g32410.1</td><td>lcl|F56F4.7 CE11272 WBGene00018983 status:Partially_confirmed SW:O01844 protein_id:AAB54204.1 /QuerySize=118</td><td>gi|6802713|ref|Os02g28670|</td></tr>
<tr><td>gi|31240487|ref|XP_320657.1|</td><td>lcl|At5g32420.1</td><td>lcl|F56G4.2 CE18757 WBGene00003977 locus:pes-2 status:Partially_confirmed SW:Q27347 protein_id:CAB04484.1 /QuerySize=3</td><td>gi|6802714|ref|Os02g28680|</td></tr>
<tr><td>gi|31240489|ref|XP_320658.1|</td><td>lcl|At5g32430.1</td><td>lcl|F56G4.3 CE18757 WBGene00010158 status:Partially_confirmed SW:Q27347 protein_id:CAB04485.1 /QuerySize=331</td><td>gi|6802715|ref|Os02g28690|</td></tr>
<tr><td>gi|31240491|ref|XP_320659.1|</td><td>lcl|At5g32480.1</td><td>lcl|F56G4.6 CE34323 WBGene00010161 status:Partially_confirmed SW:Q9XUY0 protein_id:CAB04488.2 /QuerySize=765</td><td>gi|6802716|ref|Os02g28700|</td></tr>
<tr><td>gi|31240495|ref|XP_320661.1|</td><td>lcl|At5g32490.1</td><td>lcl|F56H11.2 CE16143 WBGene00010175 status:Predicted SW:Q20902 protein_id:CAA92959.1 /QuerySize=152</td><td>gi|6802717|ref|Os02g28710|</td></tr>
<tr><td>gi|31240505|ref|XP_320666.1|</td><td>lcl|At5g32500.1</td><td>lcl|F56H6.3 CE16131 WBGene00010164 status:Predicted SW:O45582 protein_id:CAB04493.1 /QuerySize=136</td><td>gi|6802719|ref|Os02g28730|</td></tr>
<tr><td>gi|31240599|ref|XP_320713.1|</td><td>lcl|At5g32510.1</td><td>lcl|F56H6.6 CE16134 WBGene00010167 status:Partially_confirmed SW:O45584 protein_id:CAB04495.1 /QuerySize=388</td><td>gi|6802721|ref|Os02g28750|</td></tr>
<tr><td>gi|31240611|ref|XP_320719.1|</td><td>lcl|At5g32540.1</td><td>lcl|F56H9.2 CE05972 WBGene00010174 status:Confirmed SW:Q20909 protein_id:CAA98950.1 /QuerySize=57</td><td>gi|6802722|ref|Os02g28760|</td></tr>
<tr><td>gi|31240641|ref|XP_320734.1|</td><td>lcl|At5g32550.1</td><td>lcl|F56H9.5 CE05975 WBGene00003011 locus:lin-25 lin-25 status:Partially_confirmed SW:Q10573 protein_id:CAA98967.1 /Query</td><td>gi|6802723|ref|Os02g28770|</td></tr>
<tr><td>gi|31240643|ref|XP_320735.1|</td><td>lcl|At5g32560.1</td><td>lcl|F57A10.4 CE16150 WBGene00010185 status:Partially_confirmed SW:O17897 protein_id:CAB09421.1 /QuerySize=206</td><td>gi|6802726|ref|Os02g28800|</td></tr>
<tr><td>gi|31240673|ref|XP_320750.1|</td><td>lcl|At5g32570.1</td><td>lcl|F57A8.1 CE34035 WBGene00010177 ETS domain status:Partially_confirmed SW:Q20918 protein_id:CAA98277.2 /QuerySize=</td><td>gi|6802730|ref|Os02g28840|</td></tr>
<tr><td>gi|31240683|ref|XP_320755.1|</td><td>lcl|At5g32590.1</td><td>lcl|F57A8.8 CE34890 WBGene00010183 status:Predicted SW:Q7YX01 protein_id:CAE17848.1 /QuerySize=78</td><td>gi|6802732|ref|Os02g28860|</td></tr>
<tr><td>gi|31240723|ref|XP_320775.1|</td><td>lcl|At5g32600.1</td><td>lcl|F57B1.5 CE11294 WBGene00010188 status:Predicted SW:Q20925 protein_id:CAB01512.1 /QuerySize=157</td><td>gi|6802734|ref|Os02g28880|</td></tr>
<tr><td>gi|31240745|ref|XP_320786.1|</td><td>lcl|At5g32605.1</td><td>lcl|F57B1.6 CE11296 WBGene00010189 status:Partially_confirmed SW:Q20926 protein_id:CAB01513.1 /QuerySize=245</td><td>gi|6802735|ref|Os02g28890|</td></tr>
<tr><td>gi|31240769|ref|XP_320798.1|</td><td>lcl|At5g32610.1</td><td>lcl|F57B1.7 CE11298 WBGene00010190 status:Partially_confirmed SW:Q20920 protein_id:CAB01507.1 /QuerySize=235</td><td>gi|6802737|ref|Os02g28910|</td></tr>
<tr><td>gi|31240791|ref|XP_320809.1|</td><td>lcl|At5g32613.1</td><td>lcl|F57B10.12 CE11320 WBGene00003184 locus:mei-2 status:Confirmed SW:O44740 protein_id:AAB96729.1 /QuerySize=280</td><td>gi|6802738|ref|Os02g28920|</td></tr>
<tr><td>gi|31240795|ref|XP_320811.1|</td><td>lcl|At5g32775.1</td><td>lcl|F57B10.14 CE31352 WBGene00019007 status:Confirmed SW:Q8MXI1 protein_id:AAM81096.1 /QuerySize=56</td><td>gi|6802739|ref|Os02g28930|</td></tr>
<tr><td>gi|31240843|ref|XP_320835.1|</td><td>lcl|At5g33230.1</td><td>lcl|F57B10.4 CE11304 WBGene00019002 status:Partially_confirmed SW:O44741 protein_id:AAB96721.1 /QuerySize=691</td><td>gi|6802745|ref|Os02g28990|</td></tr>
<tr><td>gi|31240871|ref|XP_320849.1|</td><td>lcl|At5g33355.1</td><td>lcl|F57B9.9 CE33114 WBGene00004133 locus:pqn-46 status:Partially_confirmed SW:Q20936 protein_id:AAA21172.3 /QuerySize=</td><td>gi|6802756|ref|Os02g29090|</td></tr>
<tr><td>gi|31240875|ref|XP_320851.1|</td><td>lcl|At5g33380.1</td><td>lcl|F57C2.3 CE16154 WBGene00010195 status:Partially_confirmed SW:O45595 protein_id:CAB05525.1 /QuerySize=251</td><td>gi|6802759|ref|Os02g29120|</td></tr>
<tr><td>gi|31240879|ref|XP_320853.1|</td><td>lcl|At5g33390.1</td><td>lcl|F57C2.4 CE16155 WBGene00010196 status:Partially_confirmed SW:O45596 protein_id:CAB05526.1 /QuerySize=88</td><td>gi|6802764|ref|Os02g29170|</td></tr>
<tr><td>gi|31240885|ref|XP_320856.1|</td><td>lcl|At5g33393.1</td><td>lcl|F57C2.6 CE35560 WBGene00010198 status:Partially_confirmed SW:O45243 protein_id:CAB05528.3 /QuerySize=545</td><td>gi|6802765|ref|Os02g29180|</td></tr>
<tr><td>gi|31240895|ref|XP_320861.1|</td><td>lcl|At5g33402.1</td><td>lcl|F57C7.5 CE36492 WBGene00010201 status:Partially_confirmed SW:Q20946 protein_id:CAA93472.2 /QuerySize=500</td><td>gi|6802767|ref|Os02g29200|</td></tr>
<tr><td>gi|31240905|ref|XP_320866.1|</td><td>lcl|At5g33431.1</td><td>lcl|F57C9.3 CE32682 WBGene00019010 status:Predicted SW:O01822 protein_id:AAK73886.2 /QuerySize=128</td><td>gi|6802771|ref|Os02g29240|</td></tr>
<tr><td>gi|31240923|ref|XP_320875.1|</td><td>lcl|At5g33442.1</td><td>lcl|F57C9.4a CE28474 WBGene00019011 status:Partially_confirmed SW:Q95R05 protein_id:AAK73887.1 /QuerySize=653</td><td>gi|6802772|ref|Os02g29250|</td></tr>
<tr><td>gi|31240943|ref|XP_320885.1|</td><td>lcl|At5g33715.1</td><td>lcl|F57C9.7 CE11334 WBGene00019013 status:Partially_confirmed SW:O01819 protein_id:AAB54190.1 /QuerySize=412</td><td>gi|6802774|ref|Os02g29270|</td></tr>
<tr><td>gi|31240961|ref|XP_320894.1|</td><td>lcl|At5g33806.1</td><td>lcl|F57E7.1 CE18763 WBGene00010202 status:Predicted SW:Q9XXT3 protein_id:CAA16166.1 /QuerySize=113</td><td>gi|6802775|ref|Os02g29280|</td></tr>
<tr><td>gi|31240971|ref|XP_320899.1|</td><td>lcl|At5g33898.1</td><td>lcl|F57E7.2 CE18764 WBGene00010203 status:Predicted SW:Q9XXT4 protein_id:CAA16165.1 /QuerySize=240</td><td>gi|6802776|ref|Os02g29290|</td></tr>
<tr><td>gi|31240975|ref|XP_320901.1|</td><td>lcl|At5g34358.1</td><td>lcl|F57F4.2 CE11340 WBGene00019016 status:Predicted SW:Q94244 protein_id:AAB09165.1 /QuerySize=327</td><td>gi|6802777|ref|Os02g29300|</td></tr>
<tr><td>gi|31240977|ref|XP_320902.1|</td><td>lcl|At5g34450.1</td><td>lcl|F57F5.3 CE06001 WBGene00010205 status:Predicted SW:Q20951 protein_id:CAB00099.1 /QuerySize=257</td><td>gi|6802778|ref|Os02g29300|</td></tr>
<tr><td>gi|31240997|ref|XP_320912.1|</td><td>lcl|At5g34581.1</td><td>lcl|F57G12.1 CE03426 WBGene00010221 status:Confirmed SW:Q20955 protein_id:CAA91534.1 /QuerySize=237</td><td>gi|6802779|ref|Os02g29310|</td></tr>
<tr><td>gi|31240999|ref|XP_320913.1|</td><td>lcl|At5g34820.1</td><td>lcl|F57G12.2 CE03427 WBGene00010222 status:Partially_confirmed SW:Q20956 protein_id:CAA91535.1 /QuerySize=226</td><td>gi|6802783|ref|Os02g29350|</td></tr>
<tr><td>gi|31241013|ref|XP_320920.1|</td><td>lcl|At5g34830.1</td><td>lcl|F57G4.2 CE18767 WBGene00010207 status:Predicted SW:Q9XUX5 protein_id:CAB04507.1 /QuerySize=423</td><td>gi|6802784|ref|Os02g29360|</td></tr>
<tr><td>gi|31241015|ref|XP_320921.1|</td><td>lcl|At5g34839.1</td><td>lcl|F57G4.3 CE18768 WBGene00010208 status:Predicted SW:Q9XUX4 protein_id:CAB04508.1 /QuerySize=304</td><td>gi|6802787|ref|Os02g29390|</td></tr>
<tr><td>gi|31241025|ref|XP_320926.1|</td><td>lcl|At5g34855.1</td><td>lcl|F57G4.5 CE18770 WBGene00010210 status:Predicted SW:Q9XUX8 protein_id:CAB04504.1 /QuerySize=259</td><td>gi|6802790|ref|Os02g29420|</td></tr>
<tr><td>gi|31241039|ref|XP_320935.1|</td><td>lcl|At5g34870.1</td><td>lcl|F57G8.7 CE18778 WBGene00010216 status:Predicted SW:Q9XU92 protein_id:CAB05531.1 /QuerySize=137</td><td>gi|6802792|ref|Os02g29440|</td></tr>
<tr><td>gi|31241069|ref|XP_320956.1|</td><td>lcl|At5g34875.1</td><td>lcl|F57G9.3 CE17919 WBGene00010219 status:Partially_confirmed SW:O62268 protein_id:CAB05751.1 /QuerySize=247</td><td>gi|6802793|ref|Os02g29450|</td></tr>
<tr><td>gi|31241075|ref|XP_320961.1|</td><td>lcl|At5g34881.1</td><td>lcl|F58A3.3 CE11356 WBGene00010223 status:Partially_confirmed SW:Q93802 protein_id:CAB02668.1 /QuerySize=181</td><td>gi|6802797|ref|Os02g29490|</td></tr>
<tr><td>gi|31241081|ref|XP_320964.1|</td><td>lcl|At5g34883.1</td><td>lcl|F58A4.1 CE20889 WBGene00010226 status:Partially_confirmed SW:P34468 protein_id:CAA80169.1 /QuerySize=254</td><td>gi|6802798|ref|Os02g29500|</td></tr>
<tr><td>gi|31241099|ref|XP_320973.1|</td><td>lcl|At5g34885.1</td><td>lcl|F58A4.11 CE00217 WBGene00001570 locus:gei-13 status:Partially_confirmed SW:P34478 protein_id:CAA80171.1 /QuerySize=</td><td>gi|6802800|ref|Os02g29520|</td></tr>
<tr><td>gi|31241105|ref|XP_320976.1|</td><td>lcl|At5g34887.1</td><td>lcl|F58A4.2 CE01017 WBGene00010227 status:Partially_confirmed SW:P34469 protein_id:CAA80168.1 /QuerySize=159</td><td>gi|6802802|ref|Os02g29540|</td></tr>
<tr><td>gi|31241107|ref|XP_320977.1|</td><td>lcl|At5g34905.1</td><td>lcl|F58A4.7d CE34038 WBGene00001955 locus:hlh-11 status:Confirmed SW:Q7YXC9 protein_id:CAD91636.1 /QuerySize=136</td><td>gi|6802803|ref|Os02g29560|</td></tr>
<tr><td>gi|31241135|ref|XP_320992.1|</td><td>lcl|At5g34910.1</td><td>lcl|F58A6.2 CE04676 WBGene00019023 status:Predicted SW:Q20957 protein_id:AAA96197.1 /QuerySize=159</td><td>gi|6802806|ref|Os02g29580|</td></tr>
<tr><td>gi|31241137|ref|XP_320994.1|</td><td>lcl|At5g34920.1</td><td>lcl|F58B4.4 CE06014 WBGene00010237 status:Predicted SW:Q20972 protein_id:CAA98494.1 /QuerySize=137</td><td>gi|6802807|ref|Os02g29590|</td></tr>
<tr><td>gi|31241143|ref|XP_320997.1|</td><td>lcl|At5g34970.1</td><td>lcl|F58D12.1 CE11368 WBGene00010249 status:Predicted SW:O02276 protein_id:CAB03144.1 /QuerySize=143</td><td>gi|6802813|ref|Os02g29650|</td></tr>
<tr><td>gi|31241153|ref|XP_321002.1|</td><td>lcl|At5g34980.1</td><td>lcl|F58D2.2 CE11364 WBGene00010241 status:Partially_confirmed SW:Q9XVM2 protein_id:CAB03147.1 /QuerySize=610</td><td>gi|6802819|ref|Os02g29710|</td></tr>
<tr><td>gi|31241157|ref|XP_321005.1|</td><td>lcl|At5g35010.1</td><td>lcl|F58D5.2a CE34487 WBGene00010242 status:Partially_confirmed SW:Q9NLD2 protein_id:CAB70237.2 /QuerySize=188</td><td>gi|6802823|ref|Os02g29750|</td></tr>
<tr><td>gi|31241159|ref|XP_321006.1|</td><td>lcl|At5g35020.1</td><td>lcl|F58D5.2b CE34488 WBGene00010242 status:Confirmed SW:Q7YTT0 protein_id:CAE11315.1 /QuerySize=62</td><td>gi|6802827|ref|Os02g29790|</td></tr>
<tr><td>gi|31241161|ref|XP_321008.1|</td><td>lcl|At5g35035.1</td><td>lcl|F58D5.5 CE30547 WBGene00010244 status:Partially_confirmed SW:Q9NLD3 protein_id:CAB70236.2 /QuerySize=159</td><td>gi|6802828|ref|Os02g29800|</td></tr>
<tr><td>gi|31241167|ref|XP_321012.1|</td><td>lcl|At5g35050.1</td><td>lcl|F58D5.7 CE25024 WBGene00010246 status:Confirmed SW:Q9NLC7 protein_id:CAB70242.1 /QuerySize=258</td><td>gi|6802832|ref|Os02g29840|</td></tr>
<tr><td>gi|31241175|ref|XP_321017.1|</td><td>lcl|At5g35054.1</td><td>lcl|F58D5.8 CE25920 WBGene00010247 status:Partially_confirmed SW:Q9NLC8 protein_id:CAC15866.1 /QuerySize=328</td><td>gi|6802833|ref|Os02g29850|</td></tr>
<tr><td>gi|31241185|ref|XP_321023.1|</td><td>lcl|At5g35060.1</td><td>lcl|F58D5.9 CE34893 WBGene00010248 status:Predicted SW:Q7YTM2 protein_id:CAE17839.1 /QuerySize=220</td><td>gi|6802834|ref|Os02g29860|</td></tr>
<tr><td>gi|31241189|ref|XP_321025.1|</td><td>lcl|At5g35070.1</td><td>lcl|F58E1.11 CE36171 WBGene00019042 status:Partially_confirmed SW:Q9TZG1 protein_id:AAC67488.2 /QuerySize=415</td><td>gi|6802835|ref|Os02g29870|</td></tr>
<tr><td>gi|31241193|ref|XP_321027.1|</td><td>lcl|At5g35090.1</td><td>lcl|F58E1.13 CE36172 WBGene00019044 status:Partially_confirmed SW:Q9TZF9 protein_id:AAC67476.2 /QuerySize=95</td><td>gi|6802838|ref|Os02g29900|</td></tr>
<tr><td>gi|31241203|ref|XP_321032.1|</td><td>lcl|At5g35110.1</td><td>lcl|F58E10.1 CE30677 WBGene00010259 status:Confirmed SW:Q9XUW6 protein_id:CAB04517.2 /QuerySize=694</td><td>gi|6802839|ref|Os02g29910|</td></tr>
<tr><td>gi|31241229|ref|XP_321045.1|</td><td>lcl|At5g35120.1</td><td>lcl|F58E10.7 CE18789 WBGene00010262 status:Confirmed SW:Q9XUW9 protein_id:CAB04514.1 /QuerySize=158</td><td>gi|6802842|ref|Os02g29940|</td></tr>
</table>

<table>
<tr><td>gi|31241253|ref|XP_321057.1|</td><td>lcl|At5g35140.1</td><td>lcl|F58E2.2 CE17130 WBGene00019046 status:Predicted SW:Q9TYZ6 protein_id:AAC68965.1 /QuerySize=141</td><td>gi|6802845|ref|Os02g29970|</td></tr>
<tr><td>gi|31241255|ref|XP_321058.1|</td><td>lcl|At5g35190.1</td><td>lcl|F58E6.11 CE34894 WBGene00010258 status:Confirmed SW:Q7YX04 protein_id:CAE17845.1 /QuerySize=188</td><td>gi|6802847|ref|Os02g29990|</td></tr>
<tr><td>gi|31241271|ref|XP_321066.1|</td><td>lcl|At5g35230.1</td><td>lcl|F58E6.6 CE06021 WBGene00010255 status:Partially_confirmed SW:Q20982 protein_id:CAA94776.1 /QuerySize=242</td><td>gi|6802849|ref|Os02g30010|</td></tr>
<tr><td>gi|31241277|ref|XP_321069.1|</td><td>lcl|At5g35270.1</td><td>lcl|F58E6.7 CE06022 WBGene00010256 status:Confirmed SW:Q20983 protein_id:CAA94777.1 /QuerySize=87</td><td>gi|6802855|ref|Os02g30070|</td></tr>
<tr><td>gi|31241279|ref|XP_321070.1|</td><td>lcl|At5g35290.1</td><td>lcl|F58E6.8 CE06023 WBGene00010257 status:Partially_confirmed SW:Q20984 protein_id:CAA94778.1 /QuerySize=228</td><td>gi|6802865|ref|Os02g30170|</td></tr>
<tr><td>gi|31241305|ref|XP_321083.1|</td><td>lcl|At5g35300.1</td><td>lcl|F58F12.2 CE07305 WBGene00019062 status:Predicted SW:Q09399 protein_id:AAC46700.2 /QuerySize=125</td><td>gi|6802867|ref|Os02g30190|</td></tr>
<tr><td>gi|31241311|ref|XP_321086.1|</td><td>lcl|At5g35310.1</td><td>lcl|F58F12.3 CE01978 WBGene00019063 status:Partially_confirmed SW:Q09400 protein_id:AAC46701.1 /QuerySize=463</td><td>gi|6802868|ref|Os02g30200|</td></tr>
<tr><td>gi|31241337|ref|XP_321099.1|</td><td>lcl|At5g35340.1</td><td>lcl|F58F6.3 CE17138 WBGene00019052 status:Predicted SW:O44172 protein_id:AAB88357.1 /QuerySize=170</td><td>gi|6802874|ref|Os02g30250|</td></tr>
<tr><td>gi|31241349|ref|XP_321105.1|</td><td>lcl|At5g35350.1</td><td>lcl|F58F6.5 CE34324 WBGene00019053 status:Partially_confirmed SW:O44176 protein_id:AAB88361.2 /QuerySize=109</td><td>gi|6802875|ref|Os02g30260|</td></tr>
<tr><td>gi|31241365|ref|XP_321113.1|</td><td>lcl|At5g35420.1</td><td>lcl|F58F9.1 CE07298 WBGene00019055 status:Partially_confirmed SW:Q20993 protein_id:AAC48087.1 /QuerySize=310</td><td>gi|6802876|ref|Os02g30270|</td></tr>
<tr><td>gi|31241373|ref|XP_321117.1|</td><td>lcl|At5g35425.1</td><td>lcl|F58F9.3a CE30155 WBGene00019057 status:Partially_confirmed SW:Q20989 protein_id:AAC48084.2 /QuerySize=181</td><td>gi|6802877|ref|Os02g30280|</td></tr>
<tr><td>gi|31241389|ref|XP_321125.1|</td><td>lcl|At5g35470.1</td><td>lcl|F58G1.4 CE17924 WBGene00010266 status:Partially_confirmed SW:O62277 protein_id:CAB04526.1 /QuerySize=223</td><td>gi|6802882|ref|Os02g30330|</td></tr>
<tr><td>gi|31241395|ref|XP_321128.1|</td><td>lcl|At5g35480.1</td><td>lcl|F58G1.7 CE17927 WBGene00010269 status:Partially_confirmed SW:O62270 protein_id:CAB04519.1 /QuerySize=569</td><td>gi|6802887|ref|Os02g30380|</td></tr>
<tr><td>gi|31241485|ref|XP_321173.1|</td><td>lcl|At5g35490.1</td><td>lcl|F58G1.9 CE17929 WBGene00010271 status:Partially_confirmed SW:O62273 protein_id:CAB04522.1 /QuerySize=87</td><td>gi|6802892|ref|Os02g30430|</td></tr>
<tr><td>gi|31241507|ref|XP_321184.1|</td><td>lcl|At5g35500.1</td><td>lcl|F58G11.3 CE11404 WBGene00010281 status:Partially_confirmed SW:P90898 protein_id:CAB03154.1 /QuerySize=666</td><td>gi|6802893|ref|Os02g30440|</td></tr>
<tr><td>gi|31241527|ref|XP_321194.1|</td><td>lcl|At5g35510.1</td><td>lcl|F58G6.6 CE25923 WBGene00000953 locus:del-2 status:Partially_confirmed SW:Q21007 protein_id:CAA92468.2 /QuerySize=5</td><td>gi|6802898|ref|Os02g30490|</td></tr>
<tr><td>gi|31241529|ref|XP_321195.1|</td><td>lcl|At5g35540.1</td><td>lcl|F58H1.2 CE06026 WBGene00010285 status:Partially_confirmed SW:Q21011 protein_id:CAB00105.1 /QuerySize=190</td><td>gi|6802899|ref|Os02g30500|</td></tr>
<tr><td>gi|31241533|ref|XP_321197.1|</td><td>lcl|At5g35603.1</td><td>lcl|F58H1.4 CE06028 WBGene00010287 status:Partially_confirmed SW:Q21013 protein_id:CAB00107.1 /QuerySize=104</td><td>gi|6802905|ref|Os02g30560|</td></tr>
<tr><td>gi|31241535|ref|XP_321198.1|</td><td>lcl|At5g35604.1</td><td>lcl|F58H1.5 CE06029 WBGene00010288 status:Confirmed SW:Q21014 protein_id:CAB00108.1 /QuerySize=323</td><td>gi|6802906|ref|Os02g30570|</td></tr>
<tr><td>gi|31241543|ref|XP_321202.1|</td><td>lcl|At5g35660.1</td><td>lcl|F58H10.1 CE36173 WBGene00010291 status:Partially_confirmed SW:Q93827 protein_id:CAB01975.2 /QuerySize=119</td><td>gi|6802908|ref|Os02g30590|</td></tr>
<tr><td>gi|31241551|ref|XP_321206.1|</td><td>lcl|At5g35760.1</td><td>lcl|F58H7.1 CE17930 WBGene00019067 status:Partially_confirmed SW:O45090 protein_id:AAC02592.1 /QuerySize=607</td><td>gi|6802909|ref|Os02g30600|</td></tr>
<tr><td>gi|31241559|ref|XP_321210.1|</td><td>lcl|At5g35775.1</td><td>lcl|F58H7.5 CE17933 WBGene00019070 status:Partially_confirmed SW:O45091 protein_id:AAC02591.1 /QuerySize=272</td><td>gi|6802914|ref|Os02g30640|</td></tr>
<tr><td>gi|31241567|ref|XP_321214.1|</td><td>lcl|At5g35792.1</td><td>lcl|F58H7.7 CE34326 WBGene00019072 status:Partially_confirmed SW:O45093 protein_id:AAC02596.2 /QuerySize=663</td><td>gi|6802917|ref|Os02g30660|</td></tr>
<tr><td>gi|31241569|ref|XP_321215.1|</td><td>lcl|At5g35800.1</td><td>lcl|F58H7.8 CE17936 WBGene00019073 status:Predicted SW:O45094 protein_id:AAC02595.1 /QuerySize=338</td><td>gi|6802928|ref|Os02g30760|</td></tr>
<tr><td>gi|31241571|ref|XP_321216.1|</td><td>lcl|At5g35850.1</td><td>lcl|F59A1.11 CE34039 WBGene00010297 status:Partially_confirmed SW:Q9XUW3 protein_id:CAB04530.2 /QuerySize=303</td><td>gi|6802930|ref|Os02g30780|</td></tr>
<tr><td>gi|31241573|ref|XP_321217.1|</td><td>lcl|At5g35880.1</td><td>lcl|F59A1.12 CE18796 WBGene00010298 status:Predicted SW:Q9XUW4 protein_id:CAB04529.1 /QuerySize=357</td><td>gi|6802931|ref|Os02g30790|</td></tr>
<tr><td>gi|31241575|ref|XP_321218.1|</td><td>lcl|At5g35890.1</td><td>lcl|F59A1.15 CE34897 WBGene00010300 status:Predicted SW:Q7YWZ9 protein_id:CAE17850.1 /QuerySize=554</td><td>gi|6802934|ref|Os02g30820|</td></tr>
<tr><td>gi|31241577|ref|XP_321219.1|</td><td>lcl|At5g35945.1</td><td>lcl|F59A1.6 CE18790 WBGene00010292 status:Predicted SW:Q9XUV9 protein_id:CAB04534.1 /QuerySize=99</td><td>gi|6802935|ref|Os02g30830|</td></tr>
<tr><td>gi|31241579|ref|XP_321220.1|</td><td>lcl|At5g35990.1</td><td>lcl|F59A2.2 CE01020 WBGene00010302 Coiled-coil protein status:Partially_confirmed SW:Q21020 protein_id:CAA84327.1 /Query</td><td>gi|6802938|ref|Os02g30860|</td></tr>
<tr><td>gi|31241583|ref|XP_321222.1|</td><td>lcl|At5g36020.1</td><td>lcl|F59A2.5 CE01023 WBGene00010305 status:Partially_confirmed SW:Q21019 protein_id:CAA84326.1 /QuerySize=123</td><td>gi|6802939|ref|Os02g30870|</td></tr>
<tr><td>gi|31241585|ref|XP_321223.1|</td><td>lcl|At5g36035.1</td><td>lcl|F59A3.10 CE11434 WBGene00019083 status:Predicted SW:P91362 protein_id:AAB37824.1 /QuerySize=117</td><td>gi|6802941|ref|Os02g30890|</td></tr>
<tr><td>gi|31241587|ref|XP_321224.1|</td><td>lcl|At5g36040.1</td><td>lcl|F59A3.2a CE11418 WBGene00019075 status:Partially_confirmed SW:P91357 protein_id:AAB37819.1 /QuerySize=975</td><td>gi|6802944|ref|Os02g30920|</td></tr>
<tr><td>gi|31241593|ref|XP_321227.1|</td><td>lcl|At5g36060.1</td><td>lcl|F59A3.2b CE33116 WBGene00019075 status:Partially_confirmed SW:Q8I7G1 protein_id:AAO12417.1 /QuerySize=687</td><td>gi|6802945|ref|Os02g30930|</td></tr>
<tr><td>gi|31241603|ref|XP_321232.1|</td><td>lcl|At5g36070.1</td><td>lcl|F59A3.7 CE11428 WBGene00019080 status:Predicted SW:P91358 protein_id:AAB37820.1 /QuerySize=260</td><td>gi|6802946|ref|Os02g30940|</td></tr>
<tr><td>gi|31241605|ref|XP_321233.1|</td><td>lcl|At5g36080.1</td><td>lcl|F59A6.2 CE01980 WBGene00019084 status:Partially_confirmed SW:Q21028 protein_id:AAK31522.1 /QuerySize=118</td><td>gi|6802947|ref|Os02g30950|</td></tr>
<tr><td>gi|31241607|ref|XP_321234.1|</td><td>lcl|At5g36100.1</td><td>lcl|F59A6.3 CE01981 WBGene00019085 Glycoprotein status:Partially_confirmed SW:Q21027 protein_id:AAK31523.1 /QuerySize=</td><td>gi|6802949|ref|Os02g30970|</td></tr>
<tr><td>gi|31241615|ref|XP_321238.1|</td><td>lcl|At5g36190.1</td><td>lcl|F59A7.2 CE11438 WBGene00019090 status:Confirmed SW:O16281 protein_id:AAB65335.1 /QuerySize=116</td><td>gi|6802953|ref|Os02g31010|</td></tr>
<tr><td>gi|31241683|ref|XP_321272.1|</td><td>lcl|At5g36280.1</td><td>lcl|F59A7.3 CE11440 WBGene00019091 locus:srab-13 status:Predicted SW:O16278 protein_id:AAB65334.1 /QuerySize=298</td><td>gi|6802958|ref|Os02g31060|</td></tr>
<tr><td>gi|31241703|ref|XP_321282.1|</td><td>lcl|At5g36650.1</td><td>lcl|F59A7.5a CE30810 WBGene00019092 status:Partially_confirmed SW:O16279 protein_id:AAD34667.2 /QuerySize=239</td><td>gi|6802962|ref|Os02g31090|</td></tr>
<tr><td>gi|31241711|ref|XP_321286.1|</td><td>lcl|At5g36660.1</td><td>lcl|F59A7.5b CE35327 WBGene00019092 status:Confirmed SW:Q7KQA4 protein_id:AAQ62449.1 /QuerySize=116</td><td>gi|6802964|ref|Os02g31110|</td></tr>
<tr><td>gi|31241719|ref|XP_321290.1|</td><td>lcl|At5g36720.1</td><td>lcl|F59B1.2 CE17943 WBGene00019097 status:Confirmed SW:O61880 protein_id:AAC17663.1 /QuerySize=161</td><td>gi|6802976|ref|Os02g31230|</td></tr>
<tr><td>gi|31241721|ref|XP_321291.1|</td><td>lcl|At5g36735.1</td><td>lcl|F59B1.6 CE17947 WBGene00019099 status:Predicted SW:O61879 protein_id:AAC17661.1 /QuerySize=359</td><td>gi|6802977|ref|Os02g31230|</td></tr>
<tr><td>gi|31241723|ref|XP_321292.1|</td><td>lcl|At5g36737.1</td><td>lcl|F59B10.2 CE01591 WBGene00010318 status:Predicted SW:Q09950 protein_id:CAA88601.1 /QuerySize=482</td><td>gi|6802978|ref|Os02g31240|</td></tr>
<tr><td>gi|31241731|ref|XP_321296.1|</td><td>lcl|At5g36810.1</td><td>lcl|F59B10.3 CE35884 WBGene00010319 cadherin transmembranous domain status:Confirmed SW:Q09951 protein_id:CAA8859</td><td>gi|6802979|ref|Os02g31250|</td></tr>
<tr><td>gi|31241733|ref|XP_321297.1|</td><td>lcl|At5g36900.1</td><td>lcl|F59B10.4a CE35885 WBGene00010320 status:Confirmed SW:Q09952 protein_id:CAA88598.2 /QuerySize=264</td><td>gi|6802982|ref|Os02g31280|</td></tr>
<tr><td>gi|31241735|ref|XP_321298.1|</td><td>lcl|At5g36920.1</td><td>lcl|F59B10.4b CE36941 WBGene00010320 status:Confirmed SW:Q6BEV1 protein_id:CAH04739.1 /QuerySize=238</td><td>gi|6802988|ref|Os02g31330|</td></tr>
<tr><td>gi|31241737|ref|XP_321299.1|</td><td>lcl|At5g36925.1</td><td>lcl|F59B10.5 CE01594 WBGene00010321 status:Confirmed SW:Q09953 protein_id:CAA88599.1 /QuerySize=96</td><td>gi|6802989|ref|Os02g31830|</td></tr>
<tr><td>gi|31241749|ref|XP_321305.1|</td><td>lcl|At5g36960.1</td><td>lcl|F59B10.6 CE01595 WBGene00010322 status:Partially_confirmed SW:Q09954 protein_id:CAA88600.1 /QuerySize=105</td><td>gi|6802990|ref|Os02g31840|</td></tr>
<tr><td>gi|31241755|ref|XP_321308.1|</td><td>lcl|At5g36980.1</td><td>lcl|F59B2.11 CE37775 WBGene00010313 status:Partially_confirmed SW:P34486 protein_id:CAA77588.2 /QuerySize=590</td><td>gi|6802992|ref|Os02g31860|</td></tr>
<tr><td>gi|31241789|ref|XP_321325.1|</td><td>lcl|At5g37010.1</td><td>lcl|F59B2.6 CE34193 WBGene00006977 locus:zif-1 status:Confirmed SW:P34482 protein_id:CAA77583.2 /QuerySize=489</td><td>gi|6802998|ref|Os02g31920|</td></tr>
<tr><td>gi|31241805|ref|XP_321333.1|</td><td>lcl|At5g37050.1</td><td>lcl|F59C6.11 CE27178 WBGene00010328 status:Confirmed SW:Q95NH9 protein_id:CAC42309.1 /QuerySize=173</td><td>gi|6802999|ref|Os02g31930|</td></tr>
<tr><td>gi|31241807|ref|XP_321334.1|</td><td>lcl|At5g37090.1</td><td>lcl|F59C6.3 CE11460 WBGene00010324 status:Predicted SW:Q93829 protein_id:CAB01874.1 /QuerySize=350</td><td>gi|6803001|ref|Os02g31950|</td></tr>
<tr><td>gi|31241823|ref|XP_321342.1|</td><td>lcl|At5g37120.1</td><td>lcl|F59C6.6 CE11466 WBGene00003742 locus:nlp-4 status:Partially_confirmed SW:Q93832 protein_id:CAB01877.1 /QuerySize=2</td><td>gi|6803006|ref|Os02g32000|</td></tr>
<tr><td>gi|31241859|ref|XP_321360.1|</td><td>lcl|At5g37190.1</td><td>lcl|F59D12.2 CE35566 WBGene00010330 status:Partially_confirmed SW:O62281 protein_id:CAB04540.2 /QuerySize=144</td><td>gi|6803008|ref|Os02g32020|</td></tr>
<tr><td>gi|31241875|ref|XP_321368.1|</td><td>lcl|At5g37220.1</td><td>lcl|F59D12.5 CE19909 WBGene00010332 status:Partially_confirmed SW:Q9XTE3 protein_id:CAA19427.1 /QuerySize=157</td><td>gi|6803014|ref|Os02g32070|</td></tr>
<tr><td>gi|31241891|ref|XP_321376.1|</td><td>lcl|At5g37240.1</td><td>lcl|F59D6.1 CE11476 WBGene00019103 status:Partially_confirmed SW:O16344 protein_id:AAB65876.1 /QuerySize=118</td><td>gi|6803015|ref|Os02g32080|</td></tr>
<tr><td>gi|31241899|ref|XP_321380.1|</td><td>lcl|At5g37330.1</td><td>lcl|F59E11.12a CE33120 WBGene00019117 status:Confirmed SW:O16754 protein_id:AAG24146.2 /QuerySize=64</td><td>gi|6803017|ref|Os02g32100|</td></tr>
<tr><td>gi|31241903|ref|XP_321382.1|</td><td>lcl|At5g37385.1</td><td>lcl|F59E11.12b CE35888 WBGene00019117 status:Confirmed SW:Q71VC4 protein_id:AAQ91902.1 /QuerySize=69</td><td>gi|6803020|ref|Os02g32130|</td></tr>
<tr><td>gi|31241907|ref|XP_321384.1|</td><td>lcl|At5g37550.1</td><td>lcl|F59E11.5 CE37776 WBGene00019111 status:Confirmed SW:O16761 protein_id:AAG24141.2 /QuerySize=212</td><td>gi|6803022|ref|Os02g32150|</td></tr>
<tr><td>gi|31241909|ref|XP_321385.1|</td><td>lcl|At5g37580.1</td><td>lcl|F59E11.6 CE11506 WBGene00019112 status:Partially_confirmed SW:O16760 protein_id:AAG24142.1 /QuerySize=222</td><td>gi|6803025|ref|Os02g32180|</td></tr>
<tr><td>gi|31241911|ref|XP_321386.1|</td><td>lcl|At5g37880.1</td><td>lcl|F59E11.7a CE28568 WBGene00019113 status:Confirmed SW:O16759 protein_id:AAG24143.2 /QuerySize=195</td><td>gi|6803026|ref|Os02g32190|</td></tr>
<tr><td>gi|31241933|ref|XP_321397.1|</td><td>lcl|At5g38035.1</td><td>lcl|F59E11.7b CE11508 WBGene00019113 status:Confirmed SW:Q8IA81 protein_id:AAO12416.1 /QuerySize=172</td><td>gi|6803028|ref|Os02g32210|</td></tr>
<tr><td>gi|31241947|ref|XP_321404.1|</td><td>lcl|At5g38080.1</td><td>lcl|F59E12.10 CE11536 WBGene00019125 status:Partially_confirmed SW:O01901 protein_id:AAB54251.1 /QuerySize=189</td><td>gi|6803031|ref|Os02g32240|</td></tr>
<tr><td>gi|31241973|ref|XP_321417.1|</td><td>lcl|At5g38090.1</td><td>lcl|F59F4.3 CE31551 WBGene00010338 status:Partially_confirmed SW:Q93840 protein_id:CAB03159.2 /QuerySize=494</td><td>gi|6803032|ref|Os02g32250|</td></tr>
<tr><td>gi|31242001|ref|XP_321431.1|</td><td>lcl|At5g38190.1</td><td>lcl|F59F5.2 CE26715 WBGene00010341 status:Confirmed SW:Q21045 protein_id:CAA90656.2 /QuerySize=179</td><td>gi|6803033|ref|Os02g32260|</td></tr>
<tr><td>gi|31242009|ref|XP_321435.1|</td><td>lcl|At5g38300.1</td><td>lcl|F59F5.4 CE03445 WBGene00010343 collagen status:Partially_confirmed SW:Q21047 protein_id:CAA90658.1 /QuerySize=93</td><td>gi|6803034|ref|Os02g32270|</td></tr>
<tr><td>gi|31242015|ref|XP_321438.1|</td><td>lcl|At5g38310.1</td><td>lcl|F59F5.5 CE03446 WBGene00010344 status:Predicted SW:Q21048 protein_id:CAA90659.1 /QuerySize=151</td><td>gi|6803035|ref|Os02g32280|</td></tr>
<tr><td>gi|31242085|ref|XP_321473.1|</td><td>lcl|At5g38320.1</td><td>lcl|F59F5.8 CE26716 WBGene00010346 status:Partially_confirmed SW:Q9BI85 protein_id:CAC35819.1 /QuerySize=396</td><td>gi|6803036|ref|Os02g32280|</td></tr>
<tr><td>gi|31242119|ref|XP_321490.1|</td><td>lcl|At5g38330.1</td><td>lcl|F59G1.8 CE26373 WBGene00019129 status:Predicted SW:Q9BI84 protein_id:AAK31534.1 /QuerySize=192</td><td>gi|6803037|ref|Os02g32290|</td></tr>
<tr><td>gi|31242125|ref|XP_321493.1|</td><td>lcl|At5g38370.1</td><td>lcl|F59H6.3 CE32683 WBGene00019134 status:Predicted SW:Q9N5Q4 protein_id:AAF39852.2 /QuerySize=71</td><td>gi|6803039|ref|Os02g32310|</td></tr>
<tr><td>gi|31242127|ref|XP_321494.1|</td><td>lcl|At5g38400.1</td><td>lcl|F59H6.6 CE20912 WBGene00019137 status:Predicted SW:Q9N5Q5 protein_id:AAF39855.1 /QuerySize=216</td><td>gi|6803040|ref|Os02g32320|</td></tr>
<tr><td>gi|31242147|ref|XP_321504.1|</td><td>lcl|At5g38595.1</td><td>lcl|H01G02.1 CE11554 WBGene00010348 status:Predicted SW:O17902 protein_id:CAB07421.1 /QuerySize=309</td><td>gi|6803041|ref|Os02g32330|</td></tr>
<tr><td>gi|31242149|ref|XP_321505.1|</td><td>lcl|At5g38760.1</td><td>lcl|H01G02.3a CE35568 WBGene00010350 status:Confirmed SW:Q7JKB9 protein_id:CAE46676.1 /QuerySize=208</td><td>gi|6803046|ref|Os02g32380|</td></tr>
<tr><td>gi|31242205|ref|XP_321533.1|</td><td>lcl|At5g38770.1</td><td>lcl|H01G02.3b CE35569 WBGene00010350 status:Confirmed SW:Q7JKB8 protein_id:CAE46677.1 /QuerySize=177</td><td>gi|6803047|ref|Os02g32390|</td></tr>
<tr><td>gi|31242207|ref|XP_321534.1|</td><td>lcl|At5g38790.1</td><td>lcl|H01M10.1 CE19912 WBGene00019143 status:Partially_confirmed SW:Q9N5P5 protein_id:AAF39864.1 /QuerySize=320</td><td>gi|6803049|ref|Os02g32410|</td></tr>
<tr><td>gi|31242209|ref|XP_321535.1|</td><td>lcl|At5g38870.1</td><td>lcl|H01M10.2 CE35729 WBGene00019144 status:Partially_confirmed SW:Q9N5P4 protein_id:AAF39863.2 /QuerySize=260</td><td>gi|6803050|ref|Os02g32420|</td></tr>
<tr><td>gi|31242213|ref|XP_321537.1|</td><td>lcl|At5g38980.1</td><td>lcl|H02F09.2 CE35730 WBGene00019145 status:Partially_confirmed SW:O76603 protein_id:AAC64623.2 /QuerySize=175</td><td>gi|6803052|ref|Os02g32440|</td></tr>
<tr><td>gi|31242217|ref|XP_321539.1|</td><td>lcl|At5g39010.1</td><td>lcl|H02F09.3 CE19479 WBGene00019146 status:Predicted SW:O76602 protein_id:AAC64622.1 /QuerySize=1275</td><td>gi|6803053|ref|Os02g32450|</td></tr>
<tr><td>gi|31242239|ref|XP_321550.1|</td><td>lcl|At5g39140.1</td><td>lcl|H02I12.5 CE16162 WBGene00010353 status:Confirmed SW:O45603 protein_id:CAB07219.1 /QuerySize=344</td><td>gi|6803055|ref|Os02g32470|</td></tr>
<tr><td>gi|31242289|ref|XP_321575.1|</td><td>lcl|At5g39170.1</td><td>lcl|H03A11.2 CE32684 WBGene00010357 status:Partially_confirmed SW:Q9XTW1 protein_id:CAB07529.2 /QuerySize=1218</td><td>gi|6803056|ref|Os02g32480|</td></tr>
<tr><td>gi|31242313|ref|XP_321587.1|</td><td>lcl|At5g39200.1</td><td>lcl|H03E18.2 CE11560 WBGene00019149 status:Partially_confirmed SW:Q94249 protein_id:AAB07558.1 /QuerySize=204</td><td>gi|6803059|ref|Os02g32510|</td></tr>
<tr><td>gi|31242317|ref|XP_321589.1|</td><td>lcl|At5g39205.1</td><td>lcl|H03G16.2 CE34042 WBGene00010359 status:Partially_confirmed SW:Q9XU24 protein_id:CAB07224.2 /QuerySize=377</td><td>gi|6803072|ref|Os02g32630|</td></tr>
<tr><td>gi|31242345|ref|XP_321603.1|</td><td>lcl|At5g39240.1</td><td>lcl|H03G16.5 CE27765 WBGene00010361 status:Predicted SW:Q9XU21 protein_id:CAB07227.2 /QuerySize=227</td><td>gi|6803073|ref|Os02g32640|</td></tr>
<tr><td>gi|31242363|ref|XP_321612.1|</td><td>lcl|At5g39470.1</td><td>lcl|H04D03.1 CE16165 WBGene00010362 status:Confirmed SW:O45607 protein_id:CAA15958.1 /QuerySize=204</td><td>gi|6803080|ref|Os02g32710|</td></tr>
<tr><td>gi|31242365|ref|XP_321613.1|</td><td>lcl|At5g39540.1</td><td>lcl|H04D03.2a CE16166 WBGene00010363 status:Partially_confirmed SW:O45606 protein_id:CAA15957.1 /QuerySize=747</td><td>gi|6803081|ref|Os02g32720|</td></tr>
<tr><td>gi|31242405|ref|XP_321633.1|</td><td>lcl|At5g39570.1</td><td>lcl|H04D03.2b CE23792 WBGene00010363 status:Partially_confirmed SW:Q9U3D9 protein_id:CAB54258.1 /QuerySize=749</td><td>gi|6803088|ref|Os02g32790|</td></tr>
<tr><td>gi|31242423|ref|XP_321642.1|</td><td>lcl|At5g39690.1</td><td>lcl|H04J21.3a CE31849 WBGene00001589 locus:gip-1 status:Confirmed SW:O61208 protein_id:AAB94969.2 /QuerySize=891</td><td>gi|6803091|ref|Os02g32820|</td></tr>
<tr><td>gi|31242425|ref|XP_321643.1|</td><td>lcl|At5g39880.1</td><td>lcl|H04J21.3b CE32450 WBGene00001589 locus:gip-1 status:Partially_confirmed SW:Q8ITY3 protein_id:AAN63399.1 /QuerySize=</td><td>gi|6803096|ref|Os02g32870|</td></tr>
<tr><td>gi|31242427|ref|XP_321644.1|</td><td>lcl|At5g39920.1</td><td>lcl|H04J21.3c CE34566 WBGene00001589 locus:gip-1 status:Confirmed SW:Q7YZW0 protein_id:AAP82651.1 /QuerySize=336</td><td>gi|6803099|ref|Os02g32900|</td></tr>
<tr><td>gi|31242447|ref|XP_321654.1|</td><td>lcl|At5g40070.1</td><td>lcl|H04M03.11 CE20930 WBGene00019155 status:Partially_confirmed SW:Q9TYR2 protein_id:AAD12796.1 /QuerySize=203</td><td>gi|6803100|ref|Os02g32910|</td></tr>
<tr><td>gi|31242455|ref|XP_321658.1|</td><td>lcl|At5g40110.1</td><td>lcl|H04M03.12 CE20931 WBGene00019156 status:Partially_confirmed SW:Q9TYQ7 protein_id:AAD12797.1 /QuerySize=260</td><td>gi|6803101|ref|Os02g32920|</td></tr>
<tr><td>gi|31242459|ref|XP_321660.1|</td><td>lcl|At5g40155.1</td><td>lcl|H05C05.1a CE37385 WBGene00019157 status:Partially_confirmed SW:Q9TXU2 protein_id:AAK29840.2 /QuerySize=820</td><td>gi|6803103|ref|Os02g32940|</td></tr>
<tr><td>gi|31242463|ref|XP_321662.1|</td><td>lcl|At5g40180.1</td><td>lcl|H05C05.1b CE32451 WBGene00019157 status:Confirmed SW:Q8ITW7 protein_id:AAN63400.1 /QuerySize=219</td><td>gi|6803113|ref|Os02g33040|</td></tr>
<tr><td>gi|31242469|ref|XP_321665.1|</td><td>lcl|At5g40450.1</td><td>lcl|H05C05.2b CE32453 WBGene00019158 status:Partially_confirmed SW:Q8ITW5 protein_id:AAN63402.1 /QuerySize=243</td><td>gi|6803114|ref|Os02g33050|</td></tr>
<tr><td>gi|31242479|ref|XP_321670.1|</td><td>lcl|At5g40460.1</td><td>lcl|H05C05.3 CE30283 WBGene00019159 status:Predicted SW:Q9TXU4 protein_id:AAK29841.2 /QuerySize=391</td><td>gi|6803115|ref|Os02g33060|</td></tr>
<tr><td>gi|31242483|ref|XP_321672.1|</td><td>lcl|At5g40595.1</td><td>lcl|H05L03.3 CE34043 WBGene00019160 status:Confirmed SW:Q95YA3 protein_id:AAK85489.2 /QuerySize=72</td><td>gi|6803116|ref|Os02g33060|</td></tr>
<tr><td>gi|31242489|ref|XP_321675.1|</td><td>lcl|At5g40600.1</td><td>lcl|H06I04.1c CE31021 WBGene00019167 status:Partially_confirmed SW:Q8MXV3 protein_id:AAM48541.1 /QuerySize=231</td><td>gi|6803117|ref|Os02g33070|</td></tr>
<tr><td>gi|31242505|ref|XP_321683.1|</td><td>lcl|At5g40620.1</td><td>lcl|H08J11.2 CE37248 WBGene00019173 status:Partially_confirmed SW:Q689A0 protein_id:AAU05565.1 /QuerySize=319</td><td>gi|6803121|ref|Os02g33100|</td></tr>
<tr><td>gi|31242509|ref|XP_321685.1|</td><td>lcl|At5g40730.1</td><td>lcl|H08J19.1 CE18806 WBGene00010372 status:Predicted SW:Q9XXK3 protein_id:CAA19426.1 /QuerySize=98</td><td>gi|6803127|ref|Os02g33150|</td></tr>
<tr><td>gi|31242523|ref|XP_321692.1|</td><td>lcl|At5g40790.1</td><td>lcl|H09G03.1 CE34570 WBGene00019174 status:Partially_confirmed SW:Q9TZ60 protein_id:AAC68808.2 /QuerySize=303</td><td>gi|6803129|ref|Os02g33170|</td></tr>
<tr><td>gi|31242525|ref|XP_321693.1|</td><td>lcl|At5g40800.1</td><td>lcl|H09I01.1 CE23799 WBGene00019175 status:Predicted SW:Q9N5N6 protein_id:AAF39872.1 /QuerySize=87</td><td>gi|6803134|ref|Os02g33220|</td></tr>
<tr><td>gi|31242527|ref|XP_321694.1|</td><td>lcl|At5g40855.1</td><td>lcl|H10D12.2 CE17145 WBGene00019176 status:Partially_confirmed SW:O44520 protein_id:AAB93487.1 /QuerySize=256</td><td>gi|6803137|ref|Os02g33250|</td></tr>
<tr><td>gi|31242543|ref|XP_321702.1|</td><td>lcl|At5g40860.1</td><td>lcl|H10D18.1 CE20956 WBGene00019177 status:Partially_confirmed SW:Q9N5N1 protein_id:AAF39873.1 /QuerySize=172</td><td>gi|6803143|ref|Os02g33300|</td></tr>
<tr><td>gi|31242573|ref|XP_321717.1|</td><td>lcl|At5g41320.1</td><td>lcl|H10E21.1 CE32456 WBGene00019182 status:Partially_confirmed SW:O76669 protein_id:AAC26919.2 /QuerySize=87</td><td>gi|6803148|ref|Os02g33350|</td></tr>
<tr><td>gi|31242583|ref|XP_321722.1|</td><td>lcl|At5g41470.1</td><td>lcl|H11E01.3 CE30442 WBGene00019188 status:Partially_confirmed SW:O61802 protein_id:AAC17017.2 /QuerySize=1464</td><td>gi|6803151|ref|Os02g33380|</td></tr>
<tr><td>gi|31242613|ref|XP_321737.1|</td><td>lcl|At5g41530.1</td><td>lcl|H11L12.1 CE20957 WBGene00019189 status:Partially_confirmed SW:Q9TYV1 protein_id:AAC78197.1 /QuerySize=252</td><td>gi|6803152|ref|Os02g33390|</td></tr>
<tr><td>gi|31242621|ref|XP_321741.1|</td><td>lcl|At5g41660.1</td><td>lcl|H12C20.2b CE18807 WBGene00004064 locus:pms-2 status:Confirmed SW:Q9XXM5 protein_id:CAA18354.1 /QuerySize=77</td><td>gi|6803154|ref|Os02g33410|</td></tr>
<tr><td>gi|31242631|ref|XP_321746.1|</td><td>lcl|At5g41765.1</td><td>lcl|H12D21.1 CE15223 WBGene00010377 status:Predicted SW:Q23303 protein_id:CAB07428.1 /QuerySize=34</td><td>gi|6803157|ref|Os02g33440|</td></tr>
<tr><td>gi|31242637|ref|XP_321749.1|</td><td>lcl|At5g41780.1</td><td>lcl|H12D21.11 CE34901 WBGene00010387 status:Predicted SW:Q7YSM8 protein_id:CAE17982.1 /QuerySize=359</td><td>gi|6803164|ref|Os02g33510|</td></tr>
<tr><td>gi|31242641|ref|XP_321751.1|</td><td>lcl|At5g41860.1</td><td>lcl|H12D21.2 CE36698 WBGene00010378 status:Predicted SW:Q9U3D8 protein_id:CAB63209.2 /QuerySize=196</td><td>gi|6803170|ref|Os02g33570|</td></tr>
<tr><td>gi|31242645|ref|XP_321753.1|</td><td>lcl|At5g41930.1</td><td>lcl|H12D21.3 CE15225 WBGene00010379 status:Predicted SW:Q23304 protein_id:CAB07424.1 /QuerySize=83</td><td>gi|6803178|ref|Os02g33640|</td></tr>
<tr><td>gi|31242661|ref|XP_321761.1|</td><td>lcl|At5g42060.1</td><td>lcl|H12D21.5 CE36177 WBGene00010381 status:Predicted SW:Q9XTW7 protein_id:CAB07431.2 /QuerySize=169</td><td>gi|6803179|ref|Os02g33650|</td></tr>
<tr><td>gi|31242669|ref|XP_321765.1|</td><td>lcl|At5g42110.1</td><td>lcl|H12D21.6 CE19919 WBGene00010382 status:Partially_confirmed SW:Q9XTW8 protein_id:CAB07430.1 /QuerySize=163</td><td>gi|6803180|ref|Os02g33660|</td></tr>
<tr><td>gi|31242685|ref|XP_321773.1|</td><td>lcl|At5g42290.1</td><td>lcl|H12D21.9 CE20959 WBGene00010385 status:Partially_confirmed SW:Q9XTW6 protein_id:CAB07432.1 /QuerySize=270</td><td>gi|6803181|ref|Os02g33670|</td></tr>
<tr><td>gi|31242687|ref|XP_321774.1|</td><td>lcl|At5g42330.1</td><td>lcl|H12I13.5 CE27183 WBGene00019193 status:Partially_confirmed SW:Q9BLB7 protein_id:AAK31536.1 /QuerySize=29</td><td>gi|6803183|ref|Os02g33690|</td></tr>
<tr><td>gi|31242691|ref|XP_321776.1|</td><td>lcl|At5g42530.1</td><td>lcl|H12I13.6 CE29972 WBGene00019194 status:Confirmed SW:Q8WTN2 protein_id:AAL32227.1 /QuerySize=75</td><td>gi|6803184|ref|Os02g33700|</td></tr>
<tr><td>gi|31242693|ref|XP_321777.1|</td><td>lcl|At5g42635.1</td><td>lcl|H12I19.8 CE34902 WBGene00010395 status:Predicted SW:Q7YWW6 protein_id:CAE17901.1 /QuerySize=95</td><td>gi|6803191|ref|Os02g33760|</td></tr>
<tr><td>gi|31242697|ref|XP_321779.1|</td><td>lcl|At5g42655.1</td><td>lcl|H13N06.3a CE19924 WBGene00001649 locus:gob-1 status:Partially_confirmed SW:Q9XTQ5 protein_id:CAB17072.1 /QuerySiz</td><td>gi|6803193|ref|Os02g33780|</td></tr>
<tr><td>gi|31242709|ref|XP_321785.1|</td><td>lcl|At5g42710.1</td><td>lcl|H13N06.3b CE29332 WBGene00001649 locus:gob-1 status:Confirmed SW:Q95QD8 protein_id:CAC70091.1 /QuerySize=431</td><td>gi|6803194|ref|Os02g33790|</td></tr>
<tr><td>gi|31242711|ref|XP_321786.1|</td><td>lcl|At5g42750.1</td><td>lcl|H14A12.3 CE28734 WBGene00019196 status:Confirmed SW:O17213 protein_id:AAB70984.2 /QuerySize=292</td><td>gi|6803195|ref|Os02g33800|</td></tr>
<tr><td>gi|31242715|ref|XP_321788.1|</td><td>lcl|At5g42785.1</td><td>lcl|H14A12.5 CE11586 WBGene00019197 status:Partially_confirmed SW:O17215 protein_id:AAB70981.1 /QuerySize=132</td><td>gi|6803196|ref|Os02g33810|</td></tr>
<tr><td>gi|31242719|ref|XP_321790.1|</td><td>lcl|At5g42900.1</td><td>lcl|H14E04.3 CE20968 WBGene00019200 status:Partially_confirmed SW:Q9TYP4 protein_id:AAD12810.1 /QuerySize=188</td><td>gi|6803197|ref|Os02g33820|</td></tr>
<tr><td>gi|31242727|ref|XP_321794.1|</td><td>lcl|At5g42900.2</td><td>lcl|H14E04.4 CE20969 WBGene00019201 status:Predicted SW:Q9TYP3 protein_id:AAD12811.1 /QuerySize=160</td><td>gi|6803205|ref|Os02g33900|</td></tr>
<tr><td>gi|31242757|ref|XP_321809.1|</td><td>lcl|At5g42955.1</td><td>lcl|H14N18.2 CE17974 WBGene00019202 status:Predicted SW:O61979 protein_id:AAC17780.1 /QuerySize=294</td><td>gi|6803207|ref|Os02g33920|</td></tr>
<tr><td>gi|31242765|ref|XP_321813.1|</td><td>lcl|At5g42957.1</td><td>lcl|H15N14.1f CE33127 WBGene00000079 locus:adr-1 status:Confirmed SW:Q8I8G8 protein_id:CAD60419.1 /QuerySize=100</td><td>gi|6803208|ref|Os02g33930|</td></tr>
<tr><td>gi|31242771|ref|XP_321816.1|</td><td>lcl|At5g43000.1</td><td>lcl|H15N14.1g CE33128 WBGene00000079 locus:adr-1 status:Confirmed SW:Q8I8G7 protein_id:CAD60420.1 /QuerySize=115</td><td>gi|6803209|ref|Os02g33940|</td></tr>
<tr><td>gi|31242775|ref|XP_321818.1|</td><td>lcl|At5g43405.1</td><td>lcl|H16D19.3 CE19928 WBGene00010401 status:Predicted SW:Q9U3D5 protein_id:CAA20942.1 /QuerySize=244</td><td>gi|6803213|ref|Os02g33980|</td></tr>
<tr><td>gi|31242785|ref|XP_321823.1|</td><td>lcl|At5g43480.1</td><td>lcl|H16D19.4 CE19929 WBGene00010402 status:Predicted SW:Q9XX93 protein_id:CAA20939.1 /QuerySize=416</td><td>gi|6803221|ref|Os02g34060|</td></tr>
<tr><td>gi|31242803|ref|XP_321832.1|</td><td>lcl|At5g43510.1</td><td>lcl|H16O14.2 CE28735 WBGene00019206 status:Partially_confirmed SW:Q966J2 protein_id:AAK82902.1 /QuerySize=90</td><td>gi|6803222|ref|Os02g34070|</td></tr>
<tr><td>gi|31242823|ref|XP_321842.1|</td><td>lcl|At5g43550.1</td><td>lcl|H17B01.2 CE11592 WBGene00000646 locus:col-70 collagen status:Predicted SW:O61209 protein_id:AAK31537.1 /QuerySize=</td><td>gi|6803224|ref|Os02g34090|</td></tr>
<tr><td>gi|31242829|ref|XP_321845.1|</td><td>lcl|At5g43770.1</td><td>lcl|H18N23.1 CE28736 WBGene00019210 status:Predicted SW:Q9N5M4 protein_id:AAF39884.2 /QuerySize=221</td><td>gi|6803225|ref|Os02g34100|</td></tr>
<tr><td>gi|31242831|ref|XP_321846.1|</td><td>lcl|At5g44040.1</td><td>lcl|H19J13.2 CE34903 WBGene00010404 status:Predicted SW:Q7YSV8 protein_id:CAE17896.1 /QuerySize=153</td><td>gi|6803226|ref|Os02g34110|</td></tr>
<tr><td>gi|31242843|ref|XP_321852.1|</td><td>lcl|At5g44313.1</td><td>lcl|H19M22.2c CE26978 WBGene00002915 locus:let-805 status:Confirmed SW:Q9BKP3 protein_id:AAK26140.1 /QuerySize=386</td><td>gi|6803227|ref|Os02g34120|</td></tr>
<tr><td>gi|31242855|ref|XP_321858.1|</td><td>lcl|At5g44350.1</td><td>lcl|H19M22.3b CE29414 WBGene00019212 status:Confirmed SW:Q95X58 protein_id:AAL06033.1 /QuerySize=101</td><td>gi|6803229|ref|Os02g34140|</td></tr>
</table>

gi|31242881|ref|XP_321871.1|	lcl|At5g44570.1	lcl|H19N07.3 CE16184 WBGene00010407 status:Confirmed SW:O45625 protein_id:CAB07398.1 /QuerySize=168	gi|6803230|ref|Os02g34150|
gi|31242897|ref|XP_321879.1|	lcl|At5g44575.1	lcl|H20E11.1b CE33664 WBGene00019213 status:Confirmed SW:Q86MG8 protein_id:AAO91709.1 /QuerySize=82	gi|6803234|ref|Os02g34190|
gi|31242909|ref|XP_321885.1|	lcl|At5g44580.1	lcl|H20J04.1 CE23811 WBGene00019216 status:Partially_confirmed SW:Q9N5L7 protein_id:AAF39887.1 /QuerySize=843	gi|6803237|ref|Os02g34220|
gi|31242951|ref|XP_321907.1|	lcl|At5g44610.1	lcl|H20J18.1a CE18818 WBGene00004739 locus:scd-1 status:Partially_confirmed SW:Q9XXK5 protein_id:CAA18898.1 /QuerySiz	gi|6803238|ref|Os02g34230|
gi|31242953|ref|XP_321908.1|	lcl|At5g44880.1	lcl|H20J18.1b CE31554 WBGene00004739 locus:scd-1 status:Partially_confirmed SW:Q8MQ12 protein_id:CAD44137.1 /QuerySiz	gi|6803239|ref|Os02g34240|
gi|31242955|ref|XP_321910.1|	lcl|At5g45010.1	lcl|H22K11.3 CE35732 WBGene00019226 status:Partially_confirmed SW:O76631 protein_id:AAK39241.2 /QuerySize=231	gi|6803240|ref|Os02g34250|
gi|31242967|ref|XP_321916.1|	lcl|At5g45310.1	lcl|H23L24.1 CE20982 WBGene00019228 status:Predicted SW:Q9N5L2 protein_id:AAF39892.1 /QuerySize=274	gi|6803243|ref|Os02g34280|
gi|31243009|ref|XP_321939.1|	lcl|At5g45573.1	lcl|H24G06.1d CE37778 WBGene00019237 status:Partially_confirmed SW:Q5TKA5 protein_id:AAV58863.1 /QuerySize=669	gi|6803244|ref|Os02g34290|
gi|31243029|ref|XP_321949.1|	lcl|At5g45830.1	lcl|H25K10.2 CE19934 WBGene00010412 status:Predicted SW:Q9XU19 protein_id:CAB07230.1 /QuerySize=101	gi|6803246|ref|Os02g34310|
gi|31243055|ref|XP_321962.1|	lcl|At5g46115.1	lcl|H25K10.4 CE19936 WBGene00010413 status:Partially_confirmed SW:Q9XU17 protein_id:CAB07232.1 /QuerySize=114	gi|6803250|ref|Os02g34350|
gi|31243061|ref|XP_321965.1|	lcl|At5g46120.1	lcl|H27M09.3 CE23830 WBGene00019247 status:Partially_confirmed SW:Q9N5K3 protein_id:AAF39905.1 /QuerySize=605	gi|6803251|ref|Os02g34360|
gi|31243067|ref|XP_321968.1|	lcl|At5g46295.1	lcl|H27M09.5 CE23827 WBGene00019248 status:Partially_confirmed SW:Q9N5K4 protein_id:AAF39904.1 /QuerySize=341	gi|6803252|ref|Os02g34370|
gi|31243069|ref|XP_321969.1|	lcl|At5g46300.1	lcl|H28G03.2a CE29419 WBGene00019250 status:Confirmed SW:Q9TXP7 protein_id:AAC67404.2 /QuerySize=548	gi|6803253|ref|Os02g34380|
gi|31243071|ref|XP_321970.1|	lcl|At5g46310.1	lcl|H28G03.2b CE31191 WBGene00019250 status:Confirmed SW:Q8MXF5 protein_id:AAM69105.1 /QuerySize=298	gi|6803254|ref|Os02g34390|
gi|31243081|ref|XP_321975.1|	lcl|At5g46320.1	lcl|H28G03.2c CE31192 WBGene00019250 status:Confirmed SW:Q8MXF4 protein_id:AAM69106.1 /QuerySize=275	gi|6803255|ref|Os02g34400|
gi|31243083|ref|XP_321976.1|	lcl|At5g46440.1	lcl|H28G03.3 CE11602 WBGene00019251 intermediate filament protein status:Predicted SW:Q9TXP5 protein_id:AAC67402.1 /Qu	gi|6803259|ref|Os02g34440|
gi|31243101|ref|XP_321987.1|	lcl|At5g46500.1	lcl|H28O16.2 CE34196 WBGene00010420 status:Partially_confirmed SW:Q9XXK2 protein_id:CAA19428.2 /QuerySize=366	gi|6803262|ref|Os02g34470|
gi|31243103|ref|XP_321988.1|	lcl|At5g46560.1	lcl|H31B20.1 CE36871 WBGene00023412 status:Predicted SW:Q69Z21 protein_id:CAH10789.1 /QuerySize=181	gi|6803263|ref|Os02g34480|
gi|31243105|ref|XP_321989.1|	lcl|At5g46730.1	lcl|H31B20.2 CE36872 WBGene00023413 status:Predicted SW:Q69Z20 protein_id:CAH10790.1 /QuerySize=124	gi|6803265|ref|Os02g34500|
gi|31243109|ref|XP_321993.1|	lcl|At5g46770.1	lcl|H31G24.1 CE21002 WBGene00019253 status:Partially_confirmed SW:Q9BLB3 protein_id:AAK21415.1 /QuerySize=109	gi|6803278|ref|Os02g34610|
gi|31243121|ref|XP_322001.1|	lcl|At5g46780.1	lcl|H31G24.3 CE23834 WBGene00019254 status:Predicted SW:Q9BLB4 protein_id:AAK21416.1 /QuerySize=241	gi|6803279|ref|Os02g34620|
gi|31243151|ref|XP_322021.1|	lcl|At5g46780.2	lcl|H32K16.2 CE34907 WBGene00010422 status:Predicted SW:Q7YWX0 protein_id:CAE17890.1 /QuerySize=70	gi|6803281|ref|Os02g34640|
gi|31243169|ref|XP_322030.1|	lcl|At5g46795.1	lcl|H32K21.1 CE37840 WBGene00044006 status:Predicted /QuerySize=72	gi|6803288|ref|Os02g34710|
gi|31243173|ref|XP_322032.1|	lcl|At5g46915.1	lcl|H34C03.1 CE19499 WBGene00019258 status:Partially_confirmed SW:Q9TYY7 protein_id:AAC68976.1 /QuerySize=478	gi|6803295|ref|Os02g34780|
gi|31243179|ref|XP_322035.1|	lcl|At5g47170.1	lcl|H34I24.1 CE19501 WBGene00019260 status:Partially_confirmed SW:O76673 protein_id:AAK18966.1 /QuerySize=258	gi|6803299|ref|Os02g34820|
gi|31243181|ref|XP_322036.1|	lcl|At5g47270.1	lcl|H34I24.2 CE26681 WBGene00019261 status:Confirmed SW:O76672 protein_id:AAK18967.1 /QuerySize=458	gi|6803304|ref|Os02g34870|
gi|31243183|ref|XP_322037.1|	lcl|At5g47290.1	lcl|H35B03.1 CE31708 WBGene00019263 status:Partially_confirmed SW:Q9TYZ4 protein_id:AAC68970.2 /QuerySize=442	gi|6803308|ref|Os02g34910|
gi|31243185|ref|XP_322038.1|	lcl|At5g47455.1	lcl|H35N09.2 CE19944 WBGene00019266 status:Predicted SW:Q9TZM7 protein_id:AAC67408.1 /QuerySize=360	gi|6803309|ref|Os02g34920|
gi|31243187|ref|XP_322039.1|	lcl|At5g47455.2	lcl|H36L18.2 CE19945 WBGene00010424 status:Confirmed SW:Q9XTV5 protein_id:CAB08560.1 /QuerySize=72	gi|6803310|ref|Os02g34930|
gi|31243189|ref|XP_322040.1|	lcl|At5g47455.3	lcl|H38K22.4 CE18832 WBGene00010429 status:Predicted SW:Q9XXA8 protein_id:CAA19706.1 /QuerySize=88	gi|6803315|ref|Os02g34980|
gi|31243191|ref|XP_322041.1|	lcl|At5g47580.1	lcl|H39E20.1 CE19948 WBGene00019267 status:Partially_confirmed SW:Q9TZM8 protein_id:AAC67406.1 /QuerySize=49	gi|6803321|ref|Os02g35040|
gi|31243193|ref|XP_322042.1|	lcl|At5g48045.1	lcl|H39E23.2 CE34908 WBGene00010432 status:Predicted SW:Q7YWY4 protein_id:CAE17867.1 /QuerySize=220	gi|6803329|ref|Os02g35120|
gi|31243195|ref|XP_322043.1|	lcl|At5g48175.1	lcl|H42K12.2 CE27387 WBGene00019271 status:Predicted SW:O17348 protein_id:AAK71372.1 /QuerySize=233	gi|6803344|ref|Os02g35250|
gi|31243197|ref|XP_322044.1|	lcl|At5g48200.1	lcl|H43E16.1 CE19949 WBGene00019273 status:Partially_confirmed SW:Q9N5K0 protein_id:AAF39909.1 /QuerySize=1203	gi|6803345|ref|Os02g35260|
gi|31243199|ref|XP_322045.1|	lcl|At5g48210.1	lcl|JC8.11 CE28343 WBGene00010441 status:Partially_confirmed SW:O62296 protein_id:CAB05233.2 /QuerySize=90	gi|6803347|ref|Os02g35280|
gi|31243201|ref|XP_322046.1|	lcl|At5g48260.1	lcl|JC8.7 CE17991 WBGene00010439 status:Partially_confirmed SW:O62291 protein_id:CAB05227.1 /QuerySize=333	gi|6803372|ref|Os02g35510|
gi|31243205|ref|XP_322048.1|	lcl|At5g48280.1	lcl|K01A11.3 CE36443 WBGene00010451 status:Predicted SW:Q18422 protein_id:CAA91345.2 /QuerySize=247	gi|6803375|ref|Os02g35540|
gi|31243207|ref|XP_322049.1|	lcl|At5g48350.1	lcl|K01A11.5 CE36444 WBGene00010452 Ubiquitin carboxyl-terminal hydrolases family 2 status:Predicted SW:Q6A4R1 protein_id	gi|6803376|ref|Os02g35550|
gi|31243223|ref|XP_322057.1|	lcl|At5g48420.1	lcl|K01A12.2 CE35734 WBGene00019286 status:Partially_confirmed SW:Q10041 protein_id:AAA64339.2 /QuerySize=89	gi|6803378|ref|Os02g35570|
gi|31243225|ref|XP_322058.1|	lcl|At5g48480.1	lcl|K01A2.1 CE35984 WBGene00019277 status:Partially_confirmed SW:Q9TZ49 protein_id:AAC69503.2 /QuerySize=287	gi|6803379|ref|Os02g35580|
gi|31243227|ref|XP_322059.1|	lcl|At5g48500.1	lcl|K01A2.10 CE19519 WBGene00019284 status:Partially_confirmed SW:Q9TZ50 protein_id:AAC69509.1 /QuerySize=257	gi|6803393|ref|Os02g35710|
gi|31243229|ref|XP_322060.1|	lcl|At5g48530.1	lcl|K01A2.2b CE33412 WBGene00001391 locus:far-7 status:Confirmed SW:Q86S19 protein_id:AAO38625.1 /QuerySize=80	gi|6803395|ref|Os02g35730|
gi|31247920|ref|XP_316557.1|	lcl|At5g48575.1	lcl|K01A2.3 CE29422 WBGene00019278 status:Partially_confirmed SW:Q7KNR0 protein_id:AAR12969.2 /QuerySize=119	gi|6803396|ref|Os02g35740|
gi|31247942|ref|XP_316570.1|	lcl|At5g48860.1	lcl|K01A2.4 CE35987 WBGene00019279 status:Partially_confirmed SW:Q7KNR1 protein_id:AAR12970.1 /QuerySize=214	gi|6803401|ref|Os02g35790|
gi|31247954|ref|XP_316576.1|	lcl|At5g48920.1	lcl|K01A2.6 CE19514 WBGene00019281 status:Predicted SW:Q9TZ59 protein_id:AAC69506.1 /QuerySize=170	gi|6803403|ref|Os02g35810|
gi|31247962|ref|XP_316582.1|	lcl|At5g49080.1	lcl|K01A2.8a CE19516 WBGene00019282 status:Confirmed SW:Q9TZ55 protein_id:AAC69501.1 /QuerySize=159	gi|6803408|ref|Os02g35860|
gi|31247966|ref|XP_316584.1|	lcl|At5g49090.1	lcl|K01A2.8b CE19517 WBGene00019282 status:Confirmed SW:Q9TZ54 protein_id:AAC69508.1 /QuerySize=159	gi|6803411|ref|Os02g35890|
gi|31247968|ref|XP_316585.1|	lcl|At5g49100.1	lcl|K01A2.8c CE33413 WBGene00019282 status:Confirmed SW:Q86S20 protein_id:AAO38626.1 /QuerySize=115	gi|6803414|ref|Os02g35920|
gi|31247972|ref|XP_316587.1|	lcl|At5g49170.1	lcl|K01A2.9 CE19518 WBGene00019283 status:Predicted SW:Q9TZ52 protein_id:AAC69500.1 /QuerySize=150	gi|6803415|ref|Os02g35930|
gi|31247974|ref|XP_316588.1|	lcl|At5g49250.1	lcl|K01A6.3 CE06036 WBGene00010445 status:Predicted SW:Q21076 protein_id:CAA92966.1 /QuerySize=320	gi|6803418|ref|Os02g35960|
gi|31247982|ref|XP_316592.1|	lcl|At5g49260.1	lcl|K01A6.4 CE06037 WBGene00010446 status:Partially_confirmed SW:Q21073 protein_id:CAA92963.1 /QuerySize=284	gi|6803420|ref|Os02g35980|
gi|31247984|ref|XP_316593.1|	lcl|At5g49280.1	lcl|K01A6.5 CE37388 WBGene00010447 status:Predicted SW:Q21078 protein_id:CAA92968.2 /QuerySize=184	gi|6803421|ref|Os02g35990|
gi|31247996|ref|XP_316601.1|	lcl|At5g49440.1	lcl|K01A6.6 CE37389 WBGene00010448 status:Confirmed SW:Q21077 protein_id:CAA92967.2 /QuerySize=451	gi|6803422|ref|Os02g36000|
gi|31248004|ref|XP_316605.1|	lcl|At5g49590.1	lcl|K01B6.3 CE27190 WBGene00010454 status:Partially_confirmed SW:P34491 protein_id:CAA80130.2 /QuerySize=410	gi|6803426|ref|Os02g36040|
gi|31248024|ref|XP_316616.1|	lcl|At5g49640.1	lcl|K01B6.4 CE34909 WBGene00010455 status:Predicted SW:Q7YWX6 protein_id:CAE17880.1 /QuerySize=125	gi|6803430|ref|Os02g36080|
gi|31248026|ref|XP_316617.1|	lcl|At5g49790.1	lcl|K01C8.5 CE35891 WBGene00001571 locus:gei-14 status:Partially_confirmed SW:Q21088 protein_id:CAA88863.2 /QuerySize=	gi|6803434|ref|Os02g36120|
gi|31248038|ref|XP_316623.1|	lcl|At5g50010.1	lcl|K01C8.6 CE02267 WBGene00010458 status:Confirmed SW:Q21083 protein_id:CAA88857.1 /QuerySize=239	gi|6803435|ref|Os02g36130|
gi|31248058|ref|XP_316635.1|	lcl|At5g50030.1	lcl|K01D12.10 CE06049 WBGene00010469 status:Partially_confirmed SW:Q21092 protein_id:CAA99869.1 /QuerySize=194	gi|6803439|ref|Os02g36160|
gi|31248084|ref|XP_319161.1|	lcl|At5g50040.1	lcl|K01D12.15 CE28043 WBGene00010474 status:Partially_confirmed SW:Q27223 protein_id:CAA99878.2 /QuerySize=118	gi|6803441|ref|Os02g36180|
gi|31248088|ref|XP_320930.1|	lcl|At5g50050.1	lcl|K01D12.5 CE34198 WBGene00010464 status:Partially_confirmed SW:Q21095 protein_id:CAA99872.2 /QuerySize=270	gi|6803443|ref|Os02g36200|
gi|31248112|ref|XP_321003.1|	lcl|At5g50190.1	lcl|K01D12.7 CE28483 WBGene00010466 status:Partially_confirmed SW:Q95QD3 protein_id:CAA99866.2 /QuerySize=136	gi|6803448|ref|Os02g36250|
gi|31248128|ref|XP_321928.1|	lcl|At5g50335.1	lcl|K01D12.8 CE34199 WBGene00010467 status:Partially_confirmed SW:Q21091 protein_id:CAA99868.2 /QuerySize=139	gi|6803452|ref|Os02g36290|
gi|31248130|ref|XP_321985.1|	lcl|At5g50500.1	lcl|K01D12.9 CE06048 WBGene00010468 status:Partially_confirmed SW:Q21089 protein_id:CAA99865.1 /QuerySize=109	gi|6803454|ref|Os02g36310|
gi|31248132|ref|XP_321986.1|	lcl|At5g50540.1	lcl|K01F9.2 CE34910 WBGene00010475 status:Predicted SW:Q7YTL1 protein_id:CAE17902.1 /QuerySize=156	gi|6803461|ref|Os02g36370|
gi|31248134|ref|XP_321990.1|	lcl|At5g50565.1	lcl|K01G12.3 CE16202 WBGene00010483 status:Predicted SW:O45633 protein_id:CAB05237.1 /QuerySize=596	gi|6803465|ref|Os02g36410|
gi|31248136|ref|XP_321991.1|	lcl|At5g50610.1	lcl|K01G5.3 CE16193 WBGene00010477 status:Confirmed SW:O17914 protein_id:CAB07239.1 /QuerySize=319	gi|6803467|ref|Os02g36430|
gi|31282100|gb|AAM66833.1|	lcl|At5g50645.1	lcl|K01H12.4 CE03456 WBGene00010486 status:Confirmed SW:Q21105 protein_id:CAA92474.1 /QuerySize=329	gi|6803470|ref|Os02g36460|
gi|31282102|gb|AAM66834.1|	lcl|At5g50665.1	lcl|K02A11.2 CE06056 WBGene00010487 status:Partially_confirmed SW:Q21108 protein_id:CAA99879.1 /QuerySize=212	gi|6803472|ref|Os02g36480|
gi|31282104|gb|AAM66835.1|	lcl|At5g50710.1	lcl|K02A2.5 CE32056 WBGene00019290 status:Confirmed SW:Q09401 protein_id:AAC46704.2 /QuerySize=91	gi|6803477|ref|Os02g36530|
gi|31282106|gb|AAM66836.1|	lcl|At5g50820.1	lcl|K02A6.1 CE02793 WBGene00019292 status:Predicted SW:Q21110 protein_id:AAC47060.1 /QuerySize=94	gi|6803478|ref|Os02g36540|
gi|31282108|gb|AAM66837.1|	lcl|At5g50830.1	lcl|K02A6.2 CE02794 WBGene00019293 status:Predicted SW:Q21111 protein_id:AAC47061.1 /QuerySize=111	gi|6803480|ref|Os02g36560|
gi|31282110|gb|AAM66838.1|	lcl|At5g50880.1	lcl|K02A6.3b CE37120 WBGene00019294 status:Confirmed SW:Q7YZW3 protein_id:AAP82648.2 /QuerySize=165	gi|6803482|ref|Os02g36580|
gi|31282112|gb|AAM66839.1|	lcl|At5g50910.1	lcl|K02B12.2 CE06063 WBGene00010495 status:Partially_confirmed SW:Q21114 protein_id:CAB00032.1 /QuerySize=196	gi|6803485|ref|Os02g36610|
gi|31282114|gb|AAM66840.1|	lcl|At5g50940.2	lcl|K02B12.4 CE36181 WBGene00010497 status:Partially_confirmed SW:Q21116 protein_id:CAB00034.3 /QuerySize=282	gi|6803486|ref|Os02g36620|
gi|31282116|gb|AAM66841.1|	lcl|At5g51090.1	lcl|K02B12.6 CE06067 WBGene00010499 status:Partially_confirmed SW:Q21113 protein_id:CAB00030.1 /QuerySize=134	gi|6803490|ref|Os02g36660|
gi|31282118|gb|AAM66842.1|	lcl|At5g51105.1	lcl|K02B2.6 CE04692 WBGene00019297 status:Predicted SW:Q21124 protein_id:AAK39247.1 /QuerySize=143	gi|6803491|ref|Os02g36670|
gi|31282120|gb|AAM66843.1|	lcl|At5g51195.1	lcl|K02B7.2 CE16204 WBGene00010490 status:Predicted SW:O45636 protein_id:CAB05539.1 /QuerySize=391	gi|6803492|ref|Os02g36680|
gi|31282122|gb|AAM66844.1|	lcl|At5g51320.1	lcl|K02B7.3 CE16205 WBGene00010491 status:Confirmed SW:O45637 protein_id:CAB05540.1 /QuerySize=119	gi|6803493|ref|Os02g36690|
gi|31282124|gb|AAM66845.1|	lcl|At5g51390.1	lcl|K02B9.1 CE16206 WBGene00010492 isocitrate lyase like status:Partially_confirmed SW:Q21126 protein_id:CAA93511.1 /Quer	gi|6803496|ref|Os02g36720|
gi|31282126|gb|AAM66846.1|	lcl|At5g51580.1	lcl|K02B9.2 CE35572 WBGene00010493 MYB-related protein like status:Partially_confirmed SW:Q21127 protein_id:CAA93509.2 /	gi|6803497|ref|Os02g36730|
gi|31282128|gb|AAM66847.1|	lcl|At5g51620.1	lcl|K02B9.3 CE06060 WBGene00010494 status:Partially_confirmed SW:Q21125 protein_id:CAA93508.1 /QuerySize=287	gi|6803499|ref|Os02g36750|
gi|31282130|gb|AAM66848.1|	lcl|At5g51650.1	lcl|K02C4.2 CE32686 WBGene00010501 status:Partially_confirmed SW:Q09403 protein_id:CAA87785.2 /QuerySize=216	gi|6803500|ref|Os02g36760|
gi|31282132|gb|AAM66849.1|	lcl|At5g51850.1	lcl|K02C4.5 CE32688 WBGene00010503 status:Confirmed SW:Q8I4H7 protein_id:CAD57701.1 /QuerySize=422	gi|6803506|ref|Os02g36820|
gi|31282134|gb|AAM66850.1|	lcl|At5g52080.1	lcl|K02D10.2 CE00242 WBGene00019302 status:Partially_confirmed SW:P34493 protein_id:AAA28083.1 /QuerySize=124	gi|6803510|ref|Os02g36860|
gi|31282136|gb|AAM66851.1|	lcl|At5g52130.1	lcl|K02D10.3 CE00243 WBGene00019303 status:Predicted SW:P34494 protein_id:AAA28081.1 /QuerySize=105	gi|6803515|ref|Os02g36900|
gi|31282138|gb|AAM66852.1|	lcl|At5g52140.1	lcl|K02D10.4 CE00244 WBGene00019304 status:Partially_confirmed SW:P34495 protein_id:AAA28079.1 /QuerySize=227	gi|6803516|ref|Os02g36910|
gi|31282140|gb|AAM66853.1|	lcl|At5g52300.1	lcl|K02D3.1 CE06069 WBGene00010504 status:Partially_confirmed SW:Q21131 protein_id:CAA94146.1 /QuerySize=183	gi|6803528|ref|Os02g37020|
gi|31282142|gb|AAM66854.1|	lcl|At5g52310.1	lcl|K02D7.4 CE33667 WBGene00001099 locus:dsc-4 status:Confirmed SW:O45104 protein_id:AAC02607.3 /QuerySize=892	gi|6803532|ref|Os02g37060|
gi|31282144|gb|AAM66855.1|	lcl|At5g52500.1	lcl|K02E10.5 CE35735 WBGene00019319 status:Partially_confirmed SW:Q21132 protein_id:AAC47071.2 /QuerySize=132	gi|6803533|ref|Os02g37070|
gi|31282146|gb|AAM66856.1|	lcl|At5g52530.1	lcl|K02E11.10 CE37979 WBGene00044083 locus:tin-9.2 status:Partially_confirmed /QuerySize=360	gi|6803536|ref|Os02g37100|
gi|31282148|gb|AAM66857.1|	lcl|At5g52530.2	lcl|K02E11.3 CE11648 WBGene00010512 status:Partially_confirmed SW:Q21141 protein_id:CAB01219.1 /QuerySize=131	gi|6803537|ref|Os02g37110|
gi|31282150|gb|AAM66858.1|	lcl|At5g52550.1	lcl|K02E11.4 CE11650 WBGene00010513 status:Partially_confirmed SW:Q21140 protein_id:CAB01218.1 /QuerySize=103	gi|6803538|ref|Os02g37120|
gi|31282152|gb|AAM66859.1|	lcl|At5g52670.1	lcl|K02E11.5 CE11652 WBGene00010514 status:Partially_confirmed SW:Q21144 protein_id:CAB01222.1 /QuerySize=126	gi|6803544|ref|Os02g37170|
gi|31282154|gb|AAM66860.1|	lcl|At5g52870.1	lcl|K02E11.6 CE37980 WBGene00010515 status:Predicted SW:Q21143 protein_id:CAB01221.1 /QuerySize=128	gi|6803545|ref|Os02g37180|
gi|31282156|gb|AAM66861.1|	lcl|At5g52890.1	lcl|K02E11.7 CE37981 WBGene00010516 status:Confirmed SW:O62297 protein_id:CAB01224.1 /QuerySize=135	gi|6803546|ref|Os02g37190|
gi|31282158|gb|AAM66862.1|	lcl|At5g52900.1	lcl|K02E11.8 CE34912 WBGene00010517 status:Predicted SW:Q7YWZ0 protein_id:CAE17860.1 /QuerySize=71	gi|6803547|ref|Os02g37200|
gi|31282160|gb|AAM66863.1|	lcl|At5g52965.1	lcl|K02E11.9 CE34913 WBGene00010518 status:Predicted SW:Q7YWY9 protein_id:CAE17861.1 /QuerySize=123	gi|6803548|ref|Os02g37210|
gi|31282162|gb|AAM66864.1|	lcl|At5g52975.1	lcl|K02E2.1 CE18836 WBGene00010506 status:Partially_confirmed SW:Q9XUV4 protein_id:CAB04545.1 /QuerySize=381	gi|6803550|ref|Os02g37230|
gi|31282164|gb|AAM66865.1|	lcl|At5g53030.1	lcl|K02E2.4 CE18839 WBGene00002118 locus:ins-35 status:Confirmed SW:Q9XUV3 protein_id:CAB04546.1 /QuerySize=94	gi|6803551|ref|Os02g37240|
gi|31282166|gb|AAM66866.1|	lcl|At5g53110.1	lcl|K02E2.7 CE18842 WBGene00010508 status:Partially_confirmed SW:Q9XUV5 protein_id:CAB04544.1 /QuerySize=164	gi|6803552|ref|Os02g37250|
gi|31282168|gb|AAM66867.1|	lcl|At5g53250.1	lcl|K02E7.11 CE28409 WBGene00019315 status:Partially_confirmed SW:O17257 protein_id:AAB71020.2 /QuerySize=136	gi|6803553|ref|Os02g37260|
gi|31282170|gb|AAM66868.1|	lcl|At5g53410.1	lcl|K02E7.12 CE33414 WBGene00019316 status:Confirmed SW:Q86LS3 protein_id:AAO38627.1 /QuerySize=55	gi|6803554|ref|Os02g37270|
gi|31282172|gb|AAM66869.1|	lcl|At5g53485.1	lcl|K02E7.2 CE11624 WBGene00019307 status:Predicted SW:O17259 protein_id:AAB71024.1 /QuerySize=411	gi|6803556|ref|Os02g37290|
gi|31282174|gb|AAM66870.1|	lcl|At5g53690.1	lcl|K02E7.4 CE33415 WBGene00019309 status:Predicted SW:O17256 protein_id:AAO38628.1 /QuerySize=233	gi|6803558|ref|Os02g37310|
gi|31282176|gb|AAM66871.1|	lcl|At5g53710.1	lcl|K02E7.7 CE11634 WBGene00019312 status:Predicted SW:O17251 protein_id:AAB71028.1 /QuerySize=196	gi|6803559|ref|Os02g37320|
gi|31282178|gb|AAM66872.1|	lcl|At5g53740.1	lcl|K02F2.5 CE31353 WBGene00019324 status:Confirmed SW:O44983 protein_id:AAB97563.2 /QuerySize=202	gi|6803561|ref|Os02g37340|
gi|31282180|gb|AAM66873.1|	lcl|At5g53820.1	lcl|K02F3.1 CE31028 WBGene00019325 helicase status:Partially_confirmed /QuerySize=514	gi|6803562|ref|Os02g37350|
gi|31282182|gb|AAM66874.1|	lcl|At5g53860.1	lcl|K02F3.10 CE01346 WBGene00019333 status:Confirmed SW:Q21154 protein_id:AAK21418.1 /QuerySize=201	gi|6803563|ref|Os02g37360|
gi|31282184|gb|AAM66875.1|	lcl|At5g53860.2	lcl|K02F3.3 CE34046 WBGene00001392 locus:far-8 status:Partially_confirmed SW:Q21152 protein_id:AAK21422.2 /QuerySize=2	gi|6803564|ref|Os02g37370|
gi|31282186|gb|AAM66876.1|	lcl|At5g53880.1	lcl|K02F3.7 CE01353 WBGene00019330 status:Partially_confirmed SW:Q21149 protein_id:AAK21419.1 /QuerySize=373	gi|6803565|ref|Os02g37380|
gi|31282188|gb|AAM66877.1|	lcl|At5g53895.1	lcl|K02F3.8 CE01354 WBGene00019331 status:Predicted SW:Q21150 protein_id:AAK21423.1 /QuerySize=289	gi|6803566|ref|Os02g37390|
gi|31282190|gb|AAM66878.1|	lcl|At5g53905.1	lcl|K02F3.9 CE01355 WBGene00019332 status:Confirmed SW:Q21151 protein_id:AAK21424.1 /QuerySize=156	gi|6803567|ref|Os02g37400|
gi|31282192|gb|AAM66879.1|	lcl|At5g53960.1	lcl|K02G10.4a CE04704 WBGene00001454 locus:flp-11 status:Confirmed SW:Q21156 protein_id:AAK39250.1 /QuerySize=110	gi|6803568|ref|Os02g37410|
gi|31282194|gb|AAM66880.1|	lcl|At5g54062.1	lcl|K02G10.4b CE31030 WBGene00001454 locus:flp-11 status:Confirmed SW:O61467 protein_id:AAM54174.1 /QuerySize=109	gi|6803571|ref|Os02g37440|
gi|31282196|gb|AAM66881.1|	lcl|At5g54067.1	lcl|K02G10.4c CE34328 WBGene00001454 locus:flp-11 status:Confirmed SW:Q7Z140 protein_id:AAP68932.1 /QuerySize=86	gi|6803572|ref|Os02g37450|
gi|31282198|gb|AAM66882.1|	lcl|At5g54095.1	lcl|K02H11.9 CE30380 WBGene00019350 status:Predicted SW:Q8T7Y5 protein_id:AAM15584.1 /QuerySize=209	gi|6803575|ref|Os02g37480|
gi|31282200|gb|AAM66883.1|	lcl|At5g54145.1	lcl|K03A11.5 CE34049 WBGene00010522 status:Partially_confirmed SW:Q9XVU7 protein_id:CAB00868.2 /QuerySize=149	gi|6803576|ref|Os02g37490|
gi|31282202|gb|AAM66884.1|	lcl|At5g54150.1	lcl|K03B4.1 CE28579 WBGene00019353 status:Confirmed SW:Q21174 protein_id:AAA97998.2 /QuerySize=388	gi|6803577|ref|Os02g37500|
gi|31282204|gb|AAM66885.1|	lcl|At5g54350.1	lcl|K03B4.2 CE07322 WBGene00019354 status:Confirmed SW:Q21173 protein_id:AAA97997.1 /QuerySize=150	gi|6803579|ref|Os02g37520|
gi|31282216|gb|AAM66891.1|	lcl|At5g54410.1	lcl|K03B4.4 CE07324 WBGene00019355 status:Partially_confirmed SW:Q21171 protein_id:AAA97995.1 /QuerySize=87	gi|6803580|ref|Os02g37530|
gi|3128483|gb|AAC16246.1|	lcl|At5g54460.1	lcl|K03B4.7a CE28581 WBGene00019357 status:Confirmed SW:Q21175 protein_id:AAA97999.2 /QuerySize=115	gi|6803581|ref|Os02g37540|
gi|3128485|gb|AAC16248.1|	lcl|At5g54585.1	lcl|K03B4.7b CE33133 WBGene00019357 status:Confirmed SW:Q86NG7 protein_id:AAO21423.1 /QuerySize=99	gi|6803582|ref|Os02g37550|
gi|3281843|emb|CAA07489.1|	lcl|At5g54740.1	lcl|K03B8.11 CE34914 WBGene00010526 status:Predicted SW:Q7YWZ1 protein_id:CAE17859.1 /QuerySize=73	gi|6803583|ref|Os02g37560|
gi|33355871|gb|AAQ16280.1|	lcl|At5g54790.1	lcl|K03B8.8 CE06082 WBGene00010525 status:Partially_confirmed SW:Q21176 protein_id:CAA98499.1 /QuerySize=172	gi|6803584|ref|Os02g37570|
gi|33355877|gb|AAQ16283.1|	lcl|At5g54850.1	lcl|K03C7.1 CE31195 WBGene00019358 status:Partially_confirmed SW:Q21186 protein_id:AAA81138.3 /QuerySize=867	gi|6803589|ref|Os02g37610|
gi|33355879|gb|AAQ16284.1|	lcl|At5g54930.1	lcl|K03D3.2 CE16208 WBGene00010528 status:Predicted SW:O45640 protein_id:CAB05239.1 /QuerySize=144	gi|6803593|ref|Os02g37650|
gi|33355883|gb|AAQ16286.1|	lcl|At5g54970.1	lcl|K03D3.5 CE16211 WBGene00010531 status:Partially_confirmed SW:O45643 protein_id:CAB05242.1 /QuerySize=106	gi|6803594|ref|Os02g37660|
gi|33355885|gb|AAQ16287.1|	lcl|At5g55010.1	lcl|K03D7.9 CE18846 WBGene00010536 status:Predicted SW:O45654 protein_id:CAB04554.1 /QuerySize=211	gi|6803609|ref|Os02g37810|
gi|3378533|emb|CAA03873.1|	lcl|At5g55132.1	lcl|K03E5.5a CE37251 WBGene00019363 status:Partially_confirmed /QuerySize=2122	gi|6803610|ref|Os02g37820|
gi|3378535|emb|CAA03874.1|	lcl|At5g55135.1	lcl|K03E5.5b CE30702 WBGene00019363 status:Partially_confirmed /QuerySize=806	gi|6803613|ref|Os02g37850|
gi|34392588|dbj|BAC82627.1|	lcl|At5g55210.1	lcl|K03F8.1 CE01356 WBGene00019366 status:Predicted SW:Q21195 protein_id:AAA50713.1 /QuerySize=170	gi|6803625|ref|Os02g37960|
gi|37677926|gb|AAQ97584.1|	lcl|At5g55410.1	lcl|K03H1.12 CE21018 WBGene00010547 status:Confirmed SW:Q9XVX7 protein_id:CAA82666.1 /QuerySize=112	gi|6803627|ref|Os02g37980|
gi|37677928|gb|AAQ97585.1|	lcl|At5g55420.1	lcl|K03H1.7 CE00573 WBGene00010542 status:Confirmed SW:Q21196 protein_id:CAA82659.1 /QuerySize=88	gi|6803644|ref|Os02g38100|
gi|37677930|gb|AAQ97586.1|	lcl|At5g55430.1	lcl|K03H1.9 CE33530 WBGene00010544 status:Confirmed SW:Q9U3C2 protein_id:CAA82661.3 /QuerySize=148	gi|6803649|ref|Os02g38150|

<table>
<tr><td>gi|37677932|gb|AAQ97587.1|</td><td>lcl|At5g55507.1</td><td>lcl|K03H4.1 CE11696 WBGene00010548 status:Predicted SW:Q9XVL6 protein_id:CAB03161.1 /QuerySize=391</td><td>gi|6803651|ref|Os02g38170|</td></tr>
<tr><td>gi|37677934|gb|AAQ97588.1|</td><td>lcl|At5g55650.1</td><td>lcl|K03H4.2 CE11698 WBGene00010549 status:Partially_confirmed SW:Q9XVL5 protein_id:CAB03162.1 /QuerySize=264</td><td>gi|6803652|ref|Os02g38180|</td></tr>
<tr><td>gi|37677936|gb|AAQ97589.1|</td><td>lcl|At5g55680.1</td><td>lcl|K03H9.1 CE01995 WBGene00019371 status:Predicted SW:Q09404 protein_id:AAC46667.1 /QuerySize=175</td><td>gi|6803665|ref|Os02g38310|</td></tr>
<tr><td>gi|40019407|emb|CAF01997.1|</td><td>lcl|At5g55750.1</td><td>lcl|K03H9.3 CE01997 WBGene00019372 status:Predicted SW:Q09579 protein_id:AAC46669.1 /QuerySize=457</td><td>gi|6803666|ref|Os02g38320|</td></tr>
<tr><td>gi|40019413|emb|CAF02000.1|</td><td>lcl|At5g55790.1</td><td>lcl|K04A8.3 CE11704 WBGene00019375 status:Partially_confirmed SW:Q94257 protein_id:AAC48049.1 /QuerySize=117</td><td>gi|6803667|ref|Os02g38330|</td></tr>
<tr><td>gi|40019419|emb|CAF02003.1|</td><td>lcl|At5g55855.1</td><td>lcl|K04B12.2a CE35576 WBGene00010550 status:Partially_confirmed SW:O45656 protein_id:CAB05754.2 /QuerySize=420</td><td>gi|6803677|ref|Os02g38420|</td></tr>
<tr><td>gi|4127301|emb|CAA76813.1|</td><td>lcl|At5g55870.1</td><td>lcl|K04B12.2b CE35577 WBGene00010550 status:Partially_confirmed SW:Q7JKG4 protein_id:CAE46672.1 /QuerySize=381</td><td>gi|6803680|ref|Os02g38450|</td></tr>
<tr><td>gi|4127303|emb|CAA76814.1|</td><td>lcl|At5g55893.1</td><td>lcl|K04C2.3a CE00721 WBGene00019381 status:Partially_confirmed SW:Q21208 protein_id:AAA50677.1 /QuerySize=82</td><td>gi|6803686|ref|Os02g38510|</td></tr>
<tr><td>gi|4127305|emb|CAA76815.1|</td><td>lcl|At5g55980.1</td><td>lcl|K04C2.3b CE32689 WBGene00019381 status:Confirmed SW:Q8IG09 protein_id:AAN65313.1 /QuerySize=49</td><td>gi|6803688|ref|Os02g38530|</td></tr>
<tr><td>gi|4127307|emb|CAA76819.1|</td><td>lcl|At5g56070.1</td><td>lcl|K04C2.5 CE00799 WBGene00019382 status:Partially_confirmed SW:Q21211 protein_id:AAA50680.1 /QuerySize=108</td><td>gi|6803689|ref|Os02g38540|</td></tr>
<tr><td>gi|4127309|emb|CAA76820.1|</td><td>lcl|At5g56100.1</td><td>lcl|K04D7.5 CE35579 WBGene00001653 locus:gon-4 status:Partially_confirmed SW:Q21216 protein_id:CAA93515.2 /QuerySize=</td><td>gi|6803695|ref|Os02g38600|</td></tr>
<tr><td>gi|4127329|emb|CAA76821.1|</td><td>lcl|At5g56210.1</td><td>lcl|K04D7.6 CE34915 WBGene00010559 status:Predicted SW:Q7YWX4 protein_id:CAE17882.1 /QuerySize=377</td><td>gi|6803696|ref|Os02g38610|</td></tr>
<tr><td>gi|4127344|emb|CAA76832.1|</td><td>lcl|At5g56520.1</td><td>lcl|K04E7.1 CE02796 WBGene00019383 status:Predicted SW:Q21220 protein_id:AAA80410.1 /QuerySize=217</td><td>gi|6803699|ref|Os02g38640|</td></tr>
<tr><td>gi|4210615|emb|CAA10258.1|</td><td>lcl|At5g56540.1</td><td>lcl|K04F1.8 CE19959 WBGene00019387 status:Predicted SW:Q9TXL2 protein_id:AAC78167.1 /QuerySize=153</td><td>gi|6803708|ref|Os02g38730|</td></tr>
<tr><td>gi|4210617|emb|CAA10259.1|</td><td>lcl|At5g56747.1</td><td>lcl|K04F1.9 CE19960 WBGene00019388 status:Partially_confirmed SW:Q9TXL3 protein_id:AAC78168.1 /QuerySize=139</td><td>gi|6803709|ref|Os02g38740|</td></tr>
<tr><td>gi|4210619|emb|CAA10260.1|</td><td>lcl|At5g56850.1</td><td>lcl|K04F10.3 CE28586 WBGene00019396 status:Partially_confirmed SW:O44769 protein_id:AAB96749.2 /QuerySize=280</td><td>gi|6803710|ref|Os02g38750|</td></tr>
<tr><td>gi|48994208|emb|CAG26920.1|</td><td>lcl|At5g56880.1</td><td>lcl|K04G11.1 CE11742 WBGene00010570 status:Partially_confirmed SW:Q93848 protein_id:CAB01761.1 /QuerySize=218</td><td>gi|6803711|ref|Os02g38760|</td></tr>
<tr><td>gi|50830872|gb|AAT81601.1|</td><td>lcl|At5g56990.1</td><td>lcl|K04G11.2 CE35893 WBGene00004764 locus:sel-7 status:Confirmed SW:Q93846 protein_id:CAE48506.1 /QuerySize=324</td><td>gi|6803712|ref|Os02g38770|</td></tr>
<tr><td>gi|51012501|gb|AAT92557.1|</td><td>lcl|At5g57070.1</td><td>lcl|K04G11.3 CE11746 WBGene00010571 status:Partially_confirmed SW:Q93845 protein_id:CAB01758.1 /QuerySize=368</td><td>gi|6803714|ref|Os02g38790|</td></tr>
<tr><td>gi|51012503|gb|AAT92558.1|</td><td>lcl|At5g57080.1</td><td>lcl|K04G2.10 CE06104 WBGene00010568 status:Partially_confirmed SW:Q21229 protein_id:CAB00047.1 /QuerySize=319</td><td>gi|6803720|ref|Os02g38850|</td></tr>
<tr><td>gi|53766643|gb|AAU93481.1|</td><td>lcl|At5g57310.1</td><td>lcl|K04G7.1 CE01358 WBGene00019400 status:Partially_confirmed SW:Q21234 protein_id:AAA62533.1 /QuerySize=558</td><td>gi|6803721|ref|Os02g38860|</td></tr>
<tr><td>gi|53766645|gb|AAU93482.1|</td><td>lcl|At5g57340.1</td><td>lcl|K04H4.5 CE00250 WBGene00010574 status:Partially_confirmed SW:P34507 protein_id:CAA81585.1 /QuerySize=251</td><td>gi|6803722|ref|Os02g38870|</td></tr>
<tr><td>gi|53766657|gb|AAU93488.1|</td><td>lcl|At5g57400.1</td><td>lcl|K04H4.7 CE36183 WBGene00010575 status:Confirmed SW:Q6LAC9 protein_id:CAE54900.1 /QuerySize=96</td><td>gi|6803723|ref|Os02g38880|</td></tr>
<tr><td>gi|53771817|gb|AAU93514.1|</td><td>lcl|At5g57510.1</td><td>lcl|K04H8.3 CE11760 WBGene00010578 status:Partially_confirmed SW:O01338 protein_id:CAB04565.1 /QuerySize=222</td><td>gi|6803724|ref|Os02g38890|</td></tr>
<tr><td>gi|53988173|gb|AAV28176.1|</td><td>lcl|At5g57650.1</td><td>lcl|K05C4.10 CE19978 WBGene00010586 status:Partially_confirmed SW:Q9XUU7 protein_id:CAB04570.1 /QuerySize=180</td><td>gi|6803726|ref|Os02g38910|</td></tr>
<tr><td>gi|53988179|gb|AAV28179.1|</td><td>lcl|At5g57730.1</td><td>lcl|K05C4.4 CE19972 WBGene00010581 status:Partially_confirmed SW:Q9XUU4 protein_id:CAB04574.1 /QuerySize=432</td><td>gi|6803732|ref|Os02g38960|</td></tr>
<tr><td>gi|53988189|gb|AAV28184.1|</td><td>lcl|At5g57760.1</td><td>lcl|K05C4.8 CE19976 WBGene00010584 status:Predicted SW:Q9XUU6 protein_id:CAB04571.1 /QuerySize=140</td><td>gi|6803735|ref|Os02g38990|</td></tr>
<tr><td>gi|53988215|gb|AAV28196.1|</td><td>lcl|At5g57770.1</td><td>lcl|K05F1.8 CE02812 WBGene00019409 status:Partially_confirmed SW:Q21245 protein_id:AAA68715.1 /QuerySize=78</td><td>gi|6803744|ref|Os02g39080|</td></tr>
<tr><td>gi|54066672|gb|AAV28542.1|</td><td>lcl|At5g57785.1</td><td>lcl|K05F6.10 CE17168 WBGene00019421 status:Predicted SW:O44870 protein_id:AAB95023.1 /QuerySize=277</td><td>gi|6803749|ref|Os02g39130|</td></tr>
<tr><td>gi|54066706|gb|AAV28543.1|</td><td>lcl|At5g57790.1</td><td>lcl|K05G3.1 CE04753 WBGene00019422 status:Partially_confirmed SW:Q21251 protein_id:AAB52496.1 /QuerySize=203</td><td>gi|6803755|ref|Os02g39190|</td></tr>
<tr><td>gi|54066728|gb|AAV28544.1|</td><td>lcl|At5g57820.1</td><td>lcl|K05G3.2 CE35739 WBGene00019423 status:Partially_confirmed SW:Q21249 protein_id:AAB52497.2 /QuerySize=244</td><td>gi|6803757|ref|Os02g39210|</td></tr>
<tr><td>gi|55233240|gb|EAA01736.2|</td><td>lcl|At5g57887.1</td><td>lcl|K06A4.2 CE06106 WBGene00010592 status:Partially_confirmed SW:Q21256 protein_id:CAA94785.1 /QuerySize=220</td><td>gi|6803761|ref|Os02g39250|</td></tr>
<tr><td>gi|55233241|gb|EAL38468.1|</td><td>lcl|At5g57910.1</td><td>lcl|K06A4.6 CE06110 WBGene00010596 status:Predicted SW:Q21255 protein_id:CAA94784.1 /QuerySize=75</td><td>gi|6803763|ref|Os02g39270|</td></tr>
<tr><td>gi|55233255|gb|EAL38469.1|</td><td>lcl|At5g58570.1</td><td>lcl|K06A4.7 CE31556 WBGene00010597 status:Partially_confirmed SW:Q8MQ10 protein_id:CAD44138.1 /QuerySize=55</td><td>gi|6803767|ref|Os02g39310|</td></tr>
<tr><td>gi|55233261|gb|EAA01530.2|</td><td>lcl|At5g58630.1</td><td>lcl|K06A5.1 CE36655 WBGene00019429 status:Partially_confirmed SW:O44553 protein_id:AAK21431.2 /QuerySize=629</td><td>gi|6803768|ref|Os02g39320|</td></tr>
<tr><td>gi|55233262|gb|EAA01563.2|</td><td>lcl|At5g58650.1</td><td>lcl|K06A5.4 CE26645 WBGene00019432 status:Partially_confirmed SW:O44548 protein_id:AAK21435.1 /QuerySize=877</td><td>gi|6803775|ref|Os02g39390|</td></tr>
<tr><td>gi|55233265|gb|EAA43041.2|</td><td>lcl|At5g58790.1</td><td>lcl|K06A9.1a CE19524 WBGene00019435 status:Partially_confirmed SW:P91365 protein_id:AAC70889.1 /QuerySize=1032</td><td>gi|6803781|ref|Os02g39450|</td></tr>
<tr><td>gi|55233270|gb|EAA01559.2|</td><td>lcl|At5g58880.1</td><td>lcl|K06A9.1b CE19525 WBGene00019435 status:Partially_confirmed SW:Q8IFX6 protein_id:AAC70890.1 /QuerySize=2232</td><td>gi|6803786|ref|Os02g39500|</td></tr>
<tr><td>gi|55233272|gb|EAL38475.1|</td><td>lcl|At5g59050.1</td><td>lcl|K06A9.1c CE34576 WBGene00019435 status:Partially_confirmed SW:Q7YZW4 protein_id:AAP82647.1 /QuerySize=825</td><td>gi|6803787|ref|Os02g39510|</td></tr>
<tr><td>gi|55233276|gb|EAL38476.1|</td><td>lcl|At5g59060.1</td><td>lcl|K06B4.3 CE11786 WBGene00010598 status:Predicted SW:O17929 protein_id:CAB05762.1 /QuerySize=83</td><td>gi|6803789|ref|Os02g39530|</td></tr>
<tr><td>gi|55233283|gb|EAL38478.1|</td><td>lcl|At5g59080.1</td><td>lcl|K06B4.4 CE11788 WBGene00010599 status:Partially_confirmed SW:O45663 protein_id:CAB05758.1 /QuerySize=298</td><td>gi|6803790|ref|Os02g39530|</td></tr>
<tr><td>gi|55233300|gb|EAL38484.1|</td><td>lcl|At5g59105.1</td><td>lcl|K06B9.4 CE34917 WBGene00019441 status:Partially_confirmed SW:Q21260 protein_id:AAQ01530.1 /QuerySize=367</td><td>gi|6803796|ref|Os02g39580|</td></tr>
<tr><td>gi|55233311|gb|EAL38486.1|</td><td>lcl|At5g59170.1</td><td>lcl|K06C4.7 CE11814 WBGene00019443 status:Predicted SW:Q9GYH0 protein_id:AAF98221.1 /QuerySize=185</td><td>gi|6803799|ref|Os02g39610|</td></tr>
<tr><td>gi|55233313|gb|EAA01458.2|</td><td>lcl|At5g59305.1</td><td>lcl|K06G5.3 CE18853 WBGene00010607 status:Predicted SW:Q9XUU0 protein_id:CAB04580.1 /QuerySize=344</td><td>gi|6803801|ref|Os02g39630|</td></tr>
<tr><td>gi|55233327|gb|EAA01455.2|</td><td>lcl|At5g59330.1</td><td>lcl|K06H7.1 CE26940 WBGene00019455 Protein kinase status:Predicted SW:P34509 protein_id:AAF99990.2 /QuerySize=316</td><td>gi|6803802|ref|Os02g39640|</td></tr>
<tr><td>gi|55233336|gb|EAL38491.1|</td><td>lcl|At5g59360.1</td><td>lcl|K06H7.7 CE00258 WBGene00019458 status:Partially_confirmed SW:P34515 protein_id:AAF99983.1 /QuerySize=322</td><td>gi|6803820|ref|Os02g39760|</td></tr>
<tr><td>gi|55233341|gb|EAA01444.3|</td><td>lcl|At5g59500.1</td><td>lcl|K07A1.1 CE18854 WBGene00010608 status:Partially_confirmed SW:P90908 protein_id:CAB03174.1 /QuerySize=367</td><td>gi|6803821|ref|Os02g39770|</td></tr>
<tr><td>gi|55233349|gb|EAA01438.3|</td><td>lcl|At5g59510.1</td><td>lcl|K07A1.13 CE18857 WBGene00010617 status:Partially_confirmed SW:Q9XVL4 protein_id:CAB03173.1 /QuerySize=176</td><td>gi|6803825|ref|Os02g39810|</td></tr>
<tr><td>gi|55233364|gb|EAA43063.2|</td><td>lcl|At5g59760.1</td><td>lcl|K07A1.4 CE11846 WBGene00010611 status:Partially_confirmed SW:O01339 protein_id:CAB03166.1 /QuerySize=295</td><td>gi|6803838|ref|Os02g39940|</td></tr>
<tr><td>gi|55233365|gb|EAL38495.1|</td><td>lcl|At5g60000.1</td><td>lcl|K07A1.9c CE32467 WBGene00010615 arginyl-tRNA protein transferase like (truncated) status:Confirmed SW:Q8I4H5 protein_i</td><td>gi|6803843|ref|Os02g39980|</td></tr>
<tr><td>gi|55233375|gb|EAL38497.1|</td><td>lcl|At5g60030.1</td><td>lcl|K07A12.5 CE25934 WBGene00010623 status:Partially_confirmed SW:P90870 protein_id:CAB03185.2 /QuerySize=307</td><td>gi|6803848|ref|Os02g40020|</td></tr>
<tr><td>gi|55233384|gb|EAA01465.2|</td><td>lcl|At5g60060.1</td><td>lcl|K07A9.3 CE17171 WBGene00019462 status:Predicted SW:Q9TXI9 protein_id:AAC68811.1 /QuerySize=189</td><td>gi|6803860|ref|Os02g40120|</td></tr>
<tr><td>gi|55233398|gb|EAL38500.1|</td><td>lcl|At5g60240.1</td><td>lcl|K07A9.4 CE31709 WBGene00019463 status:Partially_confirmed SW:Q8MXE8 protein_id:AAM98005.1 /QuerySize=31</td><td>gi|6803862|ref|Os02g40140|</td></tr>
<tr><td>gi|55233402|gb|EAL38501.1|</td><td>lcl|At5g60260.1</td><td>lcl|K07B1.6a CE11886 WBGene00019466 status:Confirmed SW:O01885 protein_id:AAB54242.1 /QuerySize=163</td><td>gi|6803863|ref|Os02g40150|</td></tr>
<tr><td>gi|55233414|gb|EAL38505.1|</td><td>lcl|At5g60290.1</td><td>lcl|K07B1.6b CE29508 WBGene00019466 status:Confirmed SW:Q95R01 protein_id:AAL08041.1 /QuerySize=42</td><td>gi|6803865|ref|Os02g40170|</td></tr>
<tr><td>gi|55233429|gb|EAA01599.3|</td><td>lcl|At5g60330.1</td><td>lcl|K07B1.8 CE28838 WBGene00019468 status:Confirmed SW:Q95R02 protein_id:AAK84539.1 /QuerySize=34</td><td>gi|6803870|ref|Os02g40210|</td></tr>
<tr><td>gi|55233433|gb|EAL38508.1|</td><td>lcl|At5g60350.1</td><td>lcl|K07C11.10 CE35335 WBGene00019482 status:Partially_confirmed SW:Q21273 protein_id:AAA96182.2 /QuerySize=125</td><td>gi|6803871|ref|Os02g40220|</td></tr>
<tr><td>gi|55233435|gb|EAA01273.2|</td><td>lcl|At5g60400.1</td><td>lcl|K07C11.8 CE37780 WBGene00019480 status:Confirmed SW:Q21269 protein_id:AAA96178.2 /QuerySize=132</td><td>gi|6803879|ref|Os02g40270|</td></tr>
<tr><td>gi|55233437|gb|EAL38509.1|</td><td>lcl|At5g60630.1</td><td>lcl|K07C5.9 CE31358 WBGene00010632 status:Confirmed SW:Q8MQ09 protein_id:CAD44139.1 /QuerySize=76</td><td>gi|6803885|ref|Os02g40330|</td></tr>
<tr><td>gi|55233439|gb|EAA01582.2|</td><td>lcl|At5g60650.1</td><td>lcl|K07C6.1 CE17172 WBGene00019469 locus:srz-89 status:Predicted SW:O44653 protein_id:AAB94244.1 /QuerySize=276</td><td>gi|6803889|ref|Os02g40370|</td></tr>
<tr><td>gi|55233454|gb|EAL38511.1|</td><td>lcl|At5g60810.1</td><td>lcl|K07D4.2 CE19526 WBGene00019483 status:Partially_confirmed SW:O76578 protein_id:AAC26286.1 /QuerySize=194</td><td>gi|6803894|ref|Os02g40420|</td></tr>
<tr><td>gi|55233503|gb|EAA01679.2|</td><td>lcl|At5g60840.1</td><td>lcl|K07D4.4 CE19528 WBGene00019484 status:Predicted SW:O76575 protein_id:AAC26288.1 /QuerySize=199</td><td>gi|6803904|ref|Os02g40520|</td></tr>
<tr><td>gi|55233518|gb|EAL38523.1|</td><td>lcl|At5g60880.1</td><td>lcl|K07D4.5 CE35991 WBGene00019485 status:Confirmed SW:O76574 protein_id:AAC26289.2 /QuerySize=54</td><td>gi|6803906|ref|Os02g40540|</td></tr>
<tr><td>gi|55233535|gb|EAA43096.2|</td><td>lcl|At5g61100.1</td><td>lcl|K07D4.6 CE19530 WBGene00019486 status:Partially_confirmed SW:O76573 protein_id:AAC26290.1 /QuerySize=389</td><td>gi|6803908|ref|Os02g40560|</td></tr>
<tr><td>gi|55233568|gb|EAL38530.1|</td><td>lcl|At5g61110.1</td><td>lcl|K07D4.9 CE32058 WBGene00019488 status:Confirmed SW:Q8ITW9 protein_id:AAN60512.1 /QuerySize=102</td><td>gi|6803911|ref|Os02g40590|</td></tr>
<tr><td>gi|55233570|gb|EAA01548.2|</td><td>lcl|At5g61120.1</td><td>lcl|K07E12.1b CE31032 WBGene00019500 status:Partially_confirmed SW:Q8MQ08 protein_id:AAM48546.1 /QuerySize=12268</td><td>gi|6803912|ref|Os02g40600|</td></tr>
<tr><td>gi|55233624|gb|EAL38541.1|</td><td>lcl|At5g61300.1</td><td>lcl|K07E12.2 CE01362 WBGene00019501 status:Partially_confirmed SW:Q21283 protein_id:AAA50716.1 /QuerySize=91</td><td>gi|6803913|ref|Os02g40610|</td></tr>
<tr><td>gi|55233627|gb|EAL38542.1|</td><td>lcl|At5g61360.1</td><td>lcl|K07E3.1 CE04718 WBGene00019490 status:Partially_confirmed SW:Q21288 protein_id:AAC69098.1 /QuerySize=1247</td><td>gi|6803914|ref|Os02g40620|</td></tr>
<tr><td>gi|55233680|gb|EAL38555.1|</td><td>lcl|At5g61605.1</td><td>lcl|K07E3.2 CE04719 WBGene00019491 status:Partially_confirmed SW:Q21287 protein_id:AAC69099.1 /QuerySize=837</td><td>gi|6803915|ref|Os02g40630|</td></tr>
<tr><td>gi|55233692|gb|EAA01365.3|</td><td>lcl|At5g61630.1</td><td>lcl|K07E8.10 CE11904 WBGene00019499 7TM chemoreceptor</td><td>gi|6803916|ref|Os02g40640|</td></tr>
<tr><td>gi|55233696|gb|EAL38561.1|</td><td>lcl|At5g61660.1</td><td>lcl|K07F5.11 CE06125 WBGene00006050 locus:ssq-1 status:Partially_confirmed SW:Q21294 protein_id:CAA94280.1 /QuerySize=</td><td>gi|6803917|ref|Os02g40650|</td></tr>
<tr><td>gi|55233738|gb|EAA01641.2|</td><td>lcl|At5g61710.1</td><td>lcl|K07G5.4 CE06132 WBGene00010643 status:Partially_confirmed SW:Q21300 protein_id:CAA95827.1 /QuerySize=325</td><td>gi|6803919|ref|Os02g40670|</td></tr>
<tr><td>gi|55233770|gb|EAA43158.2|</td><td>lcl|At5g61865.1</td><td>lcl|K07G6.1 CE21030 WBGene00019502 status:Partially_confirmed SW:Q9TYQ3 protein_id:AAD12802.1 /QuerySize=133</td><td>gi|6803921|ref|Os02g40690|</td></tr>
<tr><td>gi|55233784|gb|EAA01342.2|</td><td>lcl|At5g62070.1</td><td>lcl|K07H8.5 CE18025 WBGene00019506 status:Predicted SW:O45178 protein_id:AAC04424.1 /QuerySize=409</td><td>gi|6803924|ref|Os02g40720|</td></tr>
<tr><td>gi|55233807|gb|EAA01327.2|</td><td>lcl|At5g62110.1</td><td>lcl|K07H8.7 CE18027 WBGene00019507 status:Predicted SW:O45175 protein_id:AAC04422.1 /QuerySize=146</td><td>gi|6803928|ref|Os02g40760|</td></tr>
<tr><td>gi|55233820|gb|EAA00869.2|</td><td>lcl|At5g62170.1</td><td>lcl|K08B4.2 CE25940 WBGene00019514 status:Partially_confirmed SW:Q9TYY6 protein_id:AAC68979.2 /QuerySize=173</td><td>gi|6803932|ref|Os02g40800|</td></tr>
<tr><td>gi|55233821|gb|EAL38578.1|</td><td>lcl|At5g62240.1</td><td>lcl|K08B4.7 CE37255 WBGene00023486 status:Confirmed SW:Q688A2 protein_id:AAU05562.1 /QuerySize=65</td><td>gi|6803934|ref|Os02g40820|</td></tr>
<tr><td>gi|55233827|gb|EAA01284.2|</td><td>lcl|At5g62280.1</td><td>lcl|K08B5.2 CE11908 WBGene00019519 status:Partially_confirmed SW:Q21308 protein_id:AAA82338.2 /QuerySize=248</td><td>gi|6803945|ref|Os02g40910|</td></tr>
<tr><td>gi|55233835|gb|EAA01286.2|</td><td>lcl|At5g62330.1</td><td>lcl|K08C7.4 CE35587 WBGene00010647 status:Partially_confirmed SW:Q21314 protein_id:CAA94294.2 /QuerySize=194</td><td>gi|6803950|ref|Os02g41450|</td></tr>
<tr><td>gi|55233836|gb|EAL38582.1|</td><td>lcl|At5g62400.1</td><td>lcl|K08C7.6 CE06139 WBGene00010648 status:Partially_confirmed SW:Q21315 protein_id:CAA94362.1 /QuerySize=175</td><td>gi|6803954|ref|Os02g41490|</td></tr>
<tr><td>gi|55233837|gb|EAA00866.2|</td><td>lcl|At5g62750.1</td><td>lcl|K08C7.7 CE21039 WBGene00010649 status:Partially_confirmed SW:Q9XVV6 protein_id:CAA94296.1 /QuerySize=298</td><td>gi|6803958|ref|Os02g41530|</td></tr>
<tr><td>gi|55233877|gb|EAA01347.2|</td><td>lcl|At5g62770.1</td><td>lcl|K08C9.1 CE18858 WBGene00010650 status:Confirmed SW:Q9XUT5 protein_id:CAB04586.1 /QuerySize=240</td><td>gi|6803959|ref|Os02g41540|</td></tr>
<tr><td>gi|55233880|gb|EAA01282.2|</td><td>lcl|At5g63050.1</td><td>lcl|K08C9.2 CE18859 WBGene00010651 status:Partially_confirmed SW:Q9XUT6 protein_id:CAB04585.1 /QuerySize=235</td><td>gi|6803965|ref|Os02g41570|</td></tr>
<tr><td>gi|55233884|gb|EAA01288.2|</td><td>lcl|At5g63150.1</td><td>lcl|K08C9.6 CE21040 WBGene00010653 status:Predicted SW:Q9XUT2 protein_id:CAB04589.1 /QuerySize=127</td><td>gi|6803968|ref|Os02g41600|</td></tr>
<tr><td>gi|55233895|gb|EAA43185.2|</td><td>lcl|At5g63300.1</td><td>lcl|K08C9.7 CE18863 WBGene00010654 Ubiquitin family status:Predicted SW:Q9XUT3 protein_id:CAB04588.1 /QuerySize=114</td><td>gi|6803969|ref|Os02g41610|</td></tr>
<tr><td>gi|55233896|gb|EAL38595.1|</td><td>lcl|At5g63340.1</td><td>lcl|K08D10.10 CE07362 WBGene00019533 status:Partially_confirmed SW:Q21316 protein_id:AAA98026.1 /QuerySize=394</td><td>gi|6803970|ref|Os02g41620|</td></tr>
<tr><td>gi|55233911|gb|EAA01587.3|</td><td>lcl|At5g63720.1</td><td>lcl|K08D10.11 CE07363 WBGene00019534 status:Predicted SW:Q21326 protein_id:AAA98036.1 /QuerySize=153</td><td>gi|6803972|ref|Os02g41640|</td></tr>
<tr><td>gi|55233914|gb|EAL38599.1|</td><td>lcl|At5g63740.1</td><td>lcl|K08D10.9 CE07361 WBGene00019532 status:Predicted SW:Q21317 protein_id:AAA98027.1 /QuerySize=259</td><td>gi|6803983|ref|Os02g41730|</td></tr>
<tr><td>gi|55233918|gb|EAL38601.1|</td><td>lcl|At5g63905.1</td><td>lcl|K08D12.4 CE21044 WBGene00019538 status:Partially_confirmed SW:Q966I9 protein_id:AAK84543.1 /QuerySize=100</td><td>gi|6803985|ref|Os02g41750|</td></tr>
<tr><td>gi|55233924|gb|EAA01556.2|</td><td>lcl|At5g64010.1</td><td>lcl|K08D12.6 CE21047 WBGene00019540 status:Confirmed SW:Q8MXU8 protein_id:AAM98004.1 /QuerySize=668</td><td>gi|6803989|ref|Os02g41790|</td></tr>
<tr><td>gi|55233957|gb|EAL38605.1|</td><td>lcl|At5g64170.1</td><td>lcl|K08D8.1 CE06140 WBGene00010655 status:Predicted SW:Q21331 protein_id:CAA97436.1 /QuerySize=144</td><td>gi|6803991|ref|Os02g41810|</td></tr>
<tr><td>gi|55233958|gb|EAL38606.1|</td><td>lcl|At5g64310.1</td><td>lcl|K08D8.2 CE21041 WBGene00010656 status:Predicted SW:Q21328 protein_id:CAA97433.1 /QuerySize=123</td><td>gi|6803993|ref|Os02g41830|</td></tr>
<tr><td>gi|55233960|gb|EAA01393.2|</td><td>lcl|At5g64340.1</td><td>lcl|K08E3.8 CE18874 WBGene00007025 locus:mdt-29 status:Partially_confirmed SW:Q9XUS2 protein_id:CAB04600.1 /QuerySize</td><td>gi|6803995|ref|Os02g41850|</td></tr>
<tr><td>gi|55233963|gb|EAA01476.3|</td><td>lcl|At5g64480.1</td><td>lcl|K08E4.3 CE03463 WBGene00010667 status:Predicted SW:Q21334 protein_id:CAA92681.1 /QuerySize=352</td><td>gi|6804000|ref|Os02g41880|</td></tr>
<tr><td>gi|55233964|gb|EAA01501.2|</td><td>lcl|At5g64540.1</td><td>lcl|K08E4.4 CE03464 WBGene00010668 status:Predicted SW:Q21332 protein_id:CAA92679.1 /QuerySize=89</td><td>gi|6804002|ref|Os02g41900|</td></tr>
<tr><td>gi|55233971|gb|EAL38607.1|</td><td>lcl|At5g64690.1</td><td>lcl|K08E7.4 CE06148 WBGene00010672 status:Predicted SW:Q21343 protein_id:CAB01227.1 /QuerySize=139</td><td>gi|6804015|ref|Os02g42030|</td></tr>
<tr><td>gi|55233973|gb|EAA01413.3|</td><td>lcl|At5g64770.1</td><td>lcl|K08E7.5 CE11924 WBGene00010673 status:Partially_confirmed SW:Q21344 protein_id:CAB01228.1 /QuerySize=1221</td><td>gi|6804017|ref|Os02g42050|</td></tr>
<tr><td>gi|55233976|gb|EAL38608.1|</td><td>lcl|At5g64800.1</td><td>lcl|K08E7.6 CE11926 WBGene00010674 status:Partially_confirmed SW:Q21345 protein_id:CAB01229.1 /QuerySize=263</td><td>gi|6804019|ref|Os02g42070|</td></tr>
<tr><td>gi|55233979|gb|EAL38609.1|</td><td>lcl|At5g64820.1</td><td>lcl|K08F11.1 CE11950 WBGene00019541 status:Predicted SW:Q94265 protein_id:AAB09159.1 /QuerySize=134</td><td>gi|6804021|ref|Os02g42090|</td></tr>
<tr><td>gi|55233982|gb|EAA43206.2|</td><td>lcl|At5g64880.1</td><td>lcl|K08F4.12 CE34923 WBGene00010680 status:Predicted SW:Q7YWZ2 protein_id:CAE17858.1 /QuerySize=93</td><td>gi|6804022|ref|Os02g42100|</td></tr>
<tr><td>gi|55233988|gb|EAL38610.1|</td><td>lcl|At5g64890.1</td><td>lcl|K08F4.5 CE06153 WBGene00010679 status:Partially_confirmed SW:Q21354 protein_id:CAA93085.1 /QuerySize=215</td><td>gi|6804024|ref|Os02g42120|</td></tr>
<tr><td>gi|55233990|gb|EAL38611.1|</td><td>lcl|At5g64900.1</td><td>lcl|K08F8.5a CE03471 WBGene00010682 status:Partially_confirmed SW:Q21365 protein_id:CAA91287.1 /QuerySize=340</td><td>gi|6804036|ref|Os02g42240|</td></tr>
<tr><td>gi|55233991|gb|EAL38612.1|</td><td>lcl|At5g64905.1</td><td>lcl|K08F8.5b CE11938 WBGene00010682 status:Partially_confirmed SW:P90926 protein_id:CAA91288.1 /QuerySize=312</td><td>gi|6804047|ref|Os02g42340|</td></tr>
<tr><td>gi|55234000|gb|EAL38615.1|</td><td>lcl|At5g65120.1</td><td>lcl|K08F8.7 CE34924 WBGene00010683 status:Predicted SW:Q7YWX2 protein_id:CAE17887.1 /QuerySize=197</td><td>gi|6804049|ref|Os02g42360|</td></tr>
<tr><td>gi|55234013|gb|EAL38617.1|</td><td>lcl|At5g65207.1</td><td>lcl|K08F9.4 CE11948 WBGene00010687 status:Partially_confirmed SW:O17941 protein_id:CAB05811.1 /QuerySize=517</td><td>gi|6804053|ref|Os02g42400|</td></tr>
<tr><td>gi|55234018|gb|EAA01333.2|</td><td>lcl|At5g65300.1</td><td>lcl|K08H10.3 CE18876 WBGene00010696 status:Predicted SW:Q9XU85 protein_id:CAB05542.1 /QuerySize=120</td><td>gi|6804059|ref|Os02g42460|</td></tr>
<tr><td>gi|55234020|gb|EAA01326.2|</td><td>lcl|At5g65390.1</td><td>lcl|K08H2.2 CE06160 WBGene00010689 status:Partially_confirmed SW:Q21366 protein_id:CAA94148.1 /QuerySize=200</td><td>gi|6804060|ref|Os02g42470|</td></tr>
<tr><td>gi|55234028|gb|EAL38621.1|</td><td>lcl|At5g65480.1</td><td>lcl|K08H2.3 CE06161 WBGene00010690 status:Partially_confirmed SW:Q21368 protein_id:CAA94150.1 /QuerySize=267</td><td>gi|6804061|ref|Os02g42480|</td></tr>
<tr><td>gi|55234044|gb|EAA01779.2|</td><td>lcl|At5g65495.1</td><td>lcl|K08H2.4 CE06162 WBGene00010691 status:Partially_confirmed SW:Q21369 protein_id:CAA94151.1 /QuerySize=142</td><td>gi|6804062|ref|Os02g42490|</td></tr>
<tr><td>gi|55234075|gb|EAA01703.2|</td><td>lcl|At5g65580.1</td><td>lcl|K08H2.7 CE06165 WBGene00010693 status:Predicted SW:Q21371 protein_id:CAA94153.1 /QuerySize=142</td><td>gi|6804063|ref|Os02g42500|</td></tr>
<tr><td>gi|55234093|gb|EAL38628.1|</td><td>lcl|At5g65610.1</td><td>lcl|K08H2.9 CE34925 WBGene00010694 status:Predicted SW:Q7YWW9 protein_id:CAE17897.1 /QuerySize=403</td><td>gi|6804064|ref|Os02g42510|</td></tr>
<tr><td>gi|55234095|gb|EAA01696.2|</td><td>lcl|At5g65660.1</td><td>lcl|K09B11.10 CE35588 WBGene00010714 status:Partially_confirmed SW:Q9U3B4 protein_id:CAB05554.3 /QuerySize=712</td><td>gi|6804073|ref|Os02g42560|</td></tr>
<tr><td>gi|55234112|gb|EAL38632.1|</td><td>lcl|At5g65880.1</td><td>lcl|K09B11.3 CE19990 WBGene00010710 status:Predicted SW:Q9XU78 protein_id:CAB05552.1 /QuerySize=114</td><td>gi|6804078|ref|Os02g42610|</td></tr>
<tr><td>gi|55234117|gb|EAL38634.1|</td><td>lcl|At5g65925.1</td><td>lcl|K09B11.4 CE19991 WBGene00010711 status:Predicted SW:Q9XU79 protein_id:CAB05551.1 /QuerySize=142</td><td>gi|6804090|ref|Os02g42720|</td></tr>
<tr><td>gi|55234128|gb|EAL38637.1|</td><td>lcl|At5g66000.1</td><td>lcl|K09C4.10 CE04738 WBGene00019554 status:Partially_confirmed SW:Q21380 protein_id:AAA83622.1 /QuerySize=1035</td><td>gi|6804091|ref|Os02g42730|</td></tr>
<tr><td>gi|55234129|gb|EAA01720.3|</td><td>lcl|At5g66052.1</td><td>lcl|K09C4.2 CE04730 WBGene00019548 status:Predicted SW:Q21384 protein_id:AAA83626.1 /QuerySize=58</td><td>gi|6804095|ref|Os02g42770|</td></tr>
<tr><td>gi|55234135|gb|EAA01690.3|</td><td>lcl|At5g66340.1</td><td>lcl|K09C4.4 CE04732 WBGene00019549 sugar transporter status:Predicted SW:Q21382 protein_id:AAA83624.1 /QuerySize=271</td><td>gi|6804108|ref|Os02g42900|</td></tr>
<tr><td>gi|55234150|gb|EAA01469.2|</td><td>lcl|At5g66370.1</td><td>lcl|K09C4.6 CE04734 WBGene00019551 status:Partially_confirmed SW:Q21379 protein_id:AAA83621.1 /QuerySize=205</td><td>gi|6804111|ref|Os02g42930|</td></tr>
<tr><td>gi|55234151|gb|EAL38640.1|</td><td>lcl|At5g66400.1</td><td>lcl|K09C4.7 CE04735 WBGene00019552 status:Predicted SW:Q21378 protein_id:AAA83620.1 /QuerySize=110</td><td>gi|6804116|ref|Os02g42980|</td></tr>
<tr><td>gi|55234159|gb|EAA01520.2|</td><td>lcl|At5g66440.1</td><td>lcl|K09C4.9 CE04737 WBGene00019553 status:Predicted SW:Q21377 protein_id:AAA83619.1 /QuerySize=107</td><td>gi|6804124|ref|Os02g43060|</td></tr>
<tr><td>gi|55234160|gb|EAA01540.2|</td><td>lcl|At5g66490.1</td><td>lcl|K09C6.2 CE11990 WBGene00019556 status:Partially_confirmed SW:O16946 protein_id:AAB70669.1 /QuerySize=344</td><td>gi|6804125|ref|Os02g43070|</td></tr>
<tr><td>gi|55234166|gb|EAA43247.2|</td><td>lcl|At5g66800.1</td><td>lcl|K09C6.3 CE11992 WBGene00019557 status:Predicted SW:O16944 protein_id:AAB70670.1 /QuerySize=115</td><td>gi|6804132|ref|Os02g43140|</td></tr>
<tr><td>gi|55234168|gb|EAA01030.2|</td><td>lcl|At5g66815.1</td><td>lcl|K09C6.9 CE35339 WBGene00019563 status:Partially_confirmed SW:O16945 protein_id:AAB70667.3 /QuerySize=153</td><td>gi|6804134|ref|Os02g43160|</td></tr>
<tr><td>gi|55234171|gb|EAL38647.1|</td><td>lcl|At5g66820.1</td><td>lcl|K09C8.2 CE12006 WBGene00010715 status:Confirmed SW:O01341 protein_id:CAA91998.1 /QuerySize=251</td><td>gi|6804139|ref|Os02g43210|</td></tr>
<tr><td>gi|55234177|gb|EAA01546.2|</td><td>lcl|At5g66985.1</td><td>lcl|K09C8.7 CE34926 WBGene00010717 status:Predicted SW:Q7YWY3 protein_id:CAE17869.1 /QuerySize=97</td><td>gi|6804141|ref|Os02g43230|</td></tr>
<tr><td>gi|55234179|gb|EAL38649.1|</td><td>lcl|At5g67350.1</td><td>lcl|K09C8.8 CE34927 WBGene00010718 status:Predicted SW:Q7YWY2 protein_id:CAE17870.1 /QuerySize=107</td><td>gi|6804142|ref|Os02g43240|</td></tr>
<tr><td>gi|55234201|gb|EAL38652.1|</td><td>lcl|At5g67600.1</td><td>lcl|K09D9.1 CE21055 WBGene00019564 status:Partially_confirmed SW:Q9N5I0 protein_id:AAF39922.1 /QuerySize=295</td><td>gi|6804149|ref|Os02g43310|</td></tr>
<tr><td>gi|55234204|gb|EAL38653.1|</td><td>lcl|At5g67640.1</td><td>lcl|K09D9.12 CE21066 WBGene00019569 status:Predicted SW:Q9N5I8 protein_id:AAF39930.1 /QuerySize=512</td><td>gi|6804150|ref|Os02g43320|</td></tr>
<tr><td>gi|55234206|gb|EAA43259.2|</td><td></td><td>lcl|K09D9.3 CE21057 WBGene00019566 status:Predicted SW:Q9N5I2 protein_id:AAF39920.1 /QuerySize=311</td><td>gi|6804157|ref|Os02g43380|</td></tr>
<tr><td>gi|55234209|gb|EAL38655.1|</td><td></td><td>lcl|K09D9.9 CE21063 WBGene00019567 status:Predicted SW:Q9N5J1 protein_id:AAF39927.1 /QuerySize=272</td><td>gi|6804161|ref|Os02g43420|</td></tr>
<tr><td>gi|55234226|gb|EAL38658.1|</td><td></td><td>lcl|K09E2.2 CE37259 WBGene00019571 status:Partially_confirmed SW:Q21393 protein_id:AAC46573.2 /QuerySize=552</td><td>gi|6804163|ref|Os02g43440|</td></tr>
<tr><td>gi|55234231|gb|EAA00576.2|</td><td></td><td>lcl|K09E2.3 CE02816 WBGene00019572 status:Partially_confirmed SW:Q21392 protein_id:AAC46574.1 /QuerySize=210</td><td>gi|6804168|ref|Os02g43490|</td></tr>
<tr><td>gi|55234233|gb|EAL38661.1|</td><td></td><td>lcl|K09E2.4b CE34053 WBGene00004369 locus:rig-1 status:Confirmed SW:Q7Z2A2 protein_id:AAP40525.1 /QuerySize=300</td><td>gi|6804173|ref|Os02g43530|</td></tr>
<tr><td>gi|55234234|gb|EAL38662.1|</td><td></td><td>lcl|K09E3.1 CE04739 WBGene00019573 S. lavendulae subtilisin inhibitor-like protein-4 (SIL-4) status:Predicted SW:Q21400 protei</td><td>gi|6804174|ref|Os02g43540|</td></tr>
</table>

<table>
<tr><td>gi|55234241|gb|EAL38664.1|</td><td>lcl|K09E3.2 CE04740 WBGene00019574 status:Predicted SW:Q21398 protein_id:AAA82375.1 /QuerySize=109</td><td>gi|6804177|ref|Os02g43570|</td></tr>
<tr><td>gi|55234250|gb|EAA00031.2|</td><td>lcl|K09E3.4 CE04742 WBGene00019575 status:Predicted SW:Q21399 protein_id:AAA82376.1 /QuerySize=31</td><td>gi|6804178|ref|Os02g43580|</td></tr>
<tr><td>gi|55234264|gb|EAA00582.2|</td><td>lcl|K09E3.7 CE04745 WBGene00019578 status:Predicted SW:Q21401 protein_id:AAA82378.1 /QuerySize=859</td><td>gi|6804182|ref|Os02g43610|</td></tr>
<tr><td>gi|55234266|gb|EAA00587.2|</td><td>lcl|K09E4.5 CE30158 WBGene00001805 locus:gur-4 status:Partially_confirmed SW:Q9XU57 protein_id:CAB05770.2 /QuerySize=</td><td>gi|6804184|ref|Os02g43630|</td></tr>
<tr><td>gi|55234268|gb|EAA00572.2|</td><td>lcl|K09E4.6 CE34928 WBGene00010723 status:Partially_confirmed SW:Q7YWX9 protein_id:CAE17873.1 /QuerySize=116</td><td>gi|6804185|ref|Os02g43640|</td></tr>
<tr><td>gi|55234279|gb|EAA00517.2|</td><td>lcl|K09E9.3 CE12014 WBGene00010726 status:Partially_confirmed SW:Q93879 protein_id:CAB01890.1 /QuerySize=474</td><td>gi|6804186|ref|Os02g43650|</td></tr>
<tr><td>gi|55234287|gb|EAL38672.1|</td><td>lcl|K09F5.1 CE02818 WBGene00019582 status:Predicted SW:Q21405 protein_id:AAB52672.1 /QuerySize=414</td><td>gi|6804189|ref|Os02g43680|</td></tr>
<tr><td>gi|55234295|gb|EAL38676.1|</td><td>lcl|K09F5.4 CE02821 WBGene00019583 status:Predicted SW:Q21402 protein_id:AAB52673.1 /QuerySize=145</td><td>gi|6804196|ref|Os02g43730|</td></tr>
<tr><td>gi|55234299|gb|EAA00535.2|</td><td>lcl|K09F6.10 CE31562 WBGene00019593 status:Partially_confirmed SW:Q8MXJ4 protein_id:AAM97998.1 /QuerySize=480</td><td>gi|6804216|ref|Os02g43880|</td></tr>
<tr><td>gi|55234302|gb|EAL38677.1|</td><td>lcl|K09F6.5 CE12032 WBGene00019588 status:Predicted SW:O16731 protein_id:AAB66195.1 /QuerySize=410</td><td>gi|6804217|ref|Os02g43890|</td></tr>
<tr><td>gi|55234303|gb|EAL38678.1|</td><td>lcl|K09F6.6 CE31564 WBGene00019589 status:Partially_confirmed SW:O16732 protein_id:AAB66196.2 /QuerySize=1010</td><td>gi|6804218|ref|Os02g43900|</td></tr>
<tr><td>gi|55234304|gb|EAL38679.1|</td><td>lcl|K09F6.8 CE12038 WBGene00019591 status:Partially_confirmed SW:O16735 protein_id:AAB66198.1 /QuerySize=405</td><td>gi|6804227|ref|Os02g43950|</td></tr>
<tr><td>gi|55234306|gb|EAL38680.1|</td><td>lcl|K09F6.9 CE31565 WBGene00019592 status:Partially_confirmed SW:Q8MXJ5 protein_id:AAM97999.1 /QuerySize=609</td><td>gi|6804228|ref|Os02g43960|</td></tr>
<tr><td>gi|55234307|gb|EAL38681.1|</td><td>lcl|K09G1.2 CE12042 WBGene00010728 status:Partially_confirmed SW:Q9XVL0 protein_id:CAB03198.1 /QuerySize=127</td><td>gi|6804230|ref|Os02g43980|</td></tr>
<tr><td>gi|55234309|gb|EAL38682.1|</td><td>lcl|K09G1.3 CE12044 WBGene00010729 status:Predicted SW:Q9XVL2 protein_id:CAB03196.1 /QuerySize=53</td><td>gi|6804231|ref|Os02g43990|</td></tr>
<tr><td>gi|55234312|gb|EAL38683.1|</td><td>lcl|K09H11.4 CE12054 WBGene00019601 status:Partially_confirmed SW:O01586 protein_id:AAB52267.1 /QuerySize=630</td><td>gi|6804234|ref|Os02g44020|</td></tr>
<tr><td>gi|55234314|gb|EAL38684.1|</td><td>lcl|K09H11.5 CE36879 WBGene00019602 status:Predicted SW:O01587 protein_id:AAB52264.2 /QuerySize=83</td><td>gi|6804235|ref|Os02g44030|</td></tr>
<tr><td>gi|55234322|gb|EAL38687.1|</td><td>lcl|K09H11.6 CE35340 WBGene00019603 status:Partially_confirmed SW:O01589 protein_id:AAB52265.2 /QuerySize=166</td><td>gi|6804242|ref|Os02g44100|</td></tr>
<tr><td>gi|55234326|gb|EAL38688.1|</td><td>lcl|K09H9.2 CE34336 WBGene00019595 status:Confirmed SW:O44992 protein_id:AAB97569.2 /QuerySize=443</td><td>gi|6804243|ref|Os02g44100|</td></tr>
<tr><td>gi|55234329|gb|EAL38689.1|</td><td>lcl|K09H9.5a CE36703 WBGene00019597 status:Partially_confirmed SW:O44990 protein_id:AAB97572.2 /QuerySize=424</td><td>gi|6804247|ref|Os02g44140|</td></tr>
<tr><td>gi|55234331|gb|EAL38691.1|</td><td>lcl|K09H9.5b CE33146 WBGene00019597 status:Confirmed SW:Q8IA63 protein_id:AAO12406.1 /QuerySize=173</td><td>gi|6804249|ref|Os02g44160|</td></tr>
<tr><td>gi|55234344|gb|EAA00791.2|</td><td>lcl|K10C2.2 CE04748 WBGene00019618 status:Partially_confirmed SW:Q94270 protein_id:AAK39257.1 /QuerySize=208</td><td>gi|6804254|ref|Os02g44210|</td></tr>
<tr><td>gi|55234347|gb|EAL38698.1|</td><td>lcl|K10C2.6 CE04752 WBGene00019621 status:Partially_confirmed SW:Q94274 protein_id:AAK39261.1 /QuerySize=114</td><td>gi|6804257|ref|Os02g44240|</td></tr>
<tr><td>gi|55234362|gb|EAL38703.1|</td><td>lcl|K10C2.7 CE34054 WBGene00019622 status:Confirmed SW:Q967F0 protein_id:AAK39262.2 /QuerySize=95</td><td>gi|6804260|ref|Os02g44270|</td></tr>
<tr><td>gi|55234371|gb|EAA00584.2|</td><td>lcl|K10C3.4 CE12080 WBGene00010731 status:Partially_confirmed SW:O45665 protein_id:CAB05771.1 /QuerySize=370</td><td>gi|6804261|ref|Os02g44270|</td></tr>
<tr><td>gi|55234374|gb|EAL38706.1|</td><td>lcl|K10C8.4 CE34929 WBGene00010737 status:Predicted SW:Q7YWY0 protein_id:CAE17872.1 /QuerySize=147</td><td>gi|6804267|ref|Os02g44300|</td></tr>
<tr><td>gi|55234384|gb|EAL38708.1|</td><td>lcl|K10C9.1 CE18043 WBGene00019623 status:Predicted SW:O61891 protein_id:AAC17669.1 /QuerySize=71</td><td>gi|6804271|ref|Os02g44340|</td></tr>
<tr><td>gi|55234385|gb|EAL38709.1|</td><td>lcl|K10C9.7 CE18048 WBGene00019626 status:Partially_confirmed SW:O61890 protein_id:AAC17671.1 /QuerySize=269</td><td>gi|6804280|ref|Os02g44430|</td></tr>
<tr><td>gi|55234399|gb|EAL38713.1|</td><td>lcl|K10D2.4 CE02016 WBGene00019630 status:Confirmed SW:Q09410 protein_id:AAA62543.1 /QuerySize=81</td><td>gi|6804291|ref|Os02g44540|</td></tr>
<tr><td>gi|55234427|gb|EAA00238.2|</td><td>lcl|K10D3.6 CE29596 WBGene00010740 status:Confirmed SW:Q8WQE3 protein_id:CAD01087.1 /QuerySize=108</td><td>gi|6804292|ref|Os02g44540|</td></tr>
<tr><td>gi|55234428|gb|EAA00575.3|</td><td>lcl|K10D6.3 CE06177 WBGene00010743 status:Partially_confirmed SW:Q21421 protein_id:CAA98510.1 /QuerySize=83</td><td>gi|6804296|ref|Os02g44580|</td></tr>
<tr><td>gi|55234432|gb|EAL38723.1|</td><td>lcl|K10D6.4 CE36185 WBGene00010744 status:Confirmed SW:Q21423 protein_id:CAA98512.2 /QuerySize=294</td><td>gi|6804300|ref|Os02g44620|</td></tr>
<tr><td>gi|55234442|gb|EAL38725.1|</td><td>lcl|K10E9.1 CE18050 WBGene00019634 status:Partially_confirmed SW:O61779 protein_id:AAC17021.1 /QuerySize=1451</td><td>gi|6804312|ref|Os02g44710|</td></tr>
<tr><td>gi|55234443|gb|EAA00581.2|</td><td>lcl|K10F12.4b CE31038 WBGene00019636 status:Partially_confirmed SW:Q8IA74 protein_id:AAN72424.1 /QuerySize=123</td><td>gi|6804313|ref|Os02g44720|</td></tr>
<tr><td>gi|55234444|gb|EAL38726.1|</td><td>lcl|K10F12.6 CE12096 WBGene00019638 status:Predicted SW:O17236 protein_id:AAB71004.1 /QuerySize=121</td><td>gi|6804316|ref|Os02g44750|</td></tr>
<tr><td>gi|55234451|gb|EAL38728.1|</td><td>lcl|K10F12.7 CE31039 WBGene00019639 status:Partially_confirmed SW:Q8IA73 protein_id:AAN72425.1 /QuerySize=129</td><td>gi|6804317|ref|Os02g44760|</td></tr>
<tr><td>gi|55234460|gb|EAL38730.1|</td><td>lcl|K10G4.3 CE16246 WBGene00010752 status:Partially_confirmed SW:O45672 protein_id:CAB07256.1 /QuerySize=202</td><td>gi|6804322|ref|Os02g44800|</td></tr>
<tr><td>gi|55234472|gb|EAA00546.3|</td><td>lcl|K10G6.2 CE12100 WBGene00019640 status:Partially_confirmed SW:O16627 protein_id:AAB66097.1 /QuerySize=275</td><td>gi|6804325|ref|Os02g44830|</td></tr>
<tr><td>gi|55234489|gb|EAL38739.1|</td><td>lcl|K10G6.3 CE32201 WBGene00004751 locus:sea-2 status:Partially_confirmed SW:O16625 protein_id:AAB66098.3 /QuerySize=</td><td>gi|6804326|ref|Os02g44840|</td></tr>
<tr><td>gi|55234490|gb|EAL38740.1|</td><td>lcl|K10G6.4 CE28602 WBGene00019641 status:Partially_confirmed SW:O16626 protein_id:AAB66099.2 /QuerySize=455</td><td>gi|6804337|ref|Os02g44950|</td></tr>
<tr><td>gi|55234492|gb|EAL38741.1|</td><td>lcl|K10G9.2 CE37538 WBGene00010756 status:Confirmed SW:Q21425 protein_id:CAA85288.3 /QuerySize=240</td><td>gi|6804338|ref|Os02g44960|</td></tr>
<tr><td>gi|55234493|gb|EAA00495.2|</td><td>lcl|K10H10.10 CE34932 WBGene00010765 status:Predicted SW:Q7YWX5 protein_id:CAE17881.1 /QuerySize=321</td><td>gi|6804342|ref|Os02g45000|</td></tr>
<tr><td>gi|55234494|gb|EAL38742.1|</td><td>lcl|K10H10.4 CE16254 WBGene00010760 status:Confirmed SW:O45681 protein_id:CAB05780.1 /QuerySize=214</td><td>gi|6804345|ref|Os02g45030|</td></tr>
<tr><td>gi|55234506|gb|EAA00514.2|</td><td>lcl|K10H10.5 CE16255 WBGene00010761 status:Partially_confirmed SW:O45682 protein_id:CAB05781.1 /QuerySize=191</td><td>gi|6804349|ref|Os02g45060|</td></tr>
<tr><td>gi|55234529|gb|EAA00491.2|</td><td>lcl|K10H10.9 CE28603 WBGene00010764 status:Predicted SW:O45683 protein_id:CAB05782.2 /QuerySize=257</td><td>gi|6804357|ref|Os02g45140|</td></tr>
<tr><td>gi|55234530|gb|EAA00206.2|</td><td>lcl|K11D12.1 CE30382 WBGene00000862 locus:cwp-4 status:Confirmed SW:O44626 protein_id:AAM15587.1 /QuerySize=402</td><td>gi|6804364|ref|Os02g45190|</td></tr>
<tr><td>gi|55234535|gb|EAL38751.1|</td><td>lcl|K11D12.8 CE12140 WBGene00019648 status:Predicted SW:O44623 protein_id:AAB94228.1 /QuerySize=303</td><td>gi|6804366|ref|Os02g45220|</td></tr>
<tr><td>gi|55234541|gb|EAL38752.1|</td><td>lcl|K11H12.5 CE18054 WBGene00019661 status:Partially_confirmed SW:P91368 protein_id:AAC24404.1 /QuerySize=290</td><td>gi|6804367|ref|Os02g45230|</td></tr>
<tr><td>gi|55234557|gb|EAL38755.1|</td><td>lcl|K11H12.6 CE34340 WBGene00019662 status:Partially_confirmed SW:P91371 protein_id:AAC24396.2 /QuerySize=132</td><td>gi|6804370|ref|Os02g45270|</td></tr>
<tr><td>gi|55234564|gb|EAA00637.3|</td><td>lcl|K11H12.8b CE31566 WBGene00019664 status:Confirmed SW:Q8MPU0 protein_id:AAM98000.1 /QuerySize=112</td><td>gi|6804371|ref|Os02g45260|</td></tr>
<tr><td>gi|55234565|gb|EAL38757.1|</td><td>lcl|K11H3.2 CE00262 WBGene00010779 status:Predicted SW:P34518 protein_id:CAA80175.1 /QuerySize=228</td><td>gi|6804373|ref|Os02g45290|</td></tr>
<tr><td>gi|55234572|gb|EAL38760.1|</td><td>lcl|K11H3.5 CE37781 WBGene00010782 status:Predicted SW:P34521 protein_id:CAA80177.2 /QuerySize=107</td><td>gi|6804374|ref|Os02g45300|</td></tr>
<tr><td>gi|55234576|gb|EAL38761.1|</td><td>lcl|K12B6.4 CE12170 WBGene00019669 status:Partially_confirmed SW:O16381 protein_id:AAB65919.1 /QuerySize=203</td><td>gi|6804378|ref|Os02g45330|</td></tr>
<tr><td>gi|55234580|gb|EAL38762.1|</td><td>lcl|K12B6.6 CE12174 WBGene00019670 status:Predicted SW:O16384 protein_id:AAB65914.1 /QuerySize=120</td><td>gi|6804381|ref|Os02g45360|</td></tr>
<tr><td>gi|55234581|gb|EAL38763.1|</td><td>lcl|K12B6.7 CE12176 WBGene00019671 status:Predicted SW:O16385 protein_id:AAB65920.1 /QuerySize=481</td><td>gi|6804386|ref|Os02g45410|</td></tr>
<tr><td>gi|55234585|gb|EAL38764.1|</td><td>lcl|K12D12.4a CE36704 WBGene00010786 status:Partially_confirmed /QuerySize=342</td><td>gi|6804389|ref|Os02g45440|</td></tr>
<tr><td>gi|55234606|gb|EAA00310.2|</td><td>lcl|K12F2.2b CE23870 WBGene00006874 locus:vab-8 kinesin-like protein status:Confirmed SW:Q9TVW0 protein_id:CAB54275.1</td><td>gi|6804392|ref|Os02g45470|</td></tr>
<tr><td>gi|55234623|gb|EAL38779.1|</td><td>lcl|K12H4.5 CE00270 WBGene00019680 status:Confirmed SW:P34526 protein_id:AAA28095.1 /QuerySize=97</td><td>gi|6804396|ref|Os02g45500|</td></tr>
<tr><td>gi|55234628|gb|EAL38780.1|</td><td>lcl|K12H4.6 CE00271 WBGene00019681 status:Predicted SW:P34527 protein_id:AAA28094.1 /QuerySize=84</td><td>gi|6804402|ref|Os02g45560|</td></tr>
<tr><td>gi|55234633|gb|EAL38784.1|</td><td>lcl|K12H6.12 CE20015 WBGene00019691 status:Partially_confirmed SW:Q9N5H8 protein_id:AAF39941.1 /QuerySize=405</td><td>gi|6804404|ref|Os02g45580|</td></tr>
<tr><td>gi|55234638|gb|EAA00018.2|</td><td>lcl|K12H6.2 CE35997 WBGene00019683 status:Partially_confirmed SW:Q9N5H7 protein_id:AAF39932.2 /QuerySize=276</td><td>gi|6804409|ref|Os02g45630|</td></tr>
<tr><td>gi|55234639|gb|EAL38788.1|</td><td>lcl|K12H6.4 CE20007 WBGene00019684 status:Partially_confirmed SW:Q9N5H3 protein_id:AAF39934.1 /QuerySize=107</td><td>gi|6804410|ref|Os02g45640|</td></tr>
<tr><td>gi|55234640|gb|EAL38789.1|</td><td>lcl|K12H6.5 CE20008 WBGene00019685 status:Predicted SW:Q9N5H1 protein_id:AAF39935.1 /QuerySize=178</td><td>gi|6804420|ref|Os02g45740|</td></tr>
<tr><td>gi|55234647|gb|EAL38792.1|</td><td>lcl|K12H6.7 CE20010 WBGene00019687 status:Partially_confirmed SW:Q9N5G8 protein_id:AAF39942.1 /QuerySize=281</td><td>gi|6804429|ref|Os02g45830|</td></tr>
<tr><td>gi|55234651|gb|EAL38794.1|</td><td>lcl|K12H6.8 CE20011 WBGene00019688 status:Predicted SW:Q9N5H0 protein_id:AAF39937.1 /QuerySize=103</td><td>gi|6804430|ref|Os02g45840|</td></tr>
<tr><td>gi|55234657|gb|EAL38797.1|</td><td>lcl|K12H6.9 CE33423 WBGene00019689 status:Partially_confirmed SW:Q9N5H2 protein_id:AAF39938.2 /QuerySize=131</td><td>gi|6804445|ref|Os02g45990|</td></tr>
<tr><td>gi|55234660|gb|EAL38798.1|</td><td>lcl|M01A12.3 CE33534 WBGene00019695 status:Predicted SW:Q86NC7 protein_id:AAO44911.1 /QuerySize=118</td><td>gi|6804448|ref|Os02g46020|</td></tr>
<tr><td>gi|55234661|gb|EAL38799.1|</td><td>lcl|M01A8.1 CE00275 WBGene00010795 status:Partially_confirmed SW:P34530 protein_id:CAA81606.1 /QuerySize=203</td><td>gi|6804463|ref|Os02g46170|</td></tr>
<tr><td>gi|55234662|gb|EAL38800.1|</td><td>lcl|M01B12.1 CE18059 WBGene00019696 status:Predicted SW:O61863 protein_id:AAC17560.1 /QuerySize=154</td><td>gi|6804471|ref|Os02g46250|</td></tr>
<tr><td>gi|55234663|gb|EAL38801.1|</td><td>lcl|M01B12.4a CE29982 WBGene00019697 status:Confirmed SW:O61862 protein_id:AAC17562.3 /QuerySize=634</td><td>gi|6804474|ref|Os02g46270|</td></tr>
<tr><td>gi|55234674|gb|EAL38804.1|</td><td>lcl|M01B12.4b CE30554 WBGene00019697 status:Partially_confirmed SW:Q8T7Y6 protein_id:AAM15592.1 /QuerySize=644</td><td>gi|6804475|ref|Os02g46280|</td></tr>
<tr><td>gi|55234687|gb|EAL38809.1|</td><td>lcl|M01B12.4c CE37031 WBGene00019697 status:Partially_confirmed SW:Q8MXG4 protein_id:AAM81100.2 /QuerySize=678</td><td>gi|6804477|ref|Os02g46300|</td></tr>
<tr><td>gi|55234696|gb|EAA00755.2|</td><td>lcl|M01D7.5 CE12268 WBGene00003750 locus:nlp-12 status:Confirmed SW:O01970 protein_id:AAB58066.1 /QuerySize=80</td><td>gi|6804480|ref|Os02g46330|</td></tr>
<tr><td>gi|55234697|gb|EAL38812.1|</td><td>lcl|M01E11.3 CE12292 WBGene00019712 status:Partially_confirmed SW:P91399 protein_id:AAB37829.1 /QuerySize=926</td><td>gi|6804488|ref|Os02g46390|</td></tr>
<tr><td>gi|55234698|gb|EAL38813.1|</td><td>lcl|M01E11.4a CE28610 WBGene00019713 locus:pqn-52 status:Confirmed SW:Q95ZL6 protein_id:AAK77613.1 /QuerySize=244</td><td>gi|6804489|ref|Os02g46400|</td></tr>
<tr><td>gi|55234701|gb|EAA00720.2|</td><td>lcl|M01E11.4b CE28611 WBGene00019713 locus:pqn-52 status:Confirmed SW:Q95ZL7 protein_id:AAK77614.1 /QuerySize=252</td><td>gi|6804490|ref|Os02g46410|</td></tr>
<tr><td>gi|55234706|gb|EAL38817.1|</td><td>lcl|M01E11.4c CE37032 WBGene00019713 locus:pqn-52 status:Confirmed /QuerySize=544</td><td>gi|6804491|ref|Os02g46420|</td></tr>
<tr><td>gi|55234717|gb|EAL38819.1|</td><td>lcl|M01E11.7d CE30558 WBGene00006508 locus:tag-163 status:Partially_confirmed SW:Q8T3B1 protein_id:AAM15595.1 /Query</td><td>gi|6804497|ref|Os02g46480|</td></tr>
<tr><td>gi|55234718|gb|EAL38820.1|</td><td>lcl|M01E5.1 CE12274 WBGene00010804 status:Predicted SW:O17964 protein_id:CAB07638.1 /QuerySize=312</td><td>gi|6804502|ref|Os02g46530|</td></tr>
<tr><td>gi|55234719|gb|EAA00317.3|</td><td>lcl|M01E5.3 CE16272 WBGene00010806 status:Partially_confirmed SW:O45692 protein_id:CAB07641.1 /QuerySize=558</td><td>gi|6804503|ref|Os02g46540|</td></tr>
<tr><td>gi|55234729|gb|EAL38823.1|</td><td>lcl|M01E5.4 CE12280 WBGene00010807 status:Partially_confirmed SW:O45690 protein_id:CAB07636.1 /QuerySize=250</td><td>gi|6804504|ref|Os02g46550|</td></tr>
<tr><td>gi|55234759|gb|EAL38831.1|</td><td>lcl|M01F1.8a CE33806 WBGene00010814 status:Confirmed SW:Q7YXB7 protein_id:CAD92392.1 /QuerySize=328</td><td>gi|6804511|ref|Os02g46620|</td></tr>
<tr><td>gi|55234762|gb|EAL38832.1|</td><td>lcl|M01F1.8b CE34206 WBGene00010814 status:Partially_confirmed SW:Q7YXB6 protein_id:CAD92393.1 /QuerySize=124</td><td>gi|6804516|ref|Os02g46670|</td></tr>
<tr><td>gi|55234770|gb|EAL38838.1|</td><td>lcl|M01F1.9 CE34938 WBGene00010815 status:Predicted SW:Q7YWW8 protein_id:CAE17898.1 /QuerySize=187</td><td>gi|6804530|ref|Os02g46790|</td></tr>
<tr><td>gi|55234791|gb|EAA00549.2|</td><td>lcl|M01G12.10 CE18073 WBGene00010823 status:Predicted SW:O62307 protein_id:CAB04609.1 /QuerySize=144</td><td>gi|6804531|ref|Os02g46800|</td></tr>
<tr><td>gi|55234822|gb|EAL38846.1|</td><td>lcl|M01G12.11 CE18074 WBGene00010824 status:Predicted SW:O62314 protein_id:CAB04617.1 /QuerySize=59</td><td>gi|6804533|ref|Os02g46820|</td></tr>
<tr><td>gi|55234832|gb|EAL38849.1|</td><td>lcl|M01G12.14 CE27588 WBGene00010825 status:Partially_confirmed SW:O62316 protein_id:CAB04619.2 /QuerySize=350</td><td>gi|6804534|ref|Os02g46830|</td></tr>
<tr><td>gi|55234865|gb|EAL38857.1|</td><td>lcl|M01G12.7 CE18071 WBGene00010821 status:Predicted SW:O62313 protein_id:CAB04616.1 /QuerySize=460</td><td>gi|6804547|ref|Os02g46950|</td></tr>
<tr><td>gi|55234887|gb|EAL38864.1|</td><td>lcl|M01G12.9 CE18072 WBGene00010822 status:Predicted SW:O62317 protein_id:CAB04620.1 /QuerySize=272</td><td>gi|6804555|ref|Os02g47030|</td></tr>
<tr><td>gi|55234891|gb|EAL38865.1|</td><td>lcl|M01G4.1 CE02829 WBGene00019714 status:Predicted SW:Q21457 protein_id:AAC46537.1 /QuerySize=63</td><td>gi|6804557|ref|Os02g47050|</td></tr>
<tr><td>gi|55234905|gb|EAA00794.3|</td><td>lcl|M01G5.1 CE19536 WBGene00019715 status:Predicted SW:O76686 protein_id:AAC26941.1 /QuerySize=889</td><td>gi|6804559|ref|Os02g47070|</td></tr>
<tr><td>gi|55234906|gb|EAA00761.2|</td><td>lcl|M01H9.3a CE28614 WBGene00019719 status:Confirmed SW:Q95ZL2 protein_id:AAK77617.1 /QuerySize=246</td><td>gi|6804560|ref|Os02g47080|</td></tr>
<tr><td>gi|55234924|gb|EAA00760.2|</td><td>lcl|M01H9.3b CE28615 WBGene00019719 status:Confirmed SW:Q95ZL3 protein_id:AAK77618.1 /QuerySize=152</td><td>gi|6804562|ref|Os02g47100|</td></tr>
<tr><td>gi|55234935|gb|EAL38876.1|</td><td>lcl|M01H9.3c CE37722 WBGene00019719 status:Confirmed SW:Q5TYL1 protein_id:AAV34789.1 /QuerySize=195</td><td>gi|6804576|ref|Os02g47240|</td></tr>
<tr><td>gi|55234936|gb|EAA00772.2|</td><td>lcl|M01H9.4 CE34581 WBGene00019720 status:Partially_confirmed SW:Q94281 protein_id:AAB09145.2 /QuerySize=225</td><td>gi|6804577|ref|Os02g47250|</td></tr>
<tr><td>gi|55234942|gb|EAL38877.1|</td><td>lcl|M01H9.5 CE31716 WBGene00019721 status:Partially_confirmed SW:Q8MPT9 protein_id:AAM97995.1 /QuerySize=228</td><td>gi|6804581|ref|Os02g47290|</td></tr>
<tr><td>gi|55234944|gb|EAL38879.1|</td><td>lcl|M02A10.1 CE07386 WBGene00019722 status:Partially_confirmed SW:Q21459 protein_id:AAC48057.1 /QuerySize=130</td><td>gi|6804582|ref|Os02g47300|</td></tr>
<tr><td>gi|55234969|gb|EAL38890.1|</td><td>lcl|M02B1.2 CE12314 WBGene00010827 status:Partially_confirmed SW:Q93891 protein_id:CAB03201.1 /QuerySize=144</td><td>gi|6804585|ref|Os02g47330|</td></tr>
<tr><td>gi|55234974|gb|EAL38891.1|</td><td>lcl|M02B1.4 CE12318 WBGene00010829 status:Partially_confirmed SW:Q93893 protein_id:CAB03203.1 /QuerySize=313</td><td>gi|6804591|ref|Os02g47390|</td></tr>
<tr><td>gi|55234975|gb|EAL38892.1|</td><td>lcl|M02B7.1 CE12320 WBGene00019723 status:Partially_confirmed SW:Q94286 protein_id:AAB09127.1 /QuerySize=305</td><td>gi|6804599|ref|Os02g47440|</td></tr>
<tr><td>gi|55234977|gb|EAL38893.1|</td><td>lcl|M02D8.2 CE35743 WBGene00019728 status:Partially_confirmed SW:Q21462 protein_id:AAA82380.2 /QuerySize=127</td><td>gi|6804607|ref|Os02g47520|</td></tr>
<tr><td>gi|55234990|gb|EAA00483.2|</td><td>lcl|M02D8.3 CE04759 WBGene00019729 status:Partially_confirmed SW:Q21461 protein_id:AAA82379.1 /QuerySize=915</td><td>gi|6804608|ref|Os02g47530|</td></tr>
<tr><td>gi|55235012|gb|EAA00807.2|</td><td>lcl|M02E1.1b CE31043 WBGene00019733 status:Partially_confirmed SW:Q95Q45 protein_id:AAM51500.1 /QuerySize=688</td><td>gi|6804610|ref|Os02g47550|</td></tr>
<tr><td>gi|55235018|gb|EAL38902.1|</td><td>lcl|M02E1.2 CE28616 WBGene00019734 status:Confirmed SW:Q95Q46 protein_id:AAK77619.1 /QuerySize=84</td><td>gi|6804619|ref|Os02g47630|</td></tr>
<tr><td>gi|55235019|gb|EAA00805.2|</td><td>lcl|M02E1.3 CE37132 WBGene00023458 status:Confirmed SW:Q6A6X0 protein_id:AAT92070.1 /QuerySize=19</td><td>gi|6804626|ref|Os02g47680|</td></tr>
<tr><td>gi|55235022|gb|EAA00804.2|</td><td>lcl|M02F4.1 CE04762 WBGene00019735 status:Predicted SW:Q21472 protein_id:AAA83204.1 /QuerySize=399</td><td>gi|6804629|ref|Os02g47710|</td></tr>
<tr><td>gi|55235028|gb|EAL38907.1|</td><td>lcl|M02F4.2 CE35744 WBGene00019736 status:Confirmed SW:Q21470 protein_id:AAA83202.2 /QuerySize=135</td><td>gi|6804632|ref|Os02g47740|</td></tr>
<tr><td>gi|55235029|gb|EAL38908.1|</td><td>lcl|M02F4.3 CE04764 WBGene00019737 status:Confirmed SW:Q21469 protein_id:AAA83201.1 /QuerySize=250</td><td>gi|6804640|ref|Os02g47820|</td></tr>
<tr><td>gi|55235036|gb|EAL38910.1|</td><td>lcl|M02F4.9 CE25059 WBGene00019739 status:Predicted SW:Q95ZR4 protein_id:AAK71379.1 /QuerySize=93</td><td>gi|6804641|ref|Os02g47830|</td></tr>
<tr><td>gi|55235037|gb|EAL38911.1|</td><td>lcl|M02G9.1 CE36944 WBGene00010830 keratin like protein status:Partially_confirmed SW:O17970 protein_id:CAB04625.2 /Quer</td><td>gi|6804649|ref|Os02g47910|</td></tr>
<tr><td>gi|55235040|gb|EAL38912.1|</td><td>lcl|M02G9.3 CE20017 WBGene00010832 status:Partially_confirmed SW:Q9XUS0 protein_id:CAB04626.1 /QuerySize=294</td><td>gi|6804651|ref|Os02g47930|</td></tr>
<tr><td>gi|55235041|gb|EAA00073.2|</td><td>lcl|M02H5.8 CE21073 WBGene00019744 status:Partially_confirmed SW:Q966I2 protein_id:AAK84558.1 /QuerySize=103</td><td>gi|6804653|ref|Os02g47950|</td></tr>
<tr><td>gi|55235047|gb|EAA00643.2|</td><td>lcl|M03A8.3 CE37133 WBGene00019749 status:Partially_confirmed /QuerySize=364</td><td>gi|6804654|ref|Os02g47960|</td></tr>
<tr><td>gi|55235051|gb|EAL38915.1|</td><td>lcl|M03A8.4 CE34940 WBGene00001572 locus:gei-15 status:Partially_confirmed SW:Q21482 protein_id:AAA83185.3 /QuerySize=</td><td>gi|6804657|ref|Os02g47990|</td></tr>
<tr><td>gi|55235053|gb|EAL38917.1|</td><td>lcl|M03B6.1 CE12334 WBGene00010833 status:Partially_confirmed SW:Q93895 protein_id:CAB01765.1 /QuerySize=150</td><td>gi|6804660|ref|Os02g48020|</td></tr>
<tr><td>gi|55235055|gb|EAL38918.1|</td><td>lcl|M03B6.3 CE23873 WBGene00010835 status:Confirmed SW:Q93894 protein_id:CAB54307.1 /QuerySize=642</td><td>gi|6804661|ref|Os02g48030|</td></tr>
<tr><td>gi|55235056|gb|EAL38919.1|</td><td>lcl|M03B6.4 CE12340 WBGene00010836 status:Partially_confirmed SW:Q93897 protein_id:CAB01767.1 /QuerySize=306</td><td>gi|6804662|ref|Os02g48040|</td></tr>
<tr><td>gi|55235060|gb|EAL38921.1|</td><td>lcl|M03B6.5 CE34941 WBGene00010837 status:Predicted SW:Q7YWZ5 protein_id:CAE17854.1 /QuerySize=106</td><td>gi|6804663|ref|Os02g48050|</td></tr>
<tr><td>gi|55235061|gb|EAL38922.1|</td><td>lcl|M03C11.3 CE03494 WBGene00010840 status:Partially_confirmed SW:Q21488 protein_id:CAA88958.1 /QuerySize=832</td><td>gi|6804665|ref|Os02g48070|</td></tr>
<tr><td>gi|55235070|gb|EAL38925.1|</td><td>lcl|M03C11.6 CE37782 WBGene00010843 status:Predicted SW:Q21485 protein_id:CAA88956.2 /QuerySize=300</td><td>gi|6804667|ref|Os02g48090|</td></tr>
<tr><td>gi|55235080|gb|EAA43333.2|</td><td>lcl|M03D4.1d CE31718 WBGene00006974 locus:zen-4 status:Partially_confirmed SW:Q8MQ03 protein_id:AAM97994.1 /QuerySiz</td><td>gi|6804674|ref|Os02g48160|</td></tr>
<tr><td>gi|55235092|gb|EAL38933.1|</td><td>lcl|M03D4.3 CE12350 WBGene00019750 status:Partially_confirmed SW:Q21491 protein_id:AAC24405.1 /QuerySize=59</td><td>gi|6804675|ref|Os02g48170|</td></tr>
<tr><td>gi|55235100|gb|EAA00613.2|</td><td>lcl|M03E7.2 CE07395 WBGene00019754 status:Partially_confirmed SW:Q21498 protein_id:AAB00725.1 /QuerySize=122</td><td>gi|6804680|ref|Os02g48220|</td></tr>
<tr><td>gi|55235112|gb|EAL38943.1|</td><td>lcl|M03E7.3 CE07396 WBGene00019755 status:Predicted SW:Q21497 protein_id:AAB00724.1 /QuerySize=110</td><td>gi|6804686|ref|Os02g48280|</td></tr>
<tr><td>gi|55235113|gb|EAA15061.2|</td><td>lcl|M03F4.4 CE12362 WBGene00019758 status:Predicted SW:Q23014 protein_id:AAB04574.1 /QuerySize=163</td><td>gi|6804699|ref|Os02g48400|</td></tr>
<tr><td>gi|55235117|gb|EAL38944.1|</td><td>lcl|M03F8.1 CE12372 WBGene00019761 status:Partially_confirmed SW:O16378 protein_id:AAB65908.1 /QuerySize=344</td><td>gi|6804703|ref|Os02g48440|</td></tr>
<tr><td>gi|55235165|gb|EAA14996.2|</td><td>lcl|M04B2.6 CE34942 WBGene00010848 status:Predicted SW:Q7YTL7 protein_id:CAE17885.1 /QuerySize=91</td><td>gi|6804707|ref|Os02g48480|</td></tr>
<tr><td>gi|55235170|gb|EAA14972.2|</td><td>lcl|M04B2.7 CE34943 WBGene00010849 status:Predicted SW:Q7YTL6 protein_id:CAE17886.1 /QuerySize=168</td><td>gi|6804708|ref|Os02g48490|</td></tr>
<tr><td>gi|55235171|gb|EAL38956.1|</td><td>lcl|M04C3.2 CE31973 WBGene00010851 status:Partially_confirmed SW:O62319 protein_id:CAB07266.2 /QuerySize=606</td><td>gi|6804709|ref|Os02g48500|</td></tr>
<tr><td>gi|55235174|gb|EAL38958.1|</td><td>lcl|M04C3.3 CE18080 WBGene00010852 status:Partially_confirmed SW:O62320 protein_id:CAB07267.1 /QuerySize=190</td><td>gi|6804710|ref|Os02g48510|</td></tr>
<tr><td>gi|55235175|gb|EAL38959.1|</td><td>lcl|M04C7.3 CE12398 WBGene00010854 status:Partially_confirmed SW:P91906 protein_id:CAB05569.1 /QuerySize=220</td><td>gi|6804711|ref|Os02g48520|</td></tr>
<tr><td>gi|55235185|gb|EAA14987.2|</td><td>lcl|M04C7.4 CE34944 WBGene00010855 status:Predicted SW:Q7YWX8 protein_id:CAE17876.1 /QuerySize=142</td><td>gi|6804712|ref|Os02g48530|</td></tr>
<tr><td>gi|55235187|gb|EAL38962.1|</td><td>lcl|M04C9.2 CE12402 WBGene00010857 status:Predicted SW:P90931 protein_id:CAB06020.1 /QuerySize=61</td><td>gi|6804713|ref|Os02g48540|</td></tr>
<tr><td>gi|55235188|gb|EAL38963.1|</td><td>lcl|M04C9.4 CE36656 WBGene00010859 status:Partially_confirmed SW:P90934 protein_id:CAB06023.2 /QuerySize=450</td><td>gi|6804714|ref|Os02g48550|</td></tr>
</table>

gi|55235189|gb|EAL38964.1|	lcl|M04D8.2 CE00577 WBGene00002105 locus:ins-22 status:Confirmed SW:Q21508 protein_id:CAA83611.1 /QuerySize=83	gi|6804717|ref|Os02g48580|
gi|55235190|gb|EAL38965.1|	lcl|M04D8.4 CE00622 WBGene00010862 status:Predicted SW:Q21509 protein_id:CAA83612.1 /QuerySize=218	gi|6804719|ref|Os02g48600|
gi|55235192|gb|EAA14970.3|	lcl|M04D8.5 CE00623 WBGene00010863 status:Partially_confirmed SW:Q21510 protein_id:CAA83613.1 /QuerySize=189	gi|6804728|ref|Os02g48690|
gi|55235193|gb|EAL38966.1|	lcl|M04D8.6 CE00579 WBGene00010864 status:Partially_confirmed SW:Q21511 protein_id:CAA83614.1 /QuerySize=200	gi|6804729|ref|Os02g48700|
gi|55235197|gb|EAL38967.1|	lcl|M04D8.7 CE00580 WBGene00010865 status:Partially_confirmed SW:Q21512 protein_id:CAA83615.1 /QuerySize=170	gi|6804730|ref|Os02g48710|
gi|55235203|gb|EAL38969.1|	lcl|M04D8.8 CE34945 WBGene00010866 status:Predicted SW:Q7YWX1 protein_id:CAE17888.1 /QuerySize=189	gi|6804735|ref|Os02g48750|
gi|55235205|gb|EAL38970.1|	lcl|M04F3.2 CE36357 WBGene00019768 status:Partially_confirmed SW:Q95Y98 protein_id:AAK71381.2 /QuerySize=255	gi|6804736|ref|Os02g48760|
gi|55235217|gb|EAA14762.2|	lcl|M04G12.4a CE36495 WBGene00010868 Zinc finger	gi|6804751|ref|Os02g48890|
gi|55235224|gb|EAL38976.1|	lcl|M04G12.4b CE36496 WBGene00010868 status:Partially_confirmed SW:Q8I4H1 protein_id:CAD56592.2 /QuerySize=550	gi|6804756|ref|Os02g48940|
gi|55235226|gb|EAL38977.1|	lcl|M05B5.3 CE28625 WBGene00010871 status:Partially_confirmed SW:Q21518 protein_id:CAA95835.2 /QuerySize=403	gi|6804764|ref|Os02g49020|
gi|55235228|gb|EAL38978.1|	lcl|M05B5.6 CE18081 WBGene00010873 status:Partially_confirmed SW:Q21517 protein_id:CAA95836.1 /QuerySize=481	gi|6804772|ref|Os02g49100|
gi|55235229|gb|EAL38979.1|	lcl|M05D6.6 CE03504 WBGene00010879 status:Confirmed SW:Q21525 protein_id:CAA91415.1 /QuerySize=161	gi|6804773|ref|Os02g49110|
gi|55235230|gb|EAL38980.1|	lcl|M05D6.9 CE34947 WBGene00010881 status:Predicted SW:Q7YWZ3 protein_id:CAE17856.1 /QuerySize=271	gi|6804775|ref|Os02g49130|
gi|55235232|gb|EAL38981.1|	lcl|M106.2 CE30680 WBGene00010910 status:Partially_confirmed SW:Q09592 protein_id:CAA87049.2 /QuerySize=519	gi|6804783|ref|Os02g49200|
gi|55235233|gb|EAA15004.2|	lcl|M110.8 CE34948 WBGene00010916 status:Predicted SW:Q7YTM0 protein_id:CAE17874.1 /QuerySize=91	gi|6804784|ref|Os02g49210|
gi|55235234|gb|EAA15003.2|	lcl|M110.9 CE34949 WBGene00010917 status:Predicted SW:Q7YTL9 protein_id:CAE17875.1 /QuerySize=144	gi|6804785|ref|Os02g49220|
gi|55235238|gb|EAA14990.2|	lcl|M116.1 CE37265 WBGene00019788 status:Confirmed SW:Q688B6 protein_id:AAU05543.1 /QuerySize=58	gi|6804791|ref|Os02g49240|
gi|55235241|gb|EAA15002.2|	lcl|M117.4 CE18085 WBGene00010920 status:Partially_confirmed SW:Q21536 protein_id:CAA98136.1 /QuerySize=662	gi|6804806|ref|Os02g49380|
gi|55235244|gb|EAL38983.1|	lcl|M151.1 CE36880 WBGene00019793 status:Partially_confirmed SW:O17121 protein_id:AAB70378.4 /QuerySize=402	gi|6804811|ref|Os02g49430|
gi|55235248|gb|EAL38985.1|	lcl|M151.2 CE12436 WBGene00019794 status:Predicted SW:O17119 protein_id:AAB70374.1 /QuerySize=675	gi|6804822|ref|Os02g49540|
gi|55235264|gb|EAL38990.1|	lcl|M151.3 CE12438 WBGene00019795 status:Predicted SW:O17118 protein_id:AAB70373.1 /QuerySize=836	gi|6804842|ref|Os02g49740|
gi|55235269|gb|EAL38991.1|	lcl|M151.4 CE28627 WBGene00019796 status:Predicted SW:O17117 protein_id:AAK77623.1 /QuerySize=591	gi|6804843|ref|Os02g49750|
gi|55235270|gb|EAL38992.1|	lcl|M151.7 CE28628 WBGene00019799 status:Partially_confirmed SW:Q965L8 protein_id:AAK77624.1 /QuerySize=416	gi|6804849|ref|Os02g49810|
gi|55235271|gb|EAL38993.1|	lcl|M153.3 CE03513 WBGene00010926 status:Predicted SW:Q21546 protein_id:CAA91945.1 /QuerySize=396	gi|6804859|ref|Os02g49910|
gi|55235272|gb|EAL38994.1|	lcl|M162.6 CE18896 WBGene00010932 status:Predicted SW:Q9XUD1 protein_id:CAB05252.1 /QuerySize=48	gi|6804862|ref|Os02g49940|
gi|55235273|gb|EAL38995.1|	lcl|M163.1 CE12446 WBGene00010935 status:Confirmed SW:Q93906 protein_id:CAB01897.1 /QuerySize=149	gi|6804870|ref|Os02g50020|
gi|55235279|gb|EAL38997.1|	lcl|M163.2 CE37393 WBGene00010936 Zinc finger	gi|6804871|ref|Os02g50030|
gi|55235280|gb|EAL38998.1|	lcl|M163.5 CE12454 WBGene00010937 status:Predicted SW:Q93900 protein_id:CAB01891.1 /QuerySize=435	gi|6804873|ref|Os02g50050|
gi|55235293|gb|EAL39000.1|	lcl|M163.6 CE12456 WBGene00010938 status:Predicted SW:Q93904 protein_id:CAB01895.1 /QuerySize=157	gi|6804875|ref|Os02g50070|
gi|55235294|gb|EAL39001.1|	lcl|M163.7 CE12458 WBGene00010939 status:Predicted SW:Q93905 protein_id:CAB01896.1 /QuerySize=259	gi|6804879|ref|Os02g50110|
gi|55235300|gb|EAA14932.2|	lcl|M176.10 CE28946 WBGene00010946 status:Partially_confirmed SW:Q8WQD9 protein_id:CAD21655.1 /QuerySize=454	gi|6804880|ref|Os02g50120|
gi|55235306|gb|EAL39004.1|	lcl|M176.11 CE33288 WBGene00010947 status:Partially_confirmed SW:Q86NF6 protein_id:CAD62041.1 /QuerySize=220	gi|6804886|ref|Os02g50180|
gi|55235307|gb|EAA15065.3|	lcl|M176.5 CE28947 WBGene00010944 status:Confirmed SW:Q21548 protein_id:CAB01654.2 /QuerySize=211	gi|6804894|ref|Os02g50250|
gi|55235310|gb|EAA14871.2|	lcl|M195.1 CE03514 WBGene00000653 locus:col-77 cuticle collagen status:Confirmed SW:Q21562 protein_id:CAA91290.1 /Query	gi|6804895|ref|Os02g50260|
gi|55235314|gb|EAL39006.1|	lcl|M195.2 CE34950 WBGene00010948 status:Partially_confirmed SW:Q21563 protein_id:CAA91291.2 /QuerySize=419	gi|6804899|ref|Os02g50300|
gi|55235316|gb|EAL39007.1|	lcl|M195.4 CE03517 WBGene00010949 status:Predicted SW:Q21564 protein_id:CAA91292.1 /QuerySize=142	gi|6804907|ref|Os02g50380|
gi|55235326|gb|EAL39010.1|	lcl|M199.3 CE20020 WBGene00010952 status:Predicted SW:Q9NAP1 protein_id:CAB70253.1 /QuerySize=67	gi|6804908|ref|Os02g50390|
gi|55235330|gb|EAL39013.1|	lcl|M199.8 CE34953 WBGene00010956 status:Predicted SW:Q7YTL2 protein_id:CAE17893.1 /QuerySize=54	gi|6804909|ref|Os02g50400|
gi|55235334|gb|EAL39015.1|	lcl|M28.10 CE32913 WBGene00010901 status:Confirmed SW:Q8I116 protein_id:CAD59151.1 /QuerySize=250	gi|6804910|ref|Os02g50410|
gi|55235341|gb|EAL39020.1|	lcl|M28.2 CE02281 WBGene00010894 status:Predicted SW:Q21572 protein_id:CAA90130.1 /QuerySize=681	gi|6804911|ref|Os02g50420|
gi|55235359|gb|EAA43368.2|	lcl|M28.4 CE32914 WBGene00010895 status:Partially_confirmed SW:Q21567 protein_id:CAA90126.2 /QuerySize=735	gi|6804912|ref|Os02g50430|
gi|55235382|gb|EAL39031.1|	lcl|M28.8 CE02286 WBGene00010899 status:Partially_confirmed SW:Q21574 protein_id:CAA90132.1 /QuerySize=831	gi|6804913|ref|Os02g50440|
gi|55235383|gb|EAL39032.1|	lcl|M28.9 CE02287 WBGene00010900 status:Partially_confirmed SW:Q21571 protein_id:CAA90129.1 /QuerySize=861	gi|6804914|ref|Os02g50450|
gi|55235396|gb|EAL39035.1|	lcl|M6.1c CE34062 WBGene00002056 locus:ifc-2 status:Partially_confirmed SW:Q7Z2A3 protein_id:AAP40524.1 /QuerySize=685	gi|6804916|ref|Os02g50470|
gi|55235397|gb|EAL39036.1|	lcl|M6.4 CE31206 WBGene00019776 status:Partially_confirmed SW:Q21577 protein_id:AAA98705.2 /QuerySize=194	gi|6804919|ref|Os02g50500|
gi|55235399|gb|EAL39037.1|	lcl|M60.4a CE34063 WBGene00019780 status:Confirmed SW:Q7Z2A7 protein_id:AAP40520.1 /QuerySize=162	gi|6804921|ref|Os02g50520|
gi|55235400|gb|EAL39038.1|	lcl|M60.4b CE02832 WBGene00019780 status:Confirmed SW:Q7Z2A6 protein_id:AAP40521.1 /QuerySize=139	gi|6804923|ref|Os02g50540|
gi|55235401|gb|EAA43373.2|	lcl|M60.6 CE31208 WBGene00019781 status:Partially_confirmed SW:Q9GYM6 protein_id:AAF99994.3 /QuerySize=147	gi|6804927|ref|Os02g50580|
gi|55235405|gb|EAA14943.2|	lcl|M70.2 CE17194 WBGene00019784 status:Predicted SW:O45186 protein_id:AAC04436.1 /QuerySize=198	gi|6804928|ref|Os02g50590|
gi|55235422|gb|EAL39041.1|	lcl|M79.2 CE03507 WBGene00010902 status:Predicted SW:Q19334 protein_id:CAA90692.1 /QuerySize=283	gi|6804934|ref|Os02g50650|
gi|55235426|gb|EAL39042.1|	lcl|M79.4 CE18890 WBGene00001462 locus:flp-19 status:Confirmed SW:Q9XVX1 protein_id:CAA90690.1 /QuerySize=89	gi|6804936|ref|Os02g50670|
gi|55235436|gb|EAA15025.2|	lcl|M88.2 CE23884 WBGene00010905 status:Confirmed SW:Q21601 protein_id:CAA84334.2 /QuerySize=228	gi|6804940|ref|Os02g50700|
gi|55235441|gb|EAA14704.2|	lcl|M88.3 CE01038 WBGene00010906 status:Partially_confirmed SW:Q21600 protein_id:CAA84333.1 /QuerySize=354	gi|6804941|ref|Os02g50710|
gi|55235442|gb|EAA15079.2|	lcl|MTCE.3 CE34072 WBGene00010957 NADH dehydrogenase ND6 status:Predicted /QuerySize=144	gi|6804942|ref|Os02g50720|
gi|55235443|gb|EAL39046.1|	lcl|MTCE.4 CE35354 WBGene00010958 NADH dehydrogenase ND4L status:Predicted /QuerySize=77	gi|6804945|ref|Os02g50750|
gi|55235444|gb|EAA15077.2|	lcl|R01B10.1b CE33149 WBGene00000534 locus:cli-1 status:Confirmed SW:Q86S25 protein_id:AAO21430.1 /QuerySize=82	gi|6804951|ref|Os02g50800|
gi|55235483|gb|EAL39053.1|	lcl|R01B10.2 CE18089 WBGene00019804 status:Predicted SW:O61972 protein_id:AAC17766.1 /QuerySize=65	gi|6804961|ref|Os02g50890|
gi|55235485|gb|EAA14679.2|	lcl|R01B10.3 CE18090 WBGene00019805 status:Partially_confirmed SW:O61974 protein_id:AAC17765.1 /QuerySize=98	gi|6804967|ref|Os02g50950|
gi|55235486|gb|EAA15083.2|	lcl|R01B10.6 CE30822 WBGene00019808 status:Partially_confirmed SW:Q8MXG1 protein_id:AAM45356.1 /QuerySize=274	gi|6804978|ref|Os02g51000|
gi|55235492|gb|EAL39055.1|	lcl|R01E6.5 CE03522 WBGene00010969 status:Confirmed SW:Q21616 protein_id:CAA92185.1 /QuerySize=245	gi|6804983|ref|Os02g51050|
gi|55235498|gb|EAL39057.1|	lcl|R01E6.7 CE03524 WBGene00010971 status:Partially_confirmed SW:Q21620 protein_id:CAA92189.1 /QuerySize=245	gi|6804995|ref|Os02g51170|
gi|55235503|gb|EAL39059.1|	lcl|R01H10.4 CE00591 WBGene00010972 status:Partially_confirmed SW:Q21622 protein_id:CAA83462.1 /QuerySize=245	gi|6804999|ref|Os02g51210|
gi|55235512|gb|EAL39060.1|	lcl|R01H10.5 CE20023 WBGene00010973 status:Partially_confirmed SW:Q21621 protein_id:CAA83464.1 /QuerySize=209	gi|6805004|ref|Os02g51260|
gi|55235518|gb|EAL39062.1|	lcl|R01H2.1 CE00728 WBGene00019809 status:Predicted SW:Q21631 protein_id:AAA50643.1 /QuerySize=104	gi|6805005|ref|Os02g51270|
gi|55235547|gb|EAL39071.1|	lcl|R01H2.4 CE00803 WBGene00019812 status:Partially_confirmed SW:Q21630 protein_id:AAA50646.1 /QuerySize=222	gi|6805012|ref|Os02g51340|
gi|55235558|gb|EAL39073.1|	lcl|R01H2.7 CE36881 WBGene00023414 status:Predicted SW:Q6F6K6 protein_id:AAT48619.1 /QuerySize=178	gi|6805014|ref|Os02g51360|
gi|55235560|gb|EAL39074.1|	lcl|R02D3.7 CE18101 WBGene00019824 status:Partially_confirmed SW:O44505 protein_id:AAB94147.1 /QuerySize=479	gi|6805016|ref|Os02g51380|
gi|55235579|gb|EAL39080.1|	lcl|R02D5.1 CE12486 WBGene00010976 status:Confirmed SW:Q21636 protein_id:CAB01433.1 /QuerySize=206	gi|6805017|ref|Os02g51390|
gi|55235609|gb|EAA14908.2|	lcl|R02D5.3 CE12490 WBGene00010977 status:Partially_confirmed SW:Q21635 protein_id:CAB01432.1 /QuerySize=351	gi|6805031|ref|Os02g51510|
gi|55235626|gb|EAA14904.2|	lcl|R02D5.4 CE12492 WBGene00010978 status:Predicted SW:Q21634 protein_id:CAB01431.1 /QuerySize=97	gi|6805036|ref|Os02g51560|
gi|55235632|gb|EAL39091.1|	lcl|R02D5.7 CE29435 WBGene00010980 status:Partially_confirmed SW:O62323 protein_id:CAB01436.2 /QuerySize=149	gi|6805038|ref|Os02g51580|
gi|55235635|gb|EAL39092.1|	lcl|R02E12.5 CE30292 WBGene00019829 status:Partially_confirmed SW:Q21640 protein_id:AAA96183.2 /QuerySize=87	gi|6805044|ref|Os02g51630|
gi|55235642|gb|EAL39094.1|	lcl|R02E12.6 CE30099 WBGene00019830 status:Partially_confirmed SW:Q21642 protein_id:AAA96185.2 /QuerySize=194	gi|6805045|ref|Os02g51640|
gi|55235652|gb|EAL39099.1|	lcl|R02E4.1 CE04777 WBGene00019826 status:Partially_confirmed /QuerySize=474	gi|6805046|ref|Os02g51650|
gi|55235655|gb|EAA14830.3|	lcl|R02E4.2 CE37924 WBGene00044021 status:Predicted /QuerySize=253	gi|6805053|ref|Os02g51720|
gi|55235665|gb|EAA14640.3|	lcl|R02F11.1 CE12496 WBGene00019839 status:Confirmed SW:O16364 protein_id:AAB65897.1 /QuerySize=176	gi|6805071|ref|Os02g51870|
gi|55235669|gb|EAL39101.1|	lcl|R02F2.5 CE00684 WBGene00019834 status:Partially_confirmed SW:Q21648 protein_id:AAA50721.1 /QuerySize=223	gi|6805076|ref|Os02g51920|
gi|55235676|gb|EAL39104.1|	lcl|R02F2.6 CE00770 WBGene00019835 status:Partially_confirmed SW:Q21647 protein_id:AAA50722.1 /QuerySize=80	gi|6805083|ref|Os02g51990|
gi|55235677|gb|EAA14634.2|	lcl|R02F2.7 CE29818 WBGene00019836 status:Partially_confirmed SW:Q21649 protein_id:AAA50723.3 /QuerySize=693	gi|6805087|ref|Os02g52030|
gi|55235678|gb|EAL39105.1|	lcl|R03A10.1 CE06207 WBGene00010981 status:Partially_confirmed SW:Q21655 protein_id:CAA93670.1 /QuerySize=126	gi|6805096|ref|Os02g52120|
gi|55235682|gb|EAA14665.2|	lcl|R03A10.2 CE06208 WBGene00010982 status:Predicted SW:Q21656 protein_id:CAA93671.1 /QuerySize=136	gi|6805102|ref|Os02g52170|
gi|55235741|gb|EAL39117.1|	lcl|R03C1.1 CE35358 WBGene00010987 status:Partially_confirmed SW:O17977 protein_id:CAB05259.2 /QuerySize=321	gi|6805105|ref|Os02g52200|
gi|55235746|gb|EAA14367.2|	lcl|R03E9.2 CE04785 WBGene00019843 status:Partially_confirmed SW:Q21662 protein_id:AAC48068.1 /QuerySize=610	gi|6805110|ref|Os02g52240|
gi|55235748|gb|EAL39119.1|	lcl|R03G5.6 CE07403 WBGene00019847 status:Partially_confirmed SW:Q21668 protein_id:AAA96066.1 /QuerySize=76	gi|6805117|ref|Os02g52300|
gi|55235750|gb|EAL39120.1|	lcl|R03G5.7 CE31212 WBGene00019848 status:Confirmed SW:Q8MQ01 protein_id:AAM69099.1 /QuerySize=78	gi|6805118|ref|Os02g52310|
gi|55235757|gb|EAL39121.1|	lcl|R03G8.2 CE03526 WBGene00010997 reverse transcriptase status:Predicted SW:Q21670 protein_id:CAA93677.1 /QuerySize=:	gi|6805120|ref|Os02g52330|
gi|55235760|gb|EAA14411.2|	lcl|R03H10.1 CE02837 WBGene00019854 status:Predicted SW:Q9GYL7 protein_id:AAF99998.1 /QuerySize=379	gi|6805122|ref|Os02g52350|
gi|55235761|gb|EAL39122.1|	lcl|R03H10.2 CE25965 WBGene00019855 status:Confirmed SW:Q9GYM4 protein_id:AAG00006.1 /QuerySize=382	gi|6805125|ref|Os02g52380|
gi|55235765|gb|EAL39124.1|	lcl|R04A9.1 CE04788 WBGene00019861 status:Predicted SW:Q21695 protein_id:AAA83288.1 /QuerySize=103	gi|6805127|ref|Os02g52400|
gi|55235797|gb|EAL39129.1|	lcl|R04A9.3 CE35747 WBGene00019863 status:Confirmed SW:Q21692 protein_id:AAA83285.2 /QuerySize=301	gi|6805134|ref|Os02g52440|
gi|55235806|gb|EAA14008.2|	lcl|R04A9.6 CE30336 WBGene00019865 status:Partially_confirmed SW:Q95QB9 protein_id:AAL06046.2 /QuerySize=135	gi|6805143|ref|Os02g52500|
gi|55235817|gb|EAL39135.1|	lcl|R04B3.1 CE37659 WBGene00019866 status:Predicted SW:Q21696 protein_id:AAA91259.2 /QuerySize=529	gi|6805150|ref|Os02g52570|
gi|55235819|gb|EAL39137.1|	lcl|R04B3.3 CE23890 WBGene00019868 status:Partially_confirmed SW:Q21698 protein_id:AAA91261.2 /QuerySize=503	gi|6805153|ref|Os02g52600|
gi|55235829|gb|EAA14083.3|	lcl|R04B5.1 CE06212 WBGene00011001 status:Predicted SW:Q21699 protein_id:CAA94838.1 /QuerySize=86	gi|6805155|ref|Os02g52620|
gi|55235831|gb|EAA43469.2|	lcl|R04B5.11 CE31975 WBGene00011008 status:Partially_confirmed SW:Q21708 protein_id:CAA94847.2 /QuerySize=87	gi|6805159|ref|Os02g52660|
gi|55235838|gb|EAA13888.2|	lcl|R04B5.7 CE06217 WBGene00011005 Weak similarity to PAB-dependent poly(A)-specific ribonuclease (Swiss Prot accession n	gi|6805169|ref|Os02g52760|
gi|55235839|gb|EAA14075.2|	lcl|R04D3.2 CE06223 WBGene00011010 status:Partially_confirmed SW:Q21712 protein_id:CAA94162.1 /QuerySize=406	gi|6805183|ref|Os02g52890|
gi|55235846|gb|EAL39142.1|	lcl|R04D3.3 CE06224 WBGene00011011 status:Partially_confirmed SW:Q21713 protein_id:CAA94163.1 /QuerySize=430	gi|6805191|ref|Os02g52950|
gi|55235847|gb|EAA13969.3|	lcl|R04D3.4 CE06225 WBGene00011012 status:Confirmed SW:Q21711 protein_id:CAA94161.1 /QuerySize=226	gi|6805197|ref|Os02g53010|
gi|55235851|gb|EAL39144.1|	lcl|R04E5.7 CE35748 WBGene00019870 status:Partially_confirmed SW:Q9GYL5 protein_id:AAG00009.2 /QuerySize=118	gi|6805205|ref|Os02g53080|
gi|55235852|gb|EAA14130.3|	lcl|R04E5.8a CE04800 WBGene00019871 status:Partially_confirmed SW:Q9GYL4 protein_id:AAG00010.1 /QuerySize=997	gi|6805206|ref|Os02g53080|
gi|55235853|gb|EAL39145.1|	lcl|R04E5.8b CE33934 WBGene00019871 status:Confirmed SW:Q86DC5 protein_id:AAP31431.1 /QuerySize=142	gi|6805207|ref|Os02g53090|
gi|55235861|gb|EAA14137.3|	lcl|R04E5.9 CE04801 WBGene00019872 status:Partially_confirmed SW:Q9GYL3 protein_id:AAG00011.1 /QuerySize=175	gi|6805215|ref|Os02g53170|
gi|55235870|gb|EAL39148.1|	lcl|R04F11.3 CE29598 WBGene00011016 status:Confirmed SW:Q21730 protein_id:CAA98958.4 /QuerySize=385	gi|6805219|ref|Os02g53190|
gi|55235871|gb|EAA14142.2|	lcl|R05A10.1 CE36884 WBGene00011018 status:Partially_confirmed SW:O45699 protein_id:CAB05263.3 /QuerySize=415	gi|6805222|ref|Os02g53220|
gi|55235883|gb|EAL39151.1|	lcl|R05A10.2 CE16288 WBGene00011019 status:Partially_confirmed SW:O45698 protein_id:CAB05262.1 /QuerySize=161	gi|6805224|ref|Os02g53240|
gi|55235886|gb|EAL39153.1|	lcl|R05A10.3 CE16289 WBGene00011020 status:Partially_confirmed SW:O45701 protein_id:CAB05265.1 /QuerySize=387	gi|6805225|ref|Os02g53250|
gi|55235891|gb|EAL39156.1|	lcl|R05D11.8 CE06243 WBGene00011036 status:Partially_confirmed SW:Q21740 protein_id:CAA99895.1 /QuerySize=566	gi|6805230|ref|Os02g53290|
gi|55235892|gb|EAL39157.1|	lcl|R05D11.9 CE32474 WBGene00011037 status:Partially_confirmed SW:Q95ZR2 protein_id:CAC42331.2 /QuerySize=640	gi|6805231|ref|Os02g53300|
gi|55235894|gb|EAL39158.1|	lcl|R05D3.5 CE28272 WBGene00019879 status:Predicted /QuerySize=60	gi|6805232|ref|Os02g53310|
gi|55235908|gb|EAL39168.1|	lcl|R05D3.8 CE00287 WBGene00019881 status:Confirmed SW:P34541 protein_id:AAK21440.1 /QuerySize=138	gi|6805236|ref|Os02g53350|
gi|55235910|gb|EAL39170.1|	lcl|R05D7.1 CE32475 WBGene00011026 status:Confirmed SW:Q9XVK8 protein_id:CAB03215.2 /QuerySize=142	gi|6805238|ref|Os02g53370|
gi|55235912|gb|EAL39172.1|	lcl|R05D7.5 CE34492 WBGene00011030 status:Partially_confirmed SW:O45708 protein_id:CAB03218.2 /QuerySize=331	gi|6805240|ref|Os02g53390|
gi|55235920|gb|EAL39177.1|	lcl|R05F9.11 CE04814 WBGene00019894 A. thaliana ELI3-2 protein status:Predicted SW:Q21749 protein_id:AAA83173.1 /QueryS	gi|6805242|ref|Os02g53410|
gi|55235930|gb|EAL39181.1|	lcl|R05F9.1d CE36498 WBGene00019888 status:Partially_confirmed SW:Q7JP41 protein_id:AAS60256.1 /QuerySize=155	gi|6805245|ref|Os02g53440|
gi|55235934|gb|EAA14232.2|	lcl|R05F9.7 CE04810 WBGene00019891 status:Predicted SW:Q21741 protein_id:AAA83162.1 /QuerySize=286	gi|6805246|ref|Os02g53450|
gi|55235958|gb|EAL39186.1|	lcl|R05F9.9 CE04812 WBGene00019892 status:Partially_confirmed SW:Q21745 protein_id:AAA83169.1 /QuerySize=256	gi|6805247|ref|Os02g53460|
gi|55235959|gb|EAA14518.3|	lcl|R05G6.1 CE07414 WBGene00019896 status:Partially_confirmed SW:Q21760 protein_id:AAB00630.1 /QuerySize=314	gi|6805249|ref|Os02g53480|
gi|55235960|gb|EAA14515.3|	lcl|R05G9.1 CE21110 WBGene00019903 status:Predicted SW:Q966H0 protein_id:AAK68391.1 /QuerySize=197	gi|6805253|ref|Os02g53510|
gi|55235962|gb|EAA14486.2|	lcl|R05G9.3 CE21108 WBGene00019905 status:Partially_confirmed SW:Q966H1 protein_id:AAK68393.1 /QuerySize=212	gi|6805259|ref|Os02g53570|
gi|55235966|gb|EAA14503.2|	lcl|R05G9R.1 CE36001 WBGene00019906 status:Partially_confirmed SW:Q965Y7 protein_id:AAK68394.2 /QuerySize=763	gi|6805260|ref|Os02g53580|
gi|55235972|gb|EAA14519.3|	lcl|R05H10.3a CE25970 WBGene00011044 status:Partially_confirmed SW:O62326 protein_id:CAC14398.1 /QuerySize=591	gi|6805261|ref|Os02g53590|
gi|55235976|gb|EAA14505.3|	lcl|R05H10.3b CE37034 WBGene00011044 status:Partially_confirmed SW:Q6EUT6 protein_id:CAH04736.1 /QuerySize=504	gi|6805268|ref|Os02g53660|
gi|55235978|gb|EAL39190.1|	lcl|R05H11.2 CE32703 WBGene00019908 status:Predicted SW:Q8IFZ7 protein_id:AAN65309.1 /QuerySize=105	gi|6805274|ref|Os02g53710|
gi|55235981|gb|EAL39192.1|	lcl|R05H5.7 CE02294 WBGene00011041 status:Partially_confirmed SW:Q21766 protein_id:CAA88730.1 /QuerySize=203	gi|6805276|ref|Os02g53730|
gi|55235995|gb|EAA14309.2|	lcl|R06A10.1 CE36707 WBGene00019909 status:Predicted SW:O44746 protein_id:AAB96732.2 /QuerySize=171	gi|6805280|ref|Os02g53760|
gi|55236004|gb|EAL39200.1|	lcl|R06A10.3 CE12516 WBGene00019910 status:Predicted SW:O44745 protein_id:AAB96731.1 /QuerySize=158	gi|6805285|ref|Os02g53800|
gi|55236018|gb|EAL39206.1|	lcl|R06A4.2 CE18109 WBGene00011047 status:Partially_confirmed SW:O62329 protein_id:CAB05583.1 /QuerySize=837	gi|6805288|ref|Os02g53830|
gi|55236033|gb|EAA14187.3|	lcl|R06B10.7 CE32916 WBGene00019916 status:Confirmed SW:Q8IA65 protein_id:AAN84806.1 /QuerySize=34	gi|6805292|ref|Os02g53870|
gi|55236036|gb|EAL39210.1|	lcl|R06B9.5 CE23900 WBGene00011056 status:Partially_confirmed SW:Q9U397 protein_id:CAB54283.1 /QuerySize=91	gi|6805293|ref|Os02g53880|
gi|55236046|gb|EAA14198.2|	lcl|R06C1.6 CE37541 WBGene00011060 status:Partially_confirmed SW:O62338 protein_id:CAD45609.2 /QuerySize=432	gi|6805294|ref|Os02g53890|
gi|55236047|gb|EAL39213.1|	lcl|R06C7.2 CE36661 WBGene00011062 status:Partially_confirmed SW:Q21772 protein_id:CAA95841.2 /QuerySize=233	gi|6805295|ref|Os02g53900|
gi|55236070|gb|EAL39217.1|	lcl|R06C7.4 CE06247 WBGene00011063 status:Partially_confirmed SW:Q21771 protein_id:CAA95840.1 /QuerySize=292	gi|6805297|ref|Os02g53920|
gi|55236075|gb|EAL39219.1|	lcl|R06F6.11 CE01617 WBGene00011072 locus:tag-209 status:Partially_confirmed SW:Q09418 protein_id:CAA86781.1 /QuerySiz	gi|6805300|ref|Os02g53950|

<table>
<tr><td>gi|55236077|gb|EAA43545.2|</td><td>lcl|R06F6.12 CE36945 WBGene00023418 status:Partially_confirmed SW:Q6BEV5 protein_id:CAH04745.1 /QuerySize=417</td><td>gi|6805309|ref|Os02g54040|</td></tr>
<tr><td>gi|55236092|gb|EAL39224.1|</td><td>lcl|R06F6.7 CE01623 WBGene00011070 status:Partially_confirmed SW:Q09416 protein_id:CAA86777.1 /QuerySize=120</td><td>gi|6805316|ref|Os02g54080|</td></tr>
<tr><td>gi|55236125|gb|EAA14104.2|</td><td>lcl|R07A4.2 CE03531 WBGene00011073 status:Partially_confirmed SW:Q21780 protein_id:CAA91762.1 /QuerySize=157</td><td>gi|6805317|ref|Os02g54080|</td></tr>
<tr><td>gi|55236130|gb|EAL39230.1|</td><td>lcl|R07A4.3 CE03532 WBGene00011074 status:Partially_confirmed SW:Q21781 protein_id:CAA91763.1 /QuerySize=295</td><td>gi|6805319|ref|Os02g54100|</td></tr>
<tr><td>gi|55236131|gb|EAL39231.1|</td><td>lcl|R07B1.11 CE34956 WBGene00011082 status:Confirmed SW:Q7YWU6 protein_id:CAE17931.1 /QuerySize=120</td><td>gi|6805335|ref|Os02g54230|</td></tr>
<tr><td>gi|55236132|gb|EAL39232.1|</td><td>lcl|R07B1.7 CE01633 WBGene00011079 status:Predicted SW:Q09421 protein_id:CAA88544.1 /QuerySize=238</td><td>gi|6805342|ref|Os02g54300|</td></tr>
<tr><td>gi|55236139|gb|EAA14038.2|</td><td>lcl|R07B1.9 CE01635 WBGene00011081 status:Partially_confirmed SW:Q09609 protein_id:CAA88546.1 /QuerySize=770</td><td>gi|6805343|ref|Os02g54310|</td></tr>
<tr><td>gi|55236151|gb|EAA14107.2|</td><td>lcl|R07B5.3 CE34582 WBGene00005703 locus:sru-40 status:Predicted SW:Q21784 protein_id:CAA96663.2 /QuerySize=326</td><td>gi|6805344|ref|Os02g54320|</td></tr>
<tr><td>gi|55236152|gb|EAA14122.3|</td><td>lcl|R07B5.4 CE06258 WBGene00005701 locus:sru-38 status:Partially_confirmed SW:Q21785 protein_id:CAA96664.1 /QuerySize=</td><td>gi|6805345|ref|Os02g54330|</td></tr>
<tr><td>gi|55236155|gb|EAA14082.2|</td><td>lcl|R07B5.9 CE31575 WBGene00011086 status:Partially_confirmed SW:Q8MPZ9 protein_id:CAD44143.1 /QuerySize=660</td><td>gi|6805348|ref|Os02g54350|</td></tr>
<tr><td>gi|55236175|gb|EAL39239.1|</td><td>lcl|R07B7.2 CE28841 WBGene00011087 status:Partially_confirmed SW:Q95QM1 protein_id:CAB00127.2 /QuerySize=651</td><td>gi|6805351|ref|Os02g54380|</td></tr>
<tr><td>gi|55236184|gb|EAL39242.1|</td><td>lcl|R07B7.3 CE06265 WBGene00004138 locus:pqn-53 status:Confirmed SW:Q21793 protein_id:CAB00112.1 /QuerySize=355</td><td>gi|6805359|ref|Os02g54460|</td></tr>
<tr><td>gi|55236194|gb|EAA13976.2|</td><td>lcl|R07B7.7 CE06269 WBGene00011091 status:Partially_confirmed SW:Q21797 protein_id:CAB00116.1 /QuerySize=110</td><td>gi|6805375|ref|Os02g54610|</td></tr>
<tr><td>gi|55236195|gb|EAL39244.1|</td><td>lcl|R07C12.1 CE17204 WBGene00019931 status:Partially_confirmed SW:O44525 protein_id:AAB94150.1 /QuerySize=337</td><td>gi|6805377|ref|Os02g54630|</td></tr>
<tr><td>gi|55236200|gb|EAL39245.1|</td><td>lcl|R07C12.2 CE33679 WBGene00019932 status:Confirmed SW:O44524 protein_id:AAL06047.2 /QuerySize=177</td><td>gi|6805380|ref|Os02g54660|</td></tr>
<tr><td>gi|55236205|gb|EAA14148.2|</td><td>lcl|R07C12.3 CE17206 WBGene00019933 status:Predicted SW:O44523 protein_id:AAB94152.1 /QuerySize=358</td><td>gi|6805381|ref|Os02g54670|</td></tr>
<tr><td>gi|55236208|gb|EAA13866.2|</td><td>lcl|R07C12.4 CE29445 WBGene00019934 status:Partially_confirmed SW:Q95X68 protein_id:AAL06048.1 /QuerySize=254</td><td>gi|6805382|ref|Os02g54680|</td></tr>
<tr><td>gi|55236209|gb|EAL39247.1|</td><td>lcl|R07C3.10 CE32706 WBGene00019926 status:Partially_confirmed SW:O16766 protein_id:AAB66239.2 /QuerySize=399</td><td>gi|6805390|ref|Os02g54750|</td></tr>
<tr><td>gi|55236210|gb|EAA43579.2|</td><td>lcl|R07C3.11 CE12542 WBGene00019927 status:Partially_confirmed SW:O16765 protein_id:AAB66240.1 /QuerySize=399</td><td>gi|6805395|ref|Os02g54800|</td></tr>
<tr><td>gi|55236211|gb|EAA43581.2|</td><td>lcl|R07C3.13 CE12546 WBGene00019929 status:Predicted SW:O16772 protein_id:AAB66242.1 /QuerySize=300</td><td>gi|6805396|ref|Os02g54810|</td></tr>
<tr><td>gi|55236228|gb|EAA14076.3|</td><td>lcl|R07C3.14 CE12548 WBGene00019930 status:Predicted SW:O16773 protein_id:AAB66243.1 /QuerySize=172</td><td>gi|6805401|ref|Os02g54840|</td></tr>
<tr><td>gi|55236233|gb|EAL39249.1|</td><td>lcl|R07C3.2 CE12524 WBGene00019918 status:Partially_confirmed SW:O16777 protein_id:AAB66232.1 /QuerySize=288</td><td>gi|6805402|ref|Os02g54850|</td></tr>
<tr><td>gi|55236234|gb|EAA14129.3|</td><td>lcl|R07C3.5 CE12530 WBGene00019921 status:Partially_confirmed SW:O16774 protein_id:AAB66235.1 /QuerySize=205</td><td>gi|6805405|ref|Os02g54870|</td></tr>
<tr><td>gi|55236251|gb|EAL39257.1|</td><td>lcl|R07C3.6 CE12532 WBGene00019922 status:Partially_confirmed SW:O16771 protein_id:AAB66236.1 /QuerySize=388</td><td>gi|6805410|ref|Os02g54920|</td></tr>
<tr><td>gi|55236256|gb|EAL39259.1|</td><td>lcl|R07C3.7 CE12534 WBGene00019923 status:Partially_confirmed SW:O16769 protein_id:AAB66237.1 /QuerySize=825</td><td>gi|6805411|ref|Os02g54930|</td></tr>
<tr><td>gi|55236270|gb|EAA14268.2|</td><td>lcl|R07C3.8 CE12536 WBGene00019924 status:Partially_confirmed SW:O16768 protein_id:AAB66238.1 /QuerySize=381</td><td>gi|6805412|ref|Os02g54940|</td></tr>
<tr><td>gi|55236290|gb|EAL39267.1|</td><td>lcl|R07C3.9 CE12538 WBGene00019925 status:Confirmed SW:O16767 protein_id:AAB66230.1 /QuerySize=288</td><td>gi|6805415|ref|Os02g54970|</td></tr>
<tr><td>gi|55236298|gb|EAL39269.1|</td><td>lcl|R07E3.2 CE02296 WBGene00011103 status:Predicted SW:Q21814 protein_id:CAA89074.1 /QuerySize=242</td><td>gi|6805424|ref|Os02g55040|</td></tr>
<tr><td>gi|55236303|gb|EAA14365.2|</td><td>lcl|R07E3.6 CE02300 WBGene00011107 status:Partially_confirmed SW:Q21811 protein_id:CAA89071.1 /QuerySize=927</td><td>gi|6805429|ref|Os02g55090|</td></tr>
<tr><td>gi|55236309|gb|EAL39272.1|</td><td>lcl|R07E3.7 CE02301 WBGene00011108 status:Partially_confirmed SW:Q21813 protein_id:CAA89073.1 /QuerySize=195</td><td>gi|6805430|ref|Os02g55100|</td></tr>
<tr><td>gi|55236323|gb|EAA14015.2|</td><td>lcl|R07E4.5 CE04820 WBGene00019938 status:Partially_confirmed SW:Q21816 protein_id:AAA80404.1 /QuerySize=817</td><td>gi|6805436|ref|Os02g55160|</td></tr>
<tr><td>gi|55236324|gb|EAA14044.2|</td><td>lcl|R07E5.11 CE00669 WBGene00011117 status:Partially_confirmed SW:Q21822 protein_id:CAA83618.1 /QuerySize=113</td><td>gi|6805440|ref|Os02g55190|</td></tr>
<tr><td>gi|55236362|gb|EAA13977.3|</td><td>lcl|R07E5.12 CE03534 WBGene00011118 status:Confirmed SW:Q21833 protein_id:CAA83627.1 /QuerySize=102</td><td>gi|6805445|ref|Os02g55240|</td></tr>
<tr><td>gi|55236365|gb|EAA13978.2|</td><td>lcl|R07E5.15 CE18126 WBGene00011120 status:Confirmed SW:O62342 protein_id:CAA83628.1 /QuerySize=253</td><td>gi|6805457|ref|Os02g55360|</td></tr>
<tr><td>gi|55236366|gb|EAL39284.1|</td><td>lcl|R07E5.17 CE34958 WBGene00011121 status:Predicted SW:Q7YWU4 protein_id:CAE17933.1 /QuerySize=361</td><td>gi|6805464|ref|Os02g55430|</td></tr>
<tr><td>gi|55236372|gb|EAA43624.2|</td><td>lcl|R07E5.6 CE18124 WBGene00011114 Coiled-coil protein status:Partially_confirmed SW:Q21823 protein_id:CAA83629.1 /Query</td><td>gi|6805466|ref|Os02g55450|</td></tr>
<tr><td>gi|55236381|gb|EAA14045.3|</td><td>lcl|R07E5.7 CE00672 WBGene00011115 status:Confirmed SW:Q21821 protein_id:CAA83617.1 /QuerySize=191</td><td>gi|6805467|ref|Os02g55460|</td></tr>
<tr><td>gi|55236395|gb|EAL39292.1|</td><td>lcl|R07G3.6 CE02025 WBGene00019942 status:Confirmed SW:Q09423 protein_id:AAK31546.1 /QuerySize=304</td><td>gi|6805471|ref|Os02g55500|</td></tr>
<tr><td>gi|55236406|gb|EAL39293.1|</td><td>lcl|R07G3.7a CE36004 WBGene00019943 status:Confirmed SW:Q09424 protein_id:AAR12972.1 /QuerySize=366</td><td>gi|6805472|ref|Os02g55510|</td></tr>
<tr><td>gi|55236441|gb|EAA13943.2|</td><td>lcl|R07G3.7b CE36005 WBGene00019943 status:Confirmed SW:Q09424 protein_id:AAR12973.1 /QuerySize=231</td><td>gi|6805480|ref|Os02g55580|</td></tr>
<tr><td>gi|55236452|gb|EAL39305.1|</td><td>lcl|R07H5.11 CE30681 WBGene00011131 status:Confirmed SW:Q8MYP7 protein_id:CAD31662.1 /QuerySize=119</td><td>gi|6805482|ref|Os02g55600|</td></tr>
<tr><td>gi|55236456|gb|EAA14301.3|</td><td>lcl|R07H5.4 CE12556 WBGene00011124 status:Confirmed SW:Q93932 protein_id:CAB03227.1 /QuerySize=206</td><td>gi|6805483|ref|Os02g55610|</td></tr>
<tr><td>gi|55236466|gb|EAL39310.1|</td><td>lcl|R07H5.9 CE30682 WBGene00011129 status:Partially_confirmed SW:Q93935 protein_id:CAB03231.2 /QuerySize=287</td><td>gi|6805487|ref|Os02g55650|</td></tr>
<tr><td>gi|55236472|gb|EAL39313.1|</td><td>lcl|R08A2.5 CE23906 WBGene00011136 status:Partially_confirmed SW:Q9U394 protein_id:CAB57903.1 /QuerySize=290</td><td>gi|6805488|ref|Os02g55660|</td></tr>
<tr><td>gi|55236475|gb|EAA14295.2|</td><td>lcl|R08A2.7 CE34959 WBGene00011137 status:Predicted SW:Q7YWU8 protein_id:CAE17929.1 /QuerySize=128</td><td>gi|6805489|ref|Os02g55670|</td></tr>
<tr><td>gi|55236518|gb|EAL39325.1|</td><td>lcl|R08B4.3 CE20025 WBGene00011139 status:Confirmed SW:Q9XVW5 protein_id:CAA92002.1 /QuerySize=154</td><td>gi|6805491|ref|Os02g55690|</td></tr>
<tr><td>gi|55236519|gb|EAL39326.1|</td><td>lcl|R08B4.4 CE31577 WBGene00011140 status:Partially_confirmed SW:Q8MPZ8 protein_id:CAD44147.1 /QuerySize=142</td><td>gi|6805492|ref|Os02g55700|</td></tr>
<tr><td>gi|55236528|gb|EAL39328.1|</td><td>lcl|R08B4.5 CE34960 WBGene00011141 status:Predicted SW:Q7YWW1 protein_id:CAE17909.1 /QuerySize=97</td><td>gi|6805493|ref|Os02g55710|</td></tr>
<tr><td>gi|55236535|gb|EAA13706.2|</td><td>lcl|R08C7.1 CE07424 WBGene00019945 status:Partially_confirmed SW:Q21845 protein_id:AAC48072.1 /QuerySize=47</td><td>gi|6805495|ref|Os02g55730|</td></tr>
<tr><td>gi|55236536|gb|EAL39331.1|</td><td>lcl|R08C7.11 CE07434 WBGene00019954 status:Predicted SW:Q21846 protein_id:AAC48079.1 /QuerySize=54</td><td>gi|6805496|ref|Os02g55740|</td></tr>
<tr><td>gi|55236537|gb|EAL39332.1|</td><td>lcl|R08E3.3a CE33809 WBGene00019959 status:Partially_confirmed SW:Q21848 protein_id:AAC48196.2 /QuerySize=455</td><td>gi|6805497|ref|Os02g55750|</td></tr>
<tr><td>gi|55236560|gb|EAL39339.1|</td><td>lcl|R08E3.3b CE35751 WBGene00019959 status:Confirmed SW:Q7JP60 protein_id:AAQ82846.1 /QuerySize=516</td><td>gi|6805498|ref|Os02g55760|</td></tr>
<tr><td>gi|55236573|gb|EAL39344.1|</td><td>lcl|R08E5.4 CE12578 WBGene00019964 status:Partially_confirmed SW:O01595 protein_id:AAB52278.1 /QuerySize=94</td><td>gi|6805499|ref|Os02g55770|</td></tr>
<tr><td>gi|55236600|gb|EAL39354.1|</td><td>lcl|R08H2.8 CE12608 WBGene00011149 status:Predicted SW:O17995 protein_id:CAB04638.1 /QuerySize=367</td><td>gi|6805501|ref|Os02g55790|</td></tr>
<tr><td>gi|55236616|gb|EAL39360.1|</td><td>lcl|R09A1.3 CE18129 WBGene00019973 status:Partially_confirmed SW:O61933 protein_id:AAC17777.1 /QuerySize=317</td><td>gi|6805502|ref|Os02g55800|</td></tr>
<tr><td>gi|55236623|gb|EAL39364.1|</td><td>lcl|R09A1.4 CE18130 WBGene00019974 status:Predicted SW:O61934 protein_id:AAC17778.1 /QuerySize=122</td><td>gi|6805503|ref|Os02g55810|</td></tr>
<tr><td>gi|55236631|gb|EAA43669.2|</td><td>lcl|R09A1.5 CE29447 WBGene00019975 status:Partially_confirmed SW:Q95X32 protein_id:AAL06049.1 /QuerySize=105</td><td>gi|6805504|ref|Os02g55820|</td></tr>
<tr><td>gi|55236632|gb|EAA13581.2|</td><td>lcl|R09A8.1 CE03537 WBGene00011152 status:Predicted SW:Q21852 protein_id:CAA92003.1 /QuerySize=1095</td><td>gi|6805506|ref|Os02g55840|</td></tr>
<tr><td>gi|55236636|gb|EAL39367.1|</td><td>lcl|R09A8.2 CE34082 WBGene00011153 status:Partially_confirmed SW:Q21853 protein_id:CAA92004.2 /QuerySize=891</td><td>gi|6805507|ref|Os02g55850|</td></tr>
<tr><td>gi|55236645|gb|EAL39368.1|</td><td>lcl|R09A8.5 CE03541 WBGene00011154 status:Partially_confirmed SW:Q21856 protein_id:CAA92007.1 /QuerySize=208</td><td>gi|6805512|ref|Os02g55900|</td></tr>
<tr><td>gi|55236647|gb|EAL39370.1|</td><td>lcl|R09B5.10 CE12636 WBGene00000559 locus:cnc-5 status:Partially_confirmed SW:O44614 protein_id:AAB94217.1 /QuerySize=</td><td>gi|6805514|ref|Os02g55920|</td></tr>
<tr><td>gi|55236667|gb|EAL39373.1|</td><td>lcl|R09B5.2 CE12620 WBGene00000555 locus:cnc-1 status:Predicted SW:O44615 protein_id:AAB94211.1 /QuerySize=62</td><td>gi|6805515|ref|Os02g55930|</td></tr>
<tr><td>gi|55236680|gb|EAL39375.1|</td><td>lcl|R09B5.3 CE12622 WBGene00000556 locus:cnc-2 status:Partially_confirmed SW:O44611 protein_id:AAB94212.1 /QuerySize=7</td><td>gi|6805534|ref|Os02g56090|</td></tr>
<tr><td>gi|55236686|gb|EAL39377.1|</td><td>lcl|R09B5.8 CE12632 WBGene00000557 locus:cnc-3 status:Confirmed SW:O44612 protein_id:AAB94209.1 /QuerySize=82</td><td>gi|6805540|ref|Os02g56150|</td></tr>
<tr><td>gi|55236696|gb|EAA13664.2|</td><td>lcl|R09B5.9 CE12634 WBGene00000558 locus:cnc-4 status:Confirmed SW:O44613 protein_id:AAB94216.1 /QuerySize=66</td><td>gi|6805548|ref|Os02g56220|</td></tr>
<tr><td>gi|55236706|gb|EAL39380.1|</td><td>lcl|R09E10.1 CE35599 WBGene00011171 status:Confirmed SW:Q21876 protein_id:CAA94302.3 /QuerySize=294</td><td>gi|6805550|ref|Os02g56240|</td></tr>
<tr><td>gi|55236709|gb|EAL39381.1|</td><td>lcl|R09E10.2 CE36603 WBGene00011172 status:Confirmed SW:Q21877 protein_id:CAA94303.2 /QuerySize=198</td><td>gi|6805561|ref|Os02g56340|</td></tr>
<tr><td>gi|55236710|gb|EAL39382.1|</td><td>lcl|R09E10.6 CE06288 WBGene00011176 status:Partially_confirmed SW:Q21871 protein_id:CAA94297.1 /QuerySize=299</td><td>gi|6805563|ref|Os02g56360|</td></tr>
<tr><td>gi|55236711|gb|EAL39383.1|</td><td>lcl|R09E10.8 CE23910 WBGene00011177 status:Partially_confirmed SW:Q9U391 protein_id:CAB54288.1 /QuerySize=78</td><td>gi|6805564|ref|Os02g56360|</td></tr>
<tr><td>gi|55236718|gb|EAL39386.1|</td><td>lcl|R09E12.5 CE12650 WBGene00019984 status:Predicted SW:O16257 protein_id:AAG24173.1 /QuerySize=411</td><td>gi|6805569|ref|Os02g56410|</td></tr>
<tr><td>gi|55236721|gb|EAL39388.1|</td><td>lcl|R09F10.3 CE33810 WBGene00019987 status:Confirmed SW:Q23026 protein_id:AAC69092.2 /QuerySize=468</td><td>gi|6805572|ref|Os02g56440|</td></tr>
<tr><td>gi|55236723|gb|EAA13518.3|</td><td>lcl|R09F10.5 CE07439 WBGene00019988 status:Partially_confirmed SW:Q23028 protein_id:AAC69094.1 /QuerySize=145</td><td>gi|6805573|ref|Os02g56450|</td></tr>
<tr><td>gi|55236725|gb|EAA13530.2|</td><td>lcl|R09F10.8 CE28756 WBGene00019989 status:Confirmed SW:Q23031 protein_id:AAC69097.2 /QuerySize=337</td><td>gi|6805575|ref|Os02g56470|</td></tr>
<tr><td>gi|55236735|gb|EAL39394.1|</td><td>lcl|R09F10.9 CE23911 WBGene00000427 locus:ced-13 status:Predicted SW:Q9TY06 protein_id:AAD34655.1 /QuerySize=98</td><td>gi|6805577|ref|Os02g56490|</td></tr>
<tr><td>gi|55236738|gb|EAA43702.2|</td><td>lcl|R09G11.1 CE12656 WBGene00006319 locus:sup-10 status:Confirmed SW:Q17374 protein_id:AAC48175.1 /QuerySize=332</td><td>gi|6805587|ref|Os02g56590|</td></tr>
<tr><td>gi|55236749|gb|EAL39397.1|</td><td>lcl|R09H10.7 CE34963 WBGene00011184 status:Predicted SW:Q7YTK3 protein_id:CAE17935.1 /QuerySize=94</td><td>gi|6805590|ref|Os02g56620|</td></tr>
<tr><td>gi|55236751|gb|EAL39399.1|</td><td>lcl|R09H3.1 CE07445 WBGene00019990 status:Predicted SW:Q21885 protein_id:AAB00611.1 /QuerySize=1073</td><td>gi|6805595|ref|Os02g56670|</td></tr>
<tr><td>gi|55236752|gb|EAA13481.3|</td><td>lcl|R09H3.3 CE20026 WBGene00019991 status:Predicted SW:Q9U390 protein_id:AAD32276.1 /QuerySize=191</td><td>gi|6805599|ref|Os02g56710|</td></tr>
<tr><td>gi|55236760|gb|EAL39404.1|</td><td>lcl|R102.10 CE36710 WBGene00011297 status:Confirmed SW:Q6A4J7 protein_id:CAH10784.1 /QuerySize=323</td><td>gi|6805610|ref|Os02g56810|</td></tr>
<tr><td>gi|55236761|gb|EAL39405.1|</td><td>lcl|R102.3 CE35600 WBGene00011290 status:Partially_confirmed SW:Q21889 protein_id:CAA94357.2 /QuerySize=358</td><td>gi|6805616|ref|Os02g56870|</td></tr>
<tr><td>gi|55236762|gb|EAL39406.1|</td><td>lcl|R102.6 CE06334 WBGene00011293 status:Partially_confirmed SW:Q21892 protein_id:CAA94360.1 /QuerySize=324</td><td>gi|6805618|ref|Os02g56890|</td></tr>
<tr><td>gi|55236770|gb|EAA13408.2|</td><td>lcl|R102.7 CE36711 WBGene00011294 status:Predicted SW:Q21893 protein_id:CAA94361.2 /QuerySize=324</td><td>gi|6805631|ref|Os02g57020|</td></tr>
<tr><td>gi|55236779|gb|EAL39413.1|</td><td>lcl|R102.8 CE23916 WBGene00011295 status:Partially_confirmed SW:Q9U388 protein_id:CAB54292.1 /QuerySize=209</td><td>gi|6805645|ref|Os02g57130|</td></tr>
<tr><td>gi|55236784|gb|EAA13396.2|</td><td>lcl|R106.1 CE07453 WBGene00020085 status:Predicted SW:Q23032 protein_id:AAB04991.1 /QuerySize=155</td><td>gi|6805650|ref|Os02g57170|</td></tr>
<tr><td>gi|55236796|gb|EAA43732.2|</td><td>lcl|R10A10.1 CE12668 WBGene00019992 status:Predicted SW:P91403 protein_id:AAB37825.1 /QuerySize=53</td><td>gi|6805654|ref|Os02g57210|</td></tr>
<tr><td>gi|55236801|gb|EAA13179.2|</td><td>lcl|R10D12.13c CE23921 WBGene00011194 status:Confirmed SW:Q9U386 protein_id:CAB54295.1 /QuerySize=226</td><td>gi|6805661|ref|Os02g57280|</td></tr>
<tr><td>gi|55236805|gb|EAA43736.2|</td><td>lcl|R10E11.9 CE33683 WBGene00011220 status:Partially_confirmed SW:P90945 protein_id:CAA82356.2 /QuerySize=120</td><td>gi|6805662|ref|Os02g57280|</td></tr>
<tr><td>gi|55236810|gb|EAL39424.1|</td><td>lcl|R10E12.2 CE00311 WBGene00011221 status:Partially_confirmed SW:P34553 protein_id:CAA82668.1 /QuerySize=395</td><td>gi|6805663|ref|Os02g57280|</td></tr>
<tr><td>gi|55236820|gb|EAA13190.2|</td><td>lcl|R10E4.2c CE34085 WBGene00011199 status:Confirmed SW:Q7Z204 protein_id:CAD91698.1 /QuerySize=150</td><td>gi|6805670|ref|Os02g57300|</td></tr>
<tr><td>gi|55236824|gb|EAA13121.2|</td><td>lcl|R10E4.3 CE03557 WBGene00011200 status:Partially_confirmed SW:Q21901 protein_id:CAA90764.1 /QuerySize=305</td><td>gi|6805672|ref|Os02g57320|</td></tr>
<tr><td>gi|55236825|gb|EAL39428.1|</td><td>lcl|R10E4.6 CE03560 WBGene00011202 status:Predicted SW:Q21903 protein_id:CAA90767.1 /QuerySize=375</td><td>gi|6805675|ref|Os02g57350|</td></tr>
<tr><td>gi|55236838|gb|EAL39431.1|</td><td>lcl|R10E4.7 CE03561 WBGene00011203 status:Partially_confirmed SW:Q21904 protein_id:CAA90768.1 /QuerySize=197</td><td>gi|6805716|ref|Os02g57680|</td></tr>
<tr><td>gi|55236843|gb|EAA13277.3|</td><td>lcl|R10E8.3 CE18911 WBGene00011209 status:Partially_confirmed SW:Q9XUR7 protein_id:CAB04644.1 /QuerySize=522</td><td>gi|6805721|ref|Os02g57730|</td></tr>
<tr><td>gi|55236847|gb|EAA13135.2|</td><td>lcl|R10E8.4 CE18912 WBGene00011210 status:Predicted SW:Q9XUR9 protein_id:CAB04641.1 /QuerySize=534</td><td>gi|6805731|ref|Os02g57830|</td></tr>
<tr><td>gi|55236857|gb|EAL39434.1|</td><td>lcl|R10E9.2 CE01051 WBGene00011214 status:Confirmed SW:Q21910 protein_id:CAA84666.1 /QuerySize=259</td><td>gi|6805734|ref|Os02g57850|</td></tr>
<tr><td>gi|55236868|gb|EAA13165.2|</td><td>lcl|R10E9.3 CE34086 WBGene00011215 status:Partially_confirmed SW:Q20115 protein_id:CAA84668.2 /QuerySize=149</td><td>gi|6805739|ref|Os02g57900|</td></tr>
<tr><td>gi|55236872|gb|EAA13308.2|</td><td>lcl|R10F2.4 CE12708 WBGene00019995 status:Predicted SW:O01910 protein_id:AAB54267.1 /QuerySize=204</td><td>gi|6805743|ref|Os02g57940|</td></tr>
<tr><td>gi|55236895|gb|EAA13185.2|</td><td>lcl|R10H1.1 CE31820 WBGene00019998 trypsin inhibitor status:Confirmed SW:Q10043 protein_id:AAM97991.1 /QuerySize=161</td><td>gi|6805744|ref|Os02g57950|</td></tr>
<tr><td>gi|55236919|gb|EAA13271.3|</td><td>lcl|R10H10.4 CE06295 WBGene00011223 status:Partially_confirmed SW:Q21914 protein_id:CAA94608.1 /QuerySize=80</td><td>gi|6805746|ref|Os02g57970|</td></tr>
<tr><td>gi|55236925|gb|EAL39450.1|</td><td>lcl|R11.2 CE03264 WBGene00011227 status:Partially_confirmed SW:O62346 protein_id:CAB04650.1 /QuerySize=90</td><td>gi|6805749|ref|Os02g58000|</td></tr>
<tr><td>gi|55236927|gb|EAL39452.1|</td><td>lcl|R11.3 CE12718 WBGene00011228 status:Predicted SW:O62345 protein_id:CAB04649.1 /QuerySize=528</td><td>gi|6805755|ref|Os02g58050|</td></tr>
<tr><td>gi|55236929|gb|EAA43770.2|</td><td>lcl|R11.4 CE12720 WBGene00011229 status:Confirmed SW:O62344 protein_id:CAB04648.1 /QuerySize=65</td><td>gi|6805756|ref|Os02g58060|</td></tr>
<tr><td>gi|55236940|gb|EAL39458.1|</td><td>lcl|R119.1 CE25971 WBGene00020087 status:Partially_confirmed SW:O61712 protein_id:AAC16430.2 /QuerySize=730</td><td>gi|6805769|ref|Os02g58180|</td></tr>
<tr><td>gi|55236942|gb|EAL39459.1|</td><td>lcl|R119.4 CE23925 WBGene00004143 locus:pqn-59 status:Confirmed SW:O61708 protein_id:AAD47125.1 /QuerySize=712</td><td>gi|6805771|ref|Os02g58200|</td></tr>
<tr><td>gi|55236947|gb|EAL39463.1|</td><td>lcl|R11A5.2 CE12724 WBGene00011230 status:Confirmed SW:O45717 protein_id:CAB05597.1 /QuerySize=293</td><td>gi|6805781|ref|Os02g58290|</td></tr>
<tr><td>gi|55236948|gb|EAL39464.1|</td><td>lcl|R11A5.3 CE12726 WBGene00011231 status:Confirmed SW:O02285 protein_id:CAB05599.1 /QuerySize=155</td><td>gi|6805788|ref|Os02g58360|</td></tr>
<tr><td>gi|55236951|gb|EAA43787.2|</td><td>lcl|R11A5.6 CE12732 WBGene00011234 status:Confirmed SW:O45716 protein_id:CAB05596.1 /QuerySize=113</td><td>gi|6805789|ref|Os02g58370|</td></tr>
<tr><td>gi|55236967|gb|EAA13025.2|</td><td>lcl|R11A8.1 CE06299 WBGene00011236 status:Partially_confirmed SW:Q21923 protein_id:CAA94366.1 /QuerySize=552</td><td>gi|6805790|ref|Os02g58380|</td></tr>
<tr><td>gi|55236983|gb|EAL39472.1|</td><td>lcl|R11D1.2 CE06307 WBGene00011243 status:Partially_confirmed SW:Q21935 protein_id:CAA99902.1 /QuerySize=335</td><td>gi|6805792|ref|Os02g58400|</td></tr>
<tr><td>gi|55236985|gb|EAA12952.3|</td><td>lcl|R11D1.3 CE06308 WBGene00011244 status:Partially_confirmed SW:Q21932 protein_id:CAA99900.1 /QuerySize=201</td><td>gi|6805794|ref|Os02g58420|</td></tr>
<tr><td>gi|55236998|gb|EAL39474.1|</td><td>lcl|R11D1.4 CE06309 WBGene00011245 status:Predicted SW:Q21931 protein_id:CAA99899.1 /QuerySize=207</td><td>gi|6805813|ref|Os02g58600|</td></tr>
<tr><td>gi|55237008|gb|EAA12982.2|</td><td>lcl|R11D1.7 CE06312 WBGene00011246 status:Predicted SW:Q21933 protein_id:CAA99901.1 /QuerySize=402</td><td>gi|6805816|ref|Os02g58630|</td></tr>
<tr><td>gi|55237021|gb|EAL39482.1|</td><td>lcl|R11E3.7b CE31721 WBGene00001060 locus:dpf-7 status:Confirmed SW:Q8MXE4 protein_id:AAM97938.1 /QuerySize=164</td><td>gi|6805822|ref|Os02g58680|</td></tr>
<tr><td>gi|55237022|gb|EAL39483.1|</td><td>lcl|R11G1.2 CE04833 WBGene00020011 status:Predicted SW:Q21947 protein_id:AAA82320.1 /QuerySize=284</td><td>gi|6805823|ref|Os02g58690|</td></tr>
<tr><td>gi|55237029|gb|EAL39485.1|</td><td>lcl|R11G10.3 CE37985 WBGene00044045 status:Predicted /QuerySize=284</td><td>gi|6805829|ref|Os02g58750|</td></tr>
<tr><td>gi|55237031|gb|EAL39486.1|</td><td>lcl|R11H6.4 CE12776 WBGene00011252 status:Predicted SW:O18002 protein_id:CAB07648.1 /QuerySize=230</td><td>gi|6805830|ref|Os02g58760|</td></tr>
<tr><td>gi|55237061|gb|EAL39500.1|</td><td>lcl|R12B2.3 CE01366 WBGene00020019 status:Predicted SW:Q21953 protein_id:AAA50741.1 /QuerySize=136</td><td>gi|6805835|ref|Os02g58800|</td></tr>
<tr><td>gi|55237066|gb|EAL39503.1|</td><td>lcl|R12B2.4 CE01367 WBGene00001869 locus:him-10 status:Partially_confirmed SW:Q21952 protein_id:AAA50742.1 /QuerySize=</td><td>gi|6900003|ref|Os03g01020|</td></tr>
<tr><td>gi|55237067|gb|EAA12935.2|</td><td>lcl|R12B2.6 CE30109 WBGene00020020 status:Predicted SW:P45898 protein_id:AAA50744.2 /QuerySize=106</td><td>gi|6900030|ref|Os03g01280|</td></tr>
<tr><td>gi|55237072|gb|EAL39506.1|</td><td>lcl|R12B2.7 CE32918 WBGene00020021 status:Partially_confirmed SW:Q8MPZ4 protein_id:AAM54165.2 /QuerySize=75</td><td>gi|6900035|ref|Os03g01330|</td></tr>
<tr><td>gi|55237075|gb|EAL39508.1|</td><td>lcl|R12C12.4 CE02849 WBGene00020024 status:Predicted SW:Q21958 protein_id:AAC46783.1 /QuerySize=71</td><td>gi|6900036|ref|Os03g01340|</td></tr>
<tr><td>gi|55237081|gb|EAA13057.2|</td><td>lcl|R12C12.9a CE02854 WBGene00020029 status:Confirmed SW:Q21956 protein_id:AAC46787.1 /QuerySize=250</td><td>gi|6900037|ref|Os03g01350|</td></tr>
<tr><td>gi|55237084|gb|EAL39511.1|</td><td>lcl|R12C12.9b CE32059 WBGene00020029 status:Confirmed SW:Q8IFZ5 protein_id:AAN60495.1 /QuerySize=245</td><td>gi|6900038|ref|Os03g01360|</td></tr>
<tr><td>gi|55237085|gb|EAL39512.1|</td><td>lcl|R12E2.14 CE18146 WBGene00020039 status:Partially_confirmed SW:O61782 protein_id:AAC17035.1 /QuerySize=74</td><td>gi|6900039|ref|Os03g01360|</td></tr>
<tr><td>gi|55237088|gb|EAL39514.1|</td><td>lcl|R12E2.15 CE18147 WBGene00020040 status:Partially_confirmed SW:O61781 protein_id:AAC17036.1 /QuerySize=74</td><td>gi|6900040|ref|Os03g01360|</td></tr>
<tr><td>gi|55237091|gb|EAA43802.2|</td><td>lcl|R12E2.6 CE18138 WBGene00020032 status:Partially_confirmed SW:O61783 protein_id:AAC17027.1 /QuerySize=74</td><td>gi|6900046|ref|Os03g01420|</td></tr>
<tr><td>gi|55237092|gb|EAL39516.1|</td><td>lcl|R12E2.7 CE18139 WBGene00020033 status:Confirmed SW:O61784 protein_id:AAC17028.1 /QuerySize=82</td><td>gi|6900047|ref|Os03g01430|</td></tr>
<tr><td>gi|55237094|gb|EAA43803.2|</td><td>lcl|R12E2.8 CE34360 WBGene00020034 status:Partially_confirmed SW:O61785 protein_id:AAC17029.3 /QuerySize=241</td><td>gi|6900049|ref|Os03g01450|</td></tr>
<tr><td>gi|55237101|gb|EAL39518.1|</td><td>lcl|R12H7.4 CE03569 WBGene00011255 status:Partially_confirmed SW:Q21967 protein_id:CAA90634.1 /QuerySize=369</td><td>gi|6900050|ref|Os03g01460|</td></tr>
<tr><td>gi|55237113|gb|EAL39520.1|</td><td>lcl|R13.4 CE06321 WBGene00011259 status:Partially_confirmed SW:Q21970 protein_id:CAA97439.1 /QuerySize=193</td><td>gi|6900051|ref|Os03g01470|</td></tr>
<tr><td>gi|55237153|gb|EAA12493.2|</td><td>lcl|R13A1.1 CE04837 WBGene00020041 status:Predicted SW:Q21979 protein_id:AAA81478.1 /QuerySize=75</td><td>gi|6900052|ref|Os03g01480|</td></tr>
<tr><td>gi|55237158|gb|EAA12552.2|</td><td>lcl|R13A1.5 CE04841 WBGene00020043 status:Confirmed SW:Q95ZQ7 protein_id:AAK84561.1 /QuerySize=155</td><td>gi|6900053|ref|Os03g01490|</td></tr>
<tr><td>gi|55237160|gb|EAL39538.1|</td><td>lcl|R13A1.7 CE04842 WBGene00020044 Na-K-Cl cotransporter status:Partially_confirmed SW:Q21976 protein_id:AAA81475.1 /Q</td><td>gi|6900054|ref|Os03g01500|</td></tr>
<tr><td>gi|55237162|gb|EAL39539.1|</td><td>lcl|R13A5.7 CE30389 WBGene00020050 status:Partially_confirmed SW:Q95Y91 protein_id:AAK84468.2 /QuerySize=388</td><td>gi|6900055|ref|Os03g01510|</td></tr>
</table>

<table>
<tr><td>gi|55237163|gb|EAL39540.1|</td><td>lcl|R13D11.10 CE28768 WBGene00020063 status:Partially_confirmed SW:Q965M0 protein_id:AAK82915.1 /QuerySize=79</td><td>gi|6900056|ref|Os03g01520|</td></tr>
<tr><td>gi|55237165|gb|EAL39541.1|</td><td>lcl|R13D7.2b CE33299 WBGene00020054 status:Confirmed SW:Q86S82 protein_id:AAO26007.1 /QuerySize=105</td><td>gi|6900060|ref|Os03g01560|</td></tr>
<tr><td>gi|55237166|gb|EAL39542.1|</td><td>lcl|R13F6.1 CE00720 WBGene00020064 locus:kbp-1 status:Confirmed SW:Q95QB8 protein_id:AAK84562.1 /QuerySize=149</td><td>gi|6900062|ref|Os03g01580|</td></tr>
<tr><td>gi|55237175|gb|EAL39545.1|</td><td>lcl|R13F6.4c CE32707 WBGene00006498 locus:tag-152 status:Partially_confirmed SW:Q8I7J1 protein_id:AAN65304.1 /QuerySize</td><td>gi|6900066|ref|Os03g01620|</td></tr>
<tr><td>gi|55237178|gb|EAA12556.3|</td><td>lcl|R13H4.6 CE12810 WBGene00011264 ATP-dependent protease status:Predicted SW:P90952 protein_id:CAB04658.1 /QuerySi</td><td>gi|6900083|ref|Os03g01740|</td></tr>
<tr><td>gi|55237180|gb|EAA12206.3|</td><td>lcl|R13H4.8 CE34968 WBGene00011266 status:Partially_confirmed SW:Q7YWV7 protein_id:CAE17915.1 /QuerySize=212</td><td>gi|6900091|ref|Os03g01780|</td></tr>
<tr><td>gi|55237186|gb|EAA12752.3|</td><td>lcl|R144.10 CE26946 WBGene00020098 status:Confirmed SW:Q9BI75 protein_id:AAK21459.1 /QuerySize=200</td><td>gi|6900098|ref|Os03g01840|</td></tr>
<tr><td>gi|55237192|gb|EAA12751.2|</td><td>lcl|R144.11 CE28949 WBGene00020099 status:Confirmed SW:Q95QB7 protein_id:AAK93858.1 /QuerySize=307</td><td>gi|6900100|ref|Os03g01860|</td></tr>
<tr><td>gi|55237203|gb|EAL39549.1|</td><td>lcl|R144.12 CE37548 WBGene00043060 status:Confirmed SW:Q629J5 protein_id:AAU87816.1 /QuerySize=333</td><td>gi|6900115|ref|Os03g01980|</td></tr>
<tr><td>gi|55237207|gb|EAL39550.1|</td><td>lcl|R144.5 CE31581 WBGene00020095 status:Partially_confirmed SW:Q09346 protein_id:AAK21454.2 /QuerySize=511</td><td>gi|6900118|ref|Os03g02000|</td></tr>
<tr><td>gi|55237209|gb|EAL39551.1|</td><td>lcl|R144.9 CE25092 WBGene00007015 locus:mdt-11 status:Partially_confirmed SW:Q9BI74 protein_id:AAK21456.1 /QuerySize=1</td><td>gi|6900126|ref|Os03g02060|</td></tr>
<tr><td>gi|55237210|gb|EAL39552.1|</td><td>lcl|R148.1 CE12848 WBGene00020100 status:Predicted SW:O17269 protein_id:AAB71036.1 /QuerySize=461</td><td>gi|6900128|ref|Os03g02080|</td></tr>
<tr><td>gi|55237214|gb|EAL39555.1|</td><td>lcl|R148.2 CE12850 WBGene00020101 status:Partially_confirmed SW:O17268 protein_id:AAB71037.1 /QuerySize=131</td><td>gi|6900129|ref|Os03g02080|</td></tr>
<tr><td>gi|55237215|gb|EAA12753.3|</td><td>lcl|R148.4 CE12854 WBGene00020103 status:Partially_confirmed SW:O17266 protein_id:AAB71035.1 /QuerySize=147</td><td>gi|6900130|ref|Os03g02090|</td></tr>
<tr><td>gi|55237216|gb|EAL39556.1|</td><td>lcl|R148.7 CE12860 WBGene00020105 status:Partially_confirmed SW:O17270 protein_id:AAB71041.1 /QuerySize=260</td><td>gi|6900134|ref|Os03g02130|</td></tr>
<tr><td>gi|55237230|gb|EAL39559.1|</td><td>lcl|R151.1 CE00746 WBGene00020106 status:Predicted SW:Q21995 protein_id:AAK29852.1 /QuerySize=377</td><td>gi|6900145|ref|Os03g02230|</td></tr>
<tr><td>gi|55237231|gb|EAL39560.1|</td><td>lcl|R153.1f CE33440 WBGene00020114 locus:pde-4 status:Partially_confirmed SW:Q86NE7 protein_id:AAO38609.1 /QuerySize=1</td><td>gi|6900147|ref|Os03g02250|</td></tr>
<tr><td>gi|55237232|gb|EAL39561.1|</td><td>lcl|R160.2 CE19552 WBGene00020119 status:Predicted SW:Q9TZD1 protein_id:AAC68737.1 /QuerySize=53</td><td>gi|6900152|ref|Os03g02300|</td></tr>
<tr><td>gi|55237233|gb|EAL39562.1|</td><td>lcl|R160.3 CE19553 WBGene00020120 status:Partially_confirmed SW:Q9TZD5 protein_id:AAC68736.1 /QuerySize=148</td><td>gi|6900172|ref|Os03g02470|</td></tr>
<tr><td>gi|55237235|gb|EAL39563.1|</td><td>lcl|R160.4 CE19554 WBGene00020121 status:Predicted SW:Q9TZD4 protein_id:AAC68735.1 /QuerySize=168</td><td>gi|6900174|ref|Os03g02490|</td></tr>
<tr><td>gi|55237236|gb|EAL39564.1|</td><td>lcl|R160.5 CE19555 WBGene00020122 status:Predicted SW:Q9TZD3 protein_id:AAC68734.1 /QuerySize=102</td><td>gi|6900187|ref|Os03g02620|</td></tr>
<tr><td>gi|55237237|gb|EAA12643.2|</td><td>lcl|R160.6 CE19556 WBGene00020123 status:Predicted SW:Q9TZD2 protein_id:AAC68733.1 /QuerySize=215</td><td>gi|6900191|ref|Os03g02660|</td></tr>
<tr><td>gi|55237238|gb|EAA12659.2|</td><td>lcl|R173.4 CE31059 WBGene00020126 status:Confirmed SW:Q8MPY9 protein_id:AAM51536.1 /QuerySize=84</td><td>gi|6900204|ref|Os03g02770|</td></tr>
<tr><td>gi|55237239|gb|EAA12663.3|</td><td>lcl|R186.8 CE34972 WBGene00011309 status:Predicted SW:Q7YTL0 protein_id:CAE17911.1 /QuerySize=116</td><td>gi|6900207|ref|Os03g02790|</td></tr>
<tr><td>gi|55237240|gb|EAA12644.2|</td><td>lcl|R193.1 CE21131 WBGene00020127 status:Predicted SW:Q9N5F4 protein_id:AAF39954.1 /QuerySize=186</td><td>gi|6900209|ref|Os03g02810|</td></tr>
<tr><td>gi|55237241|gb|EAA12750.2|</td><td>lcl|R193.3 CE23930 WBGene00020129 status:Predicted SW:Q9N5F5 protein_id:AAF39957.1 /QuerySize=370</td><td>gi|6900217|ref|Os03g02870|</td></tr>
<tr><td>gi|55237245|gb|EAL39566.1|</td><td>lcl|R52.4 CE12830 WBGene00020076 status:Predicted SW:O17289 protein_id:AAB71062.1 /QuerySize=168</td><td>gi|6900218|ref|Os03g02880|</td></tr>
<tr><td>gi|55237249|gb|EAA12781.2|</td><td>lcl|R53.8 CE03578 WBGene00011277 status:Partially_confirmed SW:Q22026 protein_id:CAA88572.1 /QuerySize=184</td><td>gi|6900219|ref|Os03g02890|</td></tr>
<tr><td>gi|55237250|gb|EAA12715.3|</td><td>lcl|R74.2 CE23931 WBGene00011278 status:Partially_confirmed SW:Q22029 protein_id:CAA85275.2 /QuerySize=400</td><td>gi|6900221|ref|Os03g02910|</td></tr>
<tr><td>gi|55237253|gb|EAA12730.2|</td><td>lcl|T01B10.2 CE07456 WBGene00001703 locus:grd-14 status:Confirmed SW:O02153 protein_id:AAB52859.2 /QuerySize=154</td><td>gi|6900229|ref|Os03g02990|</td></tr>
<tr><td>gi|55237261|gb|EAA12673.2|</td><td>lcl|T01B10.5 CE35754 WBGene00020137 status:Partially_confirmed SW:O02152 protein_id:AAB52860.2 /QuerySize=215</td><td>gi|6900231|ref|Os03g03010|</td></tr>
<tr><td>gi|55237279|gb|EAA12714.3|</td><td>lcl|T01B4.3 CE34973 WBGene00011310 status:Predicted SW:Q7YTK9 protein_id:CAE17914.1 /QuerySize=155</td><td>gi|6900235|ref|Os03g03050|</td></tr>
<tr><td>gi|55237293|gb|EAA12528.2|</td><td>lcl|T01B6.1 CE37141 WBGene00020134 status:Partially_confirmed /QuerySize=238</td><td>gi|6900238|ref|Os03g03080|</td></tr>
<tr><td>gi|55237304|gb|EAA12529.3|</td><td>lcl|T01B6.4 CE12884 WBGene00020136 status:Partially_confirmed SW:Q95ZK8 protein_id:AAK84565.1 /QuerySize=138</td><td>gi|6900239|ref|Os03g03090|</td></tr>
<tr><td>gi|55237340|gb|EAL39588.1|</td><td>lcl|T01B7.6 CE35362 WBGene00011312 status:Partially_confirmed SW:Q22047 protein_id:CAA91299.2 /QuerySize=765</td><td>gi|6900242|ref|Os03g03110|</td></tr>
<tr><td>gi|55237341|gb|EAL39589.1|</td><td>lcl|T01B7.8 CE03592 WBGene00011313 status:Partially_confirmed SW:Q22048 protein_id:CAA91301.1 /QuerySize=164</td><td>gi|6900250|ref|Os03g03190|</td></tr>
<tr><td>gi|55237344|gb|EAA12284.2|</td><td>lcl|T01C1.2 CE03593 WBGene00011315 status:Predicted SW:Q22052 protein_id:CAA92010.1 /QuerySize=192</td><td>gi|6900251|ref|Os03g03200|</td></tr>
<tr><td>gi|55237350|gb|EAL39590.1|</td><td>lcl|T01C1.4 CE31584 WBGene00011317 status:Partially_confirmed SW:Q8MPY7 protein_id:CAD44146.1 /QuerySize=92</td><td>gi|6900261|ref|Os03g03300|</td></tr>
<tr><td>gi|55237355|gb|EAA43851.2|</td><td>lcl|T01C3.11 CE34976 WBGene00011325 status:Predicted SW:Q7YWW2 protein_id:CAE17908.1 /QuerySize=118</td><td>gi|6900262|ref|Os03g03310|</td></tr>
<tr><td>gi|55237400|gb|EAL39601.1|</td><td>lcl|T01C3.2 CE23935 WBGene00011319 status:Confirmed SW:Q22056 protein_id:CAB01660.2 /QuerySize=98</td><td>gi|6900263|ref|Os03g03320|</td></tr>
<tr><td>gi|55237424|gb|EAL39604.1|</td><td>lcl|T01C3.9 CE12924 WBGene00011324 status:Partially_confirmed SW:Q22058 protein_id:CAB01662.1 /QuerySize=305</td><td>gi|6900264|ref|Os03g03330|</td></tr>
<tr><td>gi|55237434|gb|EAA12653.2|</td><td>lcl|T01C8.2 CE07459 WBGene00020143 status:Confirmed SW:Q22065 protein_id:AAB00576.1 /QuerySize=80</td><td>gi|6900265|ref|Os03g03340|</td></tr>
<tr><td>gi|55237444|gb|EAA12586.2|</td><td>lcl|T01C8.3 CE32205 WBGene00020144 status:Predicted SW:Q22064 protein_id:AAB00575.2 /QuerySize=215</td><td>gi|6900273|ref|Os03g03420|</td></tr>
<tr><td>gi|55237450|gb|EAL39613.1|</td><td>lcl|T01D1.1 CE12940 WBGene00020147 status:Predicted SW:P91417 protein_id:AAB37885.1 /QuerySize=135</td><td>gi|6900276|ref|Os03g03440|</td></tr>
<tr><td>gi|55237468|gb|EAL39619.1|</td><td>lcl|T01D1.3 CE12946 WBGene00020148 status:Confirmed SW:P91415 protein_id:AAB37883.1 /QuerySize=216</td><td>gi|6900277|ref|Os03g03440|</td></tr>
<tr><td>gi|55237472|gb|EAA12469.2|</td><td>lcl|T01D1.5 CE12950 WBGene00020150 status:Predicted SW:P91418 protein_id:AAB37886.1 /QuerySize=337</td><td>gi|6900283|ref|Os03g03490|</td></tr>
<tr><td>gi|55237482|gb|EAL39624.1|</td><td>lcl|T01E8.5 CE18165 WBGene00011333 status:Partially_confirmed SW:Q20749 protein_id:CAA88749.1 /QuerySize=1270</td><td>gi|6900286|ref|Os03g03520|</td></tr>
<tr><td>gi|55237488|gb|EAA12519.2|</td><td>lcl|T01E8.8 CE34977 WBGene00011335 status:Partially_confirmed SW:Q7YWV0 protein_id:CAE17927.1 /QuerySize=205</td><td>gi|6900297|ref|Os03g03630|</td></tr>
<tr><td>gi|55237491|gb|EAL39626.1|</td><td>lcl|T01G1.2 CE12970 WBGene00011337 status:Partially_confirmed SW:O45723 protein_id:CAB07272.1 /QuerySize=157</td><td>gi|6900298|ref|Os03g03640|</td></tr>
<tr><td>gi|55237505|gb|EAL39630.1|</td><td>lcl|T01G9.1 CE13010 WBGene00011343 status:Partially_confirmed SW:Q93966 protein_id:CAB00055.1 /QuerySize=220</td><td>gi|6900309|ref|Os03g03750|</td></tr>
<tr><td>gi|55237506|gb|EAL39631.1|</td><td>lcl|T01H3.5 CE35755 WBGene00011351 status:Partially_confirmed SW:Q22091 protein_id:CAA92689.2 /QuerySize=165</td><td>gi|6900312|ref|Os03g03780|</td></tr>
<tr><td>gi|55237508|gb|EAL39632.1|</td><td>lcl|T02B11.3a CE33300 WBGene00020155 status:Confirmed SW:Q86S53 protein_id:AAO26005.1 /QuerySize=198</td><td>gi|6900314|ref|Os03g03800|</td></tr>
<tr><td>gi|55237514|gb|EAL39635.1|</td><td>lcl|T02B11.3b CE33301 WBGene00020155 status:Confirmed SW:Q86S52 protein_id:AAO26006.1 /QuerySize=106</td><td>gi|6900318|ref|Os03g03840|</td></tr>
<tr><td>gi|55237522|gb|EAA43888.2|</td><td>lcl|T02B11.4 CE33302 WBGene00020156 status:Confirmed SW:O16973 protein_id:AAB69902.2 /QuerySize=198</td><td>gi|6900338|ref|Os03g04010|</td></tr>
<tr><td>gi|55237537|gb|EAL39638.1|</td><td>lcl|T02B5.2 CE13026 WBGene00011363 status:Predicted SW:P92014 protein_id:CAB03274.1 /QuerySize=185</td><td>gi|6900345|ref|Os03g04080|</td></tr>
<tr><td>gi|55237541|gb|EAA43890.2|</td><td>lcl|T02C12.4 CE30825 WBGene00011369 status:Predicted SW:Q8MM82 protein_id:CAD36496.1 /QuerySize=134</td><td>gi|6900346|ref|Os03g04090|</td></tr>
<tr><td>gi|55237545|gb|EAL39642.1|</td><td>lcl|T02C5.2 CE04849 WBGene00020159 status:Partially_confirmed SW:Q22094 protein_id:AAB36869.1 /QuerySize=117</td><td>gi|6900360|ref|Os03g04230|</td></tr>
<tr><td>gi|55237546|gb|EAL39643.1|</td><td>lcl|T02C5.3 CE34589 WBGene00020160 status:Partially_confirmed SW:Q22096 protein_id:AAB36866.2 /QuerySize=589</td><td>gi|6900365|ref|Os03g04280|</td></tr>
<tr><td>gi|55237547|gb|EAL39644.1|</td><td>lcl|T02C5.5d CE34591 WBGene00006742 locus:unc-2 status:Confirmed SW:Q7YZH2 protein_id:AAP82641.1 /QuerySize=286</td><td>gi|6900369|ref|Os03g04320|</td></tr>
<tr><td>gi|55237548|gb|EAL39645.1|</td><td>lcl|T02D1.7 CE16327 WBGene00011373 status:Partially_confirmed SW:O45726 protein_id:CAB05905.1 /QuerySize=74</td><td>gi|6900370|ref|Os03g04330|</td></tr>
<tr><td>gi|55237560|gb|EAL39648.1|</td><td>lcl|T02E1.1 CE13046 WBGene00004966 locus:spe-12 spe-12 status:Partially_confirmed SW:Q17387 protein_id:CAB04665.1 /Que</td><td>gi|6900372|ref|Os03g04350|</td></tr>
<tr><td>gi|55237565|gb|EAL39649.1|</td><td>lcl|T02E1.2 CE32480 WBGene00011375 status:Partially_confirmed SW:O02293 protein_id:CAB04663.2 /QuerySize=568</td><td>gi|6900379|ref|Os03g04420|</td></tr>
<tr><td>gi|55237567|gb|EAA12566.2|</td><td>lcl|T02E1.6 CE13056 WBGene00011378 status:Partially_confirmed SW:O02291 protein_id:CAB04661.1 /QuerySize=165</td><td>gi|6900393|ref|Os03g04540|</td></tr>
<tr><td>gi|55237568|gb|EAL39650.1|</td><td>lcl|T02E1.8 CE34981 WBGene00011380 status:Predicted SW:Q7YSH8 protein_id:CAE18064.1 /QuerySize=166</td><td>gi|6900394|ref|Os03g04550|</td></tr>
<tr><td>gi|55237570|gb|EAA12419.2|</td><td>lcl|T02E9.5 CE31981 WBGene00011383 status:Partially_confirmed SW:Q9U382 protein_id:CAB54299.2 /QuerySize=188</td><td>gi|6900400|ref|Os03g04610|</td></tr>
<tr><td>gi|55237578|gb|EAA12577.2|</td><td>lcl|T02G5.1 CE04853 WBGene00020161 status:Predicted SW:Q22110 protein_id:AAA82407.1 /QuerySize=117</td><td>gi|6900407|ref|Os03g04670|</td></tr>
<tr><td>gi|55237579|gb|EAL39653.1|</td><td>lcl|T02G5.14 CE29989 WBGene00020170 status:Partially_confirmed SW:Q8WQD4 protein_id:AAL38967.1 /QuerySize=111</td><td>gi|6900410|ref|Os03g04700|</td></tr>
<tr><td>gi|55237580|gb|EAA12539.2|</td><td>lcl|T02G5.2 CE04854 WBGene00020162 status:Partially_confirmed SW:Q22108 protein_id:AAA82405.1 /QuerySize=122</td><td>gi|6900422|ref|Os03g04820|</td></tr>
<tr><td>gi|55237600|gb|EAL39657.1|</td><td>lcl|T02G5.3 CE04855 WBGene00020163 status:Predicted SW:Q22107 protein_id:AAA82404.1 /QuerySize=238</td><td>gi|6900423|ref|Os03g04830|</td></tr>
<tr><td>gi|55237651|gb|EAL39668.1|</td><td>lcl|T02G6.1 CE18172 WBGene00011384 status:Predicted SW:O62356 protein_id:CAB04671.1 /QuerySize=234</td><td>gi|6900425|ref|Os03g04850|</td></tr>
<tr><td>gi|55237659|gb|EAL39671.1|</td><td>lcl|T02G6.2 CE18173 WBGene00011385 status:Predicted SW:O62355 protein_id:CAB04670.1 /QuerySize=314</td><td>gi|6900428|ref|Os03g04880|</td></tr>
<tr><td>gi|55237661|gb|EAL39673.1|</td><td>lcl|T02G6.3 CE28072 WBGene00011386 status:Predicted SW:O62357 protein_id:CAB04672.2 /QuerySize=160</td><td>gi|6900449|ref|Os03g05050|</td></tr>
<tr><td>gi|55237664|gb|EAA12766.2|</td><td>lcl|T02G6.4 CE18175 WBGene00011387 status:Predicted SW:O62354 protein_id:CAB04669.1 /QuerySize=323</td><td>gi|6900452|ref|Os03g05080|</td></tr>
<tr><td>gi|55237665|gb|EAA12762.2|</td><td>lcl|T02G6.6 CE18177 WBGene00011389 status:Predicted SW:O62359 protein_id:CAB04674.1 /QuerySize=397</td><td>gi|6900453|ref|Os03g05090|</td></tr>
<tr><td>gi|55237666|gb|EAA12764.2|</td><td>lcl|T02H6.10 CE21146 WBGene00020180 status:Predicted SW:Q9N5F1 protein_id:AAF39959.1 /QuerySize=116</td><td>gi|6900454|ref|Os03g05100|</td></tr>
<tr><td>gi|55237669|gb|EAA12432.3|</td><td>lcl|T02H6.3 CE21139 WBGene00020173 status:Predicted SW:Q9N5E5 protein_id:AAF39963.1 /QuerySize=1306</td><td>gi|6900456|ref|Os03g05120|</td></tr>
<tr><td>gi|55237673|gb|EAA12433.2|</td><td>lcl|T02H6.6 CE36888 WBGene00020176 status:Predicted SW:Q9N5E8 protein_id:AAF39965.2 /QuerySize=68</td><td>gi|6900457|ref|Os03g05130|</td></tr>
<tr><td>gi|55237681|gb|EAL39676.1|</td><td>lcl|T02H6.8 CE21144 WBGene00020178 status:Predicted SW:Q9N5F0 protein_id:AAF39960.1 /QuerySize=147</td><td>gi|6900459|ref|Os03g05150|</td></tr>
<tr><td>gi|55237683|gb|EAA12763.2|</td><td>lcl|T03D3.5 CE33823 WBGene00020183 status:Confirmed SW:O16979 protein_id:AAG24188.3 /QuerySize=343</td><td>gi|6900461|ref|Os03g05170|</td></tr>
<tr><td>gi|55237685|gb|EAL39678.1|</td><td>lcl|T03D8.1b CE18919 WBGene00003830 locus:num-1 status:Confirmed SW:Q9XTY6 protein_id:CAB07407.1 /QuerySize=401</td><td>gi|6900467|ref|Os03g05220|</td></tr>
<tr><td>gi|55237702|gb|EAL39683.1|</td><td>lcl|T03D8.7 CE18925 WBGene00011394 status:Partially_confirmed SW:Q9XTY0 protein_id:CAB07411.1 /QuerySize=84</td><td>gi|6900468|ref|Os03g05230|</td></tr>
<tr><td>gi|55237714|gb|EAL39686.1|</td><td>lcl|T03F1.6a CE13106 WBGene00020188 status:Confirmed SW:P91421 protein_id:AAB42234.1 /QuerySize=342</td><td>gi|6900482|ref|Os03g05370|</td></tr>
<tr><td>gi|55237723|gb|EAA43921.2|</td><td>lcl|T03F1.6b CE28248 WBGene00020188 status:Confirmed SW:Q95Q44 protein_id:AAK68396.1 /QuerySize=345</td><td>gi|6900483|ref|Os03g05380|</td></tr>
<tr><td>gi|55237729|gb|EAA12786.2|</td><td>lcl|T03F1.9 CE13112 WBGene00001832 locus:hcp-4 status:Confirmed SW:P91426 protein_id:AAB42237.1 /QuerySize=866</td><td>gi|6900488|ref|Os03g05410|</td></tr>
<tr><td>gi|55237749|gb|EAA12790.2|</td><td>lcl|T03F7.5 CE06348 WBGene00011402 status:Partially_confirmed SW:Q22117 protein_id:CAA98520.1 /QuerySize=255</td><td>gi|6900497|ref|Os03g05490|</td></tr>
<tr><td>gi|55237752|gb|EAA12791.3|</td><td>lcl|T03F7.6 CE06349 WBGene00011403 status:Predicted SW:Q22118 protein_id:CAA98521.1 /QuerySize=237</td><td>gi|6900503|ref|Os03g05550|</td></tr>
<tr><td>gi|55237757|gb|EAL39693.1|</td><td>lcl|T03G11.1 CE31226 WBGene00004145 locus:pqn-62 status:Partially_confirmed SW:Q22126 protein_id:AAA82452.4 /QuerySize</td><td>gi|6900506|ref|Os03g05580|</td></tr>
<tr><td>gi|55237771|gb|EAL39699.1|</td><td>lcl|T03G11.5 CE04873 WBGene00020198 exopolygalacturonase status:Partially_confirmed SW:Q22121 protein_id:AAA82447.1 /C</td><td>gi|6900508|ref|Os03g05600|</td></tr>
<tr><td>gi|55237776|gb|EAL39700.1|</td><td>lcl|T03G6.1 CE35756 WBGene00020194 status:Confirmed SW:Q22128 protein_id:AAA81705.2 /QuerySize=333</td><td>gi|6900511|ref|Os03g05630|</td></tr>
<tr><td>gi|55237788|gb|EAL39703.1|</td><td>lcl|T04A6.1a CE31585 WBGene00020200 status:Confirmed SW:Q8MPY3 protein_id:AAM97940.1 /QuerySize=175</td><td>gi|6900513|ref|Os03g05650|</td></tr>
<tr><td>gi|55237789|gb|EAL39704.1|</td><td>lcl|T04A6.1b CE31586 WBGene00020200 status:Confirmed SW:Q8MPY2 protein_id:AAM97941.1 /QuerySize=28</td><td>gi|6900518|ref|Os03g05700|</td></tr>
<tr><td>gi|55237798|gb|EAA43936.2|</td><td>lcl|T04A6.2 CE00758 WBGene00020201 status:Partially_confirmed SW:Q22131 protein_id:AAA50673.1 /QuerySize=165</td><td>gi|6900537|ref|Os03g05890|</td></tr>
<tr><td>gi|55237838|gb|EAA12700.2|</td><td>lcl|T04A6.3 CE00840 WBGene00020202 status:Predicted SW:Q22130 protein_id:AAA50674.1 /QuerySize=456</td><td>gi|6900543|ref|Os03g05940|</td></tr>
<tr><td>gi|55237855|gb|EAA12718.2|</td><td>lcl|T04A8.10 CE01065 WBGene00011411 non-histone chromosomal protein HMG-1 status:Confirmed SW:Q22139 protein_id:CAA</td><td>gi|6900544|ref|Os03g05950|</td></tr>
<tr><td>gi|55237866|gb|EAA12677.2|</td><td>lcl|T04A8.13 CE23943 WBGene00011414 neurofilament triplet M protein status:Partially_confirmed SW:Q22142 protein_id:CAA84</td><td>gi|6900546|ref|Os03g05970|</td></tr>
<tr><td>gi|55237873|gb|EAL39725.1|</td><td>lcl|T04A8.15 CE33538 WBGene00011415 glucose-6-phosphate 1-dehydrogenase status:Partially_confirmed SW:Q22145 protein_</td><td>gi|6900552|ref|Os03g06030|</td></tr>
<tr><td>gi|55237877|gb|EAL39726.1|</td><td>lcl|T04B2.3 CE23944 WBGene00011422 status:Partially_confirmed SW:Q22147 protein_id:CAA92610.2 /QuerySize=412</td><td>gi|6900570|ref|Os03g06210|</td></tr>
<tr><td>gi|55237881|gb|EAL39727.1|</td><td>lcl|T04B2.7 CE36499 WBGene00011425 status:Predicted SW:Q86GC1 protein_id:CAD60422.2 /QuerySize=367</td><td>gi|6900580|ref|Os03g06280|</td></tr>
<tr><td>gi|55237898|gb|EAL39731.1|</td><td>lcl|T04B2.8 CE37847 WBGene00044018 status:Predicted /QuerySize=111</td><td>gi|6900584|ref|Os03g06320|</td></tr>
<tr><td>gi|55237911|gb|EAA12808.2|</td><td>lcl|T04B8.4 CE18192 WBGene00020206 status:Predicted SW:O44845 protein_id:AAB95010.1 /QuerySize=52</td><td>gi|6900588|ref|Os03g06360|</td></tr>
<tr><td>gi|55237912|gb|EAA12445.3|</td><td>lcl|T04C10.3 CE06354 WBGene00011426 status:Predicted SW:Q22154 protein_id:CAA93755.1 /QuerySize=278</td><td>gi|6900592|ref|Os03g06380|</td></tr>
<tr><td>gi|55237914|gb|EAL39734.1|</td><td>lcl|T04C12.1 CE13142 WBGene00011427 status:Partially_confirmed SW:O02295 protein_id:CAB04679.1 /QuerySize=217</td><td>gi|6900594|ref|Os03g06400|</td></tr>
<tr><td>gi|55237915|gb|EAL39735.1|</td><td>lcl|T04C12.3 CE13146 WBGene00011428 status:Confirmed SW:O45745 protein_id:CAB04677.1 /QuerySize=76</td><td>gi|6900596|ref|Os03g06420|</td></tr>
<tr><td>gi|55237919|gb|EAA12819.2|</td><td>lcl|T04C12.7 CE34982 WBGene00011429 status:Predicted SW:Q7YWW4 protein_id:CAE17905.1 /QuerySize=202</td><td>gi|6900597|ref|Os03g06420|</td></tr>
<tr><td>gi|55237928|gb|EAA12810.2|</td><td>lcl|T04C12.8 CE34983 WBGene00011430 status:Partially_confirmed SW:Q7YWW3 protein_id:CAE17906.1 /QuerySize=77</td><td>gi|6900600|ref|Os03g06450|</td></tr>
<tr><td>gi|55237933|gb|EAA12826.2|</td><td>lcl|T04C9.2 CE04877 WBGene00020210 status:Predicted SW:Q9GP91 protein_id:AAG01555.1 /QuerySize=116</td><td>gi|6900602|ref|Os03g06470|</td></tr>
<tr><td>gi|55237943|gb|EAA12742.2|</td><td>lcl|T04C9.3 CE04878 WBGene00020211 dual bar protein (weak) status:Predicted SW:Q9GP92 protein_id:AAG01556.1 /QuerySize</td><td>gi|6900603|ref|Os03g06480|</td></tr>
<tr><td>gi|55237947|gb|EAA12822.3|</td><td>lcl|T04D3.1 CE13153 WBGene00011431 status:Partially_confirmed SW:O18697 protein_id:CAB03285.1 /QuerySize=454</td><td>gi|6900604|ref|Os03g06490|</td></tr>
<tr><td>gi|55237949|gb|EAL39740.1|</td><td>lcl|T04D3.2 CE13155 WBGene00011432 status:Partially_confirmed SW:O02297 protein_id:CAB03286.1 /QuerySize=604</td><td>gi|6900605|ref|Os03g06500|</td></tr>
<tr><td>gi|55237950|gb|EAL39741.1|</td><td>lcl|T04D3.5 CE13161 WBGene00011434 status:Partially_confirmed SW:O02299 protein_id:CAB03287.1 /QuerySize=300</td><td>gi|6900608|ref|Os03g06530|</td></tr>
<tr><td>gi|55237952|gb|EAA12823.3|</td><td>lcl|T04D3.8 CE27434 WBGene00011435 status:Confirmed SW:Q95ZQ2 protein_id:CAC42332.1 /QuerySize=89</td><td>gi|6900613|ref|Os03g06580|</td></tr>
<tr><td>gi|55237970|gb|EAA12634.2|</td><td>lcl|T04F8.7 CE03619 WBGene00011445 status:Partially_confirmed SW:Q22166 protein_id:CAA91481.1 /QuerySize=317</td><td>gi|6900614|ref|Os03g06590|</td></tr>
<tr><td>gi|55237971|gb|EAA12616.2|</td><td>lcl|T04F8.8 CE18927 WBGene00011446 status:Confirmed SW:Q22168 protein_id:CAA91483.1 /QuerySize=165</td><td>gi|6900615|ref|Os03g06600|</td></tr>
<tr><td>gi|55237972|gb|EAA12626.2|</td><td>lcl|T04F8.9 CE34984 WBGene00011447 status:Predicted SW:Q7YWV2 protein_id:CAE17924.1 /QuerySize=287</td><td>gi|6900623|ref|Os03g06640|</td></tr>
<tr><td>gi|55237985|gb|EAL39747.1|</td><td>lcl|T04G9.1 CE04882 WBGene00020214 status:Partially_confirmed SW:Q22173 protein_id:AAA82463.1 /QuerySize=1610</td><td>gi|6900624|ref|Os03g06650|</td></tr>
<tr><td>gi|55237987|gb|EAL39749.1|</td><td>lcl|T04G9.6 CE04887 WBGene00020217 status:Predicted SW:Q22172 protein_id:AAA82462.1 /QuerySize=601</td><td>gi|6900628|ref|Os03g06690|</td></tr>
<tr><td>gi|55237992|gb|EAA12099.2|</td><td>lcl|T04G9.7 CE30161 WBGene00020218 status:Partially_confirmed SW:Q9TXQ6 protein_id:AAD50511.2 /QuerySize=131</td><td>gi|6900635|ref|Os03g06750|</td></tr>
<tr><td>gi|55238003|gb|EAA12136.2|</td><td>lcl|T05A10.1b CE36396 WBGene00004862 locus:sma-9 status:Partially_confirmed SW:Q8MPY1 protein_id:CAD44152.2 /QuerySi</td><td>gi|6900637|ref|Os03g06770|</td></tr>
<tr><td>gi|55238006|gb|EAL39753.1|</td><td>lcl|T05A10.2 CE03634 WBGene00000525 locus:clc-4 status:Partially_confirmed SW:Q22191 protein_id:CAA92134.1 /QuerySize=</td><td>gi|6900638|ref|Os03g06780|</td></tr>
<tr><td>gi|55238007|gb|EAA12097.2|</td><td>lcl|T05A12.3 CE04898 WBGene00020234 status:Partially_confirmed SW:Q9GYK8 protein_id:AAF98587.1 /QuerySize=655</td><td>gi|6900639|ref|Os03g06790|</td></tr>
<tr><td>gi|55238008|gb|EAA12135.2|</td><td>lcl|T05A7.1 CE04888 WBGene00020219 status:Predicted SW:Q22207 protein_id:AAA81119.1 /QuerySize=242</td><td>gi|6900640|ref|Os03g06800|</td></tr>
<tr><td>gi|55238009|gb|EAL39754.1|</td><td>lcl|T05A7.4 CE04890 WBGene00001976 locus:hmg-11 nuclear phosphoprotein (weak) status:Confirmed SW:Q22204 protein_id:A</td><td>gi|6900641|ref|Os03g06810|</td></tr>
<tr><td>gi|55238020|gb|EAA12040.2|</td><td>lcl|T05A7.7 CE04893 WBGene00020224 locus:sre-39 glycoprotein H precursor status:Partially_confirmed SW:Q22208 protein_id:</td><td>gi|6900642|ref|Os03g06820|</td></tr>
<tr><td>gi|55238043|gb|EAA12075.3|</td><td>lcl|T05A8.1 CE18197 WBGene00020227 status:Predicted SW:O44860 protein_id:AAB95018.1 /QuerySize=159</td><td>gi|6900643|ref|Os03g06830|</td></tr>
<tr><td>gi|55238050|gb|EAL39766.1|</td><td>lcl|T05A8.2 CE18198 WBGene00020228 status:Predicted SW:O44859 protein_id:AAB95019.1 /QuerySize=221</td><td>gi|6900647|ref|Os03g06870|</td></tr>
<tr><td>gi|55238069|gb|EAA12105.3|</td><td>lcl|T05A8.3 CE18199 WBGene00020229 status:Confirmed SW:O44858 protein_id:AAB95020.1 /QuerySize=103</td><td>gi|6900670|ref|Os03g07090|</td></tr>
<tr><td>gi|55238072|gb|EAL39768.1|</td><td>lcl|T05A8.6 CE18202 WBGene00020232 status:Predicted SW:O44855 protein_id:AAB95017.1 /QuerySize=210</td><td>gi|6900680|ref|Os03g07180|</td></tr>
<tr><td>gi|55238080|gb|EAA12114.3|</td><td>lcl|T05A8.7 CE25979 WBGene00020233 status:Predicted SW:Q965K7 protein_id:AAK84566.1 /QuerySize=402</td><td>gi|6900684|ref|Os03g07210|</td></tr>
<tr><td>gi|55238097|gb|EAL39776.1|</td><td>lcl|T05B11.3 CE13219 WBGene00020246 locus:clic-1 status:Confirmed SW:P90961 protein_id:AAB37069.1 /QuerySize=226</td><td>gi|6900690|ref|Os03g07270|</td></tr>
<tr><td>gi|55238107|gb|EAA12013.2|</td><td>lcl|T05B4.4 CE30826 WBGene00020238 status:Partially_confirmed SW:O16423 protein_id:AAC69056.2 /QuerySize=178</td><td>gi|6900691|ref|Os03g07280|</td></tr>
<tr><td>gi|55238115|gb|EAL39785.1|</td><td>lcl|T05B9.1 CE02310 WBGene00011464 status:Confirmed SW:Q22215 protein_id:CAA88962.1 /QuerySize=262</td><td>gi|6900702|ref|Os03g07390|</td></tr>
<tr><td>gi|55238117|gb|EAL39786.1|</td><td>lcl|T05B9.2 CE02311 WBGene00011465 status:Partially_confirmed SW:Q22214 protein_id:CAA88961.1 /QuerySize=177</td><td>gi|6900704|ref|Os03g07410|</td></tr>
<tr><td>gi|55238118|gb|EAA44008.2|</td><td>lcl|T05C1.1 CE02859 WBGene00020248 status:Partially_confirmed SW:Q22217 protein_id:AAA68389.1 /QuerySize=283</td><td>gi|6900709|ref|Os03g07460|</td></tr>
<tr><td>gi|55238119|gb|EAL39787.1|</td><td>lcl|T05C1.2 CE02860 WBGene00020249 status:Predicted SW:Q22218 protein_id:AAA68390.1 /QuerySize=145</td><td>gi|6900712|ref|Os03g07490|</td></tr>
</table>

<table>
<tr><td>gi|55238121|gb|EAL39788.1|</td><td>lcl|T05C1.3 CE02861 WBGene00020250 status:Partially_confirmed SW:Q22219 protein_id:AAA68391.1 /QuerySize=233</td><td>gi|6900713|ref|Os03g07500|</td></tr>
<tr><td>gi|55238124|gb|EAL39790.1|</td><td>lcl|T05C12.11 CE34990 WBGene00011473 status:Predicted SW:Q7YWU2 protein_id:CAE17939.1 /QuerySize=312</td><td>gi|6900715|ref|Os03g07520|</td></tr>
<tr><td>gi|55238128|gb|EAL39791.1|</td><td>lcl|T05C12.4 CE32927 WBGene00011468 status:Partially_confirmed SW:Q22225 protein_id:CAA91305.2 /QuerySize=630</td><td>gi|6900718|ref|Os03g07550|</td></tr>
<tr><td>gi|55238134|gb|EAL39792.1|</td><td>lcl|T05C12.8 CE02320 WBGene00011471 status:Partially_confirmed SW:Q22229 protein_id:CAA91309.1 /QuerySize=318</td><td>gi|6900719|ref|Os03g07560|</td></tr>
<tr><td>gi|55238141|gb|EAL39793.1|</td><td>lcl|T05C12.9 CE02321 WBGene00011472 status:Predicted SW:Q22231 protein_id:CAA91311.1 /QuerySize=182</td><td>gi|6900722|ref|Os03g07590|</td></tr>
<tr><td>gi|55238146|gb|EAL39796.1|</td><td>lcl|T05C3.3 CE13225 WBGene00020255 status:Predicted SW:O16300 protein_id:AAB65360.1 /QuerySize=931</td><td>gi|6900726|ref|Os03g07630|</td></tr>
<tr><td>gi|55238152|gb|EAL39799.1|</td><td>lcl|T05C7.1 CE18204 WBGene00020257 status:Predicted SW:O76362 protein_id:AAC19187.1 /QuerySize=180</td><td>gi|6900728|ref|Os03g07650|</td></tr>
<tr><td>gi|55238159|gb|EAA10961.3|</td><td>lcl|T05D4.2 CE16342 WBGene00011475 status:Partially_confirmed SW:O45748 protein_id:CAB03292.1 /QuerySize=314</td><td>gi|6900732|ref|Os03g07690|</td></tr>
<tr><td>gi|55238173|gb|EAL39803.1|</td><td>lcl|T05D4.3 CE33168 WBGene00011476 status:Confirmed SW:O45749 protein_id:CAB03293.2 /QuerySize=486</td><td>gi|6900733|ref|Os03g07700|</td></tr>
<tr><td>gi|55238181|gb|EAL39805.1|</td><td>lcl|T05D4.4 CE34087 WBGene00011477 status:Partially_confirmed SW:O45750 protein_id:CAB03294.2 /QuerySize=651</td><td>gi|6900734|ref|Os03g07710|</td></tr>
<tr><td>gi|55238182|gb|EAA11820.3|</td><td>lcl|T05E11.2 CE35611 WBGene00011479 status:Partially_confirmed SW:Q22234 protein_id:CAA92972.2 /QuerySize=193</td><td>gi|6900735|ref|Os03g07720|</td></tr>
<tr><td>gi|55238183|gb|EAA11838.2|</td><td>lcl|T05E7.4 CE34362 WBGene00020260 status:Partially_confirmed SW:O01859 protein_id:AAB54216.2 /QuerySize=185</td><td>gi|6900736|ref|Os03g07730|</td></tr>
<tr><td>gi|55238193|gb|EAL39809.1|</td><td>lcl|T05E7.5 CE13239 WBGene00020261 status:Partially_confirmed SW:O01861 protein_id:AAB54217.1 /QuerySize=381</td><td>gi|6900737|ref|Os03g07740|</td></tr>
<tr><td>gi|55238203|gb|EAL39811.1|</td><td>lcl|T05E8.2 CE34363 WBGene00001859 locus:hil-8 status:Partially_confirmed SW:O01597 protein_id:AAB52426.2 /QuerySize=15</td><td>gi|6900738|ref|Os03g07750|</td></tr>
<tr><td>gi|55238210|gb|EAA11129.3|</td><td>lcl|T05F1.2 CE18929 WBGene00011489 status:Confirmed SW:Q9XUR4 protein_id:CAB04690.1 /QuerySize=579</td><td>gi|6900739|ref|Os03g07750|</td></tr>
<tr><td>gi|55238212|gb|EAA11149.3|</td><td>lcl|T05F1.4 CE13261 WBGene00011490 status:Confirmed SW:O18036 protein_id:CAB04691.1 /QuerySize=203</td><td>gi|6900740|ref|Os03g07760|</td></tr>
<tr><td>gi|55238218|gb|EAL39814.1|</td><td>lcl|T05F1.5 CE23949 WBGene00011491 status:Partially_confirmed SW:O18034 protein_id:CAB04688.2 /QuerySize=366</td><td>gi|6900745|ref|Os03g07810|</td></tr>
<tr><td>gi|55238219|gb|EAL39815.1|</td><td>lcl|T05F1.7 CE13255 WBGene00011492 status:Predicted SW:O18032 protein_id:CAB04696.1 /QuerySize=484</td><td>gi|6900750|ref|Os03g07860|</td></tr>
<tr><td>gi|55238221|gb|EAL39816.1|</td><td>lcl|T05F1.9 CE20037 WBGene00011494 status:Partially_confirmed SW:Q9XUR3 protein_id:CAB04693.1 /QuerySize=359</td><td>gi|6900756|ref|Os03g07900|</td></tr>
<tr><td>gi|55238225|gb|EAA11911.2|</td><td>lcl|T05G5.11 CE28077 WBGene00011504 status:Partially_confirmed SW:Q95ZP9 protein_id:CAC42341.1 /QuerySize=115</td><td>gi|6900758|ref|Os03g07920|</td></tr>
<tr><td>gi|55238231|gb|EAL39820.1|</td><td>lcl|T05G5.12 CE31363 WBGene00000932 locus:dao-6 status:Confirmed SW:Q8MPY0 protein_id:CAD44154.1 /QuerySize=92</td><td>gi|6900761|ref|Os03g07950|</td></tr>
<tr><td>gi|55238238|gb|EAL39821.1|</td><td>lcl|T05G5.4 CE00316 WBGene00011499 status:Partially_confirmed SW:P34557 protein_id:CAA81591.1 /QuerySize=78</td><td>gi|6900770|ref|Os03g08030|</td></tr>
<tr><td>gi|55238249|gb|EAA10873.2|</td><td>lcl|T05H10.3 CE01640 WBGene00011508 status:Confirmed SW:Q09347 protein_id:CAA87790.1 /QuerySize=158</td><td>gi|6900771|ref|Os03g08040|</td></tr>
<tr><td>gi|55238252|gb|EAL39823.1|</td><td>lcl|T05H10.4 CE01641 WBGene00011509 status:Partially_confirmed SW:Q09348 protein_id:CAA87791.1 /QuerySize=436</td><td>gi|6900782|ref|Os03g08130|</td></tr>
<tr><td>gi|55238256|gb|EAL39825.1|</td><td>lcl|T05H4.11 CE27440 WBGene00020274 status:Confirmed SW:O16516 protein_id:AAB66017.2 /QuerySize=210</td><td>gi|6900788|ref|Os03g08180|</td></tr>
<tr><td>gi|55238280|gb|EAL39833.1|</td><td>lcl|T05H4.12 CE13291 WBGene00020275 status:Confirmed SW:O16517 protein_id:AAB66018.1 /QuerySize=129</td><td>gi|6900789|ref|Os03g08190|</td></tr>
<tr><td>gi|55238281|gb|EAL39834.1|</td><td>lcl|T05H4.15a CE13297 WBGene00020276 status:Confirmed SW:O16523 protein_id:AAB66020.1 /QuerySize=409</td><td>gi|6900791|ref|Os03g08210|</td></tr>
<tr><td>gi|55238282|gb|EAL39835.1|</td><td>lcl|T05H4.15b CE33308 WBGene00020276 status:Confirmed SW:Q86S56 protein_id:AAO26004.1 /QuerySize=193</td><td>gi|6900794|ref|Os03g08240|</td></tr>
<tr><td>gi|55238287|gb|EAA11135.2|</td><td>lcl|T06A1.4 CE18208 WBGene00020279 status:Predicted SW:O61928 protein_id:AAC17771.1 /QuerySize=110</td><td>gi|6900795|ref|Os03g08250|</td></tr>
<tr><td>gi|55238288|gb|EAL39836.1|</td><td>lcl|T06A4.2 CE29464 WBGene00020282 status:Partially_confirmed SW:Q9TZH3 protein_id:AAC67472.2 /QuerySize=167</td><td>gi|6900796|ref|Os03g08260|</td></tr>
<tr><td>gi|55238289|gb|EAA11136.3|</td><td>lcl|T06C10.4 CE04902 WBGene00001453 locus:flp-10 status:Confirmed SW:Q22242 protein_id:AAA82299.1 /QuerySize=67</td><td>gi|6900799|ref|Os03g08290|</td></tr>
<tr><td>gi|55238293|gb|EAL39837.1|</td><td>lcl|T06C12.12 CE16362 WBGene00011519 status:Predicted SW:O18047 protein_id:CAB03308.1 /QuerySize=112</td><td>gi|6900804|ref|Os03g08340|</td></tr>
<tr><td>gi|55238294|gb|EAL39838.1|</td><td>lcl|T06D10.1 CE25101 WBGene00011531 status:Confirmed SW:Q9U379 protein_id:CAB63307.1 /QuerySize=171</td><td>gi|6900827|ref|Os03g08510|</td></tr>
<tr><td>gi|55238296|gb|EAL39839.1|</td><td>lcl|T06D4.1 CE13301 WBGene00020290 status:Partially_confirmed SW:O16649 protein_id:AAB66121.1 /QuerySize=441</td><td>gi|6900830|ref|Os03g08540|</td></tr>
<tr><td>gi|55238306|gb|EAA44033.2|</td><td>lcl|T06D4.2 CE36890 WBGene00020291 status:Predicted SW:O16650 protein_id:AAB66122.2 /QuerySize=366</td><td>gi|6900834|ref|Os03g08580|</td></tr>
<tr><td>gi|55238308|gb|EAL39844.1|</td><td>lcl|T06E4.10 CE06376 WBGene00011537 status:Partially_confirmed SW:Q22264 protein_id:CAA94796.1 /QuerySize=228</td><td>gi|6900835|ref|Os03g08580|</td></tr>
<tr><td>gi|55238322|gb|EAA11137.2|</td><td>lcl|T06E4.11 CE06377 WBGene00004146 locus:pqn-63 status:Partially_confirmed SW:Q22265 protein_id:CAA94797.1 /QuerySize</td><td>gi|6900836|ref|Os03g08590|</td></tr>
<tr><td>gi|55238329|gb|EAA11138.2|</td><td>lcl|T06E4.12 CE37848 WBGene00044011 status:Partially_confirmed /QuerySize=228</td><td>gi|6900842|ref|Os03g08650|</td></tr>
<tr><td>gi|55238372|gb|EAL39861.1|</td><td>lcl|T06E4.8 CE23951 WBGene00011535 status:Partially_confirmed SW:Q22262 protein_id:CAA94794.2 /QuerySize=199</td><td>gi|6900844|ref|Os03g08670|</td></tr>
<tr><td>gi|55238374|gb|EAL39862.1|</td><td>lcl|T06E4.9 CE23952 WBGene00011536 status:Partially_confirmed SW:Q22263 protein_id:CAA94795.2 /QuerySize=212</td><td>gi|6900846|ref|Os03g08690|</td></tr>
<tr><td>gi|55238383|gb|EAA11013.2|</td><td>lcl|T06E8.2 CE06379 WBGene00011544 status:Predicted SW:Q22266 protein_id:CAA98275.1 /QuerySize=104</td><td>gi|6900856|ref|Os03g08780|</td></tr>
<tr><td>gi|55238390|gb|EAL39867.1|</td><td>lcl|T06F4.1a CE23953 WBGene00020294 status:Partially_confirmed SW:Q95ZP7 protein_id:AAK71398.1 /QuerySize=177</td><td>gi|6900868|ref|Os03g08890|</td></tr>
<tr><td>gi|55238399|gb|EAA11073.2|</td><td>lcl|T06F4.1b CE23954 WBGene00020294 status:Partially_confirmed SW:Q95ZP6 protein_id:AAK71399.1 /QuerySize=184</td><td>gi|6900873|ref|Os03g08940|</td></tr>
<tr><td>gi|55238400|gb|EAA11060.2|</td><td>lcl|T06G6.1 CE13333 WBGene00005049 locus:sra-23 7TM chemoreceptor</td><td>gi|6900876|ref|Os03g08970|</td></tr>
<tr><td>gi|55238401|gb|EAL39869.1|</td><td>lcl|T06G6.12 CE34994 WBGene00011551 status:Predicted SW:Q7YWV1 protein_id:CAE17926.1 /QuerySize=109</td><td>gi|6900884|ref|Os03g09030|</td></tr>
<tr><td>gi|55238409|gb|EAA11546.2|</td><td>lcl|T06G6.2 CE33445 WBGene00005050 locus:sra-24 7TM chemoreceptor</td><td>gi|6900885|ref|Os03g09040|</td></tr>
<tr><td>gi|55238412|gb|EAA11040.2|</td><td>lcl|T06G6.3a CE32489 WBGene00011545 status:Partially_confirmed SW:O18051 protein_id:CAB04708.2 /QuerySize=511</td><td>gi|6900886|ref|Os03g09050|</td></tr>
<tr><td>gi|55238413|gb|EAA11016.2|</td><td>lcl|T06G6.3b CE37142 WBGene00011545 status:Partially_confirmed SW:Q69Z10 protein_id:CAH10780.1 /QuerySize=483</td><td>gi|6900894|ref|Os03g09130|</td></tr>
<tr><td>gi|55238415|gb|EAL39873.1|</td><td>lcl|T06G6.5 CE13341 WBGene00011547 status:Predicted SW:O62369 protein_id:CAB04703.1 /QuerySize=203</td><td>gi|6900898|ref|Os03g09160|</td></tr>
<tr><td>gi|55238428|gb|EAA44055.2|</td><td>lcl|T06G6.6 CE37143 WBGene00011548 status:Confirmed SW:O18052 protein_id:CAB04705.2 /QuerySize=247</td><td>gi|6900912|ref|Os03g09240|</td></tr>
<tr><td>gi|55238431|gb|EAL39875.1|</td><td>lcl|T06H11.2 CE02332 WBGene00011552 status:Predicted SW:Q22272 protein_id:CAA90067.1 /QuerySize=76</td><td>gi|6900922|ref|Os03g09830|</td></tr>
<tr><td>gi|55238439|gb|EAA11358.2|</td><td>lcl|T06H11.5 CE34995 WBGene00011553 status:Predicted SW:Q7YTK6 protein_id:CAE17921.1 /QuerySize=155</td><td>gi|6900930|ref|Os03g09890|</td></tr>
<tr><td>gi|55238451|gb|EAL39878.1|</td><td>lcl|T07A5.5 CE36502 WBGene00011558 status:Predicted SW:Q6BEV3 protein_id:CAH04721.1 /QuerySize=35</td><td>gi|6900939|ref|Os03g09960|</td></tr>
<tr><td>gi|55238466|gb|EAA11730.3|</td><td>lcl|T07A9.12a CE27204 WBGene00020299 status:Partially_confirmed SW:Q95X70 protein_id:AAK39285.1 /QuerySize=145</td><td>gi|6900944|ref|Os03g10010|</td></tr>
<tr><td>gi|55238467|gb|EAL39882.1|</td><td>lcl|T07A9.12b CE31597 WBGene00020299 status:Partially_confirmed SW:Q8MXI5 protein_id:AAM97947.1 /QuerySize=139</td><td>gi|6900947|ref|Os03g10040|</td></tr>
<tr><td>gi|55238477|gb|EAL39886.1|</td><td>lcl|T07A9.13a CE31598 WBGene00020300 status:Partially_confirmed SW:Q95X71 protein_id:AAK39286.2 /QuerySize=111</td><td>gi|6900956|ref|Os03g10130|</td></tr>
<tr><td>gi|55238480|gb|EAL39887.1|</td><td>lcl|T07A9.13b CE37788 WBGene00020300 status:Confirmed protein_id:AAV58861.1 /QuerySize=105</td><td>gi|6900959|ref|Os03g10160|</td></tr>
<tr><td>gi|55238481|gb|EAA11245.2|</td><td>lcl|T07C12.10 CE06392 WBGene00011574 status:Predicted SW:Q22278 protein_id:CAA98281.1 /QuerySize=88</td><td>gi|6900960|ref|Os03g10170|</td></tr>
<tr><td>gi|55238490|gb|EAL39890.1|</td><td>lcl|T07C12.13 CE34998 WBGene00011577 status:Predicted SW:Q7YWT7 protein_id:CAE17945.1 /QuerySize=74</td><td>gi|6900968|ref|Os03g10250|</td></tr>
<tr><td>gi|55238491|gb|EAL39891.1|</td><td>lcl|T07C4.3a CE00602 WBGene00011560 status:Confirmed SW:Q22290 protein_id:CAA82576.1 /QuerySize=649</td><td>gi|6900969|ref|Os03g10260|</td></tr>
<tr><td>gi|55238502|gb|EAA11081.3|</td><td>lcl|T07C4.3b CE21158 WBGene00011560 status:Confirmed SW:Q9U376 protein_id:CAA82581.1 /QuerySize=648</td><td>gi|6900972|ref|Os03g10290|</td></tr>
<tr><td>gi|55238520|gb|EAL39896.1|</td><td>lcl|T07D1.5 CE36321 WBGene00020303 status:Predicted SW:Q95QB4 protein_id:AAR85903.1 /QuerySize=210</td><td>gi|6900974|ref|Os03g10310|</td></tr>
<tr><td>gi|55238525|gb|EAL39898.1|</td><td>lcl|T07D10.1 CE21161 WBGene00011581 status:Partially_confirmed SW:O45765 protein_id:CAB04716.1 /QuerySize=281</td><td>gi|6900976|ref|Os03g10330|</td></tr>
<tr><td>gi|55238541|gb|EAA10955.2|</td><td>lcl|T07D10.3 CE34209 WBGene00011583 zinc finger protein status:Partially_confirmed SW:O02303 protein_id:CAB04715.2 /Quer</td><td>gi|6900981|ref|Os03g10380|</td></tr>
<tr><td>gi|55238548|gb|EAA10874.3|</td><td>lcl|T07D10.6 CE33541 WBGene00011586 status:Confirmed SW:Q86G91 protein_id:CAD82918.1 /QuerySize=86</td><td>gi|6900989|ref|Os03g10430|</td></tr>
<tr><td>gi|55238556|gb|EAL39908.1|</td><td>lcl|T07E3.2 CE01383 WBGene00020313 status:Partially_confirmed SW:Q22313 protein_id:AAA21081.1 /QuerySize=89</td><td>gi|6900995|ref|Os03g10480|</td></tr>
<tr><td>gi|55238564|gb|EAA10879.2|</td><td>lcl|T07E3.3 CE01384 WBGene00020314 status:Predicted SW:Q22312 protein_id:AAA21082.1 /QuerySize=312</td><td>gi|6901000|ref|Os03g10530|</td></tr>
<tr><td>gi|55238565|gb|EAL39912.1|</td><td>lcl|T07E3.6a CE30165 WBGene00020317 status:Partially_confirmed SW:Q8WQD1 protein_id:AAL56619.1 /QuerySize=182</td><td>gi|6901003|ref|Os03g10550|</td></tr>
<tr><td>gi|55238567|gb|EAA10871.2|</td><td>lcl|T07E3.6b CE30166 WBGene00020317 status:Partially_confirmed SW:Q8WQD2 protein_id:AAL56620.1 /QuerySize=180</td><td>gi|6901004|ref|Os03g10560|</td></tr>
<tr><td>gi|55238568|gb|EAL39913.1|</td><td>lcl|T07F10.2 CE21163 WBGene00011588 status:Partially_confirmed SW:Q22320 protein_id:CAB01245.1 /QuerySize=176</td><td>gi|6901006|ref|Os03g10580|</td></tr>
<tr><td>gi|55238577|gb|EAL39915.1|</td><td>lcl|T07F10.5 CE06398 WBGene00011591 status:Partially_confirmed SW:Q22319 protein_id:CAB01244.1 /QuerySize=59</td><td>gi|6901007|ref|Os03g10590|</td></tr>
<tr><td>gi|55238589|gb|EAL39918.1|</td><td>lcl|T07F8.3a CE28651 WBGene00001597 locus:gld-3 status:Partially_confirmed SW:Q95ZK7 protein_id:AAK77629.1 /QuerySize=</td><td>gi|6901011|ref|Os03g10630|</td></tr>
<tr><td>gi|55238590|gb|EAA10875.2|</td><td>lcl|T07F8.3b CE28652 WBGene00001597 locus:gld-3 status:Partially_confirmed SW:Q95ZK6 protein_id:AAK77630.1 /QuerySize=</td><td>gi|6901014|ref|Os03g10660|</td></tr>
<tr><td>gi|55238592|gb|EAL39920.1|</td><td>lcl|T07G12.10 CE29521 WBGene00011600 Zinc finger</td><td>gi|6901015|ref|Os03g10670|</td></tr>
<tr><td>gi|55238614|gb|EAL39932.1|</td><td>lcl|T07G12.12 CE36715 WBGene00011602 Zinc finger</td><td>gi|6901018|ref|Os03g10700|</td></tr>
<tr><td>gi|55238624|gb|EAA44082.2|</td><td>lcl|T07G12.13 CE35614 WBGene00011603 status:Partially_confirmed SW:Q8WQD0 protein_id:CAD18876.2 /QuerySize=218</td><td>gi|6901019|ref|Os03g10710|</td></tr>
<tr><td>gi|55238625|gb|EAA10840.2|</td><td>lcl|T07G12.6 CE13397 WBGene00011597 status:Partially_confirmed SW:O18064 protein_id:CAB05277.1 /QuerySize=380</td><td>gi|6901021|ref|Os03g10730|</td></tr>
<tr><td>gi|55238633|gb|EAL39941.1|</td><td>lcl|T07G12.8 CE13401 WBGene00011599 status:Partially_confirmed SW:O18060 protein_id:CAB05273.1 /QuerySize=147</td><td>gi|6901022|ref|Os03g10740|</td></tr>
<tr><td>gi|55238639|gb|EAL39943.1|</td><td>lcl|T08A9.11 CE31230 WBGene00020339 status:Confirmed SW:Q8MPX6 protein_id:AAM69084.1 /QuerySize=34</td><td>gi|6901024|ref|Os03g10760|</td></tr>
<tr><td>gi|55238640|gb|EAA44088.2|</td><td>lcl|T08A9.5 CE04920 WBGene00020337 status:Predicted SW:Q22334 protein_id:AAA81412.1 /QuerySize=157</td><td>gi|6901031|ref|Os03g10830|</td></tr>
<tr><td>gi|55238653|gb|EAL39944.1|</td><td>lcl|T08A9.6 CE04921 WBGene00020338 status:Partially_confirmed SW:Q22333 protein_id:AAA81411.1 /QuerySize=114</td><td>gi|6901032|ref|Os03g10840|</td></tr>
<tr><td>gi|55238657|gb|EAA11548.2|</td><td>lcl|T08B1.5 CE35762 WBGene00020342 status:Confirmed SW:O44559 protein_id:AAB94179.2 /QuerySize=146</td><td>gi|6901044|ref|Os03g10960|</td></tr>
<tr><td>gi|55238675|gb|EAL39949.1|</td><td>lcl|T08B2.11 CE13439 WBGene00020349 status:Partially_confirmed SW:Q9GYS6 protein_id:AAG00018.1 /QuerySize=416</td><td>gi|6901045|ref|Os03g10970|</td></tr>
<tr><td>gi|55238679|gb|EAA11530.2|</td><td>lcl|T08B2.12 CE13441 WBGene00020350 status:Predicted SW:Q9GYS3 protein_id:AAG00019.1 /QuerySize=152</td><td>gi|6901048|ref|Os03g11000|</td></tr>
<tr><td>gi|55238700|gb|EAA11042.3|</td><td>lcl|T08B2.3 CE13423 WBGene00020344 status:Predicted SW:Q9GYS4 protein_id:AAG00013.1 /QuerySize=179</td><td>gi|6901057|ref|Os03g11080|</td></tr>
<tr><td>gi|55238701|gb|EAA11052.3|</td><td>lcl|T08B2.4 CE13425 WBGene00020345 status:Predicted SW:Q9GYS5 protein_id:AAG00014.1 /QuerySize=119</td><td>gi|6901058|ref|Os03g11090|</td></tr>
<tr><td>gi|55238723|gb|EAA11053.2|</td><td>lcl|T08B2.5d CE13429 WBGene00020346 status:Confirmed SW:Q8MNW1 protein_id:AAM22070.1 /QuerySize=163</td><td>gi|6901065|ref|Os03g11150|</td></tr>
<tr><td>gi|55238743|gb|EAL39960.1|</td><td>lcl|T08B2.5e CE33170 WBGene00020346 status:Confirmed SW:Q86NK1 protein_id:AAO21391.1 /QuerySize=185</td><td>gi|6901069|ref|Os03g11190|</td></tr>
<tr><td>gi|55238748|gb|EAL39961.1|</td><td>lcl|T08B6.1 CE30300 WBGene00020351 status:Partially_confirmed SW:O44531 protein_id:AAB94153.2 /QuerySize=96</td><td>gi|6901077|ref|Os03g11270|</td></tr>
<tr><td>gi|55238762|gb|EAA11692.2|</td><td>lcl|T08B6.2 CE17223 WBGene00020352 status:Predicted SW:O44529 protein_id:AAB94154.1 /QuerySize=388</td><td>gi|6901078|ref|Os03g11280|</td></tr>
<tr><td>gi|55238763|gb|EAA11805.3|</td><td>lcl|T08B6.4 CE37550 WBGene00020353 status:Partially_confirmed SW:O44527 protein_id:AAB94160.2 /QuerySize=631</td><td>gi|6901090|ref|Os03g11390|</td></tr>
<tr><td>gi|55238783|gb|EAA11247.2|</td><td>lcl|T08B6.9 CE33691 WBGene00020356 status:Partially_confirmed SW:Q86MH6 protein_id:AAO91700.1 /QuerySize=531</td><td>gi|6901099|ref|Os03g11480|</td></tr>
<tr><td>gi|55238786|gb|EAL39968.1|</td><td>lcl|T08D10.3 CE03644 WBGene00011616 status:Predicted SW:Q22344 protein_id:CAA90638.1 /QuerySize=473</td><td>gi|6901117|ref|Os03g11640|</td></tr>
<tr><td>gi|55238799|gb|EAA11256.2|</td><td>lcl|T08D2.5 CE21171 WBGene00011610 status:Predicted SW:Q9XTX8 protein_id:CAB07414.1 /QuerySize=325</td><td>gi|6901129|ref|Os03g11740|</td></tr>
<tr><td>gi|55238819|gb|EAA11250.2|</td><td>lcl|T08E11.5 CE33448 WBGene00020361 status:Partially_confirmed SW:O76639 protein_id:AAC26318.3 /QuerySize=347</td><td>gi|6901130|ref|Os03g11750|</td></tr>
<tr><td>gi|55238834|gb|EAA44135.2|</td><td>lcl|T08G11.3 CE13447 WBGene00011630 status:Partially_confirmed SW:Q94012 protein_id:CAB02306.1 /QuerySize=601</td><td>gi|6901136|ref|Os03g11800|</td></tr>
<tr><td>gi|55238838|gb|EAA10995.2|</td><td>lcl|T08G3.11 CE18942 WBGene00011620 status:Predicted SW:Q9XU54 protein_id:CAB05790.1 /QuerySize=108</td><td>gi|6901137|ref|Os03g11810|</td></tr>
<tr><td>gi|55238840|gb|EAA10991.2|</td><td>lcl|T08G3.6 CE18938 WBGene00011618 status:Predicted SW:Q9XU51 protein_id:CAB05796.1 /QuerySize=304</td><td>gi|6901138|ref|Os03g11820|</td></tr>
<tr><td>gi|55238845|gb|EAL39983.1|</td><td>lcl|T08G5.1 CE18943 WBGene00011622 status:Partially_confirmed SW:Q9XUQ7 protein_id:CAB04717.1 /QuerySize=272</td><td>gi|6901140|ref|Os03g11840|</td></tr>
<tr><td>gi|55238846|gb|EAL39984.1|</td><td>lcl|T08G5.10 CE25109 WBGene00003474 locus:mtl-2 status:Confirmed SW:P17512 protein_id:CAB61028.1 /QuerySize=63</td><td>gi|6901142|ref|Os03g11860|</td></tr>
<tr><td>gi|55238849|gb|EAL39985.1|</td><td>lcl|T08G5.3 CE18945 WBGene00011624 status:Partially_confirmed SW:Q9XUQ6 protein_id:CAB04718.1 /QuerySize=215</td><td>gi|6901148|ref|Os03g11920|</td></tr>
<tr><td>gi|55238862|gb|EAA10992.2|</td><td>lcl|T08G5.7 CE18947 WBGene00011626 Zinc finger</td><td>gi|6901149|ref|Os03g11930|</td></tr>
<tr><td>gi|55238873|gb|EAA11002.2|</td><td>lcl|T08H10.3 CE07476 WBGene00020370 status:Partially_confirmed SW:Q22349 protein_id:AAA97989.1 /QuerySize=168</td><td>gi|6901150|ref|Os03g11940|</td></tr>
<tr><td>gi|55238874|gb|EAL39994.1|</td><td>lcl|T08H4.2 CE29471 WBGene00020367 status:Partially_confirmed SW:Q22353 protein_id:AAC71119.2 /QuerySize=189</td><td>gi|6901157|ref|Os03g12010|</td></tr>
<tr><td>gi|55238879|gb|EAL39995.1|</td><td>lcl|T09A12.1 CE35002 WBGene00020372 status:Partially_confirmed SW:O61221 protein_id:AAC04433.3 /QuerySize=339</td><td>gi|6901160|ref|Os03g12040|</td></tr>
<tr><td>gi|55238888|gb|EAL39999.1|</td><td>lcl|T09A5.12 CE03646 WBGene00011639 Zinc finger</td><td>gi|6901161|ref|Os03g12050|</td></tr>
<tr><td>gi|55238889|gb|EAA44154.2|</td><td>lcl|T09A5.14 CE35003 WBGene00011640 status:Predicted SW:Q7YTK8 protein_id:CAE17918.1 /QuerySize=68</td><td>gi|6901162|ref|Os03g12060|</td></tr>
<tr><td>gi|55238890|gb|EAA11608.2|</td><td>lcl|T09A5.15 CE35004 WBGene00011641 status:Partially_confirmed SW:Q7YTK7 protein_id:CAE17919.1 /QuerySize=92</td><td>gi|6901165|ref|Os03g12080|</td></tr>
<tr><td>gi|55238892|gb|EAA11609.3|</td><td>lcl|T09A5.2b CE36407 WBGene00002216 locus:klp-3 status:Partially_confirmed SW:Q7JMP3 protein_id:CAF31496.1 /QuerySize=</td><td>gi|6901170|ref|Os03g12130|</td></tr>
<tr><td>gi|55238904|gb|EAL40001.1|</td><td>lcl|T09A5.2c CE36408 WBGene00002216 locus:klp-3 status:Partially_confirmed SW:Q7JMP4 protein_id:CAF31495.1 /QuerySize=</td><td>gi|6901174|ref|Os03g12170|</td></tr>
<tr><td>gi|55238906|gb|EAA11088.3|</td><td>lcl|T09A5.4 CE01085 WBGene00011633 status:Partially_confirmed SW:P45964 protein_id:CAA85332.1 /QuerySize=297</td><td>gi|6901178|ref|Os03g12210|</td></tr>
<tr><td>gi|55238911|gb|EAL40003.1|</td><td>lcl|T09A5.5 CE01086 WBGene00011634 status:Confirmed SW:P45965 protein_id:CAA85333.1 /QuerySize=163</td><td>gi|6901185|ref|Os03g12280|</td></tr>
<tr><td>gi|55238915|gb|EAL40004.1|</td><td>lcl|T09B4.3 CE13461 WBGene00020377 status:Partially_confirmed SW:O02160 protein_id:AAB53006.1 /QuerySize=125</td><td>gi|6901190|ref|Os03g12330|</td></tr>
<tr><td>gi|55238922|gb|EAL40005.1|</td><td>lcl|T09B4.5a CE13465 WBGene00020379 status:Confirmed SW:O02155 protein_id:AAB53007.1 /QuerySize=327</td><td>gi|6901199|ref|Os03g12380|</td></tr>
<tr><td>gi|55238924|gb|EAL40006.1|</td><td>lcl|T09B4.5b CE33175 WBGene00020379 status:Confirmed SW:Q86NC3 protein_id:AAO21395.1 /QuerySize=77</td><td>gi|6901201|ref|Os03g12400|</td></tr>
<tr><td>gi|55238932|gb|EAL40008.1|</td><td>lcl|T09D3.3 CE13481 WBGene00020384 status:Partially_confirmed SW:Q23036 protein_id:AAG24196.1 /QuerySize=592</td><td>gi|6901215|ref|Os03g12540|</td></tr>
<tr><td>gi|55238940|gb|EAA11224.2|</td><td>lcl|T09E8.1a CE31987 WBGene00011647 status:Confirmed SW:Q7YSP6 protein_id:CAB54298.2 /QuerySize=747</td><td>gi|6901218|ref|Os03g12560|</td></tr>
<tr><td>gi|55238944|gb|EAA44169.2|</td><td>lcl|T09E8.1b CE31988 WBGene00011647 status:Confirmed SW:P90906 protein_id:CAB54297.2 /QuerySize=689</td><td>gi|6901220|ref|Os03g12580|</td></tr>
<tr><td>gi|55238956|gb|EAL40016.1|</td><td>lcl|T09E8.1c CE31989 WBGene00011647 status:Partially_confirmed SW:Q8I0T9 protein_id:CAD54155.1 /QuerySize=771</td><td>gi|6901225|ref|Os03g12630|</td></tr>
<tr><td>gi|55238960|gb|EAL40017.1|</td><td>lcl|T09E8.1d CE31990 WBGene00011647 status:Confirmed SW:Q8I4G1 protein_id:CAD54154.1 /QuerySize=547</td><td>gi|6901226|ref|Os03g12640|</td></tr>
<tr><td>gi|55238963|gb|EAA10821.2|</td><td>lcl|T09E8.1e CE31991 WBGene00011647 status:Partially_confirmed SW:Q8I0Q4 protein_id:CAD54156.1 /QuerySize=507</td><td>gi|6901227|ref|Os03g12650|</td></tr>
<tr><td>gi|55238964|gb|EAL40018.1|</td><td>lcl|T09E8.2 CE13489 WBGene00001874 locus:him-17 status:Partially_confirmed SW:Q22360 protein_id:CAB01515.1 /QuerySize=</td><td>gi|6901229|ref|Os03g12670|</td></tr>
<tr><td>gi|55238965|gb|EAA11845.3|</td><td>lcl|T09F3.4 CE35187 WBGene00011663 status:Predicted SW:Q22363 protein_id:CAA88871.2 /QuerySize=223</td><td>gi|6901231|ref|Os03g12690|</td></tr>
<tr><td>gi|55238966|gb|EAA11236.3|</td><td>lcl|T09F5.2 CE13523 WBGene00011666 status:Predicted SW:O62373 protein_id:CAB05802.1 /QuerySize=182</td><td>gi|6901238|ref|Os03g12750|</td></tr>
<tr><td>gi|55238967|gb|EAL40019.1|</td><td>lcl|T09F5.7 CE13533 WBGene00011667 reverse transcriptase status:Predicted SW:O18073 protein_id:CAB05805.1 /QuerySize=2</td><td>gi|6901240|ref|Os03g12770|</td></tr>
<tr><td>gi|55238968|gb|EAA11220.2|</td><td>lcl|T10B10.6 CE06404 WBGene00011683 status:Partially_confirmed SW:Q22373 protein_id:CAA96678.1 /QuerySize=204</td><td>gi|6901241|ref|Os03g12780|</td></tr>
<tr><td>gi|55238977|gb|EAL40020.1|</td><td>lcl|T10B10.9 CE23964 WBGene00011685 status:Partially_confirmed SW:Q9U374 protein_id:CAB54309.1 /QuerySize=106</td><td>gi|6901243|ref|Os03g12800|</td></tr>
<tr><td>gi|55239002|gb|EAL40025.1|</td><td>lcl|T10B11.4 CE18243 WBGene00020400 status:Predicted SW:Q9TZI0 protein_id:AAC67464.1 /QuerySize=360</td><td>gi|6901246|ref|Os03g12830|</td></tr>
<tr><td>gi|55239004|gb|EAL40027.1|</td><td>lcl|T10B11.7 CE34365 WBGene00020403 status:Partially_confirmed SW:Q9TZH7 protein_id:AAC67469.2 /QuerySize=562</td><td>gi|6901248|ref|Os03g12850|</td></tr>
<tr><td>gi|55239007|gb|EAA11116.2|</td><td>lcl|T10B11.8a CE37405 WBGene00020404 status:Confirmed SW:Q9TZH5 protein_id:AAC67463.3 /QuerySize=469</td><td>gi|6901250|ref|Os03g12870|</td></tr>
<tr><td>gi|55239008|gb|EAA11578.2|</td><td>lcl|T10B11.8b CE33178 WBGene00020404 status:Partially_confirmed SW:Q86S23 protein_id:AAO21396.1 /QuerySize=421</td><td>gi|6901251|ref|Os03g12880|</td></tr>
<tr><td>gi|55239009|gb|EAA11574.2|</td><td>lcl|T10B5.10 CE33314 WBGene00020396 status:Partially_confirmed SW:Q86S30 protein_id:AAO25992.1 /QuerySize=255</td><td>gi|6901256|ref|Os03g12920|</td></tr>
<tr><td>gi|55239010|gb|EAA11124.2|</td><td>lcl|T10B5.2 CE36197 WBGene00020388 status:Partially_confirmed SW:O76408 protein_id:AAO25993.2 /QuerySize=380</td><td>gi|6901260|ref|Os03g12960|</td></tr>
<tr><td>gi|55239011|gb|EAA11117.2|</td><td>lcl|T10B5.6 CE18236 WBGene00020392 locus:knl-3 status:Partially_confirmed SW:O76406 protein_id:AAC19225.1 /QuerySize=3</td><td>gi|6901261|ref|Os03g12970|</td></tr>
</table>

gi|55239012|gb|EAL40028.1|	lcl|T10B9.9 CE31992 WBGene00011678 status:Confirmed SW:Q09352 protein_id:CAA88608.2 /QuerySize=124	gi|6901262|ref|Os03g12980|
gi|55239013|gb|EAA11121.2|	lcl|T10D4.11 CE20051 WBGene00020410 status:Predicted SW:Q9TZE7 protein_id:AAC68721.1 /QuerySize=250	gi|6901266|ref|Os03g13020|
gi|55239014|gb|EAL40029.1|	lcl|T10E9.2 CE13545 WBGene00020412 status:Partially_confirmed SW:O01605 protein_id:AAB52470.1 /QuerySize=720	gi|6901271|ref|Os03g13060|
gi|55239023|gb|EAA44188.2|	lcl|T10E9.5 CE13551 WBGene00020415 status:Predicted SW:O01599 protein_id:AAB52468.1 /QuerySize=237	gi|6901277|ref|Os03g13120|
gi|55239025|gb|EAA11596.2|	lcl|T10E9.8 CE13557 WBGene00020418 status:Partially_confirmed SW:O01604 protein_id:AAB52475.1 /QuerySize=225	gi|6901286|ref|Os03g13190|
gi|55239049|gb|EAL40035.1|	lcl|T10G3.1 CE31065 WBGene00011692 status:Confirmed SW:P92018 protein_id:CAB03324.2 /QuerySize=318	gi|6901296|ref|Os03g13290|
gi|55239062|gb|EAL40038.1|	lcl|T10G3.2 CE13563 WBGene00011693 status:Partially_confirmed SW:P92019 protein_id:CAB03325.1 /QuerySize=346	gi|6901300|ref|Os03g13330|
gi|55239063|gb|EAL40039.1|	lcl|T10G3.3 CE37988 WBGene00011694 status:Partially_confirmed SW:P92020 protein_id:CAB03326.2 /QuerySize=297	gi|6901301|ref|Os03g13340|
gi|55239065|gb|EAL40040.1|	lcl|T10G3.4 CE13567 WBGene00011695 7TM receptor-like status:Predicted SW:P92166 protein_id:CAB03327.1 /QuerySize=309	gi|6901303|ref|Os03g13360|
gi|55239070|gb|EAL40042.1|	lcl|T11A5.1 CE06407 WBGene00011700 status:Predicted SW:Q22381 protein_id:CAA96681.1 /QuerySize=419	gi|6901309|ref|Os03g13410|
gi|55239087|gb|EAL40044.1|	lcl|T11A5.2 CE36198 WBGene00011701 locus:srab-17 status:Partially_confirmed SW:Q22382 protein_id:CAA96682.3 /QuerySize	gi|6901311|ref|Os03g13430|
gi|55239091|gb|EAL40046.1|	lcl|T11A5.3 CE34092 WBGene00011702 locus:srab-18 status:Partially_confirmed SW:Q22383 protein_id:CAA96683.2 /QuerySize	gi|6901316|ref|Os03g13470|
gi|55239099|gb|EAL40050.1|	lcl|T11A5.4 CE37552 WBGene00011703 locus:srab-19 status:Partially_confirmed SW:Q22384 protein_id:CAA96684.4 /QuerySize	gi|6901319|ref|Os03g13500|
gi|55239101|gb|EAL40051.1|	lcl|T11A5.5 CE06411 WBGene00011704 status:Partially_confirmed SW:Q22385 protein_id:CAA96685.1 /QuerySize=288	gi|6901320|ref|Os03g13510|
gi|55239104|gb|EAL40052.1|	lcl|T11B7.2 CE03648 WBGene00011707 status:Partially_confirmed SW:Q22388 protein_id:CAA90988.1 /QuerySize=94	gi|6901323|ref|Os03g13530|
gi|55239134|gb|EAA10825.2|	lcl|T11B7.5 CE03651 WBGene00011708 status:Confirmed SW:Q22390 protein_id:CAA90990.1 /QuerySize=155	gi|6901328|ref|Os03g13580|
gi|55239135|gb|EAL40059.1|	lcl|T11F1.3 CE20054 WBGene00020429 status:Predicted SW:Q9U8C6 protein_id:AAC68710.1 /QuerySize=96	gi|6901330|ref|Os03g13600|
gi|55239137|gb|EAL40061.1|	lcl|T11F8.1 CE17234 WBGene00020433 status:Partially_confirmed SW:O44194 protein_id:AAB88555.1 /QuerySize=261	gi|6901331|ref|Os03g13600|
gi|55239141|gb|EAA11500.2|	lcl|T11F8.2 CE35005 WBGene00020434 status:Partially_confirmed SW:O44193 protein_id:AAB88556.2 /QuerySize=424	gi|6901335|ref|Os03g13640|
gi|55239143|gb|EAL40062.1|	lcl|T11F9.10 CE06422 WBGene00011712 status:Partially_confirmed SW:Q22394 protein_id:CAA98526.1 /QuerySize=325	gi|6901337|ref|Os03g13660|
gi|55239144|gb|EAA11506.2|	lcl|T11F9.12 CE18953 WBGene00011713 status:Partially_confirmed SW:Q22402 protein_id:CAA98537.1 /QuerySize=688	gi|6901353|ref|Os03g13810|
gi|55239148|gb|EAL40065.1|	lcl|T11F9.13 CE06425 WBGene00011714 status:Predicted SW:Q22404 protein_id:CAA98535.1 /QuerySize=116	gi|6901354|ref|Os03g13810|
gi|55239149|gb|EAL40066.1|	lcl|T11F9.14 CE06426 WBGene00011715 status:Predicted SW:Q22403 protein_id:CAA98534.1 /QuerySize=45	gi|6901360|ref|Os03g13870|
gi|55239156|gb|EAL40067.1|	lcl|T11F9.19 CE35007 WBGene00011716 status:Predicted SW:Q7YWV9 protein_id:CAE17912.1 /QuerySize=227	gi|6901373|ref|Os03g13980|
gi|55239158|gb|EAL40068.1|	lcl|T11F9.7 CE06419 WBGene00011710 status:Partially_confirmed SW:Q22399 protein_id:CAA98531.1 /QuerySize=249	gi|6901374|ref|Os03g13990|
gi|55239162|gb|EAL40069.1|	lcl|T11F9.8 CE06420 WBGene00011711 zinc metalloprotease status:Partially_confirmed SW:Q22400 protein_id:CAA98532.1 /Que	gi|6901385|ref|Os03g14070|
gi|55239169|gb|EAL40072.1|	lcl|T11G6.5 CE32932 WBGene00011720 status:Partially_confirmed SW:Q22411 protein_id:CAA93420.2 /QuerySize=1406	gi|6901389|ref|Os03g14110|
gi|55239172|gb|EAA11191.3|	lcl|T11G6.7 CE06431 WBGene00011721 status:Partially_confirmed SW:Q22407 protein_id:CAA93415.1 /QuerySize=75	gi|6901396|ref|Os03g14160|
gi|55239173|gb|EAA11154.3|	lcl|T12A2.3 CE01396 WBGene00020438 status:Partially_confirmed SW:Q22415 protein_id:AAC24443.1 /QuerySize=206	gi|6901400|ref|Os03g14200|
gi|55239175|gb|EAL40073.1|	lcl|T12A2.5 CE01398 WBGene00020439 status:Predicted SW:Q22414 protein_id:AAC24444.1 /QuerySize=994	gi|6901402|ref|Os03g14220|
gi|55239183|gb|EAA11156.2|	lcl|T12A2.6 CE30395 WBGene00020440 status:Partially_confirmed SW:Q22416 protein_id:AAC24445.2 /QuerySize=689	gi|6901403|ref|Os03g14230|
gi|55239205|gb|EAL40079.1|	lcl|T12A7.2 CE23979 WBGene00011723 status:Partially_confirmed SW:Q22423 protein_id:CAA98142.2 /QuerySize=483	gi|6901404|ref|Os03g14240|
gi|55239208|gb|EAL40080.1|	lcl|T12A7.6 CE27446 WBGene00011727 status:Partially_confirmed SW:Q95ZP0 protein_id:CAC42338.1 /QuerySize=350	gi|6901410|ref|Os03g14300|
gi|55239209|gb|EAL40081.1|	lcl|T12C9.7 CE28662 WBGene00020461 status:Partially_confirmed SW:Q95QB2 protein_id:AAK77633.1 /QuerySize=284	gi|6901411|ref|Os03g14310|
gi|55239213|gb|EAA11094.3|	lcl|T12D8.5 CE33832 WBGene00011733 status:Confirmed SW:O45783 protein_id:CAB03345.2 /QuerySize=135	gi|6901412|ref|Os03g14310|
gi|55239230|gb|EAL40087.1|	lcl|T12D8.9a CE34093 WBGene00011736 status:Confirmed SW:O45785 protein_id:CAB05819.3 /QuerySize=1181	gi|6901413|ref|Os03g14320|
gi|55239232|gb|EAL40088.1|	lcl|T12D8.9b CE37146 WBGene00011736 status:Confirmed SW:Q69YY4 protein_id:CAH10791.1 /QuerySize=1183	gi|6901414|ref|Os03g14330|
gi|55239236|gb|EAL40089.1|	lcl|T12E12.3 CE13587 WBGene00020464 status:Partially_confirmed SW:Q22429 protein_id:AAB03119.1 /QuerySize=818	gi|6901422|ref|Os03g14390|
gi|55239237|gb|EAA11105.2|	lcl|T12F5.1 CE13595 WBGene00020466 status:Partially_confirmed SW:O44761 protein_id:AAB96743.1 /QuerySize=249	gi|6901426|ref|Os03g14430|
gi|55239238|gb|EAA11099.2|	lcl|T12F5.2 CE13597 WBGene00020467 status:Confirmed SW:O44759 protein_id:AAB96744.1 /QuerySize=329	gi|6901427|ref|Os03g14440|
gi|55239245|gb|EAL40092.1|	lcl|T12G3.2a CE06439 WBGene00011738 status:Partially_confirmed SW:Q22435 protein_id:CAA92981.1 /QuerySize=928	gi|6901431|ref|Os03g14480|
gi|55239253|gb|EAL40094.1|	lcl|T12G3.2b CE36199 WBGene00011738 status:Partially_confirmed SW:Q7JLZ0 protein_id:CAE54904.1 /QuerySize=912	gi|6901440|ref|Os03g14570|
gi|55239256|gb|EAL40096.1|	lcl|T12G3.2c CE36200 WBGene00011738 status:Partially_confirmed SW:Q7JLY9 protein_id:CAE54905.1 /QuerySize=888	gi|6901446|ref|Os03g14620|
gi|55239257|gb|EAL40097.1|	lcl|T12G3.6 CE06442 WBGene00011741 status:Partially_confirmed SW:Q22434 protein_id:CAA92980.1 /QuerySize=217	gi|6901447|ref|Os03g14630|
gi|55239258|gb|EAA11092.2|	lcl|T12G3.7 CE18257 WBGene00011742 status:Partially_confirmed SW:O62430 protein_id:CAA19510.1 /QuerySize=354	gi|6901448|ref|Os03g14640|
gi|55239269|gb|EAL40101.1|	lcl|T13A10.1 CE07483 WBGene00020468 status:Partially_confirmed SW:Q22449 protein_id:AAB38121.1 /QuerySize=155	gi|6901449|ref|Os03g14650|
gi|55239270|gb|EAL40102.1|	lcl|T13A10.5a CE31727 WBGene00003754 locus:nlp-16 status:Confirmed SW:Q22442 protein_id:AAB38119.2 /QuerySize=326	gi|6901452|ref|Os03g14680|
gi|55239271|gb|EAL40103.1|	lcl|T13B5.8 CE36017 WBGene00020475 status:Partially_confirmed SW:O17378 protein_id:AAB71291.2 /QuerySize=234	gi|6901458|ref|Os03g14740|
gi|55239274|gb|EAA11564.2|	lcl|T13C2.2 CE37046 WBGene00020478 status:Confirmed SW:Q6AHP7 protein_id:AAT81189.1 /QuerySize=525	gi|6901462|ref|Os03g14780|
gi|55239280|gb|EAL40108.1|	lcl|T13C2.3a CE33451 WBGene00020479 status:Partially_confirmed SW:Q22454 protein_id:AAA81135.3 /QuerySize=431	gi|6901466|ref|Os03g14820|
gi|55239296|gb|EAA11565.2|	lcl|T13C2.3b CE36018 WBGene00020479 status:Partially_confirmed SW:Q7JP78 protein_id:AAR12976.1 /QuerySize=474	gi|6901471|ref|Os03g14870|
gi|55239300|gb|EAA11291.2|	lcl|T13C2.7 CE34371 WBGene00020482 status:Predicted SW:Q7JP79 protein_id:AAR12978.1 /QuerySize=298	gi|6901499|ref|Os03g15060|
gi|55239318|gb|EAA10947.2|	lcl|T13F2.4 CE13625 WBGene00011744 status:Predicted SW:Q94047 protein_id:CAB03355.1 /QuerySize=233	gi|6901500|ref|Os03g15070|
gi|55239319|gb|EAA10937.2|	lcl|T13F2.6 CE13629 WBGene00011746 status:Partially_confirmed SW:Q94049 protein_id:CAB03357.1 /QuerySize=579	gi|6901501|ref|Os03g15080|
gi|55239320|gb|EAL40116.1|	lcl|T13F2.7 CE13631 WBGene00011747 status:Confirmed SW:Q94050 protein_id:CAB03358.1 /QuerySize=663	gi|6901502|ref|Os03g15090|
gi|55239324|gb|EAL40117.1|	lcl|T13F2.9 CE13635 WBGene00011748 status:Partially_confirmed SW:Q94052 protein_id:CAB03360.1 /QuerySize=255	gi|6901503|ref|Os03g15100|
gi|55239325|gb|EAL40118.1|	lcl|T13F3.6 CE16412 WBGene00011753 status:Partially_confirmed SW:O18089 protein_id:CAB07673.1 /QuerySize=191	gi|6901507|ref|Os03g15130|
gi|55239332|gb|EAL40120.1|	lcl|T13F3.7 CE35011 WBGene00011754 status:Confirmed SW:Q7YTK5 protein_id:CAE17923.1 /QuerySize=324	gi|6901509|ref|Os03g15150|
gi|55239336|gb|EAA10974.3|	lcl|T13G4.5 CE25996 WBGene00020492 status:Predicted SW:Q966F4 protein_id:AAK84574.1 /QuerySize=264	gi|6901511|ref|Os03g15170|
gi|55239337|gb|EAL40122.1|	lcl|T13G4.6 CE21181 WBGene00020493 status:Predicted SW:Q966F3 protein_id:AAK84575.1 /QuerySize=254	gi|6901514|ref|Os03g15200|
gi|55239350|gb|EAL40124.1|	lcl|T13G4.7 CE25995 WBGene00020494 status:Predicted SW:Q966F2 protein_id:AAK84576.1 /QuerySize=65	gi|6901520|ref|Os03g15260|
gi|55239351|gb|EAA44263.2|	lcl|T13H2.3 CE31236 WBGene00020495 zinc finger containing protein status:Partially_confirmed SW:Q22463 protein_id:AAM6906	gi|6901522|ref|Os03g15280|
gi|55239358|gb|EAA10952.2|	lcl|T13H2.4a CE30179 WBGene00004148 locus:pqn-65 status:Partially_confirmed SW:Q8WQC8 protein_id:AAL56623.1 /QuerySi	gi|6901524|ref|Os03g15300|
gi|55239384|gb|EAA44268.2|	lcl|T13H2.4b CE30180 WBGene00004148 locus:pqn-65 status:Partially_confirmed SW:Q8WQC7 protein_id:AAL56624.1 /QuerySi	gi|6901525|ref|Os03g15310|
gi|55239415|gb|EAA11489.3|	lcl|T13H5.6 CE23982 WBGene00011760 status:Partially_confirmed SW:Q9U369 protein_id:CAB54303.1 /QuerySize=475	gi|6901527|ref|Os03g15330|
gi|55239419|gb|EAL40133.1|	lcl|T14A8.1 CE27448 WBGene00004363 locus:ric-3 status:Partially_confirmed SW:Q22472 protein_id:AAA93437.2 /QuerySize=37	gi|6901540|ref|Os03g15460|
gi|55239421|gb|EAA11492.3|	lcl|T14A8.2 CE29475 WBGene00020497 status:Partially_confirmed SW:Q22473 protein_id:AAA93438.2 /QuerySize=202	gi|6901541|ref|Os03g15470|
gi|55239423|gb|EAL40134.1|	lcl|T14B1.1 CE01662 WBGene00011763 status:Confirmed SW:Q22474 protein_id:CAA86862.1 /QuerySize=375	gi|6901543|ref|Os03g15490|
gi|55239426|gb|EAA11320.3|	lcl|T14B4.5 CE02048 WBGene00020501 status:Partially_confirmed SW:Q22478 protein_id:AAK31553.1 /QuerySize=194	gi|6901544|ref|Os03g15500|
gi|55239431|gb|EAA11402.2|	lcl|T14B4.8 CE02051 WBGene00020502 status:Partially_confirmed SW:Q22480 protein_id:AAK31554.1 /QuerySize=304	gi|6901547|ref|Os03g15530|
gi|55239432|gb|EAL40135.1|	lcl|T14C1.2 CE06446 WBGene00011766 reverse transcriptase status:Predicted SW:Q22486 protein_id:CAA94617.1 /QuerySize=8	gi|6901558|ref|Os03g15640|
gi|55239433|gb|EAA11502.2|	lcl|T14E8.4 CE37676 WBGene00043981 status:Predicted SW:Q5WRS0 protein_id:AAV28332.1 /QuerySize=475	gi|6901561|ref|Os03g15670|
gi|55239436|gb|EAA11485.2|	lcl|T14F9.2 CE07498 WBGene00020508 status:Partially_confirmed SW:Q22493 protein_id:AAA91265.1 /QuerySize=395	gi|6901573|ref|Os03g15760|
gi|55239447|gb|EAA11268.2|	lcl|T14G10.8 CE06454 WBGene00011777 status:Confirmed SW:Q22497 protein_id:CAA93094.1 /QuerySize=181	gi|6901581|ref|Os03g15820|
gi|55239448|gb|EAL40140.1|	lcl|T14G11.1 CE04959 WBGene00020510 status:Predicted SW:Q22506 protein_id:AAA82346.1 /QuerySize=91	gi|6901599|ref|Os03g15980|
gi|55239453|gb|EAL40143.1|	lcl|T14G12.3 CE04964 WBGene00006408 locus:tag-18 status:Partially_confirmed SW:Q22508 protein_id:AAA82434.1 /QuerySize	gi|6901611|ref|Os03g16100|
gi|55239455|gb|EAL40145.1|	lcl|T14G8.2 CE03658 WBGene00011770 status:Partially_confirmed SW:Q22513 protein_id:CAA91807.1 /QuerySize=347	gi|6901618|ref|Os03g16160|
gi|55239469|gb|EAA44290.2|	lcl|T14G8.4 CE03660 WBGene00011772 status:Partially_confirmed SW:Q22514 protein_id:CAA91808.1 /QuerySize=429	gi|6901622|ref|Os03g16200|
gi|55239497|gb|EAL40158.1|	lcl|T15B7.10 CE13664 WBGene00020522 status:Predicted SW:O17027 protein_id:AAB69971.1 /QuerySize=231	gi|6901626|ref|Os03g16240|
gi|55239498|gb|EAA10637.2|	lcl|T15B7.14 CE35770 WBGene00020526 status:Partially_confirmed SW:O17037 protein_id:AAB69967.2 /QuerySize=650	gi|6901627|ref|Os03g16250|
gi|55239500|gb|EAA10638.2|	lcl|T15B7.15 CE13674 WBGene00020527 status:Partially_confirmed SW:O17039 protein_id:AAB69968.1 /QuerySize=375	gi|6901630|ref|Os03g16270|
gi|55239505|gb|EAL40162.1|	lcl|T15B7.6 CE13658 WBGene00020518 status:Partially_confirmed SW:O17033 protein_id:AAB69962.1 /QuerySize=74	gi|6901639|ref|Os03g16360|
gi|55239508|gb|EAA10607.2|	lcl|T15D6.4 CE13686 WBGene00011779 status:Predicted SW:O02320 protein_id:CAB05626.1 /QuerySize=424	gi|6901640|ref|Os03g16370|
gi|55239513|gb|EAL40164.1|	lcl|T15H9.2 CE01665 WBGene00011788 coiled-coil status:Partially_confirmed SW:Q10006 protein_id:CAA87415.1 /QuerySize=25	gi|6901644|ref|Os03g16410|
gi|55239520|gb|EAA10594.2|	lcl|T15H9.5 CE01668 WBGene00011790 status:Partially_confirmed SW:Q09355 protein_id:CAA87418.1 /QuerySize=173	gi|6901651|ref|Os03g16470|
gi|55239566|gb|EAL40176.1|	lcl|T16A1.3 CE13708 WBGene00020534 status:Predicted SW:P91446 protein_id:AAB37874.1 /QuerySize=216	gi|6901652|ref|Os03g16470|
gi|55239567|gb|EAL40177.1|	lcl|T16A1.4 CE13710 WBGene00020535 status:Predicted SW:P91444 protein_id:AAB37872.1 /QuerySize=137	gi|6901654|ref|Os03g16490|
gi|55239582|gb|EAA10526.2|	lcl|T16A1.5 CE32723 WBGene00020536 status:Predicted SW:P91445 protein_id:AAB37873.2 /QuerySize=59	gi|6901660|ref|Os03g16550|
gi|55239586|gb|EAL40182.1|	lcl|T16A1.7 CE32724 WBGene00004149 locus:pqn-66 status:Predicted SW:P91448 protein_id:AAB37876.3 /QuerySize=896	gi|6901661|ref|Os03g16560|
gi|55239598|gb|EAL40190.1|	lcl|T16D1.1 CE04968 WBGene00020539 status:Predicted SW:Q22526 protein_id:AAA80131.1 /QuerySize=92	gi|6901665|ref|Os03g16580|
gi|55239599|gb|EAA10370.3|	lcl|T16G1.1 CE20071 WBGene00004150 locus:pqn-67 status:Partially_confirmed SW:Q9XUQ0 protein_id:CAB04725.1 /QuerySize	gi|6901666|ref|Os03g16590|
gi|55239601|gb|EAA10387.3|	lcl|T16G1.2 CE20072 WBGene00011796 status:Partially_confirmed SW:Q9XUP9 protein_id:CAB04726.1 /QuerySize=407	gi|6901670|ref|Os03g16630|
gi|55239604|gb|EAL40192.1|	lcl|T16G12.3 CE34094 WBGene00011804 status:Partially_confirmed SW:Q22532 protein_id:CAA82972.2 /QuerySize=477	gi|6901671|ref|Os03g16640|
gi|55239631|gb|EAL40197.1|	lcl|T16G12.4 CE00582 WBGene00011805 status:Partially_confirmed SW:Q22527 protein_id:CAA82967.1 /QuerySize=213	gi|6901672|ref|Os03g16650|
gi|55239642|gb|EAL40199.1|	lcl|T16G12.8 CE23987 WBGene00011809 status:Partially_confirmed SW:Q9U366 protein_id:CAB54306.1 /QuerySize=378	gi|6901673|ref|Os03g16660|
gi|55239643|gb|EAL40200.1|	lcl|T16G12.9 CE35014 WBGene00011810 status:Predicted SW:Q7YSH2 protein_id:CAE17937.1 /QuerySize=358	gi|6901675|ref|Os03g16680|
gi|55239651|gb|EAA10372.2|	lcl|T16H12.1 CE00322 WBGene00011811 status:Partially_confirmed SW:P34564 protein_id:CAA83137.1 /QuerySize=344	gi|6901681|ref|Os03g16720|
gi|55239665|gb|EAA44334.2|	lcl|T16H12.11 CE35015 WBGene00011819 status:Partially_confirmed SW:Q7YWU3 protein_id:CAE17936.1 /QuerySize=295	gi|6901684|ref|Os03g16750|
gi|55239673|gb|EAL40206.1|	lcl|T16H12.2 CE00323 WBGene00011812 status:Partially_confirmed SW:P34565 protein_id:CAA83135.1 /QuerySize=181	gi|6901686|ref|Os03g16770|
gi|55239690|gb|EAA10395.2|	lcl|T16H12.3a CE37790 WBGene00011813 status:Confirmed SW:P34566 protein_id:CAA83136.2 /QuerySize=278	gi|6901688|ref|Os03g16790|
gi|55239705|gb|EAL40213.1|	lcl|T16H12.3b CE35947 WBGene00011813 status:Confirmed SW:Q7JMR5 protein_id:CAE52907.1 /QuerySize=297	gi|6901690|ref|Os03g16810|
gi|55239722|gb|EAA10338.2|	lcl|T17A3.12 CE31608 WBGene00020548 status:Confirmed SW:Q8MXF8 protein_id:AAM97950.1 /QuerySize=43	gi|6901693|ref|Os03g16840|
gi|55239723|gb|EAL40217.1|	lcl|T17A3.2 CE37151 WBGene00020540 status:Confirmed /QuerySize=305	gi|6901707|ref|Os03g16970|
gi|55239741|gb|EAL40222.1|	lcl|T17H7.4a CE28670 WBGene00001573 locus:gei-16 status:Confirmed SW:Q95QA6 protein_id:AAK77638.1 /QuerySize=663	gi|6901710|ref|Os03g16990|
gi|55239742|gb|EAL40223.1|	lcl|T17H7.4b CE28671 WBGene00001573 locus:gei-16 status:Partially_confirmed SW:Q95QA7 protein_id:AAK77639.1 /QuerySize	gi|6901718|ref|Os03g17040|
gi|55239745|gb|EAL40225.1|	lcl|T17H7.4c CE28672 WBGene00001573 locus:gei-16 status:Partially_confirmed SW:Q95QA8 protein_id:AAK77640.1 /QuerySize	gi|6901725|ref|Os03g17090|
gi|55239749|gb|EAL40227.1|	lcl|T17H7.4d CE35016 WBGene00001573 locus:gei-16 status:Partially_confirmed SW:Q22534 protein_id:AAC48169.2 /QuerySize	gi|6901736|ref|Os03g17190|
gi|55239757|gb|EAL40231.1|	lcl|T17H7.4e CE31612 WBGene00001573 locus:gei-16 status:Partially_confirmed SW:Q8MPW6 protein_id:AAM97953.1 /QuerySi	gi|6901743|ref|Os03g17260|
gi|55239773|gb|EAL40237.1|	lcl|T17H7.4f CE31613 WBGene00001573 locus:gei-16 status:Partially_confirmed SW:Q8MPW4 protein_id:AAM97954.1 /QuerySiz	gi|6901746|ref|Os03g17290|
gi|55239777|gb|EAL40239.1|	lcl|T17H7.4g CE31614 WBGene00001573 locus:gei-16 status:Partially_confirmed SW:Q8MPW5 protein_id:AAM97955.1 /QuerySi	gi|6901749|ref|Os03g17320|
gi|55239779|gb|EAA10054.2|	lcl|T17H7.4h CE31615 WBGene00001573 locus:gei-16 status:Partially_confirmed SW:Q8MPW8 protein_id:AAM97956.1 /QuerySi	gi|6901750|ref|Os03g17330|
gi|55239787|gb|EAA10330.2|	lcl|T17H7.4i CE31616 WBGene00001573 locus:gei-16 status:Partially_confirmed SW:Q8MPW7 protein_id:AAM97957.1 /QuerySiz	gi|6901753|ref|Os03g17360|
gi|55239797|gb|EAL40246.1|	lcl|T17H7.4j CE31617 WBGene00001573 locus:gei-16 status:Partially_confirmed SW:Q8MPX0 protein_id:AAM97958.1 /QuerySize	gi|6901755|ref|Os03g17380|
gi|55239808|gb|EAL40247.1|	lcl|T17H7.4k CE31618 WBGene00001573 locus:gei-16 status:Partially_confirmed SW:Q8MPW9 protein_id:AAM97959.1 /QuerySi	gi|6901756|ref|Os03g17390|
gi|55239810|gb|EAL40248.1|	lcl|T17H7.4l CE33939 WBGene00001573 locus:gei-16 status:Partially_confirmed SW:Q86DC7 protein_id:AAP31429.1 /QuerySize	gi|6901757|ref|Os03g17400|
gi|55239814|gb|EAA10222.3|	lcl|T17H7.7 CE32725 WBGene00020551 status:Partially_confirmed SW:Q22539 protein_id:AAC48172.3 /QuerySize=469	gi|6901758|ref|Os03g17410|
gi|55239825|gb|EAL40251.1|	lcl|T18D3.5 CE03666 WBGene00011822 status:Predicted SW:Q22542 protein_id:CAA92194.1 /QuerySize=131	gi|6901759|ref|Os03g17420|
gi|55239831|gb|EAA10153.2|	lcl|T18D3.6 CE03667 WBGene00011823 status:Partially_confirmed SW:Q22543 protein_id:CAA92195.1 /QuerySize=271	gi|6901768|ref|Os03g17500|
gi|55239834|gb|EAL40252.1|	lcl|T18D3.8 CE03669 WBGene00011825 status:Predicted SW:Q22546 protein_id:CAA92205.1 /QuerySize=134	gi|6901771|ref|Os03g17530|
gi|55239835|gb|EAA10335.2|	lcl|T18H9.6 CE04974 WBGene00007023 locus:mdt-27 status:Partially_confirmed SW:Q22550 protein_id:AAA83333.1 /QuerySize=	gi|6901785|ref|Os03g17660|
gi|55239840|gb|EAA44358.2|	lcl|T19A5.3a CE37678 WBGene00020554 status:Confirmed SW:Q22552 protein_id:AAC69039.4 /QuerySize=715	gi|6901786|ref|Os03g17670|
gi|55239847|gb|EAL40254.1|	lcl|T19A5.3b CE35773 WBGene00020554 status:Confirmed SW:Q7JNX5 protein_id:AAQ91899.1 /QuerySize=422	gi|6901787|ref|Os03g17680|
gi|55239848|gb|EAL40255.1|	lcl|T19B10.2 CE16413 WBGene00011831 status:Confirmed SW:Q22562 protein_id:CAA98547.1 /QuerySize=368	gi|6901794|ref|Os03g17750|
gi|55239851|gb|EAL40257.1|	lcl|T19B10.4a CE16414 WBGene00004152 locus:pqn-70 status:Confirmed SW:Q7JLH5 protein_id:CAA98545.1 /QuerySize=243	gi|6901801|ref|Os03g17820|
gi|55239866|gb|EAA10266.3|	lcl|T19B10.4b CE16415 WBGene00004152 locus:pqn-70 status:Confirmed SW:Q22558 protein_id:CAA98546.1 /QuerySize=125	gi|6901802|ref|Os03g17830|
gi|55239867|gb|EAA10264.2|	lcl|T19B10.9 CE16416 WBGene00011836 max protein like status:Partially_confirmed SW:Q22563 protein_id:CAA98544.1 /QueryS	gi|6901803|ref|Os03g17840|
gi|55239874|gb|EAL40264.1|	lcl|T19B4.2 CE13738 WBGene00003793 locus:npp-7 status:Partially_confirmed SW:P91457 protein_id:AAB37636.1 /QuerySize=1	gi|6901807|ref|Os03g17880|
gi|55239886|gb|EAL40269.1|	lcl|T19B4.5 CE13744 WBGene00020558 status:Partially_confirmed SW:P91453 protein_id:AAB37631.1 /QuerySize=400	gi|6901808|ref|Os03g17890|
gi|55239892|gb|EAL40272.1|	lcl|T19C3.2 CE02053 WBGene00020560 status:Partially_confirmed SW:Q10008 protein_id:AAC46593.1 /QuerySize=269	gi|6901816|ref|Os03g17970|
gi|55239894|gb|EAL40273.1|	lcl|T19C3.3 CE30183 WBGene00020561 status:Confirmed SW:Q10009 protein_id:AAC46594.2 /QuerySize=92	gi|6901824|ref|Os03g18040|
gi|55239903|gb|EAL40274.1|	lcl|T19C3.6 CE32726 WBGene00020564 status:Partially_confirmed SW:Q10012 protein_id:AAC46597.2 /QuerySize=201	gi|6901830|ref|Os03g18090|
gi|55239904|gb|EAL40275.1|	lcl|T19C3.7 CE29055 WBGene00020565 protein phosphatase 2c status:Partially_confirmed SW:Q95QA1 protein_id:AAK71403.2 /	gi|6901831|ref|Os03g18100|
gi|55239907|gb|EAL40278.1|	lcl|T19C4.1 CE06465 WBGene00011838 status:Partially_confirmed SW:Q22570 protein_id:CAA99917.1 /QuerySize=424	gi|6901844|ref|Os03g18230|
gi|55239911|gb|EAA10312.3|	lcl|T19C4.7 CE27777 WBGene00003771 locus:nlp-33 status:Confirmed SW:Q95ZN4 protein_id:CAC42343.1 /QuerySize=74	gi|6901845|ref|Os03g18240|

<table>
<tr><td>gi|55239932|gb|EAL40282.1|</td><td>lcl|T19C9.8 CE16425 WBGene00011844 status:Predicted SW:O45792 protein_id:CAB07486.1 /QuerySize=402</td><td>gi|6901846|ref|Os03g18250|</td></tr>
<tr><td>gi|55239933|gb|EAL40283.1|</td><td>lcl|T19D12.1 CE28677 WBGene00020576 A. faecalis PHB depolymerase\; zinc finger protein 40 status:Partially_confirmed SW:Q2</td><td>gi|6901847|ref|Os03g18260|</td></tr>
<tr><td>gi|55239934|gb|EAA10241.2|</td><td>lcl|T19D12.2a CE33453 WBGene00020577 A. faecalis PHB depolymerase status:Confirmed SW:Q86ND6 protein_id:AAO38593.1</td><td>gi|6901848|ref|Os03g18270|</td></tr>
<tr><td>gi|55239937|gb|EAL40284.1|</td><td>lcl|T19D12.2b CE33454 WBGene00020577 status:Confirmed SW:Q86ND3 protein_id:AAO38594.1 /QuerySize=422</td><td>gi|6901849|ref|Os03g18280|</td></tr>
<tr><td>gi|55239946|gb|EAL40288.1|</td><td>lcl|T19D12.2c CE33455 WBGene00020577 status:Confirmed SW:Q86ND4 protein_id:AAO38595.1 /QuerySize=516</td><td>gi|6901850|ref|Os03g18290|</td></tr>
<tr><td>gi|55239948|gb|EAL40290.1|</td><td>lcl|T19D12.2d CE33456 WBGene00020577 status:Confirmed SW:Q86ND5 protein_id:AAO38596.1 /QuerySize=518</td><td>gi|6901854|ref|Os03g18320|</td></tr>
<tr><td>gi|55239951|gb|EAL40292.1|</td><td>lcl|T19D12.3 CE04978 WBGene00020578 status:Predicted SW:Q22577 protein_id:AAC24424.1 /QuerySize=179</td><td>gi|6901856|ref|Os03g18340|</td></tr>
<tr><td>gi|55239956|gb|EAL40294.1|</td><td>lcl|T19D12.4b CE33457 WBGene00020579 status:Partially_confirmed SW:Q86ND7 protein_id:AAO38597.1 /QuerySize=479</td><td>gi|6901862|ref|Os03g18400|</td></tr>
<tr><td>gi|55239957|gb|EAL40295.1|</td><td>lcl|T19D12.7 CE04982 WBGene00020582 status:Partially_confirmed SW:Q22575 protein_id:AAC24427.1 /QuerySize=400</td><td>gi|6901874|ref|Os03g18490|</td></tr>
<tr><td>gi|55239963|gb|EAL40296.1|</td><td>lcl|T19D2.3 CE07516 WBGene00020568 status:Partially_confirmed SW:Q22581 protein_id:AAA83601.1 /QuerySize=108</td><td>gi|6901887|ref|Os03g18610|</td></tr>
<tr><td>gi|55239966|gb|EAL40297.1|</td><td>lcl|T19D7.2 CE07518 WBGene00020570 status:Partially_confirmed SW:Q22584 protein_id:AAK39296.1 /QuerySize=143</td><td>gi|6901891|ref|Os03g18650|</td></tr>
<tr><td>gi|55239985|gb|EAL40303.1|</td><td>lcl|T19D7.3 CE07519 WBGene00020571 status:Partially_confirmed SW:Q22583 protein_id:AAK39295.1 /QuerySize=118</td><td>gi|6901893|ref|Os03g18670|</td></tr>
<tr><td>gi|55239989|gb|EAA10245.3|</td><td>lcl|T19D7.5 CE07521 WBGene00020573 status:Partially_confirmed SW:Q22586 protein_id:AAK39297.1 /QuerySize=142</td><td>gi|6901900|ref|Os03g18730|</td></tr>
<tr><td>gi|55240000|gb|EAL40307.1|</td><td>lcl|T19H12.3b CE30836 WBGene00020589 status:Confirmed SW:Q8MPT1 protein_id:AAM45361.1 /QuerySize=133</td><td>gi|6901902|ref|Os03g18750|</td></tr>
<tr><td>gi|55240015|gb|EAA10227.2|</td><td>lcl|T19H5.4 CE01675 WBGene00011849 status:Confirmed SW:Q09356 protein_id:CAA87666.1 /QuerySize=313</td><td>gi|6901903|ref|Os03g18760|</td></tr>
<tr><td>gi|55240018|gb|EAA10305.2|</td><td>lcl|T20B12.4 CE01410 WBGene00020602 status:Predicted SW:P41844 protein_id:AAA19057.1 /QuerySize=335</td><td>gi|6901906|ref|Os03g18780|</td></tr>
<tr><td>gi|55240021|gb|EAL40314.1|</td><td>lcl|T20B12.5 CE01411 WBGene00020603 status:Predicted SW:P41845 protein_id:AAA19058.1 /QuerySize=131</td><td>gi|6901907|ref|Os03g18800|</td></tr>
<tr><td>gi|55240024|gb|EAL40315.1|</td><td>lcl|T20B3.14 CE32937 WBGene00011854 status:Confirmed SW:Q8I109 protein_id:CAD59162.1 /QuerySize=64</td><td>gi|6901917|ref|Os03g18900|</td></tr>
<tr><td>gi|55240035|gb|EAL40319.1|</td><td>lcl|T20B5.2 CE28681 WBGene00020595 status:Confirmed SW:Q22600 protein_id:AAA68331.2 /QuerySize=238</td><td>gi|6901919|ref|Os03g18920|</td></tr>
<tr><td>gi|55240048|gb|EAL40325.1|</td><td>lcl|T20B6.3 CE13778 WBGene00020599 status:Predicted SW:O02049 protein_id:AAB52890.1 /QuerySize=259</td><td>gi|6901920|ref|Os03g18930|</td></tr>
<tr><td>gi|55240049|gb|EAL40326.1|</td><td>lcl|T20D3.3 CE28683 WBGene00011857 status:Confirmed SW:O01255 protein_id:CAA92486.3 /QuerySize=293</td><td>gi|6901931|ref|Os03g19030|</td></tr>
<tr><td>gi|55240050|gb|EAA10006.3|</td><td>lcl|T20D4.1 CE36507 WBGene00020607 locus:srab-20 status:Predicted SW:P91475 protein_id:AAB37597.2 /QuerySize=330</td><td>gi|6901938|ref|Os03g19100|</td></tr>
<tr><td>gi|55240051|gb|EAL40327.1|</td><td>lcl|T20D4.16 CE13808 WBGene00020621 status:Predicted SW:P91461 protein_id:AAB37583.1 /QuerySize=56</td><td>gi|6901942|ref|Os03g19140|</td></tr>
<tr><td>gi|55240052|gb|EAL40328.1|</td><td>lcl|T20D4.18 CE36508 WBGene00020623 locus:srab-21 status:Predicted SW:P91473 protein_id:AAB37595.2 /QuerySize=330</td><td>gi|6901946|ref|Os03g19180|</td></tr>
<tr><td>gi|55240053|gb|EAL40329.1|</td><td>lcl|T20D4.2 CE36509 WBGene00020608 locus:srab-22 status:Predicted SW:P91474 protein_id:AAB37596.2 /QuerySize=330</td><td>gi|6901949|ref|Os03g19210|</td></tr>
<tr><td>gi|55240058|gb|EAA09990.2|</td><td>lcl|T20F10.2 CE21191 WBGene00011864 status:Confirmed SW:O18092 protein_id:CAB04747.1 /QuerySize=282</td><td>gi|6901951|ref|Os03g19230|</td></tr>
<tr><td>gi|55240061|gb|EAA09956.2|</td><td>lcl|T20F10.4 CE13850 WBGene00011865 status:Predicted SW:O18094 protein_id:CAB04746.1 /QuerySize=416</td><td>gi|6901954|ref|Os03g19260|</td></tr>
<tr><td>gi|55240072|gb|EAL40335.1|</td><td>lcl|T20F10.5 CE35189 WBGene00011866 status:Partially_confirmed SW:Q9NAN4 protein_id:CAB76410.2 /QuerySize=441</td><td>gi|6901961|ref|Os03g19320|</td></tr>
<tr><td>gi|55240075|gb|EAA09880.2|</td><td>lcl|T20F5.4 CE13822 WBGene00020626 status:Confirmed SW:P91479 protein_id:AAB37665.1 /QuerySize=333</td><td>gi|6901965|ref|Os03g19360|</td></tr>
<tr><td>gi|55240082|gb|EAL40338.1|</td><td>lcl|T20F5.5 CE13824 WBGene00020627 status:Partially_confirmed SW:P91481 protein_id:AAB37667.1 /QuerySize=226</td><td>gi|6901974|ref|Os03g19440|</td></tr>
<tr><td>gi|55240089|gb|EAL40341.1|</td><td>lcl|T20F7.1 CE33698 WBGene00020630 status:Partially_confirmed SW:O02169 protein_id:AAK18978.2 /QuerySize=773</td><td>gi|6901979|ref|Os03g19490|</td></tr>
<tr><td>gi|55240095|gb|EAL40343.1|</td><td>lcl|T20F7.5 CE30187 WBGene00020632 status:Partially_confirmed SW:O02165 protein_id:AAK18983.2 /QuerySize=1065</td><td>gi|6901985|ref|Os03g19540|</td></tr>
<tr><td>gi|55240117|gb|EAL40348.1|</td><td>lcl|T20G5.14 CE35948 WBGene00011875 status:Confirmed SW:Q7JMS0 protein_id:CAE52908.1 /QuerySize=89</td><td>gi|6901986|ref|Os03g19550|</td></tr>
<tr><td>gi|55240157|gb|EAA09894.2|</td><td>lcl|T20H12.1 CE32729 WBGene00020643 status:Predicted SW:O16618 protein_id:AAB66088.2 /QuerySize=162</td><td>gi|6901991|ref|Os03g19600|</td></tr>
<tr><td>gi|55240159|gb|EAL40358.1|</td><td>lcl|T20H4.2 CE00749 WBGene00020635 status:Partially_confirmed SW:Q22621 protein_id:AAA50659.1 /QuerySize=235</td><td>gi|6901993|ref|Os03g19620|</td></tr>
<tr><td>gi|55240181|gb|EAL40369.1|</td><td>lcl|T21B10.3 CE23993 WBGene00011885 status:Partially_confirmed SW:Q22624 protein_id:CAA92693.2 /QuerySize=1170</td><td>gi|6901994|ref|Os03g19630|</td></tr>
<tr><td>gi|55240182|gb|EAL40370.1|</td><td>lcl|T21B10.4 CE03686 WBGene00011886 status:Partially_confirmed SW:Q22625 protein_id:CAA92694.1 /QuerySize=418</td><td>gi|6901995|ref|Os03g19640|</td></tr>
<tr><td>gi|55240195|gb|EAL40372.1|</td><td>lcl|T21B4.15 CE37557 WBGene00043054 status:Partially_confirmed SW:Q5WRM7 protein_id:CAH60752.1 /QuerySize=241</td><td>gi|6901999|ref|Os03g19660|</td></tr>
<tr><td>gi|55240197|gb|EAA09832.2|</td><td>lcl|T21B6.4 CE03682 WBGene00011881 status:Predicted SW:Q22632 protein_id:CAA92015.1 /QuerySize=119</td><td>gi|6902004|ref|Os03g19710|</td></tr>
<tr><td>gi|55240201|gb|EAL40375.1|</td><td>lcl|T21C12.3 CE26725 WBGene00011899 status:Confirmed SW:Q22634 protein_id:CAA90316.2 /QuerySize=145</td><td>gi|6902006|ref|Os03g19730|</td></tr>
<tr><td>gi|55240211|gb|EAL40381.1|</td><td>lcl|T21C12.4 CE37791 WBGene00011900 status:Partially_confirmed SW:Q22635 protein_id:CAA90317.3 /QuerySize=192</td><td>gi|6902007|ref|Os03g19740|</td></tr>
<tr><td>gi|55240216|gb|EAL40382.1|</td><td>lcl|T21C12.7 CE35019 WBGene00011901 status:Predicted SW:Q7YWT9 protein_id:CAE17942.1 /QuerySize=94</td><td>gi|6902010|ref|Os03g19770|</td></tr>
<tr><td>gi|55240217|gb|EAL40383.1|</td><td>lcl|T21C12.8 CE37792 WBGene00043998 status:Partially_confirmed protein_id:CAI06050.1 /QuerySize=122</td><td>gi|6902012|ref|Os03g19790|</td></tr>
<tr><td>gi|55240218|gb|EAL40384.1|</td><td>lcl|T21C9.11 CE06480 WBGene00011896 status:Partially_confirmed SW:Q22646 protein_id:CAA97338.1 /QuerySize=225</td><td>gi|6902013|ref|Os03g19800|</td></tr>
<tr><td>gi|55240222|gb|EAL40387.1|</td><td>lcl|T21C9.13 CE35621 WBGene00011898 status:Confirmed SW:Q22648 protein_id:CAA97340.2 /QuerySize=246</td><td>gi|6902014|ref|Os03g19810|</td></tr>
<tr><td>gi|55240223|gb|EAL40388.1|</td><td>lcl|T21C9.5 CE06474 WBGene00003065 locus:lpd-9 status:Confirmed SW:Q22641 protein_id:CAA97333.1 /QuerySize=131</td><td>gi|6902015|ref|Os03g19820|</td></tr>
<tr><td>gi|55240237|gb|EAA09769.3|</td><td>lcl|T21C9.9 CE35020 WBGene00011895 status:Partially_confirmed SW:Q22644 protein_id:CAA97336.2 /QuerySize=391</td><td>gi|6902016|ref|Os03g19830|</td></tr>
<tr><td>gi|55240238|gb|EAL40395.1|</td><td>lcl|T21D12.11 CE18274 WBGene00020650 status:Partially_confirmed SW:O16783 protein_id:AAC48093.1 /QuerySize=709</td><td>gi|6902018|ref|Os03g19850|</td></tr>
<tr><td>gi|55240243|gb|EAL40400.1|</td><td>lcl|T21D9.2 CE04990 WBGene00020645 status:Predicted SW:Q22652 protein_id:AAA81694.1 /QuerySize=120</td><td>gi|6902019|ref|Os03g19860|</td></tr>
<tr><td>gi|55240244|gb|EAL40401.1|</td><td>lcl|T21E12.5 CE13886 WBGene00020656 status:Partially_confirmed SW:P91485 protein_id:AAK21471.1 /QuerySize=186</td><td>gi|6902023|ref|Os03g19880|</td></tr>
<tr><td>gi|55240256|gb|EAA09736.2|</td><td>lcl|T21E3.2 CE34373 WBGene00020653 status:Partially_confirmed SW:O01766 protein_id:AAB54137.2 /QuerySize=207</td><td>gi|6902026|ref|Os03g19910|</td></tr>
<tr><td>gi|55240264|gb|EAL40406.1|</td><td>lcl|T21E8.4 CE06486 WBGene00011902 status:Predicted SW:Q22653 protein_id:CAA94217.1 /QuerySize=390</td><td>gi|6902030|ref|Os03g19950|</td></tr>
<tr><td>gi|55240271|gb|EAL40407.1|</td><td>lcl|T21E8.5 CE06487 WBGene00011903 status:Partially_confirmed SW:Q22654 protein_id:CAA94218.1 /QuerySize=98</td><td>gi|6902038|ref|Os03g20000|</td></tr>
<tr><td>gi|55240309|gb|EAL40413.1|</td><td>lcl|T21G5.2 CE34374 WBGene00020660 status:Partially_confirmed SW:O02062 protein_id:AAB52898.2 /QuerySize=109</td><td>gi|6902041|ref|Os03g20030|</td></tr>
<tr><td>gi|55240319|gb|EAL40418.1|</td><td>lcl|T21H3.5 CE13906 WBGene00020664 status:Partially_confirmed SW:O16306 protein_id:AAB65366.1 /QuerySize=383</td><td>gi|6902042|ref|Os03g20040|</td></tr>
<tr><td>gi|55240348|gb|EAL40427.1|</td><td>lcl|T21H8.2 CE03691 WBGene00005063 locus:sra-37 7TM receptor like status:Partially_confirmed SW:Q94072 protein_id:CAB01</td><td>gi|6902044|ref|Os03g20060|</td></tr>
<tr><td>gi|55240360|gb|EAA09600.2|</td><td>lcl|T21H8.4 CE16438 WBGene00005065 locus:sra-39 7TM receptor like status:Partially_confirmed SW:O62420 protein_id:CAA19</td><td>gi|6902045|ref|Os03g20070|</td></tr>
<tr><td>gi|55240375|gb|EAA09598.3|</td><td>lcl|T21H8.5 CE18956 WBGene00011905 status:Partially_confirmed SW:Q94075 protein_id:CAB01770.1 /QuerySize=176</td><td>gi|6902049|ref|Os03g20110|</td></tr>
<tr><td>gi|55240377|gb|EAL40435.1|</td><td>lcl|T22A3.3 CE13916 WBGene00003083 locus:lst-1 status:Partially_confirmed SW:P91820 protein_id:CAB03381.1 /QuerySize=41</td><td>gi|6902051|ref|Os03g20130|</td></tr>
<tr><td>gi|55240400|gb|EAL40442.1|</td><td>lcl|T22B11.1 CE36610 WBGene00020675 status:Partially_confirmed SW:O44564 protein_id:AAB94181.2 /QuerySize=472</td><td>gi|6902054|ref|Os03g20160|</td></tr>
<tr><td>gi|55240402|gb|EAL40443.1|</td><td>lcl|T22B11.4a CE17243 WBGene00020678 status:Partially_confirmed SW:O44561 protein_id:AAB94184.1 /QuerySize=553</td><td>gi|6902060|ref|Os03g20220|</td></tr>
<tr><td>gi|55240407|gb|EAA09588.2|</td><td>lcl|T22B11.4b CE33701 WBGene00020678 status:Partially_confirmed SW:Q86MJ7 protein_id:AAO91670.1 /QuerySize=615</td><td>gi|6902062|ref|Os03g20240|</td></tr>
<tr><td>gi|55240427|gb|EAL40448.1|</td><td>lcl|T22B2.2 CE13930 WBGene00020666 status:Predicted SW:O45194 protein_id:AAC04441.1 /QuerySize=185</td><td>gi|6902063|ref|Os03g20250|</td></tr>
<tr><td>gi|55240435|gb|EAA09334.2|</td><td>lcl|T22B2.3 CE13932 WBGene00020667 status:Predicted SW:O45193 protein_id:AAC04440.1 /QuerySize=385</td><td>gi|6902066|ref|Os03g20280|</td></tr>
<tr><td>gi|55240442|gb|EAA09332.2|</td><td>lcl|T22B2.5 CE35781 WBGene00020669 status:Partially_confirmed SW:O45191 protein_id:AAC04443.2 /QuerySize=263</td><td>gi|6902071|ref|Os03g20320|</td></tr>
<tr><td>gi|55240447|gb|EAA09365.3|</td><td>lcl|T22B2.6 CE13938 WBGene00020670 status:Partially_confirmed SW:O45192 protein_id:AAC04444.1 /QuerySize=145</td><td>gi|6902074|ref|Os03g20350|</td></tr>
<tr><td>gi|55240448|gb|EAL40452.1|</td><td>lcl|T22B2.7 CE18276 WBGene00020671 status:Predicted SW:O45190 protein_id:AAC04445.1 /QuerySize=84</td><td>gi|6902075|ref|Os03g20360|</td></tr>
<tr><td>gi|55240451|gb|EAA44503.2|</td><td>lcl|T22B3.1 CE03694 WBGene00001079 locus:dpy-20 DPY-20 protein status:Partially_confirmed SW:P34807 protein_id:CAA9261</td><td>gi|6902081|ref|Os03g20390|</td></tr>
<tr><td>gi|55240455|gb|EAL40454.1|</td><td>lcl|T22C1.10a CE06498 WBGene00004318 locus:rbg-2 status:Partially_confirmed SW:Q22670 protein_id:CAA99926.1 /QuerySize</td><td>gi|6902082|ref|Os03g20400|</td></tr>
<tr><td>gi|55240456|gb|EAL40455.1|</td><td>lcl|T22C1.10b CE36203 WBGene00004318 locus:rbg-2 status:Partially_confirmed SW:Q7JLF2 protein_id:CAE54924.1 /QuerySize</td><td>gi|6902090|ref|Os03g20480|</td></tr>
<tr><td>gi|55240467|gb|EAL40456.1|</td><td>lcl|T22C1.11 CE24001 WBGene00011920 status:Partially_confirmed SW:Q22671 protein_id:CAA99927.2 /QuerySize=444</td><td>gi|6902093|ref|Os03g20510|</td></tr>
<tr><td>gi|55240484|gb|EAL40462.1|</td><td>lcl|T22C1.12 CE35022 WBGene00011921 status:Predicted SW:Q7YWS7 protein_id:CAE17966.1 /QuerySize=133</td><td>gi|6902096|ref|Os03g20540|</td></tr>
<tr><td>gi|55240493|gb|EAA44517.2|</td><td>lcl|T22C1.4 CE06492 WBGene00011915 status:Partially_confirmed SW:Q22664 protein_id:CAA99922.1 /QuerySize=96</td><td>gi|6902101|ref|Os03g20590|</td></tr>
<tr><td>gi|55240494|gb|EAL40464.1|</td><td>lcl|T22C1.5 CE13954 WBGene00011916 status:Partially_confirmed SW:Q22665 protein_id:CAA99929.1 /QuerySize=167</td><td>gi|6902102|ref|Os03g20600|</td></tr>
<tr><td>gi|55240498|gb|EAA44518.2|</td><td>lcl|T22C1.9 CE32498 WBGene00011919 status:Confirmed SW:Q22669 protein_id:CAA99925.2 /QuerySize=195</td><td>gi|6902103|ref|Os03g20610|</td></tr>
<tr><td>gi|55240511|gb|EAL40468.1|</td><td>lcl|T22C8.6 CE02354 WBGene00011927 status:Partially_confirmed SW:Q22679 protein_id:CAA88878.1 /QuerySize=300</td><td>gi|6902104|ref|Os03g20620|</td></tr>
<tr><td>gi|55240530|gb|EAL40472.1|</td><td>lcl|T22D1.2 CE17246 WBGene00020681 status:Predicted SW:Q9GZH1 protein_id:AAF98625.1 /QuerySize=302</td><td>gi|6902117|ref|Os03g20740|</td></tr>
<tr><td>gi|55240531|gb|EAL40473.1|</td><td>lcl|T22D1.5 CE17249 WBGene00020684 status:Partially_confirmed SW:Q9GZH6 protein_id:AAF98627.1 /QuerySize=391</td><td>gi|6902120|ref|Os03g20770|</td></tr>
<tr><td>gi|55240534|gb|EAL40474.1|</td><td>lcl|T22D1.6 CE32499 WBGene00020685 status:Predicted SW:Q9GZH8 protein_id:AAF98628.2 /QuerySize=87</td><td>gi|6902124|ref|Os03g20800|</td></tr>
<tr><td>gi|55240535|gb|EAL40475.1|</td><td>lcl|T22D1.8 CE17252 WBGene00020686 status:Partially_confirmed SW:Q9GZH7 protein_id:AAF98629.1 /QuerySize=400</td><td>gi|6902127|ref|Os03g20830|</td></tr>
<tr><td>gi|55240544|gb|EAA09564.2|</td><td>lcl|T22E5.1 CE04991 WBGene00020690 status:Partially_confirmed SW:Q22683 protein_id:AAA83616.1 /QuerySize=68</td><td>gi|6902143|ref|Os03g20960|</td></tr>
<tr><td>gi|55240548|gb|EAA09567.3|</td><td>lcl|T22E5.6 CE04995 WBGene00020692 status:Partially_confirmed SW:Q22684 protein_id:AAA83617.1 /QuerySize=249</td><td>gi|6902147|ref|Os03g20990|</td></tr>
<tr><td>gi|55240550|gb|EAL40477.1|</td><td>lcl|T22F7.4 CE01418 WBGene00020703 transposase status:Predicted SW:Q8MP14 protein_id:AAA20673.1 /QuerySize=286</td><td>gi|6902150|ref|Os03g21010|</td></tr>
<tr><td>gi|55240551|gb|EAL40478.1|</td><td>lcl|T22H2.4 CE14002 WBGene00011934 status:Partially_confirmed SW:O18111 protein_id:CAB04751.1 /QuerySize=91</td><td>gi|6902157|ref|Os03g21070|</td></tr>
<tr><td>gi|55240569|gb|EAL40483.1|</td><td>lcl|T22H6.5 CE03700 WBGene00000016 locus:abf-5 status:Confirmed SW:Q22690 protein_id:CAA90673.1 /QuerySize=129</td><td>gi|6902166|ref|Os03g21150|</td></tr>
<tr><td>gi|55240575|gb|EAL40486.1|</td><td>lcl|T22H6.7 CE30355 WBGene00000017 locus:abf-6 status:Confirmed SW:Q8T3C5 protein_id:CAD27609.1 /QuerySize=195</td><td>gi|6902171|ref|Os03g21200|</td></tr>
<tr><td>gi|55240578|gb|EAA44539.2|</td><td>lcl|T23B12.10 CE14044 WBGene00020724 status:Predicted SW:O17009 protein_id:AAB69940.1 /QuerySize=293</td><td>gi|6902173|ref|Os03g21220|</td></tr>
<tr><td>gi|55240601|gb|EAA09355.2|</td><td>lcl|T23B12.5 CE14034 WBGene00020720 status:Partially_confirmed SW:O17000 protein_id:AAB69937.1 /QuerySize=224</td><td>gi|6902182|ref|Os03g21300|</td></tr>
<tr><td>gi|55240609|gb|EAA44543.2|</td><td>lcl|T23B3.1 CE14008 WBGene00020709 status:Partially_confirmed SW:P91492 protein_id:AAB42332.1 /QuerySize=389</td><td>gi|6902186|ref|Os03g21340|</td></tr>
<tr><td>gi|55240619|gb|EAL40493.1|</td><td>lcl|T23B3.3 CE14012 WBGene00020711 status:Predicted SW:P91490 protein_id:AAB42330.1 /QuerySize=256</td><td>gi|6902188|ref|Os03g21360|</td></tr>
<tr><td>gi|55240627|gb|EAA09310.2|</td><td>lcl|T23B3.5 CE14016 WBGene00020713 status:Confirmed SW:P91488 protein_id:AAB42334.1 /QuerySize=211</td><td>gi|6902195|ref|Os03g21410|</td></tr>
<tr><td>gi|55240628|gb|EAA09314.2|</td><td>lcl|T23B5.3a CE35622 WBGene00011940 status:Confirmed SW:O45800 protein_id:CAB07675.4 /QuerySize=816</td><td>gi|6902197|ref|Os03g21430|</td></tr>
<tr><td>gi|55240636|gb|EAA09371.2|</td><td>lcl|T23B5.3b CE35623 WBGene00011940 status:Confirmed SW:Q7JKA3 protein_id:CAE46674.1 /QuerySize=540</td><td>gi|6902198|ref|Os03g21440|</td></tr>
<tr><td>gi|55240656|gb|EAA09517.3|</td><td>lcl|T23C6.1 CE07526 WBGene00007011 locus:mdt-1.2 status:Partially_confirmed SW:O02042 protein_id:AAB52882.1 /QuerySize</td><td>gi|6902205|ref|Os03g21500|</td></tr>
<tr><td>gi|55240667|gb|EAL40501.1|</td><td>lcl|T23C6.3 CE07528 WBGene00020725 status:Partially_confirmed SW:O02039 protein_id:AAB52884.1 /QuerySize=533</td><td>gi|6902214|ref|Os03g21590|</td></tr>
<tr><td>gi|55240672|gb|EAL40502.1|</td><td>lcl|T23C6.4 CE07529 WBGene00020726 status:Partially_confirmed SW:O02040 protein_id:AAB52883.2 /QuerySize=341</td><td>gi|6902215|ref|Os03g21600|</td></tr>
<tr><td>gi|55240692|gb|EAL40507.1|</td><td>lcl|T23D8.9b CE27209 WBGene00011946 status:Partially_confirmed SW:Q95ZN3 protein_id:CAC42344.1 /QuerySize=239</td><td>gi|6902216|ref|Os03g21600|</td></tr>
<tr><td>gi|55240695|gb|EAL40508.1|</td><td>lcl|T23E1.2 CE33837 WBGene00020729 status:Partially_confirmed SW:O45204 protein_id:AAP13766.1 /QuerySize=499</td><td>gi|6902217|ref|Os03g21610|</td></tr>
<tr><td>gi|55240710|gb|EAL40510.1|</td><td>lcl|T23E1.3 CE33838 WBGene00020730 status:Partially_confirmed SW:Q86FL9 protein_id:AAP13767.1 /QuerySize=222</td><td>gi|6902222|ref|Os03g21650|</td></tr>
<tr><td>gi|55240716|gb|EAL40512.1|</td><td>lcl|T23E7.2d CE24008 WBGene00020732 status:Confirmed SW:Q9TXR8 protein_id:AAD47130.1 /QuerySize=118</td><td>gi|6902224|ref|Os03g21670|</td></tr>
<tr><td>gi|55240726|gb|EAL40517.1|</td><td>lcl|T23E7.3 CE14068 WBGene00020733 status:Partially_confirmed SW:O17340 protein_id:AAB71259.1 /QuerySize=196</td><td>gi|6902232|ref|Os03g21750|</td></tr>
<tr><td>gi|55240727|gb|EAA09100.3|</td><td>lcl|T23E7.4 CE37274 WBGene00003744 locus:nlp-6 status:Partially_confirmed SW:O17341 protein_id:AAB71255.2 /QuerySize=94</td><td>gi|6902233|ref|Os03g21760|</td></tr>
<tr><td>gi|55240731|gb|EAA09101.3|</td><td>lcl|T23F1.5 CE14078 WBGene00011948 status:Partially_confirmed SW:O18117 protein_id:CAB03404.1 /QuerySize=1262</td><td>gi|6902243|ref|Os03g21860|</td></tr>
<tr><td>gi|55240737|gb|EAL40520.1|</td><td>lcl|T23F11.2 CE01095 WBGene00011954 status:Confirmed SW:Q22694 protein_id:CAA86457.1 /QuerySize=253</td><td>gi|6902245|ref|Os03g21880|</td></tr>
<tr><td>gi|55240768|gb|EAL40530.1|</td><td>lcl|T23F2.2a CE31245 WBGene00020735 status:Partially_confirmed SW:Q8MPV7 protein_id:AAM69069.1 /QuerySize=1534</td><td>gi|6902250|ref|Os03g21930|</td></tr>
<tr><td>gi|55240771|gb|EAA09099.2|</td><td>lcl|T23F2.2b CE31246 WBGene00020735 status:Partially_confirmed SW:Q8MPV6 protein_id:AAM69070.1 /QuerySize=1538</td><td>gi|6902257|ref|Os03g22000|</td></tr>
<tr><td>gi|55240798|gb|EAA09257.2|</td><td>lcl|T23F4.2 CE32070 WBGene00020740 status:Partially_confirmed SW:O17261 protein_id:AAB71034.2 /QuerySize=210</td><td>gi|6902260|ref|Os03g22030|</td></tr>
<tr><td>gi|55240816|gb|EAL40540.1|</td><td>lcl|T23F6.1 CE18960 WBGene00011949 status:Confirmed SW:Q9XU68 protein_id:CAB05630.1 /QuerySize=160</td><td>gi|6902264|ref|Os03g22070|</td></tr>
<tr><td>gi|55240817|gb|EAA09065.3|</td><td>lcl|T23F6.2 CE26007 WBGene00011950 status:Predicted SW:Q9XU66 protein_id:CAB05632.2 /QuerySize=252</td><td>gi|6902272|ref|Os03g22130|</td></tr>
<tr><td>gi|55240819|gb|EAA09026.2|</td><td>lcl|T23F6.5 CE18964 WBGene00011952 status:Predicted SW:Q9XU70 protein_id:CAB05628.1 /QuerySize=441</td><td>gi|6902274|ref|Os03g22150|</td></tr>
<tr><td>gi|55240825|gb|EAA09034.2|</td><td>lcl|T23G11.1 CE14092 WBGene00011968 status:Partially_confirmed SW:Q9XVI1 protein_id:CAB03413.1 /QuerySize=168</td><td>gi|6902283|ref|Os03g22230|</td></tr>
<tr><td>gi|55240826|gb|EAL40541.1|</td><td>lcl|T23G11.10 CE35026 WBGene00011973 status:Predicted SW:Q7YTJ6 protein_id:CAE17967.1 /QuerySize=199</td><td>gi|6902284|ref|Os03g22240|</td></tr>
<tr><td>gi|55240853|gb|EAA09294.3|</td><td>lcl|T23G11.7a CE16459 WBGene00011972 status:Confirmed SW:P90969 protein_id:CAB03420.1 /QuerySize=361</td><td>gi|6902285|ref|Os03g22250|</td></tr>
<tr><td>gi|55240869|gb|EAA09049.2|</td><td>lcl|T23G4.1 CE16453 WBGene00006580 locus:tlp-1 status:Confirmed SW:O18124 protein_id:CAB05282.1 /QuerySize=354</td><td>gi|6902297|ref|Os03g22360|</td></tr>
<tr><td>gi|55240870|gb|EAL40549.1|</td><td>lcl|T23G4.2 CE16454 WBGene00011958 status:Partially_confirmed SW:O18126 protein_id:CAB05284.1 /QuerySize=128</td><td>gi|6902298|ref|Os03g22370|</td></tr>
<tr><td>gi|55240871|gb|EAL40550.1|</td><td>lcl|T23G4.3 CE16455 WBGene00011959 status:Partially_confirmed SW:O18125 protein_id:CAB05283.1 /QuerySize=485</td><td>gi|6902299|ref|Os03g22370|</td></tr>
<tr><td>gi|55240881|gb|EAL40552.1|</td><td>lcl|T23G5.6 CE37794 WBGene00011964 status:Confirmed SW:Q03615 protein_id:CAA79572.2 /QuerySize=305</td><td>gi|6902316|ref|Os03g22500|</td></tr>
<tr><td>gi|55240883|gb|EAL40553.1|</td><td>lcl|T24A11.2 CE34097 WBGene00011974 status:Partially_confirmed SW:Q22709 protein_id:CAA88881.2 /QuerySize=331</td><td>gi|6902318|ref|Os03g22520|</td></tr>
<tr><td>gi|55240890|gb|EAL40555.1|</td><td>lcl|T24A6.1 CE14124 WBGene00020744 status:Predicted SW:O61951 protein_id:AAC17791.1 /QuerySize=124</td><td>gi|6902329|ref|Os03g22630|</td></tr>
<tr><td>gi|55240900|gb|EAA44602.2|</td><td>lcl|T24A6.17 CE14156 WBGene00020754 status:Predicted SW:O61953 protein_id:AAC17804.1 /QuerySize=49</td><td>gi|6902331|ref|Os03g22650|</td></tr>
<tr><td>gi|55240908|gb|EAL40557.1|</td><td>lcl|T24A6.2 CE14126 WBGene00020745 status:Predicted SW:O61950 protein_id:AAC17790.1 /QuerySize=155</td><td>gi|6902338|ref|Os03g22710|</td></tr>
<tr><td>gi|55240914|gb|EAL40558.1|</td><td>lcl|T24B8.3a CE35910 WBGene00011977 status:Confirmed SW:Q22719 protein_id:CAA92760.2 /QuerySize=105</td><td>gi|6902339|ref|Os03g22720|</td></tr>
<tr><td>gi|55240917|gb|EAL40559.1|</td><td>lcl|T24B8.3b CE35911 WBGene00011977 status:Partially_confirmed SW:Q7JLZ9 protein_id:CAE48509.1 /QuerySize=104</td><td>gi|6902344|ref|Os03g22760|</td></tr>
<tr><td>gi|55240939|gb|EAL40564.1|</td><td>lcl|T24B8.4 CE03712 WBGene00011978 status:Partially_confirmed SW:Q22715 protein_id:CAA92756.1 /QuerySize=914</td><td>gi|6902351|ref|Os03g22820|</td></tr>
<tr><td>gi|55240951|gb|EAL40567.1|</td><td>lcl|T24C12.1 CE05001 WBGene00020764 status:Predicted SW:Q22722 protein_id:AAA82389.1 /QuerySize=378</td><td>gi|6902355|ref|Os03g22860|</td></tr>
<tr><td>gi|55240955|gb|EAL40568.1|</td><td>lcl|T24C12.4 CE05004 WBGene00020766 status:Predicted SW:Q22723 protein_id:AAA82390.1 /QuerySize=218</td><td>gi|6902360|ref|Os03g22910|</td></tr>
<tr><td>gi|55240959|gb|EAA08998.2|</td><td>lcl|T24C2.2 CE03716 WBGene00011981 status:Partially_confirmed SW:Q22725 protein_id:CAA92200.1 /QuerySize=218</td><td>gi|6902362|ref|Os03g22930|</td></tr>
<tr><td>gi|55240961|gb|EAL40572.1|</td><td>lcl|T24C2.3 CE03717 WBGene00011982 status:Partially_confirmed SW:Q22726 protein_id:CAA92201.1 /QuerySize=266</td><td>gi|6902363|ref|Os03g22940|</td></tr>
<tr><td>gi|55240962|gb|EAL40573.1|</td><td>lcl|T24C2.5 CE03719 WBGene00011984 status:Partially_confirmed SW:Q22728 protein_id:CAA92203.1 /QuerySize=177</td><td>gi|6902365|ref|Os03g22960|</td></tr>
<tr><td>gi|55240967|gb|EAL40575.1|</td><td>lcl|T24C4.2 CE19593 WBGene00020758 status:Partially_confirmed SW:Q9TZ37 protein_id:AAC68921.1 /QuerySize=423</td><td>gi|6902369|ref|Os03g23000|</td></tr>
<tr><td>gi|55240968|gb|EAL40576.1|</td><td>lcl|T24C4.3 CE19594 WBGene00020759 status:Partially_confirmed SW:Q9TZ36 protein_id:AAC68922.1 /QuerySize=155</td><td>gi|6902377|ref|Os03g23040|</td></tr>
<tr><td>gi|55240970|gb|EAA44630.2|</td><td>lcl|T24D3.2 CE37414 WBGene00023492 status:Confirmed SW:Q5WRP8 protein_id:CAH60754.1 /QuerySize=70</td><td>gi|6902379|ref|Os03g23060|</td></tr>
<tr><td>gi|55240979|gb|EAA08875.2|</td><td>lcl|T24D5.1 CE03721 WBGene00011989 status:Predicted SW:Q22732 protein_id:CAA92016.1 /QuerySize=304</td><td>gi|6902382|ref|Os03g23090|</td></tr>
<tr><td>gi|55240982|gb|EAL40579.1|</td><td>lcl|T24D5.2 CE03722 WBGene00011990 status:Partially_confirmed SW:Q22736 protein_id:CAA92020.1 /QuerySize=408</td><td>gi|6902386|ref|Os03g23130|</td></tr>
</table>

gi|55240984|gb|EAA08874.2|	lcl|T24D5.3 CE03723 WBGene00011991 status:Partially_confirmed SW:Q22733 protein_id:CAA92017.1 /QuerySize=269	gi|6902390|ref|Os03g23170|
gi|55240996|gb|EAA44637.2|	lcl|T24D5.5 CE03725 WBGene00011993 status:Predicted SW:Q22735 protein_id:CAA92019.1 /QuerySize=408	gi|6902394|ref|Os03g23210|
gi|55241008|gb|EAL40583.1|	lcl|T24D8.2 CE37153 WBGene00020768 status:Confirmed SW:Q22740 protein_id:AAA81447.3 /QuerySize=62	gi|6902397|ref|Os03g23240|
gi|55241009|gb|EAL40584.1|	lcl|T24D8.3 CE05007 WBGene00003760 locus:nlp-22 status:Predicted SW:Q22737 protein_id:AAA81444.1 /QuerySize=102	gi|6902398|ref|Os03g23250|
gi|55241010|gb|EAL40585.1|	lcl|T24D8.4 CE05008 WBGene00003761 locus:nlp-23 status:Predicted SW:Q22738 protein_id:AAA81445.1 /QuerySize=137	gi|6902400|ref|Os03g23760|
gi|55241025|gb|EAA08798.2|	lcl|T24D8.5 CE05009 WBGene00003740 locus:nlp-2 status:Confirmed SW:Q22739 protein_id:AAA81446.1 /QuerySize=171	gi|6902406|ref|Os03g23820|
gi|55241035|gb|EAA08878.2|	lcl|T24D8.6 CE05010 WBGene00020769 status:Partially_confirmed SW:Q22742 protein_id:AAA81449.1 /QuerySize=63	gi|6902409|ref|Os03g23850|
gi|55241038|gb|EAL40591.1|	lcl|T24E12.1 CE17259 WBGene00020771 status:Predicted SW:O44885 protein_id:AAB95039.1 /QuerySize=507	gi|6902411|ref|Os03g23870|
gi|55241040|gb|EAL40592.1|	lcl|T24E12.10 CE17267 WBGene00020777 status:Predicted SW:O44881 protein_id:AAB95047.1 /QuerySize=632	gi|6902412|ref|Os03g23880|
gi|55241041|gb|EAL40593.1|	lcl|T24E12.11 CE17268 WBGene00020778 status:Partially_confirmed SW:O44883 protein_id:AAB95045.1 /QuerySize=195	gi|6902414|ref|Os03g23900|
gi|55241056|gb|EAL40596.1|	lcl|T24E12.2 CE17260 WBGene00020772 status:Predicted SW:O44884 protein_id:AAB95040.1 /QuerySize=251	gi|6902415|ref|Os03g23900|
gi|55241084|gb|EAA08809.2|	lcl|T24E12.3 CE17261 WBGene00020773 status:Predicted SW:O44882 protein_id:AAB95038.1 /QuerySize=288	gi|6902417|ref|Os03g23920|
gi|55241088|gb|EAL40601.1|	lcl|T24E12.6 CE35626 WBGene00020775 status:Partially_confirmed SW:O44878 protein_id:AAB95046.3 /QuerySize=125	gi|6902418|ref|Os03g23920|
gi|55241089|gb|EAL40602.1|	lcl|T24E12.9 CE24013 WBGene00020776 status:Partially_confirmed SW:O44880 protein_id:AAB95044.2 /QuerySize=565	gi|6902420|ref|Os03g23940|
gi|55241095|gb|EAA08891.2|	lcl|T24F1.4 CE02364 WBGene00011996 status:Predicted SW:Q22745 protein_id:CAA90138.1 /QuerySize=149	gi|6902422|ref|Os03g23960|
gi|55241102|gb|EAA08892.2|	lcl|T24F1.5 CE02365 WBGene00011997 status:Partially_confirmed SW:Q22746 protein_id:CAA90139.1 /QuerySize=64	gi|6902435|ref|Os03g24080|
gi|55241121|gb|EAA08902.2|	lcl|T24F1.7 CE35029 WBGene00011999 status:Predicted SW:Q7YWR6 protein_id:CAE17988.1 /QuerySize=78	gi|6902436|ref|Os03g24090|
gi|55241131|gb|EAL40609.1|	lcl|T24H10.5 CE03730 WBGene00012003 status:Predicted SW:Q22755 protein_id:CAA90947.1 /QuerySize=421	gi|6902440|ref|Os03g24130|
gi|55241139|gb|EAA08819.3|	lcl|T24H10.8 CE35031 WBGene00012006 status:Predicted SW:Q7YTK1 protein_id:CAE17949.1 /QuerySize=72	gi|6902441|ref|Os03g24140|
gi|55241143|gb|EAA08823.3|	lcl|T24H7.4 CE02886 WBGene00020783 status:Partially_confirmed SW:Q22756 protein_id:AAA68350.1 /QuerySize=106	gi|6902442|ref|Os03g24150|
gi|55241144|gb|EAA44672.2|	lcl|T24H7.5c CE21205 WBGene00020784 locus:tat-4 status:Confirmed SW:Q7JPE4 protein_id:AAD31558.1 /QuerySize=239	gi|6902447|ref|Os03g24200|
gi|55241145|gb|EAL40612.1|	lcl|T25B2.2 CE31249 WBGene00020786 status:Partially_confirmed SW:O61751 protein_id:AAC17000.2 /QuerySize=462	gi|6902448|ref|Os03g24210|
gi|55241147|gb|EAA08826.2|	lcl|T25B6.1 CE05011 WBGene00020787 status:Predicted SW:Q22764 protein_id:AAC48217.1 /QuerySize=185	gi|6902451|ref|Os03g24230|
gi|55241149|gb|EAL40613.1|	lcl|T25B6.3 CE05013 WBGene00020789 status:Partially_confirmed SW:Q22762 protein_id:AAC48218.1 /QuerySize=150	gi|6902453|ref|Os03g24250|
gi|55241161|gb|EAL40617.1|	lcl|T25B6.4 CE05014 WBGene00020790 status:Partially_confirmed SW:Q22759 protein_id:AAC48219.1 /QuerySize=96	gi|6902457|ref|Os03g24290|
gi|55241172|gb|EAL40620.1|	lcl|T25B6.5 CE05015 WBGene00020791 status:Predicted SW:Q22760 protein_id:AAC48221.1 /QuerySize=94	gi|6902459|ref|Os03g24310|
gi|55241179|gb|EAL40622.1|	lcl|T25B6.6 CE30191 WBGene00020792 status:Confirmed SW:Q22761 protein_id:AAC48220.2 /QuerySize=112	gi|6902460|ref|Os03g24320|
gi|55241197|gb|EAA08795.2|	lcl|T25B9.3a CE36717 WBGene00012009 status:Partially_confirmed SW:Q22767 protein_id:CAH04716.1 /QuerySize=263	gi|6902468|ref|Os03g24400|
gi|55241206|gb|EAA08737.2|	lcl|T25B9.3b CE36718 WBGene00012009 status:Confirmed SW:Q6BET9 protein_id:CAH04719.1 /QuerySize=154	gi|6902470|ref|Os03g24420|
gi|55241219|gb|EAL40635.1|	lcl|T25B9.6 CE35628 WBGene00012012 status:Partially_confirmed SW:Q22769 protein_id:CAA94377.2 /QuerySize=588	gi|6902477|ref|Os03g24470|
gi|55241222|gb|EAL40638.1|	lcl|T25C12.1a CE03733 WBGene00003003 locus:lin-14 embryonic nuclear protein lin-14 form B2 status:Partially_confirmed SW:Q	gi|6902478|ref|Os03g24480|
gi|55241223|gb|EAL40639.1|	lcl|T25C12.1b CE03734 WBGene00003003 locus:lin-14 embryonic nuclear protein lin-14 form A status:Partially_confirmed SW:Q2	gi|6902480|ref|Os03g24500|
gi|55241224|gb|EAL40640.1|	lcl|T25D1.1 CE05017 WBGene00020793 status:Predicted SW:Q22779 protein_id:AAA82466.1 /QuerySize=612	gi|6902483|ref|Os03g24530|
gi|55241228|gb|EAA08582.2|	lcl|T25D10.4 CE02061 WBGene00020801 status:Partially_confirmed SW:Q10019 protein_id:AAC46591.1 /QuerySize=246	gi|6902484|ref|Os03g24540|
gi|55241242|gb|EAA08673.3|	lcl|T25E12.11 CE33839 WBGene00012026 locus:srz-100 DUF-216 family status:Partially_confirmed SW:O45825 protein_id:CAB0	gi|6902485|ref|Os03g24550|
gi|55241249|gb|EAL40646.1|	lcl|T25E12.4b CE18283 WBGene00012019 status:Partially_confirmed SW:O62166 protein_id:CAB07477.1 /QuerySize=220	gi|6902486|ref|Os03g24560|
gi|55241254|gb|EAL40648.1|	lcl|T25E4.1 CE28493 WBGene00020803 status:Confirmed SW:Q10014 protein_id:AAC46730.2 /QuerySize=231	gi|6902487|ref|Os03g24570|
gi|55241275|gb|EAA08494.2|	lcl|T25F10.1 CE07532 WBGene00020805 status:Confirmed SW:Q23051 protein_id:AAB04990.1 /QuerySize=94	gi|6902498|ref|Os03g24670|
gi|55241279|gb|EAL40652.1|	lcl|T25G12.1 CE07538 WBGene00020809 status:Partially_confirmed SW:Q22788 protein_id:AAC69017.1 /QuerySize=223	gi|6902505|ref|Os03g24740|
gi|55241310|gb|EAL40660.1|	lcl|T25G12.11 CE30400 WBGene00020817 status:Confirmed SW:Q8T3C3 protein_id:AAM15610.1 /QuerySize=83	gi|6902506|ref|Os03g24750|
gi|55241311|gb|EAA08520.3|	lcl|T25G12.3 CE07540 WBGene00020811 status:Partially_confirmed SW:Q22784 protein_id:AAC69019.1 /QuerySize=80	gi|6902508|ref|Os03g24770|
gi|55241322|gb|EAA08603.3|	lcl|T25G12.8 CE07545 WBGene00020814 status:Predicted SW:Q22786 protein_id:AAC69021.1 /QuerySize=241	gi|6902512|ref|Os03g24810|
gi|55241323|gb|EAL40663.1|	lcl|T25G3.1 CE34378 WBGene00012029 status:Partially_confirmed SW:Q22790 protein_id:CAA96687.2 /QuerySize=156	gi|6902515|ref|Os03g24840|
gi|55241325|gb|EAA08545.2|	lcl|T26A5.2a CE00703 WBGene00020819 status:Partially_confirmed SW:Q22801 protein_id:AAC77505.1 /QuerySize=779	gi|6902536|ref|Os03g25020|
gi|55241341|gb|EAA08598.3|	lcl|T26A5.2b CE32732 WBGene00020819 status:Partially_confirmed SW:Q8IFY8 protein_id:AAN65290.1 /QuerySize=641	gi|6902540|ref|Os03g25060|
gi|55241361|gb|EAL40669.1|	lcl|T26A8.1 CE07547 WBGene00020824 status:Confirmed SW:Q22806 protein_id:AAA85757.1 /QuerySize=708	gi|6902543|ref|Os03g25090|
gi|55241363|gb|EAL40671.1|	lcl|T26A8.2 CE07548 WBGene00020825 status:Predicted SW:Q22804 protein_id:AAA85755.1 /QuerySize=301	gi|6902557|ref|Os03g25230|
gi|55241378|gb|EAL40674.1|	lcl|T26A8.3 CE07549 WBGene00020826 status:Predicted SW:Q22803 protein_id:AAA85754.1 /QuerySize=162	gi|6902565|ref|Os03g25310|
gi|55241382|gb|EAL40675.1|	lcl|T26A8.5 CE37276 WBGene00023488 locus:srz-84 status:Confirmed SW:Q65ZI2 protein_id:AAU05549.1 /QuerySize=41	gi|6902575|ref|Os03g25410|
gi|55241392|gb|EAA44751.2|	lcl|T26C11.2 CE05021 WBGene00020828 E. gracilis major membrane skeletal protein status:Predicted SW:Q22807 protein_id:AA	gi|6902585|ref|Os03g25510|
gi|55241403|gb|EAL40683.1|	lcl|T26C11.3 CE05022 WBGene00020829 status:Predicted SW:Q22808 protein_id:AAC48212.1 /QuerySize=97	gi|6902586|ref|Os03g25520|
gi|55241415|gb|EAL40686.1|	lcl|T26C11.4 CE05023 WBGene00020830 status:Partially_confirmed SW:Q22809 protein_id:AAC48213.1 /QuerySize=548	gi|6902587|ref|Os03g25530|
gi|55241417|gb|EAL40687.1|	lcl|T26C12.2 CE18286 WBGene00020832 status:Partially_confirmed SW:O61857 protein_id:AAC17554.1 /QuerySize=240	gi|6902588|ref|Os03g25540|
gi|55241421|gb|EAL40690.1|	lcl|T26C5.4 CE03740 WBGene00012034 status:Predicted SW:Q22817 protein_id:CAA90729.1 /QuerySize=135	gi|6902590|ref|Os03g25560|
gi|55241431|gb|EAA08372.3|	lcl|T26E3.10 CE35034 WBGene00012042 status:Predicted SW:Q7YTJ9 protein_id:CAE17954.1 /QuerySize=93	gi|6902596|ref|Os03g25610|
gi|55241440|gb|EAL40698.1|	lcl|T26E3.5 CE14192 WBGene00012038 status:Partially_confirmed SW:O45826 protein_id:CAB04831.1 /QuerySize=158	gi|6902598|ref|Os03g25630|
gi|55241444|gb|EAL40701.1|	lcl|T26E3.6 CE14194 WBGene00012039 fibrillin status:Partially_confirmed SW:O45828 protein_id:CAB04833.1 /QuerySize=103	gi|6902599|ref|Os03g25640|
gi|55241447|gb|EAL40702.1|	lcl|T26E3.8 CE21207 WBGene00012041 status:Partially_confirmed SW:O18130 protein_id:CAB04838.1 /QuerySize=232	gi|6902601|ref|Os03g25660|
gi|55241453|gb|EAA08373.2|	lcl|T26E3.9 CE33461 WBGene00005051 locus:sra-25 7TM chemoreceptor	gi|6902604|ref|Os03g25690|
gi|55241480|gb|EAA08325.2|	lcl|T26E4.10 CE16481 WBGene00012052 status:Predicted SW:O45834 protein_id:CAB03429.1 /QuerySize=90	gi|6902608|ref|Os03g25730|
gi|55241482|gb|EAL40710.1|	lcl|T26E4.2 CE16474 WBGene00012044 status:Partially_confirmed SW:O45836 protein_id:CAB03431.1 /QuerySize=125	gi|6902614|ref|Os03g25780|
gi|55241488|gb|EAL40714.1|	lcl|T26E4.4 CE16476 WBGene00012046 status:Predicted SW:O45839 protein_id:CAB03434.1 /QuerySize=365	gi|6902617|ref|Os03g25800|
gi|55241498|gb|EAA08329.3|	lcl|T26E4.9 CE16480 WBGene00012051 status:Predicted SW:O45833 protein_id:CAB03428.1 /QuerySize=86	gi|6902618|ref|Os03g25810|
gi|55241514|gb|EAL40718.1|	lcl|T26F2.2 CE14203 WBGene00012058 status:Predicted SW:O18133 protein_id:CAB04841.1 /QuerySize=501	gi|6902624|ref|Os03g25870|
gi|55241515|gb|EAL40719.1|	lcl|T26G10.4 CE00340 WBGene00012061 status:Predicted SW:P34583 protein_id:CAA82361.1 /QuerySize=347	gi|6902628|ref|Os03g25910|
gi|55241520|gb|EAL40721.1|	lcl|T26G10.5 CE00341 WBGene00012062 status:Predicted SW:P34584 protein_id:CAA82364.1 /QuerySize=125	gi|6902633|ref|Os03g25950|
gi|55241521|gb|EAA08324.2|	lcl|T26H5.4 CE18292 WBGene00012069 status:Partially_confirmed SW:O62385 protein_id:CAB04855.1 /QuerySize=102	gi|6902646|ref|Os03g26060|
gi|55241531|gb|EAA08328.2|	lcl|T26H8.3 CE18970 WBGene00005940 locus:srx-49 status:Predicted SW:Q9XUN1 protein_id:CAB04859.1 /QuerySize=168	gi|6902647|ref|Os03g26070|
gi|55241540|gb|EAL40723.1|	lcl|T27A1.1 CE21209 WBGene00005324 locus:srh-105 7TM chemoreceptor	gi|6902651|ref|Os03g26110|
gi|55241547|gb|EAA08321.2|	lcl|T27A1.3 CE14209 WBGene00020835 status:Partially_confirmed SW:O17272 protein_id:AAB71044.1 /QuerySize=248	gi|6902652|ref|Os03g26120|
gi|55241554|gb|EAA08359.2|	lcl|T27A10.2 CE07552 WBGene00020844 status:Partially_confirmed SW:Q22821 protein_id:AAB52501.1 /QuerySize=274	gi|6902654|ref|Os03g26140|
gi|55241563|gb|EAL40728.1|	lcl|T27A10.5 CE07554 WBGene00020845 status:Predicted SW:Q22819 protein_id:AAB52503.1 /QuerySize=108	gi|6902655|ref|Os03g26150|
gi|55241602|gb|EAA08312.2|	lcl|T27A10.6 CE30193 WBGene00020846 status:Partially_confirmed SW:Q22822 protein_id:AAB52504.3 /QuerySize=870	gi|6902658|ref|Os03g26180|
gi|55241613|gb|EAL40738.1|	lcl|T27A3.4 CE14223 WBGene00020840 status:Confirmed SW:P91497 protein_id:AAB42294.1 /QuerySize=203	gi|6902659|ref|Os03g26190|
gi|55241618|gb|EAL40742.1|	lcl|T27A8.4 CE03744 WBGene00012076 status:Predicted SW:Q22828 protein_id:CAA92225.1 /QuerySize=97	gi|6902665|ref|Os03g26240|
gi|55241642|gb|EAL40748.1|	lcl|T27C4.1 CE33329 WBGene00020854 status:Confirmed SW:O61906 protein_id:AAC17698.2 /QuerySize=243	gi|6902668|ref|Os03g26270|
gi|55241645|gb|EAL40751.1|	lcl|T27C4.2 CE18296 WBGene00020855 status:Partially_confirmed SW:O61904 protein_id:AAC17699.1 /QuerySize=643	gi|6902669|ref|Os03g26280|
gi|55241660|gb|EAL40756.1|	lcl|T27D12.4 CE26472 WBGene00003979 locus:pes-5 status:Partially_confirmed SW:Q9GNA9 protein_id:CAC15867.1 /QuerySize	gi|6902672|ref|Os03g26310|
gi|55241661|gb|EAL40757.1|	lcl|T27E4.4 CE14253 WBGene00020862 status:Confirmed SW:Q23052 protein_id:AAB04835.1 /QuerySize=89	gi|6902673|ref|Os03g26320|
gi|55241665|gb|EAL40761.1|	lcl|T27E4.5 CE35787 WBGene00020863 status:Partially_confirmed SW:Q23053 protein_id:AAB04836.3 /QuerySize=213	gi|6902675|ref|Os03g26340|
gi|55241666|gb|EAL40762.1|	lcl|T27E4.7 CE14259 WBGene00020865 status:Confirmed SW:Q23055 protein_id:AAB04838.1 /QuerySize=73	gi|6902679|ref|Os03g26380|
gi|55241667|gb|EAA44815.2|	lcl|T27E7.1 CE16505 WBGene00012087 status:Partially_confirmed SW:O45855 protein_id:CAB05287.1 /QuerySize=226	gi|6902682|ref|Os03g26410|
gi|55241668|gb|EAL40763.1|	lcl|T27E7.3 CE16507 WBGene00012088 status:Predicted SW:O45858 protein_id:CAB05290.1 /QuerySize=402	gi|6902689|ref|Os03g26470|
gi|55241669|gb|EAL40764.1|	lcl|T27E7.4 CE20107 WBGene00012089 status:Partially_confirmed SW:Q9XUC5 protein_id:CAB05293.1 /QuerySize=350	gi|6902690|ref|Os03g26480|
gi|55241679|gb|EAL40770.1|	lcl|T27E7.5 CE16508 WBGene00012090 status:Predicted SW:O45856 protein_id:CAB05288.1 /QuerySize=258	gi|6902691|ref|Os03g26490|
gi|55241682|gb|EAL40772.1|	lcl|T27E7.6 CE16509 WBGene00012091 status:Partially_confirmed SW:O45859 protein_id:CAB05291.1 /QuerySize=418	gi|6902692|ref|Os03g26500|
gi|55241691|gb|EAL40774.1|	lcl|T27E7.9 CE35036 WBGene00012093 status:Predicted SW:Q7YWS1 protein_id:CAE17979.1 /QuerySize=342	gi|6902693|ref|Os03g26510|
gi|55241692|gb|EAL40775.1|	lcl|T27E9.6 CE34099 WBGene00012096 status:Partially_confirmed SW:O45863 protein_id:CAB04872.2 /QuerySize=201	gi|6902694|ref|Os03g26520|
gi|55241696|gb|EAA07905.2|	lcl|T27F2.4 CE06522 WBGene00012101 status:Confirmed SW:Q22835 protein_id:CAA98551.1 /QuerySize=165	gi|6902697|ref|Os03g26550|
gi|55241697|gb|EAA07960.3|	lcl|T27F6.4 CE16513 WBGene00012104 status:Partially_confirmed SW:O45868 protein_id:CAB04883.1 /QuerySize=206	gi|6902700|ref|Os03g26580|
gi|55241698|gb|EAL40776.1|	lcl|T27F6.7 CE16516 WBGene00012106 status:Confirmed SW:O45871 protein_id:CAB04886.1 /QuerySize=257	gi|6902707|ref|Os03g26640|
gi|55241699|gb|EAA44829.2|	lcl|T28A11.4 CE14287 WBGene00020871 status:Predicted SW:P91520 protein_id:AAC48114.1 /QuerySize=90	gi|6902709|ref|Os03g26660|
gi|55241706|gb|EAA07934.2|	lcl|T28A11.6 CE14291 WBGene00020873 status:Predicted SW:P91514 protein_id:AAC48117.1 /QuerySize=129	gi|6902710|ref|Os03g26670|
gi|55241722|gb|EAA07908.2|	lcl|T28B4.2 CE14323 WBGene00020885 status:Partially_confirmed SW:O17343 protein_id:AAK39306.1 /QuerySize=260	gi|6902711|ref|Os03g26680|
gi|55241734|gb|EAL40785.1|	lcl|T28B4.4 CE33711 WBGene00020887 status:Confirmed SW:O17346 protein_id:AAK39305.3 /QuerySize=167	gi|6902715|ref|Os03g26720|
gi|55241739|gb|EAA07796.2|	lcl|T28B8.1 CE14329 WBGene00012113 status:Confirmed SW:P91833 protein_id:CAB03441.1 /QuerySize=170	gi|6902720|ref|Os03g26770|
gi|55241744|gb|EAL40787.1|	lcl|T28C12.6 CE14349 WBGene00020893 status:Predicted SW:O16699 protein_id:AAB66161.1 /QuerySize=333	gi|6902721|ref|Os03g26780|
gi|55241746|gb|EAL40788.1|	lcl|T28C6.1 CE35630 WBGene00012118 status:Confirmed SW:Q22843 protein_id:CAA90992.2 /QuerySize=281	gi|6902722|ref|Os03g26790|
gi|55241751|gb|EAL40791.1|	lcl|T28C6.3 CE03748 WBGene00012119 status:Partially_confirmed SW:Q22845 protein_id:CAA90994.1 /QuerySize=200	gi|6902724|ref|Os03g26810|
gi|55241755|gb|EAL40792.1|	lcl|T28C6.5 CE35631 WBGene00012120 status:Partially_confirmed SW:Q22846 protein_id:CAA90996.2 /QuerySize=307	gi|6902726|ref|Os03g26830|
gi|55241756|gb|EAL40793.1|	lcl|T28C6.8 CE26012 WBGene00012122 status:Partially_confirmed SW:Q9XVW9 protein_id:CAA90999.2 /QuerySize=119	gi|6902727|ref|Os03g26840|
gi|55241757|gb|EAA07797.3|	lcl|T28D6.5a CE32216 WBGene00012125 status:Partially_confirmed SW:O18151 protein_id:CAD56610.1 /QuerySize=795	gi|6902728|ref|Os03g26850|
gi|55241777|gb|EAL40797.1|	lcl|T28D6.5b CE32506 WBGene00012125 status:Partially_confirmed SW:Q8I055 protein_id:CAD56611.1 /QuerySize=879	gi|6902734|ref|Os03g26900|
gi|55241779|gb|EAA07541.2|	lcl|T28D9.1 CE28092 WBGene00020894 status:Confirmed SW:Q10020 protein_id:AAA68316.2 /QuerySize=129	gi|6902738|ref|Os03g26950|
gi|55241793|gb|EAA07561.3|	lcl|T28D9.11 CE02066 WBGene00020899 status:Partially_confirmed SW:Q10027 protein_id:AAA68315.1 /QuerySize=64	gi|6902739|ref|Os03g26940|
gi|55241795|gb|EAL40798.1|	lcl|T28D9.2c CE28094 WBGene00004702 locus:rsp-5 status:Partially_confirmed SW:Q10021 protein_id:AAK68400.1 /QuerySize=	gi|6902742|ref|Os03g26980|
gi|55241797|gb|EAL40800.1|	lcl|T28D9.4 CE36024 WBGene00020896 status:Partially_confirmed SW:Q10023 protein_id:AAA68310.2 /QuerySize=468	gi|6902749|ref|Os03g27030|
gi|55241816|gb|EAA07597.3|	lcl|T28F12.1 CE21218 WBGene00020904 status:Partially_confirmed SW:Q9N5D4 protein_id:AAF39973.1 /QuerySize=431	gi|6902752|ref|Os03g27050|
gi|55241817|gb|EAA07542.3|	lcl|T28F12.2d CE31074 WBGene00006796 locus:unc-62 status:Confirmed SW:Q8MXU6 protein_id:AAM48548.1 /QuerySize=111	gi|6902753|ref|Os03g27060|
gi|55241823|gb|EAL40803.1|	lcl|T28F2.2 CE31075 WBGene00020901 status:Confirmed SW:O01659 protein_id:AAB53058.2 /QuerySize=175	gi|6902754|ref|Os03g27070|
gi|55241824|gb|EAA07593.3|	lcl|T28F2.4b CE37050 WBGene00020902 status:Confirmed SW:Q6A599 protein_id:AAT81147.1 /QuerySize=125	gi|6902757|ref|Os03g27100|
gi|55241831|gb|EAA07615.3|	lcl|T28F3.7 CE28691 WBGene00012133 status:Predicted SW:Q9XUC1 protein_id:CAB05300.2 /QuerySize=498	gi|6902760|ref|Os03g27120|
gi|55241832|gb|EAL40805.1|	lcl|T28F3.8 CE20118 WBGene00012134 status:Partially_confirmed SW:Q9XUC0 protein_id:CAB05301.1 /QuerySize=548	gi|6902761|ref|Os03g27130|
gi|55241840|gb|EAA07619.3|	lcl|T28F4.1 CE06523 WBGene00012136 status:Confirmed SW:Q22849 protein_id:CAA96691.1 /QuerySize=285	gi|6902764|ref|Os03g27160|
gi|55241848|gb|EAL40809.1|	lcl|T28F4.4 CE06526 WBGene00012139 status:Partially_confirmed SW:Q22853 protein_id:CAA96695.1 /QuerySize=717	gi|6902766|ref|Os03g27180|
gi|55241860|gb|EAL40817.1|	lcl|T28F4.5 CE06527 WBGene00012140 status:Confirmed SW:Q22850 protein_id:CAA96692.1 /QuerySize=105	gi|6902768|ref|Os03g27200|
gi|55241861|gb|EAL40818.1|	lcl|T28H10.4 CE35037 WBGene00012145 status:Predicted SW:Q7YTJ1 protein_id:CAE17986.1 /QuerySize=66	gi|6902770|ref|Os03g27220|
gi|55241874|gb|EAA07998.2|	lcl|T28H11.7 CE14380 WBGene00020905 status:Confirmed SW:Q23061 protein_id:AAB04603.1 /QuerySize=194	gi|6902775|ref|Os03g27270|
gi|55241889|gb|EAL40822.1|	lcl|VB0393L.2 CE32101 WBGene00012146 status:Predicted SW:Q9U3S4 protein_id:CAB54318.2 /QuerySize=133	gi|6902778|ref|Os03g27300|
gi|55241892|gb|EAA07996.2|	lcl|VC5.1 CE28354 WBGene00020907 status:Predicted SW:Q9TYU2 protein_id:AAC78209.2 /QuerySize=94	gi|6902784|ref|Os03g27330|
gi|55241902|gb|EAA08032.3|	lcl|VC5.3a CE21224 WBGene00003786 locus:npa-1 status:Confirmed SW:Q9TYU6 protein_id:AAC78208.1 /QuerySize=1440	gi|6902791|ref|Os03g27380|
gi|55241906|gb|EAA07760.3|	lcl|VC5.3b CE33332 WBGene00003786 locus:npa-1 status:Confirmed SW:Q86S16 protein_id:AAO25985.1 /QuerySize=399	gi|6902792|ref|Os03g27390|
gi|55241923|gb|EAL40828.1|	lcl|VC5.3c CE36211 WBGene00003786 locus:npa-1 status:Partially_confirmed SW:Q7KNM6 protein_id:AAR25643.1 /QuerySize=1	gi|6902793|ref|Os03g27390|
gi|55241933|gb|EAA07753.2|	lcl|VH15N14R.1 CE20120 WBGene00012151 status:Predicted SW:Q9GNJ5 protein_id:CAB76736.1 /QuerySize=222	gi|6902794|ref|Os03g27390|
gi|55241961|gb|EAA07777.3|	lcl|VK10D6R.1 CE35038 WBGene00012152 status:Predicted SW:Q7YWT4 protein_id:CAE17951.1 /QuerySize=89	gi|6902796|ref|Os03g27410|
gi|55241968|gb|EAA07453.2|	lcl|VW02B12L.4 CE26728 WBGene00012157 status:Confirmed SW:Q23129 protein_id:CAA91404.2 /QuerySize=217	gi|6902799|ref|Os03g27440|
gi|55241987|gb|EAA07780.3|	lcl|VY35H6BL.2 CE32736 WBGene00012161 status:Predicted SW:Q8I134 protein_id:CAD57706.1 /QuerySize=121	gi|6902804|ref|Os03g27470|
gi|55241988|gb|EAL40839.1|	lcl|VZK822L.2 CE32943 WBGene00012163 status:Confirmed SW:Q8I108 protein_id:CAD59158.1 /QuerySize=83	gi|6902806|ref|Os03g27490|
gi|55241992|gb|EAL40841.1|	lcl|W01A11.3 CE31077 WBGene00006815 locus:unc-83 status:Confirmed SW:Q23064 protein_id:AAB04966.2 /QuerySize=1041	gi|6902807|ref|Os03g27500|
gi|55242014|gb|EAA07779.2|	lcl|W01A11.7 CE37796 WBGene00020912 status:Confirmed SW:Q23070 protein_id:AAB04972.2 /QuerySize=186	gi|6902813|ref|Os03g27560|
gi|55242016|gb|EAA07873.2|	lcl|W01A8.1a CE18303 WBGene00007024 locus:mdt-28 status:Confirmed SW:Q23095 protein_id:CAA95854.1 /QuerySize=415	gi|6902818|ref|Os03g27600|
gi|55242021|gb|EAL40844.1|	lcl|W01A8.1b CE06531 WBGene00007024 locus:mdt-28 status:Confirmed SW:Q65ZB1 protein_id:CAH19105.1 /QuerySize=385	gi|6902820|ref|Os03g27620|
gi|55242032|gb|EAA44883.2|	lcl|W01A8.4 CE32507 WBGene00012166 status:Confirmed SW:Q23098 protein_id:CAA95851.2 /QuerySize=172	gi|6902821|ref|Os03g27630|
gi|55242036|gb|EAL40848.1|	lcl|W01A8.5 CE06535 WBGene00012167 status:Partially_confirmed SW:Q23096 protein_id:CAA95849.1 /QuerySize=311	gi|6902822|ref|Os03g27640|

gi|55242037|gb|EAA07698.3| lcl|W01B11.1 CE18305 WBGene00020913 status:Partially_confirmed SW:O61215 protein_id:AAK21474.1 /QuerySize=128 gi|6902824|ref|Os03g27660|
gi|55242039|gb|EAL40849.1| lcl|W01B11.4a CE18309 WBGene00020916 status:Partially_confirmed SW:O45014 protein_id:AAK21477.1 /QuerySize=57 gi|6902826|ref|Os03g27680|
gi|55242040|gb|EAL40850.1| lcl|W01B11.4b CE18308 WBGene00020916 status:Partially_confirmed SW:O45013 protein_id:AAK21476.1 /QuerySize=70 gi|6902827|ref|Os03g27690|
gi|55242046|gb|EAL40852.1| lcl|W01B11.5 CE34380 WBGene00004154 locus:pqn-72 possible trans-splice site at 11908 status:Partially_confirmed SW:O45015 gi|6902828|ref|Os03g27700|
gi|55242048|gb|EAL40853.1| lcl|W01B6.4 CE03756 WBGene00012171 status:Partially_confirmed SW:Q23101 protein_id:CAA92622.1 /QuerySize=63 gi|6902832|ref|Os03g27740|
gi|55242049|gb|EAA07730.2| lcl|W01B6.8 CE03760 WBGene00012174 status:Predicted SW:Q23100 protein_id:CAA92621.1 /QuerySize=170 gi|6902833|ref|Os03g27750|
gi|55242060|gb|EAA44890.2| lcl|W01C8.1 CE05029 WBGene00020918 status:Predicted SW:Q9GYG6 protein_id:AAG00023.1 /QuerySize=98 gi|6902839|ref|Os03g27810|
gi|55242062|gb|EAL40857.1| lcl|W01C9.1 CE35912 WBGene00012175 status:Partially_confirmed SW:Q23114 protein_id:CAA90266.2 /QuerySize=232 gi|6902841|ref|Os03g27830|
gi|55242073|gb|EAL40860.1| lcl|W01C9.2 CE02368 WBGene00012176 status:Predicted SW:Q23118 protein_id:CAA90270.1 /QuerySize=564 gi|6902847|ref|Os03g27890|
gi|55242085|gb|EAA07704.3| lcl|W01C9.3 CE02369 WBGene00004155 locus:pqn-73 status:Partially_confirmed SW:Q23115 protein_id:CAA90267.1 /QuerySize gi|6902850|ref|Os03g27920|
gi|55242093|gb|EAL40864.1| lcl|W01D2.3 CE20126 WBGene00012180 status:Predicted SW:Q9XU65 protein_id:CAB05634.1 /QuerySize=216 gi|6902852|ref|Os03g27940|
gi|55242112|gb|EAA08002.3| lcl|W01D2.5 CE20128 WBGene00012182 status:Partially_confirmed SW:Q9XU63 protein_id:CAB05639.1 /QuerySize=348 gi|6902860|ref|Os03g28000|
gi|55242117|gb|EAL40868.1| lcl|W01D2.6 CE35039 WBGene00012183 status:Predicted SW:Q7YTK2 protein_id:CAE17947.1 /QuerySize=72 gi|6902862|ref|Os03g28020|
gi|55242118|gb|EAL40869.1| lcl|W01G7.4 CE21230 WBGene00012188 status:Confirmed SW:Q9XVH5 protein_id:CAB03456.1 /QuerySize=251 gi|6902863|ref|Os03g28030|
gi|55242122|gb|EAA08003.2| lcl|W01H2.2 CE07561 WBGene00020922 status:Predicted SW:O02045 protein_id:AAB52886.1 /QuerySize=59 gi|6902865|ref|Os03g28050|
gi|55242124|gb|EAL40870.1| lcl|W02A2.8 CE21238 WBGene00012191 status:Partially_confirmed SW:Q9XUB1 protein_id:CAB05311.1 /QuerySize=130 gi|6902867|ref|Os03g28070|
gi|55242130|gb|EAL40874.1| lcl|W02B3.3 CE02075 WBGene00020923 status:Predicted SW:Q09340 protein_id:AAA64321.1 /QuerySize=111 gi|6902871|ref|Os03g28100|
gi|55242134|gb|EAL40875.1| lcl|W02B3.7 CE02079 WBGene00020927 status:Partially_confirmed SW:Q09343 protein_id:AAA64326.1 /QuerySize=162 gi|6902876|ref|Os03g28150|
gi|55242146|gb|EAL40879.1| lcl|W02B8.1 CE24027 WBGene00012197 status:Partially_confirmed SW:Q9TVV2 protein_id:CAA21672.3 /QuerySize=179 gi|6902879|ref|Os03g28180|
gi|55242147|gb|EAA07631.3| lcl|W02C12.2 CE30197 WBGene00020929 status:Confirmed SW:P91525 protein_id:AAB37996.2 /QuerySize=100 gi|6902883|ref|Os03g28220|
gi|55242149|gb|EAL40880.1| lcl|W02D3.10a CE34383 WBGene00020935 status:Partially_confirmed SW:Q7Z151 protein_id:AAP68909.1 /QuerySize=1437 gi|6902885|ref|Os03g28240|
gi|55242152|gb|EAL40881.1| lcl|W02D3.10b CE34384 WBGene00020935 status:Partially_confirmed SW:Q7Z150 protein_id:AAP68910.1 /QuerySize=946 gi|6902886|ref|Os03g28250|
gi|55242153|gb|EAL40882.1| lcl|W02D7.11 CE31081 WBGene00020946 status:Partially_confirmed SW:Q8MQH0 protein_id:AAM48551.1 /QuerySize=83 gi|6902889|ref|Os03g28280|
gi|55242154|gb|EAL40883.1| lcl|W02D7.3 CE14440 WBGene00020939 status:Partially_confirmed SW:O17524 protein_id:AAB63933.1 /QuerySize=143 gi|6902890|ref|Os03g28290|
gi|55242155|gb|EAL40884.1| lcl|W02D7.6 CE31082 WBGene00020942 status:Confirmed SW:O17527 protein_id:AAB63936.2 /QuerySize=322 gi|6902893|ref|Os03g28320|
gi|55242156|gb|EAL40885.1| lcl|W02D7.8 CE35794 WBGene00020943 status:Partially_confirmed SW:O17529 protein_id:AAB63939.2 /QuerySize=135 gi|6902895|ref|Os03g28340|
gi|55242163|gb|EAA07739.2| lcl|W02D7.9 CE14452 WBGene00020944 status:Predicted SW:O17531 protein_id:AAB63938.1 /QuerySize=66 gi|6902896|ref|Os03g28350|
gi|55242179|gb|EAL40891.1| lcl|W02D9.10 CE14472 WBGene00012216 status:Confirmed SW:Q9XVG7 protein_id:CAB03466.1 /QuerySize=182 gi|6902897|ref|Os03g28360|
gi|55242184|gb|EAA07695.3| lcl|W02D9.4 CE14460 WBGene00012210 status:Predicted SW:O02336 protein_id:CAB03471.1 /QuerySize=338 gi|6902900|ref|Os03g28390|
gi|55242187|gb|EAL40892.1| lcl|W02D9.6 CE14464 WBGene00012212 status:Partially_confirmed SW:Q9XVH0 protein_id:CAB03463.1 /QuerySize=86 gi|6902902|ref|Os03g28900|
gi|55242277|gb|EAL40910.1| lcl|W02D9.7 CE14466 WBGene00012213 status:Partially_confirmed SW:Q9XVG9 protein_id:CAB03464.1 /QuerySize=97 gi|6902903|ref|Os03g28910|
gi|55242283|gb|EAL40912.1| lcl|W02D9.8 CE14468 WBGene00012214 status:Predicted SW:Q9XVG5 protein_id:CAB03468.1 /QuerySize=84 gi|6902904|ref|Os03g28920|
gi|55242285|gb|EAL40913.1| lcl|W02D9.9 CE14470 WBGene00012215 status:Predicted SW:Q9XVG6 protein_id:CAB03467.1 /QuerySize=73 gi|6902911|ref|Os03g28970|
gi|55242290|gb|EAL40915.1| lcl|W02F12.3 CE14476 WBGene00020948 status:Partially_confirmed SW:O45146 protein_id:AAC04459.1 /QuerySize=388 gi|6902914|ref|Os03g29000|
gi|55242297|gb|EAA44925.2| lcl|W02F12.4b CE35795 WBGene00020949 status:Partially_confirmed SW:Q71UV4 protein_id:AAQ91897.1 /QuerySize=113 gi|6902917|ref|Os03g29030|
gi|55242306|gb|EAL40919.1| lcl|W02F12.4c CE37159 WBGene00020949 status:Partially_confirmed SW:Q6A581 protein_id:AAT92057.1 /QuerySize=117 gi|6902919|ref|Os03g29050|
gi|55242323|gb|EAA44929.2| lcl|W02F12.6 CE31084 WBGene00020951 status:Confirmed SW:O45149 protein_id:AAC04461.2 /QuerySize=186 gi|6902920|ref|Os03g29060|
gi|55242326|gb|EAA07981.2| lcl|W02H3.1 CE03773 WBGene00012217 status:Partially_confirmed SW:Q23132 protein_id:CAA92022.1 /QuerySize=201 gi|6902921|ref|Os03g29070|
gi|55242327|gb|EAL40923.1| lcl|W02H3.2 CE03774 WBGene00012218 status:Predicted SW:Q23131 protein_id:CAA92021.1 /QuerySize=213 gi|6902922|ref|Os03g29080|
gi|55242328|gb|EAL40924.1| lcl|W02H5.1 CE21250 WBGene00020956 status:Partially_confirmed SW:Q9N5C5 protein_id:AAF60388.1 /QuerySize=93 gi|6902925|ref|Os03g29110|
gi|55242333|gb|EAL40925.1| lcl|W02H5.2 CE21247 WBGene00020957 status:Predicted SW:Q9N5C8 protein_id:AAF60385.1 /QuerySize=201 gi|6902928|ref|Os03g29140|
gi|55242343|gb|EAL40927.1| lcl|W02H5.9 CE35796 WBGene00020963 status:Partially_confirmed SW:Q7KX59 protein_id:AAQ91896.1 /QuerySize=281 gi|6902930|ref|Os03g29160|
gi|55242347|gb|EAL40930.1| lcl|W03A5.2 CE29360 WBGene00020967 status:Predicted SW:Q23135 protein_id:AAL02529.1 /QuerySize=224 gi|6902935|ref|Os03g29200|
gi|55242378|gb|EAL40933.1| lcl|W03A5.5 CE30198 WBGene00020969 status:Partially_confirmed SW:Q23139 protein_id:AAA50729.2 /QuerySize=189 gi|6902938|ref|Os03g29230|
gi|55242379|gb|EAA08090.3| lcl|W03A5.6 CE01424 WBGene00020970 status:Predicted SW:Q23140 protein_id:AAA50732.1 /QuerySize=80 gi|6902942|ref|Os03g29270|
gi|55242380|gb|EAA44945.2| lcl|W03B1.1 CE14488 WBGene00020971 status:Partially_confirmed SW:Q23143 protein_id:AAC24436.1 /QuerySize=118 gi|6902943|ref|Os03g29280|
gi|55242382|gb|EAA07922.2| lcl|W03B1.3 CE07567 WBGene00020973 status:Confirmed SW:Q23142 protein_id:AAC24438.2 /QuerySize=256 gi|6902945|ref|Os03g29300|
gi|55242403|gb|EAL40936.1| lcl|W03B1.5 CE14496 WBGene00020974 status:Predicted SW:O45885 protein_id:AAC24433.1 /QuerySize=581 gi|6902947|ref|Os03g29320|
gi|55242411|gb|EAA07504.2| lcl|W03C9.1 CE32947 WBGene00012219 status:Partially_confirmed SW:Q23147 protein_id:CAA91358.2 /QuerySize=284 gi|6902954|ref|Os03g29370|
gi|55242413|gb|EAL40939.1| lcl|W03C9.2 CE16532 WBGene00012220 status:Partially_confirmed SW:Q23144 protein_id:CAA91360.1 /QuerySize=418 gi|6902955|ref|Os03g29380|
gi|55242419|gb|EAL40940.1| lcl|W03C9.5 CE03779 WBGene00012221 status:Confirmed SW:Q23145 protein_id:CAA91356.1 /QuerySize=79 gi|6902956|ref|Os03g29390|
gi|55242427|gb|EAA44949.2| lcl|W03C9.8 CE32949 WBGene00012223 status:Partially_confirmed SW:Q8I105 protein_id:CAD59163.1 /QuerySize=86 gi|6902957|ref|Os03g29400|
gi|55242451|gb|EAA07477.2| lcl|W03D2.1a CE28501 WBGene00004157 locus:pqn-75 status:Partially_confirmed SW:O02123 protein_id:AAC48255.2 /QuerySiz gi|6902959|ref|Os03g29420|
gi|55242475|gb|EAA07832.3| lcl|W03D2.1b CE31729 WBGene00004157 locus:pqn-75 status:Partially_confirmed SW:Q8MQG8 protein_id:AAM97960.1 /QueryS gi|6902962|ref|Os03g29450|
gi|55242482|gb|EAL40948.1| lcl|W03D2.1c CE14506 WBGene00004157 locus:pqn-75 status:Partially_confirmed SW:Q8MQG9 protein_id:AAM97961.1 /QueryS gi|6902966|ref|Os03g29490|
gi|55242504|gb|EAL40954.1| lcl|W03D2.2 CE14508 WBGene00020979 status:Partially_confirmed SW:O02121 protein_id:AAC48256.1 /QuerySize=239 gi|6902967|ref|Os03g29500|
gi|55242506|gb|EAL40956.1| lcl|W03D2.3 CE14510 WBGene00020980 status:Predicted SW:O02117 protein_id:AAC48254.1 /QuerySize=94 gi|6902968|ref|Os03g29510|
gi|55242508|gb|EAA44974.2| lcl|W03D2.5 CE14514 WBGene00006951 locus:wrt-5 status:Confirmed SW:O02116 protein_id:AAC48258.1 /QuerySize=176 gi|6902970|ref|Os03g29530|
gi|55242512|gb|EAL40959.1| lcl|W03D2.6 CE33718 WBGene00020981 status:Partially_confirmed SW:O02118 protein_id:AAC48261.4 /QuerySize=226 gi|6902976|ref|Os03g29580|
gi|55242516|gb|EAA07422.2| lcl|W03D2.7 CE28845 WBGene00020982 status:Partially_confirmed SW:O02119 protein_id:AAC48260.2 /QuerySize=93 gi|6902977|ref|Os03g29590|
gi|55242535|gb|EAL40964.1| lcl|W03D2.9 CE14522 WBGene00020983 status:Partially_confirmed SW:O02122 protein_id:AAC48253.1 /QuerySize=115 gi|6902979|ref|Os03g29610|
gi|55242536|gb|EAL40965.1| lcl|W03D8.1 CE32224 WBGene00020984 status:Predicted SW:O45004 protein_id:AAK21496.2 /QuerySize=474 gi|6902982|ref|Os03g29640|
gi|55242544|gb|EAA07298.2| lcl|W03D8.3 CE26953 WBGene00020986 status:Partially_confirmed SW:O61214 protein_id:AAK21498.1 /QuerySize=130 gi|6902985|ref|Os03g29670|
gi|55242560|gb|EAL40972.1| lcl|W03F11.5a CE36720 WBGene00021008 status:Partially_confirmed SW:O01781 protein_id:AAK18988.2 /QuerySize=288 gi|6902987|ref|Os03g29690|
gi|55242575|gb|EAL40976.1| lcl|W03F11.5b CE26954 WBGene00021008 status:Partially_confirmed SW:Q9BIB9 protein_id:AAK18991.1 /QuerySize=254 gi|6902988|ref|Os03g29700|
gi|55242594|gb|EAL40979.1| lcl|W03F8.10 CE34385 WBGene00020998 status:Partially_confirmed SW:Q7YZM5 protein_id:AAP46270.1 /QuerySize=587 gi|6902989|ref|Os03g29710|
gi|55242610|gb|EAL40988.1| lcl|W03F8.8 CE35041 WBGene00020996 status:Partially_confirmed SW:O44569 protein_id:AAB94186.2 /QuerySize=57 gi|6902999|ref|Os03g29780|
gi|55242612|gb|EAA07341.2| lcl|W03F9.2a CE14526 WBGene00021000 status:Confirmed SW:O16998 protein_id:AAG24200.1 /QuerySize=236 gi|6903001|ref|Os03g29800|
gi|55242621|gb|EAL40991.1| lcl|W03F9.2b CE24030 WBGene00021000 status:Confirmed SW:Q9TXS2 protein_id:AAG24208.1 /QuerySize=41 gi|6903003|ref|Os03g29820|
gi|55242630|gb|EAL40993.1| lcl|W03F9.3 CE35797 WBGene00021001 status:Partially_confirmed SW:O16996 protein_id:AAG24201.2 /QuerySize=80 gi|6903005|ref|Os03g29840|
gi|55242637|gb|EAA07336.2| lcl|W03G1.2 CE17280 WBGene00021010 status:Partially_confirmed SW:Q9UAY5 protein_id:AAD14750.1 /QuerySize=350 gi|6903008|ref|Os03g29870|
gi|55242638|gb|EAA07309.2| lcl|W03G1.5 CE17283 WBGene00021011 status:Partially_confirmed SW:Q9UAY0 protein_id:AAD14753.1 /QuerySize=471 gi|6903010|ref|Os03g29890|
gi|55242643|gb|EAL40994.1| lcl|W03G1.8 CE17286 WBGene00021013 status:Predicted SW:Q9UAY6 protein_id:AAD14756.1 /QuerySize=214 gi|6903015|ref|Os03g29930|
gi|55242647|gb|EAL40997.1| lcl|W03G11.4 CE18331 WBGene00012226 status:Partially_confirmed SW:Q18002 protein_id:CAA91547.1 /QuerySize=484 gi|6903018|ref|Os03g29960|
gi|55242651|gb|EAL40998.1| lcl|W03G9.2 CE14556 WBGene00021014 status:Partially_confirmed SW:O44749 protein_id:AAB96736.1 /QuerySize=515 gi|6903022|ref|Os03g29990|
gi|55242659|gb|EAL41000.1| lcl|W03G9.3 CE14558 WBGene00021015 status:Partially_confirmed SW:O44748 protein_id:AAB96737.1 /QuerySize=462 gi|6903024|ref|Os03g30010|
gi|55242660|gb|EAA07216.2| lcl|W03H9.2 CE20135 WBGene00012228 status:Partially_confirmed SW:Q9U351 protein_id:CAB63319.1 /QuerySize=167 gi|6903025|ref|Os03g30020|
gi|55242665|gb|EAL41001.1| lcl|W03H9.3 CE20136 WBGene00012229 status:Predicted SW:Q9U352 protein_id:CAB63318.1 /QuerySize=96 gi|6903029|ref|Os03g30060|
gi|55242683|gb|EAA07103.2| lcl|W04A4.2 CE36957 WBGene00012231 status:Predicted SW:O45888 protein_id:CAB04896.2 /QuerySize=218 gi|6903032|ref|Os03g30090|
gi|55242696|gb|EAL41009.1| lcl|W04A4.6 CE35042 WBGene00012235 status:Predicted SW:Q7YWR9 protein_id:CAE17983.1 /QuerySize=185 gi|6903035|ref|Os03g30120|
gi|55242698|gb|EAL41010.1| lcl|W04A8.2 CE16536 WBGene00012237 status:Predicted SW:Q9XUM3 protein_id:CAB04903.1 /QuerySize=103 gi|6903038|ref|Os03g30140|
gi|55242702|gb|EAL41011.1| lcl|W04A8.3 CE16537 WBGene00012238 status:Partially_confirmed SW:Q9XUM2 protein_id:CAB04904.1 /QuerySize=315 gi|6903039|ref|Os03g30150|
gi|55242706|gb|EAL41013.1| lcl|W04A8.6 CE16540 WBGene00012241 status:Partially_confirmed SW:Q9XUM0 protein_id:CAB04906.1 /QuerySize=740 gi|6903045|ref|Os03g30210|
gi|55242709|gb|EAL41014.1| lcl|W04B5.1 CE28846 WBGene00021018 status:Confirmed SW:Q9UA59 protein_id:AAC68914.2 /QuerySize=56 gi|6903047|ref|Os03g30230|
gi|55242716|gb|EAL41017.1| lcl|W04B5.2 CE20139 WBGene00021019 status:Predicted SW:Q9UA60 protein_id:AAC68915.1 /QuerySize=352 gi|6903051|ref|Os03g30270|
gi|55242732|gb|EAL41020.1| lcl|W04B5.6 CE20143 WBGene00021023 status:Predicted SW:Q9UA58 protein_id:AAC68919.1 /QuerySize=179 gi|6903052|ref|Os03g30280|
gi|55242734|gb|EAL41021.1| lcl|W04C9.2 CE18333 WBGene00021024 status:Partially_confirmed SW:Q9TZE0 protein_id:AAC68725.1 /QuerySize=102 gi|6903053|ref|Os03g30290|
gi|55242750|gb|EAA07220.2| lcl|W04C9.4 CE18335 WBGene00021026 status:Confirmed SW:Q9TZE4 protein_id:AAC68727.1 /QuerySize=150 gi|6903056|ref|Os03g30310|
gi|55242777|gb|EAA07195.2| lcl|W04D12.1 CE01425 WBGene00021029 status:Predicted SW:Q23154 protein_id:AAA50745.1 /QuerySize=85 gi|6903058|ref|Os03g30330|
gi|55242778|gb|EAL41033.1| lcl|W04E12.2 CE18997 WBGene00012247 status:Partially_confirmed SW:Q9XUL2 protein_id:CAB04914.1 /QuerySize=199 gi|6903059|ref|Os03g30340|
gi|55242782|gb|EAL41035.1| lcl|W04E12.3 CE18998 WBGene00012248 status:Predicted SW:Q9XUL4 protein_id:CAB04912.1 /QuerySize=75 gi|6903063|ref|Os03g30380|
gi|55242793|gb|EAL41039.1| lcl|W04E12.4 CE34100 WBGene00012249 status:Partially_confirmed SW:Q9XUL3 protein_id:CAB04913.2 /QuerySize=227 gi|6903066|ref|Os03g30410|
gi|55242799|gb|EAL41041.1| lcl|W04E12.5 CE34101 WBGene00012250 status:Partially_confirmed SW:Q9XUL1 protein_id:CAB04915.2 /QuerySize=235 gi|6903069|ref|Os03g30440|
gi|55242808|gb|EAA07197.2| lcl|W04E12.9 CE35043 WBGene00012254 status:Predicted SW:Q7YSV2 protein_id:CAE17980.1 /QuerySize=323 gi|6903073|ref|Os03g30480|
gi|55242817|gb|EAA45012.2| lcl|W04G5.3 CE14586 WBGene00012264 status:Predicted SW:O18168 protein_id:CAB07680.1 /QuerySize=72 gi|6903074|ref|Os03g30490|
gi|55242818|gb|EAA07183.2| lcl|W04H10.1 CE14600 WBGene00021030 status:Partially_confirmed SW:O44852 protein_id:AAB95012.1 /QuerySize=70 gi|6903075|ref|Os03g30500|
gi|55242828|gb|EAL41053.1| lcl|W04H10.2 CE14602 WBGene00021031 status:Predicted SW:O44851 protein_id:AAB95015.1 /QuerySize=154 gi|6903078|ref|Os03g30530|
gi|55242829|gb|EAA07163.2| lcl|W05B10.3 CE06546 WBGene00012278 status:Confirmed SW:Q23172 protein_id:CAA98964.1 /QuerySize=95 gi|6903080|ref|Os03g30540|
gi|55242831|gb|EAL41054.1| lcl|W05B2.7 CE21259 WBGene00012273 status:Partially_confirmed SW:Q9XVG2 protein_id:CAB03476.1 /QuerySize=315 gi|6903082|ref|Os03g30560|
gi|55242832|gb|EAL41055.1| lcl|W05B5.1 CE19005 WBGene00012274 status:Partially_confirmed SW:Q9XUK9 protein_id:CAB04917.1 /QuerySize=209 gi|6903083|ref|Os03g30570|
gi|55242834|gb|EAL41057.1| lcl|W05E10.2 CE06549 WBGene00012281 status:Partially_confirmed SW:Q23174 protein_id:CAB01248.1 /QuerySize=115 gi|6903085|ref|Os03g30590|
gi|55242848|gb|EAL41062.1| lcl|W05E7.3 CE34104 WBGene00001702 locus:grd-13 status:Confirmed SW:Q7Z2A9 protein_id:AAP40518.1 /QuerySize=156 gi|6903086|ref|Os03g30600|
gi|55242850|gb|EAA06974.2| lcl|W05F2.2a CE14614 WBGene00021034 status:Partially_confirmed SW:Q9TYT9 protein_id:AAC78213.1 /QuerySize=457 gi|6903088|ref|Os03g30620|
gi|55242851|gb|EAL41063.1| lcl|W05F2.2b CE28505 WBGene00021034 status:Partially_confirmed SW:Q95X16 protein_id:AAK73901.1 /QuerySize=302 gi|6903089|ref|Os03g30630|
gi|55242856|gb|EAA07060.3| lcl|W05F2.3 CE14616 WBGene00021035 status:Confirmed SW:Q9TYT8 protein_id:AAC78214.1 /QuerySize=222 gi|6903090|ref|Os03g30640|
gi|55242859|gb|EAL41064.1| lcl|W05F2.6 CE21260 WBGene00021038 status:Partially_confirmed SW:Q9TYT7 protein_id:AAC78219.1 /QuerySize=456 gi|6903091|ref|Os03g30650|
gi|55242860|gb|EAL41065.1| lcl|W05F2.7 CE32227 WBGene00021039 status:Predicted SW:Q9TYU0 protein_id:AAC78217.2 /QuerySize=1324 gi|6903092|ref|Os03g30660|
gi|55242862|gb|EAA07011.2| lcl|W05H5.2 CE14638 WBGene00012284 status:Predicted SW:P91839 protein_id:CAB03478.1 /QuerySize=51 gi|6903093|ref|Os03g30670|
gi|55242891|gb|EAL41072.1| lcl|W05H7.1 CE28507 WBGene00021044 status:Confirmed SW:O02176 protein_id:AAB52863.2 /QuerySize=131 gi|6903094|ref|Os03g30680|
gi|55242894|gb|EAL41074.1| lcl|W05H7.2 CE30202 WBGene00021045 status:Predicted SW:O02172 protein_id:AAB52866.2 /QuerySize=109 gi|6903095|ref|Os03g30690|
gi|55242915|gb|EAA06976.3| lcl|W05H7.4c CE31252 WBGene00021047 status:Partially_confirmed SW:Q8MPS8 protein_id:AAM69072.1 /QuerySize=73 gi|6903101|ref|Os03g30750|
gi|55242916|gb|EAL41079.1| lcl|W05H7.4d CE33719 WBGene00021047 status:Confirmed SW:Q86MJ0 protein_id:AAO91680.1 /QuerySize=38 gi|6903104|ref|Os03g30780|
gi|55242925|gb|EAA07015.2| lcl|W05H9.1 CE05036 WBGene00021048 status:Confirmed SW:Q23179 protein_id:AAA81726.1 /QuerySize=414 gi|6903110|ref|Os03g30840|
gi|55242927|gb|EAL41083.1| lcl|W05H9.2 CE33720 WBGene00021049 status:Partially_confirmed SW:Q23178 protein_id:AAO91681.1 /QuerySize=851 gi|6903111|ref|Os03g30850|
gi|55242931|gb|EAL41085.1| lcl|W05H9.3 CE05038 WBGene00021050 status:Confirmed SW:Q23177 protein_id:AAA81724.1 /QuerySize=45 gi|6903114|ref|Os03g30880|
gi|55242939|gb|EAL41087.1| lcl|W05H9.4 CE33721 WBGene00021051 status:Partially_confirmed SW:Q86MI9 protein_id:AAO91682.1 /QuerySize=780 gi|6903116|ref|Os03g30900|
gi|55242947|gb|EAL41088.1| lcl|W06A11.3 CE05041 WBGene00021054 status:Partially_confirmed SW:Q23182 protein_id:AAA82411.1 /QuerySize=261 gi|6903120|ref|Os03g30940|
gi|55242951|gb|EAL41090.1| lcl|W06A11.4 CE05042 WBGene00021055 status:Partially_confirmed SW:Q23180 protein_id:AAA82409.1 /QuerySize=347 gi|6903122|ref|Os03g30960|
gi|55242960|gb|EAL41094.1| lcl|W06A7.2 CE14664 WBGene00012291 status:Partially_confirmed SW:Q23186 protein_id:CAB01521.1 /QuerySize=176 gi|6903128|ref|Os03g31030|
gi|55242964|gb|EAL41096.1| lcl|W06A7.5 CE14672 WBGene00012294 status:Confirmed SW:Q23184 protein_id:CAB01519.1 /QuerySize=34 gi|6903133|ref|Os03g31050|
gi|55242965|gb|EAL41097.1| lcl|W06B11.3 CE31254 WBGene00021060 status:Confirmed SW:Q23190 protein_id:AAA81076.2 /QuerySize=236 gi|6903136|ref|Os03g31080|
gi|55242966|gb|EAL41098.1| lcl|W06B4.1 CE02890 WBGene00021056 status:Partially_confirmed SW:Q23196 protein_id:AAC46817.1 /QuerySize=432 gi|6903139|ref|Os03g31100|
gi|55242970|gb|EAL41099.1| lcl|W06D11.1 CE06552 WBGene00012299 status:Predicted SW:Q17623 protein_id:CAA93539.1 /QuerySize=236 gi|6903141|ref|Os03g31120|
gi|55242983|gb|EAA45030.2| lcl|W06D11.2 CE06553 WBGene00012300 status:Partially_confirmed SW:Q23197 protein_id:CAA93535.1 /QuerySize=119 gi|6903148|ref|Os03g31160|
gi|55243003|gb|EAA06793.2| lcl|W06D11.3 CE06554 WBGene00012301 status:Partially_confirmed SW:Q23198 protein_id:CAA93536.1 /QuerySize=139 gi|6903153|ref|Os03g31190|
gi|55243007|gb|EAL41110.1| lcl|W06D11.5 CE06556 WBGene00012303 status:Predicted SW:Q23199 protein_id:CAA93537.1 /QuerySize=147 gi|6903156|ref|Os03g31220|
gi|55243017|gb|EAL41111.1| lcl|W06D12.6 CE36615 WBGene00012305 status:Partially_confirmed SW:O45895 protein_id:CAB04927.2 /QuerySize=208 gi|6903160|ref|Os03g31250|
gi|55243019|gb|EAA06795.2| lcl|W06D4.2 CE16544 WBGene00012296 status:Partially_confirmed SW:Q9XW44 protein_id:CAA22250.1 /QuerySize=250 gi|6903162|ref|Os03g31270|
gi|55243027|gb|EAL41114.1| lcl|W06E11.2 CE01427 WBGene00021062 status:Partially_confirmed SW:Q23205 protein_id:AAK29862.1 /QuerySize=196 gi|6903163|ref|Os03g31280|
gi|55243029|gb|EAA06935.3| lcl|W06E11.7 CE29056 WBGene00021065 status:Partially_confirmed SW:Q95ZK0 protein_id:AAK95884.1 /QuerySize=100 gi|6903171|ref|Os03g31330|
gi|55243041|gb|EAA06944.2| lcl|W06F12.2a CE24033 WBGene00012306 status:Partially_confirmed SW:Q9U345 protein_id:CAB54314.1 /QuerySize=562 gi|6903173|ref|Os03g31350|
gi|55243055|gb|EAL41120.1| lcl|W06F12.2b CE24034 WBGene00012306 status:Partially_confirmed SW:Q9U344 protein_id:CAB54315.1 /QuerySize=568 gi|6903175|ref|Os03g31370|
gi|55243066|gb|EAL41123.1| lcl|W06F12.2c CE29479 WBGene00012306 status:Partially_confirmed SW:Q8WQB8 protein_id:CAD18877.1 /QuerySize=502 gi|6903176|ref|Os03g31380|
gi|55243072|gb|EAL41127.1| lcl|W06G6.10 CE20155 WBGene00012312 status:Partially_confirmed SW:Q9U341 protein_id:CAB63324.1 /QuerySize=101 gi|6903177|ref|Os03g31390|

gi|55243076|gb|EAL41128.1|	lcl|W06G6.11 CE20156 WBGene00012313 status:Predicted SW:Q9U342 protein_id:CAB63323.1 /QuerySize=138	gi|6903179|ref|Os03g31410|
gi|55243077|gb|EAL41129.1|	lcl|W06G6.12 CE20157 WBGene00012314 status:Predicted SW:Q9U337 protein_id:CAB05644.1 /QuerySize=256	gi|6903180|ref|Os03g31420|
gi|55243080|gb|EAL41131.1|	lcl|W06G6.2 CE20152 WBGene00012309 status:Predicted SW:Q9U339 protein_id:CAB63326.1 /QuerySize=84	gi|6903182|ref|Os03g31440|
gi|55243094|gb|EAA06962.3|	lcl|W06G6.9 CE36411 WBGene00012311 status:Predicted SW:Q9U340 protein_id:CAB63325.2 /QuerySize=100	gi|6903185|ref|Os03g31470|
gi|55243097|gb|EAA06886.2|	lcl|W06H3.1 CE36215 WBGene00012315 [031110 dl] Modified prediction based on EST data	gi|6903188|ref|Os03g31500|
gi|55243098|gb|EAL41133.1|	lcl|W06H8.4 CE21265 WBGene00021067 status:Partially_confirmed SW:Q9N5C1 protein_id:AAF39976.1 /QuerySize=351	gi|6903190|ref|Os03g31520|
gi|55243102|gb|EAA45051.2|	lcl|W06H8.6 CE21264 WBGene00021068 status:Partially_confirmed SW:Q9N5B5 protein_id:AAF39983.2 /QuerySize=327	gi|6903192|ref|Os03g31540|
gi|55243106|gb|EAA06958.2|	lcl|W07A12.8 CE35051 WBGene00012325 status:Predicted SW:Q7YSL9 protein_id:CAE17961.1 /QuerySize=151	gi|6903197|ref|Os03g31580|
gi|55243108|gb|EAL41135.1|	lcl|W07B3.2a CE17290 WBGene00001561 locus:gei-4 status:Confirmed SW:Q9TZ39 protein_id:AAC68913.1 /QuerySize=545	gi|6903198|ref|Os03g31590|
gi|55243114|gb|EAL41137.1|	lcl|W07B3.2b CE17291 WBGene00001561 locus:gei-4 status:Confirmed SW:Q9TZ38 protein_id:AAC68910.1 /QuerySize=568	gi|6903199|ref|Os03g31600|
gi|55243118|gb|EAL41139.1|	lcl|W07B3.2c CE34603 WBGene00001561 locus:gei-4 status:Confirmed SW:Q9TZ40 protein_id:AAP82634.1 /QuerySize=542	gi|6903206|ref|Os03g31670|
gi|55243134|gb|EAA06949.2|	lcl|W07B3.2d CE32743 WBGene00001561 locus:gei-4 status:Confirmed SW:Q8ITW3 protein_id:AAN65284.1 /QuerySize=279	gi|6903207|ref|Os03g31680|
gi|55243144|gb|EAL41147.1|	lcl|W07B3.2e CE34604 WBGene00001561 locus:gei-4 status:Partially_confirmed SW:Q7YZW8 protein_id:AAP82635.1 /QuerySize	gi|6903209|ref|Os03g31700|
gi|55243160|gb|EAA06783.2|	lcl|W07B3.2f CE37419 WBGene00001561 locus:gei-4 status:Confirmed SW:Q65CL5 protein_id:AAU20837.1 /QuerySize=564	gi|6903210|ref|Os03g31710|
gi|55243162|gb|EAL41152.1|	lcl|W07E11.2 CE02373 WBGene00001446 locus:flp-3 FMRFamide neuropeptide repeats status:Confirmed SW:Q23212 protein_id:	gi|6903211|ref|Os03g31720|
gi|55243177|gb|EAA06382.2|	lcl|W07E11.3a CE02374 WBGene00001445 locus:flp-2 status:Confirmed SW:Q23213 protein_id:CAA90031.1 /QuerySize=106	gi|6903212|ref|Os03g31720|
gi|55243178|gb|EAA06534.2|	lcl|W07E11.3b CE28258 WBGene00001445 locus:flp-2 status:Confirmed SW:O61465 protein_id:CAC42354.1 /QuerySize=75	gi|6903215|ref|Os03g31740|
gi|55243181|gb|EAA06677.2|	lcl|W07E11.4 CE35052 WBGene00012327 status:Predicted SW:Q7YWT6 protein_id:CAE17946.1 /QuerySize=71	gi|6903219|ref|Os03g31770|
gi|55243188|gb|EAA45061.2|	lcl|W07E6.5 CE17297 WBGene00021076 status:Predicted SW:Q9TYV4 protein_id:AAC78180.1 /QuerySize=241	gi|6903221|ref|Os03g31790|
gi|55243193|gb|EAA06681.2|	lcl|W07G1.7 CE24041 WBGene00012334 status:Partially_confirmed SW:Q9XUJ8 protein_id:CAB04939.1 /QuerySize=169	gi|6903222|ref|Os03g31800|
gi|55243197|gb|EAL41156.1|	lcl|W07G4.1 CE14684 WBGene00012335 status:Partially_confirmed SW:Q23216 protein_id:CAB01445.1 /QuerySize=126	gi|6903223|ref|Os03g31810|
gi|55243203|gb|EAA06331.2|	lcl|W08A12.2 CE14698 WBGene00021079 status:Confirmed SW:O01620 protein_id:AAB52321.1 /QuerySize=59	gi|6903224|ref|Os03g31820|
gi|55243212|gb|EAA45070.2|	lcl|W08A12.3 CE14700 WBGene00021080 status:Partially_confirmed SW:O01621 protein_id:AAB52319.1 /QuerySize=198	gi|6903225|ref|Os03g31830|
gi|55243221|gb|EAL41161.1|	lcl|W08D2.3 CE06558 WBGene00012340 status:Partially_confirmed SW:Q23220 protein_id:CAA94232.1 /QuerySize=562	gi|6903226|ref|Os03g31840|
gi|55243223|gb|EAL41163.1|	lcl|W08E12.2 CE21279 WBGene00021083 status:Partially_confirmed SW:Q9N5B0 protein_id:AAF39989.1 /QuerySize=120	gi|6903228|ref|Os03g31860|
gi|55243225|gb|EAA06148.2|	lcl|W08E12.3 CE21278 WBGene00021084 status:Confirmed SW:Q9N5B1 protein_id:AAF39988.1 /QuerySize=140	gi|6903229|ref|Os03g31870|
gi|55243236|gb|EAA06278.2|	lcl|W08E12.4 CE21277 WBGene00021085 status:Predicted SW:Q9N2L6 protein_id:AAF39987.1 /QuerySize=141	gi|6903232|ref|Os03g31900|
gi|55243243|gb|EAL41167.1|	lcl|W08E12.5 CE21277 WBGene00021086 status:Predicted SW:Q9N2L6 protein_id:AAF39986.1 /QuerySize=141	gi|6903237|ref|Os03g31950|
gi|55243251|gb|EAL41170.1|	lcl|W08E12.6 CE21276 WBGene00021087 status:Partially_confirmed SW:Q9N5B2 protein_id:AAF39985.1 /QuerySize=215	gi|6903238|ref|Os03g31960|
gi|55243266|gb|EAA06171.3|	lcl|W08E12.8 CE24042 WBGene00021089 status:Partially_confirmed SW:Q9N5B4 protein_id:AAF39991.1 /QuerySize=151	gi|6903239|ref|Os03g31970|
gi|55243267|gb|EAA45079.2|	lcl|W08E12.9 CE24043 WBGene00021090 status:Predicted SW:Q9N5A9 protein_id:AAF39992.1 /QuerySize=111	gi|6903243|ref|Os03g32010|
gi|55243271|gb|EAL41176.1|	lcl|W08E3.2 CE26028 WBGene00012343 status:Confirmed SW:P91916 protein_id:CAB07130.2 /QuerySize=584	gi|6903245|ref|Os03g32030|
gi|55243278|gb|EAL41179.1|	lcl|W08E3.4 CE32508 WBGene00012345 status:Partially_confirmed SW:P91915 protein_id:CAB07129.2 /QuerySize=127	gi|6903250|ref|Os03g32080|
gi|55243287|gb|EAA45090.2|	lcl|W08F4.1 CE14712 WBGene00021091 status:Predicted SW:O02114 protein_id:AAC71168.1 /QuerySize=97	gi|6903253|ref|Os03g32110|
gi|55243289|gb|EAL41183.1|	lcl|W08F4.10 CE14730 WBGene00021099 status:Predicted SW:O02112 protein_id:AAC71174.1 /QuerySize=207	gi|6903254|ref|Os03g32120|
gi|55243290|gb|EAL41184.1|	lcl|W08F4.11 CE14732 WBGene00021100 status:Predicted SW:O02113 protein_id:AAC71175.1 /QuerySize=112	gi|6903256|ref|Os03g32140|
gi|55243292|gb|EAL41185.1|	lcl|W08F4.12 CE30207 WBGene00021101 status:Confirmed SW:Q8WQH4 protein_id:AAL65799.1 /QuerySize=92	gi|6903263|ref|Os03g32200|
gi|55243314|gb|EAA06603.2|	lcl|W08F4.5 CE14720 WBGene00021094 status:Predicted SW:O02105 protein_id:AAC71170.1 /QuerySize=193	gi|6903264|ref|Os03g32210|
gi|55243316|gb|EAA06646.2|	lcl|W08F4.7 CE14724 WBGene00021096 status:Predicted SW:O02107 protein_id:AAC71176.1 /QuerySize=138	gi|6903267|ref|Os03g32240|
gi|55243325|gb|EAL41195.1|	lcl|W08G11.5 CE35053 WBGene00012349 status:Predicted SW:Q7YTJ5 protein_id:CAE17970.1 /QuerySize=129	gi|6903268|ref|Os03g32250|
gi|55243328|gb|EAL41197.1|	lcl|W09B6.3 CE32072 WBGene00021103 status:Partially_confirmed SW:Q9GZI7 protein_id:AAG00030.3 /QuerySize=578	gi|6903269|ref|Os03g32260|
gi|55243330|gb|EAL41199.1|	lcl|W09B6.4 CE33469 WBGene00021104 status:Partially_confirmed SW:Q9GZI5 protein_id:AAG00031.2 /QuerySize=271	gi|6903271|ref|Os03g32280|
gi|55243342|gb|EAL41201.1|	lcl|W09B6.5 CE14742 WBGene00021105 status:Predicted SW:Q9GZI4 protein_id:AAG00032.1 /QuerySize=270	gi|6903272|ref|Os03g32290|
gi|55243353|gb|EAA45096.2|	lcl|W09B7.1 CE21283 WBGene00021106 status:Predicted SW:Q9N5A5 protein_id:AAF60392.1 /QuerySize=346	gi|6903273|ref|Os03g32300|
gi|55243354|gb|EAA06584.2|	lcl|W09B7.2 CE21282 WBGene00021107 status:Predicted SW:Q9N5A6 protein_id:AAF60391.1 /QuerySize=316	gi|6903275|ref|Os03g32320|
gi|55243361|gb|EAA06594.2|	lcl|W09C3.2 CE14746 WBGene00021110 status:Predicted SW:P91531 protein_id:AAC24411.1 /QuerySize=460	gi|6903277|ref|Os03g32340|
gi|55243362|gb|EAA06558.3|	lcl|W09C3.7 CE14756 WBGene00021114 status:Confirmed SW:P91532 protein_id:AAC24415.1 /QuerySize=172	gi|6903278|ref|Os03g32350|
gi|55243363|gb|EAA45097.2|	lcl|W09C3.8 CE14758 WBGene00021115 status:Predicted SW:P91533 protein_id:AAC24416.1 /QuerySize=373	gi|6903282|ref|Os03g32390|
gi|55243368|gb|EAA06123.3|	lcl|W09C5.9 CE20172 WBGene00012355 status:Predicted SW:Q9U328 protein_id:CAB63335.1 /QuerySize=46	gi|6903283|ref|Os03g32400|
gi|55243372|gb|EAL41206.1|	lcl|W09D10.5 CE37798 WBGene00012363 status:Partially_confirmed SW:O18184 protein_id:CAB07861.3 /QuerySize=321	gi|6903284|ref|Os03g32410|
gi|55243374|gb|EAL41207.1|	lcl|W09D12.2 CE33198 WBGene00005212 locus:srg-55 G-protein coupled receptor	gi|6903285|ref|Os03g32420|
gi|55243394|gb|EAA06590.2|	lcl|W09D12.3 CE33199 WBGene00005211 locus:srg-54 G-protein coupled receptor	gi|6903286|ref|Os03g32430|
gi|55243396|gb|EAA06753.2|	lcl|W09D6.4 CE19018 WBGene00012357 status:Partially_confirmed SW:Q9XUJ5 protein_id:CAB04943.1 /QuerySize=148	gi|6903287|ref|Os03g32440|
gi|55243404|gb|EAL41213.1|	lcl|W09D6.5 CE19019 WBGene00012358 status:Confirmed SW:Q9XUJ2 protein_id:CAB04946.1 /QuerySize=257	gi|6903288|ref|Os03g32450|
gi|55243412|gb|EAL41216.1|	lcl|W09G12.6 CE18345 WBGene00021120 status:Partially_confirmed SW:O45197 protein_id:AAC04448.1 /QuerySize=178	gi|6903289|ref|Os03g32460|
gi|55243415|gb|EAL41217.1|	lcl|W09G12.7 CE18346 WBGene00021121 status:Partially_confirmed SW:O45198 protein_id:AAC04447.1 /QuerySize=368	gi|6903300|ref|Os03g32570|
gi|55243416|gb|EAL41218.1|	lcl|W09G12.9 CE18348 WBGene00021123 status:Partially_confirmed SW:O45200 protein_id:AAC04452.1 /QuerySize=137	gi|6903304|ref|Os03g32610|
gi|55243429|gb|EAA06686.2|	lcl|W09G3.3 CE34215 WBGene00012367 status:Partially_confirmed SW:Q7YTU9 protein_id:CAD92380.1 /QuerySize=200	gi|6903307|ref|Os03g32640|
gi|55243434|gb|EAA06609.2|	lcl|W09G3.7b CE34217 WBGene00012370 status:Partially_confirmed SW:Q7YTU8 protein_id:CAD92381.1 /QuerySize=98	gi|6903309|ref|Os03g32660|
gi|55243438|gb|EAA45112.2|	lcl|W09H1.3 CE16573 WBGene00012373 status:Partially_confirmed SW:O45901 protein_id:CAB04956.1 /QuerySize=105	gi|6903310|ref|Os03g32670|
gi|55243440|gb|EAL41224.1|	lcl|W09H1.4 CE16574 WBGene00012374 status:Predicted SW:O45902 protein_id:CAB04957.1 /QuerySize=134	gi|6903311|ref|Os03g32680|
gi|55243447|gb|EAL41225.1|	lcl|W10C4.1 CE32510 WBGene00021124 status:Predicted SW:Q9N5A3 protein_id:AAF39993.2 /QuerySize=223	gi|6903314|ref|Os03g32710|
gi|55243454|gb|EAA45114.2|	lcl|W10C8.3 CE32744 WBGene00021126 status:Predicted SW:O45008 protein_id:AAK21501.2 /QuerySize=68	gi|6903318|ref|Os03g32750|
gi|55243459|gb|EAL41227.1|	lcl|W10C8.4b CE33586 WBGene00021127 status:Confirmed SW:Q86GV4 protein_id:AAO61419.1 /QuerySize=41	gi|6903319|ref|Os03g32760|
gi|55243460|gb|EAA06174.3|	lcl|W10D9.1 CE14786 WBGene00021129 status:Predicted SW:O17284 protein_id:AAB71055.1 /QuerySize=199	gi|6903321|ref|Os03g32780|
gi|55243461|gb|EAA06169.2|	lcl|W10D9.2 CE36027 WBGene00021130 status:Partially_confirmed SW:O17283 protein_id:AAB71056.3 /QuerySize=157	gi|6903324|ref|Os03g32800|
gi|55243466|gb|EAL41228.1|	lcl|W10D9.3 CE14790 WBGene00021131 status:Partially_confirmed SW:O17285 protein_id:AAB71057.1 /QuerySize=394	gi|6903326|ref|Os03g32820|
gi|55243473|gb|EAA06170.2|	lcl|W10G11.17 CE14832 WBGene00021147 status:Partially_confirmed SW:O44918 protein_id:AAG24223.1 /QuerySize=439	gi|6903327|ref|Os03g32830|
gi|55243477|gb|EAL41231.1|	lcl|W10G11.19 CE30208 WBGene00021148 status:Confirmed SW:O44923 protein_id:AAK82922.2 /QuerySize=508	gi|6903332|ref|Os03g32880|
gi|55243481|gb|EAL41232.1|	lcl|W10G11.20 CE28781 WBGene00021149 status:Confirmed SW:Q965K5 protein_id:AAK82923.1 /QuerySize=123	gi|6903333|ref|Os03g32890|
gi|55243485|gb|EAA06746.2|	lcl|W10G6.1 CE14796 WBGene00012378 status:Predicted SW:Q94364 protein_id:CAB03484.1 /QuerySize=85	gi|6903335|ref|Os03g32910|
gi|55243487|gb|EAL41233.1|	lcl|Y102A11A.1 CE26480 WBGene00022411 status:Partially_confirmed SW:Q95XJ6 protein_id:AAK39313.1 /QuerySize=296	gi|6903342|ref|Os03g32980|
gi|55243490|gb|EAL41234.1|	lcl|Y102A11A.2a CE29093 WBGene00022412 status:Partially_confirmed SW:Q95XK1 protein_id:AAK39314.2 /QuerySize=772	gi|6903343|ref|Os03g32990|
gi|55243498|gb|EAL41235.1|	lcl|Y102A11A.2b CE36332 WBGene00022412 status:Partially_confirmed SW:Q7KWZ7 protein_id:AAR85899.1 /QuerySize=753	gi|6903344|ref|Os03g33000|
gi|55243503|gb|EAL41236.1|	lcl|Y102A11A.3 CE31255 WBGene00022413 status:Partially_confirmed SW:Q95XK2 protein_id:AAM69073.1 /QuerySize=1515	gi|6903345|ref|Os03g33010|
gi|55243514|gb|EAA06167.2|	lcl|Y102A11A.5 CE26476 WBGene00022415 status:Confirmed SW:Q95XK0 protein_id:AAK39311.1 /QuerySize=132	gi|6903347|ref|Os03g33030|
gi|55243517|gb|EAA06164.3|	lcl|Y102A11A.7 CE26474 WBGene00022417 status:Partially_confirmed SW:Q95XJ8 protein_id:AAK39310.1 /QuerySize=172	gi|6903351|ref|Os03g33070|
gi|55243519|gb|EAL41237.1|	lcl|Y102A11A.9 CE31256 WBGene00022419 status:Partially_confirmed SW:Q8MXR9 protein_id:AAM69074.1 /QuerySize=339	gi|6903355|ref|Os03g33110|
gi|55243520|gb|EAL41238.1|	lcl|Y102A5C.2 CE19288 WBGene00013611 status:Partially_confirmed SW:Q9XX92 protein_id:CAA20943.1 /QuerySize=172	gi|6903359|ref|Os03g33150|
gi|55243522|gb|EAL41240.1|	lcl|Y102A5C.27 CE20399 WBGene00013628 status:Predicted SW:Q9XX67 protein_id:CAA20968.1 /QuerySize=262	gi|6903364|ref|Os03g33200|
gi|55243523|gb|EAL41241.1|	lcl|Y102A5C.33 CE20404 WBGene00013629 locus:srz-95 status:Predicted SW:Q9XX65 protein_id:CAA20970.1 /QuerySize=326	gi|6903365|ref|Os03g33210|
gi|55243524|gb|EAL41242.1|	lcl|Y102A5D.1 CE19291 WBGene00013630 status:Predicted SW:Q9XX64 protein_id:CAA20973.1 /QuerySize=416	gi|6903375|ref|Os03g33310|
gi|55243534|gb|EAL41244.1|	lcl|Y102E9.1a CE28101 WBGene00003851 locus:odr-4 status:Confirmed SW:Q23233 protein_id:AAA93427.2 /QuerySize=445	gi|6903377|ref|Os03g33330|
gi|55243543|gb|EAL41245.1|	lcl|Y102E9.1b CE32953 WBGene00003851 locus:odr-4 status:Confirmed SW:Q8I7F8 protein_id:AAN84808.1 /QuerySize=475	gi|6903379|ref|Os03g33350|
gi|55243544|gb|EAA06711.2|	lcl|Y102E9.1c CE32954 WBGene00003851 locus:odr-4 status:Confirmed SW:Q8I7F7 protein_id:AAN84809.1 /QuerySize=473	gi|6903381|ref|Os03g33370|
gi|55243546|gb|EAL41246.1|	lcl|Y102E9.3 CE07575 WBGene00022421 status:Partially_confirmed SW:Q23234 protein_id:AAA93428.1 /QuerySize=123	gi|6903382|ref|Os03g33380|
gi|55243547|gb|EAA06748.2|	lcl|Y102E9.4 CE07576 WBGene00022422 status:Predicted SW:Q23235 protein_id:AAA93429.1 /QuerySize=59	gi|6903383|ref|Os03g33390|
gi|55243552|gb|EAL41247.1|	lcl|Y102F5A.1 CE19293 WBGene00013631 status:Predicted SW:Q9XWT8 protein_id:CAA21557.1 /QuerySize=399	gi|6903384|ref|Os03g33400|
gi|55243565|gb|EAL41248.1|	lcl|Y104H12D.2 CE31631 WBGene00022426 status:Confirmed SW:Q9N5A2 protein_id:AAM97962.1 /QuerySize=327	gi|6903385|ref|Os03g33410|
gi|55243566|gb|EAA06435.2|	lcl|Y104H12D.4 CE31633 WBGene00022428 status:Confirmed SW:Q8MXU4 protein_id:AAM97963.1 /QuerySize=107	gi|6903386|ref|Os03g33420|
gi|55243570|gb|EAA06202.2|	lcl|Y105C5A.1 CE23184 WBGene00013632 status:Partially_confirmed SW:Q9NF71 protein_id:CAB54981.1 /QuerySize=767	gi|6903387|ref|Os03g33430|
gi|55243589|gb|EAL41253.1|	lcl|Y105C5A.10 CE23191 WBGene00013635 status:Partially_confirmed SW:Q9NF66 protein_id:CAB54989.1 /QuerySize=46	gi|6903388|ref|Os03g33440|
gi|55243594|gb|EAA06227.2|	lcl|Y105C5A.12 CE23193 WBGene00013636 status:Predicted SW:Q7K702 protein_id:CAB54991.1 /QuerySize=76	gi|6903389|ref|Os03g33450|
gi|55243601|gb|EAA06353.3|	lcl|Y105C5A.13 CE36217 WBGene00013637 status:Confirmed SW:Q9N678 protein_id:CAB54992.2 /QuerySize=114	gi|6903391|ref|Os03g33470|
gi|55243617|gb|EAL41262.1|	lcl|Y105C5A.22 CE23201 WBGene00013641 status:Predicted SW:Q9NF65 protein_id:CAB55000.1 /QuerySize=174	gi|6903392|ref|Os03g33480|
gi|55243623|gb|EAL41263.1|	lcl|Y105C5A.25 CE24057 WBGene00013644 status:Partially_confirmed SW:Q9NF63 protein_id:CAB55005.1 /QuerySize=296	gi|6903395|ref|Os03g33510|
gi|55243626|gb|EAA06731.2|	lcl|Y105C5A.8 CE36219 WBGene00013633 status:Partially_confirmed SW:Q9NF68 protein_id:CAB54987.2 /QuerySize=324	gi|6903398|ref|Os03g33540|
gi|55243630|gb|EAL41265.1|	lcl|Y105C5A.9 CE23190 WBGene00013634 status:Predicted SW:Q9NF67 protein_id:CAB54988.1 /QuerySize=108	gi|6903399|ref|Os03g33550|
gi|55243634|gb|EAA06206.2|	lcl|Y105C5B.1 CE35643 WBGene00013645 status:Partially_confirmed SW:Q9U318 protein_id:CAB60310.2 /QuerySize=311	gi|6903400|ref|Os03g33560|
gi|55243643|gb|EAA06217.2|	lcl|Y105C5B.20 CE24070 WBGene00013659 status:Partially_confirmed SW:Q9NAM1 protein_id:CAB54366.1 /QuerySize=222	gi|6903406|ref|Os03g33620|
gi|55243646|gb|EAA45150.2|	lcl|Y105C5B.27 CE24077 WBGene00013664 status:Predicted SW:Q9NAL6 protein_id:CAB54375.1 /QuerySize=413	gi|6903407|ref|Os03g33630|
gi|55243662|gb|EAL41273.1|	lcl|Y105C5B.8 CE35645 WBGene00013649 status:Partially_confirmed SW:Q9NAM8 protein_id:CAB54354.2 /QuerySize=447	gi|6903410|ref|Os03g33660|
gi|55243663|gb|EAL41274.1|	lcl|Y105E8A.13 CE25169 WBGene00013673 status:Predicted SW:Q9NEY3 protein_id:CAC48133.1 /QuerySize=325	gi|6903411|ref|Os03g33670|
gi|55243670|gb|EAA06722.2|	lcl|Y105E8A.15 CE29834 WBGene00013675 status:Predicted SW:Q8WQA9 protein_id:CAD21664.1 /QuerySize=94	gi|6903413|ref|Os03g33690|
gi|55243680|gb|EAL41277.1|	lcl|Y105E8A.25 CE29843 WBGene00013682 status:Partially_confirmed SW:Q8WQA2 protein_id:CAD21671.1 /QuerySize=418	gi|6903421|ref|Os03g33770|
gi|55243687|gb|EAA45157.2|	lcl|Y105E8A.27 CE34222 WBGene00013684 status:Partially_confirmed SW:Q7YTU3 protein_id:CAD92402.1 /QuerySize=260	gi|6903422|ref|Os03g33780|
gi|55243688|gb|EAA06133.2|	lcl|Y105E8A.28 CE34223 WBGene00013685 status:Predicted SW:Q7YTU2 protein_id:CAD92403.1 /QuerySize=210	gi|6903424|ref|Os03g33800|
gi|55243689|gb|EAA45158.2|	lcl|Y105E8A.29 CE35056 WBGene00013686 status:Partially_confirmed SW:Q7YTJ0 protein_id:CAE17987.1 /QuerySize=405	gi|6903425|ref|Os03g33810|
gi|55243690|gb|EAA06135.2|	lcl|Y105E8A.7c CE33723 WBGene00001147 locus:eat-18 status:Confirmed SW:Q86DB4 protein_id:CAD88218.1 /QuerySize=71	gi|6903434|ref|Os03g33900|
gi|55243692|gb|EAL41279.1|	lcl|Y105E8A.7d CE37055 WBGene00001147 locus:eat-18 status:Confirmed SW:Q6EUT2 protein_id:CAH04765.1 /QuerySize=78	gi|6903435|ref|Os03g33910|
gi|55243696|gb|EAL41280.1|	lcl|Y105E8B.6 CE24100 WBGene00013691 status:Partially_confirmed SW:Q9NF10 protein_id:CAB60854.1 /QuerySize=71	gi|6903436|ref|Os03g33920|
gi|55243699|gb|EAL41281.1|	lcl|Y106G6A.2a CE30567 WBGene00013695 status:Confirmed SW:Q9XWQ4 protein_id:CAA21595.2 /QuerySize=428	gi|6903439|ref|Os03g33950|
gi|55243701|gb|EAL41283.1|	lcl|Y106G6A.4 CE30569 WBGene00013696 status:Partially_confirmed SW:Q9XWQ2 protein_id:CAA21597.2 /QuerySize=175	gi|6903441|ref|Os03g33970|
gi|55243711|gb|EAL41285.1|	lcl|Y106G6A.5 CE19298 WBGene00013697 status:Partially_confirmed SW:Q9XWQ1 protein_id:CAA21598.1 /QuerySize=210	gi|6903442|ref|Os03g33980|
gi|55243715|gb|EAL41286.1|	lcl|Y106G6D.1 CE19299 WBGene00013698 status:Partially_confirmed SW:Q9XX63 protein_id:CAA20974.1 /QuerySize=287	gi|6903443|ref|Os03g33990|
gi|55243718|gb|EAA06579.3|	lcl|Y106G6D.2 CE19300 WBGene00013699 status:Partially_confirmed SW:Q9XX62 protein_id:CAA20975.1 /QuerySize=248	gi|6903445|ref|Os03g34010|
gi|55243721|gb|EAA06136.2|	lcl|Y106G6D.3 CE19301 WBGene00013700 status:Partially_confirmed SW:Q9XX61 protein_id:CAA20976.1 /QuerySize=555	gi|6903446|ref|Os03g34020|
gi|55243724|gb|EAL41287.1|	lcl|Y106G6E.2 CE20406 WBGene00013706 status:Partially_confirmed SW:Q8WPJ8 protein_id:CAA21729.1 /QuerySize=238	gi|6903447|ref|Os03g34030|
gi|55243726|gb|EAL41288.1|	lcl|Y106G6E.3 CE20407 WBGene00013707 status:Predicted SW:Q9XWE4 protein_id:CAA21730.1 /QuerySize=223	gi|6903449|ref|Os03g34050|
gi|55243730|gb|EAL41289.1|	lcl|Y106G6G.1 CE23211 WBGene00013710 status:Partially_confirmed SW:Q23311 protein_id:CAB00066.1 /QuerySize=368	gi|6903451|ref|Os03g34070|
gi|55243732|gb|EAA06566.3|	lcl|Y106G6G.4 CE23214 WBGene00013713 status:Partially_confirmed SW:Q9XWA9 protein_id:CAA22054.1 /QuerySize=315	gi|6903452|ref|Os03g34080|
gi|55243737|gb|EAL41291.1|	lcl|Y106G6H.1 CE20411 WBGene00013716 status:Partially_confirmed SW:Q9U301 protein_id:CAB63336.1 /QuerySize=453	gi|6903457|ref|Os03g34130|
gi|55243738|gb|EAL41292.1|	lcl|Y106G6H.13 CE20422 WBGene00013723 status:Partially_confirmed SW:Q9XWS1 protein_id:CAA21576.1 /QuerySize=138	gi|6903459|ref|Os03g34150|
gi|55243760|gb|EAL41297.1|	lcl|Y106G6H.16 CE23216 WBGene00013726 status:Partially_confirmed SW:Q9XWR9 protein_id:CAA21579.1 /QuerySize=137	gi|6903460|ref|Os03g34160|
gi|55243773|gb|EAL41302.1|	lcl|Y108F1.3 CE31827 WBGene00022431 status:Predicted SW:Q8MXK5 protein_id:AAN01434.1 /QuerySize=262	gi|6903466|ref|Os03g34220|
gi|55243783|gb|EAA06220.2|	lcl|Y108G3AL.5 CE36512 WBGene00022436 status:Predicted SW:Q95Y31 protein_id:AAK72071.2 /QuerySize=134	gi|6903471|ref|Os03g34270|
gi|55243788|gb|EAL41305.1|	lcl|Y108G3AL.6 CE29605 WBGene00022437 status:Confirmed SW:Q95Y34 protein_id:AAL13326.1 /QuerySize=16	gi|6903472|ref|Os03g34280|
gi|55243795|gb|EAA06548.2|	lcl|Y110A2AL.10 CE23218 WBGene00022446 status:Predicted SW:Q9N496 protein_id:AAF60396.1 /QuerySize=269	gi|6903473|ref|Os03g34290|
gi|55243796|gb|EAA06145.2|	lcl|Y110A2AL.4a CE33472 WBGene00022441 status:Confirmed SW:Q86DM1 protein_id:AAO38586.1 /QuerySize=181	gi|6903476|ref|Os03g34310|
gi|55243801|gb|EAA06612.3|	lcl|Y110A2AL.4b CE33473 WBGene00022441 status:Confirmed SW:Q86DM0 protein_id:AAO38587.1 /QuerySize=163	gi|6903477|ref|Os03g34310|
gi|55243808|gb|EAL41311.1|	lcl|Y110A2AL.6 CE36029 WBGene00022443 status:Partially_confirmed SW:Q9N4A3 protein_id:AAF60399.2 /QuerySize=160	gi|6903478|ref|Os03g34320|
gi|55243809|gb|EAL41312.1|	lcl|Y110A2AL.7 CE23221 WBGene00022444 status:Partially_confirmed SW:Q9N4A2 protein_id:AAF60398.1 /QuerySize=162	gi|6903482|ref|Os03g35350|
gi|55243826|gb|EAL41319.1|	lcl|Y110A2AM.1 CE33474 WBGene00022449 status:Partially_confirmed SW:Q965Y4 protein_id:AAK85498.2 /QuerySize=318	gi|6903483|ref|Os03g35360|
gi|55243846|gb|EAA45184.2|	lcl|Y110A2AM.4 CE34393 WBGene00022451 status:Confirmed SW:Q7Z149 protein_id:AAP68911.1 /QuerySize=38	gi|6903484|ref|Os03g35370|

<table>
<tr><td>gi|55243856|gb|EAL41324.1|</td><td>lcl|Y110A7A.11 CE34394 WBGene00022460 status:Partially_confirmed SW:Q9N598 protein_id:AAF60428.2 /QuerySize=254</td><td>gi|6903488|ref|Os03g35410|</td></tr>
<tr><td>gi|55243860|gb|EAA05947.2|</td><td>lcl|Y110A7A.15b CE33206 WBGene00022462 status:Confirmed SW:Q8IAB5 protein_id:AAO12404.1 /QuerySize=65</td><td>gi|6903489|ref|Os03g35420|</td></tr>
<tr><td>gi|55243867|gb|EAL41327.1|</td><td>lcl|Y111B2A.14 CE28848 WBGene00004161 locus:pqn-80 status:Partially_confirmed SW:Q9NEX0 protein_id:CAC35843.2 /Query</td><td>gi|6903491|ref|Os03g35440|</td></tr>
<tr><td>gi|55243868|gb|EAL41328.1|</td><td>lcl|Y111B2A.21 CE23261 WBGene00013741 status:Predicted SW:Q9NEL0 protein_id:CAC35850.1 /QuerySize=240</td><td>gi|6903493|ref|Os03g35460|</td></tr>
<tr><td>gi|55243870|gb|EAA05961.2|</td><td>lcl|Y111B2A.27 CE35058 WBGene00013744 status:Predicted SW:Q7YTJ7 protein_id:CAE17963.1 /QuerySize=237</td><td>gi|6903494|ref|Os03g35470|</td></tr>
<tr><td>gi|55243875|gb|EAL41329.1|</td><td>lcl|Y111B2A.3 CE23259 WBGene00013729 status:Partially_confirmed SW:Q9NEM5 protein_id:CAB60972.1 /QuerySize=718</td><td>gi|6903496|ref|Os03g35490|</td></tr>
<tr><td>gi|55243876|gb|EAA06073.2|</td><td>lcl|Y111B2A.4 CE37278 WBGene00013730 status:Partially_confirmed SW:Q9BHL8 protein_id:CAC35834.2 /QuerySize=459</td><td>gi|6903497|ref|Os03g35500|</td></tr>
<tr><td>gi|55243877|gb|EAA06119.2|</td><td>lcl|Y111B2A.5a CE37279 WBGene00013731 status:Partially_confirmed /QuerySize=434</td><td>gi|6903499|ref|Os03g35520|</td></tr>
<tr><td>gi|55243899|gb|EAA05958.2|</td><td>lcl|Y111B2A.9a CE28698 WBGene00013733 status:Partially_confirmed SW:Q9BHL5 protein_id:CAC35837.2 /QuerySize=406</td><td>gi|6903500|ref|Os03g35530|</td></tr>
<tr><td>gi|55243900|gb|EAA06083.2|</td><td>lcl|Y111B2A.9b CE34504 WBGene00013733 status:Partially_confirmed SW:Q7YTV1 protein_id:CAD91706.2 /QuerySize=326</td><td>gi|6903502|ref|Os03g35550|</td></tr>
<tr><td>gi|55243901|gb|EAA06067.3|</td><td>lcl|Y113G7A.12 CE36453 WBGene00013749 status:Partially_confirmed SW:Q6EUT5 protein_id:CAH04763.1 /QuerySize=133</td><td>gi|6903506|ref|Os03g35590|</td></tr>
<tr><td>gi|55243902|gb|EAA06082.2|</td><td>lcl|Y113G7A.13 CE24142 WBGene00013750 status:Partially_confirmed SW:Q9U2Y3 protein_id:CAB60485.1 /QuerySize=107</td><td>gi|6903509|ref|Os03g35620|</td></tr>
<tr><td>gi|55243904|gb|EAA06068.2|</td><td>lcl|Y113G7A.14 CE23283 WBGene00013751 status:Partially_confirmed SW:Q9U2Y4 protein_id:CAB60484.1 /QuerySize=204</td><td>gi|6903510|ref|Os03g35630|</td></tr>
<tr><td>gi|55243912|gb|EAL41336.1|</td><td>lcl|Y113G7A.15 CE27229 WBGene00013752 status:Confirmed SW:Q95ZJ6 protein_id:CAC42356.1 /QuerySize=110</td><td>gi|6903511|ref|Os03g35640|</td></tr>
<tr><td>gi|55243922|gb|EAA06056.3|</td><td>lcl|Y113G7A.16 CE32004 WBGene00013753 status:Confirmed SW:Q8I4F0 protein_id:CAD54159.1 /QuerySize=151</td><td>gi|6903512|ref|Os03g35650|</td></tr>
<tr><td>gi|55243928|gb|EAL41341.1|</td><td>lcl|Y113G7B.18 CE23298 WBGene00007017 locus:mdt-17 status:Partially_confirmed SW:Q9NAL4 protein_id:CAB76740.1 /Query</td><td>gi|6903518|ref|Os03g35710|</td></tr>
<tr><td>gi|55243933|gb|EAL41342.1|</td><td>lcl|Y113G7B.21 CE24143 WBGene00013767 status:Partially_confirmed SW:Q9U2W7 protein_id:CAB54339.1 /QuerySize=171</td><td>gi|6903522|ref|Os03g35750|</td></tr>
<tr><td>gi|55243948|gb|EAA06116.2|</td><td>lcl|Y113G7B.25 CE32006 WBGene00013769 status:Partially_confirmed SW:Q8I4E9 protein_id:CAD54161.1 /QuerySize=49</td><td>gi|6903523|ref|Os03g35760|</td></tr>
<tr><td>gi|55243963|gb|EAL41347.1|</td><td>lcl|Y116A8B.1 CE16679 WBGene00013779 status:Partially_confirmed SW:O62401 protein_id:CAA16287.1 /QuerySize=207</td><td>gi|6903524|ref|Os03g35770|</td></tr>
<tr><td>gi|55243965|gb|EAA05953.2|</td><td>lcl|Y116A8B.4 CE16681 WBGene00013781 status:Predicted SW:O62402 protein_id:CAA16289.1 /QuerySize=284</td><td>gi|6903526|ref|Os03g35790|</td></tr>
<tr><td>gi|55243969|gb|EAL41348.1|</td><td>lcl|Y116A8C.11 CE23322 WBGene00013791 status:Partially_confirmed SW:Q9U2T3 protein_id:CAB55144.1 /QuerySize=281</td><td>gi|6903528|ref|Os03g35810|</td></tr>
<tr><td>gi|55243977|gb|EAL41349.1|</td><td>lcl|Y116A8C.15 CE29061 WBGene00013793 status:Partially_confirmed SW:Q9U2V6 protein_id:CAB55121.3 /QuerySize=173</td><td>gi|6903529|ref|Os03g35820|</td></tr>
<tr><td>gi|55243981|gb|EAA06113.3|</td><td>lcl|Y116A8C.23 CE24149 WBGene00013800 status:Partially_confirmed SW:Q9U2S2 protein_id:CAB55155.1 /QuerySize=264</td><td>gi|6903532|ref|Os03g35850|</td></tr>
<tr><td>gi|55243984|gb|EAL41351.1|</td><td>lcl|Y116A8C.29 CE35648 WBGene00013806 status:Partially_confirmed SW:Q9U2U2 protein_id:CAB55135.2 /QuerySize=86</td><td>gi|6903535|ref|Os03g35880|</td></tr>
<tr><td>gi|55243991|gb|EAL41353.1|</td><td>lcl|Y116A8C.33 CE24151 WBGene00013809 status:Partially_confirmed SW:Q9U2S0 protein_id:CAB55157.1 /QuerySize=712</td><td>gi|6903536|ref|Os03g35890|</td></tr>
<tr><td>gi|55243997|gb|EAA06014.2|</td><td>lcl|Y116A8C.43 CE28699 WBGene00013814 status:Predicted SW:Q9U2U4 protein_id:CAB55133.2 /QuerySize=241</td><td>gi|6903537|ref|Os03g35900|</td></tr>
<tr><td>gi|55244016|gb|EAA05944.3|</td><td>lcl|Y116A8C.44 CE35060 WBGene00013815 status:Predicted SW:Q7YWS0 protein_id:CAE17981.1 /QuerySize=74</td><td>gi|6903539|ref|Os03g35920|</td></tr>
<tr><td>gi|55244021|gb|EAL41361.1|</td><td>lcl|Y116A8C.6 CE23317 WBGene00013787 status:Partially_confirmed SW:Q9U2S9 protein_id:CAB55148.1 /QuerySize=105</td><td>gi|6903540|ref|Os03g35930|</td></tr>
<tr><td>gi|55244028|gb|EAL41364.1|</td><td>lcl|Y116F11A.3 CE28970 WBGene00013817 status:Partially_confirmed SW:Q9U3T3 protein_id:CAB60761.2 /QuerySize=119</td><td>gi|6903544|ref|Os03g35970|</td></tr>
<tr><td>gi|55244029|gb|EAL41365.1|</td><td>lcl|Y116F11B.11 CE26045 WBGene00013827 status:Predicted SW:Q9GRV6 protein_id:CAC14393.1 /QuerySize=511</td><td>gi|6903545|ref|Os03g35980|</td></tr>
<tr><td>gi|55244030|gb|EAL41366.1|</td><td>lcl|Y116F11B.14 CE25729 WBGene00013829 status:Predicted SW:Q9NEJ3 protein_id:CAB81984.1 /QuerySize=266</td><td>gi|6903546|ref|Os03g35990|</td></tr>
<tr><td>gi|55244058|gb|EAL41375.1|</td><td>lcl|Y116F11B.6 CE24168 WBGene00013822 status:Predicted SW:Q9NEK3 protein_id:CAB81974.1 /QuerySize=345</td><td>gi|6903548|ref|Os03g36010|</td></tr>
<tr><td>gi|55244067|gb|EAL41376.1|</td><td>lcl|Y116F11B.8 CE24165 WBGene00013824 status:Predicted SW:Q9NEK0 protein_id:CAB81977.1 /QuerySize=367</td><td>gi|6903551|ref|Os03g36040|</td></tr>
<tr><td>gi|55244069|gb|EAA05856.2|</td><td>lcl|Y119C1A.1 CE34395 WBGene00022466 status:Partially_confirmed SW:Q9N582 protein_id:AAF60432.2 /QuerySize=256</td><td>gi|6903554|ref|Os03g36070|</td></tr>
<tr><td>gi|55244099|gb|EAL41381.1|</td><td>lcl|Y119C1B.1 CE29607 WBGene00022467 status:Partially_confirmed SW:Q95Y76 protein_id:AAK39322.2 /QuerySize=273</td><td>gi|6903555|ref|Os03g36080|</td></tr>
<tr><td>gi|55244100|gb|EAL41382.1|</td><td>lcl|Y119C1B.10 CE27232 WBGene00022475 status:Partially_confirmed SW:Q95Y75 protein_id:AAK39327.1 /QuerySize=304</td><td>gi|6903556|ref|Os03g36090|</td></tr>
<tr><td>gi|55244104|gb|EAL41384.1|</td><td>lcl|Y119C1B.6 CE25215 WBGene00022472 status:Partially_confirmed SW:Q95Y81 protein_id:AAK39319.1 /QuerySize=80</td><td>gi|6903557|ref|Os03g36100|</td></tr>
<tr><td>gi|55244106|gb|EAL41385.1|</td><td>lcl|Y119C1B.9 CE25213 WBGene00022474 status:Predicted SW:Q95Y79 protein_id:AAK39318.1 /QuerySize=274</td><td>gi|6903558|ref|Os03g36110|</td></tr>
<tr><td>gi|55244109|gb|EAA45242.2|</td><td>lcl|Y119D3B.11 CE32745 WBGene00022488 status:Partially_confirmed SW:Q95Y69 protein_id:AAK29718.3 /QuerySize=612</td><td>gi|6903565|ref|Os03g36180|</td></tr>
<tr><td>gi|55244119|gb|EAL41391.1|</td><td>lcl|Y119D3B.21 CE29608 WBGene00022497 status:Confirmed SW:Q95Y68 protein_id:AAL11465.1 /QuerySize=66</td><td>gi|6903566|ref|Os03g36190|</td></tr>
<tr><td>gi|55244123|gb|EAA05831.2|</td><td>lcl|Y119D3B.3 CE37280 WBGene00022481 locus:srz-9 status:Predicted SW:Q9BLB2 protein_id:AAK29723.2 /QuerySize=281</td><td>gi|6903567|ref|Os03g36200|</td></tr>
<tr><td>gi|55244124|gb|EAL41392.1|</td><td>lcl|Y119D3B.5 CE37280 WBGene00022483 locus:srz-8 status:Predicted SW:Q9BLB2 protein_id:AAK29721.3 /QuerySize=281</td><td>gi|6903568|ref|Os03g36210|</td></tr>
<tr><td>gi|55244132|gb|EAA05737.2|</td><td>lcl|Y11D7A.10 CE20193 WBGene00012434 status:Confirmed SW:Q9U2R7 protein_id:CAB63344.1 /QuerySize=70</td><td>gi|6903572|ref|Os03g36250|</td></tr>
<tr><td>gi|55244133|gb|EAA05744.2|</td><td>lcl|Y11D7A.5 CE20188 WBGene00012429 status:Partially_confirmed SW:Q9XWQ8 protein_id:CAA21590.1 /QuerySize=161</td><td>gi|6903573|ref|Os03g36260|</td></tr>
<tr><td>gi|55244154|gb|EAA05749.3|</td><td>lcl|Y11D7A.6 CE20189 WBGene00012430 reverse transcriptase status:Predicted SW:Q9U2R8 protein_id:CAB63343.1 /QuerySize</td><td>gi|6903579|ref|Os03g36320|</td></tr>
<tr><td>gi|55244157|gb|EAA05788.3|</td><td>lcl|Y11D7A.7 CE20190 WBGene00012431 status:Predicted SW:Q9XWR5 protein_id:CAA21583.1 /QuerySize=199</td><td>gi|6903581|ref|Os03g36340|</td></tr>
<tr><td>gi|55244158|gb|EAA05814.2|</td><td>lcl|Y11D7A.9 CE20192 WBGene00012433 status:Partially_confirmed SW:Q9XWR3 protein_id:CAA21585.1 /QuerySize=297</td><td>gi|6903586|ref|Os03g36390|</td></tr>
<tr><td>gi|55244162|gb|EAL41401.1|</td><td>lcl|Y12A6A.1 CE19031 WBGene00012439 status:Confirmed SW:Q9XXJ4 protein_id:CAA19444.1 /QuerySize=209</td><td>gi|6903588|ref|Os03g36410|</td></tr>
<tr><td>gi|55244170|gb|EAA05738.2|</td><td>lcl|Y12A6A.2 CE19032 WBGene00012440 status:Confirmed SW:Q9XXJ3 protein_id:CAA19445.1 /QuerySize=75</td><td>gi|6903589|ref|Os03g36420|</td></tr>
<tr><td>gi|55244171|gb|EAA05732.2|</td><td>lcl|Y13C8A.1 CE24172 WBGene00021188 status:Predicted SW:Q9N581 protein_id:AAF60433.1 /QuerySize=107</td><td>gi|6903591|ref|Os03g36440|</td></tr>
<tr><td>gi|55244172|gb|EAA45265.2|</td><td>lcl|Y13C8A.2 CE21363 WBGene00021189 status:Partially_confirmed SW:Q7KX53 protein_id:AAR85898.1 /QuerySize=89</td><td>gi|6903593|ref|Os03g36460|</td></tr>
<tr><td>gi|55244173|gb|EAL41405.1|</td><td>lcl|Y14H12A.1 CE26051 WBGene00021190 status:Partially_confirmed SW:Q966E1 protein_id:AAK84582.1 /QuerySize=136</td><td>gi|6903597|ref|Os03g36500|</td></tr>
<tr><td>gi|55244174|gb|EAA05734.2|</td><td>lcl|Y14H12B.1a CE21365 WBGene00021191 zinc-finger protein status:Confirmed SW:Q9TYP7 protein_id:AAD12808.1 /QuerySize</td><td>gi|6903598|ref|Os03g36510|</td></tr>
<tr><td>gi|55244175|gb|EAA05726.2|</td><td>lcl|Y14H12B.1b CE32075 WBGene00021191 status:Confirmed SW:Q8ITV5 protein_id:AAN60502.1 /QuerySize=217</td><td>gi|6903599|ref|Os03g36520|</td></tr>
<tr><td>gi|55244176|gb|EAA05807.2|</td><td>lcl|Y15E3A.3 CE24174 WBGene00012442 status:Predicted SW:Q9U2R4 protein_id:CAB60331.1 /QuerySize=624</td><td>gi|6903611|ref|Os03g36640|</td></tr>
<tr><td>gi|55244177|gb|EAL41406.1|</td><td>lcl|Y15E3A.5 CE31634 WBGene00012444 status:Confirmed SW:Q8MPS4 protein_id:CAD44159.1 /QuerySize=83</td><td>gi|6903618|ref|Os03g36710|</td></tr>
<tr><td>gi|55244183|gb|EAL41407.1|</td><td>lcl|Y17D7B.2 CE16591 WBGene00012450 status:Predicted SW:O45909 protein_id:CAA16298.1 /QuerySize=249</td><td>gi|6903624|ref|Os03g36770|</td></tr>
<tr><td>gi|55244192|gb|EAL41410.1|</td><td>lcl|Y17D7B.3 CE16592 WBGene00012451 status:Partially_confirmed SW:O45911 protein_id:CAA16300.1 /QuerySize=259</td><td>gi|6903627|ref|Os03g36800|</td></tr>
<tr><td>gi|55244193|gb|EAL41411.1|</td><td>lcl|Y17D7B.4 CE16593 WBGene00012452 status:Confirmed SW:O62404 protein_id:CAA16301.1 /QuerySize=149</td><td>gi|6903628|ref|Os03g36810|</td></tr>
<tr><td>gi|55244203|gb|EAA05790.2|</td><td>lcl|Y17D7C.1 CE16596 WBGene00012455 status:Predicted SW:O62407 protein_id:CAA16295.1 /QuerySize=241</td><td>gi|6903629|ref|Os03g36820|</td></tr>
<tr><td>gi|55244220|gb|EAL41417.1|</td><td>lcl|Y17D7C.2 CE28972 WBGene00012456 status:Predicted SW:O62408 protein_id:CAA16296.2 /QuerySize=240</td><td>gi|6903631|ref|Os03g36840|</td></tr>
<tr><td>gi|55244223|gb|EAL41419.1|</td><td>lcl|Y17G7A.1 CE21378 WBGene00001977 locus:hmg-12 status:Confirmed SW:O45912 protein_id:CAA15962.1 /QuerySize=315</td><td>gi|6903632|ref|Os03g36850|</td></tr>
<tr><td>gi|55244224|gb|EAL41420.1|</td><td>lcl|Y17G7B.20 CE32515 WBGene00012471 status:Confirmed SW:Q8I4E8 protein_id:CAD56603.1 /QuerySize=294</td><td>gi|6903634|ref|Os03g36870|</td></tr>
<tr><td>gi|55244228|gb|EAL41421.1|</td><td>lcl|Y17G7B.21 CE32516 WBGene00012472 status:Partially_confirmed SW:Q8I4E7 protein_id:CAD56604.1 /QuerySize=196</td><td>gi|6903641|ref|Os03g36940|</td></tr>
<tr><td>gi|55244229|gb|EAL41422.1|</td><td>lcl|Y17G7B.22 CE32517 WBGene00012473 status:Confirmed SW:Q8I4E6 protein_id:CAD56605.1 /QuerySize=167</td><td>gi|6903642|ref|Os03g36950|</td></tr>
<tr><td>gi|55244230|gb|EAL41423.1|</td><td>lcl|Y17G7B.23 CE37937 WBGene00044030 status:Predicted /QuerySize=308</td><td>gi|6903646|ref|Os03g36990|</td></tr>
<tr><td>gi|55244232|gb|EAL41424.1|</td><td>lcl|Y17G7B.24 CE37938 WBGene00044033 status:Predicted /QuerySize=117</td><td>gi|6903647|ref|Os03g37000|</td></tr>
<tr><td>gi|55244233|gb|EAA05664.3|</td><td>lcl|Y17G7B.5b CE36030 WBGene00003154 locus:mcm-2 status:Confirmed SW:Q7K7J6 protein_id:CAE53737.1 /QuerySize=125</td><td>gi|6903649|ref|Os03g37020|</td></tr>
<tr><td>gi|55244240|gb|EAA05661.3|</td><td>lcl|Y17G9A.2 CE27467 WBGene00021195 status:Partially_confirmed SW:Q9N486 protein_id:AAF60437.2 /QuerySize=240</td><td>gi|6903651|ref|Os03g37040|</td></tr>
<tr><td>gi|55244244|gb|EAA05683.2|</td><td>lcl|Y17G9A.3 CE24177 WBGene00021196 status:Partially_confirmed SW:Q9N485 protein_id:AAF60439.1 /QuerySize=234</td><td>gi|6903653|ref|Os03g37060|</td></tr>
<tr><td>gi|55244246|gb|EAL41427.1|</td><td>lcl|Y17G9A.4 CE24178 WBGene00021197 status:Predicted SW:Q9N484 protein_id:AAF60440.1 /QuerySize=384</td><td>gi|6903658|ref|Os03g37110|</td></tr>
<tr><td>gi|55244248|gb|EAL41428.1|</td><td>lcl|Y17G9B.1 CE34605 WBGene00021198 status:Partially_confirmed SW:Q9N572 protein_id:AAF60443.2 /QuerySize=480</td><td>gi|6903662|ref|Os03g37150|</td></tr>
<tr><td>gi|55244250|gb|EAA05680.2|</td><td>lcl|Y17G9B.2 CE24182 WBGene00021199 status:Partially_confirmed SW:Q9N573 protein_id:AAF60451.1 /QuerySize=37</td><td>gi|6903663|ref|Os03g37160|</td></tr>
<tr><td>gi|55244251|gb|EAA05682.3|</td><td>lcl|Y17G9B.6 CE24185 WBGene00021203 status:Predicted SW:Q9N578 protein_id:AAF60447.1 /QuerySize=159</td><td>gi|6903664|ref|Os03g37170|</td></tr>
<tr><td>gi|55244261|gb|EAA05676.2|</td><td>lcl|Y17G9B.9 CE24188 WBGene00021206 status:Confirmed SW:Q9N575 protein_id:AAF60450.1 /QuerySize=346</td><td>gi|6903669|ref|Os03g37220|</td></tr>
<tr><td>gi|55244264|gb|EAL41430.1|</td><td>lcl|Y18D10A.1 CE21397 WBGene00012474 status:Partially_confirmed SW:Q9XW25 protein_id:CAA22308.1 /QuerySize=1634</td><td>gi|6903670|ref|Os03g37230|</td></tr>
<tr><td>gi|55244274|gb|EAA05718.2|</td><td>lcl|Y18D10A.11 CE21407 WBGene00012481 status:Confirmed SW:Q9XW20 protein_id:CAA22314.1 /QuerySize=248</td><td>gi|6903676|ref|Os03g37280|</td></tr>
<tr><td>gi|55244275|gb|EAA05674.2|</td><td>lcl|Y18D10A.21 CE36229 WBGene00012486 status:Partially_confirmed SW:Q9XW04 protein_id:CAA22331.2 /QuerySize=286</td><td>gi|6903678|ref|Os03g37300|</td></tr>
<tr><td>gi|55244294|gb|EAL41437.1|</td><td>lcl|Y18D10A.26 CE37165 WBGene00023459 status:Predicted SW:Q67X93 protein_id:CAH19089.1 /QuerySize=274</td><td>gi|6903679|ref|Os03g37310|</td></tr>
<tr><td>gi|55244297|gb|EAL41440.1|</td><td>lcl|Y18D10A.4 CE21400 WBGene00012477 status:Predicted SW:Q9XW23 protein_id:CAA22310.1 /QuerySize=158</td><td>gi|6903682|ref|Os03g37340|</td></tr>
<tr><td>gi|55244298|gb|EAA05656.2|</td><td>lcl|Y18H1A.2 CE21438 WBGene00021208 status:Confirmed SW:Q9BL93 protein_id:AAK21506.1 /QuerySize=270</td><td>gi|6903684|ref|Os03g37360|</td></tr>
<tr><td>gi|55244307|gb|EAA05585.2|</td><td>lcl|Y18H1A.8 CE30211 WBGene00021212 status:Partially_confirmed SW:Q9BL87 protein_id:AAK21511.2 /QuerySize=151</td><td>gi|6903689|ref|Os03g37410|</td></tr>
<tr><td>gi|55244309|gb|EAL41444.1|</td><td>lcl|Y19D10A.1 CE21453 WBGene00021216 status:Predicted SW:Q966D0 protein_id:AAK68409.1 /QuerySize=117</td><td>gi|6903692|ref|Os03g37440|</td></tr>
<tr><td>gi|55244313|gb|EAL41446.1|</td><td>lcl|Y19D10A.14 CE21443 WBGene00021229 status:Predicted SW:Q966D1 protein_id:AAK68414.1 /QuerySize=63</td><td>gi|6903693|ref|Os03g37450|</td></tr>
<tr><td>gi|55244318|gb|EAL41451.1|</td><td>lcl|Y19D10B.1 CE27595 WBGene00021230 status:Predicted SW:Q966C9 protein_id:AAK68423.1 /QuerySize=341</td><td>gi|6903694|ref|Os03g37460|</td></tr>
<tr><td>gi|55244330|gb|EAL41452.1|</td><td>lcl|Y19D10B.7 CE25224 WBGene00021236 status:Confirmed SW:Q9N565 protein_id:AAF59424.1 /QuerySize=151</td><td>gi|6903697|ref|Os03g37480|</td></tr>
<tr><td>gi|55244334|gb|EAL41453.1|</td><td>lcl|Y19D2B.2 CE35065 WBGene00012490 status:Predicted SW:Q7YSR6 protein_id:CAE17976.1 /QuerySize=166</td><td>gi|6903700|ref|Os03g37510|</td></tr>
<tr><td>gi|55244341|gb|EAA05608.2|</td><td>lcl|Y1A5A.2 CE20176 WBGene00012380 status:Predicted SW:Q9XXT6 protein_id:CAA15979.1 /QuerySize=83</td><td>gi|6903701|ref|Os03g37520|</td></tr>
<tr><td>gi|55244379|gb|EAL41467.1|</td><td>lcl|Y1B5A.1 CE24189 WBGene00021150 status:Partially_confirmed SW:Q9N479 protein_id:AAF59431.1 /QuerySize=90</td><td>gi|6903702|ref|Os03g37530|</td></tr>
<tr><td>gi|55244400|gb|EAA45308.2|</td><td>lcl|Y20C6A.3 CE16601 WBGene00012493 status:Predicted SW:O45914 protein_id:CAA16304.1 /QuerySize=301</td><td>gi|6903703|ref|Os03g37540|</td></tr>
<tr><td>gi|55244401|gb|EAA05596.2|</td><td>lcl|Y20F4.2 CE36335 WBGene00021237 status:Partially_confirmed SW:Q95XA8 protein_id:AAL77187.3 /QuerySize=473</td><td>gi|6903705|ref|Os03g37560|</td></tr>
<tr><td>gi|55244402|gb|EAL41469.1|</td><td>lcl|Y20F4.4 CE30313 WBGene00021239 status:Partially_confirmed SW:Q95XA7 protein_id:AAL00866.2 /QuerySize=1319</td><td>gi|6903706|ref|Os03g37570|</td></tr>
<tr><td>gi|55244404|gb|EAA05573.3|</td><td>lcl|Y22D7AL.1 CE23425 WBGene00021241 status:Predicted SW:Q965P3 protein_id:AAK84583.1 /QuerySize=123</td><td>gi|6903708|ref|Os03g37590|</td></tr>
<tr><td>gi|55244411|gb|EAL41471.1|</td><td>lcl|Y22D7AL.12 CE26493 WBGene00021250 status:Predicted SW:Q965P5 protein_id:AAK84586.1 /QuerySize=85</td><td>gi|6903709|ref|Os03g37600|</td></tr>
<tr><td>gi|55244412|gb|EAL41472.1|</td><td>lcl|Y22D7AL.13 CE23437 WBGene00021251 status:Predicted SW:Q965P4 protein_id:AAK84587.1 /QuerySize=61</td><td>gi|6903711|ref|Os03g37620|</td></tr>
<tr><td>gi|55244432|gb|EAL41477.1|</td><td>lcl|Y22D7AL.2 CE26509 WBGene00021242 status:Predicted SW:Q965P6 protein_id:AAK84591.1 /QuerySize=184</td><td>gi|6903715|ref|Os03g37660|</td></tr>
<tr><td>gi|55244451|gb|EAL41481.1|</td><td>lcl|Y22D7AL.3 CE26508 WBGene00021243 status:Predicted SW:Q965P7 protein_id:AAK84592.1 /QuerySize=90</td><td>gi|6903717|ref|Os03g37680|</td></tr>
<tr><td>gi|55244452|gb|EAA05532.2|</td><td>lcl|Y22D7AL.4 CE27243 WBGene00021244 status:Partially_confirmed SW:Q965P9 protein_id:AAK84593.1 /QuerySize=451</td><td>gi|6903718|ref|Os03g37690|</td></tr>
<tr><td>gi|55244461|gb|EAA05471.2|</td><td>lcl|Y22D7AL.6 CE26503 WBGene00021245 status:Partially_confirmed SW:Q965Q3 protein_id:AAK84595.1 /QuerySize=153</td><td>gi|6903719|ref|Os03g37700|</td></tr>
<tr><td>gi|55244462|gb|EAL41483.1|</td><td>lcl|Y22D7AL.7 CE27245 WBGene00021246 status:Partially_confirmed SW:Q965Q5 protein_id:AAK84596.1 /QuerySize=385</td><td>gi|6903722|ref|Os03g37730|</td></tr>
<tr><td>gi|55244463|gb|EAL41484.1|</td><td>lcl|Y22D7AR.10 CE27246 WBGene00021263 status:Partially_confirmed SW:Q9BKX4 protein_id:AAK29877.1 /QuerySize=69</td><td>gi|6903725|ref|Os03g37760|</td></tr>
<tr><td>gi|55244466|gb|EAA05602.2|</td><td>lcl|Y22D7AR.3 CE23432 WBGene00021257 status:Predicted SW:Q9BKY1 protein_id:AAK29872.1 /QuerySize=65</td><td>gi|6903726|ref|Os03g37770|</td></tr>
<tr><td>gi|55244469|gb|EAL41486.1|</td><td>lcl|Y22D7AR.4 CE23431 WBGene00021258 status:Partially_confirmed SW:Q9BKY0 protein_id:AAK29871.1 /QuerySize=181</td><td>gi|6903727|ref|Os03g37780|</td></tr>
<tr><td>gi|55244470|gb|EAL41487.1|</td><td>lcl|Y23B4A.1 CE24190 WBGene00021267 status:Predicted SW:Q9N564 protein_id:AAF60453.1 /QuerySize=124</td><td>gi|6903729|ref|Os03g37800|</td></tr>
<tr><td>gi|55244477|gb|EAL41489.1|</td><td>lcl|Y23B4A.2 CE21455 WBGene00021268 status:Confirmed SW:Q9N563 protein_id:AAF60452.1 /QuerySize=86</td><td>gi|6903730|ref|Os03g37810|</td></tr>
<tr><td>gi|55244479|gb|EAA05622.2|</td><td>lcl|Y23H5A.1b CE31095 WBGene00000801 locus:crs-2 status:Confirmed SW:Q8MPQ4 protein_id:AAM54171.1 /QuerySize=55</td><td>gi|6903731|ref|Os03g37810|</td></tr>
<tr><td>gi|55244480|gb|EAL41490.1|</td><td>lcl|Y23H5A.2 CE18358 WBGene00021269 status:Partially_confirmed SW:O76617 protein_id:AAC64628.1 /QuerySize=197</td><td>gi|6903732|ref|Os03g37820|</td></tr>
<tr><td>gi|55244493|gb|EAL41491.1|</td><td>lcl|Y23H5A.3 CE18359 WBGene00021270 status:Partially_confirmed SW:O76616 protein_id:AAC64630.1 /QuerySize=307</td><td>gi|6903735|ref|Os03g37850|</td></tr>
<tr><td>gi|55244494|gb|EAL41492.1|</td><td>lcl|Y23H5B.1 CE23009 WBGene00021272 status:Predicted SW:Q9N471 protein_id:AAF60461.1 /QuerySize=369</td><td>gi|6903737|ref|Os03g37870|</td></tr>
<tr><td>gi|55244495|gb|EAA05493.2|</td><td>lcl|Y23H5B.2 CE23008 WBGene00021273 status:Predicted SW:Q9N473 protein_id:AAF60460.1 /QuerySize=139</td><td>gi|6903738|ref|Os03g37880|</td></tr>
<tr><td>gi|55244505|gb|EAL41496.1|</td><td>lcl|Y23H5B.3 CE23007 WBGene00021274 status:Predicted SW:Q9N475 protein_id:AAF60459.1 /QuerySize=196</td><td>gi|6903741|ref|Os03g37910|</td></tr>
<tr><td>gi|55244506|gb|EAA05413.3|</td><td>lcl|Y23H5B.8 CE23004 WBGene00021279 status:Partially_confirmed SW:Q9N474 protein_id:AAF60455.1 /QuerySize=174</td><td>gi|6903744|ref|Os03g37940|</td></tr>
<tr><td>gi|55244522|gb|EAA05409.2|</td><td>lcl|Y24D9A.1a CE21464 WBGene00021281 status:Confirmed SW:Q9N554 protein_id:AAF60470.1 /QuerySize=670</td><td>gi|6903748|ref|Os03g37980|</td></tr>
<tr><td>gi|55244530|gb|EAA05406.2|</td><td>lcl|Y24D9A.1b CE29994 WBGene00021281 status:Confirmed SW:Q8WTN0 protein_id:AAL32234.1 /QuerySize=487</td><td>gi|6903753|ref|Os03g38030|</td></tr>
<tr><td>gi|55244532|gb|EAL41500.1|</td><td>lcl|Y24D9A.5 CE21458 WBGene00021283 status:Partially_confirmed SW:Q9N558 protein_id:AAF60464.1 /QuerySize=282</td><td>gi|6903754|ref|Os03g38040|</td></tr>
<tr><td>gi|55244544|gb|EAA05384.2|</td><td>lcl|Y24D9A.6 CE21457 WBGene00021284 status:Partially_confirmed SW:Q9N556 protein_id:AAF60463.1 /QuerySize=157</td><td>gi|6903756|ref|Os03g38060|</td></tr>
<tr><td>gi|55244545|gb|EAL41507.1|</td><td>lcl|Y24D9A.7 CE21456 WBGene00021285 status:Partially_confirmed SW:Q9N555 protein_id:AAF60462.1 /QuerySize=77</td><td>gi|6903759|ref|Os03g38090|</td></tr>
<tr><td>gi|55244546|gb|EAA05387.3|</td><td>lcl|Y24D9B.1 CE31635 WBGene00021287 status:Partially_confirmed SW:Q9TYQ2 protein_id:AAD12803.2 /QuerySize=550</td><td>gi|6903762|ref|Os03g38120|</td></tr>
<tr><td>gi|55244555|gb|EAA05329.2|</td><td>lcl|Y24F12A.3 CE24194 WBGene00012498 status:Predicted SW:Q9U2Q6 protein_id:CAB60326.1 /QuerySize=244</td><td>gi|6903764|ref|Os03g38140|</td></tr>
<tr><td>gi|55244559|gb|EAL41512.1|</td><td>lcl|Y24F12A.4 CE24195 WBGene00012499 status:Predicted SW:Q9U2Q5 protein_id:CAB60327.1 /QuerySize=248</td><td>gi|6903765|ref|Os03g38150|</td></tr>
<tr><td>gi|55244563|gb|EAL41513.1|</td><td>lcl|Y25C1A.6 CE21473 WBGene00021293 status:Confirmed SW:Q9TYM3 protein_id:AAD12837.1 /QuerySize=189</td><td>gi|6903766|ref|Os03g38160|</td></tr>
<tr><td>gi|55244568|gb|EAL41515.1|</td><td>lcl|Y25C1A.8b CE32241 WBGene00021295 status:Confirmed SW:Q8ITV3 protein_id:AAN60503.1 /QuerySize=219</td><td>gi|6903768|ref|Os03g38180|</td></tr>
<tr><td>gi|55244574|gb|EAA45332.2|</td><td>lcl|Y26D4A.10 CE21490 WBGene00012507 status:Predicted SW:Q9U2P8 protein_id:CAB54346.1 /QuerySize=546</td><td>gi|6903769|ref|Os03g38190|</td></tr>
<tr><td>gi|55244582|gb|EAL41518.1|</td><td>lcl|Y26D4A.13 CE36513 WBGene00012510 status:Predicted SW:Q7YWT2 protein_id:CAE17956.2 /QuerySize=315</td><td>gi|6903770|ref|Os03g38200|</td></tr>
<tr><td>gi|55244592|gb|EAL41520.1|</td><td>lcl|Y26D4A.14 CE35068 WBGene00012511 status:Predicted SW:Q7YWT1 protein_id:CAE17957.1 /QuerySize=716</td><td>gi|6903772|ref|Os03g38220|</td></tr>
<tr><td>gi|55244599|gb|EAA05362.2|</td><td>lcl|Y26D4A.5 CE21486 WBGene00012503 status:Partially_confirmed SW:Q9U2Q1 protein_id:CAB54343.1 /QuerySize=117</td><td>gi|6903774|ref|Os03g38240|</td></tr>
<tr><td>gi|55244600|gb|EAA05347.2|</td><td>lcl|Y26D4A.8 CE21488 WBGene00012505 status:Predicted SW:Q9U2P6 protein_id:CAB54348.1 /QuerySize=380</td><td>gi|6903780|ref|Os03g38300|</td></tr>
<tr><td>gi|55244613|gb|EAA05318.2|</td><td>lcl|Y26E6A.1 CE16607 WBGene00012512 status:Partially_confirmed SW:O62418 protein_id:CAA19472.1 /QuerySize=449</td><td>gi|6903784|ref|Os03g38340|</td></tr>
<tr><td>gi|55244619|gb|EAA05030.2|</td><td>lcl|Y26E6A.2 CE16608 WBGene00012513 status:Confirmed SW:O62419 protein_id:CAA19473.1 /QuerySize=413</td><td>gi|6903786|ref|Os03g38360|</td></tr>
<tr><td>gi|55244627|gb|EAL41526.1|</td><td>lcl|Y27F2A.8 CE33729 WBGene00021299 status:Partially_confirmed SW:Q86MI6 protein_id:AAO91687.1 /QuerySize=154</td><td>gi|6903788|ref|Os03g38380|</td></tr>
<tr><td>gi|55244628|gb|EAL41527.1|</td><td>lcl|Y27F2A.9 CE36231 WBGene00021300 status:Partially_confirmed SW:Q6AW17 protein_id:AAT92060.1 /QuerySize=78</td><td>gi|6903791|ref|Os03g38410|</td></tr>
<tr><td>gi|55244630|gb|EAL41528.1|</td><td>lcl|Y2C2A.1 CE25234 WBGene00006453 locus:tag-80 status:Partially_confirmed SW:Q9N541 protein_id:AAF60483.1 /QuerySize=</td><td>gi|6903792|ref|Os03g38420|</td></tr>
<tr><td>gi|55244641|gb|EAL41532.1|</td><td>lcl|Y32B12A.5 CE20202 WBGene00012520 status:Predicted SW:Q9N6E3 protein_id:CAB76747.1 /QuerySize=99</td><td>gi|6903793|ref|Os03g38430|</td></tr>
<tr><td>gi|55244654|gb|EAA05057.3|</td><td>lcl|Y32B12B.1 CE19062 WBGene00012521 status:Predicted SW:Q9XX36 protein_id:CAA21004.1 /QuerySize=248</td><td>gi|6903794|ref|Os03g38440|</td></tr>
</table>

gi|55244655|gb|EAA05176.3|	lcl|Y32B12B.2 CE32518 WBGene00012522 status:Partially_confirmed SW:Q9XX35 protein_id:CAA21005.2 /QuerySize=789	gi|6903796|ref|Os03g38460|
gi|55244658|gb|EAL41536.1|	lcl|Y32B12B.4 CE24197 WBGene00012524 status:Partially_confirmed SW:Q9XX31 protein_id:CAA21009.1 /QuerySize=1545	gi|6903801|ref|Os03g38510|
gi|55244674|gb|EAL41544.1|	lcl|Y32G9A.10 CE26753 WBGene00021307 status:Predicted SW:Q9BPM7 protein_id:AAG53698.1 /QuerySize=214	gi|6903813|ref|Os03g38620|
gi|55244679|gb|EAL41545.1|	lcl|Y32G9A.11 CE32242 WBGene00021308 status:Predicted SW:Q8MXQ6 protein_id:AAM48554.2 /QuerySize=115	gi|6903814|ref|Os03g38630|
gi|55244680|gb|EAL41546.1|	lcl|Y32G9A.2 CE21503 WBGene00021301 status:Predicted SW:Q9BPM5 protein_id:AAG53707.1 /QuerySize=109	gi|6903815|ref|Os03g38640|
gi|55244694|gb|EAL41552.1|	lcl|Y32G9A.3 CE26761 WBGene00021302 status:Predicted SW:Q9BPM8 protein_id:AAG53706.1 /QuerySize=142	gi|6903816|ref|Os03g38650|
gi|55244696|gb|EAA05062.2|	lcl|Y32G9A.5 CE26759 WBGene00021303 status:Predicted SW:Q9BPN1 protein_id:AAG53704.1 /QuerySize=275	gi|6903817|ref|Os03g38660|
gi|55244697|gb|EAA05034.3|	lcl|Y32G9B.1 CE28113 WBGene00021309 status:Partially_confirmed SW:Q965Y2 protein_id:AAK71405.1 /QuerySize=394	gi|6903818|ref|Os03g38670|
gi|55244700|gb|EAL41553.1|	lcl|Y32H12A.9 CE32960 WBGene00021317 status:Predicted SW:Q8IAB1 protein_id:AAN84812.1 /QuerySize=43	gi|6903819|ref|Os03g38680|
gi|55244707|gb|EAA05051.2|	lcl|Y34B4A.10 CE30214 WBGene00021326 status:Partially_confirmed SW:Q8WSP0 protein_id:AAL65800.1 /QuerySize=498	gi|6903820|ref|Os03g38690|
gi|55244711|gb|EAA05162.2|	lcl|Y34B4A.2 CE21522 WBGene00021319 status:Predicted SW:Q9N465 protein_id:AAF59641.1 /QuerySize=344	gi|6903828|ref|Os03g38750|
gi|55244721|gb|EAA05059.2|	lcl|Y34D9A.3a CE37059 WBGene00021329 status:Partially_confirmed SW:Q6AW13 protein_id:AAT81174.1 /QuerySize=574	gi|6903829|ref|Os03g38760|
gi|55244722|gb|EAA05327.2|	lcl|Y34D9A.3b CE37060 WBGene00021329 status:Partially_confirmed SW:Q6AW12 protein_id:AAT81175.1 /QuerySize=227	gi|6903831|ref|Os03g38780|
gi|55244731|gb|EAA05175.2|	lcl|Y34D9A.8 CE21525 WBGene00021333 status:Partially_confirmed SW:Q9N455 protein_id:AAK29879.1 /QuerySize=128	gi|6903834|ref|Os03g38810|
gi|55244741|gb|EAA05090.2|	lcl|Y34F4.1 CE26643 WBGene00021336 status:Partially_confirmed SW:Q9GR66 protein_id:AAG37944.1 /QuerySize=86	gi|6903837|ref|Os03g38840|
gi|55244745|gb|EAA05122.2|	lcl|Y34F4.2 CE26642 WBGene00021337 status:Partially_confirmed SW:Q9GR68 protein_id:AAG37943.1 /QuerySize=198	gi|6903839|ref|Os03g38860|
gi|55244751|gb|EAL41563.1|	lcl|Y34F4.4 CE32961 WBGene00021339 status:Partially_confirmed SW:Q95XC3 protein_id:AAK66023.2 /QuerySize=84	gi|6903841|ref|Os03g38880|
gi|55244753|gb|EAL41564.1|	lcl|Y34F4.5 CE32243 WBGene00021340 status:Partially_confirmed SW:Q95XC2 protein_id:AAK66024.2 /QuerySize=191	gi|6903842|ref|Os03g38890|
gi|55244769|gb|EAA05126.2|	lcl|Y35H6.1 CE26764 WBGene00021341 status:Predicted SW:Q9BKT3 protein_id:AAK31562.1 /QuerySize=161	gi|6903843|ref|Os03g38900|
gi|55244785|gb|EAL41576.1|	lcl|Y35H6.3 CE26762 WBGene00021342 status:Partially_confirmed SW:Q9BKT4 protein_id:AAK31560.1 /QuerySize=239	gi|6903844|ref|Os03g38910|
gi|55244786|gb|EAL41577.1|	lcl|Y37A1B.4 CE19074 WBGene00012537 status:Predicted SW:Q9XXG0 protein_id:CAA19489.1 /QuerySize=95	gi|6903845|ref|Os03g38920|
gi|55244788|gb|EAL41579.1|	lcl|Y37A1B.6 CE19076 WBGene00012539 status:Predicted SW:Q9XXG6 protein_id:CAA19483.1 /QuerySize=144	gi|6903847|ref|Os03g38940|
gi|55244797|gb|EAL41583.1|	lcl|Y37A1B.7 CE19077 WBGene00012540 status:Partially_confirmed SW:Q9XXG7 protein_id:CAA19482.1 /QuerySize=175	gi|6903862|ref|Os03g39030|
gi|55244798|gb|EAA05159.2|	lcl|Y37A1C.1b CE19087 WBGene00012543 status:Partially_confirmed SW:Q9XTC4 protein_id:CAA16318.1 /QuerySize=262	gi|6903863|ref|Os03g39040|
gi|55244799|gb|EAA05118.3|	lcl|Y37D8A.12a CE20216 WBGene00012552 status:Partially_confirmed SW:Q9XWV9 protein_id:CAA21531.1 /QuerySize=458	gi|6903865|ref|Os03g39060|
gi|55244800|gb|EAA05141.2|	lcl|Y37D8A.12b CE28421 WBGene00012552 status:Partially_confirmed SW:Q95ZJ5 protein_id:CAC44300.1 /QuerySize=140	gi|6903866|ref|Os03g39070|
gi|55244806|gb|EAA05104.2|	lcl|Y37D8A.15 CE20219 WBGene00001457 locus:flp-14 status:Partially_confirmed SW:Q9XWV7 protein_id:CAA21533.1 /QuerySi	gi|6903867|ref|Os03g39080|
gi|55244807|gb|EAA05297.2|	lcl|Y37D8A.16 CE20220 WBGene00012554 status:Confirmed SW:Q9XWV6 protein_id:CAA21534.1 /QuerySize=163	gi|6903868|ref|Os03g39090|
gi|55244808|gb|EAA05295.2|	lcl|Y37D8A.19 CE20223 WBGene00012557 status:Confirmed SW:Q9XWU9 protein_id:CAA21542.1 /QuerySize=101	gi|6903870|ref|Os03g39110|
gi|55244809|gb|EAA05120.2|	lcl|Y37D8A.3 CE20208 WBGene00012545 status:Confirmed SW:Q9XWV1 protein_id:CAA21540.1 /QuerySize=306	gi|6903871|ref|Os03g39120|
gi|55244810|gb|EAL41585.1|	lcl|Y37E11AL.2 CE30218 WBGene00021358 status:Partially_confirmed SW:Q9N443 protein_id:AAF59454.2 /QuerySize=215	gi|6903872|ref|Os03g39130|
gi|55244817|gb|EAL41588.1|	lcl|Y37E11AL.3a CE27599 WBGene00021359 status:Confirmed SW:Q9N444 protein_id:AAF59453.2 /QuerySize=305	gi|6903873|ref|Os03g39140|
gi|55244837|gb|EAL41593.1|	lcl|Y37E11AL.3b CE33851 WBGene00021359 status:Partially_confirmed SW:Q86FM4 protein_id:AAP13760.1 /QuerySize=207	gi|6903876|ref|Os03g39170|
gi|55244838|gb|EAL41594.1|	lcl|Y37E11AL.9 CE26061 WBGene00021364 status:Predicted SW:Q9N442 protein_id:AAF59445.1 /QuerySize=133	gi|6903877|ref|Os03g39180|
gi|55244854|gb|EAL41599.1|	lcl|Y37E11AM.2 CE28517 WBGene00021366 status:Partially_confirmed SW:Q9N439 protein_id:AAK73905.1 /QuerySize=182	gi|6903879|ref|Os03g39200|
gi|55244878|gb|EAL41603.1|	lcl|Y37E11AM.3 CE29996 WBGene00021367 status:Confirmed SW:Q8WTM5 protein_id:AAL32236.1 /QuerySize=109	gi|6903881|ref|Os03g39220|
gi|55244879|gb|EAL41604.1|	lcl|Y37E11B.10a CE28114 WBGene00021381 status:Confirmed SW:Q9TYM8 protein_id:AAD12819.2 /QuerySize=511	gi|6903883|ref|Os03g39240|
gi|55244880|gb|EAL41605.1|	lcl|Y37E11B.10b CE33857 WBGene00021381 status:Partially_confirmed SW:Q86FM2 protein_id:AAP13763.1 /QuerySize=525	gi|6903885|ref|Os03g39260|
gi|55244893|gb|EAA05099.2|	lcl|Y37E11B.10c CE33858 WBGene00021381 status:Partially_confirmed SW:Q86FM1 protein_id:AAP13764.1 /QuerySize=173	gi|6903886|ref|Os03g39270|
gi|55244894|gb|EAA05306.2|	lcl|Y37E11B.2 CE36337 WBGene00021375 status:Confirmed SW:Q9TYN6 protein_id:AAD12822.2 /QuerySize=83	gi|6903887|ref|Os03g39280|
gi|55244896|gb|EAL41610.1|	lcl|Y37E11B.3 CE19613 WBGene00021376 status:Confirmed SW:Q9TYN4 protein_id:AAD12823.1 /QuerySize=157	gi|6903889|ref|Os03g39300|
gi|55244898|gb|EAL41611.1|	lcl|Y37E11B.6 CE19616 WBGene00021378 status:Partially_confirmed SW:Q9TYN1 protein_id:AAD12826.1 /QuerySize=136	gi|6903890|ref|Os03g39310|
gi|55244901|gb|EAA05124.3|	lcl|Y37E11B.7 CE19617 WBGene00021379 status:Confirmed SW:Q9TYN0 protein_id:AAD12820.1 /QuerySize=105	gi|6903891|ref|Os03g39320|
gi|55244908|gb|EAL41614.1|	lcl|Y37E11B.9 CE34610 WBGene00021380 status:Partially_confirmed SW:Q9TYM9 protein_id:AAD12827.2 /QuerySize=210	gi|6903893|ref|Os03g39340|
gi|55244909|gb|EAL41615.1|	lcl|Y37F4.1 CE29849 WBGene00021382 status:Partially_confirmed SW:Q95XA0 protein_id:AAL27248.1 /QuerySize=226	gi|6903896|ref|Os03g39370|
gi|55244919|gb|EAL41622.1|	lcl|Y37F4.2 CE29850 WBGene00021383 status:Predicted SW:Q95XA1 protein_id:AAL27249.1 /QuerySize=76	gi|6903898|ref|Os03g39390|
gi|55244922|gb|EAL41623.1|	lcl|Y37F4.4 CE29852 WBGene00021385 status:Predicted SW:Q95XA2 protein_id:AAL27251.1 /QuerySize=96	gi|6903900|ref|Os03g39410|
gi|55244924|gb|EAL41624.1|	lcl|Y37F4.5 CE36032 WBGene00021386 status:Partially_confirmed SW:Q75KS2 protein_id:AAR12980.1 /QuerySize=263	gi|6903901|ref|Os03g39420|
gi|55244928|gb|EAL41625.1|	lcl|Y37F4.6a CE29854 WBGene00021387 status:Partially_confirmed SW:Q95X98 protein_id:AAL27253.1 /QuerySize=433	gi|6903902|ref|Os03g39430|
gi|55244933|gb|EAL41627.1|	lcl|Y37F4.6b CE33214 WBGene00021387 status:Partially_confirmed SW:Q86S59 protein_id:AAO21408.1 /QuerySize=262	gi|6903908|ref|Os03g39490|
gi|55244934|gb|EAL41628.1|	lcl|Y37F4.7 CE36033 WBGene00021388 status:Confirmed SW:Q75KS1 protein_id:AAR12981.1 /QuerySize=93	gi|6903910|ref|Os03g39510|
gi|55244935|gb|EAL41629.1|	lcl|Y37H2A.11 CE25241 WBGene00012571 status:Predicted SW:Q9U2N2 protein_id:CAB60578.1 /QuerySize=417	gi|6903912|ref|Os03g39530|
gi|55244936|gb|EAL41630.1|	lcl|Y37H2A.9 CE24215 WBGene00012569 status:Predicted SW:Q9U2N9 protein_id:CAB60571.1 /QuerySize=275	gi|6903914|ref|Os03g39550|
gi|55244937|gb|EAL41631.1|	lcl|Y37H2B.1 CE19088 WBGene00012572 status:Predicted SW:Q9XXF5 protein_id:CAA19498.1 /QuerySize=161	gi|6903921|ref|Os03g39620|
gi|55244938|gb|EAL41632.1|	lcl|Y37H9A.1a CE20227 WBGene00012576 status:Partially_confirmed SW:Q9U2M9 protein_id:CAB63349.1 /QuerySize=514	gi|6903922|ref|Os03g39630|
gi|55244947|gb|EAL41635.1|	lcl|Y37H9A.1b CE37562 WBGene00012576 status:Partially_confirmed SW:Q5ZEQ4 protein_id:CAH60795.1 /QuerySize=498	gi|6903925|ref|Os03g39660|
gi|55244948|gb|EAL41636.1|	lcl|Y37H9A.2 CE20228 WBGene00012577 status:Predicted SW:Q9XWW6 protein_id:CAA21523.1 /QuerySize=326	gi|6903929|ref|Os03g39700|
gi|55244950|gb|EAL41637.1|	lcl|Y38C1AA.12 CE33859 WBGene00021402 status:Partially_confirmed SW:Q86FM6 protein_id:AAP13757.1 /QuerySize=279	gi|6903932|ref|Os03g39730|
gi|55244952|gb|EAA05021.2|	lcl|Y38C1AA.1b CE31642 WBGene00021394 locus:prdx-6 status:Confirmed SW:Q8MXT2 protein_id:AAM97970.1 /QuerySize=98	gi|6903935|ref|Os03g39750|
gi|55244953|gb|EAL41638.1|	lcl|Y38C1AA.4 CE31644 WBGene00006561 locus:tcl-2 status:Confirmed SW:Q9N428 protein_id:AAF59468.3 /QuerySize=435	gi|6903937|ref|Os03g39770|
gi|55244958|gb|EAL41642.1|	lcl|Y38C1AA.7 CE25245 WBGene00021398 status:Confirmed SW:Q9N431 protein_id:AAF59465.1 /QuerySize=159	gi|6903941|ref|Os03g39810|
gi|55244960|gb|EAL41643.1|	lcl|Y38C1AA.8 CE36617 WBGene00021399 status:Predicted SW:Q9N433 protein_id:AAF59464.2 /QuerySize=82	gi|6903946|ref|Os03g39860|
gi|55244963|gb|EAL41646.1|	lcl|Y38C1AB.2 CE25253 WBGene00021404 status:Predicted SW:Q9N2K9 protein_id:AAF60498.1 /QuerySize=778	gi|6903947|ref|Os03g39870|
gi|55244973|gb|EAA04991.2|	lcl|Y38C1AB.3 CE18182 WBGene00021405 status:Predicted SW:Q9N2L3 protein_id:AAF60496.1 /QuerySize=239	gi|6903948|ref|Os03g39880|
gi|55244988|gb|EAL41659.1|	lcl|Y38C1AB.6 CE25253 WBGene00021408 status:Predicted SW:Q9N2K9 protein_id:AAF60501.1 /QuerySize=778	gi|6903955|ref|Os03g39950|
gi|55244990|gb|EAA04873.2|	lcl|Y38C1AB.7 CE18182 WBGene00021409 status:Predicted SW:Q9N2L3 protein_id:AAF60495.1 /QuerySize=239	gi|6903956|ref|Os03g39960|
gi|55244996|gb|EAL41661.1|	lcl|Y38C1BA.1 CE21573 WBGene00021410 status:Partially_confirmed SW:Q9N529 protein_id:AAF60503.1 /QuerySize=255	gi|6903957|ref|Os03g39970|
gi|55245003|gb|EAL41665.1|	lcl|Y38E10A.1 CE21581 WBGene00012579 status:Predicted SW:Q9NAK9 protein_id:CAB54392.1 /QuerySize=96	gi|6903958|ref|Os03g39980|
gi|55245028|gb|EAL41671.1|	lcl|Y38E10A.14 CE21594 WBGene00012592 status:Confirmed SW:Q9NAJ7 protein_id:CAB54405.1 /QuerySize=127	gi|6903959|ref|Os03g39990|
gi|55245039|gb|EAL41673.1|	lcl|Y38E10A.17 CE21597 WBGene00012595 status:Partially_confirmed SW:Q9NAJ4 protein_id:CAB54408.1 /QuerySize=586	gi|6903960|ref|Os03g40000|
gi|55245048|gb|EAA45374.2|	lcl|Y38E10A.2 CE21582 WBGene00012580 status:Predicted SW:Q9NAK8 protein_id:CAB54393.1 /QuerySize=267	gi|6903963|ref|Os03g40030|
gi|55245070|gb|EAL41681.1|	lcl|Y38E10A.20 CE21601 WBGene00012598 status:Predicted SW:Q9NAJ1 protein_id:CAB54412.1 /QuerySize=195	gi|6903964|ref|Os03g40040|
gi|55245072|gb|EAA04853.2|	lcl|Y38E10A.23 CE21604 WBGene00012601 status:Predicted SW:Q9NAI8 protein_id:CAB54415.1 /QuerySize=153	gi|6903965|ref|Os03g40050|
gi|55245074|gb|EAA04892.2|	lcl|Y38E10A.24 CE21605 WBGene00012602 status:Confirmed SW:Q9NAI7 protein_id:CAB54416.1 /QuerySize=68	gi|6903966|ref|Os03g40060|
gi|55245075|gb|EAL41684.1|	lcl|Y38E10A.28 CE37940 WBGene00044034 status:Predicted /QuerySize=397	gi|6903969|ref|Os03g40090|
gi|55245077|gb|EAL41685.1|	lcl|Y38E10A.3 CE21583 WBGene00012581 status:Partially_confirmed SW:Q9NAK7 protein_id:CAB54394.1 /QuerySize=243	gi|6903972|ref|Os03g40120|
gi|55245078|gb|EAL41686.1|	lcl|Y38F1A.1 CE32962 WBGene00012605 status:Partially_confirmed SW:Q9XWM5 protein_id:CAA21628.2 /QuerySize=269	gi|6903974|ref|Os03g40140|
gi|55245079|gb|EAL41687.1|	lcl|Y38F1A.7 CE21612 WBGene00012609 status:Predicted SW:Q9XWM3 protein_id:CAA21632.1 /QuerySize=149	gi|6903981|ref|Os03g40210|
gi|55245085|gb|EAA04880.2|	lcl|Y38F1A.8 CE21613 WBGene00012610 status:Confirmed SW:Q9XWM2 protein_id:CAA21633.1 /QuerySize=303	gi|6903983|ref|Os03g40230|
gi|55245088|gb|EAL41688.1|	lcl|Y38F2AL.6 CE21618 WBGene00021418 status:Partially_confirmed SW:Q8WTM4 protein_id:AAL32237.1 /QuerySize=83	gi|6903984|ref|Os03g40240|
gi|55245110|gb|EAA04828.2|	lcl|Y38F2AR.13 CE33864 WBGene00021431 status:Partially_confirmed SW:Q86FM8 protein_id:AAP13754.1 /QuerySize=301	gi|6903988|ref|Os03g40280|
gi|55245115|gb|EAA04878.3|	lcl|Y38F2AR.6 CE27615 WBGene00021424 status:Predicted SW:Q95XS6 protein_id:AAK68447.1 /QuerySize=282	gi|6903990|ref|Os03g40300|
gi|55245116|gb|EAL41695.1|	lcl|Y38H6A.1 CE19093 WBGene00012612 status:Predicted SW:Q9XWT9 protein_id:CAA21555.1 /QuerySize=218	gi|6903994|ref|Os03g40340|
gi|55245124|gb|EAL41697.1|	lcl|Y38H6A.2 CE19094 WBGene00012613 status:Predicted SW:Q9XWU0 protein_id:CAA21554.1 /QuerySize=208	gi|6903995|ref|Os03g40350|
gi|55245126|gb|EAL41698.1|	lcl|Y38H6A.3 CE20231 WBGene00012614 status:Partially_confirmed SW:Q9XWU1 protein_id:CAA21553.1 /QuerySize=247	gi|6903997|ref|Os03g40370|
gi|55245127|gb|EAL41699.1|	lcl|Y38H6C.1 CE19095 WBGene00012615 status:Confirmed SW:Q9XX57 protein_id:CAA20981.1 /QuerySize=90	gi|6903998|ref|Os03g40380|
gi|55245135|gb|EAL41701.1|	lcl|Y38H6C.10 CE19104 WBGene00012623 status:Partially_confirmed SW:Q9XX51 protein_id:CAA20988.1 /QuerySize=345	gi|6903999|ref|Os03g40390|
gi|55245141|gb|EAL41702.1|	lcl|Y38H6C.14 CE19108 WBGene00012626 status:Partially_confirmed SW:Q9XX47 protein_id:CAA20992.1 /QuerySize=814	gi|6904001|ref|Os03g40410|
gi|55245153|gb|EAA04794.3|	lcl|Y38H6C.15 CE19109 WBGene00012627 status:Partially_confirmed SW:Q9XX46 protein_id:CAA20993.1 /QuerySize=185	gi|6904003|ref|Os03g40430|
gi|55245154|gb|EAL41704.1|	lcl|Y38H6C.16 CE19110 WBGene00012628 status:Partially_confirmed SW:Q9XX45 protein_id:CAA20994.1 /QuerySize=244	gi|6904004|ref|Os03g40440|
gi|55245155|gb|EAL41705.1|	lcl|Y38H6C.18 CE19112 WBGene00012630 status:Predicted SW:Q9XX43 protein_id:CAA20996.1 /QuerySize=158	gi|6904007|ref|Os03g40470|
gi|55245172|gb|EAA04028.2|	lcl|Y38H6C.21 CE19115 WBGene00012633 status:Predicted SW:Q9XX37 protein_id:CAA21002.1 /QuerySize=278	gi|6904009|ref|Os03g40490|
gi|55245175|gb|EAL41712.1|	lcl|Y38H6C.22 CE19116 WBGene00000015 locus:abf-4 status:Predicted SW:Q9XX40 protein_id:CAA20999.1 /QuerySize=123	gi|6904012|ref|Os03g40520|
gi|55245191|gb|EAA04230.3|	lcl|Y38H6C.3 CE19097 WBGene00012616 status:Partially_confirmed SW:Q9XX55 protein_id:CAA20983.1 /QuerySize=140	gi|6904013|ref|Os03g40530|
gi|55245193|gb|EAA04054.2|	lcl|Y38H6C.4 CE19098 WBGene00012617 status:Partially_confirmed SW:Q9XX54 protein_id:CAA20984.1 /QuerySize=65	gi|6904019|ref|Os03g40590|
gi|55245217|gb|EAL41722.1|	lcl|Y38H6C.7 CE19101 WBGene00012620 status:Partially_confirmed SW:Q9XX52 protein_id:CAA20987.1 /QuerySize=97	gi|6904022|ref|Os03g40620|
gi|55245228|gb|EAA04052.3|	lcl|Y38H8A.1 CE19118 WBGene00012635 status:Partially_confirmed SW:Q9XXR8 protein_id:CAA16348.1 /QuerySize=272	gi|6904023|ref|Os03g40630|
gi|55245234|gb|EAA04548.2|	lcl|Y38H8A.7 CE35657 WBGene00012640 status:Partially_confirmed SW:Q7K7Q1 protein_id:CAE46681.1 /QuerySize=139	gi|6904024|ref|Os03g40640|
gi|55245242|gb|EAL41727.1|	lcl|Y39A1A.10 CE19129 WBGene00012649 status:Predicted SW:Q9XX23 protein_id:CAA21017.1 /QuerySize=118	gi|6904026|ref|Os03g40660|
gi|55245251|gb|EAL41729.1|	lcl|Y39A1A.18 CE19138 WBGene00012656 status:Partially_confirmed SW:Q9XX29 protein_id:CAA21011.1 /QuerySize=139	gi|6904028|ref|Os03g40680|
gi|55245252|gb|EAL41730.1|	lcl|Y39A1A.2 CE37800 WBGene00012642 status:Predicted SW:Q9XX22 protein_id:CAA21018.2 /QuerySize=176	gi|6904036|ref|Os03g40760|
gi|55245264|gb|EAA04216.2|	lcl|Y39A1A.20 CE34114 WBGene00012657 status:Partially_confirmed SW:Q9XX30 protein_id:CAA21010.2 /QuerySize=113	gi|6904039|ref|Os03g40790|
gi|55245266|gb|EAA04056.2|	lcl|Y39A1A.24 CE19124 WBGene00012660 status:Confirmed SW:Q9XX20 protein_id:CAA21020.1 /QuerySize=126	gi|6904040|ref|Os03g40800|
gi|55245267|gb|EAL41739.1|	lcl|Y39A1A.3 CE19121 WBGene00012643 status:Confirmed SW:Q9XTC1 protein_id:CAA21495.1 /QuerySize=179	gi|6904041|ref|Os03g40810|
gi|55245271|gb|EAA04068.2|	lcl|Y39A1A.7 CE19126 WBGene00012646 status:Partially_confirmed SW:Q9XX25 protein_id:CAA21015.1 /QuerySize=187	gi|6904042|ref|Os03g40820|
gi|55245286|gb|EAL41743.1|	lcl|Y39A1C.1 CE19146 WBGene00012662 status:Partially_confirmed SW:Q9XXE7 protein_id:CAA19507.1 /QuerySize=117	gi|6904045|ref|Os03g40850|
gi|55245297|gb|EAA04492.2|	lcl|Y39A3B.3 CE32522 WBGene00021438 status:Predicted SW:Q9N518 protein_id:AAF60515.2 /QuerySize=215	gi|6904046|ref|Os03g40860|
gi|55245303|gb|EAA03937.2|	lcl|Y39A3CL.1 CE32963 WBGene00021440 status:Partially_confirmed SW:Q9N412 protein_id:AAK93862.2 /QuerySize=598	gi|6904048|ref|Os03g40880|
gi|55245310|gb|EAA04490.2|	lcl|Y39A3CL.3 CE21651 WBGene00021441 status:Partially_confirmed SW:Q9N416 protein_id:AAF60518.1 /QuerySize=50	gi|6904049|ref|Os03g40890|
gi|55245316|gb|EAA04489.3|	lcl|Y39A3CL.7a CE28979 WBGene00021443 status:Partially_confirmed SW:Q965X1 protein_id:AAK93863.1 /QuerySize=480	gi|6904050|ref|Os03g40900|
gi|55245323|gb|EAA04491.2|	lcl|Y39A3CL.7b CE37426 WBGene00021443 status:Partially_confirmed SW:Q65XX5 protein_id:AAU20836.1 /QuerySize=404	gi|6904054|ref|Os03g40940|
gi|55245334|gb|EAL41752.1|	lcl|Y39A3CR.1b CE33943 WBGene00004886 locus:smi-1 status:Partially_confirmed SW:Q86DC9 protein_id:AAP31427.1 /QueryS	gi|6904055|ref|Os03g40950|
gi|55245350|gb|EAL41757.1|	lcl|Y39A3CR.5 CE33944 WBGene00021445 status:Partially_confirmed SW:Q9N409 protein_id:AAF60524.2 /QuerySize=66	gi|6904059|ref|Os03g40990|
gi|55245352|gb|EAL41758.1|	lcl|Y39A3CR.7 CE29106 WBGene00004163 locus:pqn-82 status:Partially_confirmed SW:Q9N411 protein_id:AAF60529.2 /QueryS	gi|6904061|ref|Os03g41010|
gi|55245359|gb|EAA04378.2|	lcl|Y39A3CR.8 CE25262 WBGene00021447 status:Predicted SW:Q9N404 protein_id:AAF60530.1 /QuerySize=165	gi|6904063|ref|Os03g41030|
gi|55245360|gb|EAL41760.1|	lcl|Y39B6A.1 CE21682 WBGene00012664 status:Confirmed SW:Q9NES7 protein_id:CAC51077.1 /QuerySize=735	gi|6904064|ref|Os03g41040|
gi|55245368|gb|EAA04475.3|	lcl|Y39B6A.10 CE21698 WBGene00012672 status:Confirmed SW:Q9NET7 protein_id:CAC51067.1 /QuerySize=256	gi|6904065|ref|Os03g41050|
gi|55245375|gb|EAA04596.3|	lcl|Y39B6A.11 CE21697 WBGene00012673 status:Partially_confirmed SW:Q9NET8 protein_id:CAC51066.1 /QuerySize=366	gi|6904069|ref|Os03g41090|
gi|55245389|gb|EAL41767.1|	lcl|Y39B6A.13 CE21694 WBGene00012675 status:Partially_confirmed SW:Q9NEU1 protein_id:CAC51063.1 /QuerySize=208	gi|6904073|ref|Os03g41130|
gi|55245390|gb|EAL41768.1|	lcl|Y39B6A.16 CE29858 WBGene00012678 status:Partially_confirmed SW:Q8MYP1 protein_id:CAD31814.1 /QuerySize=283	gi|6904074|ref|Os03g41140|
gi|55245400|gb|EAL41771.1|	lcl|Y39B6A.26 CE29867 WBGene00012685 status:Confirmed SW:Q8MYN3 protein_id:CAD31823.1 /QuerySize=73	gi|6904075|ref|Os03g41150|
gi|55245416|gb|EAL41774.1|	lcl|Y39B6A.28 CE29869 WBGene00012687 status:Predicted SW:Q8MYN2 protein_id:CAD31824.1 /QuerySize=265	gi|6904082|ref|Os03g41220|
gi|55245428|gb|EAL41777.1|	lcl|Y39B6A.31 CE21689 WBGene00012690 status:Partially_confirmed SW:Q9NEU6 protein_id:CAC51049.1 /QuerySize=429	gi|6904083|ref|Os03g41230|
gi|55245429|gb|EAL41778.1|	lcl|Y39B6A.32 CE29871 WBGene00012691 status:Predicted SW:Q8MYN0 protein_id:CAD31826.1 /QuerySize=135	gi|6904085|ref|Os03g41250|
gi|55245439|gb|EAL41782.1|	lcl|Y39B6A.37 CE29874 WBGene00012695 status:Partially_confirmed SW:Q8MYM7 protein_id:CAD31829.1 /QuerySize=314	gi|6904086|ref|Os03g41260|
gi|55245440|gb|EAL41783.1|	lcl|Y39B6A.4 CE21674 WBGene00012667 status:Predicted SW:Q9NET1 protein_id:CAC51073.1 /QuerySize=161	gi|6904087|ref|Os03g41270|
gi|55245463|gb|EAL41790.1|	lcl|Y39B6A.42 CE29877 WBGene00012700 status:Confirmed SW:Q8MYM4 protein_id:CAD31832.1 /QuerySize=105	gi|6904088|ref|Os03g41280|
gi|55245472|gb|EAL41794.1|	lcl|Y39B6A.43a CE32013 WBGene00012701 status:Partially_confirmed SW:Q8MYM3 protein_id:CAD31833.2 /QuerySize=750	gi|6904091|ref|Os03g41310|
gi|55245474|gb|EAA04544.3|	lcl|Y39B6A.43b CE32014 WBGene00012701 status:Partially_confirmed SW:Q8I0Z8 protein_id:CAD57708.1 /QuerySize=753	gi|6904092|ref|Os03g41320|
gi|55245476|gb|EAL41796.1|	lcl|Y39B6A.46 CE29881 WBGene00012702 status:Partially_confirmed SW:Q8MYM0 protein_id:CAD31836.1 /QuerySize=248	gi|6904094|ref|Os03g41340|
gi|55245489|gb|EAL41798.1|	lcl|Y39B6A.5 CE21672 WBGene00012668 status:Confirmed SW:Q9NET2 protein_id:CAC51072.1 /QuerySize=82	gi|6904099|ref|Os03g41380|
gi|55245520|gb|EAL41802.1|	lcl|Y39C12A.9 CE25265 WBGene00012710 status:Partially_confirmed SW:Q9U2L1 protein_id:CAB60495.1 /QuerySize=144	gi|6904101|ref|Os03g41400|
gi|55245523|gb|EAL41803.1|	lcl|Y39E4A.1 CE16615 WBGene00012711 status:Partially_confirmed SW:O45921 protein_id:CAA16326.1 /QuerySize=133	gi|6904105|ref|Os03g41440|
gi|55245532|gb|EAL41808.1|	lcl|Y39E4B.10 CE21721 WBGene00012719 status:Partially_confirmed SW:Q9U2K2 protein_id:CAB54431.1 /QuerySize=581	gi|6904109|ref|Os03g41480|

<table>
<tr><td>gi|55245534|gb|EAL41809.1|</td><td>lcl|Y39E4B.11 CE21722 WBGene00012720 status:Partially_confirmed SW:Q9U2K9 protein_id:CAB54423.1 /QuerySize=245</td><td>gi|6904110|ref|Os03g41490|</td></tr>
<tr><td>gi|55245541|gb|EAA04386.2|</td><td>lcl|Y39E4B.13 CE21723 WBGene00012721 status:Partially_confirmed SW:Q9U2L0 protein_id:CAB54422.1 /QuerySize=148</td><td>gi|6904111|ref|Os03g41500|</td></tr>
<tr><td>gi|55245548|gb|EAL41810.1|</td><td>lcl|Y39E4B.2 CE21714 WBGene00012715 status:Partially_confirmed SW:Q9U2K7 protein_id:CAB54425.1 /QuerySize=445</td><td>gi|6904115|ref|Os03g41520|</td></tr>
<tr><td>gi|55245549|gb|EAL41811.1|</td><td>lcl|Y39E4B.6 CE21718 WBGene00012717 status:Confirmed SW:Q9U2K4 protein_id:CAB54428.1 /QuerySize=237</td><td>gi|6904119|ref|Os03g41540|</td></tr>
<tr><td>gi|55245558|gb|EAL41813.1|</td><td>lcl|Y39G10AL.1 CE26987 WBGene00021458 status:Confirmed SW:Q9N402 protein_id:AAF59490.2 /QuerySize=160</td><td>gi|6904120|ref|Os03g41550|</td></tr>
<tr><td>gi|55245562|gb|EAL41816.1|</td><td>lcl|Y39G10AR.10 CE26989 WBGene00021468 status:Confirmed SW:Q95XR4 protein_id:AAK39618.1 /QuerySize=690</td><td>gi|6904121|ref|Os03g41560|</td></tr>
<tr><td>gi|55245563|gb|EAL41817.1|</td><td>lcl|Y39G10AR.11 CE26525 WBGene00021469 status:Partially_confirmed SW:Q95XR6 protein_id:AAK39606.1 /QuerySize=788</td><td>gi|6904124|ref|Os03g41590|</td></tr>
<tr><td>gi|55245564|gb|EAA04488.2|</td><td>lcl|Y39G10AR.12a CE26990 WBGene00021470 status:Partially_confirmed SW:Q95XR5 protein_id:AAK39617.1 /QuerySize=507</td><td>gi|6904130|ref|Os03g41650|</td></tr>
<tr><td>gi|55245567|gb|EAA04323.2|</td><td>lcl|Y39G10AR.12b CE29999 WBGene00021470 status:Partially_confirmed SW:Q8WSN6 protein_id:AAL32238.1 /QuerySize=482</td><td>gi|6904131|ref|Os03g41660|</td></tr>
<tr><td>gi|55245568|gb|EAL41818.1|</td><td>lcl|Y39G10AR.15 CE26523 WBGene00021471 status:Partially_confirmed SW:Q95XQ7 protein_id:AAK39604.1 /QuerySize=704</td><td>gi|6904132|ref|Os03g41670|</td></tr>
<tr><td>gi|55245569|gb|EAA04388.2|</td><td>lcl|Y39G10AR.16 CE21755 WBGene00021472 status:Partially_confirmed SW:Q95XQ6 protein_id:AAK39603.1 /QuerySize=227</td><td>gi|6904134|ref|Os03g41690|</td></tr>
<tr><td>gi|55245570|gb|EAA04329.2|</td><td>lcl|Y39G10AR.17 CE36733 WBGene00021473 status:Partially_confirmed SW:Q95XQ1 protein_id:AAK39602.2 /QuerySize=549</td><td>gi|6904137|ref|Os03g41720|</td></tr>
<tr><td>gi|55245571|gb|EAA04393.2|</td><td>lcl|Y39G10AR.18b CE36735 WBGene00021474 status:Partially_confirmed SW:Q6AW05 protein_id:AAT39978.1 /QuerySize=586</td><td>gi|6904139|ref|Os03g41740|</td></tr>
<tr><td>gi|55245573|gb|EAA04324.2|</td><td>lcl|Y39G10AR.2 CE26992 WBGene00021460 locus:zwl-1 status:Partially_confirmed SW:Q95XP9 protein_id:AAK39613.1 /QuerySi</td><td>gi|6904140|ref|Os03g41750|</td></tr>
<tr><td>gi|55245574|gb|EAA45421.2|</td><td>lcl|Y39G10AR.7 CE21764 WBGene00021465 status:Partially_confirmed SW:Q95XQ9 protein_id:AAK39607.1 /QuerySize=474</td><td>gi|6904141|ref|Os03g41760|</td></tr>
<tr><td>gi|55245584|gb|EAL41821.1|</td><td>lcl|Y39H10A.1 CE26072 WBGene00021478 status:Partially_confirmed SW:Q9N3Z1 protein_id:AAF59487.2 /QuerySize=131</td><td>gi|6904146|ref|Os03g41810|</td></tr>
<tr><td>gi|55245585|gb|EAA04405.3|</td><td>lcl|Y39H10A.4 CE21781 WBGene00021480 status:Partially_confirmed SW:Q9N3Z9 protein_id:AAF59482.1 /QuerySize=261</td><td>gi|6904147|ref|Os03g41820|</td></tr>
<tr><td>gi|55245587|gb|EAL41822.1|</td><td>lcl|Y39H10A.5 CE26074 WBGene00021481 status:Predicted SW:Q9N3Z7 protein_id:AAF59481.2 /QuerySize=120</td><td>gi|6904150|ref|Os03g41850|</td></tr>
<tr><td>gi|55245599|gb|EAA45425.2|</td><td>lcl|Y40A1A.2 CE35798 WBGene00021484 status:Partially_confirmed SW:Q8IU11 protein_id:AAN73855.2 /QuerySize=71</td><td>gi|6904151|ref|Os03g41860|</td></tr>
<tr><td>gi|55245609|gb|EAL41828.1|</td><td>lcl|Y40A1A.3 CE35799 WBGene00021485 status:Confirmed SW:Q86N74 protein_id:AAQ91894.1 /QuerySize=88</td><td>gi|6904152|ref|Os03g41870|</td></tr>
<tr><td>gi|55245610|gb|EAL41829.1|</td><td>lcl|Y40B10A.3 CE26079 WBGene00021488 locus:srab-23 status:Predicted SW:Q965W7 protein_id:AAK70658.1 /QuerySize=243</td><td>gi|6904153|ref|Os03g41880|</td></tr>
<tr><td>gi|55245611|gb|EAL41830.1|</td><td>lcl|Y40B10A.5 CE26081 WBGene00021490 status:Predicted SW:Q965W5 protein_id:AAK70660.1 /QuerySize=148</td><td>gi|6904154|ref|Os03g41890|</td></tr>
<tr><td>gi|55245612|gb|EAL41831.1|</td><td>lcl|Y40B10A.9 CE26083 WBGene00021493 status:Confirmed SW:Q965W0 protein_id:AAK70664.1 /QuerySize=433</td><td>gi|6904155|ref|Os03g41900|</td></tr>
<tr><td>gi|55245613|gb|EAL41832.1|</td><td>lcl|Y40B1A.1 CE21786 WBGene00012732 status:Predicted SW:Q9XW29 protein_id:CAA22304.1 /QuerySize=75</td><td>gi|6904156|ref|Os03g41910|</td></tr>
<tr><td>gi|55245615|gb|EAL41834.1|</td><td>lcl|Y40B1A.3 CE34508 WBGene00012734 status:Partially_confirmed SW:Q9XW27 protein_id:CAA22306.2 /QuerySize=233</td><td>gi|6904157|ref|Os03g41920|</td></tr>
<tr><td>gi|55245616|gb|EAL41835.1|</td><td>lcl|Y40B1A.5 CE35082 WBGene00012736 status:Predicted SW:Q7YWR5 protein_id:CAE17989.1 /QuerySize=134</td><td>gi|6904158|ref|Os03g41930|</td></tr>
<tr><td>gi|55245619|gb|EAL41837.1|</td><td>lcl|Y40B1B.5 CE20239 WBGene00012738 status:Partially_confirmed SW:Q9XWP7 protein_id:CAA21603.1 /QuerySize=212</td><td>gi|6904159|ref|Os03g41940|</td></tr>
<tr><td>gi|55245624|gb|EAL41839.1|</td><td>lcl|Y40B1B.7 CE21790 WBGene00012739 status:Confirmed SW:Q9XWP4 protein_id:CAA21606.1 /QuerySize=123</td><td>gi|6904160|ref|Os03g41950|</td></tr>
<tr><td>gi|55245632|gb|EAA04304.3|</td><td>lcl|Y40C5A.3 CE24257 WBGene00021496 status:Partially_confirmed SW:Q9N3Y8 protein_id:AAF60538.1 /QuerySize=2344</td><td>gi|6904162|ref|Os03g41970|</td></tr>
<tr><td>gi|55245637|gb|EAL41841.1|</td><td>lcl|Y40C7B.1 CE24260 WBGene00021498 status:Predicted SW:Q9N506 protein_id:AAF60541.1 /QuerySize=923</td><td>gi|6904169|ref|Os03g42030|</td></tr>
<tr><td>gi|55245641|gb|EAL41842.1|</td><td>lcl|Y40C7B.3 CE24262 WBGene00021499 status:Predicted SW:Q9N507 protein_id:AAF60543.1 /QuerySize=416</td><td>gi|6904172|ref|Os03g42060|</td></tr>
<tr><td>gi|55245648|gb|EAL41844.1|</td><td>lcl|Y40C7B.4 CE24263 WBGene00021500 status:Partially_confirmed SW:Q9N509 protein_id:AAF60544.1 /QuerySize=163</td><td>gi|6904174|ref|Os03g42080|</td></tr>
<tr><td>gi|55245666|gb|EAL41850.1|</td><td>lcl|Y40C7B.5 CE24264 WBGene00021501 status:Predicted SW:Q9N505 protein_id:AAF60545.1 /QuerySize=610</td><td>gi|6904175|ref|Os03g42090|</td></tr>
<tr><td>gi|55245667|gb|EAL41851.1|</td><td>lcl|Y40H7A.7 CE21818 WBGene00012745 status:Predicted SW:Q9XW99 protein_id:CAA22067.1 /QuerySize=218</td><td>gi|6904179|ref|Os03g42120|</td></tr>
<tr><td>gi|55245668|gb|EAA04510.3|</td><td>lcl|Y41C4A.1 CE31833 WBGene00012749 status:Partially_confirmed SW:Q9U2I9 protein_id:CAB63352.2 /QuerySize=444</td><td>gi|6904181|ref|Os03g42140|</td></tr>
<tr><td>gi|55245669|gb|EAL41852.1|</td><td>lcl|Y41C4A.12a CE20252 WBGene00012758 status:Confirmed SW:Q9U2I3 protein_id:CAB63358.1 /QuerySize=278</td><td>gi|6904184|ref|Os03g42170|</td></tr>
<tr><td>gi|55245670|gb|EAL41853.1|</td><td>lcl|Y41C4A.12b CE32523 WBGene00012758 status:Partially_confirmed SW:Q8I4D6 protein_id:CAD56606.1 /QuerySize=301</td><td>gi|6904185|ref|Os03g42180|</td></tr>
<tr><td>gi|55245671|gb|EAA04513.2|</td><td>lcl|Y41C4A.13 CE20253 WBGene00012759 status:Confirmed SW:Q9XWU2 protein_id:CAA21550.1 /QuerySize=103</td><td>gi|6904186|ref|Os03g42190|</td></tr>
<tr><td>gi|55245676|gb|EAL41856.1|</td><td>lcl|Y41C4A.17 CE36455 WBGene00012760 status:Partially_confirmed SW:Q8I4D5 protein_id:CAD56607.2 /QuerySize=89</td><td>gi|6904188|ref|Os03g42210|</td></tr>
<tr><td>gi|55245684|gb|EAL41858.1|</td><td>lcl|Y41C4A.18 CE32525 WBGene00012761 status:Confirmed SW:Q8I4D4 protein_id:CAD56608.1 /QuerySize=100</td><td>gi|6904194|ref|Os03g42260|</td></tr>
<tr><td>gi|55245694|gb|EAL41861.1|</td><td>lcl|Y41C4A.3 CE20243 WBGene00012751 status:Predicted SW:Q9XWU6 protein_id:CAA21546.1 /QuerySize=162</td><td>gi|6904198|ref|Os03g42300|</td></tr>
<tr><td>gi|55245702|gb|EAL41863.1|</td><td>lcl|Y41C4A.6 CE31836 WBGene00012753 status:Partially_confirmed SW:Q9U2I6 protein_id:CAB63355.2 /QuerySize=118</td><td>gi|6904199|ref|Os03g42310|</td></tr>
<tr><td>gi|55245703|gb|EAA04511.2|</td><td>lcl|Y41D4A.1 CE27618 WBGene00021504 status:Partially_confirmed SW:Q95Y21 protein_id:AAK68451.1 /QuerySize=251</td><td>gi|6904201|ref|Os03g42330|</td></tr>
<tr><td>gi|55245720|gb|EAL41868.1|</td><td>lcl|Y41D4A.3 CE31646 WBGene00021505 status:Partially_confirmed SW:Q8WTM1 protein_id:AAK68453.4 /QuerySize=88</td><td>gi|6904204|ref|Os03g42360|</td></tr>
<tr><td>gi|55245726|gb|EAL41869.1|</td><td>lcl|Y41D4A.7 CE21837 WBGene00021509 status:Predicted SW:Q95Y23 protein_id:AAK68457.3 /QuerySize=259</td><td>gi|6904207|ref|Os03g42390|</td></tr>
<tr><td>gi|55245737|gb|EAL41871.1|</td><td>lcl|Y41D4B.1 CE26087 WBGene00021511 status:Predicted SW:Q95Y02 protein_id:AAK68459.1 /QuerySize=235</td><td>gi|6904208|ref|Os03g42400|</td></tr>
<tr><td>gi|55245748|gb|EAL41874.1|</td><td>lcl|Y41D4B.11 CE26095 WBGene00021514 status:Confirmed SW:Q95Y11 protein_id:AAK68461.1 /QuerySize=349</td><td>gi|6904212|ref|Os03g42440|</td></tr>
<tr><td>gi|55245753|gb|EAA04135.2|</td><td>lcl|Y41D4B.14 CE29109 WBGene00021516 status:Confirmed SW:Q95Y14 protein_id:AAK68464.2 /QuerySize=96</td><td>gi|6904213|ref|Os03g42450|</td></tr>
<tr><td>gi|55245755|gb|EAA04145.3|</td><td>lcl|Y41D4B.22 CE21856 WBGene00021523 status:Partially_confirmed SW:Q95Y01 protein_id:AAK68474.1 /QuerySize=89</td><td>gi|6904216|ref|Os03g42480|</td></tr>
<tr><td>gi|55245764|gb|EAL41878.1|</td><td>lcl|Y41D4B.26 CE36740 WBGene00021525 status:Partially_confirmed SW:Q8WSN8 protein_id:AAL32241.2 /QuerySize=201</td><td>gi|6904217|ref|Os03g42490|</td></tr>
<tr><td>gi|55245775|gb|EAA04016.2|</td><td>lcl|Y41D4B.6 CE27628 WBGene00021513 status:Partially_confirmed SW:Q95Y06 protein_id:AAK68480.1 /QuerySize=372</td><td>gi|6904218|ref|Os03g42500|</td></tr>
<tr><td>gi|55245780|gb|EAA04009.3|</td><td>lcl|Y41E3.13 CE18381 WBGene00012771 status:Predicted SW:Q9U2H6 protein_id:CAB63363.1 /QuerySize=187</td><td>gi|6904220|ref|Os03g42520|</td></tr>
<tr><td>gi|55245781|gb|EAA04014.2|</td><td>lcl|Y41E3.8 CE18376 WBGene00012766 status:Confirmed SW:O62437 protein_id:CAB09004.1 /QuerySize=110</td><td>gi|6904223|ref|Os03g42550|</td></tr>
<tr><td>gi|55245783|gb|EAA04010.2|</td><td>lcl|Y41E3.9 CE18377 WBGene00012767 status:Partially_confirmed SW:Q9U2H4 protein_id:CAB63365.1 /QuerySize=1306</td><td>gi|6904224|ref|Os03g42560|</td></tr>
<tr><td>gi|55245789|gb|EAL41882.1|</td><td>lcl|Y41G9A.2 CE35801 WBGene00021526 status:Partially_confirmed SW:Q9N500 protein_id:AAF60548.2 /QuerySize=242</td><td>gi|6904226|ref|Os03g42580|</td></tr>
<tr><td>gi|55245792|gb|EAA04019.2|</td><td>lcl|Y41G9A.5 CE21861 WBGene00021529 status:Partially_confirmed SW:Q9N503 protein_id:AAF60547.1 /QuerySize=402</td><td>gi|6904227|ref|Os03g42590|</td></tr>
<tr><td>gi|55245795|gb|EAL41884.1|</td><td>lcl|Y41G9A.6 CE28362 WBGene00021530 status:Partially_confirmed SW:Q9N4Z8 protein_id:AAF60552.2 /QuerySize=524</td><td>gi|6904228|ref|Os03g42600|</td></tr>
<tr><td>gi|55245799|gb|EAA45440.2|</td><td>lcl|Y42A5A.3 CE20257 WBGene00012778 status:Confirmed SW:Q9XWZ5 protein_id:CAA21486.1 /QuerySize=114</td><td>gi|6904230|ref|Os03g42620|</td></tr>
<tr><td>gi|55245809|gb|EAA03990.3|</td><td>lcl|Y42G9A.1 CE24274 WBGene00021531 status:Confirmed SW:Q9N4Z2 protein_id:AAF60556.1 /QuerySize=301</td><td>gi|6904232|ref|Os03g42640|</td></tr>
<tr><td>gi|55245831|gb|EAL41891.1|</td><td>lcl|Y42G9A.2 CE24275 WBGene00021532 status:Confirmed SW:Q9N4Z4 protein_id:AAF60557.1 /QuerySize=121</td><td>gi|6904236|ref|Os03g42670|</td></tr>
<tr><td>gi|55245842|gb|EAL41894.1|</td><td>lcl|Y42H9AR.4 CE37682 WBGene00021539 status:Partially_confirmed SW:Q9N3Y3 protein_id:AAF60561.3 /QuerySize=627</td><td>gi|6904237|ref|Os03g42680|</td></tr>
<tr><td>gi|55245845|gb|EAL41896.1|</td><td>lcl|Y42H9AR.5 CE29112 WBGene00021540 status:Confirmed SW:Q95XZ8 protein_id:AAL00867.1 /QuerySize=119</td><td>gi|6904239|ref|Os03g42700|</td></tr>
<tr><td>gi|55245847|gb|EAL41897.1|</td><td>lcl|Y42H9B.3 CE25279 WBGene00021541 status:Partially_confirmed SW:Q9N3X7 protein_id:AAF60565.1 /QuerySize=295</td><td>gi|6904241|ref|Os03g42720|</td></tr>
<tr><td>gi|55245849|gb|EAL41899.1|</td><td>lcl|Y43B11AR.1 CE27633 WBGene00021543 status:Partially_confirmed SW:Q9N3X4 protein_id:AAF60572.2 /QuerySize=214</td><td>gi|6904242|ref|Os03g42730|</td></tr>
<tr><td>gi|55245864|gb|EAL41900.1|</td><td>lcl|Y43C5A.3 CE19152 WBGene00012783 status:Confirmed SW:Q9XXF1 protein_id:CAA19503.1 /QuerySize=108</td><td>gi|6904249|ref|Os03g42800|</td></tr>
<tr><td>gi|55245865|gb|EAL41901.1|</td><td>lcl|Y43C5A.4 CE19153 WBGene00012784 status:Partially_confirmed SW:Q9XXF0 protein_id:CAA19504.1 /QuerySize=218</td><td>gi|6904256|ref|Os03g42850|</td></tr>
<tr><td>gi|55245866|gb|EAL41902.1|</td><td>lcl|Y43D4A.3 CE21876 WBGene00012789 status:Predicted SW:Q9NF28 protein_id:CAB60344.1 /QuerySize=327</td><td>gi|6904258|ref|Os03g42870|</td></tr>
<tr><td>gi|55245867|gb|EAA04302.2|</td><td>lcl|Y43D4A.4 CE21877 WBGene00012790 status:Predicted SW:Q9NF27 protein_id:CAB60345.1 /QuerySize=266</td><td>gi|6904263|ref|Os03g42910|</td></tr>
<tr><td>gi|55245871|gb|EAL41905.1|</td><td>lcl|Y43D4A.7 CE28121 WBGene00012793 status:Predicted SW:Q95ZJ0 protein_id:CAC42373.1 /QuerySize=86</td><td>gi|6904264|ref|Os03g42920|</td></tr>
<tr><td>gi|55245872|gb|EAA04301.2|</td><td>lcl|Y43F11A.1 CE21916 WBGene00012841 status:Predicted SW:Q9TVU3 protein_id:CAB55038.1 /QuerySize=189</td><td>gi|6904266|ref|Os03g42940|</td></tr>
<tr><td>gi|55245875|gb|EAL41906.1|</td><td>lcl|Y43F11A.2 CE36412 WBGene00012842 status:Partially_confirmed SW:Q9U2H0 protein_id:CAB55039.2 /QuerySize=155</td><td>gi|6904270|ref|Os03g42980|</td></tr>
<tr><td>gi|55245876|gb|EAA04401.2|</td><td>lcl|Y43F11A.4 CE21916 WBGene00012844 status:Predicted SW:Q9TVU3 protein_id:CAB55041.1 /QuerySize=189</td><td>gi|6904271|ref|Os03g42990|</td></tr>
<tr><td>gi|55245883|gb|EAL41907.1|</td><td>lcl|Y43F4A.3 CE16624 WBGene00012798 status:Predicted SW:O45929 protein_id:CAB16473.1 /QuerySize=172</td><td>gi|6904272|ref|Os03g43000|</td></tr>
<tr><td>gi|55245910|gb|EAL41912.1|</td><td>lcl|Y43F4B.2 CE28790 WBGene00012801 status:Partially_confirmed SW:O45931 protein_id:CAA16331.2 /QuerySize=557</td><td>gi|6904275|ref|Os03g43030|</td></tr>
<tr><td>gi|55245915|gb|EAA45460.2|</td><td>lcl|Y43F4B.3 CE16628 WBGene00012802 status:Partially_confirmed SW:O45932 protein_id:CAA16332.1 /QuerySize=216</td><td>gi|6904277|ref|Os03g43050|</td></tr>
<tr><td>gi|55245923|gb|EAA45461.2|</td><td>lcl|Y43F4B.8 CE16633 WBGene00012805 status:Confirmed SW:O45937 protein_id:CAA16337.1 /QuerySize=205</td><td>gi|6904278|ref|Os03g43060|</td></tr>
<tr><td>gi|55245925|gb|EAL41917.1|</td><td>lcl|Y43F4B.9 CE28123 WBGene00012806 status:Confirmed SW:Q95Q24 protein_id:CAC42370.1 /QuerySize=127</td><td>gi|6904282|ref|Os03g43100|</td></tr>
<tr><td>gi|55245935|gb|EAL41920.1|</td><td>lcl|Y43F8A.1 CE21880 WBGene00012808 status:Predicted SW:Q9XWL6 protein_id:CAA21640.1 /QuerySize=62</td><td>gi|6904283|ref|Os03g43110|</td></tr>
<tr><td>gi|55245938|gb|EAA45465.2|</td><td>lcl|Y43F8A.2 CE24284 WBGene00012809 status:Partially_confirmed SW:Q9U2G6 protein_id:CAA21643.2 /QuerySize=502</td><td>gi|6904284|ref|Os03g43120|</td></tr>
<tr><td>gi|55245939|gb|EAA45466.2|</td><td>lcl|Y43F8B.10 CE32751 WBGene00012820 status:Predicted SW:Q9XWY0 protein_id:CAA21506.2 /QuerySize=300</td><td>gi|6904285|ref|Os03g43130|</td></tr>
<tr><td>gi|55245943|gb|EAL41921.1|</td><td>lcl|Y43F8B.11 CE21895 WBGene00012821 status:Partially_confirmed SW:Q9XWY1 protein_id:CAA21505.1 /QuerySize=178</td><td>gi|6904286|ref|Os03g43140|</td></tr>
<tr><td>gi|55245944|gb|EAL41922.1|</td><td>lcl|Y43F8B.12 CE21896 WBGene00012822 status:Partially_confirmed SW:Q9XWX0 protein_id:CAA21517.1 /QuerySize=147</td><td>gi|6904288|ref|Os03g43160|</td></tr>
<tr><td>gi|55245949|gb|EAL41924.1|</td><td>lcl|Y43F8B.13 CE21897 WBGene00012823 status:Partially_confirmed SW:Q9XWY2 protein_id:CAA21504.1 /QuerySize=465</td><td>gi|6904291|ref|Os03g43190|</td></tr>
<tr><td>gi|55245950|gb|EAL41925.1|</td><td>lcl|Y43F8B.14 CE21898 WBGene00012824 status:Partially_confirmed SW:Q9XWW9 protein_id:CAA21518.1 /QuerySize=593</td><td>gi|6904292|ref|Os03g43200|</td></tr>
<tr><td>gi|55245952|gb|EAA45474.2|</td><td>lcl|Y43F8B.1a CE32015 WBGene00012812 status:Confirmed SW:Q9XWX8 protein_id:CAA21508.2 /QuerySize=309</td><td>gi|6904303|ref|Os03g43310|</td></tr>
<tr><td>gi|55245966|gb|EAL41928.1|</td><td>lcl|Y43F8B.1b CE32016 WBGene00012812 status:Confirmed SW:Q8I4D3 protein_id:CAD54165.1 /QuerySize=312</td><td>gi|6904304|ref|Os03g43320|</td></tr>
<tr><td>gi|55245967|gb|EAA04242.2|</td><td>lcl|Y43F8B.6 CE21891 WBGene00012817 status:Predicted SW:Q9XWX9 protein_id:CAA21507.1 /QuerySize=63</td><td>gi|6904305|ref|Os03g43330|</td></tr>
<tr><td>gi|55245990|gb|EAL41938.1|</td><td>lcl|Y43F8B.7 CE21892 WBGene00012818 status:Partially_confirmed SW:Q9XWX2 protein_id:CAA21514.1 /QuerySize=123</td><td>gi|6904306|ref|Os03g43340|</td></tr>
<tr><td>gi|55246013|gb|EAA04058.2|</td><td>lcl|Y43F8B.9 CE21893 WBGene00012819 status:Partially_confirmed SW:Q9XWX1 protein_id:CAA21516.1 /QuerySize=111</td><td>gi|6904307|ref|Os03g43350|</td></tr>
<tr><td>gi|55246015|gb|EAL41945.1|</td><td>lcl|Y43F8C.1 CE21899 WBGene00003763 locus:nlp-25 status:Partially_confirmed SW:Q9XWP3 protein_id:CAA21608.1 /QuerySiz</td><td>gi|6904309|ref|Os03g43370|</td></tr>
<tr><td>gi|55246017|gb|EAL41947.1|</td><td>lcl|Y43F8C.11 CE21909 WBGene00012833 status:Partially_confirmed SW:Q9XWN8 protein_id:CAA21613.1 /QuerySize=96</td><td>gi|6904317|ref|Os03g43450|</td></tr>
<tr><td>gi|55246027|gb|EAA04182.2|</td><td>lcl|Y43F8C.15 CE21913 WBGene00012836 status:Partially_confirmed SW:Q9XWM9 protein_id:CAA21623.1 /QuerySize=117</td><td>gi|6904318|ref|Os03g43460|</td></tr>
<tr><td>gi|55246036|gb|EAA04053.2|</td><td>lcl|Y43F8C.16 CE34118 WBGene00012837 status:Partially_confirmed SW:Q9XWN5 protein_id:CAA21616.2 /QuerySize=370</td><td>gi|6904320|ref|Os03g43480|</td></tr>
<tr><td>gi|55246042|gb|EAL41951.1|</td><td>lcl|Y43F8C.17 CE21915 WBGene00012838 status:Predicted SW:Q9XWM8 protein_id:CAA21624.1 /QuerySize=169</td><td>gi|6904321|ref|Os03g43490|</td></tr>
<tr><td>gi|55246048|gb|EAL41953.1|</td><td>lcl|Y43F8C.18 CE36456 WBGene00012839 status:Partially_confirmed SW:Q8I4D1 protein_id:CAD54170.3 /QuerySize=140</td><td>gi|6904322|ref|Os03g43500|</td></tr>
<tr><td>gi|55246050|gb|EAL41954.1|</td><td>lcl|Y43F8C.2 CE21900 WBGene00003764 locus:nlp-26 status:Confirmed SW:Q9XWN4 protein_id:CAA21617.1 /QuerySize=90</td><td>gi|6904325|ref|Os03g43520|</td></tr>
<tr><td>gi|55246059|gb|EAA04449.2|</td><td>lcl|Y43F8C.20 CE35086 WBGene00012840 status:Partially_confirmed SW:Q7YWQ8 protein_id:CAE17998.1 /QuerySize=193</td><td>gi|6904326|ref|Os03g43530|</td></tr>
<tr><td>gi|55246061|gb|EAA04441.3|</td><td>lcl|Y43F8C.4 CE34119 WBGene00012826 status:Partially_confirmed SW:Q9XWN2 protein_id:CAA21619.2 /QuerySize=534</td><td>gi|6904327|ref|Os03g43540|</td></tr>
<tr><td>gi|55246062|gb|EAL41956.1|</td><td>lcl|Y43F8C.5 CE21903 WBGene00012827 status:Partially_confirmed SW:Q9XWP2 protein_id:CAA21609.1 /QuerySize=245</td><td>gi|6904329|ref|Os03g43560|</td></tr>
<tr><td>gi|55246067|gb|EAA04447.3|</td><td>lcl|Y43F8C.9 CE21907 WBGene00012831 status:Partially_confirmed SW:Q9XWN0 protein_id:CAA21621.1 /QuerySize=108</td><td>gi|6904334|ref|Os03g43600|</td></tr>
<tr><td>gi|55246073|gb|EAL41961.1|</td><td>lcl|Y43H11AL.1 CE32968 WBGene00021545 status:Confirmed SW:Q8IAA7 protein_id:AAN39662.2 /QuerySize=367</td><td>gi|6904336|ref|Os03g43620|</td></tr>
<tr><td>gi|55246074|gb|EAA04450.2|</td><td>lcl|Y44A6C.1 CE28852 WBGene00012850 status:Partially_confirmed SW:O62452 protein_id:CAA16369.2 /QuerySize=325</td><td>gi|6904337|ref|Os03g43630|</td></tr>
<tr><td>gi|55246078|gb|EAL41963.1|</td><td>lcl|Y44A6C.2 CE18389 WBGene00012851 status:Confirmed SW:O62453 protein_id:CAA16370.1 /QuerySize=333</td><td>gi|6904338|ref|Os03g43640|</td></tr>
<tr><td>gi|55246079|gb|EAA04423.2|</td><td>lcl|Y44A6D.1 CE19159 WBGene00012852 status:Partially_confirmed SW:Q9XXE3 protein_id:CAA19514.1 /QuerySize=44</td><td>gi|6904343|ref|Os03g43690|</td></tr>
<tr><td>gi|55246089|gb|EAL41967.1|</td><td>lcl|Y44A6D.2 CE19160 WBGene00012853 status:Confirmed SW:Q9XXE2 protein_id:CAA19515.1 /QuerySize=76</td><td>gi|6904344|ref|Os03g43700|</td></tr>
<tr><td>gi|55246090|gb|EAL41968.1|</td><td>lcl|Y44A6D.6 CE19163 WBGene00012856 status:Predicted SW:Q9XXE1 protein_id:CAA19516.1 /QuerySize=123</td><td>gi|6904345|ref|Os03g43710|</td></tr>
<tr><td>gi|55246096|gb|EAL41971.1|</td><td>lcl|Y44E3A.1a CE21926 WBGene00021547 status:Partially_confirmed SW:Q9TXZ1 protein_id:AAC78227.1 /QuerySize=154</td><td>gi|6904352|ref|Os03g43740|</td></tr>
<tr><td>gi|55246110|gb|EAL41975.1|</td><td>lcl|Y44E3A.1b CE33225 WBGene00021547 status:Partially_confirmed SW:Q86S15 protein_id:AAO21410.1 /QuerySize=83</td><td>gi|6904353|ref|Os03g43750|</td></tr>
<tr><td>gi|55246117|gb|EAA04462.2|</td><td>lcl|Y44E3A.6a CE31372 WBGene00021551 status:Confirmed SW:Q8ITV7 protein_id:AAN73852.1 /QuerySize=841</td><td>gi|6904356|ref|Os03g43780|</td></tr>
<tr><td>gi|55246122|gb|EAL41980.1|</td><td>lcl|Y44E3A.6b CE31373 WBGene00021551 status:Confirmed SW:Q8ITV6 protein_id:AAN73853.1 /QuerySize=843</td><td>gi|6904357|ref|Os03g43790|</td></tr>
<tr><td>gi|55246124|gb|EAA04460.2|</td><td>lcl|Y44E3B.1a CE18390 WBGene00021552 status:Confirmed SW:O76709 protein_id:AAK21516.1 /QuerySize=318</td><td>gi|6904359|ref|Os03g43810|</td></tr>
<tr><td>gi|55246125|gb|EAL41981.1|</td><td>lcl|Y44E3B.1b CE28364 WBGene00021552 status:Confirmed SW:Q965I4 protein_id:AAK68487.1 /QuerySize=295</td><td>gi|6904360|ref|Os03g43820|</td></tr>
<tr><td>gi|55246151|gb|EAA03899.2|</td><td>lcl|Y45F10A.1 CE18392 WBGene00012865 status:Partially_confirmed SW:O62458 protein_id:CAA16365.1 /QuerySize=292</td><td>gi|6904361|ref|Os03g43830|</td></tr>
<tr><td>gi|55246160|gb|EAA03892.2|</td><td>lcl|Y45F10A.5 CE18396 WBGene00003755 locus:nlp-17 status:Confirmed SW:O62455 protein_id:CAA16362.1 /QuerySize=83</td><td>gi|6904365|ref|Os03g43870|</td></tr>
<tr><td>gi|55246167|gb|EAL41991.1|</td><td>lcl|Y45F10B.13a CE32022 WBGene00012875 status:Confirmed SW:O62465 protein_id:CAA16360.3 /QuerySize=633</td><td>gi|6904371|ref|Os03g43920|</td></tr>
<tr><td>gi|55246183|gb|EAA03856.3|</td><td>lcl|Y45F10B.13b CE37429 WBGene00012875 status:Confirmed SW:Q5ZEQ6 protein_id:CAH60766.1 /QuerySize=618</td><td>gi|6904373|ref|Os03g43940|</td></tr>
<tr><td>gi|55246187|gb|EAL41997.1|</td><td>lcl|Y45F10B.2 CE18401 WBGene00012870 status:Partially_confirmed SW:O62467 protein_id:CAA16353.1 /QuerySize=413</td><td>gi|6904374|ref|Os03g43950|</td></tr>
<tr><td>gi|55246191|gb|EAA03844.3|</td><td>lcl|Y45F10B.3 CE18402 WBGene00012871 status:Partially_confirmed SW:O62468 protein_id:CAA16354.1 /QuerySize=75</td><td>gi|6904375|ref|Os03g43960|</td></tr>
<tr><td>gi|55246192|gb|EAA03872.2|</td><td>lcl|Y45F10D.14 CE35088 WBGene00012892 status:Predicted SW:Q7YWQ7 protein_id:CAE17999.1 /QuerySize=282</td><td>gi|6904382|ref|Os03g44030|</td></tr>
<tr><td>gi|55246195|gb|EAA03833.2|</td><td>lcl|Y45F10D.15 CE35089 WBGene00012893 status:Predicted SW:Q7YSN8 protein_id:CAE18000.1 /QuerySize=308</td><td>gi|6904383|ref|Os03g44040|</td></tr>
<tr><td>gi|55246200|gb|EAL42001.1|</td><td>lcl|Y45F10D.2 CE16641 WBGene00012883 status:Partially_confirmed SW:O45945 protein_id:CAA16377.1 /QuerySize=179</td><td>gi|6904387|ref|Os03g44080|</td></tr>
<tr><td>gi|55246211|gb|EAL42005.1|</td><td>lcl|Y45F10D.6 CE16644 WBGene00012886 status:Predicted SW:O62476 protein_id:CAA16381.1 /QuerySize=202</td><td>gi|6904391|ref|Os03g44120|</td></tr>
<tr><td>gi|55246222|gb|EAA03733.2|</td><td>lcl|Y45F10D.9 CE16647 WBGene00012888 locus:sas-6 status:Partially_confirmed SW:O62479 protein_id:CAA16384.1 /QuerySize</td><td>gi|6904395|ref|Os03g44160|</td></tr>
<tr><td>gi|55246227|gb|EAL42007.1|</td><td>lcl|Y45F3A.1 CE19164 WBGene00012859 status:Confirmed SW:Q9XTF1 protein_id:CAA21496.1 /QuerySize=329</td><td>gi|6904397|ref|Os03g44180|</td></tr>
<tr><td>gi|55246235|gb|EAA03668.2|</td><td>lcl|Y45F3A.4 CE33226 WBGene00012861 status:Confirmed SW:Q9XWZ1 protein_id:CAA21491.2 /QuerySize=147</td><td>gi|6904398|ref|Os03g44190|</td></tr>
<tr><td>gi|55246267|gb|EAL42014.1|</td><td>lcl|Y45F3A.5 CE19168 WBGene00012862 status:Predicted SW:Q9XWY9 protein_id:CAA21493.1 /QuerySize=81</td><td>gi|6904401|ref|Os03g44220|</td></tr>
<tr><td>gi|55246305|gb|EAL42025.1|</td><td>lcl|Y45G5AL.1a CE30008 WBGene00021554 status:Confirmed SW:Q8WTL9 protein_id:AAL32245.1 /QuerySize=273</td><td>gi|6904405|ref|Os03g44260|</td></tr>
<tr><td>gi|55246311|gb|EAL42028.1|</td><td>lcl|Y45G5AL.1b CE30009 WBGene00021554 status:Confirmed SW:Q8WTM0 protein_id:AAL32246.1 /QuerySize=275</td><td>gi|6904412|ref|Os03g44320|</td></tr>
<tr><td>gi|55246317|gb|EAL42030.1|</td><td>lcl|Y45G5AM.3 CE26405 WBGene00021556 status:Partially_confirmed SW:Q9GUI8 protein_id:AAG23365.1 /QuerySize=410</td><td>gi|6904413|ref|Os03g44330|</td></tr>
<tr><td>gi|55246326|gb|EAL42033.1|</td><td>lcl|Y45G5AM.5 CE33342 WBGene00021557 status:Partially_confirmed SW:Q9GUJ1 protein_id:AAG23363.2 /QuerySize=167</td><td>gi|6904414|ref|Os03g44340|</td></tr>
<tr><td>gi|55246332|gb|EAL42035.1|</td><td>lcl|Y45G5AM.9a CE30010 WBGene00021561 status:Partially_confirmed SW:Q9GUI5 protein_id:AAG23358.2 /QuerySize=499</td><td>gi|6904415|ref|Os03g44350|</td></tr>
<tr><td>gi|55246343|gb|EAL42038.1|</td><td>lcl|Y45G5AM.9b CE33343 WBGene00021561 status:Partially_confirmed SW:Q86N73 protein_id:AAO25978.1 /QuerySize=423</td><td>gi|6904421|ref|Os03g44410|</td></tr>
<tr><td>gi|55246345|gb|EAL42039.1|</td><td>lcl|Y46B2A.1 CE21951 WBGene00021576 status:Partially_confirmed SW:Q9N4X2 protein_id:AAF60595.1 /QuerySize=508</td><td>gi|6904436|ref|Os03g44550|</td></tr>
<tr><td>gi|55246346|gb|EAA03680.2|</td><td>lcl|Y46D2A.3 CE25301 WBGene00021591 status:Partially_confirmed SW:Q9N4W9 protein_id:AAF60608.1 /QuerySize=85</td><td>gi|6904441|ref|Os03g44570|</td></tr>
</table>

<table>
<tr><td>gi|55246358|gb|EAL42042.1|</td><td>lcl|Y46E12A.1 CE26996 WBGene00000560 locus:cnc-6 status:Partially_confirmed SW:Q9N3V6 protein_id:AAF60614.2 /QuerySiz</td><td>gi|6904443|ref|Os03g44600|</td></tr>
<tr><td>gi|55246359|gb|EAA03656.2|</td><td>lcl|Y46E12A.2 CE23404 WBGene00021592 status:Predicted SW:Q9N3V7 protein_id:AAF60613.1 /QuerySize=163</td><td>gi|6904452|ref|Os03g44680|</td></tr>
<tr><td>gi|55246361|gb|EAA03750.2|</td><td>lcl|Y46E12BR.1 CE37170 WBGene00021598 status:Partially_confirmed SW:Q965N5 protein_id:AAK68492.2 /QuerySize=185</td><td>gi|6904453|ref|Os03g44690|</td></tr>
<tr><td>gi|55246372|gb|EAA03746.2|</td><td>lcl|Y46G5A.10 CE37941 WBGene00012901 status:Partially_confirmed SW:Q9U2D6 protein_id:CAB60376.2 /QuerySize=732</td><td>gi|6904454|ref|Os03g44700|</td></tr>
<tr><td>gi|55246377|gb|EAL42044.1|</td><td>lcl|Y46G5A.14 CE21989 WBGene00012905 status:Confirmed SW:Q9U2F4 protein_id:CAB60357.1 /QuerySize=100</td><td>gi|6904456|ref|Os03g44720|</td></tr>
<tr><td>gi|55246381|gb|EAA03620.2|</td><td>lcl|Y46G5A.15 CE35666 WBGene00012906 status:Confirmed SW:Q9U2F3 protein_id:CAB60358.2 /QuerySize=304</td><td>gi|6904457|ref|Os03g44730|</td></tr>
<tr><td>gi|55246398|gb|EAL42050.1|</td><td>lcl|Y46G5A.18 CE21995 WBGene00012908 status:Partially_confirmed SW:Q9U2F1 protein_id:CAB60360.1 /QuerySize=375</td><td>gi|6904459|ref|Os03g44750|</td></tr>
<tr><td>gi|55246403|gb|EAA45562.2|</td><td>lcl|Y46G5A.20 CE24293 WBGene00012910 status:Confirmed SW:Q9U2E9 protein_id:CAB60362.1 /QuerySize=109</td><td>gi|6904463|ref|Os03g44770|</td></tr>
<tr><td>gi|55246404|gb|EAL42051.1|</td><td>lcl|Y46G5A.22a CE21999 WBGene00012912 status:Confirmed SW:Q9U2E7 protein_id:CAB60364.1 /QuerySize=295</td><td>gi|6904465|ref|Os03g44790|</td></tr>
<tr><td>gi|55246406|gb|EAA03605.2|</td><td>lcl|Y46G5A.22b CE24295 WBGene00012912 status:Confirmed SW:Q7K712 protein_id:CAB60365.1 /QuerySize=272</td><td>gi|6904471|ref|Os03g44850|</td></tr>
<tr><td>gi|55246410|gb|EAL42053.1|</td><td>lcl|Y46G5A.23 CE21968 WBGene00012913 status:Partially_confirmed SW:Q9U2E5 protein_id:CAB60366.1 /QuerySize=108</td><td>gi|6904472|ref|Os03g44860|</td></tr>
<tr><td>gi|55246432|gb|EAL42057.1|</td><td>lcl|Y46G5A.26b CE31117 WBGene00012915 status:Partially_confirmed SW:Q8MPP4 protein_id:CAD36500.1 /QuerySize=145</td><td>gi|6904473|ref|Os03g44870|</td></tr>
<tr><td>gi|55246449|gb|EAL42061.1|</td><td>lcl|Y46G5A.34 CE35090 WBGene00012919 status:Partially_confirmed SW:Q7YWP6 protein_id:CAE18011.1 /QuerySize=100</td><td>gi|6904477|ref|Os03g44910|</td></tr>
<tr><td>gi|55246455|gb|EAL42063.1|</td><td>lcl|Y46H3A.5 CE24308 WBGene00021601 status:Partially_confirmed SW:Q9N4W6 protein_id:AAF60619.1 /QuerySize=129</td><td>gi|6904478|ref|Os03g44920|</td></tr>
<tr><td>gi|55246463|gb|EAL42065.1|</td><td>lcl|Y46H3C.5 CE24316 WBGene00021605 status:Predicted SW:Q9N4W2 protein_id:AAF60627.1 /QuerySize=172</td><td>gi|6904479|ref|Os03g44930|</td></tr>
<tr><td>gi|55246466|gb|EAL42066.1|</td><td>lcl|Y46H3C.6 CE35806 WBGene00021606 status:Partially_confirmed SW:Q9N4W1 protein_id:AAF60628.2 /QuerySize=354</td><td>gi|6904482|ref|Os03g44960|</td></tr>
<tr><td>gi|55246468|gb|EAA03525.2|</td><td>lcl|Y46H3C.7 CE36340 WBGene00021607 status:Partially_confirmed SW:Q7KX43 protein_id:AAR85896.1 /QuerySize=88</td><td>gi|6904483|ref|Os03g44970|</td></tr>
<tr><td>gi|55246469|gb|EAL42067.1|</td><td>lcl|Y46H3D.1 CE24318 WBGene00021608 status:Partially_confirmed SW:Q966B2 protein_id:AAK72304.1 /QuerySize=58</td><td>gi|6904484|ref|Os03g44980|</td></tr>
<tr><td>gi|55246470|gb|EAL42068.1|</td><td>lcl|Y47A7.2 CE27257 WBGene00021614 status:Partially_confirmed SW:Q9BKR3 protein_id:AAK27878.1 /QuerySize=255</td><td>gi|6904485|ref|Os03g44990|</td></tr>
<tr><td>gi|55246475|gb|EAL42070.1|</td><td>lcl|Y47D3A.13 CE24329 WBGene00012926 status:Partially_confirmed SW:Q9U2C8 protein_id:CAB57893.1 /QuerySize=136</td><td>gi|6904486|ref|Os03g45000|</td></tr>
<tr><td>gi|55246479|gb|EAL42072.1|</td><td>lcl|Y47D3A.20 CE34121 WBGene00012931 status:Partially_confirmed SW:Q7YXC5 protein_id:CAD91710.1 /QuerySize=121</td><td>gi|6904487|ref|Os03g45010|</td></tr>
<tr><td>gi|55246485|gb|EAA03503.2|</td><td>lcl|Y47D3A.30 CE30360 WBGene00001246 locus:elo-8 status:Partially_confirmed SW:Q8T3A8 protein_id:CAD27612.1 /QuerySiz</td><td>gi|6904488|ref|Os03g45020|</td></tr>
<tr><td>gi|55246494|gb|EAL42076.1|</td><td>lcl|Y47D3A.31 CE22031 WBGene00012937 status:Confirmed SW:Q7YWP8 protein_id:CAE18009.1 /QuerySize=196</td><td>gi|6904492|ref|Os03g45060|</td></tr>
<tr><td>gi|55246498|gb|EAL42078.1|</td><td>lcl|Y47D3A.32 CE35091 WBGene00012938 status:Predicted SW:Q7YWP7 protein_id:CAE18010.1 /QuerySize=98</td><td>gi|6904494|ref|Os03g45080|</td></tr>
<tr><td>gi|55246502|gb|EAL42080.1|</td><td>lcl|Y47D3A.5 CE34122 WBGene00012924 status:Partially_confirmed SW:Q9U2D1 protein_id:CAB55095.3 /QuerySize=222</td><td>gi|6904495|ref|Os03g45090|</td></tr>
<tr><td>gi|55246509|gb|EAA03470.2|</td><td>lcl|Y47D3B.2a CE19175 WBGene00003759 locus:nlp-21 status:Confirmed SW:Q9XX02 protein_id:CAA21040.1 /QuerySize=164</td><td>gi|6904496|ref|Os03g45100|</td></tr>
<tr><td>gi|55246520|gb|EAL42086.1|</td><td>lcl|Y47D3B.2b CE24343 WBGene00003759 locus:nlp-21 status:Confirmed SW:Q9U2B9 protein_id:CAB54383.1 /QuerySize=149</td><td>gi|6904499|ref|Os03g45130|</td></tr>
<tr><td>gi|55246521|gb|EAL42087.1|</td><td>lcl|Y47D7A.10 CE24347 WBGene00021622 status:Predicted SW:Q9N3U5 protein_id:AAF60633.1 /QuerySize=47</td><td>gi|6904501|ref|Os03g45150|</td></tr>
<tr><td>gi|55246536|gb|EAA03425.2|</td><td>lcl|Y47D7A.11 CE35807 WBGene00021623 status:Partially_confirmed SW:Q9N3U4 protein_id:AAF60632.2 /QuerySize=129</td><td>gi|6904502|ref|Os03g45160|</td></tr>
<tr><td>gi|55246540|gb|EAA45610.2|</td><td>lcl|Y47D7A.12 CE22061 WBGene00021624 status:Partially_confirmed SW:Q9N3U3 protein_id:AAF60631.1 /QuerySize=199</td><td>gi|6904509|ref|Os03g45220|</td></tr>
<tr><td>gi|55246545|gb|EAL42091.1|</td><td>lcl|Y47D7A.13 CE22060 WBGene00021625 status:Partially_confirmed SW:Q9N3U2 protein_id:AAL32247.1 /QuerySize=311</td><td>gi|6904510|ref|Os03g45230|</td></tr>
<tr><td>gi|55246561|gb|EAL42098.1|</td><td>lcl|Y47D7A.15 CE36236 WBGene00021627 status:Partially_confirmed SW:Q8WTL8 protein_id:AAL32248.2 /QuerySize=97</td><td>gi|6904511|ref|Os03g45240|</td></tr>
<tr><td>gi|55246565|gb|EAA03392.2|</td><td>lcl|Y47D7A.2 CE26109 WBGene00021616 status:Partially_confirmed SW:Q9N3V0 protein_id:AAF60640.2 /QuerySize=119</td><td>gi|6904515|ref|Os03g45280|</td></tr>
<tr><td>gi|55246566|gb|EAA03391.2|</td><td>lcl|Y47D7A.6 CE24349 WBGene00021619 status:Predicted SW:Q9N3V2 protein_id:AAF60637.1 /QuerySize=446</td><td>gi|6904516|ref|Os03g45280|</td></tr>
<tr><td>gi|55246567|gb|EAA03393.3|</td><td>lcl|Y47D7A.7 CE24348 WBGene00021620 status:Partially_confirmed SW:Q9N3V1 protein_id:AAF60636.1 /QuerySize=121</td><td>gi|6904519|ref|Os03g45310|</td></tr>
<tr><td>gi|55246579|gb|EAA03368.2|</td><td>lcl|Y47D7A.9 CE22056 WBGene00021621 status:Partially_confirmed SW:Q9N3U7 protein_id:AAF60634.1 /QuerySize=91</td><td>gi|6904523|ref|Os03g45350|</td></tr>
<tr><td>gi|55246583|gb|EAL42104.1|</td><td>lcl|Y47D9A.3 CE34398 WBGene00021630 status:Partially_confirmed SW:Q9N4V6 protein_id:AAF60645.2 /QuerySize=74</td><td>gi|6904524|ref|Os03g45360|</td></tr>
<tr><td>gi|55246585|gb|EAL42105.1|</td><td>lcl|Y47D9A.4 CE34399 WBGene00021631 status:Partially_confirmed SW:Q9N4V7 protein_id:AAF60644.2 /QuerySize=89</td><td>gi|6904526|ref|Os03g45380|</td></tr>
<tr><td>gi|55246596|gb|EAL42108.1|</td><td>lcl|Y47D9A.5 CE24354 WBGene00021632 status:Partially_confirmed SW:Q9N4V5 protein_id:AAF60649.1 /QuerySize=447</td><td>gi|6904531|ref|Os03g45430|</td></tr>
<tr><td>gi|55246600|gb|EAA03329.3|</td><td>lcl|Y47G6A.1 CE27258 WBGene00002143 locus:inx-21 status:Partially_confirmed SW:Q9N3R4 protein_id:AAF60654.2 /QuerySiz</td><td>gi|6904532|ref|Os03g45440|</td></tr>
<tr><td>gi|55246612|gb|EAA03312.2|</td><td>lcl|Y47G6A.15a CE37431 WBGene00021641 status:Partially_confirmed SW:Q65XX3 protein_id:AAU20832.1 /QuerySize=275</td><td>gi|6904534|ref|Os03g45460|</td></tr>
<tr><td>gi|55246616|gb|EAA03308.3|</td><td>lcl|Y47G6A.15b CE22105 WBGene00021641 status:Confirmed SW:Q65XX4 protein_id:AAU20833.1 /QuerySize=278</td><td>gi|6904535|ref|Os03g45470|</td></tr>
<tr><td>gi|55246617|gb|EAA03307.2|</td><td>lcl|Y47G6A.16 CE36744 WBGene00021642 status:Predicted SW:Q9N3T1 protein_id:AAF60663.2 /QuerySize=559</td><td>gi|6904538|ref|Os03g45500|</td></tr>
<tr><td>gi|55246620|gb|EAL42115.1|</td><td>lcl|Y47G6A.24 CE24370 WBGene00021648 locus:mis-12 status:Partially_confirmed SW:Q9N3R7 protein_id:AAF60669.1 /QuerySi</td><td>gi|6904544|ref|Os03g45560|</td></tr>
<tr><td>gi|55246629|gb|EAL42116.1|</td><td>lcl|Y47G6A.25 CE34403 WBGene00021649 status:Partially_confirmed SW:Q9N3R6 protein_id:AAF60670.2 /QuerySize=440</td><td>gi|6904548|ref|Os03g45600|</td></tr>
<tr><td>gi|55246630|gb|EAA03284.2|</td><td>lcl|Y47G6A.26 CE22084 WBGene00021650 status:Partially_confirmed SW:Q9N3R3 protein_id:AAF60671.1 /QuerySize=196</td><td>gi|6904550|ref|Os03g45620|</td></tr>
<tr><td>gi|55246635|gb|EAL42119.1|</td><td>lcl|Y47G6A.28 CE28986 WBGene00006440 locus:tag-63 status:Partially_confirmed SW:Q95XZ1 protein_id:AAK95891.1 /QuerySi</td><td>gi|6904551|ref|Os03g45630|</td></tr>
<tr><td>gi|55246641|gb|EAA03263.2|</td><td>lcl|Y47G6A.3 CE22076 WBGene00021633 status:Partially_confirmed SW:Q9N3R8 protein_id:AAF60655.1 /QuerySize=181</td><td>gi|6904552|ref|Os03g45640|</td></tr>
<tr><td>gi|55246643|gb|EAA03255.3|</td><td>lcl|Y47G6A.30 CE34404 WBGene00021653 status:Partially_confirmed SW:Q7Z148 protein_id:AAP68912.1 /QuerySize=85</td><td>gi|6904553|ref|Os03g45650|</td></tr>
<tr><td>gi|55246645|gb|EAA03253.2|</td><td>lcl|Y47G6A.4 CE24371 WBGene00021634 status:Partially_confirmed SW:Q9N3S2 protein_id:AAF60656.1 /QuerySize=627</td><td>gi|6904554|ref|Os03g45660|</td></tr>
<tr><td>gi|55246651|gb|EAL42122.1|</td><td>lcl|Y47G6A.9 CE22108 WBGene00021638 status:Partially_confirmed SW:Q9N3T3 protein_id:AAF60652.1 /QuerySize=230</td><td>gi|6904556|ref|Os03g45680|</td></tr>
<tr><td>gi|55246652|gb|EAA03251.2|</td><td>lcl|Y47G7B.2 CE19624 WBGene00021654 status:Partially_confirmed SW:Q9TZD6 protein_id:AAC68731.1 /QuerySize=422</td><td>gi|6904558|ref|Os03g45700|</td></tr>
<tr><td>gi|55246659|gb|EAL42125.1|</td><td>lcl|Y47H10A.3 CE34512 WBGene00012959 status:Partially_confirmed SW:Q9U2B6 protein_id:CAB60385.2 /QuerySize=333</td><td>gi|6904566|ref|Os03g45780|</td></tr>
<tr><td>gi|55246662|gb|EAA03238.2|</td><td>lcl|Y47H10A.4 CE24377 WBGene00012960 status:Partially_confirmed SW:Q9U2B5 protein_id:CAB60386.1 /QuerySize=327</td><td>gi|6904567|ref|Os03g45790|</td></tr>
<tr><td>gi|55246674|gb|EAA03221.2|</td><td>lcl|Y47H10A.5 CE22113 WBGene00012961 status:Partially_confirmed SW:Q9U2B4 protein_id:CAB60387.1 /QuerySize=331</td><td>gi|6904572|ref|Os03g45840|</td></tr>
<tr><td>gi|55246690|gb|EAA45668.2|</td><td>lcl|Y47H9C.12 CE20272 WBGene00012955 status:Partially_confirmed SW:Q9XWD3 protein_id:CAA21742.1 /QuerySize=72</td><td>gi|6904575|ref|Os03g45870|</td></tr>
<tr><td>gi|55246692|gb|EAA03199.2|</td><td>lcl|Y47H9C.15 CE28706 WBGene00012957 status:Predicted SW:Q95Q21 protein_id:CAC42375.2 /QuerySize=190</td><td>gi|6904576|ref|Os03g45880|</td></tr>
<tr><td>gi|55246701|gb|EAA03185.2|</td><td>lcl|Y47H9C.8 CE20268 WBGene00012951 status:Partially_confirmed SW:Q9U2B1 protein_id:CAB63370.1 /QuerySize=389</td><td>gi|6904577|ref|Os03g45890|</td></tr>
<tr><td>gi|55246706|gb|EAA03168.2|</td><td>lcl|Y48A6B.10 CE24378 WBGene00012971 status:Partially_confirmed SW:Q9U2B0 protein_id:CAA19525.2 /QuerySize=446</td><td>gi|6904578|ref|Os03g45900|</td></tr>
<tr><td>gi|55246727|gb|EAL42141.1|</td><td>lcl|Y48A6B.11a CE24379 WBGene00012972 status:Partially_confirmed SW:Q9TVY5 protein_id:CAA19535.2 /QuerySize=947</td><td>gi|6904581|ref|Os03g45930|</td></tr>
<tr><td>gi|55246734|gb|EAA03128.2|</td><td>lcl|Y48A6B.11b CE34514 WBGene00012972 status:Partially_confirmed SW:Q7YSJ2 protein_id:CAE11312.1 /QuerySize=949</td><td>gi|6904582|ref|Os03g45940|</td></tr>
<tr><td>gi|55246738|gb|EAL42144.1|</td><td>lcl|Y48A6B.13 CE19195 WBGene00012973 status:Partially_confirmed SW:Q9XXC7 protein_id:CAA19534.1 /QuerySize=396</td><td>gi|6904583|ref|Os03g45950|</td></tr>
<tr><td>gi|55246750|gb|EAL42148.1|</td><td>lcl|Y48A6B.2 CE19185 WBGene00012963 status:Partially_confirmed SW:Q9XXD6 protein_id:CAA19524.1 /QuerySize=182</td><td>gi|6904587|ref|Os03g45990|</td></tr>
<tr><td>gi|55246751|gb|EAL42149.1|</td><td>lcl|Y48A6B.4 CE19187 WBGene00012965 status:Confirmed SW:Q9XXD3 protein_id:CAA19528.1 /QuerySize=47</td><td>gi|6904588|ref|Os03g45990|</td></tr>
<tr><td>gi|55246759|gb|EAL42153.1|</td><td>lcl|Y48A6B.8 CE19191 WBGene00012969 status:Partially_confirmed SW:Q9XXC9 protein_id:CAA19532.1 /QuerySize=551</td><td>gi|6904589|ref|Os03g46000|</td></tr>
<tr><td>gi|55246760|gb|EAA03092.3|</td><td>lcl|Y48B6A.6b CE32971 WBGene00012980 status:Partially_confirmed SW:Q8I0Z7 protein_id:CAD59175.1 /QuerySize=533</td><td>gi|6904590|ref|Os03g46010|</td></tr>
<tr><td>gi|55246767|gb|EAL42157.1|</td><td>lcl|Y48C3A.1 CE22133 WBGene00012985 status:Predicted SW:Q9NAG8 protein_id:CAB55099.1 /QuerySize=115</td><td>gi|6904591|ref|Os03g46020|</td></tr>
<tr><td>gi|55246769|gb|EAL42158.1|</td><td>lcl|Y48C3A.5a CE32754 WBGene00012989 status:Partially_confirmed SW:Q8I0Z6 protein_id:CAD57712.1 /QuerySize=510</td><td>gi|6904592|ref|Os03g46030|</td></tr>
<tr><td>gi|55246774|gb|EAL42159.1|</td><td>lcl|Y48C3A.5b CE32755 WBGene00012989 status:Partially_confirmed SW:Q8I0Z5 protein_id:CAD57713.1 /QuerySize=593</td><td>gi|6904594|ref|Os03g46050|</td></tr>
<tr><td>gi|55246775|gb|EAA03068.2|</td><td>lcl|Y48C3A.8 CE22141 WBGene00012990 status:Partially_confirmed SW:Q9NAG3 protein_id:CAB55107.1 /QuerySize=291</td><td>gi|6904604|ref|Os03g46110|</td></tr>
<tr><td>gi|55246781|gb|EAA45699.2|</td><td>lcl|Y48D7A.2a CE24387 WBGene00001461 locus:flp-18 status:Partially_confirmed SW:Q9N4V0 protein_id:AAF60678.1 /QuerySiz</td><td>gi|6904606|ref|Os03g46130|</td></tr>
<tr><td>gi|55246791|gb|EAA03040.3|</td><td>lcl|Y48D7A.2b CE33736 WBGene00001461 locus:flp-18 status:Partially_confirmed SW:Q9N4V0 protein_id:AAO91690.1 /QuerySiz</td><td>gi|6904607|ref|Os03g46140|</td></tr>
<tr><td>gi|55246809|gb|EAL42170.1|</td><td>lcl|Y48E1B.11 CE14872 WBGene00013009 status:Predicted SW:O18201 protein_id:CAB07695.1 /QuerySize=190</td><td>gi|6904610|ref|Os03g46160|</td></tr>
<tr><td>gi|55246813|gb|EAA02972.3|</td><td>lcl|Y48E1B.12 CE14874 WBGene00000804 locus:csc-1 status:Confirmed SW:O45952 protein_id:CAB07696.1 /QuerySize=249</td><td>gi|6904616|ref|Os03g46210|</td></tr>
<tr><td>gi|55246815|gb|EAA02965.2|</td><td>lcl|Y48E1B.15 CE36674 WBGene00013012 status:Predicted SW:Q7JKA0 protein_id:CAE54925.2 /QuerySize=158</td><td>gi|6904620|ref|Os03g46240|</td></tr>
<tr><td>gi|55246816|gb|EAA02967.2|</td><td>lcl|Y48E1B.4 CE14858 WBGene00013003 status:Partially_confirmed SW:O18198 protein_id:CAB07690.1 /QuerySize=611</td><td>gi|6904625|ref|Os03g46290|</td></tr>
<tr><td>gi|55246818|gb|EAA02962.2|</td><td>lcl|Y48E1B.6 CE14862 WBGene00013005 status:Partially_confirmed SW:O18200 protein_id:CAB07692.1 /QuerySize=89</td><td>gi|6904628|ref|Os03g46320|</td></tr>
<tr><td>gi|55246826|gb|EAA02922.2|</td><td>lcl|Y48E1C.1a CE25310 WBGene00013014 status:Partially_confirmed SW:Q9TW55 protein_id:CAB61035.1 /QuerySize=447</td><td>gi|6904639|ref|Os03g46420|</td></tr>
<tr><td>gi|55246828|gb|EAL42175.1|</td><td>lcl|Y48E1C.1c CE32975 WBGene00013014 status:Confirmed SW:Q8I0S7 protein_id:CAD59169.1 /QuerySize=196</td><td>gi|6904640|ref|Os03g46430|</td></tr>
<tr><td>gi|55246872|gb|EAA02799.2|</td><td>lcl|Y48G10A.2 CE25313 WBGene00013019 status:Partially_confirmed SW:Q9U284 protein_id:CAB63371.1 /QuerySize=375</td><td>gi|6904652|ref|Os03g46530|</td></tr>
<tr><td>gi|55246873|gb|EAL42195.1|</td><td>lcl|Y48G10A.4 CE25315 WBGene00013021 status:Partially_confirmed SW:Q9U282 protein_id:CAB63373.1 /QuerySize=778</td><td>gi|6904653|ref|Os03g46540|</td></tr>
<tr><td>gi|55246886|gb|EAA02782.2|</td><td>lcl|Y48G10A.6 CE35092 WBGene00013023 status:Predicted SW:Q7YWQ6 protein_id:CAE18001.1 /QuerySize=91</td><td>gi|6904657|ref|Os03g46580|</td></tr>
<tr><td>gi|55246894|gb|EAL42202.1|</td><td>lcl|Y48G1A.2 CE26998 WBGene00021658 status:Confirmed SW:Q9N3Q6 protein_id:AAF60685.2 /QuerySize=137</td><td>gi|6904667|ref|Os03g46670|</td></tr>
<tr><td>gi|55246908|gb|EAL42207.1|</td><td>lcl|Y48G1BL.1 CE27784 WBGene00021662 status:Predicted SW:Q9N3Q1 protein_id:AAF60694.2 /QuerySize=318</td><td>gi|6904668|ref|Os03g46680|</td></tr>
<tr><td>gi|55246911|gb|EAL42210.1|</td><td>lcl|Y48G1BL.5 CE22179 WBGene00021664 status:Predicted SW:Q9N3P8 protein_id:AAF60688.1 /QuerySize=162</td><td>gi|6904669|ref|Os03g46690|</td></tr>
<tr><td>gi|55246912|gb|EAL42211.1|</td><td>lcl|Y48G1BL.6 CE25318 WBGene00021665 status:Predicted SW:Q9N3P7 protein_id:AAF60687.1 /QuerySize=91</td><td>gi|6904673|ref|Os03g46730|</td></tr>
<tr><td>gi|55246914|gb|EAL42213.1|</td><td>lcl|Y48G1BM.5 CE26117 WBGene00021670 status:Partially_confirmed SW:Q965V1 protein_id:AAK68497.1 /QuerySize=994</td><td>gi|6904678|ref|Os03g46760|</td></tr>
<tr><td>gi|55246924|gb|EAA02698.2|</td><td>lcl|Y48G1BM.7 CE26115 WBGene00021672 status:Predicted SW:Q965U9 protein_id:AAK68499.1 /QuerySize=111</td><td>gi|6904681|ref|Os03g46780|</td></tr>
<tr><td>gi|55246930|gb|EAL42218.1|</td><td>lcl|Y48G1BM.8 CE26114 WBGene00021673 status:Predicted SW:Q965U7 protein_id:AAK68500.1 /QuerySize=320</td><td>gi|6904685|ref|Os03g46820|</td></tr>
<tr><td>gi|55246934|gb|EAA02666.3|</td><td>lcl|Y48G1BR.1 CE37432 WBGene00021675 status:Partially_confirmed SW:Q9N3P6 protein_id:AAF60695.2 /QuerySize=256</td><td>gi|6904687|ref|Os03g46840|</td></tr>
<tr><td>gi|55246941|gb|EAL42226.1|</td><td>lcl|Y48G1C.1 CE30019 WBGene00021676 status:Partially_confirmed SW:Q9N3P1 protein_id:AAK29893.2 /QuerySize=454</td><td>gi|6904688|ref|Os03g46850|</td></tr>
<tr><td>gi|55246943|gb|EAA02656.2|</td><td>lcl|Y48G1C.10 CE32264 WBGene00021683 status:Predicted SW:Q8IU07 protein_id:AAN60504.1 /QuerySize=353</td><td>gi|6904690|ref|Os03g46870|</td></tr>
<tr><td>gi|55246949|gb|EAL42230.1|</td><td>lcl|Y48G1C.11 CE32265 WBGene00021684 status:Partially_confirmed SW:Q8IU06 protein_id:AAN60505.1 /QuerySize=359</td><td>gi|6904693|ref|Os03g46900|</td></tr>
<tr><td>gi|55246951|gb|EAL42232.1|</td><td>lcl|Y48G1C.7 CE27266 WBGene00021680 status:Partially_confirmed SW:Q9BL75 protein_id:AAK29896.1 /QuerySize=623</td><td>gi|6904695|ref|Os03g46920|</td></tr>
<tr><td>gi|55246953|gb|EAA02625.3|</td><td>lcl|Y48G1C.8 CE36240 WBGene00021681 status:Partially_confirmed SW:Q9N3N9 protein_id:AAK29890.2 /QuerySize=828</td><td>gi|6904696|ref|Os03g46930|</td></tr>
<tr><td>gi|55246954|gb|EAA02626.2|</td><td>lcl|Y48G8AL.12 CE27271 WBGene00021690 status:Confirmed SW:Q9BL10 protein_id:AAK29900.1 /QuerySize=78</td><td>gi|6904697|ref|Os03g46940|</td></tr>
<tr><td>gi|55246955|gb|EAA02628.2|</td><td>lcl|Y48G8AL.7 CE27275 WBGene00021687 status:Confirmed SW:Q9BL18 protein_id:AAK29909.1 /QuerySize=191</td><td>gi|6904706|ref|Os03g47030|</td></tr>
<tr><td>gi|55246956|gb|EAA02627.2|</td><td>lcl|Y48G8AL.9 CE27276 WBGene00021688 status:Confirmed SW:Q9BL21 protein_id:AAK29910.1 /QuerySize=295</td><td>gi|6904708|ref|Os03g47050|</td></tr>
<tr><td>gi|55246968|gb|EAA02611.3|</td><td>lcl|Y48G8AR.3 CE22202 WBGene00021694 status:Partially_confirmed SW:Q9N3N5 protein_id:AAF60706.1 /QuerySize=112</td><td>gi|6904709|ref|Os03g47060|</td></tr>
<tr><td>gi|55246970|gb|EAL42238.1|</td><td>lcl|Y48G9A.11 CE25324 WBGene00021704 status:Predicted SW:Q9N3M8 protein_id:AAK29913.1 /QuerySize=356</td><td>gi|6904713|ref|Os03g47090|</td></tr>
<tr><td>gi|55246997|gb|EAL42249.1|</td><td>lcl|Y48G9A.12 CE25323 WBGene00021705 status:Predicted SW:Q9N3M4 protein_id:AAK29912.1 /QuerySize=254</td><td>gi|6904714|ref|Os03g47100|</td></tr>
<tr><td>gi|55246998|gb|EAL42250.1|</td><td>lcl|Y48G9A.2 CE25331 WBGene00021696 status:Predicted SW:Q9N3M3 protein_id:AAK29919.1 /QuerySize=149</td><td>gi|6904715|ref|Os03g47110|</td></tr>
<tr><td>gi|55246999|gb|EAA45809.2|</td><td>lcl|Y48G9A.6 CE35094 WBGene00021700 status:Partially_confirmed SW:Q9N3M7 protein_id:AAK29916.2 /QuerySize=288</td><td>gi|6904717|ref|Os03g47130|</td></tr>
<tr><td>gi|55247002|gb|EAL42253.1|</td><td>lcl|Y48G9A.7 CE22767 WBGene00021701 status:Partially_confirmed SW:Q9N3M9 protein_id:AAK29915.1 /QuerySize=76</td><td>gi|6904719|ref|Os03g47150|</td></tr>
<tr><td>gi|55247005|gb|EAL42255.1|</td><td>lcl|Y49E10.10 CE32980 WBGene00013033 status:Partially_confirmed SW:Q9XTT6 protein_id:CAB11562.2 /QuerySize=425</td><td>gi|6904720|ref|Os03g47160|</td></tr>
<tr><td>gi|55247011|gb|EAA02518.2|</td><td>lcl|Y49E10.21 CE28707 WBGene00013040 status:Partially_confirmed SW:Q9XTU2 protein_id:CAB11555.2 /QuerySize=387</td><td>gi|6904721|ref|Os03g47170|</td></tr>
<tr><td>gi|55247015|gb|EAL42258.1|</td><td>lcl|Y49F6A.5 CE24394 WBGene00021713 status:Predicted SW:Q9N3L6 protein_id:AAF60726.1 /QuerySize=416</td><td>gi|6904722|ref|Os03g47180|</td></tr>
<tr><td>gi|55247029|gb|EAL42263.1|</td><td>lcl|Y49F6B.5 CE25339 WBGene00021717 status:Predicted SW:Q9N4U8 protein_id:AAF60732.1 /QuerySize=193</td><td>gi|6904723|ref|Os03g47190|</td></tr>
<tr><td>gi|55247037|gb|EAL42268.1|</td><td>lcl|Y49F6B.6 CE22258 WBGene00021718 status:Predicted SW:Q9N4U9 protein_id:AAF60727.1 /QuerySize=76</td><td>gi|6904724|ref|Os03g47200|</td></tr>
<tr><td>gi|55247039|gb|EAA02477.2|</td><td>lcl|Y49F6C.2 CE22266 WBGene00021724 status:Partially_confirmed SW:Q9TYI9 protein_id:AAD12863.1 /QuerySize=148</td><td>gi|6904725|ref|Os03g47210|</td></tr>
<tr><td>gi|55247046|gb|EAL42271.1|</td><td>lcl|Y49F6C.7 CE22271 WBGene00021729 status:Predicted SW:Q9TYJ4 protein_id:AAD12867.1 /QuerySize=241</td><td>gi|6904726|ref|Os03g47220|</td></tr>
<tr><td>gi|55247060|gb|EAA02444.2|</td><td>lcl|Y49F6C.8 CE22272 WBGene00021730 status:Partially_confirmed SW:Q9TYJ3 protein_id:AAD12860.1 /QuerySize=163</td><td>gi|6904728|ref|Os03g47240|</td></tr>
<tr><td>gi|55247081|gb|EAL42290.1|</td><td>lcl|Y4C6A.4 CE31738 WBGene00021154 status:Partially_confirmed SW:Q8MXU0 protein_id:AAM97975.1 /QuerySize=98</td><td>gi|6904733|ref|Os03g47290|</td></tr>
<tr><td>gi|55247091|gb|EAA02411.2|</td><td>lcl|Y4C6B.1 CE21305 WBGene00021155 status:Partially_confirmed SW:Q9UB01 protein_id:AAD14764.1 /QuerySize=442</td><td>gi|6904737|ref|Os03g47330|</td></tr>
<tr><td>gi|55247118|gb|EAL42306.1|</td><td>lcl|Y50D4A.1 CE27637 WBGene00021735 status:Confirmed SW:Q95Y57 protein_id:AAK68502.1 /QuerySize=821</td><td>gi|6904738|ref|Os03g47340|</td></tr>
<tr><td>gi|55247138|gb|EAL42317.1|</td><td>lcl|Y50D4A.2 CE30026 WBGene00021736 status:Confirmed SW:Q8WTM8 protein_id:AAK68503.2 /QuerySize=192</td><td>gi|6904739|ref|Os03g47350|</td></tr>
<tr><td>gi|55247146|gb|EAA02319.2|</td><td>lcl|Y50D4A.5 CE36961 WBGene00021739 status:Confirmed SW:Q7KX42 protein_id:AAQ23125.2 /QuerySize=58</td><td>gi|6904740|ref|Os03g47360|</td></tr>
<tr><td>gi|55247147|gb|EAL42323.1|</td><td>lcl|Y50D4B.1 CE26132 WBGene00021740 status:Predicted SW:Q9N4S8 protein_id:AAF59497.2 /QuerySize=199</td><td>gi|6904741|ref|Os03g47370|</td></tr>
<tr><td>gi|55247152|gb|EAA02309.2|</td><td>lcl|Y50D4C.5 CE27640 WBGene00021749 status:Partially_confirmed SW:Q965Q6 protein_id:AAK68510.1 /QuerySize=540</td><td>gi|6904743|ref|Os03g47390|</td></tr>
<tr><td>gi|55247159|gb|EAL42330.1|</td><td>lcl|Y50D4C.6 CE31120 WBGene00021750 status:Partially_confirmed SW:Q8IAA1 protein_id:AAN73848.1 /QuerySize=184</td><td>gi|6904744|ref|Os03g47400|</td></tr>
<tr><td>gi|55247169|gb|EAA02266.2|</td><td>lcl|Y50D7A.5 CE26142 WBGene00021755 status:Partially_confirmed SW:Q965T5 protein_id:AAK68512.1 /QuerySize=176</td><td>gi|6904747|ref|Os03g47430|</td></tr>
<tr><td>gi|55247170|gb|EAL42337.1|</td><td>lcl|Y50D7A.8 CE30029 WBGene00021756 status:Partially_confirmed SW:Q9N3K5 protein_id:AAF59643.3 /QuerySize=372</td><td>gi|6904748|ref|Os03g47440|</td></tr>
<tr><td>gi|55247174|gb|EAA45870.2|</td><td>lcl|Y50E8A.1 CE22303 WBGene00013046 status:Partially_confirmed SW:Q9NAF6 protein_id:CAB55044.1 /QuerySize=86</td><td>gi|6904749|ref|Os03g47450|</td></tr>
<tr><td>gi|55247189|gb|EAL42343.1|</td><td>lcl|Y50E8A.10 CE22317 WBGene00013053 status:Partially_confirmed SW:Q9NAE8 protein_id:CAB55058.1 /QuerySize=209</td><td>gi|6904752|ref|Os03g47480|</td></tr>
<tr><td>gi|55247200|gb|EAA02223.2|</td><td>lcl|Y50E8A.11 CE22318 WBGene00013054 status:Partially_confirmed SW:Q9NAE7 protein_id:CAB55059.1 /QuerySize=221</td><td>gi|6904753|ref|Os03g47490|</td></tr>
<tr><td>gi|55247206|gb|EAA02212.2|</td><td>lcl|Y50E8A.12 CE22319 WBGene00013055 status:Partially_confirmed SW:Q9NAE6 protein_id:CAB55060.1 /QuerySize=183</td><td>gi|6904756|ref|Os03g47510|</td></tr>
<tr><td>gi|55247212|gb|EAA45875.2|</td><td>lcl|Y50E8A.15 CE22313 WBGene00013057 status:Predicted SW:Q9NAF0 protein_id:CAB55054.1 /QuerySize=203</td><td>gi|6904757|ref|Os03g47520|</td></tr>
<tr><td>gi|55247223|gb|EAA45878.2|</td><td>lcl|Y50E8A.8 CE22312 WBGene00013051 status:Predicted SW:Q9NAF1 protein_id:CAB55053.1 /QuerySize=225</td><td>gi|6904759|ref|Os03g47540|</td></tr>
<tr><td>gi|55247227|gb|EAL42360.1|</td><td>lcl|Y50F7A.2 CE22322 WBGene00021760 status:Predicted SW:Q9TYM5 protein_id:AAD12831.1 /QuerySize=108</td><td>gi|6904770|ref|Os03g47630|</td></tr>
<tr><td>gi|55247234|gb|EAA02169.2|</td><td>lcl|Y51A2A.4 CE25360 WBGene00013060 status:Partially_confirmed SW:Q9U269 protein_id:CAB63378.1 /QuerySize=120</td><td>gi|6904773|ref|Os03g47660|</td></tr>
<tr><td>gi|55247255|gb|EAL42371.1|</td><td>lcl|Y51A2A.5 CE20276 WBGene00013061 status:Predicted SW:Q9U273 protein_id:CAB63374.1 /QuerySize=635</td><td>gi|6904783|ref|Os03g47750|</td></tr>
<tr><td>gi|55247271|gb|EAL42379.1|</td><td>lcl|Y51A2B.1 CE16655 WBGene00013065 status:Partially_confirmed SW:O45956 protein_id:CAA16392.1 /QuerySize=98</td><td>gi|6904793|ref|Os03g47850|</td></tr>
<tr><td>gi|55247275|gb|EAL42382.1|</td><td>lcl|Y51A2B.8 CE35103 WBGene00013071 status:Predicted SW:Q7YWN8 protein_id:CAE18020.1 /QuerySize=56</td><td>gi|6904800|ref|Os03g47920|</td></tr>
<tr><td>gi|55247289|gb|EAA02086.2|</td><td>lcl|Y51A2D.14 CE19210 WBGene00013081 status:Confirmed SW:Q9XXR2 protein_id:CAA16401.1 /QuerySize=54</td><td>gi|6904811|ref|Os03g48020|</td></tr>
</table>

gi|55247290|gb|EAL42386.1|	lcl|Y51A2D.20 CE32532 WBGene00013084 status:Partially_confirmed SW:Q8I4C8 protein_id:CAD56613.1 /QuerySize=138	gi|6904815|ref|Os03g48070|
gi|55247293|gb|EAL42389.1|	lcl|Y51A2D.7a CE19203 WBGene00013075 status:Partially_confirmed SW:Q7K7P3 protein_id:CAA16406.1 /QuerySize=680	gi|6904821|ref|Os03g48130|
gi|55247300|gb|EAL42394.1|	lcl|Y51A2D.7b CE24410 WBGene00013075 status:Partially_confirmed SW:Q9U267 protein_id:CAA16410.2 /QuerySize=860	gi|6904823|ref|Os03g48150|
gi|55247301|gb|EAL42395.1|	lcl|Y51B11A.1 CE22326 WBGene00021761 reverse transcriptase status:Predicted SW:Q9N4S7 protein_id:AAF60743.1 /QuerySiz	gi|6904824|ref|Os03g48160|
gi|55247302|gb|EAL42396.1|	lcl|Y51B9A.5 CE19215 WBGene00013087 status:Partially_confirmed SW:Q9XXC2 protein_id:CAA19540.1 /QuerySize=90	gi|6904827|ref|Os03g48190|
gi|55247313|gb|EAL42400.1|	lcl|Y51B9A.7 CE19217 WBGene00013089 status:Predicted SW:Q9XXC5 protein_id:CAA19537.1 /QuerySize=111	gi|6904829|ref|Os03g48210|
gi|55247334|gb|EAA45921.2|	lcl|Y51F10.10 CE31380 WBGene00021771 status:Confirmed SW:Q8WTK0 protein_id:AAL35728.2 /QuerySize=337	gi|6904833|ref|Os03g48240|
gi|55247335|gb|EAA01987.2|	lcl|Y51F10.3 CE32268 WBGene00021764 status:Partially_confirmed SW:Q8WTJ9 protein_id:AAL35730.2 /QuerySize=337	gi|6904837|ref|Os03g48290|
gi|55247348|gb|EAL42409.1|	lcl|Y51F10.6 CE30036 WBGene00021767 status:Partially_confirmed SW:Q8WTK3 protein_id:AAL35733.1 /QuerySize=220	gi|6904851|ref|Os03g48400|
gi|55247349|gb|EAL42410.1|	lcl|Y51F10.7 CE30321 WBGene00021768 status:Confirmed SW:Q8TA47 protein_id:AAL77192.1 /QuerySize=189	gi|6904852|ref|Os03g48410|
gi|55247350|gb|EAA01951.2|	lcl|Y51F10.8 CE30038 WBGene00021769 status:Predicted SW:Q8WTJ7 protein_id:AAL35735.1 /QuerySize=204	gi|6904853|ref|Os03g48420|
gi|55247363|gb|EAL42415.1|	lcl|Y51F10.9 CE32270 WBGene00021770 status:Predicted SW:Q8WTJ5 protein_id:AAL35736.2 /QuerySize=94	gi|6904855|ref|Os03g48440|
gi|55247366|gb|EAA45933.2|	lcl|Y51H1A.6 CE20288 WBGene00013096 Zinc finger	gi|6904858|ref|Os03g48470|
gi|55247368|gb|EAA45934.2|	lcl|Y51H4A.1 CE35667 WBGene00013098 status:Partially_confirmed SW:Q9NAE5 protein_id:CAB61134.2 /QuerySize=214	gi|6904866|ref|Os03g48550|
gi|55247373|gb|EAA01922.3|	lcl|Y51H4A.10 CE28708 WBGene00013105 status:Confirmed SW:Q9NAD9 protein_id:CAB61144.2 /QuerySize=61	gi|6904868|ref|Os03g48570|
gi|55247378|gb|EAA01916.2|	lcl|Y51H4A.15 CE22340 WBGene00013109 status:Partially_confirmed SW:Q9NAD8 protein_id:CAB61147.1 /QuerySize=225	gi|6904869|ref|Os03g48580|
gi|55247391|gb|EAL42425.1|	lcl|Y51H4A.16 CE22341 WBGene00013110 status:Predicted SW:Q9NAD7 protein_id:CAB61148.1 /QuerySize=136	gi|6904878|ref|Os03g48640|
gi|55247395|gb|EAA01900.3|	lcl|Y51H4A.2 CE25364 WBGene00013099 status:Predicted SW:Q9U265 protein_id:CAB63380.1 /QuerySize=190	gi|6904879|ref|Os03g48650|
gi|55247413|gb|EAA01868.2|	lcl|Y51H4A.20 CE22345 WBGene00013114 status:Predicted SW:Q9NAD3 protein_id:CAB61152.1 /QuerySize=110	gi|6904884|ref|Os03g48700|
gi|55247424|gb|EAL42436.1|	lcl|Y51H4A.21 CE25365 WBGene00013115 status:Predicted SW:Q9U260 protein_id:CAB63385.1 /QuerySize=131	gi|6904887|ref|Os03g48720|
gi|55247427|gb|EAL42439.1|	lcl|Y51H4A.22 CE35669 WBGene00013116 status:Partially_confirmed SW:Q9NAD2 protein_id:CAB61154.2 /QuerySize=283	gi|6904888|ref|Os03g48730|
gi|55247431|gb|EAL42441.1|	lcl|Y51H4A.23 CE25366 WBGene00013117 status:Predicted SW:Q9U259 protein_id:CAB63386.1 /QuerySize=257	gi|6904895|ref|Os03g48800|
gi|55247437|gb|EAA01835.2|	lcl|Y51H4A.24 CE25367 WBGene00013118 status:Partially_confirmed SW:Q9U258 protein_id:CAB63387.1 /QuerySize=485	gi|6904897|ref|Os03g48820|
gi|56067561|gb|AAV70248.1|	lcl|Y51H4A.26 CE28709 WBGene00013120 status:Partially_confirmed SW:Q95Q19 protein_id:CAC70119.1 /QuerySize=107	gi|6904901|ref|Os03g48860|
gi|56067563|gb|AAV70249.1|	lcl|Y51H4A.28 CE35197 WBGene00000709 locus:col-136 cuticular collagen status:Predicted SW:Q7YTG0 protein_id:CAE45096.1	gi|6904904|ref|Os03g48870|
gi|56067565|gb|AAV70250.1|	lcl|Y51H4A.6 CE25370 WBGene00013102 status:Predicted SW:Q9U264 protein_id:CAB63381.1 /QuerySize=76	gi|6904914|ref|Os03g48960|
gi|56067567|gb|AAV70251.1|	lcl|Y51H4A.8 CE36242 WBGene00013104 status:Confirmed SW:Q9NAE1 protein_id:CAB61142.2 /QuerySize=186	gi|6904919|ref|Os03g48980|
gi|56067569|gb|AAV70252.1|	lcl|Y51H7BR.3 CE25372 WBGene00021775 status:Partially_confirmed SW:Q9N3J8 protein_id:AAF60746.1 /QuerySize=162	gi|6904922|ref|Os03g49010|
gi|56067571|gb|AAV70253.1|	lcl|Y51H7BR.4 CE36038 WBGene00021776 status:Partially_confirmed SW:Q9N3J9 protein_id:AAF60747.2 /QuerySize=143	gi|6904923|ref|Os03g49020|
gi|56067573|gb|AAV70254.1|	lcl|Y51H7BR.5 CE25374 WBGene00021777 status:Predicted SW:Q9N3K0 protein_id:AAF60748.1 /QuerySize=141	gi|6904928|ref|Os03g49070|
gi|56067575|gb|AAV70255.1|	lcl|Y51H7C.1 CE22358 WBGene00021779 status:Partially_confirmed SW:Q9N3I0 protein_id:AAK39336.1 /QuerySize=448	gi|6904929|ref|Os03g49080|
gi|56067577|gb|AAV70256.1|	lcl|Y51H7C.10 CE36039 WBGene00021788 status:Partially_confirmed SW:Q95XY5 protein_id:AAK39341.2 /QuerySize=237	gi|6904938|ref|Os03g49160|
gi|56067579|gb|AAV70257.1|	lcl|Y51H7C.13 CE36040 WBGene00021791 status:Partially_confirmed SW:Q9N3I1 protein_id:AAK39331.2 /QuerySize=344	gi|6904946|ref|Os03g49240|
gi|56067581|gb|AAV70258.1|	lcl|Y51H7C.3 CE25385 WBGene00021781 status:Confirmed SW:Q9N3I5 protein_id:AAK39334.1 /QuerySize=141	gi|6904949|ref|Os03g49270|
gi|56067583|gb|AAV70259.1|	lcl|Y51H7C.5 CE32079 WBGene00021783 status:Partially_confirmed SW:Q95XY7 protein_id:AAK39344.2 /QuerySize=338	gi|6904950|ref|Os03g49280|
gi|56067585|gb|AAV70260.1|	lcl|Y51H7C.8 CE22362 WBGene00021786 status:Predicted SW:Q9N3J5 protein_id:AAK39332.1 /QuerySize=72	gi|6904951|ref|Os03g49290|
gi|56067587|gb|AAV70261.1|	lcl|Y52B11A.1 CE34228 WBGene00013121 status:Partially_confirmed SW:Q9XWF5 protein_id:CAA21717.2 /QuerySize=179	gi|6904952|ref|Os03g49300|
gi|56067589|gb|AAV70262.1|	lcl|Y52B11B.1 CE19220 WBGene00013130 status:Partially_confirmed SW:Q9XWM7 protein_id:CAA21626.1 /QuerySize=92	gi|6904953|ref|Os03g49310|
gi|56067591|gb|AAV70263.1|	lcl|Y53C10A.10 CE22378 WBGene00013138 status:Predicted SW:Q9XW47 protein_id:CAA22144.1 /QuerySize=627	gi|6904954|ref|Os03g49320|
gi|56067593|gb|AAV70264.1|	lcl|Y53C10A.2 CE22370 WBGene00013133 status:Partially_confirmed SW:Q9XW55 protein_id:CAA22136.1 /QuerySize=139	gi|6904958|ref|Os03g49360|
gi|56067595|gb|AAV70265.1|	lcl|Y53C10A.6 CE22374 WBGene00013137 status:Partially_confirmed SW:Q9XW51 protein_id:CAA22140.1 /QuerySize=344	gi|6904959|ref|Os03g49370|
gi|56067597|gb|AAV70266.1|	lcl|Y53C12A.6 CE14892 WBGene00013141 status:Partially_confirmed SW:O18208 protein_id:CAB16483.1 /QuerySize=130	gi|6904962|ref|Os03g49390|
gi|56067599|gb|AAV70267.1|	lcl|Y53C12A.7 CE35104 WBGene00013142 status:Predicted SW:Q7YWN2 protein_id:CAE18042.1 /QuerySize=134	gi|6904964|ref|Os03g49410|
gi|56067601|gb|AAV70268.1|	lcl|Y53C12B.5b CE32983 WBGene00003100 locus:mab-3 status:Partially_confirmed SW:Q8I0Z0 protein_id:CAD59170.1 /QuerySi	gi|6904970|ref|Os03g49450|
gi|56067603|gb|AAV70269.1|	lcl|Y53C12B.7 CE35105 WBGene00013146 status:Predicted SW:Q7YWN6 protein_id:CAE18037.1 /QuerySize=133	gi|6904972|ref|Os03g49470|
gi|56067605|gb|AAV70270.1|	lcl|Y53F4A.2 CE22382 WBGene00013148 status:Partially_confirmed SW:Q9NAC9 protein_id:CAB54462.1 /QuerySize=87	gi|6904979|ref|Os03g49530|
gi|56067607|gb|AAV70271.1|	lcl|Y53F4B.10 CE22398 WBGene00013157 status:Partially_confirmed SW:Q9NAC2 protein_id:CAB61079.1 /QuerySize=471	gi|6904982|ref|Os03g49560|
gi|56067609|gb|AAV70272.1|	lcl|Y53F4B.11 CE22400 WBGene00013158 status:Partially_confirmed SW:Q9NAC0 protein_id:CAB61081.1 /QuerySize=254	gi|6904986|ref|Os03g49590|
gi|56067611|gb|AAV70273.1|	lcl|Y53F4B.14 CE25395 WBGene00013161 status:Confirmed SW:Q9NAA4 protein_id:CAB70105.1 /QuerySize=90	gi|6904995|ref|Os03g49650|
gi|56067613|gb|AAV70274.1|	lcl|Y53F4B.16 CE24423 WBGene00013162 status:Predicted SW:Q9NAB8 protein_id:CAB61086.1 /QuerySize=304	gi|6904997|ref|Os03g49670|
gi|56067615|gb|AAV70275.1|	lcl|Y53F4B.17 CE25397 WBGene00013163 status:Predicted SW:Q9NAA2 protein_id:CAB70107.1 /QuerySize=340	gi|6904998|ref|Os03g49680|
gi|56067617|gb|AAV70276.1|	lcl|Y53F4B.23 CE35676 WBGene00013169 status:Partially_confirmed SW:Q9NA97 protein_id:CAB70111.2 /QuerySize=165	gi|6904999|ref|Os03g49690|
gi|56067619|gb|AAV70277.1|	lcl|Y53F4B.26 CE25406 WBGene00013172 status:Predicted SW:Q9N671 protein_id:CAB70113.2 /QuerySize=152	gi|6905004|ref|Os03g49740|
gi|56067621|gb|AAV70278.1|	lcl|Y53F4B.27 CE37435 WBGene00013173 status:Partially_confirmed SW:Q9NA93 protein_id:CAB61101.3 /QuerySize=574	gi|6905006|ref|Os03g49760|
gi|56067623|gb|AAV70279.1|	lcl|Y53F4B.36 CE35677 WBGene00013175 status:Partially_confirmed SW:Q9NA95 protein_id:CAB61110.3 /QuerySize=297	gi|6905008|ref|Os03g49780|
gi|56067625|gb|AAV70280.1|	lcl|Y53F4B.6 CE36415 WBGene00013153 status:Partially_confirmed SW:Q9NAC3 protein_id:CAB61075.2 /QuerySize=108	gi|6905011|ref|Os03g49810|
gi|56067627|gb|AAV70281.1|	lcl|Y53F4B.7 CE25406 WBGene00013154 status:Partially_confirmed SW:Q9N671 protein_id:CAB70102.1 /QuerySize=152	gi|6905012|ref|Os03g49820|
gi|56067629|gb|AAV70282.1|	lcl|Y53G8AL.1 CE32534 WBGene00021799 status:Partially_confirmed SW:Q9N3H4 protein_id:AAF59529.4 /QuerySize=538	gi|6905013|ref|Os03g49830|
gi|56067631|gb|AAV70283.1|	lcl|Y53G8AL.3 CE26164 WBGene00021801 status:Predicted SW:Q95XY2 protein_id:AAK84614.1 /QuerySize=252	gi|6905014|ref|Os03g49840|
gi|56067633|gb|AAV70284.1|	lcl|Y53G8AM.2 CE25417 WBGene00021803 status:Predicted SW:Q9N3G6 protein_id:AAK29929.1 /QuerySize=73	gi|6905015|ref|Os03g49850|
gi|56067635|gb|AAV70285.1|	lcl|Y53G8AM.6 CE25414 WBGene00021806 status:Predicted SW:Q9N3G9 protein_id:AAK29925.1 /QuerySize=183	gi|6905016|ref|Os03g49860|
gi|56067637|gb|AAV70286.1|	lcl|Y53G8AM.7 CE25413 WBGene00021807 status:Partially_confirmed SW:Q9N3G8 protein_id:AAK29924.1 /QuerySize=252	gi|6905019|ref|Os03g49890|
gi|56067639|gb|AAV70287.1|	lcl|Y53G8AR.1 CE25426 WBGene00021809 status:Partially_confirmed SW:Q9N3F5 protein_id:AAF59547.1 /QuerySize=272	gi|6905022|ref|Os03g49920|
gi|56067641|gb|AAV70288.1|	lcl|Y53G8AR.7b CE34126 WBGene00021814 status:Partially_confirmed SW:Q7YZM9 protein_id:AAP40516.1 /QuerySize=73	gi|6905029|ref|Os03g49950|
gi|56067643|gb|AAV70289.1|	lcl|Y53G8B.3 CE30041 WBGene00021819 status:Partially_confirmed /QuerySize=321	gi|6905030|ref|Os03g49970|
gi|56067645|gb|AAV70290.1|	lcl|Y53H1A.1 CE25431 WBGene00013177 status:Partially_confirmed SW:Q9U252 protein_id:CAB63392.1 /QuerySize=613	gi|6905033|ref|Os03g50000|
gi|56067647|gb|AAV70291.1|	lcl|Y53H1A.4 CE36041 WBGene00002119 locus:ins-36 status:Predicted SW:Q7K6X2 protein_id:CAE46684.2 /QuerySize=103	gi|6905040|ref|Os03g50050|
gi|56067649|gb|AAV70292.1|	lcl|Y53H1B.2 CE24431 WBGene00013181 status:Partially_confirmed SW:Q9TVV4 protein_id:CAB60416.1 /QuerySize=222	gi|6905041|ref|Os03g50060|
gi|56067651|gb|AAV70293.1|	lcl|Y53H1B.4 CE22435 WBGene00013182 status:Predicted SW:Q9U249 protein_id:CAB60412.1 /QuerySize=180	gi|6905053|ref|Os03g50170|
gi|56067653|gb|AAV70294.1|	lcl|Y53H1B.5 CE24432 WBGene00013183 status:Predicted SW:Q9U248 protein_id:CAB60413.1 /QuerySize=87	gi|6905055|ref|Os03g50190|
gi|56067655|gb|AAV70295.1|	lcl|Y54C5A.1 CE19626 WBGene00021822 status:Predicted SW:Q9TZE5 protein_id:AAC68723.1 /QuerySize=52	gi|6905056|ref|Os03g50200|
gi|56067657|gb|AAV70296.1|	lcl|Y54E10A.1 CE24438 WBGene00021824 status:Partially_confirmed SW:Q9N3E1 protein_id:AAF60760.1 /QuerySize=295	gi|6905060|ref|Os03g50240|
gi|56067659|gb|AAV70297.1|	lcl|Y54E10A.12 CE24441 WBGene00021832 status:Partially_confirmed SW:Q9N3E8 protein_id:AAF60766.1 /QuerySize=547	gi|6905062|ref|Os03g50260|
gi|5834922|ref|NP_008079.1|	lcl|Y54E10A.13 CE24442 WBGene00021833 status:Predicted SW:Q9N3E7 protein_id:AAF60767.1 /QuerySize=165	gi|6905073|ref|Os03g50360|
gi|7362608|emb|CAB40379.2|	lcl|Y54E10A.15a CE24444 WBGene00000413 locus:cdt-1 status:Partially_confirmed SW:Q9N3E4 protein_id:AAF60769.1 /QueryS	gi|6905074|ref|Os03g50370|
gi|7432566|pir||	lcl|Y54E10A.15b CE22447 WBGene00000413 locus:cdt-1 status:Partially_confirmed SW:Q86S68 protein_id:AAO21412.2 /QueryS	gi|6905078|ref|Os03g50400|
	lcl|Y54E10A.16a CE24445 WBGene00021834 status:Partially_confirmed SW:Q9N3E2 protein_id:AAF60770.1 /QuerySize=427	gi|6905084|ref|Os03g50460|
	lcl|Y54E10A.16b CE33235 WBGene00021834 status:Confirmed SW:Q86S67 protein_id:AAO21413.1 /QuerySize=137	gi|6905085|ref|Os03g50470|
	lcl|Y54E10A.2 CE34407 WBGene00021825 status:Partially_confirmed SW:Q9N3E3 protein_id:AAF60761.3 /QuerySize=522	gi|6905096|ref|Os03g50570|
	lcl|Y54E10BL.1 CE34409 WBGene00021837 status:Partially_confirmed SW:Q7Z147 protein_id:AAP68913.1 /QuerySize=90	gi|6905097|ref|Os03g50580|
	lcl|Y54E10BL.3 CE25435 WBGene00021838 status:Partially_confirmed SW:Q9N3D8 protein_id:AAF60778.1 /QuerySize=209	gi|6905098|ref|Os03g50590|
	lcl|Y54E2A.10 CE20309 WBGene00013195 status:Partially_confirmed SW:Q9XWI3 protein_id:CAA21684.1 /QuerySize=305	gi|6905099|ref|Os03g50600|
	lcl|Y54E2A.3 CE20302 WBGene00006381 locus:tac-1 status:Confirmed SW:Q9XWJ0 protein_id:CAA21677.1 /QuerySize=260	gi|6905100|ref|Os03g50610|
	lcl|Y54E2A.4 CE22440 WBGene00013189 status:Partially_confirmed SW:Q9XWI1 protein_id:CAA21686.1 /QuerySize=177	gi|6905102|ref|Os03g50630|
	lcl|Y54E2A.5 CE20304 WBGene00013190 status:Partially_confirmed SW:Q9XWI9 protein_id:CAA21678.1 /QuerySize=237	gi|6905104|ref|Os03g50650|
	lcl|Y54E2A.8 CE20307 WBGene00013193 status:Partially_confirmed SW:Q9XWI7 protein_id:CAA21680.1 /QuerySize=485	gi|6905107|ref|Os03g50680|
	lcl|Y54E5A.2 CE20313 WBGene00013198 status:Predicted SW:Q9XWK0 protein_id:CAA21660.1 /QuerySize=197	gi|6905109|ref|Os03g50700|
	lcl|Y54E5A.3 CE24455 WBGene00013199 status:Partially_confirmed SW:Q9U246 protein_id:CAA21661.2 /QuerySize=404	gi|6905110|ref|Os03g50710|
	lcl|Y54E5A.4 CE20315 WBGene00003790 locus:npp-4 status:Partially_confirmed SW:Q9XWK3 protein_id:CAA21657.1 /QuerySiz	gi|6905111|ref|Os03g50720|
	lcl|Y54E5A.8a CE20319 WBGene00013203 status:Confirmed SW:Q9XTC0 protein_id:CAA21663.1 /QuerySize=263	gi|6905115|ref|Os03g50750|
	lcl|Y54E5A.8b CE20320 WBGene00013203 status:Partially_confirmed SW:Q9XTG5 protein_id:CAA21664.1 /QuerySize=243	gi|6905117|ref|Os03g50770|
	lcl|Y54F10AL.2b CE27003 WBGene00001339 locus:est-1 status:Confirmed SW:Q9BL69 protein_id:AAK27881.1 /QuerySize=461	gi|6905119|ref|Os03g50790|
	lcl|Y54F10AM.6 CE26176 WBGene00021850 status:Partially_confirmed SW:Q9BL06 protein_id:AAK29935.1 /QuerySize=254	gi|6905133|ref|Os03g50910|
	lcl|Y54F10AR.2 CE35107 WBGene00021855 status:Partially_confirmed SW:Q9N3C2 protein_id:AAF59560.2 /QuerySize=309	gi|6905135|ref|Os03g50930|
	lcl|Y54F10BM.12 CE37438 WBGene00021866 status:Confirmed SW:Q95XK6 protein_id:AAK68891.2 /QuerySize=273	gi|6905137|ref|Os03g50950|
	lcl|Y54F10BM.9 CE29121 WBGene00021863 status:Partially_confirmed SW:Q95XL0 protein_id:AAK68900.2 /QuerySize=1142	gi|6905141|ref|Os03g50990|
	lcl|Y54G11A.1 CE22472 WBGene00013212 status:Predicted SW:Q9XVZ7 protein_id:CAA22455.1 /QuerySize=625	gi|6905150|ref|Os03g51070|
	lcl|Y54G11A.14 CE30712 WBGene00013221 status:Predicted SW:Q8MYL8 protein_id:CAD31697.1 /QuerySize=450	gi|6905153|ref|Os03g51100|
	lcl|Y54G2A.10a CE27482 WBGene00021875 status:Partially_confirmed SW:Q95XX7 protein_id:AAK68523.1 /QuerySize=188	gi|6905156|ref|Os03g51130|
	lcl|Y54G2A.10b CE33883 WBGene00021875 status:Confirmed SW:Q86FN9 protein_id:AAP13739.1 /QuerySize=63	gi|6905160|ref|Os03g51170|
	lcl|Y54G2A.11a CE27483 WBGene00021876 status:Confirmed SW:Q95XX8 protein_id:AAK68524.1 /QuerySize=482	gi|6905162|ref|Os03g51190|
	lcl|Y54G2A.11b CE33884 WBGene00021876 status:Confirmed SW:Q86FN8 protein_id:AAP13740.1 /QuerySize=74	gi|6905170|ref|Os03g51260|
	lcl|Y54G2A.13 CE32535 WBGene00021878 status:Partially_confirmed SW:Q9N3C0 protein_id:AAF59585.2 /QuerySize=610	gi|6905179|ref|Os03g51340|
	lcl|Y54G2A.15 CE25465 WBGene00021880 status:Partially_confirmed SW:Q9N3B8 protein_id:AAF59583.1 /QuerySize=157	gi|6905182|ref|Os03g51370|
	lcl|Y54G2A.16 CE34412 WBGene00021881 status:Partially_confirmed SW:Q9N3B7 protein_id:AAF59582.2 /QuerySize=167	gi|6905183|ref|Os03g51380|
	lcl|Y54G2A.19 CE25461 WBGene00021884 status:Partially_confirmed SW:Q9N3B4 protein_id:AAF59579.1 /QuerySize=107	gi|6905184|ref|Os03g51390|
	lcl|Y54G2A.20 CE25460 WBGene00021885 status:Predicted SW:Q9N3B3 protein_id:AAF59578.1 /QuerySize=608	gi|6905185|ref|Os03g51400|
	lcl|Y54G2A.22 CE34413 WBGene00021887 status:Partially_confirmed SW:Q9N3B1 protein_id:AAF59576.2 /QuerySize=340	gi|6905186|ref|Os03g51410|
	lcl|Y54G2A.26a CE30050 WBGene00021890 status:Confirmed SW:Q9N3A3 protein_id:AAF59568.4 /QuerySize=611	gi|6905187|ref|Os03g51420|
	lcl|Y54G2A.26b CE33888 WBGene00021890 status:Confirmed SW:Q86FN6 protein_id:AAP13744.1 /QuerySize=549	gi|6905190|ref|Os03g51450|
	lcl|Y54G2A.27 CE25446 WBGene00021891 status:Predicted SW:Q9N399 protein_id:AAF59564.1 /QuerySize=229	gi|6905192|ref|Os03g51470|
	lcl|Y54G2A.28 CE36897 WBGene00021892 status:Predicted SW:Q9N398 protein_id:AAF59563.3 /QuerySize=366	gi|6905193|ref|Os03g51470|
	lcl|Y54G2A.29 CE34416 WBGene00021893 status:Partially_confirmed SW:Q9N397 protein_id:AAF59562.4 /QuerySize=536	gi|6905197|ref|Os03g51490|
	lcl|Y54G2A.3 CE27491 WBGene00021869 status:Confirmed SW:Q95XW9 protein_id:AAK68528.1 /QuerySize=166	gi|6905198|ref|Os03g51500|
	lcl|Y54G2A.32 CE29123 WBGene00021894 status:Confirmed SW:Q95XX6 protein_id:AAL00869.1 /QuerySize=187	gi|6905202|ref|Os03g51530|
	lcl|Y54G2A.36 CE34417 WBGene00021898 status:Partially_confirmed SW:Q7Z073 protein_id:AAP68916.1 /QuerySize=128	gi|6905206|ref|Os03g51570|
	lcl|Y54G2A.37 CE37293 WBGene00023484 status:Partially_confirmed SW:Q688Z5 protein_id:AAU05552.1 /QuerySize=281	gi|6905208|ref|Os03g51590|
	lcl|Y54G2A.5a CE30054 WBGene00021871 status:Partially_confirmed SW:Q8WTL0 protein_id:AAK68529.2 /QuerySize=249	gi|6905212|ref|Os03g51630|
	lcl|Y54G2A.5b CE33889 WBGene00021871 status:Confirmed SW:Q86FN5 protein_id:AAP13745.1 /QuerySize=232	gi|6905215|ref|Os03g51660|
	lcl|Y54G9A.1 CE19229 WBGene00013205 status:Predicted SW:Q9XWH1 protein_id:CAA21697.1 /QuerySize=99	gi|6905218|ref|Os03g51680|
	lcl|Y54G9A.2 CE19230 WBGene00013206 status:Predicted SW:Q9XWH2 protein_id:CAA21696.1 /QuerySize=124	gi|6905222|ref|Os03g51720|
	lcl|Y54G9A.5 CE20321 WBGene00013208 status:Partially_confirmed SW:Q9XWG8 protein_id:CAA21700.1 /QuerySize=257	gi|6905225|ref|Os03g51750|
	lcl|Y54G9A.7 CE26735 WBGene00013210 status:Confirmed SW:Q9BHB3 protein_id:CAC35867.1 /QuerySize=244	gi|6905239|ref|Os03g51890|
	lcl|Y54G9A.9 CE32761 WBGene00013211 status:Confirmed SW:Q8I4C6 protein_id:CAD57711.1 /QuerySize=93	gi|6905244|ref|Os03g51940|
	lcl|Y55B1AL.1 CE25471 WBGene00021903 status:Partially_confirmed SW:Q9N391 protein_id:AAF59589.1 /QuerySize=223	gi|6905249|ref|Os03g51980|
	lcl|Y55B1AR.2a CE34129 WBGene00021906 status:Partially_confirmed SW:Q9N385 protein_id:AAK68901.2 /QuerySize=456	gi|6905253|ref|Os03g52020|
	lcl|Y55B1AR.2b CE35109 WBGene00021906 status:Partially_confirmed SW:Q7YZG7 protein_id:AAP86610.1 /QuerySize=588	gi|6905254|ref|Os03g52030|
	lcl|Y55B1AR.3 CE25475 WBGene00021907 status:Predicted SW:Q9N383 protein_id:AAF60787.1 /QuerySize=300	gi|6905256|ref|Os03g52050|
	lcl|Y55B1AR.4 CE22490 WBGene00021908 status:Partially_confirmed SW:Q9N382 protein_id:AAF60784.1 /QuerySize=337	gi|6905257|ref|Os03g52060|
	lcl|Y55B1BL.1 CE22511 WBGene00021909 status:Partially_confirmed SW:Q9N381 protein_id:AAF59593.1 /QuerySize=117	gi|6905266|ref|Os03g52140|
	lcl|Y55B1BR.1 CE22506 WBGene00021911 status:Predicted SW:Q95XW6 protein_id:AAK85501.1 /QuerySize=543	gi|6905277|ref|Os03g52220|
	lcl|Y55B1BR.2 CE25478 WBGene00021912 status:Partially_confirmed SW:Q95XW7 protein_id:AAK85502.1 /QuerySize=740	gi|6905278|ref|Os03g52240|
	lcl|Y55D5A.1a CE27294 WBGene00021915 status:Confirmed SW:Q968Z0 protein_id:AAK39621.1 /QuerySize=285	gi|6905281|ref|Os03g52270|
	lcl|Y55D5A.1b CE34131 WBGene00021915 status:Confirmed SW:Q7Z2B3 protein_id:AAP40511.1 /QuerySize=162	gi|6905282|ref|Os03g52280|

lcl|Y55D5A.2 CE22518 WBGene00021916 status:Predicted SW:Q968Z1 protein_id:AAK29950.2 /QuerySize=99 gi|6905284|ref|Os03g52300|
lcl|Y55D5A.4 CE22516 WBGene00021918 status:Partially_confirmed SW:Q9GUH8 protein_id:AAK29948.2 /QuerySize=199 gi|6905287|ref|Os03g52330|
lcl|Y55F3AM.13 CE22542 WBGene00021930 status:Confirmed SW:Q9N362 protein_id:AAF60790.1 /QuerySize=354 gi|6905289|ref|Os03g52350|
lcl|Y55F3AM.3b CE25485 WBGene00021921 status:Confirmed SW:Q9N367 protein_id:AAF60796.1 /QuerySize=49 gi|6905290|ref|Os03g52360|
lcl|Y55F3AM.5 CE36752 WBGene00021923 status:Predicted SW:Q9N372 protein_id:AAF60804.2 /QuerySize=309 gi|6905291|ref|Os03g52370|
lcl|Y55F3BL.1 CE36753 WBGene00021935 status:Predicted SW:Q9N355 protein_id:AAF60808.2 /QuerySize=419 gi|6905292|ref|Os03g52380|
lcl|Y55F3BR.7 CE22538 WBGene00021944 status:Predicted SW:Q9N349 protein_id:AAF59603.1 /QuerySize=211 gi|6905296|ref|Os03g52420|
lcl|Y55H10B.1 CE22571 WBGene00021954 status:Partially_confirmed SW:Q9N4R8 protein_id:AAF60812.1 /QuerySize=507 gi|6905298|ref|Os03g52440|
lcl|Y55H10B.2 CE22570 WBGene00021955 status:Confirmed SW:Q9N4R9 protein_id:AAF60811.1 /QuerySize=76 gi|6905304|ref|Os03g52500|
lcl|Y56A3A.29b CE28139 WBGene00013241 status:Confirmed SW:Q95Q13 protein_id:CAC42381.1 /QuerySize=95 gi|6905305|ref|Os03g52510|
lcl|Y56A3A.31 CE22596 WBGene00013243 status:Partially_confirmed SW:Q9U230 protein_id:CAB60510.1 /QuerySize=413 gi|6905315|ref|Os03g52610|
lcl|Y56A3A.6 CE22577 WBGene00013227 status:Partially_confirmed SW:Q9U225 protein_id:CAB60515.1 /QuerySize=798 gi|6905324|ref|Os03g52680|
lcl|Y56A3A.7 CE28140 WBGene00013228 status:Partially_confirmed SW:Q9U222 protein_id:CAB60519.2 /QuerySize=457 gi|6905344|ref|Os03g52850|
lcl|Y56A3A.9 CE25498 WBGene00013229 status:Predicted SW:Q9U215 protein_id:CAB60526.1 /QuerySize=373 gi|6905348|ref|Os03g52890|
lcl|Y57A10A.14 CE22617 WBGene00013257 status:Predicted SW:Q9NA82 protein_id:CAB55017.1 /QuerySize=228 gi|6905349|ref|Os03g52900|
lcl|Y57A10A.22 CE25505 WBGene00013262 status:Predicted SW:Q9U212 protein_id:CAB60766.1 /QuerySize=73 gi|6905351|ref|Os03g52920|
lcl|Y57A10A.24 CE25506 WBGene00013264 status:Partially_confirmed SW:Q9U211 protein_id:CAB60767.1 /QuerySize=534 gi|6905352|ref|Os03g52930|
lcl|Y57A10A.27 CE25507 WBGene00013267 status:Partially_confirmed SW:Q9U210 protein_id:CAB60768.1 /QuerySize=388 gi|6905353|ref|Os03g52940|
lcl|Y57A10A.32 CE22612 WBGene00013271 status:Partially_confirmed SW:Q9NA70 protein_id:CAB55037.1 /QuerySize=153 gi|6905355|ref|Os03g52960|
lcl|Y57A10A.9 CE22628 WBGene00013254 status:Confirmed SW:Q9NA77 protein_id:CAB55028.1 /QuerySize=158 gi|6905359|ref|Os03g52990|
lcl|Y57A10B.2 CE20323 WBGene00013274 status:Predicted SW:Q9XWH9 protein_id:CAA21689.1 /QuerySize=178 gi|6905363|ref|Os03g53030|
lcl|Y57A10B.7 CE35689 WBGene00013279 status:Partially_confirmed SW:Q9XWH4 protein_id:CAA21694.2 /QuerySize=271 gi|6905364|ref|Os03g53040|
lcl|Y57A10C.1 CE22632 WBGene00013280 status:Confirmed SW:O62487 protein_id:CAA19553.1 /QuerySize=239 gi|6905380|ref|Os03g53160|
lcl|Y57A10C.11 CE37943 WBGene00044047 status:Predicted /QuerySize=262 gi|6905382|ref|Os03g53180|
lcl|Y57E12AL.2 CE22640 WBGene00021957 status:Predicted SW:Q9N338 protein_id:AAF59615.1 /QuerySize=121 gi|6905385|ref|Os03g53210|
lcl|Y57E12AL.4 CE26192 WBGene00021958 status:Predicted SW:Q95XV5 protein_id:AAK71408.1 /QuerySize=167 gi|6905388|ref|Os03g53240|
lcl|Y57E12AL.6 CE22635 WBGene00021959 status:Confirmed SW:Q9N336 protein_id:AAF59611.1 /QuerySize=130 gi|6905393|ref|Os03g53290|
lcl|Y57E12B.1 CE22642 WBGene00021961 status:Predicted SW:Q95XU9 protein_id:AAK68535.1 /QuerySize=78 gi|6905396|ref|Os03g53320|
lcl|Y57E12B.2 CE24471 WBGene00021962 status:Partially_confirmed SW:Q95XV2 protein_id:AAK68536.1 /QuerySize=31 gi|6905397|ref|Os03g53340|
lcl|Y57G11A.1b CE33893 WBGene00013289 LIM domain containing proteins status:Partially_confirmed SW:Q86CZ7 protein_id:C gi|6905401|ref|Os03g53330|
lcl|Y57G11A.4 CE32991 WBGene00013292 status:Partially_confirmed SW:O18222 protein_id:CAB16497.2 /QuerySize=232 gi|6905404|ref|Os03g53370|
lcl|Y57G11B.1 CE16665 WBGene00013294 status:Partially_confirmed SW:O18223 protein_id:CAB16502.1 /QuerySize=418 gi|6905405|ref|Os03g53380|
lcl|Y57G11B.5 CE14924 WBGene00013297 status:Partially_confirmed SW:O18224 protein_id:CAB16499.1 /QuerySize=225 gi|6905409|ref|Os03g53420|
lcl|Y57G11B.6 CE35114 WBGene00013298 status:Predicted SW:Q7YWP1 protein_id:CAE18017.1 /QuerySize=129 gi|6905411|ref|Os03g53440|
lcl|Y57G11C.14 CE14952 WBGene00013310 status:Partially_confirmed SW:O18238 protein_id:CAB16515.1 /QuerySize=242 gi|6905412|ref|Os03g53450|
lcl|Y57G11C.18 CE14960 WBGene00013313 status:Predicted SW:O18242 protein_id:CAB16519.1 /QuerySize=343 gi|6905413|ref|Os03g53460|
lcl|Y57G11C.20 CE14964 WBGene00013315 status:Predicted SW:O18244 protein_id:CAB16521.1 /QuerySize=649 gi|6905414|ref|Os03g53470|
lcl|Y57G11C.21 CE14966 WBGene00013316 status:Partially_confirmed SW:O18245 protein_id:CAB16522.1 /QuerySize=236 gi|6905415|ref|Os03g53480|
lcl|Y57G11C.32 CE35692 WBGene00013322 status:Partially_confirmed SW:Q9U205 protein_id:CAB54455.2 /QuerySize=512 gi|6905419|ref|Os03g53520|
lcl|Y57G11C.40 CE35117 WBGene00013329 status:Predicted SW:Q7YTI7 protein_id:CAE18023.1 /QuerySize=181 gi|6905437|ref|Os03g53680|
lcl|Y57G11C.41 CE35118 WBGene00013330 status:Predicted SW:Q7YTI6 protein_id:CAE18024.1 /QuerySize=181 gi|6905454|ref|Os03g53830|
lcl|Y57G11C.42 CE35119 WBGene00013331 status:Predicted SW:Q7YTI5 protein_id:CAE18025.1 /QuerySize=421 gi|6905455|ref|Os03g53840|
lcl|Y57G11C.45 CE35122 WBGene00013334 status:Predicted SW:Q7YTI2 protein_id:CAE18028.1 /QuerySize=159 gi|6905460|ref|Os03g53870|
lcl|Y57G11C.46 CE35123 WBGene00013335 status:Predicted SW:Q7YTI1 protein_id:CAE18029.1 /QuerySize=377 gi|6905467|ref|Os03g53930|
lcl|Y57G11C.48 CE35125 WBGene00013337 status:Predicted SW:Q7YTH9 protein_id:CAE18031.1 /QuerySize=133 gi|6905468|ref|Os03g53940|
lcl|Y57G11C.8 CE14940 WBGene00013306 status:Partially_confirmed SW:O18234 protein_id:CAB16510.1 /QuerySize=313 gi|6905475|ref|Os03g54010|
lcl|Y57G11C.9c CE28142 WBGene00013307 status:Confirmed SW:Q95Q12 protein_id:CAC42379.1 /QuerySize=77 gi|6905480|ref|Os03g54060|
lcl|Y57G7A.1 CE19630 WBGene00021965 status:Confirmed SW:O76628 protein_id:AAC26296.1 /QuerySize=137 gi|6905481|ref|Os03g54070|
lcl|Y57G7A.11 CE29893 WBGene00021974 status:Confirmed SW:Q95X26 protein_id:AAL27258.1 /QuerySize=152 gi|6905485|ref|Os03g54110|
lcl|Y57G7A.2 CE19631 WBGene00021966 status:Partially_confirmed SW:O76627 protein_id:AAC26297.1 /QuerySize=177 gi|6905486|ref|Os03g54120|
lcl|Y57G7A.5 CE19634 WBGene00021968 status:Partially_confirmed SW:O76621 protein_id:AAC26294.1 /QuerySize=244 gi|6905488|ref|Os03g54140|
lcl|Y57G7A.8 CE19637 WBGene00021971 status:Predicted SW:O76626 protein_id:AAC26301.1 /QuerySize=354 gi|6905492|ref|Os03g54180|
lcl|Y58A7A.2 CE26195 WBGene00021976 status:Predicted SW:Q966A7 protein_id:AAK84616.1 /QuerySize=109 gi|6905493|ref|Os03g54190|
lcl|Y58A7A.3 CE26196 WBGene00021977 status:Partially_confirmed SW:Q966A6 protein_id:AAK84617.1 /QuerySize=883 gi|6905494|ref|Os03g54200|
lcl|Y58A7A.4 CE26197 WBGene00021978 status:Partially_confirmed SW:Q966A5 protein_id:AAK84618.1 /QuerySize=904 gi|6905495|ref|Os03g54210|
lcl|Y58A7A.5 CE26198 WBGene00021979 status:Predicted SW:Q966A4 protein_id:AAK84619.1 /QuerySize=349 gi|6905496|ref|Os03g54220|
lcl|Y59A8A.3 CE26201 WBGene00013340 status:Confirmed SW:Q9GRZ9 protein_id:CAC14400.1 /QuerySize=676 gi|6905506|ref|Os03g54800|
lcl|Y59A8B.12 CE24505 WBGene00013349 status:Partially_confirmed SW:Q9NEQ5 protein_id:CAB60960.1 /QuerySize=118 gi|6905508|ref|Os03g54820|
lcl|Y59A8B.19 CE26209 WBGene00013351 status:Partially_confirmed SW:Q9GRY4 protein_id:CAC14415.1 /QuerySize=390 gi|6905509|ref|Os03g54830|
lcl|Y59A8B.20 CE36755 WBGene00013352 status:Partially_confirmed SW:Q9NER7 protein_id:CAB60948.2 /QuerySize=152 gi|6905510|ref|Os03g54840|
lcl|Y59A8B.24 CE24491 WBGene00013356 status:Predicted SW:Q9NES3 protein_id:CAB60942.1 /QuerySize=173 gi|6905526|ref|Os03g54970|
lcl|Y59C2A.3 CE37171 WBGene00021986 status:Partially_confirmed /QuerySize=782 gi|6905535|ref|Os03g55060|
lcl|Y59E1B.1 CE35808 WBGene00021990 status:Partially_confirmed SW:Q9N331 protein_id:AAF60817.2 /QuerySize=105 gi|6905553|ref|Os03g55190|
lcl|Y59E1B.2 CE24511 WBGene00021991 status:Predicted SW:Q9N332 protein_id:AAF60818.1 /QuerySize=99 gi|6905564|ref|Os03g55300|
lcl|Y59E9AL.3a CE22659 WBGene00021993 status:Partially_confirmed SW:Q9N2L4 protein_id:AAF60821.1 /QuerySize=381 gi|6905578|ref|Os03g55430|
lcl|Y59E9AL.3b CE22659 WBGene00021993 status:Partially_confirmed SW:Q9N2L4 protein_id:AAF60820.1 /QuerySize=381 gi|6905579|ref|Os03g55440|
lcl|Y59E9AL.3c CE22659 WBGene00021993 status:Partially_confirmed SW:Q9N2L4 protein_id:AAM97978.1 /QuerySize=381 gi|6905581|ref|Os03g55460|
lcl|Y59E9AL.5 CE22661 WBGene00021995 status:Predicted SW:Q9N329 protein_id:AAF60819.1 /QuerySize=99 gi|6905583|ref|Os03g55480|
lcl|Y59E9AL.6 CE25514 WBGene00021996 status:Partially_confirmed SW:Q9N328 protein_id:AAF60823.1 /QuerySize=214 gi|6905585|ref|Os03g55500|
lcl|Y59E9AR.2 CE22665 WBGene00021998 status:Predicted SW:Q9N2L8 protein_id:AAF60827.1 /QuerySize=383 gi|6905586|ref|Os03g55510|
lcl|Y59E9AR.8 CE22665 WBGene00022003 status:Partially_confirmed SW:Q9N2L8 protein_id:AAF60825.1 /QuerySize=383 gi|6905587|ref|Os03g55520|
lcl|Y59H11AM.4 CE25518 WBGene00022008 status:Partially_confirmed SW:Q95XU8 protein_id:AAK68542.1 /QuerySize=185 gi|6905604|ref|Os03g55650|
lcl|Y59H11AR.1 CE26556 WBGene00022009 status:Predicted SW:Q9N320 protein_id:AAF59623.1 /QuerySize=37 gi|6905609|ref|Os03g55700|
lcl|Y59H11AR.3 CE26554 WBGene00022011 status:Predicted SW:Q9N322 protein_id:AAF59621.1 /QuerySize=128 gi|6905615|ref|Os03g55740|
lcl|Y5H2B.3 CE21320 WBGene00021165 status:Partially_confirmed SW:Q9N4Q9 protein_id:AAF59633.1 /QuerySize=182 gi|6905616|ref|Os03g55750|
lcl|Y60A3A.21 CE32027 WBGene00013366 status:Confirmed SW:Q8I4C1 protein_id:CAD54173.1 /QuerySize=97 gi|6905618|ref|Os03g55770|
lcl|Y60A3A.23 CE35127 WBGene00013367 status:Predicted SW:Q7YWP2 protein_id:CAE18016.1 /QuerySize=228 gi|6905619|ref|Os03g55780|
lcl|Y60A3A.24 CE37444 WBGene00023519 status:Partially_confirmed SW:Q5ZEQ0 protein_id:CAH60778.1 /QuerySize=402 gi|6905622|ref|Os03g55810|
lcl|Y60A9.1 CE24534 WBGene00013368 status:Predicted SW:Q9U1Y4 protein_id:CAB60408.1 /QuerySize=268 gi|6905630|ref|Os03g55880|
lcl|Y60A9A.1 CE35128 WBGene00013371 status:Predicted SW:Q7YWN7 protein_id:CAE18032.1 /QuerySize=109 gi|6905632|ref|Os03g55900|
lcl|Y61A9LA.11 CE29129 WBGene00022022 status:Confirmed SW:Q95XU5 protein_id:AAL00870.1 /QuerySize=177 gi|6905633|ref|Os03g55910|
lcl|Y61A9LA.3a CE27412 WBGene00022015 status:Partially_confirmed SW:Q9N313 protein_id:AAF60843.2 /QuerySize=743 gi|6905636|ref|Os03g55940|
lcl|Y61A9LA.3b CE36964 WBGene00022015 status:Confirmed SW:Q9N310 protein_id:AAF60844.3 /QuerySize=525 gi|6905640|ref|Os03g55980|
lcl|Y61A9LA.3c CE31122 WBGene00022015 status:Confirmed SW:Q8MXS5 protein_id:AAM48560.1 /QuerySize=726 gi|6905643|ref|Os03g56010|
lcl|Y61A9LA.4 CE29131 WBGene00022016 status:Predicted SW:Q9N315 protein_id:AAF60842.2 /QuerySize=199 gi|6905645|ref|Os03g56030|
lcl|Y61A9LA.7 CE22680 WBGene00022018 status:Partially_confirmed SW:Q9N316 protein_id:AAF60838.1 /QuerySize=110 gi|6905650|ref|Os03g56080|
lcl|Y61A9LA.9 CE22673 WBGene00022020 status:Partially_confirmed SW:Q9N312 protein_id:AAF60836.1 /QuerySize=263 gi|6905652|ref|Os03g56100|
lcl|Y62E10A.14 CE22704 WBGene00013380 status:Partially_confirmed SW:Q9U1X3 protein_id:CAB60601.1 /QuerySize=345 gi|6905654|ref|Os03g56120|
lcl|Y62E10A.18 CE35129 WBGene00013384 status:Predicted SW:Q7YTH7 protein_id:CAE18035.1 /QuerySize=126 gi|6905657|ref|Os03g56150|
lcl|Y62E10A.19 CE35130 WBGene00013385 status:Predicted SW:Q7YTH6 protein_id:CAE18036.1 /QuerySize=197 gi|6905658|ref|Os03g56150|
lcl|Y62E10A.3 CE22691 WBGene00013374 status:Predicted SW:Q9U1Y1 protein_id:CAB60593.1 /QuerySize=176 gi|6905659|ref|Os03g56150|
lcl|Y62F5A.9 CE22710 WBGene00013387 status:Confirmed SW:Q8T3A5 protein_id:CAD30454.1 /QuerySize=197 gi|6905664|ref|Os03g56200|
lcl|Y62H9A.11 CE19247 WBGene00013399 status:Predicted SW:Q9XWS5 protein_id:CAA21570.1 /QuerySize=296 gi|6905665|ref|Os03g56210|
lcl|Y62H9A.12 CE19248 WBGene00013400 status:Partially_confirmed SW:Q9XWS8 protein_id:CAA21567.1 /QuerySize=260 gi|6905675|ref|Os03g56290|
lcl|Y62H9A.13 CE19249 WBGene00013401 status:Predicted SW:Q9XWS7 protein_id:CAA21568.1 /QuerySize=348 gi|6905681|ref|Os03g56330|
lcl|Y62H9A.14 CE35695 WBGene00013402 status:Partially_confirmed SW:Q9XTG2 protein_id:CAA21571.2 /QuerySize=274 gi|6905683|ref|Os03g56350|
lcl|Y62H9A.2 CE19238 WBGene00013390 status:Predicted SW:Q9XWT6 protein_id:CAA21559.1 /QuerySize=217 gi|6905692|ref|Os03g56430|
lcl|Y62H9A.3 CE19239 WBGene00013391 status:Partially_confirmed SW:Q9XWT5 protein_id:CAA21560.1 /QuerySize=214 gi|6905693|ref|Os03g56440|
lcl|Y62H9A.4 CE19240 WBGene00013392 status:Partially_confirmed SW:Q9XWT4 protein_id:CAA21561.1 /QuerySize=212 gi|6905694|ref|Os03g56450|
lcl|Y62H9A.5 CE31124 WBGene00013393 status:Confirmed SW:Q9XWS6 protein_id:CAA21569.2 /QuerySize=165 gi|6905698|ref|Os03g56480|
lcl|Y62H9A.6 CE19242 WBGene00013394 status:Confirmed SW:Q9XWT3 protein_id:CAA21562.1 /QuerySize=181 gi|6905699|ref|Os03g56490|
lcl|Y62H9A.7 CE19243 WBGene00013395 status:Predicted SW:Q9XWT2 protein_id:CAA21563.1 /QuerySize=241 gi|6905700|ref|Os03g56500|
lcl|Y62H9A.8 CE19244 WBGene00013396 status:Partially_confirmed SW:Q9XWT1 protein_id:CAA21564.1 /QuerySize=170 gi|6905702|ref|Os03g56520|
lcl|Y62H9A.9 CE19245 WBGene00013397 status:Predicted SW:Q9XWT0 protein_id:CAA21565.1 /QuerySize=369 gi|6905704|ref|Os03g56550|
lcl|Y64G10A.2 CE36246 WBGene00013412 status:Partially_confirmed SW:Q9NA66 protein_id:CAB54467.2 /QuerySize=187 gi|6905710|ref|Os03g56600|
lcl|Y64G10A.3 CE22714 WBGene00013413 status:Partially_confirmed SW:Q9NA65 protein_id:CAB54468.1 /QuerySize=254 gi|6905715|ref|Os03g56650|
lcl|Y64G10A.6 CE22716 WBGene00013415 status:Partially_confirmed SW:Q9NA64 protein_id:CAB54470.1 /QuerySize=846 gi|6905716|ref|Os03g56660|
lcl|Y64G10A.8 CE36247 WBGene00013417 locus:srab-24 status:Predicted SW:Q7YWP3 protein_id:CAE18014.2 /QuerySize=336 gi|6905722|ref|Os03g56710|
lcl|Y64H9A.2 CE29132 WBGene00022024 status:Confirmed SW:Q95Y56 protein_id:AAL00871.1 /QuerySize=101 gi|6905723|ref|Os03g56720|
lcl|Y65A5A.1 CE22718 WBGene00013418 status:Confirmed SW:Q9XTW5 protein_id:CAB07433.1 /QuerySize=123 gi|6905724|ref|Os03g56730|
lcl|Y65A5A.2 CE22719 WBGene00013419 status:Predicted SW:Q9XTW3 protein_id:CAB07435.1 /QuerySize=226 gi|6905726|ref|Os03g56750|
lcl|Y65A5A.3 CE22720 WBGene00013420 status:Predicted SW:Q9XTW4 protein_id:CAB07434.1 /QuerySize=48 gi|6905727|ref|Os03g56760|
lcl|Y65B4A.4 CE27301 WBGene00022028 status:Predicted SW:Q9BL63 protein_id:AAK29957.1 /QuerySize=124 gi|6905728|ref|Os03g56770|
lcl|Y65B4BL.2 CE25536 WBGene00022034 status:Partially_confirmed SW:Q9N303 protein_id:AAF60852.1 /QuerySize=619 gi|6905729|ref|Os03g56780|
lcl|Y65B4BL.3 CE34420 WBGene00022035 status:Partially_confirmed SW:Q9N305 protein_id:AAF60850.2 /QuerySize=347 gi|6905740|ref|Os03g56860|
lcl|Y65B4BL.4 CE22745 WBGene00022036 status:Partially_confirmed SW:Q9N304 protein_id:AAF60851.1 /QuerySize=324 gi|6905741|ref|Os03g56870|
lcl|Y65B4BL.6 CE22747 WBGene00022038 status:Partially_confirmed SW:Q9N301 protein_id:AAF60847.1 /QuerySize=63 gi|6905743|ref|Os03g56890|
lcl|Y65B4BL.7 CE36756 WBGene00022039 status:Partially_confirmed SW:Q95XU4 protein_id:AAK84623.2 /QuerySize=214 gi|6905746|ref|Os03g56920|
lcl|Y66A7A.3 CE20342 WBGene00013423 status:Predicted SW:Q9XWY6 protein_id:CAC35864.1 /QuerySize=95 gi|6905747|ref|Os03g56930|
lcl|Y66A7A.7 CE20344 WBGene00013426 status:Predicted SW:Q9XWY4 protein_id:CAC35861.1 /QuerySize=117 gi|6905748|ref|Os03g56940|
lcl|Y66D12A.1 CE15689 WBGene00013427 status:Predicted SW:Q9BI35 protein_id:CAC35913.1 /QuerySize=236 gi|6905754|ref|Os03g56980|
lcl|Y66D12A.11 CE28793 WBGene00013437 status:Partially_confirmed SW:Q95Q04 protein_id:CAC70133.1 /QuerySize=340 gi|6905756|ref|Os03g57000|
lcl|Y66D12A.16 CE26448 WBGene00013442 status:Partially_confirmed SW:Q95Q01 protein_id:CAC70136.2 /QuerySize=193 gi|6905757|ref|Os03g57010|
lcl|Y66D12A.18 CE26445 WBGene00013444 status:Partially_confirmed SW:Q95PJ9 protein_id:CAC35895.1 /QuerySize=286 gi|6905758|ref|Os03g57020|
lcl|Y66D12A.19 CE28999 WBGene00013445 status:Partially_confirmed SW:Q95PZ6 protein_id:CAC70142.1 /QuerySize=169 gi|6905759|ref|Os03g57030|
lcl|Y66D12A.24 CE31843 WBGene00013448 status:Partially_confirmed SW:Q95PZ5 protein_id:CAC70143.2 /QuerySize=261 gi|6905762|ref|Os03g57050|
lcl|Y66D12A.3 CE28803 WBGene00013429 status:Confirmed SW:Q95Q09 protein_id:CAC70128.1 /QuerySize=313 gi|6905763|ref|Os03g57060|
lcl|Y66D12A.5 CE28805 WBGene00013431 status:Partially_confirmed SW:Q95Q08 protein_id:CAC70129.1 /QuerySize=257 gi|6905778|ref|Os03g57180|
lcl|Y66D12A.6 CE26458 WBGene00013432 status:Partially_confirmed SW:Q9BI39 protein_id:CAC35908.1 /QuerySize=136 gi|6905783|ref|Os03g57230|
lcl|Y66H1A.5 CE22756 WBGene00022047 status:Confirmed SW:Q9TYK0 protein_id:AAD12854.1 /QuerySize=217 gi|6905785|ref|Os03g57250|
lcl|Y67A10A.6 CE24567 WBGene00013455 locus:ssu-2 status:Partially_confirmed SW:Q9U1V3 protein_id:CAB60447.1 /QuerySiz gi|6905797|ref|Os03g57350|
lcl|Y67A6A.1 CE20349 WBGene00013449 status:Partially_confirmed SW:Q9XWJ2 protein_id:CAA21674.1 /QuerySize=123 gi|6905801|ref|Os03g57390|
lcl|Y67D8B.4 CE36344 WBGene00022063 status:Confirmed SW:Q95XT9 protein_id:AAK68549.3 /QuerySize=54 gi|6905806|ref|Os03g57430|
lcl|Y67D8B.5 CE31663 WBGene00022064 status:Partially_confirmed SW:Q95XT6 protein_id:AAL27260.2 /QuerySize=152 gi|6905818|ref|Os03g57540|

lcl|Y67D8C.1 CE22784 WBGene00022065 status:Predicted SW:Q9GUN2 protein_id:AAG23382.1 /QuerySize=77	gi|6905819|ref|Os03g57550|
lcl|Y67D8C.2 CE22783 WBGene00022066 status:Predicted SW:Q9GUN4 protein_id:AAG23381.1 /QuerySize=460	gi|6905821|ref|Os03g57570|
lcl|Y67D8C.3a CE29902 WBGene00022067 transposase status:Partially_confirmed SW:Q9GUN8 protein_id:AAG23379.2 /QueryS	gi|6905822|ref|Os03g57580|
lcl|Y67D8C.3b CE33951 WBGene00022067 transposase status:Partially_confirmed SW:Q86DD2 protein_id:AAP31423.1 /QuerySi	gi|6905823|ref|Os03g57580|
lcl|Y67D8C.4 CE26234 WBGene00022068 status:Predicted SW:Q9GUN9 protein_id:AAG23378.1 /QuerySize=323	gi|6905824|ref|Os03g57580|
lcl|Y67D8C.6 CE22778 WBGene00022070 status:Predicted SW:Q9GUN7 protein_id:AAG23374.1 /QuerySize=264	gi|6905825|ref|Os03g57600|
lcl|Y67D8C.7 CE22777 WBGene00022071 status:Partially_confirmed SW:Q9GUN5 protein_id:AAG23373.1 /QuerySize=192	gi|6905826|ref|Os03g57590|
lcl|Y67D8C.8 CE27506 WBGene00022072 status:Confirmed SW:Q95XP7 protein_id:AAK68552.1 /QuerySize=69	gi|6905828|ref|Os03g57620|
lcl|Y67H2A.10 CE33897 WBGene00013465 status:Partially_confirmed SW:Q86DA8 protein_id:CAD89753.1 /QuerySize=558	gi|6905829|ref|Os03g57630|
lcl|Y67H2A.4b CE32273 WBGene00013462 status:Confirmed SW:Q8I0Y9 protein_id:CAD56257.1 /QuerySize=169	gi|6905831|ref|Os03g57650|
lcl|Y67H2A.5 CE28376 WBGene00013463 status:Confirmed SW:Q95PZ1 protein_id:CAC44306.1 /QuerySize=145	gi|6905836|ref|Os03g57700|
lcl|Y68A4B.3 CE19263 WBGene00013472 status:Partially_confirmed SW:Q9XXB6 protein_id:CAA19556.1 /QuerySize=85	gi|6905837|ref|Os03g57710|
lcl|Y69A2AR.12 CE22796 WBGene00022083 status:Predicted SW:Q95XH9 protein_id:AAK68556.1 /QuerySize=190	gi|6905838|ref|Os03g57720|
lcl|Y69A2AR.13 CE30463 WBGene00022084 status:Predicted SW:Q95XI0 protein_id:AAK68557.2 /QuerySize=207	gi|6905841|ref|Os03g57750|
lcl|Y69A2AR.14 CE26568 WBGene00022085 status:Partially_confirmed SW:Q95XI3 protein_id:AAK68558.1 /QuerySize=555	gi|6905844|ref|Os03g57770|
lcl|Y69A2AR.22 CE22799 WBGene00022093 status:Predicted SW:Q95XI6 protein_id:AAK68567.1 /QuerySize=78	gi|6905848|ref|Os03g57810|
lcl|Y69A2AR.23 CE22795 WBGene00022094 status:Partially_confirmed SW:Q95XI4 protein_id:AAK68568.1 /QuerySize=87	gi|6905850|ref|Os03g57830|
lcl|Y69A2AR.24 CE22794 WBGene00022095 status:Predicted SW:Q95XI2 protein_id:AAK68569.1 /QuerySize=94	gi|6905863|ref|Os03g57930|
lcl|Y69A2AR.25 CE22793 WBGene00022096 status:Predicted SW:Q95XI1 protein_id:AAK68570.1 /QuerySize=337	gi|6905871|ref|Os03g58000|
lcl|Y69A2AR.27 CE22806 WBGene00022098 status:Predicted SW:Q95XH3 protein_id:AAK68572.1 /QuerySize=130	gi|6905885|ref|Os03g58090|
lcl|Y69A2AR.28 CE26562 WBGene00022099 status:Confirmed SW:Q95XH0 protein_id:AAK68573.1 /QuerySize=108	gi|6905894|ref|Os03g58170|
lcl|Y69A2AR.2b CE33952 WBGene00004367 locus:ric-8 status:Confirmed SW:Q86DD4 protein_id:AAP31421.1 /QuerySize=157	gi|6905897|ref|Os03g58180|
lcl|Y69A2AR.3 CE27518 WBGene00022075 status:Confirmed SW:Q95XG6 protein_id:AAK68575.1 /QuerySize=83	gi|6905900|ref|Os03g58210|
lcl|Y69A2AR.31 CE27519 WBGene00022100 status:Partially_confirmed SW:Q95XG5 protein_id:AAK68577.1 /QuerySize=1219	gi|6905901|ref|Os03g58220|
lcl|Y69A2AR.32b CE36369 WBGene00022101 status:Confirmed SW:Q8T871 protein_id:AAM15621.2 /QuerySize=216	gi|6905908|ref|Os03g58280|
lcl|Y69A2AR.8 CE22804 WBGene00022079 status:Partially_confirmed SW:Q95XH4 protein_id:AAK68582.1 /QuerySize=120	gi|6905911|ref|Os03g58310|
lcl|Y69A2AR.9 CE22803 WBGene00022080 status:Predicted SW:Q95XH5 protein_id:AAK68583.1 /QuerySize=300	gi|6905913|ref|Os03g58330|
lcl|Y69E1A.2 CE22809 WBGene00013474 status:Confirmed SW:Q9XW38 protein_id:CAA22257.1 /QuerySize=366	gi|6905917|ref|Os03g58360|
lcl|Y69E1A.8 CE35696 WBGene00013478 status:Partially_confirmed SW:Q9XW35 protein_id:CAA22260.2 /QuerySize=364	gi|6905925|ref|Os03g58440|
lcl|Y69H2.1 CE37446 WBGene00013479 status:Partially_confirmed SW:Q9XTT0 protein_id:CAB11569.2 /QuerySize=343	gi|6905926|ref|Os03g58450|
lcl|Y69H2.7 CE37450 WBGene00013482 status:Partially_confirmed SW:Q9U1U3 protein_id:CAB63404.2 /QuerySize=411	gi|6905927|ref|Os03g58460|
lcl|Y69H2.9 CE22827 WBGene00013484 status:Partially_confirmed SW:Q9U1U1 protein_id:CAB63406.1 /QuerySize=314	gi|6905933|ref|Os03g58500|
lcl|Y6B3B.1 CE20177 WBGene00012387 status:Predicted SW:O18164 protein_id:CAA21727.1 /QuerySize=1829	gi|6905934|ref|Os03g58510|
lcl|Y6B3B.12 CE37580 WBGene00043050 status:Partially_confirmed SW:Q5ZEQ2 protein_id:CAH60798.1 /QuerySize=329	gi|6905939|ref|Os03g58550|
lcl|Y6B3B.3 CE20178 WBGene00012388 status:Predicted SW:Q9XWE7 protein_id:CAA21726.1 /QuerySize=995	gi|6905940|ref|Os03g58560|
lcl|Y6D1A.1 CE21324 WBGene00012395 status:Partially_confirmed SW:Q9U1S8 protein_id:CAB60580.1 /QuerySize=681	gi|6905945|ref|Os03g58610|
lcl|Y6E2A.7 CE16583 WBGene00012400 status:Partially_confirmed SW:O45974 protein_id:CAA15968.1 /QuerySize=363	gi|6905949|ref|Os03g58650|
lcl|Y6E2A.8 CE16584 WBGene00012401 status:Partially_confirmed SW:O45975 protein_id:CAA15969.1 /QuerySize=219	gi|6905952|ref|Os03g58680|
lcl|Y70C5B.1 CE16672 WBGene00013491 status:Predicted SW:O45980 protein_id:CAA16431.1 /QuerySize=170	gi|6905953|ref|Os03g58690|
lcl|Y71A12B.11 CE24598 WBGene00013508 status:Confirmed SW:Q9NEN0 protein_id:CAB82002.1 /QuerySize=286	gi|6905957|ref|Os03g58730|
lcl|Y71A12B.13b CE29068 WBGene00013510 status:Predicted SW:Q95PY6 protein_id:CAC70126.1 /QuerySize=148	gi|6905961|ref|Os03g58770|
lcl|Y71A12B.7 CE26240 WBGene00013504 status:Predicted SW:Q9GRV3 protein_id:CAC14422.1 /QuerySize=913	gi|6905970|ref|Os03g58850|
lcl|Y71F9AL.11 CE22875 WBGene00022116 status:Predicted SW:Q9N4I7 protein_id:AAF35996.1 /QuerySize=196	gi|6905971|ref|Os03g58860|
lcl|Y71F9AL.6 CE22840 WBGene00022111 status:Partially_confirmed SW:Q9N4H8 protein_id:AAF36000.1 /QuerySize=271	gi|6905972|ref|Os03g58870|
lcl|Y71F9AL.7 CE22861 WBGene00022112 status:Partially_confirmed SW:Q9N4H9 protein_id:AAF35999.1 /QuerySize=284	gi|6905981|ref|Os03g58950|
lcl|Y71F9AL.8 CE25560 WBGene00022113 status:Partially_confirmed SW:Q9N4I2 protein_id:AAF36006.1 /QuerySize=332	gi|6905988|ref|Os03g59000|
lcl|Y71F9AL.9 CE22858 WBGene00022114 status:Partially_confirmed SW:Q9N4I3 protein_id:AAF35998.1 /QuerySize=314	gi|6906002|ref|Os03g59130|
lcl|Y71F9AR.3 CE26245 WBGene00022124 status:Partially_confirmed SW:Q95XT1 protein_id:AAK68586.1 /QuerySize=746	gi|6906013|ref|Os03g59230|
lcl|Y71F9B.1 CE24627 WBGene00022125 status:Partially_confirmed SW:Q9N4G1 protein_id:AAF36017.1 /QuerySize=139	gi|6906030|ref|Os03g59380|
lcl|Y71F9B.10a CE28808 WBGene00004946 locus:sop-3 status:Confirmed SW:Q9N4G4 protein_id:AAF36027.2 /QuerySize=1475	gi|6906032|ref|Os03g59400|
lcl|Y71F9B.10b CE29912 WBGene00004946 locus:sop-3 status:Confirmed SW:Q95Y43 protein_id:AAL27266.1 /QuerySize=544	gi|6906043|ref|Os03g59490|
lcl|Y71F9B.13a CE25566 WBGene00022131 status:Confirmed SW:Q9N4F9 protein_id:AAF36024.1 /QuerySize=220	gi|6906050|ref|Os03g59560|
lcl|Y71F9B.13b CE25567 WBGene00022131 status:Confirmed SW:Q9N4G0 protein_id:AAF36025.1 /QuerySize=192	gi|6906058|ref|Os03g59630|
lcl|Y71F9B.13c CE33244 WBGene00022131 status:Confirmed SW:Q8IAB0 protein_id:AAO12395.1 /QuerySize=95	gi|6906076|ref|Os03g59780|
lcl|Y71F9B.14 CE22852 WBGene00022132 status:Partially_confirmed SW:Q9N4F8 protein_id:AAF36026.1 /QuerySize=430	gi|6906078|ref|Os03g59800|
lcl|Y71F9B.15 CE22851 WBGene00022133 status:Predicted SW:Q9N4F7 protein_id:AAF36013.1 /QuerySize=224	gi|6906080|ref|Os03g59820|
lcl|Y71G10AL.1a CE31666 WBGene00022134 status:Partially_confirmed SW:Q8MXS3 protein_id:AAM97980.1 /QuerySize=472	gi|6906081|ref|Os03g59830|
lcl|Y71G10AL.1b CE37585 WBGene00022134 status:Partially_confirmed /QuerySize=698	gi|6906083|ref|Os03g59850|
lcl|Y71G10AR.3 CE36900 WBGene00022137 status:Partially_confirmed SW:Q9N2Y5 protein_id:AAF60868.4 /QuerySize=235	gi|6906089|ref|Os03g59910|
lcl|Y71G12A.2 CE36759 WBGene00022138 status:Partially_confirmed SW:Q9BL51 protein_id:AAK31568.6 /QuerySize=611	gi|6906090|ref|Os03g59920|
lcl|Y71G12B.13 CE26259 WBGene00022153 status:Predicted SW:Q95XN5 protein_id:AAK73912.1 /QuerySize=553	gi|6906091|ref|Os03g59930|
lcl|Y71G12B.2 CE36761 WBGene00022142 status:Predicted SW:Q95XM0 protein_id:AAK73920.2 /QuerySize=257	gi|6906092|ref|Os03g59940|
lcl|Y71G12B.20b CE33246 WBGene00003111 locus:mab-20 status:Confirmed SW:Q86LT8 protein_id:AAO21421.1 /QuerySize=1	gi|6906095|ref|Os03g59970|
lcl|Y71G12B.21 CE22925 WBGene00004168 locus:pqn-88 status:Confirmed SW:Q95XP2 protein_id:AAK73922.1 /QuerySize=198	gi|6906096|ref|Os03g59980|
lcl|Y71G12B.22 CE34425 WBGene00022157 status:Partially_confirmed SW:Q95XP1 protein_id:AAK73923.2 /QuerySize=238	gi|6906097|ref|Os03g59990|
lcl|Y71G12B.26 CE22902 WBGene00022161 status:Partially_confirmed SW:Q95XM7 protein_id:AAK73927.1 /QuerySize=488	gi|6906102|ref|Os03g60040|
lcl|Y71G12B.28 CE28859 WBGene00022163 status:Partially_confirmed SW:Q95XL7 protein_id:AAK84624.1 /QuerySize=49	gi|6906104|ref|Os03g60060|
lcl|Y71G12B.3 CE22909 WBGene00022143 status:Predicted SW:Q95XM1 protein_id:AAK73929.1 /QuerySize=178	gi|6906105|ref|Os03g60070|
lcl|Y71G12B.5 CE36763 WBGene00022145 status:Predicted SW:Q95XM3 protein_id:AAK73931.2 /QuerySize=1098	gi|6906127|ref|Os03g60280|
lcl|Y71G12B.7 CE36764 WBGene00022147 status:Predicted SW:Q95XM6 protein_id:AAK73933.2 /QuerySize=129	gi|6906130|ref|Os03g60310|
lcl|Y71G12B.9a CE29139 WBGene00022149 status:Partially_confirmed SW:Q95XN0 protein_id:AAK73935.2 /QuerySize=516	gi|6906131|ref|Os03g60320|
lcl|Y71G12B.9b CE31387 WBGene00022149 status:Confirmed SW:Q8IU04 protein_id:AAN73851.1 /QuerySize=511	gi|6906142|ref|Os03g60410|
lcl|Y71H2AL.2 CE22931 WBGene00022165 status:Predicted SW:Q9N2Y0 protein_id:AAF60876.1 /QuerySize=239	gi|6906143|ref|Os03g60420|
lcl|Y71H2AM.4 CE22940 WBGene00022169 status:Confirmed SW:Q9BL33 protein_id:AAK29975.1 /QuerySize=139	gi|6906145|ref|Os03g60440|
lcl|Y71H2AM.7 CE26270 WBGene00022172 status:Partially_confirmed SW:Q9BL45 protein_id:AAK29972.1 /QuerySize=325	gi|6906146|ref|Os03g60450|
lcl|Y71H2B.11 CE34427 WBGene00022198 status:Partially_confirmed SW:Q95Y42 protein_id:AAK93868.3 /QuerySize=227	gi|6906148|ref|Os03g60470|
lcl|Y71H2B.4 CE24631 WBGene00022194 status:Confirmed SW:Q9N4F0 protein_id:AAF36033.1 /QuerySize=90	gi|6906150|ref|Os03g60490|
lcl|Y72A10A.1 CE37172 WBGene00022202 status:Partially_confirmed /QuerySize=199	gi|6906169|ref|Os03g60640|
lcl|Y73B3A.1 CE27324 WBGene00022203 status:Partially_confirmed SW:Q95XE2 protein_id:AAK39359.1 /QuerySize=659	gi|6906171|ref|Os03g60660|
lcl|Y73B3A.10 CE37297 WBGene00022212 status:Confirmed SW:Q95XG0 protein_id:AAK39355.2 /QuerySize=95	gi|6906173|ref|Os03g60680|
lcl|Y73B3A.11 CE26582 WBGene00022213 status:Predicted SW:Q95XF8 protein_id:AAK39354.1 /QuerySize=131	gi|6906183|ref|Os03g60760|
lcl|Y73B3A.20 CE33247 WBGene00022221 status:Confirmed SW:Q95XE3 protein_id:AAK39367.3 /QuerySize=72	gi|6906184|ref|Os03g60770|
lcl|Y73B3A.7 CE26579 WBGene00022209 status:Predicted SW:Q95XF5 protein_id:AAK39357.1 /QuerySize=205	gi|6906191|ref|Os03g60830|
lcl|Y73B3A.8 CE26586 WBGene00022210 status:Predicted SW:Q95XF7 protein_id:AAK39356.1 /QuerySize=60	gi|6906194|ref|Os03g60860|
lcl|Y73B3A.9 CE27335 WBGene00022211 status:Predicted SW:Q95XF9 protein_id:AAK39363.1 /QuerySize=315	gi|6906207|ref|Os03g60980|
lcl|Y73B3B.3 CE26596 WBGene00022225 status:Predicted SW:Q95XD9 protein_id:AAK39372.1 /QuerySize=61	gi|6906209|ref|Os03g61000|
lcl|Y73B3B.5 CE28523 WBGene00022227 status:Partially_confirmed SW:Q95XD6 protein_id:AAK39371.2 /QuerySize=87	gi|6906218|ref|Os03g61070|
lcl|Y73B6A.3 CE22960 WBGene00022230 status:Partially_confirmed SW:Q9N4E4 protein_id:AAF36039.1 /QuerySize=126	gi|6906220|ref|Os03g61090|
lcl|Y73B6BL.14 CE26288 WBGene00022237 status:Predicted SW:Q9GUG2 protein_id:AAG23474.1 /QuerySize=99	gi|6906227|ref|Os03g61160|
lcl|Y73B6BL.16 CE26286 WBGene00022239 status:Predicted SW:Q9GUG0 protein_id:AAG23472.1 /QuerySize=270	gi|6906235|ref|Os03g61230|
lcl|Y73B6BL.20 CE26282 WBGene00022241 status:Predicted SW:Q9GUF6 protein_id:AAG23468.1 /QuerySize=120	gi|6906236|ref|Os03g61240|
lcl|Y73B6BL.22 CE26280 WBGene00022243 status:Partially_confirmed SW:Q9GUF4 protein_id:AAG23466.1 /QuerySize=105	gi|6906245|ref|Os03g61300|
lcl|Y73B6BL.23 CE27530 WBGene00022244 status:Partially_confirmed SW:Q95XC9 protein_id:AAK68593.1 /QuerySize=197	gi|6906254|ref|Os03g61380|
lcl|Y73B6BL.27 CE29925 WBGene00022248 status:Partially_confirmed SW:Q9GUE9 protein_id:AAG23461.3 /QuerySize=131	gi|6906257|ref|Os03g61410|
lcl|Y73B6BL.28 CE27532 WBGene00022249 status:Partially_confirmed SW:Q95XC5 protein_id:AAK68594.1 /QuerySize=112	gi|6906258|ref|Os03g61420|
lcl|Y73B6BL.35 CE29929 WBGene00022254 status:Confirmed SW:Q8MXR0 protein_id:AAM44391.1 /QuerySize=88	gi|6906262|ref|Os03g61460|
lcl|Y73B6BL.36 CE29930 WBGene00022255 status:Predicted SW:Q8MXQ9 protein_id:AAM44392.1 /QuerySize=155	gi|6906264|ref|Os03g61480|
lcl|Y73B6BL.5a CE29147 WBGene00022234 status:Partially_confirmed SW:Q8MXR5 protein_id:AAM44395.1 /QuerySize=571	gi|6906267|ref|Os03g61510|
lcl|Y73B6BL.5b CE29148 WBGene00022234 status:Partially_confirmed SW:Q8MXR4 protein_id:AAM44396.1 /QuerySize=526	gi|6906268|ref|Os03g61520|
lcl|Y73B6BL.5c CE31129 WBGene00022234 status:Partially_confirmed SW:Q8IU02 protein_id:AAN63425.1 /QuerySize=391	gi|6906275|ref|Os03g61590|
lcl|Y73B6BL.5d CE31130 WBGene00022234 status:Confirmed SW:Q8IU01 protein_id:AAN63426.1 /QuerySize=104	gi|6906277|ref|Os03g61610|
lcl|Y73B6BL.9b CE31131 WBGene00001853 locus:hil-2 histone H1.2 status:Partially_confirmed /QuerySize=173	gi|6906281|ref|Os03g61650|
lcl|Y73B6BR.1a CE25586 WBGene00004169 locus:pqn-89 status:Confirmed SW:Q9N2X8 protein_id:AAF60877.1 /QuerySize=481	gi|6906294|ref|Os03g61770|
lcl|Y73B6BR.1b CE33954 WBGene00004169 locus:pqn-89 status:Partially_confirmed SW:Q86DD5 protein_id:AAP31420.1 /Query	gi|6906305|ref|Os03g61860|
lcl|Y73C8C.3 CE20359 WBGene00022262 status:Predicted SW:Q9TXW6 protein_id:AAC69345.1 /QuerySize=826	gi|6906310|ref|Os03g61900|
lcl|Y73E7A.2 CE32277 WBGene00022269 status:Partially_confirmed SW:Q9GUM5 protein_id:AAG23388.3 /QuerySize=463	gi|6906332|ref|Os03g62040|
lcl|Y73F8A.10 CE36250 WBGene00013519 status:Partially_confirmed SW:Q9NA60 protein_id:CAB60535.2 /QuerySize=205	gi|6906343|ref|Os03g62130|
lcl|Y73F8A.12 CE25590 WBGene00013521 status:Partially_confirmed SW:Q9NA37 protein_id:CAB70228.1 /QuerySize=129	gi|6906346|ref|Os03g62160|
lcl|Y73F8A.13 CE36251 WBGene00013522 status:Partially_confirmed SW:Q9NA58 protein_id:CAB60538.2 /QuerySize=284	gi|6906352|ref|Os03g62220|
lcl|Y73F8A.14 CE25591 WBGene00013523 status:Partially_confirmed SW:Q9NA36 protein_id:CAB70229.1 /QuerySize=129	gi|6906358|ref|Os03g62280|
lcl|Y73F8A.15 CE22984 WBGene00013524 status:Partially_confirmed SW:Q9NA57 protein_id:CAB60540.1 /QuerySize=122	gi|6906359|ref|Os03g62290|
lcl|Y73F8A.25a CE32999 WBGene00013530 status:Confirmed SW:Q9NA34 protein_id:CAB70231.2 /QuerySize=640	gi|6906360|ref|Os03g62300|
lcl|Y73F8A.25b CE33000 WBGene00013530 status:Partially_confirmed SW:Q8I0Y8 protein_id:CAD59165.1 /QuerySize=323	gi|6906363|ref|Os03g62330|
lcl|Y73F8A.32 CE36252 WBGene00013536 status:Partially_confirmed SW:Q9NA42 protein_id:CAB60566.2 /QuerySize=60	gi|6906365|ref|Os03g62350|
lcl|Y73F8A.33 CE24651 WBGene00013537 status:Predicted SW:Q9NA44 protein_id:CAB60561.1 /QuerySize=874	gi|6906366|ref|Os03g62360|
lcl|Y74C10AR.2 CE30326 WBGene00022282 status:Partially_confirmed SW:Q965S9 protein_id:AAK68602.2 /QuerySize=166	gi|6906369|ref|Os03g62390|
lcl|Y74C9A.1 CE34428 WBGene00022275 status:Partially_confirmed SW:Q9N4D5 protein_id:AAF36048.2 /QuerySize=243	gi|6906375|ref|Os03g62440|
lcl|Y74C9A.2 CE24660 WBGene00022276 status:Confirmed SW:Q9N4D8 protein_id:AAF36049.1 /QuerySize=123	gi|6906385|ref|Os03g62530|
lcl|Y75B12A.2 CE19269 WBGene00013566 status:Partially_confirmed SW:Q9XTE8 protein_id:CAA21758.1 /QuerySize=238	gi|6906386|ref|Os03g62540|
lcl|Y75B12B.1 CE20370 WBGene00013567 status:Partially_confirmed SW:Q9XWB8 protein_id:CAA21764.1 /QuerySize=296	gi|6906387|ref|Os03g62550|
lcl|Y75B12B.11 CE35138 WBGene00013573 status:Predicted SW:Q7YWP9 protein_id:CAE18008.1 /QuerySize=188	gi|6906388|ref|Os03g62560|
lcl|Y75B12B.3 CE20372 WBGene00013568 status:Partially_confirmed SW:Q9XWC0 protein_id:CAA21759.1 /QuerySize=94	gi|6906389|ref|Os03g62570|
lcl|Y75B12B.8 CE17539 WBGene00013570 status:Predicted SW:O62097 protein_id:CAA21763.1 /QuerySize=226	gi|6906425|ref|Os03g62760|
lcl|Y75B7AL.2 CE26303 WBGene00022285 status:Partially_confirmed SW:Q965S7 protein_id:AAK68604.1 /QuerySize=252	gi|6906426|ref|Os03g62770|
lcl|Y75B7AR.1 CE27416 WBGene00022287 status:Partially_confirmed SW:Q9N2X6 protein_id:AAF60880.2 /QuerySize=146	gi|6906433|ref|Os03g62820|
lcl|Y75B7B.1 CE24665 WBGene00022288 status:Predicted SW:Q9N4D3 protein_id:AAF36054.1 /QuerySize=365	gi|6906435|ref|Os03g62840|
lcl|Y75B8A.11 CE23023 WBGene00013548 status:Predicted SW:Q9XW71 protein_id:CAA22099.1 /QuerySize=208	gi|6906440|ref|Os03g62880|
lcl|Y75B8A.13 CE23025 WBGene00013549 status:Partially_confirmed SW:Q9XW69 protein_id:CAA22101.1 /QuerySize=540	gi|6906441|ref|Os03g62890|
lcl|Y75B8A.17 CE23028 WBGene00013552 status:Partially_confirmed SW:Q9XW85 protein_id:CAA22084.1 /QuerySize=180	gi|6906445|ref|Os03g62920|
lcl|Y75B8A.18 CE23029 WBGene00013553 status:Predicted SW:Q9XW84 protein_id:CAA22085.1 /QuerySize=170	gi|6906446|ref|Os03g62930|
lcl|Y75B8A.19 CE23030 WBGene00013554 status:Partially_confirmed SW:Q9XW67 protein_id:CAA22104.1 /QuerySize=696	gi|6906447|ref|Os03g62940|
lcl|Y75B8A.23 CE23034 WBGene00013556 status:Predicted SW:Q9XW64 protein_id:CAA22107.1 /QuerySize=86	gi|6906448|ref|Os03g62950|
lcl|Y75B8A.25 CE23036 WBGene00013558 status:Partially_confirmed SW:Q9XW62 protein_id:CAA22109.1 /QuerySize=570	gi|6906449|ref|Os03g62960|
lcl|Y75B8A.27 CE37298 WBGene00004172 locus:pqn-92 status:Partially_confirmed SW:Q9XW82 protein_id:CAA22087.2 /QueryS	gi|6906450|ref|Os03g62970|

lcl|Y75B8A.28 CE23039 WBGene00013559 status:Predicted SW:Q9XW81 protein_id:CAA22088.1 /QuerySize=363 gi|6906451|ref|Os03g62980|
lcl|Y75B8A.32 CE23043 WBGene00013562 status:Partially_confirmed SW:Q9XW77 protein_id:CAA22092.1 /QuerySize=444 gi|6906452|ref|Os03g62990|
lcl|Y75B8A.33 CE23044 WBGene00013563 status:Partially_confirmed SW:Q9XW60 protein_id:CAA22111.1 /QuerySize=134 gi|6906460|ref|Os03g63050|
lcl|Y75B8A.35a CE24668 WBGene00006986 locus:zip-1 status:Confirmed SW:Q9U1S5 protein_id:CAB54500.1 /QuerySize=480 gi|6906467|ref|Os03g63100|
lcl|Y75B8A.35b CE23046 WBGene00006986 locus:zip-1 status:Confirmed SW:Q9XTL0 protein_id:CAA22113.1 /QuerySize=463 gi|6906471|ref|Os03g63130|
lcl|Y75B8A.39 CE37299 WBGene00023483 status:Predicted SW:Q67X95 protein_id:CAH19095.1 /QuerySize=222 gi|6906475|ref|Os03g63160|
lcl|Y75B8A.8 CE34135 WBGene00013545 status:Partially_confirmed SW:Q9XW73 protein_id:CAA22097.2 /QuerySize=715 gi|6906476|ref|Os03g63170|
lcl|Y75D11A.1 CE18431 WBGene00022290 status:Predicted SW:O76706 protein_id:AAF36057.1 /QuerySize=71 gi|6906478|ref|Os03g63190|
lcl|Y75D11A.2 CE23051 WBGene00022291 status:Predicted SW:Q9N4D1 protein_id:AAF36058.1 /QuerySize=180 gi|6906480|ref|Os03g63210|
lcl|Y75D11A.3 CE23050 WBGene00022292 status:Confirmed SW:Q9N4D0 protein_id:AAF36056.1 /QuerySize=180 gi|6906493|ref|Os03g63340|
lcl|Y76A2B.4 CE19276 WBGene00013576 status:Partially_confirmed SW:Q9XWD0 protein_id:CAA21745.1 /QuerySize=152 gi|6906494|ref|Os03g63350|
lcl|Y76B12C.4 CE23061 WBGene00022298 status:Partially_confirmed SW:Q9N4C7 protein_id:AAF36065.1 /QuerySize=252 gi|6906516|ref|Os03g63540|
lcl|Y76B12C.6a CE25608 WBGene00022300 status:Partially_confirmed SW:Q9N4C4 protein_id:AAF36066.1 /QuerySize=580 gi|6906519|ref|Os03g63570|
lcl|Y76B12C.6b CE34618 WBGene00022300 status:Partially_confirmed SW:Q7YZX1 protein_id:AAP82631.1 /QuerySize=487 gi|6906523|ref|Os03g63610|
lcl|Y76B12C.8 CE23063 WBGene00022302 status:Confirmed SW:Q95Y40 protein_id:AAL00878.1 /QuerySize=85 gi|6906552|ref|Os03g63840|
lcl|Y76B12C.9 CE29933 WBGene00022303 status:Confirmed SW:Q95Y39 protein_id:AAL27270.1 /QuerySize=65 gi|6906557|ref|Os03g63880|
lcl|Y76G2A.1 CE07952 WBGene00022304 status:Predicted SW:O01475 protein_id:AAC78488.1 /QuerySize=137 gi|6906567|ref|Os03g63960|
lcl|Y76G2A.2 CE07957 WBGene00022305 status:Predicted SW:O01476 protein_id:AAC78489.2 /QuerySize=79 gi|6906569|ref|Os03g63980|
lcl|Y77E11A.14 CE28150 WBGene00022314 status:Confirmed SW:Q95Y38 protein_id:AAK67241.1 /QuerySize=74 gi|6906571|ref|Os03g64000|
lcl|Y77E11A.3 CE36901 WBGene00022308 status:Predicted SW:Q9N4B0 protein_id:AAF36073.2 /QuerySize=117 gi|6906572|ref|Os03g64010|
lcl|Y77E11A.7a CE34430 WBGene00022310 status:Partially_confirmed SW:Q9N4B6 protein_id:AAF36077.2 /QuerySize=605 gi|6906575|ref|Os03g64040|
lcl|Y77E11A.7b CE36902 WBGene00022310 status:Partially_confirmed SW:Q8MXT7 protein_id:AAM97985.2 /QuerySize=603 gi|6906581|ref|Os03g64100|
lcl|Y77E11A.7c CE37300 WBGene00022310 status:Partially_confirmed SW:Q688Z9 protein_id:AAU05554.1 /QuerySize=577 gi|6906587|ref|Os03g64160|
lcl|Y79H2A.12 CE37803 WBGene00043997 status:Predicted SW:Q5TEB3 protein_id:CAI06051.1 /QuerySize=175 gi|6906589|ref|Os03g64180|
lcl|Y79H2A.1a CE23090 WBGene00000273 locus:brp-1 status:Confirmed SW:Q9U1S2 protein_id:CAB54505.1 /QuerySize=552 gi|6906603|ref|Os03g64270|
lcl|Y79H2A.1b CE37301 WBGene00000273 locus:brp-1 status:Confirmed SW:Q67X91 protein_id:CAH19094.1 /QuerySize=466 gi|6906607|ref|Os03g64310|
lcl|Y79H2A.2 CE37804 WBGene00013579 status:Confirmed SW:Q9U1R9 protein_id:CAB54508.2 /QuerySize=167 gi|6906617|ref|Os03g64390|
lcl|Y79H2A.3a CE23092 WBGene00013580 status:Partially_confirmed SW:Q9U1R8 protein_id:CAB54509.1 /QuerySize=2129 gi|7000001|ref|Os04g01010|
lcl|Y79H2A.3b CE35811 WBGene00013580 status:Partially_confirmed SW:Q7K705 protein_id:CAE47474.1 /QuerySize=1271 gi|7000002|ref|Os04g01020|
lcl|Y7A5A.2 CE21327 WBGene00012408 status:Confirmed SW:Q9XVZ1 protein_id:CAA22462.1 /QuerySize=144 gi|7000005|ref|Os04g01050|
lcl|Y7A5A.3 CE21328 WBGene00012409 status:Partially_confirmed SW:Q9XVZ0 protein_id:CAA22464.1 /QuerySize=129 gi|7000017|ref|Os04g01170|
lcl|Y7A5A.5 CE21330 WBGene00012411 status:Predicted SW:Q9XVY9 protein_id:CAA22465.1 /QuerySize=56 gi|7000026|ref|Os04g01260|
lcl|Y7A5A.6 CE21331 WBGene00012412 status:Predicted SW:Q9XVY8 protein_id:CAA22466.1 /QuerySize=130 gi|7000027|ref|Os04g01270|
lcl|Y7A5A.7 CE21332 WBGene00012413 status:Partially_confirmed SW:Q9XVY7 protein_id:CAA22467.1 /QuerySize=383 gi|7000033|ref|Os04g01330|
lcl|Y7A5A.8 CE21333 WBGene00012414 status:Predicted SW:Q9XVY6 protein_id:CAA22468.1 /QuerySize=157 gi|7000039|ref|Os04g01390|
lcl|Y7A5A.9 CE21334 WBGene00012415 status:Predicted SW:Q9XVY5 protein_id:CAA22469.1 /QuerySize=353 gi|7000042|ref|Os04g01420|
lcl|Y7A9A.2 CE14844 WBGene00012417 status:Predicted SW:O18255 protein_id:CAB16540.1 /QuerySize=48 gi|7000044|ref|Os04g01440|
lcl|Y7A9D.1 CE14850 WBGene00012420 status:Confirmed SW:O18259 protein_id:CAB16544.1 /QuerySize=115 gi|7000045|ref|Os04g01450|
lcl|Y80D3A.10 CE27787 WBGene00013588 status:Confirmed SW:Q95ZI9 protein_id:CAC42383.1 /QuerySize=92 gi|7000046|ref|Os04g01460|
lcl|Y80D3A.11 CE36515 WBGene00013589 status:Predicted SW:Q7YWQ2 protein_id:CAE18005.2 /QuerySize=120 gi|7000052|ref|Os04g01500|
lcl|Y81B9A.1 CE23116 WBGene00022315 status:Predicted SW:Q9N4P6 protein_id:AAF60881.1 /QuerySize=579 gi|7000058|ref|Os04g01560|
lcl|Y81B9A.2 CE24679 WBGene00022316 status:Predicted SW:Q9N4P7 protein_id:AAF60882.1 /QuerySize=269 gi|7000060|ref|Os04g01580|
lcl|Y81B9A.3 CE24680 WBGene00022317 status:Predicted SW:Q9N4P8 protein_id:AAF60883.1 /QuerySize=88 gi|7000063|ref|Os04g01610|
lcl|Y81G3A.1 CE23117 WBGene00013590 status:Predicted SW:Q9XVY3 protein_id:CAA22516.1 /QuerySize=243 gi|7000066|ref|Os04g01640|
lcl|Y81G3A.4 CE23119 WBGene00013592 status:Partially_confirmed SW:Q9XVY2 protein_id:CAA22517.1 /QuerySize=282 gi|7000075|ref|Os04g01730|
lcl|Y82E9BL.1 CE27539 WBGene00022318 status:Predicted SW:Q95XB1 protein_id:AAK66030.1 /QuerySize=124 gi|7000077|ref|Os04g01750|
lcl|Y82E9BL.2 CE23418 WBGene00022319 status:Predicted SW:Q9GR29 protein_id:AAG37969.1 /QuerySize=235 gi|7000078|ref|Os04g01760|
lcl|Y82E9BL.3 CE23419 WBGene00022320 status:Partially_confirmed SW:Q9GR30 protein_id:AAG37970.1 /QuerySize=252 gi|7000082|ref|Os04g01800|
lcl|Y82E9BL.5 CE27547 WBGene00022322 status:Partially_confirmed SW:Q95XB3 protein_id:AAK66032.1 /QuerySize=250 gi|7000085|ref|Os04g01830|
lcl|Y82E9BL.6 CE27548 WBGene00022323 status:Partially_confirmed SW:Q95XB4 protein_id:AAK66033.1 /QuerySize=253 gi|7000086|ref|Os04g01840|
lcl|Y82E9BR.1 CE27035 WBGene00022334 status:Partially_confirmed SW:Q9BKR6 protein_id:AAK26151.1 /QuerySize=502 gi|7000094|ref|Os04g01920|
lcl|Y82E9BR.10 CE27036 WBGene00022343 status:Predicted SW:Q9BKS9 protein_id:AAK26144.1 /QuerySize=98 gi|7000096|ref|Os04g01940|
lcl|Y82E9BR.11 CE27037 WBGene00022344 status:Predicted SW:Q9BKS8 protein_id:AAK26143.1 /QuerySize=105 gi|7000104|ref|Os04g02010|
lcl|Y82E9BR.13 CE38010 WBGene00022346 status:Partially_confirmed SW:Q9BKS4 protein_id:AAK26141.1 /QuerySize=251 gi|7000105|ref|Os04g02020|
lcl|Y82E9BR.17a CE27337 WBGene00022349 status:Partially_confirmed SW:Q9BKR7 protein_id:AAK26159.1 /QuerySize=481 gi|7000107|ref|Os04g02040|
lcl|Y82E9BR.17b CE34434 WBGene00022349 status:Partially_confirmed SW:Q7Z1Q7 protein_id:AAP46263.1 /QuerySize=179 gi|7000109|ref|Os04g02060|
lcl|Y82E9BR.19 CE27043 WBGene00022351 status:Predicted SW:Q9BKR4 protein_id:AAK26158.1 /QuerySize=139 gi|7000110|ref|Os04g02070|
lcl|Y82E9BR.20 CE29934 WBGene00022352 status:Partially_confirmed SW:Q95XB0 protein_id:AAL16323.1 /QuerySize=44 gi|7000112|ref|Os04g02090|
lcl|Y82E9BR.21 CE34435 WBGene00022353 status:Partially_confirmed SW:Q7Z1Q6 protein_id:AAP46264.1 /QuerySize=296 gi|7000113|ref|Os04g02100|
lcl|Y82E9BR.22 CE36517 WBGene00022354 status:Predicted SW:Q7KWZ2 protein_id:AAS60255.1 /QuerySize=69 gi|7000117|ref|Os04g02130|
lcl|Y82E9BR.4 CE34436 WBGene00022337 status:Predicted SW:Q9BKS3 protein_id:AAP46265.1 /QuerySize=544 gi|7000120|ref|Os04g02160|
lcl|Y82E9BR.5 CE27046 WBGene00022338 status:Partially_confirmed SW:Q9BKS5 protein_id:AAK26153.1 /QuerySize=41 gi|7000121|ref|Os04g02170|
lcl|Y82E9BR.6 CE23391 WBGene00022339 status:Partially_confirmed SW:Q9BKS6 protein_id:AAK26148.1 /QuerySize=46 gi|7000131|ref|Os04g02270|
lcl|Y82E9BR.7 CE23439 WBGene00022340 status:Predicted SW:Q9BKT0 protein_id:AAK26147.1 /QuerySize=536 gi|7000137|ref|Os04g02330|
lcl|Y82E9BR.8 CE27047 WBGene00022341 status:Predicted SW:Q9BKT2 protein_id:AAK26146.1 /QuerySize=390 gi|7000142|ref|Os04g02380|
lcl|Y82E9BR.9 CE23415 WBGene00022342 status:Predicted SW:Q9BKT1 protein_id:AAK26145.1 /QuerySize=139 gi|7000144|ref|Os04g02400|
lcl|Y87G2A.1 CE24681 WBGene00013593 status:Partially_confirmed SW:Q9U1Q6 protein_id:CAB60426.1 /QuerySize=591 gi|7000145|ref|Os04g02410|
lcl|Y87G2A.7 CE23136 WBGene00013596 status:Partially_confirmed SW:Q9NA28 protein_id:CAB54490.1 /QuerySize=489 gi|7000148|ref|Os04g02440|
lcl|Y8A9A.4 CE36346 WBGene00021173 status:Partially_confirmed SW:Q9TYK7 protein_id:AAK18993.2 /QuerySize=107 gi|7000151|ref|Os04g02470|
lcl|Y92C3A.2 CE30840 WBGene00022355 status:Predicted SW:Q9BL25 protein_id:AAK31570.2 /QuerySize=396 gi|7000152|ref|Os04g02480|
lcl|Y92C3A.3 CE26310 WBGene00022356 status:Partially_confirmed SW:Q9BL23 protein_id:AAK31571.1 /QuerySize=169 gi|7000153|ref|Os04g02490|
lcl|Y92C3B.1 CE34437 WBGene00022357 locus:kbp-4 status:Confirmed SW:Q9N2X3 protein_id:AAK29987.2 /QuerySize=97 gi|7000156|ref|Os04g02520|
lcl|Y92H12A.3 CE36765 WBGene00022359 status:Partially_confirmed SW:Q9BKW5 protein_id:AAK29731.2 /QuerySize=249 gi|7000157|ref|Os04g02530|
lcl|Y92H12A.6 CE32282 WBGene00022362 status:Predicted SW:Q9BKW7 protein_id:AAK29734.2 /QuerySize=372 gi|7000160|ref|Os04g02560|
lcl|Y92H12BL.2 CE36768 WBGene00022364 status:Predicted SW:Q9BKW1 protein_id:AAK19004.2 /QuerySize=64 gi|7000161|ref|Os04g02570|
lcl|Y92H12BM.1 CE26646 WBGene00022367 status:Predicted SW:Q9GR27 protein_id:AAG37982.1 /QuerySize=71 gi|7000163|ref|Os04g02590|
lcl|Y92H12BR.2 CE26782 WBGene00022368 status:Confirmed SW:Q9BPN9 protein_id:AAK09077.1 /QuerySize=177 gi|7000165|ref|Os04g02610|
lcl|Y92H12BR.3 CE29936 WBGene00022369 status:Partially_confirmed SW:Q9BPN8 protein_id:AAK09080.2 /QuerySize=637 gi|7000167|ref|Os04g02630|
lcl|Y92H12BR.4 CE26784 WBGene00022370 status:Partially_confirmed SW:Q9BPN7 protein_id:AAK09081.1 /QuerySize=61 gi|7000170|ref|Os04g02660|
lcl|Y92H12BR.7 CE26786 WBGene00022372 status:Partially_confirmed SW:Q9BPP2 protein_id:AAK09079.1 /QuerySize=598 gi|7000183|ref|Os04g02790|
lcl|Y94H6A.10 CE36575 WBGene00022382 status:Partially_confirmed SW:Q75MI6 protein_id:AAS80337.1 /QuerySize=181 gi|7000184|ref|Os04g02800|
lcl|Y94H6A.3 CE27552 WBGene00022376 status:Confirmed SW:Q9N2X1 protein_id:AAF60894.2 /QuerySize=186 gi|7000185|ref|Os04g02810|
lcl|Y95B8A.11 CE34441 WBGene00022390 status:Partially_confirmed SW:Q9N2V7 protein_id:AAF60907.2 /QuerySize=435 gi|7000188|ref|Os04g02840|
lcl|Y95B8A.2 CE24691 WBGene00022383 status:Confirmed SW:Q9N2V3 protein_id:AAF60902.1 /QuerySize=128 gi|7000193|ref|Os04g02880|
lcl|Y95B8A.4 CE36771 WBGene00022385 status:Predicted SW:Q9N2V5 protein_id:AAF60900.2 /QuerySize=214 gi|7000201|ref|Os04g02940|
lcl|Y95B8A.8 CE24694 WBGene00022388 status:Predicted SW:Q9N2W1 protein_id:AAF60906.1 /QuerySize=187 gi|7000208|ref|Os04g03010|
lcl|Y95D11A.1 CE23166 WBGene00013605 status:Confirmed SW:Q9U1P7 protein_id:CAB60435.1 /QuerySize=144 gi|7000209|ref|Os04g03020|
lcl|Y97E10AM.1 CE25640 WBGene00022395 status:Partially_confirmed SW:Q9N2V1 protein_id:AAF60909.1 /QuerySize=135 gi|7000214|ref|Os04g03070|
lcl|Y97E10B.8 CE26328 WBGene00022407 status:Predicted SW:Q965R4 protein_id:AAK71416.1 /QuerySize=162 gi|7000216|ref|Os04g03090|
lcl|Y97E10C.1 CE31135 WBGene00022410 status:Partially_confirmed SW:Q8MXT9 protein_id:AAM48561.1 /QuerySize=100 gi|7000220|ref|Os04g03130|
lcl|Y9C2UA.1a CE33005 WBGene00012421 status:Confirmed SW:Q9XXU0 protein_id:CAA15517.2 /QuerySize=343 gi|7000221|ref|Os04g03140|
lcl|Y9C2UA.1b CE33006 WBGene00012421 status:Confirmed SW:Q8I0C5 protein_id:CAD59164.1 /QuerySize=384 gi|7000223|ref|Os04g03160|
lcl|Y9C2UA.2 CE21341 WBGene00012422 status:Partially_confirmed SW:Q9XXT9 protein_id:CAA15518.1 /QuerySize=103 gi|7000224|ref|Os04g03170|
lcl|Y9D1A.1 CE33250 WBGene00021186 status:Confirmed SW:Q9TYK9 protein_id:AAD12847.2 /QuerySize=77 gi|7000230|ref|Os04g03220|
lcl|Y9D1A.2 CE21358 WBGene00021187 status:Predicted SW:Q9TYK8 protein_id:AAD12846.1 /QuerySize=151 gi|7000231|ref|Os04g03230|
lcl|ZC116.1 CE32031 WBGene00013853 status:Confirmed SW:Q23241 protein_id:CAA98556.2 /QuerySize=178 gi|7000236|ref|Os04g03280|
lcl|ZC123.1 CE28152 WBGene00022517 status:Partially_confirmed SW:O45021 protein_id:AAB97604.2 /QuerySize=730 gi|7000237|ref|Os04g03290|
lcl|ZC13.2 CE29614 WBGene00022503 status:Confirmed SW:Q95Q41 protein_id:AAL13328.1 /QuerySize=321 gi|7000239|ref|Os04g03310|
lcl|ZC13.3 CE14988 WBGene00022504 status:Partially_confirmed SW:Q95Q40 protein_id:AAL13329.1 /QuerySize=732 gi|7000247|ref|Os04g03390|
lcl|ZC132.2 CE15038 WBGene00022521 status:Partially_confirmed SW:O17519 protein_id:AAB63924.1 /QuerySize=98 gi|7000251|ref|Os04g03430|
lcl|ZC132.3 CE15040 WBGene00022522 status:Partially_confirmed SW:O17515 protein_id:AAB63926.1 /QuerySize=578 gi|7000252|ref|Os04g03440|
lcl|ZC132.4 CE15042 WBGene00022523 status:Predicted SW:O17516 protein_id:AAB63927.1 /QuerySize=831 gi|7000254|ref|Os04g03460|
lcl|ZC132.6 CE38012 WBGene00022525 status:Predicted SW:O17518 protein_id:AAB63928.1 /QuerySize=302 gi|7000256|ref|Os04g03480|
lcl|ZC132.8 CE26610 WBGene00022526 status:Predicted SW:O17522 protein_id:AAB63930.2 /QuerySize=166 gi|7000258|ref|Os04g03500|
lcl|ZC132.9 CE26611 WBGene00022527 status:Partially_confirmed SW:O17523 protein_id:AAB63931.2 /QuerySize=246 gi|7000259|ref|Os04g03510|
lcl|ZC15.1 CE16683 WBGene00013830 status:Predicted SW:O18265 protein_id:CAB07709.1 /QuerySize=875 gi|7000260|ref|Os04g03520|
lcl|ZC15.3 CE16685 WBGene00013832 status:Confirmed SW:O18267 protein_id:CAB07711.1 /QuerySize=343 gi|7000266|ref|Os04g03580|
lcl|ZC15.5 CE16686 WBGene00013833 status:Predicted SW:O18268 protein_id:CAB07712.1 /QuerySize=597 gi|7000273|ref|Os04g03650|
lcl|ZC15.8 CE16689 WBGene00004173 locus:pqn-94 status:Confirmed SW:O18271 protein_id:CAB07715.1 /QuerySize=109 gi|7000284|ref|Os04g03760|
lcl|ZC155.5a CE33007 WBGene00022533 status:Partially_confirmed SW:Q8I7F6 protein_id:AAN84795.1 /QuerySize=232 gi|7000290|ref|Os04g03820|
lcl|ZC155.5b CE33008 WBGene00022533 status:Partially_confirmed SW:Q8I7F5 protein_id:AAN84796.1 /QuerySize=237 gi|7000292|ref|Os04g03840|
lcl|ZC168.3 CE35700 WBGene00013857 status:Partially_confirmed SW:Q23249 protein_id:CAA94386.2 /QuerySize=396 gi|7000296|ref|Os04g03880|
lcl|ZC190.10 CE18432 WBGene00022544 status:Predicted SW:O76707 protein_id:AAC26963.1 /QuerySize=132 gi|7000298|ref|Os04g03900|
lcl|ZC190.5 CE18428 WBGene00022539 status:Predicted SW:O76700 protein_id:AAC26964.1 /QuerySize=311 gi|7000299|ref|Os04g03910|
lcl|ZC190.6 CE19640 WBGene00022540 status:Predicted SW:O76701 protein_id:AAC26966.1 /QuerySize=547 gi|7000300|ref|Os04g03920|
lcl|ZC190.7 CE28156 WBGene00022541 status:Confirmed SW:O76704 protein_id:AAC26959.2 /QuerySize=52 gi|7000301|ref|Os04g03930|
lcl|ZC190.8 CE18430 WBGene00022542 status:Predicted SW:O76705 protein_id:AAC26958.1 /QuerySize=227 gi|7000304|ref|Os04g03960|
lcl|ZC190.9 CE18431 WBGene00022543 status:Predicted SW:O76706 protein_id:AAC26962.1 /QuerySize=71 gi|7000305|ref|Os04g03970|
lcl|ZC196.1 CE36055 WBGene00022545 status:Partially_confirmed SW:O01631 protein_id:AAB52294.2 /QuerySize=139 gi|7000313|ref|Os04g04040|
lcl|ZC196.6 CE36057 WBGene00022550 status:Partially_confirmed SW:O01626 protein_id:AAB52297.2 /QuerySize=491 gi|7000316|ref|Os04g04070|
lcl|ZC204.13 CE15108 WBGene00022563 status:Predicted SW:P91545 protein_id:AAB37919.1 /QuerySize=156 gi|7000318|ref|Os04g04090|
lcl|ZC204.14 CE15110 WBGene00022564 status:Partially_confirmed SW:P91546 protein_id:AAB37920.1 /QuerySize=151 gi|7000326|ref|Os04g04170|
lcl|ZC204.6 CE15094 WBGene00022556 status:Predicted SW:P91537 protein_id:AAB37916.1 /QuerySize=170 gi|7000330|ref|Os04g04210|
lcl|ZC21.3 CE34442 WBGene00022505 Dual bar protein status:Partially_confirmed SW:P34587 protein_id:AAA28169.3 /QuerySize gi|7000331|ref|Os04g04220|
lcl|ZC21.6a CE33253 WBGene00022506 status:Partially_confirmed SW:Q6LED9 protein_id:AAO12393.1 /QuerySize=285 gi|7000341|ref|Os04g04310|
lcl|ZC21.8 CE00559 WBGene00022507 status:Partially_confirmed SW:P34592 protein_id:AAA28170.2 /QuerySize=114 gi|7000345|ref|Os04g04350|
lcl|ZC247.2 CE15148 WBGene00013860 status:Partially_confirmed SW:Q94379 protein_id:CAB02309.1 /QuerySize=690 gi|7000346|ref|Os04g04360|
lcl|ZC262.1 CE28272 WBGene00022578 status:Predicted /QuerySize=60 gi|7000347|ref|Os04g04370|
lcl|ZC262.2a CE00350 WBGene00022579 status:Partially_confirmed SW:P34594 protein_id:AAK29991.1 /QuerySize=233 gi|7000351|ref|Os04g04410|
lcl|ZC262.2b CE33010 WBGene00022579 status:Confirmed SW:Q8I7F4 protein_id:AAN84797.1 /QuerySize=63 gi|7000360|ref|Os04g04500|
lcl|ZC262.4 CE34443 WBGene00022581 status:Partially_confirmed SW:P34596 protein_id:AAK29992.3 /QuerySize=149 gi|7000364|ref|Os04g04540|
lcl|ZC266.1 CE07590 WBGene00022584 status:Partially_confirmed SW:Q23254 protein_id:AAA96102.1 /QuerySize=134 gi|7000366|ref|Os04g04560|
lcl|ZC266.2 CE07591 WBGene00022585 status:Partially_confirmed SW:Q23253 protein_id:AAA96101.1 /QuerySize=276 gi|7000367|ref|Os04g04570|
lcl|ZC302.3 CE06575 WBGene00013863 status:Partially_confirmed SW:Q23257 protein_id:CAA98294.1 /QuerySize=236 gi|7000370|ref|Os04g04600|

lcl|ZC308.4 CE15165 WBGene00022586 status:Partially_confirmed SW:O17085 protein_id:AAB70344.1 /QuerySize=329	gi|7000371|ref|Os04g04610|
lcl|ZC317.2 CE15169 WBGene00022588 status:Confirmed SW:Q23075 protein_id:AAB04960.1 /QuerySize=131	gi|7000372|ref|Os04g04620|
lcl|ZC317.6 CE15177 WBGene00022589 status:Predicted SW:Q23078 protein_id:AAB04963.1 /QuerySize=308	gi|7000375|ref|Os04g04650|
lcl|ZC317.7 CE15179 WBGene00022590 status:Partially_confirmed SW:Q23079 protein_id:AAB04964.1 /QuerySize=574	gi|7000376|ref|Os04g04660|
lcl|ZC328.1 CE27347 WBGene00022591 status:Confirmed SW:O02051 protein_id:AAK39380.1 /QuerySize=202	gi|7000378|ref|Os04g04680|
lcl|ZC328.4 CE34445 WBGene00004721 locus:san-1 status:Confirmed SW:O02053 protein_id:AAK39378.2 /QuerySize=390	gi|7000386|ref|Os04g04760|
lcl|ZC328.5 CE15189 WBGene00022594 status:Partially_confirmed SW:O02052 protein_id:AAK39379.1 /QuerySize=191	gi|7000388|ref|Os04g04780|
lcl|ZC373.2 CE02375 WBGene00013867 status:Partially_confirmed SW:Q23258 protein_id:CAA88974.1 /QuerySize=122	gi|7000391|ref|Os04g04810|
lcl|ZC373.6 CE02379 WBGene00000930 locus:dao-4 status:Partially_confirmed SW:Q23262 protein_id:CAA88978.1 /QuerySize=2	gi|7000393|ref|Os04g04830|
lcl|ZC376.6 CE15203 WBGene00013877 status:Partially_confirmed SW:Q23271 protein_id:CAB00882.1 /QuerySize=1152	gi|7000394|ref|Os04g04840|
lcl|ZC395.1 CE01435 WBGene00022595 status:Partially_confirmed SW:Q9GYF8 protein_id:AAG00034.1 /QuerySize=213	gi|7000397|ref|Os04g04870|
lcl|ZC395.11 CE01437 WBGene00022600 status:Predicted SW:Q9GYF9 protein_id:AAG00039.1 /QuerySize=55	gi|7000399|ref|Os04g04890|
lcl|ZC395.4 CE01440 WBGene00022596 status:Predicted SW:Q9GYG0 protein_id:AAG00036.1 /QuerySize=169	gi|7000401|ref|Os04g04910|
lcl|ZC395.5 CE01441 WBGene00022597 status:Confirmed SW:Q9GYG2 protein_id:AAG00037.1 /QuerySize=74	gi|7000407|ref|Os04g04970|
lcl|ZC404.1 CE07592 WBGene00022601 status:Predicted SW:Q23291 protein_id:AAA97967.1 /QuerySize=184	gi|7000410|ref|Os04g05000|
lcl|ZC404.3a CE07594 WBGene00004975 locus:spe-39 status:Partially_confirmed SW:Q23288 protein_id:AAA97964.1 /QuerySize	gi|7000417|ref|Os04g05070|
lcl|ZC404.3b CE29620 WBGene00004975 locus:spe-39 status:Confirmed SW:Q95PY2 protein_id:AAL11104.1 /QuerySize=84	gi|7000419|ref|Os04g05090|
lcl|ZC404.7 CE07597 WBGene00022602 status:Predicted SW:Q23286 protein_id:AAA97962.1 /QuerySize=110	gi|7000420|ref|Os04g05100|
lcl|ZC410.5a CE15209 WBGene00013882 microfilarial antigen like status:Partially_confirmed SW:P91844 protein_id:CAA92570.1 /	gi|7000422|ref|Os04g05120|
lcl|ZC410.5b CE35703 WBGene00013882 status:Partially_confirmed SW:Q7JM19 protein_id:CAE46688.1 /QuerySize=158	gi|7000423|ref|Os04g05130|
lcl|ZC410.7a CE26842 WBGene00003066 locus:lpl-1 status:Confirmed SW:O62503 protein_id:CAA92571.2 /QuerySize=109	gi|7000428|ref|Os04g05180|
lcl|ZC411.1 CE07602 WBGene00022607 status:Predicted SW:Q23301 protein_id:AAA96111.1 /QuerySize=348	gi|7000429|ref|Os04g05190|
lcl|ZC412.3 CE15215 WBGene00013884 status:Confirmed SW:Q23307 protein_id:CAB01530.1 /QuerySize=250	gi|7000433|ref|Os04g05230|
lcl|ZC412.4 CE15217 WBGene00013885 status:Partially_confirmed SW:Q23308 protein_id:CAB01531.1 /QuerySize=223	gi|7000435|ref|Os04g05250|
lcl|ZC412.5 CE15219 WBGene00013886 status:Partially_confirmed SW:Q23309 protein_id:CAB01532.1 /QuerySize=168	gi|7000436|ref|Os04g05260|
lcl|ZC412.6 CE15221 WBGene00013887 status:Confirmed SW:Q23306 protein_id:CAB01529.1 /QuerySize=34	gi|7000442|ref|Os04g05320|
lcl|ZC412.7 CE15223 WBGene00013888 status:Predicted SW:Q23303 protein_id:CAB01526.1 /QuerySize=34	gi|7000448|ref|Os04g05380|
lcl|ZC412.8 CE15225 WBGene00013889 status:Predicted SW:Q23304 protein_id:CAB01527.1 /QuerySize=83	gi|7000449|ref|Os04g05390|
lcl|ZC412.9 CE15227 WBGene00013890 status:Predicted SW:Q23302 protein_id:CAB01525.1 /QuerySize=132	gi|7000454|ref|Os04g05440|
lcl|ZC416.2 CE17304 WBGene00022609 status:Predicted SW:O44184 protein_id:AAB88367.1 /QuerySize=125	gi|7000456|ref|Os04g05460|
lcl|ZC434.3 CE06578 WBGene00013891 status:Partially_confirmed SW:Q23313 protein_id:CAB00059.1 /QuerySize=474	gi|7000459|ref|Os04g05490|
lcl|ZC434.7 CE06582 WBGene00013893 status:Partially_confirmed SW:Q23317 protein_id:CAB00061.1 /QuerySize=471	gi|7000460|ref|Os04g05500|
lcl|ZC443.3 CE06587 WBGene00013898 status:Partially_confirmed SW:Q23321 protein_id:CAA99948.1 /QuerySize=407	gi|7000461|ref|Os04g05510|
lcl|ZC443.4 CE06588 WBGene00013899 status:Confirmed SW:Q23319 protein_id:CAA99946.1 /QuerySize=389	gi|7000464|ref|Os04g05540|
lcl|ZC449.4 CE37688 WBGene00022614 status:Confirmed SW:Q23329 protein_id:AAK39381.2 /QuerySize=76	gi|7000465|ref|Os04g05550|
lcl|ZC449.5 CE27350 WBGene00022615 status:Partially_confirmed SW:Q23330 protein_id:AAK39385.1 /QuerySize=493	gi|7000470|ref|Os04g05600|
lcl|ZC455.1 CE32034 WBGene00013902 status:Partially_confirmed SW:Q23332 protein_id:CAA99953.3 /QuerySize=681	gi|7000473|ref|Os04g05630|
lcl|ZC477.4 CE05060 WBGene00022619 status:Predicted SW:Q23341 protein_id:AAK19010.1 /QuerySize=80	gi|7000475|ref|Os04g05650|
lcl|ZC477.5 CE28164 WBGene00022620 status:Confirmed SW:Q23342 protein_id:AAK19011.2 /QuerySize=339	gi|7000484|ref|Os04g05740|
lcl|ZC477.7 CE05063 WBGene00022621 status:Predicted SW:Q23346 protein_id:AAK19013.1 /QuerySize=194	gi|7000485|ref|Os04g05750|
lcl|ZC487.3 CE26335 WBGene00022625 status:Predicted SW:Q23350 protein_id:AAA92539.2 /QuerySize=79	gi|7000488|ref|Os04g05780|
lcl|ZC504.5 CE30223 WBGene00001804 locus:gur-3 status:Confirmed SW:O18280 protein_id:CAA90343.3 /QuerySize=447	gi|7000492|ref|Os04g05820|
lcl|ZC513.12 CE31137 WBGene00006060 locus:sth-1 status:Confirmed SW:Q95YG4 protein_id:AAM48562.1 /QuerySize=119	gi|7000493|ref|Os04g05830|
lcl|ZC513.3 CE07610 WBGene00022628 status:Partially_confirmed SW:Q23363 protein_id:AAC48265.1 /QuerySize=619	gi|7000494|ref|Os04g05840|
lcl|ZC518.4 CE06604 WBGene00013922 status:Predicted SW:Q23367 protein_id:CAA92987.1 /QuerySize=240	gi|7000495|ref|Os04g05850|
lcl|ZC53.1 CE35813 WBGene00022508 status:Partially_confirmed SW:Q23377 protein_id:AAB52616.2 /QuerySize=448	gi|7000497|ref|Os04g05870|
lcl|ZC53.2 CE07584 WBGene00022509 status:Predicted SW:Q23374 protein_id:AAB52617.1 /QuerySize=171	gi|7000501|ref|Os04g05910|
lcl|ZC581.3 CE31399 WBGene00022633 status:Partially_confirmed SW:O01772 protein_id:AAB54141.4 /QuerySize=737	gi|7000505|ref|Os04g05950|
lcl|ZC8.4c CE33745 WBGene00022500 locus:lfi-1 status:Partially_confirmed SW:Q86MI1 protein_id:AAO91693.1 /QuerySize=249	gi|7000506|ref|Os04g05960|
lcl|ZC84.7 CE35147 WBGene00013850 status:Predicted SW:Q7YWM4 protein_id:CAE18055.1 /QuerySize=59	gi|7000512|ref|Os04g06020|
lcl|ZC97.1b CE33258 WBGene00022516 status:Confirmed SW:Q8I7F3 protein_id:AAO12391.1 /QuerySize=55	gi|7000527|ref|Os04g06170|
lcl|ZK1010.5 CE15503 WBGene00014179 status:Partially_confirmed SW:O18284 protein_id:CAB04971.1 /QuerySize=756	gi|7000537|ref|Os04g06270|
lcl|ZK1010.8 CE15509 WBGene00014181 status:Partially_confirmed SW:O18287 protein_id:CAB04974.1 /QuerySize=388	gi|7000539|ref|Os04g06290|
lcl|ZK1025.5 CE19332 WBGene00014185 status:Predicted SW:Q9U1P2 protein_id:CAA18363.1 /QuerySize=108	gi|7000542|ref|Os04g06320|
lcl|ZK1053.2 CE15517 WBGene00014197 status:Partially_confirmed SW:O45994 protein_id:CAB04976.1 /QuerySize=536	gi|7000544|ref|Os04g06340|
lcl|ZK1053.3 CE15519 WBGene00014198 status:Partially_confirmed SW:O62505 protein_id:CAB04977.1 /QuerySize=780	gi|7000545|ref|Os04g06350|
lcl|ZK1053.4 CE16763 WBGene00014199 status:Partially_confirmed SW:O45995 protein_id:CAB04980.1 /QuerySize=667	gi|7000548|ref|Os04g06380|
lcl|ZK1055.3 CE18472 WBGene00022844 status:Partially_confirmed SW:O76445 protein_id:AAC19261.1 /QuerySize=101	gi|7000554|ref|Os04g06440|
lcl|ZK1055.6a CE18475 WBGene00022847 status:Partially_confirmed SW:O76448 protein_id:AAC19264.1 /QuerySize=531	gi|7000556|ref|Os04g06460|
lcl|ZK1055.6b CE29154 WBGene00022847 status:Partially_confirmed SW:Q95X28 protein_id:AAL00880.1 /QuerySize=564	gi|7000557|ref|Os04g06470|
lcl|ZK1055.6c CE31138 WBGene00022847 status:Confirmed SW:Q8MXF9 protein_id:AAM48563.1 /QuerySize=53	gi|7000559|ref|Os04g06490|
lcl|ZK1055.7 CE18476 WBGene00022848 status:Partially_confirmed SW:O76449 protein_id:AAC19265.1 /QuerySize=570	gi|7000562|ref|Os04g06520|
lcl|ZK1058.9 CE01107 WBGene00014207 status:Partially_confirmed SW:Q23385 protein_id:CAA84682.1 /QuerySize=304	gi|7000563|ref|Os04g06530|
lcl|ZK1067.3 CE33012 WBGene00014209 status:Confirmed SW:Q23389 protein_id:CAA93885.2 /QuerySize=399	gi|7000567|ref|Os04g06570|
lcl|ZK1067.7 CE03844 WBGene00004174 locus:pqn-95 status:Confirmed SW:Q23390 protein_id:CAA93886.1 /QuerySize=314	gi|7000575|ref|Os04g06650|
lcl|ZK1067.8 CE32288 WBGene00014212 status:Predicted SW:Q8MPS0 protein_id:CAD36503.2 /QuerySize=192	gi|7000581|ref|Os04g06710|
lcl|ZK1073.2 CE24713 WBGene00014214 status:Partially_confirmed SW:Q23393 protein_id:CAA92228.2 /QuerySize=194	gi|7000582|ref|Os04g06720|
lcl|ZK1086.2 CE06681 WBGene00014216 status:Partially_confirmed SW:Q23396 protein_id:CAA94224.1 /QuerySize=143	gi|7000583|ref|Os04g06730|
lcl|ZK1098.2 CE34139 WBGene00014219 status:Partially_confirmed SW:P34601 protein_id:CAD91716.1 /QuerySize=626	gi|7000584|ref|Os04g06740|
lcl|ZK1098.6 CE00369 WBGene00014223 Coiled coil protein status:Partially_confirmed SW:P34606 protein_id:CAA80134.1 /Quer	gi|7000585|ref|Os04g06750|
lcl|ZK1098.9 CE00371 WBGene00014225 status:Predicted SW:P34608 protein_id:CAA80138.1 /QuerySize=220	gi|7000586|ref|Os04g06760|
lcl|ZK112.3 CE00374 WBGene00022659 status:Predicted SW:P34612 protein_id:AAA28186.1 /QuerySize=160	gi|7000588|ref|Os04g06780|
lcl|ZK112.4 CE00375 WBGene00022660 status:Partially_confirmed SW:P34613 protein_id:AAA28187.1 /QuerySize=100	gi|7000590|ref|Os04g06800|
lcl|ZK112.5 CE00376 WBGene00022661 status:Partially_confirmed SW:P34614 protein_id:AAA28184.1 /QuerySize=159	gi|7000593|ref|Os04g06830|
lcl|ZK112.6 CE00377 WBGene00022662 status:Predicted SW:P34615 protein_id:AAA28183.1 /QuerySize=69	gi|7000603|ref|Os04g06930|
lcl|ZK1127.12 CE33491 WBGene00022857 status:Partially_confirmed SW:Q23401 protein_id:AAB93434.2 /QuerySize=246	gi|7000605|ref|Os04g06950|
lcl|ZK1127.4 CE07643 WBGene00022851 status:Confirmed SW:Q23402 protein_id:AAB93435.1 /QuerySize=349	gi|7000615|ref|Os04g07050|
lcl|ZK1128.3 CE01685 WBGene00014229 status:Partially_confirmed SW:Q09358 protein_id:CAA87422.1 /QuerySize=360	gi|7000618|ref|Os04g07080|
lcl|ZK1193.4 CE05129 WBGene00022860 status:Predicted SW:Q23412 protein_id:AAA83294.1 /QuerySize=188	gi|7000623|ref|Os04g07130|
lcl|ZK1225.4 CE19343 WBGene00014238 status:Partially_confirmed SW:Q9XXL5 protein_id:CAA18371.1 /QuerySize=519	gi|7000624|ref|Os04g07140|
lcl|ZK1225.5 CE19344 WBGene00014239 status:Partially_confirmed SW:Q9XXL1 protein_id:CAA18376.1 /QuerySize=511	gi|7000625|ref|Os04g07150|
lcl|ZK1236.3 CE00532 WBGene00023405 status:Partially_confirmed SW:P34619 protein_id:AAA28193.1 /QuerySize=1000	gi|7000628|ref|Os04g07180|
lcl|ZK1236.5a CE37595 WBGene00022864 status:Partially_confirmed SW:Q629J9 protein_id:AAU87804.1 /QuerySize=306	gi|7000629|ref|Os04g07190|
lcl|ZK1236.5b CE37596 WBGene00022864 status:Confirmed SW:Q629J8 protein_id:AAU87805.1 /QuerySize=60	gi|7000631|ref|Os04g07210|
lcl|ZK1236.6 CE00383 WBGene00004175 locus:pqn-96 status:Partially_confirmed SW:P34622 protein_id:AAA28189.1 /QuerySize	gi|7000632|ref|Os04g07220|
lcl|ZK1240.4 CE15535 WBGene00022869 status:Predicted SW:O16678 protein_id:AAB66142.1 /QuerySize=134	gi|7000638|ref|Os04g07280|
lcl|ZK1248.11 CE02895 WBGene00022881 status:Confirmed SW:Q23420 protein_id:AAC71092.1 /QuerySize=201	gi|7000641|ref|Os04g07310|
lcl|ZK1248.13 CE33497 WBGene00022882 status:Partially_confirmed SW:Q23421 protein_id:AAC71093.3 /QuerySize=604	gi|7000643|ref|Os04g07330|
lcl|ZK1248.8 CE02908 WBGene00022878 status:Predicted SW:Q23416 protein_id:AAC71089.1 /QuerySize=76	gi|7000649|ref|Os04g07390|
lcl|ZK1251.7 CE03854 WBGene00014242 status:Partially_confirmed SW:Q23434 protein_id:CAA92503.1 /QuerySize=138	gi|7000651|ref|Os04g07410|
lcl|ZK1290.11 CE02081 WBGene00022892 status:Partially_confirmed SW:Q09338 protein_id:AAB93317.1 /QuerySize=141	gi|7000652|ref|Os04g07420|
lcl|ZK1290.13 CE32084 WBGene00022893 status:Confirmed SW:Q8I7F2 protein_id:AAN60508.1 /QuerySize=284	gi|7000654|ref|Os04g07440|
lcl|ZK1290.7 CE36070 WBGene00022889 status:Partially_confirmed SW:Q09634 protein_id:AAB93314.2 /QuerySize=270	gi|7000656|ref|Os04g07460|
lcl|ZK1290.8 CE02087 WBGene00006956 locus:wrt-10 status:Confirmed SW:Q09635 protein_id:AAB93318.1 /QuerySize=258	gi|7000660|ref|Os04g07500|
lcl|ZK1307.2 CE01691 WBGene00014245 status:Partially_confirmed SW:Q09360 protein_id:CAA87430.1 /QuerySize=127	gi|7000662|ref|Os04g07520|
lcl|ZK131.11a CE32036 WBGene00013934 status:Confirmed SW:O62022 protein_id:CAB05840.2 /QuerySize=337	gi|7000663|ref|Os04g07530|
lcl|ZK131.11b CE32037 WBGene00013934 status:Confirmed SW:Q8I0M4 protein_id:CAD54180.1 /QuerySize=449	gi|7000664|ref|Os04g07540|
lcl|ZK1320.10 CE01699 WBGene00003749 locus:nlp-11 status:Confirmed SW:Q09367 protein_id:CAA87048.1 /QuerySize=134	gi|7000667|ref|Os04g07570|
lcl|ZK1320.11 CE29941 WBGene00014259 status:Partially_confirmed SW:Q8WQ98 protein_id:CAD18881.1 /QuerySize=204	gi|7000671|ref|Os04g07610|
lcl|ZK1320.13 CE37864 WBGene00044007 status:Predicted /QuerySize=68	gi|7000672|ref|Os04g07620|
lcl|ZK1320.5 CE01703 WBGene00014255 status:Partially_confirmed SW:Q09366 protein_id:CAA87043.1 /QuerySize=414	gi|7000673|ref|Os04g07630|
lcl|ZK1321.1 CE01708 WBGene00014260 status:Predicted SW:Q09368 protein_id:CAA88475.1 /QuerySize=158	gi|7000674|ref|Os04g07640|
lcl|ZK154.1 CE31266 WBGene00022665 status:Confirmed SW:Q94312 protein_id:AAB09093.2 /QuerySize=209	gi|7000678|ref|Os04g07680|
lcl|ZK154.4 CE28176 WBGene00022666 status:Partially_confirmed SW:Q94313 protein_id:AAB09094.2 /QuerySize=203	gi|7000679|ref|Os04g07690|
lcl|ZK154.5 CE28177 WBGene00022667 status:Partially_confirmed SW:Q94314 protein_id:AAB09095.2 /QuerySize=1105	gi|7000680|ref|Os04g07700|
lcl|ZK154.6a CE28178 WBGene00022668 status:Confirmed SW:Q94315 protein_id:AAB09096.2 /QuerySize=148	gi|7000682|ref|Os04g07720|
lcl|ZK154.6b CE35816 WBGene00022668 status:Confirmed SW:Q7JNL6 protein_id:AAQ91891.1 /QuerySize=119	gi|7000686|ref|Os04g07760|
lcl|ZK177.1 CE02089 WBGene00022669 status:Partially_confirmed SW:Q09370 protein_id:AAG00044.1 /QuerySize=460	gi|7000688|ref|Os04g07780|
lcl|ZK177.4a CE29069 WBGene00022672 status:Confirmed SW:Q09372 protein_id:AAG00052.2 /QuerySize=655	gi|7000692|ref|Os04g07820|
lcl|ZK177.9 CE02098 WBGene00022674 status:Predicted SW:Q09375 protein_id:AAG00047.1 /QuerySize=121	gi|7000693|ref|Os04g07830|
lcl|ZK180.5a CE07623 WBGene00022679 status:Partially_confirmed SW:Q23446 protein_id:AAB52969.3 /QuerySize=542	gi|7000695|ref|Os04g07850|
lcl|ZK180.5b CE31746 WBGene00022679 status:Partially_confirmed SW:Q8MPR7 protein_id:AAM97986.1 /QuerySize=497	gi|7000698|ref|Os04g07880|
lcl|ZK180.5c CE28179 WBGene00022679 status:Partially_confirmed SW:Q8MPR8 protein_id:AAM97987.1 /QuerySize=399	gi|7000699|ref|Os04g07890|
lcl|ZK180.6 CE07624 WBGene00022680 status:Partially_confirmed SW:Q23447 protein_id:AAB52970.1 /QuerySize=718	gi|7000700|ref|Os04g07900|
lcl|ZK185.1 CE17310 WBGene00022681 status:Partially_confirmed SW:O44197 protein_id:AAB88558.1 /QuerySize=285	gi|7000701|ref|Os04g07910|
lcl|ZK185.3 CE31748 WBGene00022683 status:Partially_confirmed SW:O44195 protein_id:AAB88560.2 /QuerySize=293	gi|7000702|ref|Os04g07920|
lcl|ZK218.4 CE16704 WBGene00013938 status:Partially_confirmed SW:O45999 protein_id:CAB04985.1 /QuerySize=180	gi|7000703|ref|Os04g07930|
lcl|ZK228.1 CE16710 WBGene00013944 status:Predicted SW:O46007 protein_id:CAB04993.1 /QuerySize=738	gi|7000706|ref|Os04g07960|
lcl|ZK262.4 CE20427 WBGene00013951 status:Partially_confirmed SW:Q9U1P0 protein_id:CAB63419.1 /QuerySize=76	gi|7000707|ref|Os04g07970|
lcl|ZK262.8 CE20429 WBGene00013953 status:Predicted SW:Q9U1N8 protein_id:CAB63421.1 /QuerySize=362	gi|7000711|ref|Os04g08010|
lcl|ZK262.9 CE19326 WBGene00013954 status:Predicted SW:Q9XTR4 protein_id:CAB16550.1 /QuerySize=231	gi|7000712|ref|Os04g08020|
lcl|ZK265.3 CE15293 WBGene00013956 status:Partially_confirmed SW:Q94401 protein_id:CAB03515.1 /QuerySize=340	gi|7000713|ref|Os04g08040|
lcl|ZK265.6 CE15299 WBGene00013958 status:Confirmed SW:Q94402 protein_id:CAB03516.1 /QuerySize=189	gi|7000717|ref|Os04g08070|
lcl|ZK265.7 CE32551 WBGene00013959 status:Confirmed SW:Q94404 protein_id:CAB03518.2 /QuerySize=370	gi|7000720|ref|Os04g08100|
lcl|ZK285.2 CE35155 WBGene00013961 status:Predicted SW:Q7YWM2 protein_id:CAE18058.1 /QuerySize=158	gi|7000721|ref|Os04g08110|
lcl|ZK287.1 CE06610 WBGene00013962 status:Partially_confirmed SW:Q23460 protein_id:CAA94805.1 /QuerySize=497	gi|7000723|ref|Os04g08130|
lcl|ZK287.3 CE06612 WBGene00013964 status:Partially_confirmed SW:Q23455 protein_id:CAA94799.1 /QuerySize=102	gi|7000724|ref|Os04g08140|
lcl|ZK287.6 CE06615 WBGene00013966 Zinc finger	gi|7000726|ref|Os04g08160|
lcl|ZK287.7 CE27557 WBGene00013967 status:Partially_confirmed SW:Q23459 protein_id:CAA94803.2 /QuerySize=387	gi|7000728|ref|Os04g08180|
lcl|ZK287.8a CE06617 WBGene00001842 locus:her-1 HER-1 protein status:Partially_confirmed SW:P34704 protein_id:CAA94804	gi|7000732|ref|Os04g08220|
lcl|ZK287.8b CE37304 WBGene00001842 locus:her-1 status:Partially_confirmed SW:Q65ZB2 protein_id:CAH19098.1 /QuerySize=	gi|7000734|ref|Os04g08240|
lcl|ZK287.9 CE35156 WBGene00013968 status:Predicted SW:Q7YWN0 protein_id:CAE18045.1 /QuerySize=82	gi|7000735|ref|Os04g08250|
lcl|ZK337.4 CE16721 WBGene00006399 locus:tag-4 status:Predicted SW:O46017 protein_id:CAB05009.1 /QuerySize=159	gi|7000736|ref|Os04g08260|
lcl|ZK353.2 CE00386 WBGene00022698 status:Partially_confirmed SW:P34625 protein_id:AAA28199.1 /QuerySize=99	gi|7000740|ref|Os04g08300|

lcl|ZK353.3 CE00387 WBGene00022699 status:Partially_confirmed SW:P34626 protein_id:AAA28200.1 /QuerySize=164	gi|7000747|ref|Os04g08360|
lcl|ZK353.4 CE00388 WBGene00022700 status:Predicted SW:P34627 protein_id:AAA28202.1 /QuerySize=305	gi|7000753|ref|Os04g08420|
lcl|ZK353.5 CE00389 WBGene00022701 status:Predicted SW:P34628 protein_id:AAA28201.1 /QuerySize=114	gi|7000757|ref|Os04g08460|
lcl|ZK354.3 CE15316 WBGene00022706 status:Partially_confirmed SW:P91570 protein_id:AAB42258.1 /QuerySize=312	gi|7000761|ref|Os04g08500|
lcl|ZK355.2a CE33501 WBGene00022712 status:Confirmed SW:Q9N4P1 protein_id:AAO38580.1 /QuerySize=366	gi|7000768|ref|Os04g08570|
lcl|ZK355.2b CE33502 WBGene00022712 status:Confirmed SW:Q86LS7 protein_id:AAO38581.1 /QuerySize=376	gi|7000770|ref|Os04g08590|
lcl|ZK355.3 CE23458 WBGene00022713 status:Partially_confirmed SW:Q9N4P0 protein_id:AAF39997.1 /QuerySize=176	gi|7000773|ref|Os04g08620|
lcl|ZK370.6 CE00398 WBGene00022720 status:Partially_confirmed SW:Q02333 protein_id:AAA28208.1 /QuerySize=164	gi|7000774|ref|Os04g08630|
lcl|ZK380.2 CE18452 WBGene00022723 status:Predicted SW:O61762 protein_id:AAC17011.1 /QuerySize=495	gi|7000775|ref|Os04g08640|
lcl|ZK380.4 CE18453 WBGene00022724 status:Predicted SW:O61763 protein_id:AAC17012.1 /QuerySize=244	gi|7000776|ref|Os04g08650|
lcl|ZK381.4 CE25689 WBGene00003992 locus:pgl-1 status:Confirmed SW:Q9TZQ3 protein_id:AAG00057.1 /QuerySize=730	gi|7000777|ref|Os04g08660|
lcl|ZK402.1 CE05073 WBGene00022728 status:Predicted SW:Q23480 protein_id:AAA81759.1 /QuerySize=262	gi|7000780|ref|Os04g08690|
lcl|ZK418.3 CE00729 WBGene00022733 status:Partially_confirmed SW:Q23481 protein_id:AAA50687.1 /QuerySize=161	gi|7000781|ref|Os04g08700|
lcl|ZK418.4 CE25690 WBGene00003022 locus:lin-37 status:Confirmed SW:Q23482 protein_id:AAA50688.2 /QuerySize=275	gi|7000784|ref|Os04g08730|
lcl|ZK470.1 CE31268 WBGene00022744 status:Partially_confirmed SW:Q23508 protein_id:AAA80398.2 /QuerySize=494	gi|7000787|ref|Os04g08750|
lcl|ZK470.2a CE05086 WBGene00022745 status:Partially_confirmed SW:Q23505 protein_id:AAA80395.1 /QuerySize=175	gi|7000788|ref|Os04g08760|
lcl|ZK470.2b CE05087 WBGene00022745 status:Partially_confirmed SW:Q23505 protein_id:AAA80396.1 /QuerySize=78	gi|7000792|ref|Os04g09290|
lcl|ZK470.2c CE31269 WBGene00022745 status:Partially_confirmed SW:Q23503 protein_id:AAA80393.2 /QuerySize=260	gi|7000793|ref|Os04g09300|
lcl|ZK470.4 CE05089 WBGene00022746 status:Predicted SW:Q23504 protein_id:AAA80394.1 /QuerySize=83	gi|7000795|ref|Os04g09320|
lcl|ZK470.6 CE05091 WBGene00022747 status:Partially_confirmed SW:Q23509 protein_id:AAA80399.1 /QuerySize=250	gi|7000797|ref|Os04g09340|
lcl|ZK484.1 CE15337 WBGene00022748 status:Confirmed /QuerySize=80	gi|7000798|ref|Os04g09350|
lcl|ZK484.3 CE15341 WBGene00022749 status:Partially_confirmed SW:O44895 protein_id:AAK39389.1 /QuerySize=332	gi|7000803|ref|Os04g09400|
lcl|ZK484.4a CE27354 WBGene00022750 status:Partially_confirmed SW:Q95X55 protein_id:AAK39392.1 /QuerySize=919	gi|7000805|ref|Os04g09420|
lcl|ZK484.4b CE27355 WBGene00022750 status:Partially_confirmed SW:Q95X56 protein_id:AAK39393.1 /QuerySize=873	gi|7000807|ref|Os04g09440|
lcl|ZK484.5 CE15345 WBGene00022751 status:Confirmed SW:O44898 protein_id:AAK39387.1 /QuerySize=99	gi|7000808|ref|Os04g09450|
lcl|ZK507.4 CE00404 WBGene00013980 status:Predicted SW:P34636 protein_id:CAA82370.1 /QuerySize=232	gi|7000813|ref|Os04g09500|
lcl|ZK512.1 CE00407 WBGene00013982 status:Predicted SW:P34639 protein_id:CAA80144.1 /QuerySize=332	gi|7000818|ref|Os04g09550|
lcl|ZK512.10 CE35158 WBGene00013988 status:Predicted SW:Q7YTH2 protein_id:CAE18051.1 /QuerySize=63	gi|7000820|ref|Os04g09570|
lcl|ZK512.11 CE35159 WBGene00013989 status:Predicted SW:Q7YTH1 protein_id:CAE18052.1 /QuerySize=110	gi|7000821|ref|Os04g09580|
lcl|ZK512.3 CE00409 WBGene00000425 locus:ced-11 status:Partially_confirmed SW:P34641 protein_id:CAA80145.1 /QuerySize=	gi|7000825|ref|Os04g09620|
lcl|ZK512.5 CE00411 WBGene00013985 status:Partially_confirmed SW:P34643 protein_id:CAA80146.1 /QuerySize=1232	gi|7000826|ref|Os04g09630|
lcl|ZK512.7 CE00413 WBGene00013986 status:Confirmed SW:P34645 protein_id:CAA80147.1 /QuerySize=141	gi|7000827|ref|Os04g09640|
lcl|ZK512.8 CE00414 WBGene00013987 status:Confirmed SW:P34646 protein_id:CAA80149.1 /QuerySize=171	gi|7000836|ref|Os04g09730|
lcl|ZK520.2 CE33908 WBGene00013991 status:Confirmed SW:O46019 protein_id:CAB07300.4 /QuerySize=311	gi|7000838|ref|Os04g09750|
lcl|ZK525.1 CE16731 WBGene00001458 locus:flp-15 status:Confirmed SW:O46023 protein_id:CAB05022.1 /QuerySize=87	gi|7000842|ref|Os04g09790|
lcl|ZK546.1a CE32088 WBGene00006997 locus:zyg-12 cytoskeletal linker protein status:Confirmed SW:Q23529 protein_id:AAA68	gi|7000845|ref|Os04g09820|
lcl|ZK546.1b CE28524 WBGene00006997 locus:zyg-12 cytoskeletal linker protein status:Confirmed /QuerySize=777	gi|7000848|ref|Os04g09850|
lcl|ZK546.1c CE36518 WBGene00006997 locus:zyg-12 cytoskeletal linker protein status:Confirmed /QuerySize=761	gi|7000851|ref|Os04g09870|
lcl|ZK546.4 CE02918 WBGene00022761 status:Partially_confirmed SW:Q23522 protein_id:AAA68737.1 /QuerySize=151	gi|7000852|ref|Os04g09880|
lcl|ZK546.7 CE02921 WBGene00022763 status:Predicted SW:Q23518 protein_id:AAA68740.1 /QuerySize=346	gi|7000853|ref|Os04g09890|
lcl|ZK563.4a CE05095 WBGene00000524 locus:clc-3 status:Confirmed SW:Q23532 protein_id:AAA81150.1 /QuerySize=222	gi|7000856|ref|Os04g09910|
lcl|ZK563.4b CE33749 WBGene00000524 locus:clc-3 status:Confirmed SW:Q86MH7 protein_id:AAO91697.1 /QuerySize=205	gi|7000860|ref|Os04g09950|
lcl|ZK563.5b CE36352 WBGene00022769 status:Partially_confirmed SW:Q7JP77 protein_id:AAR85895.1 /QuerySize=115	gi|7000861|ref|Os04g09960|
lcl|ZK593.2 CE06623 WBGene00014002 status:Predicted SW:Q23540 protein_id:CAA93425.1 /QuerySize=205	gi|7000864|ref|Os04g09990|
lcl|ZK593.3 CE06624 WBGene00014003 status:Partially_confirmed SW:Q23537 protein_id:CAA93422.1 /QuerySize=147	gi|7000869|ref|Os04g10040|
lcl|ZK596.3 CE35705 WBGene00014008 status:Partially_confirmed SW:Q23547 protein_id:CAA93432.2 /QuerySize=322	gi|7000870|ref|Os04g10050|
lcl|ZK616.2 CE26351 WBGene00022772 status:Partially_confirmed SW:Q9N4N1 protein_id:AAF60949.1 /QuerySize=97	gi|7000874|ref|Os04g10090|
lcl|ZK616.5 CE27558 WBGene00022775 status:Partially_confirmed SW:Q965Z2 protein_id:AAK68624.1 /QuerySize=197	gi|7000879|ref|Os04g10140|
lcl|ZK617.3 CE28723 WBGene00004971 locus:spe-17 spermatogenesis-defective protein SPE-17 like status:Partially_confirmed /(	gi|7000880|ref|Os04g10150|
lcl|ZK617.4 CE35162 WBGene00014010 status:Predicted SW:Q7YWM6 protein_id:CAE18053.1 /QuerySize=178	gi|7000882|ref|Os04g10170|
lcl|ZK622.2 CE30225 WBGene00001806 locus:gur-5 status:Predicted SW:Q23553 protein_id:AAK71421.2 /QuerySize=408	gi|7000883|ref|Os04g10180|
lcl|ZK632.13 CE01711 WBGene00003035 locus:lin-52 status:Confirmed SW:Q10120 protein_id:CAA80188.1 /QuerySize=161	gi|7000886|ref|Os04g10210|
lcl|ZK632.14 CE35163 WBGene00014020 status:Partially_confirmed SW:Q7YWL9 protein_id:CAE18061.1 /QuerySize=206	gi|7000887|ref|Os04g10220|
lcl|ZK632.9 CE28193 WBGene00014016 status:Confirmed SW:P34654 protein_id:CAA80189.2 /QuerySize=62	gi|7000890|ref|Os04g10250|
lcl|ZK637.12 CE18455 WBGene00014029 status:Partially_confirmed SW:P34658 protein_id:CAA77475.1 /QuerySize=213	gi|7000894|ref|Os04g10290|
lcl|ZK637.15 CE06640 WBGene00014032 status:Predicted SW:Q23556 protein_id:CAA77457.1 /QuerySize=172	gi|7000897|ref|Os04g10320|
lcl|ZK637.4 CE00435 WBGene00014024 status:Partially_confirmed SW:P30637 protein_id:CAA77451.1 /QuerySize=95	gi|7000898|ref|Os04g10330|
lcl|ZK643.5 CE24732 WBGene00014036 status:Partially_confirmed SW:P30651 protein_id:CAA77474.2 /QuerySize=487	gi|7000899|ref|Os04g10340|
lcl|ZK643.7 CE00445 WBGene00014038 status:Predicted SW:P30653 protein_id:CAA77469.1 /QuerySize=109	gi|7000902|ref|Os04g10370|
lcl|ZK643.8 CE35164 WBGene00001734 locus:grl-25 status:Partially_confirmed SW:Q868B4 protein_id:CAD88221.2 /QuerySize=	gi|7000911|ref|Os04g10450|
lcl|ZK652.6b CE34462 WBGene00022785 status:Confirmed SW:Q7Z143 protein_id:AAP68927.1 /QuerySize=229	gi|7000913|ref|Os04g10470|
lcl|ZK652.8 CE29164 WBGene00022786 status:Partially_confirmed SW:P34665 protein_id:AAA28217.2 /QuerySize=171	gi|7000914|ref|Os04g10480|
lcl|ZK662.2 CE15377 WBGene00014039 status:Partially_confirmed SW:Q94406 protein_id:CAB01898.1 /QuerySize=222	gi|7000915|ref|Os04g10490|
lcl|ZK662.5 CE24733 WBGene00014040 status:Predicted SW:Q9U1N7 protein_id:CAB60456.1 /QuerySize=598	gi|7000916|ref|Os04g10500|
lcl|ZK666.1 CE02386 WBGene00014041 status:Predicted SW:Q23559 protein_id:CAA88981.1 /QuerySize=137	gi|7000917|ref|Os04g10510|
lcl|ZK666.11 CE35165 WBGene00014049 status:Predicted SW:Q7YWM8 protein_id:CAE18049.1 /QuerySize=141	gi|7000921|ref|Os04g10550|
lcl|ZK666.12 CE35166 WBGene00014050 status:Predicted SW:Q7YWM7 protein_id:CAE18050.1 /QuerySize=163	gi|7000922|ref|Os04g10560|
lcl|ZK666.2 CE02387 WBGene00014042 status:Partially_confirmed SW:Q23560 protein_id:CAA88982.1 /QuerySize=64	gi|7000926|ref|Os04g10600|
lcl|ZK666.4 CE02389 WBGene00014044 status:Partially_confirmed SW:Q23562 protein_id:CAA88983.1 /QuerySize=144	gi|7000932|ref|Os04g10660|
lcl|ZK669.7 CE35167 WBGene00014056 status:Predicted SW:Q7YWM5 protein_id:CAE18054.1 /QuerySize=126	gi|7000933|ref|Os04g10670|
lcl|ZK673.10 CE16744 WBGene00014064 status:Partially_confirmed SW:O18299 protein_id:CAA88484.1 /QuerySize=142	gi|7000943|ref|Os04g10760|
lcl|ZK673.11 CE33019 WBGene00014065 status:Confirmed SW:Q8I0Y7 protein_id:CAD59166.1 /QuerySize=347	gi|7000944|ref|Os04g10770|
lcl|ZK673.3 CE16743 WBGene00014059 status:Partially_confirmed SW:Q09377 protein_id:CAA88485.1 /QuerySize=322	gi|7000945|ref|Os04g10780|
lcl|ZK673.4 CE01716 WBGene00014060 status:Predicted SW:Q09663 protein_id:CAA88480.1 /QuerySize=476	gi|7000946|ref|Os04g10790|
lcl|ZK673.5 CE02394 WBGene00014061 status:Partially_confirmed SW:Q09664 protein_id:CAA88483.1 /QuerySize=146	gi|7000950|ref|Os04g10830|
lcl|ZK673.6 CE01718 WBGene00014062 status:Partially_confirmed SW:Q09378 protein_id:CAA88481.1 /QuerySize=284	gi|7000955|ref|Os04g10880|
lcl|ZK675.4 CE01723 WBGene00014067 status:Partially_confirmed SW:Q09380 protein_id:CAA86846.1 /QuerySize=196	gi|7000958|ref|Os04g10910|
lcl|ZK678.1 CE15383 WBGene00023498 locus:lin-15A LIN-15A protein status:Confirmed SW:Q27365 protein_id:CAB01904.1 /Que	gi|7000959|ref|Os04g10920|
lcl|ZK682.7 CE36080 WBGene00022790 status:Partially_confirmed SW:Q23581 protein_id:AAA82420.2 /QuerySize=250	gi|7000960|ref|Os04g10930|
lcl|ZK686.1 CE34463 WBGene00022791 ATP-dependent RNA helicase status:Confirmed SW:P34667 protein_id:AAA28221.2 /Que	gi|7000961|ref|Os04g10940|
lcl|ZK688.1 CE29165 WBGene00022796 status:Partially_confirmed SW:P34671 protein_id:AAA28226.2 /QuerySize=192	gi|7000964|ref|Os04g10970|
lcl|ZK688.4 CE00462 WBGene00022799 status:Partially_confirmed SW:P34674 protein_id:AAA28229.1 /QuerySize=67	gi|7000968|ref|Os04g11010|
lcl|ZK697.1 CE15395 WBGene00022804 status:Predicted SW:O44580 protein_id:AAC48249.1 /QuerySize=352	gi|7000969|ref|Os04g11020|
lcl|ZK697.3 CE15399 WBGene00022806 status:Predicted SW:O44575 protein_id:AAC48246.1 /QuerySize=307	gi|7000970|ref|Os04g11030|
lcl|ZK721.3 CE35818 WBGene00022810 status:Partially_confirmed SW:Q9GYF2 protein_id:AAG01565.2 /QuerySize=317	gi|7000971|ref|Os04g11040|
lcl|ZK757.3c CE24738 WBGene00006449 locus:tag-76 status:Confirmed SW:P34681 protein_id:CAB54245.1 /QuerySize=55	gi|7000972|ref|Os04g11050|
lcl|ZK783.3 CE00842 WBGene00022818 status:Predicted SW:Q23589 protein_id:AAC24420.1 /QuerySize=211	gi|7000973|ref|Os04g11060|
lcl|ZK795.2 CE18463 WBGene00014082 status:Partially_confirmed SW:O62520 protein_id:CAB05843.1 /QuerySize=171	gi|7000977|ref|Os04g11100|
lcl|ZK809.5a CE28874 WBGene00014087 status:Partially_confirmed SW:Q23600 protein_id:CAA92639.2 /QuerySize=706	gi|7000978|ref|Os04g11110|
lcl|ZK809.5b CE33911 WBGene00014087 status:Partially_confirmed SW:Q86D01 protein_id:CAD89754.1 /QuerySize=708	gi|7000979|ref|Os04g11120|
lcl|ZK809.8 CE33912 WBGene00014088 status:Confirmed SW:Q86D00 protein_id:CAD89755.1 /QuerySize=55	gi|7000981|ref|Os04g11140|
lcl|ZK813.2 CE05111 WBGene00022821 status:Partially_confirmed SW:Q23605 protein_id:AAB52653.1 /QuerySize=121	gi|7000986|ref|Os04g11190|
lcl|ZK813.3 CE29944 WBGene00022822 status:Partially_confirmed SW:Q23604 protein_id:AAB52655.2 /QuerySize=188	gi|7000990|ref|Os04g11230|
lcl|ZK813.4 CE29168 WBGene00022823 status:Confirmed SW:Q95PW1 protein_id:AAL00885.1 /QuerySize=171	gi|7000992|ref|Os04g11250|
lcl|ZK816.1 CE05114 WBGene00022825 status:Predicted SW:Q23611 protein_id:AAA82326.1 /QuerySize=104	gi|7000993|ref|Os04g11260|
lcl|ZK816.3 CE05116 WBGene00022826 status:Partially_confirmed SW:Q23609 protein_id:AAA82324.1 /QuerySize=66	gi|7000994|ref|Os04g11270|
lcl|ZK822.1 CE06644 WBGene00014089 status:Partially_confirmed SW:Q23613 protein_id:CAA98066.1 /QuerySize=881	gi|7000996|ref|Os04g11290|
lcl|ZK829.3 CE27561 WBGene00014094 status:Partially_confirmed SW:Q23620 protein_id:CAA98073.2 /QuerySize=448	gi|7000999|ref|Os04g11320|
lcl|ZK836.3 CE35171 WBGene00014099 status:Predicted SW:Q7YWM9 protein_id:CAE18048.1 /QuerySize=105	gi|7001000|ref|Os04g11330|
lcl|ZK84.1 CE02924 WBGene00022649 status:Partially_confirmed SW:Q23635 protein_id:AAC48204.1 /QuerySize=801	gi|7001005|ref|Os04g11380|
lcl|ZK84.2 CE02925 WBGene00022650 status:Partially_confirmed SW:Q23633 protein_id:AAC48205.1 /QuerySize=196	gi|7001006|ref|Os04g11390|
lcl|ZK84.3 CE36081 WBGene00002088 locus:ins-5 status:Partially_confirmed /QuerySize=84	gi|7001007|ref|Os04g11400|
lcl|ZK84.4 CE02927 WBGene00022651 status:Predicted SW:Q23632 protein_id:AAC48206.1 /QuerySize=72	gi|7001011|ref|Os04g11440|
lcl|ZK84.5 CE02928 WBGene00022652 status:Partially_confirmed SW:Q23634 protein_id:AAC48207.1 /QuerySize=84	gi|7001012|ref|Os04g11450|
lcl|ZK849.6 CE35172 WBGene00014104 status:Predicted SW:Q7YWM3 protein_id:CAE18057.1 /QuerySize=65	gi|7001014|ref|Os04g11470|
lcl|ZK856.12 CE06669 WBGene00014113 status:Partially_confirmed SW:Q23647 protein_id:CAA94860.1 /QuerySize=623	gi|7001019|ref|Os04g11520|
lcl|ZK856.14 CE37869 WBGene00044020 status:Predicted /QuerySize=178	gi|7001024|ref|Os04g11570|
lcl|ZK856.4 CE06661 WBGene00014105 status:Predicted SW:Q23638 protein_id:CAA94851.1 /QuerySize=195	gi|7001028|ref|Os04g11610|
lcl|ZK856.7 CE06664 WBGene00014108 status:Confirmed SW:Q23642 protein_id:CAA94855.1 /QuerySize=121	gi|7001030|ref|Os04g11630|
lcl|ZK858.2 CE15423 WBGene00014116 status:Partially_confirmed SW:Q94418 protein_id:CAB02137.1 /QuerySize=255	gi|7001032|ref|Os04g11650|
lcl|ZK858.8 CE35173 WBGene00014121 status:Predicted SW:Q7YTH3 protein_id:CAE18047.1 /QuerySize=239	gi|7001035|ref|Os04g11670|
lcl|ZK867.1a CE29652 WBGene00022828 zinc finger status:Partially_confirmed SW:Q95PV4 protein_id:AAL11488.1 /QuerySize=3	gi|7001042|ref|Os04g11740|
lcl|ZK867.1b CE28198 WBGene00022828 status:Confirmed SW:Q95PV3 protein_id:AAK68626.1 /QuerySize=135	gi|7001043|ref|Os04g11750|
lcl|ZK892.1e CE32783 WBGene00002266 locus:lec-3 status:Confirmed SW:Q8I4A3 protein_id:CAD57722.1 /QuerySize=45	gi|7001044|ref|Os04g11760|
lcl|ZK892.5 CE36519 WBGene00014129 status:Predicted SW:Q23657 protein_id:CAA88573.2 /QuerySize=268	gi|7001047|ref|Os04g11790|
lcl|ZK892.7 CE01729 WBGene00014131 Zinc finger	gi|7001048|ref|Os04g11800|
lcl|ZK899.1 CE01118 WBGene00014140 status:Partially_confirmed SW:Q23659 protein_id:CAA85496.1 /QuerySize=161	gi|7001052|ref|Os04g11840|
lcl|ZK899.2 CE15467 WBGene00014141 status:Partially_confirmed SW:Q23660 protein_id:CAA85502.1 /QuerySize=452	gi|7001054|ref|Os04g11860|
lcl|ZK899.3 CE01120 WBGene00014142 status:Partially_confirmed SW:Q23663 protein_id:CAA85500.1 /QuerySize=189	gi|7001055|ref|Os04g11870|
lcl|ZK899.5 CE01122 WBGene00014143 status:Partially_confirmed SW:Q23661 protein_id:CAA85498.1 /QuerySize=308	gi|7001058|ref|Os04g11900|
lcl|ZK899.6 CE01123 WBGene00014144 status:Partially_confirmed SW:Q23662 protein_id:CAA85499.1 /QuerySize=312	gi|7001062|ref|Os04g11940|
lcl|ZK899.7 CE15469 WBGene00014145 status:Partially_confirmed SW:O18306 protein_id:CAA85501.1 /QuerySize=190	gi|7001064|ref|Os04g11960|
lcl|ZK909.6 CE34468 WBGene00014150 status:Partially_confirmed SW:Q7YTT9 protein_id:CAD98732.1 /QuerySize=156	gi|7001066|ref|Os04g11980|
lcl|ZK930.3b CE37306 WBGene00014153 status:Partially_confirmed SW:Q65ZB3 protein_id:CAH19092.1 /QuerySize=199	gi|7001067|ref|Os04g11990|
lcl|ZK930.4 CE06674 WBGene00014154 status:Partially_confirmed SW:Q23666 protein_id:CAA94172.1 /QuerySize=194	gi|7001071|ref|Os04g12020|
lcl|ZK930.5 CE06675 WBGene00014155 status:Predicted SW:Q23667 protein_id:CAA94173.1 /QuerySize=212	gi|7001074|ref|Os04g12050|
lcl|ZK930.6 CE06676 WBGene00014156 status:Predicted SW:Q23668 protein_id:CAA94174.1 /QuerySize=256	gi|7001076|ref|Os04g12070|
lcl|ZK930.7 CE16752 WBGene00014157 status:Partially_confirmed SW:O46027 protein_id:CAA94176.1 /QuerySize=167	gi|7001081|ref|Os04g12120|
lcl|ZK945.4 CE35922 WBGene00014166 status:Partially_confirmed SW:Q09381 protein_id:CAA88438.2 /QuerySize=239	gi|7001083|ref|Os04g12140|
lcl|ZK945.6a CE01737 WBGene00014168 status:Partially_confirmed SW:Q09382 protein_id:CAA88439.1 /QuerySize=216	gi|7001084|ref|Os04g12150|
lcl|ZK945.6b CE35923 WBGene00014168 status:Partially_confirmed SW:Q6LAA9 protein_id:CAE48820.1 /QuerySize=214	gi|7001089|ref|Os04g12200|
lcl|ZK945.7 CE01738 WBGene00014169 status:Confirmed SW:Q09383 protein_id:CAA88440.1 /QuerySize=249	gi|7001099|ref|Os04g12300|
lcl|ZK970.7 CE02407 WBGene00014173 OV-17 antigen precursor status:Partially_confirmed SW:Q23683 protein_id:CAA88891.1	gi|7001102|ref|Os04g12330|
lcl|ZK971.1 CE02408 WBGene00014175 status:Predicted SW:Q20750 protein_id:CAA88894.1 /QuerySize=176	gi|7001103|ref|Os04g12340|

lcl|ZK973.4 CE24750 WBGene00022833 status:Predicted SW:Q9N4M2 protein_id:AAF40008.1 /QuerySize=154
lcl|ZK993.3 CE36775 WBGene00022839 status:Predicted SW:Q9N4L3 protein_id:AAF40016.2 /QuerySize=44
lcl|ZK994.6 CE15493 WBGene00022842 status:Partially_confirmed SW:O44088 protein_id:AAB88612.1 /QuerySize=113
lcl|cTel7X.1 CE20618 WBGene00008351 status:Predicted SW:Q9XXA0 protein_id:CAA20336.1 /QuerySize=86

gi|7001105|ref|Os04g12360|
gi|7001106|ref|Os04g12370|
gi|7001107|ref|Os04g12380|
gi|7001112|ref|Os04g12430|
gi|7001114|ref|Os04g12450|
gi|7001116|ref|Os04g12470|
gi|7001118|ref|Os04g12490|
gi|7001124|ref|Os04g12550|
gi|7001126|ref|Os04g12570|
gi|7001128|ref|Os04g12590|
gi|7001131|ref|Os04g12620|
gi|7001133|ref|Os04g12640|
gi|7001134|ref|Os04g12650|
gi|7001136|ref|Os04g12670|
gi|7001142|ref|Os04g12730|
gi|7001143|ref|Os04g12740|
gi|7001144|ref|Os04g12750|
gi|7001148|ref|Os04g12790|
gi|7001149|ref|Os04g12800|
gi|7001151|ref|Os04g12820|
gi|7001154|ref|Os04g12850|
gi|7001160|ref|Os04g12910|
gi|7001161|ref|Os04g12920|
gi|7001174|ref|Os04g13050|
gi|7001175|ref|Os04g13060|
gi|7001176|ref|Os04g13070|
gi|7001182|ref|Os04g13130|
gi|7001188|ref|Os04g13190|
gi|7001189|ref|Os04g13200|
gi|7001192|ref|Os04g13230|
gi|7001195|ref|Os04g13260|
gi|7001196|ref|Os04g13270|
gi|7001197|ref|Os04g13280|
gi|7001199|ref|Os04g13300|
gi|7001205|ref|Os04g13360|
gi|7001210|ref|Os04g13410|
gi|7001211|ref|Os04g13420|
gi|7001215|ref|Os04g13460|
gi|7001218|ref|Os04g13490|
gi|7001219|ref|Os04g13500|
gi|7001226|ref|Os04g13570|
gi|7001227|ref|Os04g13580|
gi|7001230|ref|Os04g13610|
gi|7001235|ref|Os04g13660|
gi|7001240|ref|Os04g13710|
gi|7001246|ref|Os04g13770|
gi|7001252|ref|Os04g13830|
gi|7001253|ref|Os04g13840|
gi|7001254|ref|Os04g13850|
gi|7001256|ref|Os04g13870|
gi|7001258|ref|Os04g13890|
gi|7001263|ref|Os04g13940|
gi|7001267|ref|Os04g13980|
gi|7001271|ref|Os04g14020|
gi|7001277|ref|Os04g14080|
gi|7001279|ref|Os04g14100|
gi|7001283|ref|Os04g14120|
gi|7001284|ref|Os04g14140|
gi|7001288|ref|Os04g14180|
gi|7001290|ref|Os04g14200|
gi|7001291|ref|Os04g14210|
gi|7001293|ref|Os04g14230|
gi|7001294|ref|Os04g14240|
gi|7001295|ref|Os04g14250|
gi|7001297|ref|Os04g14270|
gi|7001299|ref|Os04g14290|
gi|7001302|ref|Os04g14320|
gi|7001303|ref|Os04g14330|
gi|7001305|ref|Os04g14350|
gi|7001306|ref|Os04g14360|
gi|7001309|ref|Os04g14390|
gi|7001312|ref|Os04g14420|
gi|7001316|ref|Os04g14440|
gi|7001317|ref|Os04g14460|
gi|7001318|ref|Os04g14470|
gi|7001319|ref|Os04g14480|
gi|7001320|ref|Os04g14490|
gi|7001323|ref|Os04g14520|
gi|7001324|ref|Os04g14530|
gi|7001331|ref|Os04g14600|
gi|7001334|ref|Os04g14630|
gi|7001335|ref|Os04g14640|
gi|7001341|ref|Os04g14700|
gi|7001344|ref|Os04g14730|
gi|7001345|ref|Os04g14740|
gi|7001346|ref|Os04g14750|
gi|7001351|ref|Os04g14800|
gi|7001353|ref|Os04g14820|
gi|7001356|ref|Os04g14850|
gi|7001359|ref|Os04g14880|
gi|7001371|ref|Os04g15000|
gi|7001373|ref|Os04g15510|
gi|7001374|ref|Os04g15520|
gi|7001381|ref|Os04g15590|
gi|7001386|ref|Os04g15640|
gi|7001389|ref|Os04g15670|
gi|7001390|ref|Os04g15680|
gi|7001392|ref|Os04g15700|
gi|7001393|ref|Os04g15710|
gi|7001394|ref|Os04g15720|
gi|7001395|ref|Os04g15730|
gi|7001396|ref|Os04g15740|
gi|7001397|ref|Os04g15750|
gi|7001399|ref|Os04g15770|
gi|7001400|ref|Os04g15780|
gi|7001403|ref|Os04g15810|
gi|7001404|ref|Os04g15820|
gi|7001408|ref|Os04g15860|
gi|7001410|ref|Os04g15880|
gi|7001412|ref|Os04g15900|
gi|7001417|ref|Os04g15950|
gi|7001418|ref|Os04g15960|
gi|7001420|ref|Os04g15980|
gi|7001424|ref|Os04g16020|
gi|7001425|ref|Os04g16030|
gi|7001427|ref|Os04g16050|
gi|7001428|ref|Os04g16060|
gi|7001429|ref|Os04g16070|
gi|7001431|ref|Os04g16090|
gi|7001436|ref|Os04g16140|
gi|7001444|ref|Os04g16220|
gi|7001445|ref|Os04g16230|
gi|7001446|ref|Os04g16240|
gi|7001448|ref|Os04g16260|
gi|7001449|ref|Os04g16270|
gi|7001454|ref|Os04g16320|
gi|7001455|ref|Os04g16330|
gi|7001456|ref|Os04g16340|
gi|7001463|ref|Os04g16410|
gi|7001472|ref|Os04g16490|
gi|7001475|ref|Os04g16520|
gi|7001481|ref|Os04g16580|
gi|7001484|ref|Os04g16610|
gi|7001485|ref|Os04g16620|

gi|7001487|ref|Os04g16640|
gi|7001490|ref|Os04g16670|
gi|7001492|ref|Os04g16690|
gi|7001493|ref|Os04g16700|
gi|7001510|ref|Os04g16870|
gi|7001515|ref|Os04g16920|
gi|7001516|ref|Os04g16930|
gi|7001519|ref|Os04g16960|
gi|7001521|ref|Os04g16980|
gi|7001523|ref|Os04g17000|
gi|7001526|ref|Os04g17030|
gi|7001527|ref|Os04g17040|
gi|7001533|ref|Os04g17080|
gi|7001534|ref|Os04g17090|
gi|7001536|ref|Os04g17110|
gi|7001540|ref|Os04g17150|
gi|7001541|ref|Os04g17160|
gi|7001545|ref|Os04g17200|
gi|7001547|ref|Os04g17220|
gi|7001556|ref|Os04g17310|
gi|7001558|ref|Os04g17330|
gi|7001561|ref|Os04g17360|
gi|7001573|ref|Os04g17480|
gi|7001574|ref|Os04g17490|
gi|7001580|ref|Os04g17550|
gi|7001584|ref|Os04g17590|
gi|7001594|ref|Os04g17670|
gi|7001595|ref|Os04g17680|
gi|7001596|ref|Os04g17690|
gi|7001598|ref|Os04g17710|
gi|7001600|ref|Os04g17730|
gi|7001614|ref|Os04g17870|
gi|7001617|ref|Os04g17900|
gi|7001622|ref|Os04g17950|
gi|7001632|ref|Os04g18050|
gi|7001633|ref|Os04g18060|
gi|7001637|ref|Os04g18100|
gi|7001639|ref|Os04g18120|
gi|7001640|ref|Os04g18130|
gi|7001644|ref|Os04g18170|
gi|7001646|ref|Os04g18190|
gi|7001648|ref|Os04g18210|
gi|7001649|ref|Os04g18220|
gi|7001652|ref|Os04g18250|
gi|7001654|ref|Os04g18270|
gi|7001655|ref|Os04g18280|
gi|7001656|ref|Os04g18290|
gi|7001659|ref|Os04g18320|
gi|7001664|ref|Os04g18370|
gi|7001670|ref|Os04g18430|
gi|7001671|ref|Os04g18440|
gi|7001676|ref|Os04g18490|
gi|7001678|ref|Os04g18510|
gi|7001680|ref|Os04g18530|
gi|7001682|ref|Os04g18550|
gi|7001683|ref|Os04g18560|
gi|7001686|ref|Os04g18590|
gi|7001698|ref|Os04g18710|
gi|7001706|ref|Os04g18790|
gi|7001707|ref|Os04g18800|
gi|7001708|ref|Os04g18810|
gi|7001716|ref|Os04g18890|
gi|7001721|ref|Os04g18940|
gi|7001722|ref|Os04g18950|
gi|7001726|ref|Os04g18990|
gi|7001728|ref|Os04g19010|
gi|7001730|ref|Os04g19030|
gi|7001732|ref|Os04g19050|
gi|7001733|ref|Os04g19060|
gi|7001734|ref|Os04g19070|
gi|7001739|ref|Os04g19120|
gi|7001740|ref|Os04g19130|
gi|7001742|ref|Os04g19150|
gi|7001743|ref|Os04g19160|
gi|7001747|ref|Os04g19200|
gi|7001749|ref|Os04g19220|
gi|7001750|ref|Os04g19230|
gi|7001758|ref|Os04g19310|
gi|7001764|ref|Os04g19370|
gi|7001768|ref|Os04g19410|
gi|7001771|ref|Os04g19440|
gi|7001772|ref|Os04g19450|
gi|7001773|ref|Os04g19460|
gi|7001774|ref|Os04g19470|
gi|7001778|ref|Os04g19510|
gi|7001782|ref|Os04g19550|
gi|7001783|ref|Os04g19560|
gi|7001785|ref|Os04g19580|
gi|7001789|ref|Os04g19620|
gi|7001790|ref|Os04g19630|
gi|7001791|ref|Os04g19640|
gi|7001792|ref|Os04g19650|
gi|7001794|ref|Os04g19670|
gi|7001795|ref|Os04g19680|
gi|7001806|ref|Os04g19790|
gi|7001810|ref|Os04g19830|
gi|7001813|ref|Os04g19860|
gi|7001814|ref|Os04g19870|
gi|7001816|ref|Os04g19890|
gi|7001823|ref|Os04g19960|
gi|7001824|ref|Os04g19970|
gi|7001827|ref|Os04g20000|
gi|7001832|ref|Os04g20050|
gi|7001838|ref|Os04g20110|
gi|7001840|ref|Os04g20130|
gi|7001847|ref|Os04g20200|
gi|7001848|ref|Os04g20210|
gi|7001852|ref|Os04g20240|
gi|7001859|ref|Os04g20290|
gi|7001862|ref|Os04g20320|
gi|7001867|ref|Os04g20370|
gi|7001868|ref|Os04g20380|
gi|7001871|ref|Os04g20410|
gi|7001873|ref|Os04g20430|
gi|7001877|ref|Os04g20470|
gi|7001879|ref|Os04g20490|
gi|7001880|ref|Os04g20500|
gi|7001882|ref|Os04g20520|
gi|7001883|ref|Os04g20530|
gi|7001885|ref|Os04g20550|
gi|7001887|ref|Os04g20570|
gi|7001890|ref|Os04g20600|
gi|7001893|ref|Os04g20630|
gi|7001895|ref|Os04g20650|
gi|7001896|ref|Os04g20660|
gi|7001897|ref|Os04g20670|
gi|7001899|ref|Os04g20690|
gi|7001901|ref|Os04g20710|
gi|7001903|ref|Os04g20730|
gi|7001904|ref|Os04g20740|
gi|7001907|ref|Os04g20770|
gi|7001913|ref|Os04g20820|
gi|7001914|ref|Os04g20830|
gi|7001918|ref|Os04g20870|

gi|7001922|ref|Os04g20910|
gi|7001933|ref|Os04g21020|
gi|7001937|ref|Os04g21060|
gi|7001938|ref|Os04g21070|
gi|7001939|ref|Os04g21080|
gi|7001943|ref|Os04g21120|
gi|7001948|ref|Os04g21170|
gi|7001950|ref|Os04g21190|
gi|7001955|ref|Os04g21240|
gi|7001964|ref|Os04g21330|
gi|7001968|ref|Os04g21370|
gi|7001974|ref|Os04g21430|
gi|7001977|ref|Os04g21460|
gi|7001980|ref|Os04g21490|
gi|7001983|ref|Os04g21520|
gi|7001990|ref|Os04g21590|
gi|7001991|ref|Os04g21600|
gi|7001993|ref|Os04g21620|
gi|7001994|ref|Os04g21630|
gi|7001995|ref|Os04g21640|
gi|7002002|ref|Os04g21710|
gi|7002008|ref|Os04g21770|
gi|7002009|ref|Os04g21780|
gi|7002011|ref|Os04g21800|
gi|7002012|ref|Os04g21810|
gi|7002014|ref|Os04g21830|
gi|7002015|ref|Os04g21840|
gi|7002016|ref|Os04g21850|
gi|7002021|ref|Os04g21900|
gi|7002025|ref|Os04g21940|
gi|7002029|ref|Os04g21980|
gi|7002035|ref|Os04g22040|
gi|7002038|ref|Os04g22070|
gi|7002044|ref|Os04g22130|
gi|7002045|ref|Os04g22140|
gi|7002046|ref|Os04g22150|
gi|7002056|ref|Os04g22250|
gi|7002057|ref|Os04g22260|
gi|7002058|ref|Os04g22270|
gi|7002059|ref|Os04g22270|
gi|7002060|ref|Os04g22280|
gi|7002061|ref|Os04g22290|
gi|7002062|ref|Os04g22300|
gi|7002064|ref|Os04g22320|
gi|7002067|ref|Os04g22350|
gi|7002073|ref|Os04g22410|
gi|7002076|ref|Os04g22440|
gi|7002077|ref|Os04g22450|
gi|7002078|ref|Os04g22460|
gi|7002080|ref|Os04g22480|
gi|7002083|ref|Os04g22510|
gi|7002084|ref|Os04g22520|
gi|7002085|ref|Os04g22530|
gi|7002086|ref|Os04g22540|
gi|7002087|ref|Os04g22550|
gi|7002088|ref|Os04g22560|
gi|7002097|ref|Os04g22650|
gi|7002099|ref|Os04g22670|
gi|7002102|ref|Os04g22700|
gi|7002107|ref|Os04g22750|
gi|7002108|ref|Os04g22760|
gi|7002109|ref|Os04g22770|
gi|7002112|ref|Os04g22800|
gi|7002114|ref|Os04g22820|
gi|7002120|ref|Os04g22880|
gi|7002121|ref|Os04g22890|
gi|7002127|ref|Os04g22940|
gi|7002133|ref|Os04g23000|
gi|7002134|ref|Os04g23010|
gi|7002137|ref|Os04g23040|
gi|7002138|ref|Os04g23050|
gi|7002142|ref|Os04g23090|
gi|7002143|ref|Os04g23100|
gi|7002145|ref|Os04g23120|
gi|7002146|ref|Os04g23130|
gi|7002147|ref|Os04g23140|
gi|7002148|ref|Os04g23150|
gi|7002149|ref|Os04g23160|
gi|7002150|ref|Os04g23170|
gi|7002153|ref|Os04g23200|
gi|7002156|ref|Os04g23230|
gi|7002165|ref|Os04g23320|
gi|7002166|ref|Os04g23330|
gi|7002179|ref|Os04g23460|
gi|7002182|ref|Os04g23490|
gi|7002183|ref|Os04g23500|
gi|7002184|ref|Os04g23510|
gi|7002187|ref|Os04g23540|
gi|7002192|ref|Os04g23590|
gi|7002194|ref|Os04g23610|
gi|7002196|ref|Os04g23630|
gi|7002199|ref|Os04g23660|
gi|7002202|ref|Os04g23690|
gi|7002204|ref|Os04g23710|
gi|7002208|ref|Os04g23750|
gi|7002214|ref|Os04g23810|
gi|7002217|ref|Os04g23840|
gi|7002218|ref|Os04g23850|
gi|7002219|ref|Os04g23860|
gi|7002227|ref|Os04g23920|
gi|7002231|ref|Os04g23960|
gi|7002232|ref|Os04g23970|
gi|7002233|ref|Os04g23980|
gi|7002235|ref|Os04g24000|
gi|7002237|ref|Os04g24020|
gi|7002238|ref|Os04g24030|
gi|7002240|ref|Os04g24050|
gi|7002244|ref|Os04g24090|
gi|7002247|ref|Os04g24120|
gi|7002250|ref|Os04g24150|
gi|7002251|ref|Os04g24160|
gi|7002258|ref|Os04g24190|
gi|7002259|ref|Os04g24200|
gi|7002264|ref|Os04g24250|
gi|7002265|ref|Os04g24260|
gi|7002266|ref|Os04g24270|
gi|7002267|ref|Os04g24280|
gi|7002270|ref|Os04g24310|
gi|7002271|ref|Os04g24320|
gi|7002272|ref|Os04g24330|
gi|7002273|ref|Os04g24340|
gi|7002275|ref|Os04g24360|
gi|7002277|ref|Os04g24380|
gi|7002279|ref|Os04g24400|
gi|7002281|ref|Os04g24420|
gi|7002284|ref|Os04g24450|
gi|7002285|ref|Os04g24460|
gi|7002286|ref|Os04g24470|
gi|7002287|ref|Os04g24480|
gi|7002293|ref|Os04g24540|
gi|7002295|ref|Os04g24560|
gi|7002301|ref|Os04g24620|
gi|7002305|ref|Os04g24660|
gi|7002306|ref|Os04g24670|

gi|7002311|ref|Os04g24720|
gi|7002315|ref|Os04g24760|
gi|7002316|ref|Os04g24770|
gi|7002322|ref|Os04g24830|
gi|7002327|ref|Os04g24880|
gi|7002328|ref|Os04g24890|
gi|7002329|ref|Os04g24900|
gi|7002330|ref|Os04g24910|
gi|7002332|ref|Os04g24930|
gi|7002334|ref|Os04g24950|
gi|7002337|ref|Os04g24980|
gi|7002339|ref|Os04g25000|
gi|7002357|ref|Os04g25180|
gi|7002359|ref|Os04g25200|
gi|7002361|ref|Os04g25220|
gi|7002362|ref|Os04g25230|
gi|7002365|ref|Os04g25260|
gi|7002366|ref|Os04g25270|
gi|7002368|ref|Os04g25290|
gi|7002370|ref|Os04g25310|
gi|7002371|ref|Os04g25320|
gi|7002374|ref|Os04g25350|
gi|7002381|ref|Os04g25420|
gi|7002382|ref|Os04g25430|
gi|7002390|ref|Os04g25510|
gi|7002392|ref|Os04g25530|
gi|7002398|ref|Os04g25580|
gi|7002402|ref|Os04g25620|
gi|7002407|ref|Os04g25670|
gi|7002412|ref|Os04g25720|
gi|7002414|ref|Os04g25740|
gi|7002415|ref|Os04g25750|
gi|7002417|ref|Os04g25770|
gi|7002419|ref|Os04g25790|
gi|7002423|ref|Os04g25830|
gi|7002426|ref|Os04g25860|
gi|7002431|ref|Os04g25910|
gi|7002435|ref|Os04g25950|
gi|7002445|ref|Os04g26050|
gi|7002448|ref|Os04g26080|
gi|7002449|ref|Os04g26090|
gi|7002450|ref|Os04g26100|
gi|7002451|ref|Os04g26110|
gi|7002452|ref|Os04g26120|
gi|7002455|ref|Os04g26150|
gi|7002456|ref|Os04g26160|
gi|7002458|ref|Os04g26180|
gi|7002460|ref|Os04g26200|
gi|7002467|ref|Os04g26260|
gi|7002468|ref|Os04g26270|
gi|7002474|ref|Os04g26330|
gi|7002478|ref|Os04g26370|
gi|7002480|ref|Os04g26390|
gi|7002481|ref|Os04g26400|
gi|7002485|ref|Os04g26440|
gi|7002486|ref|Os04g26450|
gi|7002487|ref|Os04g26460|
gi|7002488|ref|Os04g26470|
gi|7002490|ref|Os04g26490|
gi|7002492|ref|Os04g26510|
gi|7002493|ref|Os04g26520|
gi|7002496|ref|Os04g26550|
gi|7002497|ref|Os04g26560|
gi|7002499|ref|Os04g26580|
gi|7002500|ref|Os04g26590|
gi|7002501|ref|Os04g26600|
gi|7002502|ref|Os04g26610|
gi|7002503|ref|Os04g26620|
gi|7002504|ref|Os04g26630|
gi|7002505|ref|Os04g26640|
gi|7002508|ref|Os04g26670|
gi|7002509|ref|Os04g26680|
gi|7002518|ref|Os04g26770|
gi|7002519|ref|Os04g26780|
gi|7002521|ref|Os04g26800|
gi|7002522|ref|Os04g26810|
gi|7002523|ref|Os04g26820|
gi|7002534|ref|Os04g26930|
gi|7002535|ref|Os04g26940|
gi|7002536|ref|Os04g26950|
gi|7002540|ref|Os04g26990|
gi|7002541|ref|Os04g27000|
gi|7002542|ref|Os04g27010|
gi|7002544|ref|Os04g27030|
gi|7002545|ref|Os04g27040|
gi|7002546|ref|Os04g27050|
gi|7002549|ref|Os04g27080|
gi|7002550|ref|Os04g27090|
gi|7002556|ref|Os04g27150|
gi|7002559|ref|Os04g27180|
gi|7002562|ref|Os04g27200|
gi|7002567|ref|Os04g27250|
gi|7002571|ref|Os04g27290|
gi|7002575|ref|Os04g27330|
gi|7002577|ref|Os04g27350|
gi|7002579|ref|Os04g27370|
gi|7002580|ref|Os04g27380|
gi|7002581|ref|Os04g27390|
gi|7002586|ref|Os04g27440|
gi|7002587|ref|Os04g27450|
gi|7002588|ref|Os04g27460|
gi|7002595|ref|Os04g27530|
gi|7002600|ref|Os04g27580|
gi|7002601|ref|Os04g27590|
gi|7002602|ref|Os04g27600|
gi|7002604|ref|Os04g27620|
gi|7002607|ref|Os04g27650|
gi|7002611|ref|Os04g27690|
gi|7002612|ref|Os04g27700|
gi|7002616|ref|Os04g27740|
gi|7002617|ref|Os04g27750|
gi|7002619|ref|Os04g27770|
gi|7002620|ref|Os04g27780|
gi|7002622|ref|Os04g27800|
gi|7002623|ref|Os04g27810|
gi|7002624|ref|Os04g27820|
gi|7002626|ref|Os04g27840|
gi|7002632|ref|Os04g27900|
gi|7002633|ref|Os04g27910|
gi|7002635|ref|Os04g27930|
gi|7002636|ref|Os04g27940|
gi|7002637|ref|Os04g27950|
gi|7002646|ref|Os04g28030|
gi|7002648|ref|Os04g28050|
gi|7002650|ref|Os04g28070|
gi|7002651|ref|Os04g28080|
gi|7002654|ref|Os04g28110|
gi|7002655|ref|Os04g28120|
gi|7002657|ref|Os04g28140|
gi|7002662|ref|Os04g28190|
gi|7002665|ref|Os04g28220|
gi|7002670|ref|Os04g28270|
gi|7002672|ref|Os04g28290|
gi|7002674|ref|Os04g28310|

gi|7002675|ref|Os04g28320|
gi|7002676|ref|Os04g28330|
gi|7002677|ref|Os04g28340|
gi|7002679|ref|Os04g28360|
gi|7002680|ref|Os04g28370|
gi|7002681|ref|Os04g28380|
gi|7002682|ref|Os04g28390|
gi|7002684|ref|Os04g28410|
gi|7002686|ref|Os04g28430|
gi|7002688|ref|Os04g28450|
gi|7002693|ref|Os04g28490|
gi|7002694|ref|Os04g28500|
gi|7002697|ref|Os04g28530|
gi|7002698|ref|Os04g28540|
gi|7002705|ref|Os04g28610|
gi|7002707|ref|Os04g28630|
gi|7002708|ref|Os04g28640|
gi|7002710|ref|Os04g28660|
gi|7002711|ref|Os04g28670|
gi|7002715|ref|Os04g28710|
gi|7002716|ref|Os04g28720|
gi|7002717|ref|Os04g28730|
gi|7002718|ref|Os04g28740|
gi|7002719|ref|Os04g28750|
gi|7002720|ref|Os04g28760|
gi|7002723|ref|Os04g28790|
gi|7002724|ref|Os04g28800|
gi|7002725|ref|Os04g28810|
gi|7002727|ref|Os04g28830|
gi|7002728|ref|Os04g28840|
gi|7002729|ref|Os04g28850|
gi|7002739|ref|Os04g28950|
gi|7002740|ref|Os04g28960|
gi|7002741|ref|Os04g28970|
gi|7002742|ref|Os04g28980|
gi|7002745|ref|Os04g29010|
gi|7002746|ref|Os04g29020|
gi|7002748|ref|Os04g29040|
gi|7002749|ref|Os04g29050|
gi|7002750|ref|Os04g29060|
gi|7002751|ref|Os04g29070|
gi|7002752|ref|Os04g29080|
gi|7002754|ref|Os04g29100|
gi|7002755|ref|Os04g29110|
gi|7002756|ref|Os04g29120|
gi|7002761|ref|Os04g29170|
gi|7002764|ref|Os04g29200|
gi|7002768|ref|Os04g29240|
gi|7002770|ref|Os04g29260|
gi|7002772|ref|Os04g29280|
gi|7002783|ref|Os04g29390|
gi|7002784|ref|Os04g29400|
gi|7002785|ref|Os04g29410|
gi|7002790|ref|Os04g29460|
gi|7002793|ref|Os04g29490|
gi|7002794|ref|Os04g29500|
gi|7002795|ref|Os04g29510|
gi|7002798|ref|Os04g29540|
gi|7002800|ref|Os04g29560|
gi|7002801|ref|Os04g29570|
gi|7002803|ref|Os04g29590|
gi|7002805|ref|Os04g29610|
gi|7002810|ref|Os04g29660|
gi|7002811|ref|Os04g29670|
gi|7002813|ref|Os04g29690|
gi|7002817|ref|Os04g29730|
gi|7002819|ref|Os04g29750|
gi|7002820|ref|Os04g29760|
gi|7002822|ref|Os04g29780|
gi|7002825|ref|Os04g29810|
gi|7002828|ref|Os04g29840|
gi|7002830|ref|Os04g29860|
gi|7002840|ref|Os04g29950|
gi|7002842|ref|Os04g29970|
gi|7002845|ref|Os04g30000|
gi|7002847|ref|Os04g30020|
gi|7002850|ref|Os04g30050|
gi|7002852|ref|Os04g30070|
gi|7002853|ref|Os04g30080|
gi|7002854|ref|Os04g30090|
gi|7002859|ref|Os04g30140|
gi|7002862|ref|Os04g30170|
gi|7002867|ref|Os04g30220|
gi|7002874|ref|Os04g30290|
gi|7002876|ref|Os04g30310|
gi|7002881|ref|Os04g30360|
gi|7002885|ref|Os04g30400|
gi|7002886|ref|Os04g30410|
gi|7002893|ref|Os04g30480|
gi|7002895|ref|Os04g30500|
gi|7002896|ref|Os04g30510|
gi|7002897|ref|Os04g30520|
gi|7002901|ref|Os04g30560|
gi|7002909|ref|Os04g30640|
gi|7002912|ref|Os04g30670|
gi|7002913|ref|Os04g30680|
gi|7002915|ref|Os04g30700|
gi|7002919|ref|Os04g30740|
gi|7002920|ref|Os04g30750|
gi|7002927|ref|Os04g30820|
gi|7002929|ref|Os04g30840|
gi|7002930|ref|Os04g30850|
gi|7002937|ref|Os04g30920|
gi|7002939|ref|Os04g30940|
gi|7002940|ref|Os04g30950|
gi|7002941|ref|Os04g30960|
gi|7002942|ref|Os04g30970|
gi|7002943|ref|Os04g30980|
gi|7002947|ref|Os04g31020|
gi|7002953|ref|Os04g31060|
gi|7002956|ref|Os04g31100|
gi|7002962|ref|Os04g31130|
gi|7002963|ref|Os04g31140|
gi|7002964|ref|Os04g31150|
gi|7002965|ref|Os04g31160|
gi|7002966|ref|Os04g31170|
gi|7002969|ref|Os04g31200|
gi|7002974|ref|Os04g31230|
gi|7002976|ref|Os04g31250|
gi|7002977|ref|Os04g31260|
gi|7002979|ref|Os04g31280|
gi|7002981|ref|Os04g31300|
gi|7002982|ref|Os04g31310|
gi|7002987|ref|Os04g31360|
gi|7002992|ref|Os04g31410|
gi|7002994|ref|Os04g31430|
gi|7002997|ref|Os04g31460|
gi|7002998|ref|Os04g31470|
gi|7002999|ref|Os04g31480|
gi|7003000|ref|Os04g31490|
gi|7003003|ref|Os04g31520|
gi|7003004|ref|Os04g31530|
gi|7003006|ref|Os04g31550|
gi|7003008|ref|Os04g31570|

gi|7003014|ref|Os04g31630|
gi|7003015|ref|Os04g31640|
gi|7003018|ref|Os04g31670|
gi|7003019|ref|Os04g31680|
gi|7003024|ref|Os04g31720|
gi|7003026|ref|Os04g31740|
gi|7003027|ref|Os04g31750|
gi|7003028|ref|Os04g31760|
gi|7003030|ref|Os04g31780|
gi|7003031|ref|Os04g31790|
gi|7003033|ref|Os04g31810|
gi|7003035|ref|Os04g31830|
gi|7003036|ref|Os04g31840|
gi|7003039|ref|Os04g31870|
gi|7003040|ref|Os04g31880|
gi|7003041|ref|Os04g31890|
gi|7003049|ref|Os04g31970|
gi|7003052|ref|Os04g32000|
gi|7003056|ref|Os04g32040|
gi|7003063|ref|Os04g32100|
gi|7003066|ref|Os04g32120|
gi|7003068|ref|Os04g32140|
gi|7003071|ref|Os04g32170|
gi|7003072|ref|Os04g32180|
gi|7003073|ref|Os04g32190|
gi|7003074|ref|Os04g32200|
gi|7003075|ref|Os04g32210|
gi|7003076|ref|Os04g32220|
gi|7003077|ref|Os04g32230|
gi|7003079|ref|Os04g32250|
gi|7003083|ref|Os04g32280|
gi|7003084|ref|Os04g32290|
gi|7003092|ref|Os04g32360|
gi|7003094|ref|Os04g32380|
gi|7003095|ref|Os04g32390|
gi|7003098|ref|Os04g32420|
gi|7003099|ref|Os04g32430|
gi|7003100|ref|Os04g32440|
gi|7003101|ref|Os04g32450|
gi|7003104|ref|Os04g32470|
gi|7003107|ref|Os04g32500|
gi|7003109|ref|Os04g32520|
gi|7003110|ref|Os04g32530|
gi|7003119|ref|Os04g32610|
gi|7003121|ref|Os04g32630|
gi|7003122|ref|Os04g32640|
gi|7003127|ref|Os04g32690|
gi|7003128|ref|Os04g32700|
gi|7003136|ref|Os04g32780|
gi|7003140|ref|Os04g32820|
gi|7003142|ref|Os04g32840|
gi|7003143|ref|Os04g32850|
gi|7003145|ref|Os04g32860|
gi|7003149|ref|Os04g32900|
gi|7003160|ref|Os04g32980|
gi|7003162|ref|Os04g33000|
gi|7003164|ref|Os04g33020|
gi|7003165|ref|Os04g33030|
gi|7003169|ref|Os04g33070|
gi|7003172|ref|Os04g33090|
gi|7003173|ref|Os04g33100|
gi|7003174|ref|Os04g33110|
gi|7003176|ref|Os04g33130|
gi|7003177|ref|Os04g33140|
gi|7003179|ref|Os04g33160|
gi|7003180|ref|Os04g33170|
gi|7003181|ref|Os04g33180|
gi|7003186|ref|Os04g33230|
gi|7003196|ref|Os04g33310|
gi|7003197|ref|Os04g33320|
gi|7003199|ref|Os04g33340|
gi|7003203|ref|Os04g33380|
gi|7003207|ref|Os04g33420|
gi|7003208|ref|Os04g33430|
gi|7003224|ref|Os04g33540|
gi|7003225|ref|Os04g33550|
gi|7003226|ref|Os04g33560|
gi|7003228|ref|Os04g33580|
gi|7003232|ref|Os04g33620|
gi|7003236|ref|Os04g33650|
gi|7003238|ref|Os04g33670|
gi|7003242|ref|Os04g33710|
gi|7003244|ref|Os04g33730|
gi|7003247|ref|Os04g33760|
gi|7003250|ref|Os04g33790|
gi|7003256|ref|Os04g33850|
gi|7003260|ref|Os04g33890|
gi|7003262|ref|Os04g33910|
gi|7003267|ref|Os04g33960|
gi|7003275|ref|Os04g34040|
gi|7003277|ref|Os04g34060|
gi|7003280|ref|Os04g34090|
gi|7003283|ref|Os04g34120|
gi|7003284|ref|Os04g34130|
gi|7003286|ref|Os04g34150|
gi|7003287|ref|Os04g34160|
gi|7003288|ref|Os04g34170|
gi|7003289|ref|Os04g34170|
gi|7003290|ref|Os04g34170|
gi|7003291|ref|Os04g34180|
gi|7003293|ref|Os04g34200|
gi|7003295|ref|Os04g34220|
gi|7003301|ref|Os04g34280|
gi|7003307|ref|Os04g34340|
gi|7003311|ref|Os04g34380|
gi|7003322|ref|Os04g34470|
gi|7003326|ref|Os04g34510|
gi|7003327|ref|Os04g34520|
gi|7003329|ref|Os04g34540|
gi|7003330|ref|Os04g34550|
gi|7003332|ref|Os04g34570|
gi|7003334|ref|Os04g34590|
gi|7003344|ref|Os04g34690|
gi|7003345|ref|Os04g34700|
gi|7003346|ref|Os04g34710|
gi|7003348|ref|Os04g34730|
gi|7003349|ref|Os04g34740|
gi|7003351|ref|Os04g34760|
gi|7003352|ref|Os04g34770|
gi|7003353|ref|Os04g34780|
gi|7003354|ref|Os04g34790|
gi|7003355|ref|Os04g34800|
gi|7003357|ref|Os04g34820|
gi|7003358|ref|Os04g34830|
gi|7003361|ref|Os04g34860|
gi|7003363|ref|Os04g34880|
gi|7003364|ref|Os04g34890|
gi|7003365|ref|Os04g34900|
gi|7003366|ref|Os04g34910|
gi|7003367|ref|Os04g34920|
gi|7003371|ref|Os04g34950|
gi|7003378|ref|Os04g35000|
gi|7003383|ref|Os04g35050|
gi|7003385|ref|Os04g35070|

gi|7003395|ref|Os04g35170|
gi|7003402|ref|Os04g35230|
gi|7003415|ref|Os04g35350|
gi|7003419|ref|Os04g35390|
gi|7003420|ref|Os04g35400|
gi|7003436|ref|Os04g35550|
gi|7003440|ref|Os04g35580|
gi|7003444|ref|Os04g35620|
gi|7003445|ref|Os04g35630|
gi|7003449|ref|Os04g35670|
gi|7003454|ref|Os04g35710|
gi|7003455|ref|Os04g35720|
gi|7003456|ref|Os04g35730|
gi|7003457|ref|Os04g35740|
gi|7003458|ref|Os04g35750|
gi|7003464|ref|Os04g35810|
gi|7003465|ref|Os04g35820|
gi|7003466|ref|Os04g35830|
gi|7003482|ref|Os04g35980|
gi|7003485|ref|Os04g36010|
gi|7003486|ref|Os04g36020|
gi|7003503|ref|Os04g36670|
gi|7003505|ref|Os04g36690|
gi|7003513|ref|Os04g36770|
gi|7003514|ref|Os04g36780|
gi|7003518|ref|Os04g36820|
gi|7003524|ref|Os04g36880|
gi|7003526|ref|Os04g36900|
gi|7003527|ref|Os04g36910|
gi|7003528|ref|Os04g37410|
gi|7003529|ref|Os04g37420|
gi|7003531|ref|Os04g37440|
gi|7003532|ref|Os04g37450|
gi|7003542|ref|Os04g37540|
gi|7003544|ref|Os04g37560|
gi|7003567|ref|Os04g37750|
gi|7003569|ref|Os04g37770|
gi|7003572|ref|Os04g37800|
gi|7003579|ref|Os04g37860|
gi|7003581|ref|Os04g37880|
gi|7003582|ref|Os04g37890|
gi|7003591|ref|Os04g37930|
gi|7003592|ref|Os04g37940|
gi|7003598|ref|Os04g38000|
gi|7003601|ref|Os04g38030|
gi|7003608|ref|Os04g38100|
gi|7003611|ref|Os04g38130|
gi|7003619|ref|Os04g38210|
gi|7003621|ref|Os04g38230|
gi|7003623|ref|Os04g38250|
gi|7003624|ref|Os04g38260|
gi|7003627|ref|Os04g38290|
gi|7003628|ref|Os04g38300|
gi|7003630|ref|Os04g38320|
gi|7003633|ref|Os04g38350|
gi|7003636|ref|Os04g38380|
gi|7003642|ref|Os04g38440|
gi|7003644|ref|Os04g38460|
gi|7003647|ref|Os04g38490|
gi|7003648|ref|Os04g38500|
gi|7003649|ref|Os04g38510|
gi|7003654|ref|Os04g38550|
gi|7003657|ref|Os04g38580|
gi|7003658|ref|Os04g38590|
gi|7003665|ref|Os04g38650|
gi|7003671|ref|Os04g38710|
gi|7003674|ref|Os04g38730|
gi|7003677|ref|Os04g38760|
gi|7003681|ref|Os04g38800|
gi|7003691|ref|Os04g38890|
gi|7003694|ref|Os04g38920|
gi|7003699|ref|Os04g38960|
gi|7003702|ref|Os04g38990|
gi|7003721|ref|Os04g39170|
gi|7003722|ref|Os04g39170|
gi|7003726|ref|Os04g39200|
gi|7003729|ref|Os04g39230|
gi|7003739|ref|Os04g39310|
gi|7003740|ref|Os04g39320|
gi|7003741|ref|Os04g39320|
gi|7003746|ref|Os04g39340|
gi|7003750|ref|Os04g39380|
gi|7003764|ref|Os04g39490|
gi|7003766|ref|Os04g39510|
gi|7003769|ref|Os04g39540|
gi|7003774|ref|Os04g39580|
gi|7003778|ref|Os04g39620|
gi|7003785|ref|Os04g39690|
gi|7003793|ref|Os04g39770|
gi|7003795|ref|Os04g39790|
gi|7003796|ref|Os04g39800|
gi|7003799|ref|Os04g39830|
gi|7003807|ref|Os04g39890|
gi|7003810|ref|Os04g39920|
gi|7003813|ref|Os04g39950|
gi|7003814|ref|Os04g39960|
gi|7003820|ref|Os04g40020|
gi|7003829|ref|Os04g40110|
gi|7003830|ref|Os04g40120|
gi|7003834|ref|Os04g40160|
gi|7003835|ref|Os04g40170|
gi|7003837|ref|Os04g40190|
gi|7003838|ref|Os04g40190|
gi|7003839|ref|Os04g40190|
gi|7003840|ref|Os04g40200|
gi|7003843|ref|Os04g40230|
gi|7003845|ref|Os04g40250|
gi|7003847|ref|Os04g40270|
gi|7003855|ref|Os04g40340|
gi|7003859|ref|Os04g40380|
gi|7003860|ref|Os04g40390|
gi|7003870|ref|Os04g40480|
gi|7003878|ref|Os04g40550|
gi|7003884|ref|Os04g40610|
gi|7003887|ref|Os04g40640|
gi|7003894|ref|Os04g40690|
gi|7003895|ref|Os04g40710|
gi|7003898|ref|Os04g40720|
gi|7003908|ref|Os04g40810|
gi|7003909|ref|Os04g40820|
gi|7003916|ref|Os04g40890|
gi|7003924|ref|Os04g40960|
gi|7003928|ref|Os04g41000|
gi|7003929|ref|Os04g41010|
gi|7003955|ref|Os04g41210|
gi|7003957|ref|Os04g41230|
gi|7003961|ref|Os04g41270|
gi|7003962|ref|Os04g41280|
gi|7003970|ref|Os04g41360|
gi|7003973|ref|Os04g41390|
gi|7003984|ref|Os04g41480|
gi|7003996|ref|Os04g41590|
gi|7004000|ref|Os04g41630|
gi|7004007|ref|Os04g41700|

gi|7004008|ref|Os04g41700|
gi|7004009|ref|Os04g41700|
gi|7004011|ref|Os04g41720|
gi|7004018|ref|Os04g41790|
gi|7004025|ref|Os04g41860|
gi|7004027|ref|Os04g41880|
gi|7004028|ref|Os04g41890|
gi|7004035|ref|Os04g41940|
gi|7004044|ref|Os04g42040|
gi|7004045|ref|Os04g42050|
gi|7004047|ref|Os04g42070|
gi|7004048|ref|Os04g42080|
gi|7004055|ref|Os04g42150|
gi|7004056|ref|Os04g42160|
gi|7004057|ref|Os04g42170|
gi|7004059|ref|Os04g42190|
gi|7004060|ref|Os04g42200|
gi|7004063|ref|Os04g42230|
gi|7004064|ref|Os04g42240|
gi|7004078|ref|Os04g42360|
gi|7004084|ref|Os04g42410|
gi|7004089|ref|Os04g42440|
gi|7004101|ref|Os04g42510|
gi|7004103|ref|Os04g42530|
gi|7004104|ref|Os04g42540|
gi|7004105|ref|Os04g42550|
gi|7004106|ref|Os04g42560|
gi|7004109|ref|Os04g42590|
gi|7004113|ref|Os04g42630|
gi|7004114|ref|Os04g42640|
gi|7004116|ref|Os04g42660|
gi|7004118|ref|Os04g42680|
gi|7004132|ref|Os04g42820|
gi|7004135|ref|Os04g42850|
gi|7004139|ref|Os04g42890|
gi|7004141|ref|Os04g42910|
gi|7004145|ref|Os04g42940|
gi|7004148|ref|Os04g42970|
gi|7004151|ref|Os04g43000|
gi|7004160|ref|Os04g43080|
gi|7004161|ref|Os04g43090|
gi|7004163|ref|Os04g43110|
gi|7004164|ref|Os04g43120|
gi|7004171|ref|Os04g43190|
gi|7004177|ref|Os04g43220|
gi|7004179|ref|Os04g43240|
gi|7004181|ref|Os04g43260|
gi|7004188|ref|Os04g43330|
gi|7004190|ref|Os04g43350|
gi|7004202|ref|Os04g43450|
gi|7004208|ref|Os04g43510|
gi|7004209|ref|Os04g43520|
gi|7004210|ref|Os04g43530|
gi|7004214|ref|Os04g43570|
gi|7004220|ref|Os04g43630|
gi|7004221|ref|Os04g43640|
gi|7004223|ref|Os04g43660|
gi|7004235|ref|Os04g43770|
gi|7004237|ref|Os04g43790|
gi|7004239|ref|Os04g43810|
gi|7004240|ref|Os04g43820|
gi|7004244|ref|Os04g43860|
gi|7004246|ref|Os04g43880|
gi|7004252|ref|Os04g43940|
gi|7004254|ref|Os04g43960|
gi|7004260|ref|Os04g44020|
gi|7004263|ref|Os04g44050|
gi|7004265|ref|Os04g44070|
gi|7004266|ref|Os04g44080|
gi|7004268|ref|Os04g44100|
gi|7004270|ref|Os04g44120|
gi|7004272|ref|Os04g44140|
gi|7004274|ref|Os04g44160|
gi|7004291|ref|Os04g44310|
gi|7004298|ref|Os04g44380|
gi|7004299|ref|Os04g44390|
gi|7004305|ref|Os04g44450|
gi|7004312|ref|Os04g44520|
gi|7004323|ref|Os04g44600|
gi|7004329|ref|Os04g44660|
gi|7004331|ref|Os04g44680|
gi|7004332|ref|Os04g44690|
gi|7004336|ref|Os04g44720|
gi|7004340|ref|Os04g44760|
gi|7004341|ref|Os04g44770|
gi|7004343|ref|Os04g44790|
gi|7004350|ref|Os04g44860|
gi|7004352|ref|Os04g44880|
gi|7004359|ref|Os04g44930|
gi|7004360|ref|Os04g44940|
gi|7004365|ref|Os04g44990|
gi|7004369|ref|Os04g45030|
gi|7004372|ref|Os04g45050|
gi|7004376|ref|Os04g45080|
gi|7004378|ref|Os04g45100|
gi|7004383|ref|Os04g45150|
gi|7004392|ref|Os04g45220|
gi|7004393|ref|Os04g45230|
gi|7004394|ref|Os04g45250|
gi|7004395|ref|Os04g45240|
gi|7004401|ref|Os04g45300|
gi|7004402|ref|Os04g45310|
gi|7004406|ref|Os04g45350|
gi|7004409|ref|Os04g45380|
gi|7004410|ref|Os04g45390|
gi|7004412|ref|Os04g45410|
gi|7004416|ref|Os04g45450|
gi|7004422|ref|Os04g45500|
gi|7004423|ref|Os04g45510|
gi|7004425|ref|Os04g45530|
gi|7004428|ref|Os04g45560|
gi|7004434|ref|Os04g45620|
gi|7004436|ref|Os04g45640|
gi|7004437|ref|Os04g45650|
gi|7004438|ref|Os04g45650|
gi|7004439|ref|Os04g45660|
gi|7004441|ref|Os04g45680|
gi|7004444|ref|Os04g45710|
gi|7004447|ref|Os04g45740|
gi|7004452|ref|Os04g45790|
gi|7004455|ref|Os04g45820|
gi|7004460|ref|Os04g45870|
gi|7004461|ref|Os04g45880|
gi|7004469|ref|Os04g45940|
gi|7004473|ref|Os04g45980|
gi|7004479|ref|Os04g46030|
gi|7004480|ref|Os04g46040|
gi|7004488|ref|Os04g46110|
gi|7004493|ref|Os04g46160|
gi|7004504|ref|Os04g46260|
gi|7004511|ref|Os04g46330|
gi|7004512|ref|Os04g46340|
gi|7004514|ref|Os04g46360|
gi|7004516|ref|Os04g46380|

gi|7004520|ref|Os04g46420|
gi|7004521|ref|Os04g46430|
gi|7004528|ref|Os04g46480|
gi|7004534|ref|Os04g46540|
gi|7004535|ref|Os04g46550|
gi|7004539|ref|Os04g46590|
gi|7004544|ref|Os04g46640|
gi|7004553|ref|Os04g46720|
gi|7004563|ref|Os04g46790|
gi|7004564|ref|Os04g46800|
gi|7004568|ref|Os04g46840|
gi|7004569|ref|Os04g46850|
gi|7004571|ref|Os04g46870|
gi|7004580|ref|Os04g46950|
gi|7004585|ref|Os04g47000|
gi|7004586|ref|Os04g47010|
gi|7004588|ref|Os04g47030|
gi|7004594|ref|Os04g47090|
gi|7004598|ref|Os04g47130|
gi|7004599|ref|Os04g47140|
gi|7004603|ref|Os04g47180|
gi|7004604|ref|Os04g47180|
gi|7004607|ref|Os04g47200|
gi|7004608|ref|Os04g47210|
gi|7004612|ref|Os04g47230|
gi|7004619|ref|Os04g47310|
gi|7004622|ref|Os04g47340|
gi|7004623|ref|Os04g47350|
gi|7004635|ref|Os04g47440|
gi|7004637|ref|Os04g47460|
gi|7004638|ref|Os04g47470|
gi|7004640|ref|Os04g47490|
gi|7004642|ref|Os04g47510|
gi|7004646|ref|Os04g47540|
gi|7004653|ref|Os04g47610|
gi|7004655|ref|Os04g47630|
gi|7004657|ref|Os04g47650|
gi|7004659|ref|Os04g47670|
gi|7004660|ref|Os04g47670|
gi|7004673|ref|Os04g47790|
gi|7004678|ref|Os04g47840|
gi|7004679|ref|Os04g47850|
gi|7004680|ref|Os04g47860|
gi|7004690|ref|Os04g47920|
gi|7004692|ref|Os04g47940|
gi|7004693|ref|Os04g47950|
gi|7004694|ref|Os04g47960|
gi|7004699|ref|Os04g47980|
gi|7004700|ref|Os04g48000|
gi|7004714|ref|Os04g48110|
gi|7004715|ref|Os04g48120|
gi|7004720|ref|Os04g48160|
gi|7004722|ref|Os04g48180|
gi|7004723|ref|Os04g48190|
gi|7004726|ref|Os04g48220|
gi|7004730|ref|Os04g48250|
gi|7004734|ref|Os04g48280|
gi|7004736|ref|Os04g48300|
gi|7004738|ref|Os04g48320|
gi|7004739|ref|Os04g48330|
gi|7004740|ref|Os04g48340|
gi|7004742|ref|Os04g48360|
gi|7004750|ref|Os04g48440|
gi|7004751|ref|Os04g48450|
gi|7004754|ref|Os04g48470|
gi|7004757|ref|Os04g48500|
gi|7004762|ref|Os04g48550|
gi|7004764|ref|Os04g48570|
gi|7004765|ref|Os04g48580|
gi|7004768|ref|Os04g48610|
gi|7004770|ref|Os04g48630|
gi|7004772|ref|Os04g48650|
gi|7004774|ref|Os04g48670|
gi|7004775|ref|Os04g48680|
gi|7004776|ref|Os04g48690|
gi|7004780|ref|Os04g48720|
gi|7004781|ref|Os04g48730|
gi|7004782|ref|Os04g48740|
gi|7004794|ref|Os04g48810|
gi|7004799|ref|Os04g48840|
gi|7004804|ref|Os04g48890|
gi|7004813|ref|Os04g48960|
gi|7004815|ref|Os04g48980|
gi|7004822|ref|Os04g49050|
gi|7004825|ref|Os04g49080|
gi|7004835|ref|Os04g49180|
gi|7004841|ref|Os04g49240|
gi|7004845|ref|Os04g49280|
gi|7004857|ref|Os04g49400|
gi|7004874|ref|Os04g49550|
gi|7004880|ref|Os04g49600|
gi|7004883|ref|Os04g49630|
gi|7004892|ref|Os04g49710|
gi|7004893|ref|Os04g49720|
gi|7004898|ref|Os04g49770|
gi|7004903|ref|Os04g49820|
gi|7004906|ref|Os04g49850|
gi|7004909|ref|Os04g49880|
gi|7004922|ref|Os04g49990|
gi|7004924|ref|Os04g50010|
gi|7004927|ref|Os04g50040|
gi|7004928|ref|Os04g50050|
gi|7004931|ref|Os04g50080|
gi|7004941|ref|Os04g50670|
gi|7004943|ref|Os04g50690|
gi|7004949|ref|Os04g50750|
gi|7004952|ref|Os04g50780|
gi|7004959|ref|Os04g50840|
gi|7004966|ref|Os04g50910|
gi|7004971|ref|Os04g50960|
gi|7004973|ref|Os04g50980|
gi|7004977|ref|Os04g51020|
gi|7004991|ref|Os04g51140|
gi|7005000|ref|Os04g51210|
gi|7005005|ref|Os04g51260|
gi|7005024|ref|Os04g51400|
gi|7005025|ref|Os04g51410|
gi|7005033|ref|Os04g51480|
gi|7005035|ref|Os04g51500|
gi|7005045|ref|Os04g51600|
gi|7005047|ref|Os04g51620|
gi|7005049|ref|Os04g51640|
gi|7005050|ref|Os04g51650|
gi|7005052|ref|Os04g51670|
gi|7005061|ref|Os04g51760|
gi|7005066|ref|Os04g51810|
gi|7005090|ref|Os04g52040|
gi|7005091|ref|Os04g52040|
gi|7005092|ref|Os04g52040|
gi|7005095|ref|Os04g52070|
gi|7005096|ref|Os04g52080|
gi|7005099|ref|Os04g52110|
gi|7005106|ref|Os04g52180|
gi|7005110|ref|Os04g52220|

gi|7005134|ref|Os04g52420|
gi|7005135|ref|Os04g52430|
gi|7005157|ref|Os04g52650|
gi|7005158|ref|Os04g52660|
gi|7005160|ref|Os04g52680|
gi|7005162|ref|Os04g52700|
gi|7005167|ref|Os04g52740|
gi|7005168|ref|Os04g52750|
gi|7005172|ref|Os04g52790|
gi|7005175|ref|Os04g52820|
gi|7005181|ref|Os04g52880|
gi|7005184|ref|Os04g52910|
gi|7005186|ref|Os04g52930|
gi|7005192|ref|Os04g52980|
gi|7005193|ref|Os04g52990|
gi|7005202|ref|Os04g53080|
gi|7005204|ref|Os04g53100|
gi|7005205|ref|Os04g53110|
gi|7005208|ref|Os04g53140|
gi|7005212|ref|Os04g53180|
gi|7005228|ref|Os04g53320|
gi|7005230|ref|Os04g53340|
gi|7005238|ref|Os04g53420|
gi|7005243|ref|Os04g53460|
gi|7005244|ref|Os04g53460|
gi|7005245|ref|Os04g53470|
gi|7005254|ref|Os04g53560|
gi|7005255|ref|Os04g53570|
gi|7005258|ref|Os04g53600|
gi|7005265|ref|Os04g53650|
gi|7005275|ref|Os04g53730|
gi|7005287|ref|Os04g53840|
gi|7005298|ref|Os04g53940|
gi|7005302|ref|Os04g53970|
gi|7005308|ref|Os04g54030|
gi|7005309|ref|Os04g54040|
gi|7005310|ref|Os04g54050|
gi|7005322|ref|Os04g54170|
gi|7005326|ref|Os04g54210|
gi|7005327|ref|Os04g54220|
gi|7005328|ref|Os04g54230|
gi|7005329|ref|Os04g54230|
gi|7005335|ref|Os04g54290|
gi|7005344|ref|Os04g54370|
gi|7005350|ref|Os04g54430|
gi|7005353|ref|Os04g54460|
gi|7005354|ref|Os04g54470|
gi|7005359|ref|Os04g54520|
gi|7005361|ref|Os04g54540|
gi|7005362|ref|Os04g54550|
gi|7005366|ref|Os04g54590|
gi|7005367|ref|Os04g54590|
gi|7005369|ref|Os04g54610|
gi|7005370|ref|Os04g54620|
gi|7005371|ref|Os04g54630|
gi|7005381|ref|Os04g54720|
gi|7005383|ref|Os04g54740|
gi|7005386|ref|Os04g54770|
gi|7005400|ref|Os04g54910|
gi|7005401|ref|Os04g54920|
gi|7005405|ref|Os04g54960|
gi|7005406|ref|Os04g54970|
gi|7005407|ref|Os04g54980|
gi|7005408|ref|Os04g54990|
gi|7005419|ref|Os04g55100|
gi|7005437|ref|Os04g55270|
gi|7005438|ref|Os04g55280|
gi|7005442|ref|Os04g55300|
gi|7005443|ref|Os04g55310|
gi|7005445|ref|Os04g55330|
gi|7005447|ref|Os04g55350|
gi|7005450|ref|Os04g55380|
gi|7005452|ref|Os04g55400|
gi|7005459|ref|Os04g55460|
gi|7005463|ref|Os04g55490|
gi|7005470|ref|Os04g55550|
gi|7005475|ref|Os04g55600|
gi|7005476|ref|Os04g55610|
gi|7005478|ref|Os04g55630|
gi|7005496|ref|Os04g55790|
gi|7005501|ref|Os04g55840|
gi|7005508|ref|Os04g55900|
gi|7005509|ref|Os04g55910|
gi|7005513|ref|Os04g55930|
gi|7005519|ref|Os04g55980|
gi|7005520|ref|Os04g55990|
gi|7005521|ref|Os04g56000|
gi|7005522|ref|Os04g56010|
gi|7005524|ref|Os04g56030|
gi|7005525|ref|Os04g56040|
gi|7005526|ref|Os04g56050|
gi|7005531|ref|Os04g56100|
gi|7005535|ref|Os04g56140|
gi|7005540|ref|Os04g56190|
gi|7005541|ref|Os04g56200|
gi|7005543|ref|Os04g56220|
gi|7005548|ref|Os04g56270|
gi|7005549|ref|Os04g56280|
gi|7005555|ref|Os04g56340|
gi|7005558|ref|Os04g56370|
gi|7005560|ref|Os04g56390|
gi|7005563|ref|Os04g56420|
gi|7005570|ref|Os04g56490|
gi|7005571|ref|Os04g56500|
gi|7005576|ref|Os04g56550|
gi|7005581|ref|Os04g56600|
gi|7005589|ref|Os04g56660|
gi|7005606|ref|Os04g56810|
gi|7005607|ref|Os04g56820|
gi|7005608|ref|Os04g56830|
gi|7005609|ref|Os04g56840|
gi|7005620|ref|Os04g56940|
gi|7005622|ref|Os04g56960|
gi|7005628|ref|Os04g57000|
gi|7005633|ref|Os04g57040|
gi|7005641|ref|Os04g57120|
gi|7005646|ref|Os04g57170|
gi|7005650|ref|Os04g57200|
gi|7005654|ref|Os04g57230|
gi|7005656|ref|Os04g57240|
gi|7005657|ref|Os04g57250|
gi|7005658|ref|Os04g57260|
gi|7005659|ref|Os04g57270|
gi|7005660|ref|Os04g57280|
gi|7005696|ref|Os04g57620|
gi|7005699|ref|Os04g57650|
gi|7005707|ref|Os04g57710|
gi|7005710|ref|Os04g57740|
gi|7005712|ref|Os04g57760|
gi|7005713|ref|Os04g57770|
gi|7005719|ref|Os04g57820|
gi|7005720|ref|Os04g57830|
gi|7005728|ref|Os04g57910|
gi|7005733|ref|Os04g57960|

gi|7005734|ref|Os04g57970|
gi|7005735|ref|Os04g57980|
gi|7005737|ref|Os04g57990|
gi|7005740|ref|Os04g58030|
gi|7005742|ref|Os04g58050|
gi|7005749|ref|Os04g58120|
gi|7005779|ref|Os04g58360|
gi|7005780|ref|Os04g58370|
gi|7005786|ref|Os04g58430|
gi|7005787|ref|Os04g58440|
gi|7005789|ref|Os04g58460|
gi|7005792|ref|Os04g58490|
gi|7005795|ref|Os04g58520|
gi|7005796|ref|Os04g58530|
gi|7005797|ref|Os04g58540|
gi|7005819|ref|Os04g58740|
gi|7005822|ref|Os04g58770|
gi|7005835|ref|Os04g58890|
gi|7005840|ref|Os04g58940|
gi|7005842|ref|Os04g58950|
gi|7005849|ref|Os04g59030|
gi|7005854|ref|Os04g59060|
gi|7005855|ref|Os04g59070|
gi|7005872|ref|Os04g59230|
gi|7005873|ref|Os04g59240|
gi|7005876|ref|Os04g59270|
gi|7005883|ref|Os04g59350|
gi|7005885|ref|Os04g59360|
gi|7005890|ref|Os04g59410|
gi|7005891|ref|Os04g59420|
gi|7300001|ref|Os07g01010|
gi|7300010|ref|Os07g01080|
gi|7300012|ref|Os07g01100|
gi|7300014|ref|Os07g01120|
gi|7300019|ref|Os07g01160|
gi|7300020|ref|Os07g01170|
gi|7300022|ref|Os07g01190|
gi|7300031|ref|Os07g01280|
gi|7300033|ref|Os07g01300|
gi|7300036|ref|Os07g01330|
gi|7300038|ref|Os07g01350|
gi|7300050|ref|Os07g01470|
gi|7300060|ref|Os07g01570|
gi|7300061|ref|Os07g01580|
gi|7300062|ref|Os07g01590|
gi|7300064|ref|Os07g01610|
gi|7300070|ref|Os07g01670|
gi|7300072|ref|Os07g01690|
gi|7300073|ref|Os07g01700|
gi|7300083|ref|Os07g01790|
gi|7300087|ref|Os07g01830|
gi|7300093|ref|Os07g01890|
gi|7300094|ref|Os07g01900|
gi|7300095|ref|Os07g01910|
gi|7300100|ref|Os07g01950|
gi|7300101|ref|Os07g01960|
gi|7300103|ref|Os07g01980|
gi|7300104|ref|Os07g01990|
gi|7300105|ref|Os07g02000|
gi|7300106|ref|Os07g02010|
gi|7300109|ref|Os07g02040|
gi|7300110|ref|Os07g02050|
gi|7300113|ref|Os07g02080|
gi|7300117|ref|Os07g02110|
gi|7300125|ref|Os07g02190|
gi|7300131|ref|Os07g02250|
gi|7300132|ref|Os07g02260|
gi|7300133|ref|Os07g02270|
gi|7300137|ref|Os07g02310|
gi|7300142|ref|Os07g02360|
gi|7300143|ref|Os07g02370|
gi|7300144|ref|Os07g02380|
gi|7300145|ref|Os07g02390|
gi|7300146|ref|Os07g02400|
gi|7300147|ref|Os07g02410|
gi|7300148|ref|Os07g02420|
gi|7300149|ref|Os07g02430|
gi|7300152|ref|Os07g02460|
gi|7300159|ref|Os07g02530|
gi|7300161|ref|Os07g02550|
gi|7300178|ref|Os07g02720|
gi|7300179|ref|Os07g02730|
gi|7300183|ref|Os07g02770|
gi|7300185|ref|Os07g02790|
gi|7300186|ref|Os07g02800|
gi|7300190|ref|Os07g02820|
gi|7300191|ref|Os07g02830|
gi|7300194|ref|Os07g02860|
gi|7300204|ref|Os07g02960|
gi|7300205|ref|Os07g02970|
gi|7300206|ref|Os07g02980|
gi|7300207|ref|Os07g02990|
gi|7300209|ref|Os07g03010|
gi|7300210|ref|Os07g03020|
gi|7300211|ref|Os07g03030|
gi|7300212|ref|Os07g03040|
gi|7300213|ref|Os07g03050|
gi|7300214|ref|Os07g03060|
gi|7300216|ref|Os07g03080|
gi|7300220|ref|Os07g03120|
gi|7300225|ref|Os07g03170|
gi|7300229|ref|Os07g03210|
gi|7300230|ref|Os07g03220|
gi|7300244|ref|Os07g03350|
gi|7300253|ref|Os07g03440|
gi|7300263|ref|Os07g03540|
gi|7300264|ref|Os07g03550|
gi|7300266|ref|Os07g03570|
gi|7300273|ref|Os07g03640|
gi|7300274|ref|Os07g03650|
gi|7300275|ref|Os07g03660|
gi|7300279|ref|Os07g03700|
gi|7300289|ref|Os07g03800|
gi|7300307|ref|Os07g03980|
gi|7300311|ref|Os07g04020|
gi|7300315|ref|Os07g04060|
gi|7300316|ref|Os07g04070|
gi|7300321|ref|Os07g04120|
gi|7300323|ref|Os07g04140|
gi|7300335|ref|Os07g04250|
gi|7300336|ref|Os07g04260|
gi|7300337|ref|Os07g04270|
gi|7300340|ref|Os07g04300|
gi|7300341|ref|Os07g04310|
gi|7300342|ref|Os07g04320|
gi|7300343|ref|Os07g04330|
gi|7300344|ref|Os07g04340|
gi|7300345|ref|Os07g04350|
gi|7300347|ref|Os07g04370|
gi|7300348|ref|Os07g04380|
gi|7300349|ref|Os07g04390|
gi|7300350|ref|Os07g04400|
gi|7300354|ref|Os07g04440|
gi|7300355|ref|Os07g04450|

gi|7300356|ref|Os07g04460|
gi|7300359|ref|Os07g04490|
gi|7300360|ref|Os07g04500|
gi|7300364|ref|Os07g04540|
gi|7300368|ref|Os07g04580|
gi|7300369|ref|Os07g04590|
gi|7300370|ref|Os07g04600|
gi|7300372|ref|Os07g04620|
gi|7300374|ref|Os07g04640|
gi|7300384|ref|Os07g04740|
gi|7300388|ref|Os07g04780|
gi|7300390|ref|Os07g04800|
gi|7300393|ref|Os07g04830|
gi|7300399|ref|Os07g04880|
gi|7300400|ref|Os07g04890|
gi|7300404|ref|Os07g04930|
gi|7300405|ref|Os07g04940|
gi|7300406|ref|Os07g04950|
gi|7300407|ref|Os07g04960|
gi|7300409|ref|Os07g04980|
gi|7300413|ref|Os07g05020|
gi|7300414|ref|Os07g05030|
gi|7300415|ref|Os07g05040|
gi|7300418|ref|Os07g05060|
gi|7300419|ref|Os07g05070|
gi|7300421|ref|Os07g05090|
gi|7300422|ref|Os07g05100|
gi|7300424|ref|Os07g05120|
gi|7300425|ref|Os07g05130|
gi|7300429|ref|Os07g05170|
gi|7300433|ref|Os07g05210|
gi|7300434|ref|Os07g05220|
gi|7300437|ref|Os07g05250|
gi|7300438|ref|Os07g05260|
gi|7300439|ref|Os07g05270|
gi|7300441|ref|Os07g05290|
gi|7300443|ref|Os07g05310|
gi|7300444|ref|Os07g05320|
gi|7300447|ref|Os07g05350|
gi|7300454|ref|Os07g05410|
gi|7300463|ref|Os07g05500|
gi|7300464|ref|Os07g05510|
gi|7300465|ref|Os07g05520|
gi|7300466|ref|Os07g05530|
gi|7300473|ref|Os07g05590|
gi|7300480|ref|Os07g05630|
gi|7300483|ref|Os07g05660|
gi|7300484|ref|Os07g05670|
gi|7300485|ref|Os07g05680|
gi|7300486|ref|Os07g05690|
gi|7300487|ref|Os07g05700|
gi|7300488|ref|Os07g05710|
gi|7300490|ref|Os07g05730|
gi|7300492|ref|Os07g05750|
gi|7300493|ref|Os07g05760|
gi|7300495|ref|Os07g05780|
gi|7300502|ref|Os07g05840|
gi|7300503|ref|Os07g05840|
gi|7300505|ref|Os07g05860|
gi|7300508|ref|Os07g05890|
gi|7300510|ref|Os07g05910|
gi|7300511|ref|Os07g05920|
gi|7300512|ref|Os07g05930|
gi|7300516|ref|Os07g05970|
gi|7300517|ref|Os07g05980|
gi|7300520|ref|Os07g06010|
gi|7300524|ref|Os07g06050|
gi|7300525|ref|Os07g06060|
gi|7300526|ref|Os07g06070|
gi|7300529|ref|Os07g06100|
gi|7300533|ref|Os07g06140|
gi|7300534|ref|Os07g06150|
gi|7300535|ref|Os07g06160|
gi|7300536|ref|Os07g06170|
gi|7300539|ref|Os07g06200|
gi|7300540|ref|Os07g06210|
gi|7300541|ref|Os07g06220|
gi|7300542|ref|Os07g06230|
gi|7300543|ref|Os07g06240|
gi|7300544|ref|Os07g06250|
gi|7300550|ref|Os07g06310|
gi|7300551|ref|Os07g06320|
gi|7300552|ref|Os07g06330|
gi|7300553|ref|Os07g06340|
gi|7300558|ref|Os07g06390|
gi|7300559|ref|Os07g06400|
gi|7300561|ref|Os07g06420|
gi|7300562|ref|Os07g06430|
gi|7300568|ref|Os07g06480|
gi|7300572|ref|Os07g06520|
gi|7300578|ref|Os07g06580|
gi|7300590|ref|Os07g06690|
gi|7300591|ref|Os07g06700|
gi|7300599|ref|Os07g06770|
gi|7300610|ref|Os07g06870|
gi|7300614|ref|Os07g06910|
gi|7300616|ref|Os07g06930|
gi|7300626|ref|Os07g07030|
gi|7300627|ref|Os07g07040|
gi|7300632|ref|Os07g07090|
gi|7300633|ref|Os07g07100|
gi|7300636|ref|Os07g07130|
gi|7300637|ref|Os07g07140|
gi|7300638|ref|Os07g07150|
gi|7300639|ref|Os07g07160|
gi|7300643|ref|Os07g07200|
gi|7300644|ref|Os07g07210|
gi|7300653|ref|Os07g07280|
gi|7300654|ref|Os07g07290|
gi|7300655|ref|Os07g07300|
gi|7300663|ref|Os07g07360|
gi|7300664|ref|Os07g07370|
gi|7300673|ref|Os07g07460|
gi|7300678|ref|Os07g07500|
gi|7300679|ref|Os07g07510|
gi|7300681|ref|Os07g07530|
gi|7300685|ref|Os07g07570|
gi|7300688|ref|Os07g07590|
gi|7300692|ref|Os07g07630|
gi|7300693|ref|Os07g07640|
gi|7300694|ref|Os07g07650|
gi|7300701|ref|Os07g07710|
gi|7300703|ref|Os07g07730|
gi|7300704|ref|Os07g07740|
gi|7300705|ref|Os07g07750|
gi|7300706|ref|Os07g07760|
gi|7300708|ref|Os07g07780|
gi|7300710|ref|Os07g07800|
gi|7300711|ref|Os07g07810|
gi|7300715|ref|Os07g07850|
gi|7300720|ref|Os07g07900|
gi|7300724|ref|Os07g07940|
gi|7300727|ref|Os07g07960|
gi|7300728|ref|Os07g07970|

gi|7300733|ref|Os07g08020|
gi|7300735|ref|Os07g08040|
gi|7300739|ref|Os07g08080|
gi|7300741|ref|Os07g08100|
gi|7300742|ref|Os07g08110|
gi|7300755|ref|Os07g08230|
gi|7300757|ref|Os07g08250|
gi|7300758|ref|Os07g08260|
gi|7300760|ref|Os07g08280|
gi|7300772|ref|Os07g08360|
gi|7300773|ref|Os07g08370|
gi|7300774|ref|Os07g08380|
gi|7300781|ref|Os07g08450|
gi|7300784|ref|Os07g08470|
gi|7300785|ref|Os07g08480|
gi|7300786|ref|Os07g08490|
gi|7300788|ref|Os07g08510|
gi|7300792|ref|Os07g08550|
gi|7300796|ref|Os07g08590|
gi|7300802|ref|Os07g08650|
gi|7300804|ref|Os07g08670|
gi|7300805|ref|Os07g08680|
gi|7300806|ref|Os07g08690|
gi|7300807|ref|Os07g08700|
gi|7300810|ref|Os07g08730|
gi|7300816|ref|Os07g08780|
gi|7300819|ref|Os07g08800|
gi|7300826|ref|Os07g08870|
gi|7300833|ref|Os07g08930|
gi|7300834|ref|Os07g08940|
gi|7300840|ref|Os07g08980|
gi|7300841|ref|Os07g08990|
gi|7300845|ref|Os07g09030|
gi|7300851|ref|Os07g09090|
gi|7300852|ref|Os07g09100|
gi|7300858|ref|Os07g09160|
gi|7300862|ref|Os07g09200|
gi|7300863|ref|Os07g09210|
gi|7300867|ref|Os07g09250|
gi|7300868|ref|Os07g09260|
gi|7300869|ref|Os07g09270|
gi|7300870|ref|Os07g09280|
gi|7300871|ref|Os07g09290|
gi|7300878|ref|Os07g09360|
gi|7300883|ref|Os07g09410|
gi|7300885|ref|Os07g09430|
gi|7300886|ref|Os07g09440|
gi|7300888|ref|Os07g09460|
gi|7300892|ref|Os07g09500|
gi|7300899|ref|Os07g09570|
gi|7300904|ref|Os07g09620|
gi|7300906|ref|Os07g09640|
gi|7300907|ref|Os07g09650|
gi|7300908|ref|Os07g09660|
gi|7300913|ref|Os07g09700|
gi|7300918|ref|Os07g09750|
gi|7300921|ref|Os07g09780|
gi|7300922|ref|Os07g09790|
gi|7300925|ref|Os07g09820|
gi|7300927|ref|Os07g09840|
gi|7300928|ref|Os07g09850|
gi|7300931|ref|Os07g09880|
gi|7300936|ref|Os07g09930|
gi|7300939|ref|Os07g09960|
gi|7300941|ref|Os07g09980|
gi|7300943|ref|Os07g10000|
gi|7300945|ref|Os07g10020|
gi|7300946|ref|Os07g10030|
gi|7300947|ref|Os07g10040|
gi|7300952|ref|Os07g10090|
gi|7300955|ref|Os07g10120|
gi|7300957|ref|Os07g10140|
gi|7300960|ref|Os07g10170|
gi|7300961|ref|Os07g10180|
gi|7300963|ref|Os07g10200|
gi|7300964|ref|Os07g10210|
gi|7300969|ref|Os07g10260|
gi|7300970|ref|Os07g10270|
gi|7300971|ref|Os07g10280|
gi|7300972|ref|Os07g10290|
gi|7300974|ref|Os07g10310|
gi|7300975|ref|Os07g10320|
gi|7300976|ref|Os07g10330|
gi|7300977|ref|Os07g10340|
gi|7300979|ref|Os07g10360|
gi|7300980|ref|Os07g10370|
gi|7300981|ref|Os07g10380|
gi|7300985|ref|Os07g10420|
gi|7300986|ref|Os07g10430|
gi|7300991|ref|Os07g10480|
gi|7300994|ref|Os07g10510|
gi|7301000|ref|Os07g10570|
gi|7301001|ref|Os07g10580|
gi|7301008|ref|Os07g10640|
gi|7301009|ref|Os07g10650|
gi|7301011|ref|Os07g10670|
gi|7301018|ref|Os07g10710|
gi|7301023|ref|Os07g10760|
gi|7301026|ref|Os07g10790|
gi|7301027|ref|Os07g10800|
gi|7301034|ref|Os07g10870|
gi|7301035|ref|Os07g10880|
gi|7301043|ref|Os07g10930|
gi|7301049|ref|Os07g10990|
gi|7301061|ref|Os07g11100|
gi|7301064|ref|Os07g11130|
gi|7301066|ref|Os07g11140|
gi|7301068|ref|Os07g11170|
gi|7301076|ref|Os07g11250|
gi|7301081|ref|Os07g11300|
gi|7301088|ref|Os07g11370|
gi|7301096|ref|Os07g11450|
gi|7301100|ref|Os07g11490|
gi|7301104|ref|Os07g11530|
gi|7301106|ref|Os07g11550|
gi|7301107|ref|Os07g11560|
gi|7301108|ref|Os07g11570|
gi|7301109|ref|Os07g11580|
gi|7301120|ref|Os07g11690|
gi|7301121|ref|Os07g11700|
gi|7301122|ref|Os07g11710|
gi|7301125|ref|Os07g11740|
gi|7301128|ref|Os07g11770|
gi|7301132|ref|Os07g11810|
gi|7301135|ref|Os07g11840|
gi|7301139|ref|Os07g11880|
gi|7301141|ref|Os07g11900|
gi|7301142|ref|Os07g11910|
gi|7301143|ref|Os07g11920|
gi|7301144|ref|Os07g11930|
gi|7301145|ref|Os07g11940|
gi|7301146|ref|Os07g11950|
gi|7301149|ref|Os07g11980|
gi|7301150|ref|Os07g11990|

gi|7301152|ref|Os07g12010|
gi|7301154|ref|Os07g12030|
gi|7301155|ref|Os07g12040|
gi|7301156|ref|Os07g12050|
gi|7301160|ref|Os07g12090|
gi|7301163|ref|Os07g12120|
gi|7301173|ref|Os07g12210|
gi|7301175|ref|Os07g12230|
gi|7301179|ref|Os07g12270|
gi|7301180|ref|Os07g12280|
gi|7301181|ref|Os07g12290|
gi|7301182|ref|Os07g12300|
gi|7301183|ref|Os07g12310|
gi|7301189|ref|Os07g12370|
gi|7301190|ref|Os07g12380|
gi|7301191|ref|Os07g12390|
gi|7301192|ref|Os07g12400|
gi|7301194|ref|Os07g12420|
gi|7301195|ref|Os07g12430|
gi|7301196|ref|Os07g12440|
gi|7301197|ref|Os07g12450|
gi|7301198|ref|Os07g12460|
gi|7301199|ref|Os07g12470|
gi|7301207|ref|Os07g12540|
gi|7301208|ref|Os07g12550|
gi|7301209|ref|Os07g12560|
gi|7301210|ref|Os07g12570|
gi|7301211|ref|Os07g12580|
gi|7301212|ref|Os07g12590|
gi|7301215|ref|Os07g12620|
gi|7301219|ref|Os07g12660|
gi|7301223|ref|Os07g12700|
gi|7301225|ref|Os07g12720|
gi|7301229|ref|Os07g12760|
gi|7301231|ref|Os07g12780|
gi|7301232|ref|Os07g12790|
gi|7301247|ref|Os07g12930|
gi|7301248|ref|Os07g12940|
gi|7301253|ref|Os07g12990|
gi|7301257|ref|Os07g13030|
gi|7301258|ref|Os07g13040|
gi|7301259|ref|Os07g13050|
gi|7301263|ref|Os07g13090|
gi|7301265|ref|Os07g13110|
gi|7301270|ref|Os07g13160|
gi|7301272|ref|Os07g13180|
gi|7301274|ref|Os07g13200|
gi|7301276|ref|Os07g13220|
gi|7301278|ref|Os07g13240|
gi|7301283|ref|Os07g13280|
gi|7301284|ref|Os07g13290|
gi|7301287|ref|Os07g13320|
gi|7301288|ref|Os07g13330|
gi|7301290|ref|Os07g13350|
gi|7301291|ref|Os07g13360|
gi|7301292|ref|Os07g13370|
gi|7301295|ref|Os07g13400|
gi|7301296|ref|Os07g13410|
gi|7301299|ref|Os07g13440|
gi|7301300|ref|Os07g13450|
gi|7301305|ref|Os07g13500|
gi|7301306|ref|Os07g13510|
gi|7301307|ref|Os07g13520|
gi|7301310|ref|Os07g13550|
gi|7301312|ref|Os07g13570|
gi|7301327|ref|Os07g13720|
gi|7301328|ref|Os07g13730|
gi|7301334|ref|Os07g13790|
gi|7301337|ref|Os07g13820|
gi|7301338|ref|Os07g13830|
gi|7301339|ref|Os07g13840|
gi|7301343|ref|Os07g13880|
gi|7301346|ref|Os07g13910|
gi|7301347|ref|Os07g13920|
gi|7301351|ref|Os07g13960|
gi|7301352|ref|Os07g13970|
gi|7301354|ref|Os07g13990|
gi|7301355|ref|Os07g14000|
gi|7301357|ref|Os07g14020|
gi|7301359|ref|Os07g14040|
gi|7301368|ref|Os07g14130|
gi|7301377|ref|Os07g14170|
gi|7301378|ref|Os07g14180|
gi|7301380|ref|Os07g14200|
gi|7301386|ref|Os07g14260|
gi|7301396|ref|Os07g14330|
gi|7301397|ref|Os07g14340|
gi|7301399|ref|Os07g14360|
gi|7301405|ref|Os07g14420|
gi|7301407|ref|Os07g14440|
gi|7301408|ref|Os07g14450|
gi|7301409|ref|Os07g14460|
gi|7301410|ref|Os07g14470|
gi|7301411|ref|Os07g14480|
gi|7301413|ref|Os07g14500|
gi|7301415|ref|Os07g14520|
gi|7301421|ref|Os07g14570|
gi|7301426|ref|Os07g14620|
gi|7301428|ref|Os07g14640|
gi|7301431|ref|Os07g14670|
gi|7301433|ref|Os07g14690|
gi|7301436|ref|Os07g14720|
gi|7301437|ref|Os07g14730|
gi|7301440|ref|Os07g14760|
gi|7301441|ref|Os07g14770|
gi|7301443|ref|Os07g14790|
gi|7301448|ref|Os07g14840|
gi|7301450|ref|Os07g14860|
gi|7301451|ref|Os07g14870|
gi|7301452|ref|Os07g14880|
gi|7301453|ref|Os07g14890|
gi|7301454|ref|Os07g14900|
gi|7301455|ref|Os07g14910|
gi|7301456|ref|Os07g14920|
gi|7301457|ref|Os07g14930|
gi|7301461|ref|Os07g14970|
gi|7301464|ref|Os07g15000|
gi|7301465|ref|Os07g15010|
gi|7301466|ref|Os07g15020|
gi|7301469|ref|Os07g15050|
gi|7301471|ref|Os07g15070|
gi|7301473|ref|Os07g15090|
gi|7301474|ref|Os07g15100|
gi|7301476|ref|Os07g15120|
gi|7301477|ref|Os07g15130|
gi|7301482|ref|Os07g15180|
gi|7301484|ref|Os07g15200|
gi|7301486|ref|Os07g15220|
gi|7301488|ref|Os07g15240|
gi|7301492|ref|Os07g15280|
gi|7301494|ref|Os07g15300|
gi|7301497|ref|Os07g15330|
gi|7301500|ref|Os07g15360|
gi|7301502|ref|Os07g15380|

gi|7301503|ref|Os07g15390|
gi|7301504|ref|Os07g15400|
gi|7301505|ref|Os07g15410|
gi|7301507|ref|Os07g15430|
gi|7301512|ref|Os07g15480|
gi|7301517|ref|Os07g15530|
gi|7301519|ref|Os07g15550|
gi|7301522|ref|Os07g15580|
gi|7301523|ref|Os07g15590|
gi|7301525|ref|Os07g15610|
gi|7301529|ref|Os07g15650|
gi|7301534|ref|Os07g15700|
gi|7301536|ref|Os07g15720|
gi|7301537|ref|Os07g15730|
gi|7301546|ref|Os07g15820|
gi|7301550|ref|Os07g15860|
gi|7301551|ref|Os07g15870|
gi|7301554|ref|Os07g15900|
gi|7301555|ref|Os07g15910|
gi|7301556|ref|Os07g15920|
gi|7301559|ref|Os07g15950|
gi|7301560|ref|Os07g15960|
gi|7301562|ref|Os07g15980|
gi|7301563|ref|Os07g15990|
gi|7301567|ref|Os07g16030|
gi|7301569|ref|Os07g16050|
gi|7301570|ref|Os07g16060|
gi|7301571|ref|Os07g16070|
gi|7301574|ref|Os07g16100|
gi|7301575|ref|Os07g16110|
gi|7301581|ref|Os07g16170|
gi|7301582|ref|Os07g16180|
gi|7301585|ref|Os07g16210|
gi|7301589|ref|Os07g16250|
gi|7301590|ref|Os07g16260|
gi|7301591|ref|Os07g16270|
gi|7301592|ref|Os07g16280|
gi|7301595|ref|Os07g16310|
gi|7301596|ref|Os07g16320|
gi|7301598|ref|Os07g16340|
gi|7301599|ref|Os07g16350|
gi|7301603|ref|Os07g16380|
gi|7301606|ref|Os07g16410|
gi|7301609|ref|Os07g16440|
gi|7301610|ref|Os07g16450|
gi|7301619|ref|Os07g16540|
gi|7301620|ref|Os07g16550|
gi|7301623|ref|Os07g16580|
gi|7301629|ref|Os07g16640|
gi|7301631|ref|Os07g16660|
gi|7301634|ref|Os07g16690|
gi|7301638|ref|Os07g16730|
gi|7301641|ref|Os07g16760|
gi|7301644|ref|Os07g16790|
gi|7301647|ref|Os07g16820|
gi|7301652|ref|Os07g16870|
gi|7301657|ref|Os07g16920|
gi|7301663|ref|Os07g16980|
gi|7301665|ref|Os07g17000|
gi|7301667|ref|Os07g17020|
gi|7301671|ref|Os07g17060|
gi|7301672|ref|Os07g17070|
gi|7301675|ref|Os07g17100|
gi|7301682|ref|Os07g17170|
gi|7301683|ref|Os07g17180|
gi|7301685|ref|Os07g17200|
gi|7301697|ref|Os07g17320|
gi|7301702|ref|Os07g17370|
gi|7301703|ref|Os07g17380|
gi|7301706|ref|Os07g17410|
gi|7301708|ref|Os07g17430|
gi|7301710|ref|Os07g17450|
gi|7301712|ref|Os07g17470|
gi|7301720|ref|Os07g17550|
gi|7301721|ref|Os07g17560|
gi|7301724|ref|Os07g17590|
gi|7301725|ref|Os07g17600|
gi|7301731|ref|Os07g17660|
gi|7301734|ref|Os07g17690|
gi|7301738|ref|Os07g17730|
gi|7301739|ref|Os07g17740|
gi|7301740|ref|Os07g17750|
gi|7301741|ref|Os07g17760|
gi|7301749|ref|Os07g17840|
gi|7301751|ref|Os07g17860|
gi|7301752|ref|Os07g17870|
gi|7301754|ref|Os07g17890|
gi|7301755|ref|Os07g17900|
gi|7301758|ref|Os07g17930|
gi|7301759|ref|Os07g17940|
gi|7301764|ref|Os07g17990|
gi|7301766|ref|Os07g18010|
gi|7301768|ref|Os07g18030|
gi|7301769|ref|Os07g18040|
gi|7301772|ref|Os07g18070|
gi|7301775|ref|Os07g18100|
gi|7301776|ref|Os07g18110|
gi|7301778|ref|Os07g18130|
gi|7301779|ref|Os07g18140|
gi|7301782|ref|Os07g18170|
gi|7301783|ref|Os07g18180|
gi|7301791|ref|Os07g18260|
gi|7301793|ref|Os07g18280|
gi|7301796|ref|Os07g18310|
gi|7301798|ref|Os07g18330|
gi|7301804|ref|Os07g18390|
gi|7301811|ref|Os07g18460|
gi|7301813|ref|Os07g18480|
gi|7301820|ref|Os07g18550|
gi|7301822|ref|Os07g18570|
gi|7301823|ref|Os07g18580|
gi|7301824|ref|Os07g18590|
gi|7301828|ref|Os07g18630|
gi|7301830|ref|Os07g18650|
gi|7301831|ref|Os07g18660|
gi|7301833|ref|Os07g18680|
gi|7301836|ref|Os07g18710|
gi|7301847|ref|Os07g18820|
gi|7301851|ref|Os07g18860|
gi|7301858|ref|Os07g18930|
gi|7301859|ref|Os07g18940|
gi|7301860|ref|Os07g18950|
gi|7301861|ref|Os07g18960|
gi|7301863|ref|Os07g18980|
gi|7301864|ref|Os07g18990|
gi|7301865|ref|Os07g19000|
gi|7301867|ref|Os07g19020|
gi|7301870|ref|Os07g19050|
gi|7301873|ref|Os07g19080|
gi|7301875|ref|Os07g19100|
gi|7301876|ref|Os07g19110|
gi|7301881|ref|Os07g19160|
gi|7301882|ref|Os07g19170|
gi|7301883|ref|Os07g19180|

gi|7301887|ref|Os07g19220|
gi|7301890|ref|Os07g19250|
gi|7301891|ref|Os07g19260|
gi|7301892|ref|Os07g19270|
gi|7301893|ref|Os07g19280|
gi|7301894|ref|Os07g19290|
gi|7301895|ref|Os07g19300|
gi|7301896|ref|Os07g19310|
gi|7301898|ref|Os07g19330|
gi|7301899|ref|Os07g19340|
gi|7301900|ref|Os07g19350|
gi|7301901|ref|Os07g19360|
gi|7301906|ref|Os07g19410|
gi|7301909|ref|Os07g19440|
gi|7301914|ref|Os07g19480|
gi|7301918|ref|Os07g19520|
gi|7301920|ref|Os07g19540|
gi|7301921|ref|Os07g19550|
gi|7301924|ref|Os07g19570|
gi|7301925|ref|Os07g19580|
gi|7301928|ref|Os07g20100|
gi|7301929|ref|Os07g20110|
gi|7301930|ref|Os07g20120|
gi|7301934|ref|Os07g20160|
gi|7301936|ref|Os07g20180|
gi|7301940|ref|Os07g20220|
gi|7301941|ref|Os07g20230|
gi|7301944|ref|Os07g20260|
gi|7301948|ref|Os07g20300|
gi|7301949|ref|Os07g20310|
gi|7301950|ref|Os07g20320|
gi|7301953|ref|Os07g20350|
gi|7301954|ref|Os07g20360|
gi|7301957|ref|Os07g20390|
gi|7301958|ref|Os07g20400|
gi|7301965|ref|Os07g20470|
gi|7301966|ref|Os07g20480|
gi|7301970|ref|Os07g20520|
gi|7301971|ref|Os07g20530|
gi|7301975|ref|Os07g20570|
gi|7301979|ref|Os07g20600|
gi|7301983|ref|Os07g20640|
gi|7301984|ref|Os07g20650|
gi|7301989|ref|Os07g20700|
gi|7301995|ref|Os07g20760|
gi|7302003|ref|Os07g20840|
gi|7302006|ref|Os07g20870|
gi|7302009|ref|Os07g20900|
gi|7302011|ref|Os07g20920|
gi|7302017|ref|Os07g21960|
gi|7302024|ref|Os07g22030|
gi|7302025|ref|Os07g22040|
gi|7302026|ref|Os07g22050|
gi|7302028|ref|Os07g22070|
gi|7302033|ref|Os07g22120|
gi|7302034|ref|Os07g22130|
gi|7302035|ref|Os07g22140|
gi|7302036|ref|Os07g22150|
gi|7302037|ref|Os07g22160|
gi|7302042|ref|Os07g22210|
gi|7302044|ref|Os07g22230|
gi|7302045|ref|Os07g22240|
gi|7302046|ref|Os07g22250|
gi|7302047|ref|Os07g22260|
gi|7302051|ref|Os07g22300|
gi|7302055|ref|Os07g22340|
gi|7302057|ref|Os07g22360|
gi|7302058|ref|Os07g22370|
gi|7302059|ref|Os07g22380|
gi|7302066|ref|Os07g22450|
gi|7302067|ref|Os07g22460|
gi|7302068|ref|Os07g22470|
gi|7302070|ref|Os07g22490|
gi|7302071|ref|Os07g22500|
gi|7302072|ref|Os07g22500|
gi|7302079|ref|Os07g22570|
gi|7302086|ref|Os07g22630|
gi|7302092|ref|Os07g22690|
gi|7302107|ref|Os07g22830|
gi|7302111|ref|Os07g22870|
gi|7302113|ref|Os07g22890|
gi|7302114|ref|Os07g22900|
gi|7302116|ref|Os07g22920|
gi|7302118|ref|Os07g22940|
gi|7302122|ref|Os07g22980|
gi|7302123|ref|Os07g22990|
gi|7302124|ref|Os07g23000|
gi|7302125|ref|Os07g23010|
gi|7302130|ref|Os07g23060|
gi|7302132|ref|Os07g23080|
gi|7302134|ref|Os07g23100|
gi|7302137|ref|Os07g23130|
gi|7302138|ref|Os07g23140|
gi|7302140|ref|Os07g23160|
gi|7302141|ref|Os07g23170|
gi|7302147|ref|Os07g23230|
gi|7302149|ref|Os07g23250|
gi|7302150|ref|Os07g23260|
gi|7302152|ref|Os07g23280|
gi|7302166|ref|Os07g23420|
gi|7302173|ref|Os07g23490|
gi|7302174|ref|Os07g23500|
gi|7302175|ref|Os07g23510|
gi|7302179|ref|Os07g23530|
gi|7302182|ref|Os07g23550|
gi|7302183|ref|Os07g23560|
gi|7302186|ref|Os07g23580|
gi|7302187|ref|Os07g23590|
gi|7302192|ref|Os07g23640|
gi|7302194|ref|Os07g23660|
gi|7302195|ref|Os07g23670|
gi|7302196|ref|Os07g23680|
gi|7302205|ref|Os07g23770|
gi|7302206|ref|Os07g23780|
gi|7302209|ref|Os07g23810|
gi|7302214|ref|Os07g23860|
gi|7302215|ref|Os07g23870|
gi|7302219|ref|Os07g23910|
gi|7302222|ref|Os07g23940|
gi|7302225|ref|Os07g23970|
gi|7302229|ref|Os07g24010|
gi|7302231|ref|Os07g24030|
gi|7302232|ref|Os07g24040|
gi|7302235|ref|Os07g24070|
gi|7302241|ref|Os07g24130|
gi|7302242|ref|Os07g24140|
gi|7302243|ref|Os07g24150|
gi|7302244|ref|Os07g24160|
gi|7302245|ref|Os07g24170|
gi|7302246|ref|Os07g24180|
gi|7302249|ref|Os07g24200|
gi|7302250|ref|Os07g24210|
gi|7302251|ref|Os07g24220|
gi|7302255|ref|Os07g24260|

gi|7302256|ref|Os07g24270|
gi|7302259|ref|Os07g24300|
gi|7302266|ref|Os07g24370|
gi|7302267|ref|Os07g24380|
gi|7302271|ref|Os07g24420|
gi|7302272|ref|Os07g24430|
gi|7302276|ref|Os07g24470|
gi|7302277|ref|Os07g24480|
gi|7302278|ref|Os07g24490|
gi|7302279|ref|Os07g24500|
gi|7302280|ref|Os07g24510|
gi|7302284|ref|Os07g24550|
gi|7302286|ref|Os07g24570|
gi|7302287|ref|Os07g24580|
gi|7302290|ref|Os07g24610|
gi|7302292|ref|Os07g24630|
gi|7302297|ref|Os07g24680|
gi|7302300|ref|Os07g24710|
gi|7302302|ref|Os07g24730|
gi|7302307|ref|Os07g24780|
gi|7302311|ref|Os07g24820|
gi|7302312|ref|Os07g24830|
gi|7302316|ref|Os07g24870|
gi|7302318|ref|Os07g24890|
gi|7302322|ref|Os07g24930|
gi|7302331|ref|Os07g25020|
gi|7302334|ref|Os07g25050|
gi|7302335|ref|Os07g25060|
gi|7302336|ref|Os07g25070|
gi|7302343|ref|Os07g25140|
gi|7302346|ref|Os07g25170|
gi|7302347|ref|Os07g25180|
gi|7302351|ref|Os07g25220|
gi|7302354|ref|Os07g25250|
gi|7302355|ref|Os07g25260|
gi|7302358|ref|Os07g25290|
gi|7302359|ref|Os07g25300|
gi|7302361|ref|Os07g25320|
gi|7302367|ref|Os07g25380|
gi|7302379|ref|Os07g25480|
gi|7302380|ref|Os07g25490|
gi|7302382|ref|Os07g25510|
gi|7302383|ref|Os07g25520|
gi|7302384|ref|Os07g25530|
gi|7302388|ref|Os07g25570|
gi|7302389|ref|Os07g25580|
gi|7302391|ref|Os07g25600|
gi|7302392|ref|Os07g25610|
gi|7302396|ref|Os07g25650|
gi|7302397|ref|Os07g25660|
gi|7302398|ref|Os07g25670|
gi|7302405|ref|Os07g25720|
gi|7302406|ref|Os07g25730|
gi|7302408|ref|Os07g25750|
gi|7302410|ref|Os07g25770|
gi|7302411|ref|Os07g25780|
gi|7302412|ref|Os07g25790|
gi|7302414|ref|Os07g25810|
gi|7302415|ref|Os07g25820|
gi|7302416|ref|Os07g25830|
gi|7302421|ref|Os07g25880|
gi|7302423|ref|Os07g25900|
gi|7302425|ref|Os07g25920|
gi|7302426|ref|Os07g25930|
gi|7302427|ref|Os07g25940|
gi|7302429|ref|Os07g25960|
gi|7302430|ref|Os07g25970|
gi|7302431|ref|Os07g25980|
gi|7302434|ref|Os07g26010|
gi|7302435|ref|Os07g26020|
gi|7302438|ref|Os07g26050|
gi|7302439|ref|Os07g26060|
gi|7302443|ref|Os07g26100|
gi|7302446|ref|Os07g26130|
gi|7302447|ref|Os07g26140|
gi|7302452|ref|Os07g26190|
gi|7302453|ref|Os07g26200|
gi|7302456|ref|Os07g26230|
gi|7302457|ref|Os07g26240|
gi|7302458|ref|Os07g26250|
gi|7302461|ref|Os07g26280|
gi|7302463|ref|Os07g26300|
gi|7302464|ref|Os07g26310|
gi|7302465|ref|Os07g26320|
gi|7302470|ref|Os07g26370|
gi|7302471|ref|Os07g26380|
gi|7302473|ref|Os07g26400|
gi|7302474|ref|Os07g26410|
gi|7302475|ref|Os07g26420|
gi|7302478|ref|Os07g26450|
gi|7302484|ref|Os07g26500|
gi|7302486|ref|Os07g26520|
gi|7302487|ref|Os07g26530|
gi|7302489|ref|Os07g26550|
gi|7302491|ref|Os07g26570|
gi|7302493|ref|Os07g26590|
gi|7302494|ref|Os07g26600|
gi|7302496|ref|Os07g26620|
gi|7302499|ref|Os07g26650|
gi|7302501|ref|Os07g26670|
gi|7302502|ref|Os07g26680|
gi|7302505|ref|Os07g26710|
gi|7302509|ref|Os07g26750|
gi|7302510|ref|Os07g26760|
gi|7302511|ref|Os07g26770|
gi|7302513|ref|Os07g26790|
gi|7302519|ref|Os07g26850|
gi|7302520|ref|Os07g26860|
gi|7302522|ref|Os07g26880|
gi|7302523|ref|Os07g26890|
gi|7302525|ref|Os07g26910|
gi|7302530|ref|Os07g26950|
gi|7302531|ref|Os07g26960|
gi|7302532|ref|Os07g26970|
gi|7302533|ref|Os07g26980|
gi|7302534|ref|Os07g26990|
gi|7302539|ref|Os07g27040|
gi|7302540|ref|Os07g27050|
gi|7302541|ref|Os07g27060|
gi|7302545|ref|Os07g27100|
gi|7302547|ref|Os07g27120|
gi|7302548|ref|Os07g27120|
gi|7302549|ref|Os07g27120|
gi|7302550|ref|Os07g27130|
gi|7302554|ref|Os07g27170|
gi|7302556|ref|Os07g27190|
gi|7302561|ref|Os07g27240|
gi|7302562|ref|Os07g27250|
gi|7302563|ref|Os07g27260|
gi|7302566|ref|Os07g27290|
gi|7302574|ref|Os07g27360|
gi|7302576|ref|Os07g27380|
gi|7302577|ref|Os07g27390|
gi|7302578|ref|Os07g27400|

gi|7302579|ref|Os07g27410|
gi|7302582|ref|Os07g27440|
gi|7302589|ref|Os07g27500|
gi|7302592|ref|Os07g27530|
gi|7302594|ref|Os07g27550|
gi|7302600|ref|Os07g27610|
gi|7302601|ref|Os07g27620|
gi|7302602|ref|Os07g27630|
gi|7302604|ref|Os07g27650|
gi|7302607|ref|Os07g27680|
gi|7302610|ref|Os07g27710|
gi|7302626|ref|Os07g27860|
gi|7302632|ref|Os07g27920|
gi|7302637|ref|Os07g27960|
gi|7302639|ref|Os07g27980|
gi|7302640|ref|Os07g27990|
gi|7302642|ref|Os07g28010|
gi|7302643|ref|Os07g28020|
gi|7302648|ref|Os07g28070|
gi|7302649|ref|Os07g28080|
gi|7302651|ref|Os07g28100|
gi|7302655|ref|Os07g28140|
gi|7302658|ref|Os07g28170|
gi|7302659|ref|Os07g28180|
gi|7302670|ref|Os07g28290|
gi|7302673|ref|Os07g28320|
gi|7302674|ref|Os07g28330|
gi|7302675|ref|Os07g28340|
gi|7302676|ref|Os07g28350|
gi|7302682|ref|Os07g28410|
gi|7302683|ref|Os07g28420|
gi|7302691|ref|Os07g28500|
gi|7302696|ref|Os07g28550|
gi|7302699|ref|Os07g28580|
gi|7302706|ref|Os07g28640|
gi|7302708|ref|Os07g28660|
gi|7302710|ref|Os07g28680|
gi|7302711|ref|Os07g28690|
gi|7302712|ref|Os07g28700|
gi|7302714|ref|Os07g28720|
gi|7302723|ref|Os07g28810|
gi|7302725|ref|Os07g28830|
gi|7302728|ref|Os07g28860|
gi|7302729|ref|Os07g28870|
gi|7302730|ref|Os07g28880|
gi|7302733|ref|Os07g28910|
gi|7302738|ref|Os07g28960|
gi|7302739|ref|Os07g28970|
gi|7302740|ref|Os07g28980|
gi|7302741|ref|Os07g28990|
gi|7302744|ref|Os07g29020|
gi|7302746|ref|Os07g29040|
gi|7302747|ref|Os07g29050|
gi|7302751|ref|Os07g29090|
gi|7302753|ref|Os07g29110|
gi|7302757|ref|Os07g29150|
gi|7302761|ref|Os07g29190|
gi|7302767|ref|Os07g29250|
gi|7302769|ref|Os07g29270|
gi|7302770|ref|Os07g29280|
gi|7302772|ref|Os07g29300|
gi|7302779|ref|Os07g29340|
gi|7302780|ref|Os07g29350|
gi|7302782|ref|Os07g29370|
gi|7302783|ref|Os07g29380|
gi|7302785|ref|Os07g29400|
gi|7302788|ref|Os07g29430|
gi|7302796|ref|Os07g29510|
gi|7302798|ref|Os07g29530|
gi|7302801|ref|Os07g29560|
gi|7302803|ref|Os07g29580|
gi|7302814|ref|Os07g29660|
gi|7302815|ref|Os07g29670|
gi|7302816|ref|Os07g29680|
gi|7302817|ref|Os07g29690|
gi|7302818|ref|Os07g29700|
gi|7302819|ref|Os07g29710|
gi|7302820|ref|Os07g29720|
gi|7302828|ref|Os07g29800|
gi|7302835|ref|Os07g29870|
gi|7302837|ref|Os07g29890|
gi|7302838|ref|Os07g29900|
gi|7302839|ref|Os07g29910|
gi|7302840|ref|Os07g29920|
gi|7302841|ref|Os07g29930|
gi|7302843|ref|Os07g29950|
gi|7302845|ref|Os07g29970|
gi|7302849|ref|Os07g30010|
gi|7302851|ref|Os07g30030|
gi|7302854|ref|Os07g30060|
gi|7302860|ref|Os07g30120|
gi|7302876|ref|Os07g30260|
gi|7302878|ref|Os07g30280|
gi|7302879|ref|Os07g30290|
gi|7302890|ref|Os07g30390|
gi|7302891|ref|Os07g30400|
gi|7302893|ref|Os07g30420|
gi|7302895|ref|Os07g30440|
gi|7302896|ref|Os07g30450|
gi|7302900|ref|Os07g30490|
gi|7302901|ref|Os07g30500|
gi|7302903|ref|Os07g30520|
gi|7302905|ref|Os07g30540|
gi|7302907|ref|Os07g30560|
gi|7302914|ref|Os07g30630|
gi|7302919|ref|Os07g30680|
gi|7302922|ref|Os07g30710|
gi|7302938|ref|Os07g30860|
gi|7302939|ref|Os07g30870|
gi|7302944|ref|Os07g30920|
gi|7302952|ref|Os07g31000|
gi|7302953|ref|Os07g31010|
gi|7302954|ref|Os07g31020|
gi|7302955|ref|Os07g31030|
gi|7302960|ref|Os07g31080|
gi|7302961|ref|Os07g31090|
gi|7302962|ref|Os07g31100|
gi|7302964|ref|Os07g31120|
gi|7302967|ref|Os07g31150|
gi|7302968|ref|Os07g31160|
gi|7302970|ref|Os07g31180|
gi|7302972|ref|Os07g31200|
gi|7302974|ref|Os07g31220|
gi|7302975|ref|Os07g31230|
gi|7302984|ref|Os07g31320|
gi|7302985|ref|Os07g31330|
gi|7302988|ref|Os07g31350|
gi|7302989|ref|Os07g31360|
gi|7302994|ref|Os07g31410|
gi|7303002|ref|Os07g31480|
gi|7303005|ref|Os07g31510|
gi|7303010|ref|Os07g31560|
gi|7303011|ref|Os07g31570|
gi|7303014|ref|Os07g31600|

gi|7303021|ref|Os07g31670|
gi|7303025|ref|Os07g31710|
gi|7303027|ref|Os07g31730|
gi|7303028|ref|Os07g31740|
gi|7303030|ref|Os07g31760|
gi|7303036|ref|Os07g31820|
gi|7303047|ref|Os07g31930|
gi|7303049|ref|Os07g31950|
gi|7303054|ref|Os07g32000|
gi|7303057|ref|Os07g32030|
gi|7303059|ref|Os07g32050|
gi|7303061|ref|Os07g32070|
gi|7303063|ref|Os07g32090|
gi|7303067|ref|Os07g32130|
gi|7303068|ref|Os07g32140|
gi|7303069|ref|Os07g32150|
gi|7303070|ref|Os07g32160|
gi|7303071|ref|Os07g32170|
gi|7303076|ref|Os07g32210|
gi|7303083|ref|Os07g32280|
gi|7303084|ref|Os07g32290|
gi|7303092|ref|Os07g32360|
gi|7303100|ref|Os07g32440|
gi|7303103|ref|Os07g32470|
gi|7303106|ref|Os07g32500|
gi|7303108|ref|Os07g32520|
gi|7303110|ref|Os07g32540|
gi|7303120|ref|Os07g32640|
gi|7303124|ref|Os07g32680|
gi|7303126|ref|Os07g32700|
gi|7303127|ref|Os07g32710|
gi|7303134|ref|Os07g32780|
gi|7303140|ref|Os07g32820|
gi|7303141|ref|Os07g32830|
gi|7303142|ref|Os07g32840|
gi|7303143|ref|Os07g32850|
gi|7303145|ref|Os07g32870|
gi|7303150|ref|Os07g32920|
gi|7303151|ref|Os07g32930|
gi|7303152|ref|Os07g32940|
gi|7303154|ref|Os07g32960|
gi|7303155|ref|Os07g32970|
gi|7303156|ref|Os07g32980|
gi|7303157|ref|Os07g32990|
gi|7303160|ref|Os07g33020|
gi|7303161|ref|Os07g33030|
gi|7303162|ref|Os07g33040|
gi|7303164|ref|Os07g33060|
gi|7303165|ref|Os07g33070|
gi|7303166|ref|Os07g33080|
gi|7303167|ref|Os07g33090|
gi|7303171|ref|Os07g33120|
gi|7303177|ref|Os07g33180|
gi|7303187|ref|Os07g33280|
gi|7303188|ref|Os07g33290|
gi|7303189|ref|Os07g33300|
gi|7303191|ref|Os07g33320|
gi|7303203|ref|Os07g33430|
gi|7303206|ref|Os07g33460|
gi|7303207|ref|Os07g33470|
gi|7303211|ref|Os07g33490|
gi|7303213|ref|Os07g33510|
gi|7303214|ref|Os07g33520|
gi|7303215|ref|Os07g33530|
gi|7303226|ref|Os07g33640|
gi|7303227|ref|Os07g33650|
gi|7303228|ref|Os07g33650|
gi|7303229|ref|Os07g33650|
gi|7303239|ref|Os07g33750|
gi|7303241|ref|Os07g33770|
gi|7303244|ref|Os07g33800|
gi|7303245|ref|Os07g33810|
gi|7303253|ref|Os07g33890|
gi|7303259|ref|Os07g33940|
gi|7303264|ref|Os07g33990|
gi|7303267|ref|Os07g34020|
gi|7303271|ref|Os07g34060|
gi|7303275|ref|Os07g34100|
gi|7303283|ref|Os07g34180|
gi|7303287|ref|Os07g34220|
gi|7303292|ref|Os07g34270|
gi|7303294|ref|Os07g34290|
gi|7303298|ref|Os07g34330|
gi|7303300|ref|Os07g34350|
gi|7303301|ref|Os07g34360|
gi|7303303|ref|Os07g34380|
gi|7303308|ref|Os07g34430|
gi|7303311|ref|Os07g34460|
gi|7303312|ref|Os07g34470|
gi|7303314|ref|Os07g34490|
gi|7303320|ref|Os07g34530|
gi|7303321|ref|Os07g34540|
gi|7303323|ref|Os07g34560|
gi|7303331|ref|Os07g34630|
gi|7303333|ref|Os07g34650|
gi|7303334|ref|Os07g34660|
gi|7303336|ref|Os07g34680|
gi|7303337|ref|Os07g34690|
gi|7303348|ref|Os07g34790|
gi|7303358|ref|Os07g34890|
gi|7303364|ref|Os07g34960|
gi|7303369|ref|Os07g34990|
gi|7303372|ref|Os07g35020|
gi|7303373|ref|Os07g35030|
gi|7303378|ref|Os07g35080|
gi|7303379|ref|Os07g35090|
gi|7303382|ref|Os07g35120|
gi|7303385|ref|Os07g35150|
gi|7303386|ref|Os07g35160|
gi|7303388|ref|Os07g35180|
gi|7303389|ref|Os07g35190|
gi|7303390|ref|Os07g35200|
gi|7303392|ref|Os07g35220|
gi|7303395|ref|Os07g35250|
gi|7303397|ref|Os07g35270|
gi|7303407|ref|Os07g35360|
gi|7303411|ref|Os07g35400|
gi|7303413|ref|Os07g35420|
gi|7303416|ref|Os07g35450|
gi|7303420|ref|Os07g35490|
gi|7303424|ref|Os07g35530|
gi|7303426|ref|Os07g35550|
gi|7303432|ref|Os07g35600|
gi|7303434|ref|Os07g35620|
gi|7303439|ref|Os07g35670|
gi|7303446|ref|Os07g35720|
gi|7303447|ref|Os07g35720|
gi|7303448|ref|Os07g35720|
gi|7303453|ref|Os07g35760|
gi|7303457|ref|Os07g35800|
gi|7303459|ref|Os07g35820|
gi|7303460|ref|Os07g35830|
gi|7303462|ref|Os07g35850|
gi|7303467|ref|Os07g35890|

gi|7303473|ref|Os07g35950|
gi|7303476|ref|Os07g35980|
gi|7303480|ref|Os07g36020|
gi|7303483|ref|Os07g36050|
gi|7303485|ref|Os07g36070|
gi|7303489|ref|Os07g36100|
gi|7303491|ref|Os07g36120|
gi|7303495|ref|Os07g36160|
gi|7303500|ref|Os07g36210|
gi|7303501|ref|Os07g36220|
gi|7303504|ref|Os07g36240|
gi|7303511|ref|Os07g36310|
gi|7303512|ref|Os07g36320|
gi|7303513|ref|Os07g36330|
gi|7303515|ref|Os07g36350|
gi|7303518|ref|Os07g36380|
gi|7303523|ref|Os07g36430|
gi|7303531|ref|Os07g36510|
gi|7303533|ref|Os07g36530|
gi|7303538|ref|Os07g36580|
gi|7303543|ref|Os07g36620|
gi|7303545|ref|Os07g36640|
gi|7303546|ref|Os07g36650|
gi|7303554|ref|Os07g36730|
gi|7303560|ref|Os07g36790|
gi|7303563|ref|Os07g36810|
gi|7303568|ref|Os07g36860|
gi|7303570|ref|Os07g36880|
gi|7303576|ref|Os07g36930|
gi|7303578|ref|Os07g36950|
gi|7303579|ref|Os07g36960|
gi|7303580|ref|Os07g36970|
gi|7303583|ref|Os07g37000|
gi|7303585|ref|Os07g37020|
gi|7303588|ref|Os07g37050|
gi|7303589|ref|Os07g37060|
gi|7303598|ref|Os07g37150|
gi|7303610|ref|Os07g37260|
gi|7303612|ref|Os07g37280|
gi|7303613|ref|Os07g37290|
gi|7303614|ref|Os07g37300|
gi|7303617|ref|Os07g37330|
gi|7303618|ref|Os07g37340|
gi|7303620|ref|Os07g37360|
gi|7303621|ref|Os07g37370|
gi|7303622|ref|Os07g37380|
gi|7303623|ref|Os07g37390|
gi|7303627|ref|Os07g37430|
gi|7303628|ref|Os07g37440|
gi|7303632|ref|Os07g37480|
gi|7303633|ref|Os07g37490|
gi|7303639|ref|Os07g37540|
gi|7303645|ref|Os07g37590|
gi|7303646|ref|Os07g37600|
gi|7303652|ref|Os07g37660|
gi|7303654|ref|Os07g37680|
gi|7303669|ref|Os07g37820|
gi|7303673|ref|Os07g37850|
gi|7303675|ref|Os07g37870|
gi|7303676|ref|Os07g37880|
gi|7303679|ref|Os07g37910|
gi|7303681|ref|Os07g37930|
gi|7303690|ref|Os07g38020|
gi|7303692|ref|Os07g38040|
gi|7303693|ref|Os07g38050|
gi|7303704|ref|Os07g38140|
gi|7303705|ref|Os07g38150|
gi|7303709|ref|Os07g38190|
gi|7303710|ref|Os07g38200|
gi|7303711|ref|Os07g38210|
gi|7303722|ref|Os07g38310|
gi|7303730|ref|Os07g38370|
gi|7303734|ref|Os07g38410|
gi|7303741|ref|Os07g38480|
gi|7303748|ref|Os07g38550|
gi|7303750|ref|Os07g38570|
gi|7303756|ref|Os07g38630|
gi|7303760|ref|Os07g38670|
gi|7303765|ref|Os07g38720|
gi|7303767|ref|Os07g38740|
gi|7303781|ref|Os07g38870|
gi|7303782|ref|Os07g38880|
gi|7303784|ref|Os07g38900|
gi|7303793|ref|Os07g38980|
gi|7303795|ref|Os07g39000|
gi|7303799|ref|Os07g39030|
gi|7303800|ref|Os07g39040|
gi|7303801|ref|Os07g39050|
gi|7303802|ref|Os07g39060|
gi|7303804|ref|Os07g39080|
gi|7303806|ref|Os07g39100|
gi|7303807|ref|Os07g39110|
gi|7303808|ref|Os07g39120|
gi|7303809|ref|Os07g39130|
gi|7303810|ref|Os07g39140|
gi|7303812|ref|Os07g39160|
gi|7303814|ref|Os07g39180|
gi|7303815|ref|Os07g39190|
gi|7303816|ref|Os07g39200|
gi|7303817|ref|Os07g39210|
gi|7303822|ref|Os07g39260|
gi|7303831|ref|Os07g39330|
gi|7303835|ref|Os07g39370|
gi|7303837|ref|Os07g39390|
gi|7303843|ref|Os07g39450|
gi|7303847|ref|Os07g39490|
gi|7303848|ref|Os07g39500|
gi|7303854|ref|Os07g39540|
gi|7303855|ref|Os07g39550|
gi|7303858|ref|Os07g39570|
gi|7303859|ref|Os07g39580|
gi|7303865|ref|Os07g39640|
gi|7303866|ref|Os07g39650|
gi|7303867|ref|Os07g39660|
gi|7303869|ref|Os07g39680|
gi|7303873|ref|Os07g39720|
gi|7303877|ref|Os07g39760|
gi|7303878|ref|Os07g39770|
gi|7303881|ref|Os07g39790|
gi|7303888|ref|Os07g39860|
gi|7303891|ref|Os07g39890|
gi|7303903|ref|Os07g40010|
gi|7303906|ref|Os07g40040|
gi|7303909|ref|Os07g40070|
gi|7303910|ref|Os07g40080|
gi|7303911|ref|Os07g40090|
gi|7303913|ref|Os07g40110|
gi|7303915|ref|Os07g40130|
gi|7303916|ref|Os07g40140|
gi|7303919|ref|Os07g40170|
gi|7303920|ref|Os07g40180|
gi|7303922|ref|Os07g40200|
gi|7303924|ref|Os07g40220|
gi|7303925|ref|Os07g40230|

gi|7303931|ref|Os07g40280|
gi|7303937|ref|Os07g40340|
gi|7303940|ref|Os07g40370|
gi|7303941|ref|Os07g40380|
gi|7303942|ref|Os07g40390|
gi|7303945|ref|Os07g40420|
gi|7303946|ref|Os07g40430|
gi|7303947|ref|Os07g40440|
gi|7303948|ref|Os07g40450|
gi|7303952|ref|Os07g40490|
gi|7303955|ref|Os07g40500|
gi|7303962|ref|Os07g40560|
gi|7303966|ref|Os07g40600|
gi|7303970|ref|Os07g40640|
gi|7303973|ref|Os07g40670|
gi|7303978|ref|Os07g40720|
gi|7303990|ref|Os07g40830|
gi|7303991|ref|Os07g40840|
gi|7303992|ref|Os07g40850|
gi|7303993|ref|Os07g40860|
gi|7303994|ref|Os07g40870|
gi|7303996|ref|Os07g40890|
gi|7303997|ref|Os07g40900|
gi|7303998|ref|Os07g40910|
gi|7304007|ref|Os07g41000|
gi|7304009|ref|Os07g41020|
gi|7304011|ref|Os07g41040|
gi|7304018|ref|Os07g41100|
gi|7304025|ref|Os07g41170|
gi|7304029|ref|Os07g41210|
gi|7304037|ref|Os07g41290|
gi|7304047|ref|Os07g41390|
gi|7304048|ref|Os07g41400|
gi|7304049|ref|Os07g41410|
gi|7304050|ref|Os07g41420|
gi|7304055|ref|Os07g41470|
gi|7304057|ref|Os07g41490|
gi|7304063|ref|Os07g41550|
gi|7304070|ref|Os07g41620|
gi|7304088|ref|Os07g41760|
gi|7304095|ref|Os07g41830|
gi|7304096|ref|Os07g41840|
gi|7304107|ref|Os07g41950|
gi|7304108|ref|Os07g41960|
gi|7304109|ref|Os07g41970|
gi|7304111|ref|Os07g41990|
gi|7304112|ref|Os07g42000|
gi|7304121|ref|Os07g42090|
gi|7304122|ref|Os07g42100|
gi|7304125|ref|Os07g42130|
gi|7304131|ref|Os07g42190|
gi|7304139|ref|Os07g42270|
gi|7304141|ref|Os07g42290|
gi|7304144|ref|Os07g42320|
gi|7304146|ref|Os07g42340|
gi|7304148|ref|Os07g42360|
gi|7304151|ref|Os07g42390|
gi|7304160|ref|Os07g42470|
gi|7304161|ref|Os07g42480|
gi|7304168|ref|Os07g42550|
gi|7304171|ref|Os07g42580|
gi|7304175|ref|Os07g42620|
gi|7304179|ref|Os07g42650|
gi|7304180|ref|Os07g42650|
gi|7304190|ref|Os07g42740|
gi|7304194|ref|Os07g42780|
gi|7304195|ref|Os07g42790|
gi|7304200|ref|Os07g42840|
gi|7304201|ref|Os07g42850|
gi|7304209|ref|Os07g42930|
gi|7304218|ref|Os07g43010|
gi|7304231|ref|Os07g43130|
gi|7304233|ref|Os07g43150|
gi|7304237|ref|Os07g43190|
gi|7304239|ref|Os07g43210|
gi|7304246|ref|Os07g43280|
gi|7304248|ref|Os07g43300|
gi|7304252|ref|Os07g43340|
gi|7304259|ref|Os07g43410|
gi|7304261|ref|Os07g43430|
gi|7304262|ref|Os07g43440|
gi|7304263|ref|Os07g43450|
gi|7304267|ref|Os07g43490|
gi|7304268|ref|Os07g43500|
gi|7304277|ref|Os07g43590|
gi|7304279|ref|Os07g43610|
gi|7304280|ref|Os07g43620|
gi|7304284|ref|Os07g43660|
gi|7304287|ref|Os07g43690|
gi|7304297|ref|Os07g43770|
gi|7304298|ref|Os07g43770|
gi|7304299|ref|Os07g43770|
gi|7304308|ref|Os07g43860|
gi|7304309|ref|Os07g43880|
gi|7304313|ref|Os07g43910|
gi|7304315|ref|Os07g43930|
gi|7304319|ref|Os07g43960|
gi|7304323|ref|Os07g44000|
gi|7304324|ref|Os07g44010|
gi|7304328|ref|Os07g44050|
gi|7304332|ref|Os07g44080|
gi|7304334|ref|Os07g44100|
gi|7304336|ref|Os07g44120|
gi|7304339|ref|Os07g44150|
gi|7304340|ref|Os07g44160|
gi|7304346|ref|Os07g44220|
gi|7304368|ref|Os07g44420|
gi|7304373|ref|Os07g44470|
gi|7304396|ref|Os07g44680|
gi|7304403|ref|Os07g44750|
gi|7304404|ref|Os07g44760|
gi|7304409|ref|Os07g44810|
gi|7304415|ref|Os07g44870|
gi|7304416|ref|Os07g44880|
gi|7304428|ref|Os07g44980|
gi|7304429|ref|Os07g44990|
gi|7304434|ref|Os07g45040|
gi|7304435|ref|Os07g45050|
gi|7304443|ref|Os07g45130|
gi|7304444|ref|Os07g45140|
gi|7304445|ref|Os07g45150|
gi|7304449|ref|Os07g45190|
gi|7304450|ref|Os07g45200|
gi|7304453|ref|Os07g45230|
gi|7304454|ref|Os07g45240|
gi|7304463|ref|Os07g45330|
gi|7304464|ref|Os07g45340|
gi|7304471|ref|Os07g45380|
gi|7304475|ref|Os07g45420|
gi|7304477|ref|Os07g45440|
gi|7304478|ref|Os07g45450|
gi|7304480|ref|Os07g45470|
gi|7304483|ref|Os07g45500|
gi|7304485|ref|Os07g45520|

gi|7304497|ref|Os07g45640|
gi|7304500|ref|Os07g45670|
gi|7304502|ref|Os07g45690|
gi|7304505|ref|Os07g45720|
gi|7304506|ref|Os07g45730|
gi|7304507|ref|Os07g45740|
gi|7304510|ref|Os07g45770|
gi|7304512|ref|Os07g45790|
gi|7304513|ref|Os07g45800|
gi|7304514|ref|Os07g45810|
gi|7304517|ref|Os07g45840|
gi|7304519|ref|Os07g45860|
gi|7304524|ref|Os07g45910|
gi|7304529|ref|Os07g45960|
gi|7304531|ref|Os07g45980|
gi|7304540|ref|Os07g46070|
gi|7304543|ref|Os07g46100|
gi|7304548|ref|Os07g46150|
gi|7304553|ref|Os07g46200|
gi|7304563|ref|Os07g46290|
gi|7304581|ref|Os07g46470|
gi|7304588|ref|Os07g46530|
gi|7304603|ref|Os07g46670|
gi|7304604|ref|Os07g46680|
gi|7304621|ref|Os07g46800|
gi|7304637|ref|Os07g46960|
gi|7304649|ref|Os07g47060|
gi|7304650|ref|Os07g47070|
gi|7304668|ref|Os07g47220|
gi|7304681|ref|Os07g47320|
gi|7304688|ref|Os07g47380|
gi|7304696|ref|Os07g47460|
gi|7304702|ref|Os07g47520|
gi|7304706|ref|Os07g47560|
gi|7304710|ref|Os07g47600|
gi|7304718|ref|Os07g47640|
gi|7304720|ref|Os07g47660|
gi|7304722|ref|Os07g47680|
gi|7304723|ref|Os07g47690|
gi|7304731|ref|Os07g47770|
gi|7304736|ref|Os07g47810|
gi|7304741|ref|Os07g47850|
gi|7304749|ref|Os07g47930|
gi|7304750|ref|Os07g47940|
gi|7304753|ref|Os07g47970|
gi|7304769|ref|Os07g48110|
gi|7304770|ref|Os07g48120|
gi|7304777|ref|Os07g48190|
gi|7304784|ref|Os07g48250|
gi|7304799|ref|Os07g48380|
gi|7304803|ref|Os07g48420|
gi|7304804|ref|Os07g48420|
gi|7304805|ref|Os07g48420|
gi|7304812|ref|Os07g48480|
gi|7304817|ref|Os07g48520|
gi|7304818|ref|Os07g48520|
gi|7304819|ref|Os07g48540|
gi|7304824|ref|Os07g48590|
gi|7304827|ref|Os07g48620|
gi|7304841|ref|Os07g48740|
gi|7304856|ref|Os07g48860|
gi|7304862|ref|Os07g48910|
gi|7304863|ref|Os07g48910|
gi|7304864|ref|Os07g48910|
gi|7304866|ref|Os07g48930|
gi|7304880|ref|Os07g49050|
gi|7304881|ref|Os07g49060|
gi|7304888|ref|Os07g49130|
gi|7304893|ref|Os07g49180|
gi|7304912|ref|Os07g49350|
gi|7304932|ref|Os07g49540|
gi|7400001|ref|Os08g01010|
gi|7400002|ref|Os08g01020|
gi|7400020|ref|Os08g01200|
gi|7400023|ref|Os08g01230|
gi|7400049|ref|Os08g01440|
gi|7400051|ref|Os08g01460|
gi|7400055|ref|Os08g01500|
gi|7400059|ref|Os08g01540|
gi|7400060|ref|Os08g01550|
gi|7400061|ref|Os08g01560|
gi|7400072|ref|Os08g01660|
gi|7400075|ref|Os08g01690|
gi|7400078|ref|Os08g01720|
gi|7400079|ref|Os08g01730|
gi|7400086|ref|Os08g01790|
gi|7400089|ref|Os08g01820|
gi|7400093|ref|Os08g01860|
gi|7400098|ref|Os08g01910|
gi|7400106|ref|Os08g01990|
gi|7400108|ref|Os08g02010|
gi|7400124|ref|Os08g02180|
gi|7400125|ref|Os08g02170|
gi|7400127|ref|Os08g02200|
gi|7400134|ref|Os08g02260|
gi|7400136|ref|Os08g02280|
gi|7400137|ref|Os08g02290|
gi|7400139|ref|Os08g02310|
gi|7400140|ref|Os08g02320|
gi|7400152|ref|Os08g02430|
gi|7400168|ref|Os08g02590|
gi|7400170|ref|Os08g02610|
gi|7400171|ref|Os08g02620|
gi|7400174|ref|Os08g02650|
gi|7400175|ref|Os08g02660|
gi|7400177|ref|Os08g02680|
gi|7400181|ref|Os08g02720|
gi|7400183|ref|Os08g02740|
gi|7400189|ref|Os08g02800|
gi|7400190|ref|Os08g02810|
gi|7400193|ref|Os08g02840|
gi|7400196|ref|Os08g02870|
gi|7400197|ref|Os08g02880|
gi|7400198|ref|Os08g02890|
gi|7400200|ref|Os08g02910|
gi|7400201|ref|Os08g02920|
gi|7400202|ref|Os08g02930|
gi|7400203|ref|Os08g02940|
gi|7400204|ref|Os08g02950|
gi|7400205|ref|Os08g02960|
gi|7400206|ref|Os08g02970|
gi|7400207|ref|Os08g02980|
gi|7400214|ref|Os08g03050|
gi|7400232|ref|Os08g03230|
gi|7400234|ref|Os08g03250|
gi|7400237|ref|Os08g03280|
gi|7400239|ref|Os08g03300|
gi|7400244|ref|Os08g03320|
gi|7400252|ref|Os08g03400|
gi|7400259|ref|Os08g03460|
gi|7400284|ref|Os08g03710|
gi|7400290|ref|Os08g03770|
gi|7400292|ref|Os08g03790|
gi|7400293|ref|Os08g03800|

gi|7400296|ref|Os08g03830|
gi|7400297|ref|Os08g03840|
gi|7400298|ref|Os08g03850|
gi|7400299|ref|Os08g03860|
gi|7400302|ref|Os08g03890|
gi|7400305|ref|Os08g03920|
gi|7400307|ref|Os08g03940|
gi|7400312|ref|Os08g03990|
gi|7400313|ref|Os08g04000|
gi|7400317|ref|Os08g04040|
gi|7400322|ref|Os08g04090|
gi|7400323|ref|Os08g04100|
gi|7400329|ref|Os08g04160|
gi|7400334|ref|Os08g04200|
gi|7400337|ref|Os08g04220|
gi|7400341|ref|Os08g04260|
gi|7400360|ref|Os08g04430|
gi|7400371|ref|Os08g04520|
gi|7400374|ref|Os08g04550|
gi|7400376|ref|Os08g04570|
gi|7400377|ref|Os08g04580|
gi|7400378|ref|Os08g04590|
gi|7400379|ref|Os08g04600|
gi|7400380|ref|Os08g04610|
gi|7400383|ref|Os08g04640|
gi|7400384|ref|Os08g04650|
gi|7400385|ref|Os08g04660|
gi|7400386|ref|Os08g04670|
gi|7400387|ref|Os08g04680|
gi|7400388|ref|Os08g04690|
gi|7400389|ref|Os08g04700|
gi|7400390|ref|Os08g04710|
gi|7400393|ref|Os08g04740|
gi|7400395|ref|Os08g04760|
gi|7400402|ref|Os08g04830|
gi|7400409|ref|Os08g04900|
gi|7400410|ref|Os08g04910|
gi|7400413|ref|Os08g04940|
gi|7400415|ref|Os08g04960|
gi|7400420|ref|Os08g05010|
gi|7400421|ref|Os08g05020|
gi|7400423|ref|Os08g05040|
gi|7400424|ref|Os08g05050|
gi|7400426|ref|Os08g05070|
gi|7400428|ref|Os08g05090|
gi|7400429|ref|Os08g05100|
gi|7400430|ref|Os08g05110|
gi|7400433|ref|Os08g05140|
gi|7400435|ref|Os08g05160|
gi|7400436|ref|Os08g05170|
gi|7400438|ref|Os08g05190|
gi|7400449|ref|Os08g05300|
gi|7400450|ref|Os08g05310|
gi|7400451|ref|Os08g05320|
gi|7400452|ref|Os08g05330|
gi|7400457|ref|Os08g05380|
gi|7400466|ref|Os08g05470|
gi|7400474|ref|Os08g05540|
gi|7400475|ref|Os08g05550|
gi|7400490|ref|Os08g05680|
gi|7400492|ref|Os08g05700|
gi|7400495|ref|Os08g05730|
gi|7400496|ref|Os08g05740|
gi|7400498|ref|Os08g05760|
gi|7400509|ref|Os08g05860|
gi|7400513|ref|Os08g05900|
gi|7400518|ref|Os08g05950|
gi|7400519|ref|Os08g05960|
gi|7400523|ref|Os08g06000|
gi|7400526|ref|Os08g06030|
gi|7400531|ref|Os08g06080|
gi|7400539|ref|Os08g06130|
gi|7400549|ref|Os08g06210|
gi|7400550|ref|Os08g06220|
gi|7400554|ref|Os08g06260|
gi|7400555|ref|Os08g06270|
gi|7400559|ref|Os08g06290|
gi|7400560|ref|Os08g06300|
gi|7400564|ref|Os08g06340|
gi|7400570|ref|Os08g06390|
gi|7400571|ref|Os08g06400|
gi|7400572|ref|Os08g06410|
gi|7400586|ref|Os08g06540|
gi|7400591|ref|Os08g06590|
gi|7400594|ref|Os08g06620|
gi|7400602|ref|Os08g06680|
gi|7400609|ref|Os08g06750|
gi|7400610|ref|Os08g06760|
gi|7400611|ref|Os08g06770|
gi|7400615|ref|Os08g06810|
gi|7400617|ref|Os08g06830|
gi|7400618|ref|Os08g06840|
gi|7400620|ref|Os08g06860|
gi|7400621|ref|Os08g06870|
gi|7400625|ref|Os08g06910|
gi|7400626|ref|Os08g06920|
gi|7400628|ref|Os08g06940|
gi|7400636|ref|Os08g07020|
gi|7400637|ref|Os08g07030|
gi|7400641|ref|Os08g07070|
gi|7400643|ref|Os08g07090|
gi|7400649|ref|Os08g07150|
gi|7400650|ref|Os08g07160|
gi|7400655|ref|Os08g07210|
gi|7400658|ref|Os08g07240|
gi|7400659|ref|Os08g07250|
gi|7400662|ref|Os08g07280|
gi|7400667|ref|Os08g07320|
gi|7400673|ref|Os08g07370|
gi|7400683|ref|Os08g07470|
gi|7400685|ref|Os08g07490|
gi|7400687|ref|Os08g07510|
gi|7400688|ref|Os08g07520|
gi|7400689|ref|Os08g07530|
gi|7400695|ref|Os08g07580|
gi|7400696|ref|Os08g07590|
gi|7400697|ref|Os08g07600|
gi|7400699|ref|Os08g07620|
gi|7400700|ref|Os08g07630|
gi|7400701|ref|Os08g07640|
gi|7400702|ref|Os08g07650|
gi|7400703|ref|Os08g07660|
gi|7400704|ref|Os08g07670|
gi|7400705|ref|Os08g07680|
gi|7400706|ref|Os08g07690|
gi|7400708|ref|Os08g07710|
gi|7400709|ref|Os08g07720|
gi|7400717|ref|Os08g07800|
gi|7400719|ref|Os08g07820|
gi|7400723|ref|Os08g07860|
gi|7400728|ref|Os08g07910|
gi|7400736|ref|Os08g07990|
gi|7400738|ref|Os08g08010|
gi|7400739|ref|Os08g08020|

gi|7400747|ref|Os08g08090|
gi|7400748|ref|Os08g08100|
gi|7400753|ref|Os08g08150|
gi|7400762|ref|Os08g08230|
gi|7400764|ref|Os08g08250|
gi|7400765|ref|Os08g08260|
gi|7400766|ref|Os08g08270|
gi|7400768|ref|Os08g08290|
gi|7400771|ref|Os08g08320|
gi|7400773|ref|Os08g08340|
gi|7400775|ref|Os08g08360|
gi|7400777|ref|Os08g08380|
gi|7400779|ref|Os08g08400|
gi|7400780|ref|Os08g08410|
gi|7400781|ref|Os08g08420|
gi|7400782|ref|Os08g08430|
gi|7400783|ref|Os08g08440|
gi|7400786|ref|Os08g08470|
gi|7400788|ref|Os08g08490|
gi|7400790|ref|Os08g08510|
gi|7400791|ref|Os08g08520|
gi|7400795|ref|Os08g08560|
gi|7400797|ref|Os08g08580|
gi|7400798|ref|Os08g08590|
gi|7400799|ref|Os08g08600|
gi|7400802|ref|Os08g08630|
gi|7400804|ref|Os08g08650|
gi|7400805|ref|Os08g08650|
gi|7400806|ref|Os08g08660|
gi|7400809|ref|Os08g08690|
gi|7400811|ref|Os08g08710|
gi|7400813|ref|Os08g08730|
gi|7400814|ref|Os08g08740|
gi|7400815|ref|Os08g08750|
gi|7400820|ref|Os08g08800|
gi|7400821|ref|Os08g08810|
gi|7400828|ref|Os08g08880|
gi|7400829|ref|Os08g08890|
gi|7400830|ref|Os08g08900|
gi|7400831|ref|Os08g08910|
gi|7400833|ref|Os08g08930|
gi|7400843|ref|Os08g09030|
gi|7400845|ref|Os08g09050|
gi|7400849|ref|Os08g09090|
gi|7400852|ref|Os08g09120|
gi|7400853|ref|Os08g09130|
gi|7400871|ref|Os08g09280|
gi|7400872|ref|Os08g09290|
gi|7400876|ref|Os08g09310|
gi|7400882|ref|Os08g09360|
gi|7400886|ref|Os08g09400|
gi|7400900|ref|Os08g09540|
gi|7400901|ref|Os08g09550|
gi|7400902|ref|Os08g09560|
gi|7400904|ref|Os08g09580|
gi|7400907|ref|Os08g09610|
gi|7400908|ref|Os08g09620|
gi|7400909|ref|Os08g09630|
gi|7400914|ref|Os08g09680|
gi|7400924|ref|Os08g09780|
gi|7400925|ref|Os08g09790|
gi|7400931|ref|Os08g09850|
gi|7400933|ref|Os08g09870|
gi|7400940|ref|Os08g09930|
gi|7400943|ref|Os08g09960|
gi|7400944|ref|Os08g09970|
gi|7400945|ref|Os08g09980|
gi|7400946|ref|Os08g09990|
gi|7400947|ref|Os08g10000|
gi|7400956|ref|Os08g10090|
gi|7400957|ref|Os08g10100|
gi|7400958|ref|Os08g10110|
gi|7400968|ref|Os08g10210|
gi|7400971|ref|Os08g10240|
gi|7400974|ref|Os08g10270|
gi|7400975|ref|Os08g10280|
gi|7400984|ref|Os08g10340|
gi|7400988|ref|Os08g10380|
gi|7400989|ref|Os08g10390|
gi|7400999|ref|Os08g10490|
gi|7401000|ref|Os08g10500|
gi|7401003|ref|Os08g10530|
gi|7401019|ref|Os08g10680|
gi|7401020|ref|Os08g10690|
gi|7401023|ref|Os08g10720|
gi|7401025|ref|Os08g10750|
gi|7401028|ref|Os08g10760|
gi|7401031|ref|Os08g10790|
gi|7401032|ref|Os08g10800|
gi|7401033|ref|Os08g10810|
gi|7401034|ref|Os08g10820|
gi|7401036|ref|Os08g10840|
gi|7401038|ref|Os08g10860|
gi|7401039|ref|Os08g10870|
gi|7401040|ref|Os08g10880|
gi|7401043|ref|Os08g10910|
gi|7401045|ref|Os08g10930|
gi|7401047|ref|Os08g10950|
gi|7401058|ref|Os08g11060|
gi|7401061|ref|Os08g11090|
gi|7401062|ref|Os08g11100|
gi|7401065|ref|Os08g11130|
gi|7401068|ref|Os08g11160|
gi|7401069|ref|Os08g11170|
gi|7401071|ref|Os08g11190|
gi|7401073|ref|Os08g11210|
gi|7401074|ref|Os08g11220|
gi|7401075|ref|Os08g11230|
gi|7401076|ref|Os08g11240|
gi|7401077|ref|Os08g11250|
gi|7401079|ref|Os08g11270|
gi|7401081|ref|Os08g11290|
gi|7401084|ref|Os08g11320|
gi|7401086|ref|Os08g11340|
gi|7401087|ref|Os08g11350|
gi|7401089|ref|Os08g11370|
gi|7401091|ref|Os08g11390|
gi|7401092|ref|Os08g11400|
gi|7401094|ref|Os08g11420|
gi|7401097|ref|Os08g11450|
gi|7401101|ref|Os08g11490|
gi|7401103|ref|Os08g11510|
gi|7401104|ref|Os08g11520|
gi|7401106|ref|Os08g11540|
gi|7401107|ref|Os08g11550|
gi|7401108|ref|Os08g11560|
gi|7401110|ref|Os08g11580|
gi|7401112|ref|Os08g11600|
gi|7401113|ref|Os08g11610|
gi|7401115|ref|Os08g11630|
gi|7401116|ref|Os08g11640|
gi|7401117|ref|Os08g11650|
gi|7401118|ref|Os08g11660|
gi|7401119|ref|Os08g11670|

gi|7401122|ref|Os08g11700|
gi|7401123|ref|Os08g11710|
gi|7401124|ref|Os08g11720|
gi|7401125|ref|Os08g11730|
gi|7401127|ref|Os08g11750|
gi|7401128|ref|Os08g11760|
gi|7401129|ref|Os08g11770|
gi|7401134|ref|Os08g11820|
gi|7401136|ref|Os08g11840|
gi|7401140|ref|Os08g11880|
gi|7401141|ref|Os08g11890|
gi|7401146|ref|Os08g11940|
gi|7401147|ref|Os08g11950|
gi|7401151|ref|Os08g11990|
gi|7401155|ref|Os08g12030|
gi|7401158|ref|Os08g12060|
gi|7401159|ref|Os08g12070|
gi|7401161|ref|Os08g12090|
gi|7401162|ref|Os08g12100|
gi|7401167|ref|Os08g12150|
gi|7401168|ref|Os08g12160|
gi|7401169|ref|Os08g12170|
gi|7401170|ref|Os08g12180|
gi|7401174|ref|Os08g12220|
gi|7401175|ref|Os08g12230|
gi|7401176|ref|Os08g12240|
gi|7401177|ref|Os08g12250|
gi|7401183|ref|Os08g12310|
gi|7401184|ref|Os08g12320|
gi|7401185|ref|Os08g12330|
gi|7401186|ref|Os08g12340|
gi|7401194|ref|Os08g12420|
gi|7401198|ref|Os08g12460|
gi|7401201|ref|Os08g12490|
gi|7401203|ref|Os08g12510|
gi|7401204|ref|Os08g12520|
gi|7401210|ref|Os08g12580|
gi|7401211|ref|Os08g12590|
gi|7401212|ref|Os08g12600|
gi|7401214|ref|Os08g12620|
gi|7401215|ref|Os08g12630|
gi|7401218|ref|Os08g12660|
gi|7401219|ref|Os08g12670|
gi|7401223|ref|Os08g12710|
gi|7401229|ref|Os08g12770|
gi|7401233|ref|Os08g12810|
gi|7401246|ref|Os08g12930|
gi|7401251|ref|Os08g12980|
gi|7401254|ref|Os08g13010|
gi|7401257|ref|Os08g13040|
gi|7401258|ref|Os08g13050|
gi|7401263|ref|Os08g13100|
gi|7401264|ref|Os08g13110|
gi|7401269|ref|Os08g13160|
gi|7401273|ref|Os08g13200|
gi|7401274|ref|Os08g13210|
gi|7401276|ref|Os08g13230|
gi|7401281|ref|Os08g13280|
gi|7401284|ref|Os08g13310|
gi|7401286|ref|Os08g13330|
gi|7401292|ref|Os08g13380|
gi|7401298|ref|Os08g13430|
gi|7401302|ref|Os08g13450|
gi|7401303|ref|Os08g13460|
gi|7401304|ref|Os08g13470|
gi|7401305|ref|Os08g13480|
gi|7401306|ref|Os08g13490|
gi|7401310|ref|Os08g13530|
gi|7401320|ref|Os08g13630|
gi|7401321|ref|Os08g13630|
gi|7401323|ref|Os08g13650|
gi|7401324|ref|Os08g13660|
gi|7401325|ref|Os08g13670|
gi|7401328|ref|Os08g13700|
gi|7401329|ref|Os08g13710|
gi|7401330|ref|Os08g13720|
gi|7401331|ref|Os08g13730|
gi|7401332|ref|Os08g13740|
gi|7401335|ref|Os08g13770|
gi|7401337|ref|Os08g13790|
gi|7401340|ref|Os08g13820|
gi|7401341|ref|Os08g13830|
gi|7401346|ref|Os08g13880|
gi|7401349|ref|Os08g13910|
gi|7401354|ref|Os08g13940|
gi|7401361|ref|Os08g14010|
gi|7401363|ref|Os08g14030|
gi|7401364|ref|Os08g14040|
gi|7401365|ref|Os08g14050|
gi|7401368|ref|Os08g14080|
gi|7401370|ref|Os08g14100|
gi|7401371|ref|Os08g14110|
gi|7401373|ref|Os08g14130|
gi|7401374|ref|Os08g14140|
gi|7401375|ref|Os08g14150|
gi|7401376|ref|Os08g14160|
gi|7401384|ref|Os08g14240|
gi|7401386|ref|Os08g14260|
gi|7401390|ref|Os08g14300|
gi|7401391|ref|Os08g14310|
gi|7401396|ref|Os08g14350|
gi|7401399|ref|Os08g14380|
gi|7401400|ref|Os08g14390|
gi|7401402|ref|Os08g14410|
gi|7401403|ref|Os08g14420|
gi|7401404|ref|Os08g14430|
gi|7401413|ref|Os08g14500|
gi|7401422|ref|Os08g14590|
gi|7401423|ref|Os08g14600|
gi|7401426|ref|Os08g14630|
gi|7401428|ref|Os08g14650|
gi|7401434|ref|Os08g14710|
gi|7401435|ref|Os08g14720|
gi|7401441|ref|Os08g14780|
gi|7401442|ref|Os08g14790|
gi|7401443|ref|Os08g14800|
gi|7401445|ref|Os08g14820|
gi|7401447|ref|Os08g14840|
gi|7401450|ref|Os08g14870|
gi|7401452|ref|Os08g14890|
gi|7401453|ref|Os08g14900|
gi|7401454|ref|Os08g14910|
gi|7401455|ref|Os08g14920|
gi|7401456|ref|Os08g14930|
gi|7401461|ref|Os08g14980|
gi|7401470|ref|Os08g15070|
gi|7401473|ref|Os08g15100|
gi|7401476|ref|Os08g15130|
gi|7401478|ref|Os08g15150|
gi|7401479|ref|Os08g15160|
gi|7401482|ref|Os08g15190|
gi|7401497|ref|Os08g15340|
gi|7401499|ref|Os08g15360|
gi|7401501|ref|Os08g15380|

gi|7401502|ref|Os08g15390|
gi|7401504|ref|Os08g15410|
gi|7401507|ref|Os08g15440|
gi|7401511|ref|Os08g15480|
gi|7401512|ref|Os08g15490|
gi|7401516|ref|Os08g15530|
gi|7401517|ref|Os08g15540|
gi|7401518|ref|Os08g15550|
gi|7401519|ref|Os08g15560|
gi|7401523|ref|Os08g15590|
gi|7401525|ref|Os08g15610|
gi|7401526|ref|Os08g15620|
gi|7401527|ref|Os08g15630|
gi|7401529|ref|Os08g15650|
gi|7401531|ref|Os08g15670|
gi|7401532|ref|Os08g15680|
gi|7401538|ref|Os08g15730|
gi|7401542|ref|Os08g15770|
gi|7401550|ref|Os08g15850|
gi|7401551|ref|Os08g15860|
gi|7401554|ref|Os08g15890|
gi|7401557|ref|Os08g15920|
gi|7401558|ref|Os08g15930|
gi|7401561|ref|Os08g15960|
gi|7401562|ref|Os08g15970|
gi|7401563|ref|Os08g15980|
gi|7401567|ref|Os08g16020|
gi|7401568|ref|Os08g16030|
gi|7401569|ref|Os08g16040|
gi|7401572|ref|Os08g16060|
gi|7401577|ref|Os08g16110|
gi|7401581|ref|Os08g16150|
gi|7401583|ref|Os08g16170|
gi|7401596|ref|Os08g16300|
gi|7401597|ref|Os08g16310|
gi|7401599|ref|Os08g16330|
gi|7401602|ref|Os08g16360|
gi|7401606|ref|Os08g16400|
gi|7401607|ref|Os08g16410|
gi|7401611|ref|Os08g16450|
gi|7401613|ref|Os08g16470|
gi|7401616|ref|Os08g16490|
gi|7401617|ref|Os08g16500|
gi|7401619|ref|Os08g16520|
gi|7401622|ref|Os08g16550|
gi|7401627|ref|Os08g16590|
gi|7401631|ref|Os08g16620|
gi|7401633|ref|Os08g16640|
gi|7401634|ref|Os08g16650|
gi|7401638|ref|Os08g16690|
gi|7401641|ref|Os08g16720|
gi|7401643|ref|Os08g16740|
gi|7401644|ref|Os08g16750|
gi|7401650|ref|Os08g16800|
gi|7401651|ref|Os08g16810|
gi|7401652|ref|Os08g16820|
gi|7401653|ref|Os08g16830|
gi|7401655|ref|Os08g16850|
gi|7401657|ref|Os08g16870|
gi|7401662|ref|Os08g16920|
gi|7401663|ref|Os08g16930|
gi|7401664|ref|Os08g16940|
gi|7401665|ref|Os08g16950|
gi|7401672|ref|Os08g17020|
gi|7401676|ref|Os08g17060|
gi|7401680|ref|Os08g17100|
gi|7401681|ref|Os08g17110|
gi|7401682|ref|Os08g17120|
gi|7401689|ref|Os08g17190|
gi|7401690|ref|Os08g17200|
gi|7401692|ref|Os08g17220|
gi|7401695|ref|Os08g17250|
gi|7401696|ref|Os08g17260|
gi|7401697|ref|Os08g17270|
gi|7401703|ref|Os08g17330|
gi|7401704|ref|Os08g17340|
gi|7401705|ref|Os08g17350|
gi|7401706|ref|Os08g17360|
gi|7401708|ref|Os08g17380|
gi|7401713|ref|Os08g17430|
gi|7401714|ref|Os08g17440|
gi|7401724|ref|Os08g17540|
gi|7401726|ref|Os08g17560|
gi|7401727|ref|Os08g17570|
gi|7401728|ref|Os08g17580|
gi|7401732|ref|Os08g17620|
gi|7401733|ref|Os08g17630|
gi|7401736|ref|Os08g17660|
gi|7401739|ref|Os08g17690|
gi|7401741|ref|Os08g17710|
gi|7401746|ref|Os08g17760|
gi|7401747|ref|Os08g17770|
gi|7401749|ref|Os08g17790|
gi|7401750|ref|Os08g17800|
gi|7401758|ref|Os08g17880|
gi|7401760|ref|Os08g17900|
gi|7401762|ref|Os08g17920|
gi|7401765|ref|Os08g17950|
gi|7401766|ref|Os08g17960|
gi|7401767|ref|Os08g17970|
gi|7401773|ref|Os08g18030|
gi|7401776|ref|Os08g18060|
gi|7401777|ref|Os08g18070|
gi|7401787|ref|Os08g18170|
gi|7401790|ref|Os08g18200|
gi|7401793|ref|Os08g18720|
gi|7401795|ref|Os08g18740|
gi|7401796|ref|Os08g18750|
gi|7401800|ref|Os08g18790|
gi|7401801|ref|Os08g18800|
gi|7401802|ref|Os08g18810|
gi|7401803|ref|Os08g18820|
gi|7401804|ref|Os08g18830|
gi|7401806|ref|Os08g18850|
gi|7401807|ref|Os08g18860|
gi|7401810|ref|Os08g18890|
gi|7401811|ref|Os08g18900|
gi|7401812|ref|Os08g18910|
gi|7401814|ref|Os08g18930|
gi|7401819|ref|Os08g18980|
gi|7401822|ref|Os08g19010|
gi|7401823|ref|Os08g19020|
gi|7401824|ref|Os08g19030|
gi|7401825|ref|Os08g19040|
gi|7401826|ref|Os08g19050|
gi|7401827|ref|Os08g19060|
gi|7401828|ref|Os08g19070|
gi|7401831|ref|Os08g19100|
gi|7401832|ref|Os08g19110|
gi|7401834|ref|Os08g19130|
gi|7401836|ref|Os08g19150|
gi|7401841|ref|Os08g19200|
gi|7401842|ref|Os08g19210|
gi|7401843|ref|Os08g19210|

gi|7401845|ref|Os08g19230|
gi|7401846|ref|Os08g19240|
gi|7401849|ref|Os08g19270|
gi|7401851|ref|Os08g19290|
gi|7401852|ref|Os08g19300|
gi|7401855|ref|Os08g19330|
gi|7401856|ref|Os08g19340|
gi|7401860|ref|Os08g19380|
gi|7401865|ref|Os08g19430|
gi|7401869|ref|Os08g19470|
gi|7401870|ref|Os08g19480|
gi|7401877|ref|Os08g19550|
gi|7401878|ref|Os08g19560|
gi|7401883|ref|Os08g19600|
gi|7401887|ref|Os08g19620|
gi|7401888|ref|Os08g19630|
gi|7401889|ref|Os08g19640|
gi|7401891|ref|Os08g19660|
gi|7401892|ref|Os08g19670|
gi|7401895|ref|Os08g19700|
gi|7401898|ref|Os08g19730|
gi|7401903|ref|Os08g19780|
gi|7401904|ref|Os08g19790|
gi|7401905|ref|Os08g19800|
gi|7401906|ref|Os08g19810|
gi|7401907|ref|Os08g19820|
gi|7401909|ref|Os08g19840|
gi|7401915|ref|Os08g19900|
gi|7401916|ref|Os08g19910|
gi|7401920|ref|Os08g19950|
gi|7401921|ref|Os08g19960|
gi|7401926|ref|Os08g20010|
gi|7401933|ref|Os08g20070|
gi|7401934|ref|Os08g20080|
gi|7401935|ref|Os08g20090|
gi|7401936|ref|Os08g20100|
gi|7401938|ref|Os08g20120|
gi|7401944|ref|Os08g20180|
gi|7401949|ref|Os08g20230|
gi|7401950|ref|Os08g20240|
gi|7401960|ref|Os08g20340|
gi|7401964|ref|Os08g20380|
gi|7401965|ref|Os08g20390|
gi|7401967|ref|Os08g20410|
gi|7401969|ref|Os08g20430|
gi|7401971|ref|Os08g20450|
gi|7401979|ref|Os08g20530|
gi|7401980|ref|Os08g20540|
gi|7401982|ref|Os08g20560|
gi|7401986|ref|Os08g20600|
gi|7401988|ref|Os08g20620|
gi|7401989|ref|Os08g20630|
gi|7401990|ref|Os08g20640|
gi|7401991|ref|Os08g20650|
gi|7401993|ref|Os08g20670|
gi|7401994|ref|Os08g20680|
gi|7401995|ref|Os08g20690|
gi|7401997|ref|Os08g20710|
gi|7402000|ref|Os08g20740|
gi|7402001|ref|Os08g20750|
gi|7402002|ref|Os08g20760|
gi|7402004|ref|Os08g20780|
gi|7402007|ref|Os08g20810|
gi|7402009|ref|Os08g20830|
gi|7402011|ref|Os08g20850|
gi|7402013|ref|Os08g20870|
gi|7402014|ref|Os08g20880|
gi|7402016|ref|Os08g20910|
gi|7402017|ref|Os08g20900|
gi|7402018|ref|Os08g20920|
gi|7402019|ref|Os08g20930|
gi|7402020|ref|Os08g20940|
gi|7402021|ref|Os08g20950|
gi|7402023|ref|Os08g20970|
gi|7402024|ref|Os08g20980|
gi|7402025|ref|Os08g20990|
gi|7402026|ref|Os08g21000|
gi|7402027|ref|Os08g21010|
gi|7402030|ref|Os08g21040|
gi|7402031|ref|Os08g21050|
gi|7402034|ref|Os08g21080|
gi|7402035|ref|Os08g21090|
gi|7402041|ref|Os08g21150|
gi|7402044|ref|Os08g21180|
gi|7402050|ref|Os08g21240|
gi|7402052|ref|Os08g21260|
gi|7402053|ref|Os08g21270|
gi|7402056|ref|Os08g21300|
gi|7402057|ref|Os08g21310|
gi|7402058|ref|Os08g21320|
gi|7402060|ref|Os08g21340|
gi|7402067|ref|Os08g21400|
gi|7402072|ref|Os08g21450|
gi|7402074|ref|Os08g21470|
gi|7402077|ref|Os08g21500|
gi|7402080|ref|Os08g21530|
gi|7402081|ref|Os08g21540|
gi|7402082|ref|Os08g21550|
gi|7402083|ref|Os08g21560|
gi|7402087|ref|Os08g21600|
gi|7402088|ref|Os08g21610|
gi|7402089|ref|Os08g21620|
gi|7402091|ref|Os08g21640|
gi|7402100|ref|Os08g21730|
gi|7402105|ref|Os08g21780|
gi|7402110|ref|Os08g21830|
gi|7402112|ref|Os08g21850|
gi|7402114|ref|Os08g21870|
gi|7402118|ref|Os08g21910|
gi|7402122|ref|Os08g21950|
gi|7402125|ref|Os08g21980|
gi|7402128|ref|Os08g22010|
gi|7402131|ref|Os08g22040|
gi|7402133|ref|Os08g22060|
gi|7402136|ref|Os08g22090|
gi|7402139|ref|Os08g22120|
gi|7402142|ref|Os08g22150|
gi|7402144|ref|Os08g22170|
gi|7402146|ref|Os08g22190|
gi|7402149|ref|Os08g22220|
gi|7402151|ref|Os08g22240|
gi|7402152|ref|Os08g22250|
gi|7402156|ref|Os08g22290|
gi|7402157|ref|Os08g22300|
gi|7402167|ref|Os08g22400|
gi|7402171|ref|Os08g22440|
gi|7402173|ref|Os08g22460|
gi|7402174|ref|Os08g22470|
gi|7402176|ref|Os08g22490|
gi|7402179|ref|Os08g22520|
gi|7402180|ref|Os08g22530|
gi|7402184|ref|Os08g22570|
gi|7402185|ref|Os08g22580|
gi|7402188|ref|Os08g22610|

gi|7402194|ref|Os08g22670|
gi|7402197|ref|Os08g22700|
gi|7402199|ref|Os08g22720|
gi|7402200|ref|Os08g22730|
gi|7402207|ref|Os08g22800|
gi|7402211|ref|Os08g22840|
gi|7402217|ref|Os08g22900|
gi|7402220|ref|Os08g22930|
gi|7402222|ref|Os08g22950|
gi|7402225|ref|Os08g22980|
gi|7402228|ref|Os08g23010|
gi|7402230|ref|Os08g23030|
gi|7402245|ref|Os08g23170|
gi|7402247|ref|Os08g23190|
gi|7402250|ref|Os08g23220|
gi|7402251|ref|Os08g23230|
gi|7402255|ref|Os08g23270|
gi|7402263|ref|Os08g23350|
gi|7402265|ref|Os08g23370|
gi|7402270|ref|Os08g23420|
gi|7402275|ref|Os08g23450|
gi|7402280|ref|Os08g23500|
gi|7402282|ref|Os08g23520|
gi|7402285|ref|Os08g23550|
gi|7402286|ref|Os08g23560|
gi|7402290|ref|Os08g23600|
gi|7402297|ref|Os08g23670|
gi|7402300|ref|Os08g23690|
gi|7402306|ref|Os08g23750|
gi|7402311|ref|Os08g23800|
gi|7402313|ref|Os08g23820|
gi|7402314|ref|Os08g23830|
gi|7402316|ref|Os08g23850|
gi|7402318|ref|Os08g23870|
gi|7402319|ref|Os08g23880|
gi|7402321|ref|Os08g23900|
gi|7402326|ref|Os08g23940|
gi|7402327|ref|Os08g23950|
gi|7402329|ref|Os08g23970|
gi|7402330|ref|Os08g23980|
gi|7402332|ref|Os08g24000|
gi|7402333|ref|Os08g24010|
gi|7402339|ref|Os08g24070|
gi|7402341|ref|Os08g24090|
gi|7402343|ref|Os08g24110|
gi|7402347|ref|Os08g24150|
gi|7402349|ref|Os08g24170|
gi|7402352|ref|Os08g24200|
gi|7402353|ref|Os08g24210|
gi|7402357|ref|Os08g24250|
gi|7402361|ref|Os08g24290|
gi|7402362|ref|Os08g24300|
gi|7402365|ref|Os08g24330|
gi|7402366|ref|Os08g24340|
gi|7402367|ref|Os08g24350|
gi|7402368|ref|Os08g24360|
gi|7402369|ref|Os08g24370|
gi|7402376|ref|Os08g24440|
gi|7402378|ref|Os08g24460|
gi|7402379|ref|Os08g24470|
gi|7402383|ref|Os08g24510|
gi|7402392|ref|Os08g24600|
gi|7402393|ref|Os08g24610|
gi|7402394|ref|Os08g24620|
gi|7402398|ref|Os08g24660|
gi|7402402|ref|Os08g24700|
gi|7402406|ref|Os08g24740|
gi|7402412|ref|Os08g24800|
gi|7402413|ref|Os08g24810|
gi|7402414|ref|Os08g24820|
gi|7402416|ref|Os08g24840|
gi|7402417|ref|Os08g24850|
gi|7402421|ref|Os08g24890|
gi|7402422|ref|Os08g24900|
gi|7402423|ref|Os08g24910|
gi|7402424|ref|Os08g24920|
gi|7402426|ref|Os08g24940|
gi|7402428|ref|Os08g24960|
gi|7402432|ref|Os08g25000|
gi|7402434|ref|Os08g25020|
gi|7402436|ref|Os08g25040|
gi|7402439|ref|Os08g25070|
gi|7402443|ref|Os08g25100|
gi|7402449|ref|Os08g25160|
gi|7402451|ref|Os08g25180|
gi|7402456|ref|Os08g25230|
gi|7402460|ref|Os08g25270|
gi|7402463|ref|Os08g25300|
gi|7402465|ref|Os08g25320|
gi|7402469|ref|Os08g25360|
gi|7402470|ref|Os08g25370|
gi|7402474|ref|Os08g25400|
gi|7402476|ref|Os08g25420|
gi|7402478|ref|Os08g25440|
gi|7402479|ref|Os08g25450|
gi|7402481|ref|Os08g25470|
gi|7402482|ref|Os08g25480|
gi|7402496|ref|Os08g25600|
gi|7402504|ref|Os08g25680|
gi|7402505|ref|Os08g25690|
gi|7402510|ref|Os08g25740|
gi|7402512|ref|Os08g25760|
gi|7402513|ref|Os08g25770|
gi|7402514|ref|Os08g25780|
gi|7402519|ref|Os08g25830|
gi|7402521|ref|Os08g25850|
gi|7402522|ref|Os08g25860|
gi|7402523|ref|Os08g25870|
gi|7402527|ref|Os08g25910|
gi|7402528|ref|Os08g25920|
gi|7402529|ref|Os08g25930|
gi|7402539|ref|Os08g26030|
gi|7402540|ref|Os08g26040|
gi|7402541|ref|Os08g26050|
gi|7402542|ref|Os08g26060|
gi|7402544|ref|Os08g26080|
gi|7402546|ref|Os08g26100|
gi|7402548|ref|Os08g26120|
gi|7402550|ref|Os08g26140|
gi|7402551|ref|Os08g26150|
gi|7402552|ref|Os08g26160|
gi|7402553|ref|Os08g26170|
gi|7402557|ref|Os08g26210|
gi|7402559|ref|Os08g26230|
gi|7402560|ref|Os08g26230|
gi|7402561|ref|Os08g26230|
gi|7402562|ref|Os08g26240|
gi|7402563|ref|Os08g26250|
gi|7402565|ref|Os08g26270|
gi|7402569|ref|Os08g26310|
gi|7402570|ref|Os08g26320|
gi|7402572|ref|Os08g26340|
gi|7402573|ref|Os08g26350|
gi|7402574|ref|Os08g26360|

gi|7402575|ref|Os08g26370|
gi|7402576|ref|Os08g26380|
gi|7402579|ref|Os08g26410|
gi|7402580|ref|Os08g26420|
gi|7402582|ref|Os08g26440|
gi|7402584|ref|Os08g26460|
gi|7402585|ref|Os08g26470|
gi|7402587|ref|Os08g26490|
gi|7402590|ref|Os08g26520|
gi|7402591|ref|Os08g26530|
gi|7402592|ref|Os08g26540|
gi|7402593|ref|Os08g26550|
gi|7402595|ref|Os08g26570|
gi|7402596|ref|Os08g26580|
gi|7402597|ref|Os08g26590|
gi|7402599|ref|Os08g26610|
gi|7402600|ref|Os08g26620|
gi|7402601|ref|Os08g26630|
gi|7402602|ref|Os08g26640|
gi|7402605|ref|Os08g26670|
gi|7402606|ref|Os08g26680|
gi|7402608|ref|Os08g26700|
gi|7402610|ref|Os08g26720|
gi|7402611|ref|Os08g26730|
gi|7402617|ref|Os08g26790|
gi|7402619|ref|Os08g26810|
gi|7402621|ref|Os08g26830|
gi|7402624|ref|Os08g26860|
gi|7402634|ref|Os08g26940|
gi|7402635|ref|Os08g26950|
gi|7402636|ref|Os08g26960|
gi|7402638|ref|Os08g26980|
gi|7402642|ref|Os08g27020|
gi|7402648|ref|Os08g27060|
gi|7402650|ref|Os08g27080|
gi|7402652|ref|Os08g27100|
gi|7402654|ref|Os08g27120|
gi|7402655|ref|Os08g27130|
gi|7402658|ref|Os08g27160|
gi|7402663|ref|Os08g27210|
gi|7402665|ref|Os08g27230|
gi|7402667|ref|Os08g27250|
gi|7402668|ref|Os08g27260|
gi|7402675|ref|Os08g27330|
gi|7402679|ref|Os08g27370|
gi|7402681|ref|Os08g27390|
gi|7402685|ref|Os08g27430|
gi|7402686|ref|Os08g27440|
gi|7402689|ref|Os08g27470|
gi|7402690|ref|Os08g27480|
gi|7402692|ref|Os08g27500|
gi|7402693|ref|Os08g27510|
gi|7402695|ref|Os08g27530|
gi|7402697|ref|Os08g27550|
gi|7402698|ref|Os08g27560|
gi|7402700|ref|Os08g27580|
gi|7402701|ref|Os08g27580|
gi|7402703|ref|Os08g27600|
gi|7402707|ref|Os08g27640|
gi|7402709|ref|Os08g27660|
gi|7402711|ref|Os08g27680|
gi|7402712|ref|Os08g27690|
gi|7402714|ref|Os08g27710|
gi|7402718|ref|Os08g27750|
gi|7402719|ref|Os08g27760|
gi|7402720|ref|Os08g27770|
gi|7402731|ref|Os08g27880|
gi|7402732|ref|Os08g27890|
gi|7402736|ref|Os08g27930|
gi|7402738|ref|Os08g27950|
gi|7402739|ref|Os08g27960|
gi|7402740|ref|Os08g27970|
gi|7402743|ref|Os08g28000|
gi|7402744|ref|Os08g28010|
gi|7402745|ref|Os08g28020|
gi|7402746|ref|Os08g28030|
gi|7402748|ref|Os08g28050|
gi|7402752|ref|Os08g28090|
gi|7402754|ref|Os08g28110|
gi|7402755|ref|Os08g28120|
gi|7402758|ref|Os08g28150|
gi|7402759|ref|Os08g28160|
gi|7402769|ref|Os08g28250|
gi|7402771|ref|Os08g28270|
gi|7402774|ref|Os08g28300|
gi|7402778|ref|Os08g28340|
gi|7402780|ref|Os08g28360|
gi|7402782|ref|Os08g28380|
gi|7402784|ref|Os08g28400|
gi|7402786|ref|Os08g28420|
gi|7402787|ref|Os08g28430|
gi|7402792|ref|Os08g28480|
gi|7402800|ref|Os08g28560|
gi|7402802|ref|Os08g28580|
gi|7402805|ref|Os08g28610|
gi|7402806|ref|Os08g28620|
gi|7402809|ref|Os08g28650|
gi|7402810|ref|Os08g28660|
gi|7402822|ref|Os08g28770|
gi|7402826|ref|Os08g28810|
gi|7402830|ref|Os08g28850|
gi|7402836|ref|Os08g28910|
gi|7402837|ref|Os08g28920|
gi|7402841|ref|Os08g28960|
gi|7402846|ref|Os08g29010|
gi|7402847|ref|Os08g29020|
gi|7402854|ref|Os08g29090|
gi|7402857|ref|Os08g29120|
gi|7402859|ref|Os08g29140|
gi|7402865|ref|Os08g29190|
gi|7402867|ref|Os08g29210|
gi|7402868|ref|Os08g29220|
gi|7402869|ref|Os08g29230|
gi|7402877|ref|Os08g29310|
gi|7402879|ref|Os08g29330|
gi|7402880|ref|Os08g29340|
gi|7402881|ref|Os08g29350|
gi|7402885|ref|Os08g29380|
gi|7402888|ref|Os08g29410|
gi|7402891|ref|Os08g29440|
gi|7402892|ref|Os08g29450|
gi|7402894|ref|Os08g29470|
gi|7402905|ref|Os08g29580|
gi|7402908|ref|Os08g29610|
gi|7402909|ref|Os08g29620|
gi|7402910|ref|Os08g29630|
gi|7402911|ref|Os08g29640|
gi|7402915|ref|Os08g29680|
gi|7402919|ref|Os08g29720|
gi|7402924|ref|Os08g29740|
gi|7402925|ref|Os08g29750|
gi|7402929|ref|Os08g29790|
gi|7402931|ref|Os08g29810|
gi|7402935|ref|Os08g29850|

gi|7402937|ref|Os08g29870|
gi|7402939|ref|Os08g29890|
gi|7402944|ref|Os08g29940|
gi|7402946|ref|Os08g29960|
gi|7402953|ref|Os08g30030|
gi|7402959|ref|Os08g30090|
gi|7402962|ref|Os08g30120|
gi|7402964|ref|Os08g30140|
gi|7402967|ref|Os08g30170|
gi|7402968|ref|Os08g30180|
gi|7402972|ref|Os08g30220|
gi|7402978|ref|Os08g30280|
gi|7402982|ref|Os08g30320|
gi|7402986|ref|Os08g30360|
gi|7402987|ref|Os08g30370|
gi|7402989|ref|Os08g30390|
gi|7402991|ref|Os08g30410|
gi|7402992|ref|Os08g30420|
gi|7402993|ref|Os08g30430|
gi|7402996|ref|Os08g30460|
gi|7402997|ref|Os08g30470|
gi|7402999|ref|Os08g30490|
gi|7403000|ref|Os08g30500|
gi|7403001|ref|Os08g30510|
gi|7403004|ref|Os08g30540|
gi|7403006|ref|Os08g30560|
gi|7403008|ref|Os08g30580|
gi|7403012|ref|Os08g30620|
gi|7403016|ref|Os08g30650|
gi|7403020|ref|Os08g30690|
gi|7403023|ref|Os08g30720|
gi|7403032|ref|Os08g30800|
gi|7403035|ref|Os08g30830|
gi|7403036|ref|Os08g30840|
gi|7403038|ref|Os08g30860|
gi|7403044|ref|Os08g30920|
gi|7403049|ref|Os08g30970|
gi|7403050|ref|Os08g30980|
gi|7403054|ref|Os08g31020|
gi|7403056|ref|Os08g31040|
gi|7403057|ref|Os08g31050|
gi|7403059|ref|Os08g31070|
gi|7403061|ref|Os08g31090|
gi|7403062|ref|Os08g31100|
gi|7403064|ref|Os08g31120|
gi|7403070|ref|Os08g31180|
gi|7403078|ref|Os08g31260|
gi|7403079|ref|Os08g31270|
gi|7403081|ref|Os08g31290|
gi|7403082|ref|Os08g31300|
gi|7403084|ref|Os08g31320|
gi|7403085|ref|Os08g31330|
gi|7403088|ref|Os08g31360|
gi|7403091|ref|Os08g31390|
gi|7403096|ref|Os08g31440|
gi|7403101|ref|Os08g31480|
gi|7403102|ref|Os08g31490|
gi|7403103|ref|Os08g31500|
gi|7403105|ref|Os08g31520|
gi|7403106|ref|Os08g31530|
gi|7403108|ref|Os08g31550|
gi|7403112|ref|Os08g31590|
gi|7403113|ref|Os08g31600|
gi|7403114|ref|Os08g31610|
gi|7403118|ref|Os08g31640|
gi|7403127|ref|Os08g31730|
gi|7403133|ref|Os08g31790|
gi|7403136|ref|Os08g31820|
gi|7403148|ref|Os08g31940|
gi|7403149|ref|Os08g31950|
gi|7403153|ref|Os08g31990|
gi|7403154|ref|Os08g32000|
gi|7403155|ref|Os08g32010|
gi|7403159|ref|Os08g32050|
gi|7403165|ref|Os08g32110|
gi|7403166|ref|Os08g32120|
gi|7403172|ref|Os08g32180|
gi|7403174|ref|Os08g32200|
gi|7403175|ref|Os08g32210|
gi|7403181|ref|Os08g32270|
gi|7403183|ref|Os08g32290|
gi|7403185|ref|Os08g32310|
gi|7403186|ref|Os08g32320|
gi|7403187|ref|Os08g32330|
gi|7403188|ref|Os08g32340|
gi|7403189|ref|Os08g32350|
gi|7403190|ref|Os08g32360|
gi|7403191|ref|Os08g32370|
gi|7403192|ref|Os08g32380|
gi|7403193|ref|Os08g32390|
gi|7403194|ref|Os08g32400|
gi|7403195|ref|Os08g32410|
gi|7403196|ref|Os08g32420|
gi|7403197|ref|Os08g32430|
gi|7403199|ref|Os08g32450|
gi|7403200|ref|Os08g32460|
gi|7403201|ref|Os08g32470|
gi|7403203|ref|Os08g32490|
gi|7403205|ref|Os08g32510|
gi|7403207|ref|Os08g32530|
gi|7403209|ref|Os08g32550|
gi|7403210|ref|Os08g32560|
gi|7403211|ref|Os08g32570|
gi|7403217|ref|Os08g32640|
gi|7403225|ref|Os08g32670|
gi|7403226|ref|Os08g32680|
gi|7403228|ref|Os08g32700|
gi|7403229|ref|Os08g32710|
gi|7403230|ref|Os08g32720|
gi|7403232|ref|Os08g32740|
gi|7403240|ref|Os08g32820|
gi|7403245|ref|Os08g32860|
gi|7403249|ref|Os08g32900|
gi|7403254|ref|Os08g32950|
gi|7403262|ref|Os08g33020|
gi|7403263|ref|Os08g33030|
gi|7403271|ref|Os08g33110|
gi|7403278|ref|Os08g33170|
gi|7403285|ref|Os08g33240|
gi|7403290|ref|Os08g33290|
gi|7403292|ref|Os08g33310|
gi|7403293|ref|Os08g33320|
gi|7403304|ref|Os08g33400|
gi|7403305|ref|Os08g33410|
gi|7403309|ref|Os08g33450|
gi|7403311|ref|Os08g33470|
gi|7403316|ref|Os08g33520|
gi|7403322|ref|Os08g33580|
gi|7403330|ref|Os08g33660|
gi|7403334|ref|Os08g33690|
gi|7403338|ref|Os08g33730|
gi|7403342|ref|Os08g33770|
gi|7403344|ref|Os08g33790|
gi|7403349|ref|Os08g33840|

gi|7403356|ref|Os08g33890|
gi|7403363|ref|Os08g33960|
gi|7403364|ref|Os08g33970|
gi|7403365|ref|Os08g33980|
gi|7403366|ref|Os08g33990|
gi|7403370|ref|Os08g34030|
gi|7403371|ref|Os08g34040|
gi|7403375|ref|Os08g34080|
gi|7403377|ref|Os08g34100|
gi|7403385|ref|Os08g34180|
gi|7403387|ref|Os08g34200|
gi|7403406|ref|Os08g34390|
gi|7403408|ref|Os08g34410|
gi|7403410|ref|Os08g34430|
gi|7403412|ref|Os08g34450|
gi|7403414|ref|Os08g34470|
gi|7403417|ref|Os08g34500|
gi|7403418|ref|Os08g34510|
gi|7403419|ref|Os08g34520|
gi|7403423|ref|Os08g34560|
gi|7403429|ref|Os08g34610|
gi|7403430|ref|Os08g34620|
gi|7403432|ref|Os08g34630|
gi|7403434|ref|Os08g34660|
gi|7403435|ref|Os08g34670|
gi|7403438|ref|Os08g34700|
gi|7403439|ref|Os08g34710|
gi|7403441|ref|Os08g34730|
gi|7403448|ref|Os08g34800|
gi|7403452|ref|Os08g34840|
gi|7403455|ref|Os08g34870|
gi|7403456|ref|Os08g34880|
gi|7403460|ref|Os08g34920|
gi|7403461|ref|Os08g34930|
gi|7403462|ref|Os08g34940|
gi|7403466|ref|Os08g34980|
gi|7403473|ref|Os08g35030|
gi|7403478|ref|Os08g35080|
gi|7403480|ref|Os08g35100|
gi|7403482|ref|Os08g35120|
gi|7403483|ref|Os08g35130|
gi|7403484|ref|Os08g35140|
gi|7403485|ref|Os08g35150|
gi|7403488|ref|Os08g35180|
gi|7403492|ref|Os08g35200|
gi|7403495|ref|Os08g35230|
gi|7403498|ref|Os08g35260|
gi|7403500|ref|Os08g35280|
gi|7403504|ref|Os08g35320|
gi|7403507|ref|Os08g35350|
gi|7403508|ref|Os08g35360|
gi|7403513|ref|Os08g35410|
gi|7403515|ref|Os08g35430|
gi|7403518|ref|Os08g35460|
gi|7403522|ref|Os08g35500|
gi|7403525|ref|Os08g35530|
gi|7403527|ref|Os08g35550|
gi|7403528|ref|Os08g35560|
gi|7403529|ref|Os08g35570|
gi|7403530|ref|Os08g35580|
gi|7403531|ref|Os08g35590|
gi|7403537|ref|Os08g35640|
gi|7403539|ref|Os08g35660|
gi|7403541|ref|Os08g35680|
gi|7403546|ref|Os08g35730|
gi|7403550|ref|Os08g35770|
gi|7403552|ref|Os08g35790|
gi|7403557|ref|Os08g35840|
gi|7403558|ref|Os08g35850|
gi|7403562|ref|Os08g35890|
gi|7403572|ref|Os08g35980|
gi|7403573|ref|Os08g35990|
gi|7403579|ref|Os08g36050|
gi|7403580|ref|Os08g36060|
gi|7403583|ref|Os08g36090|
gi|7403584|ref|Os08g36100|
gi|7403586|ref|Os08g36120|
gi|7403592|ref|Os08g36180|
gi|7403595|ref|Os08g36210|
gi|7403597|ref|Os08g36230|
gi|7403598|ref|Os08g36240|
gi|7403601|ref|Os08g36270|
gi|7403604|ref|Os08g36300|
gi|7403611|ref|Os08g36350|
gi|7403613|ref|Os08g36370|
gi|7403616|ref|Os08g36400|
gi|7403619|ref|Os08g36430|
gi|7403622|ref|Os08g36460|
gi|7403623|ref|Os08g36470|
gi|7403628|ref|Os08g36510|
gi|7403635|ref|Os08g36580|
gi|7403637|ref|Os08g36600|
gi|7403638|ref|Os08g36610|
gi|7403641|ref|Os08g36640|
gi|7403643|ref|Os08g36660|
gi|7403648|ref|Os08g36710|
gi|7403657|ref|Os08g36800|
gi|7403658|ref|Os08g36810|
gi|7403660|ref|Os08g36830|
gi|7403662|ref|Os08g36850|
gi|7403666|ref|Os08g36890|
gi|7403673|ref|Os08g36950|
gi|7403675|ref|Os08g36970|
gi|7403681|ref|Os08g37020|
gi|7403686|ref|Os08g37070|
gi|7403687|ref|Os08g37080|
gi|7403688|ref|Os08g37090|
gi|7403690|ref|Os08g37110|
gi|7403695|ref|Os08g37160|
gi|7403698|ref|Os08g37190|
gi|7403701|ref|Os08g37220|
gi|7403702|ref|Os08g37230|
gi|7403705|ref|Os08g37260|
gi|7403706|ref|Os08g37260|
gi|7403711|ref|Os08g37300|
gi|7403716|ref|Os08g37340|
gi|7403718|ref|Os08g37360|
gi|7403723|ref|Os08g37410|
gi|7403724|ref|Os08g37420|
gi|7403728|ref|Os08g37460|
gi|7403733|ref|Os08g37510|
gi|7403742|ref|Os08g37590|
gi|7403745|ref|Os08g37620|
gi|7403746|ref|Os08g37630|
gi|7403747|ref|Os08g37640|
gi|7403748|ref|Os08g37650|
gi|7403751|ref|Os08g37680|
gi|7403752|ref|Os08g37690|
gi|7403754|ref|Os08g37710|
gi|7403755|ref|Os08g37720|
gi|7403756|ref|Os08g37720|
gi|7403761|ref|Os08g37770|
gi|7403767|ref|Os08g37830|
gi|7403773|ref|Os08g37890|

gi|7403775|ref|Os08g37910|
gi|7403784|ref|Os08g37980|
gi|7403785|ref|Os08g37990|
gi|7403786|ref|Os08g38000|
gi|7403787|ref|Os08g38010|
gi|7403789|ref|Os08g38030|
gi|7403791|ref|Os08g38050|
gi|7403793|ref|Os08g38070|
gi|7403796|ref|Os08g38100|
gi|7403801|ref|Os08g38150|
gi|7403805|ref|Os08g38190|
gi|7403809|ref|Os08g38230|
gi|7403812|ref|Os08g38250|
gi|7403813|ref|Os08g38260|
gi|7403818|ref|Os08g38300|
gi|7403823|ref|Os08g38350|
gi|7403827|ref|Os08g38390|
gi|7403831|ref|Os08g38420|
gi|7403832|ref|Os08g38430|
gi|7403834|ref|Os08g38450|
gi|7403836|ref|Os08g38470|
gi|7403837|ref|Os08g38480|
gi|7403838|ref|Os08g38490|
gi|7403839|ref|Os08g38500|
gi|7403840|ref|Os08g38510|
gi|7403842|ref|Os08g38530|
gi|7403843|ref|Os08g38540|
gi|7403844|ref|Os08g38550|
gi|7403851|ref|Os08g38620|
gi|7403852|ref|Os08g38620|
gi|7403853|ref|Os08g38620|
gi|7403854|ref|Os08g38630|
gi|7403858|ref|Os08g38670|
gi|7403860|ref|Os08g38690|
gi|7403865|ref|Os08g38730|
gi|7403867|ref|Os08g38750|
gi|7403875|ref|Os08g38830|
gi|7403876|ref|Os08g38840|
gi|7403878|ref|Os08g38860|
gi|7403879|ref|Os08g38870|
gi|7403884|ref|Os08g38890|
gi|7403886|ref|Os08g38900|
gi|7403893|ref|Os08g38950|
gi|7403894|ref|Os08g38960|
gi|7403900|ref|Os08g39000|
gi|7403901|ref|Os08g39010|
gi|7403902|ref|Os08g39020|
gi|7403903|ref|Os08g39030|
gi|7403912|ref|Os08g39110|
gi|7403921|ref|Os08g39190|
gi|7403922|ref|Os08g39200|
gi|7403925|ref|Os08g39230|
gi|7403930|ref|Os08g39280|
gi|7403936|ref|Os08g39320|
gi|7403937|ref|Os08g39330|
gi|7403938|ref|Os08g39330|
gi|7403939|ref|Os08g39340|
gi|7403941|ref|Os08g39360|
gi|7403945|ref|Os08g39400|
gi|7403953|ref|Os08g39470|
gi|7403957|ref|Os08g39510|
gi|7403959|ref|Os08g39530|
gi|7403967|ref|Os08g39610|
gi|7403975|ref|Os08g39680|
gi|7403976|ref|Os08g39690|
gi|7403982|ref|Os08g39750|
gi|7403984|ref|Os08g39770|
gi|7404000|ref|Os08g39900|
gi|7404003|ref|Os08g39930|
gi|7404014|ref|Os08g40040|
gi|7404016|ref|Os08g40060|
gi|7404017|ref|Os08g40070|
gi|7404018|ref|Os08g40080|
gi|7404022|ref|Os08g40120|
gi|7404023|ref|Os08g40130|
gi|7404033|ref|Os08g40210|
gi|7404034|ref|Os08g40220|
gi|7404044|ref|Os08g40320|
gi|7404047|ref|Os08g40350|
gi|7404052|ref|Os08g40400|
gi|7404059|ref|Os08g40470|
gi|7404062|ref|Os08g40500|
gi|7404063|ref|Os08g40510|
gi|7404066|ref|Os08g40540|
gi|7404084|ref|Os08g40700|
gi|7404087|ref|Os08g40730|
gi|7404092|ref|Os08g40780|
gi|7404108|ref|Os08g40910|
gi|7404112|ref|Os08g40950|
gi|7404125|ref|Os08g41030|
gi|7404127|ref|Os08g41050|
gi|7404128|ref|Os08g41060|
gi|7404130|ref|Os08g41080|
gi|7404154|ref|Os08g41310|
gi|7404164|ref|Os08g41400|
gi|7404169|ref|Os08g41450|
gi|7404176|ref|Os08g41480|
gi|7404179|ref|Os08g41510|
gi|7404193|ref|Os08g41650|
gi|7404194|ref|Os08g41660|
gi|7404196|ref|Os08g41680|
gi|7404205|ref|Os08g41770|
gi|7404212|ref|Os08g41840|
gi|7404213|ref|Os08g41850|
gi|7404214|ref|Os08g41860|
gi|7404215|ref|Os08g41870|
gi|7404226|ref|Os08g41970|
gi|7404236|ref|Os08g42060|
gi|7404237|ref|Os08g42070|
gi|7404248|ref|Os08g42180|
gi|7404252|ref|Os08g42220|
gi|7404254|ref|Os08g42240|
gi|7404256|ref|Os08g42260|
gi|7404260|ref|Os08g42300|
gi|7404262|ref|Os08g42320|
gi|7404263|ref|Os08g42330|
gi|7404264|ref|Os08g42340|
gi|7404266|ref|Os08g42360|
gi|7404277|ref|Os08g42430|
gi|7404279|ref|Os08g42450|
gi|7404280|ref|Os08g42460|
gi|7404282|ref|Os08g42480|
gi|7404285|ref|Os08g42510|
gi|7404286|ref|Os08g42520|
gi|7404298|ref|Os08g42630|
gi|7404303|ref|Os08g42680|
gi|7404316|ref|Os08g42770|
gi|7404317|ref|Os08g42780|
gi|7404318|ref|Os08g42790|
gi|7404319|ref|Os08g42800|
gi|7404320|ref|Os08g42810|
gi|7404321|ref|Os08g42820|
gi|7404327|ref|Os08g42880|
gi|7404328|ref|Os08g42890|

gi|7404329|ref|Os08g42900|
gi|7404333|ref|Os08g42940|
gi|7404336|ref|Os08g42970|
gi|7404338|ref|Os08g42990|
gi|7404350|ref|Os08g43100|
gi|7404354|ref|Os08g43140|
gi|7404355|ref|Os08g43150|
gi|7404362|ref|Os08g43220|
gi|7404364|ref|Os08g43240|
gi|7404366|ref|Os08g43260|
gi|7404371|ref|Os08g43310|
gi|7404373|ref|Os08g43330|
gi|7404383|ref|Os08g43420|
gi|7404387|ref|Os08g43460|
gi|7404400|ref|Os08g43590|
gi|7404401|ref|Os08g43600|
gi|7404403|ref|Os08g43620|
gi|7404404|ref|Os08g43630|
gi|7404412|ref|Os08g43710|
gi|7404413|ref|Os08g43720|
gi|7404425|ref|Os08g43840|
gi|7404429|ref|Os08g43880|
gi|7404444|ref|Os08g44030|
gi|7404447|ref|Os08g44060|
gi|7404449|ref|Os08g44080|
gi|7404452|ref|Os08g44110|
gi|7404453|ref|Os08g44120|
gi|7404457|ref|Os08g44160|
gi|7404463|ref|Os08g44220|
gi|7404494|ref|Os08g44460|
gi|7404495|ref|Os08g44460|
gi|7404509|ref|Os08g44600|
gi|7404510|ref|Os08g44610|
gi|7404524|ref|Os08g44730|
gi|7404525|ref|Os08g44740|
gi|7404530|ref|Os08g44780|
gi|7404532|ref|Os08g44800|
gi|7404545|ref|Os08g44880|
gi|7404547|ref|Os08g44900|
gi|7404556|ref|Os08g44980|
gi|7404560|ref|Os08g45020|
gi|7404563|ref|Os08g45050|
gi|7404565|ref|Os08g45070|
gi|7404568|ref|Os08g45090|
gi|7404569|ref|Os08g45100|
gi|7404571|ref|Os08g45120|
gi|7404580|ref|Os08g45200|
gi|7404588|ref|Os08g45270|
gi|7404589|ref|Os08g45280|
gi|7600001|ref|Os10g01010|
gi|7600003|ref|Os10g01030|
gi|7600004|ref|Os10g01040|
gi|7600007|ref|Os10g01070|
gi|7600009|ref|Os10g01090|
gi|7600012|ref|Os10g01120|
gi|7600015|ref|Os10g01150|
gi|7600016|ref|Os10g01160|
gi|7600018|ref|Os10g01180|
gi|7600019|ref|Os10g01190|
gi|7600022|ref|Os10g01220|
gi|7600025|ref|Os10g01250|
gi|7600026|ref|Os10g01260|
gi|7600031|ref|Os10g01310|
gi|7600033|ref|Os10g01330|
gi|7600034|ref|Os10g01340|
gi|7600037|ref|Os10g01370|
gi|7600041|ref|Os10g01400|
gi|7600042|ref|Os10g01410|
gi|7600043|ref|Os10g01420|
gi|7600044|ref|Os10g01430|
gi|7600045|ref|Os10g01440|
gi|7600047|ref|Os10g01460|
gi|7600050|ref|Os10g01490|
gi|7600051|ref|Os10g01500|
gi|7600061|ref|Os10g01580|
gi|7600062|ref|Os10g01590|
gi|7600065|ref|Os10g01620|
gi|7600066|ref|Os10g01630|
gi|7600070|ref|Os10g01670|
gi|7600073|ref|Os10g01700|
gi|7600074|ref|Os10g01710|
gi|7600076|ref|Os10g01730|
gi|7600077|ref|Os10g01740|
gi|7600084|ref|Os10g01810|
gi|7600085|ref|Os10g01820|
gi|7600086|ref|Os10g01830|
gi|7600087|ref|Os10g01840|
gi|7600091|ref|Os10g01880|
gi|7600100|ref|Os10g01970|
gi|7600105|ref|Os10g02020|
gi|7600108|ref|Os10g02050|
gi|7600109|ref|Os10g02060|
gi|7600114|ref|Os10g02110|
gi|7600117|ref|Os10g02140|
gi|7600118|ref|Os10g02150|
gi|7600123|ref|Os10g02200|
gi|7600132|ref|Os10g02270|
gi|7600136|ref|Os10g02310|
gi|7600137|ref|Os10g02320|
gi|7600138|ref|Os10g02330|
gi|7600145|ref|Os10g02400|
gi|7600146|ref|Os10g02410|
gi|7600150|ref|Os10g02450|
gi|7600151|ref|Os10g02460|
gi|7600158|ref|Os10g02530|
gi|7600165|ref|Os10g02590|
gi|7600167|ref|Os10g02610|
gi|7600174|ref|Os10g02670|
gi|7600176|ref|Os10g02690|
gi|7600178|ref|Os10g02710|
gi|7600181|ref|Os10g02740|
gi|7600186|ref|Os10g02780|
gi|7600188|ref|Os10g02800|
gi|7600190|ref|Os10g02820|
gi|7600191|ref|Os10g02830|
gi|7600195|ref|Os10g02870|
gi|7600197|ref|Os10g02890|
gi|7600198|ref|Os10g02900|
gi|7600202|ref|Os10g02940|
gi|7600203|ref|Os10g02950|
gi|7600209|ref|Os10g03010|
gi|7600211|ref|Os10g03030|
gi|7600217|ref|Os10g03090|
gi|7600220|ref|Os10g03120|
gi|7600223|ref|Os10g03150|
gi|7600226|ref|Os10g03180|
gi|7600227|ref|Os10g03190|
gi|7600228|ref|Os10g03200|
gi|7600229|ref|Os10g03210|
gi|7600231|ref|Os10g03230|
gi|7600232|ref|Os10g03240|
gi|7600234|ref|Os10g03260|
gi|7600235|ref|Os10g03270|
gi|7600236|ref|Os10g03280|

gi|7600245|ref|Os10g03370|
gi|7600246|ref|Os10g03380|
gi|7600249|ref|Os10g03410|
gi|7600251|ref|Os10g03430|
gi|7600253|ref|Os10g03450|
gi|7600258|ref|Os10g03500|
gi|7600259|ref|Os10g03510|
gi|7600260|ref|Os10g03520|
gi|7600263|ref|Os10g03550|
gi|7600266|ref|Os10g03580|
gi|7600267|ref|Os10g03590|
gi|7600268|ref|Os10g03600|
gi|7600271|ref|Os10g03630|
gi|7600272|ref|Os10g03640|
gi|7600280|ref|Os10g03720|
gi|7600285|ref|Os10g03770|
gi|7600287|ref|Os10g03790|
gi|7600288|ref|Os10g03800|
gi|7600290|ref|Os10g03820|
gi|7600292|ref|Os10g03840|
gi|7600295|ref|Os10g03860|
gi|7600298|ref|Os10g03890|
gi|7600303|ref|Os10g03940|
gi|7600305|ref|Os10g03960|
gi|7600311|ref|Os10g04020|
gi|7600312|ref|Os10g04030|
gi|7600317|ref|Os10g04080|
gi|7600319|ref|Os10g04100|
gi|7600326|ref|Os10g04170|
gi|7600329|ref|Os10g04200|
gi|7600330|ref|Os10g04210|
gi|7600335|ref|Os10g04260|
gi|7600337|ref|Os10g04280|
gi|7600339|ref|Os10g04300|
gi|7600342|ref|Os10g04330|
gi|7600343|ref|Os10g04340|
gi|7600344|ref|Os10g04350|
gi|7600346|ref|Os10g04370|
gi|7600348|ref|Os10g04390|
gi|7600350|ref|Os10g04410|
gi|7600355|ref|Os10g04460|
gi|7600359|ref|Os10g04500|
gi|7600362|ref|Os10g04530|
gi|7600365|ref|Os10g04560|
gi|7600368|ref|Os10g04590|
gi|7600369|ref|Os10g04600|
gi|7600370|ref|Os10g04600|
gi|7600371|ref|Os10g04610|
gi|7600372|ref|Os10g04610|
gi|7600374|ref|Os10g04630|
gi|7600377|ref|Os10g04660|
gi|7600379|ref|Os10g04680|
gi|7600380|ref|Os10g04690|
gi|7600382|ref|Os10g04710|
gi|7600386|ref|Os10g04750|
gi|7600387|ref|Os10g04760|
gi|7600388|ref|Os10g04770|
gi|7600389|ref|Os10g04780|
gi|7600390|ref|Os10g04790|
gi|7600391|ref|Os10g04800|
gi|7600393|ref|Os10g04820|
gi|7600394|ref|Os10g04830|
gi|7600395|ref|Os10g04840|
gi|7600398|ref|Os10g04870|
gi|7600399|ref|Os10g04880|
gi|7600400|ref|Os10g04890|
gi|7600402|ref|Os10g04910|
gi|7600404|ref|Os10g04930|
gi|7600405|ref|Os10g04940|
gi|7600406|ref|Os10g04950|
gi|7600407|ref|Os10g04960|
gi|7600408|ref|Os10g04970|
gi|7600409|ref|Os10g04980|
gi|7600410|ref|Os10g04990|
gi|7600412|ref|Os10g05010|
gi|7600414|ref|Os10g05030|
gi|7600420|ref|Os10g05090|
gi|7600422|ref|Os10g05110|
gi|7600423|ref|Os10g05120|
gi|7600424|ref|Os10g05130|
gi|7600425|ref|Os10g05140|
gi|7600426|ref|Os10g05150|
gi|7600431|ref|Os10g05200|
gi|7600438|ref|Os10g05260|
gi|7600439|ref|Os10g05270|
gi|7600440|ref|Os10g05280|
gi|7600443|ref|Os10g05310|
gi|7600445|ref|Os10g05330|
gi|7600447|ref|Os10g05350|
gi|7600448|ref|Os10g05360|
gi|7600449|ref|Os10g05370|
gi|7600451|ref|Os10g05390|
gi|7600454|ref|Os10g05420|
gi|7600455|ref|Os10g05430|
gi|7600457|ref|Os10g05450|
gi|7600458|ref|Os10g05460|
gi|7600459|ref|Os10g05470|
gi|7600462|ref|Os10g05500|
gi|7600463|ref|Os10g05510|
gi|7600468|ref|Os10g05550|
gi|7600469|ref|Os10g05560|
gi|7600471|ref|Os10g05580|
gi|7600474|ref|Os10g05610|
gi|7600476|ref|Os10g05630|
gi|7600477|ref|Os10g05640|
gi|7600488|ref|Os10g05740|
gi|7600490|ref|Os10g05760|
gi|7600491|ref|Os10g05770|
gi|7600497|ref|Os10g05830|
gi|7600501|ref|Os10g05870|
gi|7600503|ref|Os10g05890|
gi|7600504|ref|Os10g05900|
gi|7600506|ref|Os10g05920|
gi|7600510|ref|Os10g05960|
gi|7600517|ref|Os10g06020|
gi|7600519|ref|Os10g06040|
gi|7600523|ref|Os10g06080|
gi|7600527|ref|Os10g06120|
gi|7600532|ref|Os10g06150|
gi|7600533|ref|Os10g06160|
gi|7600538|ref|Os10g06210|
gi|7600539|ref|Os10g06220|
gi|7600541|ref|Os10g06240|
gi|7600545|ref|Os10g06280|
gi|7600547|ref|Os10g06300|
gi|7600549|ref|Os10g06320|
gi|7600553|ref|Os10g06360|
gi|7600556|ref|Os10g06390|
gi|7600561|ref|Os10g06440|
gi|7600564|ref|Os10g06470|
gi|7600565|ref|Os10g06480|
gi|7600567|ref|Os10g06500|
gi|7600575|ref|Os10g06570|
gi|7600578|ref|Os10g06600|

gi|7600580|ref|Os10g06620|
gi|7600583|ref|Os10g06650|
gi|7600587|ref|Os10g06690|
gi|7600591|ref|Os10g06730|
gi|7600593|ref|Os10g06750|
gi|7600597|ref|Os10g06790|
gi|7600599|ref|Os10g06810|
gi|7600600|ref|Os10g06820|
gi|7600603|ref|Os10g06850|
gi|7600605|ref|Os10g06870|
gi|7600607|ref|Os10g06890|
gi|7600608|ref|Os10g06900|
gi|7600610|ref|Os10g06920|
gi|7600612|ref|Os10g06940|
gi|7600614|ref|Os10g06960|
gi|7600620|ref|Os10g07020|
gi|7600621|ref|Os10g07030|
gi|7600623|ref|Os10g07050|
gi|7600626|ref|Os10g07080|
gi|7600629|ref|Os10g07110|
gi|7600630|ref|Os10g07120|
gi|7600633|ref|Os10g07150|
gi|7600644|ref|Os10g07250|
gi|7600648|ref|Os10g07280|
gi|7600652|ref|Os10g07320|
gi|7600654|ref|Os10g07340|
gi|7600655|ref|Os10g07350|
gi|7600656|ref|Os10g07360|
gi|7600658|ref|Os10g07380|
gi|7600659|ref|Os10g07390|
gi|7600662|ref|Os10g07420|
gi|7600663|ref|Os10g07430|
gi|7600664|ref|Os10g07440|
gi|7600665|ref|Os10g07450|
gi|7600667|ref|Os10g07470|
gi|7600669|ref|Os10g07490|
gi|7600670|ref|Os10g07500|
gi|7600672|ref|Os10g07520|
gi|7600674|ref|Os10g08030|
gi|7600678|ref|Os10g08070|
gi|7600682|ref|Os10g08110|
gi|7600684|ref|Os10g08130|
gi|7600688|ref|Os10g08170|
gi|7600691|ref|Os10g08200|
gi|7600695|ref|Os10g08240|
gi|7600700|ref|Os10g08290|
gi|7600703|ref|Os10g08320|
gi|7600707|ref|Os10g08360|
gi|7600709|ref|Os10g08380|
gi|7600711|ref|Os10g08400|
gi|7600714|ref|Os10g08430|
gi|7600715|ref|Os10g08440|
gi|7600717|ref|Os10g08460|
gi|7600720|ref|Os10g08490|
gi|7600721|ref|Os10g08500|
gi|7600723|ref|Os10g08520|
gi|7600724|ref|Os10g08530|
gi|7600731|ref|Os10g08590|
gi|7600733|ref|Os10g08610|
gi|7600735|ref|Os10g08630|
gi|7600736|ref|Os10g08640|
gi|7600744|ref|Os10g08720|
gi|7600745|ref|Os10g08730|
gi|7600747|ref|Os10g08750|
gi|7600748|ref|Os10g08760|
gi|7600749|ref|Os10g08770|
gi|7600750|ref|Os10g08780|
gi|7600752|ref|Os10g08800|
gi|7600755|ref|Os10g08830|
gi|7600760|ref|Os10g08860|
gi|7600770|ref|Os10g08950|
gi|7600775|ref|Os10g09000|
gi|7600776|ref|Os10g09010|
gi|7600777|ref|Os10g09020|
gi|7600779|ref|Os10g09040|
gi|7600780|ref|Os10g09050|
gi|7600783|ref|Os10g09080|
gi|7600788|ref|Os10g09120|
gi|7600790|ref|Os10g09140|
gi|7600791|ref|Os10g09150|
gi|7600793|ref|Os10g09170|
gi|7600794|ref|Os10g09180|
gi|7600795|ref|Os10g09190|
gi|7600796|ref|Os10g09200|
gi|7600797|ref|Os10g09210|
gi|7600800|ref|Os10g09240|
gi|7600801|ref|Os10g09250|
gi|7600802|ref|Os10g09260|
gi|7600803|ref|Os10g09270|
gi|7600805|ref|Os10g09290|
gi|7600806|ref|Os10g09300|
gi|7600808|ref|Os10g09320|
gi|7600810|ref|Os10g09340|
gi|7600817|ref|Os10g09410|
gi|7600818|ref|Os10g09420|
gi|7600828|ref|Os10g09520|
gi|7600830|ref|Os10g09540|
gi|7600832|ref|Os10g09560|
gi|7600835|ref|Os10g09590|
gi|7600839|ref|Os10g09630|
gi|7600841|ref|Os10g09650|
gi|7600842|ref|Os10g09660|
gi|7600843|ref|Os10g09670|
gi|7600844|ref|Os10g09680|
gi|7600849|ref|Os10g09730|
gi|7600850|ref|Os10g09740|
gi|7600851|ref|Os10g09750|
gi|7600852|ref|Os10g09760|
gi|7600853|ref|Os10g09770|
gi|7600854|ref|Os10g09780|
gi|7600855|ref|Os10g09790|
gi|7600856|ref|Os10g09800|
gi|7600859|ref|Os10g09830|
gi|7600860|ref|Os10g09840|
gi|7600867|ref|Os10g09910|
gi|7600874|ref|Os10g09980|
gi|7600876|ref|Os10g10000|
gi|7600877|ref|Os10g10010|
gi|7600881|ref|Os10g10050|
gi|7600883|ref|Os10g10070|
gi|7600887|ref|Os10g10090|
gi|7600895|ref|Os10g10140|
gi|7600897|ref|Os10g10160|
gi|7600906|ref|Os10g10230|
gi|7600911|ref|Os10g10280|
gi|7600912|ref|Os10g10290|
gi|7600914|ref|Os10g10310|
gi|7600916|ref|Os10g10330|
gi|7600917|ref|Os10g10340|
gi|7600920|ref|Os10g10370|
gi|7600922|ref|Os10g10390|
gi|7600923|ref|Os10g10400|
gi|7600924|ref|Os10g10410|
gi|7600928|ref|Os10g10450|

gi|7600934|ref|Os10g10510|
gi|7600936|ref|Os10g10530|
gi|7600938|ref|Os10g10550|
gi|7600940|ref|Os10g10570|
gi|7600943|ref|Os10g10590|
gi|7600948|ref|Os10g10640|
gi|7600949|ref|Os10g10650|
gi|7600955|ref|Os10g10710|
gi|7600958|ref|Os10g10740|
gi|7600960|ref|Os10g10760|
gi|7600963|ref|Os10g10790|
gi|7600964|ref|Os10g10800|
gi|7600969|ref|Os10g10850|
gi|7600970|ref|Os10g10860|
gi|7600972|ref|Os10g10880|
gi|7600973|ref|Os10g10890|
gi|7600974|ref|Os10g10900|
gi|7600975|ref|Os10g10910|
gi|7600977|ref|Os10g10930|
gi|7600978|ref|Os10g10940|
gi|7600980|ref|Os10g10960|
gi|7600987|ref|Os10g11010|
gi|7600988|ref|Os10g11020|
gi|7600989|ref|Os10g11030|
gi|7600996|ref|Os10g11100|
gi|7600998|ref|Os10g11120|
gi|7600999|ref|Os10g11130|
gi|7601003|ref|Os10g11170|
gi|7601010|ref|Os10g11240|
gi|7601020|ref|Os10g11320|
gi|7601024|ref|Os10g11360|
gi|7601027|ref|Os10g11390|
gi|7601030|ref|Os10g11420|
gi|7601032|ref|Os10g11440|
gi|7601033|ref|Os10g11450|
gi|7601034|ref|Os10g11460|
gi|7601036|ref|Os10g11480|
gi|7601039|ref|Os10g11510|
gi|7601042|ref|Os10g11540|
gi|7601043|ref|Os10g11550|
gi|7601044|ref|Os10g11560|
gi|7601045|ref|Os10g11570|
gi|7601048|ref|Os10g11600|
gi|7601049|ref|Os10g11610|
gi|7601051|ref|Os10g11630|
gi|7601052|ref|Os10g11640|
gi|7601059|ref|Os10g11710|
gi|7601060|ref|Os10g11720|
gi|7601064|ref|Os10g11760|
gi|7601065|ref|Os10g11770|
gi|7601071|ref|Os10g11820|
gi|7601072|ref|Os10g11830|
gi|7601073|ref|Os10g11840|
gi|7601074|ref|Os10g11850|
gi|7601078|ref|Os10g11890|
gi|7601079|ref|Os10g11900|
gi|7601083|ref|Os10g11940|
gi|7601086|ref|Os10g11970|
gi|7601093|ref|Os10g12040|
gi|7601095|ref|Os10g12060|
gi|7601096|ref|Os10g12070|
gi|7601099|ref|Os10g12100|
gi|7601102|ref|Os10g12130|
gi|7601104|ref|Os10g12150|
gi|7601107|ref|Os10g12180|
gi|7601110|ref|Os10g12210|
gi|7601114|ref|Os10g12250|
gi|7601118|ref|Os10g12290|
gi|7601119|ref|Os10g12300|
gi|7601120|ref|Os10g12310|
gi|7601121|ref|Os10g12320|
gi|7601124|ref|Os10g12350|
gi|7601126|ref|Os10g12370|
gi|7601127|ref|Os10g12380|
gi|7601128|ref|Os10g12390|
gi|7601131|ref|Os10g12420|
gi|7601132|ref|Os10g12430|
gi|7601133|ref|Os10g12440|
gi|7601134|ref|Os10g12450|
gi|7601138|ref|Os10g12490|
gi|7601140|ref|Os10g12510|
gi|7601149|ref|Os10g12600|
gi|7601150|ref|Os10g12610|
gi|7601153|ref|Os10g12640|
gi|7601154|ref|Os10g12650|
gi|7601155|ref|Os10g12660|
gi|7601157|ref|Os10g12680|
gi|7601159|ref|Os10g12700|
gi|7601161|ref|Os10g12720|
gi|7601162|ref|Os10g12730|
gi|7601167|ref|Os10g12780|
gi|7601168|ref|Os10g12790|
gi|7601171|ref|Os10g13310|
gi|7601173|ref|Os10g13330|
gi|7601178|ref|Os10g13380|
gi|7601181|ref|Os10g13410|
gi|7601186|ref|Os10g13460|
gi|7601192|ref|Os10g13520|
gi|7601194|ref|Os10g13540|
gi|7601198|ref|Os10g13560|
gi|7601201|ref|Os10g13590|
gi|7601204|ref|Os10g13620|
gi|7601206|ref|Os10g13640|
gi|7601207|ref|Os10g13650|
gi|7601208|ref|Os10g13660|
gi|7601213|ref|Os10g13710|
gi|7601214|ref|Os10g13720|
gi|7601216|ref|Os10g13740|
gi|7601217|ref|Os10g13750|
gi|7601218|ref|Os10g13760|
gi|7601220|ref|Os10g13780|
gi|7601221|ref|Os10g13790|
gi|7601225|ref|Os10g13820|
gi|7601227|ref|Os10g13840|
gi|7601231|ref|Os10g13880|
gi|7601232|ref|Os10g13890|
gi|7601233|ref|Os10g13900|
gi|7601234|ref|Os10g13910|
gi|7601236|ref|Os10g13930|
gi|7601238|ref|Os10g13950|
gi|7601244|ref|Os10g14010|
gi|7601248|ref|Os10g14050|
gi|7601249|ref|Os10g14060|
gi|7601250|ref|Os10g14070|
gi|7601253|ref|Os10g14100|
gi|7601254|ref|Os10g14110|
gi|7601259|ref|Os10g14160|
gi|7601260|ref|Os10g14170|
gi|7601263|ref|Os10g14200|
gi|7601264|ref|Os10g14210|
gi|7601270|ref|Os10g14270|
gi|7601272|ref|Os10g14290|
gi|7601274|ref|Os10g14800|
gi|7601277|ref|Os10g14830|

gi|7601278|ref|Os10g14840|
gi|7601279|ref|Os10g14850|
gi|7601283|ref|Os10g14890|
gi|7601285|ref|Os10g14910|
gi|7601290|ref|Os10g14940|
gi|7601296|ref|Os10g15000|
gi|7601298|ref|Os10g15020|
gi|7601299|ref|Os10g15030|
gi|7601301|ref|Os10g15050|
gi|7601304|ref|Os10g15080|
gi|7601306|ref|Os10g15100|
gi|7601307|ref|Os10g15110|
gi|7601308|ref|Os10g15120|
gi|7601310|ref|Os10g15140|
gi|7601311|ref|Os10g15150|
gi|7601319|ref|Os10g15230|
gi|7601322|ref|Os10g15260|
gi|7601327|ref|Os10g15310|
gi|7601328|ref|Os10g15320|
gi|7601331|ref|Os10g15350|
gi|7601332|ref|Os10g15360|
gi|7601333|ref|Os10g15370|
gi|7601334|ref|Os10g15380|
gi|7601337|ref|Os10g16400|
gi|7601346|ref|Os10g16490|
gi|7601347|ref|Os10g16500|
gi|7601348|ref|Os10g16510|
gi|7601350|ref|Os10g16530|
gi|7601354|ref|Os10g16570|
gi|7601355|ref|Os10g16580|
gi|7601358|ref|Os10g16610|
gi|7601359|ref|Os10g16620|
gi|7601372|ref|Os10g16740|
gi|7601375|ref|Os10g16770|
gi|7601377|ref|Os10g16790|
gi|7601379|ref|Os10g16810|
gi|7601380|ref|Os10g16820|
gi|7601387|ref|Os10g16890|
gi|7601390|ref|Os10g16920|
gi|7601391|ref|Os10g16930|
gi|7601392|ref|Os10g16940|
gi|7601394|ref|Os10g16960|
gi|7601397|ref|Os10g16990|
gi|7601398|ref|Os10g17000|
gi|7601399|ref|Os10g17010|
gi|7601400|ref|Os10g17020|
gi|7601402|ref|Os10g17040|
gi|7601406|ref|Os10g17080|
gi|7601408|ref|Os10g17100|
gi|7601410|ref|Os10g17120|
gi|7601411|ref|Os10g17130|
gi|7601412|ref|Os10g17140|
gi|7601414|ref|Os10g17160|
gi|7601418|ref|Os10g17200|
gi|7601420|ref|Os10g17220|
gi|7601422|ref|Os10g17240|
gi|7601425|ref|Os10g17270|
gi|7601427|ref|Os10g17290|
gi|7601431|ref|Os10g17330|
gi|7601436|ref|Os10g17380|
gi|7601437|ref|Os10g17390|
gi|7601440|ref|Os10g17420|
gi|7601443|ref|Os10g17450|
gi|7601450|ref|Os10g17520|
gi|7601458|ref|Os10g17600|
gi|7601460|ref|Os10g17620|
gi|7601462|ref|Os10g17640|
gi|7601465|ref|Os10g17670|
gi|7601468|ref|Os10g17700|
gi|7601473|ref|Os10g17750|
gi|7601478|ref|Os10g17800|
gi|7601479|ref|Os10g17810|
gi|7601481|ref|Os10g17830|
gi|7601486|ref|Os10g17880|
gi|7601499|ref|Os10g18010|
gi|7601501|ref|Os10g18030|
gi|7601503|ref|Os10g18050|
gi|7601505|ref|Os10g18070|
gi|7601506|ref|Os10g18080|
gi|7601507|ref|Os10g18090|
gi|7601508|ref|Os10g18100|
gi|7601510|ref|Os10g18120|
gi|7601511|ref|Os10g18130|
gi|7601516|ref|Os10g18180|
gi|7601521|ref|Os10g18230|
gi|7601522|ref|Os10g18240|
gi|7601523|ref|Os10g18250|
gi|7601525|ref|Os10g18270|
gi|7601528|ref|Os10g18300|
gi|7601529|ref|Os10g18310|
gi|7601530|ref|Os10g18320|
gi|7601531|ref|Os10g18330|
gi|7601536|ref|Os10g18380|
gi|7601537|ref|Os10g18390|
gi|7601539|ref|Os10g18410|
gi|7601543|ref|Os10g18450|
gi|7601544|ref|Os10g18460|
gi|7601545|ref|Os10g18470|
gi|7601548|ref|Os10g18500|
gi|7601550|ref|Os10g18520|
gi|7601552|ref|Os10g18540|
gi|7601555|ref|Os10g18570|
gi|7601560|ref|Os10g18620|
gi|7601561|ref|Os10g18630|
gi|7601568|ref|Os10g18700|
gi|7601570|ref|Os10g18720|
gi|7601573|ref|Os10g18750|
gi|7601578|ref|Os10g18800|
gi|7601579|ref|Os10g18810|
gi|7601583|ref|Os10g18850|
gi|7601585|ref|Os10g18870|
gi|7601586|ref|Os10g18880|
gi|7601589|ref|Os10g18910|
gi|7601591|ref|Os10g18930|
gi|7601593|ref|Os10g18950|
gi|7601596|ref|Os10g18980|
gi|7601601|ref|Os10g19030|
gi|7601602|ref|Os10g19040|
gi|7601603|ref|Os10g19050|
gi|7601606|ref|Os10g19080|
gi|7601607|ref|Os10g19090|
gi|7601608|ref|Os10g19100|
gi|7601613|ref|Os10g19150|
gi|7601615|ref|Os10g19170|
gi|7601618|ref|Os10g19200|
gi|7601623|ref|Os10g19250|
gi|7601624|ref|Os10g19260|
gi|7601628|ref|Os10g19300|
gi|7601630|ref|Os10g19810|
gi|7601631|ref|Os10g19820|
gi|7601633|ref|Os10g19840|
gi|7601636|ref|Os10g19870|
gi|7601642|ref|Os10g19930|
gi|7601646|ref|Os10g19970|

gi|7601647|ref|Os10g19980|
gi|7601648|ref|Os10g19990|
gi|7601655|ref|Os10g20060|
gi|7601657|ref|Os10g20080|
gi|7601661|ref|Os10g20100|
gi|7601662|ref|Os10g20110|
gi|7601665|ref|Os10g20140|
gi|7601672|ref|Os10g20210|
gi|7601674|ref|Os10g20230|
gi|7601678|ref|Os10g20270|
gi|7601680|ref|Os10g20290|
gi|7601681|ref|Os10g20300|
gi|7601685|ref|Os10g20340|
gi|7601687|ref|Os10g20360|
gi|7601688|ref|Os10g20370|
gi|7601693|ref|Os10g20420|
gi|7601697|ref|Os10g20460|
gi|7601699|ref|Os10g20480|
gi|7601700|ref|Os10g20480|
gi|7601706|ref|Os10g20520|
gi|7601707|ref|Os10g20530|
gi|7601708|ref|Os10g20540|
gi|7601713|ref|Os10g20590|
gi|7601718|ref|Os10g20640|
gi|7601722|ref|Os10g20680|
gi|7601723|ref|Os10g20690|
gi|7601724|ref|Os10g20700|
gi|7601726|ref|Os10g20720|
gi|7601727|ref|Os10g20730|
gi|7601728|ref|Os10g20740|
gi|7601736|ref|Os10g20820|
gi|7601739|ref|Os10g20850|
gi|7601740|ref|Os10g20860|
gi|7601741|ref|Os10g20870|
gi|7601749|ref|Os10g20950|
gi|7601757|ref|Os10g21030|
gi|7601758|ref|Os10g21040|
gi|7601767|ref|Os10g21130|
gi|7601768|ref|Os10g21140|
gi|7601770|ref|Os10g21160|
gi|7601771|ref|Os10g21170|
gi|7601772|ref|Os10g21180|
gi|7601773|ref|Os10g21190|
gi|7601777|ref|Os10g21230|
gi|7601792|ref|Os10g21370|
gi|7601794|ref|Os10g21390|
gi|7601796|ref|Os10g21410|
gi|7601797|ref|Os10g21420|
gi|7601798|ref|Os10g21430|
gi|7601799|ref|Os10g21440|
gi|7601800|ref|Os10g21450|
gi|7601803|ref|Os10g21480|
gi|7601804|ref|Os10g21490|
gi|7601805|ref|Os10g21500|
gi|7601807|ref|Os10g21520|
gi|7601810|ref|Os10g21550|
gi|7601813|ref|Os10g21570|
gi|7601814|ref|Os10g21580|
gi|7601818|ref|Os10g21600|
gi|7601819|ref|Os10g21610|
gi|7601820|ref|Os10g21620|
gi|7601821|ref|Os10g21630|
gi|7601822|ref|Os10g21640|
gi|7601824|ref|Os10g21660|
gi|7601829|ref|Os10g21710|
gi|7601830|ref|Os10g21720|
gi|7601832|ref|Os10g21740|
gi|7601833|ref|Os10g21750|
gi|7601834|ref|Os10g21760|
gi|7601835|ref|Os10g21770|
gi|7601836|ref|Os10g21780|
gi|7601840|ref|Os10g21820|
gi|7601844|ref|Os10g21860|
gi|7601845|ref|Os10g21870|
gi|7601846|ref|Os10g21880|
gi|7601848|ref|Os10g21900|
gi|7601856|ref|Os10g21970|
gi|7601858|ref|Os10g21990|
gi|7601861|ref|Os10g22020|
gi|7601864|ref|Os10g22050|
gi|7601865|ref|Os10g22060|
gi|7601867|ref|Os10g22080|
gi|7601868|ref|Os10g22090|
gi|7601869|ref|Os10g22100|
gi|7601871|ref|Os10g22120|
gi|7601874|ref|Os10g22150|
gi|7601877|ref|Os10g22180|
gi|7601878|ref|Os10g22190|
gi|7601879|ref|Os10g22200|
gi|7601886|ref|Os10g22270|
gi|7601887|ref|Os10g22280|
gi|7601891|ref|Os10g22320|
gi|7601898|ref|Os10g22390|
gi|7601899|ref|Os10g22400|
gi|7601903|ref|Os10g22440|
gi|7601907|ref|Os10g22480|
gi|7601909|ref|Os10g22500|
gi|7601917|ref|Os10g22580|
gi|7601920|ref|Os10g22610|
gi|7601922|ref|Os10g22630|
gi|7601925|ref|Os10g22660|
gi|7601926|ref|Os10g22670|
gi|7601927|ref|Os10g22680|
gi|7601928|ref|Os10g22690|
gi|7601930|ref|Os10g22710|
gi|7601931|ref|Os10g22720|
gi|7601933|ref|Os10g22740|
gi|7601934|ref|Os10g22750|
gi|7601935|ref|Os10g22760|
gi|7601936|ref|Os10g22770|
gi|7601937|ref|Os10g22780|
gi|7601938|ref|Os10g22790|
gi|7601941|ref|Os10g22820|
gi|7601942|ref|Os10g22830|
gi|7601944|ref|Os10g22850|
gi|7601946|ref|Os10g22870|
gi|7601947|ref|Os10g22880|
gi|7601949|ref|Os10g22900|
gi|7601950|ref|Os10g22910|
gi|7601951|ref|Os10g22920|
gi|7601953|ref|Os10g22940|
gi|7601956|ref|Os10g22970|
gi|7601961|ref|Os10g23000|
gi|7601962|ref|Os10g23010|
gi|7601963|ref|Os10g23020|
gi|7601964|ref|Os10g23030|
gi|7601967|ref|Os10g23060|
gi|7601968|ref|Os10g23070|
gi|7601969|ref|Os10g23080|
gi|7601986|ref|Os10g23240|
gi|7601987|ref|Os10g23250|
gi|7601989|ref|Os10g23270|
gi|7601990|ref|Os10g23280|
gi|7601992|ref|Os10g23300|

gi|7601994|ref|Os10g23320|
gi|7601998|ref|Os10g23850|
gi|7602002|ref|Os10g23890|
gi|7602004|ref|Os10g23910|
gi|7602005|ref|Os10g23920|
gi|7602007|ref|Os10g23940|
gi|7602009|ref|Os10g23960|
gi|7602010|ref|Os10g23970|
gi|7602011|ref|Os10g23980|
gi|7602013|ref|Os10g24000|
gi|7602016|ref|Os10g24030|
gi|7602019|ref|Os10g24060|
gi|7602021|ref|Os10g24080|
gi|7602022|ref|Os10g24090|
gi|7602023|ref|Os10g24100|
gi|7602025|ref|Os10g24120|
gi|7602026|ref|Os10g24130|
gi|7602027|ref|Os10g24140|
gi|7602031|ref|Os10g24180|
gi|7602034|ref|Os10g24210|
gi|7602037|ref|Os10g24240|
gi|7602038|ref|Os10g24250|
gi|7602039|ref|Os10g24260|
gi|7602040|ref|Os10g24270|
gi|7602043|ref|Os10g24300|
gi|7602046|ref|Os10g24330|
gi|7602047|ref|Os10g24340|
gi|7602048|ref|Os10g24350|
gi|7602049|ref|Os10g24360|
gi|7602052|ref|Os10g24390|
gi|7602054|ref|Os10g24410|
gi|7602056|ref|Os10g24430|
gi|7602063|ref|Os10g24500|
gi|7602065|ref|Os10g24520|
gi|7602072|ref|Os10g24590|
gi|7602074|ref|Os10g24610|
gi|7602076|ref|Os10g24630|
gi|7602078|ref|Os10g24650|
gi|7602081|ref|Os10g24680|
gi|7602082|ref|Os10g24690|
gi|7602085|ref|Os10g24720|
gi|7602090|ref|Os10g24770|
gi|7602093|ref|Os10g24800|
gi|7602097|ref|Os10g24840|
gi|7602098|ref|Os10g24850|
gi|7602099|ref|Os10g24860|
gi|7602100|ref|Os10g24870|
gi|7602101|ref|Os10g24880|
gi|7602102|ref|Os10g24890|
gi|7602106|ref|Os10g24930|
gi|7602111|ref|Os10g24980|
gi|7602112|ref|Os10g24990|
gi|7602119|ref|Os10g25060|
gi|7602123|ref|Os10g25100|
gi|7602125|ref|Os10g25120|
gi|7602129|ref|Os10g25160|
gi|7602138|ref|Os10g25250|
gi|7602139|ref|Os10g25260|
gi|7602143|ref|Os10g25300|
gi|7602144|ref|Os10g25300|
gi|7602149|ref|Os10g25330|
gi|7602151|ref|Os10g25350|
gi|7602153|ref|Os10g25370|
gi|7602155|ref|Os10g25390|
gi|7602157|ref|Os10g25410|
gi|7602160|ref|Os10g25440|
gi|7602163|ref|Os10g25470|
gi|7602168|ref|Os10g25520|
gi|7602174|ref|Os10g25580|
gi|7602177|ref|Os10g25610|
gi|7602179|ref|Os10g25630|
gi|7602180|ref|Os10g25640|
gi|7602181|ref|Os10g25650|
gi|7602183|ref|Os10g25670|
gi|7602185|ref|Os10g25690|
gi|7602186|ref|Os10g25700|
gi|7602189|ref|Os10g25730|
gi|7602197|ref|Os10g25800|
gi|7602198|ref|Os10g25810|
gi|7602201|ref|Os10g25840|
gi|7602202|ref|Os10g25850|
gi|7602205|ref|Os10g25880|
gi|7602208|ref|Os10g25910|
gi|7602214|ref|Os10g25970|
gi|7602228|ref|Os10g26100|
gi|7602230|ref|Os10g26120|
gi|7602236|ref|Os10g26150|
gi|7602239|ref|Os10g26180|
gi|7602240|ref|Os10g26190|
gi|7602241|ref|Os10g26200|
gi|7602244|ref|Os10g26230|
gi|7602245|ref|Os10g26240|
gi|7602246|ref|Os10g26250|
gi|7602247|ref|Os10g26260|
gi|7602250|ref|Os10g26290|
gi|7602251|ref|Os10g26300|
gi|7602252|ref|Os10g26310|
gi|7602253|ref|Os10g26320|
gi|7602259|ref|Os10g26380|
gi|7602265|ref|Os10g26440|
gi|7602266|ref|Os10g26450|
gi|7602269|ref|Os10g26480|
gi|7602270|ref|Os10g26490|
gi|7602272|ref|Os10g26510|
gi|7602276|ref|Os10g26550|
gi|7602277|ref|Os10g26560|
gi|7602279|ref|Os10g26580|
gi|7602288|ref|Os10g26670|
gi|7602293|ref|Os10g26710|
gi|7602296|ref|Os10g26740|
gi|7602297|ref|Os10g26750|
gi|7602300|ref|Os10g26780|
gi|7602301|ref|Os10g26790|
gi|7602302|ref|Os10g26800|
gi|7602304|ref|Os10g26820|
gi|7602308|ref|Os10g26860|
gi|7602311|ref|Os10g26890|
gi|7602319|ref|Os10g26970|
gi|7602320|ref|Os10g26980|
gi|7602322|ref|Os10g27000|
gi|7602330|ref|Os10g27080|
gi|7602332|ref|Os10g27100|
gi|7602338|ref|Os10g27160|
gi|7602340|ref|Os10g27180|
gi|7602342|ref|Os10g27200|
gi|7602348|ref|Os10g27250|
gi|7602350|ref|Os10g27270|
gi|7602352|ref|Os10g27290|
gi|7602353|ref|Os10g27300|
gi|7602354|ref|Os10g27310|
gi|7602355|ref|Os10g27320|
gi|7602359|ref|Os10g27360|
gi|7602361|ref|Os10g27380|
gi|7602363|ref|Os10g27400|

gi|7602364|ref|Os10g27410|
gi|7602365|ref|Os10g27420|
gi|7602366|ref|Os10g27430|
gi|7602367|ref|Os10g27440|
gi|7602369|ref|Os10g27460|
gi|7602375|ref|Os10g28010|
gi|7602381|ref|Os10g28070|
gi|7602383|ref|Os10g28090|
gi|7602384|ref|Os10g28100|
gi|7602385|ref|Os10g28110|
gi|7602393|ref|Os10g28190|
gi|7602400|ref|Os10g28260|
gi|7602401|ref|Os10g28270|
gi|7602402|ref|Os10g28280|
gi|7602403|ref|Os10g28290|
gi|7602404|ref|Os10g28300|
gi|7602416|ref|Os10g28390|
gi|7602417|ref|Os10g28400|
gi|7602420|ref|Os10g28430|
gi|7602425|ref|Os10g28480|
gi|7602426|ref|Os10g28490|
gi|7602427|ref|Os10g28500|
gi|7602428|ref|Os10g28510|
gi|7602430|ref|Os10g28530|
gi|7602432|ref|Os10g28550|
gi|7602433|ref|Os10g28560|
gi|7602434|ref|Os10g28570|
gi|7602439|ref|Os10g28620|
gi|7602447|ref|Os10g28690|
gi|7602449|ref|Os10g28710|
gi|7602453|ref|Os10g28750|
gi|7602458|ref|Os10g28800|
gi|7602461|ref|Os10g28830|
gi|7602466|ref|Os10g28880|
gi|7602468|ref|Os10g28900|
gi|7602470|ref|Os10g28920|
gi|7602472|ref|Os10g28940|
gi|7602474|ref|Os10g28960|
gi|7602478|ref|Os10g29000|
gi|7602481|ref|Os10g29030|
gi|7602482|ref|Os10g29040|
gi|7602485|ref|Os10g29060|
gi|7602487|ref|Os10g29080|
gi|7602492|ref|Os10g29130|
gi|7602493|ref|Os10g29140|
gi|7602500|ref|Os10g29200|
gi|7602504|ref|Os10g29240|
gi|7602505|ref|Os10g29250|
gi|7602507|ref|Os10g29270|
gi|7602510|ref|Os10g29300|
gi|7602515|ref|Os10g29350|
gi|7602531|ref|Os10g29500|
gi|7602533|ref|Os10g29520|
gi|7602534|ref|Os10g29530|
gi|7602538|ref|Os10g29570|
gi|7602539|ref|Os10g29580|
gi|7602540|ref|Os10g29590|
gi|7602544|ref|Os10g29630|
gi|7602545|ref|Os10g29640|
gi|7602549|ref|Os10g29670|
gi|7602550|ref|Os10g29680|
gi|7602551|ref|Os10g29690|
gi|7602552|ref|Os10g29700|
gi|7602553|ref|Os10g29710|
gi|7602554|ref|Os10g29720|
gi|7602558|ref|Os10g29760|
gi|7602560|ref|Os10g29780|
gi|7602562|ref|Os10g29800|
gi|7602569|ref|Os10g29870|
gi|7602570|ref|Os10g29880|
gi|7602571|ref|Os10g29890|
gi|7602572|ref|Os10g29900|
gi|7602573|ref|Os10g29910|
gi|7602576|ref|Os10g29940|
gi|7602578|ref|Os10g29960|
gi|7602585|ref|Os10g30030|
gi|7602592|ref|Os10g30080|
gi|7602601|ref|Os10g30170|
gi|7602605|ref|Os10g30210|
gi|7602606|ref|Os10g30220|
gi|7602607|ref|Os10g30230|
gi|7602611|ref|Os10g30270|
gi|7602629|ref|Os10g30440|
gi|7602631|ref|Os10g30460|
gi|7602632|ref|Os10g30470|
gi|7602633|ref|Os10g30480|
gi|7602634|ref|Os10g30490|
gi|7602635|ref|Os10g30500|
gi|7602649|ref|Os10g30630|
gi|7602656|ref|Os10g30700|
gi|7602657|ref|Os10g30710|
gi|7602658|ref|Os10g30720|
gi|7602659|ref|Os10g30730|
gi|7602664|ref|Os10g30780|
gi|7602667|ref|Os10g30800|
gi|7602669|ref|Os10g30820|
gi|7602676|ref|Os10g30880|
gi|7602677|ref|Os10g30890|
gi|7602678|ref|Os10g30900|
gi|7602681|ref|Os10g30920|
gi|7602682|ref|Os10g30930|
gi|7602685|ref|Os10g30960|
gi|7602694|ref|Os10g31050|
gi|7602696|ref|Os10g31070|
gi|7602698|ref|Os10g31090|
gi|7602699|ref|Os10g31100|
gi|7602700|ref|Os10g31110|
gi|7602704|ref|Os10g31150|
gi|7602707|ref|Os10g31180|
gi|7602708|ref|Os10g31190|
gi|7602712|ref|Os10g31230|
gi|7602715|ref|Os10g31260|
gi|7602716|ref|Os10g31270|
gi|7602717|ref|Os10g31280|
gi|7602719|ref|Os10g31300|
gi|7602721|ref|Os10g31320|
gi|7602722|ref|Os10g31320|
gi|7602723|ref|Os10g31330|
gi|7602724|ref|Os10g31330|
gi|7602725|ref|Os10g31340|
gi|7602728|ref|Os10g31370|
gi|7602729|ref|Os10g31380|
gi|7602730|ref|Os10g31390|
gi|7602731|ref|Os10g31400|
gi|7602733|ref|Os10g31420|
gi|7602734|ref|Os10g31430|
gi|7602735|ref|Os10g31440|
gi|7602736|ref|Os10g31450|
gi|7602737|ref|Os10g31460|
gi|7602738|ref|Os10g31470|
gi|7602739|ref|Os10g31480|
gi|7602740|ref|Os10g31490|
gi|7602741|ref|Os10g31500|
gi|7602742|ref|Os10g31510|

gi|7602744|ref|Os10g31530|
gi|7602745|ref|Os10g31540|
gi|7602746|ref|Os10g31550|
gi|7602747|ref|Os10g31560|
gi|7602748|ref|Os10g31570|
gi|7602752|ref|Os10g31610|
gi|7602753|ref|Os10g31620|
gi|7602754|ref|Os10g31630|
gi|7602755|ref|Os10g31640|
gi|7602757|ref|Os10g31660|
gi|7602758|ref|Os10g31670|
gi|7602759|ref|Os10g31680|
gi|7602760|ref|Os10g31690|
gi|7602761|ref|Os10g31700|
gi|7602762|ref|Os10g31710|
gi|7602763|ref|Os10g31720|
gi|7602764|ref|Os10g31730|
gi|7602765|ref|Os10g31740|
gi|7602766|ref|Os10g31750|
gi|7602767|ref|Os10g31760|
gi|7602772|ref|Os10g31800|
gi|7602776|ref|Os10g31840|
gi|7602782|ref|Os10g31870|
gi|7602784|ref|Os10g31890|
gi|7602787|ref|Os10g31920|
gi|7602788|ref|Os10g31930|
gi|7602791|ref|Os10g31960|
gi|7602795|ref|Os10g32000|
gi|7602796|ref|Os10g32010|
gi|7602797|ref|Os10g32020|
gi|7602804|ref|Os10g32090|
gi|7602805|ref|Os10g32100|
gi|7602809|ref|Os10g32140|
gi|7602813|ref|Os10g32180|
gi|7602815|ref|Os10g32200|
gi|7602816|ref|Os10g32210|
gi|7602818|ref|Os10g32230|
gi|7602822|ref|Os10g32270|
gi|7602823|ref|Os10g32280|
gi|7602826|ref|Os10g32310|
gi|7602829|ref|Os10g32340|
gi|7602832|ref|Os10g32360|
gi|7602833|ref|Os10g32370|
gi|7602836|ref|Os10g32400|
gi|7602838|ref|Os10g32420|
gi|7602840|ref|Os10g32440|
gi|7602841|ref|Os10g32450|
gi|7602842|ref|Os10g32460|
gi|7602843|ref|Os10g32470|
gi|7602844|ref|Os10g32480|
gi|7602846|ref|Os10g32500|
gi|7602847|ref|Os10g32510|
gi|7602848|ref|Os10g32520|
gi|7602849|ref|Os10g32530|
gi|7602857|ref|Os10g32610|
gi|7602858|ref|Os10g32620|
gi|7602861|ref|Os10g32650|
gi|7602863|ref|Os10g32670|
gi|7602865|ref|Os10g32690|
gi|7602870|ref|Os10g32720|
gi|7602877|ref|Os10g32790|
gi|7602878|ref|Os10g32800|
gi|7602885|ref|Os10g32860|
gi|7602888|ref|Os10g32890|
gi|7602890|ref|Os10g32910|
gi|7602892|ref|Os10g32930|
gi|7602893|ref|Os10g32940|
gi|7602901|ref|Os10g33020|
gi|7602904|ref|Os10g33050|
gi|7602911|ref|Os10g33120|
gi|7602914|ref|Os10g33150|
gi|7602915|ref|Os10g33160|
gi|7602917|ref|Os10g33180|
gi|7602918|ref|Os10g33190|
gi|7602919|ref|Os10g33200|
gi|7602925|ref|Os10g33260|
gi|7602932|ref|Os10g33320|
gi|7602933|ref|Os10g33330|
gi|7602934|ref|Os10g33340|
gi|7602936|ref|Os10g33360|
gi|7602938|ref|Os10g33390|
gi|7602939|ref|Os10g33380|
gi|7602940|ref|Os10g33400|
gi|7602941|ref|Os10g33410|
gi|7602943|ref|Os10g33430|
gi|7602950|ref|Os10g33500|
gi|7602951|ref|Os10g33510|
gi|7602953|ref|Os10g33530|
gi|7602958|ref|Os10g33580|
gi|7602959|ref|Os10g33590|
gi|7602960|ref|Os10g33600|
gi|7602961|ref|Os10g33610|
gi|7602967|ref|Os10g33670|
gi|7602970|ref|Os10g33700|
gi|7602975|ref|Os10g33730|
gi|7602977|ref|Os10g33750|
gi|7602986|ref|Os10g33840|
gi|7602988|ref|Os10g33860|
gi|7602989|ref|Os10g33870|
gi|7602991|ref|Os10g33890|
gi|7602997|ref|Os10g33950|
gi|7603005|ref|Os10g34010|
gi|7603014|ref|Os10g34100|
gi|7603020|ref|Os10g34160|
gi|7603024|ref|Os10g34200|
gi|7603025|ref|Os10g34190|
gi|7603029|ref|Os10g34240|
gi|7603030|ref|Os10g34250|
gi|7603031|ref|Os10g34260|
gi|7603032|ref|Os10g34270|
gi|7603033|ref|Os10g34280|
gi|7603039|ref|Os10g34330|
gi|7603040|ref|Os10g34340|
gi|7603051|ref|Os10g34440|
gi|7603052|ref|Os10g34450|
gi|7603053|ref|Os10g34460|
gi|7603059|ref|Os10g34500|
gi|7603060|ref|Os10g34510|
gi|7603064|ref|Os10g34550|
gi|7603065|ref|Os10g34560|
gi|7603066|ref|Os10g34570|
gi|7603069|ref|Os10g34600|
gi|7603070|ref|Os10g34610|
gi|7603075|ref|Os10g34650|
gi|7603079|ref|Os10g34690|
gi|7603080|ref|Os10g34700|
gi|7603088|ref|Os10g34770|
gi|7603089|ref|Os10g34780|
gi|7603091|ref|Os10g34800|
gi|7603094|ref|Os10g34830|
gi|7603095|ref|Os10g34840|
gi|7603096|ref|Os10g34850|
gi|7603098|ref|Os10g34870|
gi|7603099|ref|Os10g34880|

gi|7603101|ref|Os10g34900|
gi|7603105|ref|Os10g34940|
gi|7603106|ref|Os10g34950|
gi|7603109|ref|Os10g34980|
gi|7603119|ref|Os10g35080|
gi|7603121|ref|Os10g35100|
gi|7603124|ref|Os10g35120|
gi|7603128|ref|Os10g35160|
gi|7603133|ref|Os10g35210|
gi|7603140|ref|Os10g35270|
gi|7603145|ref|Os10g35310|
gi|7603148|ref|Os10g35340|
gi|7603150|ref|Os10g35360|
gi|7603177|ref|Os10g35590|
gi|7603178|ref|Os10g35600|
gi|7603179|ref|Os10g35610|
gi|7603180|ref|Os10g35620|
gi|7603188|ref|Os10g35700|
gi|7603195|ref|Os10g35740|
gi|7603199|ref|Os10g35780|
gi|7603203|ref|Os10g35820|
gi|7603204|ref|Os10g35830|
gi|7603212|ref|Os10g35900|
gi|7603214|ref|Os10g35910|
gi|7603226|ref|Os10g36010|
gi|7603227|ref|Os10g36020|
gi|7603229|ref|Os10g36040|
gi|7603230|ref|Os10g36050|
gi|7603233|ref|Os10g36080|
gi|7603237|ref|Os10g36120|
gi|7603238|ref|Os10g36130|
gi|7603243|ref|Os10g36180|
gi|7603247|ref|Os10g36220|
gi|7603248|ref|Os10g36230|
gi|7603249|ref|Os10g36240|
gi|7603252|ref|Os10g36260|
gi|7603255|ref|Os10g36290|
gi|7603257|ref|Os10g36310|
gi|7603259|ref|Os10g36330|
gi|7603263|ref|Os10g36360|
gi|7603268|ref|Os10g36410|
gi|7603280|ref|Os10g36510|
gi|7603281|ref|Os10g36520|
gi|7603283|ref|Os10g36540|
gi|7603288|ref|Os10g36580|
gi|7603289|ref|Os10g36590|
gi|7603290|ref|Os10g36600|
gi|7603291|ref|Os10g36610|
gi|7603292|ref|Os10g36620|
gi|7603293|ref|Os10g36630|
gi|7603294|ref|Os10g36640|
gi|7603304|ref|Os10g36730|
gi|7603308|ref|Os10g36770|
gi|7603311|ref|Os10g36790|
gi|7603314|ref|Os10g36820|
gi|7603322|ref|Os10g36890|
gi|7603323|ref|Os10g36900|
gi|7603325|ref|Os10g36920|
gi|7603326|ref|Os10g36930|
gi|7603333|ref|Os10g37000|
gi|7603334|ref|Os10g37010|
gi|7603336|ref|Os10g37030|
gi|7603342|ref|Os10g37090|
gi|7603346|ref|Os10g37130|
gi|7603347|ref|Os10g37140|
gi|7603348|ref|Os10g37150|
gi|7603353|ref|Os10g37200|
gi|7603356|ref|Os10g37220|
gi|7603360|ref|Os10g37250|
gi|7603362|ref|Os10g37270|
gi|7603364|ref|Os10g37290|
gi|7603365|ref|Os10g37300|
gi|7603367|ref|Os10g37320|
gi|7603370|ref|Os10g37350|
gi|7603371|ref|Os10g37360|
gi|7603373|ref|Os10g37380|
gi|7603380|ref|Os10g37440|
gi|7603381|ref|Os10g37450|
gi|7603387|ref|Os10g37510|
gi|7603391|ref|Os10g37540|
gi|7603392|ref|Os10g37550|
gi|7603395|ref|Os10g37580|
gi|7603396|ref|Os10g37590|
gi|7603406|ref|Os10g37680|
gi|7603408|ref|Os10g37700|
gi|7603413|ref|Os10g37750|
gi|7603416|ref|Os10g37780|
gi|7603417|ref|Os10g37790|
gi|7603428|ref|Os10g37860|
gi|7603429|ref|Os10g37870|
gi|7603439|ref|Os10g37970|
gi|7603441|ref|Os10g37990|
gi|7603443|ref|Os10g38010|
gi|7603444|ref|Os10g38020|
gi|7603450|ref|Os10g38070|
gi|7603456|ref|Os10g38130|
gi|7603461|ref|Os10g38170|
gi|7603463|ref|Os10g38190|
gi|7603482|ref|Os10g38380|
gi|7603483|ref|Os10g38390|
gi|7603485|ref|Os10g38410|
gi|7603488|ref|Os10g38440|
gi|7603490|ref|Os10g38460|
gi|7603495|ref|Os10g38510|
gi|7603496|ref|Os10g38520|
gi|7603497|ref|Os10g38530|
gi|7603506|ref|Os10g38620|
gi|7603509|ref|Os10g38650|
gi|7603512|ref|Os10g38680|
gi|7603520|ref|Os10g38760|
gi|7603523|ref|Os10g38790|
gi|7603525|ref|Os10g38810|
gi|7603528|ref|Os10g38840|
gi|7603545|ref|Os10g38990|
gi|7603546|ref|Os10g39000|
gi|7603551|ref|Os10g39040|
gi|7603552|ref|Os10g39050|
gi|7603553|ref|Os10g39060|
gi|7603555|ref|Os10g39080|
gi|7603556|ref|Os10g39090|
gi|7603557|ref|Os10g39090|
gi|7603569|ref|Os10g39200|
gi|7603570|ref|Os10g39200|
gi|7603574|ref|Os10g39240|
gi|7603575|ref|Os10g39250|
gi|7603578|ref|Os10g39280|
gi|7603579|ref|Os10g39290|
gi|7603587|ref|Os10g39370|
gi|7603598|ref|Os10g39470|
gi|7603599|ref|Os10g39480|
gi|7603600|ref|Os10g39490|
gi|7603601|ref|Os10g39500|
gi|7603602|ref|Os10g39510|
gi|7603606|ref|Os10g39550|

gi|7603609|ref|Os10g39580|
gi|7603611|ref|Os10g39600|
gi|7603618|ref|Os10g39630|
gi|7603620|ref|Os10g39650|
gi|7603624|ref|Os10g39690|
gi|7603628|ref|Os10g39730|
gi|7603647|ref|Os10g39920|
gi|7603649|ref|Os10g39940|
gi|7603651|ref|Os10g39960|
gi|7603656|ref|Os10g40010|
gi|7603667|ref|Os10g40080|
gi|7603681|ref|Os10g40210|
gi|7603682|ref|Os10g40220|
gi|7603683|ref|Os10g40230|
gi|7603688|ref|Os10g40280|
gi|7603690|ref|Os10g40300|
gi|7603691|ref|Os10g40310|
gi|7603692|ref|Os10g40320|
gi|7603693|ref|Os10g40330|
gi|7603695|ref|Os10g40350|
gi|7603697|ref|Os10g40370|
gi|7603698|ref|Os10g40380|
gi|7603700|ref|Os10g40400|
gi|7603702|ref|Os10g40420|
gi|7603705|ref|Os10g40450|
gi|7603723|ref|Os10g40620|
gi|7603724|ref|Os10g40630|
gi|7603728|ref|Os10g40670|
gi|7603730|ref|Os10g40690|
gi|7603737|ref|Os10g40750|
gi|7603738|ref|Os10g40760|
gi|7603739|ref|Os10g40770|
gi|7603742|ref|Os10g40790|
gi|7603745|ref|Os10g40820|
gi|7603754|ref|Os10g40910|
gi|7603764|ref|Os10g40970|
gi|7603765|ref|Os10g40980|
gi|7603768|ref|Os10g41010|
gi|7603776|ref|Os10g41090|
gi|7603781|ref|Os10g41140|
gi|7603788|ref|Os10g41210|
gi|7603793|ref|Os10g41250|
gi|7603797|ref|Os10g41280|
gi|7603801|ref|Os10g41320|
gi|7603812|ref|Os10g41420|
gi|7603830|ref|Os10g41580|
gi|7603833|ref|Os10g41600|
gi|7603836|ref|Os10g41630|
gi|7603840|ref|Os10g41670|
gi|7603856|ref|Os10g41800|
gi|7603857|ref|Os10g41810|
gi|7603862|ref|Os10g41850|
gi|7603866|ref|Os10g41890|
gi|7603868|ref|Os10g41910|
gi|7603869|ref|Os10g41920|
gi|7603875|ref|Os10g41980|
gi|7603876|ref|Os10g41990|
gi|7603877|ref|Os10g42000|
gi|7603878|ref|Os10g42010|
gi|7603880|ref|Os10g42030|
gi|7603881|ref|Os10g42040|
gi|7603884|ref|Os10g42070|
gi|7603885|ref|Os10g42080|
gi|7603889|ref|Os10g42120|
gi|7603891|ref|Os10g42140|
gi|7603892|ref|Os10g42150|
gi|7603906|ref|Os10g42260|
gi|7603914|ref|Os10g42340|
gi|7603915|ref|Os10g42350|
gi|7603918|ref|Os10g42380|
gi|7603934|ref|Os10g42520|
gi|7603938|ref|Os10g42560|
gi|7603952|ref|Os10g42680|
gi|7603975|ref|Os10g42810|
gi|7603985|ref|Os10g42890|
gi|7603988|ref|Os10g42920|
gi|7603989|ref|Os10g42930|
gi|7604000|ref|Os10g42980|
gi|7604003|ref|Os10g43010|
gi|8000004|ref|Os06g01040|
gi|8000005|ref|Os06g01050|
gi|8000006|ref|Os06g01060|
gi|8000007|ref|Os06g01070|
gi|8000008|ref|Os06g01080|
gi|8000011|ref|Os06g01110|
gi|8000014|ref|Os06g01140|
gi|8000015|ref|Os06g01150|
gi|8000016|ref|Os06g01160|
gi|8000019|ref|Os06g01190|
gi|8000022|ref|Os06g01220|
gi|8000024|ref|Os06g01240|
gi|8000028|ref|Os06g01270|
gi|8000029|ref|Os06g01280|
gi|8000032|ref|Os06g01310|
gi|8000034|ref|Os06g01330|
gi|8000039|ref|Os06g01380|
gi|8000054|ref|Os06g01510|
gi|8000055|ref|Os06g01520|
gi|8000058|ref|Os06g01550|
gi|8000063|ref|Os06g01600|
gi|8000076|ref|Os06g01710|
gi|8000078|ref|Os06g01730|
gi|8000079|ref|Os06g01740|
gi|8000080|ref|Os06g01750|
gi|8000088|ref|Os06g01810|
gi|8000089|ref|Os06g01820|
gi|8000090|ref|Os06g01830|
gi|8000094|ref|Os06g01870|
gi|8000095|ref|Os06g01880|
gi|8000097|ref|Os06g01900|
gi|8000103|ref|Os06g01960|
gi|8000108|ref|Os06g02010|
gi|8000111|ref|Os06g02040|
gi|8000113|ref|Os06g02060|
gi|8000115|ref|Os06g02080|
gi|8000121|ref|Os06g02140|
gi|8000126|ref|Os06g02190|
gi|8000134|ref|Os06g02270|
gi|8000135|ref|Os06g02280|
gi|8000136|ref|Os06g02290|
gi|8000138|ref|Os06g02310|
gi|8000139|ref|Os06g02320|
gi|8000140|ref|Os06g02330|
gi|8000144|ref|Os06g02370|
gi|8000145|ref|Os06g02370|
gi|8000150|ref|Os06g02410|
gi|8000154|ref|Os06g02450|
gi|8000155|ref|Os06g02460|
gi|8000161|ref|Os06g02520|
gi|8000174|ref|Os06g02630|
gi|8000175|ref|Os06g02640|
gi|8000177|ref|Os06g02660|
gi|8000178|ref|Os06g02670|
gi|8000180|ref|Os06g02690|

gi|8000182|ref|Os06g02710|
gi|8000187|ref|Os06g02760|
gi|8000188|ref|Os06g02770|
gi|8000190|ref|Os06g02790|
gi|8000191|ref|Os06g02800|
gi|8000192|ref|Os06g02810|
gi|8000195|ref|Os06g02840|
gi|8000197|ref|Os06g02860|
gi|8000198|ref|Os06g02870|
gi|8000201|ref|Os06g02890|
gi|8000202|ref|Os06g02910|
gi|8000203|ref|Os06g02920|
gi|8000204|ref|Os06g02930|
gi|8000205|ref|Os06g02940|
gi|8000207|ref|Os06g02960|
gi|8000208|ref|Os06g02970|
gi|8000210|ref|Os06g02990|
gi|8000212|ref|Os06g03010|
gi|8000213|ref|Os06g03020|
gi|8000214|ref|Os06g03030|
gi|8000215|ref|Os06g03040|
gi|8000217|ref|Os06g03060|
gi|8000218|ref|Os06g03070|
gi|8000221|ref|Os06g03100|
gi|8000222|ref|Os06g03110|
gi|8000224|ref|Os06g03130|
gi|8000227|ref|Os06g03160|
gi|8000231|ref|Os06g03200|
gi|8000232|ref|Os06g03210|
gi|8000233|ref|Os06g03220|
gi|8000234|ref|Os06g03230|
gi|8000235|ref|Os06g03240|
gi|8000238|ref|Os06g03270|
gi|8000240|ref|Os06g03290|
gi|8000244|ref|Os06g03330|
gi|8000246|ref|Os06g03350|
gi|8000248|ref|Os06g03370|
gi|8000250|ref|Os06g03390|
gi|8000251|ref|Os06g03400|
gi|8000254|ref|Os06g03430|
gi|8000255|ref|Os06g03440|
gi|8000259|ref|Os06g03480|
gi|8000260|ref|Os06g03490|
gi|8000262|ref|Os06g03510|
gi|8000266|ref|Os06g03550|
gi|8000270|ref|Os06g03590|
gi|8000274|ref|Os06g03620|
gi|8000275|ref|Os06g03630|
gi|8000277|ref|Os06g03650|
gi|8000285|ref|Os06g03730|
gi|8000294|ref|Os06g03810|
gi|8000300|ref|Os06g03870|
gi|8000304|ref|Os06g03880|
gi|8000311|ref|Os06g03950|
gi|8000312|ref|Os06g03960|
gi|8000314|ref|Os06g03980|
gi|8000320|ref|Os06g04050|
gi|8000325|ref|Os06g04060|
gi|8000330|ref|Os06g04100|
gi|8000332|ref|Os06g04120|
gi|8000334|ref|Os06g04140|
gi|8000337|ref|Os06g04170|
gi|8000341|ref|Os06g04210|
gi|8000342|ref|Os06g04220|
gi|8000343|ref|Os06g04230|
gi|8000344|ref|Os06g04240|
gi|8000346|ref|Os06g04260|
gi|8000352|ref|Os06g04320|
gi|8000353|ref|Os06g04330|
gi|8000354|ref|Os06g04340|
gi|8000355|ref|Os06g04350|
gi|8000356|ref|Os06g04360|
gi|8000360|ref|Os06g04400|
gi|8000361|ref|Os06g04410|
gi|8000363|ref|Os06g04430|
gi|8000364|ref|Os06g04440|
gi|8000367|ref|Os06g04470|
gi|8000368|ref|Os06g04470|
gi|8000370|ref|Os06g04490|
gi|8000376|ref|Os06g04550|
gi|8000378|ref|Os06g04570|
gi|8000384|ref|Os06g04630|
gi|8000392|ref|Os06g04700|
gi|8000394|ref|Os06g04720|
gi|8000395|ref|Os06g04730|
gi|8000396|ref|Os06g04740|
gi|8000397|ref|Os06g04750|
gi|8000398|ref|Os06g04760|
gi|8000408|ref|Os06g04860|
gi|8000414|ref|Os06g04890|
gi|8000419|ref|Os06g04930|
gi|8000443|ref|Os06g05120|
gi|8000454|ref|Os06g05220|
gi|8000465|ref|Os06g05330|
gi|8000468|ref|Os06g05360|
gi|8000473|ref|Os06g05410|
gi|8000474|ref|Os06g05420|
gi|8000475|ref|Os06g05430|
gi|8000476|ref|Os06g05440|
gi|8000477|ref|Os06g05450|
gi|8000479|ref|Os06g05470|
gi|8000480|ref|Os06g05480|
gi|8000481|ref|Os06g05490|
gi|8000483|ref|Os06g05510|
gi|8000485|ref|Os06g05530|
gi|8000486|ref|Os06g05540|
gi|8000488|ref|Os06g05560|
gi|8000489|ref|Os06g05570|
gi|8000498|ref|Os06g05650|
gi|8000506|ref|Os06g05710|
gi|8000508|ref|Os06g05730|
gi|8000512|ref|Os06g05770|
gi|8000516|ref|Os06g05810|
gi|8000529|ref|Os06g05930|
gi|8000533|ref|Os06g05960|
gi|8000539|ref|Os06g06010|
gi|8000553|ref|Os06g06140|
gi|8000560|ref|Os06g06170|
gi|8000564|ref|Os06g06210|
gi|8000565|ref|Os06g06220|
gi|8000567|ref|Os06g06240|
gi|8000576|ref|Os06g06330|
gi|8000577|ref|Os06g06340|
gi|8000590|ref|Os06g06470|
gi|8000601|ref|Os06g06570|
gi|8000603|ref|Os06g06590|
gi|8000606|ref|Os06g06610|
gi|8000607|ref|Os06g06620|
gi|8000608|ref|Os06g06630|
gi|8000610|ref|Os06g06650|
gi|8000614|ref|Os06g06690|
gi|8000615|ref|Os06g06700|
gi|8000635|ref|Os06g06890|
gi|8000637|ref|Os06g06910|

gi|8000641|ref|Os06g06950|
gi|8000643|ref|Os06g06970|
gi|8000645|ref|Os06g06990|
gi|8000652|ref|Os06g07050|
gi|8000653|ref|Os06g07060|
gi|8000662|ref|Os06g07150|
gi|8000664|ref|Os06g07170|
gi|8000665|ref|Os06g07180|
gi|8000674|ref|Os06g07270|
gi|8000681|ref|Os06g07340|
gi|8000683|ref|Os06g07360|
gi|8000688|ref|Os06g07410|
gi|8000692|ref|Os06g07450|
gi|8000694|ref|Os06g07470|
gi|8000708|ref|Os06g07610|
gi|8000709|ref|Os06g07620|
gi|8000714|ref|Os06g07670|
gi|8000716|ref|Os06g07690|
gi|8000720|ref|Os06g07730|
gi|8000722|ref|Os06g07750|
gi|8000728|ref|Os06g07800|
gi|8000732|ref|Os06g07840|
gi|8000765|ref|Os06g08130|
gi|8000768|ref|Os06g08160|
gi|8000771|ref|Os06g08190|
gi|8000772|ref|Os06g08200|
gi|8000773|ref|Os06g08210|
gi|8000774|ref|Os06g08220|
gi|8000775|ref|Os06g08230|
gi|8000776|ref|Os06g08240|
gi|8000777|ref|Os06g08250|
gi|8000778|ref|Os06g08260|
gi|8000779|ref|Os06g08270|
gi|8000790|ref|Os06g08370|
gi|8000797|ref|Os06g08430|
gi|8000805|ref|Os06g08510|
gi|8000814|ref|Os06g08590|
gi|8000836|ref|Os06g08780|
gi|8000845|ref|Os06g08870|
gi|8000850|ref|Os06g08920|
gi|8000856|ref|Os06g08980|
gi|8000857|ref|Os06g08990|
gi|8000858|ref|Os06g09000|
gi|8000859|ref|Os06g09010|
gi|8000860|ref|Os06g09020|
gi|8000861|ref|Os06g09020|
gi|8000862|ref|Os06g09030|
gi|8000863|ref|Os06g09040|
gi|8000867|ref|Os06g09080|
gi|8000869|ref|Os06g09100|
gi|8000870|ref|Os06g09110|
gi|8000875|ref|Os06g09160|
gi|8000879|ref|Os06g09200|
gi|8000885|ref|Os06g09260|
gi|8000889|ref|Os06g09300|
gi|8000898|ref|Os06g09360|
gi|8000899|ref|Os06g09380|
gi|8000901|ref|Os06g09400|
gi|8000902|ref|Os06g09410|
gi|8000904|ref|Os06g09430|
gi|8000912|ref|Os06g09500|
gi|8000913|ref|Os06g09510|
gi|8000914|ref|Os06g09520|
gi|8000917|ref|Os06g09550|
gi|8000921|ref|Os06g09590|
gi|8000922|ref|Os06g09600|
gi|8000925|ref|Os06g09620|
gi|8000931|ref|Os06g09680|
gi|8000935|ref|Os06g09720|
gi|8000940|ref|Os06g09770|
gi|8000943|ref|Os06g09800|
gi|8000950|ref|Os06g09870|
gi|8000958|ref|Os06g09920|
gi|8000960|ref|Os06g09940|
gi|8000961|ref|Os06g09950|
gi|8000962|ref|Os06g09960|
gi|8000963|ref|Os06g09970|
gi|8000964|ref|Os06g09980|
gi|8000965|ref|Os06g09990|
gi|8000966|ref|Os06g10000|
gi|8000967|ref|Os06g10010|
gi|8000968|ref|Os06g10020|
gi|8000969|ref|Os06g10030|
gi|8000970|ref|Os06g10040|
gi|8000971|ref|Os06g10050|
gi|8000972|ref|Os06g10060|
gi|8000973|ref|Os06g10070|
gi|8000975|ref|Os06g10090|
gi|8000976|ref|Os06g10100|
gi|8000977|ref|Os06g10110|
gi|8000978|ref|Os06g10120|
gi|8000979|ref|Os06g10130|
gi|8000981|ref|Os06g10150|
gi|8000989|ref|Os06g10220|
gi|8000992|ref|Os06g10240|
gi|8000993|ref|Os06g10250|
gi|8001000|ref|Os06g10320|
gi|8001008|ref|Os06g10400|
gi|8001013|ref|Os06g10450|
gi|8001014|ref|Os06g10460|
gi|8001017|ref|Os06g10490|
gi|8001019|ref|Os06g10510|
gi|8001021|ref|Os06g10530|
gi|8001022|ref|Os06g10540|
gi|8001027|ref|Os06g10590|
gi|8001029|ref|Os06g10610|
gi|8001032|ref|Os06g10640|
gi|8001036|ref|Os06g10680|
gi|8001040|ref|Os06g10700|
gi|8001041|ref|Os06g10710|
gi|8001042|ref|Os06g10720|
gi|8001043|ref|Os06g10730|
gi|8001054|ref|Os06g10840|
gi|8001057|ref|Os06g10870|
gi|8001070|ref|Os06g11000|
gi|8001076|ref|Os06g11060|
gi|8001082|ref|Os06g11120|
gi|8001086|ref|Os06g11160|
gi|8001093|ref|Os06g11230|
gi|8001106|ref|Os06g11360|
gi|8001109|ref|Os06g11390|
gi|8001117|ref|Os06g11470|
gi|8001118|ref|Os06g11480|
gi|8001121|ref|Os06g11510|
gi|8001123|ref|Os06g11530|
gi|8001128|ref|Os06g11580|
gi|8001129|ref|Os06g11590|
gi|8001137|ref|Os06g11670|
gi|8001139|ref|Os06g11690|
gi|8001145|ref|Os06g11750|
gi|8001146|ref|Os06g11760|
gi|8001147|ref|Os06g11770|
gi|8001152|ref|Os06g11820|
gi|8001155|ref|Os06g11850|

gi|8001157|ref|Os06g11870|
gi|8001170|ref|Os06g12000|
gi|8001171|ref|Os06g12010|
gi|8001185|ref|Os06g12130|
gi|8001186|ref|Os06g12140|
gi|8001192|ref|Os06g12200|
gi|8001196|ref|Os06g12240|
gi|8001199|ref|Os06g12270|
gi|8001202|ref|Os06g12300|
gi|8001207|ref|Os06g12340|
gi|8001211|ref|Os06g12380|
gi|8001215|ref|Os06g12420|
gi|8001216|ref|Os06g12430|
gi|8001220|ref|Os06g12470|
gi|8001222|ref|Os06g12490|
gi|8001225|ref|Os06g12520|
gi|8001229|ref|Os06g12550|
gi|8001231|ref|Os06g12570|
gi|8001236|ref|Os06g12620|
gi|8001239|ref|Os06g12650|
gi|8001246|ref|Os06g12700|
gi|8001249|ref|Os06g12730|
gi|8001252|ref|Os06g12760|
gi|8001265|ref|Os06g12890|
gi|8001268|ref|Os06g12920|
gi|8001271|ref|Os06g12950|
gi|8001290|ref|Os06g13130|
gi|8001292|ref|Os06g13150|
gi|8001296|ref|Os06g13190|
gi|8001300|ref|Os06g13230|
gi|8001302|ref|Os06g13250|
gi|8001303|ref|Os06g13260|
gi|8001306|ref|Os06g13290|
gi|8001307|ref|Os06g13300|
gi|8001311|ref|Os06g13340|
gi|8001314|ref|Os06g13370|
gi|8001315|ref|Os06g13380|
gi|8001318|ref|Os06g13410|
gi|8001321|ref|Os06g13440|
gi|8001325|ref|Os06g13480|
gi|8001336|ref|Os06g13580|
gi|8001337|ref|Os06g13590|
gi|8001339|ref|Os06g13610|
gi|8001340|ref|Os06g13620|
gi|8001348|ref|Os06g13690|
gi|8001349|ref|Os06g13700|
gi|8001357|ref|Os06g13780|
gi|8001359|ref|Os06g13800|
gi|8001371|ref|Os06g13880|
gi|8001374|ref|Os06g13910|
gi|8001377|ref|Os06g13940|
gi|8001380|ref|Os06g13970|
gi|8001381|ref|Os06g13980|
gi|8001385|ref|Os06g14020|
gi|8001392|ref|Os06g14090|
gi|8001401|ref|Os06g14180|
gi|8001405|ref|Os06g14210|
gi|8001407|ref|Os06g14230|
gi|8001414|ref|Os06g14300|
gi|8001418|ref|Os06g14340|
gi|8001422|ref|Os06g14380|
gi|8001435|ref|Os06g14500|
gi|8001437|ref|Os06g14520|
gi|8001438|ref|Os06g14530|
gi|8001441|ref|Os06g14560|
gi|8001443|ref|Os06g14580|
gi|8001451|ref|Os06g14660|
gi|8001454|ref|Os06g14690|
gi|8001462|ref|Os06g14760|
gi|8001463|ref|Os06g14770|
gi|8001465|ref|Os06g14790|
gi|8001466|ref|Os06g14800|
gi|8001468|ref|Os06g14820|
gi|8001469|ref|Os06g14830|
gi|8001472|ref|Os06g14860|
gi|8001473|ref|Os06g14870|
gi|8001475|ref|Os06g14890|
gi|8001476|ref|Os06g14900|
gi|8001479|ref|Os06g14930|
gi|8001482|ref|Os06g14960|
gi|8001484|ref|Os06g14980|
gi|8001487|ref|Os06g15010|
gi|8001494|ref|Os06g15080|
gi|8001497|ref|Os06g15110|
gi|8001498|ref|Os06g15120|
gi|8001499|ref|Os06g15130|
gi|8001505|ref|Os06g15190|
gi|8001507|ref|Os06g15210|
gi|8001508|ref|Os06g15220|
gi|8001509|ref|Os06g15230|
gi|8001510|ref|Os06g15240|
gi|8001512|ref|Os06g15260|
gi|8001515|ref|Os06g15290|
gi|8001518|ref|Os06g15320|
gi|8001529|ref|Os06g15430|
gi|8001530|ref|Os06g15440|
gi|8001531|ref|Os06g15450|
gi|8001535|ref|Os06g15490|
gi|8001544|ref|Os06g15580|
gi|8001547|ref|Os06g15610|
gi|8001552|ref|Os06g15650|
gi|8001553|ref|Os06g15660|
gi|8001554|ref|Os06g15670|
gi|8001561|ref|Os06g15740|
gi|8001565|ref|Os06g15780|
gi|8001567|ref|Os06g15800|
gi|8001570|ref|Os06g15830|
gi|8001573|ref|Os06g15860|
gi|8001575|ref|Os06g15880|
gi|8001576|ref|Os06g15890|
gi|8001577|ref|Os06g15900|
gi|8001581|ref|Os06g15940|
gi|8001583|ref|Os06g15960|
gi|8001585|ref|Os06g15980|
gi|8001589|ref|Os06g16020|
gi|8001592|ref|Os06g16040|
gi|8001599|ref|Os06g16110|
gi|8001600|ref|Os06g16120|
gi|8001602|ref|Os06g16140|
gi|8001606|ref|Os06g16180|
gi|8001607|ref|Os06g16190|
gi|8001609|ref|Os06g16210|
gi|8001622|ref|Os06g16340|
gi|8001624|ref|Os06g16360|
gi|8001626|ref|Os06g16380|
gi|8001633|ref|Os06g16440|
gi|8001635|ref|Os06g16460|
gi|8001637|ref|Os06g16480|
gi|8001639|ref|Os06g16500|
gi|8001642|ref|Os06g16530|
gi|8001644|ref|Os06g16550|
gi|8001645|ref|Os06g16560|
gi|8001648|ref|Os06g16590|
gi|8001650|ref|Os06g16610|

gi|8001651|ref|Os06g16620|
gi|8001652|ref|Os06g16630|
gi|8001654|ref|Os06g16650|
gi|8001662|ref|Os06g16730|
gi|8001663|ref|Os06g16740|
gi|8001664|ref|Os06g16750|
gi|8001666|ref|Os06g16770|
gi|8001667|ref|Os06g16780|
gi|8001669|ref|Os06g16800|
gi|8001671|ref|Os06g16820|
gi|8001681|ref|Os06g16920|
gi|8001682|ref|Os06g16930|
gi|8001686|ref|Os06g16970|
gi|8001691|ref|Os06g17010|
gi|8001695|ref|Os06g17050|
gi|8001698|ref|Os06g17080|
gi|8001703|ref|Os06g17130|
gi|8001708|ref|Os06g17170|
gi|8001709|ref|Os06g17180|
gi|8001718|ref|Os06g17270|
gi|8001721|ref|Os06g17300|
gi|8001727|ref|Os06g17360|
gi|8001728|ref|Os06g17370|
gi|8001729|ref|Os06g17380|
gi|8001731|ref|Os06g17400|
gi|8001734|ref|Os06g17430|
gi|8001736|ref|Os06g17450|
gi|8001738|ref|Os06g17470|
gi|8001741|ref|Os06g17500|
gi|8001742|ref|Os06g17510|
gi|8001744|ref|Os06g17530|
gi|8001746|ref|Os06g17550|
gi|8001747|ref|Os06g17560|
gi|8001749|ref|Os06g17580|
gi|8001750|ref|Os06g17590|
gi|8001754|ref|Os06g17630|
gi|8001756|ref|Os06g17650|
gi|8001757|ref|Os06g17660|
gi|8001760|ref|Os06g17690|
gi|8001763|ref|Os06g17720|
gi|8001765|ref|Os06g17740|
gi|8001767|ref|Os06g17760|
gi|8001768|ref|Os06g17770|
gi|8001777|ref|Os06g17850|
gi|8001778|ref|Os06g17860|
gi|8001781|ref|Os06g17890|
gi|8001786|ref|Os06g17940|
gi|8001788|ref|Os06g17960|
gi|8001790|ref|Os06g17980|
gi|8001791|ref|Os06g17990|
gi|8001794|ref|Os06g18020|
gi|8001796|ref|Os06g18040|
gi|8001800|ref|Os06g18080|
gi|8001804|ref|Os06g18120|
gi|8001807|ref|Os06g18150|
gi|8001812|ref|Os06g18680|
gi|8001813|ref|Os06g18690|
gi|8001814|ref|Os06g18700|
gi|8001816|ref|Os06g18720|
gi|8001819|ref|Os06g18750|
gi|8001821|ref|Os06g18770|
gi|8001825|ref|Os06g18810|
gi|8001829|ref|Os06g18850|
gi|8001831|ref|Os06g18870|
gi|8001832|ref|Os06g18880|
gi|8001833|ref|Os06g18890|
gi|8001835|ref|Os06g18910|
gi|8001836|ref|Os06g18920|
gi|8001837|ref|Os06g18930|
gi|8001840|ref|Os06g18960|
gi|8001841|ref|Os06g18970|
gi|8001842|ref|Os06g18980|
gi|8001844|ref|Os06g19000|
gi|8001845|ref|Os06g19010|
gi|8001846|ref|Os06g19020|
gi|8001847|ref|Os06g19030|
gi|8001849|ref|Os06g19050|
gi|8001850|ref|Os06g19060|
gi|8001855|ref|Os06g19110|
gi|8001856|ref|Os06g19120|
gi|8001862|ref|Os06g19180|
gi|8001863|ref|Os06g19190|
gi|8001864|ref|Os06g19200|
gi|8001865|ref|Os06g19210|
gi|8001866|ref|Os06g19220|
gi|8001870|ref|Os06g19260|
gi|8001871|ref|Os06g19270|
gi|8001873|ref|Os06g19290|
gi|8001874|ref|Os06g19300|
gi|8001876|ref|Os06g19320|
gi|8001878|ref|Os06g19340|
gi|8001880|ref|Os06g19360|
gi|8001882|ref|Os06g19370|
gi|8001887|ref|Os06g19420|
gi|8001888|ref|Os06g19430|
gi|8001891|ref|Os06g19460|
gi|8001894|ref|Os06g19490|
gi|8001902|ref|Os06g19570|
gi|8001903|ref|Os06g19580|
gi|8001905|ref|Os06g19600|
gi|8001916|ref|Os06g19700|
gi|8001918|ref|Os06g19720|
gi|8001920|ref|Os06g19740|
gi|8001921|ref|Os06g19750|
gi|8001922|ref|Os06g19760|
gi|8001923|ref|Os06g19770|
gi|8001924|ref|Os06g19780|
gi|8001925|ref|Os06g19790|
gi|8001927|ref|Os06g19810|
gi|8001932|ref|Os06g19860|
gi|8001934|ref|Os06g19880|
gi|8001937|ref|Os06g19910|
gi|8001940|ref|Os06g19940|
gi|8001943|ref|Os06g19970|
gi|8001946|ref|Os06g20000|
gi|8001952|ref|Os06g20060|
gi|8001953|ref|Os06g20070|
gi|8001954|ref|Os06g20080|
gi|8001956|ref|Os06g20100|
gi|8001964|ref|Os06g20180|
gi|8001970|ref|Os06g20240|
gi|8001972|ref|Os06g20260|
gi|8001973|ref|Os06g20270|
gi|8001975|ref|Os06g20290|
gi|8001976|ref|Os06g20300|
gi|8001977|ref|Os06g20310|
gi|8001978|ref|Os06g20310|
gi|8001980|ref|Os06g20330|
gi|8001983|ref|Os06g20350|
gi|8001986|ref|Os06g20380|
gi|8001992|ref|Os06g20420|
gi|8001995|ref|Os06g20450|
gi|8001997|ref|Os06g20470|
gi|8001998|ref|Os06g20480|

gi|8002003|ref|Os06g20530|
gi|8002004|ref|Os06g20540|
gi|8002005|ref|Os06g20550|
gi|8002006|ref|Os06g20560|
gi|8002010|ref|Os06g20600|
gi|8002017|ref|Os06g20670|
gi|8002019|ref|Os06g20690|
gi|8002024|ref|Os06g20740|
gi|8002026|ref|Os06g20760|
gi|8002032|ref|Os06g20820|
gi|8002033|ref|Os06g20830|
gi|8002035|ref|Os06g20850|
gi|8002039|ref|Os06g20890|
gi|8002040|ref|Os06g20900|
gi|8002041|ref|Os06g20910|
gi|8002043|ref|Os06g20930|
gi|8002051|ref|Os06g21010|
gi|8002057|ref|Os06g21070|
gi|8002059|ref|Os06g21090|
gi|8002060|ref|Os06g21100|
gi|8002061|ref|Os06g21110|
gi|8002062|ref|Os06g21120|
gi|8002063|ref|Os06g21130|
gi|8002064|ref|Os06g21140|
gi|8002071|ref|Os06g21210|
gi|8002072|ref|Os06g21220|
gi|8002073|ref|Os06g21230|
gi|8002074|ref|Os06g21240|
gi|8002075|ref|Os06g21240|
gi|8002076|ref|Os06g21250|
gi|8002078|ref|Os06g21270|
gi|8002080|ref|Os06g21290|
gi|8002082|ref|Os06g21310|
gi|8002083|ref|Os06g21320|
gi|8002087|ref|Os06g21350|
gi|8002092|ref|Os06g21400|
gi|8002093|ref|Os06g21410|
gi|8002098|ref|Os06g21460|
gi|8002117|ref|Os06g21630|
gi|8002119|ref|Os06g21650|
gi|8002123|ref|Os06g21690|
gi|8002125|ref|Os06g21710|
gi|8002131|ref|Os06g21770|
gi|8002133|ref|Os06g21790|
gi|8002134|ref|Os06g21800|
gi|8002135|ref|Os06g21810|
gi|8002139|ref|Os06g21840|
gi|8002140|ref|Os06g21850|
gi|8002142|ref|Os06g21870|
gi|8002143|ref|Os06g21880|
gi|8002144|ref|Os06g21890|
gi|8002146|ref|Os06g21910|
gi|8002149|ref|Os06g21940|
gi|8002151|ref|Os06g21960|
gi|8002152|ref|Os06g21970|
gi|8002156|ref|Os06g22000|
gi|8002160|ref|Os06g22040|
gi|8002161|ref|Os06g22050|
gi|8002172|ref|Os06g22120|
gi|8002173|ref|Os06g22130|
gi|8002175|ref|Os06g22150|
gi|8002176|ref|Os06g22160|
gi|8002177|ref|Os06g22170|
gi|8002178|ref|Os06g22180|
gi|8002179|ref|Os06g22190|
gi|8002180|ref|Os06g22200|
gi|8002181|ref|Os06g22210|
gi|8002186|ref|Os06g22260|
gi|8002187|ref|Os06g22270|
gi|8002188|ref|Os06g22280|
gi|8002191|ref|Os06g22310|
gi|8002196|ref|Os06g22360|
gi|8002197|ref|Os06g22370|
gi|8002199|ref|Os06g22390|
gi|8002200|ref|Os06g22400|
gi|8002201|ref|Os06g22410|
gi|8002202|ref|Os06g22420|
gi|8002205|ref|Os06g22450|
gi|8002207|ref|Os06g22470|
gi|8002213|ref|Os06g22530|
gi|8002214|ref|Os06g22540|
gi|8002221|ref|Os06g22610|
gi|8002222|ref|Os06g22620|
gi|8002223|ref|Os06g22630|
gi|8002224|ref|Os06g22640|
gi|8002226|ref|Os06g22660|
gi|8002228|ref|Os06g22680|
gi|8002238|ref|Os06g22780|
gi|8002240|ref|Os06g22800|
gi|8002243|ref|Os06g22830|
gi|8002244|ref|Os06g22840|
gi|8002246|ref|Os06g22860|
gi|8002248|ref|Os06g22880|
gi|8002251|ref|Os06g22910|
gi|8002254|ref|Os06g22940|
gi|8002255|ref|Os06g22950|
gi|8002257|ref|Os06g22970|
gi|8002259|ref|Os06g22990|
gi|8002260|ref|Os06g23000|
gi|8002262|ref|Os06g23020|
gi|8002264|ref|Os06g23040|
gi|8002266|ref|Os06g23060|
gi|8002269|ref|Os06g23090|
gi|8002270|ref|Os06g23100|
gi|8002273|ref|Os06g23130|
gi|8002275|ref|Os06g23150|
gi|8002278|ref|Os06g23180|
gi|8002285|ref|Os06g23250|
gi|8002287|ref|Os06g23270|
gi|8002294|ref|Os06g23300|
gi|8002295|ref|Os06g23310|
gi|8002297|ref|Os06g23330|
gi|8002300|ref|Os06g23360|
gi|8002301|ref|Os06g23370|
gi|8002302|ref|Os06g23380|
gi|8002303|ref|Os06g23390|
gi|8002304|ref|Os06g23400|
gi|8002305|ref|Os06g23410|
gi|8002309|ref|Os06g23450|
gi|8002311|ref|Os06g23470|
gi|8002312|ref|Os06g23480|
gi|8002315|ref|Os06g23510|
gi|8002316|ref|Os06g23520|
gi|8002321|ref|Os06g23570|
gi|8002322|ref|Os06g23580|
gi|8002323|ref|Os06g23590|
gi|8002324|ref|Os06g23600|
gi|8002326|ref|Os06g23620|
gi|8002328|ref|Os06g23640|
gi|8002330|ref|Os06g23660|
gi|8002333|ref|Os06g23690|
gi|8002336|ref|Os06g23720|
gi|8002338|ref|Os06g23740|
gi|8002339|ref|Os06g23750|

gi|8002341|ref|Os06g23770|
gi|8002343|ref|Os06g23790|
gi|8002345|ref|Os06g23810|
gi|8002349|ref|Os06g23850|
gi|8002352|ref|Os06g23880|
gi|8002354|ref|Os06g23900|
gi|8002355|ref|Os06g23910|
gi|8002357|ref|Os06g23930|
gi|8002360|ref|Os06g23960|
gi|8002361|ref|Os06g23970|
gi|8002364|ref|Os06g24000|
gi|8002365|ref|Os06g24010|
gi|8002366|ref|Os06g24020|
gi|8002368|ref|Os06g24040|
gi|8002374|ref|Os06g24090|
gi|8002377|ref|Os06g24120|
gi|8002378|ref|Os06g24130|
gi|8002380|ref|Os06g24150|
gi|8002384|ref|Os06g24190|
gi|8002386|ref|Os06g24210|
gi|8002387|ref|Os06g24220|
gi|8002389|ref|Os06g24240|
gi|8002390|ref|Os06g24250|
gi|8002391|ref|Os06g24260|
gi|8002393|ref|Os06g24280|
gi|8002395|ref|Os06g24300|
gi|8002402|ref|Os06g24370|
gi|8002404|ref|Os06g24390|
gi|8002408|ref|Os06g24430|
gi|8002412|ref|Os06g24470|
gi|8002413|ref|Os06g24480|
gi|8002415|ref|Os06g24500|
gi|8002416|ref|Os06g24510|
gi|8002417|ref|Os06g24520|
gi|8002420|ref|Os06g24550|
gi|8002421|ref|Os06g24560|
gi|8002423|ref|Os06g24580|
gi|8002425|ref|Os06g24600|
gi|8002426|ref|Os06g24610|
gi|8002427|ref|Os06g24620|
gi|8002429|ref|Os06g24640|
gi|8002431|ref|Os06g24660|
gi|8002434|ref|Os06g24690|
gi|8002441|ref|Os06g24740|
gi|8002442|ref|Os06g24750|
gi|8002444|ref|Os06g24770|
gi|8002450|ref|Os06g24830|
gi|8002453|ref|Os06g24860|
gi|8002456|ref|Os06g24880|
gi|8002457|ref|Os06g24890|
gi|8002458|ref|Os06g24900|
gi|8002459|ref|Os06g24910|
gi|8002461|ref|Os06g24930|
gi|8002465|ref|Os06g24970|
gi|8002470|ref|Os06g25020|
gi|8002472|ref|Os06g25040|
gi|8002475|ref|Os06g25070|
gi|8002477|ref|Os06g25090|
gi|8002483|ref|Os06g25150|
gi|8002487|ref|Os06g25190|
gi|8002491|ref|Os06g25230|
gi|8002492|ref|Os06g25240|
gi|8002494|ref|Os06g25260|
gi|8002501|ref|Os06g25330|
gi|8002502|ref|Os06g25340|
gi|8002506|ref|Os06g25380|
gi|8002507|ref|Os06g25390|
gi|8002509|ref|Os06g25410|
gi|8002510|ref|Os06g25420|
gi|8002514|ref|Os06g25460|
gi|8002515|ref|Os06g25470|
gi|8002519|ref|Os06g25510|
gi|8002520|ref|Os06g25520|
gi|8002521|ref|Os06g25530|
gi|8002522|ref|Os06g25540|
gi|8002525|ref|Os06g25570|
gi|8002527|ref|Os06g25590|
gi|8002528|ref|Os06g25600|
gi|8002530|ref|Os06g25620|
gi|8002532|ref|Os06g25640|
gi|8002537|ref|Os06g25690|
gi|8002542|ref|Os06g25740|
gi|8002543|ref|Os06g25750|
gi|8002547|ref|Os06g25790|
gi|8002555|ref|Os06g25870|
gi|8002562|ref|Os06g25940|
gi|8002564|ref|Os06g25960|
gi|8002566|ref|Os06g25980|
gi|8002570|ref|Os06g26020|
gi|8002573|ref|Os06g26050|
gi|8002576|ref|Os06g26080|
gi|8002579|ref|Os06g26110|
gi|8002580|ref|Os06g26120|
gi|8002584|ref|Os06g26160|
gi|8002585|ref|Os06g26170|
gi|8002586|ref|Os06g26180|
gi|8002587|ref|Os06g26190|
gi|8002589|ref|Os06g26210|
gi|8002591|ref|Os06g26230|
gi|8002593|ref|Os06g26250|
gi|8002596|ref|Os06g26280|
gi|8002597|ref|Os06g26290|
gi|8002598|ref|Os06g26300|
gi|8002601|ref|Os06g26330|
gi|8002606|ref|Os06g27360|
gi|8002607|ref|Os06g27370|
gi|8002608|ref|Os06g27380|
gi|8002610|ref|Os06g27400|
gi|8002617|ref|Os06g27470|
gi|8002621|ref|Os06g27510|
gi|8002622|ref|Os06g27520|
gi|8002623|ref|Os06g27530|
gi|8002625|ref|Os06g27550|
gi|8002627|ref|Os06g27570|
gi|8002629|ref|Os06g27590|
gi|8002631|ref|Os06g27610|
gi|8002632|ref|Os06g27620|
gi|8002634|ref|Os06g27640|
gi|8002636|ref|Os06g27660|
gi|8002637|ref|Os06g27670|
gi|8002641|ref|Os06g27710|
gi|8002645|ref|Os06g27750|
gi|8002649|ref|Os06g27780|
gi|8002652|ref|Os06g27810|
gi|8002653|ref|Os06g27820|
gi|8002657|ref|Os06g27860|
gi|8002662|ref|Os06g27910|
gi|8002667|ref|Os06g27960|
gi|8002675|ref|Os06g28040|
gi|8002685|ref|Os06g28110|
gi|8002687|ref|Os06g28130|
gi|8002691|ref|Os06g28170|
gi|8002695|ref|Os06g28210|
gi|8002696|ref|Os06g28220|

gi|8002697|ref|Os06g28230|
gi|8002698|ref|Os06g28240|
gi|8002700|ref|Os06g28260|
gi|8002701|ref|Os06g28270|
gi|8002702|ref|Os06g28280|
gi|8002703|ref|Os06g28290|
gi|8002710|ref|Os06g28360|
gi|8002716|ref|Os06g28420|
gi|8002717|ref|Os06g28430|
gi|8002721|ref|Os06g28470|
gi|8002724|ref|Os06g28500|
gi|8002725|ref|Os06g28510|
gi|8002726|ref|Os06g28520|
gi|8002730|ref|Os06g28560|
gi|8002731|ref|Os06g28570|
gi|8002732|ref|Os06g28580|
gi|8002734|ref|Os06g28600|
gi|8002735|ref|Os06g28610|
gi|8002736|ref|Os06g28620|
gi|8002742|ref|Os06g28680|
gi|8002743|ref|Os06g28690|
gi|8002744|ref|Os06g28700|
gi|8002746|ref|Os06g28720|
gi|8002747|ref|Os06g28730|
gi|8002750|ref|Os06g28760|
gi|8002751|ref|Os06g28770|
gi|8002753|ref|Os06g28790|
gi|8002754|ref|Os06g28800|
gi|8002758|ref|Os06g28840|
gi|8002760|ref|Os06g28860|
gi|8002761|ref|Os06g28870|
gi|8002763|ref|Os06g28890|
gi|8002764|ref|Os06g28900|
gi|8002765|ref|Os06g28910|
gi|8002768|ref|Os06g28940|
gi|8002770|ref|Os06g28960|
gi|8002771|ref|Os06g28970|
gi|8002775|ref|Os06g29010|
gi|8002782|ref|Os06g29070|
gi|8002784|ref|Os06g29090|
gi|8002785|ref|Os06g29100|
gi|8002792|ref|Os06g29170|
gi|8002794|ref|Os06g29190|
gi|8002798|ref|Os06g29230|
gi|8002800|ref|Os06g29250|
gi|8002801|ref|Os06g29260|
gi|8002803|ref|Os06g29280|
gi|8002811|ref|Os06g29360|
gi|8002812|ref|Os06g29370|
gi|8002814|ref|Os06g29390|
gi|8002819|ref|Os06g29440|
gi|8002820|ref|Os06g29450|
gi|8002825|ref|Os06g29490|
gi|8002826|ref|Os06g29490|
gi|8002827|ref|Os06g29510|
gi|8002830|ref|Os06g29540|
gi|8002833|ref|Os06g29570|
gi|8002835|ref|Os06g29590|
gi|8002838|ref|Os06g29620|
gi|8002843|ref|Os06g29670|
gi|8002844|ref|Os06g29680|
gi|8002849|ref|Os06g29730|
gi|8002853|ref|Os06g29770|
gi|8002854|ref|Os06g29780|
gi|8002859|ref|Os06g29820|
gi|8002860|ref|Os06g29830|
gi|8002863|ref|Os06g29860|
gi|8002864|ref|Os06g29870|
gi|8002865|ref|Os06g29880|
gi|8002868|ref|Os06g29910|
gi|8002870|ref|Os06g29930|
gi|8002871|ref|Os06g29940|
gi|8002874|ref|Os06g29970|
gi|8002876|ref|Os06g29990|
gi|8002881|ref|Os06g30040|
gi|8002882|ref|Os06g30050|
gi|8002889|ref|Os06g30120|
gi|8002891|ref|Os06g30140|
gi|8002898|ref|Os06g30210|
gi|8002900|ref|Os06g30230|
gi|8002901|ref|Os06g30240|
gi|8002902|ref|Os06g30250|
gi|8002903|ref|Os06g30260|
gi|8002905|ref|Os06g30280|
gi|8002906|ref|Os06g30290|
gi|8002907|ref|Os06g30300|
gi|8002911|ref|Os06g30340|
gi|8002913|ref|Os06g30360|
gi|8002922|ref|Os06g30450|
gi|8002924|ref|Os06g30470|
gi|8002925|ref|Os06g30480|
gi|8002926|ref|Os06g30490|
gi|8002930|ref|Os06g30530|
gi|8002931|ref|Os06g30540|
gi|8002932|ref|Os06g30550|
gi|8002936|ref|Os06g30590|
gi|8002937|ref|Os06g30600|
gi|8002939|ref|Os06g30620|
gi|8002940|ref|Os06g30630|
gi|8002942|ref|Os06g30650|
gi|8002943|ref|Os06g30660|
gi|8002946|ref|Os06g30690|
gi|8002950|ref|Os06g30720|
gi|8002952|ref|Os06g30740|
gi|8002959|ref|Os06g30800|
gi|8002961|ref|Os06g30820|
gi|8002964|ref|Os06g30850|
gi|8002967|ref|Os06g30880|
gi|8002971|ref|Os06g30920|
gi|8002975|ref|Os06g30960|
gi|8002977|ref|Os06g30980|
gi|8002985|ref|Os06g31060|
gi|8002986|ref|Os06g31070|
gi|8002990|ref|Os06g31110|
gi|8002996|ref|Os06g31170|
gi|8003002|ref|Os06g31230|
gi|8003005|ref|Os06g31260|
gi|8003008|ref|Os06g31290|
gi|8003011|ref|Os06g31320|
gi|8003013|ref|Os06g31340|
gi|8003015|ref|Os06g31360|
gi|8003023|ref|Os06g31440|
gi|8003025|ref|Os06g31460|
gi|8003026|ref|Os06g31470|
gi|8003028|ref|Os06g31490|
gi|8003029|ref|Os06g31500|
gi|8003030|ref|Os06g31510|
gi|8003031|ref|Os06g31520|
gi|8003032|ref|Os06g31530|
gi|8003034|ref|Os06g31550|
gi|8003035|ref|Os06g31560|
gi|8003037|ref|Os06g31580|
gi|8003038|ref|Os06g31590|
gi|8003039|ref|Os06g31600|

gi|8003040|ref|Os06g31610|
gi|8003042|ref|Os06g31630|
gi|8003045|ref|Os06g31660|
gi|8003047|ref|Os06g31680|
gi|8003050|ref|Os06g31710|
gi|8003051|ref|Os06g31720|
gi|8003053|ref|Os06g31740|
gi|8003055|ref|Os06g31760|
gi|8003056|ref|Os06g31770|
gi|8003057|ref|Os06g31780|
gi|8003058|ref|Os06g31790|
gi|8003060|ref|Os06g31810|
gi|8003061|ref|Os06g31820|
gi|8003065|ref|Os06g31860|
gi|8003067|ref|Os06g31880|
gi|8003069|ref|Os06g31900|
gi|8003073|ref|Os06g31940|
gi|8003076|ref|Os06g31970|
gi|8003077|ref|Os06g31980|
gi|8003079|ref|Os06g32000|
gi|8003083|ref|Os06g32040|
gi|8003086|ref|Os06g32070|
gi|8003092|ref|Os06g32130|
gi|8003099|ref|Os06g32200|
gi|8003100|ref|Os06g32210|
gi|8003104|ref|Os06g32250|
gi|8003108|ref|Os06g32290|
gi|8003109|ref|Os06g32300|
gi|8003110|ref|Os06g32310|
gi|8003111|ref|Os06g32320|
gi|8003115|ref|Os06g32360|
gi|8003118|ref|Os06g32390|
gi|8003119|ref|Os06g32400|
gi|8003120|ref|Os06g32410|
gi|8003121|ref|Os06g32420|
gi|8003122|ref|Os06g32430|
gi|8003123|ref|Os06g32440|
gi|8003125|ref|Os06g32460|
gi|8003127|ref|Os06g32480|
gi|8003129|ref|Os06g32500|
gi|8003131|ref|Os06g32520|
gi|8003133|ref|Os06g32540|
gi|8003135|ref|Os06g32560|
gi|8003136|ref|Os06g32570|
gi|8003138|ref|Os06g32590|
gi|8003140|ref|Os06g32610|
gi|8003143|ref|Os06g32640|
gi|8003144|ref|Os06g32650|
gi|8003147|ref|Os06g32680|
gi|8003148|ref|Os06g32690|
gi|8003149|ref|Os06g32700|
gi|8003150|ref|Os06g32710|
gi|8003154|ref|Os06g32750|
gi|8003163|ref|Os06g32840|
gi|8003171|ref|Os06g32920|
gi|8003172|ref|Os06g32930|
gi|8003180|ref|Os06g33010|
gi|8003185|ref|Os06g33060|
gi|8003190|ref|Os06g33110|
gi|8003192|ref|Os06g33130|
gi|8003193|ref|Os06g33140|
gi|8003201|ref|Os06g33220|
gi|8003209|ref|Os06g33300|
gi|8003217|ref|Os06g33380|
gi|8003218|ref|Os06g33390|
gi|8003219|ref|Os06g33400|
gi|8003220|ref|Os06g33410|
gi|8003225|ref|Os06g33460|
gi|8003226|ref|Os06g33470|
gi|8003235|ref|Os06g33560|
gi|8003238|ref|Os06g33590|
gi|8003241|ref|Os06g33620|
gi|8003242|ref|Os06g33630|
gi|8003244|ref|Os06g33650|
gi|8003245|ref|Os06g33660|
gi|8003247|ref|Os06g33680|
gi|8003249|ref|Os06g33700|
gi|8003253|ref|Os06g33730|
gi|8003254|ref|Os06g33740|
gi|8003256|ref|Os06g33760|
gi|8003258|ref|Os06g33780|
gi|8003259|ref|Os06g33790|
gi|8003260|ref|Os06g33800|
gi|8003268|ref|Os06g33880|
gi|8003271|ref|Os06g33910|
gi|8003276|ref|Os06g33960|
gi|8003280|ref|Os06g33990|
gi|8003284|ref|Os06g34030|
gi|8003289|ref|Os06g34080|
gi|8003291|ref|Os06g34100|
gi|8003292|ref|Os06g34110|
gi|8003298|ref|Os06g34160|
gi|8003300|ref|Os06g34180|
gi|8003301|ref|Os06g34190|
gi|8003302|ref|Os06g34200|
gi|8003305|ref|Os06g34230|
gi|8003307|ref|Os06g34250|
gi|8003308|ref|Os06g34260|
gi|8003309|ref|Os06g34270|
gi|8003310|ref|Os06g34280|
gi|8003311|ref|Os06g34290|
gi|8003312|ref|Os06g34300|
gi|8003313|ref|Os06g34310|
gi|8003315|ref|Os06g34330|
gi|8003316|ref|Os06g34340|
gi|8003317|ref|Os06g34350|
gi|8003321|ref|Os06g34390|
gi|8003323|ref|Os06g34410|
gi|8003328|ref|Os06g34460|
gi|8003336|ref|Os06g34540|
gi|8003337|ref|Os06g34550|
gi|8003340|ref|Os06g34570|
gi|8003343|ref|Os06g34600|
gi|8003344|ref|Os06g34610|
gi|8003346|ref|Os06g34630|
gi|8003350|ref|Os06g34670|
gi|8003353|ref|Os06g34700|
gi|8003355|ref|Os06g34720|
gi|8003356|ref|Os06g34730|
gi|8003357|ref|Os06g34740|
gi|8003358|ref|Os06g34750|
gi|8003360|ref|Os06g34770|
gi|8003363|ref|Os06g34800|
gi|8003364|ref|Os06g34810|
gi|8003368|ref|Os06g34850|
gi|8003373|ref|Os06g34900|
gi|8003375|ref|Os06g34920|
gi|8003377|ref|Os06g34940|
gi|8003385|ref|Os06g35020|
gi|8003389|ref|Os06g35060|
gi|8003390|ref|Os06g35070|
gi|8003391|ref|Os06g35080|
gi|8003392|ref|Os06g35090|
gi|8003393|ref|Os06g35100|

gi|8003396|ref|Os06g35130|
gi|8003400|ref|Os06g35170|
gi|8003407|ref|Os06g35230|
gi|8003408|ref|Os06g35240|
gi|8003415|ref|Os06g35310|
gi|8003417|ref|Os06g35330|
gi|8003418|ref|Os06g35340|
gi|8003419|ref|Os06g35350|
gi|8003424|ref|Os06g35400|
gi|8003426|ref|Os06g35420|
gi|8003435|ref|Os06g35500|
gi|8003436|ref|Os06g35510|
gi|8003442|ref|Os06g35570|
gi|8003452|ref|Os06g35670|
gi|8003453|ref|Os06g35680|
gi|8003454|ref|Os06g35690|
gi|8003457|ref|Os06g35720|
gi|8003460|ref|Os06g35750|
gi|8003461|ref|Os06g35760|
gi|8003462|ref|Os06g35770|
gi|8003464|ref|Os06g35790|
gi|8003468|ref|Os06g35830|
gi|8003473|ref|Os06g35880|
gi|8003474|ref|Os06g35890|
gi|8003477|ref|Os06g35920|
gi|8003482|ref|Os06g35970|
gi|8003483|ref|Os06g35980|
gi|8003484|ref|Os06g35990|
gi|8003492|ref|Os06g36070|
gi|8003508|ref|Os06g36230|
gi|8003510|ref|Os06g36250|
gi|8003511|ref|Os06g36260|
gi|8003520|ref|Os06g36340|
gi|8003526|ref|Os06g36370|
gi|8003527|ref|Os06g36380|
gi|8003528|ref|Os06g36390|
gi|8003529|ref|Os06g36390|
gi|8003531|ref|Os06g36410|
gi|8003533|ref|Os06g36430|
gi|8003536|ref|Os06g36460|
gi|8003541|ref|Os06g36510|
gi|8003545|ref|Os06g36550|
gi|8003549|ref|Os06g36580|
gi|8003551|ref|Os06g36600|
gi|8003552|ref|Os06g36610|
gi|8003554|ref|Os06g36630|
gi|8003560|ref|Os06g36690|
gi|8003566|ref|Os06g36750|
gi|8003570|ref|Os06g36780|
gi|8003571|ref|Os06g36790|
gi|8003573|ref|Os06g36810|
gi|8003595|ref|Os06g37020|
gi|8003606|ref|Os06g37130|
gi|8003607|ref|Os06g37140|
gi|8003610|ref|Os06g37170|
gi|8003612|ref|Os06g37190|
gi|8003613|ref|Os06g37200|
gi|8003614|ref|Os06g37210|
gi|8003617|ref|Os06g37240|
gi|8003618|ref|Os06g37250|
gi|8003620|ref|Os06g37270|
gi|8003621|ref|Os06g37280|
gi|8003624|ref|Os06g37310|
gi|8003629|ref|Os06g37360|
gi|8003630|ref|Os06g37370|
gi|8003632|ref|Os06g37390|
gi|8003640|ref|Os06g37450|
gi|8003645|ref|Os06g37490|
gi|8003649|ref|Os06g37530|
gi|8003650|ref|Os06g37540|
gi|8003651|ref|Os06g37550|
gi|8003654|ref|Os06g37580|
gi|8003661|ref|Os06g37650|
gi|8003664|ref|Os06g37680|
gi|8003666|ref|Os06g37700|
gi|8003667|ref|Os06g37710|
gi|8003670|ref|Os06g37740|
gi|8003673|ref|Os06g37770|
gi|8003674|ref|Os06g37780|
gi|8003675|ref|Os06g37790|
gi|8003676|ref|Os06g37800|
gi|8003680|ref|Os06g37840|
gi|8003683|ref|Os06g37870|
gi|8003684|ref|Os06g37880|
gi|8003686|ref|Os06g37900|
gi|8003688|ref|Os06g37920|
gi|8003689|ref|Os06g37930|
gi|8003691|ref|Os06g37950|
gi|8003692|ref|Os06g37960|
gi|8003694|ref|Os06g37980|
gi|8003696|ref|Os06g38000|
gi|8003698|ref|Os06g38020|
gi|8003707|ref|Os06g38110|
gi|8003709|ref|Os06g38120|
gi|8003711|ref|Os06g38140|
gi|8003712|ref|Os06g38150|
gi|8003714|ref|Os06g38170|
gi|8003715|ref|Os06g38180|
gi|8003716|ref|Os06g38190|
gi|8003717|ref|Os06g38200|
gi|8003718|ref|Os06g38210|
gi|8003720|ref|Os06g38230|
gi|8003722|ref|Os06g38250|
gi|8003725|ref|Os06g38280|
gi|8003728|ref|Os06g38310|
gi|8003733|ref|Os06g38350|
gi|8003734|ref|Os06g38360|
gi|8003735|ref|Os06g38370|
gi|8003736|ref|Os06g38380|
gi|8003737|ref|Os06g38390|
gi|8003741|ref|Os06g38430|
gi|8003742|ref|Os06g38440|
gi|8003748|ref|Os06g38500|
gi|8003755|ref|Os06g38570|
gi|8003756|ref|Os06g38580|
gi|8003758|ref|Os06g38600|
gi|8003764|ref|Os06g38660|
gi|8003766|ref|Os06g38680|
gi|8003769|ref|Os06g38710|
gi|8003770|ref|Os06g38720|
gi|8003772|ref|Os06g38740|
gi|8003773|ref|Os06g38750|
gi|8003775|ref|Os06g38770|
gi|8003777|ref|Os06g38790|
gi|8003778|ref|Os06g38800|
gi|8003779|ref|Os06g38810|
gi|8003782|ref|Os06g38840|
gi|8003783|ref|Os06g38850|
gi|8003785|ref|Os06g38870|
gi|8003786|ref|Os06g38880|
gi|8003789|ref|Os06g38910|
gi|8003798|ref|Os06g39000|
gi|8003799|ref|Os06g39010|
gi|8003801|ref|Os06g39030|

gi|8003808|ref|Os06g39100|
gi|8003809|ref|Os06g39110|
gi|8003810|ref|Os06g39120|
gi|8003811|ref|Os06g39130|
gi|8003818|ref|Os06g39200|
gi|8003820|ref|Os06g39220|
gi|8003827|ref|Os06g39290|
gi|8003828|ref|Os06g39300|
gi|8003830|ref|Os06g39320|
gi|8003836|ref|Os06g39380|
gi|8003839|ref|Os06g39410|
gi|8003840|ref|Os06g39420|
gi|8003841|ref|Os06g39430|
gi|8003847|ref|Os06g39490|
gi|8003848|ref|Os06g39500|
gi|8003849|ref|Os06g39510|
gi|8003852|ref|Os06g39530|
gi|8003854|ref|Os06g39550|
gi|8003855|ref|Os06g39560|
gi|8003857|ref|Os06g39580|
gi|8003860|ref|Os06g39610|
gi|8003866|ref|Os06g39670|
gi|8003877|ref|Os06g39770|
gi|8003880|ref|Os06g39800|
gi|8003881|ref|Os06g39810|
gi|8003883|ref|Os06g39830|
gi|8003885|ref|Os06g39850|
gi|8003886|ref|Os06g39860|
gi|8003889|ref|Os06g39890|
gi|8003910|ref|Os06g40100|
gi|8003913|ref|Os06g40130|
gi|8003914|ref|Os06g40140|
gi|8003916|ref|Os06g40160|
gi|8003921|ref|Os06g40210|
gi|8003923|ref|Os06g40230|
gi|8003925|ref|Os06g40250|
gi|8003929|ref|Os06g40290|
gi|8003930|ref|Os06g40300|
gi|8003932|ref|Os06g40320|
gi|8003939|ref|Os06g40390|
gi|8003940|ref|Os06g40400|
gi|8003942|ref|Os06g40420|
gi|8003943|ref|Os06g40430|
gi|8003947|ref|Os06g40450|
gi|8003948|ref|Os06g40460|
gi|8003954|ref|Os06g40520|
gi|8003960|ref|Os06g40580|
gi|8003963|ref|Os06g40610|
gi|8003973|ref|Os06g40660|
gi|8003974|ref|Os06g40670|
gi|8003976|ref|Os06g40690|
gi|8003983|ref|Os06g40760|
gi|8003987|ref|Os06g40800|
gi|8003988|ref|Os06g40810|
gi|8003994|ref|Os06g40870|
gi|8004000|ref|Os06g40930|
gi|8004005|ref|Os06g40970|
gi|8004007|ref|Os06g40990|
gi|8004008|ref|Os06g41000|
gi|8004020|ref|Os06g41120|
gi|8004021|ref|Os06g41130|
gi|8004022|ref|Os06g41140|
gi|8004023|ref|Os06g41150|
gi|8004024|ref|Os06g41160|
gi|8004026|ref|Os06g41180|
gi|8004029|ref|Os06g41210|
gi|8004030|ref|Os06g41220|
gi|8004032|ref|Os06g41240|
gi|8004034|ref|Os06g41260|
gi|8004035|ref|Os06g41270|
gi|8004036|ref|Os06g41280|
gi|8004037|ref|Os06g41290|
gi|8004038|ref|Os06g41300|
gi|8004039|ref|Os06g41310|
gi|8004041|ref|Os06g41330|
gi|8004043|ref|Os06g41350|
gi|8004049|ref|Os06g41370|
gi|8004053|ref|Os06g41400|
gi|8004063|ref|Os06g41490|
gi|8004066|ref|Os06g41520|
gi|8004067|ref|Os06g41530|
gi|8004068|ref|Os06g41540|
gi|8004069|ref|Os06g41550|
gi|8004071|ref|Os06g41570|
gi|8004072|ref|Os06g41580|
gi|8004074|ref|Os06g41600|
gi|8004076|ref|Os06g41620|
gi|8004077|ref|Os06g41630|
gi|8004079|ref|Os06g41650|
gi|8004082|ref|Os06g41680|
gi|8004086|ref|Os06g41720|
gi|8004088|ref|Os06g41740|
gi|8004090|ref|Os06g41760|
gi|8004101|ref|Os06g41870|
gi|8004103|ref|Os06g41890|
gi|8004106|ref|Os06g41920|
gi|8004111|ref|Os06g41950|
gi|8004120|ref|Os06g42040|
gi|8004121|ref|Os06g42050|
gi|8004122|ref|Os06g42060|
gi|8004123|ref|Os06g42070|
gi|8004126|ref|Os06g42100|
gi|8004127|ref|Os06g42110|
gi|8004131|ref|Os06g42150|
gi|8004133|ref|Os06g42170|
gi|8004134|ref|Os06g42180|
gi|8004143|ref|Os06g42270|
gi|8004148|ref|Os06g42320|
gi|8004149|ref|Os06g42330|
gi|8004150|ref|Os06g42340|
gi|8004155|ref|Os06g42390|
gi|8004160|ref|Os06g42440|
gi|8004161|ref|Os06g42450|
gi|8004162|ref|Os06g42460|
gi|8004163|ref|Os06g42470|
gi|8004164|ref|Os06g42480|
gi|8004170|ref|Os06g42540|
gi|8004171|ref|Os06g42550|
gi|8004179|ref|Os06g42620|
gi|8004185|ref|Os06g42680|
gi|8004186|ref|Os06g42680|
gi|8004187|ref|Os06g42690|
gi|8004192|ref|Os06g42740|
gi|8004193|ref|Os06g42750|
gi|8004194|ref|Os06g42760|
gi|8004196|ref|Os06g42780|
gi|8004201|ref|Os06g42830|
gi|8004202|ref|Os06g42840|
gi|8004206|ref|Os06g42880|
gi|8004210|ref|Os06g42920|
gi|8004211|ref|Os06g42930|
gi|8004213|ref|Os06g42950|
gi|8004218|ref|Os06g43000|
gi|8004219|ref|Os06g43010|

gi|8004220|ref|Os06g43020|
gi|8004224|ref|Os06g43060|
gi|8004225|ref|Os06g43070|
gi|8004226|ref|Os06g43080|
gi|8004228|ref|Os06g43100|
gi|8004229|ref|Os06g43110|
gi|8004230|ref|Os06g43120|
gi|8004242|ref|Os06g43230|
gi|8004244|ref|Os06g43250|
gi|8004245|ref|Os06g43260|
gi|8004255|ref|Os06g43360|
gi|8004269|ref|Os06g43500|
gi|8004273|ref|Os06g43530|
gi|8004279|ref|Os06g43580|
gi|8004295|ref|Os06g43720|
gi|8004296|ref|Os06g43730|
gi|8004298|ref|Os06g43750|
gi|8004304|ref|Os06g43810|
gi|8004305|ref|Os06g43820|
gi|8004306|ref|Os06g43830|
gi|8004311|ref|Os06g43870|
gi|8004313|ref|Os06g43890|
gi|8004316|ref|Os06g43920|
gi|8004318|ref|Os06g43940|
gi|8004319|ref|Os06g43950|
gi|8004320|ref|Os06g43960|
gi|8004321|ref|Os06g43970|
gi|8004322|ref|Os06g43980|
gi|8004323|ref|Os06g43990|
gi|8004324|ref|Os06g44000|
gi|8004326|ref|Os06g44020|
gi|8004333|ref|Os06g44090|
gi|8004343|ref|Os06g44190|
gi|8004354|ref|Os06g44280|
gi|8004359|ref|Os06g44330|
gi|8004361|ref|Os06g44350|
gi|8004362|ref|Os06g44360|
gi|8004365|ref|Os06g44390|
gi|8004366|ref|Os06g44400|
gi|8004368|ref|Os06g44420|
gi|8004375|ref|Os06g44490|
gi|8004377|ref|Os06g44510|
gi|8004378|ref|Os06g44520|
gi|8004379|ref|Os06g44530|
gi|8004381|ref|Os06g44550|
gi|8004387|ref|Os06g44610|
gi|8004389|ref|Os06g44630|
gi|8004390|ref|Os06g44640|
gi|8004391|ref|Os06g44650|
gi|8004393|ref|Os06g44670|
gi|8004394|ref|Os06g44680|
gi|8004396|ref|Os06g44700|
gi|8004398|ref|Os06g44720|
gi|8004402|ref|Os06g44760|
gi|8004403|ref|Os06g44770|
gi|8004404|ref|Os06g44780|
gi|8004407|ref|Os06g44810|
gi|8004409|ref|Os06g44830|
gi|8004413|ref|Os06g44870|
gi|8004419|ref|Os06g44930|
gi|8004430|ref|Os06g45040|
gi|8004432|ref|Os06g45060|
gi|8004436|ref|Os06g45090|
gi|8004437|ref|Os06g45090|
gi|8004441|ref|Os06g45130|
gi|8004451|ref|Os06g45220|
gi|8004461|ref|Os06g45320|
gi|8004474|ref|Os06g45420|
gi|8004479|ref|Os06g45470|
gi|8004482|ref|Os06g45490|
gi|8004489|ref|Os06g45550|
gi|8004494|ref|Os06g45600|
gi|8004496|ref|Os06g45620|
gi|8004502|ref|Os06g45680|
gi|8004504|ref|Os06g45700|
gi|8004512|ref|Os06g45780|
gi|8004537|ref|Os06g46010|
gi|8004538|ref|Os06g46020|
gi|8004540|ref|Os06g46040|
gi|8004541|ref|Os06g46050|
gi|8004542|ref|Os06g46060|
gi|8004543|ref|Os06g46070|
gi|8004544|ref|Os06g46080|
gi|8004545|ref|Os06g46090|
gi|8004546|ref|Os06g46100|
gi|8004547|ref|Os06g46110|
gi|8004550|ref|Os06g46140|
gi|8004551|ref|Os06g46150|
gi|8004552|ref|Os06g46160|
gi|8004555|ref|Os06g46190|
gi|8004556|ref|Os06g46200|
gi|8004557|ref|Os06g46210|
gi|8004559|ref|Os06g46230|
gi|8004562|ref|Os06g46260|
gi|8004565|ref|Os06g46290|
gi|8004566|ref|Os06g46300|
gi|8004569|ref|Os06g46320|
gi|8004574|ref|Os06g46360|
gi|8004580|ref|Os06g46420|
gi|8004581|ref|Os06g46430|
gi|8004584|ref|Os06g46460|
gi|8004585|ref|Os06g46470|
gi|8004587|ref|Os06g46490|
gi|8004596|ref|Os06g46580|
gi|8004602|ref|Os06g46640|
gi|8004603|ref|Os06g46650|
gi|8004612|ref|Os06g46730|
gi|8004615|ref|Os06g46760|
gi|8004623|ref|Os06g46830|
gi|8004626|ref|Os06g46860|
gi|8004629|ref|Os06g46890|
gi|8004637|ref|Os06g46970|
gi|8004638|ref|Os06g46980|
gi|8004639|ref|Os06g46990|
gi|8004641|ref|Os06g47010|
gi|8004643|ref|Os06g47030|
gi|8004645|ref|Os06g47050|
gi|8004648|ref|Os06g47080|
gi|8004649|ref|Os06g47090|
gi|8004655|ref|Os06g47140|
gi|8004657|ref|Os06g47160|
gi|8004658|ref|Os06g47170|
gi|8004664|ref|Os06g47230|
gi|8004665|ref|Os06g47240|
gi|8004669|ref|Os06g47280|
gi|8004685|ref|Os06g47410|
gi|8004686|ref|Os06g47420|
gi|8004687|ref|Os06g47430|
gi|8004689|ref|Os06g47450|
gi|8004690|ref|Os06g47460|
gi|8004692|ref|Os06g47480|
gi|8004693|ref|Os06g47490|
gi|8004696|ref|Os06g47520|
gi|8004698|ref|Os06g47540|

gi|8004710|ref|Os06g47660|
gi|8004711|ref|Os06g47670|
gi|8004713|ref|Os06g47690|
gi|8004715|ref|Os06g47710|
gi|8004745|ref|Os06g47980|
gi|8004754|ref|Os06g48050|
gi|8004761|ref|Os06g48120|
gi|8004764|ref|Os06g48150|
gi|8004768|ref|Os06g48180|
gi|8004769|ref|Os06g48190|
gi|8004772|ref|Os06g48220|
gi|8004777|ref|Os06g48260|
gi|8004778|ref|Os06g48270|
gi|8004779|ref|Os06g48280|
gi|8004785|ref|Os06g48340|
gi|8004787|ref|Os06g48360|
gi|8004801|ref|Os06g48500|
gi|8004802|ref|Os06g48500|
gi|8004806|ref|Os06g48540|
gi|8004807|ref|Os06g48550|
gi|8004821|ref|Os06g48670|
gi|8004822|ref|Os06g48680|
gi|8004823|ref|Os06g48690|
gi|8004827|ref|Os06g48730|
gi|8004828|ref|Os06g48740|
gi|8004834|ref|Os06g48790|
gi|8004838|ref|Os06g48830|
gi|8004843|ref|Os06g48880|
gi|8004844|ref|Os06g48890|
gi|8004846|ref|Os06g48910|
gi|8004847|ref|Os06g48920|
gi|8004890|ref|Os06g49280|
gi|8004891|ref|Os06g49290|
gi|8004895|ref|Os06g49330|
gi|8004899|ref|Os06g49370|
gi|8004903|ref|Os06g49410|
gi|8004925|ref|Os06g49570|
gi|8004927|ref|Os06g49590|
gi|8004928|ref|Os06g49600|
gi|8004929|ref|Os06g49610|
gi|8004930|ref|Os06g49620|
gi|8004938|ref|Os06g49680|
gi|8004939|ref|Os06g49690|
gi|8004940|ref|Os06g49700|
gi|8004941|ref|Os06g49700|
gi|8004944|ref|Os06g49730|
gi|8004950|ref|Os06g49780|
gi|8004957|ref|Os06g49850|
gi|8004958|ref|Os06g49850|
gi|8004964|ref|Os06g49900|
gi|8004966|ref|Os06g49920|
gi|8004984|ref|Os06g50070|
gi|8004986|ref|Os06g50090|
gi|8005007|ref|Os06g50250|
gi|8005025|ref|Os06g50430|
gi|8005026|ref|Os06g50440|
gi|8005028|ref|Os06g50460|
gi|8005029|ref|Os06g50470|
gi|8005031|ref|Os06g50490|
gi|8005032|ref|Os06g50500|
gi|8005035|ref|Os06g50530|
gi|8005036|ref|Os06g50540|
gi|8005037|ref|Os06g50550|
gi|8005039|ref|Os06g50570|
gi|8005040|ref|Os06g50580|
gi|8005041|ref|Os06g50580|
gi|8005042|ref|Os06g50590|
gi|8005044|ref|Os06g50610|
gi|8005045|ref|Os06g50620|
gi|8005047|ref|Os06g50640|
gi|8005049|ref|Os06g50660|
gi|8005050|ref|Os06g50670|
gi|8005051|ref|Os06g50670|
gi|8005052|ref|Os06g50680|
gi|8005054|ref|Os06g50700|
gi|8005055|ref|Os06g50710|
gi|8005056|ref|Os06g50720|
gi|8005058|ref|Os06g50740|
gi|8005059|ref|Os06g50750|
gi|8005060|ref|Os06g50760|
gi|8005061|ref|Os06g50770|
gi|8005063|ref|Os06g50790|
gi|8005065|ref|Os06g50810|
gi|8005066|ref|Os06g50820|
gi|8005085|ref|Os06g50990|
gi|8005090|ref|Os06g51040|
gi|8005095|ref|Os06g51090|
gi|8005099|ref|Os06g51120|
gi|8005101|ref|Os06g51140|
gi|8005113|ref|Os06g51230|
gi|8005114|ref|Os06g51240|
gi|8005115|ref|Os06g51240|
gi|8005124|ref|Os06g51300|
gi|8005135|ref|Os06g51400|
gi|8005139|ref|Os06g51440|
gi|8005146|ref|Os06g51500|
gi|8005155|ref|Os06g51560|
gi|8100005|ref|Os09g01050|
gi|8100008|ref|Os09g01080|
gi|8100013|ref|Os09g01130|
gi|8100014|ref|Os09g01140|
gi|8100015|ref|Os09g01150|
gi|8100016|ref|Os09g01160|
gi|8100018|ref|Os09g01180|
gi|8100020|ref|Os09g01200|
gi|8100021|ref|Os09g01210|
gi|8100022|ref|Os09g01220|
gi|8100031|ref|Os09g01310|
gi|8100032|ref|Os09g01320|
gi|8100033|ref|Os09g01330|
gi|8100040|ref|Os09g01400|
gi|8100042|ref|Os09g01420|
gi|8100044|ref|Os09g01440|
gi|8100047|ref|Os09g01470|
gi|8100050|ref|Os09g01500|
gi|8100051|ref|Os09g01510|
gi|8100058|ref|Os09g01580|
gi|8100064|ref|Os09g01610|
gi|8100069|ref|Os09g01660|
gi|8100077|ref|Os09g01730|
gi|8100078|ref|Os09g01740|
gi|8100083|ref|Os09g01790|
gi|8100090|ref|Os09g01860|
gi|8100097|ref|Os09g01930|
gi|8100098|ref|Os09g01940|
gi|8100099|ref|Os09g01950|
gi|8100101|ref|Os09g01970|
gi|8100104|ref|Os09g02000|
gi|8100105|ref|Os09g02010|
gi|8100110|ref|Os09g02060|
gi|8100116|ref|Os09g02120|
gi|8100119|ref|Os09g02150|
gi|8100120|ref|Os09g02160|
gi|8100122|ref|Os09g02180|

gi|8100124|ref|Os09g02200|
gi|8100127|ref|Os09g02230|
gi|8100128|ref|Os09g02240|
gi|8100134|ref|Os09g02300|
gi|8100135|ref|Os09g02310|
gi|8100136|ref|Os09g02320|
gi|8100137|ref|Os09g02330|
gi|8100138|ref|Os09g02340|
gi|8100139|ref|Os09g02350|
gi|8100141|ref|Os09g02370|
gi|8100142|ref|Os09g02380|
gi|8100143|ref|Os09g02390|
gi|8100148|ref|Os09g02420|
gi|8100149|ref|Os09g02430|
gi|8100151|ref|Os09g02450|
gi|8100153|ref|Os09g02470|
gi|8100155|ref|Os09g02490|
gi|8100157|ref|Os09g02510|
gi|8100158|ref|Os09g02520|
gi|8100161|ref|Os09g02550|
gi|8100162|ref|Os09g02560|
gi|8100163|ref|Os09g02570|
gi|8100164|ref|Os09g02580|
gi|8100167|ref|Os09g02610|
gi|8100168|ref|Os09g02620|
gi|8100170|ref|Os09g02640|
gi|8100173|ref|Os09g02660|
gi|8100174|ref|Os09g02670|
gi|8100176|ref|Os09g02690|
gi|8100184|ref|Os09g02770|
gi|8100187|ref|Os09g02800|
gi|8100189|ref|Os09g02820|
gi|8100192|ref|Os09g02850|
gi|8100193|ref|Os09g02860|
gi|8100195|ref|Os09g02880|
gi|8100197|ref|Os09g02900|
gi|8100198|ref|Os09g02910|
gi|8100204|ref|Os09g02970|
gi|8100209|ref|Os09g03020|
gi|8100212|ref|Os09g03050|
gi|8100214|ref|Os09g03070|
gi|8100219|ref|Os09g03120|
gi|8100220|ref|Os09g03130|
gi|8100224|ref|Os09g03170|
gi|8100226|ref|Os09g03190|
gi|8100227|ref|Os09g03200|
gi|8100228|ref|Os09g03210|
gi|8100229|ref|Os09g03220|
gi|8100230|ref|Os09g03230|
gi|8100232|ref|Os09g03250|
gi|8100234|ref|Os09g03270|
gi|8100239|ref|Os09g03320|
gi|8100246|ref|Os09g03390|
gi|8100253|ref|Os09g03460|
gi|8100255|ref|Os09g03480|
gi|8100258|ref|Os09g03510|
gi|8100260|ref|Os09g03530|
gi|8100262|ref|Os09g03550|
gi|8100263|ref|Os09g03560|
gi|8100265|ref|Os09g03580|
gi|8100272|ref|Os09g03640|
gi|8100275|ref|Os09g03670|
gi|8100282|ref|Os09g03740|
gi|8100284|ref|Os09g03760|
gi|8100287|ref|Os09g03790|
gi|8100288|ref|Os09g03800|
gi|8100289|ref|Os09g03810|
gi|8100290|ref|Os09g03820|
gi|8100293|ref|Os09g03850|
gi|8100299|ref|Os09g03910|
gi|8100302|ref|Os09g03940|
gi|8100304|ref|Os09g03960|
gi|8100305|ref|Os09g03970|
gi|8100307|ref|Os09g03990|
gi|8100311|ref|Os09g04030|
gi|8100314|ref|Os09g04060|
gi|8100317|ref|Os09g04090|
gi|8100323|ref|Os09g04120|
gi|8100332|ref|Os09g04190|
gi|8100335|ref|Os09g04220|
gi|8100336|ref|Os09g04230|
gi|8100338|ref|Os09g04250|
gi|8100342|ref|Os09g04290|
gi|8100343|ref|Os09g04300|
gi|8100344|ref|Os09g04310|
gi|8100346|ref|Os09g04330|
gi|8100349|ref|Os09g04360|
gi|8100350|ref|Os09g04370|
gi|8100351|ref|Os09g04380|
gi|8100352|ref|Os09g04390|
gi|8100356|ref|Os09g04430|
gi|8100359|ref|Os09g04450|
gi|8100361|ref|Os09g04470|
gi|8100362|ref|Os09g04480|
gi|8100364|ref|Os09g04500|
gi|8100365|ref|Os09g04510|
gi|8100370|ref|Os09g04560|
gi|8100373|ref|Os09g04590|
gi|8100374|ref|Os09g04600|
gi|8100378|ref|Os09g04640|
gi|8100379|ref|Os09g04650|
gi|8100384|ref|Os09g04690|
gi|8100385|ref|Os09g04700|
gi|8100388|ref|Os09g04720|
gi|8100392|ref|Os09g04750|
gi|8100393|ref|Os09g04760|
gi|8100394|ref|Os09g04770|
gi|8100395|ref|Os09g04780|
gi|8100399|ref|Os09g04820|
gi|8100403|ref|Os09g04860|
gi|8100404|ref|Os09g04870|
gi|8100408|ref|Os09g04900|
gi|8100409|ref|Os09g04910|
gi|8100415|ref|Os09g04970|
gi|8100419|ref|Os09g05010|
gi|8100422|ref|Os09g05040|
gi|8100429|ref|Os09g05110|
gi|8100443|ref|Os09g06240|
gi|8100449|ref|Os09g06300|
gi|8100450|ref|Os09g06310|
gi|8100452|ref|Os09g06330|
gi|8100455|ref|Os09g06360|
gi|8100457|ref|Os09g06380|
gi|8100461|ref|Os09g06420|
gi|8100463|ref|Os09g06440|
gi|8100472|ref|Os09g06530|
gi|8100474|ref|Os09g06550|
gi|8100476|ref|Os09g06570|
gi|8100486|ref|Os09g06670|
gi|8100488|ref|Os09g06690|
gi|8100489|ref|Os09g06700|
gi|8100491|ref|Os09g06720|
gi|8100492|ref|Os09g06730|
gi|8100494|ref|Os09g06750|

gi|8100495|ref|Os09g06760|
gi|8100502|ref|Os09g06830|
gi|8100504|ref|Os09g06850|
gi|8100505|ref|Os09g06860|
gi|8100507|ref|Os09g06880|
gi|8100509|ref|Os09g06900|
gi|8100512|ref|Os09g06930|
gi|8100513|ref|Os09g06940|
gi|8100514|ref|Os09g06950|
gi|8100515|ref|Os09g06960|
gi|8100518|ref|Os09g06990|
gi|8100519|ref|Os09g07000|
gi|8100522|ref|Os09g07030|
gi|8100523|ref|Os09g07040|
gi|8100529|ref|Os09g07100|
gi|8100532|ref|Os09g07130|
gi|8100534|ref|Os09g07150|
gi|8100535|ref|Os09g07160|
gi|8100543|ref|Os09g07240|
gi|8100545|ref|Os09g07260|
gi|8100547|ref|Os09g07280|
gi|8100550|ref|Os09g07310|
gi|8100557|ref|Os09g07370|
gi|8100559|ref|Os09g07390|
gi|8100568|ref|Os09g07470|
gi|8100570|ref|Os09g07490|
gi|8100574|ref|Os09g07520|
gi|8100577|ref|Os09g07550|
gi|8100578|ref|Os09g07560|
gi|8100584|ref|Os09g07620|
gi|8100585|ref|Os09g07630|
gi|8100587|ref|Os09g07650|
gi|8100594|ref|Os09g07720|
gi|8100599|ref|Os09g07770|
gi|8100601|ref|Os09g07790|
gi|8100603|ref|Os09g07810|
gi|8100607|ref|Os09g07840|
gi|8100616|ref|Os09g07930|
gi|8100617|ref|Os09g07940|
gi|8100622|ref|Os09g07990|
gi|8100623|ref|Os09g08000|
gi|8100628|ref|Os09g08050|
gi|8100630|ref|Os09g08070|
gi|8100633|ref|Os09g08100|
gi|8100634|ref|Os09g08110|
gi|8100638|ref|Os09g08140|
gi|8100639|ref|Os09g08150|
gi|8100644|ref|Os09g08200|
gi|8100645|ref|Os09g08210|
gi|8100649|ref|Os09g08250|
gi|8100650|ref|Os09g08260|
gi|8100652|ref|Os09g08280|
gi|8100654|ref|Os09g08300|
gi|8100656|ref|Os09g08320|
gi|8100657|ref|Os09g08330|
gi|8100660|ref|Os09g08360|
gi|8100662|ref|Os09g08380|
gi|8100665|ref|Os09g08400|
gi|8100666|ref|Os09g08410|
gi|8100673|ref|Os09g08480|
gi|8100675|ref|Os09g08500|
gi|8100677|ref|Os09g08520|
gi|8100678|ref|Os09g08530|
gi|8100680|ref|Os09g08550|
gi|8100681|ref|Os09g08560|
gi|8100682|ref|Os09g08570|
gi|8100683|ref|Os09g08580|
gi|8100686|ref|Os09g08610|
gi|8100687|ref|Os09g08620|
gi|8100694|ref|Os09g08690|
gi|8100695|ref|Os09g08700|
gi|8100696|ref|Os09g08710|
gi|8100699|ref|Os09g08740|
gi|8100702|ref|Os09g08770|
gi|8100706|ref|Os09g08810|
gi|8100707|ref|Os09g08820|
gi|8100709|ref|Os09g08840|
gi|8100720|ref|Os09g08950|
gi|8100722|ref|Os09g08970|
gi|8100726|ref|Os09g09010|
gi|8100728|ref|Os09g09030|
gi|8100729|ref|Os09g09040|
gi|8100739|ref|Os09g09140|
gi|8100742|ref|Os09g09170|
gi|8100743|ref|Os09g09180|
gi|8100744|ref|Os09g09190|
gi|8100745|ref|Os09g09200|
gi|8100746|ref|Os09g09210|
gi|8100751|ref|Os09g09260|
gi|8100759|ref|Os09g09330|
gi|8100760|ref|Os09g09340|
gi|8100761|ref|Os09g09350|
gi|8100765|ref|Os09g09380|
gi|8100768|ref|Os09g09410|
gi|8100782|ref|Os09g09530|
gi|8100786|ref|Os09g09570|
gi|8100790|ref|Os09g09610|
gi|8100794|ref|Os09g09650|
gi|8100801|ref|Os09g09720|
gi|8100802|ref|Os09g09730|
gi|8100803|ref|Os09g09740|
gi|8100811|ref|Os09g09820|
gi|8100821|ref|Os09g09920|
gi|8100823|ref|Os09g09940|
gi|8100826|ref|Os09g09970|
gi|8100830|ref|Os09g10010|
gi|8100835|ref|Os09g10060|
gi|8100838|ref|Os09g10090|
gi|8100840|ref|Os09g10110|
gi|8100842|ref|Os09g10130|
gi|8100844|ref|Os09g10150|
gi|8100847|ref|Os09g10180|
gi|8100850|ref|Os09g10210|
gi|8100854|ref|Os09g10250|
gi|8100857|ref|Os09g10280|
gi|8100858|ref|Os09g10290|
gi|8100860|ref|Os09g10310|
gi|8100862|ref|Os09g10330|
gi|8100865|ref|Os09g10360|
gi|8100866|ref|Os09g10370|
gi|8100869|ref|Os09g10400|
gi|8100871|ref|Os09g10420|
gi|8100872|ref|Os09g10430|
gi|8100873|ref|Os09g10440|
gi|8100875|ref|Os09g10460|
gi|8100877|ref|Os09g10480|
gi|8100878|ref|Os09g10490|
gi|8100880|ref|Os09g10510|
gi|8100881|ref|Os09g10520|
gi|8100885|ref|Os09g10560|
gi|8100886|ref|Os09g10570|
gi|8100887|ref|Os09g10580|
gi|8100888|ref|Os09g10590|
gi|8100890|ref|Os09g10610|

gi|8100891|ref|Os09g10620|
gi|8100892|ref|Os09g10630|
gi|8100895|ref|Os09g10660|
gi|8100896|ref|Os09g10670|
gi|8100897|ref|Os09g10680|
gi|8100898|ref|Os09g10690|
gi|8100899|ref|Os09g10700|
gi|8100908|ref|Os09g10780|
gi|8100910|ref|Os09g10800|
gi|8100911|ref|Os09g10810|
gi|8100913|ref|Os09g10830|
gi|8100916|ref|Os09g10860|
gi|8100918|ref|Os09g10880|
gi|8100933|ref|Os09g10970|
gi|8100935|ref|Os09g10990|
gi|8100936|ref|Os09g11000|
gi|8100941|ref|Os09g11050|
gi|8100944|ref|Os09g11080|
gi|8100945|ref|Os09g11090|
gi|8100949|ref|Os09g11130|
gi|8100950|ref|Os09g11140|
gi|8100951|ref|Os09g11150|
gi|8100952|ref|Os09g11160|
gi|8100954|ref|Os09g11180|
gi|8100955|ref|Os09g11190|
gi|8100956|ref|Os09g11200|
gi|8100957|ref|Os09g11210|
gi|8100964|ref|Os09g11280|
gi|8100966|ref|Os09g11300|
gi|8100967|ref|Os09g11310|
gi|8100968|ref|Os09g11320|
gi|8100969|ref|Os09g11330|
gi|8100973|ref|Os09g11370|
gi|8100983|ref|Os09g11470|
gi|8100989|ref|Os09g11530|
gi|8100990|ref|Os09g11540|
gi|8100992|ref|Os09g11560|
gi|8100995|ref|Os09g11590|
gi|8101001|ref|Os09g11650|
gi|8101002|ref|Os09g11660|
gi|8101003|ref|Os09g11670|
gi|8101004|ref|Os09g11680|
gi|8101005|ref|Os09g11690|
gi|8101006|ref|Os09g11700|
gi|8101008|ref|Os09g11720|
gi|8101013|ref|Os09g11770|
gi|8101014|ref|Os09g11780|
gi|8101015|ref|Os09g11790|
gi|8101016|ref|Os09g11800|
gi|8101017|ref|Os09g11810|
gi|8101018|ref|Os09g11820|
gi|8101019|ref|Os09g11830|
gi|8101020|ref|Os09g11840|
gi|8101024|ref|Os09g11880|
gi|8101026|ref|Os09g11900|
gi|8101029|ref|Os09g11930|
gi|8101032|ref|Os09g11960|
gi|8101033|ref|Os09g11970|
gi|8101035|ref|Os09g11990|
gi|8101036|ref|Os09g12000|
gi|8101037|ref|Os09g12010|
gi|8101042|ref|Os09g12060|
gi|8101048|ref|Os09g12120|
gi|8101051|ref|Os09g12150|
gi|8101053|ref|Os09g12170|
gi|8101056|ref|Os09g12200|
gi|8101057|ref|Os09g12210|
gi|8101058|ref|Os09g12220|
gi|8101061|ref|Os09g12250|
gi|8101062|ref|Os09g12260|
gi|8101072|ref|Os09g12360|
gi|8101075|ref|Os09g12390|
gi|8101076|ref|Os09g12400|
gi|8101079|ref|Os09g12430|
gi|8101080|ref|Os09g12440|
gi|8101081|ref|Os09g12450|
gi|8101082|ref|Os09g12460|
gi|8101083|ref|Os09g12470|
gi|8101085|ref|Os09g12490|
gi|8101087|ref|Os09g12510|
gi|8101088|ref|Os09g12520|
gi|8101094|ref|Os09g12580|
gi|8101097|ref|Os09g12610|
gi|8101099|ref|Os09g12630|
gi|8101100|ref|Os09g12640|
gi|8101105|ref|Os09g12680|
gi|8101106|ref|Os09g12690|
gi|8101107|ref|Os09g12700|
gi|8101122|ref|Os09g12850|
gi|8101123|ref|Os09g12860|
gi|8101127|ref|Os09g12900|
gi|8101128|ref|Os09g12910|
gi|8101132|ref|Os09g12950|
gi|8101133|ref|Os09g12960|
gi|8101136|ref|Os09g12990|
gi|8101137|ref|Os09g13000|
gi|8101138|ref|Os09g13010|
gi|8101139|ref|Os09g13020|
gi|8101142|ref|Os09g13050|
gi|8101145|ref|Os09g13080|
gi|8101146|ref|Os09g13090|
gi|8101149|ref|Os09g13120|
gi|8101150|ref|Os09g13130|
gi|8101153|ref|Os09g13160|
gi|8101154|ref|Os09g13170|
gi|8101156|ref|Os09g13190|
gi|8101157|ref|Os09g13200|
gi|8101159|ref|Os09g13220|
gi|8101161|ref|Os09g13240|
gi|8101162|ref|Os09g13250|
gi|8101163|ref|Os09g13260|
gi|8101165|ref|Os09g13280|
gi|8101166|ref|Os09g13290|
gi|8101168|ref|Os09g13310|
gi|8101175|ref|Os09g13380|
gi|8101176|ref|Os09g13390|
gi|8101177|ref|Os09g13400|
gi|8101179|ref|Os09g13420|
gi|8101180|ref|Os09g13430|
gi|8101181|ref|Os09g13440|
gi|8101182|ref|Os09g13440|
gi|8101183|ref|Os09g13450|
gi|8101184|ref|Os09g13460|
gi|8101187|ref|Os09g13490|
gi|8101190|ref|Os09g13520|
gi|8101195|ref|Os09g13570|
gi|8101197|ref|Os09g13590|
gi|8101198|ref|Os09g13600|
gi|8101202|ref|Os09g13620|
gi|8101204|ref|Os09g13650|
gi|8101211|ref|Os09g13720|
gi|8101215|ref|Os09g13760|
gi|8101216|ref|Os09g13770|
gi|8101220|ref|Os09g13810|

gi|8101222|ref|Os09g13830|
gi|8101223|ref|Os09g13840|
gi|8101225|ref|Os09g13860|
gi|8101230|ref|Os09g13910|
gi|8101231|ref|Os09g13920|
gi|8101232|ref|Os09g13930|
gi|8101234|ref|Os09g13950|
gi|8101235|ref|Os09g13960|
gi|8101239|ref|Os09g14000|
gi|8101241|ref|Os09g14020|
gi|8101244|ref|Os09g14050|
gi|8101246|ref|Os09g14070|
gi|8101248|ref|Os09g14090|
gi|8101252|ref|Os09g14130|
gi|8101253|ref|Os09g14140|
gi|8101256|ref|Os09g14170|
gi|8101260|ref|Os09g14210|
gi|8101263|ref|Os09g14240|
gi|8101269|ref|Os09g14300|
gi|8101274|ref|Os09g14350|
gi|8101277|ref|Os09g14380|
gi|8101279|ref|Os09g14400|
gi|8101281|ref|Os09g14420|
gi|8101285|ref|Os09g14460|
gi|8101296|ref|Os09g14560|
gi|8101304|ref|Os09g14640|
gi|8101316|ref|Os09g14760|
gi|8101326|ref|Os09g14860|
gi|8101329|ref|Os09g14890|
gi|8101331|ref|Os09g14910|
gi|8101332|ref|Os09g14920|
gi|8101335|ref|Os09g14950|
gi|8101337|ref|Os09g14970|
gi|8101338|ref|Os09g14980|
gi|8101344|ref|Os09g15040|
gi|8101347|ref|Os09g15070|
gi|8101351|ref|Os09g15110|
gi|8101355|ref|Os09g15150|
gi|8101358|ref|Os09g15180|
gi|8101361|ref|Os09g15210|
gi|8101363|ref|Os09g15230|
gi|8101366|ref|Os09g15260|
gi|8101369|ref|Os09g15290|
gi|8101371|ref|Os09g15310|
gi|8101376|ref|Os09g15350|
gi|8101377|ref|Os09g15360|
gi|8101382|ref|Os09g15410|
gi|8101387|ref|Os09g15450|
gi|8101391|ref|Os09g15490|
gi|8101394|ref|Os09g15520|
gi|8101395|ref|Os09g15530|
gi|8101396|ref|Os09g15540|
gi|8101397|ref|Os09g15550|
gi|8101399|ref|Os09g15570|
gi|8101402|ref|Os09g15600|
gi|8101407|ref|Os09g15650|
gi|8101408|ref|Os09g15660|
gi|8101410|ref|Os09g15680|
gi|8101413|ref|Os09g15710|
gi|8101414|ref|Os09g15720|
gi|8101415|ref|Os09g15730|
gi|8101416|ref|Os09g15740|
gi|8101425|ref|Os09g15830|
gi|8101428|ref|Os09g15860|
gi|8101429|ref|Os09g15870|
gi|8101430|ref|Os09g15880|
gi|8101433|ref|Os09g15910|
gi|8101435|ref|Os09g15930|
gi|8101439|ref|Os09g15970|
gi|8101440|ref|Os09g15980|
gi|8101444|ref|Os09g16020|
gi|8101449|ref|Os09g16070|
gi|8101450|ref|Os09g16080|
gi|8101454|ref|Os09g16120|
gi|8101455|ref|Os09g16130|
gi|8101456|ref|Os09g16140|
gi|8101459|ref|Os09g16170|
gi|8101460|ref|Os09g16180|
gi|8101461|ref|Os09g16190|
gi|8101463|ref|Os09g16210|
gi|8101465|ref|Os09g16230|
gi|8101467|ref|Os09g16250|
gi|8101469|ref|Os09g16270|
gi|8101470|ref|Os09g16280|
gi|8101472|ref|Os09g16300|
gi|8101473|ref|Os09g16310|
gi|8101474|ref|Os09g16320|
gi|8101481|ref|Os09g16390|
gi|8101483|ref|Os09g16410|
gi|8101484|ref|Os09g16420|
gi|8101488|ref|Os09g16460|
gi|8101489|ref|Os09g16470|
gi|8101492|ref|Os09g16500|
gi|8101498|ref|Os09g16560|
gi|8101499|ref|Os09g16570|
gi|8101503|ref|Os09g16610|
gi|8101508|ref|Os09g16660|
gi|8101511|ref|Os09g16690|
gi|8101513|ref|Os09g16710|
gi|8101514|ref|Os09g16720|
gi|8101516|ref|Os09g16740|
gi|8101517|ref|Os09g16750|
gi|8101519|ref|Os09g16770|
gi|8101520|ref|Os09g16780|
gi|8101522|ref|Os09g16800|
gi|8101524|ref|Os09g16820|
gi|8101539|ref|Os09g16960|
gi|8101540|ref|Os09g16970|
gi|8101542|ref|Os09g16990|
gi|8101547|ref|Os09g17040|
gi|8101548|ref|Os09g17050|
gi|8101549|ref|Os09g17060|
gi|8101550|ref|Os09g17070|
gi|8101553|ref|Os09g17100|
gi|8101559|ref|Os09g17160|
gi|8101561|ref|Os09g17180|
gi|8101564|ref|Os09g17210|
gi|8101565|ref|Os09g17220|
gi|8101566|ref|Os09g17230|
gi|8101567|ref|Os09g17240|
gi|8101569|ref|Os09g17260|
gi|8101570|ref|Os09g17270|
gi|8101573|ref|Os09g17300|
gi|8101574|ref|Os09g17310|
gi|8101575|ref|Os09g17320|
gi|8101578|ref|Os09g17350|
gi|8101579|ref|Os09g17360|
gi|8101586|ref|Os09g17430|
gi|8101587|ref|Os09g17440|
gi|8101589|ref|Os09g17460|
gi|8101593|ref|Os09g17500|
gi|8101598|ref|Os09g17550|
gi|8101600|ref|Os09g17570|
gi|8101602|ref|Os09g17590|

gi|8101609|ref|Os09g17640|
gi|8101610|ref|Os09g17650|
gi|8101612|ref|Os09g17670|
gi|8101614|ref|Os09g17690|
gi|8101615|ref|Os09g17700|
gi|8101617|ref|Os09g17720|
gi|8101623|ref|Os09g17780|
gi|8101624|ref|Os09g17790|
gi|8101627|ref|Os09g17820|
gi|8101635|ref|Os09g17900|
gi|8101638|ref|Os09g17930|
gi|8101639|ref|Os09g17940|
gi|8101643|ref|Os09g17980|
gi|8101650|ref|Os09g18050|
gi|8101651|ref|Os09g18060|
gi|8101662|ref|Os09g18170|
gi|8101664|ref|Os09g18190|
gi|8101670|ref|Os09g18250|
gi|8101672|ref|Os09g18270|
gi|8101674|ref|Os09g18290|
gi|8101675|ref|Os09g18300|
gi|8101676|ref|Os09g18310|
gi|8101678|ref|Os09g18330|
gi|8101679|ref|Os09g18340|
gi|8101687|ref|Os09g18420|
gi|8101691|ref|Os09g18460|
gi|8101693|ref|Os09g18480|
gi|8101696|ref|Os09g18510|
gi|8101701|ref|Os09g18560|
gi|8101703|ref|Os09g18580|
gi|8101705|ref|Os09g18600|
gi|8101708|ref|Os09g18630|
gi|8101709|ref|Os09g19130|
gi|8101713|ref|Os09g19170|
gi|8101716|ref|Os09g19200|
gi|8101717|ref|Os09g19210|
gi|8101722|ref|Os09g19260|
gi|8101723|ref|Os09g19270|
gi|8101728|ref|Os09g19320|
gi|8101729|ref|Os09g19330|
gi|8101733|ref|Os09g19370|
gi|8101738|ref|Os09g19420|
gi|8101742|ref|Os09g19460|
gi|8101743|ref|Os09g19470|
gi|8101744|ref|Os09g19480|
gi|8101745|ref|Os09g19490|
gi|8101748|ref|Os09g19520|
gi|8101754|ref|Os09g19580|
gi|8101755|ref|Os09g19590|
gi|8101762|ref|Os09g19660|
gi|8101765|ref|Os09g19690|
gi|8101773|ref|Os09g19760|
gi|8101775|ref|Os09g19780|
gi|8101781|ref|Os09g19840|
gi|8101784|ref|Os09g19870|
gi|8101793|ref|Os09g19960|
gi|8101794|ref|Os09g19970|
gi|8101802|ref|Os09g20050|
gi|8101807|ref|Os09g20100|
gi|8101808|ref|Os09g20110|
gi|8101811|ref|Os09g20140|
gi|8101814|ref|Os09g20170|
gi|8101815|ref|Os09g20180|
gi|8101816|ref|Os09g20190|
gi|8101817|ref|Os09g20200|
gi|8101818|ref|Os09g20210|
gi|8101820|ref|Os09g20230|
gi|8101824|ref|Os09g20270|
gi|8101828|ref|Os09g20310|
gi|8101833|ref|Os09g20360|
gi|8101834|ref|Os09g20370|
gi|8101837|ref|Os09g20400|
gi|8101838|ref|Os09g20410|
gi|8101839|ref|Os09g20420|
gi|8101845|ref|Os09g20470|
gi|8101855|ref|Os09g20570|
gi|8101858|ref|Os09g20600|
gi|8101860|ref|Os09g20620|
gi|8101866|ref|Os09g20670|
gi|8101874|ref|Os09g20750|
gi|8101875|ref|Os09g20760|
gi|8101878|ref|Os09g20790|
gi|8101883|ref|Os09g20840|
gi|8101886|ref|Os09g20870|
gi|8101888|ref|Os09g20890|
gi|8101891|ref|Os09g20920|
gi|8101892|ref|Os09g20930|
gi|8101893|ref|Os09g20940|
gi|8101895|ref|Os09g20960|
gi|8101900|ref|Os09g21010|
gi|8101902|ref|Os09g21030|
gi|8101903|ref|Os09g21040|
gi|8101904|ref|Os09g21050|
gi|8101905|ref|Os09g21060|
gi|8101906|ref|Os09g21070|
gi|8101907|ref|Os09g21080|
gi|8101908|ref|Os09g21090|
gi|8101912|ref|Os09g21130|
gi|8101913|ref|Os09g21140|
gi|8101914|ref|Os09g21150|
gi|8101921|ref|Os09g21220|
gi|8101924|ref|Os09g21240|
gi|8101927|ref|Os09g21270|
gi|8101928|ref|Os09g21280|
gi|8101929|ref|Os09g21290|
gi|8101930|ref|Os09g21300|
gi|8101933|ref|Os09g21330|
gi|8101936|ref|Os09g21360|
gi|8101944|ref|Os09g21440|
gi|8101948|ref|Os09g21480|
gi|8101950|ref|Os09g21500|
gi|8101954|ref|Os09g21530|
gi|8101955|ref|Os09g21540|
gi|8101956|ref|Os09g21550|
gi|8101960|ref|Os09g21590|
gi|8101964|ref|Os09g21630|
gi|8101966|ref|Os09g21650|
gi|8101967|ref|Os09g21660|
gi|8101968|ref|Os09g21670|
gi|8101970|ref|Os09g21690|
gi|8101973|ref|Os09g21720|
gi|8101974|ref|Os09g21730|
gi|8101976|ref|Os09g21750|
gi|8101981|ref|Os09g21790|
gi|8101983|ref|Os09g21810|
gi|8101985|ref|Os09g21830|
gi|8101989|ref|Os09g21870|
gi|8101990|ref|Os09g21880|
gi|8101992|ref|Os09g21900|
gi|8101994|ref|Os09g21920|
gi|8101995|ref|Os09g21930|
gi|8101999|ref|Os09g21970|
gi|8102004|ref|Os09g22020|
gi|8102012|ref|Os09g22100|

gi|8102015|ref|Os09g22130|
gi|8102016|ref|Os09g22140|
gi|8102018|ref|Os09g22160|
gi|8102019|ref|Os09g22170|
gi|8102020|ref|Os09g22180|
gi|8102023|ref|Os09g22210|
gi|8102026|ref|Os09g22240|
gi|8102027|ref|Os09g22250|
gi|8102028|ref|Os09g22260|
gi|8102029|ref|Os09g22270|
gi|8102030|ref|Os09g22280|
gi|8102031|ref|Os09g22290|
gi|8102039|ref|Os09g22370|
gi|8102040|ref|Os09g22380|
gi|8102041|ref|Os09g22390|
gi|8102042|ref|Os09g22400|
gi|8102044|ref|Os09g22420|
gi|8102045|ref|Os09g22430|
gi|8102049|ref|Os09g22470|
gi|8102050|ref|Os09g22480|
gi|8102051|ref|Os09g22490|
gi|8102052|ref|Os09g22500|
gi|8102053|ref|Os09g22510|
gi|8102054|ref|Os09g22520|
gi|8102055|ref|Os09g22530|
gi|8102057|ref|Os09g22550|
gi|8102058|ref|Os09g23050|
gi|8102059|ref|Os09g23060|
gi|8102070|ref|Os09g23170|
gi|8102071|ref|Os09g23180|
gi|8102074|ref|Os09g23210|
gi|8102078|ref|Os09g23250|
gi|8102080|ref|Os09g23270|
gi|8102082|ref|Os09g23290|
gi|8102086|ref|Os09g23330|
gi|8102087|ref|Os09g23340|
gi|8102093|ref|Os09g23390|
gi|8102095|ref|Os09g23410|
gi|8102096|ref|Os09g23420|
gi|8102097|ref|Os09g23430|
gi|8102098|ref|Os09g23440|
gi|8102099|ref|Os09g23450|
gi|8102100|ref|Os09g23460|
gi|8102101|ref|Os09g23470|
gi|8102104|ref|Os09g23500|
gi|8102105|ref|Os09g23510|
gi|8102106|ref|Os09g23520|
gi|8102113|ref|Os09g23580|
gi|8102114|ref|Os09g23590|
gi|8102116|ref|Os09g23610|
gi|8102118|ref|Os09g23630|
gi|8102123|ref|Os09g23680|
gi|8102125|ref|Os09g23700|
gi|8102126|ref|Os09g23710|
gi|8102130|ref|Os09g23750|
gi|8102131|ref|Os09g23760|
gi|8102132|ref|Os09g23770|
gi|8102143|ref|Os09g23880|
gi|8102144|ref|Os09g23890|
gi|8102145|ref|Os09g23900|
gi|8102146|ref|Os09g23910|
gi|8102148|ref|Os09g23930|
gi|8102149|ref|Os09g23940|
gi|8102150|ref|Os09g23950|
gi|8102151|ref|Os09g23960|
gi|8102152|ref|Os09g23970|
gi|8102154|ref|Os09g23990|
gi|8102155|ref|Os09g24000|
gi|8102156|ref|Os09g24010|
gi|8102157|ref|Os09g24020|
gi|8102158|ref|Os09g24030|
gi|8102159|ref|Os09g24040|
gi|8102162|ref|Os09g24070|
gi|8102165|ref|Os09g24100|
gi|8102166|ref|Os09g24110|
gi|8102167|ref|Os09g24120|
gi|8102168|ref|Os09g24130|
gi|8102169|ref|Os09g24140|
gi|8102170|ref|Os09g24150|
gi|8102171|ref|Os09g24160|
gi|8102172|ref|Os09g24170|
gi|8102174|ref|Os09g24190|
gi|8102184|ref|Os09g24280|
gi|8102186|ref|Os09g24300|
gi|8102191|ref|Os09g24350|
gi|8102193|ref|Os09g24370|
gi|8102195|ref|Os09g24390|
gi|8102196|ref|Os09g24400|
gi|8102199|ref|Os09g24430|
gi|8102202|ref|Os09g24460|
gi|8102206|ref|Os09g24500|
gi|8102212|ref|Os09g24550|
gi|8102216|ref|Os09g24590|
gi|8102218|ref|Os09g24610|
gi|8102219|ref|Os09g24620|
gi|8102220|ref|Os09g24630|
gi|8102230|ref|Os09g24720|
gi|8102231|ref|Os09g24730|
gi|8102241|ref|Os09g24830|
gi|8102244|ref|Os09g24860|
gi|8102245|ref|Os09g24870|
gi|8102247|ref|Os09g24890|
gi|8102253|ref|Os09g24940|
gi|8102254|ref|Os09g24950|
gi|8102260|ref|Os09g25010|
gi|8102261|ref|Os09g25020|
gi|8102262|ref|Os09g25030|
gi|8102268|ref|Os09g25080|
gi|8102273|ref|Os09g25130|
gi|8102280|ref|Os09g25180|
gi|8102283|ref|Os09g25210|
gi|8102290|ref|Os09g25270|
gi|8102298|ref|Os09g25350|
gi|8102304|ref|Os09g25410|
gi|8102305|ref|Os09g25410|
gi|8102309|ref|Os09g25450|
gi|8102311|ref|Os09g25470|
gi|8102312|ref|Os09g25480|
gi|8102317|ref|Os09g25530|
gi|8102328|ref|Os09g25630|
gi|8102334|ref|Os09g25690|
gi|8102335|ref|Os09g25700|
gi|8102336|ref|Os09g25710|
gi|8102337|ref|Os09g25720|
gi|8102339|ref|Os09g25740|
gi|8102340|ref|Os09g25750|
gi|8102349|ref|Os09g25820|
gi|8102350|ref|Os09g25830|
gi|8102351|ref|Os09g25840|
gi|8102355|ref|Os09g25880|
gi|8102364|ref|Os09g25970|
gi|8102368|ref|Os09g26010|
gi|8102371|ref|Os09g26040|
gi|8102373|ref|Os09g26060|

gi|8102374|ref|Os09g26070|
gi|8102378|ref|Os09g26110|
gi|8102394|ref|Os09g26270|
gi|8102399|ref|Os09g26320|
gi|8102402|ref|Os09g26350|
gi|8102408|ref|Os09g26410|
gi|8102413|ref|Os09g26450|
gi|8102419|ref|Os09g26510|
gi|8102420|ref|Os09g26520|
gi|8102421|ref|Os09g26530|
gi|8102426|ref|Os09g26550|
gi|8102427|ref|Os09g26550|
gi|8102432|ref|Os09g26600|
gi|8102439|ref|Os09g26670|
gi|8102443|ref|Os09g26710|
gi|8102446|ref|Os09g26740|
gi|8102447|ref|Os09g26750|
gi|8102451|ref|Os09g26790|
gi|8102456|ref|Os09g26840|
gi|8102457|ref|Os09g26850|
gi|8102465|ref|Os09g26890|
gi|8102467|ref|Os09g26910|
gi|8102469|ref|Os09g26930|
gi|8102476|ref|Os09g27000|
gi|8102478|ref|Os09g27020|
gi|8102490|ref|Os09g27120|
gi|8102494|ref|Os09g27160|
gi|8102495|ref|Os09g27170|
gi|8102498|ref|Os09g27200|
gi|8102506|ref|Os09g27280|
gi|8102507|ref|Os09g27290|
gi|8102509|ref|Os09g27310|
gi|8102514|ref|Os09g27360|
gi|8102518|ref|Os09g27400|
gi|8102519|ref|Os09g27410|
gi|8102521|ref|Os09g27430|
gi|8102522|ref|Os09g27440|
gi|8102524|ref|Os09g27460|
gi|8102525|ref|Os09g27470|
gi|8102526|ref|Os09g27480|
gi|8102527|ref|Os09g27490|
gi|8102530|ref|Os09g27520|
gi|8102531|ref|Os09g27530|
gi|8102533|ref|Os09g27550|
gi|8102534|ref|Os09g27560|
gi|8102541|ref|Os09g27630|
gi|8102544|ref|Os09g27660|
gi|8102552|ref|Os09g27670|
gi|8102553|ref|Os09g27680|
gi|8102558|ref|Os09g27720|
gi|8102560|ref|Os09g27740|
gi|8102574|ref|Os09g27870|
gi|8102575|ref|Os09g27880|
gi|8102579|ref|Os09g27920|
gi|8102585|ref|Os09g27970|
gi|8102599|ref|Os09g28090|
gi|8102607|ref|Os09g28170|
gi|8102609|ref|Os09g28190|
gi|8102614|ref|Os09g28240|
gi|8102615|ref|Os09g28250|
gi|8102617|ref|Os09g28270|
gi|8102619|ref|Os09g28290|
gi|8102622|ref|Os09g28320|
gi|8102628|ref|Os09g28380|
gi|8102631|ref|Os09g28410|
gi|8102642|ref|Os09g28520|
gi|8102643|ref|Os09g28530|
gi|8102644|ref|Os09g28540|
gi|8102645|ref|Os09g28550|
gi|8102650|ref|Os09g28600|
gi|8102657|ref|Os09g28670|
gi|8102660|ref|Os09g28700|
gi|8102661|ref|Os09g28710|
gi|8102668|ref|Os09g28780|
gi|8102670|ref|Os09g28800|
gi|8102672|ref|Os09g28820|
gi|8102686|ref|Os09g28920|
gi|8102689|ref|Os09g28950|
gi|8102692|ref|Os09g28980|
gi|8102697|ref|Os09g29030|
gi|8102698|ref|Os09g29040|
gi|8102706|ref|Os09g29110|
gi|8102709|ref|Os09g29140|
gi|8102710|ref|Os09g29150|
gi|8102715|ref|Os09g29180|
gi|8102722|ref|Os09g29250|
gi|8102724|ref|Os09g29270|
gi|8102727|ref|Os09g29300|
gi|8102732|ref|Os09g29350|
gi|8102735|ref|Os09g29380|
gi|8102737|ref|Os09g29400|
gi|8102741|ref|Os09g29440|
gi|8102751|ref|Os09g29530|
gi|8102753|ref|Os09g29550|
gi|8102755|ref|Os09g29570|
gi|8102757|ref|Os09g29590|
gi|8102759|ref|Os09g29610|
gi|8102762|ref|Os09g29640|
gi|8102763|ref|Os09g29650|
gi|8102775|ref|Os09g29770|
gi|8102776|ref|Os09g29780|
gi|8102778|ref|Os09g29800|
gi|8102785|ref|Os09g29860|
gi|8102786|ref|Os09g29870|
gi|8102787|ref|Os09g29880|
gi|8102790|ref|Os09g29900|
gi|8102791|ref|Os09g29910|
gi|8102800|ref|Os09g29990|
gi|8102801|ref|Os09g30000|
gi|8102804|ref|Os09g30030|
gi|8102805|ref|Os09g30040|
gi|8102806|ref|Os09g30050|
gi|8102807|ref|Os09g30060|
gi|8102808|ref|Os09g30070|
gi|8102818|ref|Os09g30170|
gi|8102827|ref|Os09g30260|
gi|8102830|ref|Os09g30290|
gi|8102831|ref|Os09g30300|
gi|8102836|ref|Os09g30350|
gi|8102850|ref|Os09g30970|
gi|8102861|ref|Os09g31060|
gi|8102862|ref|Os09g31070|
gi|8102863|ref|Os09g31080|
gi|8102864|ref|Os09g31080|
gi|8102865|ref|Os09g31080|
gi|8102868|ref|Os09g31100|
gi|8102875|ref|Os09g31170|
gi|8102876|ref|Os09g31170|
gi|8102882|ref|Os09g31220|
gi|8102883|ref|Os09g31220|
gi|8102884|ref|Os09g31220|
gi|8102887|ref|Os09g31250|
gi|8102891|ref|Os09g31290|
gi|8102896|ref|Os09g31330|

gi|8102897|ref|Os09g31340|
gi|8102906|ref|Os09g31420|
gi|8102910|ref|Os09g31940|
gi|8102915|ref|Os09g31990|
gi|8102923|ref|Os09g32060|
gi|8102924|ref|Os09g32070|
gi|8102928|ref|Os09g32110|
gi|8102929|ref|Os09g32120|
gi|8102930|ref|Os09g32130|
gi|8102931|ref|Os09g32140|
gi|8102932|ref|Os09g32150|
gi|8102933|ref|Os09g32160|
gi|8102934|ref|Os09g32170|
gi|8102953|ref|Os09g32330|
gi|8102954|ref|Os09g32340|
gi|8102955|ref|Os09g32350|
gi|8102960|ref|Os09g32400|
gi|8102962|ref|Os09g32420|
gi|8102968|ref|Os09g32480|
gi|8102976|ref|Os09g32540|
gi|8102981|ref|Os09g32590|
gi|8102991|ref|Os09g32690|
gi|8103001|ref|Os09g32790|
gi|8103010|ref|Os09g32850|
gi|8103013|ref|Os09g32880|
gi|8103014|ref|Os09g32890|
gi|8103017|ref|Os09g32920|
gi|8103033|ref|Os09g33540|
gi|8103036|ref|Os09g33560|
gi|8103044|ref|Os09g33640|
gi|8103045|ref|Os09g33650|
gi|8103046|ref|Os09g33660|
gi|8103051|ref|Os09g33700|
gi|8103054|ref|Os09g33730|
gi|8103057|ref|Os09g33760|
gi|8103065|ref|Os09g33840|
gi|8103070|ref|Os09g33890|
gi|8103077|ref|Os09g33960|
gi|8103083|ref|Os09g34020|
gi|8103084|ref|Os09g34030|
gi|8103086|ref|Os09g34050|
gi|8103093|ref|Os09g34120|
gi|8103098|ref|Os09g34170|
gi|8103105|ref|Os09g34240|
gi|8103107|ref|Os09g34260|
gi|8103118|ref|Os09g34340|
gi|8103126|ref|Os09g34890|
gi|8103131|ref|Os09g34940|
gi|8103138|ref|Os09g35010|
gi|8103142|ref|Os09g35540|
gi|8103143|ref|Os09g35550|
gi|8103144|ref|Os09g35560|
gi|8103145|ref|Os09g35570|
gi|8103147|ref|Os09g35590|
gi|8103150|ref|Os09g35620|
gi|8103152|ref|Os09g35640|
gi|8103163|ref|Os09g35740|
gi|8103164|ref|Os09g35750|
gi|8103167|ref|Os09g35780|
gi|8103172|ref|Os09g35820|
gi|8103173|ref|Os09g35830|
gi|8103180|ref|Os09g35900|
gi|8103185|ref|Os09g35930|
gi|8103190|ref|Os09g35980|
gi|8103191|ref|Os09g35980|
gi|8103193|ref|Os09g36000|
gi|8103194|ref|Os09g36010|
gi|8103198|ref|Os09g36050|
gi|8103210|ref|Os09g36170|
gi|8103220|ref|Os09g36260|
gi|8103225|ref|Os09g36310|
gi|8103227|ref|Os09g36330|
gi|8103232|ref|Os09g36380|
gi|8103233|ref|Os09g36390|
gi|8103234|ref|Os09g36400|
gi|8103237|ref|Os09g36430|
gi|8103241|ref|Os09g36470|
gi|8103243|ref|Os09g36490|
gi|8103245|ref|Os09g36510|
gi|8103257|ref|Os09g36620|
gi|8103258|ref|Os09g36630|
gi|8103259|ref|Os09g36640|
gi|8103260|ref|Os09g36650|
gi|8103261|ref|Os09g36660|
gi|8103267|ref|Os09g36720|
gi|8103273|ref|Os09g36780|
gi|8103274|ref|Os09g36790|
gi|8103279|ref|Os09g36840|
gi|8103280|ref|Os09g36850|
gi|8103287|ref|Os09g36910|
gi|8103291|ref|Os09g36950|
gi|8103292|ref|Os09g36960|
gi|8103293|ref|Os09g36970|
gi|8103304|ref|Os09g37080|
gi|8103305|ref|Os09g37090|
gi|8103307|ref|Os09g37110|
gi|8103309|ref|Os09g37130|
gi|8103310|ref|Os09g37140|
gi|8103322|ref|Os09g37260|
gi|8103327|ref|Os09g37290|
gi|8103332|ref|Os09g37340|
gi|8103335|ref|Os09g37370|
gi|8103343|ref|Os09g37450|
gi|8103351|ref|Os09g37530|
gi|8103354|ref|Os09g37560|
gi|8103365|ref|Os09g37670|
gi|8103366|ref|Os09g37680|
gi|8103373|ref|Os09g37750|
gi|8103375|ref|Os09g37770|
gi|8103381|ref|Os09g37830|
gi|8103385|ref|Os09g37870|
gi|8103388|ref|Os09g37900|
gi|8103392|ref|Os09g37940|
gi|8103397|ref|Os09g37990|
gi|8103403|ref|Os09g38050|
gi|8103410|ref|Os09g38120|
gi|8103414|ref|Os09g38150|
gi|8103415|ref|Os09g38160|
gi|8103421|ref|Os09g38220|
gi|8103426|ref|Os09g38270|
gi|8103435|ref|Os09g38360|
gi|8103439|ref|Os09g38400|
gi|8103442|ref|Os09g38430|
gi|8103444|ref|Os09g38450|
gi|8103445|ref|Os09g38460|
gi|8103446|ref|Os09g38470|
gi|8103447|ref|Os09g38480|
gi|8103448|ref|Os09g38490|
gi|8103460|ref|Os09g38590|
gi|8103461|ref|Os09g38600|
gi|8103466|ref|Os09g38650|
gi|8103469|ref|Os09g38680|
gi|8103487|ref|Os09g38860|
gi|8103488|ref|Os09g38870|

gi|8103489|ref|Os09g38880|
gi|8103493|ref|Os09g38920|
gi|8103503|ref|Os09g38990|
gi|8103505|ref|Os09g39010|
gi|8103507|ref|Os09g39030|
gi|8103508|ref|Os09g39040|
gi|8103512|ref|Os09g39080|
gi|8103517|ref|Os09g39130|
gi|8103528|ref|Os09g39240|
gi|8103529|ref|Os09g39250|
gi|8103532|ref|Os09g39280|
gi|8103535|ref|Os09g39310|
gi|8103538|ref|Os09g39340|
gi|8103539|ref|Os09g39350|
gi|8103540|ref|Os09g39360|
gi|8103550|ref|Os09g39450|
gi|8103553|ref|Os09g39480|
gi|8103565|ref|Os09g39600|
gi|8103566|ref|Os09g39610|
gi|8103587|ref|Os09g39800|
gi|8103595|ref|Os09g39880|
gi|8200001|ref|Os05g01010|
gi|8200007|ref|Os05g01070|
gi|8200008|ref|Os05g01080|
gi|8200011|ref|Os05g01100|
gi|8200025|ref|Os05g01210|
gi|8200026|ref|Os05g01220|
gi|8200039|ref|Os05g01340|
gi|8200066|ref|Os05g01540|
gi|8200085|ref|Os05g01720|
gi|8200092|ref|Os05g01770|
gi|8200099|ref|Os05g01830|
gi|8200100|ref|Os05g01840|
gi|8200102|ref|Os05g01860|
gi|8200103|ref|Os05g01870|
gi|8200104|ref|Os05g01880|
gi|8200105|ref|Os05g01890|
gi|8200106|ref|Os05g01900|
gi|8200109|ref|Os05g01930|
gi|8200112|ref|Os05g01960|
gi|8200125|ref|Os05g02070|
gi|8200129|ref|Os05g02100|
gi|8200135|ref|Os05g02160|
gi|8200136|ref|Os05g02170|
gi|8200137|ref|Os05g02180|
gi|8200143|ref|Os05g02230|
gi|8200146|ref|Os05g02260|
gi|8200147|ref|Os05g02260|
gi|8200148|ref|Os05g02260|
gi|8200151|ref|Os05g02290|
gi|8200158|ref|Os05g02360|
gi|8200159|ref|Os05g02370|
gi|8200165|ref|Os05g02430|
gi|8200166|ref|Os05g02440|
gi|8200169|ref|Os05g02470|
gi|8200176|ref|Os05g02540|
gi|8200178|ref|Os05g02560|
gi|8200182|ref|Os05g02600|
gi|8200183|ref|Os05g02610|
gi|8200184|ref|Os05g02620|
gi|8200185|ref|Os05g02630|
gi|8200192|ref|Os05g02700|
gi|8200194|ref|Os05g02720|
gi|8200198|ref|Os05g02760|
gi|8200199|ref|Os05g02770|
gi|8200200|ref|Os05g02780|
gi|8200201|ref|Os05g02780|
gi|8200206|ref|Os05g02830|
gi|8200207|ref|Os05g02840|
gi|8200215|ref|Os05g02900|
gi|8200216|ref|Os05g02910|
gi|8200217|ref|Os05g02920|
gi|8200222|ref|Os05g02970|
gi|8200223|ref|Os05g02980|
gi|8200226|ref|Os05g03010|
gi|8200242|ref|Os05g03150|
gi|8200249|ref|Os05g03220|
gi|8200250|ref|Os05g03230|
gi|8200251|ref|Os05g03240|
gi|8200252|ref|Os05g03250|
gi|8200253|ref|Os05g03260|
gi|8200257|ref|Os05g03300|
gi|8200258|ref|Os05g03310|
gi|8200260|ref|Os05g03330|
gi|8200261|ref|Os05g03340|
gi|8200265|ref|Os05g03380|
gi|8200270|ref|Os05g03420|
gi|8200273|ref|Os05g03440|
gi|8200279|ref|Os05g03490|
gi|8200281|ref|Os05g03510|
gi|8200282|ref|Os05g03520|
gi|8200286|ref|Os05g03560|
gi|8200287|ref|Os05g03570|
gi|8200288|ref|Os05g03580|
gi|8200289|ref|Os05g03590|
gi|8200300|ref|Os05g03700|
gi|8200303|ref|Os05g03730|
gi|8200305|ref|Os05g03750|
gi|8200307|ref|Os05g03770|
gi|8200309|ref|Os05g03790|
gi|8200320|ref|Os05g03870|
gi|8200326|ref|Os05g03930|
gi|8200329|ref|Os05g03960|
gi|8200333|ref|Os05g04000|
gi|8200334|ref|Os05g04010|
gi|8200338|ref|Os05g04050|
gi|8200343|ref|Os05g04100|
gi|8200344|ref|Os05g04110|
gi|8200346|ref|Os05g04130|
gi|8200347|ref|Os05g04140|
gi|8200355|ref|Os05g04200|
gi|8200360|ref|Os05g04250|
gi|8200362|ref|Os05g04270|
gi|8200366|ref|Os05g04310|
gi|8200374|ref|Os05g04390|
gi|8200390|ref|Os05g04540|
gi|8200392|ref|Os05g04560|
gi|8200393|ref|Os05g04570|
gi|8200406|ref|Os05g04650|
gi|8200408|ref|Os05g04670|
gi|8200409|ref|Os05g04680|
gi|8200411|ref|Os05g04700|
gi|8200413|ref|Os05g04720|
gi|8200417|ref|Os05g04760|
gi|8200419|ref|Os05g04780|
gi|8200421|ref|Os05g04800|
gi|8200422|ref|Os05g04810|
gi|8200424|ref|Os05g04830|
gi|8200425|ref|Os05g04840|
gi|8200429|ref|Os05g04880|
gi|8200430|ref|Os05g04890|
gi|8200444|ref|Os05g05020|
gi|8200445|ref|Os05g05030|
gi|8200446|ref|Os05g05040|

gi|8200448|ref|Os05g05060|
gi|8200449|ref|Os05g05070|
gi|8200450|ref|Os05g05080|
gi|8200452|ref|Os05g05100|
gi|8200453|ref|Os05g05110|
gi|8200454|ref|Os05g05120|
gi|8200461|ref|Os05g05190|
gi|8200472|ref|Os05g05290|
gi|8200477|ref|Os05g05340|
gi|8200481|ref|Os05g05380|
gi|8200484|ref|Os05g05410|
gi|8200486|ref|Os05g05430|
gi|8200493|ref|Os05g05500|
gi|8200494|ref|Os05g05510|
gi|8200498|ref|Os05g05550|
gi|8200500|ref|Os05g05570|
gi|8200505|ref|Os05g05610|
gi|8200508|ref|Os05g05640|
gi|8200522|ref|Os05g05750|
gi|8200523|ref|Os05g05760|
gi|8200524|ref|Os05g05770|
gi|8200533|ref|Os05g05850|
gi|8200541|ref|Os05g05930|
gi|8200544|ref|Os05g05960|
gi|8200548|ref|Os05g06000|
gi|8200549|ref|Os05g06010|
gi|8200550|ref|Os05g06020|
gi|8200551|ref|Os05g06030|
gi|8200552|ref|Os05g06040|
gi|8200557|ref|Os05g06090|
gi|8200566|ref|Os05g06180|
gi|8200570|ref|Os05g06220|
gi|8200571|ref|Os05g06230|
gi|8200575|ref|Os05g06270|
gi|8200588|ref|Os05g06370|
gi|8200591|ref|Os05g06400|
gi|8200592|ref|Os05g06410|
gi|8200603|ref|Os05g06520|
gi|8200604|ref|Os05g06530|
gi|8200605|ref|Os05g06540|
gi|8200606|ref|Os05g06550|
gi|8200611|ref|Os05g06600|
gi|8200613|ref|Os05g06620|
gi|8200614|ref|Os05g06630|
gi|8200619|ref|Os05g06680|
gi|8200625|ref|Os05g06730|
gi|8200631|ref|Os05g06790|
gi|8200632|ref|Os05g06800|
gi|8200635|ref|Os05g06820|
gi|8200636|ref|Os05g06830|
gi|8200646|ref|Os05g06930|
gi|8200648|ref|Os05g06950|
gi|8200649|ref|Os05g06960|
gi|8200655|ref|Os05g07020|
gi|8200675|ref|Os05g07150|
gi|8200677|ref|Os05g07170|
gi|8200680|ref|Os05g07200|
gi|8200683|ref|Os05g07230|
gi|8200686|ref|Os05g07260|
gi|8200688|ref|Os05g07260|
gi|8200694|ref|Os05g07320|
gi|8200698|ref|Os05g07360|
gi|8200705|ref|Os05g07430|
gi|8200706|ref|Os05g07440|
gi|8200708|ref|Os05g07460|
gi|8200710|ref|Os05g07480|
gi|8200713|ref|Os05g07510|
gi|8200715|ref|Os05g07530|
gi|8200718|ref|Os05g07560|
gi|8200720|ref|Os05g07580|
gi|8200722|ref|Os05g07600|
gi|8200723|ref|Os05g07610|
gi|8200724|ref|Os05g07620|
gi|8200728|ref|Os05g07660|
gi|8200749|ref|Os05g07840|
gi|8200756|ref|Os05g07910|
gi|8200758|ref|Os05g07930|
gi|8200761|ref|Os05g07960|
gi|8200762|ref|Os05g07970|
gi|8200764|ref|Os05g07990|
gi|8200765|ref|Os05g08000|
gi|8200766|ref|Os05g08010|
gi|8200768|ref|Os05g08030|
gi|8200769|ref|Os05g08040|
gi|8200774|ref|Os05g08090|
gi|8200776|ref|Os05g08110|
gi|8200786|ref|Os05g08210|
gi|8200788|ref|Os05g08230|
gi|8200789|ref|Os05g08240|
gi|8200797|ref|Os05g08320|
gi|8200798|ref|Os05g08330|
gi|8200799|ref|Os05g08340|
gi|8200800|ref|Os05g08350|
gi|8200803|ref|Os05g08380|
gi|8200808|ref|Os05g08420|
gi|8200812|ref|Os05g08450|
gi|8200814|ref|Os05g08470|
gi|8200822|ref|Os05g08550|
gi|8200828|ref|Os05g08580|
gi|8200835|ref|Os05g08630|
gi|8200840|ref|Os05g08680|
gi|8200843|ref|Os05g08710|
gi|8200845|ref|Os05g08730|
gi|8200848|ref|Os05g08760|
gi|8200850|ref|Os05g08780|
gi|8200855|ref|Os05g08830|
gi|8200858|ref|Os05g08860|
gi|8200862|ref|Os05g08900|
gi|8200863|ref|Os05g08910|
gi|8200872|ref|Os05g09000|
gi|8200873|ref|Os05g09000|
gi|8200879|ref|Os05g09060|
gi|8200885|ref|Os05g09120|
gi|8200889|ref|Os05g09160|
gi|8200892|ref|Os05g09190|
gi|8200893|ref|Os05g09200|
gi|8200902|ref|Os05g09290|
gi|8200904|ref|Os05g09310|
gi|8200908|ref|Os05g09350|
gi|8200913|ref|Os05g09400|
gi|8200915|ref|Os05g09420|
gi|8200916|ref|Os05g09430|
gi|8200918|ref|Os05g09450|
gi|8200919|ref|Os05g09460|
gi|8200920|ref|Os05g09470|
gi|8200926|ref|Os05g09510|
gi|8200927|ref|Os05g09520|
gi|8200929|ref|Os05g09540|
gi|8200931|ref|Os05g09560|
gi|8200933|ref|Os05g09580|
gi|8200934|ref|Os05g09590|
gi|8200935|ref|Os05g09600|
gi|8200936|ref|Os05g09610|
gi|8200943|ref|Os05g09670|

gi|8200946|ref|Os05g09700|
gi|8200947|ref|Os05g09710|
gi|8200956|ref|Os05g10290|
gi|8200957|ref|Os05g10300|
gi|8200959|ref|Os05g10320|
gi|8200962|ref|Os05g10350|
gi|8200963|ref|Os05g10360|
gi|8200969|ref|Os05g10420|
gi|8200970|ref|Os05g10430|
gi|8200972|ref|Os05g10450|
gi|8200975|ref|Os05g10480|
gi|8200976|ref|Os05g10490|
gi|8200977|ref|Os05g10500|
gi|8200982|ref|Os05g10550|
gi|8200983|ref|Os05g10560|
gi|8200984|ref|Os05g10570|
gi|8200985|ref|Os05g10570|
gi|8200987|ref|Os05g10590|
gi|8200988|ref|Os05g10600|
gi|8200989|ref|Os05g10610|
gi|8200997|ref|Os05g10680|
gi|8201001|ref|Os05g10720|
gi|8201006|ref|Os05g10750|
gi|8201010|ref|Os05g10790|
gi|8201011|ref|Os05g10800|
gi|8201013|ref|Os05g10820|
gi|8201016|ref|Os05g10850|
gi|8201017|ref|Os05g10860|
gi|8201022|ref|Os05g10910|
gi|8201028|ref|Os05g10970|
gi|8201030|ref|Os05g10990|
gi|8201031|ref|Os05g11000|
gi|8201033|ref|Os05g11020|
gi|8201043|ref|Os05g11100|
gi|8201048|ref|Os05g11150|
gi|8201049|ref|Os05g11160|
gi|8201050|ref|Os05g11170|
gi|8201051|ref|Os05g11180|
gi|8201053|ref|Os05g11200|
gi|8201055|ref|Os05g11220|
gi|8201056|ref|Os05g11230|
gi|8201057|ref|Os05g11240|
gi|8201058|ref|Os05g11250|
gi|8201059|ref|Os05g11260|
gi|8201064|ref|Os05g11310|
gi|8201067|ref|Os05g11340|
gi|8201068|ref|Os05g11350|
gi|8201072|ref|Os05g11390|
gi|8201077|ref|Os05g11430|
gi|8201078|ref|Os05g11440|
gi|8201080|ref|Os05g11460|
gi|8201083|ref|Os05g11490|
gi|8201084|ref|Os05g11500|
gi|8201087|ref|Os05g11530|
gi|8201088|ref|Os05g11540|
gi|8201093|ref|Os05g11580|
gi|8201094|ref|Os05g11590|
gi|8201095|ref|Os05g11600|
gi|8201096|ref|Os05g11610|
gi|8201097|ref|Os05g11620|
gi|8201099|ref|Os05g11640|
gi|8201101|ref|Os05g11660|
gi|8201109|ref|Os05g11740|
gi|8201111|ref|Os05g11760|
gi|8201115|ref|Os05g11800|
gi|8201119|ref|Os05g11840|
gi|8201123|ref|Os05g11880|
gi|8201124|ref|Os05g11890|
gi|8201125|ref|Os05g11900|
gi|8201127|ref|Os05g11920|
gi|8201128|ref|Os05g11930|
gi|8201131|ref|Os05g11960|
gi|8201136|ref|Os05g12000|
gi|8201137|ref|Os05g12010|
gi|8201138|ref|Os05g12020|
gi|8201139|ref|Os05g12030|
gi|8201141|ref|Os05g12050|
gi|8201143|ref|Os05g12070|
gi|8201144|ref|Os05g12080|
gi|8201146|ref|Os05g12100|
gi|8201147|ref|Os05g12110|
gi|8201148|ref|Os05g12120|
gi|8201152|ref|Os05g12160|
gi|8201156|ref|Os05g12200|
gi|8201158|ref|Os05g12220|
gi|8201159|ref|Os05g12230|
gi|8201161|ref|Os05g12250|
gi|8201163|ref|Os05g12270|
gi|8201164|ref|Os05g12280|
gi|8201166|ref|Os05g12300|
gi|8201167|ref|Os05g12300|
gi|8201174|ref|Os05g12370|
gi|8201176|ref|Os05g12390|
gi|8201179|ref|Os05g12420|
gi|8201180|ref|Os05g12430|
gi|8201181|ref|Os05g12440|
gi|8201183|ref|Os05g12460|
gi|8201184|ref|Os05g12470|
gi|8201187|ref|Os05g12500|
gi|8201188|ref|Os05g12510|
gi|8201191|ref|Os05g12540|
gi|8201193|ref|Os05g12560|
gi|8201196|ref|Os05g12590|
gi|8201197|ref|Os05g12600|
gi|8201198|ref|Os05g12610|
gi|8201202|ref|Os05g12640|
gi|8201205|ref|Os05g12660|
gi|8201206|ref|Os05g12670|
gi|8201208|ref|Os05g12690|
gi|8201209|ref|Os05g12700|
gi|8201210|ref|Os05g12710|
gi|8201211|ref|Os05g12720|
gi|8201212|ref|Os05g12730|
gi|8201218|ref|Os05g12790|
gi|8201219|ref|Os05g12800|
gi|8201221|ref|Os05g13310|
gi|8201225|ref|Os05g13350|
gi|8201226|ref|Os05g13360|
gi|8201230|ref|Os05g13400|
gi|8201231|ref|Os05g13410|
gi|8201233|ref|Os05g13430|
gi|8201237|ref|Os05g13470|
gi|8201240|ref|Os05g13500|
gi|8201243|ref|Os05g13530|
gi|8201244|ref|Os05g13540|
gi|8201245|ref|Os05g13550|
gi|8201246|ref|Os05g13560|
gi|8201250|ref|Os05g13600|
gi|8201254|ref|Os05g13640|
gi|8201257|ref|Os05g13670|
gi|8201258|ref|Os05g13680|
gi|8201259|ref|Os05g13690|
gi|8201266|ref|Os05g13760|
gi|8201269|ref|Os05g13790|

gi|8201271|ref|Os05g13810|
gi|8201272|ref|Os05g13820|
gi|8201273|ref|Os05g13830|
gi|8201274|ref|Os05g13840|
gi|8201275|ref|Os05g13850|
gi|8201276|ref|Os05g13860|
gi|8201277|ref|Os05g13870|
gi|8201278|ref|Os05g13880|
gi|8201279|ref|Os05g13890|
gi|8201280|ref|Os05g13900|
gi|8201281|ref|Os05g13910|
gi|8201283|ref|Os05g13930|
gi|8201284|ref|Os05g13940|
gi|8201288|ref|Os05g13980|
gi|8201293|ref|Os05g14030|
gi|8201295|ref|Os05g14050|
gi|8201296|ref|Os05g14060|
gi|8201297|ref|Os05g14070|
gi|8201303|ref|Os05g14130|
gi|8201304|ref|Os05g14140|
gi|8201306|ref|Os05g14160|
gi|8201311|ref|Os05g14210|
gi|8201313|ref|Os05g14230|
gi|8201315|ref|Os05g14250|
gi|8201317|ref|Os05g14270|
gi|8201319|ref|Os05g14290|
gi|8201321|ref|Os05g14310|
gi|8201326|ref|Os05g14360|
gi|8201329|ref|Os05g14390|
gi|8201333|ref|Os05g14430|
gi|8201337|ref|Os05g14470|
gi|8201338|ref|Os05g14480|
gi|8201339|ref|Os05g14490|
gi|8201344|ref|Os05g14540|
gi|8201352|ref|Os05g14620|
gi|8201355|ref|Os05g14650|
gi|8201356|ref|Os05g14660|
gi|8201359|ref|Os05g14690|
gi|8201360|ref|Os05g14700|
gi|8201361|ref|Os05g14710|
gi|8201362|ref|Os05g14720|
gi|8201366|ref|Os05g14760|
gi|8201367|ref|Os05g14770|
gi|8201373|ref|Os05g14830|
gi|8201374|ref|Os05g14840|
gi|8201377|ref|Os05g14870|
gi|8201379|ref|Os05g14890|
gi|8201381|ref|Os05g14910|
gi|8201382|ref|Os05g14920|
gi|8201383|ref|Os05g14930|
gi|8201385|ref|Os05g14950|
gi|8201387|ref|Os05g14970|
gi|8201389|ref|Os05g14990|
gi|8201390|ref|Os05g15000|
gi|8201391|ref|Os05g15010|
gi|8201392|ref|Os05g15020|
gi|8201397|ref|Os05g15050|
gi|8201398|ref|Os05g15060|
gi|8201399|ref|Os05g15070|
gi|8201400|ref|Os05g15080|
gi|8201402|ref|Os05g15100|
gi|8201403|ref|Os05g15110|
gi|8201404|ref|Os05g15120|
gi|8201420|ref|Os05g15280|
gi|8201422|ref|Os05g15300|
gi|8201423|ref|Os05g15310|
gi|8201425|ref|Os05g15330|
gi|8201427|ref|Os05g15350|
gi|8201431|ref|Os05g15390|
gi|8201441|ref|Os05g15490|
gi|8201442|ref|Os05g15500|
gi|8201453|ref|Os05g15610|
gi|8201454|ref|Os05g15620|
gi|8201457|ref|Os05g15650|
gi|8201459|ref|Os05g15670|
gi|8201460|ref|Os05g15680|
gi|8201462|ref|Os05g15700|
gi|8201463|ref|Os05g15710|
gi|8201466|ref|Os05g15740|
gi|8201468|ref|Os05g15760|
gi|8201470|ref|Os05g15780|
gi|8201471|ref|Os05g15790|
gi|8201472|ref|Os05g15800|
gi|8201479|ref|Os05g15870|
gi|8201482|ref|Os05g15900|
gi|8201483|ref|Os05g15910|
gi|8201492|ref|Os05g16000|
gi|8201495|ref|Os05g16030|
gi|8201496|ref|Os05g16040|
gi|8201497|ref|Os05g16050|
gi|8201498|ref|Os05g16060|
gi|8201499|ref|Os05g16070|
gi|8201501|ref|Os05g16090|
gi|8201502|ref|Os05g16100|
gi|8201503|ref|Os05g16110|
gi|8201506|ref|Os05g16140|
gi|8201507|ref|Os05g16150|
gi|8201508|ref|Os05g16160|
gi|8201513|ref|Os05g16210|
gi|8201514|ref|Os05g16220|
gi|8201515|ref|Os05g16230|
gi|8201516|ref|Os05g16240|
gi|8201524|ref|Os05g16320|
gi|8201530|ref|Os05g16380|
gi|8201536|ref|Os05g16440|
gi|8201537|ref|Os05g16450|
gi|8201538|ref|Os05g16460|
gi|8201539|ref|Os05g16470|
gi|8201540|ref|Os05g16480|
gi|8201541|ref|Os05g16490|
gi|8201543|ref|Os05g16510|
gi|8201544|ref|Os05g16520|
gi|8201545|ref|Os05g16530|
gi|8201546|ref|Os05g16540|
gi|8201551|ref|Os05g16590|
gi|8201563|ref|Os05g16710|
gi|8201567|ref|Os05g16750|
gi|8201569|ref|Os05g16770|
gi|8201573|ref|Os05g16810|
gi|8201576|ref|Os05g16840|
gi|8201578|ref|Os05g16860|
gi|8201579|ref|Os05g16870|
gi|8201581|ref|Os05g16890|
gi|8201582|ref|Os05g16900|
gi|8201589|ref|Os05g16970|
gi|8201591|ref|Os05g16990|
gi|8201594|ref|Os05g17020|
gi|8201598|ref|Os05g17060|
gi|8201599|ref|Os05g17070|
gi|8201600|ref|Os05g17080|
gi|8201612|ref|Os05g17200|
gi|8201613|ref|Os05g17210|
gi|8201614|ref|Os05g17220|
gi|8201615|ref|Os05g17230|

gi|8201616|ref|Os05g17240|
gi|8201617|ref|Os05g17250|
gi|8201622|ref|Os05g17300|
gi|8201623|ref|Os05g17310|
gi|8201629|ref|Os05g17370|
gi|8201630|ref|Os05g17380|
gi|8201631|ref|Os05g17390|
gi|8201632|ref|Os05g17400|
gi|8201635|ref|Os05g17430|
gi|8201636|ref|Os05g17440|
gi|8201641|ref|Os05g17490|
gi|8201643|ref|Os05g17510|
gi|8201644|ref|Os05g17520|
gi|8201645|ref|Os05g17530|
gi|8201647|ref|Os05g17550|
gi|8201648|ref|Os05g17560|
gi|8201651|ref|Os05g17590|
gi|8201658|ref|Os05g17620|
gi|8201661|ref|Os05g17650|
gi|8201662|ref|Os05g17660|
gi|8201663|ref|Os05g17670|
gi|8201664|ref|Os05g17680|
gi|8201665|ref|Os05g17690|
gi|8201667|ref|Os05g17710|
gi|8201669|ref|Os05g17730|
gi|8201670|ref|Os05g17740|
gi|8201672|ref|Os05g17760|
gi|8201673|ref|Os05g17770|
gi|8201682|ref|Os05g17860|
gi|8201684|ref|Os05g17880|
gi|8201698|ref|Os05g18020|
gi|8201704|ref|Os05g18080|
gi|8201707|ref|Os05g18110|
gi|8201708|ref|Os05g18120|
gi|8201709|ref|Os05g18130|
gi|8201710|ref|Os05g18140|
gi|8201711|ref|Os05g18150|
gi|8201718|ref|Os05g18220|
gi|8201721|ref|Os05g18250|
gi|8201726|ref|Os05g18300|
gi|8201728|ref|Os05g18320|
gi|8201729|ref|Os05g18330|
gi|8201731|ref|Os05g18350|
gi|8201735|ref|Os05g18390|
gi|8201736|ref|Os05g18400|
gi|8201738|ref|Os05g18420|
gi|8201739|ref|Os05g18430|
gi|8201740|ref|Os05g18440|
gi|8201741|ref|Os05g18450|
gi|8201742|ref|Os05g18460|
gi|8201746|ref|Os05g18500|
gi|8201749|ref|Os05g18530|
gi|8201755|ref|Os05g18590|
gi|8201756|ref|Os05g18600|
gi|8201760|ref|Os05g18640|
gi|8201761|ref|Os05g18650|
gi|8201766|ref|Os05g18700|
gi|8201768|ref|Os05g18720|
gi|8201776|ref|Os05g18800|
gi|8201780|ref|Os05g18840|
gi|8201783|ref|Os05g18870|
gi|8201793|ref|Os05g18970|
gi|8201795|ref|Os05g18990|
gi|8201803|ref|Os05g19060|
gi|8201804|ref|Os05g19070|
gi|8201805|ref|Os05g19080|
gi|8201808|ref|Os05g19110|
gi|8201809|ref|Os05g19120|
gi|8201810|ref|Os05g19130|
gi|8201820|ref|Os05g19230|
gi|8201826|ref|Os05g19290|
gi|8201829|ref|Os05g19320|
gi|8201831|ref|Os05g19340|
gi|8201833|ref|Os05g19360|
gi|8201837|ref|Os05g19400|
gi|8201839|ref|Os05g19420|
gi|8201841|ref|Os05g19440|
gi|8201846|ref|Os05g19490|
gi|8201848|ref|Os05g19510|
gi|8201859|ref|Os05g19620|
gi|8201863|ref|Os05g19640|
gi|8201865|ref|Os05g19660|
gi|8201867|ref|Os05g19680|
gi|8201868|ref|Os05g19690|
gi|8201870|ref|Os05g19710|
gi|8201871|ref|Os05g19720|
gi|8201876|ref|Os05g19770|
gi|8201878|ref|Os05g19790|
gi|8201879|ref|Os05g19800|
gi|8201881|ref|Os05g19820|
gi|8201882|ref|Os05g19830|
gi|8201883|ref|Os05g19840|
gi|8201885|ref|Os05g19860|
gi|8201887|ref|Os05g19880|
gi|8201892|ref|Os05g19920|
gi|8201893|ref|Os05g19930|
gi|8201898|ref|Os05g19980|
gi|8201901|ref|Os05g20010|
gi|8201904|ref|Os05g20040|
gi|8201908|ref|Os05g20060|
gi|8201909|ref|Os05g20070|
gi|8201910|ref|Os05g20080|
gi|8201911|ref|Os05g20090|
gi|8201913|ref|Os05g20110|
gi|8201915|ref|Os05g20130|
gi|8201916|ref|Os05g20140|
gi|8201919|ref|Os05g20170|
gi|8201921|ref|Os05g20190|
gi|8201923|ref|Os05g20210|
gi|8201924|ref|Os05g20220|
gi|8201926|ref|Os05g20240|
gi|8201932|ref|Os05g20300|
gi|8201937|ref|Os05g20350|
gi|8201940|ref|Os05g20380|
gi|8201944|ref|Os05g20420|
gi|8201946|ref|Os05g20440|
gi|8201949|ref|Os05g20470|
gi|8201954|ref|Os05g20520|
gi|8201956|ref|Os05g20540|
gi|8201961|ref|Os05g20590|
gi|8201966|ref|Os05g20640|
gi|8201967|ref|Os05g20650|
gi|8201968|ref|Os05g20660|
gi|8201974|ref|Os05g20720|
gi|8201976|ref|Os05g20740|
gi|8201981|ref|Os05g20790|
gi|8201983|ref|Os05g20810|
gi|8201988|ref|Os05g20860|
gi|8201989|ref|Os05g20870|
gi|8201992|ref|Os05g20900|
gi|8201993|ref|Os05g20910|
gi|8202000|ref|Os05g20980|
gi|8202003|ref|Os05g21010|
gi|8202005|ref|Os05g21030|

gi|8202007|ref|Os05g21050|
gi|8202008|ref|Os05g21060|
gi|8202023|ref|Os05g21200|
gi|8202027|ref|Os05g21240|
gi|8202028|ref|Os05g21250|
gi|8202033|ref|Os05g22270|
gi|8202034|ref|Os05g22280|
gi|8202035|ref|Os05g22290|
gi|8202042|ref|Os05g22360|
gi|8202043|ref|Os05g22370|
gi|8202044|ref|Os05g22380|
gi|8202057|ref|Os05g22510|
gi|8202067|ref|Os05g22610|
gi|8202070|ref|Os05g22640|
gi|8202072|ref|Os05g22660|
gi|8202080|ref|Os05g22740|
gi|8202084|ref|Os05g22780|
gi|8202085|ref|Os05g22790|
gi|8202088|ref|Os05g22820|
gi|8202090|ref|Os05g22840|
gi|8202094|ref|Os05g22880|
gi|8202095|ref|Os05g22890|
gi|8202097|ref|Os05g22910|
gi|8202104|ref|Os05g22980|
gi|8202106|ref|Os05g23000|
gi|8202109|ref|Os05g23030|
gi|8202110|ref|Os05g23040|
gi|8202112|ref|Os05g23060|
gi|8202116|ref|Os05g23100|
gi|8202122|ref|Os05g23160|
gi|8202126|ref|Os05g23200|
gi|8202129|ref|Os05g23230|
gi|8202133|ref|Os05g23270|
gi|8202137|ref|Os05g23310|
gi|8202140|ref|Os05g23340|
gi|8202145|ref|Os05g23390|
gi|8202146|ref|Os05g23400|
gi|8202147|ref|Os05g23410|
gi|8202151|ref|Os05g23450|
gi|8202154|ref|Os05g23480|
gi|8202155|ref|Os05g23490|
gi|8202157|ref|Os05g23510|
gi|8202158|ref|Os05g23520|
gi|8202162|ref|Os05g23540|
gi|8202164|ref|Os05g23560|
gi|8202165|ref|Os05g23570|
gi|8202166|ref|Os05g23580|
gi|8202171|ref|Os05g23630|
gi|8202173|ref|Os05g23650|
gi|8202177|ref|Os05g23680|
gi|8202181|ref|Os05g23710|
gi|8202185|ref|Os05g23760|
gi|8202194|ref|Os05g23850|
gi|8202199|ref|Os05g23900|
gi|8202202|ref|Os05g23930|
gi|8202207|ref|Os05g23980|
gi|8202212|ref|Os05g24030|
gi|8202217|ref|Os05g24080|
gi|8202218|ref|Os05g24090|
gi|8202220|ref|Os05g24110|
gi|8202227|ref|Os05g24180|
gi|8202230|ref|Os05g24210|
gi|8202232|ref|Os05g24230|
gi|8202235|ref|Os05g24260|
gi|8202237|ref|Os05g24280|
gi|8202243|ref|Os05g24340|
gi|8202244|ref|Os05g24350|
gi|8202248|ref|Os05g24390|
gi|8202251|ref|Os05g24420|
gi|8202254|ref|Os05g24450|
gi|8202258|ref|Os05g24490|
gi|8202260|ref|Os05g24510|
gi|8202263|ref|Os05g24540|
gi|8202272|ref|Os05g24630|
gi|8202279|ref|Os05g24700|
gi|8202283|ref|Os05g24740|
gi|8202290|ref|Os05g24810|
gi|8202291|ref|Os05g24820|
gi|8202292|ref|Os05g24830|
gi|8202296|ref|Os05g24870|
gi|8202299|ref|Os05g24900|
gi|8202303|ref|Os05g24940|
gi|8202308|ref|Os05g24980|
gi|8202310|ref|Os05g25000|
gi|8202313|ref|Os05g25040|
gi|8202314|ref|Os05g25040|
gi|8202315|ref|Os05g25030|
gi|8202316|ref|Os05g25050|
gi|8202320|ref|Os05g25090|
gi|8202323|ref|Os05g25120|
gi|8202333|ref|Os05g25220|
gi|8202334|ref|Os05g25230|
gi|8202335|ref|Os05g25240|
gi|8202336|ref|Os05g25250|
gi|8202337|ref|Os05g25260|
gi|8202338|ref|Os05g25270|
gi|8202345|ref|Os05g25340|
gi|8202347|ref|Os05g25360|
gi|8202352|ref|Os05g25410|
gi|8202353|ref|Os05g25420|
gi|8202355|ref|Os05g25440|
gi|8202357|ref|Os05g25460|
gi|8202361|ref|Os05g25500|
gi|8202362|ref|Os05g25510|
gi|8202363|ref|Os05g25520|
gi|8202364|ref|Os05g25530|
gi|8202369|ref|Os05g25580|
gi|8202370|ref|Os05g25590|
gi|8202373|ref|Os05g25620|
gi|8202376|ref|Os05g25650|
gi|8202378|ref|Os05g25670|
gi|8202379|ref|Os05g25680|
gi|8202380|ref|Os05g25690|
gi|8202382|ref|Os05g25710|
gi|8202383|ref|Os05g25720|
gi|8202387|ref|Os05g25760|
gi|8202392|ref|Os05g25810|
gi|8202397|ref|Os05g25860|
gi|8202402|ref|Os05g25900|
gi|8202404|ref|Os05g25920|
gi|8202406|ref|Os05g25940|
gi|8202408|ref|Os05g25960|
gi|8202409|ref|Os05g25970|
gi|8202410|ref|Os05g25980|
gi|8202411|ref|Os05g25990|
gi|8202419|ref|Os05g26070|
gi|8202425|ref|Os05g26120|
gi|8202426|ref|Os05g26130|
gi|8202427|ref|Os05g26140|
gi|8202428|ref|Os05g26150|
gi|8202432|ref|Os05g26190|
gi|8202435|ref|Os05g26220|
gi|8202437|ref|Os05g26240|
gi|8202438|ref|Os05g26250|

gi|8202441|ref|Os05g26280|
gi|8202448|ref|Os05g26350|
gi|8202449|ref|Os05g26360|
gi|8202450|ref|Os05g26370|
gi|8202451|ref|Os05g26380|
gi|8202452|ref|Os05g26390|
gi|8202453|ref|Os05g26400|
gi|8202456|ref|Os05g26430|
gi|8202457|ref|Os05g26440|
gi|8202459|ref|Os05g26460|
gi|8202461|ref|Os05g26480|
gi|8202462|ref|Os05g26490|
gi|8202465|ref|Os05g26520|
gi|8202466|ref|Os05g26530|
gi|8202467|ref|Os05g26540|
gi|8202474|ref|Os05g26610|
gi|8202475|ref|Os05g26620|
gi|8202476|ref|Os05g26630|
gi|8202480|ref|Os05g26670|
gi|8202482|ref|Os05g26690|
gi|8202483|ref|Os05g26700|
gi|8202484|ref|Os05g26710|
gi|8202485|ref|Os05g26720|
gi|8202488|ref|Os05g26750|
gi|8202489|ref|Os05g26760|
gi|8202490|ref|Os05g26770|
gi|8202491|ref|Os05g26780|
gi|8202496|ref|Os05g26830|
gi|8202498|ref|Os05g26850|
gi|8202499|ref|Os05g26860|
gi|8202506|ref|Os05g26910|
gi|8202511|ref|Os05g26960|
gi|8202513|ref|Os05g26980|
gi|8202515|ref|Os05g27000|
gi|8202527|ref|Os05g27110|
gi|8202532|ref|Os05g27160|
gi|8202534|ref|Os05g27180|
gi|8202536|ref|Os05g27200|
gi|8202537|ref|Os05g27210|
gi|8202538|ref|Os05g27220|
gi|8202540|ref|Os05g27240|
gi|8202541|ref|Os05g27250|
gi|8202545|ref|Os05g27290|
gi|8202549|ref|Os05g27330|
gi|8202551|ref|Os05g27350|
gi|8202552|ref|Os05g27360|
gi|8202553|ref|Os05g27370|
gi|8202554|ref|Os05g27380|
gi|8202555|ref|Os05g27390|
gi|8202560|ref|Os05g27440|
gi|8202562|ref|Os05g27460|
gi|8202567|ref|Os05g27510|
gi|8202580|ref|Os05g27640|
gi|8202583|ref|Os05g27670|
gi|8202584|ref|Os05g27680|
gi|8202590|ref|Os05g27740|
gi|8202594|ref|Os05g27780|
gi|8202596|ref|Os05g27800|
gi|8202597|ref|Os05g27810|
gi|8202599|ref|Os05g27830|
gi|8202600|ref|Os05g27840|
gi|8202607|ref|Os05g27900|
gi|8202608|ref|Os05g27910|
gi|8202619|ref|Os05g28000|
gi|8202621|ref|Os05g28020|
gi|8202622|ref|Os05g28030|
gi|8202625|ref|Os05g28060|
gi|8202634|ref|Os05g28150|
gi|8202645|ref|Os05g28260|
gi|8202650|ref|Os05g28310|
gi|8202652|ref|Os05g28330|
gi|8202655|ref|Os05g28360|
gi|8202657|ref|Os05g28380|
gi|8202658|ref|Os05g28390|
gi|8202659|ref|Os05g28400|
gi|8202660|ref|Os05g28410|
gi|8202663|ref|Os05g28440|
gi|8202664|ref|Os05g28450|
gi|8202666|ref|Os05g28470|
gi|8202677|ref|Os05g28570|
gi|8202678|ref|Os05g28580|
gi|8202679|ref|Os05g28590|
gi|8202681|ref|Os05g28610|
gi|8202684|ref|Os05g28640|
gi|8202685|ref|Os05g28650|
gi|8202687|ref|Os05g28670|
gi|8202696|ref|Os05g28760|
gi|8202700|ref|Os05g28800|
gi|8202701|ref|Os05g28810|
gi|8202705|ref|Os05g28850|
gi|8202708|ref|Os05g28880|
gi|8202720|ref|Os05g28970|
gi|8202722|ref|Os05g28990|
gi|8202723|ref|Os05g29000|
gi|8202727|ref|Os05g29040|
gi|8202729|ref|Os05g29060|
gi|8202730|ref|Os05g29070|
gi|8202731|ref|Os05g29080|
gi|8202732|ref|Os05g29090|
gi|8202738|ref|Os05g29150|
gi|8202740|ref|Os05g29170|
gi|8202742|ref|Os05g29680|
gi|8202747|ref|Os05g29730|
gi|8202748|ref|Os05g29740|
gi|8202751|ref|Os05g29770|
gi|8202757|ref|Os05g29830|
gi|8202761|ref|Os05g29870|
gi|8202763|ref|Os05g29890|
gi|8202768|ref|Os05g29940|
gi|8202769|ref|Os05g29950|
gi|8202770|ref|Os05g29960|
gi|8202774|ref|Os05g30000|
gi|8202776|ref|Os05g30020|
gi|8202777|ref|Os05g30030|
gi|8202784|ref|Os05g30100|
gi|8202785|ref|Os05g30110|
gi|8202790|ref|Os05g30160|
gi|8202791|ref|Os05g30170|
gi|8202792|ref|Os05g30180|
gi|8202794|ref|Os05g30200|
gi|8202797|ref|Os05g30230|
gi|8202808|ref|Os05g30340|
gi|8202812|ref|Os05g30370|
gi|8202815|ref|Os05g30400|
gi|8202818|ref|Os05g30430|
gi|8202819|ref|Os05g30440|
gi|8202822|ref|Os05g30470|
gi|8202825|ref|Os05g30500|
gi|8202826|ref|Os05g30510|
gi|8202835|ref|Os05g30600|
gi|8202837|ref|Os05g30620|
gi|8202838|ref|Os05g30630|
gi|8202840|ref|Os05g30650|
gi|8202842|ref|Os05g30670|

gi|8202845|ref|Os05g30700|
gi|8202848|ref|Os05g30730|
gi|8202861|ref|Os05g30860|
gi|8202866|ref|Os05g30910|
gi|8202868|ref|Os05g30930|
gi|8202869|ref|Os05g30940|
gi|8202871|ref|Os05g30960|
gi|8202877|ref|Os05g31010|
gi|8202879|ref|Os05g31030|
gi|8202881|ref|Os05g31050|
gi|8202885|ref|Os05g31090|
gi|8202886|ref|Os05g31100|
gi|8202892|ref|Os05g31150|
gi|8202896|ref|Os05g31180|
gi|8202897|ref|Os05g31190|
gi|8202905|ref|Os05g31270|
gi|8202908|ref|Os05g31300|
gi|8202909|ref|Os05g31310|
gi|8202910|ref|Os05g31320|
gi|8202913|ref|Os05g31350|
gi|8202914|ref|Os05g31360|
gi|8202915|ref|Os05g31370|
gi|8202917|ref|Os05g31390|
gi|8202918|ref|Os05g31400|
gi|8202923|ref|Os05g31450|
gi|8202924|ref|Os05g31460|
gi|8202925|ref|Os05g31470|
gi|8202928|ref|Os05g31500|
gi|8202930|ref|Os05g31520|
gi|8202934|ref|Os05g31560|
gi|8202936|ref|Os05g31580|
gi|8202937|ref|Os05g31590|
gi|8202942|ref|Os05g31640|
gi|8202943|ref|Os05g31650|
gi|8202944|ref|Os05g31660|
gi|8202948|ref|Os05g31700|
gi|8202960|ref|Os05g31790|
gi|8202961|ref|Os05g31800|
gi|8202962|ref|Os05g31810|
gi|8202963|ref|Os05g31820|
gi|8202965|ref|Os05g31840|
gi|8202968|ref|Os05g31870|
gi|8202971|ref|Os05g31900|
gi|8202977|ref|Os05g31940|
gi|8202978|ref|Os05g31950|
gi|8202984|ref|Os05g32010|
gi|8202985|ref|Os05g32020|
gi|8202987|ref|Os05g32040|
gi|8202992|ref|Os05g32090|
gi|8202993|ref|Os05g32100|
gi|8202999|ref|Os05g32150|
gi|8203000|ref|Os05g32160|
gi|8203004|ref|Os05g32200|
gi|8203008|ref|Os05g32240|
gi|8203009|ref|Os05g32250|
gi|8203010|ref|Os05g32260|
gi|8203012|ref|Os05g32280|
gi|8203014|ref|Os05g32300|
gi|8203028|ref|Os05g32420|
gi|8203031|ref|Os05g32450|
gi|8203035|ref|Os05g32490|
gi|8203039|ref|Os05g32520|
gi|8203043|ref|Os05g32560|
gi|8203049|ref|Os05g32620|
gi|8203051|ref|Os05g32640|
gi|8203052|ref|Os05g32650|
gi|8203056|ref|Os05g32690|
gi|8203062|ref|Os05g32750|
gi|8203064|ref|Os05g32770|
gi|8203065|ref|Os05g32780|
gi|8203066|ref|Os05g32790|
gi|8203067|ref|Os05g32800|
gi|8203070|ref|Os05g32830|
gi|8203071|ref|Os05g32840|
gi|8203073|ref|Os05g32860|
gi|8203074|ref|Os05g32870|
gi|8203078|ref|Os05g32910|
gi|8203079|ref|Os05g32920|
gi|8203080|ref|Os05g32930|
gi|8203084|ref|Os05g32970|
gi|8203085|ref|Os05g32980|
gi|8203086|ref|Os05g32990|
gi|8203094|ref|Os05g33060|
gi|8203095|ref|Os05g33070|
gi|8203097|ref|Os05g33090|
gi|8203106|ref|Os05g33180|
gi|8203114|ref|Os05g33250|
gi|8203119|ref|Os05g33290|
gi|8203125|ref|Os05g33350|
gi|8203138|ref|Os05g33470|
gi|8203144|ref|Os05g33530|
gi|8203147|ref|Os05g33560|
gi|8203149|ref|Os05g33580|
gi|8203162|ref|Os05g33660|
gi|8203164|ref|Os05g33680|
gi|8203175|ref|Os05g33780|
gi|8203177|ref|Os05g33800|
gi|8203190|ref|Os05g33930|
gi|8203192|ref|Os05g33950|
gi|8203194|ref|Os05g33970|
gi|8203199|ref|Os05g34020|
gi|8203203|ref|Os05g34060|
gi|8203207|ref|Os05g34100|
gi|8203209|ref|Os05g34120|
gi|8203210|ref|Os05g34130|
gi|8203225|ref|Os05g34250|
gi|8203228|ref|Os05g34280|
gi|8203230|ref|Os05g34300|
gi|8203236|ref|Os05g34360|
gi|8203240|ref|Os05g34400|
gi|8203249|ref|Os05g34490|
gi|8203250|ref|Os05g34500|
gi|8203251|ref|Os05g34510|
gi|8203253|ref|Os05g34530|
gi|8203256|ref|Os05g34560|
gi|8203258|ref|Os05g34580|
gi|8203261|ref|Os05g34610|
gi|8203266|ref|Os05g34660|
gi|8203270|ref|Os05g34690|
gi|8203272|ref|Os05g34710|
gi|8203274|ref|Os05g34730|
gi|8203275|ref|Os05g34740|
gi|8203281|ref|Os05g34800|
gi|8203286|ref|Os05g34840|
gi|8203291|ref|Os05g34890|
gi|8203295|ref|Os05g34930|
gi|8203299|ref|Os05g34960|
gi|8203300|ref|Os05g34970|
gi|8203302|ref|Os05g34990|
gi|8203305|ref|Os05g35020|
gi|8203306|ref|Os05g35030|
gi|8203307|ref|Os05g35040|
gi|8203311|ref|Os05g35080|
gi|8203316|ref|Os05g35130|

gi|8203318|ref|Os05g35150|
gi|8203323|ref|Os05g35180|
gi|8203326|ref|Os05g35210|
gi|8203327|ref|Os05g35220|
gi|8203329|ref|Os05g35240|
gi|8203330|ref|Os05g35250|
gi|8203340|ref|Os05g35350|
gi|8203347|ref|Os05g35420|
gi|8203351|ref|Os05g35450|
gi|8203355|ref|Os05g35490|
gi|8203357|ref|Os05g35510|
gi|8203362|ref|Os05g35550|
gi|8203363|ref|Os05g35560|
gi|8203368|ref|Os05g35610|
gi|8203369|ref|Os05g35620|
gi|8203370|ref|Os05g35630|
gi|8203371|ref|Os05g35640|
gi|8203373|ref|Os05g35660|
gi|8203375|ref|Os05g35680|
gi|8203377|ref|Os05g35700|
gi|8203388|ref|Os05g35780|
gi|8203390|ref|Os05g35800|
gi|8203391|ref|Os05g35810|
gi|8203393|ref|Os05g35830|
gi|8203395|ref|Os05g35850|
gi|8203397|ref|Os05g35870|
gi|8203400|ref|Os05g35900|
gi|8203402|ref|Os05g35920|
gi|8203404|ref|Os05g35940|
gi|8203408|ref|Os05g35980|
gi|8203409|ref|Os05g35990|
gi|8203413|ref|Os05g36020|
gi|8203419|ref|Os05g36080|
gi|8203427|ref|Os05g36130|
gi|8203428|ref|Os05g36140|
gi|8203435|ref|Os05g36210|
gi|8203438|ref|Os05g36240|
gi|8203439|ref|Os05g36250|
gi|8203445|ref|Os05g36300|
gi|8203447|ref|Os05g36320|
gi|8203448|ref|Os05g36330|
gi|8203449|ref|Os05g36340|
gi|8203452|ref|Os05g36370|
gi|8203453|ref|Os05g36380|
gi|8203462|ref|Os05g36950|
gi|8203465|ref|Os05g36980|
gi|8203467|ref|Os05g37000|
gi|8203468|ref|Os05g37010|
gi|8203469|ref|Os05g37020|
gi|8203476|ref|Os05g37090|
gi|8203477|ref|Os05g37100|
gi|8203478|ref|Os05g37110|
gi|8203489|ref|Os05g37180|
gi|8203497|ref|Os05g37260|
gi|8203502|ref|Os05g37310|
gi|8203507|ref|Os05g37360|
gi|8203514|ref|Os05g37420|
gi|8203516|ref|Os05g37440|
gi|8203518|ref|Os05g37460|
gi|8203521|ref|Os05g37480|
gi|8203522|ref|Os05g37490|
gi|8203524|ref|Os05g37510|
gi|8203529|ref|Os05g37560|
gi|8203534|ref|Os05g37610|
gi|8203536|ref|Os05g37630|
gi|8203538|ref|Os05g37650|
gi|8203540|ref|Os05g37670|
gi|8203541|ref|Os05g37680|
gi|8203544|ref|Os05g37710|
gi|8203545|ref|Os05g37720|
gi|8203550|ref|Os05g37770|
gi|8203551|ref|Os05g37780|
gi|8203552|ref|Os05g37790|
gi|8203554|ref|Os05g37810|
gi|8203558|ref|Os05g37840|
gi|8203560|ref|Os05g37860|
gi|8203563|ref|Os05g37890|
gi|8203567|ref|Os05g37920|
gi|8203571|ref|Os05g37940|
gi|8203578|ref|Os05g37990|
gi|8203581|ref|Os05g38020|
gi|8203582|ref|Os05g38030|
gi|8203583|ref|Os05g38040|
gi|8203584|ref|Os05g38040|
gi|8203585|ref|Os05g38050|
gi|8203588|ref|Os05g38080|
gi|8203589|ref|Os05g38090|
gi|8203593|ref|Os05g38130|
gi|8203615|ref|Os05g38300|
gi|8203633|ref|Os05g38440|
gi|8203640|ref|Os05g38510|
gi|8203658|ref|Os05g38650|
gi|8203659|ref|Os05g38660|
gi|8203663|ref|Os05g38700|
gi|8203669|ref|Os05g38750|
gi|8203680|ref|Os05g38840|
gi|8203682|ref|Os05g38860|
gi|8203683|ref|Os05g38870|
gi|8203684|ref|Os05g38880|
gi|8203687|ref|Os05g38910|
gi|8203689|ref|Os05g38930|
gi|8203690|ref|Os05g38940|
gi|8203697|ref|Os05g39010|
gi|8203706|ref|Os05g39100|
gi|8203710|ref|Os05g39140|
gi|8203711|ref|Os05g39150|
gi|8203712|ref|Os05g39160|
gi|8203713|ref|Os05g39170|
gi|8203714|ref|Os05g39180|
gi|8203715|ref|Os05g39190|
gi|8203716|ref|Os05g39200|
gi|8203717|ref|Os05g39210|
gi|8203725|ref|Os05g39290|
gi|8203734|ref|Os05g39370|
gi|8203737|ref|Os05g39400|
gi|8203743|ref|Os05g39440|
gi|8203750|ref|Os05g39510|
gi|8203763|ref|Os05g39630|
gi|8203764|ref|Os05g39640|
gi|8203766|ref|Os05g39660|
gi|8203770|ref|Os05g39700|
gi|8203775|ref|Os05g39750|
gi|8203787|ref|Os05g39860|
gi|8203792|ref|Os05g39910|
gi|8203801|ref|Os05g39970|
gi|8203802|ref|Os05g39980|
gi|8203807|ref|Os05g40030|
gi|8203808|ref|Os05g40040|
gi|8203816|ref|Os05g40120|
gi|8203817|ref|Os05g40130|
gi|8203821|ref|Os05g40170|
gi|8203825|ref|Os05g40210|
gi|8203828|ref|Os05g40240|
gi|8203829|ref|Os05g40250|

gi|8203846|ref|Os05g40340|
gi|8203847|ref|Os05g40350|
gi|8203848|ref|Os05g40360|
gi|8203849|ref|Os05g40370|
gi|8203850|ref|Os05g40380|
gi|8203852|ref|Os05g40400|
gi|8203857|ref|Os05g40430|
gi|8203865|ref|Os05g40510|
gi|8203866|ref|Os05g40520|
gi|8203867|ref|Os05g40530|
gi|8203870|ref|Os05g40560|
gi|8203872|ref|Os05g40580|
gi|8203873|ref|Os05g40590|
gi|8203876|ref|Os05g40620|
gi|8203878|ref|Os05g40640|
gi|8203882|ref|Os05g40680|
gi|8203883|ref|Os05g40690|
gi|8203896|ref|Os05g40800|
gi|8203900|ref|Os05g40830|
gi|8203901|ref|Os05g40840|
gi|8203902|ref|Os05g40850|
gi|8203905|ref|Os05g40880|
gi|8203906|ref|Os05g40890|
gi|8203907|ref|Os05g40890|
gi|8203908|ref|Os05g40900|
gi|8203910|ref|Os05g40920|
gi|8203912|ref|Os05g40940|
gi|8203915|ref|Os05g40970|
gi|8203920|ref|Os05g41020|
gi|8203923|ref|Os05g41050|
gi|8203934|ref|Os05g41140|
gi|8203951|ref|Os05g41300|
gi|8203959|ref|Os05g41380|
gi|8203962|ref|Os05g41410|
gi|8203964|ref|Os05g41430|
gi|8203969|ref|Os05g41470|
gi|8203973|ref|Os05g41500|
gi|8203978|ref|Os05g41550|
gi|8203979|ref|Os05g41550|
gi|8203980|ref|Os05g41550|
gi|8203981|ref|Os05g41550|
gi|8203983|ref|Os05g41570|
gi|8203995|ref|Os05g41650|
gi|8204003|ref|Os05g41730|
gi|8204004|ref|Os05g41740|
gi|8204013|ref|Os05g41820|
gi|8204015|ref|Os05g41840|
gi|8204018|ref|Os05g41870|
gi|8204020|ref|Os05g41890|
gi|8204025|ref|Os05g41920|
gi|8204030|ref|Os05g41960|
gi|8204043|ref|Os05g42080|
gi|8204044|ref|Os05g42090|
gi|8204051|ref|Os05g42160|
gi|8204052|ref|Os05g42170|
gi|8204055|ref|Os05g42200|
gi|8204059|ref|Os05g42240|
gi|8204061|ref|Os05g42260|
gi|8204069|ref|Os05g42340|
gi|8204071|ref|Os05g42360|
gi|8204073|ref|Os05g42380|
gi|8204074|ref|Os05g42390|
gi|8204075|ref|Os05g42400|
gi|8204077|ref|Os05g42420|
gi|8204080|ref|Os05g42940|
gi|8204084|ref|Os05g42980|
gi|8204085|ref|Os05g42990|
gi|8204089|ref|Os05g43030|
gi|8204093|ref|Os05g43070|
gi|8204104|ref|Os05g43180|
gi|8204105|ref|Os05g43190|
gi|8204106|ref|Os05g43200|
gi|8204107|ref|Os05g43210|
gi|8204116|ref|Os05g43290|
gi|8204119|ref|Os05g43310|
gi|8204120|ref|Os05g43320|
gi|8204121|ref|Os05g43330|
gi|8204128|ref|Os05g43400|
gi|8204139|ref|Os05g43500|
gi|8204153|ref|Os05g43640|
gi|8204155|ref|Os05g43660|
gi|8204159|ref|Os05g43700|
gi|8204161|ref|Os05g43720|
gi|8204164|ref|Os05g43750|
gi|8204169|ref|Os05g43800|
gi|8204175|ref|Os05g43860|
gi|8204176|ref|Os05g43870|
gi|8204178|ref|Os05g43890|
gi|8204179|ref|Os05g43900|
gi|8204187|ref|Os05g43980|
gi|8204192|ref|Os05g44040|
gi|8204195|ref|Os05g44060|
gi|8204201|ref|Os05g44110|
gi|8204202|ref|Os05g44120|
gi|8204207|ref|Os05g44150|
gi|8204208|ref|Os05g44160|
gi|8204211|ref|Os05g44190|
gi|8204214|ref|Os05g44220|
gi|8204235|ref|Os05g44370|
gi|8204240|ref|Os05g44420|
gi|8204241|ref|Os05g44430|
gi|8204242|ref|Os05g44440|
gi|8204243|ref|Os05g44450|
gi|8204246|ref|Os05g44480|
gi|8204261|ref|Os05g44610|
gi|8204262|ref|Os05g44610|
gi|8204264|ref|Os05g44630|
gi|8204265|ref|Os05g44640|
gi|8204266|ref|Os05g44650|
gi|8204268|ref|Os05g44670|
gi|8204270|ref|Os05g44690|
gi|8204272|ref|Os05g44710|
gi|8204273|ref|Os05g44720|
gi|8204276|ref|Os05g44750|
gi|8204279|ref|Os05g44780|
gi|8204281|ref|Os05g44800|
gi|8204282|ref|Os05g44800|
gi|8204284|ref|Os05g44820|
gi|8204285|ref|Os05g44830|
gi|8204286|ref|Os05g44840|
gi|8204292|ref|Os05g44900|
gi|8204300|ref|Os05g44980|
gi|8204323|ref|Os05g45190|
gi|8204331|ref|Os05g45250|
gi|8204332|ref|Os05g45260|
gi|8204335|ref|Os05g45290|
gi|8204340|ref|Os05g45330|
gi|8204343|ref|Os05g45360|
gi|8204345|ref|Os05g45380|
gi|8204346|ref|Os05g45390|
gi|8204357|ref|Os05g45470|
gi|8204360|ref|Os05g45500|
gi|8204363|ref|Os05g45530|
gi|8204364|ref|Os05g45540|

gi|8204366|ref|Os05g45560|
gi|8204368|ref|Os05g45580|
gi|8204371|ref|Os05g45610|
gi|8204390|ref|Os05g45780|
gi|8204408|ref|Os05g45940|
gi|8204412|ref|Os05g45980|
gi|8204424|ref|Os05g46100|
gi|8204426|ref|Os05g46120|
gi|8204431|ref|Os05g46170|
gi|8204432|ref|Os05g46180|
gi|8204433|ref|Os05g46190|
gi|8204449|ref|Os05g46310|
gi|8204450|ref|Os05g46320|
gi|8204451|ref|Os05g46320|
gi|8204453|ref|Os05g46340|
gi|8204454|ref|Os05g46340|
gi|8204459|ref|Os05g46380|
gi|8204462|ref|Os05g46410|
gi|8204466|ref|Os05g46440|
gi|8204473|ref|Os05g46470|
gi|8204474|ref|Os05g46480|
gi|8204477|ref|Os05g46500|
gi|8204480|ref|Os05g46530|
gi|8204481|ref|Os05g46540|
gi|8204486|ref|Os05g46590|
gi|8204487|ref|Os05g46600|
gi|8204490|ref|Os05g46630|
gi|8204491|ref|Os05g46640|
gi|8204492|ref|Os05g46650|
gi|8204494|ref|Os05g46670|
gi|8204495|ref|Os05g46680|
gi|8204497|ref|Os05g46700|
gi|8204501|ref|Os05g46730|
gi|8204507|ref|Os05g46790|
gi|8204508|ref|Os05g46800|
gi|8204510|ref|Os05g46820|
gi|8204511|ref|Os05g46830|
gi|8204512|ref|Os05g46840|
gi|8204515|ref|Os05g46870|
gi|8204516|ref|Os05g46880|
gi|8204517|ref|Os05g46890|
gi|8204518|ref|Os05g46890|
gi|8204519|ref|Os05g46900|
gi|8204522|ref|Os05g46930|
gi|8204524|ref|Os05g46950|
gi|8204527|ref|Os05g47470|
gi|8204533|ref|Os05g47520|
gi|8204542|ref|Os05g47600|
gi|8204550|ref|Os05g47680|
gi|8204554|ref|Os05g47720|
gi|8204558|ref|Os05g47760|
gi|8204561|ref|Os05g47790|
gi|8204563|ref|Os05g47810|
gi|8204564|ref|Os05g47820|
gi|8204565|ref|Os05g47830|
gi|8204568|ref|Os05g47860|
gi|8204569|ref|Os05g47870|
gi|8204579|ref|Os05g47960|
gi|8204583|ref|Os05g48000|
gi|8204591|ref|Os05g48080|
gi|8204596|ref|Os05g48120|
gi|8204597|ref|Os05g48130|
gi|8204598|ref|Os05g48140|
gi|8204604|ref|Os05g48180|
gi|8204605|ref|Os05g48190|
gi|8204608|ref|Os05g48210|
gi|8204613|ref|Os05g48250|
gi|8204624|ref|Os05g48350|
gi|8204630|ref|Os05g48400|
gi|8204633|ref|Os05g48430|
gi|8204635|ref|Os05g48460|
gi|8204639|ref|Os05g48470|
gi|8204641|ref|Os05g48490|
gi|8204647|ref|Os05g48540|
gi|8204649|ref|Os05g48560|
gi|8204650|ref|Os05g48570|
gi|8204654|ref|Os05g48610|
gi|8204662|ref|Os05g48680|
gi|8204665|ref|Os05g48710|
gi|8204667|ref|Os05g48730|
gi|8204668|ref|Os05g48740|
gi|8204672|ref|Os05g48780|
gi|8204673|ref|Os05g48790|
gi|8204679|ref|Os05g48830|
gi|8204690|ref|Os05g48920|
gi|8204691|ref|Os05g48930|
gi|8204708|ref|Os05g49080|
gi|8204719|ref|Os05g49190|
gi|8204738|ref|Os05g49330|
gi|8204739|ref|Os05g49340|
gi|8204740|ref|Os05g49350|
gi|8204741|ref|Os05g49360|
gi|8204742|ref|Os05g49370|
gi|8204744|ref|Os05g49390|
gi|8204755|ref|Os05g49490|
gi|8204756|ref|Os05g49500|
gi|8204759|ref|Os05g49530|
gi|8204769|ref|Os05g49630|
gi|8204770|ref|Os05g49640|
gi|8204772|ref|Os05g49660|
gi|8204773|ref|Os05g49670|
gi|8204775|ref|Os05g49690|
gi|8204778|ref|Os05g49720|
gi|8204805|ref|Os05g49950|
gi|8204806|ref|Os05g49960|
gi|8204809|ref|Os05g49990|
gi|8204810|ref|Os05g50000|
gi|8204815|ref|Os05g50050|
gi|8204820|ref|Os05g50100|
gi|8204825|ref|Os05g50150|
gi|8204835|ref|Os05g50230|
gi|8204836|ref|Os05g50240|
gi|8204844|ref|Os05g50320|
gi|8204845|ref|Os05g50330|
gi|8204851|ref|Os05g50390|
gi|8204852|ref|Os05g50400|
gi|8204854|ref|Os05g50420|
gi|8204855|ref|Os05g50430|
gi|8204856|ref|Os05g50440|
gi|8204857|ref|Os05g50450|
gi|8204859|ref|Os05g50470|
gi|8204872|ref|Os05g50590|
gi|8204878|ref|Os05g50650|
gi|8204880|ref|Os05g50670|
gi|8204886|ref|Os05g50730|
gi|8204890|ref|Os05g50760|
gi|8204896|ref|Os05g50820|
gi|8204899|ref|Os05g50850|
gi|8204919|ref|Os05g51030|
gi|8204924|ref|Os05g51080|
gi|8204938|ref|Os05g51210|
gi|8204944|ref|Os05g51270|
gi|8204951|ref|Os05g51340|
gi|8204955|ref|Os05g51380|

gi|8204962|ref|Os05g51410|
gi|8204963|ref|Os05g51430|
gi|8204981|ref|Os05g51580|
gi|8204984|ref|Os05g51600|
gi|8205001|ref|Os05g51720|
gi|8205010|ref|Os05g51810|
gi|8205012|ref|Os05g51830|
gi|8205016|ref|Os05g51870|
gi|8205019|ref|Os05g51900|
gi|8205021|ref|Os05g51920|
gi|8205023|ref|Os05g51940|
gi|8205025|ref|Os05g51960|
gi|8205026|ref|Os05g51970|
gi|8205027|ref|Os05g51980|
gi|8205032|ref|Os05g52030|
gi|8205034|ref|Os05g52050|
gi|8205035|ref|Os05g52060|
gi|8205037|ref|Os05g52080|
gi|8205038|ref|Os05g52090|
gi|8205039|ref|Os05g52100|
gi|8205041|ref|Os05g52120|
gi|8600005|ref|Os12g01050|
gi|8600007|ref|Os12g01070|
gi|8600011|ref|Os12g01110|
gi|8600013|ref|Os12g01130|
gi|8600015|ref|Os12g01150|
gi|8600025|ref|Os12g01250|
gi|8600028|ref|Os12g01280|
gi|8600030|ref|Os12g01300|
gi|8600031|ref|Os12g01310|
gi|8600035|ref|Os12g01350|
gi|8600042|ref|Os12g01410|
gi|8600064|ref|Os12g01590|
gi|8600065|ref|Os12g01590|
gi|8600067|ref|Os12g01610|
gi|8600068|ref|Os12g01620|
gi|8600069|ref|Os12g01630|
gi|8600073|ref|Os12g01670|
gi|8600078|ref|Os12g01720|
gi|8600088|ref|Os12g01810|
gi|8600090|ref|Os12g01840|
gi|8600095|ref|Os12g01880|
gi|8600104|ref|Os12g01960|
gi|8600106|ref|Os12g01980|
gi|8600107|ref|Os12g01990|
gi|8600108|ref|Os12g02000|
gi|8600112|ref|Os12g02040|
gi|8600113|ref|Os12g02050|
gi|8600120|ref|Os12g02110|
gi|8600123|ref|Os12g02140|
gi|8600125|ref|Os12g02160|
gi|8600126|ref|Os12g02170|
gi|8600127|ref|Os12g02180|
gi|8600128|ref|Os12g02190|
gi|8600133|ref|Os12g02240|
gi|8600148|ref|Os12g02360|
gi|8600152|ref|Os12g02410|
gi|8600161|ref|Os12g02480|
gi|8600168|ref|Os12g02560|
gi|8600170|ref|Os12g02580|
gi|8600171|ref|Os12g02590|
gi|8600173|ref|Os12g02610|
gi|8600177|ref|Os12g02650|
gi|8600180|ref|Os12g02680|
gi|8600181|ref|Os12g02690|
gi|8600182|ref|Os12g02690|
gi|8600183|ref|Os12g02690|
gi|8600184|ref|Os12g02690|
gi|8600194|ref|Os12g02790|
gi|8600202|ref|Os12g02850|
gi|8600206|ref|Os12g02890|
gi|8600209|ref|Os12g02920|
gi|8600210|ref|Os12g02930|
gi|8600211|ref|Os12g02940|
gi|8600217|ref|Os12g02990|
gi|8600225|ref|Os12g03070|
gi|8600233|ref|Os12g03120|
gi|8600235|ref|Os12g03140|
gi|8600243|ref|Os12g03220|
gi|8600254|ref|Os12g03320|
gi|8600255|ref|Os12g03330|
gi|8600260|ref|Os12g03380|
gi|8600262|ref|Os12g03400|
gi|8600264|ref|Os12g03420|
gi|8600273|ref|Os12g03490|
gi|8600275|ref|Os12g03510|
gi|8600279|ref|Os12g03550|
gi|8600282|ref|Os12g03580|
gi|8600283|ref|Os12g03590|
gi|8600285|ref|Os12g03610|
gi|8600302|ref|Os12g03780|
gi|8600304|ref|Os12g03800|
gi|8600315|ref|Os12g03900|
gi|8600316|ref|Os12g03910|
gi|8600318|ref|Os12g03930|
gi|8600319|ref|Os12g03940|
gi|8600323|ref|Os12g03980|
gi|8600332|ref|Os12g04070|
gi|8600348|ref|Os12g04230|
gi|8600350|ref|Os12g04250|
gi|8600359|ref|Os12g04330|
gi|8600365|ref|Os12g04390|
gi|8600366|ref|Os12g04400|
gi|8600373|ref|Os12g04470|
gi|8600375|ref|Os12g04490|
gi|8600379|ref|Os12g04530|
gi|8600390|ref|Os12g04630|
gi|8600396|ref|Os12g04690|
gi|8600398|ref|Os12g04710|
gi|8600399|ref|Os12g04720|
gi|8600400|ref|Os12g04740|
gi|8600403|ref|Os12g04730|
gi|8600405|ref|Os12g04760|
gi|8600408|ref|Os12g04790|
gi|8600409|ref|Os12g04800|
gi|8600411|ref|Os12g04820|
gi|8600414|ref|Os12g04850|
gi|8600425|ref|Os12g04950|
gi|8600426|ref|Os12g04960|
gi|8600434|ref|Os12g05010|
gi|8600436|ref|Os12g05030|
gi|8600439|ref|Os12g05060|
gi|8600440|ref|Os12g05070|
gi|8600443|ref|Os12g05100|
gi|8600447|ref|Os12g05130|
gi|8600448|ref|Os12g05140|
gi|8600451|ref|Os12g05170|
gi|8600453|ref|Os12g05190|
gi|8600456|ref|Os12g05220|
gi|8600459|ref|Os12g05250|
gi|8600464|ref|Os12g05300|
gi|8600466|ref|Os12g05320|
gi|8600468|ref|Os12g05340|
gi|8600469|ref|Os12g05350|

gi|8600479|ref|Os12g05450|
gi|8600482|ref|Os12g05480|
gi|8600487|ref|Os12g05530|
gi|8600492|ref|Os12g05580|
gi|8600496|ref|Os12g05620|
gi|8600500|ref|Os12g05670|
gi|8600505|ref|Os12g05690|
gi|8600508|ref|Os12g05720|
gi|8600510|ref|Os12g05740|
gi|8600513|ref|Os12g05770|
gi|8600515|ref|Os12g05790|
gi|8600516|ref|Os12g05800|
gi|8600518|ref|Os12g05820|
gi|8600525|ref|Os12g05890|
gi|8600531|ref|Os12g05950|
gi|8600532|ref|Os12g05960|
gi|8600533|ref|Os12g05970|
gi|8600545|ref|Os12g06090|
gi|8600548|ref|Os12g06120|
gi|8600549|ref|Os12g06130|
gi|8600550|ref|Os12g06140|
gi|8600553|ref|Os12g06170|
gi|8600555|ref|Os12g06190|
gi|8600558|ref|Os12g06210|
gi|8600561|ref|Os12g06240|
gi|8600562|ref|Os12g06250|
gi|8600565|ref|Os12g06280|
gi|8600566|ref|Os12g06280|
gi|8600567|ref|Os12g06290|
gi|8600568|ref|Os12g06300|
gi|8600569|ref|Os12g06310|
gi|8600577|ref|Os12g06390|
gi|8600579|ref|Os12g06400|
gi|8600581|ref|Os12g06420|
gi|8600582|ref|Os12g06430|
gi|8600584|ref|Os12g06450|
gi|8600585|ref|Os12g06460|
gi|8600586|ref|Os12g06470|
gi|8600589|ref|Os12g06500|
gi|8600594|ref|Os12g06550|
gi|8600610|ref|Os12g06700|
gi|8600613|ref|Os12g06730|
gi|8600616|ref|Os12g06750|
gi|8600617|ref|Os12g06760|
gi|8600618|ref|Os12g06770|
gi|8600620|ref|Os12g06790|
gi|8600624|ref|Os12g06820|
gi|8600625|ref|Os12g06830|
gi|8600635|ref|Os12g06900|
gi|8600639|ref|Os12g06930|
gi|8600640|ref|Os12g06940|
gi|8600641|ref|Os12g06950|
gi|8600642|ref|Os12g06960|
gi|8600645|ref|Os12g06990|
gi|8600646|ref|Os12g07000|
gi|8600653|ref|Os12g07070|
gi|8600655|ref|Os12g07090|
gi|8600656|ref|Os12g07100|
gi|8600666|ref|Os12g07180|
gi|8600672|ref|Os12g07240|
gi|8600673|ref|Os12g07250|
gi|8600679|ref|Os12g07310|
gi|8600682|ref|Os12g07340|
gi|8600684|ref|Os12g07370|
gi|8600685|ref|Os12g07370|
gi|8600686|ref|Os12g07370|
gi|8600687|ref|Os12g07360|
gi|8600688|ref|Os12g07380|
gi|8600689|ref|Os12g07380|
gi|8600690|ref|Os12g07390|
gi|8600691|ref|Os12g07400|
gi|8600692|ref|Os12g07410|
gi|8600693|ref|Os12g07420|
gi|8600694|ref|Os12g07430|
gi|8600695|ref|Os12g07440|
gi|8600697|ref|Os12g07460|
gi|8600698|ref|Os12g07470|
gi|8600701|ref|Os12g07500|
gi|8600702|ref|Os12g07510|
gi|8600704|ref|Os12g07530|
gi|8600706|ref|Os12g07550|
gi|8600707|ref|Os12g07560|
gi|8600708|ref|Os12g07570|
gi|8600712|ref|Os12g07600|
gi|8600714|ref|Os12g07620|
gi|8600718|ref|Os12g07660|
gi|8600724|ref|Os12g07710|
gi|8600729|ref|Os12g07760|
gi|8600733|ref|Os12g07790|
gi|8600739|ref|Os12g07850|
gi|8600743|ref|Os12g07890|
gi|8600746|ref|Os12g07920|
gi|8600748|ref|Os12g07940|
gi|8600750|ref|Os12g07960|
gi|8600756|ref|Os12g08010|
gi|8600757|ref|Os12g08020|
gi|8600758|ref|Os12g08030|
gi|8600767|ref|Os12g08120|
gi|8600770|ref|Os12g08150|
gi|8600771|ref|Os12g08160|
gi|8600775|ref|Os12g08200|
gi|8600779|ref|Os12g08240|
gi|8600780|ref|Os12g08250|
gi|8600787|ref|Os12g08290|
gi|8600789|ref|Os12g08310|
gi|8600790|ref|Os12g08320|
gi|8600792|ref|Os12g08340|
gi|8600793|ref|Os12g08350|
gi|8600796|ref|Os12g08380|
gi|8600797|ref|Os12g08390|
gi|8600798|ref|Os12g08400|
gi|8600800|ref|Os12g08420|
gi|8600806|ref|Os12g08480|
gi|8600812|ref|Os12g08540|
gi|8600813|ref|Os12g08550|
gi|8600815|ref|Os12g08570|
gi|8600821|ref|Os12g08630|
gi|8600826|ref|Os12g08680|
gi|8600827|ref|Os12g08690|
gi|8600828|ref|Os12g08700|
gi|8600829|ref|Os12g08710|
gi|8600833|ref|Os12g08750|
gi|8600839|ref|Os12g08790|
gi|8600846|ref|Os12g08840|
gi|8600848|ref|Os12g08860|
gi|8600849|ref|Os12g08870|
gi|8600855|ref|Os12g08930|
gi|8600856|ref|Os12g08940|
gi|8600857|ref|Os12g08950|
gi|8600859|ref|Os12g08970|
gi|8600860|ref|Os12g08980|
gi|8600861|ref|Os12g08990|
gi|8600863|ref|Os12g09010|
gi|8600866|ref|Os12g09040|

gi|8600867|ref|Os12g09050|
gi|8600869|ref|Os12g09070|
gi|8600873|ref|Os12g09100|
gi|8600876|ref|Os12g09130|
gi|8600877|ref|Os12g09140|
gi|8600878|ref|Os12g09150|
gi|8600879|ref|Os12g09160|
gi|8600880|ref|Os12g09170|
gi|8600881|ref|Os12g09180|
gi|8600883|ref|Os12g09200|
gi|8600886|ref|Os12g09230|
gi|8600895|ref|Os12g09310|
gi|8600898|ref|Os12g09340|
gi|8600900|ref|Os12g09360|
gi|8600901|ref|Os12g09370|
gi|8600902|ref|Os12g09380|
gi|8600908|ref|Os12g09440|
gi|8600909|ref|Os12g09450|
gi|8600911|ref|Os12g09470|
gi|8600915|ref|Os12g09510|
gi|8600916|ref|Os12g09520|
gi|8600919|ref|Os12g09550|
gi|8600920|ref|Os12g09560|
gi|8600924|ref|Os12g09600|
gi|8600929|ref|Os12g09650|
gi|8600930|ref|Os12g09660|
gi|8600932|ref|Os12g09680|
gi|8600933|ref|Os12g09690|
gi|8600939|ref|Os12g09750|
gi|8600942|ref|Os12g09780|
gi|8600945|ref|Os12g09810|
gi|8600946|ref|Os12g09820|
gi|8600947|ref|Os12g09830|
gi|8600952|ref|Os12g09880|
gi|8600959|ref|Os12g09950|
gi|8600961|ref|Os12g09970|
gi|8600962|ref|Os12g09980|
gi|8600964|ref|Os12g10000|
gi|8600965|ref|Os12g10010|
gi|8600966|ref|Os12g10020|
gi|8600968|ref|Os12g10040|
gi|8600972|ref|Os12g10080|
gi|8600974|ref|Os12g10100|
gi|8600976|ref|Os12g10120|
gi|8600979|ref|Os12g10150|
gi|8600985|ref|Os12g10210|
gi|8600986|ref|Os12g10220|
gi|8600987|ref|Os12g10230|
gi|8600993|ref|Os12g10290|
gi|8600994|ref|Os12g10300|
gi|8600999|ref|Os12g10350|
gi|8601001|ref|Os12g10370|
gi|8601002|ref|Os12g10380|
gi|8601009|ref|Os12g10450|
gi|8601012|ref|Os12g10480|
gi|8601015|ref|Os12g10510|
gi|8601017|ref|Os12g10530|
gi|8601026|ref|Os12g10620|
gi|8601033|ref|Os12g10690|
gi|8601040|ref|Os12g10750|
gi|8601041|ref|Os12g10760|
gi|8601046|ref|Os12g10810|
gi|8601047|ref|Os12g10820|
gi|8601049|ref|Os12g10840|
gi|8601053|ref|Os12g10880|
gi|8601057|ref|Os12g10920|
gi|8601060|ref|Os12g10950|
gi|8601061|ref|Os12g10960|
gi|8601063|ref|Os12g10980|
gi|8601066|ref|Os12g11010|
gi|8601069|ref|Os12g11040|
gi|8601070|ref|Os12g11050|
gi|8601071|ref|Os12g11060|
gi|8601072|ref|Os12g11070|
gi|8601074|ref|Os12g11090|
gi|8601075|ref|Os12g11100|
gi|8601079|ref|Os12g11140|
gi|8601081|ref|Os12g11160|
gi|8601082|ref|Os12g11170|
gi|8601087|ref|Os12g11220|
gi|8601089|ref|Os12g11240|
gi|8601092|ref|Os12g11270|
gi|8601093|ref|Os12g11280|
gi|8601097|ref|Os12g11320|
gi|8601100|ref|Os12g11350|
gi|8601101|ref|Os12g11360|
gi|8601104|ref|Os12g11390|
gi|8601105|ref|Os12g11400|
gi|8601107|ref|Os12g11420|
gi|8601109|ref|Os12g11440|
gi|8601110|ref|Os12g11450|
gi|8601114|ref|Os12g11490|
gi|8601117|ref|Os12g11520|
gi|8601118|ref|Os12g11530|
gi|8601119|ref|Os12g11540|
gi|8601120|ref|Os12g11550|
gi|8601126|ref|Os12g11610|
gi|8601127|ref|Os12g11620|
gi|8601131|ref|Os12g11660|
gi|8601134|ref|Os12g11690|
gi|8601135|ref|Os12g11700|
gi|8601136|ref|Os12g11710|
gi|8601144|ref|Os12g11790|
gi|8601147|ref|Os12g11820|
gi|8601148|ref|Os12g11830|
gi|8601149|ref|Os12g11840|
gi|8601150|ref|Os12g11850|
gi|8601153|ref|Os12g11870|
gi|8601154|ref|Os12g11880|
gi|8601155|ref|Os12g11890|
gi|8601157|ref|Os12g11910|
gi|8601161|ref|Os12g11950|
gi|8601162|ref|Os12g11960|
gi|8601164|ref|Os12g11980|
gi|8601165|ref|Os12g11990|
gi|8601166|ref|Os12g12000|
gi|8601168|ref|Os12g12020|
gi|8601171|ref|Os12g12050|
gi|8601173|ref|Os12g12070|
gi|8601174|ref|Os12g12080|
gi|8601175|ref|Os12g12090|
gi|8601177|ref|Os12g12110|
gi|8601180|ref|Os12g12140|
gi|8601189|ref|Os12g12220|
gi|8601190|ref|Os12g12230|
gi|8601192|ref|Os12g12250|
gi|8601195|ref|Os12g12280|
gi|8601198|ref|Os12g12310|
gi|8601199|ref|Os12g12320|
gi|8601200|ref|Os12g12330|
gi|8601202|ref|Os12g12350|
gi|8601207|ref|Os12g12390|
gi|8601214|ref|Os12g12460|
gi|8601224|ref|Os12g12550|

gi|8601226|ref|Os12g12570|
gi|8601230|ref|Os12g12610|
gi|8601232|ref|Os12g12630|
gi|8601233|ref|Os12g12640|
gi|8601235|ref|Os12g12660|
gi|8601238|ref|Os12g12690|
gi|8601244|ref|Os12g12750|
gi|8601252|ref|Os12g12830|
gi|8601259|ref|Os12g12900|
gi|8601261|ref|Os12g12920|
gi|8601264|ref|Os12g12950|
gi|8601265|ref|Os12g12960|
gi|8601267|ref|Os12g12980|
gi|8601269|ref|Os12g13000|
gi|8601273|ref|Os12g13040|
gi|8601274|ref|Os12g13050|
gi|8601277|ref|Os12g13080|
gi|8601278|ref|Os12g13090|
gi|8601283|ref|Os12g13140|
gi|8601289|ref|Os12g13190|
gi|8601290|ref|Os12g13200|
gi|8601292|ref|Os12g13220|
gi|8601293|ref|Os12g13230|
gi|8601294|ref|Os12g13240|
gi|8601295|ref|Os12g13250|
gi|8601297|ref|Os12g13270|
gi|8601298|ref|Os12g13280|
gi|8601299|ref|Os12g13290|
gi|8601305|ref|Os12g13350|
gi|8601311|ref|Os12g13410|
gi|8601317|ref|Os12g13470|
gi|8601318|ref|Os12g13480|
gi|8601320|ref|Os12g13500|
gi|8601321|ref|Os12g13510|
gi|8601322|ref|Os12g13520|
gi|8601323|ref|Os12g13530|
gi|8601324|ref|Os12g13540|
gi|8601326|ref|Os12g13560|
gi|8601328|ref|Os12g13580|
gi|8601330|ref|Os12g13600|
gi|8601331|ref|Os12g13610|
gi|8601333|ref|Os12g13630|
gi|8601335|ref|Os12g13650|
gi|8601338|ref|Os12g13680|
gi|8601339|ref|Os12g13690|
gi|8601340|ref|Os12g13700|
gi|8601341|ref|Os12g13710|
gi|8601343|ref|Os12g13730|
gi|8601344|ref|Os12g13740|
gi|8601346|ref|Os12g13760|
gi|8601347|ref|Os12g13770|
gi|8601351|ref|Os12g13810|
gi|8601354|ref|Os12g13840|
gi|8601355|ref|Os12g13850|
gi|8601356|ref|Os12g13860|
gi|8601359|ref|Os12g13890|
gi|8601360|ref|Os12g13890|
gi|8601361|ref|Os12g13900|
gi|8601362|ref|Os12g13910|
gi|8601368|ref|Os12g13970|
gi|8601369|ref|Os12g13980|
gi|8601371|ref|Os12g14000|
gi|8601372|ref|Os12g14010|
gi|8601373|ref|Os12g14020|
gi|8601377|ref|Os12g14060|
gi|8601380|ref|Os12g14090|
gi|8601384|ref|Os12g14130|
gi|8601392|ref|Os12g14210|
gi|8601393|ref|Os12g14220|
gi|8601394|ref|Os12g14230|
gi|8601401|ref|Os12g14300|
gi|8601402|ref|Os12g14310|
gi|8601403|ref|Os12g14320|
gi|8601405|ref|Os12g14340|
gi|8601408|ref|Os12g14370|
gi|8601412|ref|Os12g14410|
gi|8601416|ref|Os12g14450|
gi|8601421|ref|Os12g14500|
gi|8601422|ref|Os12g14510|
gi|8601423|ref|Os12g14520|
gi|8601424|ref|Os12g14530|
gi|8601425|ref|Os12g14540|
gi|8601426|ref|Os12g14550|
gi|8601427|ref|Os12g14560|
gi|8601428|ref|Os12g14570|
gi|8601430|ref|Os12g14590|
gi|8601431|ref|Os12g14600|
gi|8601433|ref|Os12g14620|
gi|8601437|ref|Os12g14660|
gi|8601438|ref|Os12g14670|
gi|8601441|ref|Os12g14700|
gi|8601442|ref|Os12g14710|
gi|8601443|ref|Os12g14720|
gi|8601444|ref|Os12g14730|
gi|8601447|ref|Os12g14760|
gi|8601448|ref|Os12g14770|
gi|8601452|ref|Os12g14810|
gi|8601457|ref|Os12g14850|
gi|8601458|ref|Os12g14860|
gi|8601459|ref|Os12g14870|
gi|8601463|ref|Os12g14910|
gi|8601464|ref|Os12g14920|
gi|8601470|ref|Os12g14980|
gi|8601471|ref|Os12g14990|
gi|8601485|ref|Os12g15130|
gi|8601489|ref|Os12g15170|
gi|8601490|ref|Os12g15180|
gi|8601493|ref|Os12g15210|
gi|8601496|ref|Os12g15240|
gi|8601497|ref|Os12g15250|
gi|8601498|ref|Os12g15260|
gi|8601499|ref|Os12g15270|
gi|8601500|ref|Os12g15280|
gi|8601502|ref|Os12g15300|
gi|8601504|ref|Os12g15320|
gi|8601505|ref|Os12g15330|
gi|8601508|ref|Os12g15360|
gi|8601509|ref|Os12g15370|
gi|8601510|ref|Os12g15380|
gi|8601511|ref|Os12g15390|
gi|8601513|ref|Os12g15410|
gi|8601515|ref|Os12g15430|
gi|8601521|ref|Os12g15480|
gi|8601524|ref|Os12g15510|
gi|8601527|ref|Os12g15540|
gi|8601529|ref|Os12g15560|
gi|8601535|ref|Os12g15610|
gi|8601538|ref|Os12g15640|
gi|8601540|ref|Os12g15660|
gi|8601545|ref|Os12g15710|
gi|8601548|ref|Os12g15740|
gi|8601550|ref|Os12g15760|
gi|8601555|ref|Os12g15810|
gi|8601557|ref|Os12g15830|

gi|8601558|ref|Os12g15840|
gi|8601560|ref|Os12g15860|
gi|8601567|ref|Os12g15930|
gi|8601570|ref|Os12g15960|
gi|8601571|ref|Os12g15970|
gi|8601572|ref|Os12g15980|
gi|8601573|ref|Os12g15990|
gi|8601576|ref|Os12g16020|
gi|8601578|ref|Os12g16040|
gi|8601582|ref|Os12g16080|
gi|8601583|ref|Os12g16090|
gi|8601586|ref|Os12g16120|
gi|8601591|ref|Os12g16170|
gi|8601594|ref|Os12g16190|
gi|8601602|ref|Os12g16260|
gi|8601603|ref|Os12g16270|
gi|8601604|ref|Os12g16280|
gi|8601608|ref|Os12g16320|
gi|8601609|ref|Os12g16330|
gi|8601614|ref|Os12g16360|
gi|8601615|ref|Os12g16370|
gi|8601617|ref|Os12g16390|
gi|8601629|ref|Os12g16510|
gi|8601631|ref|Os12g16530|
gi|8601633|ref|Os12g16550|
gi|8601635|ref|Os12g16570|
gi|8601638|ref|Os12g16600|
gi|8601639|ref|Os12g16610|
gi|8601642|ref|Os12g16640|
gi|8601644|ref|Os12g16660|
gi|8601645|ref|Os12g16670|
gi|8601646|ref|Os12g16680|
gi|8601654|ref|Os12g16760|
gi|8601655|ref|Os12g16770|
gi|8601659|ref|Os12g16810|
gi|8601665|ref|Os12g16870|
gi|8601666|ref|Os12g16880|
gi|8601667|ref|Os12g16890|
gi|8601669|ref|Os12g16910|
gi|8601671|ref|Os12g16930|
gi|8601672|ref|Os12g16940|
gi|8601673|ref|Os12g16950|
gi|8601675|ref|Os12g16970|
gi|8601677|ref|Os12g16990|
gi|8601678|ref|Os12g17000|
gi|8601679|ref|Os12g17010|
gi|8601680|ref|Os12g17020|
gi|8601681|ref|Os12g17030|
gi|8601684|ref|Os12g17060|
gi|8601690|ref|Os12g17120|
gi|8601692|ref|Os12g17140|
gi|8601693|ref|Os12g17150|
gi|8601698|ref|Os12g17200|
gi|8601701|ref|Os12g17230|
gi|8601702|ref|Os12g17240|
gi|8601703|ref|Os12g17250|
gi|8601706|ref|Os12g17280|
gi|8601711|ref|Os12g17330|
gi|8601715|ref|Os12g17370|
gi|8601716|ref|Os12g17380|
gi|8601722|ref|Os12g17440|
gi|8601728|ref|Os12g17500|
gi|8601730|ref|Os12g17520|
gi|8601731|ref|Os12g17530|
gi|8601734|ref|Os12g17560|
gi|8601736|ref|Os12g17580|
gi|8601737|ref|Os12g17590|
gi|8601739|ref|Os12g17610|
gi|8601743|ref|Os12g17650|
gi|8601744|ref|Os12g17660|
gi|8601745|ref|Os12g17670|
gi|8601746|ref|Os12g17680|
gi|8601748|ref|Os12g17700|
gi|8601751|ref|Os12g17730|
gi|8601755|ref|Os12g17770|
gi|8601757|ref|Os12g17790|
gi|8601758|ref|Os12g17800|
gi|8601759|ref|Os12g17810|
gi|8601764|ref|Os12g17860|
gi|8601766|ref|Os12g17870|
gi|8601768|ref|Os12g17890|
gi|8601771|ref|Os12g17920|
gi|8601772|ref|Os12g17930|
gi|8601773|ref|Os12g17940|
gi|8601774|ref|Os12g17950|
gi|8601775|ref|Os12g17960|
gi|8601779|ref|Os12g18000|
gi|8601786|ref|Os12g18070|
gi|8601788|ref|Os12g18090|
gi|8601798|ref|Os12g18160|
gi|8601799|ref|Os12g18170|
gi|8601805|ref|Os12g18230|
gi|8601806|ref|Os12g18240|
gi|8601809|ref|Os12g18270|
gi|8601810|ref|Os12g18280|
gi|8601811|ref|Os12g18290|
gi|8601815|ref|Os12g18330|
gi|8601816|ref|Os12g18340|
gi|8601824|ref|Os12g18410|
gi|8601825|ref|Os12g18410|
gi|8601827|ref|Os12g18430|
gi|8601829|ref|Os12g18450|
gi|8601833|ref|Os12g18490|
gi|8601834|ref|Os12g18500|
gi|8601835|ref|Os12g18510|
gi|8601836|ref|Os12g18520|
gi|8601839|ref|Os12g18550|
gi|8601841|ref|Os12g18570|
gi|8601842|ref|Os12g18580|
gi|8601846|ref|Os12g18620|
gi|8601855|ref|Os12g18670|
gi|8601856|ref|Os12g18680|
gi|8601857|ref|Os12g18690|
gi|8601858|ref|Os12g18700|
gi|8601859|ref|Os12g18710|
gi|8601860|ref|Os12g18720|
gi|8601868|ref|Os12g18780|
gi|8601869|ref|Os12g18790|
gi|8601871|ref|Os12g18810|
gi|8601873|ref|Os12g18830|
gi|8601875|ref|Os12g18850|
gi|8601877|ref|Os12g18870|
gi|8601879|ref|Os12g18890|
gi|8601882|ref|Os12g18920|
gi|8601883|ref|Os12g18930|
gi|8601884|ref|Os12g18940|
gi|8601889|ref|Os12g18980|
gi|8601890|ref|Os12g18990|
gi|8601896|ref|Os12g19050|
gi|8601897|ref|Os12g19060|
gi|8601899|ref|Os12g19080|
gi|8601901|ref|Os12g19100|
gi|8601902|ref|Os12g19110|
gi|8601903|ref|Os12g19120|

gi|8601905|ref|Os12g19140|
gi|8601907|ref|Os12g19160|
gi|8601908|ref|Os12g19170|
gi|8601912|ref|Os12g19210|
gi|8601914|ref|Os12g19230|
gi|8601915|ref|Os12g19240|
gi|8601918|ref|Os12g19270|
gi|8601919|ref|Os12g19280|
gi|8601924|ref|Os12g19320|
gi|8601925|ref|Os12g19330|
gi|8601928|ref|Os12g19360|
gi|8601937|ref|Os12g19440|
gi|8601938|ref|Os12g19450|
gi|8601946|ref|Os12g19510|
gi|8601949|ref|Os12g19540|
gi|8601951|ref|Os12g19560|
gi|8601952|ref|Os12g19570|
gi|8601955|ref|Os12g19600|
gi|8601956|ref|Os12g19610|
gi|8601961|ref|Os12g19660|
gi|8601963|ref|Os12g19680|
gi|8601964|ref|Os12g19690|
gi|8601966|ref|Os12g19710|
gi|8601978|ref|Os12g19820|
gi|8601983|ref|Os12g19870|
gi|8601984|ref|Os12g19880|
gi|8601987|ref|Os12g19910|
gi|8601989|ref|Os12g19930|
gi|8601992|ref|Os12g19960|
gi|8601993|ref|Os12g19970|
gi|8601999|ref|Os12g20030|
gi|8602001|ref|Os12g20050|
gi|8602003|ref|Os12g20070|
gi|8602005|ref|Os12g20090|
gi|8602006|ref|Os12g20100|
gi|8602008|ref|Os12g20120|
gi|8602009|ref|Os12g20130|
gi|8602010|ref|Os12g20140|
gi|8602012|ref|Os12g20160|
gi|8602013|ref|Os12g20180|
gi|8602016|ref|Os12g20200|
gi|8602022|ref|Os12g20260|
gi|8602024|ref|Os12g20280|
gi|8602029|ref|Os12g20330|
gi|8602032|ref|Os12g20360|
gi|8602033|ref|Os12g20370|
gi|8602034|ref|Os12g20380|
gi|8602035|ref|Os12g20390|
gi|8602038|ref|Os12g20410|
gi|8602040|ref|Os12g20430|
gi|8602041|ref|Os12g20440|
gi|8602043|ref|Os12g20460|
gi|8602046|ref|Os12g21480|
gi|8602055|ref|Os12g21570|
gi|8602057|ref|Os12g21590|
gi|8602059|ref|Os12g21610|
gi|8602061|ref|Os12g21630|
gi|8602063|ref|Os12g21650|
gi|8602065|ref|Os12g21670|
gi|8602066|ref|Os12g21680|
gi|8602067|ref|Os12g21690|
gi|8602072|ref|Os12g21740|
gi|8602073|ref|Os12g21750|
gi|8602074|ref|Os12g21760|
gi|8602077|ref|Os12g21790|
gi|8602079|ref|Os12g21810|
gi|8602082|ref|Os12g21840|
gi|8602084|ref|Os12g21860|
gi|8602090|ref|Os12g21920|
gi|8602094|ref|Os12g21960|
gi|8602099|ref|Os12g22010|
gi|8602100|ref|Os12g22020|
gi|8602102|ref|Os12g22040|
gi|8602104|ref|Os12g22060|
gi|8602107|ref|Os12g22090|
gi|8602108|ref|Os12g22090|
gi|8602109|ref|Os12g22100|
gi|8602113|ref|Os12g22140|
gi|8602115|ref|Os12g22160|
gi|8602121|ref|Os12g22220|
gi|8602133|ref|Os12g22340|
gi|8602134|ref|Os12g22350|
gi|8602135|ref|Os12g22360|
gi|8602138|ref|Os12g22390|
gi|8602144|ref|Os12g22450|
gi|8602145|ref|Os12g22460|
gi|8602148|ref|Os12g22490|
gi|8602149|ref|Os12g22500|
gi|8602152|ref|Os12g22530|
gi|8602153|ref|Os12g22540|
gi|8602154|ref|Os12g22550|
gi|8602163|ref|Os12g22640|
gi|8602165|ref|Os12g22660|
gi|8602166|ref|Os12g22670|
gi|8602172|ref|Os12g22730|
gi|8602174|ref|Os12g22750|
gi|8602176|ref|Os12g22770|
gi|8602177|ref|Os12g22780|
gi|8602178|ref|Os12g22790|
gi|8602179|ref|Os12g22800|
gi|8602183|ref|Os12g22840|
gi|8602184|ref|Os12g22850|
gi|8602185|ref|Os12g22860|
gi|8602186|ref|Os12g22870|
gi|8602187|ref|Os12g22880|
gi|8602188|ref|Os12g22890|
gi|8602189|ref|Os12g22900|
gi|8602191|ref|Os12g22920|
gi|8602192|ref|Os12g22920|
gi|8602193|ref|Os12g22930|
gi|8602194|ref|Os12g22940|
gi|8602195|ref|Os12g22950|
gi|8602196|ref|Os12g22960|
gi|8602197|ref|Os12g22970|
gi|8602205|ref|Os12g23050|
gi|8602207|ref|Os12g23070|
gi|8602208|ref|Os12g23080|
gi|8602209|ref|Os12g23090|
gi|8602210|ref|Os12g23100|
gi|8602216|ref|Os12g23160|
gi|8602221|ref|Os12g23210|
gi|8602226|ref|Os12g23260|
gi|8602227|ref|Os12g23270|
gi|8602230|ref|Os12g23290|
gi|8602231|ref|Os12g23300|
gi|8602234|ref|Os12g23330|
gi|8602235|ref|Os12g23340|
gi|8602242|ref|Os12g23410|
gi|8602246|ref|Os12g23450|
gi|8602247|ref|Os12g23460|
gi|8602249|ref|Os12g23480|
gi|8602252|ref|Os12g23510|
gi|8602256|ref|Os12g23550|
gi|8602258|ref|Os12g23570|

gi|8602261|ref|Os12g23600|
gi|8602263|ref|Os12g23620|
gi|8602268|ref|Os12g23670|
gi|8602269|ref|Os12g23680|
gi|8602270|ref|Os12g23690|
gi|8602272|ref|Os12g23710|
gi|8602273|ref|Os12g23720|
gi|8602275|ref|Os12g23740|
gi|8602276|ref|Os12g23750|
gi|8602279|ref|Os12g23780|
gi|8602283|ref|Os12g23820|
gi|8602287|ref|Os12g23860|
gi|8602290|ref|Os12g23890|
gi|8602291|ref|Os12g23900|
gi|8602297|ref|Os12g23960|
gi|8602298|ref|Os12g23970|
gi|8602302|ref|Os12g24010|
gi|8602304|ref|Os12g24030|
gi|8602308|ref|Os12g24070|
gi|8602310|ref|Os12g24090|
gi|8602314|ref|Os12g24130|
gi|8602316|ref|Os12g24150|
gi|8602324|ref|Os12g24230|
gi|8602328|ref|Os12g24270|
gi|8602329|ref|Os12g24280|
gi|8602332|ref|Os12g24310|
gi|8602335|ref|Os12g24340|
gi|8602336|ref|Os12g24350|
gi|8602337|ref|Os12g24360|
gi|8602339|ref|Os12g24380|
gi|8602345|ref|Os12g24440|
gi|8602346|ref|Os12g24450|
gi|8602347|ref|Os12g24460|
gi|8602349|ref|Os12g24480|
gi|8602351|ref|Os12g24500|
gi|8602358|ref|Os12g24570|
gi|8602360|ref|Os12g24590|
gi|8602365|ref|Os12g24640|
gi|8602367|ref|Os12g24660|
gi|8602368|ref|Os12g24670|
gi|8602373|ref|Os12g24720|
gi|8602374|ref|Os12g24730|
gi|8602386|ref|Os12g24850|
gi|8602390|ref|Os12g24890|
gi|8602391|ref|Os12g24900|
gi|8602392|ref|Os12g24910|
gi|8602394|ref|Os12g24930|
gi|8602398|ref|Os12g24970|
gi|8602399|ref|Os12g24980|
gi|8602402|ref|Os12g25010|
gi|8602403|ref|Os12g25020|
gi|8602406|ref|Os12g25050|
gi|8602410|ref|Os12g25090|
gi|8602411|ref|Os12g25090|
gi|8602413|ref|Os12g25110|
gi|8602415|ref|Os12g25130|
gi|8602416|ref|Os12g25140|
gi|8602417|ref|Os12g25150|
gi|8602420|ref|Os12g25180|
gi|8602425|ref|Os12g25230|
gi|8602426|ref|Os12g25240|
gi|8602429|ref|Os12g25270|
gi|8602430|ref|Os12g25280|
gi|8602433|ref|Os12g25310|
gi|8602437|ref|Os12g25350|
gi|8602438|ref|Os12g25360|
gi|8602439|ref|Os12g25370|
gi|8602442|ref|Os12g25400|
gi|8602444|ref|Os12g25420|
gi|8602445|ref|Os12g25430|
gi|8602446|ref|Os12g25440|
gi|8602449|ref|Os12g25470|
gi|8602452|ref|Os12g25500|
gi|8602456|ref|Os12g25540|
gi|8602463|ref|Os12g25610|
gi|8602464|ref|Os12g25620|
gi|8602467|ref|Os12g25650|
gi|8602469|ref|Os12g25670|
gi|8602479|ref|Os12g25750|
gi|8602480|ref|Os12g25760|
gi|8602481|ref|Os12g25770|
gi|8602482|ref|Os12g25780|
gi|8602483|ref|Os12g25790|
gi|8602484|ref|Os12g25800|
gi|8602485|ref|Os12g25810|
gi|8602488|ref|Os12g25840|
gi|8602492|ref|Os12g25880|
gi|8602493|ref|Os12g25890|
gi|8602496|ref|Os12g25920|
gi|8602499|ref|Os12g25950|
gi|8602504|ref|Os12g26000|
gi|8602508|ref|Os12g26040|
gi|8602509|ref|Os12g26050|
gi|8602513|ref|Os12g26090|
gi|8602514|ref|Os12g26100|
gi|8602520|ref|Os12g26160|
gi|8602521|ref|Os12g26170|
gi|8602524|ref|Os12g26200|
gi|8602526|ref|Os12g26220|
gi|8602530|ref|Os12g26260|
gi|8602531|ref|Os12g26270|
gi|8602532|ref|Os12g26280|
gi|8602535|ref|Os12g26300|
gi|8602538|ref|Os12g26330|
gi|8602540|ref|Os12g26350|
gi|8602541|ref|Os12g26360|
gi|8602544|ref|Os12g26390|
gi|8602546|ref|Os12g26410|
gi|8602548|ref|Os12g26430|
gi|8602551|ref|Os12g26460|
gi|8602553|ref|Os12g26480|
gi|8602555|ref|Os12g26490|
gi|8602557|ref|Os12g26510|
gi|8602559|ref|Os12g26530|
gi|8602573|ref|Os12g26670|
gi|8602574|ref|Os12g26680|
gi|8602576|ref|Os12g26700|
gi|8602583|ref|Os12g26770|
gi|8602586|ref|Os12g26800|
gi|8602595|ref|Os12g26890|
gi|8602597|ref|Os12g26910|
gi|8602599|ref|Os12g26930|
gi|8602602|ref|Os12g26960|
gi|8602608|ref|Os12g27020|
gi|8602609|ref|Os12g27030|
gi|8602610|ref|Os12g27040|
gi|8602614|ref|Os12g27080|
gi|8602615|ref|Os12g27090|
gi|8602618|ref|Os12g27110|
gi|8602625|ref|Os12g27180|
gi|8602626|ref|Os12g27190|
gi|8602627|ref|Os12g27200|
gi|8602628|ref|Os12g27210|
gi|8602635|ref|Os12g27280|

gi|8602636|ref|Os12g27290|
gi|8602641|ref|Os12g27340|
gi|8602643|ref|Os12g27360|
gi|8602650|ref|Os12g27430|
gi|8602651|ref|Os12g27440|
gi|8602652|ref|Os12g27450|
gi|8602654|ref|Os12g27470|
gi|8602655|ref|Os12g27480|
gi|8602657|ref|Os12g27500|
gi|8602663|ref|Os12g27540|
gi|8602664|ref|Os12g27550|
gi|8602665|ref|Os12g27560|
gi|8602666|ref|Os12g27570|
gi|8602668|ref|Os12g27590|
gi|8602674|ref|Os12g27650|
gi|8602675|ref|Os12g27660|
gi|8602676|ref|Os12g27670|
gi|8602677|ref|Os12g27680|
gi|8602678|ref|Os12g27690|
gi|8602681|ref|Os12g27720|
gi|8602683|ref|Os12g27740|
gi|8602684|ref|Os12g27750|
gi|8602686|ref|Os12g27770|
gi|8602690|ref|Os12g27810|
gi|8602691|ref|Os12g27820|
gi|8602693|ref|Os12g27840|
gi|8602694|ref|Os12g27850|
gi|8602695|ref|Os12g27860|
gi|8602698|ref|Os12g27890|
gi|8602699|ref|Os12g27900|
gi|8602701|ref|Os12g27920|
gi|8602703|ref|Os12g27940|
gi|8602704|ref|Os12g27950|
gi|8602705|ref|Os12g27960|
gi|8602706|ref|Os12g27970|
gi|8602709|ref|Os12g28000|
gi|8602710|ref|Os12g28010|
gi|8602711|ref|Os12g28020|
gi|8602712|ref|Os12g28030|
gi|8602715|ref|Os12g28060|
gi|8602717|ref|Os12g28080|
gi|8602718|ref|Os12g28090|
gi|8602720|ref|Os12g28110|
gi|8602722|ref|Os12g28130|
gi|8602726|ref|Os12g28170|
gi|8602730|ref|Os12g28210|
gi|8602732|ref|Os12g28230|
gi|8602739|ref|Os12g28300|
gi|8602740|ref|Os12g28310|
gi|8602746|ref|Os12g28370|
gi|8602748|ref|Os12g28390|
gi|8602752|ref|Os12g28430|
gi|8602753|ref|Os12g28440|
gi|8602757|ref|Os12g28480|
gi|8602759|ref|Os12g28500|
gi|8602761|ref|Os12g28520|
gi|8602763|ref|Os12g28540|
gi|8602769|ref|Os12g28600|
gi|8602774|ref|Os12g28650|
gi|8602775|ref|Os12g28660|
gi|8602777|ref|Os12g28680|
gi|8602779|ref|Os12g28700|
gi|8602781|ref|Os12g28720|
gi|8602785|ref|Os12g28760|
gi|8602787|ref|Os12g28780|
gi|8602788|ref|Os12g28790|
gi|8602791|ref|Os12g28820|
gi|8602793|ref|Os12g28840|
gi|8602795|ref|Os12g28860|
gi|8602796|ref|Os12g28870|
gi|8602800|ref|Os12g28910|
gi|8602802|ref|Os12g28930|
gi|8602804|ref|Os12g28950|
gi|8602807|ref|Os12g28980|
gi|8602810|ref|Os12g29010|
gi|8602813|ref|Os12g29040|
gi|8602814|ref|Os12g29050|
gi|8602817|ref|Os12g29080|
gi|8602820|ref|Os12g29110|
gi|8602823|ref|Os12g29140|
gi|8602825|ref|Os12g29160|
gi|8602826|ref|Os12g29170|
gi|8602827|ref|Os12g29180|
gi|8602833|ref|Os12g29240|
gi|8602836|ref|Os12g29270|
gi|8602841|ref|Os12g29320|
gi|8602843|ref|Os12g29340|
gi|8602848|ref|Os12g29390|
gi|8602850|ref|Os12g29410|
gi|8602851|ref|Os12g29420|
gi|8602853|ref|Os12g29440|
gi|8602855|ref|Os12g29460|
gi|8602860|ref|Os12g29510|
gi|8602864|ref|Os12g29540|
gi|8602869|ref|Os12g29590|
gi|8602870|ref|Os12g29600|
gi|8602878|ref|Os12g29680|
gi|8602882|ref|Os12g29720|
gi|8602883|ref|Os12g29730|
gi|8602884|ref|Os12g29740|
gi|8602888|ref|Os12g29780|
gi|8602889|ref|Os12g29790|
gi|8602892|ref|Os12g29820|
gi|8602893|ref|Os12g29830|
gi|8602894|ref|Os12g29840|
gi|8602898|ref|Os12g29880|
gi|8602912|ref|Os12g30000|
gi|8602913|ref|Os12g30010|
gi|8602924|ref|Os12g30110|
gi|8602926|ref|Os12g30130|
gi|8602929|ref|Os12g30160|
gi|8602930|ref|Os12g30170|
gi|8602932|ref|Os12g30190|
gi|8602934|ref|Os12g30210|
gi|8602936|ref|Os12g30230|
gi|8602940|ref|Os12g30270|
gi|8602945|ref|Os12g30320|
gi|8602946|ref|Os12g30330|
gi|8602951|ref|Os12g30380|
gi|8602954|ref|Os12g30410|
gi|8602956|ref|Os12g30430|
gi|8602958|ref|Os12g30450|
gi|8602959|ref|Os12g30460|
gi|8602960|ref|Os12g30470|
gi|8602961|ref|Os12g30480|
gi|8602969|ref|Os12g30560|
gi|8602971|ref|Os12g30580|
gi|8602978|ref|Os12g30650|
gi|8602979|ref|Os12g30660|
gi|8602982|ref|Os12g30690|
gi|8602984|ref|Os12g30710|
gi|8602986|ref|Os12g30730|
gi|8602987|ref|Os12g30740|
gi|8602988|ref|Os12g30750|

gi|8602994|ref|Os12g30810|
gi|8602997|ref|Os12g30840|
gi|8602999|ref|Os12g30860|
gi|8603000|ref|Os12g30870|
gi|8603005|ref|Os12g30920|
gi|8603006|ref|Os12g30930|
gi|8603007|ref|Os12g30940|
gi|8603008|ref|Os12g30950|
gi|8603009|ref|Os12g30960|
gi|8603010|ref|Os12g30970|
gi|8603011|ref|Os12g30980|
gi|8603012|ref|Os12g30990|
gi|8603019|ref|Os12g31060|
gi|8603021|ref|Os12g31080|
gi|8603022|ref|Os12g31090|
gi|8603023|ref|Os12g31100|
gi|8603024|ref|Os12g31110|
gi|8603035|ref|Os12g31220|
gi|8603037|ref|Os12g31240|
gi|8603039|ref|Os12g31260|
gi|8603040|ref|Os12g31270|
gi|8603042|ref|Os12g31290|
gi|8603043|ref|Os12g31300|
gi|8603044|ref|Os12g31310|
gi|8603046|ref|Os12g31330|
gi|8603047|ref|Os12g31340|
gi|8603049|ref|Os12g31360|
gi|8603052|ref|Os12g31390|
gi|8603053|ref|Os12g31400|
gi|8603055|ref|Os12g31420|
gi|8603056|ref|Os12g31430|
gi|8603064|ref|Os12g31510|
gi|8603065|ref|Os12g31520|
gi|8603068|ref|Os12g31550|
gi|8603071|ref|Os12g31580|
gi|8603081|ref|Os12g31680|
gi|8603082|ref|Os12g31690|
gi|8603085|ref|Os12g31720|
gi|8603088|ref|Os12g31750|
gi|8603090|ref|Os12g31770|
gi|8603106|ref|Os12g31870|
gi|8603109|ref|Os12g31900|
gi|8603110|ref|Os12g31910|
gi|8603113|ref|Os12g31940|
gi|8603114|ref|Os12g31950|
gi|8603120|ref|Os12g32010|
gi|8603121|ref|Os12g32020|
gi|8603122|ref|Os12g32030|
gi|8603124|ref|Os12g32050|
gi|8603125|ref|Os12g32060|
gi|8603127|ref|Os12g32080|
gi|8603134|ref|Os12g32150|
gi|8603135|ref|Os12g32160|
gi|8603136|ref|Os12g32170|
gi|8603146|ref|Os12g32260|
gi|8603147|ref|Os12g32270|
gi|8603150|ref|Os12g32300|
gi|8603151|ref|Os12g32310|
gi|8603152|ref|Os12g32320|
gi|8603153|ref|Os12g32330|
gi|8603154|ref|Os12g32340|
gi|8603156|ref|Os12g32350|
gi|8603157|ref|Os12g32370|
gi|8603159|ref|Os12g32390|
gi|8603165|ref|Os12g32450|
gi|8603167|ref|Os12g32470|
gi|8603168|ref|Os12g32480|
gi|8603169|ref|Os12g32490|
gi|8603171|ref|Os12g32510|
gi|8603172|ref|Os12g32520|
gi|8603173|ref|Os12g32530|
gi|8603174|ref|Os12g32540|
gi|8603175|ref|Os12g32550|
gi|8603176|ref|Os12g32560|
gi|8603177|ref|Os12g32570|
gi|8603178|ref|Os12g32580|
gi|8603180|ref|Os12g32600|
gi|8603181|ref|Os12g32610|
gi|8603184|ref|Os12g32630|
gi|8603192|ref|Os12g32700|
gi|8603199|ref|Os12g32770|
gi|8603200|ref|Os12g32780|
gi|8603203|ref|Os12g32810|
gi|8603205|ref|Os12g32830|
gi|8603206|ref|Os12g32840|
gi|8603208|ref|Os12g32860|
gi|8603209|ref|Os12g32870|
gi|8603211|ref|Os12g32890|
gi|8603213|ref|Os12g32910|
gi|8603218|ref|Os12g32960|
gi|8603225|ref|Os12g33030|
gi|8603226|ref|Os12g33040|
gi|8603233|ref|Os12g33110|
gi|8603234|ref|Os12g33120|
gi|8603235|ref|Os12g33130|
gi|8603237|ref|Os12g33150|
gi|8603240|ref|Os12g33180|
gi|8603247|ref|Os12g33250|
gi|8603249|ref|Os12g33270|
gi|8603251|ref|Os12g33290|
gi|8603253|ref|Os12g33310|
gi|8603257|ref|Os12g33350|
gi|8603262|ref|Os12g33400|
gi|8603263|ref|Os12g33410|
gi|8603265|ref|Os12g33430|
gi|8603269|ref|Os12g33470|
gi|8603270|ref|Os12g33480|
gi|8603273|ref|Os12g33510|
gi|8603276|ref|Os12g33540|
gi|8603277|ref|Os12g33550|
gi|8603279|ref|Os12g33570|
gi|8603295|ref|Os12g33730|
gi|8603297|ref|Os12g33750|
gi|8603298|ref|Os12g33760|
gi|8603299|ref|Os12g33770|
gi|8603300|ref|Os12g33780|
gi|8603301|ref|Os12g33790|
gi|8603303|ref|Os12g33810|
gi|8603306|ref|Os12g33840|
gi|8603308|ref|Os12g33860|
gi|8603310|ref|Os12g33880|
gi|8603314|ref|Os12g33920|
gi|8603316|ref|Os12g33940|
gi|8603319|ref|Os12g33970|
gi|8603328|ref|Os12g34060|
gi|8603332|ref|Os12g34100|
gi|8603334|ref|Os12g34120|
gi|8603337|ref|Os12g34150|
gi|8603339|ref|Os12g34170|
gi|8603345|ref|Os12g34230|
gi|8603350|ref|Os12g34280|
gi|8603364|ref|Os12g34400|
gi|8603367|ref|Os12g34430|
gi|8603368|ref|Os12g34440|

gi|8603371|ref|Os12g34470|
gi|8603372|ref|Os12g34480|
gi|8603373|ref|Os12g34490|
gi|8603374|ref|Os12g34500|
gi|8603380|ref|Os12g34560|
gi|8603386|ref|Os12g34620|
gi|8603388|ref|Os12g34640|
gi|8603390|ref|Os12g34660|
gi|8603392|ref|Os12g34680|
gi|8603393|ref|Os12g34690|
gi|8603396|ref|Os12g34720|
gi|8603399|ref|Os12g34750|
gi|8603402|ref|Os12g34780|
gi|8603403|ref|Os12g34790|
gi|8603404|ref|Os12g34800|
gi|8603405|ref|Os12g34810|
gi|8603407|ref|Os12g34830|
gi|8603414|ref|Os12g34900|
gi|8603415|ref|Os12g34910|
gi|8603417|ref|Os12g34930|
gi|8603418|ref|Os12g34940|
gi|8603420|ref|Os12g34960|
gi|8603426|ref|Os12g35020|
gi|8603430|ref|Os12g35050|
gi|8603431|ref|Os12g35060|
gi|8603432|ref|Os12g35070|
gi|8603433|ref|Os12g35080|
gi|8603434|ref|Os12g35090|
gi|8603439|ref|Os12g35140|
gi|8603441|ref|Os12g35160|
gi|8603442|ref|Os12g35170|
gi|8603443|ref|Os12g35180|
gi|8603448|ref|Os12g35230|
gi|8603451|ref|Os12g35260|
gi|8603452|ref|Os12g35270|
gi|8603456|ref|Os12g35310|
gi|8603464|ref|Os12g35390|
gi|8603467|ref|Os12g35420|
gi|8603469|ref|Os12g35440|
gi|8603470|ref|Os12g35450|
gi|8603472|ref|Os12g35470|
gi|8603473|ref|Os12g35480|
gi|8603474|ref|Os12g35490|
gi|8603475|ref|Os12g35500|
gi|8603476|ref|Os12g35510|
gi|8603477|ref|Os12g35520|
gi|8603478|ref|Os12g35530|
gi|8603487|ref|Os12g35590|
gi|8603497|ref|Os12g35680|
gi|8603499|ref|Os12g35700|
gi|8603504|ref|Os12g35750|
gi|8603506|ref|Os12g35770|
gi|8603508|ref|Os12g35790|
gi|8603511|ref|Os12g35820|
gi|8603512|ref|Os12g35830|
gi|8603514|ref|Os12g35850|
gi|8603515|ref|Os12g35860|
gi|8603516|ref|Os12g35870|
gi|8603520|ref|Os12g35910|
gi|8603521|ref|Os12g35920|
gi|8603524|ref|Os12g35950|
gi|8603527|ref|Os12g35980|
gi|8603528|ref|Os12g35990|
gi|8603529|ref|Os12g36000|
gi|8603530|ref|Os12g36010|
gi|8603532|ref|Os12g36030|
gi|8603534|ref|Os12g36050|
gi|8603535|ref|Os12g36060|
gi|8603536|ref|Os12g36070|
gi|8603537|ref|Os12g36080|
gi|8603542|ref|Os12g36130|
gi|8603545|ref|Os12g36160|
gi|8603548|ref|Os12g36190|
gi|8603554|ref|Os12g36250|
gi|8603556|ref|Os12g36270|
gi|8603557|ref|Os12g36280|
gi|8603558|ref|Os12g36290|
gi|8603559|ref|Os12g36300|
gi|8603560|ref|Os12g36310|
gi|8603561|ref|Os12g36320|
gi|8603562|ref|Os12g36330|
gi|8603563|ref|Os12g36340|
gi|8603572|ref|Os12g36430|
gi|8603573|ref|Os12g36430|
gi|8603575|ref|Os12g36450|
gi|8603576|ref|Os12g36460|
gi|8603580|ref|Os12g36500|
gi|8603584|ref|Os12g36540|
gi|8603595|ref|Os12g36650|
gi|8603598|ref|Os12g36680|
gi|8603600|ref|Os12g36700|
gi|8603601|ref|Os12g36710|
gi|8603611|ref|Os12g36800|
gi|8603613|ref|Os12g36820|
gi|8603630|ref|Os12g36960|
gi|8603631|ref|Os12g36970|
gi|8603632|ref|Os12g36980|
gi|8603638|ref|Os12g37040|
gi|8603639|ref|Os12g37050|
gi|8603642|ref|Os12g37080|
gi|8603643|ref|Os12g37090|
gi|8603646|ref|Os12g37120|
gi|8603655|ref|Os12g37210|
gi|8603657|ref|Os12g37230|
gi|8603659|ref|Os12g37250|
gi|8603666|ref|Os12g37310|
gi|8603680|ref|Os12g37440|
gi|8603683|ref|Os12g37470|
gi|8603688|ref|Os12g37520|
gi|8603701|ref|Os12g37620|
gi|8603708|ref|Os12g37670|
gi|8603709|ref|Os12g37680|
gi|8603715|ref|Os12g37730|
gi|8603717|ref|Os12g37750|
gi|8603721|ref|Os12g37790|
gi|8603722|ref|Os12g37800|
gi|8603724|ref|Os12g37820|
gi|8603742|ref|Os12g37990|
gi|8603750|ref|Os12g38070|
gi|8603757|ref|Os12g38140|
gi|8603762|ref|Os12g38190|
gi|8603763|ref|Os12g38200|
gi|8603765|ref|Os12g38220|
gi|8603766|ref|Os12g38230|
gi|8603767|ref|Os12g38240|
gi|8603768|ref|Os12g38250|
gi|8603769|ref|Os12g38260|
gi|8603770|ref|Os12g38270|
gi|8603776|ref|Os12g38330|
gi|8603777|ref|Os12g38340|
gi|8603778|ref|Os12g38350|
gi|8603779|ref|Os12g38360|
gi|8603780|ref|Os12g38370|
gi|8603783|ref|Os12g38390|

gi|8603784|ref|Os12g38410|
gi|8603785|ref|Os12g38420|
gi|8603791|ref|Os12g38470|
gi|8603792|ref|Os12g38480|
gi|8603796|ref|Os12g38520|
gi|8603798|ref|Os12g38540|
gi|8603799|ref|Os12g38550|
gi|8603800|ref|Os12g38560|
gi|8603802|ref|Os12g38580|
gi|8603814|ref|Os12g38660|
gi|8603818|ref|Os12g38700|
gi|8603820|ref|Os12g38720|
gi|8603834|ref|Os12g38820|
gi|8603835|ref|Os12g38830|
gi|8603841|ref|Os12g38890|
gi|8603843|ref|Os12g38910|
gi|8603846|ref|Os12g38940|
gi|8603847|ref|Os12g38950|
gi|8603850|ref|Os12g38980|
gi|8603852|ref|Os12g39000|
gi|8603856|ref|Os12g39040|
gi|8603857|ref|Os12g39050|
gi|8603858|ref|Os12g39060|
gi|8603866|ref|Os12g39130|
gi|8603867|ref|Os12g39140|
gi|8603877|ref|Os12g39230|
gi|8603879|ref|Os12g39250|
gi|8603881|ref|Os12g39270|
gi|8603882|ref|Os12g39280|
gi|8603889|ref|Os12g39350|
gi|8603896|ref|Os12g39410|
gi|8603903|ref|Os12g39430|
gi|8603904|ref|Os12g39440|
gi|8603905|ref|Os12g39440|
gi|8603906|ref|Os12g39450|
gi|8603907|ref|Os12g39460|
gi|8603908|ref|Os12g39470|
gi|8603909|ref|Os12g39480|
gi|8603910|ref|Os12g39490|
gi|8603911|ref|Os12g39500|
gi|8603912|ref|Os12g39510|
gi|8603914|ref|Os12g39530|
gi|8603916|ref|Os12g39550|
gi|8603917|ref|Os12g39560|
gi|8603928|ref|Os12g39670|
gi|8603929|ref|Os12g39680|
gi|8603930|ref|Os12g39690|
gi|8603932|ref|Os12g39710|
gi|8603933|ref|Os12g39710|
gi|8603934|ref|Os12g39720|
gi|8603935|ref|Os12g39730|
gi|8603937|ref|Os12g39750|
gi|8603939|ref|Os12g39770|
gi|8603940|ref|Os12g39780|
gi|8603941|ref|Os12g39790|
gi|8603943|ref|Os12g39810|
gi|8603944|ref|Os12g39820|
gi|8603949|ref|Os12g39870|
gi|8603950|ref|Os12g39880|
gi|8603951|ref|Os12g39890|
gi|8603953|ref|Os12g39910|
gi|8603954|ref|Os12g39920|
gi|8603956|ref|Os12g39940|
gi|8603957|ref|Os12g39950|
gi|8603958|ref|Os12g39960|
gi|8603959|ref|Os12g39970|
gi|8603962|ref|Os12g40000|
gi|8603977|ref|Os12g40150|
gi|8603978|ref|Os12g40160|
gi|8603979|ref|Os12g40170|
gi|8603980|ref|Os12g40180|
gi|8603983|ref|Os12g40210|
gi|8603984|ref|Os12g40220|
gi|8603986|ref|Os12g40240|
gi|8603989|ref|Os12g40270|
gi|8603993|ref|Os12g40290|
gi|8603997|ref|Os12g40330|
gi|8604002|ref|Os12g40380|
gi|8604004|ref|Os12g40400|
gi|8604005|ref|Os12g40410|
gi|8604039|ref|Os12g40720|
gi|8604041|ref|Os12g40740|
gi|8604042|ref|Os12g40750|
gi|8604043|ref|Os12g40760|
gi|8604047|ref|Os12g40800|
gi|8604051|ref|Os12g40840|
gi|8604052|ref|Os12g40840|
gi|8604053|ref|Os12g40850|
gi|8604060|ref|Os12g40910|
gi|8604063|ref|Os12g40930|
gi|8604064|ref|Os12g40940|
gi|8604065|ref|Os12g40950|
gi|8604066|ref|Os12g40960|
gi|8604068|ref|Os12g40980|
gi|8604069|ref|Os12g40990|
gi|8604073|ref|Os12g41030|
gi|8604075|ref|Os12g41050|
gi|8604077|ref|Os12g41070|
gi|8604080|ref|Os12g41100|
gi|8604082|ref|Os12g41120|
gi|8604083|ref|Os12g41130|
gi|8604085|ref|Os12g41150|
gi|8604095|ref|Os12g41240|
gi|8604105|ref|Os12g41320|
gi|8604106|ref|Os12g41330|
gi|8604107|ref|Os12g41340|
gi|8604109|ref|Os12g41360|
gi|8604118|ref|Os12g41430|
gi|8604119|ref|Os12g41430|
gi|8604120|ref|Os12g41430|
gi|8604124|ref|Os12g41470|
gi|8604125|ref|Os12g41480|
gi|8604132|ref|Os12g41550|
gi|8604133|ref|Os12g41560|
gi|8604134|ref|Os12g41570|
gi|8604135|ref|Os12g41580|
gi|8604137|ref|Os12g41600|
gi|8604138|ref|Os12g41610|
gi|8604141|ref|Os12g41640|
gi|8604156|ref|Os12g41770|
gi|8604160|ref|Os12g41800|
gi|8604164|ref|Os12g41840|
gi|8604165|ref|Os12g41850|
gi|8604176|ref|Os12g41930|
gi|8604183|ref|Os12g41980|
gi|8604185|ref|Os12g42000|
gi|8604186|ref|Os12g42010|
gi|8604193|ref|Os12g42080|
gi|8604203|ref|Os12g42170|
gi|8604208|ref|Os12g42220|
gi|8604209|ref|Os12g42220|
gi|8604211|ref|Os12g42240|
gi|8604217|ref|Os12g42290|
gi|8604221|ref|Os12g42330|

gi|8604222|ref|Os12g42340|
gi|8604224|ref|Os12g42360|
gi|8604226|ref|Os12g42380|
gi|8604227|ref|Os12g42390|
gi|8604229|ref|Os12g42410|
gi|8604235|ref|Os12g42470|
gi|8604236|ref|Os12g42480|
gi|8604237|ref|Os12g42490|
gi|8604238|ref|Os12g42500|
gi|8604239|ref|Os12g42510|
gi|8604241|ref|Os12g42530|
gi|8604244|ref|Os12g42560|
gi|8604247|ref|Os12g42590|
gi|8604250|ref|Os12g42620|
gi|8604253|ref|Os12g42650|
gi|8604255|ref|Os12g42670|
gi|8604259|ref|Os12g42710|
gi|8604264|ref|Os12g42740|
gi|8604267|ref|Os12g42770|
gi|8604268|ref|Os12g42780|
gi|8604287|ref|Os12g42920|
gi|8604290|ref|Os12g42950|
gi|8604296|ref|Os12g43010|
gi|8604298|ref|Os12g43030|
gi|8604304|ref|Os12g43090|
gi|8604310|ref|Os12g43140|
gi|8604311|ref|Os12g43150|
gi|8604312|ref|Os12g43160|
gi|8604313|ref|Os12g43170|
gi|8604315|ref|Os12g43190|
gi|8604316|ref|Os12g43200|
gi|8604317|ref|Os12g43210|
gi|8604318|ref|Os12g43220|
gi|8604320|ref|Os12g43240|
gi|8604322|ref|Os12g43260|
gi|8604327|ref|Os12g43310|
gi|8604328|ref|Os12g43320|
gi|8604329|ref|Os12g43330|
gi|8604331|ref|Os12g43350|
gi|8604336|ref|Os12g43400|
gi|8604338|ref|Os12g43420|
gi|8604347|ref|Os12g43510|
gi|8604350|ref|Os12g43540|
gi|8604353|ref|Os12g43570|
gi|8604354|ref|Os12g43580|
gi|8604357|ref|Os12g43610|
gi|8604366|ref|Os12g43690|
gi|8604368|ref|Os12g43710|
gi|8604372|ref|Os12g43750|
gi|8604373|ref|Os12g43760|
gi|8604375|ref|Os12g43780|
gi|8604376|ref|Os12g43790|
gi|8604377|ref|Os12g43800|
gi|8604378|ref|Os12g43810|
gi|8604384|ref|Os12g43870|
gi|8604389|ref|Os12g43920|
gi|8604394|ref|Os12g43960|
gi|8604396|ref|Os12g43980|
gi|8604397|ref|Os12g43990|
gi|8604402|ref|Os12g44040|
gi|8604410|ref|Os12g44120|
gi|8604411|ref|Os12g44130|
gi|8604424|ref|Os12g44260|
gi|8604425|ref|Os12g44270|
gi|8604426|ref|Os12g44280|
gi|8700006|ref|Os11g01060|
gi|8700007|ref|Os11g01070|
gi|8700011|ref|Os11g01110|
gi|8700012|ref|Os11g01120|
gi|8700015|ref|Os11g01150|
gi|8700023|ref|Os11g01230|
gi|8700025|ref|Os11g01250|
gi|8700026|ref|Os11g01260|
gi|8700028|ref|Os11g01280|
gi|8700031|ref|Os11g01310|
gi|8700032|ref|Os11g01320|
gi|8700035|ref|Os11g01350|
gi|8700040|ref|Os11g01400|
gi|8700043|ref|Os11g01430|
gi|8700051|ref|Os11g01500|
gi|8700054|ref|Os11g01520|
gi|8700058|ref|Os11g01560|
gi|8700066|ref|Os11g01640|
gi|8700067|ref|Os11g01650|
gi|8700068|ref|Os11g01660|
gi|8700069|ref|Os11g01670|
gi|8700070|ref|Os11g01680|
gi|8700071|ref|Os11g01690|
gi|8700073|ref|Os11g01710|
gi|8700077|ref|Os11g01750|
gi|8700078|ref|Os11g01760|
gi|8700079|ref|Os11g01770|
gi|8700082|ref|Os11g01800|
gi|8700092|ref|Os11g01900|
gi|8700093|ref|Os11g01910|
gi|8700096|ref|Os11g01940|
gi|8700097|ref|Os11g01950|
gi|8700098|ref|Os11g01960|
gi|8700101|ref|Os11g01990|
gi|8700102|ref|Os11g02000|
gi|8700103|ref|Os11g02010|
gi|8700104|ref|Os11g02020|
gi|8700105|ref|Os11g02030|
gi|8700106|ref|Os11g02040|
gi|8700107|ref|Os11g02050|
gi|8700110|ref|Os11g02080|
gi|8700111|ref|Os11g02090|
gi|8700114|ref|Os11g02120|
gi|8700116|ref|Os11g02140|
gi|8700121|ref|Os11g02170|
gi|8700124|ref|Os11g02200|
gi|8700125|ref|Os11g02210|
gi|8700126|ref|Os11g02220|
gi|8700127|ref|Os11g02230|
gi|8700132|ref|Os11g02280|
gi|8700133|ref|Os11g02290|
gi|8700139|ref|Os11g02330|
gi|8700140|ref|Os11g02330|
gi|8700141|ref|Os11g02340|
gi|8700143|ref|Os11g02360|
gi|8700145|ref|Os11g02380|
gi|8700148|ref|Os11g02410|
gi|8700156|ref|Os11g02490|
gi|8700157|ref|Os11g02500|
gi|8700158|ref|Os11g02510|
gi|8700163|ref|Os11g02560|
gi|8700177|ref|Os11g02680|
gi|8700178|ref|Os11g02690|
gi|8700181|ref|Os11g02720|
gi|8700188|ref|Os11g02790|
gi|8700190|ref|Os11g02810|
gi|8700194|ref|Os11g02850|
gi|8700196|ref|Os11g02870|
gi|8700201|ref|Os11g02920|

gi|8700202|ref|Os11g02930|
gi|8700203|ref|Os11g02940|
gi|8700205|ref|Os11g02960|
gi|8700218|ref|Os11g03080|
gi|8700222|ref|Os11g03120|
gi|8700224|ref|Os11g03140|
gi|8700225|ref|Os11g03150|
gi|8700227|ref|Os11g03170|
gi|8700228|ref|Os11g03180|
gi|8700229|ref|Os11g03190|
gi|8700236|ref|Os11g03260|
gi|8700242|ref|Os11g03320|
gi|8700244|ref|Os11g03340|
gi|8700245|ref|Os11g03350|
gi|8700266|ref|Os11g03560|
gi|8700272|ref|Os11g03620|
gi|8700273|ref|Os11g03630|
gi|8700274|ref|Os11g03640|
gi|8700276|ref|Os11g03660|
gi|8700278|ref|Os11g03680|
gi|8700282|ref|Os11g03720|
gi|8700285|ref|Os11g03750|
gi|8700287|ref|Os11g03770|
gi|8700291|ref|Os11g03800|
gi|8700298|ref|Os11g03870|
gi|8700304|ref|Os11g03930|
gi|8700311|ref|Os11g04000|
gi|8700319|ref|Os11g04050|
gi|8700323|ref|Os11g04090|
gi|8700325|ref|Os11g04110|
gi|8700326|ref|Os11g04120|
gi|8700340|ref|Os11g04250|
gi|8700342|ref|Os11g04270|
gi|8700349|ref|Os11g04340|
gi|8700357|ref|Os11g04420|
gi|8700358|ref|Os11g04430|
gi|8700360|ref|Os11g04450|
gi|8700379|ref|Os11g04630|
gi|8700386|ref|Os11g04700|
gi|8700392|ref|Os11g04750|
gi|8700396|ref|Os11g04790|
gi|8700398|ref|Os11g04810|
gi|8700400|ref|Os11g04830|
gi|8700404|ref|Os11g04870|
gi|8700409|ref|Os11g04920|
gi|8700414|ref|Os11g04970|
gi|8700420|ref|Os11g05020|
gi|8700424|ref|Os11g05060|
gi|8700437|ref|Os11g05180|
gi|8700444|ref|Os11g05250|
gi|8700447|ref|Os11g05270|
gi|8700451|ref|Os11g05310|
gi|8700453|ref|Os11g05330|
gi|8700462|ref|Os11g05420|
gi|8700463|ref|Os11g05430|
gi|8700464|ref|Os11g05440|
gi|8700465|ref|Os11g05450|
gi|8700466|ref|Os11g05460|
gi|8700469|ref|Os11g05490|
gi|8700470|ref|Os11g05500|
gi|8700473|ref|Os11g05530|
gi|8700475|ref|Os11g05550|
gi|8700482|ref|Os11g05620|
gi|8700488|ref|Os11g05680|
gi|8700491|ref|Os11g05710|
gi|8700495|ref|Os11g05750|
gi|8700501|ref|Os11g05810|
gi|8700503|ref|Os11g05830|
gi|8700509|ref|Os11g05870|
gi|8700516|ref|Os11g05940|
gi|8700517|ref|Os11g05950|
gi|8700518|ref|Os11g05960|
gi|8700526|ref|Os11g06030|
gi|8700529|ref|Os11g06050|
gi|8700531|ref|Os11g06070|
gi|8700532|ref|Os11g06080|
gi|8700533|ref|Os11g06090|
gi|8700538|ref|Os11g06120|
gi|8700540|ref|Os11g06150|
gi|8700546|ref|Os11g06200|
gi|8700552|ref|Os11g06250|
gi|8700561|ref|Os11g06330|
gi|8700563|ref|Os11g06350|
gi|8700564|ref|Os11g06360|
gi|8700565|ref|Os11g06370|
gi|8700576|ref|Os11g06430|
gi|8700578|ref|Os11g06450|
gi|8700580|ref|Os11g06470|
gi|8700581|ref|Os11g06480|
gi|8700584|ref|Os11g06510|
gi|8700586|ref|Os11g06530|
gi|8700587|ref|Os11g06540|
gi|8700588|ref|Os11g06550|
gi|8700589|ref|Os11g06560|
gi|8700591|ref|Os11g06580|
gi|8700594|ref|Os11g06610|
gi|8700599|ref|Os11g06660|
gi|8700605|ref|Os11g06710|
gi|8700614|ref|Os11g06800|
gi|8700617|ref|Os11g06830|
gi|8700619|ref|Os11g06850|
gi|8700629|ref|Os11g06950|
gi|8700631|ref|Os11g06970|
gi|8700634|ref|Os11g07000|
gi|8700644|ref|Os11g07070|
gi|8700647|ref|Os11g07100|
gi|8700657|ref|Os11g07200|
gi|8700667|ref|Os11g07290|
gi|8700668|ref|Os11g07300|
gi|8700669|ref|Os11g07310|
gi|8700670|ref|Os11g07320|
gi|8700671|ref|Os11g07330|
gi|8700672|ref|Os11g07340|
gi|8700673|ref|Os11g07350|
gi|8700674|ref|Os11g07360|
gi|8700675|ref|Os11g07370|
gi|8700676|ref|Os11g07380|
gi|8700677|ref|Os11g07390|
gi|8700678|ref|Os11g07400|
gi|8700679|ref|Os11g07410|
gi|8700680|ref|Os11g07420|
gi|8700681|ref|Os11g07430|
gi|8700692|ref|Os11g07530|
gi|8700693|ref|Os11g07540|
gi|8700694|ref|Os11g07550|
gi|8700695|ref|Os11g07560|
gi|8700696|ref|Os11g07570|
gi|8700697|ref|Os11g07580|
gi|8700698|ref|Os11g07590|
gi|8700701|ref|Os11g07620|
gi|8700702|ref|Os11g07630|
gi|8700703|ref|Os11g07640|
gi|8700705|ref|Os11g07660|
gi|8700709|ref|Os11g07700|

gi|8700714|ref|Os11g07750|
gi|8700715|ref|Os11g07760|
gi|8700717|ref|Os11g07780|
gi|8700721|ref|Os11g07820|
gi|8700725|ref|Os11g07860|
gi|8700727|ref|Os11g07880|
gi|8700729|ref|Os11g07900|
gi|8700733|ref|Os11g07940|
gi|8700734|ref|Os11g07950|
gi|8700739|ref|Os11g08000|
gi|8700745|ref|Os11g08060|
gi|8700753|ref|Os11g08130|
gi|8700754|ref|Os11g08140|
gi|8700757|ref|Os11g08170|
gi|8700763|ref|Os11g08220|
gi|8700765|ref|Os11g08240|
gi|8700767|ref|Os11g08260|
gi|8700768|ref|Os11g08270|
gi|8700769|ref|Os11g08280|
gi|8700770|ref|Os11g08290|
gi|8700776|ref|Os11g08350|
gi|8700777|ref|Os11g08360|
gi|8700783|ref|Os11g08420|
gi|8700784|ref|Os11g08430|
gi|8700790|ref|Os11g08490|
gi|8700791|ref|Os11g08500|
gi|8700793|ref|Os11g08520|
gi|8700802|ref|Os11g08600|
gi|8700806|ref|Os11g08640|
gi|8700810|ref|Os11g08680|
gi|8700812|ref|Os11g08700|
gi|8700813|ref|Os11g08710|
gi|8700814|ref|Os11g08720|
gi|8700816|ref|Os11g08740|
gi|8700820|ref|Os11g08780|
gi|8700822|ref|Os11g08800|
gi|8700824|ref|Os11g08820|
gi|8700826|ref|Os11g08840|
gi|8700827|ref|Os11g08850|
gi|8700828|ref|Os11g08860|
gi|8700830|ref|Os11g08880|
gi|8700832|ref|Os11g08900|
gi|8700833|ref|Os11g08910|
gi|8700834|ref|Os11g08920|
gi|8700835|ref|Os11g08930|
gi|8700839|ref|Os11g08970|
gi|8700841|ref|Os11g08990|
gi|8700845|ref|Os11g09030|
gi|8700847|ref|Os11g09050|
gi|8700848|ref|Os11g09060|
gi|8700849|ref|Os11g09070|
gi|8700850|ref|Os11g09080|
gi|8700852|ref|Os11g09100|
gi|8700854|ref|Os11g09120|
gi|8700857|ref|Os11g09150|
gi|8700860|ref|Os11g09180|
gi|8700862|ref|Os11g09200|
gi|8700863|ref|Os11g09210|
gi|8700878|ref|Os11g09350|
gi|8700881|ref|Os11g09380|
gi|8700882|ref|Os11g09390|
gi|8700888|ref|Os11g09450|
gi|8700890|ref|Os11g09470|
gi|8700896|ref|Os11g09530|
gi|8700897|ref|Os11g09540|
gi|8700899|ref|Os11g09560|
gi|8700900|ref|Os11g09570|
gi|8700904|ref|Os11g09610|
gi|8700909|ref|Os11g09650|
gi|8700912|ref|Os11g09680|
gi|8700913|ref|Os11g09680|
gi|8700919|ref|Os11g09740|
gi|8700922|ref|Os11g09770|
gi|8700923|ref|Os11g09780|
gi|8700925|ref|Os11g09800|
gi|8700926|ref|Os11g09810|
gi|8700927|ref|Os11g09820|
gi|8700928|ref|Os11g09830|
gi|8700929|ref|Os11g09840|
gi|8700930|ref|Os11g09850|
gi|8700931|ref|Os11g09860|
gi|8700932|ref|Os11g09870|
gi|8700933|ref|Os11g09880|
gi|8700934|ref|Os11g09890|
gi|8700935|ref|Os11g09900|
gi|8700936|ref|Os11g09910|
gi|8700938|ref|Os11g09930|
gi|8700939|ref|Os11g09940|
gi|8700943|ref|Os11g09980|
gi|8700946|ref|Os11g10010|
gi|8700948|ref|Os11g10030|
gi|8700953|ref|Os11g10080|
gi|8700957|ref|Os11g10120|
gi|8700966|ref|Os11g10210|
gi|8700968|ref|Os11g10230|
gi|8700970|ref|Os11g10250|
gi|8700972|ref|Os11g10270|
gi|8700977|ref|Os11g10320|
gi|8700980|ref|Os11g10350|
gi|8700993|ref|Os11g10470|
gi|8701001|ref|Os11g10530|
gi|8701002|ref|Os11g10540|
gi|8701004|ref|Os11g10560|
gi|8701007|ref|Os11g10590|
gi|8701008|ref|Os11g10600|
gi|8701010|ref|Os11g10620|
gi|8701013|ref|Os11g10650|
gi|8701014|ref|Os11g10660|
gi|8701015|ref|Os11g10670|
gi|8701018|ref|Os11g10700|
gi|8701021|ref|Os11g10730|
gi|8701027|ref|Os11g10790|
gi|8701029|ref|Os11g10810|
gi|8701036|ref|Os11g10880|
gi|8701037|ref|Os11g10890|
gi|8701041|ref|Os11g10930|
gi|8701042|ref|Os11g10940|
gi|8701043|ref|Os11g10950|
gi|8701044|ref|Os11g10960|
gi|8701051|ref|Os11g11030|
gi|8701052|ref|Os11g11040|
gi|8701058|ref|Os11g11080|
gi|8701059|ref|Os11g11090|
gi|8701062|ref|Os11g11120|
gi|8701063|ref|Os11g11130|
gi|8701066|ref|Os11g11160|
gi|8701069|ref|Os11g11190|
gi|8701070|ref|Os11g11200|
gi|8701073|ref|Os11g11230|
gi|8701075|ref|Os11g11250|
gi|8701077|ref|Os11g11270|
gi|8701078|ref|Os11g11280|
gi|8701081|ref|Os11g11310|
gi|8701085|ref|Os11g11350|

gi|8701086|ref|Os11g11360|
gi|8701090|ref|Os11g11400|
gi|8701092|ref|Os11g11420|
gi|8701095|ref|Os11g11450|
gi|8701096|ref|Os11g11460|
gi|8701097|ref|Os11g11470|
gi|8701098|ref|Os11g11480|
gi|8701101|ref|Os11g11510|
gi|8701102|ref|Os11g11520|
gi|8701106|ref|Os11g11560|
gi|8701107|ref|Os11g11570|
gi|8701109|ref|Os11g11590|
gi|8701113|ref|Os11g11630|
gi|8701114|ref|Os11g11640|
gi|8701115|ref|Os11g11650|
gi|8701116|ref|Os11g11660|
gi|8701117|ref|Os11g11670|
gi|8701121|ref|Os11g11710|
gi|8701123|ref|Os11g11730|
gi|8701124|ref|Os11g11740|
gi|8701125|ref|Os11g11750|
gi|8701140|ref|Os11g11900|
gi|8701141|ref|Os11g11910|
gi|8701143|ref|Os11g11930|
gi|8701147|ref|Os11g11970|
gi|8701148|ref|Os11g11980|
gi|8701152|ref|Os11g12020|
gi|8701153|ref|Os11g12030|
gi|8701157|ref|Os11g12070|
gi|8701158|ref|Os11g12080|
gi|8701165|ref|Os11g12150|
gi|8701178|ref|Os11g12280|
gi|8701187|ref|Os11g12370|
gi|8701188|ref|Os11g12380|
gi|8701189|ref|Os11g12390|
gi|8701197|ref|Os11g12470|
gi|8701201|ref|Os11g12510|
gi|8701204|ref|Os11g12540|
gi|8701205|ref|Os11g12550|
gi|8701209|ref|Os11g12590|
gi|8701210|ref|Os11g12600|
gi|8701211|ref|Os11g12610|
gi|8701213|ref|Os11g12630|
gi|8701217|ref|Os11g12670|
gi|8701218|ref|Os11g12680|
gi|8701225|ref|Os11g12750|
gi|8701236|ref|Os11g12860|
gi|8701239|ref|Os11g13380|
gi|8701240|ref|Os11g13390|
gi|8701246|ref|Os11g13450|
gi|8701247|ref|Os11g13460|
gi|8701250|ref|Os11g13490|
gi|8701253|ref|Os11g13520|
gi|8701256|ref|Os11g13550|
gi|8701259|ref|Os11g13580|
gi|8701260|ref|Os11g13590|
gi|8701263|ref|Os11g13620|
gi|8701267|ref|Os11g13660|
gi|8701269|ref|Os11g13680|
gi|8701272|ref|Os11g13710|
gi|8701277|ref|Os11g13760|
gi|8701285|ref|Os11g13830|
gi|8701292|ref|Os11g13880|
gi|8701296|ref|Os11g13900|
gi|8701297|ref|Os11g13910|
gi|8701301|ref|Os11g13950|
gi|8701306|ref|Os11g14000|
gi|8701309|ref|Os11g14030|
gi|8701312|ref|Os11g14060|
gi|8701316|ref|Os11g14100|
gi|8701318|ref|Os11g14120|
gi|8701326|ref|Os11g14190|
gi|8701327|ref|Os11g14190|
gi|8701328|ref|Os11g14200|
gi|8701329|ref|Os11g14210|
gi|8701335|ref|Os11g14250|
gi|8701339|ref|Os11g14290|
gi|8701341|ref|Os11g14310|
gi|8701342|ref|Os11g14320|
gi|8701346|ref|Os11g14360|
gi|8701347|ref|Os11g14370|
gi|8701349|ref|Os11g14390|
gi|8701354|ref|Os11g14440|
gi|8701359|ref|Os11g14490|
gi|8701360|ref|Os11g14500|
gi|8701363|ref|Os11g14530|
gi|8701374|ref|Os11g14620|
gi|8701376|ref|Os11g14640|
gi|8701378|ref|Os11g14660|
gi|8701379|ref|Os11g14670|
gi|8701381|ref|Os11g14690|
gi|8701382|ref|Os11g14700|
gi|8701383|ref|Os11g14710|
gi|8701384|ref|Os11g14720|
gi|8701385|ref|Os11g14730|
gi|8701389|ref|Os11g14770|
gi|8701390|ref|Os11g14780|
gi|8701391|ref|Os11g14790|
gi|8701392|ref|Os11g14800|
gi|8701393|ref|Os11g14810|
gi|8701394|ref|Os11g14820|
gi|8701397|ref|Os11g14850|
gi|8701398|ref|Os11g14860|
gi|8701399|ref|Os11g14870|
gi|8701400|ref|Os11g14880|
gi|8701401|ref|Os11g14890|
gi|8701406|ref|Os11g14920|
gi|8701407|ref|Os11g14930|
gi|8701408|ref|Os11g14940|
gi|8701414|ref|Os11g15000|
gi|8701416|ref|Os11g15020|
gi|8701419|ref|Os11g15050|
gi|8701425|ref|Os11g15110|
gi|8701434|ref|Os11g15200|
gi|8701437|ref|Os11g15230|
gi|8701439|ref|Os11g15250|
gi|8701442|ref|Os11g15280|
gi|8701446|ref|Os11g15320|
gi|8701453|ref|Os11g15390|
gi|8701456|ref|Os11g15420|
gi|8701461|ref|Os11g15470|
gi|8701462|ref|Os11g15480|
gi|8701463|ref|Os11g15490|
gi|8701467|ref|Os11g15530|
gi|8701468|ref|Os11g15540|
gi|8701469|ref|Os11g15550|
gi|8701472|ref|Os11g15580|
gi|8701473|ref|Os11g15590|
gi|8701474|ref|Os11g15600|
gi|8701475|ref|Os11g15610|
gi|8701480|ref|Os11g15660|
gi|8701482|ref|Os11g15680|
gi|8701485|ref|Os11g15710|
gi|8701486|ref|Os11g15720|

gi|8701487|ref|Os11g15730|
gi|8701488|ref|Os11g15740|
gi|8701496|ref|Os11g16300|
gi|8701500|ref|Os11g16340|
gi|8701501|ref|Os11g16350|
gi|8701502|ref|Os11g16360|
gi|8701504|ref|Os11g16380|
gi|8701512|ref|Os11g16440|
gi|8701518|ref|Os11g16500|
gi|8701519|ref|Os11g16510|
gi|8701520|ref|Os11g16520|
gi|8701525|ref|Os11g16570|
gi|8701528|ref|Os11g16600|
gi|8701533|ref|Os11g16650|
gi|8701534|ref|Os11g16660|
gi|8701535|ref|Os11g16670|
gi|8701537|ref|Os11g16690|
gi|8701538|ref|Os11g16700|
gi|8701540|ref|Os11g16720|
gi|8701546|ref|Os11g16780|
gi|8701548|ref|Os11g16800|
gi|8701552|ref|Os11g16840|
gi|8701553|ref|Os11g16850|
gi|8701562|ref|Os11g16940|
gi|8701563|ref|Os11g16950|
gi|8701565|ref|Os11g16970|
gi|8701567|ref|Os11g16990|
gi|8701569|ref|Os11g17010|
gi|8701571|ref|Os11g17030|
gi|8701572|ref|Os11g17040|
gi|8701574|ref|Os11g17060|
gi|8701575|ref|Os11g17070|
gi|8701577|ref|Os11g17090|
gi|8701578|ref|Os11g17100|
gi|8701584|ref|Os11g17160|
gi|8701589|ref|Os11g17210|
gi|8701590|ref|Os11g17220|
gi|8701593|ref|Os11g17250|
gi|8701597|ref|Os11g17290|
gi|8701602|ref|Os11g17320|
gi|8701604|ref|Os11g17340|
gi|8701605|ref|Os11g17350|
gi|8701606|ref|Os11g17360|
gi|8701615|ref|Os11g17450|
gi|8701620|ref|Os11g17500|
gi|8701622|ref|Os11g17520|
gi|8701627|ref|Os11g17550|
gi|8701628|ref|Os11g17560|
gi|8701629|ref|Os11g17570|
gi|8701631|ref|Os11g17590|
gi|8701634|ref|Os11g17620|
gi|8701635|ref|Os11g17630|
gi|8701636|ref|Os11g17640|
gi|8701637|ref|Os11g17650|
gi|8701638|ref|Os11g17660|
gi|8701639|ref|Os11g17670|
gi|8701640|ref|Os11g17680|
gi|8701642|ref|Os11g17700|
gi|8701645|ref|Os11g17730|
gi|8701648|ref|Os11g17760|
gi|8701652|ref|Os11g17800|
gi|8701653|ref|Os11g17810|
gi|8701654|ref|Os11g17820|
gi|8701658|ref|Os11g17860|
gi|8701659|ref|Os11g17870|
gi|8701660|ref|Os11g17880|
gi|8701663|ref|Os11g17910|
gi|8701664|ref|Os11g17920|
gi|8701666|ref|Os11g17940|
gi|8701670|ref|Os11g17980|
gi|8701672|ref|Os11g18000|
gi|8701673|ref|Os11g18010|
gi|8701674|ref|Os11g18020|
gi|8701678|ref|Os11g18060|
gi|8701680|ref|Os11g18080|
gi|8701681|ref|Os11g18090|
gi|8701682|ref|Os11g18100|
gi|8701685|ref|Os11g18130|
gi|8701688|ref|Os11g18160|
gi|8701691|ref|Os11g18190|
gi|8701692|ref|Os11g18200|
gi|8701694|ref|Os11g18220|
gi|8701695|ref|Os11g18230|
gi|8701699|ref|Os11g18270|
gi|8701700|ref|Os11g18280|
gi|8701702|ref|Os11g18300|
gi|8701704|ref|Os11g18320|
gi|8701705|ref|Os11g18330|
gi|8701712|ref|Os11g18400|
gi|8701713|ref|Os11g18410|
gi|8701718|ref|Os11g18460|
gi|8701719|ref|Os11g18470|
gi|8701720|ref|Os11g18480|
gi|8701721|ref|Os11g18490|
gi|8701722|ref|Os11g18500|
gi|8701725|ref|Os11g18530|
gi|8701726|ref|Os11g18540|
gi|8701731|ref|Os11g18590|
gi|8701733|ref|Os11g18610|
gi|8701735|ref|Os11g18630|
gi|8701738|ref|Os11g18660|
gi|8701740|ref|Os11g18680|
gi|8701746|ref|Os11g18740|
gi|8701747|ref|Os11g18750|
gi|8701748|ref|Os11g18760|
gi|8701750|ref|Os11g18780|
gi|8701752|ref|Os11g18800|
gi|8701757|ref|Os11g18850|
gi|8701758|ref|Os11g18860|
gi|8701763|ref|Os11g18890|
gi|8701764|ref|Os11g18900|
gi|8701767|ref|Os11g18930|
gi|8701771|ref|Os11g18970|
gi|8701776|ref|Os11g19020|
gi|8701778|ref|Os11g19040|
gi|8701779|ref|Os11g19050|
gi|8701781|ref|Os11g19070|
gi|8701782|ref|Os11g19080|
gi|8701783|ref|Os11g19090|
gi|8701792|ref|Os11g19170|
gi|8701793|ref|Os11g19180|
gi|8701801|ref|Os11g19260|
gi|8701802|ref|Os11g19270|
gi|8701803|ref|Os11g19280|
gi|8701805|ref|Os11g19300|
gi|8701807|ref|Os11g19320|
gi|8701811|ref|Os11g19360|
gi|8701812|ref|Os11g19370|
gi|8701815|ref|Os11g19400|
gi|8701816|ref|Os11g19410|
gi|8701819|ref|Os11g19440|
gi|8701820|ref|Os11g19450|
gi|8701822|ref|Os11g19470|
gi|8701825|ref|Os11g19500|

gi|8701826|ref|Os11g19510|
gi|8701828|ref|Os11g19530|
gi|8701829|ref|Os11g19540|
gi|8701833|ref|Os11g19580|
gi|8701842|ref|Os11g19670|
gi|8701843|ref|Os11g19680|
gi|8701846|ref|Os11g19710|
gi|8701849|ref|Os11g19740|
gi|8701850|ref|Os11g19750|
gi|8701851|ref|Os11g19760|
gi|8701860|ref|Os11g19850|
gi|8701864|ref|Os11g19890|
gi|8701865|ref|Os11g19900|
gi|8701867|ref|Os11g19920|
gi|8701868|ref|Os11g19930|
gi|8701872|ref|Os11g19970|
gi|8701873|ref|Os11g19980|
gi|8701876|ref|Os11g20010|
gi|8701877|ref|Os11g20020|
gi|8701880|ref|Os11g20050|
gi|8701881|ref|Os11g20060|
gi|8701882|ref|Os11g20070|
gi|8701885|ref|Os11g20100|
gi|8701888|ref|Os11g20130|
gi|8701889|ref|Os11g20140|
gi|8701892|ref|Os11g20170|
gi|8701893|ref|Os11g20180|
gi|8701894|ref|Os11g20190|
gi|8701895|ref|Os11g20200|
gi|8701898|ref|Os11g20230|
gi|8701900|ref|Os11g20250|
gi|8701903|ref|Os11g20280|
gi|8701907|ref|Os11g20320|
gi|8701908|ref|Os11g20330|
gi|8701909|ref|Os11g20340|
gi|8701910|ref|Os11g20350|
gi|8701913|ref|Os11g20370|
gi|8701920|ref|Os11g20440|
gi|8701925|ref|Os11g20490|
gi|8701926|ref|Os11g20500|
gi|8701929|ref|Os11g20530|
gi|8701934|ref|Os11g20580|
gi|8701936|ref|Os11g20600|
gi|8701939|ref|Os11g20630|
gi|8701942|ref|Os11g20660|
gi|8701943|ref|Os11g20670|
gi|8701945|ref|Os11g20690|
gi|8701949|ref|Os11g20730|
gi|8701950|ref|Os11g20740|
gi|8701951|ref|Os11g20750|
gi|8701953|ref|Os11g20770|
gi|8701954|ref|Os11g20780|
gi|8701958|ref|Os11g21810|
gi|8701961|ref|Os11g21840|
gi|8701963|ref|Os11g21860|
gi|8701968|ref|Os11g21910|
gi|8701969|ref|Os11g21920|
gi|8701970|ref|Os11g21930|
gi|8701981|ref|Os11g22040|
gi|8701982|ref|Os11g22050|
gi|8701983|ref|Os11g22060|
gi|8701984|ref|Os11g22070|
gi|8701986|ref|Os11g22090|
gi|8701988|ref|Os11g22110|
gi|8701989|ref|Os11g22120|
gi|8701994|ref|Os11g22170|
gi|8701995|ref|Os11g22180|
gi|8701997|ref|Os11g22200|
gi|8701998|ref|Os11g22210|
gi|8702002|ref|Os11g22250|
gi|8702004|ref|Os11g22270|
gi|8702006|ref|Os11g22290|
gi|8702013|ref|Os11g22360|
gi|8702015|ref|Os11g22380|
gi|8702016|ref|Os11g22390|
gi|8702019|ref|Os11g22420|
gi|8702021|ref|Os11g22440|
gi|8702022|ref|Os11g22450|
gi|8702023|ref|Os11g22460|
gi|8702024|ref|Os11g22470|
gi|8702026|ref|Os11g22490|
gi|8702028|ref|Os11g22510|
gi|8702033|ref|Os11g22560|
gi|8702040|ref|Os11g22630|
gi|8702041|ref|Os11g22640|
gi|8702042|ref|Os11g22650|
gi|8702043|ref|Os11g22660|
gi|8702046|ref|Os11g22690|
gi|8702053|ref|Os11g22760|
gi|8702054|ref|Os11g22770|
gi|8702057|ref|Os11g22800|
gi|8702058|ref|Os11g22810|
gi|8702059|ref|Os11g22820|
gi|8702063|ref|Os11g22860|
gi|8702065|ref|Os11g22880|
gi|8702066|ref|Os11g22890|
gi|8702067|ref|Os11g22900|
gi|8702073|ref|Os11g22960|
gi|8702074|ref|Os11g22970|
gi|8702077|ref|Os11g23000|
gi|8702080|ref|Os11g23030|
gi|8702082|ref|Os11g23050|
gi|8702084|ref|Os11g23070|
gi|8702085|ref|Os11g23080|
gi|8702086|ref|Os11g23080|
gi|8702093|ref|Os11g23150|
gi|8702094|ref|Os11g23160|
gi|8702096|ref|Os11g23180|
gi|8702097|ref|Os11g23190|
gi|8702099|ref|Os11g23210|
gi|8702101|ref|Os11g23230|
gi|8702107|ref|Os11g23780|
gi|8702117|ref|Os11g23850|
gi|8702118|ref|Os11g23860|
gi|8702122|ref|Os11g23900|
gi|8702123|ref|Os11g23910|
gi|8702124|ref|Os11g23920|
gi|8702125|ref|Os11g23930|
gi|8702131|ref|Os11g23990|
gi|8702134|ref|Os11g24020|
gi|8702135|ref|Os11g24030|
gi|8702140|ref|Os11g24080|
gi|8702143|ref|Os11g24110|
gi|8702144|ref|Os11g24120|
gi|8702151|ref|Os11g24190|
gi|8702153|ref|Os11g24210|
gi|8702155|ref|Os11g24230|
gi|8702158|ref|Os11g24260|
gi|8702163|ref|Os11g24310|
gi|8702168|ref|Os11g24360|
gi|8702170|ref|Os11g24380|
gi|8702171|ref|Os11g24390|
gi|8702174|ref|Os11g24420|
gi|8702175|ref|Os11g24430|

gi|8702176|ref|Os11g24440|
gi|8702178|ref|Os11g24460|
gi|8702184|ref|Os11g24520|
gi|8702191|ref|Os11g24600|
gi|8702194|ref|Os11g24620|
gi|8702196|ref|Os11g24640|
gi|8702198|ref|Os11g24660|
gi|8702200|ref|Os11g24680|
gi|8702202|ref|Os11g24700|
gi|8702206|ref|Os11g24740|
gi|8702211|ref|Os11g24790|
gi|8702214|ref|Os11g24820|
gi|8702221|ref|Os11g24890|
gi|8702222|ref|Os11g24900|
gi|8702223|ref|Os11g24910|
gi|8702226|ref|Os11g24940|
gi|8702227|ref|Os11g24950|
gi|8702230|ref|Os11g24980|
gi|8702240|ref|Os11g25080|
gi|8702241|ref|Os11g25090|
gi|8702243|ref|Os11g25110|
gi|8702245|ref|Os11g25130|
gi|8702246|ref|Os11g25140|
gi|8702247|ref|Os11g25150|
gi|8702248|ref|Os11g25160|
gi|8702256|ref|Os11g25240|
gi|8702262|ref|Os11g25300|
gi|8702263|ref|Os11g25310|
gi|8702266|ref|Os11g25340|
gi|8702269|ref|Os11g25370|
gi|8702274|ref|Os11g25420|
gi|8702276|ref|Os11g25440|
gi|8702279|ref|Os11g25470|
gi|8702280|ref|Os11g25480|
gi|8702281|ref|Os11g25490|
gi|8702285|ref|Os11g25530|
gi|8702286|ref|Os11g25540|
gi|8702289|ref|Os11g25570|
gi|8702292|ref|Os11g25600|
gi|8702293|ref|Os11g25610|
gi|8702294|ref|Os11g25620|
gi|8702295|ref|Os11g25630|
gi|8702300|ref|Os11g25680|
gi|8702306|ref|Os11g25740|
gi|8702307|ref|Os11g25750|
gi|8702308|ref|Os11g25760|
gi|8702309|ref|Os11g25770|
gi|8702311|ref|Os11g25790|
gi|8702314|ref|Os11g25820|
gi|8702316|ref|Os11g25840|
gi|8702319|ref|Os11g25870|
gi|8702320|ref|Os11g25880|
gi|8702321|ref|Os11g25890|
gi|8702323|ref|Os11g25910|
gi|8702328|ref|Os11g25960|
gi|8702329|ref|Os11g25970|
gi|8702334|ref|Os11g26020|
gi|8702338|ref|Os11g26060|
gi|8702339|ref|Os11g26070|
gi|8702351|ref|Os11g26190|
gi|8702352|ref|Os11g26200|
gi|8702353|ref|Os11g26210|
gi|8702354|ref|Os11g26220|
gi|8702356|ref|Os11g26240|
gi|8702357|ref|Os11g26250|
gi|8702360|ref|Os11g26280|
gi|8702365|ref|Os11g26330|
gi|8702366|ref|Os11g26340|
gi|8702367|ref|Os11g26350|
gi|8702372|ref|Os11g26400|
gi|8702374|ref|Os11g26420|
gi|8702375|ref|Os11g26430|
gi|8702379|ref|Os11g26470|
gi|8702381|ref|Os11g26490|
gi|8702385|ref|Os11g26530|
gi|8702388|ref|Os11g26560|
gi|8702389|ref|Os11g26570|
gi|8702392|ref|Os11g26600|
gi|8702393|ref|Os11g26610|
gi|8702394|ref|Os11g26620|
gi|8702395|ref|Os11g26630|
gi|8702396|ref|Os11g26640|
gi|8702398|ref|Os11g26660|
gi|8702401|ref|Os11g26690|
gi|8702402|ref|Os11g26700|
gi|8702403|ref|Os11g26710|
gi|8702404|ref|Os11g26720|
gi|8702405|ref|Os11g26730|
gi|8702407|ref|Os11g26750|
gi|8702412|ref|Os11g26800|
gi|8702416|ref|Os11g26840|
gi|8702420|ref|Os11g26870|
gi|8702424|ref|Os11g26900|
gi|8702427|ref|Os11g26930|
gi|8702431|ref|Os11g26970|
gi|8702432|ref|Os11g26980|
gi|8702433|ref|Os11g26990|
gi|8702434|ref|Os11g27000|
gi|8702435|ref|Os11g27010|
gi|8702436|ref|Os11g27020|
gi|8702438|ref|Os11g27040|
gi|8702443|ref|Os11g27090|
gi|8702445|ref|Os11g27110|
gi|8702446|ref|Os11g27120|
gi|8702448|ref|Os11g27140|
gi|8702456|ref|Os11g27220|
gi|8702463|ref|Os11g27280|
gi|8702466|ref|Os11g27310|
gi|8702471|ref|Os11g27360|
gi|8702474|ref|Os11g27390|
gi|8702477|ref|Os11g27420|
gi|8702481|ref|Os11g27460|
gi|8702483|ref|Os11g27480|
gi|8702484|ref|Os11g27490|
gi|8702486|ref|Os11g27510|
gi|8702490|ref|Os11g27550|
gi|8702491|ref|Os11g27560|
gi|8702493|ref|Os11g27580|
gi|8702495|ref|Os11g27600|
gi|8702498|ref|Os11g27630|
gi|8702499|ref|Os11g27640|
gi|8702501|ref|Os11g27660|
gi|8702502|ref|Os11g27670|
gi|8702503|ref|Os11g27680|
gi|8702506|ref|Os11g27710|
gi|8702507|ref|Os11g27720|
gi|8702510|ref|Os11g27740|
gi|8702514|ref|Os11g27780|
gi|8702517|ref|Os11g27810|
gi|8702520|ref|Os11g27840|
gi|8702522|ref|Os11g27860|
gi|8702526|ref|Os11g27900|
gi|8702529|ref|Os11g27930|
gi|8702530|ref|Os11g27940|

gi|8702533|ref|Os11g27970|
gi|8702536|ref|Os11g28000|
gi|8702537|ref|Os11g28010|
gi|8702543|ref|Os11g28070|
gi|8702544|ref|Os11g28080|
gi|8702547|ref|Os11g28110|
gi|8702549|ref|Os11g28130|
gi|8702552|ref|Os11g28160|
gi|8702553|ref|Os11g28170|
gi|8702554|ref|Os11g28180|
gi|8702556|ref|Os11g28200|
gi|8702558|ref|Os11g28220|
gi|8702562|ref|Os11g28260|
gi|8702564|ref|Os11g28280|
gi|8702568|ref|Os11g28320|
gi|8702569|ref|Os11g28330|
gi|8702573|ref|Os11g28370|
gi|8702574|ref|Os11g28380|
gi|8702575|ref|Os11g28390|
gi|8702578|ref|Os11g28420|
gi|8702579|ref|Os11g28430|
gi|8702581|ref|Os11g28450|
gi|8702582|ref|Os11g28460|
gi|8702584|ref|Os11g28480|
gi|8702587|ref|Os11g28510|
gi|8702588|ref|Os11g28520|
gi|8702595|ref|Os11g28590|
gi|8702596|ref|Os11g28600|
gi|8702600|ref|Os11g28640|
gi|8702603|ref|Os11g28670|
gi|8702604|ref|Os11g28680|
gi|8702607|ref|Os11g28710|
gi|8702611|ref|Os11g28750|
gi|8702612|ref|Os11g28760|
gi|8702616|ref|Os11g28800|
gi|8702618|ref|Os11g28820|
gi|8702619|ref|Os11g28830|
gi|8702622|ref|Os11g28860|
gi|8702623|ref|Os11g28870|
gi|8702624|ref|Os11g28880|
gi|8702625|ref|Os11g28890|
gi|8702626|ref|Os11g28900|
gi|8702627|ref|Os11g28910|
gi|8702628|ref|Os11g28920|
gi|8702629|ref|Os11g28930|
gi|8702630|ref|Os11g28940|
gi|8702632|ref|Os11g28960|
gi|8702642|ref|Os11g29060|
gi|8702646|ref|Os11g29100|
gi|8702650|ref|Os11g29130|
gi|8702651|ref|Os11g29140|
gi|8702653|ref|Os11g29160|
gi|8702658|ref|Os11g29210|
gi|8702659|ref|Os11g29220|
gi|8702662|ref|Os11g29250|
gi|8702663|ref|Os11g29260|
gi|8702664|ref|Os11g29270|
gi|8702665|ref|Os11g29280|
gi|8702667|ref|Os11g29300|
gi|8702678|ref|Os11g29410|
gi|8702680|ref|Os11g29430|
gi|8702681|ref|Os11g29440|
gi|8702683|ref|Os11g29470|
gi|8702684|ref|Os11g29460|
gi|8702685|ref|Os11g29480|
gi|8702687|ref|Os11g29500|
gi|8702691|ref|Os11g29540|
gi|8702692|ref|Os11g29550|
gi|8702695|ref|Os11g29580|
gi|8702699|ref|Os11g29620|
gi|8702700|ref|Os11g29630|
gi|8702704|ref|Os11g29670|
gi|8702705|ref|Os11g29680|
gi|8702712|ref|Os11g29740|
gi|8702714|ref|Os11g29760|
gi|8702718|ref|Os11g29800|
gi|8702719|ref|Os11g29810|
gi|8702720|ref|Os11g29820|
gi|8702721|ref|Os11g29830|
gi|8702722|ref|Os11g29840|
gi|8702724|ref|Os11g29860|
gi|8702727|ref|Os11g29890|
gi|8702731|ref|Os11g29930|
gi|8702732|ref|Os11g29940|
gi|8702748|ref|Os11g30100|
gi|8702750|ref|Os11g30120|
gi|8702751|ref|Os11g30130|
gi|8702752|ref|Os11g30140|
gi|8702753|ref|Os11g30150|
gi|8702757|ref|Os11g30190|
gi|8702758|ref|Os11g30200|
gi|8702760|ref|Os11g30220|
gi|8702761|ref|Os11g30230|
gi|8702763|ref|Os11g30250|
gi|8702766|ref|Os11g30280|
gi|8702768|ref|Os11g30300|
gi|8702770|ref|Os11g30320|
gi|8702771|ref|Os11g30330|
gi|8702772|ref|Os11g30340|
gi|8702781|ref|Os11g30430|
gi|8702783|ref|Os11g30450|
gi|8702786|ref|Os11g30480|
gi|8702789|ref|Os11g30510|
gi|8702790|ref|Os11g30520|
gi|8702796|ref|Os11g30570|
gi|8702797|ref|Os11g30580|
gi|8702799|ref|Os11g30600|
gi|8702800|ref|Os11g30610|
gi|8702801|ref|Os11g30620|
gi|8702802|ref|Os11g30630|
gi|8702803|ref|Os11g30640|
gi|8702806|ref|Os11g30670|
gi|8702807|ref|Os11g30680|
gi|8702808|ref|Os11g30690|
gi|8702810|ref|Os11g30710|
gi|8702811|ref|Os11g30720|
gi|8702814|ref|Os11g30750|
gi|8702815|ref|Os11g30760|
gi|8702816|ref|Os11g30770|
gi|8702817|ref|Os11g30780|
gi|8702821|ref|Os11g30820|
gi|8702826|ref|Os11g30870|
gi|8702827|ref|Os11g30880|
gi|8702832|ref|Os11g30930|
gi|8702833|ref|Os11g30940|
gi|8702839|ref|Os11g31000|
gi|8702840|ref|Os11g31010|
gi|8702842|ref|Os11g31030|
gi|8702843|ref|Os11g31040|
gi|8702845|ref|Os11g31060|
gi|8702846|ref|Os11g31070|
gi|8702847|ref|Os11g31080|
gi|8702851|ref|Os11g31120|
gi|8702852|ref|Os11g31130|

gi|8702853|ref|Os11g31140|
gi|8702854|ref|Os11g31150|
gi|8702859|ref|Os11g31200|
gi|8702864|ref|Os11g31250|
gi|8702867|ref|Os11g31280|
gi|8702876|ref|Os11g31370|
gi|8702878|ref|Os11g31390|
gi|8702879|ref|Os11g31400|
gi|8702881|ref|Os11g31420|
gi|8702883|ref|Os11g31440|
gi|8702884|ref|Os11g31450|
gi|8702900|ref|Os11g31600|
gi|8702901|ref|Os11g31610|
gi|8702906|ref|Os11g31660|
gi|8702907|ref|Os11g31670|
gi|8702909|ref|Os11g31690|
gi|8702911|ref|Os11g31710|
gi|8702913|ref|Os11g31730|
gi|8702914|ref|Os11g31740|
gi|8702915|ref|Os11g31750|
gi|8702918|ref|Os11g31780|
gi|8702919|ref|Os11g31790|
gi|8702920|ref|Os11g31800|
gi|8702921|ref|Os11g31810|
gi|8702922|ref|Os11g31820|
gi|8702925|ref|Os11g31850|
gi|8702934|ref|Os11g31930|
gi|8702936|ref|Os11g31950|
gi|8702942|ref|Os11g32010|
gi|8702943|ref|Os11g32020|
gi|8702945|ref|Os11g32040|
gi|8702946|ref|Os11g32050|
gi|8702954|ref|Os11g32130|
gi|8702960|ref|Os11g32190|
gi|8702963|ref|Os11g32220|
gi|8702964|ref|Os11g32230|
gi|8702966|ref|Os11g32250|
gi|8702971|ref|Os11g32300|
gi|8702972|ref|Os11g32310|
gi|8702974|ref|Os11g32330|
gi|8702975|ref|Os11g32340|
gi|8702976|ref|Os11g32350|
gi|8702978|ref|Os11g32370|
gi|8702979|ref|Os11g32380|
gi|8702980|ref|Os11g32390|
gi|8702981|ref|Os11g32400|
gi|8702982|ref|Os11g32410|
gi|8702984|ref|Os11g32430|
gi|8702985|ref|Os11g32440|
gi|8702986|ref|Os11g32450|
gi|8702987|ref|Os11g32460|
gi|8702994|ref|Os11g32530|
gi|8702997|ref|Os11g32560|
gi|8702998|ref|Os11g32570|
gi|8703001|ref|Os11g32600|
gi|8703004|ref|Os11g32630|
gi|8703005|ref|Os11g32640|
gi|8703011|ref|Os11g32700|
gi|8703012|ref|Os11g32710|
gi|8703014|ref|Os11g32730|
gi|8703015|ref|Os11g32740|
gi|8703017|ref|Os11g32760|
gi|8703019|ref|Os11g32780|
gi|8703021|ref|Os11g32800|
gi|8703023|ref|Os11g32820|
gi|8703025|ref|Os11g32840|
gi|8703027|ref|Os11g32860|
gi|8703028|ref|Os11g32870|
gi|8703034|ref|Os11g32910|
gi|8703035|ref|Os11g32920|
gi|8703036|ref|Os11g32930|
gi|8703043|ref|Os11g32990|
gi|8703044|ref|Os11g33000|
gi|8703047|ref|Os11g33030|
gi|8703048|ref|Os11g33040|
gi|8703059|ref|Os11g33150|
gi|8703060|ref|Os11g33160|
gi|8703061|ref|Os11g33170|
gi|8703063|ref|Os11g33190|
gi|8703064|ref|Os11g33200|
gi|8703065|ref|Os11g33210|
gi|8703066|ref|Os11g33220|
gi|8703067|ref|Os11g33230|
gi|8703069|ref|Os11g33250|
gi|8703072|ref|Os11g33280|
gi|8703080|ref|Os11g33360|
gi|8703081|ref|Os11g33370|
gi|8703082|ref|Os11g33380|
gi|8703085|ref|Os11g33410|
gi|8703093|ref|Os11g33980|
gi|8703094|ref|Os11g33990|
gi|8703095|ref|Os11g34000|
gi|8703096|ref|Os11g34010|
gi|8703097|ref|Os11g34020|
gi|8703098|ref|Os11g34030|
gi|8703099|ref|Os11g34040|
gi|8703102|ref|Os11g34070|
gi|8703103|ref|Os11g34080|
gi|8703105|ref|Os11g34100|
gi|8703112|ref|Os11g34170|
gi|8703118|ref|Os11g34220|
gi|8703119|ref|Os11g34230|
gi|8703120|ref|Os11g34230|
gi|8703121|ref|Os11g34230|
gi|8703122|ref|Os11g34230|
gi|8703129|ref|Os11g34280|
gi|8703134|ref|Os11g34330|
gi|8703135|ref|Os11g34340|
gi|8703139|ref|Os11g34380|
gi|8703148|ref|Os11g34470|
gi|8703149|ref|Os11g34480|
gi|8703154|ref|Os11g34530|
gi|8703157|ref|Os11g34560|
gi|8703159|ref|Os11g34580|
gi|8703165|ref|Os11g34640|
gi|8703166|ref|Os11g34650|
gi|8703175|ref|Os11g34740|
gi|8703179|ref|Os11g34780|
gi|8703180|ref|Os11g34790|
gi|8703181|ref|Os11g34800|
gi|8703182|ref|Os11g34810|
gi|8703183|ref|Os11g34810|
gi|8703190|ref|Os11g34870|
gi|8703192|ref|Os11g34890|
gi|8703193|ref|Os11g34900|
gi|8703194|ref|Os11g34910|
gi|8703198|ref|Os11g34950|
gi|8703199|ref|Os11g34960|
gi|8703201|ref|Os11g34980|
gi|8703202|ref|Os11g34990|
gi|8703203|ref|Os11g35000|
gi|8703210|ref|Os11g35070|
gi|8703211|ref|Os11g35080|
gi|8703214|ref|Os11g35110|

gi|8703218|ref|Os11g35150|
gi|8703219|ref|Os11g35160|
gi|8703221|ref|Os11g35180|
gi|8703228|ref|Os11g35250|
gi|8703231|ref|Os11g35280|
gi|8703233|ref|Os11g35300|
gi|8703238|ref|Os11g35350|
gi|8703241|ref|Os11g35370|
gi|8703242|ref|Os11g35380|
gi|8703247|ref|Os11g35430|
gi|8703248|ref|Os11g35440|
gi|8703258|ref|Os11g35540|
gi|8703264|ref|Os11g35600|
gi|8703265|ref|Os11g35610|
gi|8703266|ref|Os11g35620|
gi|8703271|ref|Os11g35670|
gi|8703276|ref|Os11g35720|
gi|8703286|ref|Os11g35820|
gi|8703296|ref|Os11g35900|
gi|8703305|ref|Os11g35990|
gi|8703307|ref|Os11g36010|
gi|8703310|ref|Os11g36040|
gi|8703314|ref|Os11g36080|
gi|8703316|ref|Os11g36100|
gi|8703319|ref|Os11g36130|
gi|8703323|ref|Os11g36170|
gi|8703327|ref|Os11g36210|
gi|8703329|ref|Os11g36230|
gi|8703332|ref|Os11g36260|
gi|8703335|ref|Os11g36290|
gi|8703336|ref|Os11g36300|
gi|8703338|ref|Os11g36320|
gi|8703339|ref|Os11g36330|
gi|8703361|ref|Os11g36520|
gi|8703363|ref|Os11g36540|
gi|8703364|ref|Os11g36550|
gi|8703366|ref|Os11g36570|
gi|8703367|ref|Os11g36580|
gi|8703369|ref|Os11g36600|
gi|8703371|ref|Os11g36620|
gi|8703375|ref|Os11g36660|
gi|8703376|ref|Os11g36670|
gi|8703377|ref|Os11g36680|
gi|8703378|ref|Os11g36690|
gi|8703380|ref|Os11g36710|
gi|8703381|ref|Os11g36720|
gi|8703384|ref|Os11g36750|
gi|8703386|ref|Os11g36770|
gi|8703387|ref|Os11g36780|
gi|8703389|ref|Os11g36800|
gi|8703390|ref|Os11g36810|
gi|8703391|ref|Os11g36820|
gi|8703392|ref|Os11g36830|
gi|8703393|ref|Os11g36840|
gi|8703399|ref|Os11g36900|
gi|8703400|ref|Os11g36910|
gi|8703401|ref|Os11g36920|
gi|8703402|ref|Os11g36930|
gi|8703407|ref|Os11g36980|
gi|8703410|ref|Os11g37010|
gi|8703415|ref|Os11g37060|
gi|8703421|ref|Os11g37120|
gi|8703424|ref|Os11g37150|
gi|8703425|ref|Os11g37160|
gi|8703431|ref|Os11g37220|
gi|8703433|ref|Os11g37240|
gi|8703434|ref|Os11g37250|
gi|8703436|ref|Os11g37270|
gi|8703438|ref|Os11g37290|
gi|8703440|ref|Os11g37310|
gi|8703441|ref|Os11g37320|
gi|8703445|ref|Os11g37360|
gi|8703446|ref|Os11g37370|
gi|8703447|ref|Os11g37380|
gi|8703449|ref|Os11g37400|
gi|8703450|ref|Os11g37410|
gi|8703456|ref|Os11g37470|
gi|8703457|ref|Os11g37480|
gi|8703458|ref|Os11g37490|
gi|8703459|ref|Os11g37500|
gi|8703462|ref|Os11g37530|
gi|8703466|ref|Os11g37570|
gi|8703467|ref|Os11g37580|
gi|8703470|ref|Os11g37610|
gi|8703471|ref|Os11g37620|
gi|8703476|ref|Os11g37670|
gi|8703487|ref|Os11g37770|
gi|8703492|ref|Os11g37810|
gi|8703495|ref|Os11g37840|
gi|8703504|ref|Os11g37910|
gi|8703520|ref|Os11g38070|
gi|8703524|ref|Os11g38110|
gi|8703528|ref|Os11g38150|
gi|8703531|ref|Os11g38180|
gi|8703532|ref|Os11g38190|
gi|8703536|ref|Os11g38230|
gi|8703540|ref|Os11g38270|
gi|8703541|ref|Os11g38280|
gi|8703542|ref|Os11g38290|
gi|8703546|ref|Os11g38330|
gi|8703550|ref|Os11g38370|
gi|8703551|ref|Os11g38380|
gi|8703552|ref|Os11g38390|
gi|8703553|ref|Os11g38400|
gi|8703554|ref|Os11g38410|
gi|8703556|ref|Os11g38430|
gi|8703560|ref|Os11g38470|
gi|8703562|ref|Os11g38490|
gi|8703564|ref|Os11g38510|
gi|8703570|ref|Os11g38570|
gi|8703572|ref|Os11g38590|
gi|8703583|ref|Os11g38680|
gi|8703586|ref|Os11g38710|
gi|8703587|ref|Os11g38720|
gi|8703588|ref|Os11g38730|
gi|8703592|ref|Os11g38770|
gi|8703594|ref|Os11g38790|
gi|8703599|ref|Os11g38830|
gi|8703600|ref|Os11g38840|
gi|8703604|ref|Os11g38880|
gi|8703612|ref|Os11g38950|
gi|8703615|ref|Os11g38970|
gi|8703621|ref|Os11g39010|
gi|8703623|ref|Os11g39030|
gi|8703624|ref|Os11g39040|
gi|8703625|ref|Os11g39050|
gi|8703626|ref|Os11g39060|
gi|8703627|ref|Os11g39070|
gi|8703628|ref|Os11g39080|
gi|8703629|ref|Os11g39090|
gi|8703630|ref|Os11g39100|
gi|8703631|ref|Os11g39110|
gi|8703632|ref|Os11g39120|
gi|8703637|ref|Os11g39150|

gi|8703640|ref|Os11g39180|
gi|8703643|ref|Os11g39210|
gi|8703647|ref|Os11g39240|
gi|8703649|ref|Os11g39260|
gi|8703658|ref|Os11g39350|
gi|8703662|ref|Os11g39380|
gi|8703663|ref|Os11g39390|
gi|8703664|ref|Os11g39400|
gi|8703665|ref|Os11g39410|
gi|8703670|ref|Os11g39460|
gi|8703671|ref|Os11g39470|
gi|8703674|ref|Os11g39500|
gi|8703676|ref|Os11g39520|
gi|8703680|ref|Os11g39560|
gi|8703682|ref|Os11g39580|
gi|8703683|ref|Os11g39590|
gi|8703685|ref|Os11g39610|
gi|8703687|ref|Os11g39630|
gi|8703693|ref|Os11g39680|
gi|8703694|ref|Os11g39690|
gi|8703699|ref|Os11g39740|
gi|8703700|ref|Os11g39750|
gi|8703701|ref|Os11g39760|
gi|8703702|ref|Os11g39770|
gi|8703703|ref|Os11g39780|
gi|8703705|ref|Os11g39800|
gi|8703706|ref|Os11g39810|
gi|8703707|ref|Os11g39820|
gi|8703708|ref|Os11g39830|
gi|8703709|ref|Os11g39840|
gi|8703710|ref|Os11g39850|
gi|8703711|ref|Os11g39860|
gi|8703713|ref|Os11g39880|
gi|8703716|ref|Os11g39910|
gi|8703721|ref|Os11g39960|
gi|8703726|ref|Os11g40010|
gi|8703729|ref|Os11g40040|
gi|8703739|ref|Os11g40120|
gi|8703740|ref|Os11g40130|
gi|8703755|ref|Os11g40270|
gi|8703759|ref|Os11g40310|
gi|8703761|ref|Os11g40330|
gi|8703763|ref|Os11g40350|
gi|8703780|ref|Os11g40520|
gi|8703781|ref|Os11g40530|
gi|8703785|ref|Os11g40560|
gi|8703789|ref|Os11g40580|
gi|8703794|ref|Os11g40620|
gi|8703797|ref|Os11g40650|
gi|8703806|ref|Os11g40740|
gi|8703808|ref|Os11g40760|
gi|8703809|ref|Os11g40770|
gi|8703811|ref|Os11g40790|
gi|8703812|ref|Os11g40800|
gi|8703817|ref|Os11g40850|
gi|8703819|ref|Os11g40870|
gi|8703820|ref|Os11g40880|
gi|8703822|ref|Os11g40900|
gi|8703823|ref|Os11g40910|
gi|8703825|ref|Os11g40930|
gi|8703833|ref|Os11g41010|
gi|8703835|ref|Os11g41030|
gi|8703836|ref|Os11g41040|
gi|8703838|ref|Os11g41060|
gi|8703839|ref|Os11g41070|
gi|8703840|ref|Os11g41080|
gi|8703841|ref|Os11g41090|
gi|8703854|ref|Os11g41200|
gi|8703856|ref|Os11g41220|
gi|8703866|ref|Os11g41320|
gi|8703867|ref|Os11g41330|
gi|8703868|ref|Os11g41340|
gi|8703873|ref|Os11g41390|
gi|8703874|ref|Os11g41400|
gi|8703876|ref|Os11g41420|
gi|8703878|ref|Os11g41440|
gi|8703880|ref|Os11g41460|
gi|8703885|ref|Os11g41510|
gi|8703886|ref|Os11g41520|
gi|8703887|ref|Os11g41530|
gi|8703889|ref|Os11g41550|
gi|8703890|ref|Os11g41560|
gi|8703892|ref|Os11g41580|
gi|8703893|ref|Os11g41590|
gi|8703901|ref|Os11g41670|
gi|8703904|ref|Os11g41700|
gi|8703906|ref|Os11g41720|
gi|8703911|ref|Os11g41770|
gi|8703913|ref|Os11g41790|
gi|8703914|ref|Os11g41800|
gi|8703915|ref|Os11g41810|
gi|8703926|ref|Os11g41920|
gi|8703927|ref|Os11g41930|
gi|8703928|ref|Os11g41940|
gi|8703929|ref|Os11g41950|
gi|8703930|ref|Os11g41960|
gi|8703931|ref|Os11g41970|
gi|8703932|ref|Os11g41980|
gi|8703937|ref|Os11g42020|
gi|8703947|ref|Os11g42110|
gi|8703948|ref|Os11g42120|
gi|8703951|ref|Os11g42150|
gi|8703953|ref|Os11g42170|
gi|8703957|ref|Os11g42210|
gi|8703959|ref|Os11g42230|
gi|8703960|ref|Os11g42240|
gi|8703962|ref|Os11g42260|
gi|8703970|ref|Os11g42340|
gi|8703973|ref|Os11g42360|
gi|8703975|ref|Os11g42380|
gi|8703977|ref|Os11g42400|
gi|8703978|ref|Os11g42410|
gi|8703986|ref|Os11g42470|
gi|8703988|ref|Os11g42490|
gi|8703993|ref|Os11g42520|
gi|8703994|ref|Os11g42530|
gi|8703998|ref|Os11g42560|
gi|8703999|ref|Os11g42570|
gi|8704009|ref|Os11g42670|
gi|8704011|ref|Os11g42690|
gi|8704012|ref|Os11g42700|
gi|8704016|ref|Os11g42740|
gi|8704018|ref|Os11g42760|
gi|8704023|ref|Os11g42810|
gi|8704024|ref|Os11g42820|
gi|8704025|ref|Os11g42830|
gi|8704026|ref|Os11g42840|
gi|8704027|ref|Os11g42850|
gi|8704029|ref|Os11g42870|
gi|8704030|ref|Os11g42880|
gi|8704031|ref|Os11g42890|
gi|8704034|ref|Os11g42920|
gi|8704035|ref|Os11g42930|
gi|8704036|ref|Os11g42940|

gi|8704037|ref|Os11g42950|
gi|8704038|ref|Os11g42960|
gi|8704039|ref|Os11g42970|
gi|8704041|ref|Os11g42990|
gi|8704043|ref|Os11g43010|
gi|8704047|ref|Os11g43050|
gi|8704049|ref|Os11g43070|
gi|8704058|ref|Os11g43160|
gi|8704064|ref|Os11g43220|
gi|8704066|ref|Os11g43240|
gi|8704073|ref|Os11g43310|
gi|8704076|ref|Os11g43330|
gi|8704077|ref|Os11g43340|
gi|8704087|ref|Os11g43440|
gi|8704092|ref|Os11g43490|
gi|8704097|ref|Os11g43540|
gi|8704100|ref|Os11g43570|
gi|8704107|ref|Os11g43630|
gi|8704109|ref|Os11g43650|
gi|8704113|ref|Os11g43690|
gi|8704115|ref|Os11g43710|
gi|8704116|ref|Os11g43720|
gi|8704125|ref|Os11g43810|
gi|8704128|ref|Os11g43840|
gi|8704136|ref|Os11g43910|
gi|8704137|ref|Os11g43920|
gi|8704138|ref|Os11g43930|
gi|8704145|ref|Os11g43990|
gi|8704146|ref|Os11g44000|
gi|8704148|ref|Os11g44020|
gi|8704149|ref|Os11g44030|
gi|8704150|ref|Os11g44040|
gi|8704154|ref|Os11g44080|
gi|8704157|ref|Os11g44110|
gi|8704158|ref|Os11g44120|
gi|8704160|ref|Os11g44140|
gi|8704166|ref|Os11g44200|
gi|8704168|ref|Os11g44220|
gi|8704169|ref|Os11g44230|
gi|8704173|ref|Os11g44270|
gi|8704174|ref|Os11g44280|
gi|8704175|ref|Os11g44290|
gi|8704176|ref|Os11g44300|
gi|8704178|ref|Os11g44320|
gi|8704182|ref|Os11g44360|
gi|8704183|ref|Os11g44370|
gi|8704184|ref|Os11g44380|
gi|8704191|ref|Os11g44450|
gi|8704192|ref|Os11g44460|
gi|8704193|ref|Os11g44470|
gi|8704195|ref|Os11g44490|
gi|8704197|ref|Os11g44510|
gi|8704203|ref|Os11g44570|
gi|8704205|ref|Os11g44590|
gi|8704206|ref|Os11g44600|
gi|8704211|ref|Os11g44650|
gi|8704216|ref|Os11g44700|
gi|8704222|ref|Os11g44760|
gi|8704229|ref|Os11g44820|
gi|8704232|ref|Os11g44850|
gi|8704240|ref|Os11g44920|
gi|8704246|ref|Os11g44980|
gi|8704250|ref|Os11g45020|
gi|8704251|ref|Os11g45030|
gi|8704252|ref|Os11g45040|
gi|8704255|ref|Os11g45070|
gi|8704256|ref|Os11g45080|
gi|8704258|ref|Os11g45100|
gi|8704259|ref|Os11g45110|
gi|8704260|ref|Os11g45120|
gi|8704263|ref|Os11g45140|
gi|8704264|ref|Os11g45150|
gi|8704266|ref|Os11g45170|
gi|8704270|ref|Os11g45210|
gi|8704275|ref|Os11g45260|
gi|8704276|ref|Os11g45270|
gi|8704280|ref|Os11g45300|
gi|8704283|ref|Os11g45310|
gi|8704284|ref|Os11g45320|
gi|8704287|ref|Os11g45350|
gi|8704288|ref|Os11g45360|
gi|8704289|ref|Os11g45370|
gi|8704297|ref|Os11g45450|
gi|8704298|ref|Os11g45460|
gi|8704301|ref|Os11g45490|
gi|8704304|ref|Os11g45520|
gi|8704307|ref|Os11g45550|
gi|8704309|ref|Os11g45570|
gi|8704310|ref|Os11g45580|
gi|8704315|ref|Os11g45630|
gi|8704316|ref|Os11g45640|
gi|8704332|ref|Os11g45770|
gi|8704335|ref|Os11g45800|
gi|8704336|ref|Os11g45810|
gi|8704337|ref|Os11g45820|
gi|8704338|ref|Os11g45830|
gi|8704342|ref|Os11g45870|
gi|8704344|ref|Os11g45890|
gi|8704346|ref|Os11g45910|
gi|8704349|ref|Os11g45940|
gi|8704351|ref|Os11g45960|
gi|8704362|ref|Os11g46050|
gi|8704363|ref|Os11g46060|
gi|8704368|ref|Os11g46110|
gi|8704369|ref|Os11g46120|
gi|8704372|ref|Os11g46150|
gi|8704375|ref|Os11g46180|
gi|8704379|ref|Os11g46220|
gi|8704382|ref|Os11g46250|
gi|8704385|ref|Os11g46280|
gi|8704387|ref|Os11g46300|
gi|8704390|ref|Os11g46820|
gi|8704393|ref|Os11g46850|
gi|8704397|ref|Os11g46890|
gi|8704407|ref|Os11g46990|
gi|8704410|ref|Os11g47020|
gi|8704412|ref|Os11g47040|
gi|8704414|ref|Os11g47060|
gi|8704418|ref|Os11g47100|
gi|8704431|ref|Os11g47230|
gi|8704446|ref|Os11g47360|
gi|8704454|ref|Os11g47440|
gi|8704455|ref|Os11g47450|
gi|8704460|ref|Os11g47480|
gi|8704476|ref|Os11g47640|
gi|8704478|ref|Os11g47660|
gi|8704484|ref|Os11g47720|
gi|8704485|ref|Os11g47730|
gi|8704486|ref|Os11g47730|
gi|8704487|ref|Os11g47740|
gi|8704488|ref|Os11g47750|
gi|8704493|ref|Os11g47790|
gi|8704499|ref|Os11g47850|
gi|8704500|ref|Os11g47860|

gi|8704508|ref|Os11g47940|
gi|8704510|ref|Os11g47960|
gi|8704514|ref|Os11g47990|
gi|8704516|ref|Os11g48010|
gi|8704528|ref|Os11g48100|

Supplementary Table 5. Human-specific proteins.

1. ENSP00000343637 gene:ENSG00000133318 transcript:ENST00000338850
MAEPSAATQSHSISSSSFGAEPSAPGGGGSPGACPALGTKSCSSSCAGTDLGNSSDYKCH
TDQLEESLLIGAVLKGLLVFGLHSI

2. ENSP00000304128 gene:ENSG00000168746 transcript:ENST00000306731
MAYYFHFYGELPSQESPHPGVYSPHPQGGWQPTADSYREWHNEDLNPRMSHWTYAQELI
GKVRPRGANQSNISGRALP

3. ENSP00000311420 gene:ENSG00000174298 transcript:ENST00000309064
MEGCAVRRGSCPLLPGPSAWRASPAGWAGRAKLRSWCRASGLPNRPYTLTGGRHGSVSL
LRHPGTTTFVQQRSLHQSWEKRIVFSACPVSRSWCPERNFSGSIPAVTPPKLPGHSKSEGP
PGKVRKRTTIRSQPLFVTRTRGFGSAVGWLPLGSPVL

4. ENSP00000346823 gene:ENSG00000197627 transcript:ENST00000354771
EEEEEMAETYRRSRQHEQLPGQRHMDLLTGYSKLIQSRLKLLLHLGSQPPVGKTFFFSCW
CHPLFHGSKTTPNVWYKFSETALSILNSCQASQLGVRKMPGDMSSSPRVREFSALVAIKE
KIHILPTNAKVGSKFGS

5. ENSP00000334405 gene:ENSG00000186056 transcript:ENST00000334039
MAAEAKGWGCRLPSCCPCPGKTGDPGPGNRRRATPMWMPKGPTQERCRPYPPPLPGMIH
APAREGKNLPAPLPLGSSGLYPGKKEVADSVTEIRLRDPLSAPALRLGRSQLCPVWVRHPV
PLCCFSASDLGLFLIPGSHP

6. ENSP00000320505 gene:ENSG00000180819 transcript:ENST00000315448
MLTPRKAFRTCSKESGLAETESCGQTHTWPRALAVLMGLWWPRDQKAGEEDLRFRERRP
GLQATATGSGEHGAFPVHSQGVWASTHWQGTAVCPLQTPPPDAFIRNNKVLS

7. ENSP00000315177 gene:ENSG00000178043 transcript:ENST00000317017
MPTGPQSASFGITGDDGSPVMAKPPSIPPFLITHLLWKICHLGFNWIRRETVGTQEQNGA
RRYVLLPMQHVKKCPTCPHLKIRPLTSQKIRYSGPGAVAHACNRNTSGGRGGRIVRSRVR
DQPGK

8. ENSP00000335616 gene:ENSG00000186354 transcript:ENST00000334490
MVRIWTTIMIVLILLLRIGPNKPSLSGRQAPAQAQTSDLVPSLFPLGLWAPGFCTWSSPD
EDKVWRPAWEQGPKGEPDPRGLRPRKPVPGTGNRDSGTRRRLQDATEQDPRPGNDVASA
ETAGPPSPSGIRAQDRAPRHRRAPPARMPVAPAPSADGEPLQEQGGGLFHRTRSVYNGLEL
NTWMKVERLFVEKFHQSFSLDN

9. ENSP00000328696 gene:ENSG00000184767 transcript:ENST00000330598
CETPRLPEIALRALPRPGVSSLGTILCVPSVPVGPIVLTSATPESSRFTRPAPSTVPEAH
RHSRILRLAPVPCRCYRPPAPAHTSRSTSLFYESPSTFNV

10. ENSP00000313049 gene:ENSG00000181563 transcript:ENST00000324328
MGIEKRFNVLKIKVTQLQCHMGRRPGCECSWELRAPVTVASFLWSPTDSGHLPLLQDTSG

PPEGTHRTFTPGRELVLGPKPTVPGKPFLASALLNV

11. ENSP00000347609 gene:ENSG00000198636 transcript:ENST00000355435
MMTQKVALLSYSHRSPSLHSFFPQSLRLRPHVLTGGFIVSSISKPLQWYLVLLSMISSLG
AGEVYIPYSVQFSKSFVCRTGSHLYIHTLWGKPRSSYTASWVYFPKHFLPPCNLPSTFCF
PQLPLFNLVAMKLLLSL

12. ENSP00000351966 gene:ENSG00000196198 transcript:ENST00000359068
MMTQKVALLSYSHRSPSLHSFFPQSLRLRPHVLTGGFIVSSISKPLQWYLVLLSMISSLG
AGEVYIPYSVQFSKSFVCRTGSHLYIHTLWGKPRSSYTASWVYFPKHFLPPCNLPSTFCF
PQLPLFNLVAMKLLLSL

13. ENSP00000331089 gene:ENSG00000100336 transcript:ENST00000328429
MGSWVQLITSVGTSGLFLGVRVREEGAGMRCSKTIQAGQWLDSSKGPLGPSPPPVPTAGY
SSSFCVHYVNLLPGVLVLSVTSQYPHLSMALCQLAAHDWPRLSCVCV

14. ENSP00000354151 gene:ENSG00000196273 transcript:ENST00000360899
MGIGTGHTSMNKGGKDVTLLELSVEKRRWRINMETSKIILEKMQSDDVLDGNRERSNERE
GRDSLSEKLKSKQNLKDEEKLRYIKTGKSIQVEGTVRAKALRWVQ

15. ENSP00000326241 gene:ENSG00000180525 transcript:ENST00000316379
MESSRWDKDPPGERRPQQSQHWRARDHGARGCGPRQPTATASPRPGLWITPAHGSHTPQ
TNTRRTQADNIFIYESWLIHHGTQMSSVLPQPPLVRGPWHNTNSPWDSWASRGKLRVCPC
R TPRLHSSGCFSSKAGTALSPSLPVPGLRPQPPFLQKPLSILAPATPPALVSPTPPKLSPG
QLSPHSVNVHWGPQGHLHLPRSGTTVLHAYLQTLSSPASHQ

16. ENSP00000348873 gene:ENSG00000197152 transcript:ENST00000356485
MPAYWRPSNRVANHVLSRLSGRPGSYSATSRPRLFPVRGSCHFRLFPPRVPISCVCSRVP
EPAPEMSTRGFRVGTNLERQWPALSGLRWLLRLGLLLRLQGRPLHGFLPSQVSRGQLLPG
GARNRILSGEDWRE

17. ENSP00000323241 gene:ENSG00000180066 transcript:ENST00000321248
MWSFLPGAESVSMGPVPGVSSLGACWTHDQDSGRAEDRPQAPRITQYTWVLSFLFTEKPQ
TRSTSPISHQGQPQTTRALSLRQPQHPSAPASGRPRPPHSSGPDLAEAAPVVDQASQAAG
RASSGLGLWEQASVSQGFRNAAFEA

18. ENSP00000354198 gene:ENSG00000196530 transcript:ENST00000360940
MAPLHSSLGDRARLRLPILKKKKKKKKCKDVIMWQGVRVKKKTRTSFPLLSQLTSRTVI
TLKSRGQRNGLQTASSGAKLKRKIPLLSLLF

19. ENSP00000334662 gene:ENSG00000186195 transcript:ENST00000334247
MCRKVCAALDLWSNPHNHQYCLRLPAGSQGLRDSIFKGICTSLSSCTLVFYSLQPRTTPQ
SFPAPYLCSDLQQNVFHLSFKSVACNLCPH

20. ENSP00000333284 gene:ENSG00000183610 transcript:ENST00000330991
MPAFFSLPAERRLQAWPQSEAPLSVSSCFQNRPPEPASFQNLRPEPASLQNLRTEPTSF

21. ENSP00000328055 gene:ENSG00000183385 transcript:ENST00000329684
MVDVSQPEGTSRRVPGMNREIPKDPKGYAERRAARHTQDQHLSVFHANPRRDTSSLLCRT
P

22. ENSP00000331202 gene:ENSG00000185700 transcript:ENST00000328819
MVDVSQPEGTSRRVPGMNREIPKDPKGYAERRAARHTQDQHLSVFHANPRRDTSSLLCRT
P

23. ENSP00000307805 gene:ENSG00000170846 transcript:ENST00000307533
MRPVDADEAREPREEPGSPLSPAPRAGRENLASLERERARAHWRARRKLLEIQSLLDAIK
SEVEAEERGARAPAPRPRAEAEERVARLCAEAERKAAEAARMGRRIVELHQRIAGCECC

24. ENSP00000351674 gene:ENSG00000196886 transcript:ENST00000358816
MLSVSILHQFACLGFWTFTKPLSQSVLVRVLQRNRTNIYIYVLRNLGWGYIPINVSQCQK
NCKLNHYKSRILCTHTHTHTHTENERESERETERERERERDWLKELCSLRIPKIYSLEAE
DPGKPMCISSLSLKT

25. ENSP00000346654 gene:ENSG00000196094 transcript:ENST00000354633
MGSIIQRSGPSYGGQVHHTAVRSIIRRSGPSYGGQVHHTAVRSVLKQKGL

26. ENSP00000268333 gene:ENSG00000140703 transcript:ENST00000268333
GWGKRGARGAGTGTGLGGPGTPESSVTPPEFPLPPATRITPNFPNTLDPAISRSSS

27. ENSP00000350316 gene:ENSG00000198012 transcript:ENST00000357687
MSKDKCWDPFFAATSKSEQQALRGAWEPLRKGHCPCLTSCPPLHSLLSYLTVTGCMLNM
VSAAPKSTRRRQGCHSDMNRSSYLNVQTSATGALGGKTSMTASLSLETFADQAGSGARV
CRRFFRTYHLSYIDVPFKAREEVWSCGELCAGNMRVCIVFLALPRLSEESIWATGVRSRHP
CLLFSR

Suppl. Table 6: Amino acid content of POFs and PDFs.

		A	C	D	E	F	G	H	I	K	L	M	N	P	Q	R	S	T	V	W	Y
Ag	PDFs	7.409	2.174	5.273	6.118	4.127	6.454	2.675	5.367	5.634	9.401	2.452	4.349	4.876	4.147	5.835	6.818	5.647	6.671	1.201	3.414
Ag	POFs	7.544	2.353	4.778	5.685	3.726	6.093	2.781	4.953	5.361	8.714	2.356	4.233	5.697	4.410	6.698	8.284	6.084	6.406	1.048	2.877
At	PDFs	6.440	1.922	5.404	6.551	4.361	6.529	2.301	5.413	6.459	9.280	2.525	4.386	4.789	3.416	5.364	8.686	5.166	6.811	1.276	2.954
At	POFs	6.174	1.783	5.245	7.101	4.129	6.274	2.218	4.936	6.752	8.978	2.704	4.179	5.181	3.473	5.817	9.661	5.215	6.463	1.212	2.555
Dm	PDFs	7.452	1.980	5.276	6.211	3.859	6.414	2.628	5.195	5.771	9.129	2.499	4.668	5.087	4.758	5.491	7.619	5.471	6.197	1.110	3.203
Dm	POFs	7.735	1.997	4.654	6.139	3.325	6.243	2.650	4.478	5.554	8.778	2.461	4.498	6.120	5.470	5.986	8.857	5.710	5.580	0.956	2.863
Hs	PDFs	7.147	2.424	4.685	6.729	3.894	6.755	2.638	4.534	5.826	9.959	2.341	3.590	5.967	4.506	5.744	7.739	5.227	6.175	1.298	2.866
Hs	POFs	7.561	2.820	3.719	6.337	3.226	7.345	2.783	3.330	5.037	10.152	2.251	2.826	7.822	4.925	6.813	9.116	5.215	5.186	1.676	1.930
Mm	PDFs	6.852	2.449	4.686	6.455	4.058	6.465	2.646	4.806	5.989	10.061	2.481	3.687	5.602	4.454	5.489	7.841	5.417	6.363	1.291	2.978
Mm	POFs	6.979	2.654	4.125	6.776	3.417	6.475	2.684	3.720	5.485	10.198	2.375	3.076	6.837	5.018	6.329	9.286	5.401	5.600	1.479	2.251
Rn	PDFs	6.945	2.375	4.690	6.517	3.981	6.540	2.617	4.656	5.944	10.081	2.446	3.645	5.644	4.426	5.664	7.845	5.417	6.451	1.293	2.898
Rn	POFs	7.099	2.351	4.353	7.107	3.212	6.431	2.646	3.657	5.557	10.115	2.385	3.116	6.720	5.123	6.352	9.067	5.431	5.681	1.437	2.210
Os	PDFs	9.159	1.917	5.511	6.145	3.778	7.324	2.540	4.495	5.261	9.021	2.459	3.516	5.605	3.528	6.502	7.460	4.923	6.783	1.372	2.726
Os	POFs	10.423	1.971	5.075	6.139	2.779	9.613	2.430	3.468	4.244	8.036	2.645	2.737	5.957	3.291	8.603	7.851	4.907	6.390	1.640	1.908
Sc	PDFs	5.998	1.367	5.745	6.466	4.388	5.499	2.157	6.495	7.478	9.311	2.165	5.712	4.359	3.908	4.582	8.262	5.728	5.915	1.108	3.425
Sc	POFs	5.003	1.418	5.291	6.355	4.638	4.304	2.208	6.292	7.688	9.767	2.360	6.198	4.253	4.223	4.798	9.641	5.999	5.256	0.983	3.418
Sp	PDFs	6.611	1.543	5.348	6.496	4.514	5.441	2.298	6.166	6.692	9.540	2.193	4.923	4.682	3.741	5.090	8.558	5.315	6.313	1.152	3.432
Sp	POFs	5.459	1.476	4.912	6.758	4.789	3.972	2.209	6.065	7.004	10.017	2.201	5.486	4.566	4.104	5.065	10.343	5.710	5.427	1.060	3.398
Ce	PDFs	6.389	2.114	5.144	6.056	5.157	5.652	2.303	6.405	6.341	8.790	2.719	4.846	4.686	3.892	5.009	7.674	5.787	6.365	1.186	3.371
Ce	POFs	6.266	2.032	5.020	6.663	4.848	5.037	2.242	5.818	6.823	8.269	2.844	4.965	5.197	4.363	5.557	8.508	5.718	5.819	0.990	3.084

POFs/PDFs:	A	C	D	E	F	G	H	I	K	L	M	N	P	Q	R	S	T	V	W	Y
Ag	1.01821	1.0823	0.9061	0.9292	0.903	0.9442	1.0395	0.9228	0.9516	0.9269	0.9606	0.9734	1.1684	1.0633	1.1478	1.215	1.0774	0.9603	0.8723	0.8428
At	0.95868	0.928	0.9706	1.0839	0.9469	0.9609	0.9641	0.912	1.0454	0.9674	1.0707	0.9528	1.0819	1.0166	1.0845	1.1123	1.0094	0.9488	0.9499	0.865
Dm	1.03794	1.0084	0.8821	0.9883	0.8616	0.9733	1.0083	0.862	0.9624	0.9615	0.9849	0.9635	1.203	1.1497	1.0902	1.1625	1.0437	0.9004	0.8614	0.8937
Hs	1.05797	1.1634	0.7938	0.9418	0.8286	1.0873	1.0548	0.7343	0.8646	1.0194	0.9615	0.7871	1.3109	1.093	1.1861	1.178	0.9976	0.8399	1.2919	0.6737
Mm	1.01849	1.0834	0.8802	1.0496	0.8419	1.0015	1.0145	0.7742	0.9158	1.0136	0.9572	0.8343	1.2204	1.1268	1.1531	1.1843	0.997	0.88	1.1454	0.756
Rn	1.02223	0.9899	0.9282	1.0906	0.8067	0.9834	1.011	0.7853	0.9349	1.0034	0.9753	0.8548	1.1907	1.1574	1.1214	1.1558	1.0027	0.8806	1.1108	0.7628
Os	1.13806	1.0281	0.9209	0.999	0.7357	1.3126	0.9565	0.7716	0.8066	0.8909	1.0757	0.7784	1.0628	0.9329	1.3231	1.0524	0.9969	0.9421	1.1946	0.6998
Sc	0.83408	1.037	0.9211	0.9828	1.0571	0.7827	1.0237	0.9688	1.0281	1.049	1.0902	1.0852	0.9757	1.0806	1.0472	1.1669	1.0473	0.8884	0.8871	0.9978
Sp	0.82569	0.9571	0.9185	1.0403	1.0609	0.73	0.9614	0.9836	1.0465	1.0499	1.0034	1.1144	0.9752	1.0971	0.995	1.2086	1.0744	0.8597	0.9208	0.9901
Ce	0.98077	0.9614	0.9759	1.1003	0.9401	0.8912	0.9734	0.9083	1.076	0.9407	1.0461	1.0245	1.109	1.1211	1.1094	1.1087	0.9881	0.9142	0.8351	0.9146

	POFs/PDFs:	A	C	D	E	F	G	H	I	K	L	M	N	P	Q	R	S	T	V	W	Y
	Mean	0.98921	1.0239	0.9098	1.0206	0.8982	0.9667	1.0007	0.8623	0.9632	0.9823	1.0126	0.9369	1.1298	1.0838	1.1258	1.1544	1.0234	0.9014	1.0069	0.8396
	Std Error	0.03052	0.0225	0.0163	0.0194	0.0333	0.0508	0.011	0.0284	0.0275	0.0169	0.0167	0.0378	0.0344	0.0213	0.028	0.0158	0.0108	0.0125	0.0517	0.036
	0.01			**					**					**	**	**	**		**		**
ANOVA	0.05			**		**			**					**	**	**	**	**	**		**

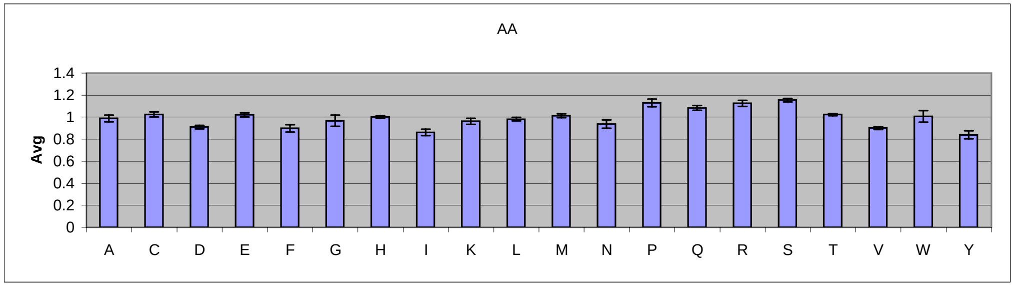

Supplement: Additional data file 1 — Supplemental Figures 1-1 through 1-10 show the relative similarity among PDFs and POFs in all proteomes studied. [file gb-2006-7-7-r57-S1.pdf]
